# Associations of host gene expression in the brain, liver, muscle with fecal and cecal microbiota composition in pigs

**DOI:** 10.1007/s10142-026-02009-5

**Published:** 2026-08-15

**Authors:** Camila Sabino de Oliveira, Bárbara Silva-Vignato, Simara Larissa Fanalli, Mariah Castro Durval, Felipe André Oliveira Freitas, Andrezza Maria Felício, Lucas Echevarria Nascimento, Fernanda Nery Ciconello, Thiago Sugizaki dos Santos, Luciana Correia de Almeida Regitano, Luiz F. Brito, Aline Silva Mello Cesar

**Affiliations:** 1 Department of Animal Science, Luiz de Queiroz College of Agriculture, University of São Paulo, Av. Pádua Dias 11, Piracicaba, SP 13418-900 Brazil; 2 School of Animal Science and Food Engineering, University of São Paulo, Av. Duque de Caxias Norte, 225, Pirassununga, SP 13635-900 Brazil; 3 Department of Animal Sciences, Purdue University, 270 S. Russell Street, West Lafayette, IN 47907 USA; 4 Embrapa Southeast-Cattle Research Center, Rod. Washington Luiz, Km 234, Fazenda Canchim, São Carlos, SP 13560-970 Brazil

**Keywords:** Gut-brain axis, Short-chain fatty acids, Multi-omics integration, Amplicon sequence variant

## Abstract

Understanding the genetic regulation underlying host–microbiota interactions is key to elucidating how microbial communities influence host metabolism, immunity, and physiology. In this context, the main objective of this study was to integrate genomic, transcriptomic, and microbial data to identify shared regulatory variants, highlight key microbial taxa, and prioritize biological pathways related to lipid metabolism, immune function, and host metabolic adaptation across multiple tissues in 72 immunocastrated male Large White pigs (S*us scrofa*). Expression quantitative trait *loci* (eQTL)-informed association analyses were performed to link the gut microbiota with host genetics. The analyses included microbial amplicon sequence variants (ASVs, ≥ 10% prevalence) from 16S rRNA sequencing, along with host cis- and trans-eQTL identified in brain, liver, and muscle tissues. Cross-tissue associations were detected between host gene expression and several bacterial genera, particularly *Oscillospira*, *Candidatus Methanoplasma*, *Roseburia*, *Anaerostipes*, and *Monoglobus*. A subset of eQTL, cis- and/or trans, showed multiple significant associations and regulated the expression of genes including *ZDHHC2*, *TMEM69*, and *CCDC167*, which have been implicated in lipid metabolism, immune regulation, and energy balance. The brain’s gene expression showed the most significant number of regulatory variants and enriched pathways related to fatty acid metabolism and immune processes, highlighting potential interactions along the gut–brain axis. These findings provide insights into the underlying host–microbiota relationships in pigs. Integrating eQTL and microbiota data across tissues revealed genetically mediated, cross-tissue regulatory networks linking microbial composition to host metabolic and immune functions. This offers a framework for functional validation and multi-omics studies in pigs.

**Supplementary Information:**

The online version contains supplementary material available at 10.1007/s10142-026-02009-5.

## Introduction

The gut microbiota plays a central role in host metabolism, immunity, and physiological homeostasis, influencing nutrient absorption, energy balance, and the inflammatory system (Tremaroli and Bäckhed 2012; Marchesi et al. 2016). Beyond responding to dietary and environmental stimuli, gut microbial communities actively shape host signaling through metabolites, such as short-chain fatty acids (SCFA) and microbial-modified bile acids, which regulate host lipid and immune metabolism. These metabolites link microbial activity to host gene expression, lipid metabolism, and immune function, embedding the microbiota within the broader regulatory framework of the organism (Tremaroli and Bäckhed 2012; Cryan et al. 2019).

In pigs, the gut microbiota contributes to productive performance, metabolic efficiency, and health status, with associations reported for traits including growth, fat deposition, and immune resilience (Crespo-Piazuelo et al. 2019; Bergamaschi et al. 2020). The pig is also a valuable biomedical model due to anatomical, physiological, and genomic similarity to humans (Huang and Chen 2023; Tang et al. 2025). Despite its importance, the genetic mechanisms by which host gene expression shapes the porcine gut microbiome across tissues remain poorly understood (Crespo-Piazuelo et al. 2019; Bergamaschi et al. 2020).

Host genetic variation affects both microbial composition and gene expression across tissues (Teng et al. 2024). Expression quantitative trait *loci* (eQTL) provide a mechanistic connection between genetic variants and gene regulation, enabling detection of tissue-specific and cross-tissue effects that underpin complex traits (Teng et al. 2024). Recent large-scale porcine resources have mapped regulatory variants across many tissues, supporting tissue-aware interpretation of genomic signals (Teng et al. 2024). In parallel, evidence supports a coordinated metabolic–immune network linking the gut, brain, liver, and muscle, mediated by microbial metabolites and host signaling pathways (Yin et al. 2022; Chew et al. 2023; Sun et al. 2024). However, despite the increasing number of studies on the porcine gut microbiota, the integration of multi-tissue host regulatory variation (e.g., eQTL) with gut microbial variation has not yet been systematically explored in pigs.

To address this gap, we integrated genomic, transcriptomic, and microbial datasets to investigate the regulatory mechanisms linking host genetics, tissue-specific gene expression, and gut microbiota composition in pigs. Using a multi-tissue eQTL dataset from brain, liver, and muscle combined with cecal and fecal microbiota profiles, we performed association analyses to identify *loci* linking gene expression and bacterial genus abundance. Our main objectives were to identify shared regulatory variants, highlight key microbial taxa, and prioritize biological pathways related to lipid metabolism, immune function, and host metabolic adaptation. This integrative framework can contribute to advancing the mechanistic understanding of host–microbiota relationships.

## Methods

The biological samples analyzed here were obtained in previous studies (Almeida et al. 2021; Freitas et al. 2024), which described the data collection procedures and experimental conditions. The study complied with the ethical principles for animal experimentation approved by the Ethics Committee on Animal Use (CEUA) of the Luiz de Queiroz School of Agriculture (ESALQ) of the University of São Paulo (USP, Piracicaba, SP, Brazil) under protocol number 2018.5.1787.11.6 and CEUA number 2018–28.

### Animal sampling

Seventy-two immunocastrated male Large White pigs with an average body weight (BW) of 6.67 ± 2.3 kg at weaning (28 days of age) were enrolled in this study. The animals are offspring of three males and 32 females belonging to a private breeding program with an estimated inbreeding rate below 14% (Almeida et al. 2021).

The experimental period began in the growth phase, when pigs weighed 28.44 ± 2.95 kg on average at an average age of 71.0 ± 1.8 days (Almeida et al. 2021; Fanalli et al. 2022; da Silva et al. 2024). The trial lasted for 98 days, covering both the growing and finishing phases. Throughout the experiment, animals had ad libitum access to feed and water, provided through three-hole dry feeders and nipple drinkers in each pen.

During the growth phase, pigs were housed in pens containing four animals, balanced by initial BW and sire, ensuring that each pen included at least one animal from each sire. At the beginning of the finishing phase, animals were redistributed into 24 pens, with three pigs per pen, maintaining the grouping established in the growing phase. Additional details are provided in Almeida et al. (2021).

### Experimental design and diets

The experiment was conducted using a completely randomized block design, with initial BW used as the blocking factor criterion. Four dietary treatments were evaluated, with six replicate pens per treatment, three pigs per pen as the experimental unit.

The experimental diets consisted of a basal corn–soybean meal formulation balanced to meet or exceed the nutritional requirements of growing and finishing pigs (Rostagno et al. 2011), with the addition of different lipid sources and inclusion levels, as described below. The detailed composition of the experimental diets is provided in Almeida et al. (2021):

- Control (CON): basal diet with 1.5% degummed soybean oil;
- Soybean (SOY): basal diet with 3% degummed soybean oil;
- Canola (CO): basal diet with 3% canola oil;
- Fish (FO): basal diet with 3% fish oil.

### Biological samples

The 72 animals were slaughtered at 169 days of age, with a final average BW of 133.9 ± 9.4 kg, according to industry standards (Almeida et al. 2021) for the collection of biological samples from the tissues (cecum, brain, liver, and muscle). Except for feces, all samples were collected at the time of slaughter (immediately *post mortem*), frozen in liquid nitrogen, and stored in an ultra-freezer at -80 °C. The biological samples used included feces (n = 71) and cecum (n = 61) for microbiota analyses (16S rRNA sequencing) and brain (middle region of the frontal lobe), liver (right hepatic lobe), and *Longissimus lumborum* muscle (between the 10th and 11th ribs) for transcriptome analyses (RNA-seq). Fecal samples from animals were collected directly from the rectum three days before slaughter (166 days of age), immediately frozen in liquid nitrogen, and stored at -80 °C. One fecal sample could not be collected because no material was available at the time of rectal sampling. In addition, logistical constraints on the slaughter line prevented cecum sampling in 11 animals.

### Microbiota library preparation and sequencing

Before downstream statistical analyses, four cecal samples were excluded because they originated from pens represented by only a single animal. Such pens could not be incorporated into the randomized complete block design without biasing the estimation of block effects. Removing these samples ensured a balanced blocking structure and valid statistical comparisons. After filtering, 58 cecal and 71 fecal samples were retained for microbiota analyses, totaling 129 samples.

Bacterial genomic DNA from the samples was extracted using the ZymoBIOMICS DNA Miniprep (Zymo Research, Irvine, CA, USA), following the manufacturer’s recommendations. DNA integrity and purity were evaluated by 0.8% agarose gel electrophoresis, concentrations were determined using a NanoDrop One spectrophotometer (Thermo Fisher Scientific, Waltham, MA, USA).

The V3–V4 hypervariable region of the 16S rRNA gene was amplified using the Quick16S™ NGS Library Prep Kit (Zymo Research, Irvine, CA, USA). PCR reactions were performed on qPCR platforms to control the numbers of cycles and minimize chimera formation. Amplified products were quantified by qPCR, pooled at equimolar concentrations, purified using the Select-a-Size DNA Clean & Concentrator™ Kit (Zymo Research, Irvine, CA), quantified with a TapeStation® (Agilent Technologies, Santa Clara, CA, USA) and Qubit® fluorometer (Thermo Fisher Scientific, Waltham, MA, USA).

The positive (ZymoBIOMICS™ Microbial Community Standard) and negative controls were included in both DNA extraction and library preparation. Sequencing was performed on the NextSeq1000 System (Illumina) with a v3 reagent kit (600 cycles) and a 10% PhiX spike-in, in paired-end mode.

### Bioinformatic processing of microorganisms and phenotypic data

The quality of the raw sequencing data was assessed using FastQC (v0.12.1; https://www.bioinformatics.babraham.ac.uk/projects/fastqc/; Andrews 2010). The forward and reverse reads of each sample were processed using the DADA2 pipeline (v1.34.0; Callahan et al. 2016). This process included filtering and trimming the sequences (removing primers and truncating to 303 bp for forward reads and 260 bp for reverse reads), setting maxEE = 2 (Maximum Expected Errors), followed by error modeling, dereplication, inference of Amplicon Sequence Variants (ASVs), read pairing. Chimeras were removed using the *removeBimeraDenovo* function. Taxonomic classification of ASVs was performed using the SILVA reference database (v138.2; Quast et al. 2013).

After generating the ASV table with its respective taxonomic classification, ASVs with low representativeness (zero counts or prevalence below 10% in the samples) were excluded from subsequent analyses. Non-bacterial ASVs or those without a phylum-level classification were also removed. The remaining ASVs had their counts (in abundance) summed at the genus level. Then, the relative abundances were transformed using the centered log-ratio (CLR) transformation (Gloor et al. 2017). The summed and adjusted genera of bacteria were used as phenotypic data in the subsequent analyses.

### RNA extraction and mRNA sequencing

The procedures for RNA extraction, RNA-seq quality control, read counting, and normalization for skeletal muscle, brain, and liver samples are described in detail in Fanalli et al. (2022) and da Silva et al. (2024). Briefly, total RNA was extracted from the brain, liver, and muscle tissues, sequenced on the Illumina HiSeq 2500 platform (San Diego, CA, USA) using TruSeq kits, following the manufacturer’s instructions. An additional purification step was performed on brain samples before library preparation. The indexed libraries were pooled and clustered using the TruSeq PE Cluster Kit v4-cBot-HS. They were then sequenced across five lanes on the HiSeq 2500 using the TruSeq SBS Kit v4-HS (200 cycles) according to the manufacturer's protocols. The RNA-seq libraries generated at least 25 million reads per sample, across tissues. The sample accession of the mRNA expression data used in the dataset is available in the European Nucleotide Archive (ENA) repository (EMBL-EBI) under the accession numbers PRJEB52665 (brain tissue; www.ebi.ac.uk/ena/data/view/PRJEB52665); PRJEB50513 (liver tissue; www.ebi.ac.uk/ena/data/view/PRJEB50513); and PRJEB52629 (skeletal muscle tissue; www.ebi.ac.uk/ena/data/view/PRJEB52629).

The sequencing data were processed using the same pipeline for all three tissues (brain, liver, and muscle). Quality control was assessed using FastQC v0.11.8 [www.bioinformatics.bbsrc.ac.uk/projects/fastqc/] (accessed on 15 July 2021). Sequencing adapters and low-complexity reads were removed using Trim Galore (v0.6.5; Krueger 2015). After trimming, reads shorter than 70 bases and with Phred scores lower than 33 were discarded. Read mapping was performed using Bowtie2 (v2.4.3; Langmead and Salzberg 2012), RNA-Seq by Expectation Maximization (RSEM v1.3.1; Li and Dewey 2011). Gene expression was estimated as transcripts per million (TPM). These normalized TPM values were used for downstream tissue-specific association analyses (Fanalli et al. 2024).

### SNP called from RNA-seq data

Single-nucleotide polymorphisms (SNPs) were identified from RNA-seq data of each tissue separately (brain, liver, or muscle) using the Genome Analysis Toolkit (*GATK* v4.1.9.0; McKenna et al. 2010) in Genomic Variant Call Format (GVCF) mode (Van der Auwera et al. 2013). Genome coverage for each of the BAM files was calculated using the samtools (v1.9) package (Danecek et al. 2021; Li et al. 2009). The *addorreplacereadgroups* tool from Picard (https://broadinstitute.github.io/picard/) was used to assign read group information. SplitNCigarReads was used to split reads that contain ‘N’ operations in their CIGAR string. Additionally, the quality scores were calibrated using GATK’s BaseRecalibrator followed by applyBQRS. For variant calling, the HaplotypeCaller algorithm was used to call variants individually, producing GVCF files for each sample. These files were then merged using the *combinegvcf* tool, the joint genotyping analysis was performed using *genotypegvcf*, generating a VCF file with all the genotyped samples (Van der Auwera et al. 2013; Franke and Crowgey 2020). Variant filtering was performed in multiple steps: SNPs were selected using the *selectvariants* function, filters were applied using bcftools and vcftools to retain only variants with a quality score ≥ 30, depth of coverage ≥ 30, minor allele frequency ≥ 0.05, less than 20% missing data per site (max-missing = 0.8) (Durval et al. 2025; Freitas et al. 2024).

### DNA extraction

The genomic DNA extraction performed in this study was described previously (Durval et al. 2025; Freitas et al. 2024). Briefly, 30 mg of liver tissue from 72 animals was macerated in liquid nitrogen and processed using the HighPrep™ Blood & Tissue DNA Plus Kit (MagBio Genomics, London, UK), which uses nucleic acid isolation technology based on magnetic bead isolation. DNA quality and quantity were assessed with a NanoDrop 2000 spectrophotometer (Thermo Fisher Scientific, Waltham, MA, USA), integrity was verified by electrophoresis on a 1.5% agarose gel stained with GelRed (Biotium, Hayward, CA, USA). Approximately 1,000 ng of DNA per sample was genotyped by NEOGEN (Pindamonhangaba, SP, Brazil) using the GeneSeek Genomic Profiler (GGP) Porcine 50 K SNP chip containing 50,915 SNPs. Raw data in Illumina format were converted to “ped” and “map” formats compatible with PLINK (v1.9; Purcell et al. 2007) using the Zanardi pipeline (Marras et al. 2017) https://github.com/bioinformatics-ptp/Zanardi. SNP genomic coordinates were updated using the most recent annotation file of the GGP Porcine 50 K chip (GGP Porcine 50 K-24 v2).

### SNP panel (genotype file)

After quality control, the SNPs from RNA-seq data (brain, liver, and muscle) and SNP chip (GeneSeek Genomic Profiler Porcine 50 K chip) were merged using PLINK v1.9 *(–bmerge).* Additional quality control steps were applied in PLINK by removing variants with MAF lower than 5%, genotyping call rate lower than 95%, missingness per individual higher than 0.20, by removing SNPs showing extreme deviation from Hardy–Weinberg equilibrium (*p* < 1 × 10⁻⁶), and pruning for linkage disequilibrium (LD; r^2^ > 0.7). LD pruning was applied using the command *–indep-pairphase* 50 5 0.7, removing one SNP from each pair with r^2^ > 0.70 within a sliding window of 50 SNPs shifted by five SNPs at each step.

### Identification of eQTL

Three eQTL analyses were performed, one for each tissue (brain, liver, and muscle), using their respective gene expression files and the same SNP panel. eQTL mapping was performed using the Matrix eQTL package in R (v4.3.0; Shabalin 2012) to identify associations between the SNPs and the gene expression levels in each tissue. Cis- and trans-eQTL were defined using a 1 Mb distance threshold. Genomic windows extending 1 Mb upstream from the beginning of each regulated gene and 1 Mb downstream from its end were used to classify cis (local) effects, whereas SNPs located more than 1 Mb away from the regulated gene were classified as trans (distant) effects (Durval et al. 2025; Freitas et al. 2024).

The Matrix eQTL R package tests the linear association between each SNP and gene expression level, assuming an additive genetic effect. It performs a separate test for each SNP–gene pair and corrects for multiple testing based on the false discovery rate (FDR). eQTL were considered significant at an FDR threshold of 0.05. The analyses were done using the modelLINEAR (linear) function for both cis- and trans-eQTL based on the following fixed linear regression model fitted:

$$Y=\beta *s+PC+SIRE+TREAT+\varepsilon \varepsilon $$

where Y is the gene expression level in normalized TPM, β is the SNP allelic substitution effect, s is the genetic marker covariate, coded as 0 (homozygous for the reference allele), 1 (heterozygous), 2 (homozygous for the alternative allele), PC represents the first four principal components fitted as linear covariates to correct for potential population stratification. These principal components were calculated in PLINK v1.9 using the final post-QC SNP genotype matrix (–pca) and together explained 38.5% of the total genetic variance. SIRE is a dummy variable representing the sire effect, TREAT is a dummy variable representing the treatment effect, ε is the random residuals with ε ∼ i.i.d.N (0, σ^2^) (Freitas et al. 2024). For downstream analyses, a tissue-specific non-redundant list of SNPs identified as significant cis- and trans-eQTL (FDR ≤ 0.05) was generated by retaining only unique SNPs.

To facilitate biological interpretation and reduce redundancy, given the large number of detected associations, eQTL presenting at least two significant associations across the genome-wide analyses, regardless of tissue or microbiota compartment, were selected for detailed characterization and further discussion.

### Functional annotation and enrichment analyses

The significant cis- and trans-eQTL identified were annotated and analyzed for functional enrichment separately. Annotation was performed using the Ensembl Variant Effect Predictor (VEP) tool (Ensembl release 114—May 2025; McLaren et al. 2016), based on the *Sus scrofa* 11.1 reference genome assembly available at Ensembl (www.ensembl. org/Sus_scrofa/Info/Index). Functional annotation of genes associated with cis- and trans-eQTL was performed separately using Ensembl BioMart (release 112). The same procedure was applied to genes associated with significant eQTL identified in the association analyses.

Functional enrichment analyses were conducted using the gprofiler2 R package (v0.2.3; Kolberg et al. 2020). Gene Ontology (GO) terms, including biological process (BP), cellular component (CC), molecular function (MF), were retrieved for this analysis. We also assessed Kyoto Encyclopedia of Genes and Genomes (KEGG) pathways and Reactome (REAC) pathways using the same gprofiler2 R package. Functional enrichment was considered significant for terms with *P*-values < 0.05, highlighting terms related to fatty acids, immunity, health, lipid metabolism.

Additionally, orthologous relationships between porcine genes with human and mouse were assessed for novel genes (without annotated gene symbols) associated with eQTL presenting at least two significant associations across the genome-wide analyses. Ortholog mapping was performed using g:Profiler (Raudvere et al. 2019) through the gprofiler2 R package (Kolberg et al. 2020). Results were further validated using Ensembl BioMart (release 115). Analyses were conducted using *Sus scrofa* as the reference species and *Homo sapiens* and *Mus musculus* as target species. Genes with annotated orthologs were retained for interpretation.

### eQTL-microbiota association analyses

CLR-transformed abundances of each bacterial genus were previously adjusted for sire and diet effects using a linear model in R. The residuals obtained from this adjustment were used as phenotypes in the subsequent association analyses. For each tissue (brain, liver, and muscle), a tissue-specific eQTL-informed SNP subset was created based on a non-redundant list of SNPs identified as significant cis- or trans-eQTL, in which each SNP was retained only once regardless of the number of associated genes. Using each tissue-specific SNP set, two independent association analyses were performed: one for cecum microbiota traits and one for fecal microbiota traits. Separate analyses were conducted for each bacterial genus. eQTL-microbiota association tests were performed in the GCTA software (Yang et al. 2011) using the *mlma-loco* approach. The genomic relationship matrix (GRM) was constructed using the full post-QC eQTL panel (RNA-seq SNPs + 50 K SNP chip). The model fitted was:

$$Y=a+\beta *x+g+\varepsilon $$

where Y is the adjusted abundance of each bacterial genus, a is the overall mean, β is the additive SNP effect, x is the indicator variable of the SNP genotype coded as 0 (homozygous for the reference allele), 1 (heterozygous), or 2 (homozygous for the reference alternative), g is the animal polygenic effect (random effect), that is, the cumulative effect of all SNPs (as modelled by the genomic relationship matrix), and ε is the residual effect.

The FDR was controlled at 0.05 to account for multiple testing. To evaluate potential inflation of test statistics, genomic inflation factors (λ) were calculated for each analysis based on the distribution of chi-square statistics. A threshold of λ > 1.05 was used to indicate potential inflation. When inflation was detected, *p*-values were adjusted using λ-corrected test statistics. FDR correction was then applied within each trait. Statistical significance for downstream biological interpretation and discussion was defined based on FDR-adjusted p-values (q < 0.05). Quantile–quantile (QQ) plots were also generated to visually assess the distribution of test statistics. In the Manhattan plots, genomic inflation-adjusted *P*-values were displayed on the y-axis, whereas SNPs corresponding to associations meeting the final significance criterion (q < 0.05) were highlighted directly.

## Results

### Cecal and fecal microbiota

A total of 6,976 and 6,912 bacterial genera were initially identified in the cecal and fecal microbiota, respectively. After quality control and prevalence filtering, 149 (2.1%) and 138 (2.0%) cecal and fecal genera, respectively, were retained for downstream analyses.

**Fig. 1:**
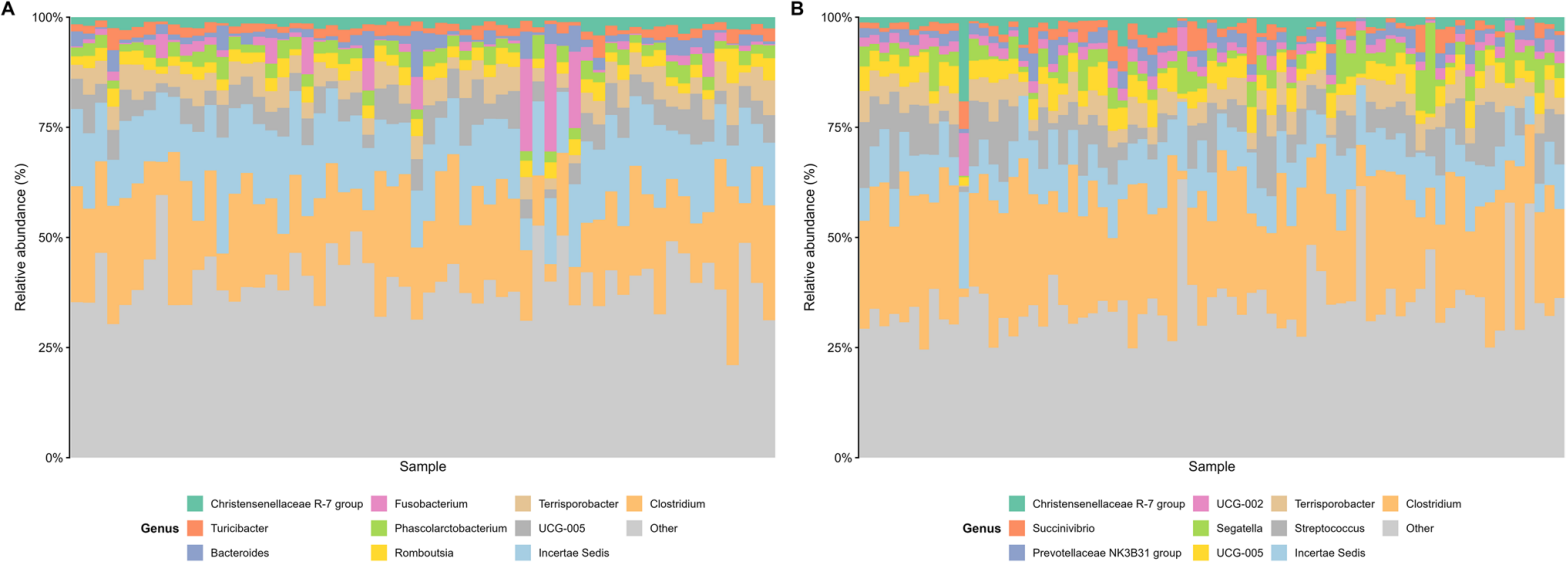
Relative abundance (%) of the 10 most abundant bacterial genera across samples in the cecal (**A**) and fecal (**B**) microbiota of pigs. Each stacked bar represents an individual sample, the remaining genera are grouped as “Other”

Overall, 164 unique bacterial genera were retained as phenotypes in the eQTL-informed microbiota association analyses across both sample types, with their CLR-transformed abundances summarized in Supplementary Table S1. Among these, 121 genera (73.8%) were shared between the cecal and fecal microbiota, according to their microbiota origin classification shown in Supplementary Table S2. The relative abundance of the 10 most abundant genera in each microbiota is shown in Fig. 1.

### Identification of SNPs

Following the merging of the 50 K (SNP chip) with SNPs identified from the expression data (RNA-seq) from brain, liver, and muscle, 197,867 variants were identified. Quality control procedures excluded 10,046 variants due to MAF, 49,971 due to missing genotypes, and 1,854 due to extreme deviation from Hardy–Weinberg equilibrium. A total of 135,996 SNPs remained for the subsequent analyses. LD pruning removed 91,997 variants, resulting in a final SNP set of 43,999 SNPs.

### Identification of eQTL

The analyses to identify eQTL in this study were performed separately for each tissue, focusing on gene expression levels in the brain, liver, and muscle. Supplementary Tables S3 and S4 provide the complete lists of significant eQTL identified in each tissue, including SNP genomic coordinates, regulated genes, their classification as cis- or trans-eQTL, the corresponding effect sizes and statistical significance metrics (p-values and FDR). A total of 13,999 eQTL were identified in the brain, including 9,278 trans-eQTL regulating 1,673 genes and 3,821 cis-eQTL regulating 1,047 genes. In the liver, 1,448 eQTL were detected, comprising 26 trans-eQTL regulating 19 genes and 1,422 cis-eQTL regulating 500 genes. In the muscle, 1,416 eQTL were identified, of which 491 trans-eQTL regulated 390 genes and 925 cis-eQTL regulated 357 genes.

To enable downstream association analyses, a tissue-specific non-redundant set of SNPs identified as significant eQTL (including both cis- and trans-eQTL) was generated by retaining each SNP only once, regardless of the number of associated genes. This resulted in 6,056, 1,221, and 885 unique SNPs for brain, liver, and muscle, respectively. The resulting SNP sets for each tissue are provided in Supplementary Table S5.

### Functional annotation and enrichment analyses of the eQTL

**Fig. 2:**
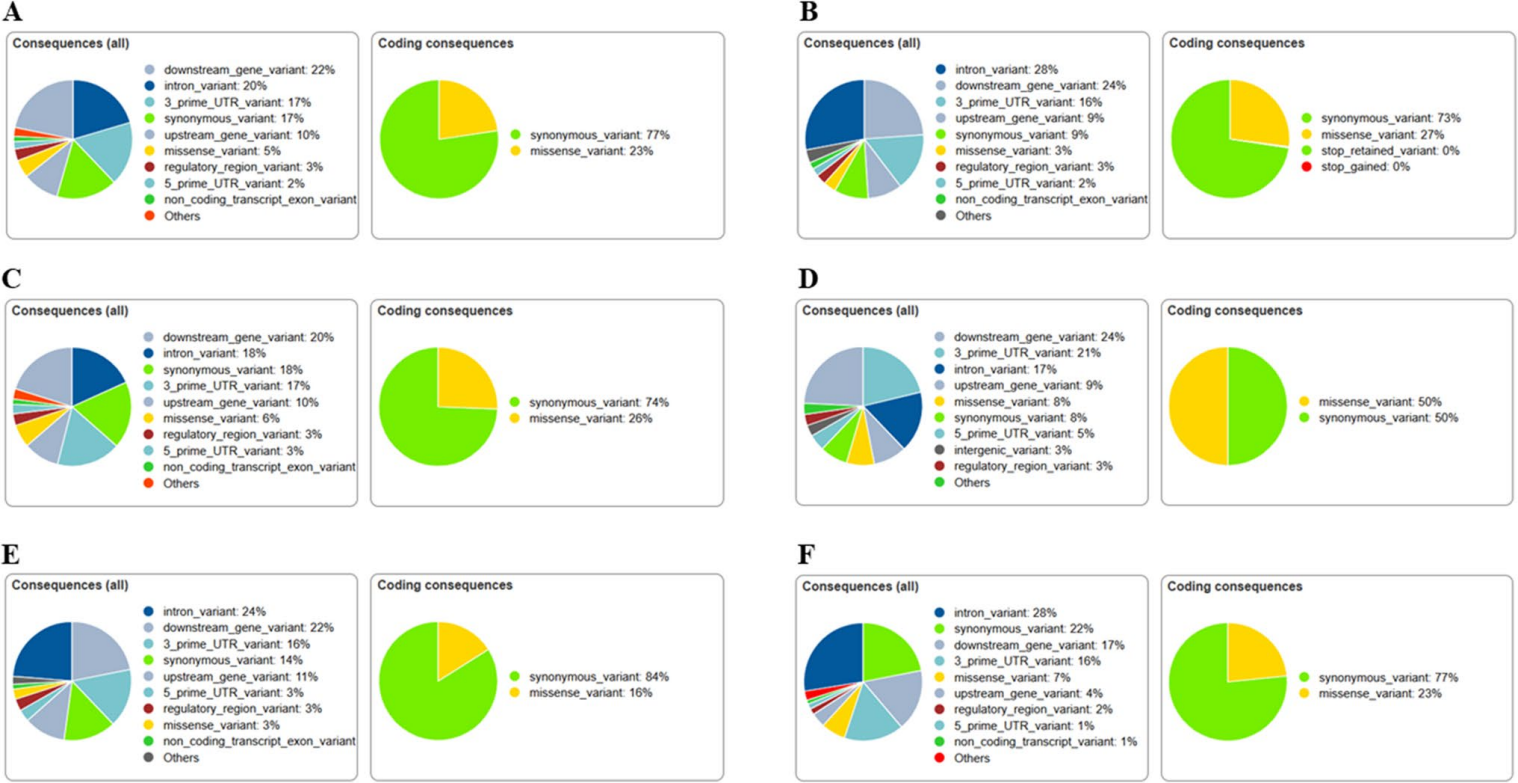
Primary functional consequences of cis- and trans-eQTL predicted by the Variant Effect Predictor (VEP) across tissues. Pie charts illustrate the proportion of eQTL variants assigned to each functional annotation category. Panels (**A**), (**C**), (**E**) correspond to cis-eQTL identified in the brain, liver, and muscle, respectively, whereas panels (**B**), (**D**), (**F**) represent trans-eQTL in the same tissues. Percentages indicate the relative contribution of each consequence class within each tissue and eQTL type

The primary consequences predicted by the Variant Effect Predictor (VEP) tool are presented in Fig. 2A-F. In the brain, 1,082 cis-eQTL (36.5%) and 1,533 trans-eQTL (44.3%) were identified as novel variants, mapping to 2,639 and 3,066 genes, respectively. In the liver, 494 cis-eQTL (40.9%) and 10 trans- (40%) were predicted as novel variants, mapping to 1,119 and 29 genes, respectively. In the muscle, 352 cis-eQTL (44.8%) and 83 trans-eQTL (63.8%) were identified as novel variants, mapping to 367 and 390 genes, respectively.

The gene counts mentioned above reflect genomic features overlapping the eQTL positions as defined by VEP. Consequently, these values do not represent unique genes, as a single eQTL may overlap multiple features. Cis-eQTL were annotated as 3,105 (brain), 725 (liver), 442 (muscle) in the respective tissues. A total of 6,514, 17, 376 trans-eQTL were annotated from the brain, liver, and muscle, respectively. The full enrichment results are available in Supplementary Tables S6 and S7, while the top 10 significantly enriched GO terms and KEGG pathways for cis- and trans-eQTL, with emphasis on lipid metabolism, immune-related processes, disease-associated pathways, are summarized in Tables 1 and 2, respectively.

**Table 1:** Top 10 significantly enriched Gene Ontology (GO) terms and KEGG pathways associated with cis-eQTL in brain, liver, and muscle tissues

Tissue	Category	Term	*P*-value
Brain	GO:MF	Small molecule binding	7.65E-07
Brain	GO:CC	Mitochondrion	8.08E-05
Brain	KEGG	Peroxisome	5.49E-03
Brain	KEGG	Herpes simplex virus 1 infection	7.39E-03
Brain	GO:BP	Small molecule metabolic process	1.08E-02
Brain	GO:MF	Oxidoreductase activity	1.42E-02
Brain	GO:CC	Lysosomal membrane	1.88E-02
Brain	GO:CC	Lytic vacuole membrane	1.88E-02
Brain	GO:CC	Peroxisome	2.76E-02
Brain	GO:CC	Microbody	2.76E-02
Liver	GO:BP	Small molecule metabolic process	1.65E-08
Liver	GO:MF	Oxidoreductase activity	1.54E-07
Liver	GO:BP	Organic acid metabolic process	2.66E-05
Liver	GO:BP	Carboxylic acid metabolic process	2.66E-04
Liver	GO:BP	Oxoacid metabolic process	3.37E-04
Liver	GO:BP	Organic acid catabolic process	1.09E-03
Liver	GO:BP	Carboxylic acid catabolic process	1.09E-03
Liver	GO:CC	Mitochondrion	4.44E-03
Liver	KEGG	Retinol metabolism	2.27E-02
Liver	KEGG	Drug metabolism—cytochrome P450	2.45E-02
Muscle	GO:CC	Cytoplasm	5.12E-06
Muscle	GO:MF	Catalytic activity	4.73E-04
Muscle	GO:MF	Transferase activity	2.34E-02
Muscle	GO:CC	Bounding membrane of organelle	3.04E-02
Muscle	GO:BP	Transport	4.17E-02

Enrichment analysis was performed using genes mapped by cis-eQTL identified in each tissue. For each tissue, the 10 most significant terms across Gene Ontology categories, including biological process (BP), molecular function (MF), cellular component (CC), as well as KEGG pathways, are shown. *P*-values indicate significance after multiple testing correction

**Table 2:** Top 10 significantly enriched Gene Ontology (GO) terms and KEGG pathways associated with trans-eQTL in brain, liver, and muscle tissues

Tissue	Category	Term	*P*-value
Brain	GO:CC	Extracellular region	1.01E-07
Brain	REAC	Keratinization	2.24E-06
Brain	KEGG	Coronavirus disease—COVID-19	7.90E-06
Brain	GO:CC	Extracellular space	4.18E-05
Brain	GO:MF	Serine-type peptidase activity	4.68E-03
Brain	GO:MF	Serine hydrolase activity	5.17E-03
Brain	REAC	Retinoid metabolism and transport	8.07E-03
Brain	REAC	Formation of the cornified envelope	1.26E-02
Brain	REAC	Metabolism of fat-soluble vitamins	1.42E-02
Brain	GO:MF	Serine-type endopeptidase inhibitor activity	1.51E-02
Liver	GO:MF	Acyloxyacyl hydrolase activity	3.93E-02
Muscle	GO:CC	Mitochondrion	1.51E-10
Muscle	GO:BP	Acetyl-CoA metabolic process	1.33E-03
Muscle	GO:BP	Phosphatidylglycerol metabolic process	4.00E-03
Muscle	GO:CC	Pyruvate dehydrogenase complex	4.70E-03
Muscle	GO:BP	Myeloid cell differentiation	5.88E-03
Muscle	GO:BP	Phospholipid metabolic process	5.88E-03
Muscle	KEGG	Thermogenesis	6.75E-03
Muscle	GO:BP	Acetyl-CoA biosynthetic process	9.39E-03
Muscle	GO:BP	Acyl-CoA metabolic process	1.41E-02
Muscle	GO:BP	Bone mineralization involved in bone maturation	1.54E-02

Enrichment analysis was performed using genes mapped by trans-eQTL identified in each tissue. For each tissue, the 10 most significant terms across Gene Ontology categories, including biological process (BP), molecular function (MF), cellular component (CC), as well as KEGG and Reactome (REAC) pathways, are shown. *P*-values indicate significance after multiple testing correction

### Association of eQTL with feces and cecum microbiota

**Fig. 3:**
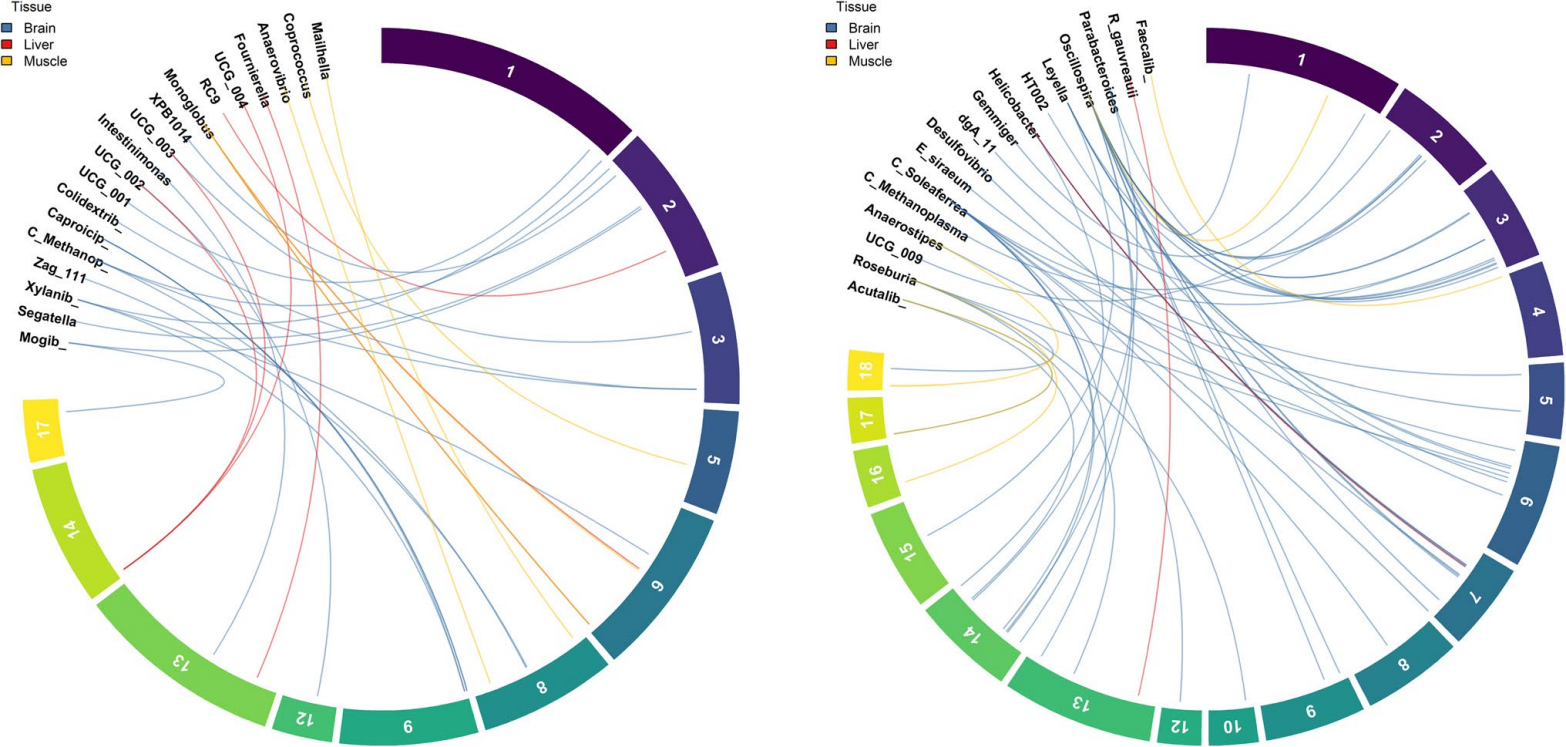
Significant associations between eQTL and bacterial genera across tissues. Panels (**A**) and (**B**) represent associations identified in the cecal and fecal microbiota, respectively. Links indicate significant associations between bacterial genera and eQTL detected in the brain (blue), liver (yellow), and muscle (red). Numbers along the outer ring correspond to chromosomes, only chromosomes with significant associations are shown. Bacterial genera are displayed on the opposite side of the diagram

Multiple eQTL-microbiota association analyses were performed, organized into six analytical blocks defined by tissue (brain, liver, muscle) and microbiota (cecum and feces). These analyses revealed associations between the cecal (*n* = 58) and fecal (*n* = 71) microbiota and eQTL identified across the three tissues. In the cecum, 31 eQTL were associated with 21 bacterial genera across the analyzed tissues. In the fecal microbiota, 58 associations involving 16 bacterial genera were detected. Significant associations are summarized in Tables 3 and 4 and visualized in Fig. 3, based on the complete set of associations provided in Supplementary Table S8. Genomic inflation factors (λ) indicated adequate control of test statistic inflation across analyses (Supplementary Table S9), which was further supported by the QQ plots shown in Supplementary Figure S1. QQ plots showed no evidence of systematic inflation (λ ≈ 1), with deviations restricted to the upper tail, consistent with true association signals.

**Table 3:** Genome-wide significant associations between microbial genera and eQTL-linked SNPs identified in the cecal microbiota across brain, liver, and muscle tissues

Tissue	Genus	SNP (Chr:Position)	Allele freq	Effect (β)	*P*-value	FDR
Brain	*Candidatus Methanoplasma*	2:4,695,744	0.0614	-1.1144	1.60E-05	0.0475
Brain	*Candidatus Methanoplasma*	3:118,016,028	0.0179	-2.5982	1.01E-06	0.0061
Brain	*Candidatus Methanoplasma*	8:74,604,767	0.1316	-0.8002	2.35E-05	0.0475
Brain	*Caproiciproducens*	9:217,643	0.0614	-1.1055	1.36E-05	0.0275
Brain	*Caproiciproducens*	9:450,977	0.1053	-1.1246	2.73E-06	0.0165
Brain	*Caproiciproducens*	9:2,976,766	0.0636	-1.1213	9.50E-06	0.0275
Brain	*Colidextribacter*	3:118,016,028	0.0179	-2.7275	2.87E-06	0.0174
Brain	Family XIII UCG 001	1:254,674,829	0.1250	-1.4365	1.60E-05	0.0486
Brain	Family XIII UCG 002	12:23,559,581	0.0431	-3.0867	2.31E-08	0.0001
Brain	*Intestinimonas*	13:81,452,573	0.0526	-1.6116	4.49E-06	0.0272
Brain	*Lachnospiraceae* UCG 003	3:53,853,269	0.0517	3.0224	7.79E-06	0.0472
Brain	*Lachnospiraceae XPB1014* group	2:15,403,524	0.0439	-1.9063	2.19E-06	0.0133
Brain	*Mogibacterium*	2:62,067,645	0.0536	-1.2164	4.25E-06	0.0168
Brain	*Mogibacterium*	17:48,221,664	0.0517	-1.3607	5.56E-06	0.0168
Brain	*Segatella*	2:59,450,319	0.0455	-2.8249	7.94E-07	0.0048
Brain	*Xylanibacter*	1:272,835,476	0.0625	-1.6445	8.53E-06	0.0172
Brain	*Xylanibacter*	6:62,442,754	0.0357	-1.9866	6.44E-06	0.0172
Brain	*Xylanibacter*	9:3,284,896	0.0439	-2.1417	1.89E-07	0.0011
Brain	*Zag 111*	8:73,421,124	0.0603	-1.7845	5.07E-06	0.0307
Liver	*Fournierella*	13:26,327,221	0.2364	-0.6300	1.27E-05	0.0155
Liver	*Monoglobus*	6:81,738,647	0.0982	-1.6267	1.40E-06	0.0017
Liver	*Monoglobus*	6:165,700,722	0.0862	-1.9871	1.08E-05	0.0066
Liver	*Rikenellaceae RC9 gut* group	2:117,034,881	0.4286	0.3613	3.86E-05	0.0471
Liver	UCG 002	14:6,325,377	0.3246	-0.4670	3.11E-05	0.0240
Liver	UCG 003	14:6,376,720	0.2589	-0.5366	9.34E-05	0.0380
Liver	UCG 004	14:6,521,475	0.2456	-0.5941	3.93E-05	0.0240
Muscle	*Anaerovibrio*	8:118,048,304	0.0982	-1.4625	3.29E-05	0.0291
Muscle	*Coprococcus*	5:63,787,481	0.4107	-0.8494	2.11E-05	0.0187
Muscle	*Mailhella*	8:12,634,540	0.5263	-1.3721	3.85E-06	0.0034
Muscle	*Monoglobus*	6:83,604,338	0.0893	-1.6691	8.15E-05	0.0361
Muscle	*Monoglobus*	6:165,700,722	0.0862	-1.9871	2.50E-06	0.0022

Associations were identified using genome-wide association analyses, SNPs are reported by chromosomal position. Effect sizes (β), allele frequencies, P-values, false discovery rates (FDR) are shown. Only associations reaching genome-wide significance after multiple testing correction are included

**Table 4:** Genome-wide significant associations between microbial genera and eQTL-linked SNPs identified in the fecal microbiota across brain, liver, and muscle tissues

Tissue	Genus	SNP (Chr:Position)	Allele freq	Effect (β)	*P*-value	FDR
Brain	*Acutalibacter*	17:5,704,977	0.1014	-1.4116	1.28E-07	0.0008
Brain	*Anaerostipes*	6:60,598,875	0.0588	2.3199	2.11E-06	0.0128
Brain	*Candidatus Methanoplasma*	2:79,413,383	0.0652	-2.0868	4.37E-06	0.0265
Brain	*Candidatus Soleaferrea*	6:17,655,847	0.0507	-1.2098	1.16E-06	0.0035
Brain	*Desulfovibrio*	7:47,762,010	0.0588	-1.0300	4.46E-06	0.0270
Brain	*dgA 11 gut* group	3:79,943,654	0.0714	-1.0860	2.80E-06	0.0170
Brain	*Eubacterium siraeum* group	5:14,851,097	0.0942	-1.0971	6.83E-06	0.0414
Brain	*Gemmiger*	1:254,208,153	0.0507	-2.6295	1.19E-06	0.0072
Brain	*Helicobacter*	7:31,189,076	0.4859	1.3309	1.43E-05	0.0291
Brain	HT002	6:88,697,664	0.0652	-2.0713	4.82E-06	0.0292
Brain	*Leyella*	6:67,383,248	0.0515	-1.3359	5.57E-06	0.0337
Brain	*Oscillospira*	3:119,454,184	0.0580	-1.3152	2.58E-06	0.0082
Brain	*Parabacteroides*	3:121,342,297	0.0915	-2.1119	5.31E-06	0.0168
Brain	*Roseburia*	10:11,842,433	0.0563	-1.6755	8.81E-06	0.0267
Brain	UCG 009	2:17,466,095	0.0797	-1.3261	1.86E-07	0.0011
Liver	*Helicobacter*	7:32,914,122	0.4859	-1.2811	2.55E-05	0.0312
Liver	*Ruminococcus gauvreauii* group	13:28,258,791	0.1765	-0.5002	3.55E-05	0.0433
Muscle	*Acutalibacter*	17:5,704,977	0.1014	-1.4116	6.56E-07	0.0006
Muscle	*Anaerostipes*	18:6,996,634	0.0588	2.5517	2.48E-05	0.0219
Muscle	*Faecalibacterium*	4:1,450,971	0.0662	-1.6093	3.21E-05	0.0285
Muscle	*Roseburia*	16:18,654,959	0.1929	-1.0038	3.30E-05	0.0292
Muscle	*Oscillospira*	1:190,724,221	0.0915	-1.1067	2.15E-05	0.0190

For genera showing multiple significant SNPs within a given tissue, only the most significant association (lowest FDR) is shown; the complete list of associations is provided in Supplementary Table S8. Columns include tissue, SNP identifier (chromosome:position), allele frequency, estimated allelic effect (β), nominal *P*-value, and false discovery rate (FDR)

**Fig. 4:**
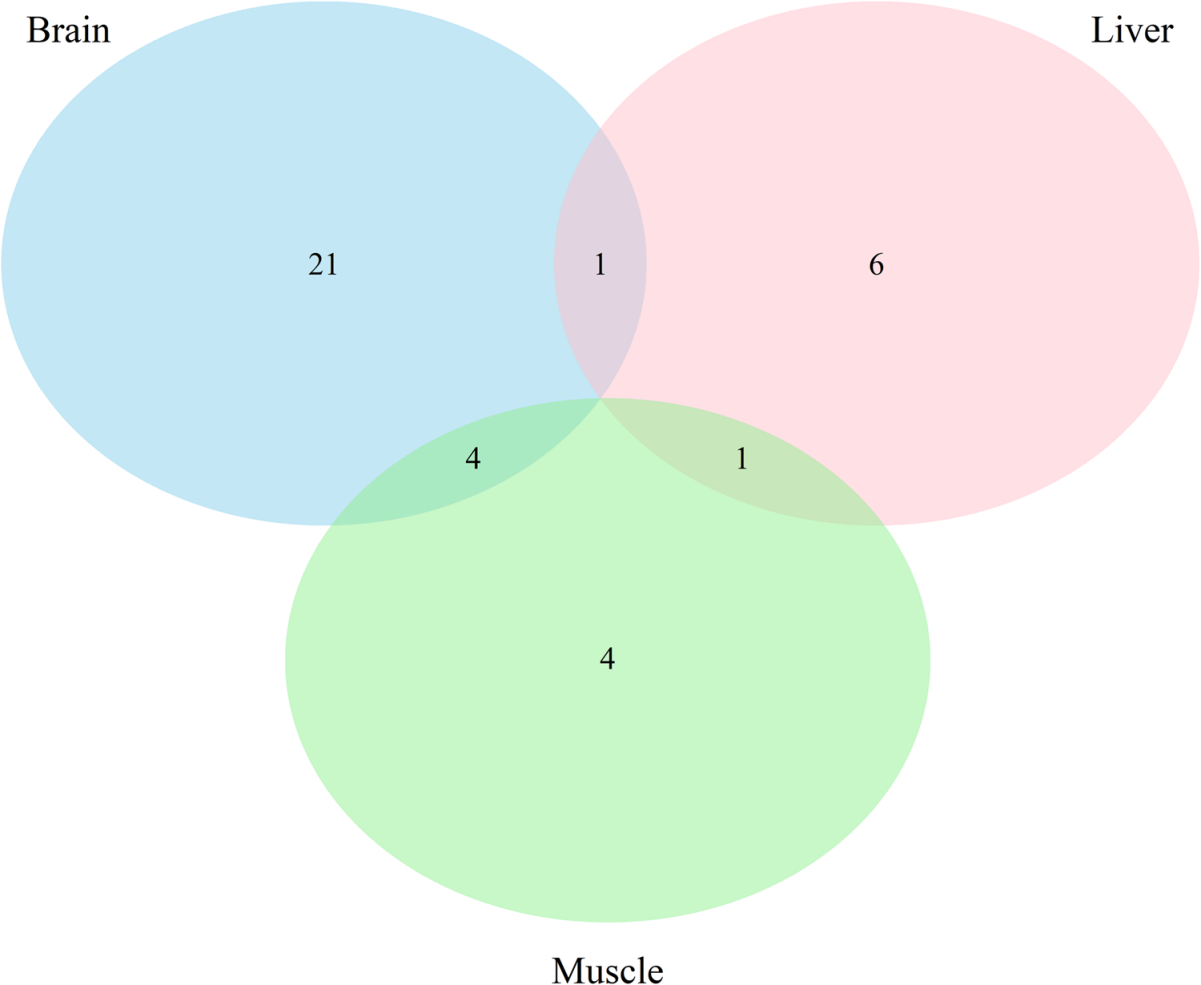
Venn diagram illustrating the overlap of significant host–microbiota associations across brain, liver, and muscle tissues. Numbers indicate the total and shared associations identified within and between tissues

A total of 6,056, 1,221, and 884 eQTL from the brain, liver, and muscle expression analyses, respectively, were used for the association analyses. Overall, the number of statistically significant eQTL–microbiota associations varied across bacterial genera, host tissues, and gut compartments, with a greater number of associations identified in the fecal than in the cecal microbiota. Only one genus, *Candidatus Methanoplasma*, was shared between the fecal and cecal microbiota. The overlap of bacterial genera associated with eQTL across tissues is illustrated in Fig. 4. The frequency of significant associations per bacterial genus and tissue is detailed in Supplementary Table S10, complementing the patterns illustrated in Fig. 4.

**Fig. 5:**
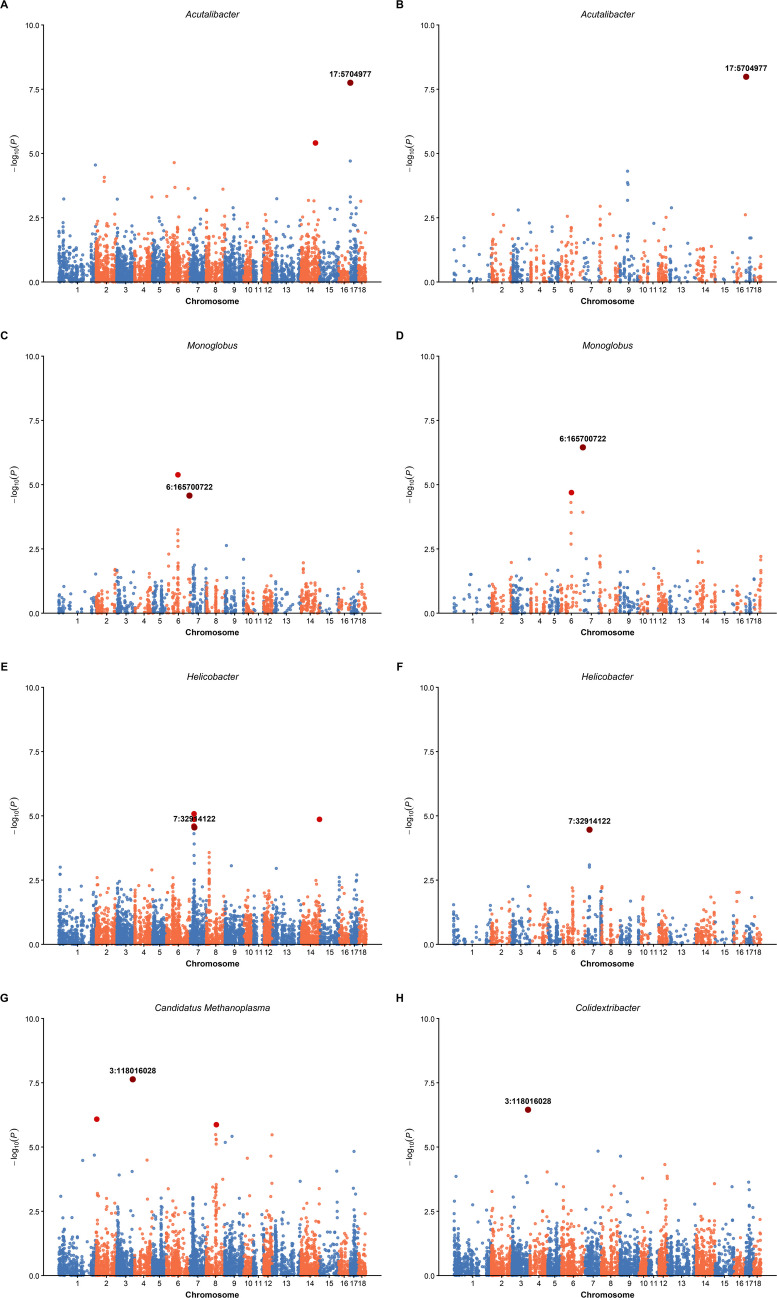
Manhattan plots illustrating eQTL–microbiota association analyses between host genetic variants and gut microbiota genera. The y-axis represents genomic inflation-adjusted *P*-values expressed as − log10(P). Associations that remained significant after false discovery rate correction within each trait (q < 0.05) are highlighted in red. The larger dark-red point and its corresponding chromosome:position label indicate the representative eQTL discussed in the paper. The remaining points are colored alternately by chromosome. The plots show cis- or trans-eQTL presenting multiple significant associations across host tissues (brain, liver, and muscle) and gut compartments (cecal and fecal microbiota)

Five eQTL met the criterion of having at least two significant associations in the eQTL-microbiota association analyses, their corresponding plots are shown in Fig. 5A-H. Three of these eQTL were associated with the same microbiota compartment and bacterial genera but across different tissues: SSC17:5,704,977 (chromosome:position) in the brain and muscle (*Acutalibacter*); SSC6:165,700,722 in the liver and muscle (*Monoglobus*); and SSC7:32,914,122 in the brain and liver (*Helicobacter*). The remaining two eQTL (SSC3:118,016,028 and SSC3:79,943,654) showed significant associations within the same tissue and microbiota. The classification of these eQTL (cis or trans), related genes, and gene symbols is summarized in Table 5.

**Table 5:** Significant eQTL associated with bacterial genera in the cecal and fecal microbiota

Chr	SNP	Microbiota compartment	Genus	Tissue	eQTL type	Gene ID	Gene symbol
3	3:118,016,028	Cecum	*Candidatus Methanoplasma; Colidextribacter*	Brain	Trans	*ENSSSCG00000002990*	*CCNP*
3	3:118,016,028	Cecum	*Candidatus Methanoplasma; Colidextribacter*	Brain	Trans	*ENSSSCG00000006425*	*OR6K6*
3	3:118,016,028	Cecum	*Candidatus Methanoplasma; Colidextribacter*	Brain	Trans	*ENSSSCG00000031587*	–
3	3:118,016,028	Cecum	*Candidatus Methanoplasma; Colidextribacter*	Brain	Trans	*ENSSSCG00000039519*	–
3	3:118,016,028	Cecum	*Candidatus Methanoplasma; Colidextribacter*	Brain	Trans	*ENSSSCG00000037703*	–
3	3:79,943,654	Feces	*Candidatus Soleaferrea; dgA.11 gut group*	Brain	Trans	*ENSSSCG00000031587*	–
6	6:165,700,722	Cecum	*Monoglobus*	Liver	Cis	*ENSSSCG00000034035*	*TMEM69*
6	6:165,700,722	Cecum	*Monoglobus*	Muscle	Cis	*ENSSSCG00000034035*	*TMEM69*
7	7:32,914,122	Feces	*Helicobacter*	Brain	Trans	*ENSSSCG00000001504*	*WDR46*
7	7:32,914,122	Feces	*Helicobacter*	Brain	Trans	*ENSSSCG00000001535*	*TCP11*
7	7:32,914,122	Feces	*Helicobacter*	Liver	Cis	*ENSSSCG00000036178*	*CCDC167*
17	17:5,704,977	Feces	*Acutalibacter*	Brain	Trans	*ENSSSCG00000008052*	–
17	17:5,704,977	Feces	*Acutalibacter*	Muscle	Cis	*ENSSSCG00000006982*	*ZDHHC2*

Significant eQTL selected based on the criterion of presenting at least two significant associations in the genome-wide analyses. eQTL were classified as cis or trans according to their genomic position relative to the associated gene. Columns indicate chromosome (Chr), SNP identifier (chromosome:position), microbiota compartment (cecal or fecal), bacterial genus, tissue in which the eQTL was identified (brain, liver, or muscle), eQTL type, related gene information (Ensembl gene ID and gene symbol). A dash (–) indicates *loci* without annotated gene symbols in the current Ensembl release. When multiple bacterial genera are listed within a single row, they represent associations sharing the same SNP, tissue, microbiota compartment, and eQTL type. Multiple genes associated with the same eQTL are displayed in separate rows

Panels (A)–(H) represent specific eQTL–microbiota associations: (A) trans-eQTL at SSC17:5,704,977 in the brain associated with fecal *Acutalibacter* abundance; (B) cis-eQTL at SSC17:5,704,977 in the muscle associated with fecal *Acutalibacter* abundance; (C) cis-eQTL at SSC6:165,700,722 in the liver associated with cecal *Monoglobus* abundance; (D) cis-eQTL at SSC6:165,700,722 in the muscle associated with cecal *Monoglobus* abundance; (E) trans-eQTL at SSC7:32,914,122 in the brain associated with fecal *Helicobacter* abundance; (F) cis-eQTL at SSC7:32,914,122 in the liver associated with fecal *Helicobacter* abundance; (G) trans-eQTL at SSC3:118,016,028 in the brain associated with cecal *Candidatus Methanoplasma* abundance; and (H) trans-eQTL at SSC3:118,016,028 in the brain associated with cecal *Colidextribacter* abundance.

Annotation of these eQTL revealed that they regulate genes with established or putative roles in metabolic and immune-related pathways (Table 5). Notably, several regulatory variants identified here are not annotated in public databases, highlighting the discovery of novel regulatory *loci* in the porcine genome. The eQTL at SSC17:5,704,977 regulated distinct genes in different tissues, acting as a trans-eQTL for a novel gene (*ENSSSCG00000008052*) in the brain and as a cis-eQTL for *ZDHHC2* (ZDHHC palmitoyltransferase 2) in muscle. The cis-eQTL at SSC6:165,700,722 regulated the *TMEM69* gene (Transmembrane Protein 69) expressed in the liver and muscle. The eQTL at SSC7:32,914,122 regulated the *TCP11* gene (T-Complex 11) in the brain, acting as a trans-eQTL, regulated the *CCDC167* gene (Coiled-Coil Domain-Containing Protein 167) in the liver, acting as a cis-eQTL. Additional eQTL-regulated genes, including *CCNP* (Cyclin P), *OR6K6* (Olfactory Receptor Family 6 Subfamily K Member 6), and other novel gene (*ENSSSCG00000031587*), act as trans-eQTL in the brain.

Ortholog analyses of the novel genes revealed that only one gene (*ENSSSCG00000008052*) presented an annotated ortholog in mouse (*Abca17*), while no orthologs were identified in humans. The remaining novel genes did not show annotated orthologs in either species.

Together, these results highlight regulatory *loci* influencing gene expression across tissues that are also associated with variation in gut microbial genera.

## Discussion

### Overview of eQTL discovery across tissues

Within this multi-tissue framework, we observed that the brain exhibited the highest number of significant eQTL (cis- and trans-) and regulated genes, whereas liver and muscle, which showed considerably fewer associations. Nearly half of these eQTL were annotated as novel variants. Similar patterns have been reported in large-scale human multi-tissue eQTL analyses, where gene regulation is highly tissue-dependent and brain tissues exhibit increased regulatory complexity and distinct expression patterns (GTEx Consortium 2015, 2017).

This pattern may reflect the intricate molecular interplay along the gut–brain axis, in which microbial metabolites (such as SCFAs) and immune signals influence central gene expression. The enrichment of lipid metabolism and immune-related pathways aligns with evidence that the gut microbiota modulates brain function through metabolic and immunological routes (O’Riordan et al. 2022; Arneth 2025). In pigs, gut microbiota colonization has been shown to alter hypothalamic transcriptomic profiles and modulate energy metabolism, supporting the role of microbial signals in brain gene regulation and homeostasis (Qi et al. 2022).

Beyond its neural implications, the gut–brain axis interacts with peripheral metabolic organs such as the liver and skeletal muscle, forming an integrated network often referred to as the microbiota–gut–liver–brain axis (Mancin et al. 2023; Li et al. 2024; Lu et al. 2024a). Microbial metabolites, including SCFAs and bile acid derivatives, reach the liver via the portal vein and modulate lipid and immune pathways that communicate with the central nervous system (Cryan et al. 2019). Systemic inflammatory and metabolic signals arising from muscle may also influence brain function, contributing to the regulation of energy homeostasis and gene expression (Przewłócka et al. 2020). Together, these interactions support a model in which gene regulation across tissues reflects coordinated host–microbiota communication rather than isolated transcriptional events.

### eQTL–microbiota associations and key microbial taxa across gut compartments

We identified associations between bacterial genera across gut compartments and gene expression in multiple tissues, highlighting the multi-tissue nature of host–microbiota regulation. *Oscillospira* showed the highest number of associations, while *Candidatus Methanoplasma* was consistently detected across both cecal and fecal microbiota. Several genera were linked to eQTL from multiple tissues, suggesting coordinated regulatory patterns across tissues, including *loci* not previously implicated in host–microbiota interactions in pigs.

Our findings are consistent with recent studies linking host genetics to gut microbiota composition in pigs, which have identified genomic regions associated with the abundance of bacterial genera and production-related traits (Crespo-Piazuelo et al. 2019; Bergamaschi et al. 2020; Hu et al. 2024; Lu et al. 2024b). However, we also observed clear differences between cecal and fecal microbiota in their genetic associations. A higher number of significant associations was detected for the fecal microbiota, whereas the cecal microbiota captured a distinct subset of associations involving metabolically relevant taxa, indicating that different gut compartments may reflect complementary layers of host genetic regulation.

In this context, recent studies have highlighted how environmental and host factors, particularly diet, drive major shifts in microbial composition along the porcine gastrointestinal tract and modulate host metabolism, immunity, epigenetic regulation (Tang et al. 2025). In parallel, previous work has emphasized both the limited number of microbiota-GWAS in pigs and the translational relevance of the porcine model due to its genetic and functional similarities to humans (Huang and Chen 2023). Building on these findings, our results extend this framework by incorporating a host-genetic regulatory layer and integrating cecal and fecal microbiota with tissue-specific eQTL, capturing complementary aspects of host–microbiota crosstalk and suggesting that spatial heterogeneity along the gastrointestinal tract may be partly genetically mediated.

Earlier studies have primarily focused on fecal microbiota due to its ease of sampling, identifying genomic regions associated with microbial taxa abundance and, in some cases, with growth or carcass traits (Crespo-Piazuelo et al. 2019; Bergamaschi et al. 2020). However, fecal samples represent a distal and more transient snapshot of the gut ecosystem, which may capture a broader but less stable set of associations. In contrast, cecal microbiota are more stable and directly involved in fermentation processes, short-chain fatty acid production, host metabolic signaling, supporting their role in reflecting functionally relevant host–microbiota interactions.

This substantial overlap between cecal and fecal microbiota suggests that fecal samples capture a large proportion of the prevalent gut microbial community, although host genetic associations may remain compartment-specific. However, potential sources of variability, including spatial heterogeneity along the gastrointestinal tract, differences in sampling time points, and pre-slaughter conditions such as stress or fasting, should be considered when interpreting differences between sample types (Thomas et al. 2015; Kim et al. 2023; Ahn et al. 2023; Levitan et al. 2023).

Among these taxa, *Oscillospira* emerged as one of the most prominent taxa associated with host genetic regulation. It is a common member of the mammalian gut microbiota and has been associated with fermentative processes (Konikoff and Gophna 2016; de Jonge et al. 2022). Although it has been linked to both beneficial roles and certain conditions in humans (Konikoff and Gophna 2016; Yang et al. 2021), our results indicate that its abundance is strongly influenced by host genetic regulation across tissues, suggesting that host genetic variation contributes to shaping *Oscillospira* abundance beyond environmental effects.

Collectively, these results support the view that distinct gut compartments capture complementary layers of host–microbiota interactions. Among the genera identified, specific taxa such as *Oscillospira* and *Candidatus Methanoplasma* emerged as particularly informative examples, providing further insight into host genetic influences on microbial composition. By integrating cecal and fecal microbiota within an eQTL-informed framework, our study provides insight into potential mechanisms through which host gene regulation may influence microbial communities along the gastrointestinal tract, with implications for metabolic and immune-related traits in pigs (Hu et al. 2024; Lu et al. 2024b).

The biological relevance of *Oscillospira* may be linked to its metabolic capabilities, including the metabolism of host-derived glycans and the production of SCFAs such as butyrate (Konikoff and Gophna 2016; Yang et al. 2021). Despite these associations, its physiological properties and ecological role remain poorly understood, as pure cultures have not yet been obtained, making it an enigmatic member of the gut microbiota (Konikoff and Gophna 2016). Across animal species, this genus has been associated with leanness, lipid metabolism, and improved metabolic performance (Miragoli et al. 2021; Liu et al. 2023), highlighting its potential relevance for host physiology. In our study, its association with eQTL detected in metabolically relevant tissues suggests that host genetic regulation may contribute to shaping its abundance. These findings support a model in which host-controlled metabolic and immune pathways may influence microbial colonization, positioning *Oscillospira* as a promising target for host–microbiota integrative studies.

Beyond confirming the role of bacterial taxa such as *Oscillospira*, our analysis also identified consistent associations involving the archaeal genus *Candidatus Methanoplasma*, a functionally distinct group with important implications for host physiology (Mi et al. 2019; Gu et al. 2021; Lendormi et al. 2022). This genus is commonly detected in the gastrointestinal tract of animals and plays a role in methane production through methylotrophic methanogenesis (Vendruscolo et al. 2020; Pyzik et al. 2018). Although pigs are monogastric, microbial fermentation in the large intestine supports archaeal methanogenesis at low but measurable levels, highlighting its potential relevance for host metabolism and environmental impact. In our study, *Candidatus Methanoplasma* showed consistent associations across both cecal and fecal microbiota, suggesting that, for this genus, fecal samples may capture a representative signal of host–microbiota interactions. These findings indicate that host genetic regulation may influence microbial pathways related to methane production, supporting the potential of microbiota-informed strategies for improving sustainability in livestock systems.

While these findings highlight compartment-specific and microbial-level associations, additional patterns emerge when considering regulatory effects across host tissues.

### Tissue-specific and cross-tissue regulatory effects

Beyond characterizing the influence of gene expression on microbial genera, our results revealed host genetic *loci* associated with microbial abundance across multiple tissues, extending regulatory effects beyond local gut interactions. *Helicobacter* provides an example of this pattern, showing overlapping associations with eQTL from both the brain and liver. Members of this genus have been widely associated with gastrointestinal disorders and, in some cases, with zoonotic risk (Gorlé et al. 2018; Stair et al. 2023; García-Ferrús et al. 2025; Li et al. 2025; Hojo et al. 2025). Moreover, *Helicobacter* infections have been linked to neurodegenerative conditions such as Parkinson’s disease, highlighting the relevance of the gut–brain axis (Kountouras et al. 2014; Noori et al. 2023; Wang et al. 2024). Although our analysis was conducted at the genus level, the observed associations suggest that microbial abundance may be influenced by systemic host genetic variation. Together, these results support a model of bidirectional communication along gut–organ axes, in which central and peripheral tissues jointly contribute to shaping the gut microbiota.

In addition to tissue-specific regulatory patterns, several bacterial genera associated with eQTL share common functional roles within the gut ecosystem. Genera such as *Monoglobus, Acutalibacter, Anaerostipes, Oscillospira*, and *Roseburia* are linked to the production of SCFAs, particularly butyrate, to the modulation of host immune and metabolic pathways (Louis and Flint 2017; Wang et al. 2021; Martín et al. 2023). SCFA-producing taxa have been shown to influence gene expression and metabolic signaling in central and peripheral tissues, including the brain, liver, and skeletal muscle, through gut–organ communication axes (Miragoli et al. 2021; O’Riordan et al. 2022; Qi et al. 2022; Arneth 2025). These results indicate that host genetic variation may contribute to coordinating microbial functions across tissues.

### SCFA-producing genera and lipid–immune pathways

Several bacterial genera associated with eQTL in this study share key functional roles within the gut ecosystem, particularly in the production of SCFAs and the modulation of host immune and metabolic pathways. Genera such as *Monoglobus, Acutalibacter, Anaerostipes, Oscillospira*, and *Roseburia* are phylogenetically diverse but functionally convergent, with established roles in butyrate production and host metabolic regulation.

In pigs, genera such as *Roseburia* have been associated with improved intestinal health, anti-inflammatory effects, and lipid metabolism, linking microbial activity to energy balance and fat deposition (Yang et al. 2018; Bergamaschi et al. 2020; Chen et al. 2023; Ortiz Sanjuán et al. 2024). Similarly, *Anaerostipes* is considered a butyrate-producing genus in other hosts and has been detected in pigs, with reported correlations between its abundance and fecal butyrate levels, although direct experimental confirmation in swine remains limited (Miragoli et al. 2021; Holman et al. 2022; Eudy et al. 2023). Microbiota-derived metabolites are also known to influence muscle physiology and growth, linking gut microbial activity to host metabolic performance (Qi et al. 2021; Mancin et al. 2023). In this study, *Roseburia* and *Anaerostipes* were associated with eQTL detected in both brain and muscle tissues, suggesting a host genetic link between microbial SCFA production and systemic metabolic regulation.

The genus *Acutalibacter* was associated with eQTL in both brain and muscle tissues, a pattern similar to other butyrate-producing genera such as *Roseburia* and *Anaerostipes*. Although it has not been widely reported in swine gut microbiota studies, members of its taxonomic family have been detected at low abundance in porcine metagenomic analyses, suggesting that this genus may belong to the rare biosphere and remain underrepresented due to limitations in current reference databases (Zhou et al. 2020; Scicchitano et al. 2024). Despite the limited evidence in pigs, species described in other hosts are known butyrate producers involved in carbohydrate fermentation and immune modulation (Lagkouvardos et al. 2016; He et al. 2024). Together, these findings suggest that *Acutalibacter* or related taxa may contribute to SCFA metabolism and host–microbiota interactions in the porcine gut, although further functional characterization is required.

We observed associations between *Monoglobus* and eQTL in both liver and muscle tissues. Originally described in the human gut as a specialized pectin-degrading bacterium (*Monoglobus pectinilyticus*), this genus plays an important role in carbohydrate fermentation and has been detected across different mammalian microbiota (Kim et al. 2017; 2019). In humans, *Monoglobus* has been associated with favorable health outcomes and immune-related pathways (Chen et al. 2023; Moriki et al. 2024; Turpin et al. 2025), whereas evidence in swine remains limited, with a single study reporting correlations with immune traits (Yuan et al. 2022). In our study, the presence of cross-tissue associations suggests that host genetic regulation may influence the abundance of this genus, highlighting *Monoglobus* as a promising target for future functional and mechanistic investigations.

Additional genera identified in the eQTL–microbiota association analysis include several SCFA-producing taxa, such as *Faecalibacterium, Coprococcus*, *Caproiciproducens,* which are associated with the production of different short-chain fatty acids, including butyrate, acetate, and caproate (Louis and Flint 2017; Wang et al. 2021; Martín et al. 2023; Notting et al. 2023; Liu et al. 2024). These metabolites act as important mediators of host lipid metabolism, immune regulation, intestinal health. Other genera, including *Fournierella, Rikenellaceae RC9 gut group, Colidextribacter*, have also been associated with lipid profiles and inflammatory states in recent studies (Mohr et al. 2024; Sebastià et al. 2024; Turpin et al. 2025). In addition, *Anaerovibrio* contributes to the degradation of triglycerides and long-chain fatty acids, linking microbial activity to energy metabolism and hepatic function (Privé et al. 2013). Together, these findings suggest that host genetic variants regulating these taxa may influence the interplay between microbial metabolite production and systemic metabolic and inflammatory processes.

### Biological relevance of key regulatory genes

Our analyses identified five key eQTL associated with multiple microbiota traits, collectively regulating 11 genes, including three novel loci. Among the annotated genes, the trans-eQTL SSC17:5,704,977 was associated with *ZDHHC2*, which encodes a palmitoyltransferase responsible for protein palmitoylation. This post-translational modification plays an important role in neuronal plasticity and synaptic function, as well as in immune signaling by modulating receptors and inflammatory pathways (Yokoi et al. 2012; Zhang et al. 2021; Sun et al. 2023; Sheng et al. 2025).

Additional genes regulated by these eQTL have been associated with cell proliferation and tumor-related pathways, including *TCP11, CCDC167, CCNP*, *OR6K6* (Kalra et al. 2020; Chen et al. 2021; Sánchez-Botet et al. 2021; Wang et al. 2023b, a; Naressi et al. 2025). Although these genes have been primarily studied in the context of oncogenic processes, their regulation in metabolically relevant tissues such as liver and muscle suggests broader roles in immune and metabolic pathways. These findings suggest that genes traditionally associated with tumor-related pathways may also contribute to broader immunometabolic processes.

Among all genes identified, only *TMEM69* was regulated by the same eQTL across more than one tissue, specifically liver and muscle. This overlap is particularly relevant given the complementary roles of these organs in energy homeostasis and immunometabolic regulation, suggesting that *TMEM69* may be involved in cross-tissue signaling linking host genetic regulation to microbial modulation. *TMEM69* encodes a predicted multi-pass transmembrane protein with broad tissue expression (Cunningham et al. 2022). In pigs, Steibel et al. (2011) reported associations between *TMEM69* expression and *loci* linked to meat quality and metabolic traits, supporting its potential role in tissue-specific transcriptional regulation and energy metabolism. The consistent detection of *TMEM69*-associated eQTL across metabolically active tissues further supports its involvement in shared regulatory processes across tissues.

Among the regulatory genes highlighted in this study, *ENSSSCG00000050714* emerged as a biologically relevant candidate due to convergent evidence from independent transcriptomic analyses. In our dataset, this gene was regulated by a cis-eQTL in the liver, indicating local genetic variation influencing its hepatic expression. Supporting this observation, Fanalli et al. (2024) identified *ENSSSCG00000050714* as a hub gene in a liver co-expression network associated with the Darkolivegreen module and the aspartate aminotransferase (AST) phenotype in pigs fed a soybean oil diet. Given that AST levels are widely used as indicators of hepatic metabolic activity and cellular integrity (Shi et al. 2022; Wang et al. 2025b), these findings suggest that genetic variation near *ENSSSCG00000050714* may contribute to transcriptional regulation linked to liver function and metabolic efficiency.

Among the novel genes investigated, only *ENSSSCG00000008052* presented an annotated ortholog in mouse (*Abca17*), while no orthologs were identified in humans. The identified mouse ortholog *Abca17* belongs to the ATP-binding cassette (ABC) transporter family, which is involved in lipid transport and metabolism. This functional context is particularly relevant given the established links between host lipid metabolism and gut microbiota composition, suggesting that regulatory variants affecting this gene may contribute to host–microbiome interactions (Kotlyarov and Kotlyarova 2021; Tarling et al. 2013). In addition, some of the novel genes identified in this study did not show annotated orthologs in either human or mouse. This may reflect species-specific regulatory elements or currently uncharacterized genomic regions in pigs, which limits functional inference but highlights the need for further annotation and functional investigation in livestock genomes.

Together, these findings highlight the biological relevance of tissue-specific and cross-tissue regulatory variation in shaping host molecular phenotypes. By integrating host genomics and microbiota data, this study supports the presence of coordinated genetic and microbial mechanisms underlying metabolic and immune processes, offering a systems-level perspective on gene–environment interactions in pigs.

## Limitations and future directions

This study also has some limitations. First, the relatively small sample size, particularly for the cecal microbiota dataset due to sample losses during data generation and quality control, may have reduced the ability to detect regulatory associations with small effect sizes, particularly trans-eQTL and cross-tissue host–microbiota associations under a substantial multiple testing burden. Because trans-eQTL generally require larger sample sizes to achieve adequate statistical power under stringent genome-wide multiple testing correction, additional biologically relevant associations may have remained undetected. Nevertheless, the identification of significant associations after stringent quality control and multiple-testing correction supports the robustness of the reported findings. Future studies with larger sample sizes will be important for validating these associations and identifying additional host–microbiota regulatory loci. In addition, the use of RNA-seq–derived SNPs restricts variant detection to expressed regions, potentially limiting the identification of regulatory variants located in non-coding or distal regions, especially for trans-eQTL. However, the integration of SNP panel data may have minimized this effect. Although the diet effect was accounted for in the statistical models, there could be diet-by-genotype (G × E) interactions that should be further investigated when larger datasets become available. Furthermore, pen-specific environmental variation was not modeled separately and therefore cannot be completely excluded as a source of residual variation. Future studies including larger sample sizes and using mixed-effect models will be important to further evaluate pen-level effects on host–microbiota associations. Despite these limitations, large-scale multi-omics datasets integrating host genetics, gene expression, and gut microbiota in pigs remain relatively scarce. Therefore, the biological coherence of the candidate genes and microbial taxa identified in this study supports the relevance of reporting these findings as an exploratory resource to guide future studies and generate new hypotheses regarding host–microbiota interactions.

Nevertheless, this study was conceived as an exploratory integrative analysis and, to the best of our knowledge, no previous work has jointly evaluated multi-tissue eQTL and bacterial genera at the ASV level in pigs. Despite these limitations, our results provide novel biological insights by demonstrating that host genetic regulation of gene expression is associated with microbial composition across multiple tissues, supporting the relevance of eQTL-informed frameworks for investigating host–microbiota interactions. The application of stringent quality control procedures, FDR correction, linear mixed models accounting for population structure and relevant covariates further strengthens the robustness of the analyses.

The use of immunocastrated males, reflecting standard commercial production practices, contributed to greater uniformity in management and physiological conditions across animals. However, this endocrine status may influence host metabolism and host–microbiota interactions. Therefore, the findings should be interpreted with caution when extrapolated to females or intact males, which present distinct hormonal profiles.

The integration of data from multiple tissues (brain, liver, and muscle) enabled the identification of consistent regulatory patterns, strengthening the biological interpretation of the findings and providing a foundation for future hypothesis-driven studies. Further integration of additional omics layers, such as metabolomics, metatranscriptomics, and proteomics, will be important to validate the associations observed here and to elucidate the mechanistic links between host genetics, gene expression, the microbiota. Experimental and longitudinal designs, together with functional validation approaches, are expected to help clarify causal relationships underlying host–microbiota interactions. Ultimately, these efforts will advance a more comprehensive understanding of the host–microbiota interface and its implications for animal physiology, health, and production traits.

## Conclusions

This study provides an integrative framework linking host genomic regulation, tissue-specific gene expression, and gut microbial composition in pigs. By coupling multi-tissue eQTL with microbiota-wide association signals, we identified genetic variants and microbial taxa jointly involved in metabolic and immune pathways, highlighting cross-tissue regulatory mechanisms that underlie host–microbiota interactions. The detection of key bacterial genera such as *Oscillospira, Candidatus Methanoplasma, Roseburia, and Anaerostipes*, along with eQTL-regulated genes such as *ZDHHC2* and *TMEM69*, reinforces the interconnected nature of lipid metabolism, immunity, and microbial modulation in metabolically active tissues. Collectively, these findings deepen the understanding of the molecular architecture governing host–microbiota relationships, with implications for improving metabolic efficiency, health, and environmental sustainability in livestock production.

## Supplementary Information

Below is the link to the electronic supplementary material.

Supplementary file1 (PDF 42447 KB)

Supplementary file2 (PDF 13763 KB)

Supplementary file3 (PDF 38291 KB)

Supplementary file4 (XLSX 194 KB)

Supplementary file5 (XLSX 18 KB)

Supplementary file6 (XLSX 490 KB)

Supplementary file7 (XLSX 593 KB)

Supplementary file8 (XLSX 173 KB)

Supplementary file9 (XLSX 20 KB)

Supplementary file10 (XLSX 26 KB)

Supplementary file11 (XLSX 23 KB)

Supplementary file12 (XLSX 61 KB)

Supplementary file13 (XLSX 16 KB)

## Data Availability

The datasets supporting the conclusions of this article are included within the article and its supplementary material. The RNA-seq datasets analyzed in this study are publicly available in the European Nucleotide Archive (ENA; EMBL-EBI) under accession numbers PRJEB52665 (brain tissue), PRJEB50513 (liver tissue), and PRJEB52629 (skeletal muscle tissue). The cecal and fecal microbiota sequencing data are also publicly available in the European Nucleotide Archive (ENA; EMBL-EBI) under accession number PRJEB105071. Other datasets generated and/or analyzed during the current study are available from the corresponding author upon reasonable request.
