# Supplementary material for: Associations of host gene expression in the brain, liver, muscle with fecal and cecal microbiota composition in pigs
Source: Funct Integr Genomics. 2026 Aug 15;26(1):226. doi: 10.1007/s10142-026-02009-5 (PMC13477493; doi:10.1007/s10142-026-02009-5)

Eubacterium_eligens_group

Cecum | Brain | . = 0.995

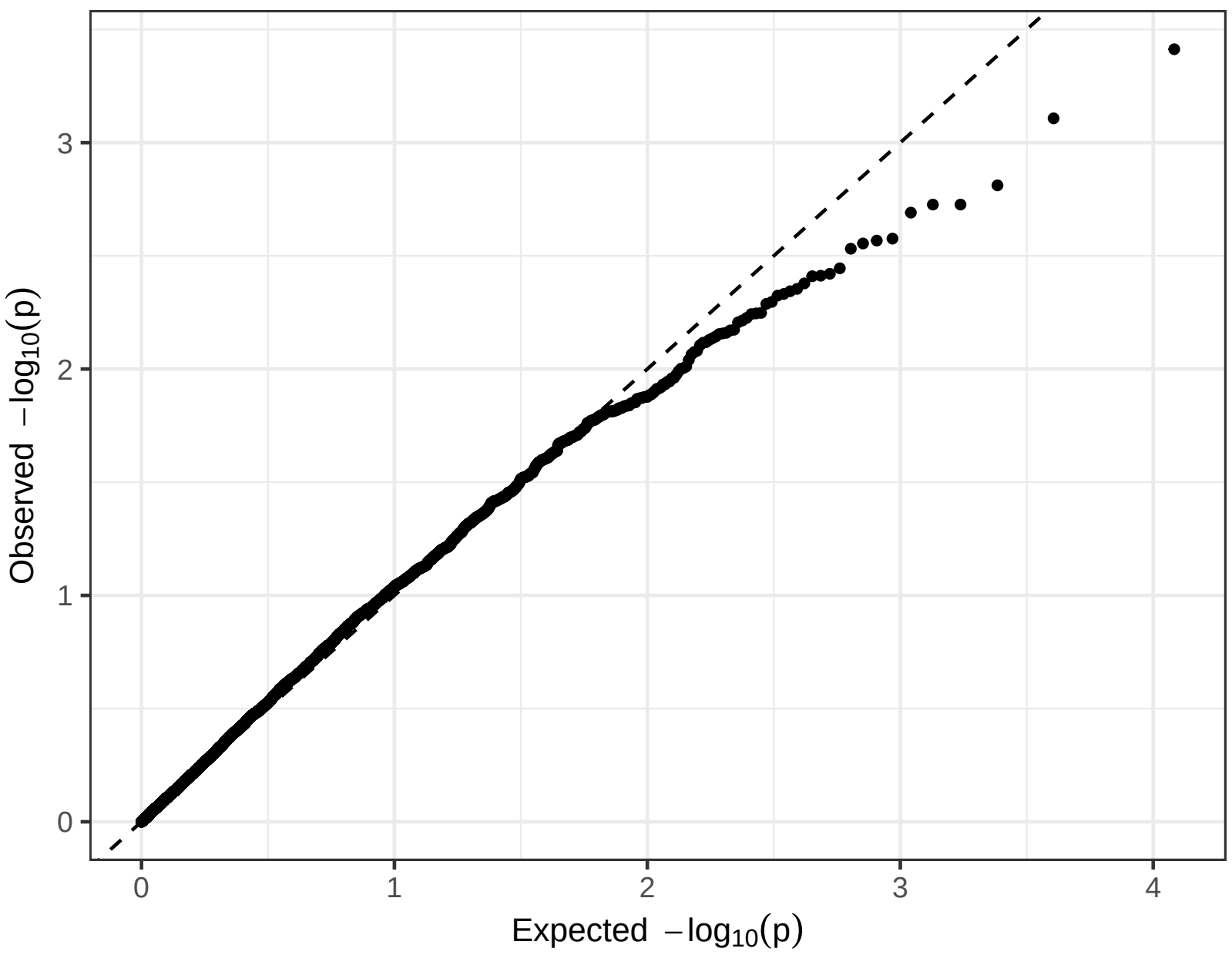

Adlercreutzia

Cecum | Brain | . = 0.875

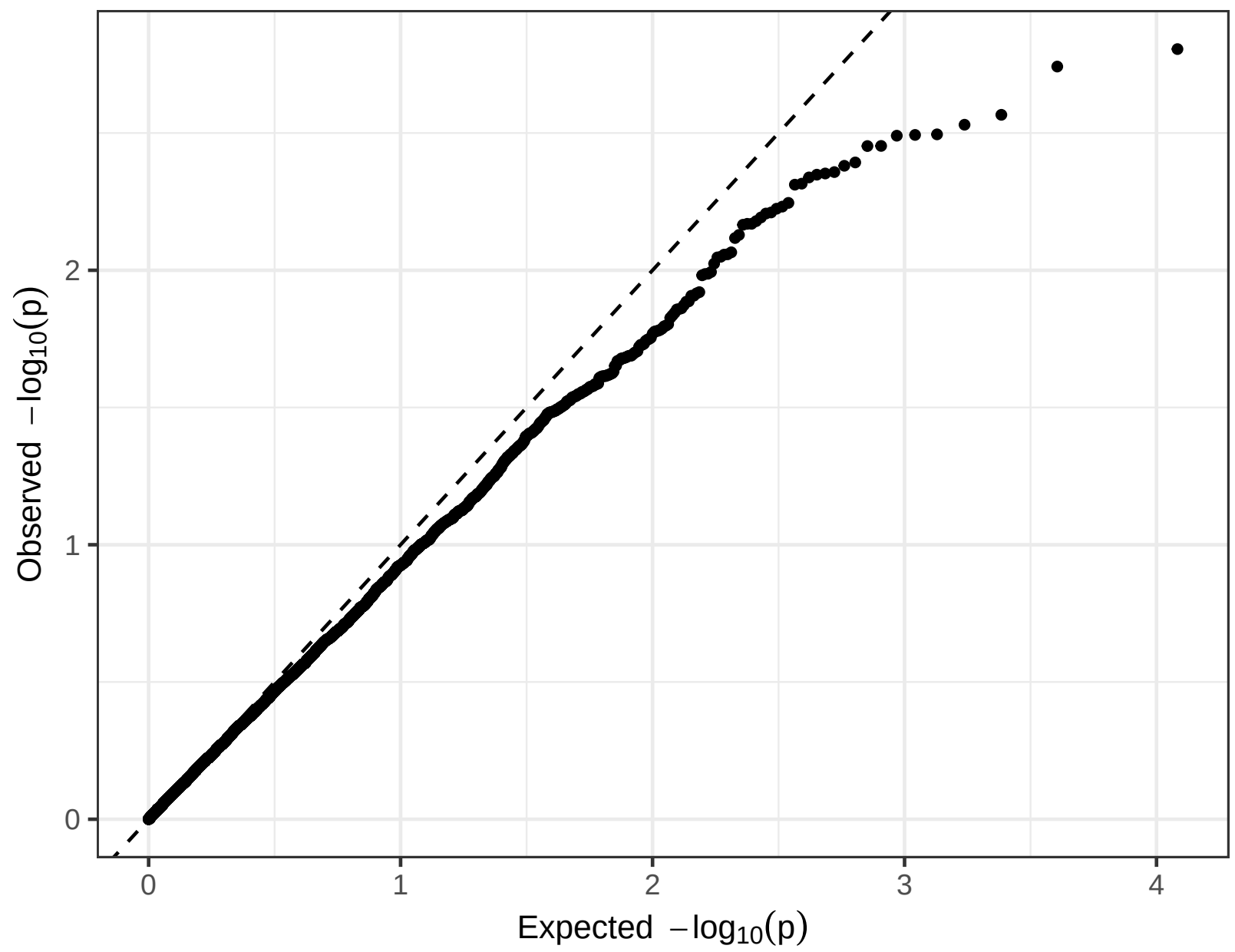

Megasphaera

Cecum | Brain | . = 0.895

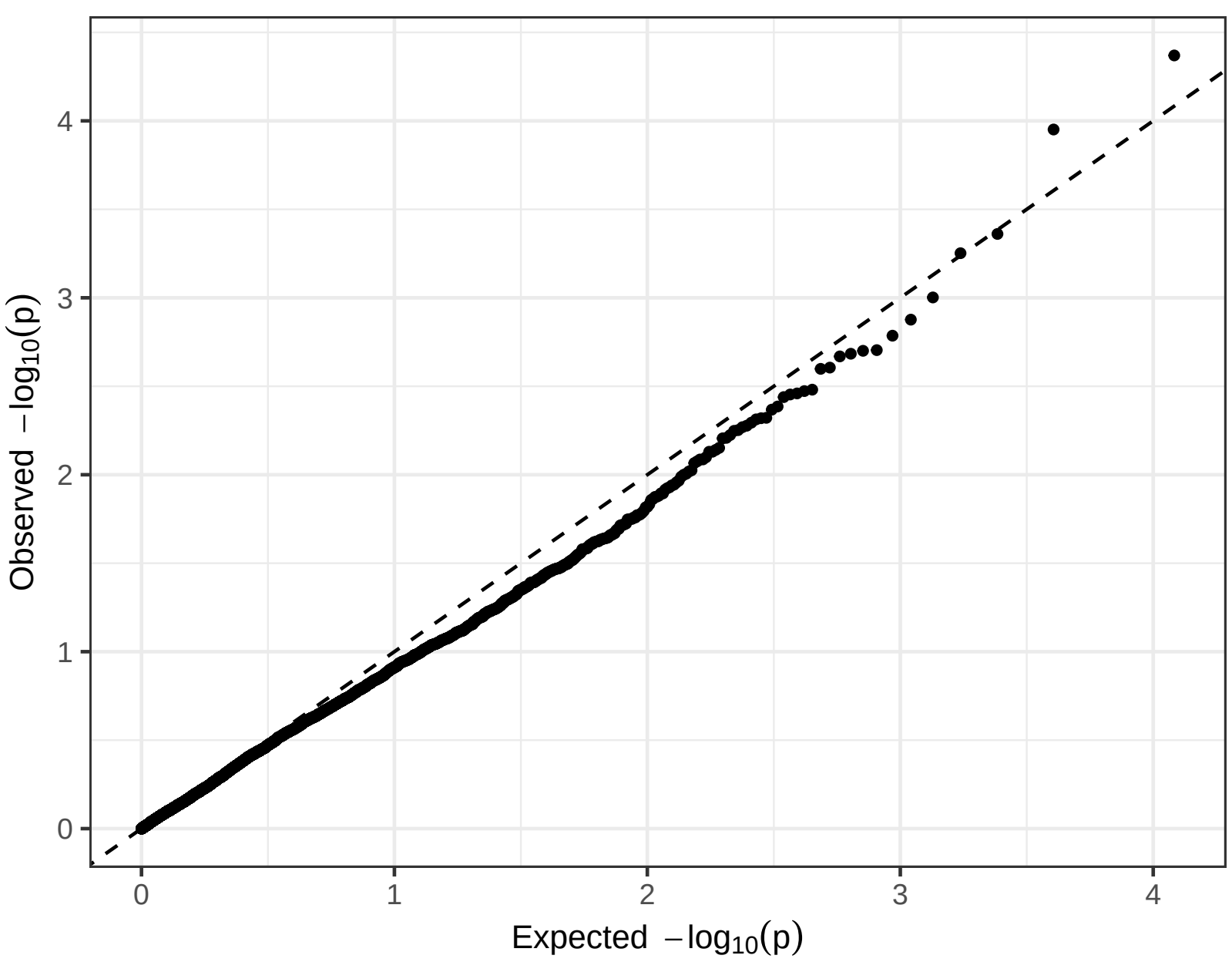

Metamycoplasma

Cecum | Brain | . = 0.917

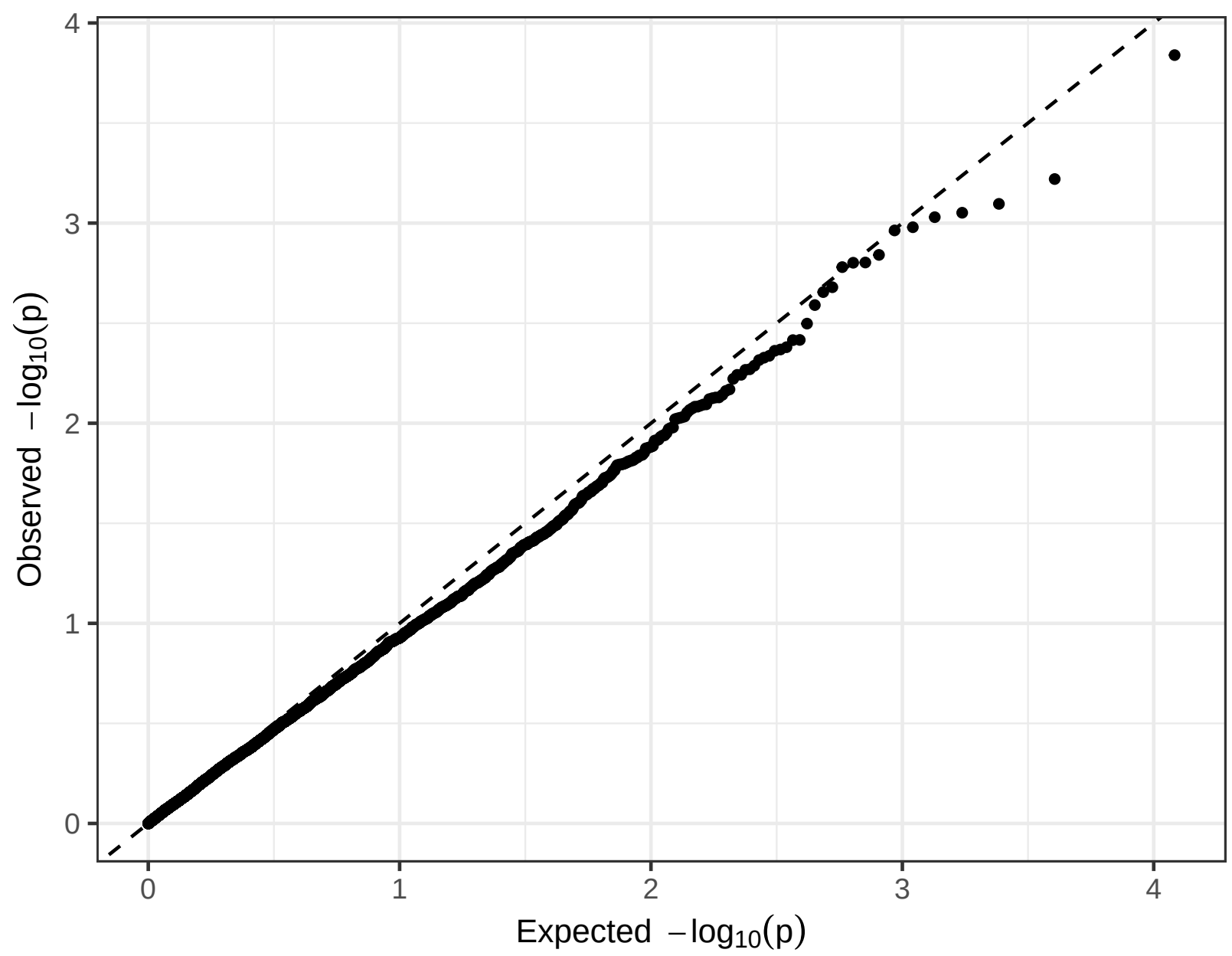

Methanobrevibacter

Cecum | Brain | . = 1.007

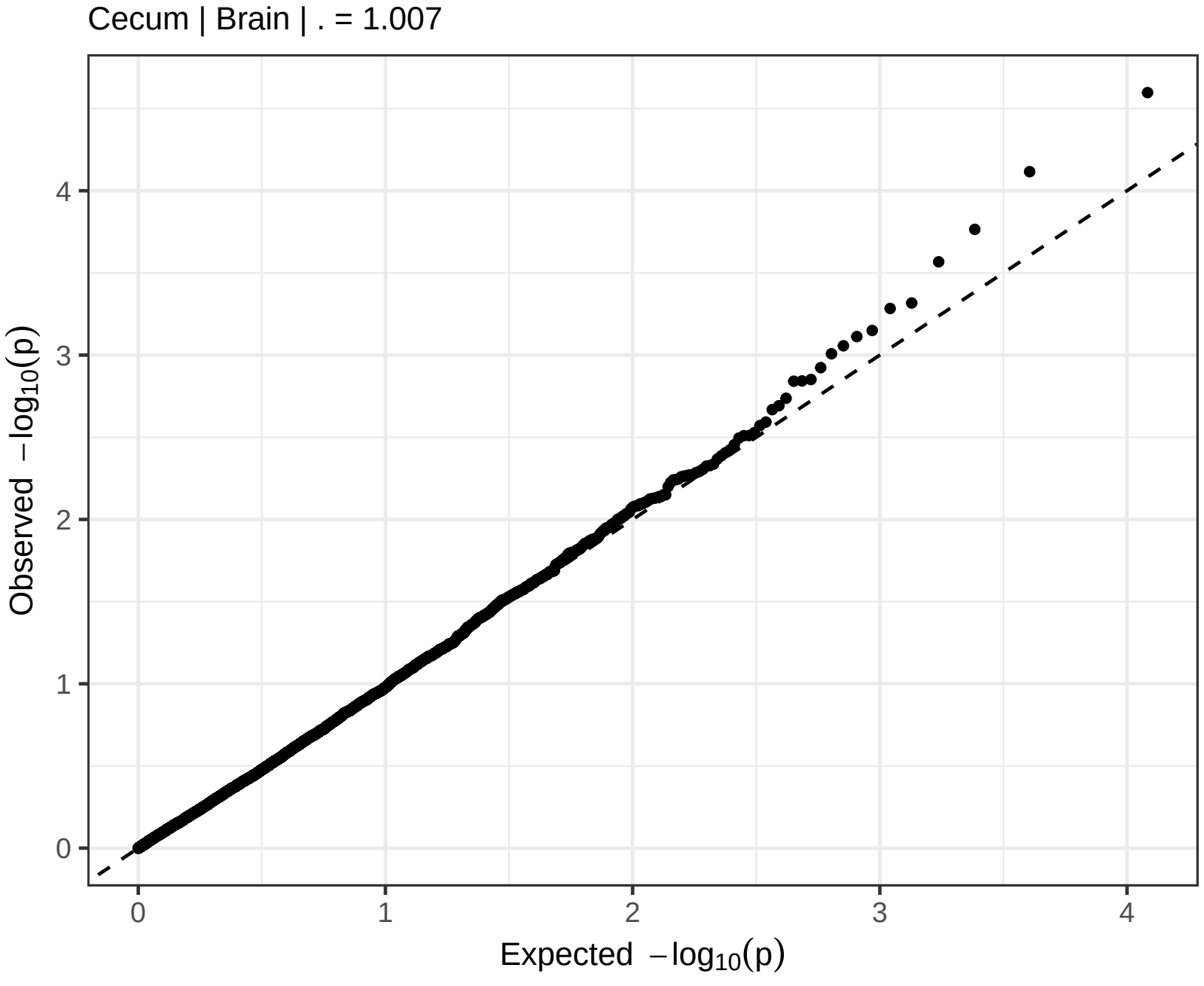

Mogibacterium

Cecum | Brain | . = 1.007

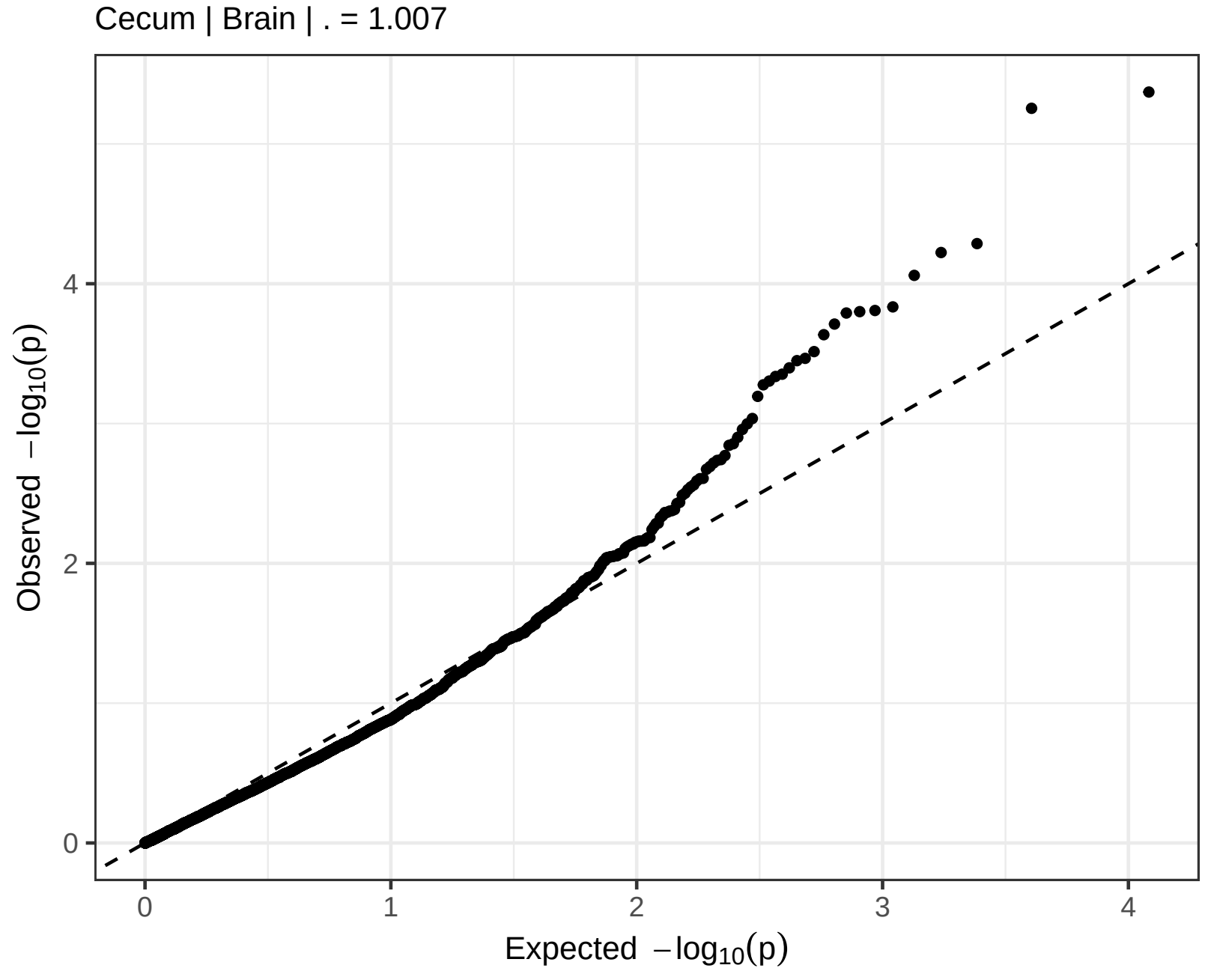

Monoglobus

Cecum | Brain | . = 1.025

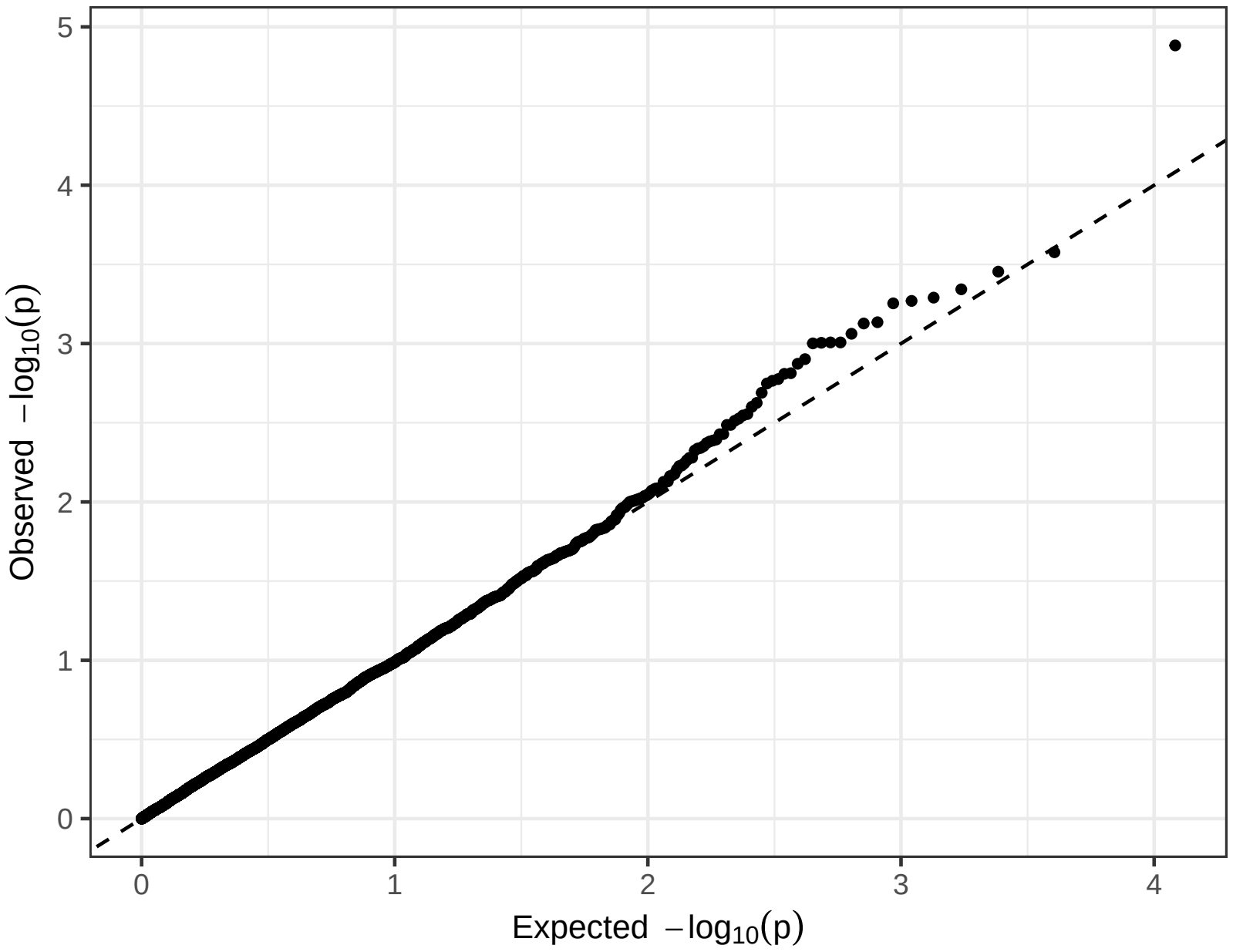

Negativibacillus

Cecum | Brain | . = 0.898

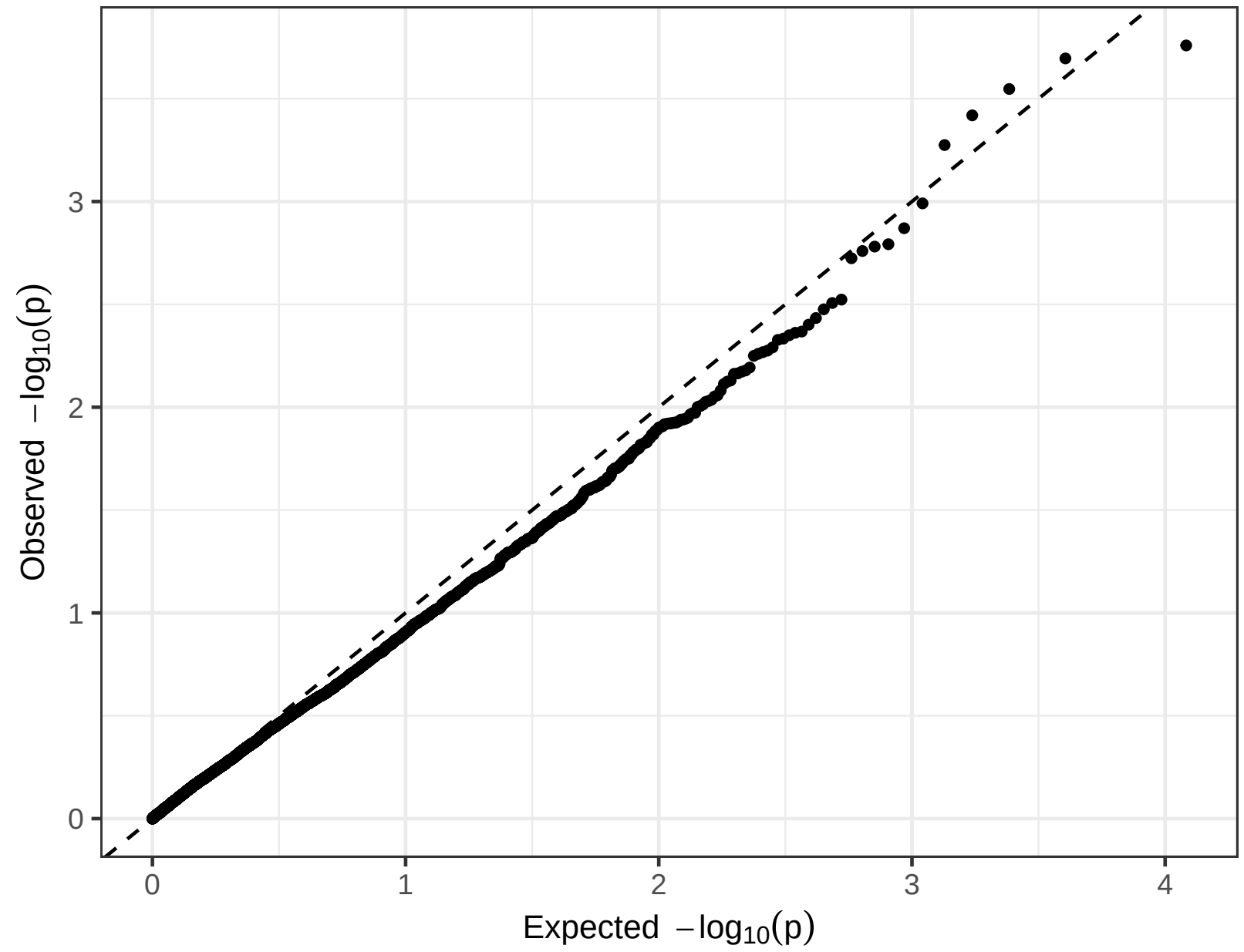

NK4A214_group

Cecum | Brain | . = 0.858

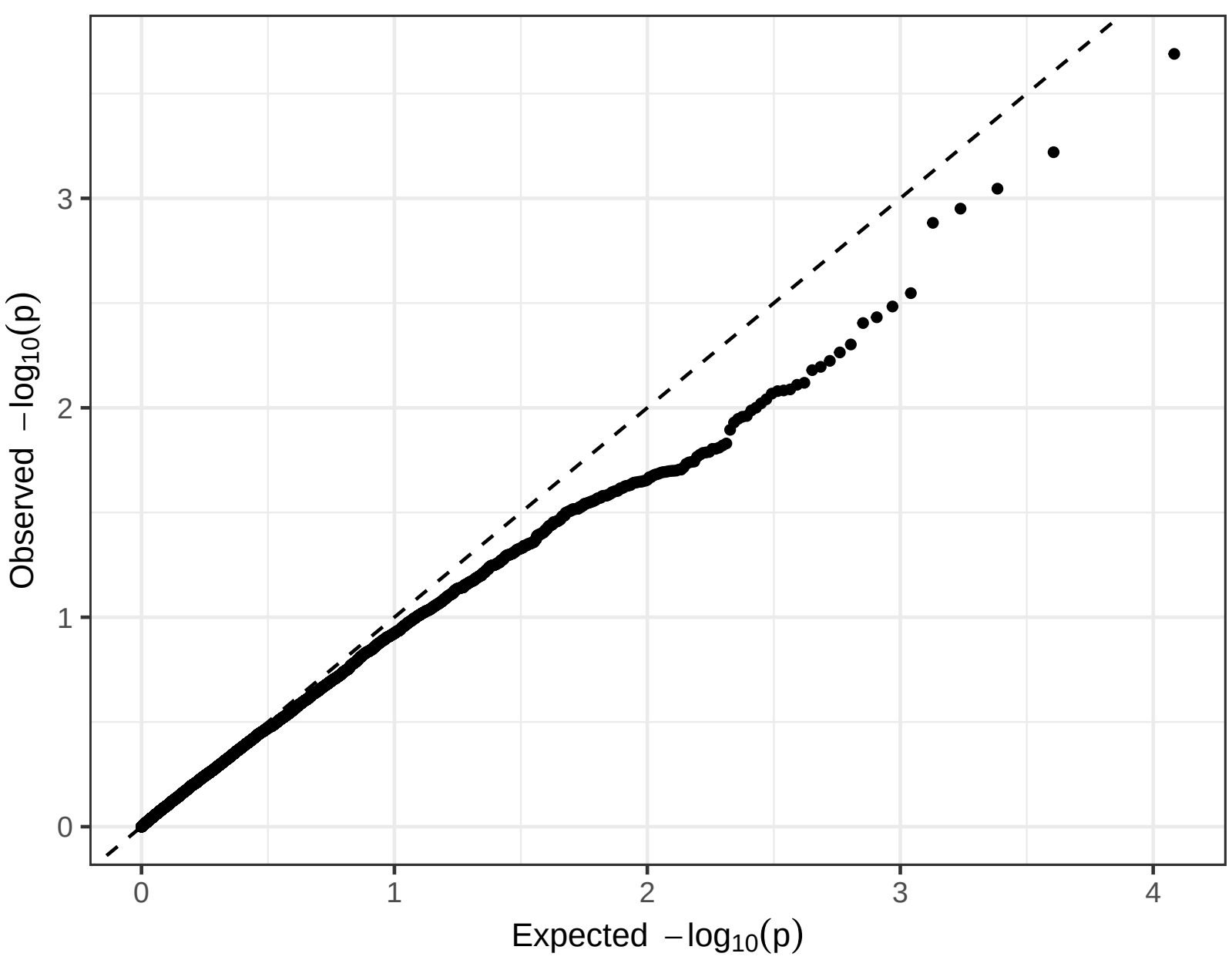

Oribacterium

Cecum | Brain | . = 0.874

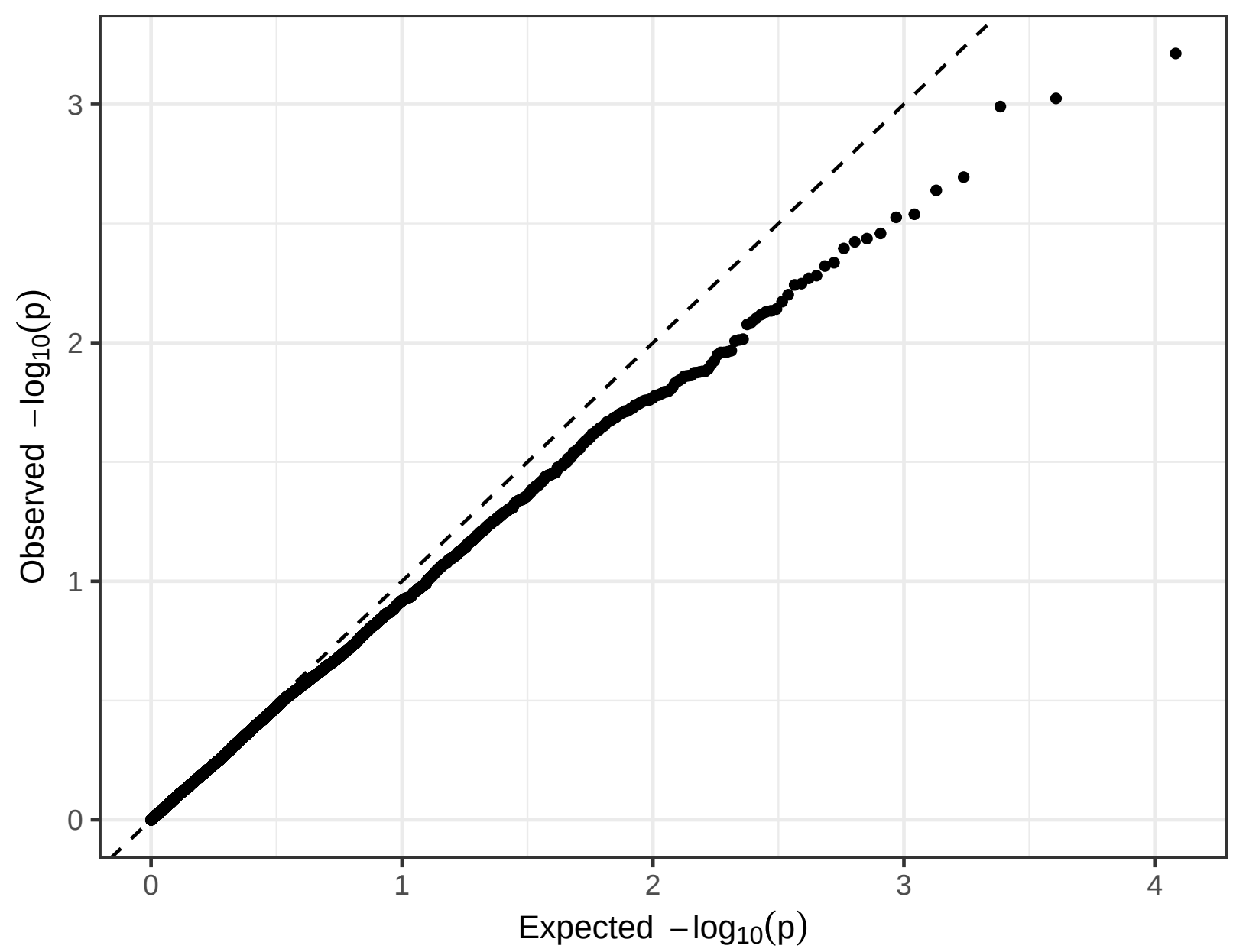

Oscillibacter

Cecum | Brain | . = 0.869

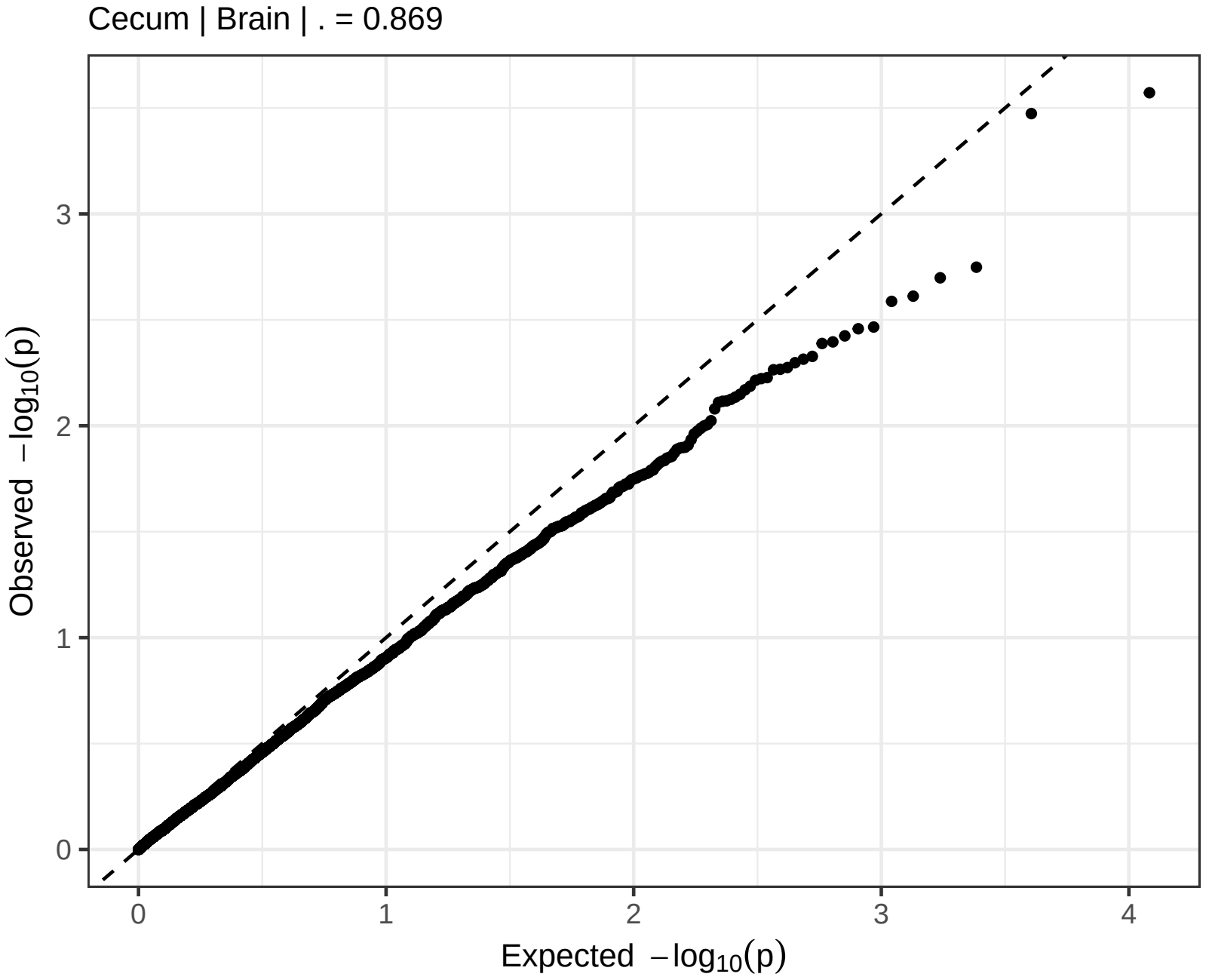

Oscillospira

Cecum | Brain | . = 0.904

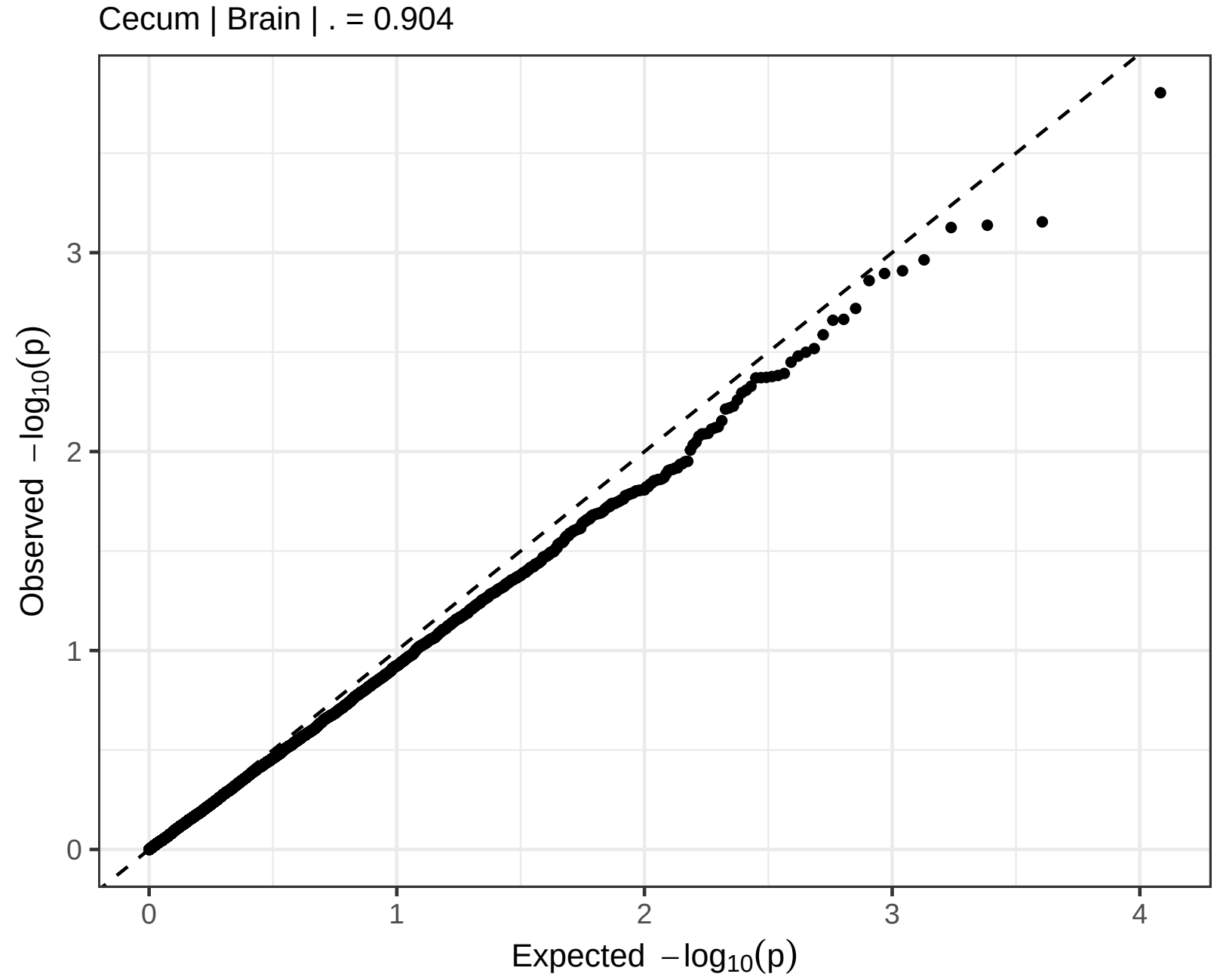

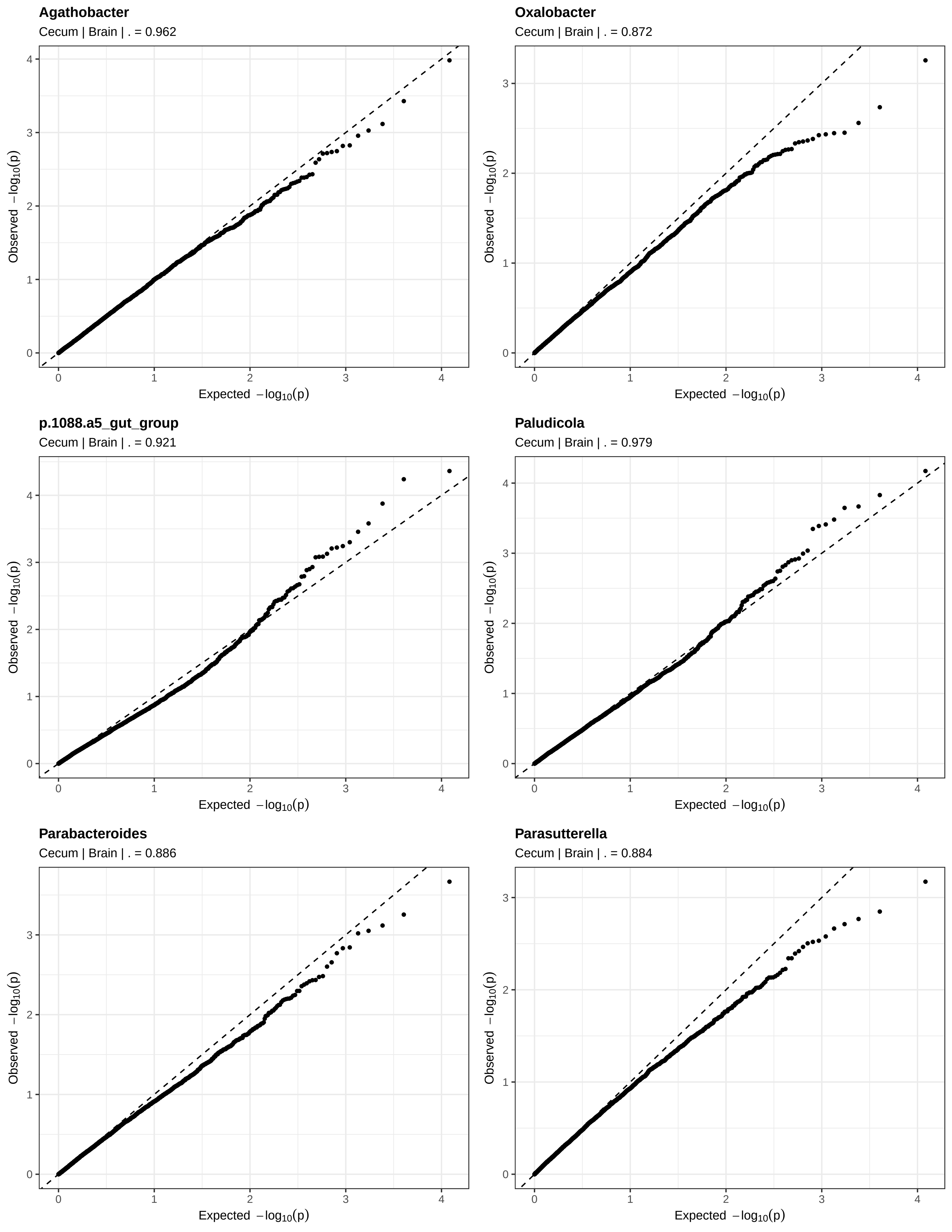
Agathobacter
Cecum | Brain | . = 0.962
Oxalobacter
Cecum | Brain | . = 0.872
p.1088.a5_gut_group
Cecum | Brain | . = 0.921
Paludicola
Cecum | Brain | . = 0.979
Parabacteroides
Cecum | Brain | . = 0.886
Parasutterella
Cecum | Brain | . = 0.884
Observed $-\log_{10}(p)$
Expected $-\log_{10}(p)$
0
1
2
3
4

Peptococcus

Cecum | Brain | . = 0.866

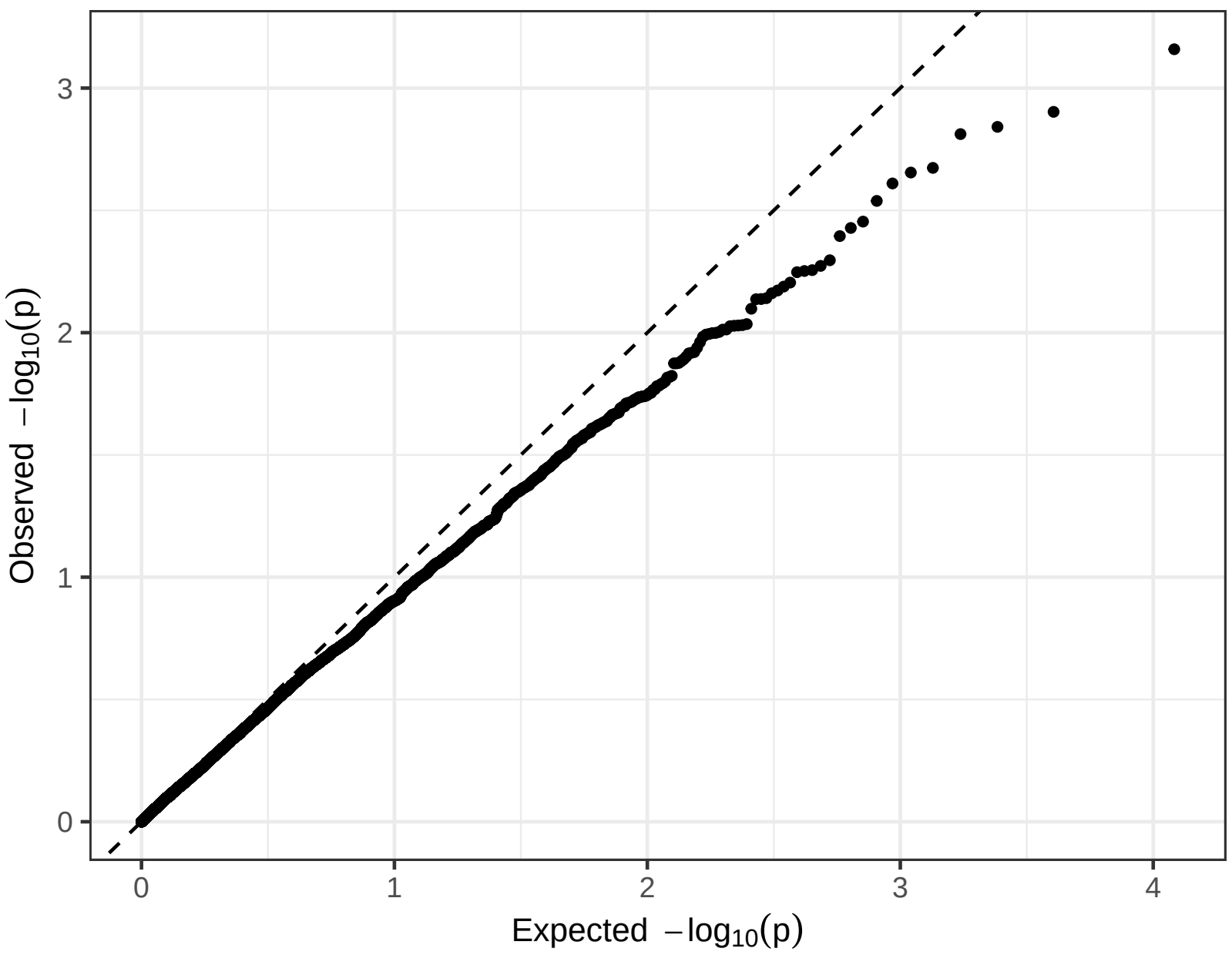

Phascolarctobacterium

Cecum | Brain | . = 0.906

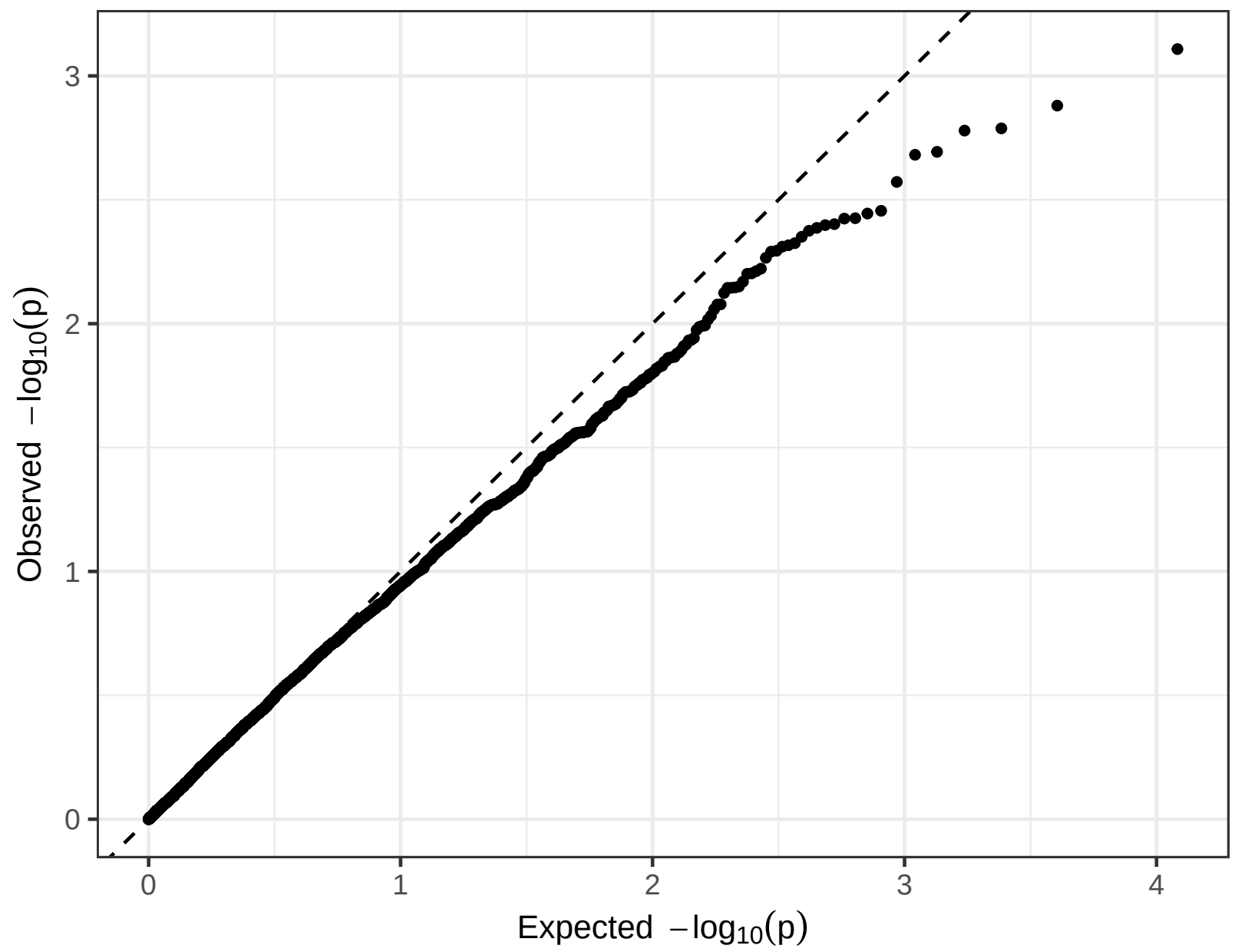

Prevotellaceae_NK3B31_group

Cecum | Brain | . = 0.959

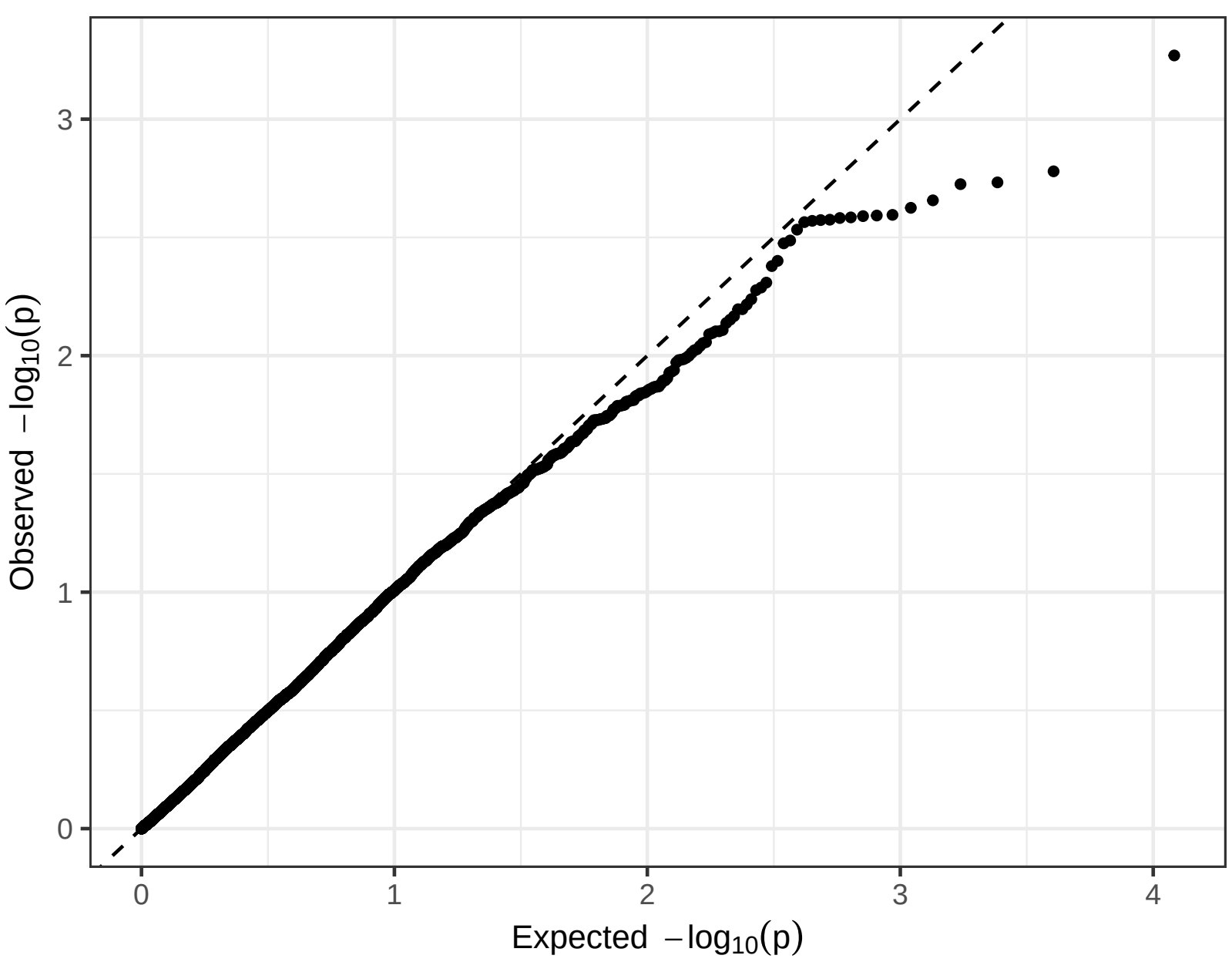

Prevotellaceae_UCG.001

Cecum | Brain | . = 0.859

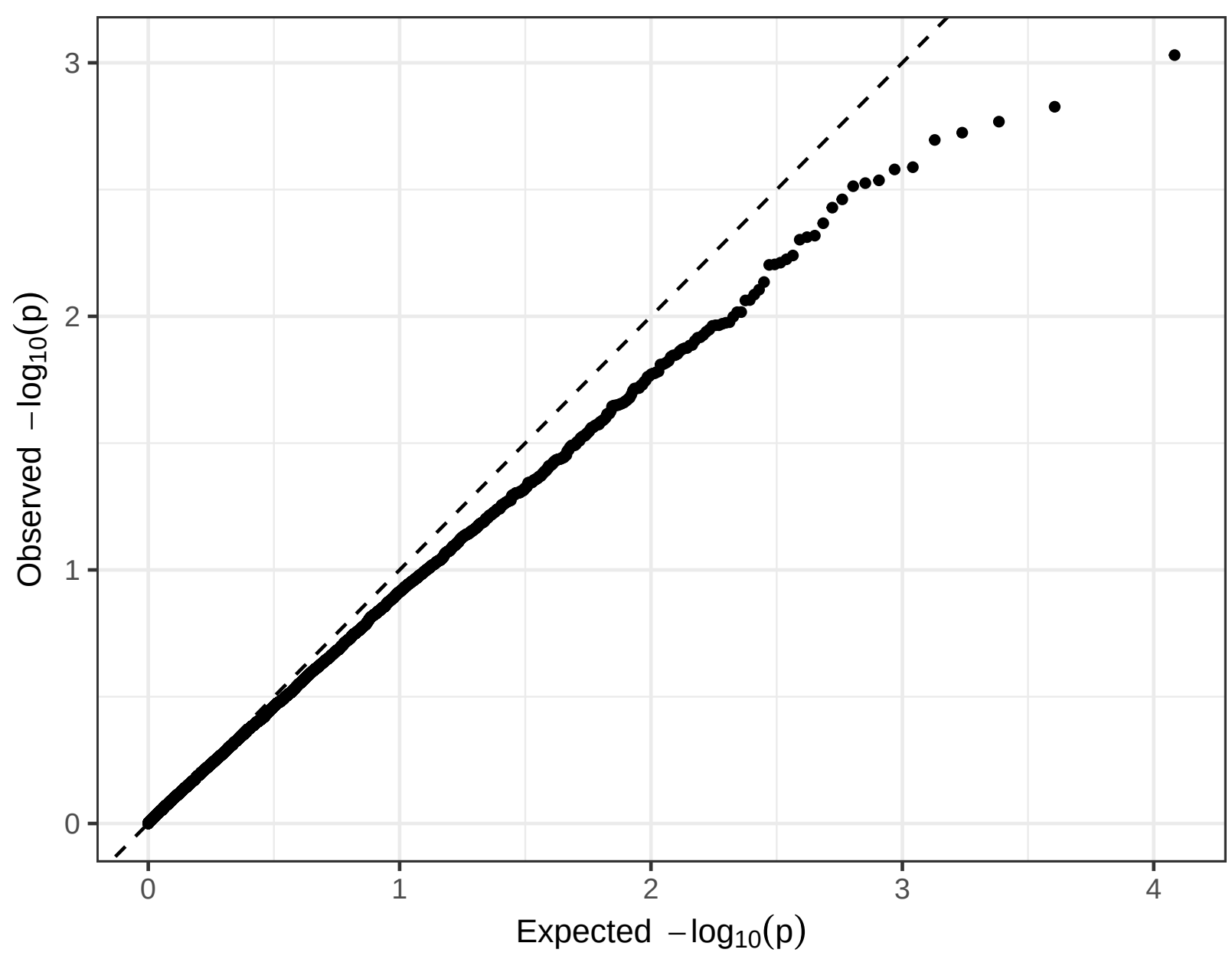

Prevotellaceae_UCG.003

Cecum | Brain | . = 0.959

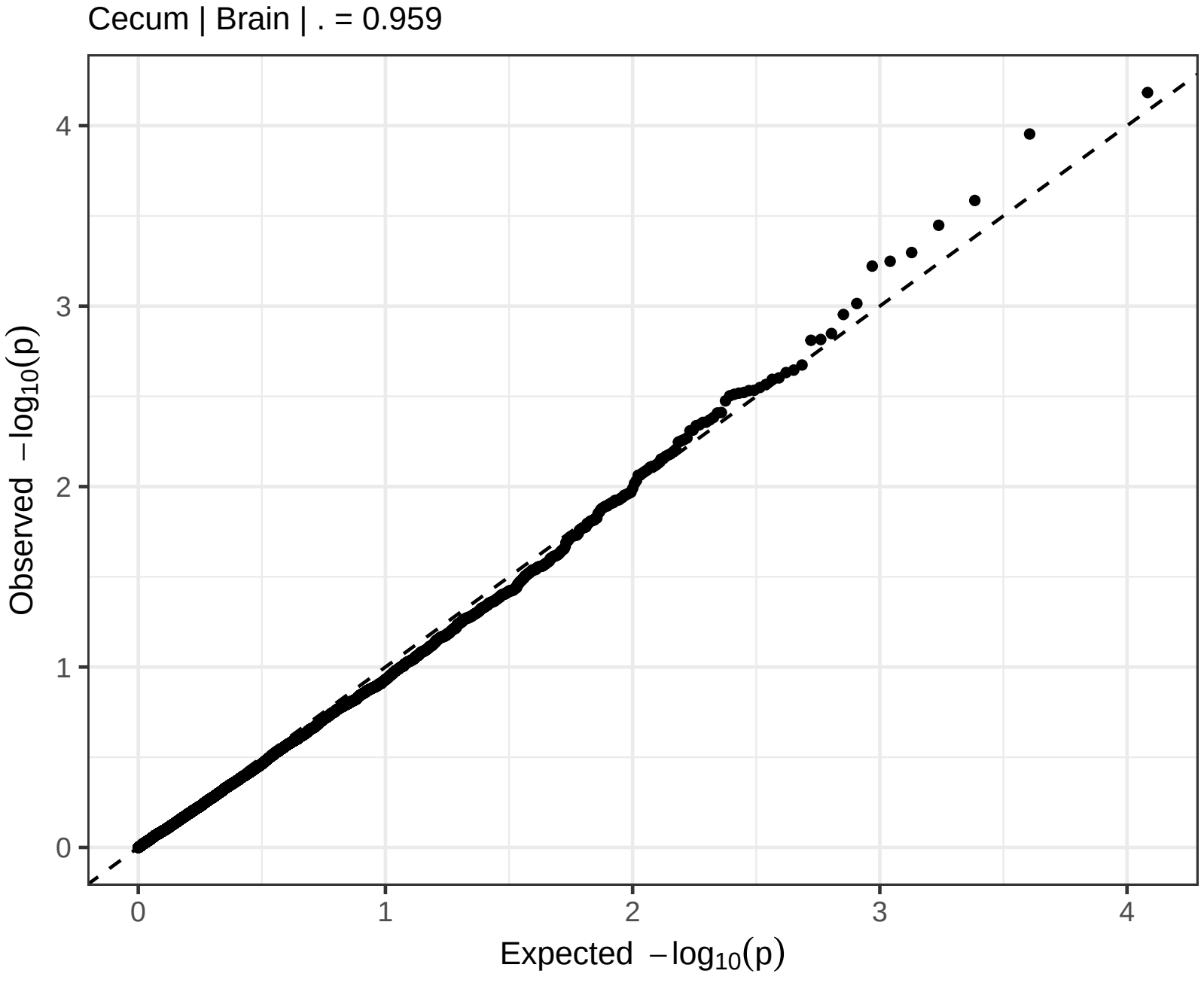

Agathobaculum

Cecum | Brain | . = 0.903

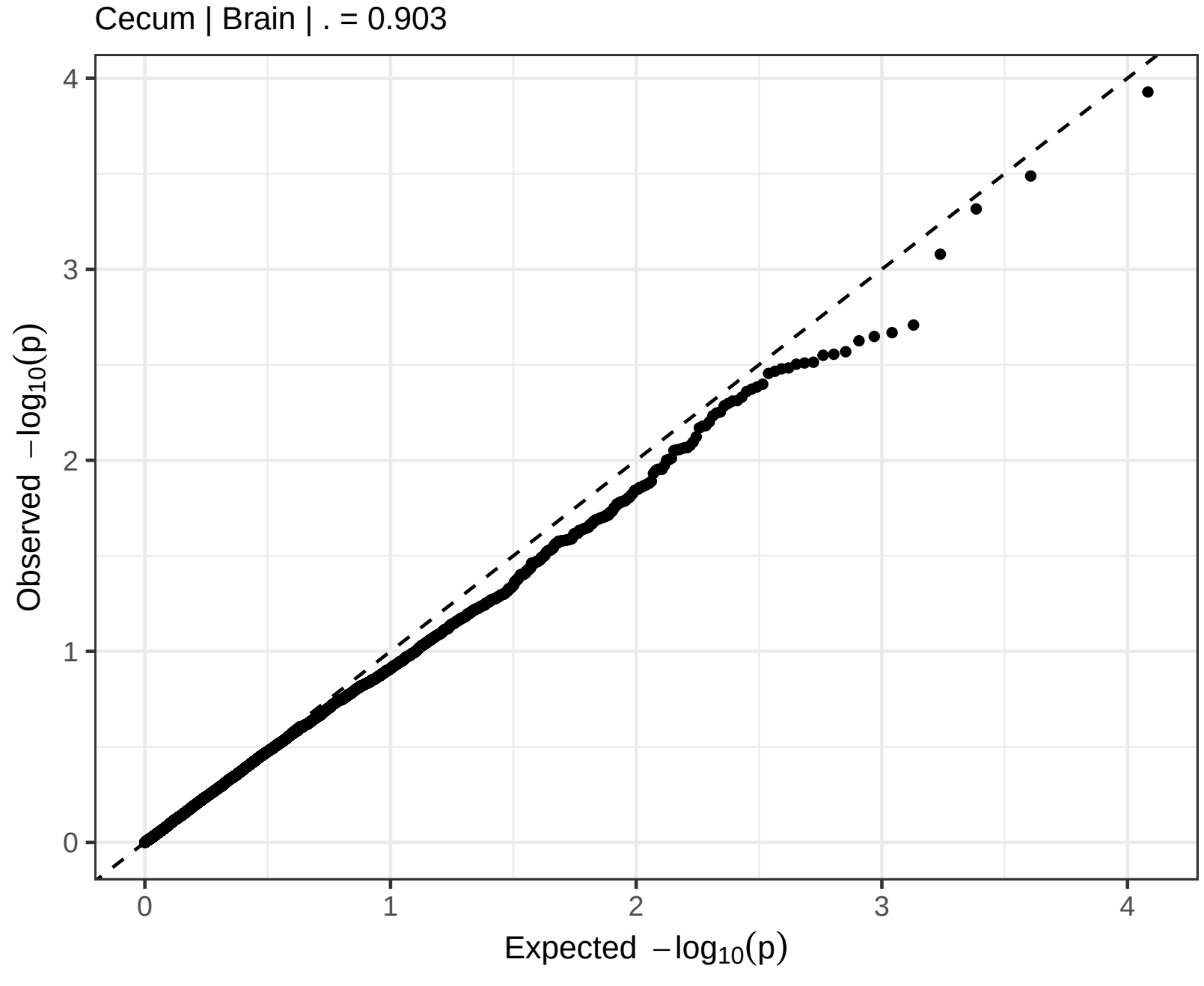

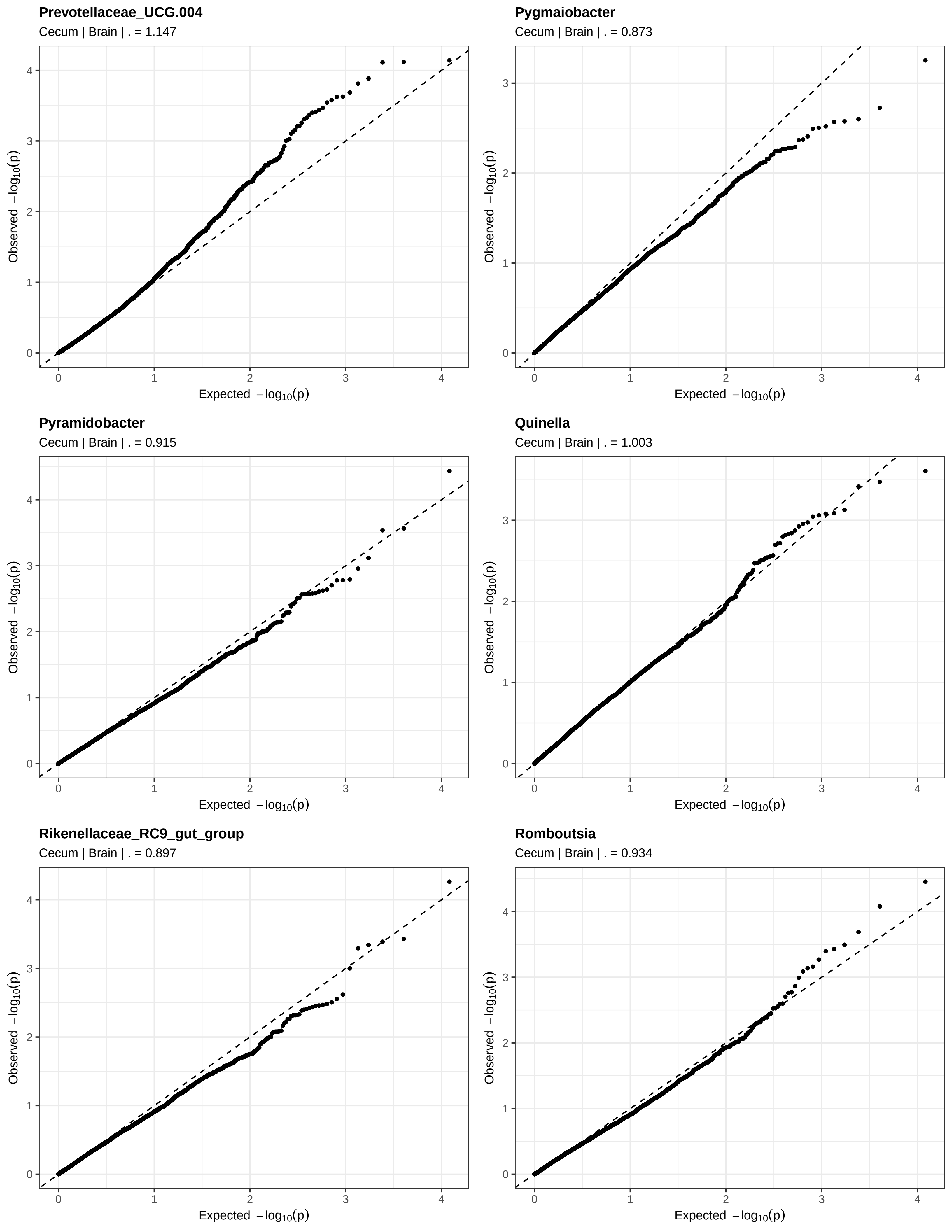

Prevotellaceae_UCG.004
Cecum | Brain | . = 1.147
Pygmaiobacter
Cecum | Brain | . = 0.873
Pyramidobacter
Cecum | Brain | . = 0.915
Quinella
Cecum | Brain | . = 1.003
Rikenellaceae_RC9_gut_group
Cecum | Brain | . = 0.897
Romboutsia
Cecum | Brain | . = 0.934
Observed $-\log_{10}(p)$
Expected $-\log_{10}(p)$

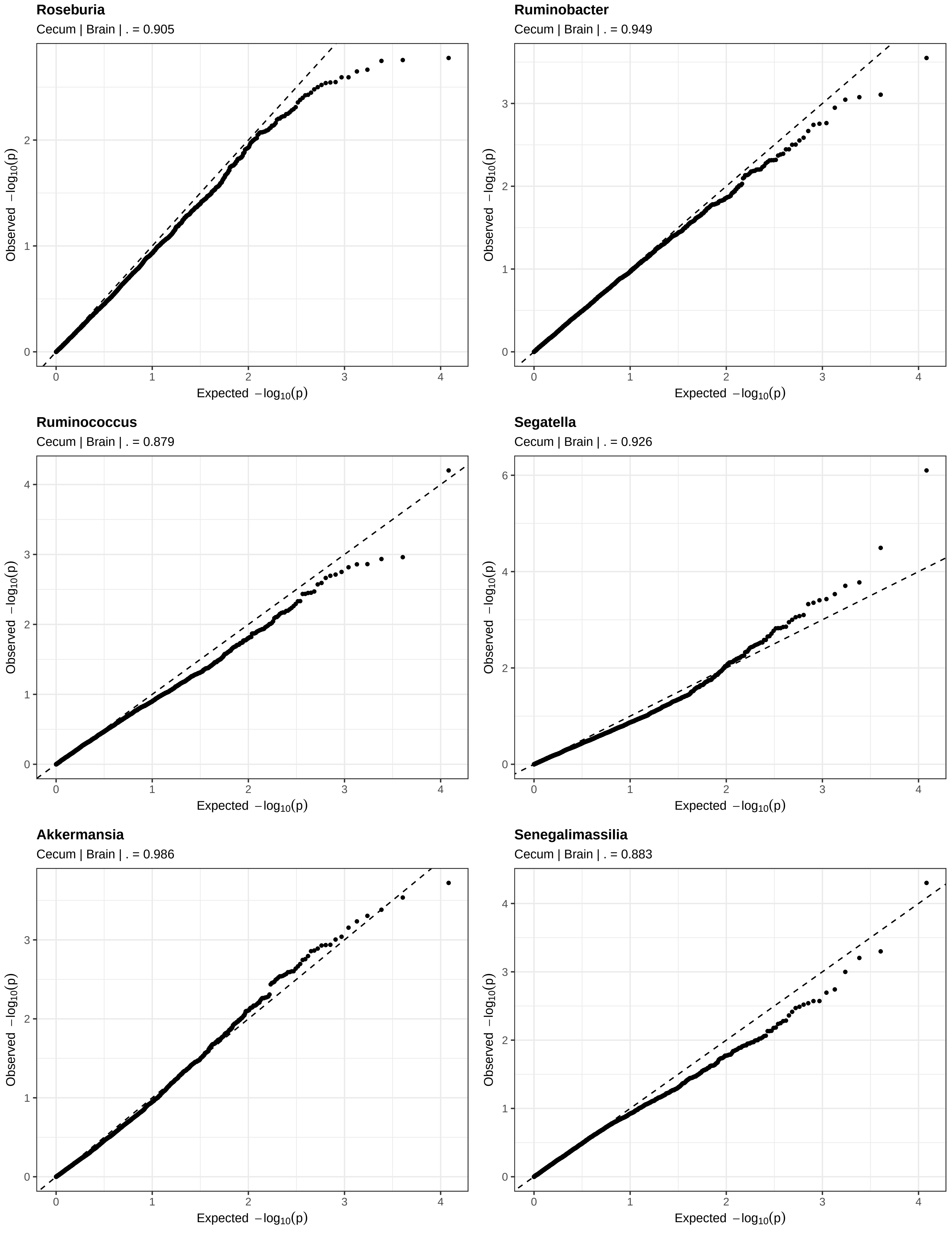
Roseburia
Cecum | Brain | . = 0.905
Ruminobacter
Cecum | Brain | . = 0.949
Ruminococcus
Cecum | Brain | . = 0.879
Segatella
Cecum | Brain | . = 0.926
Akkermansia
Cecum | Brain | . = 0.986
Senegalimassilia
Cecum | Brain | . = 0.883
Observed $-\log_{10}(p)$
Expected $-\log_{10}(p)$

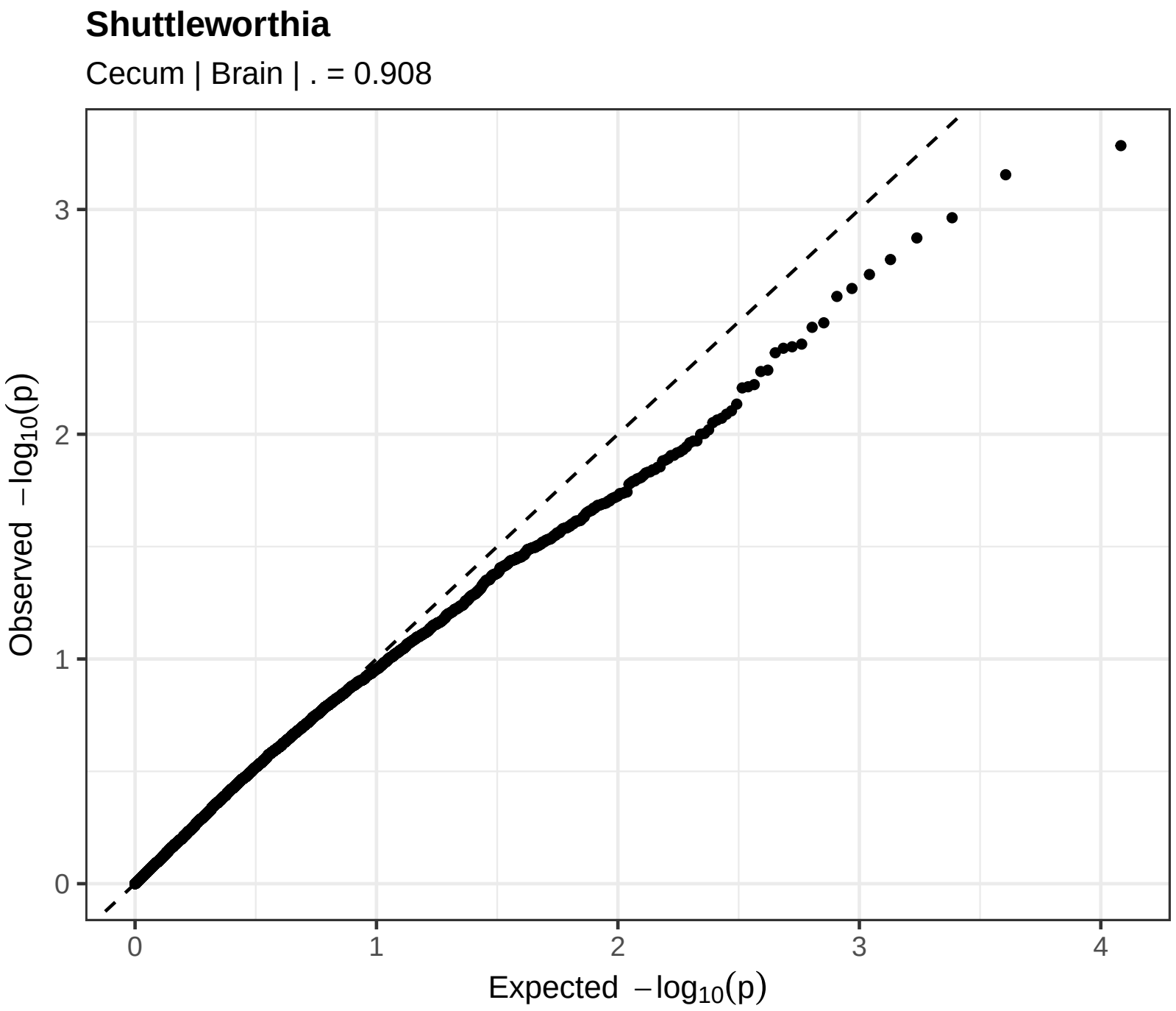
Shuttleworthia
Cecum | Brain | . = 0.908
Observed −log10(p)
Expected −log10(p)
0
1
2
3
4

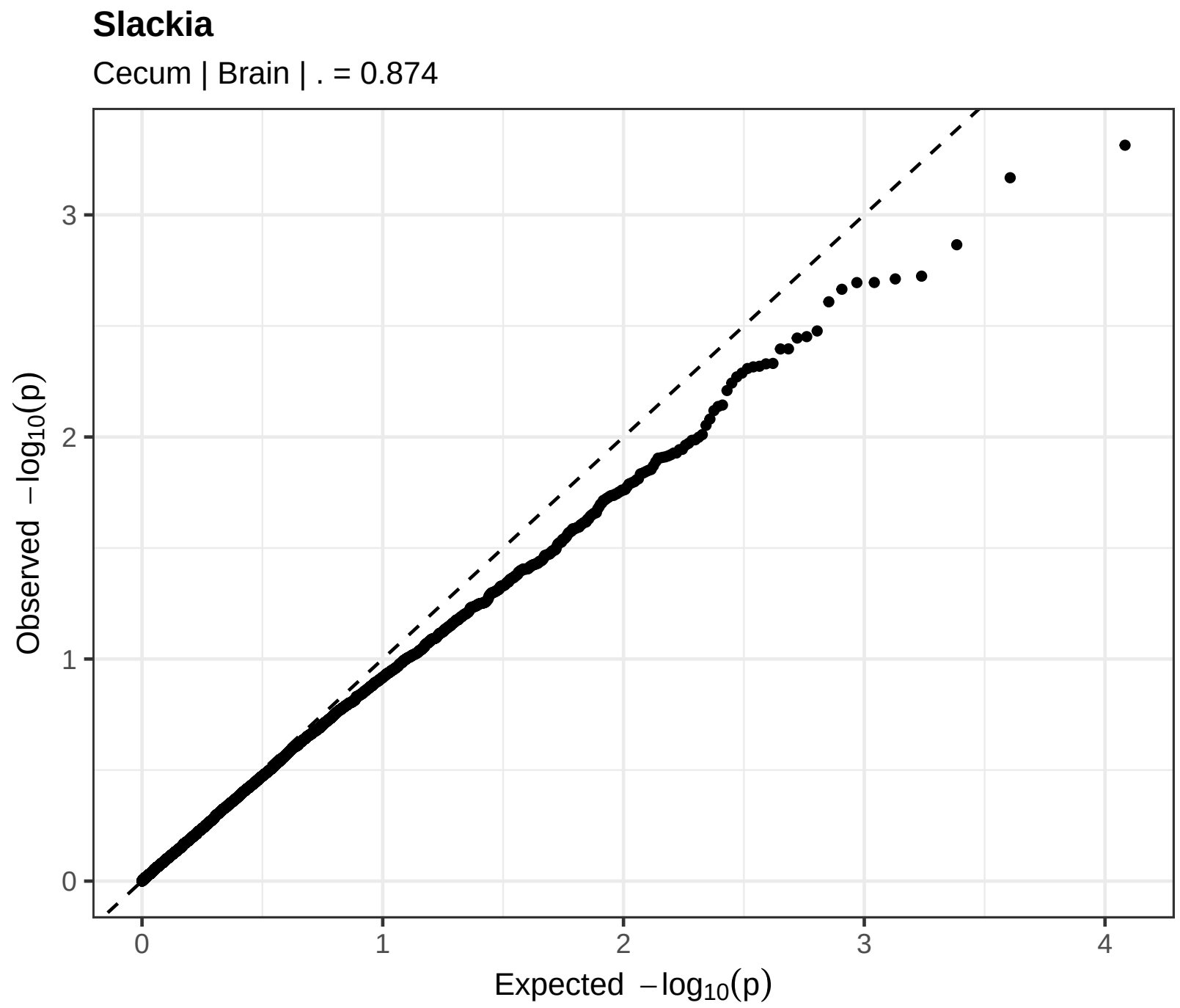
Slackia
Cecum | Brain | . = 0.874
Observed −log10(p)
Expected −log10(p)
0
1
2
3
4

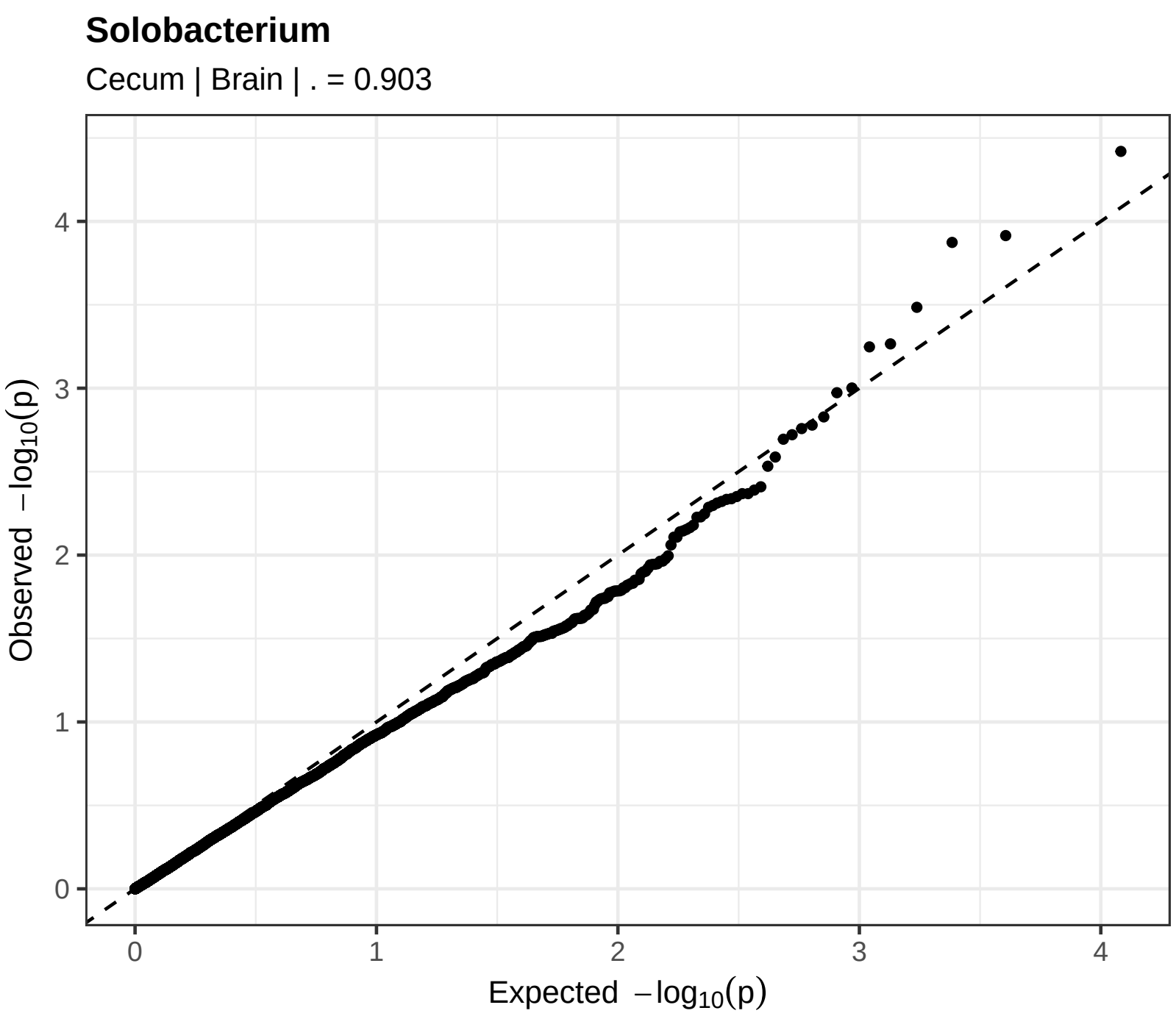
Solobacterium
Cecum | Brain | . = 0.903
Observed −log10(p)
Expected −log10(p)
0
1
2
3
4

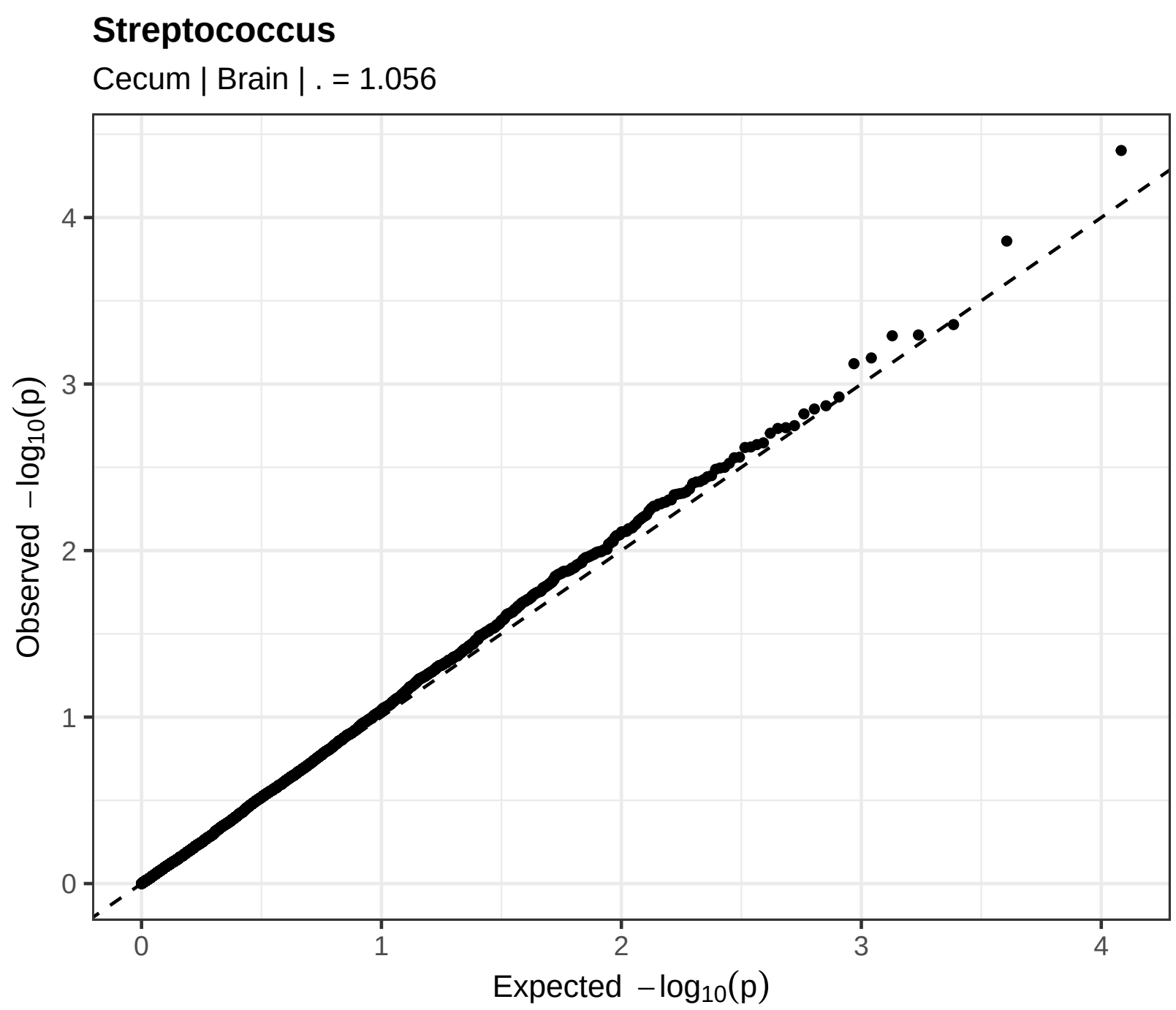
Streptococcus
Cecum | Brain | . = 1.056
Observed −log10(p)
Expected −log10(p)
0
1
2
3
4

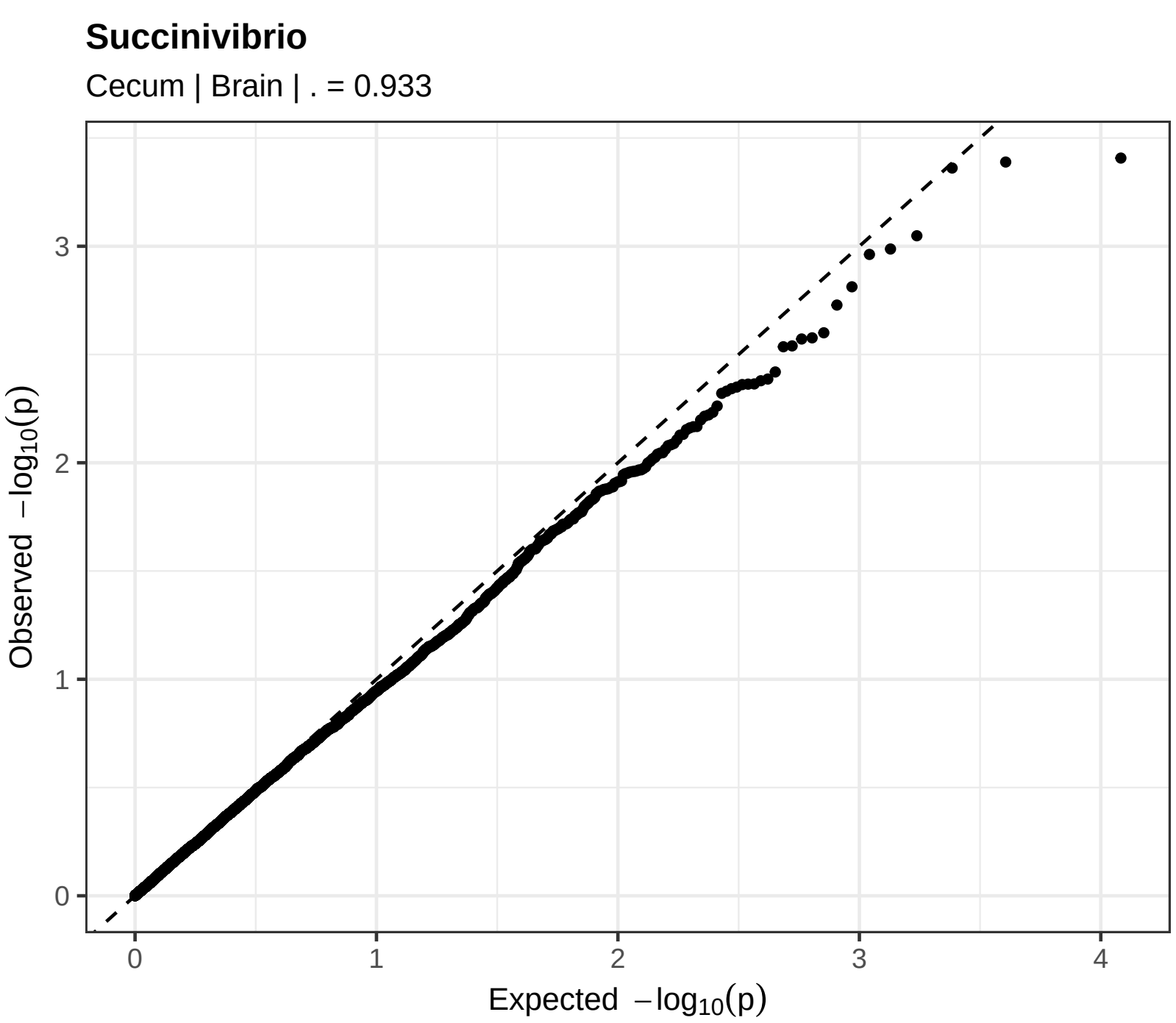
Succinivibrio
Cecum | Brain | . = 0.933
Observed −log10(p)
Expected −log10(p)
0
1
2
3
4

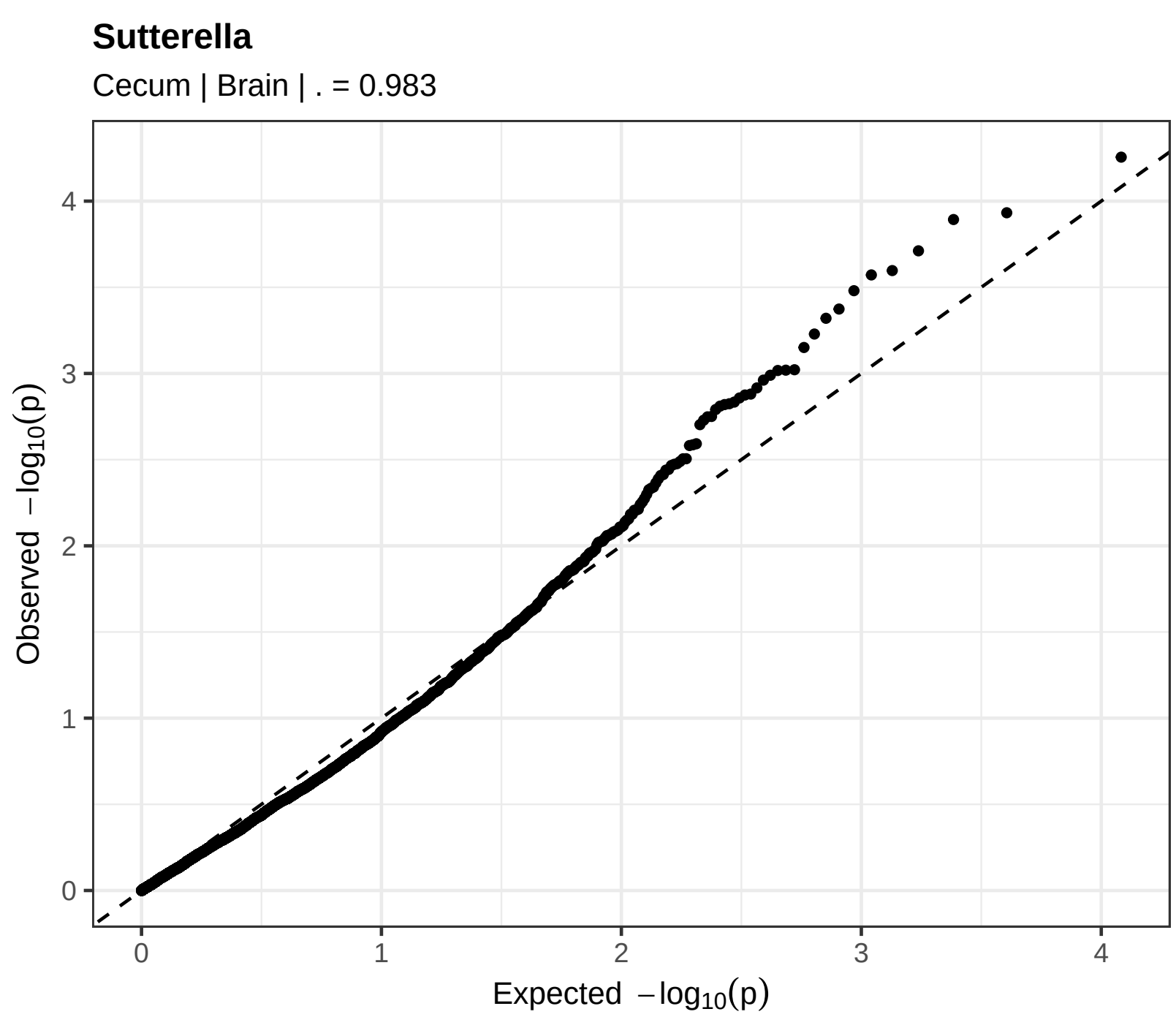
Sutterella
Cecum | Brain | . = 0.983
Observed −log10(p)
Expected −log10(p)
0
1
2
3
4

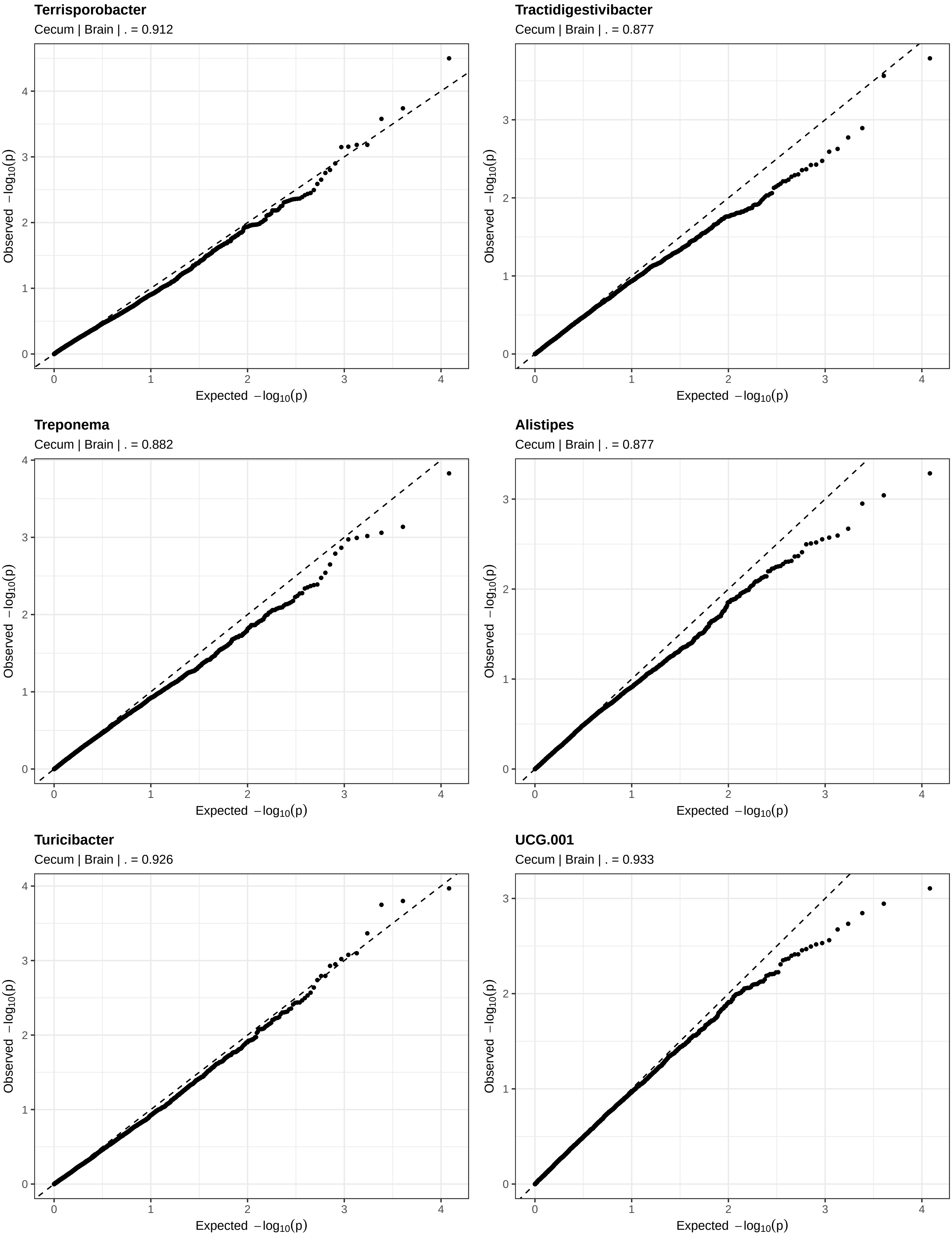
Terrisporobacter
Cecum | Brain | . = 0.912
Tractidigestivibacter
Cecum | Brain | . = 0.877
Treponema
Cecum | Brain | . = 0.882
Alistipes
Cecum | Brain | . = 0.877
Turicibacter
Cecum | Brain | . = 0.926
UCG.001
Cecum | Brain | . = 0.933
Observed $-\log_{10}(p)$
Expected $-\log_{10}(p)$

UCG.002

Cecum | Brain | . = 0.987

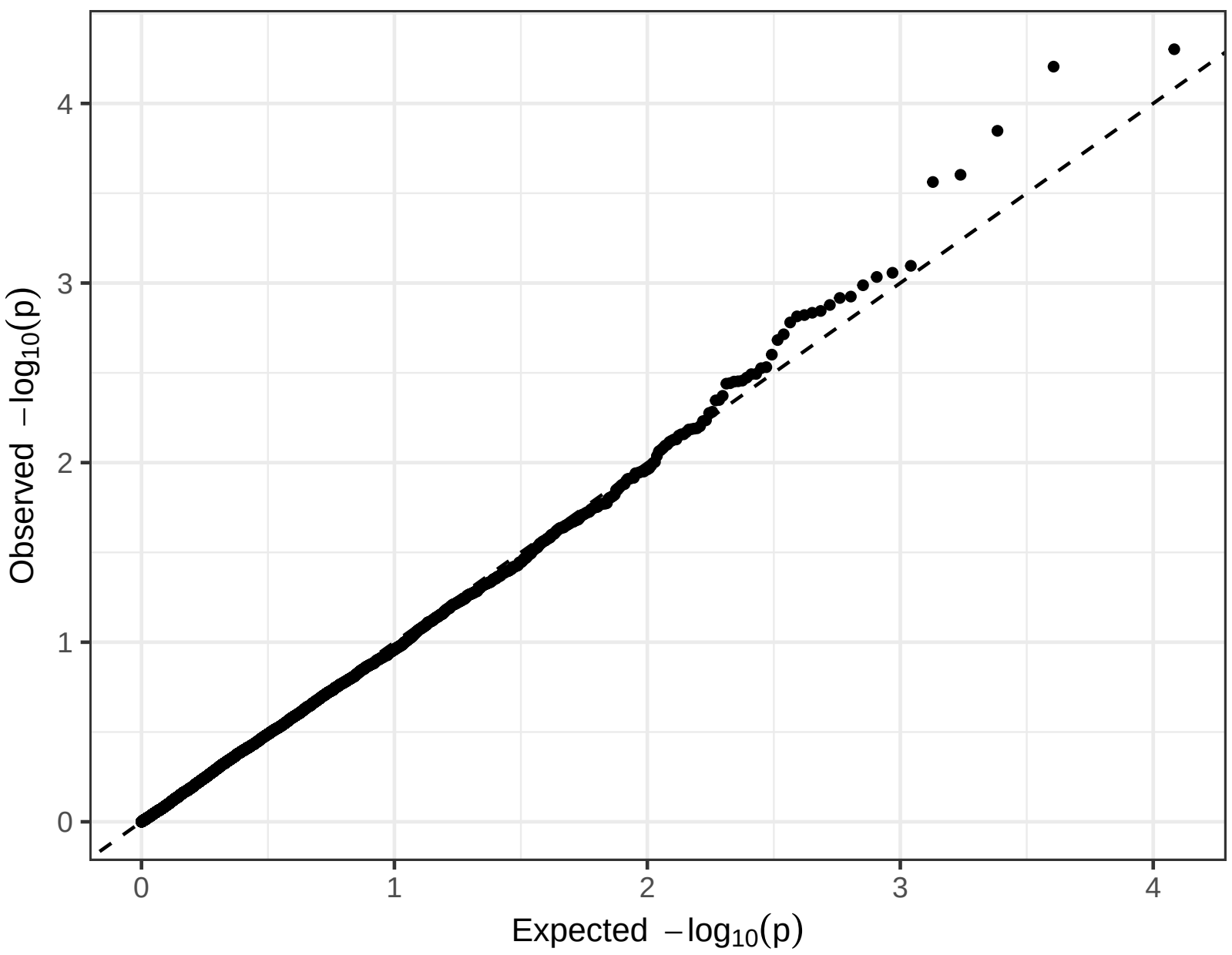

UCG.004

Cecum | Brain | . = 0.906

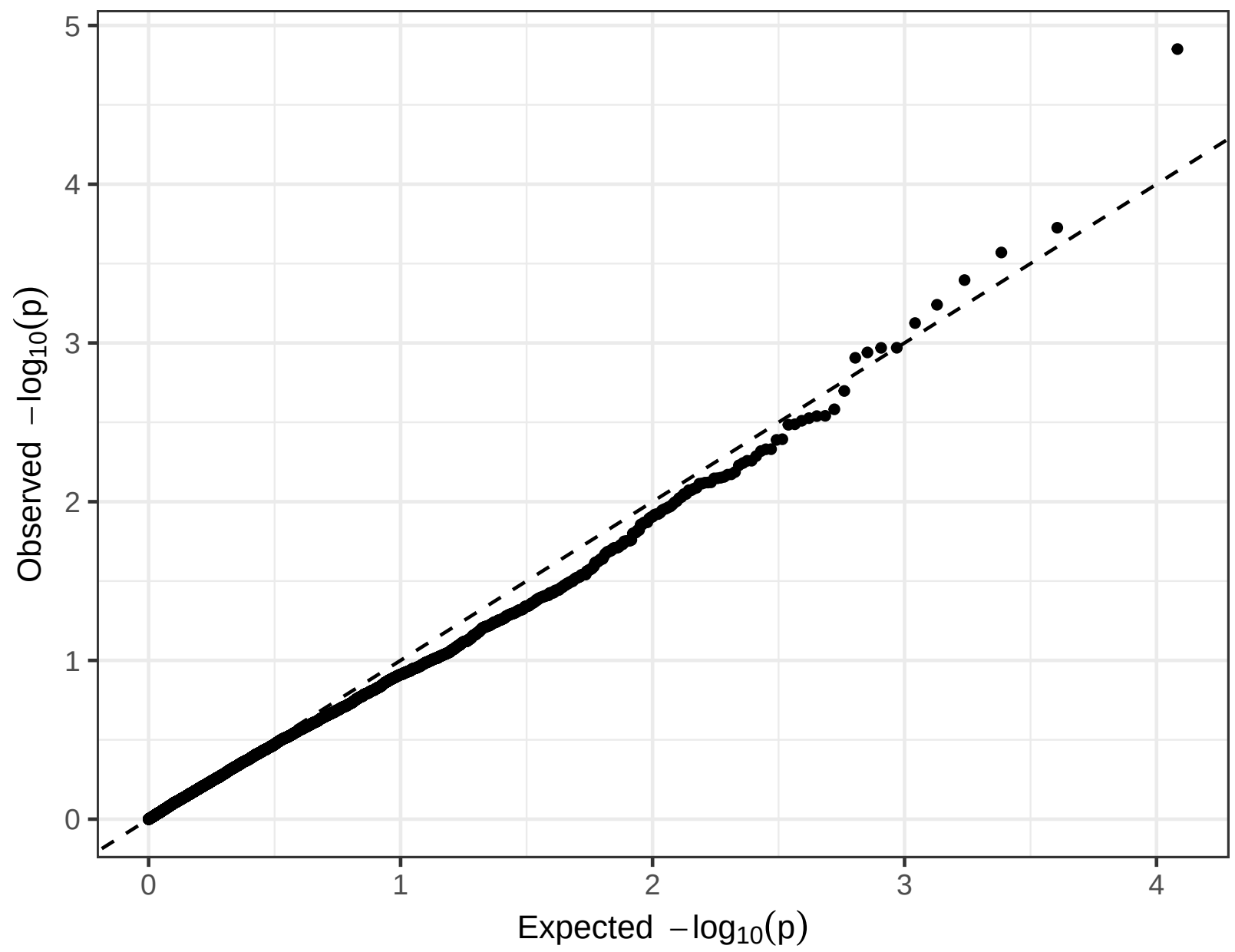

UCG.005

Cecum | Brain | . = 0.936

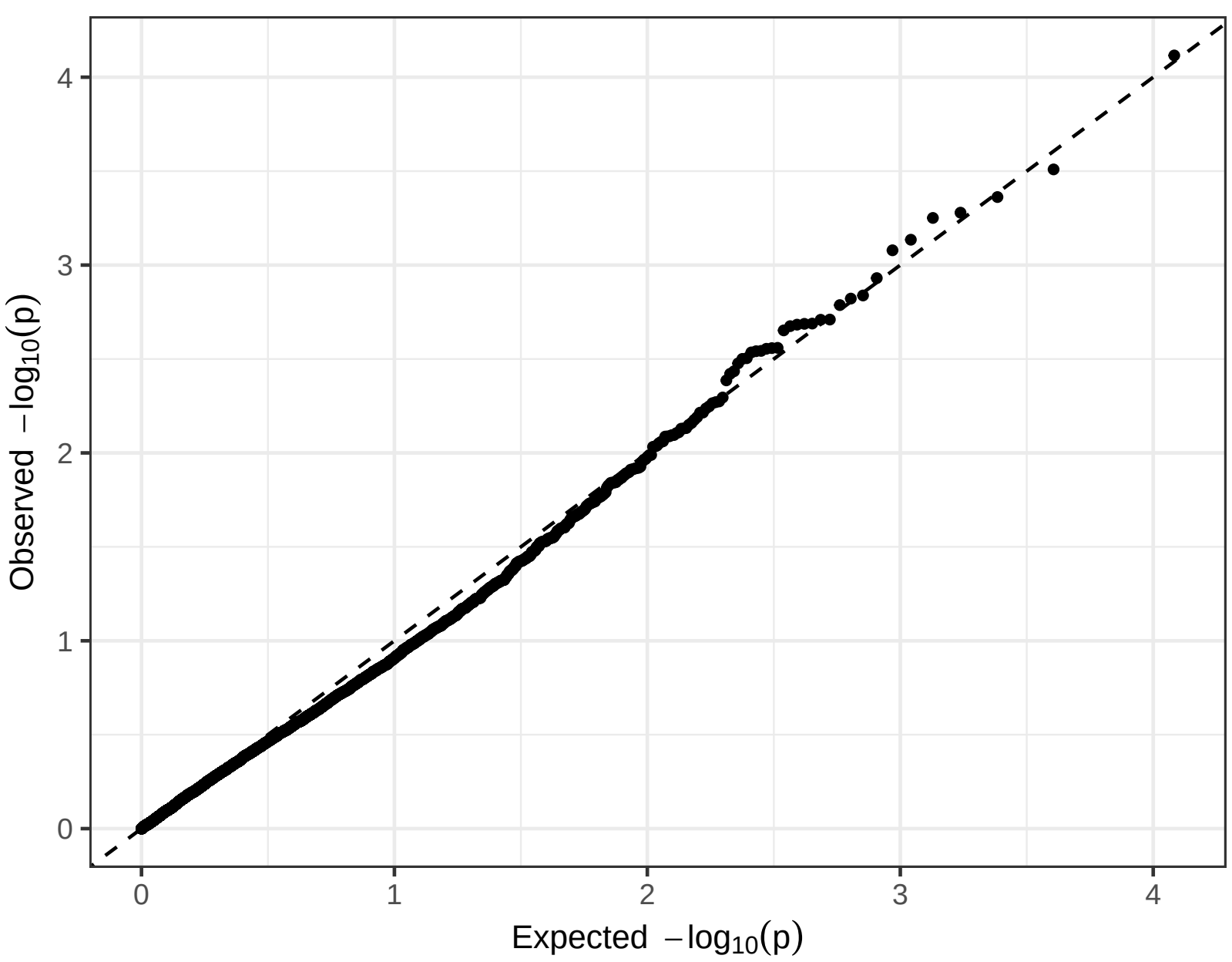

UCG.008

Cecum | Brain | . = 0.901

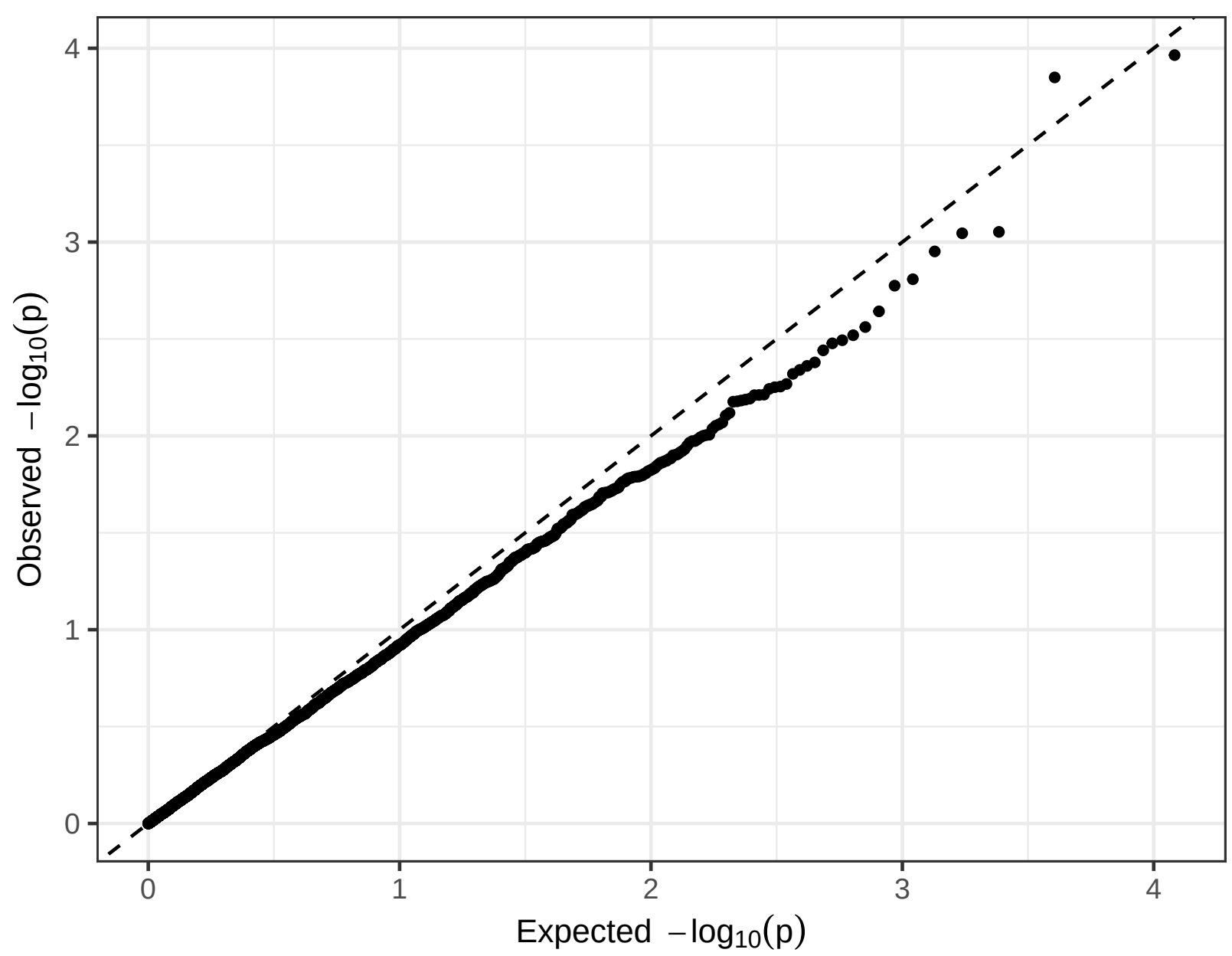

UCG.009

Cecum | Brain | . = 0.901

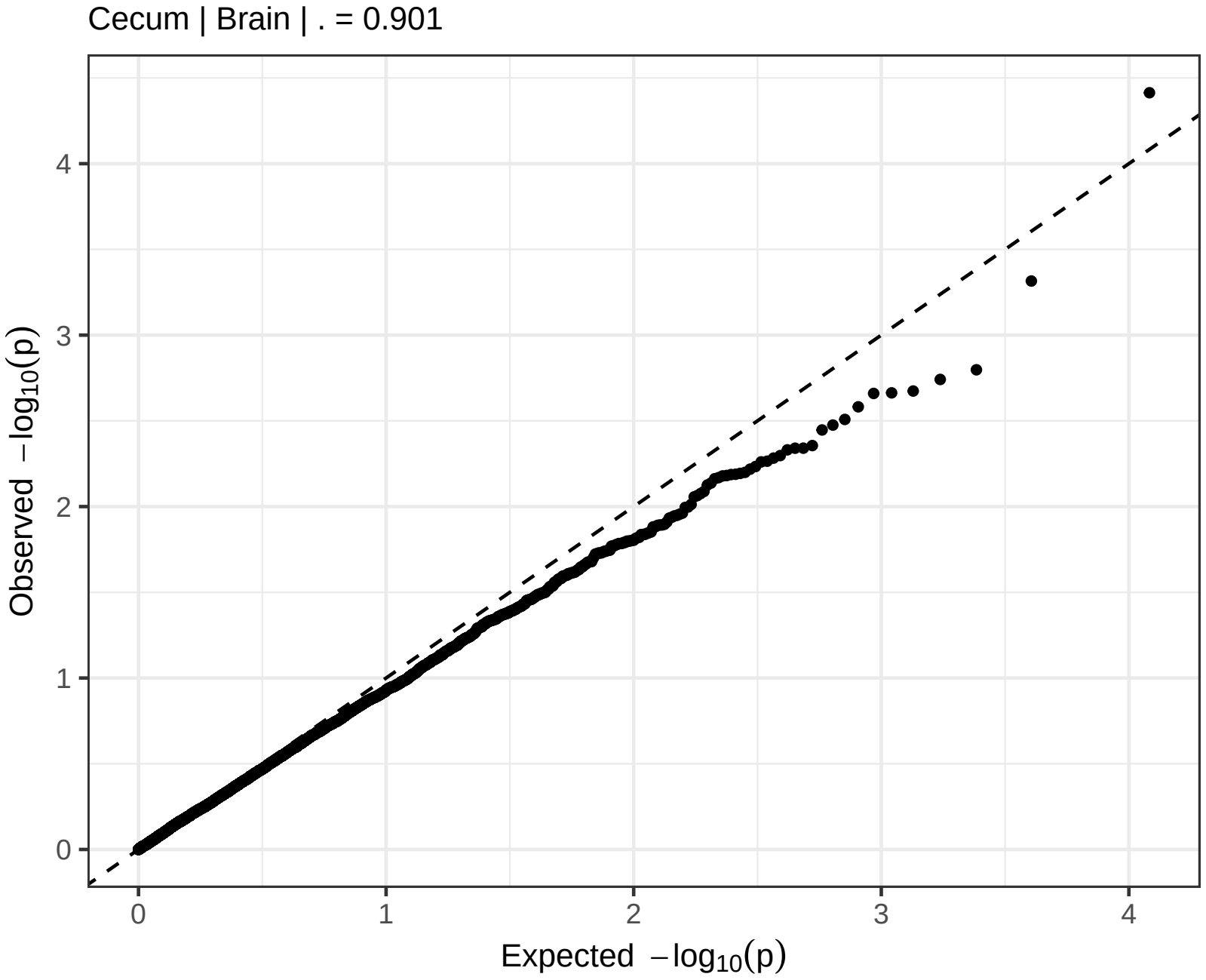

Xylanibacter

Cecum | Brain | . = 0.983

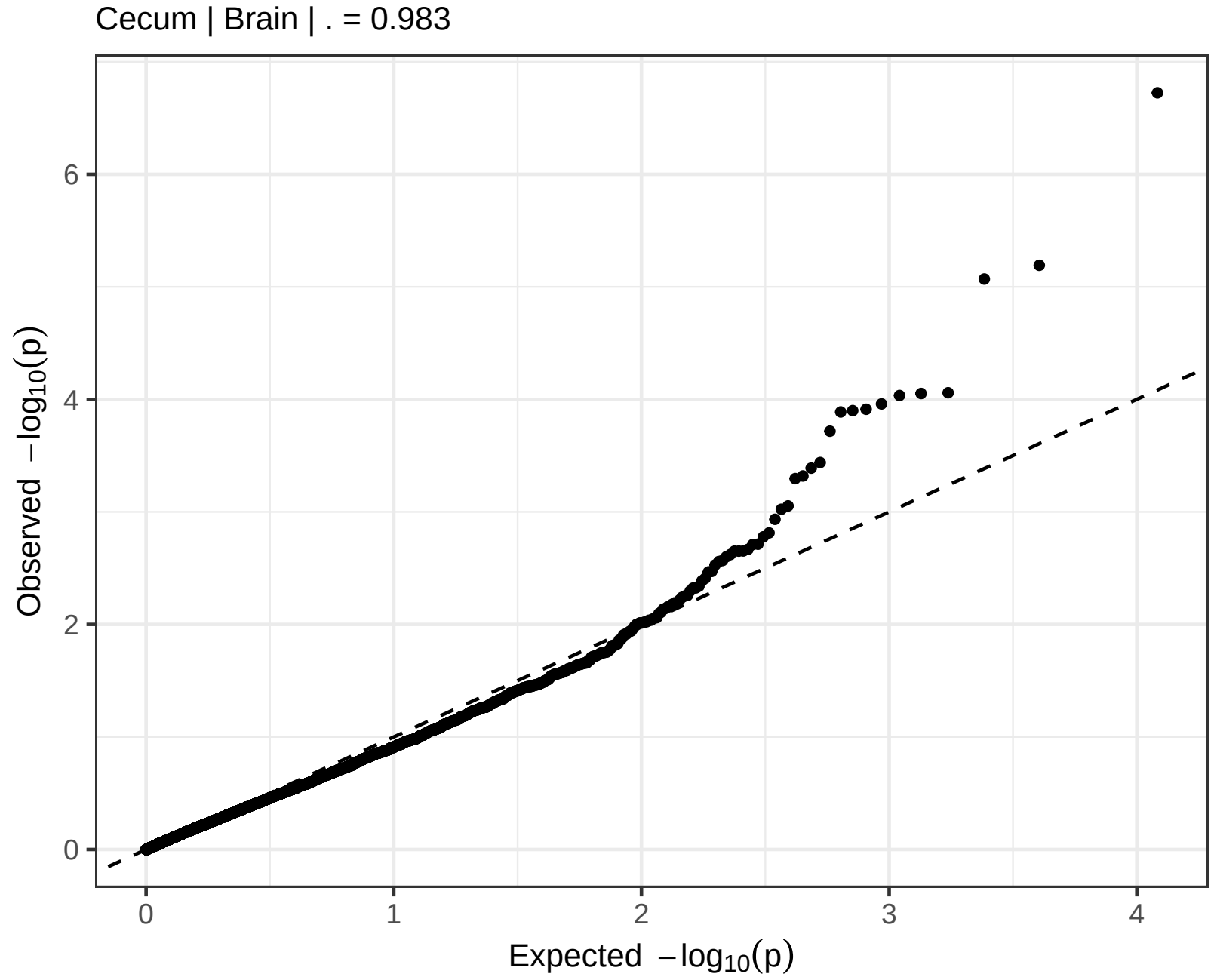

Z20

Cecum | Brain | . = 0.881

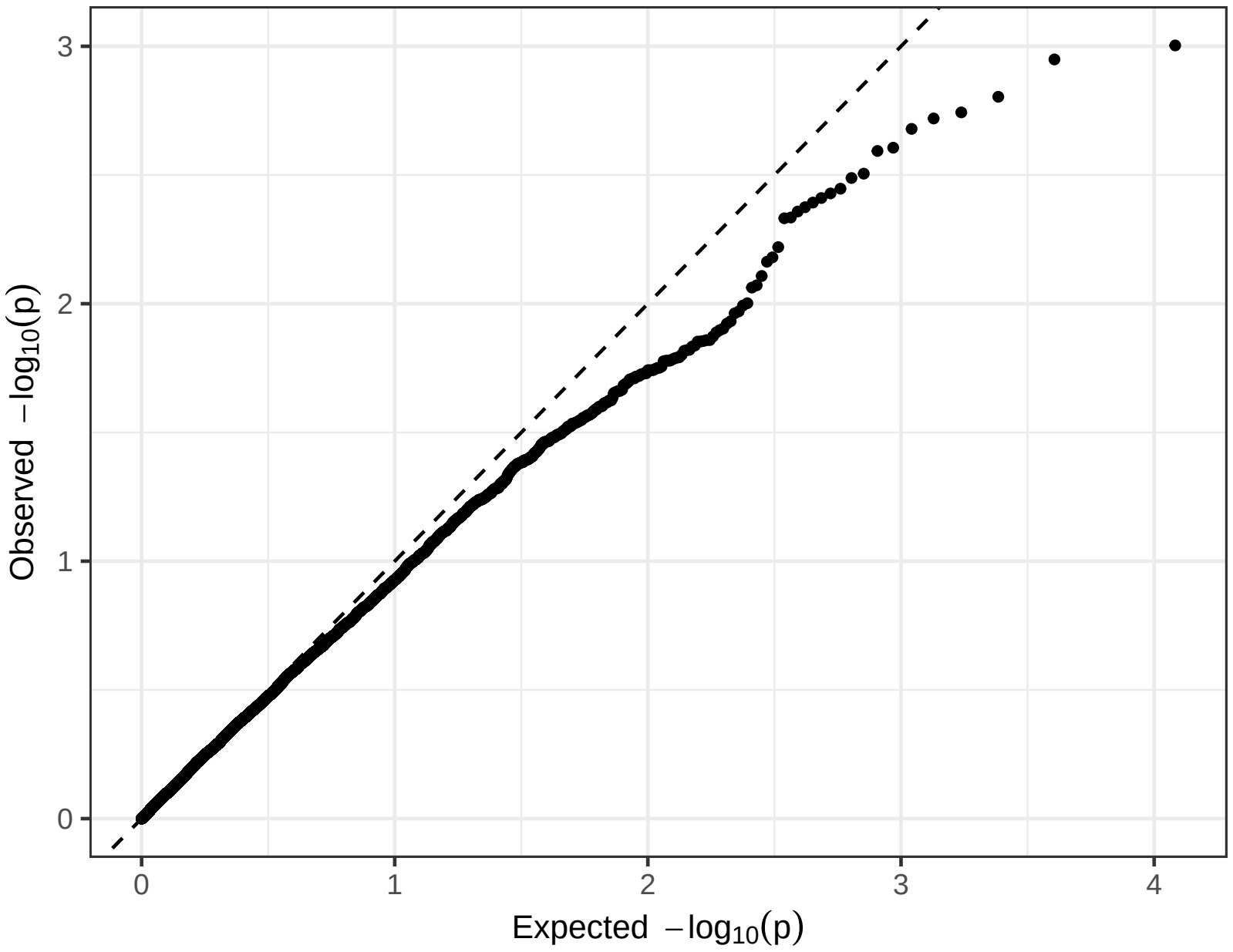

Zag_111

Cecum | Brain | . = 0.981

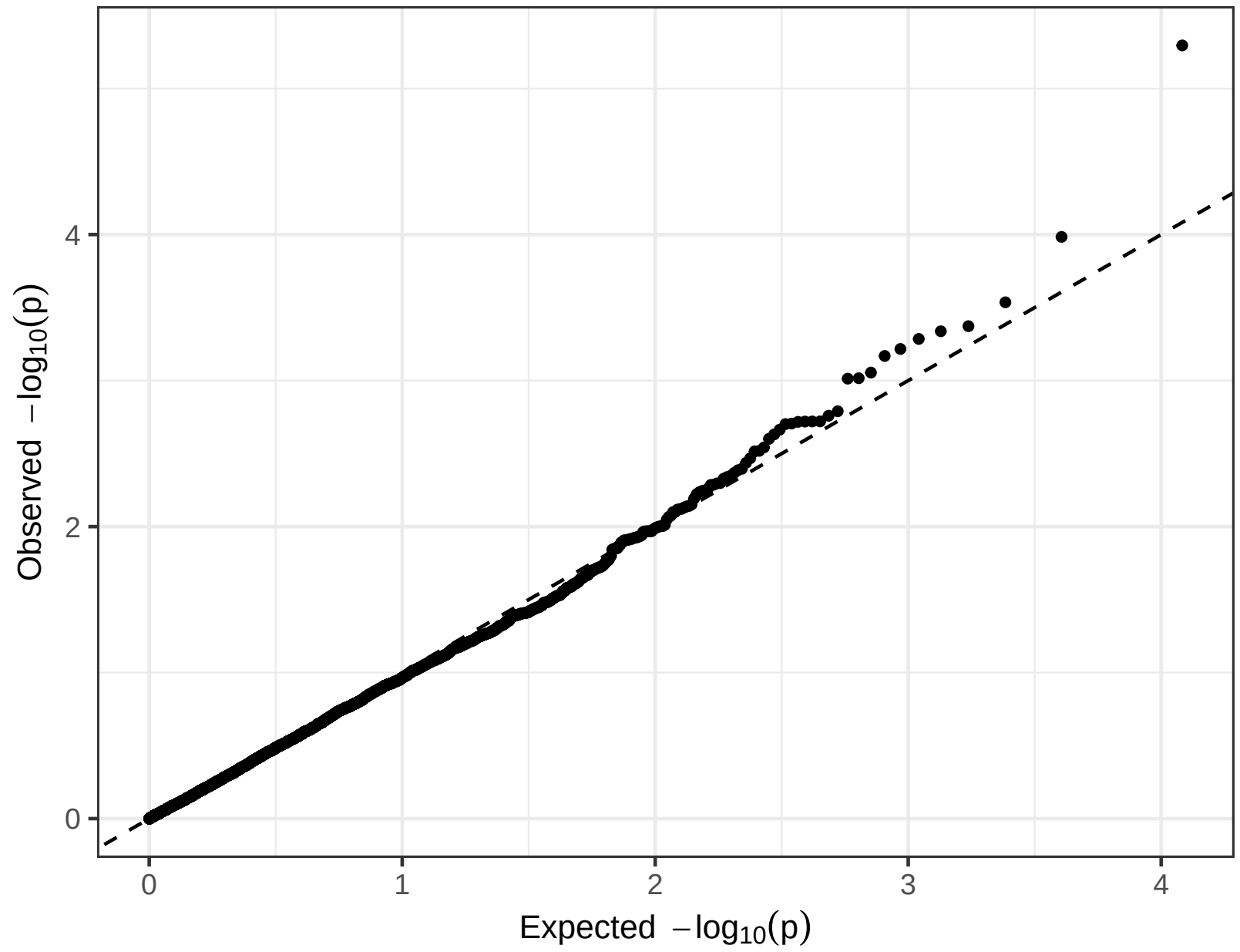

Alloprevotella

Cecum | Brain | . = 0.983

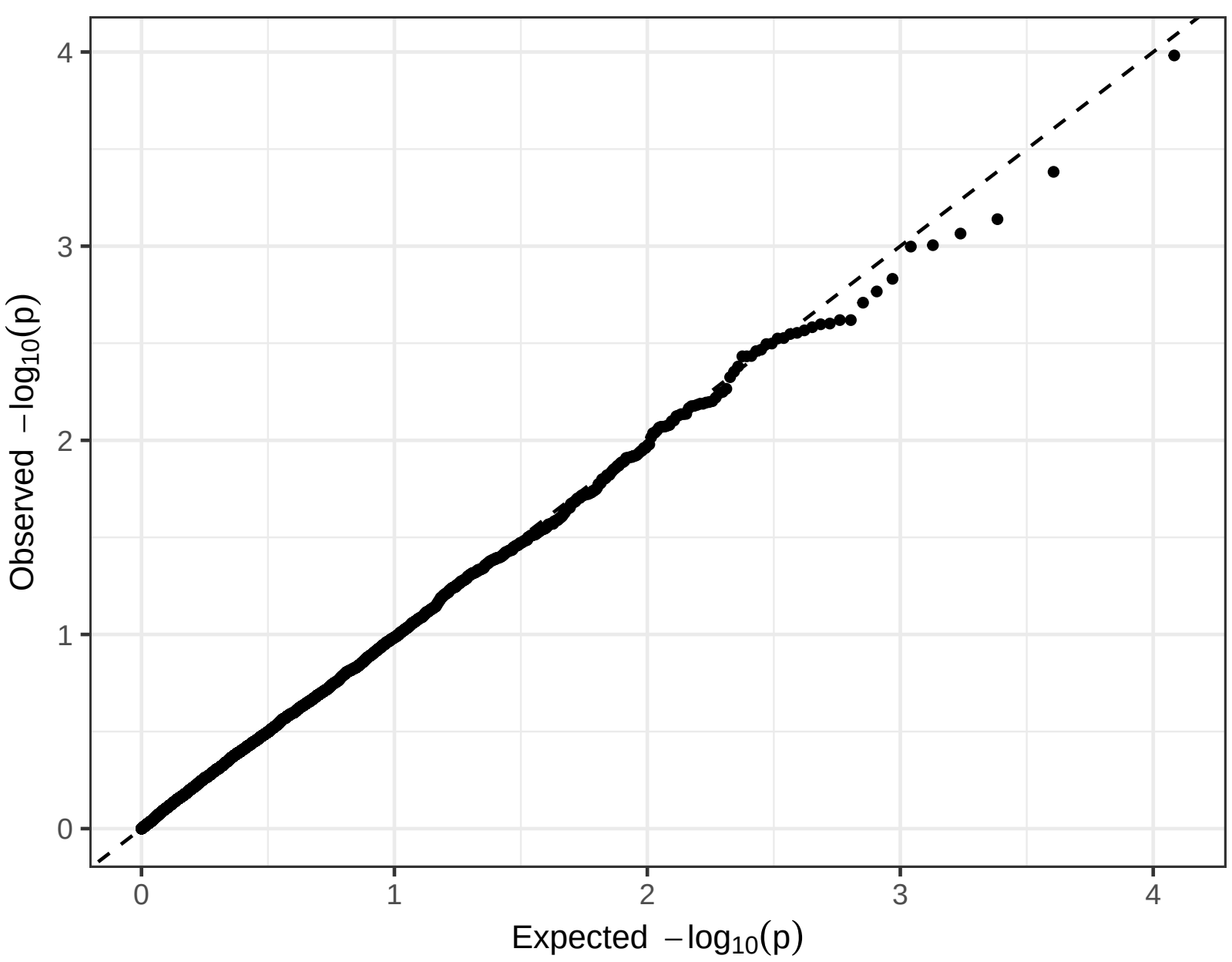

Aminipila

Cecum | Brain | . = 0.85

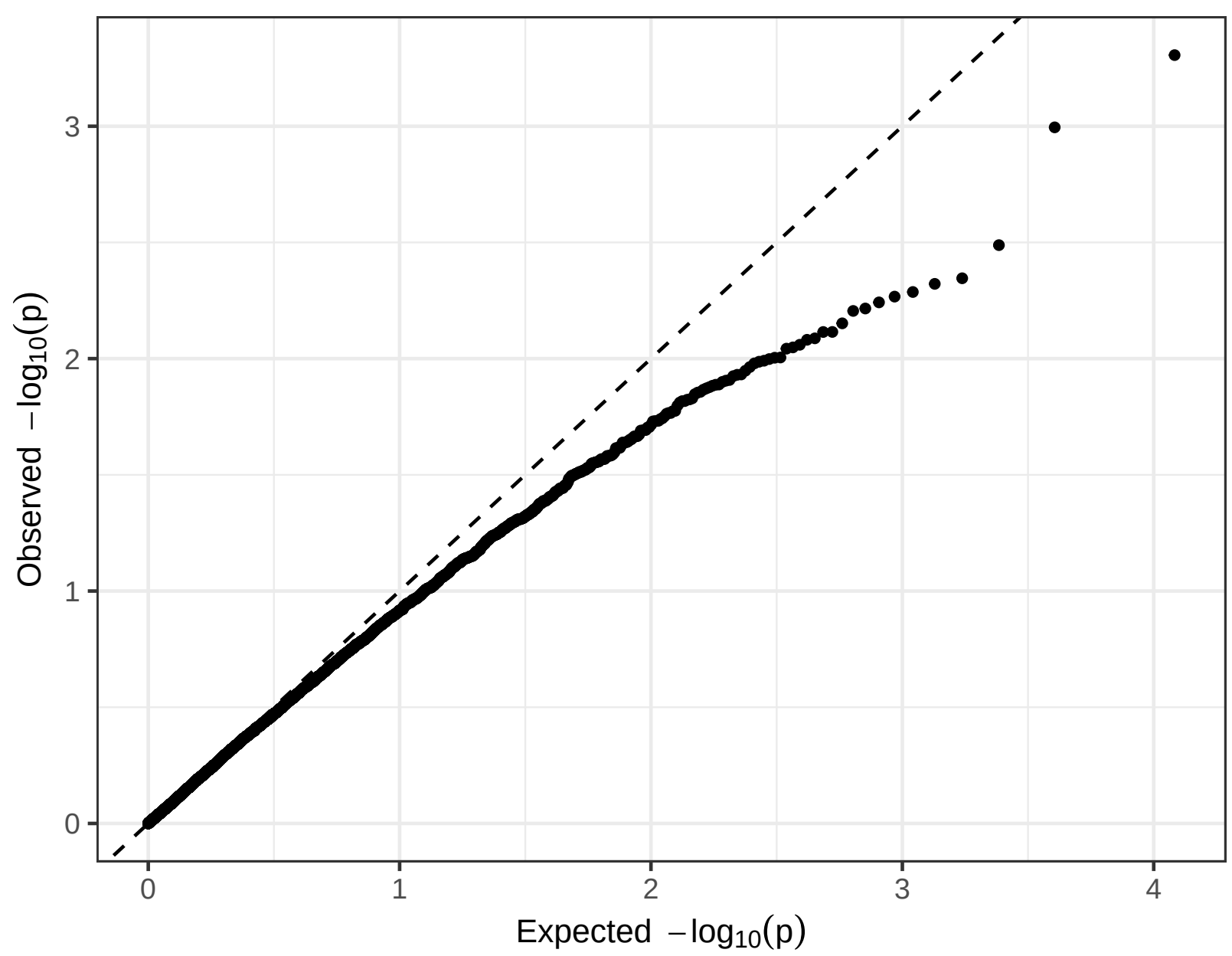

Anaerobiospirillum

Cecum | Brain | . = 0.9

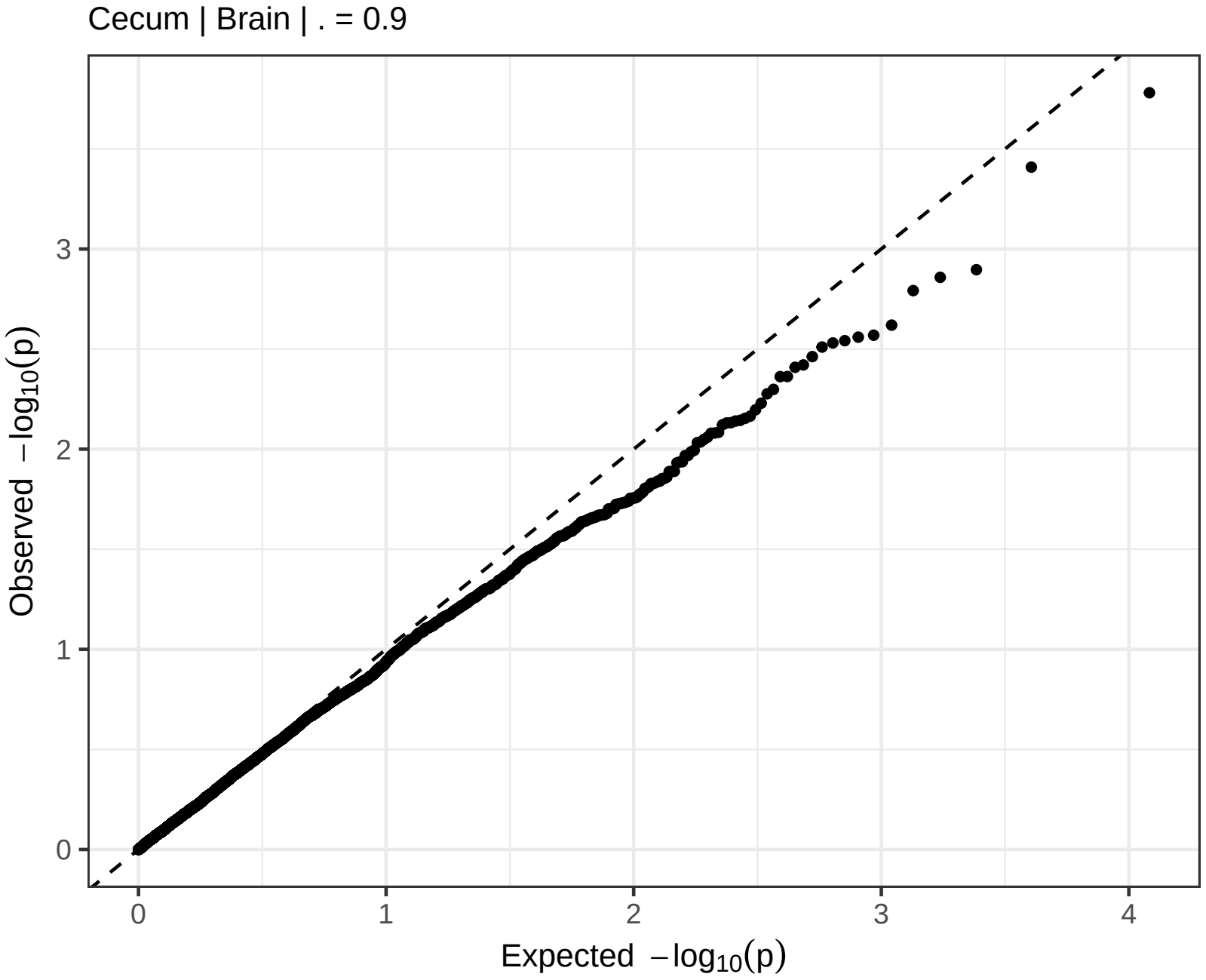

Anaerobutyricum

Cecum | Brain | . = 0.898

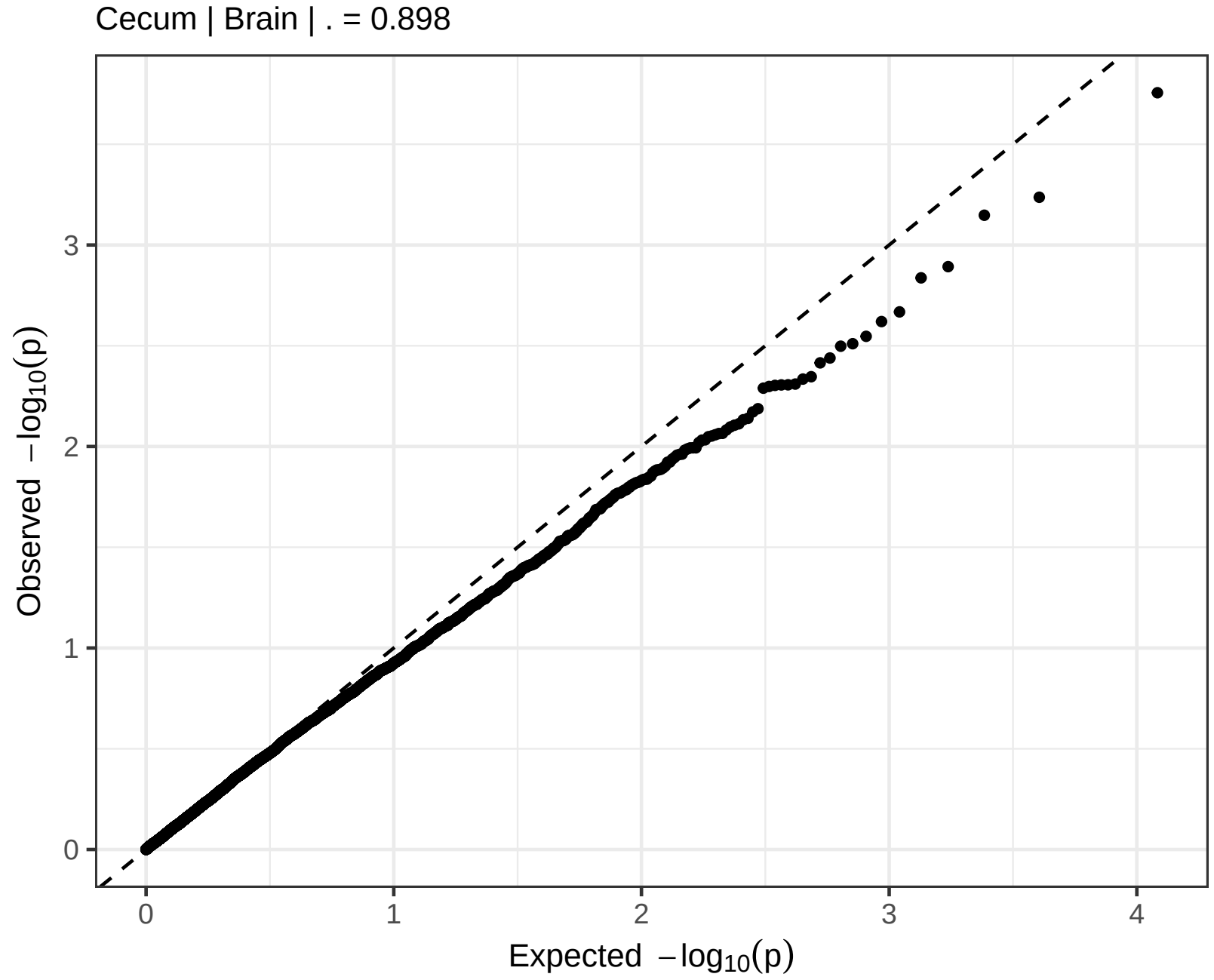

Anaeroplasma

Cecum | Brain | . = 0.9

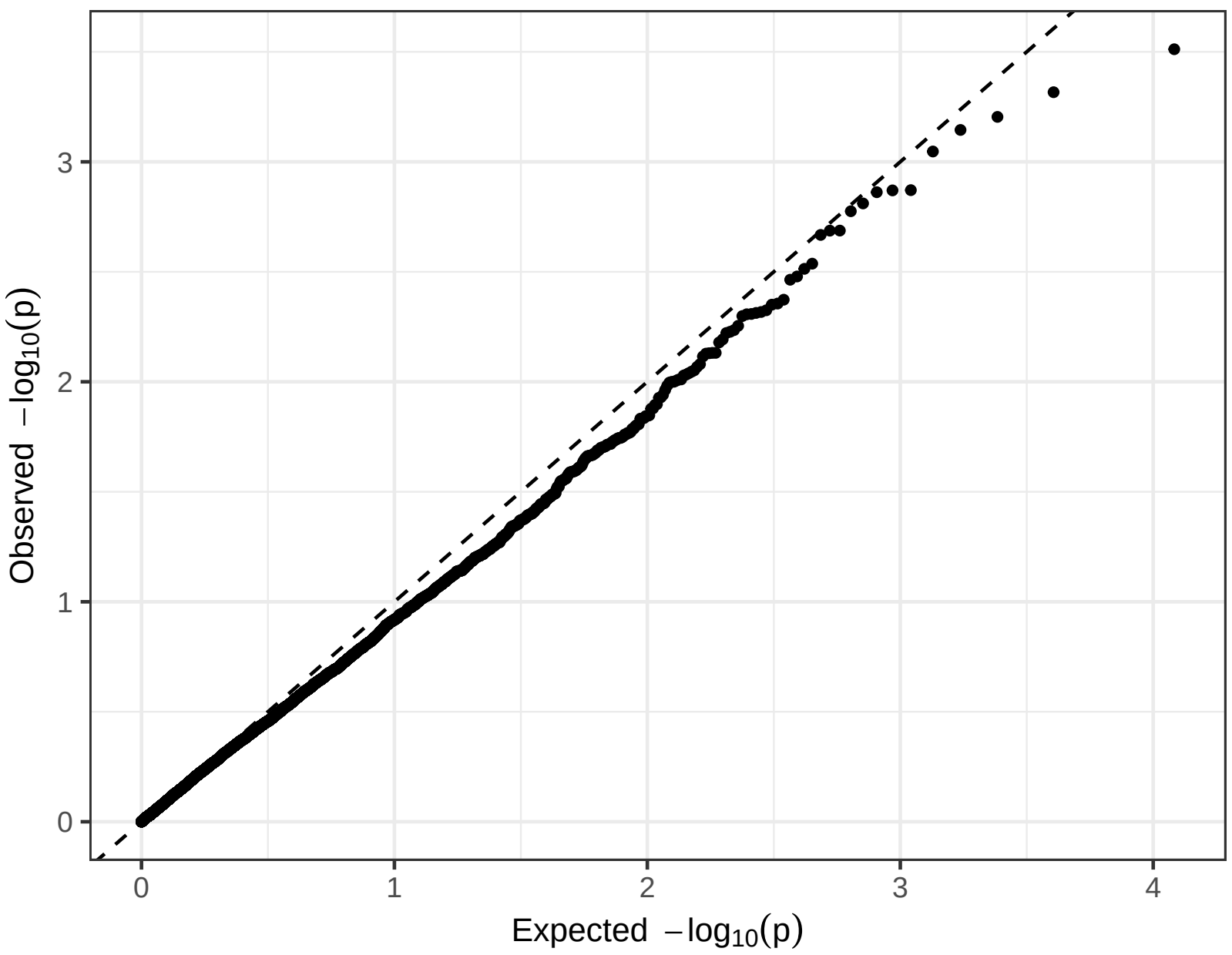

Eubacterium_ruminantium_group

Cecum | Brain | . = 0.9

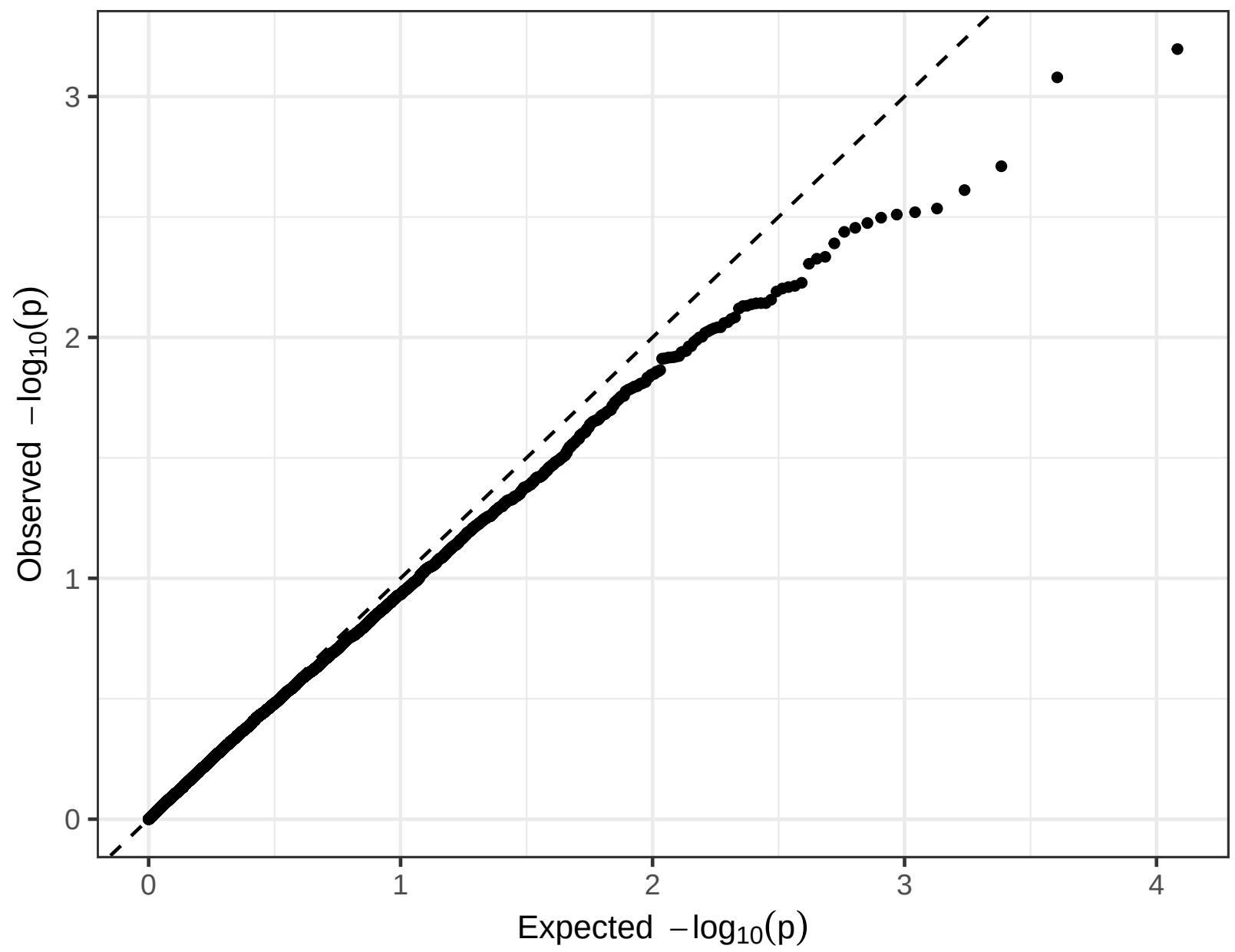

Anaerorhabdus

Cecum | Brain | . = 0.941

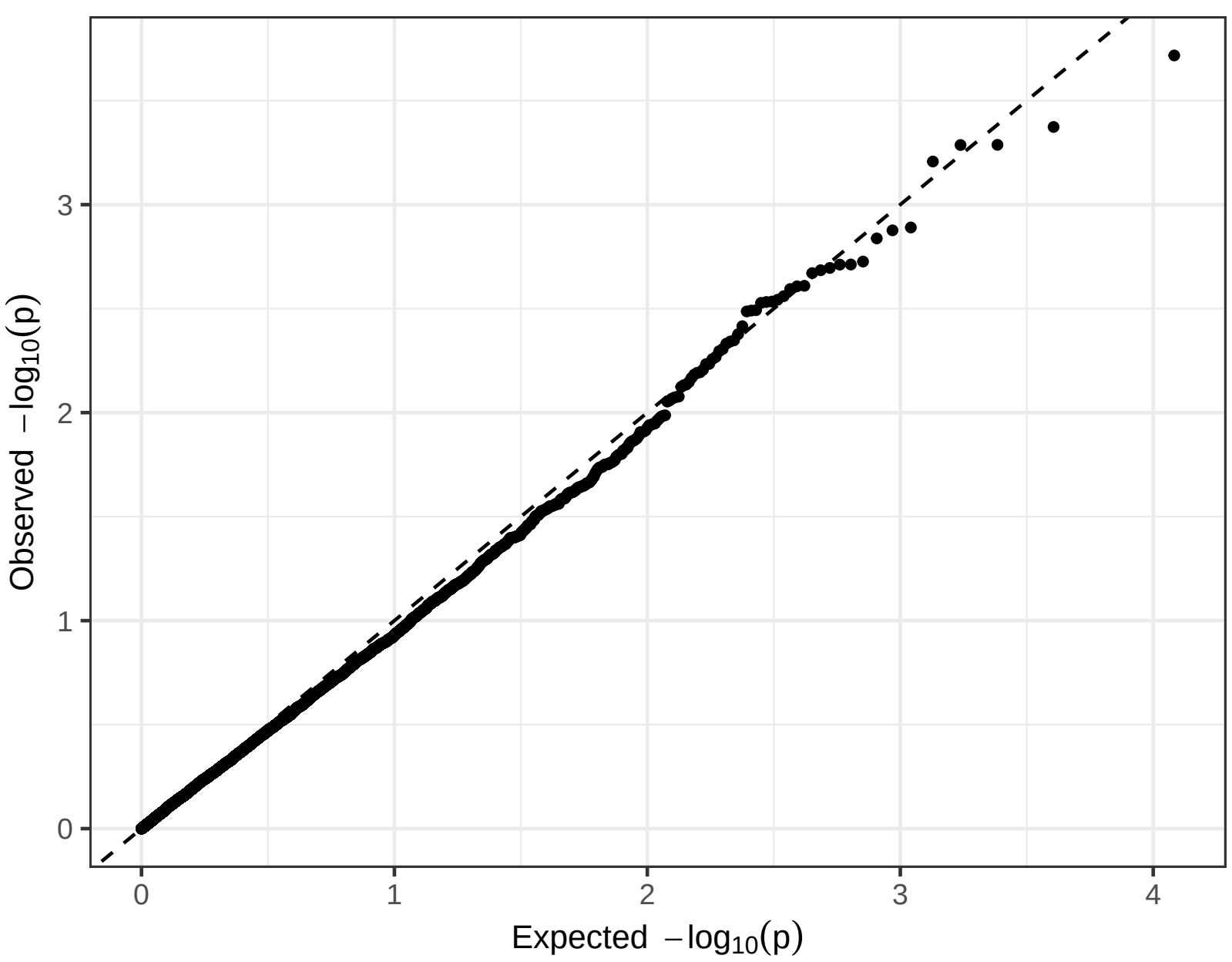

Anaerovibrio

Cecum | Brain | . = 1.047

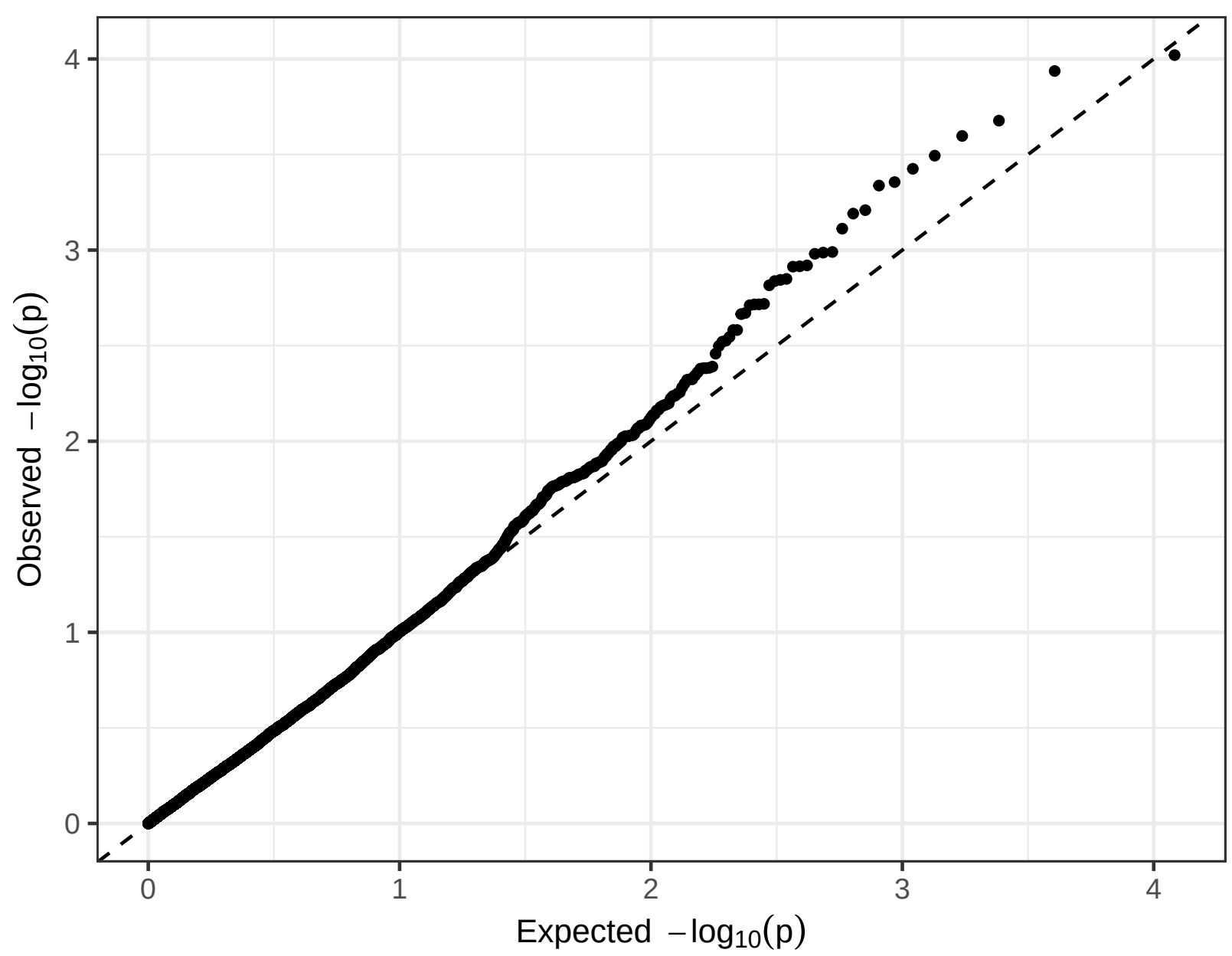

Anaerovorax

Cecum | Brain | . = 0.873

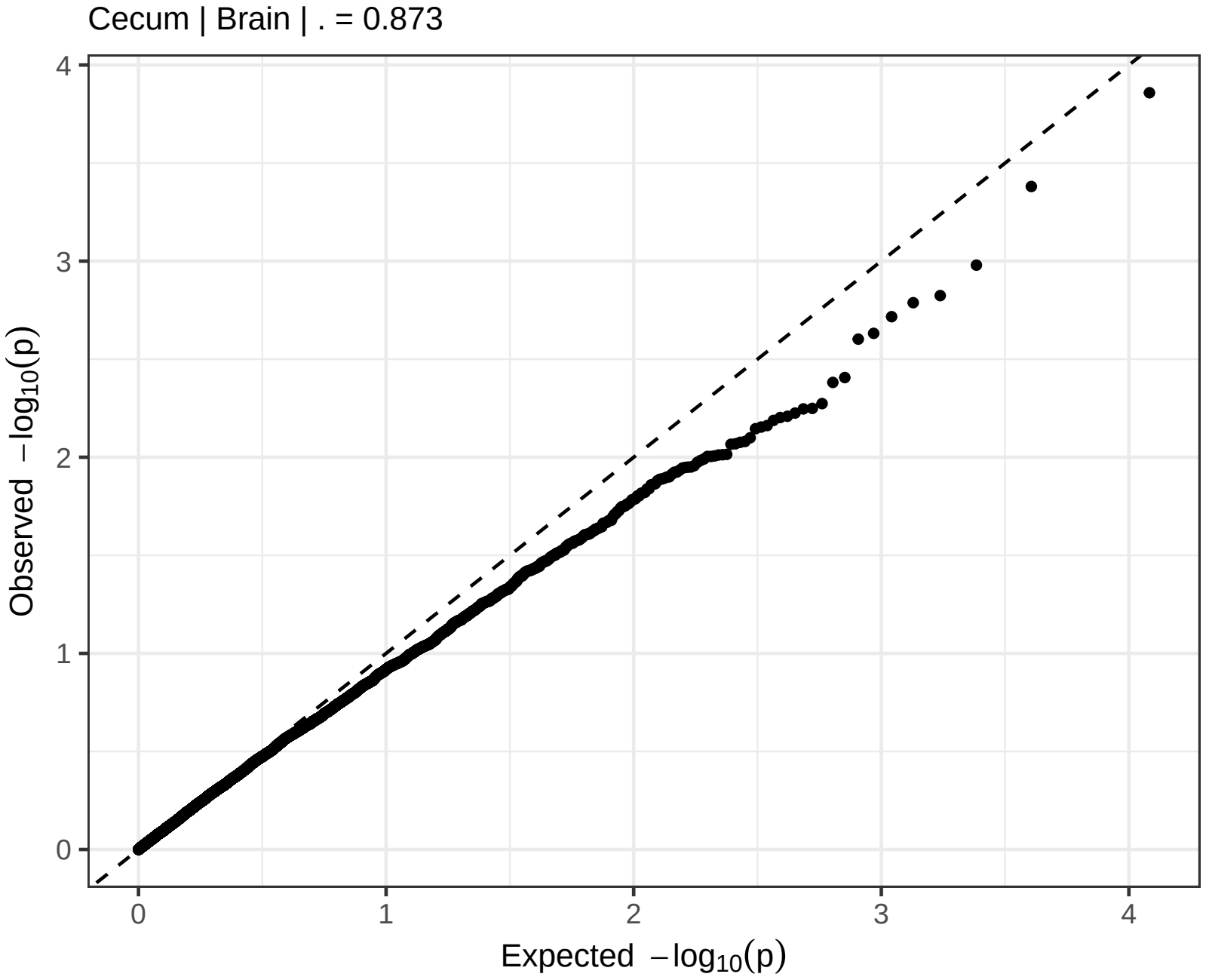

Angelakisella

Cecum | Brain | . = 0.895

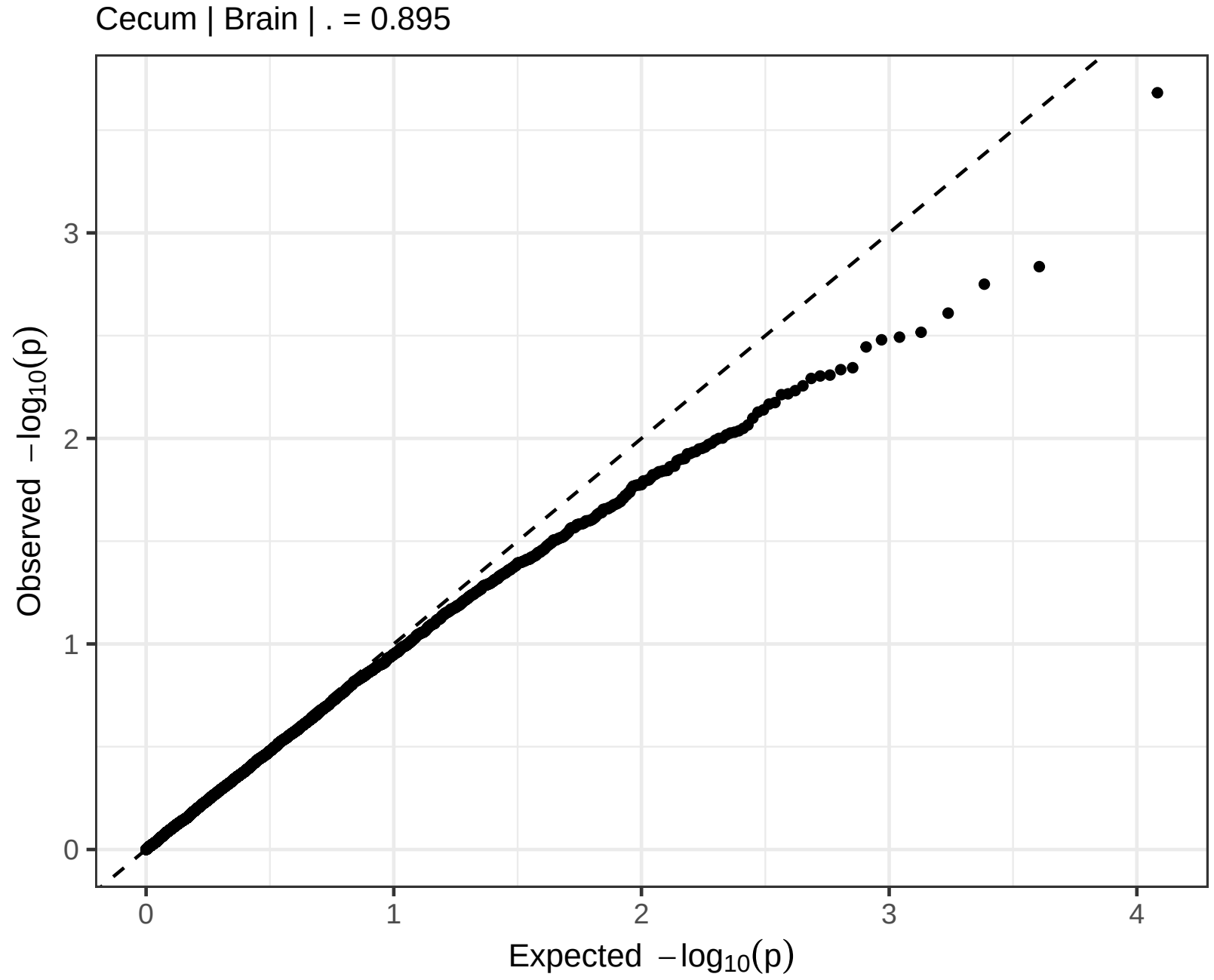

Bacteroides

Cecum | Brain | . = 0.897

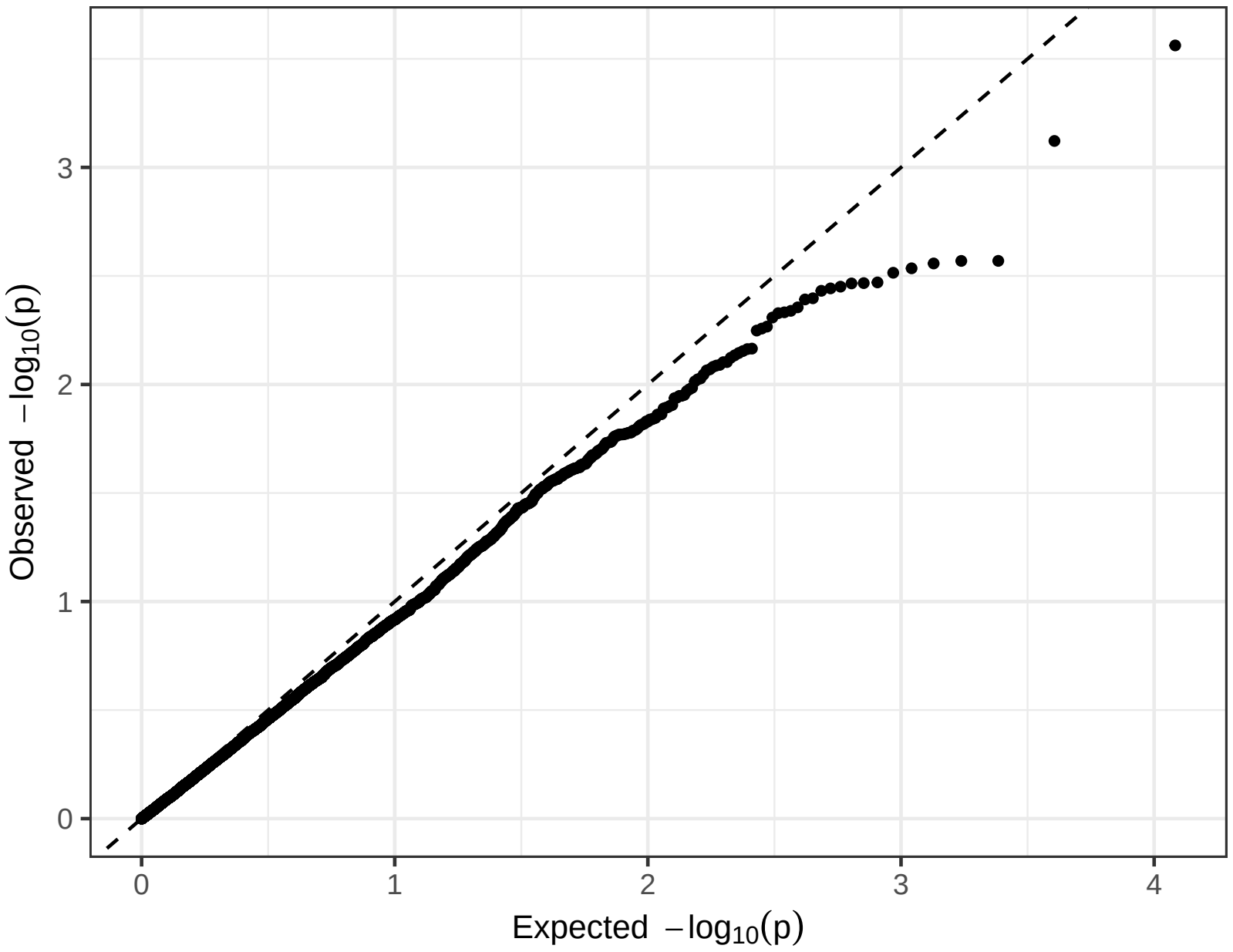

Blautia

Cecum | Brain | . = 0.898

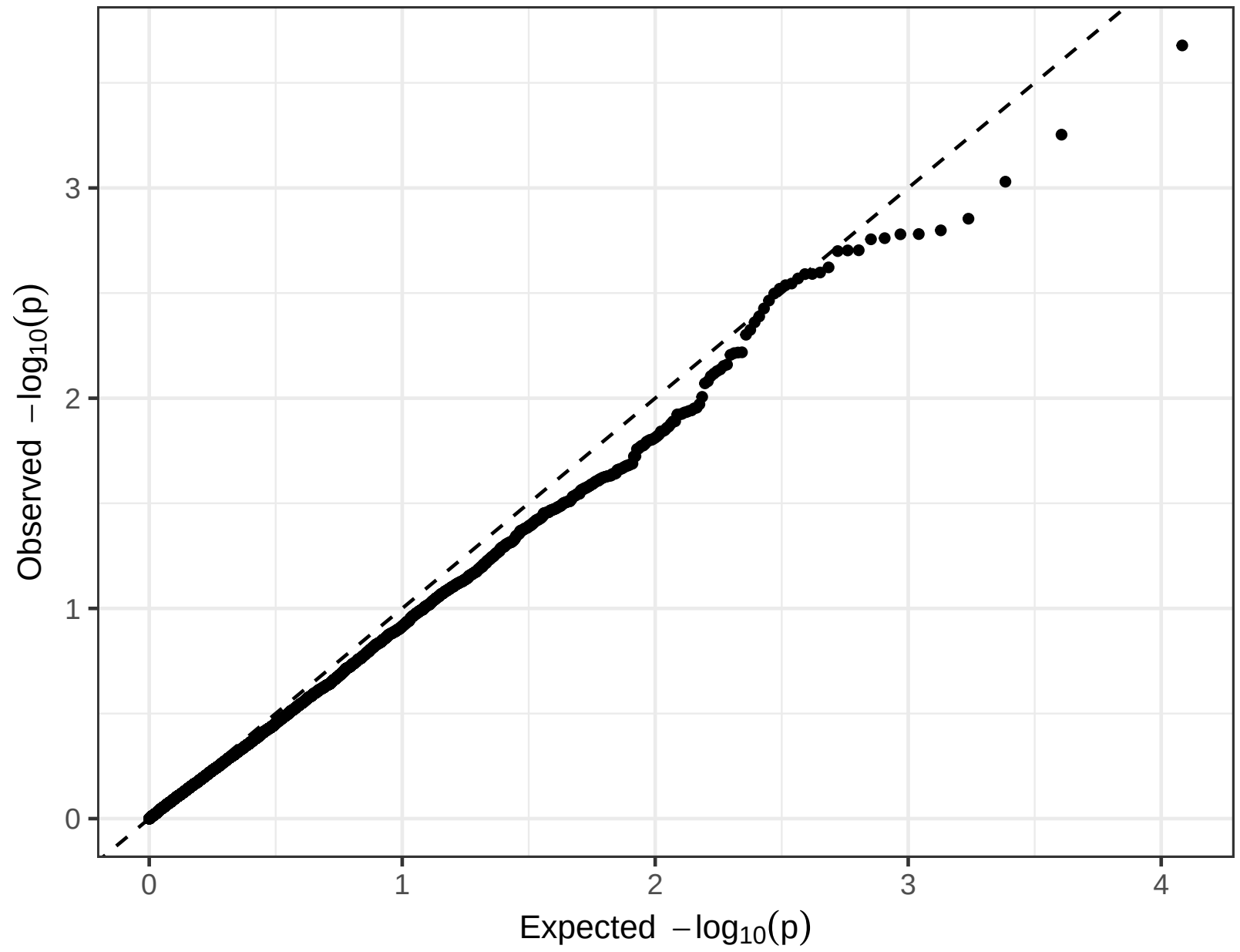

Breznakia

Cecum | Brain | . = 0.901

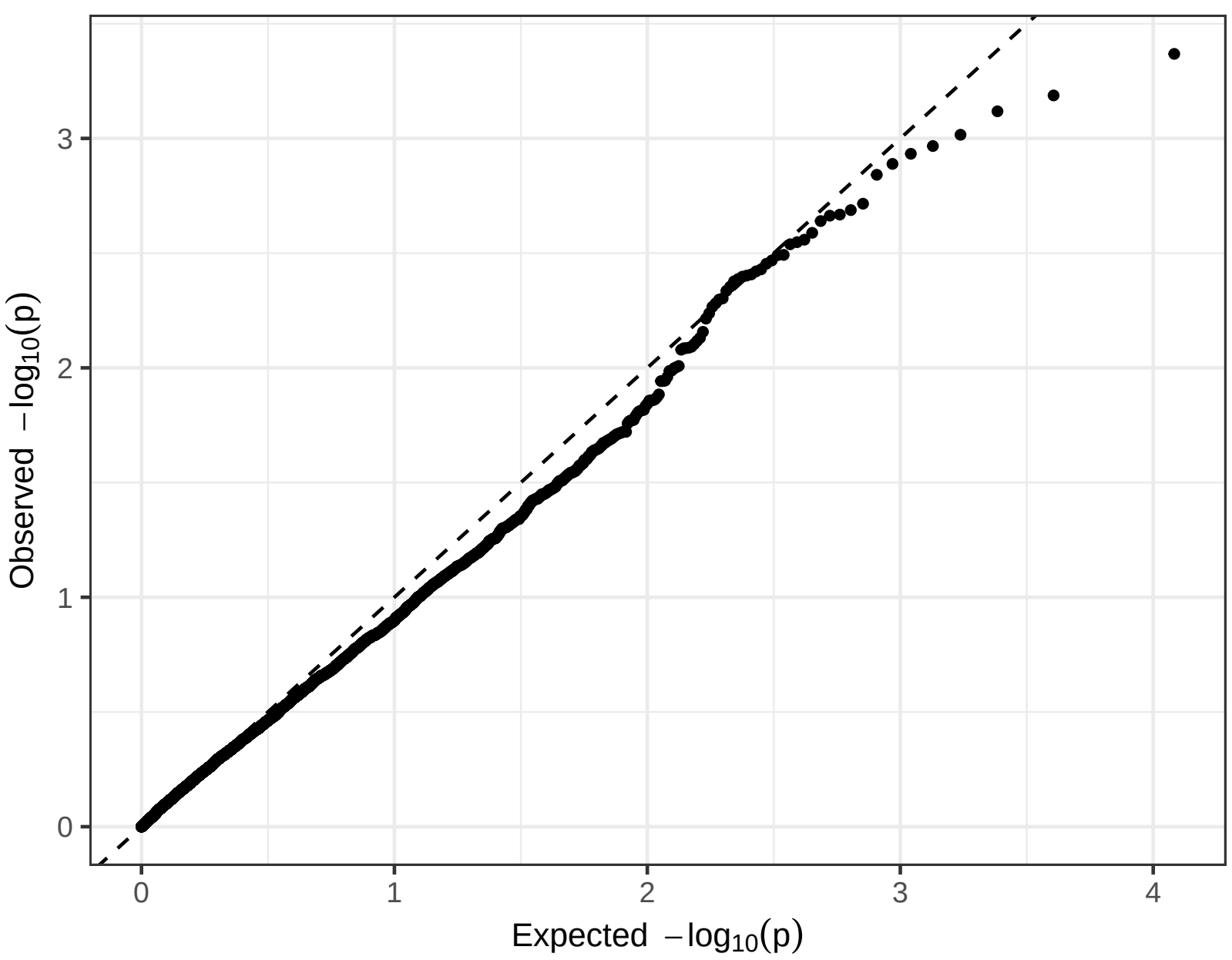

Butyribacter

Cecum | Brain | . = 0.838

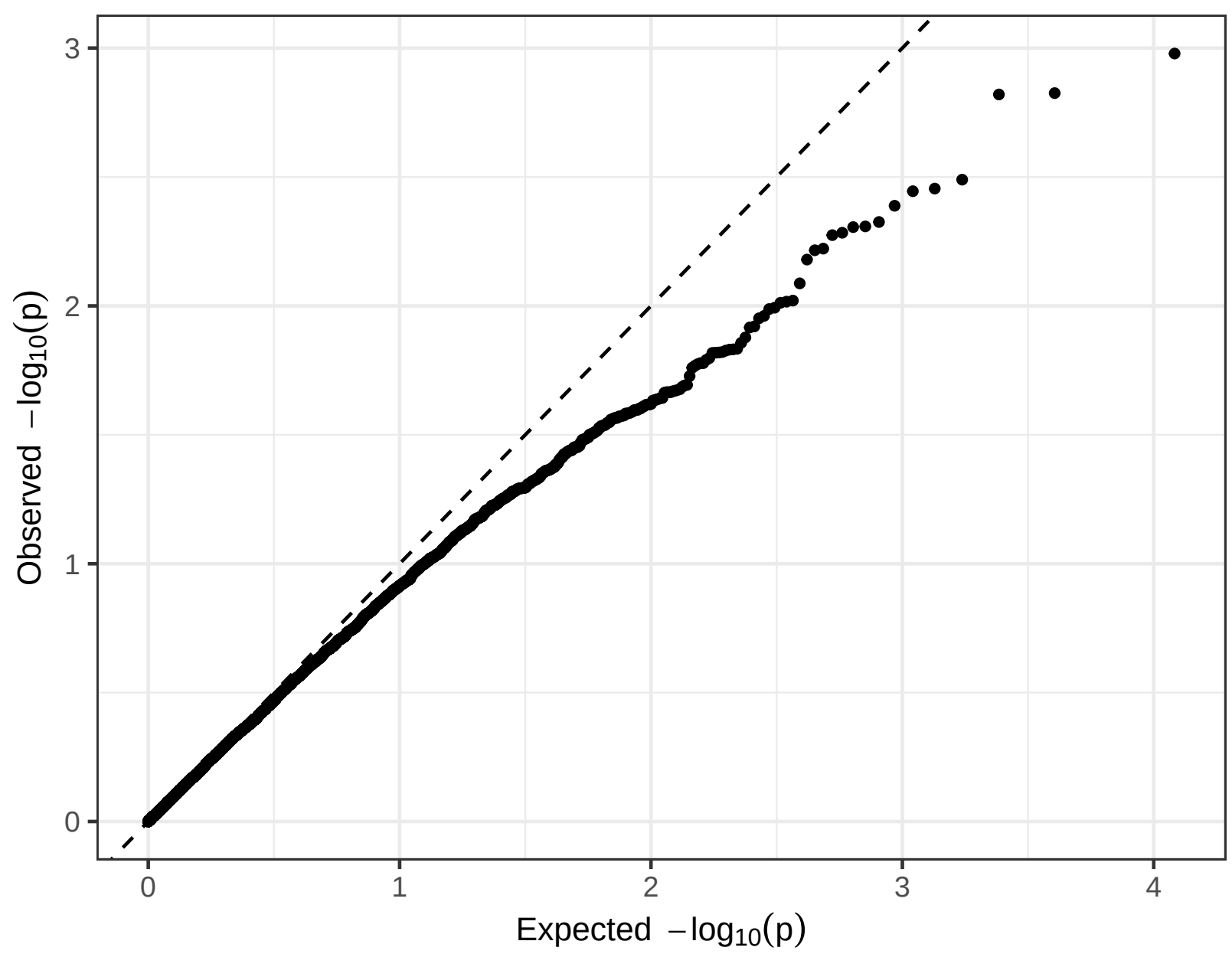

CAG.196

Cecum | Brain | . = 1.01

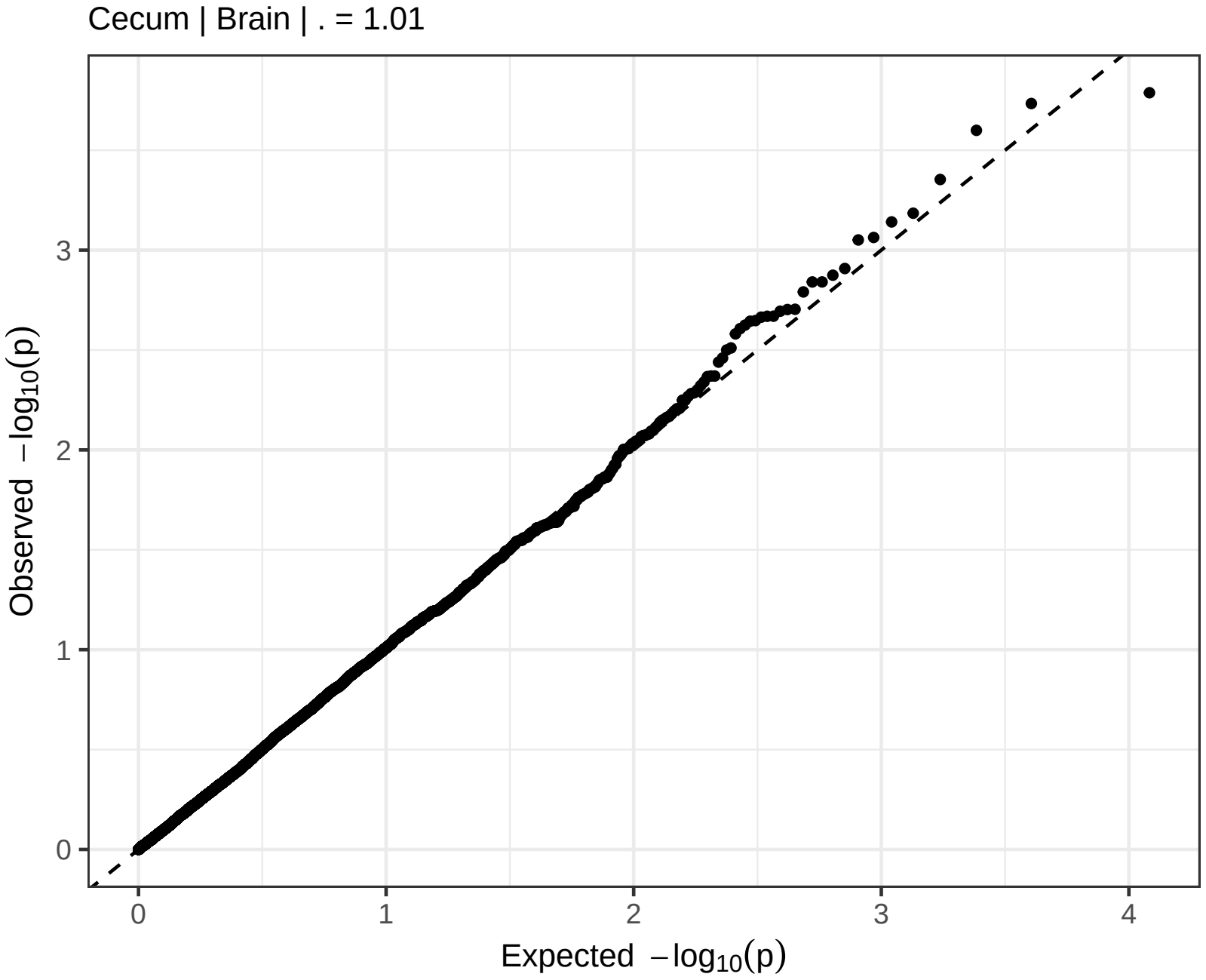

CAG.56

Cecum | Brain | . = 0.884

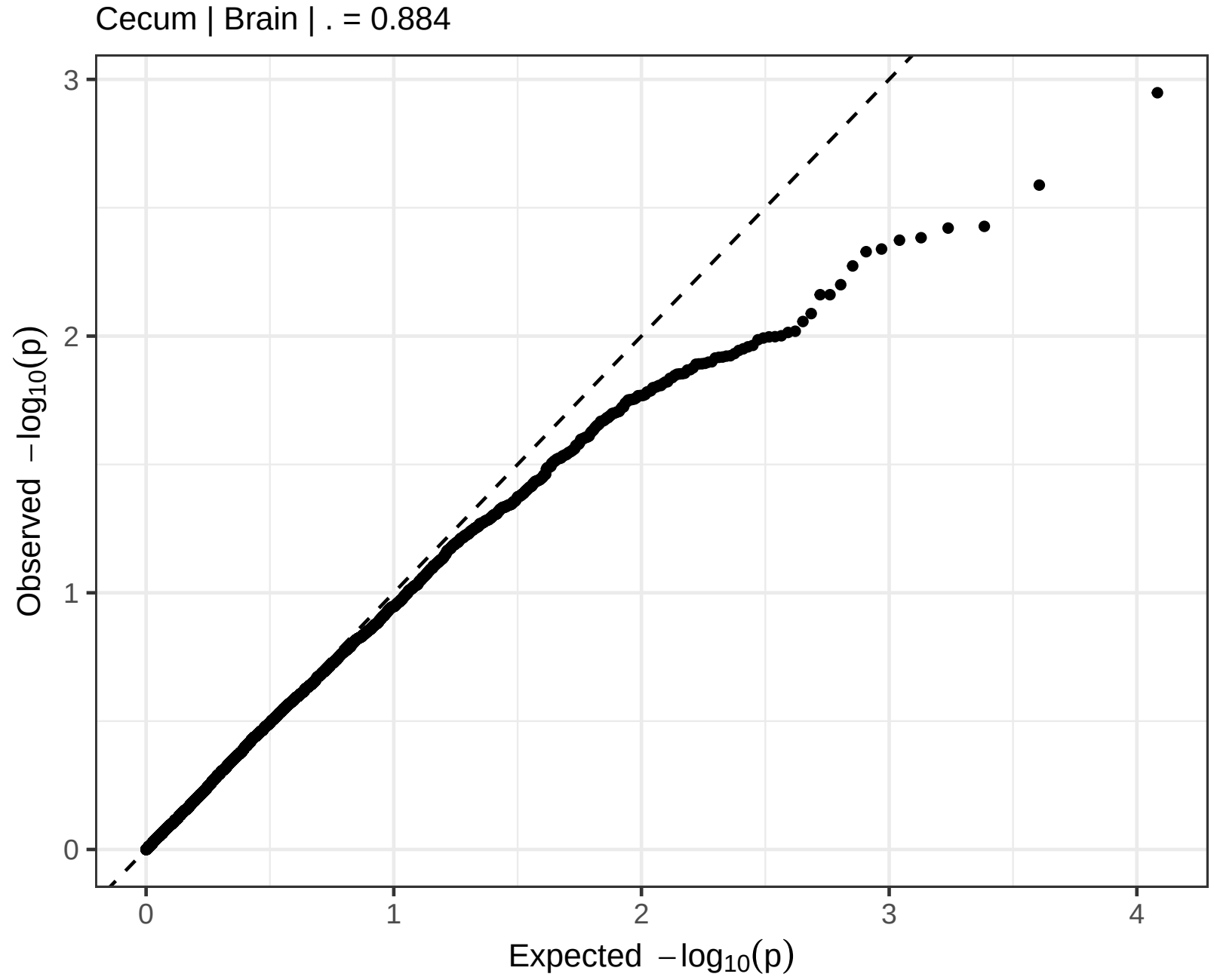

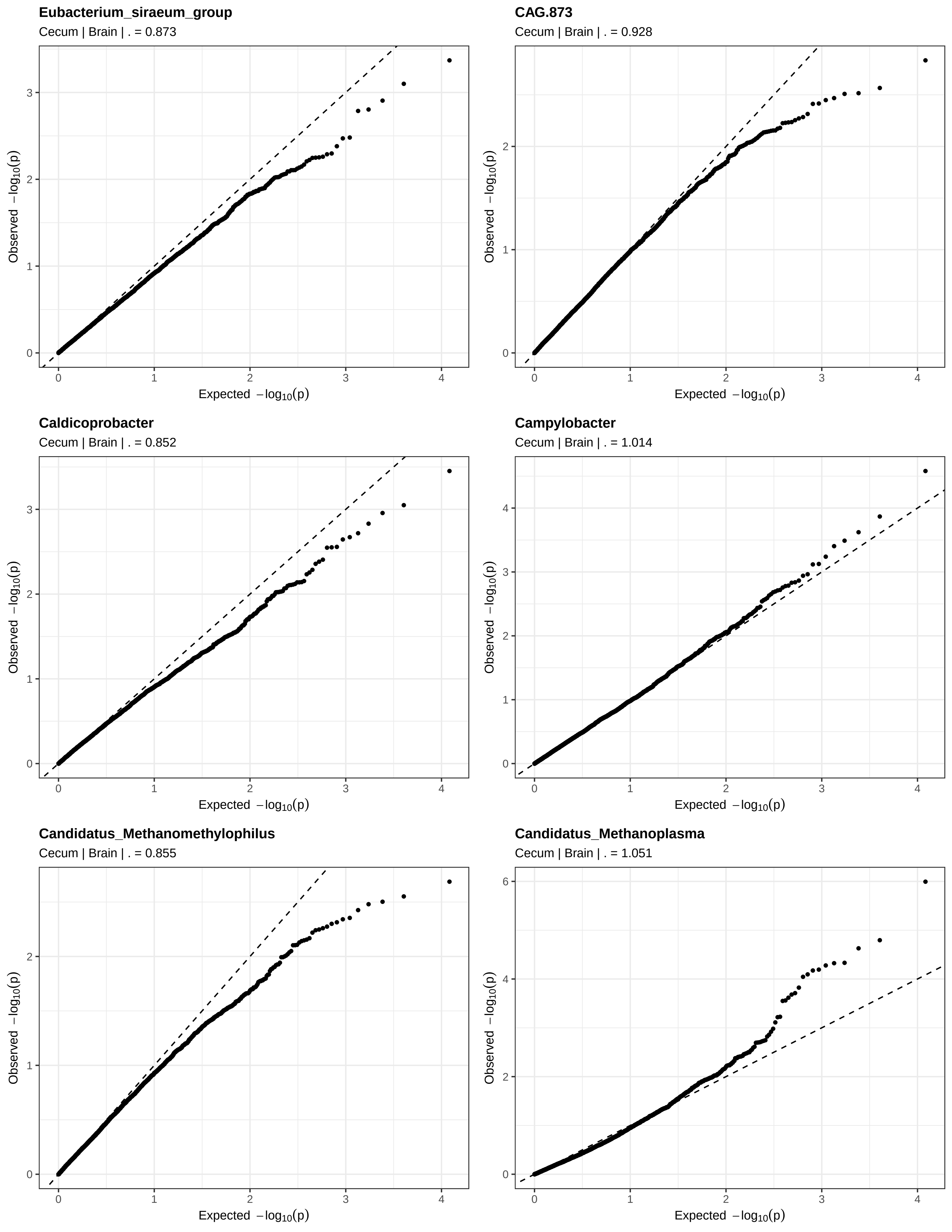

Eubacterium_siraeum_group
Cecum | Brain | . = 0.873
CAG.873
Cecum | Brain | . = 0.928
Caldicoprobacter
Cecum | Brain | . = 0.852
Campylobacter
Cecum | Brain | . = 1.014
Candidatus_Methanomethylophilus
Cecum | Brain | . = 0.855
Candidatus_Methanoplasma
Cecum | Brain | . = 1.051
Observed $-\log_{10}(p)$
Expected $-\log_{10}(p)$

Candidatus_Saccharimonas

Cecum | Brain | . = 0.866

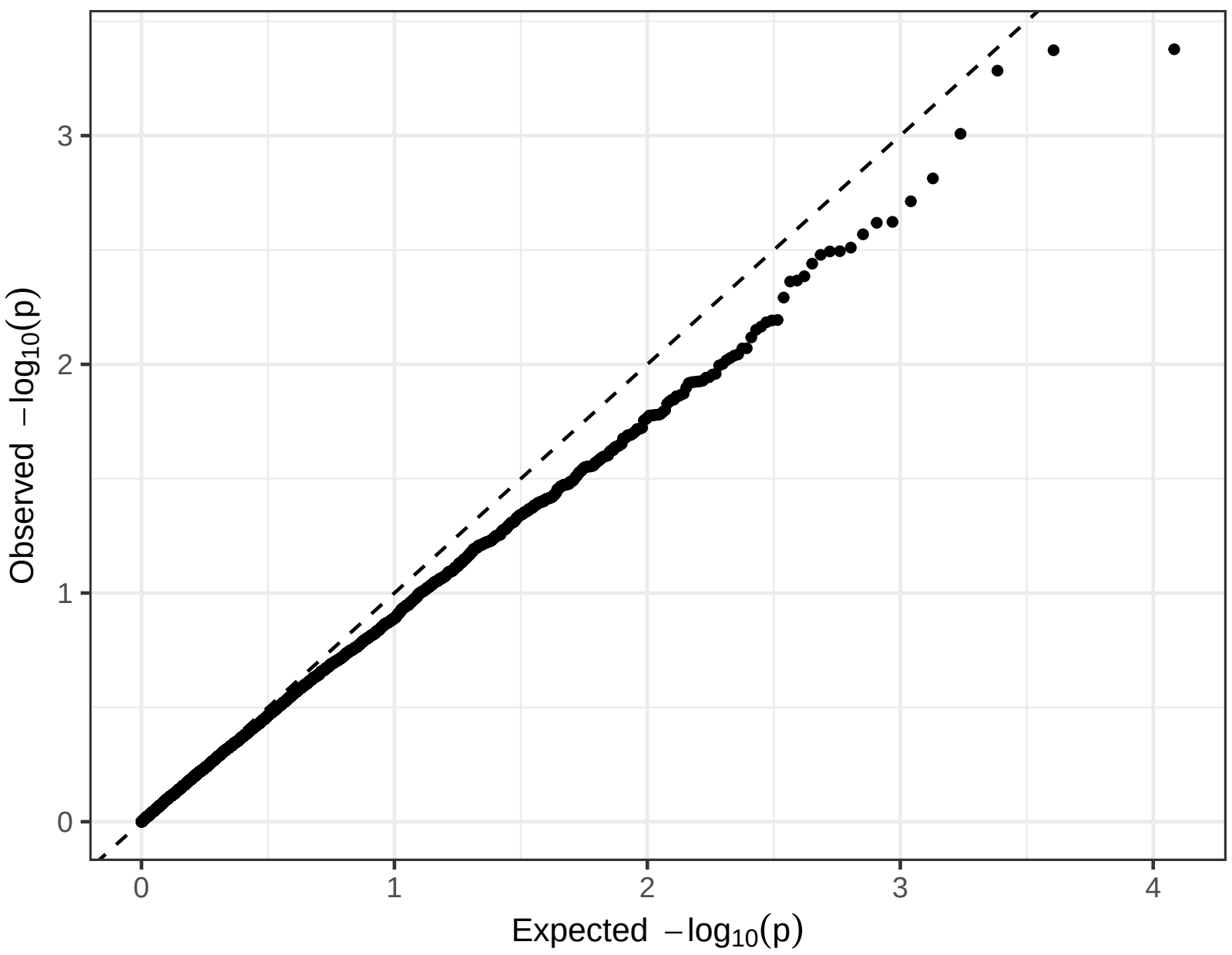

Candidatus_Soleaferrea

Cecum | Brain | . = 0.901

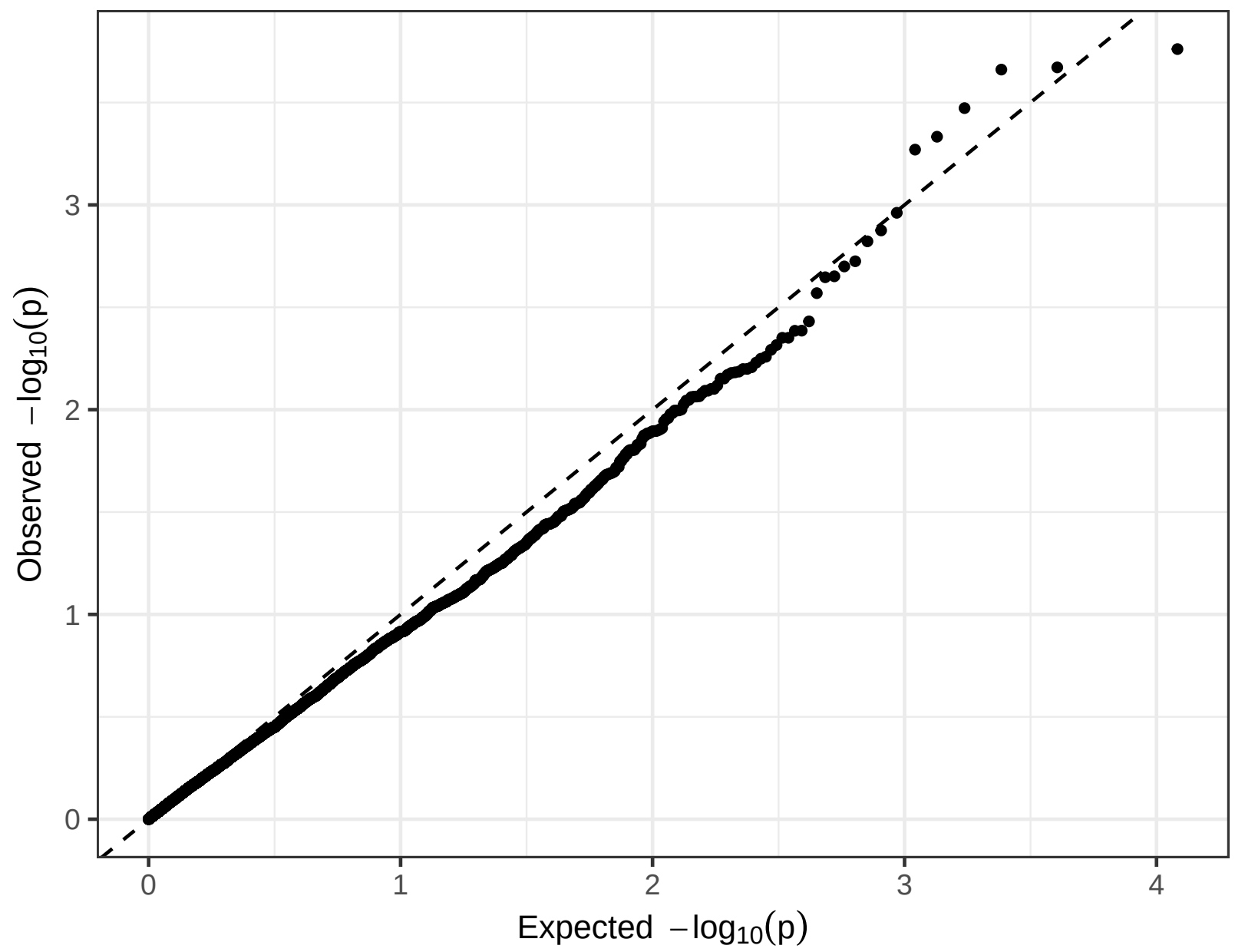

Caproiciproducens

Cecum | Brain | . = 1.128

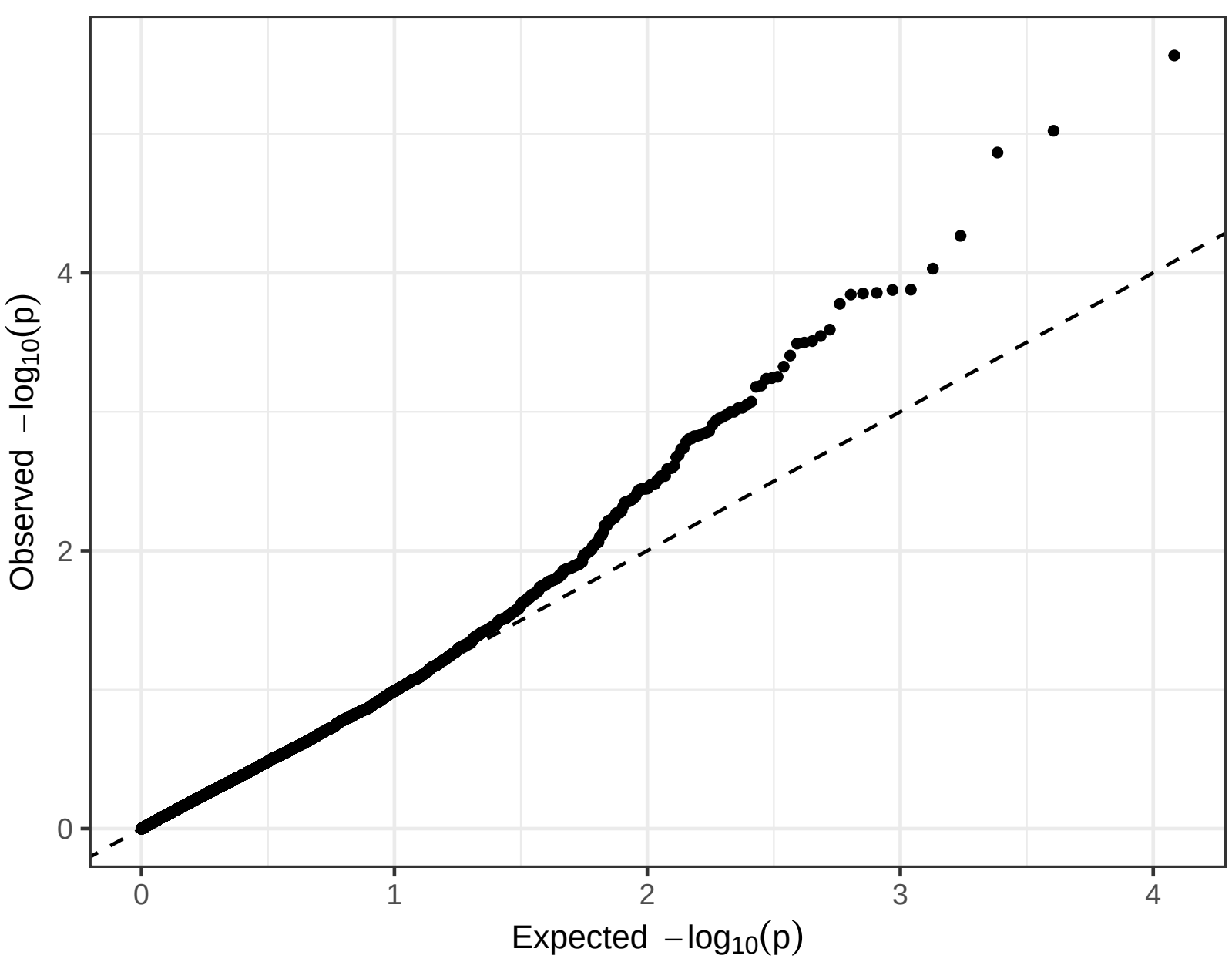

Catenibacterium

Cecum | Brain | . = 0.94

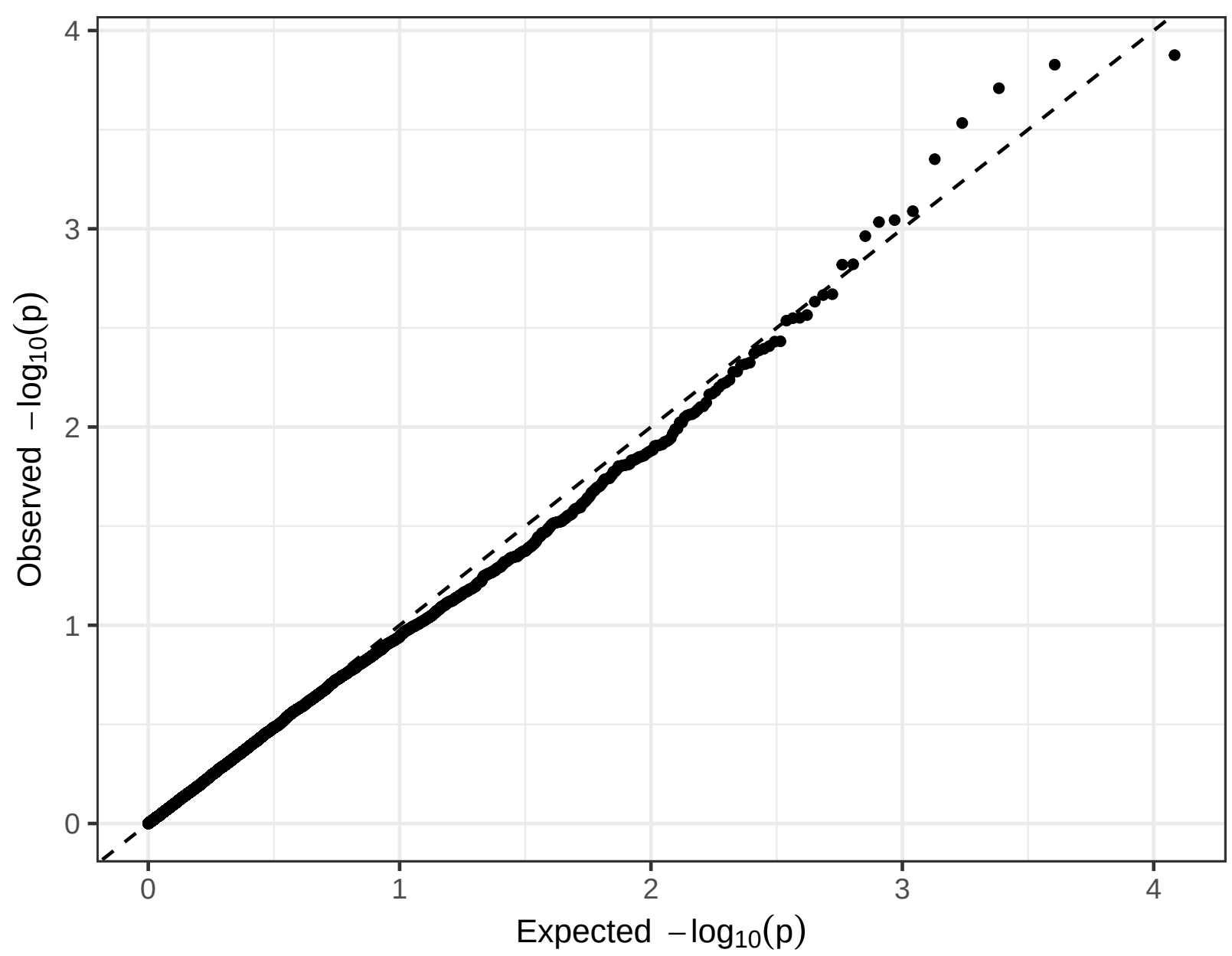

Catenisphaera

Cecum | Brain | . = 0.924

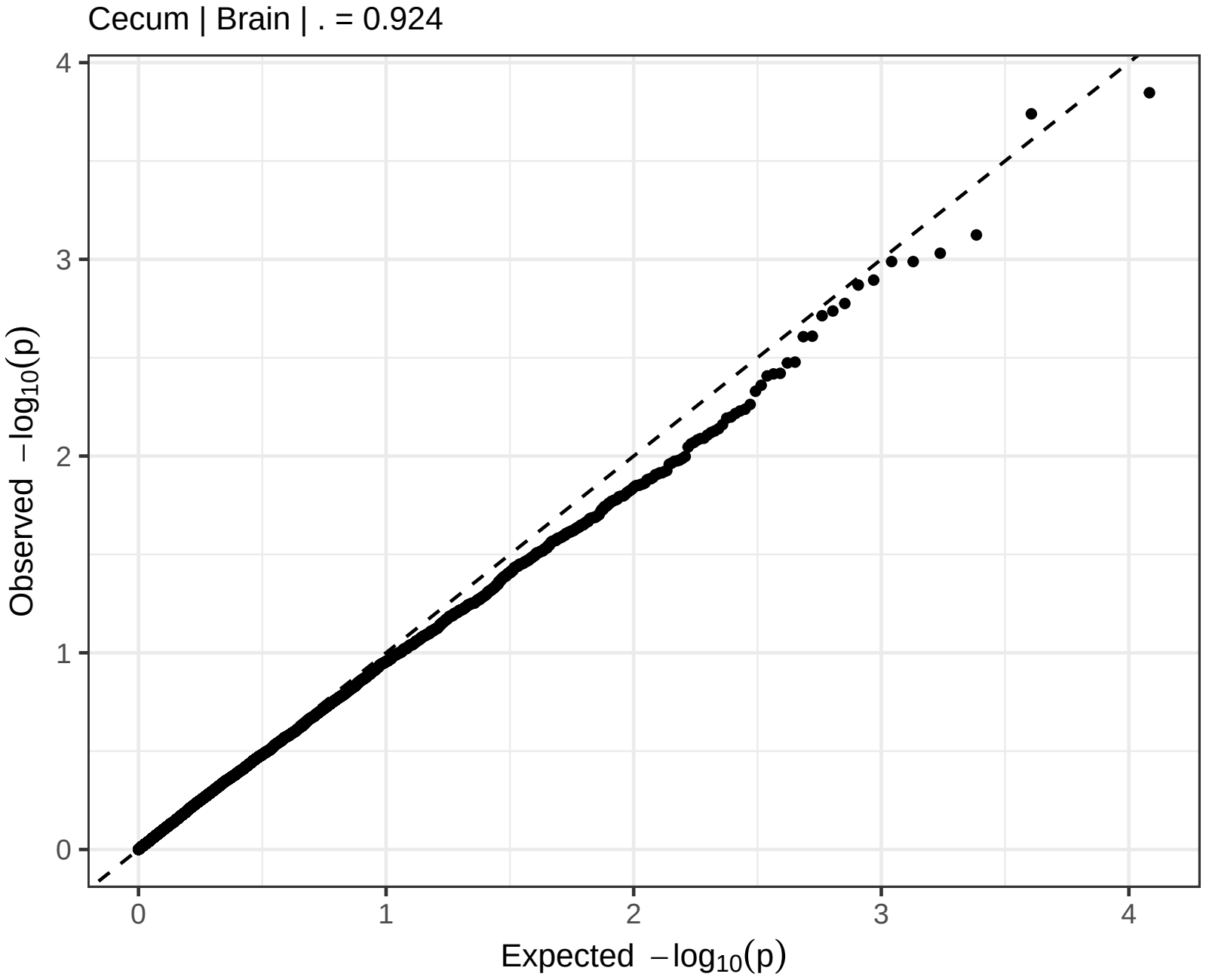

Eubacterium_xylanophilum_group

Cecum | Brain | . = 0.865

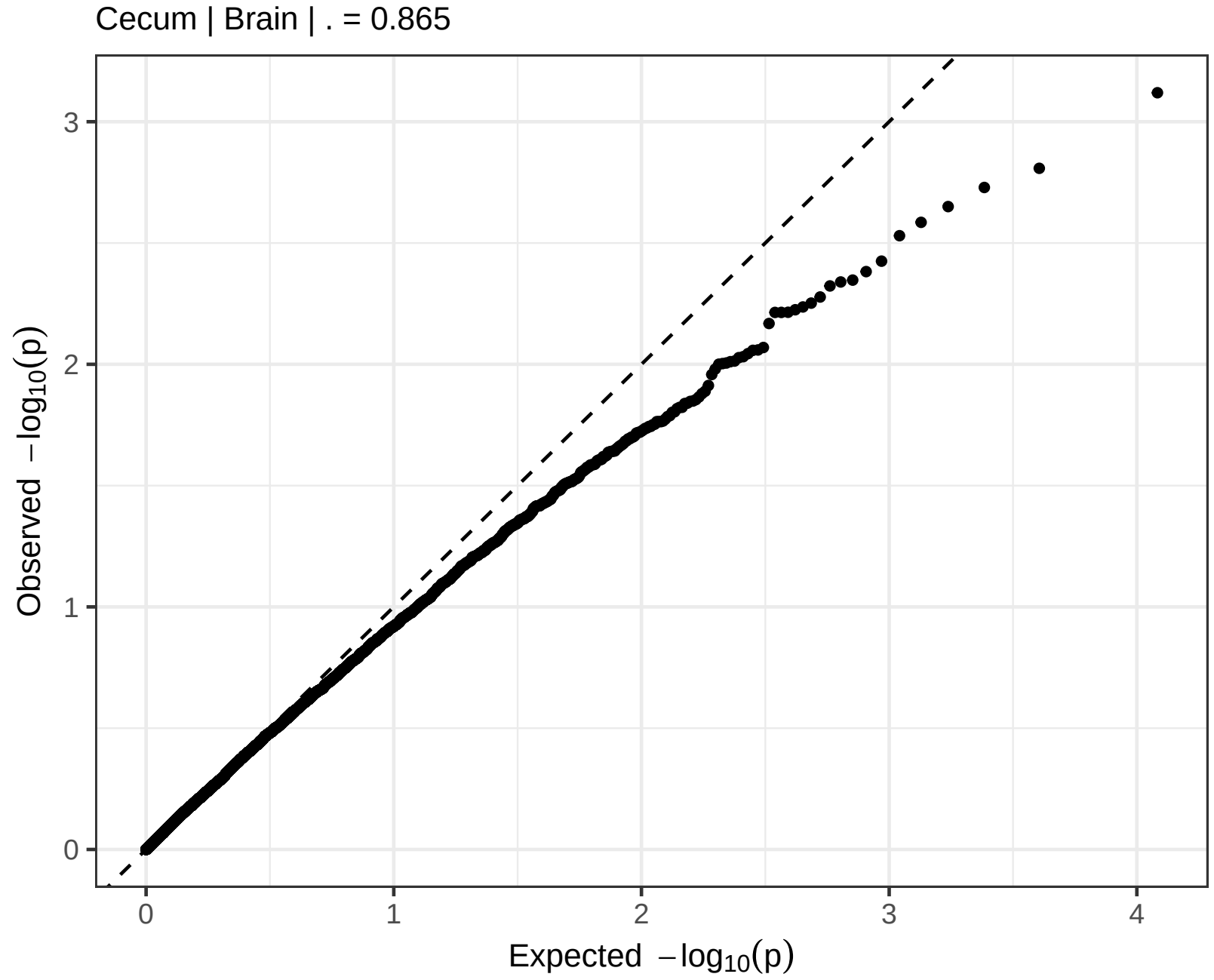

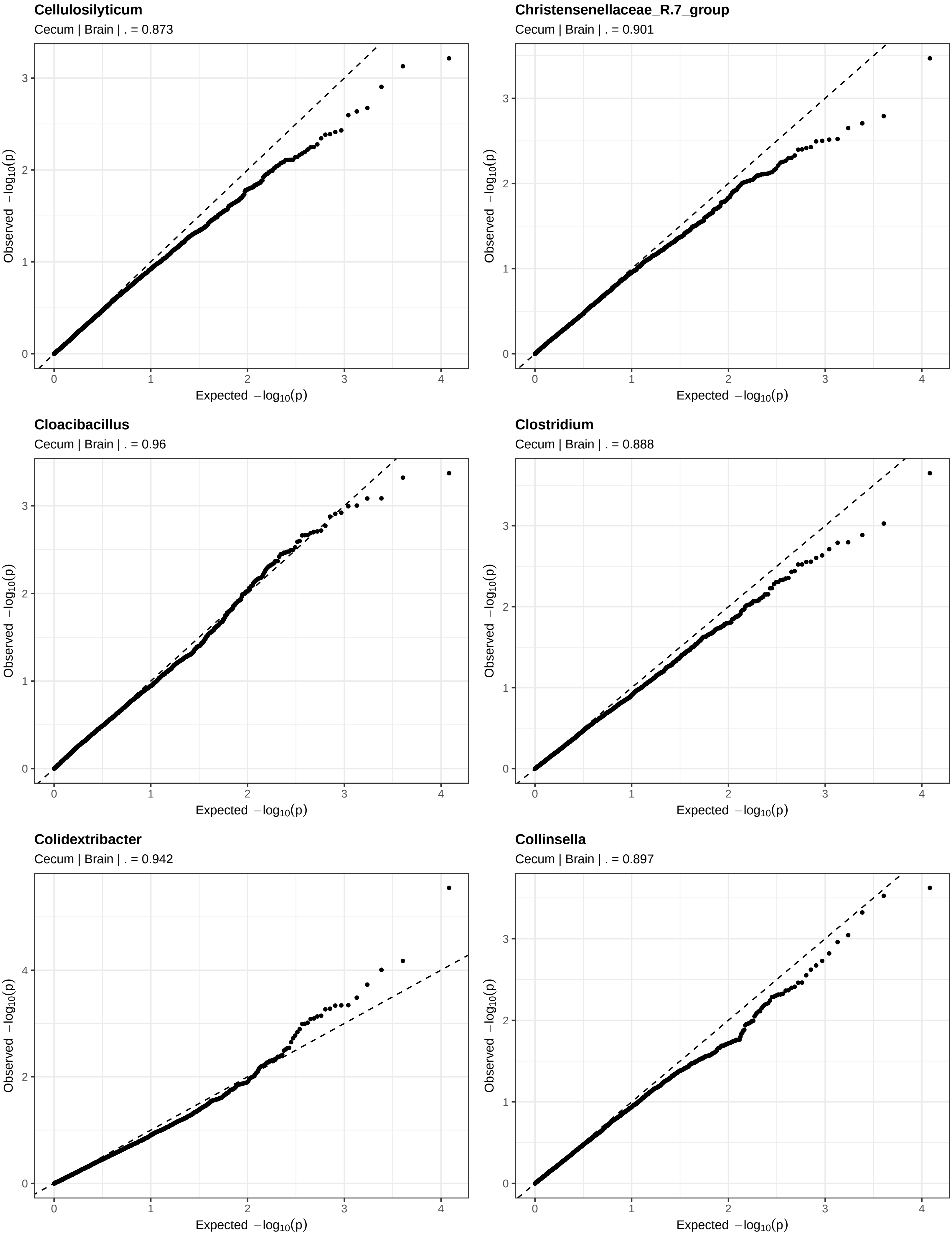
Cellulosilyticum
Cecum | Brain | . = 0.873
Christensenellaceae_R.7_group
Cecum | Brain | . = 0.901
Cloacibacillus
Cecum | Brain | . = 0.96
Clostridium
Cecum | Brain | . = 0.888
Colidextribacter
Cecum | Brain | . = 0.942
Collinsella
Cecum | Brain | . = 0.897
Observed $-\log_{10}(p)$
Expected $-\log_{10}(p)$

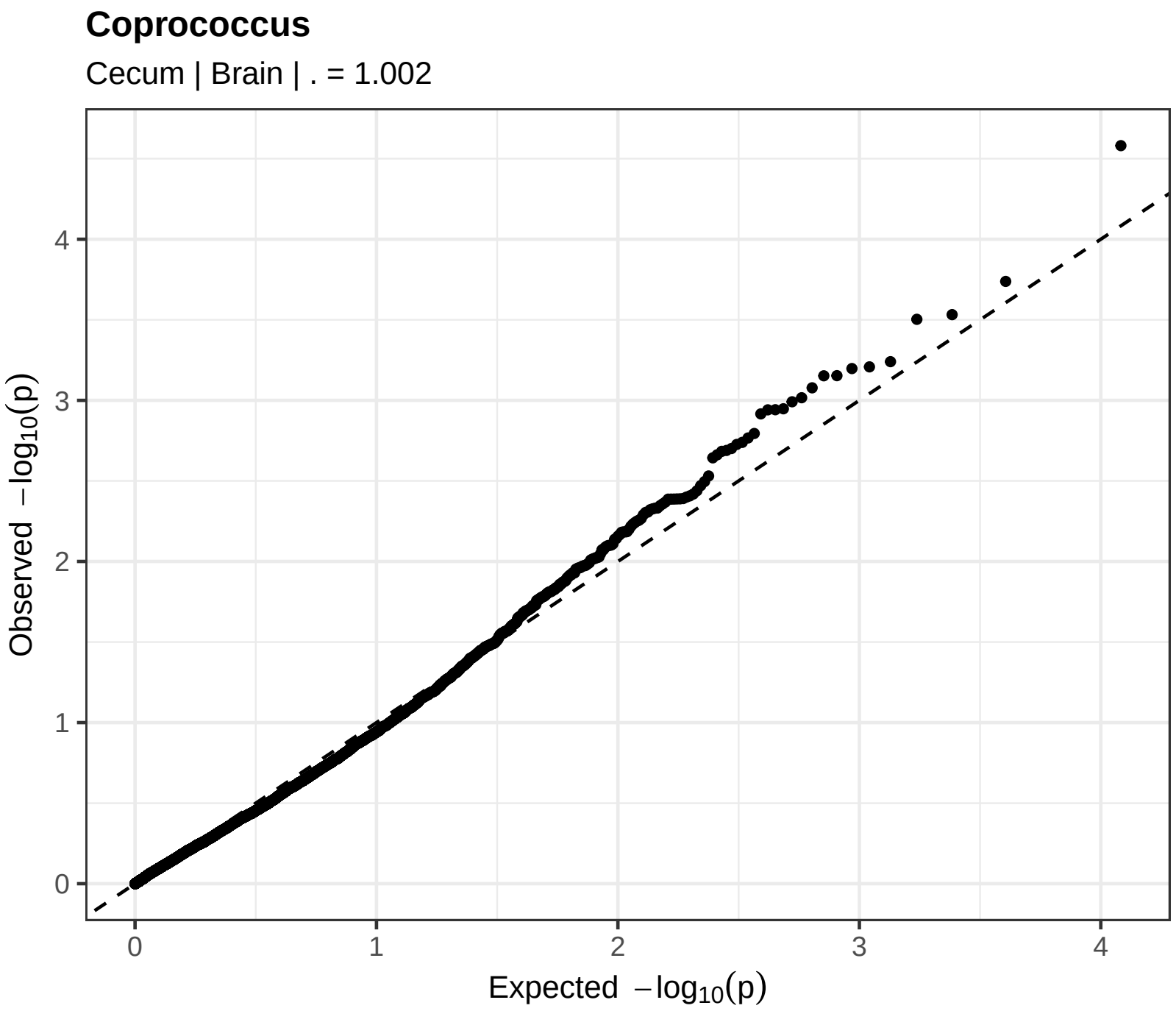
Coprococcus
Cecum | Brain | . = 1.002
Observed −log10(p)
Expected −log10(p)

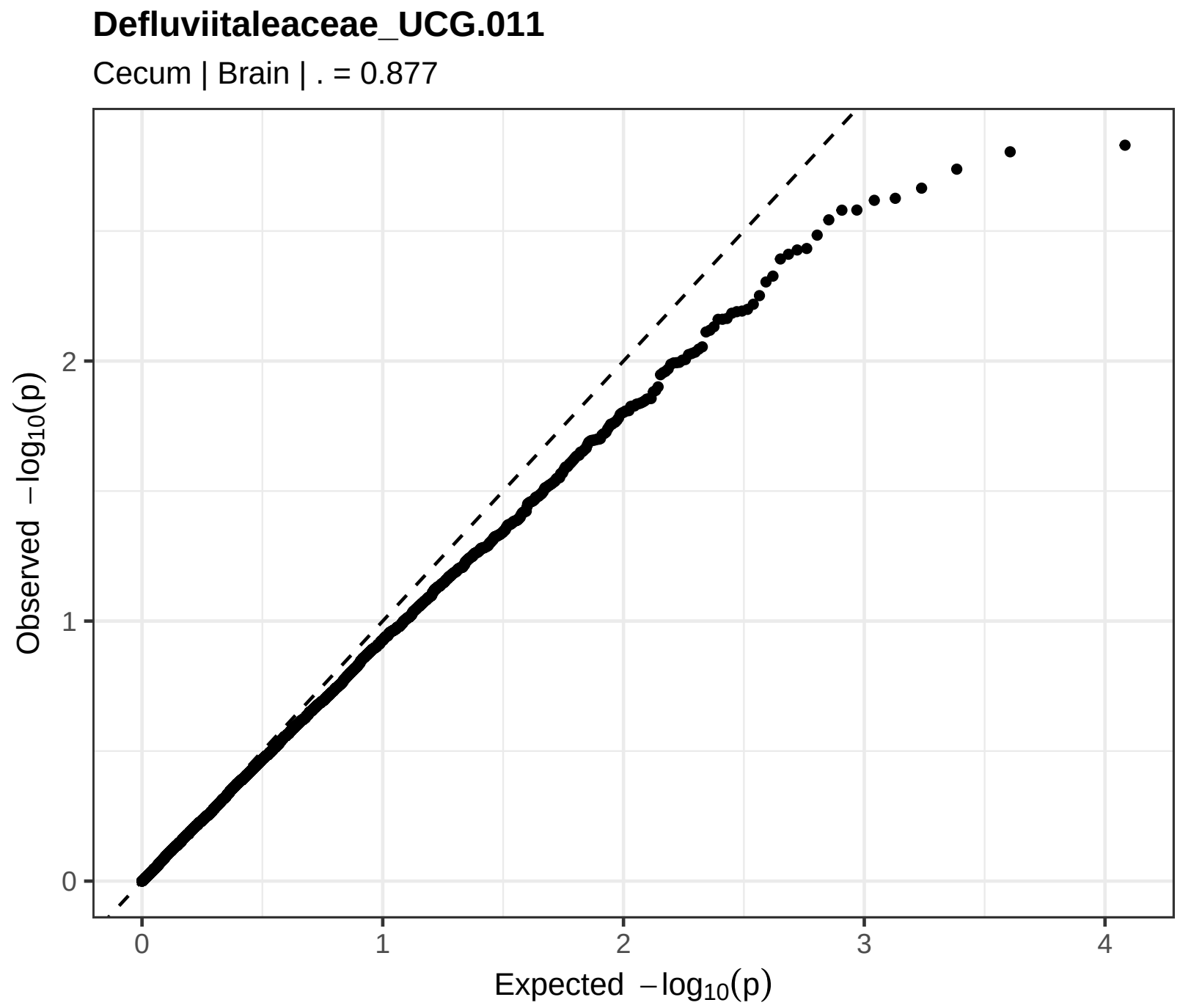
Defluviitaleaceae_UCG.011
Cecum | Brain | . = 0.877
Observed −log10(p)
Expected −log10(p)

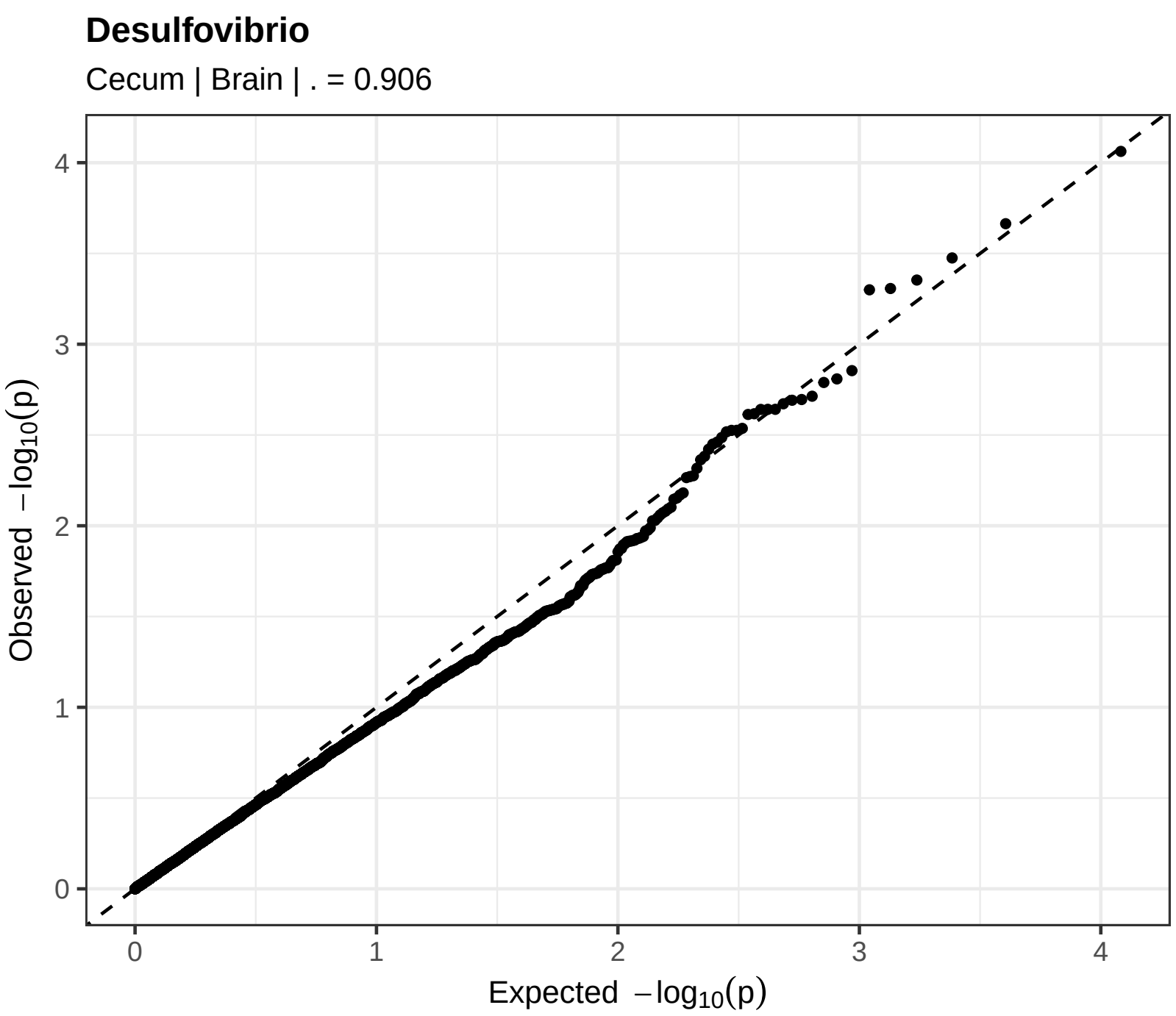
Desulfovibrio
Cecum | Brain | . = 0.906
Observed −log10(p)
Expected −log10(p)

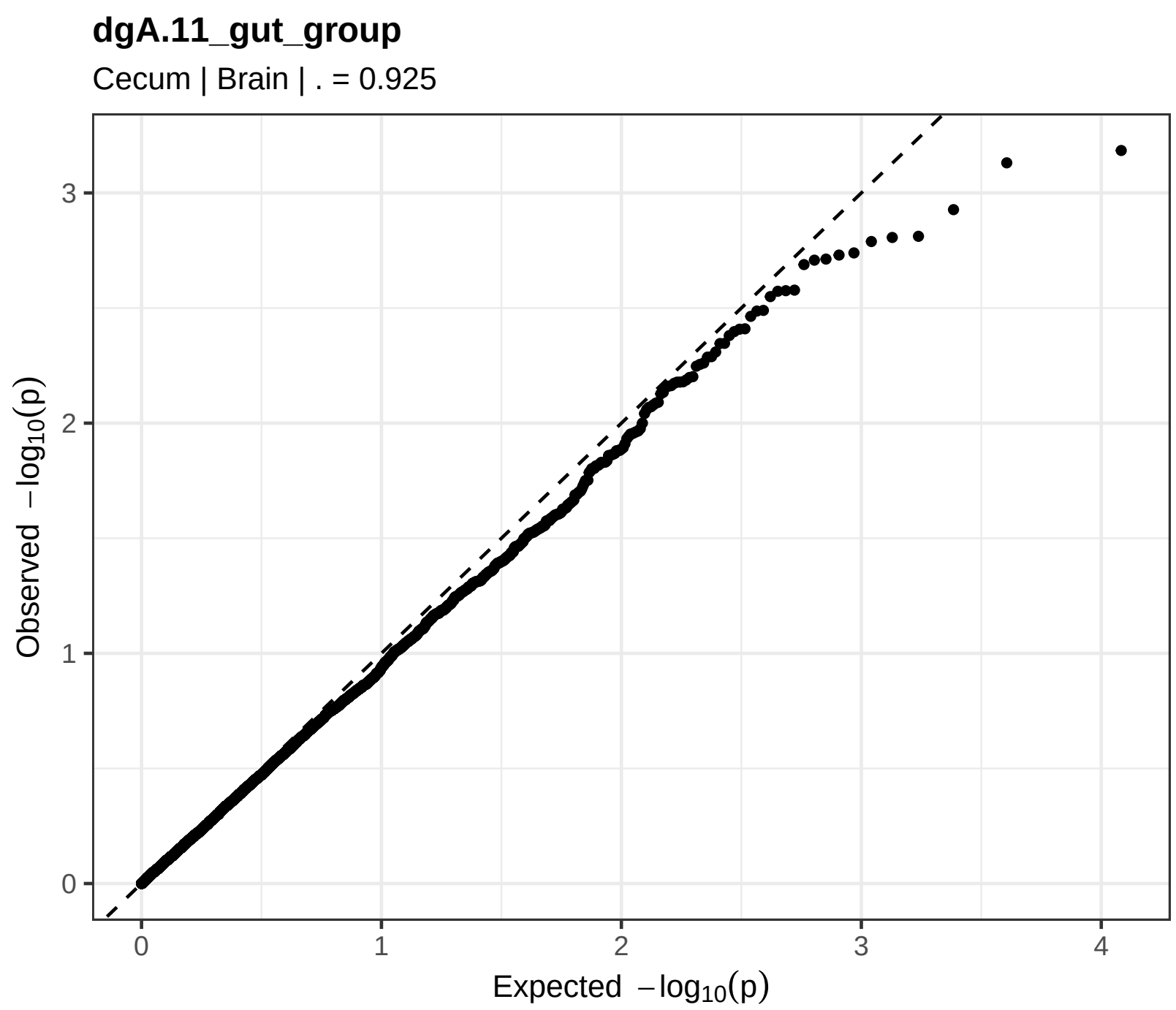
dgA.11_gut_group
Cecum | Brain | . = 0.925
Observed −log10(p)
Expected −log10(p)

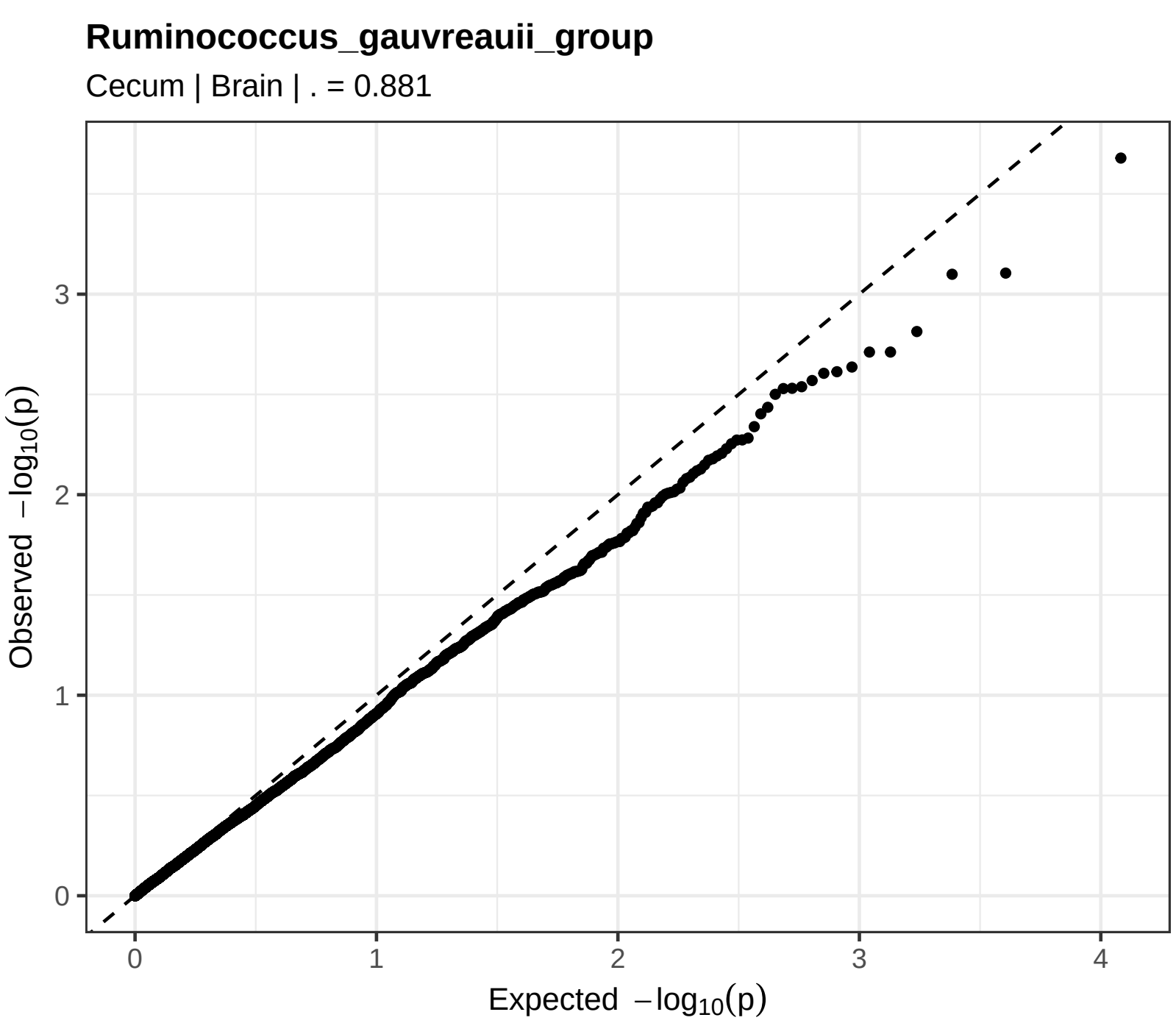
Ruminococcus_gauvreauii_group
Cecum | Brain | . = 0.881
Observed −log10(p)
Expected −log10(p)

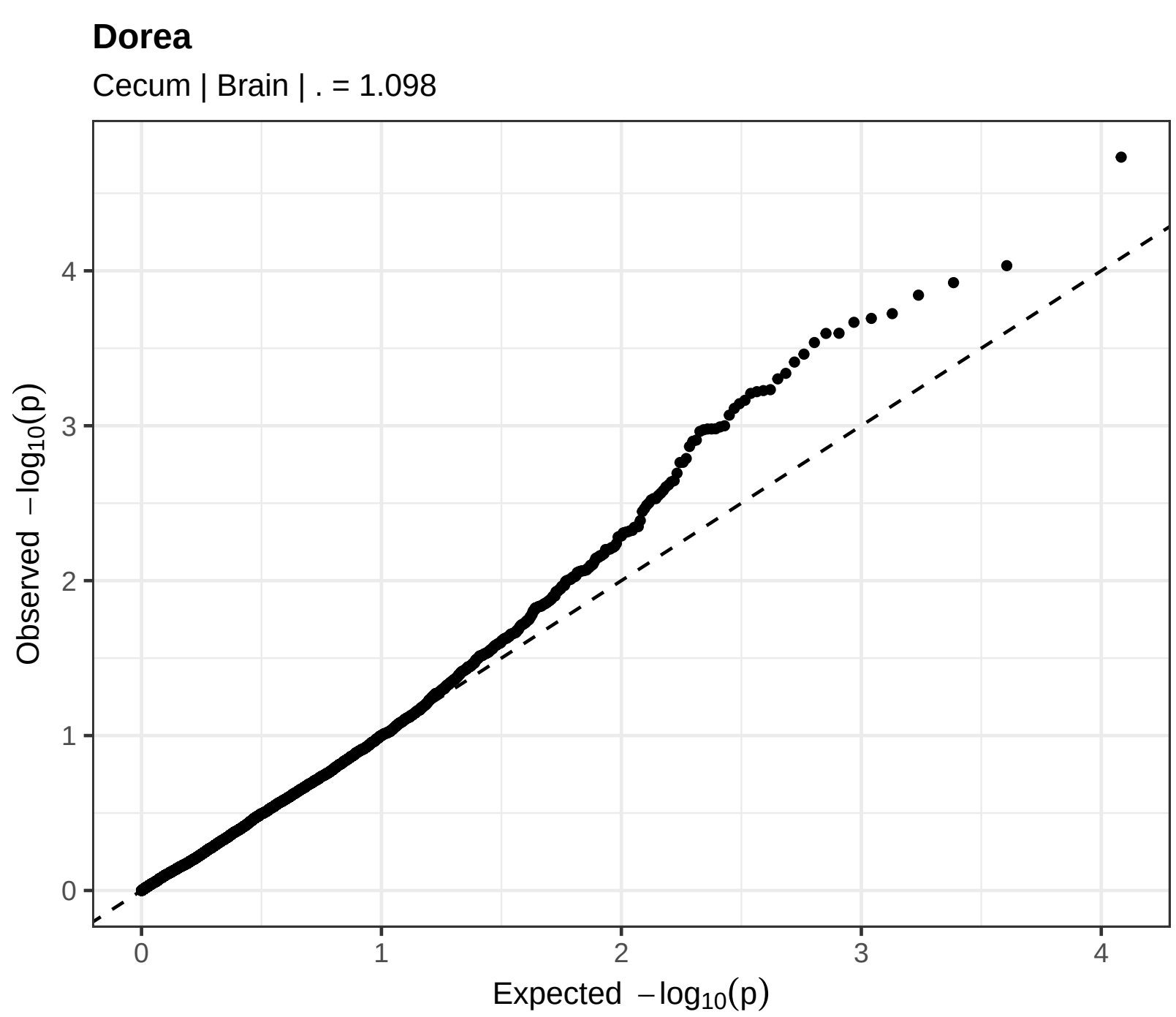
Dorea
Cecum | Brain | . = 1.098
Observed −log10(p)
Expected −log10(p)

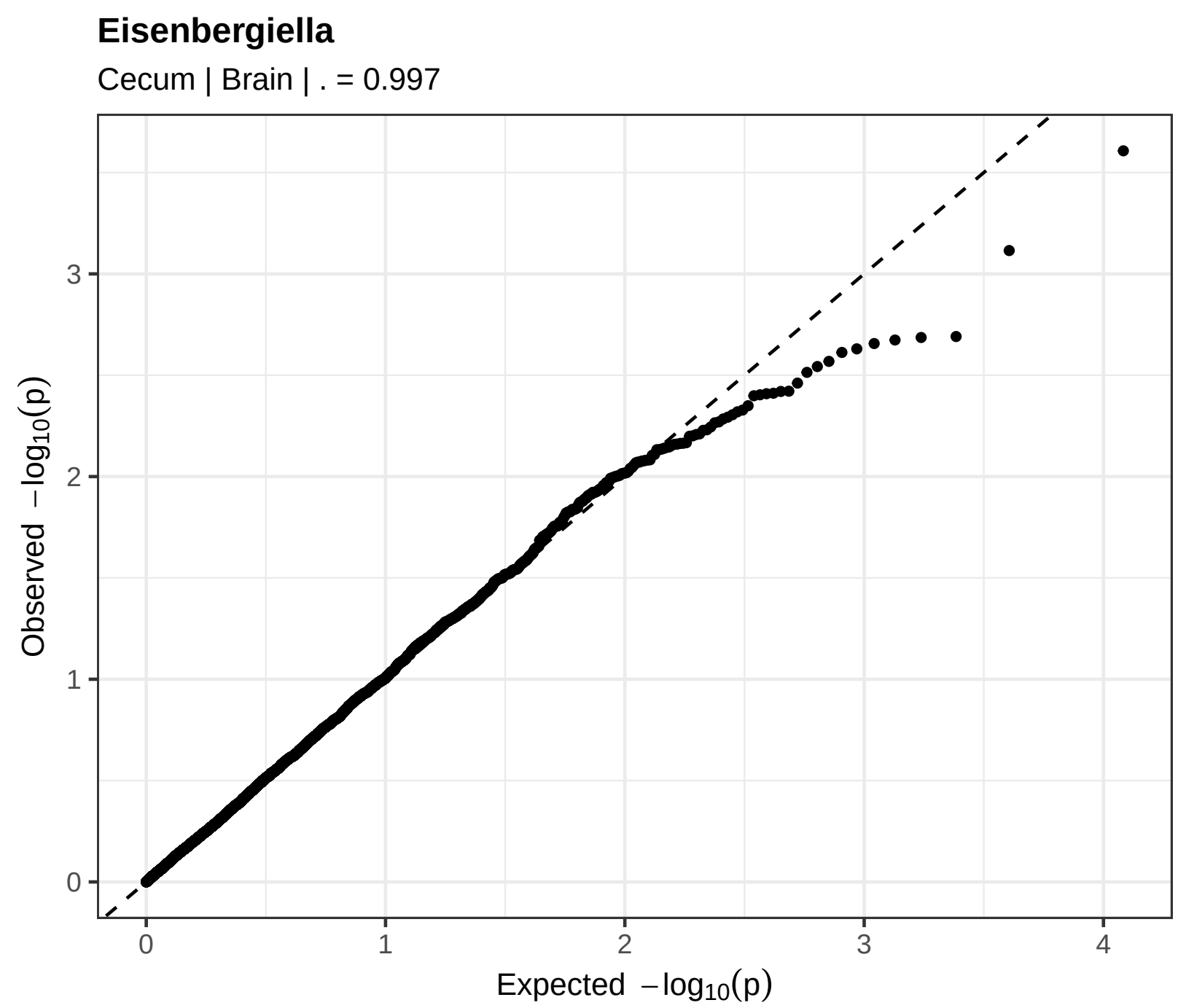
Eisenbergiella
Cecum | Brain | . = 0.997
Observed −log10(p)
Expected −log10(p)

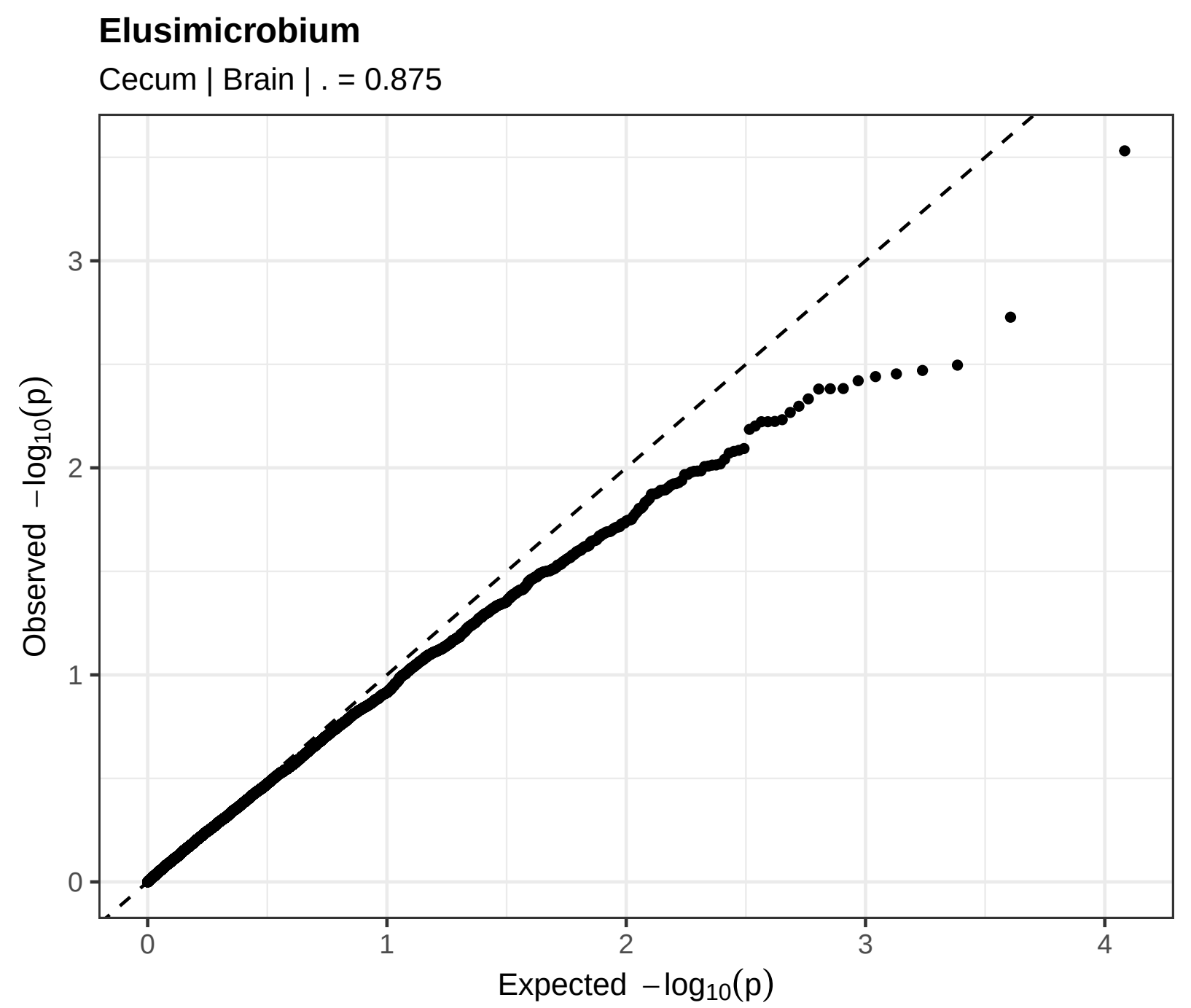
Elusimicrobium
Cecum | Brain | . = 0.875
Observed −log10(p)
Expected −log10(p)

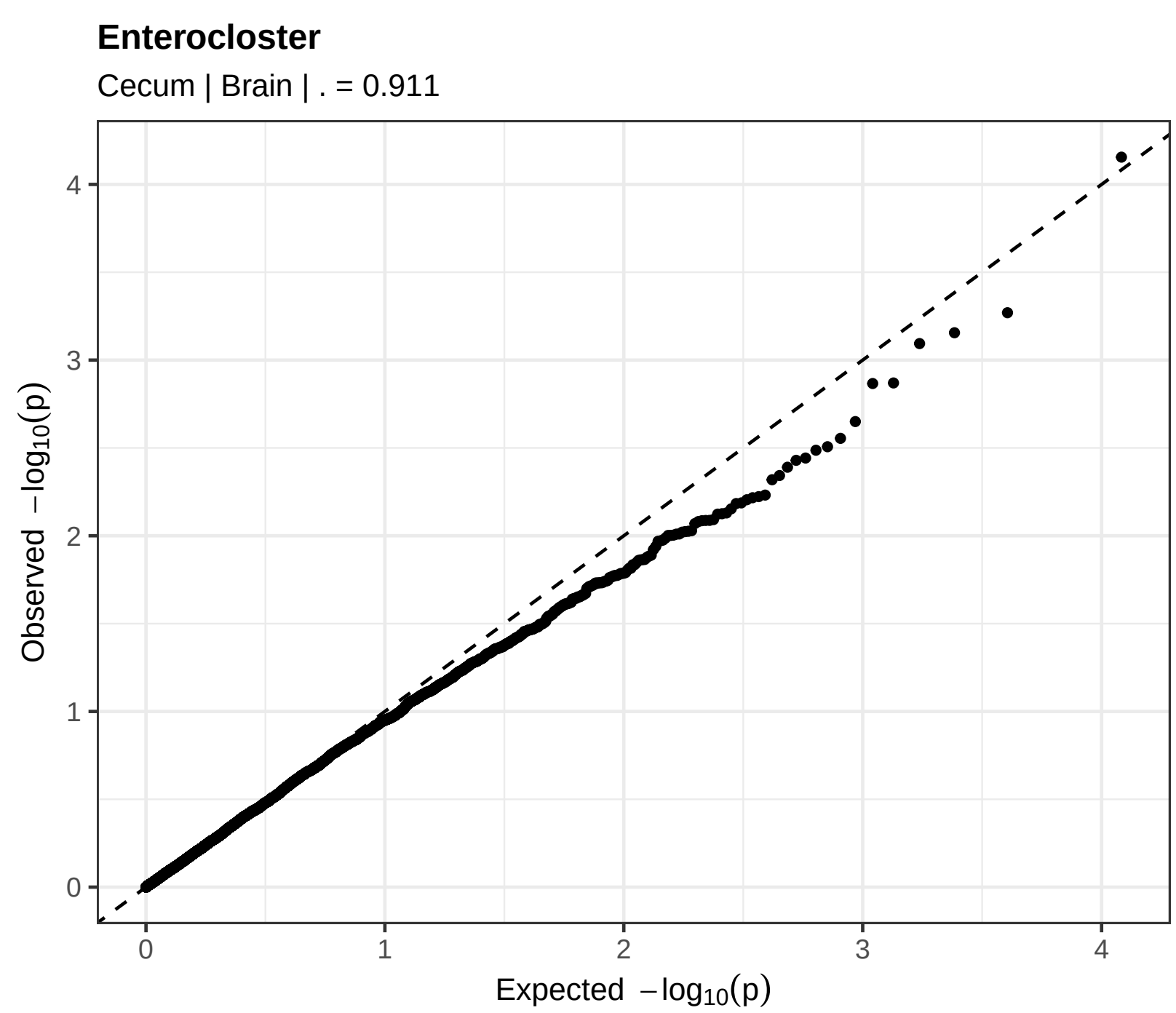
Enterocloster
Cecum | Brain | . = 0.911
Observed −log10(p)
Expected −log10(p)

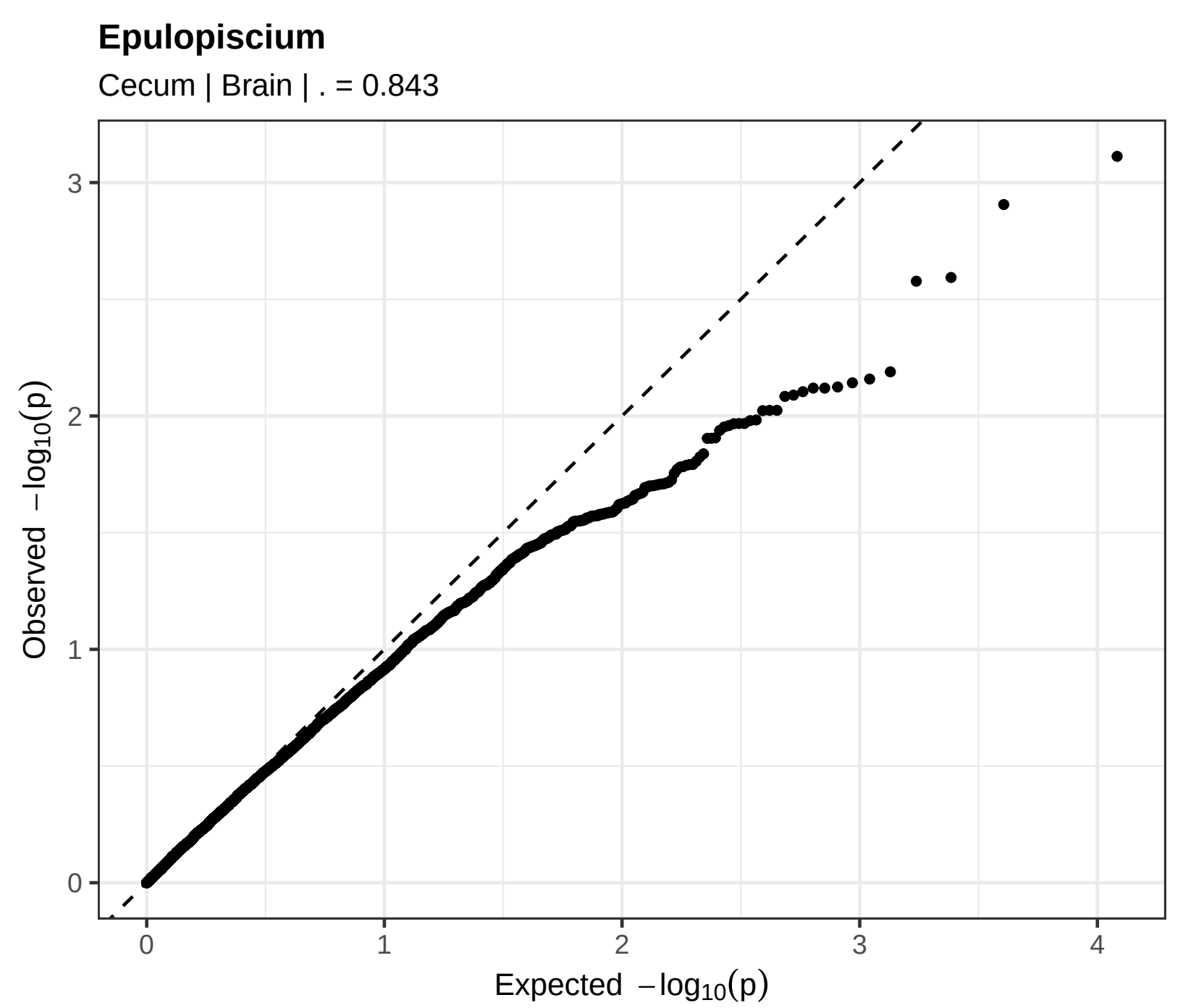
Epulopiscium
Cecum | Brain | . = 0.843
Observed −log10(p)
Expected −log10(p)

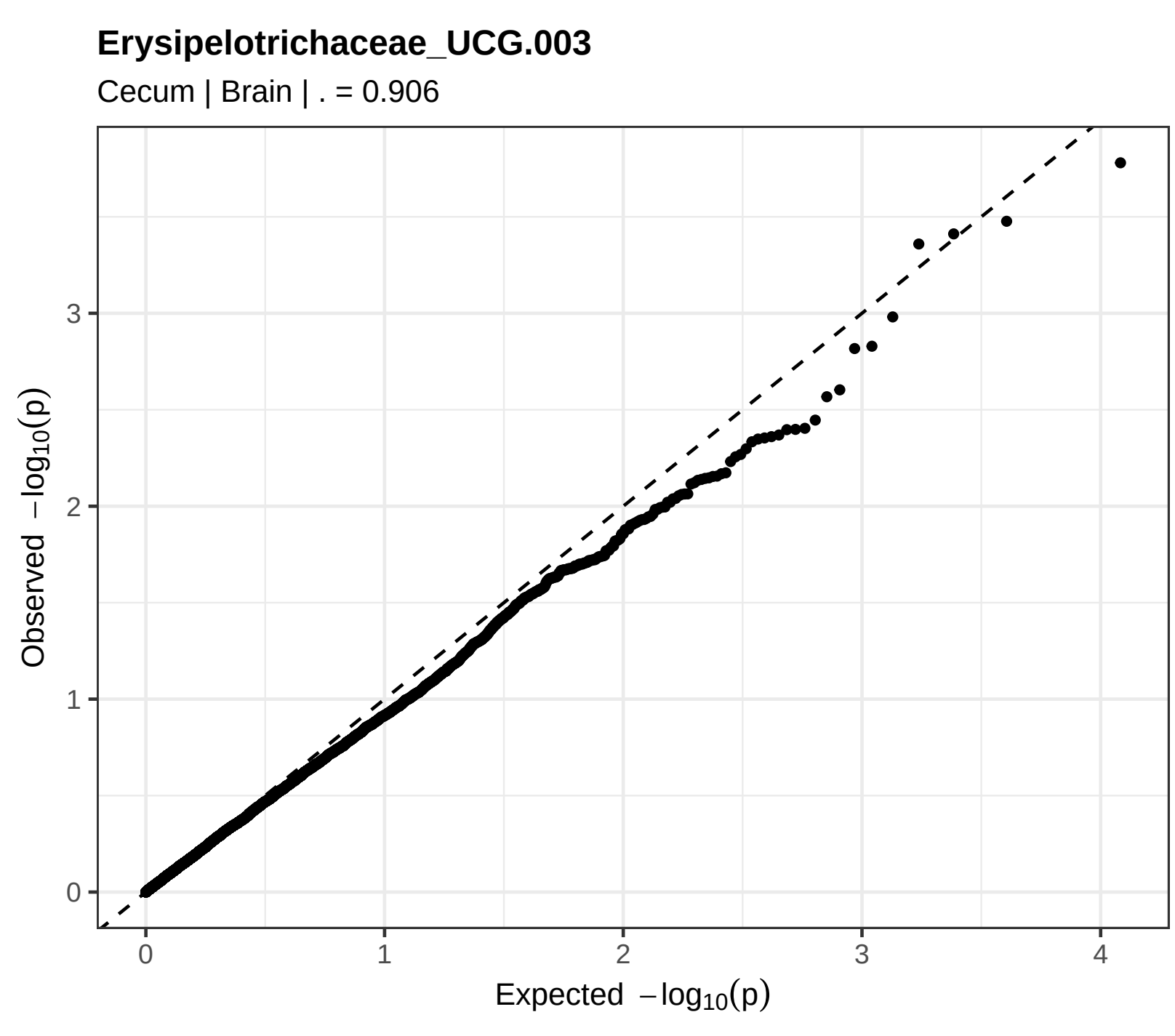
Erysipelotrichaceae_UCG.003
Cecum | Brain | . = 0.906
Observed −log10(p)
Expected −log10(p)

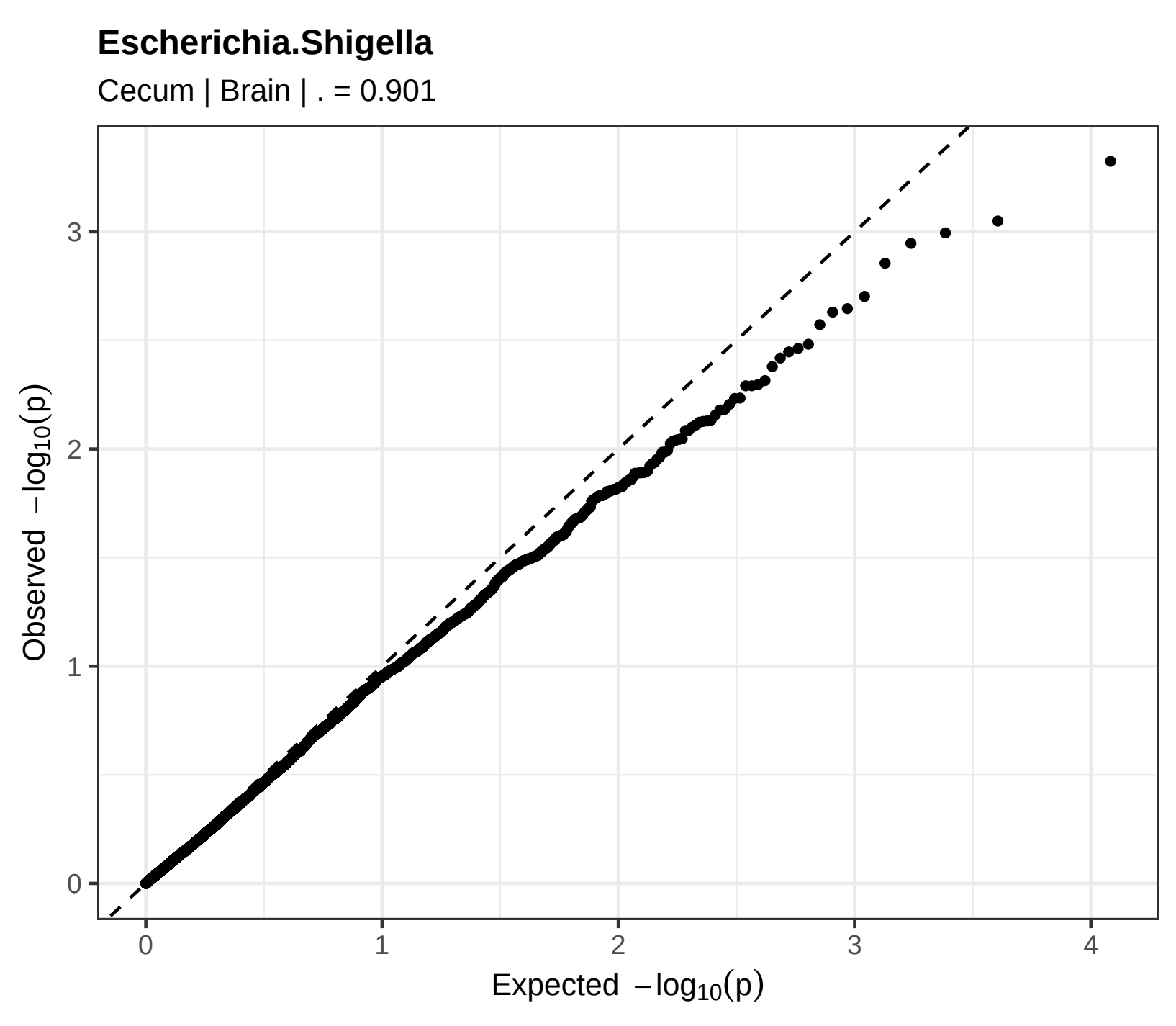
Escherichia.Shigella
Cecum | Brain | . = 0.901
Observed −log10(p)
Expected −log10(p)

Extibacter

Cecum | Brain | . = 0.937

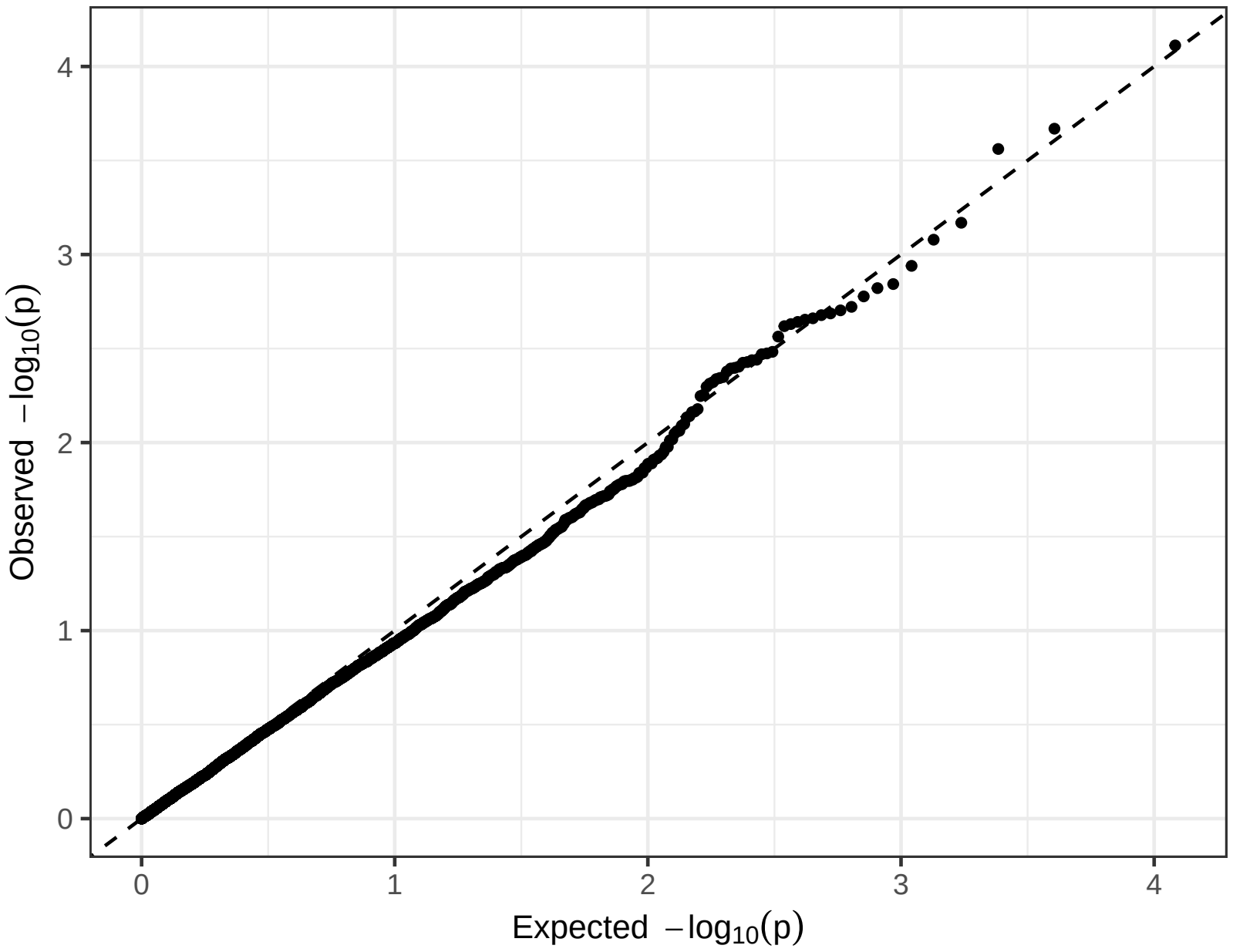

Faecalibacterium

Cecum | Brain | . = 0.927

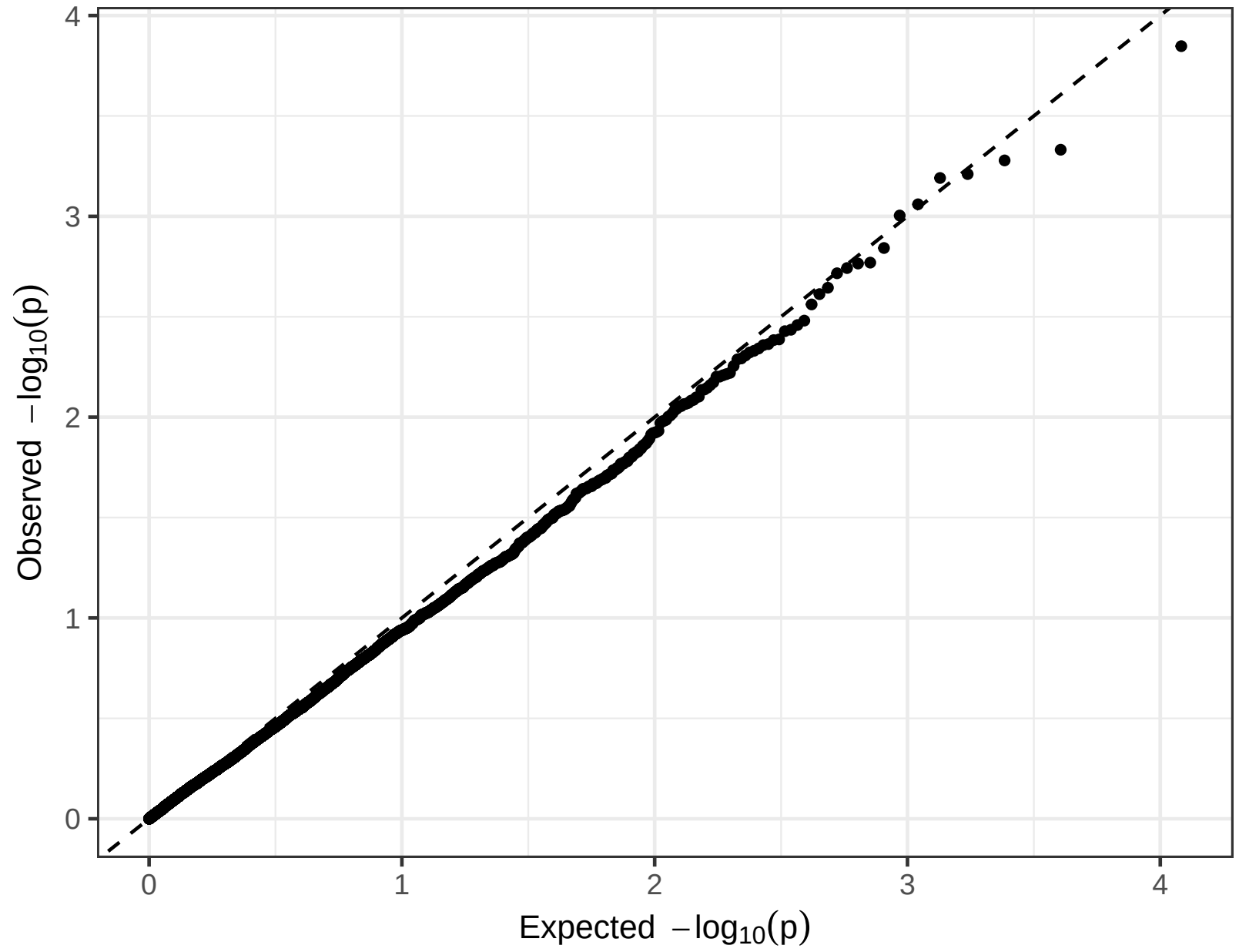

Faecalicatena

Cecum | Brain | . = 1.073

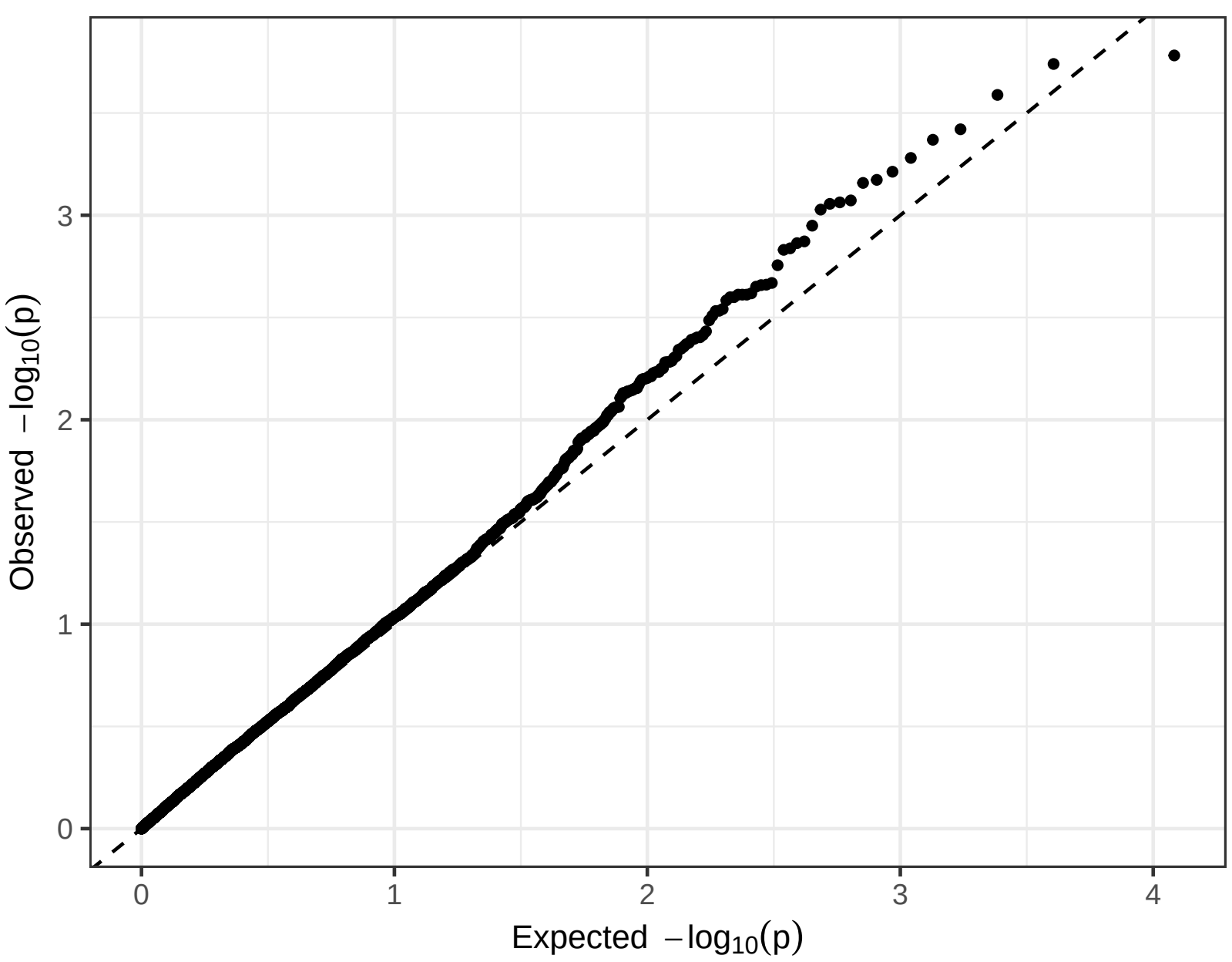

Ruminococcus_gnavus_group

Cecum | Brain | . = 0.93

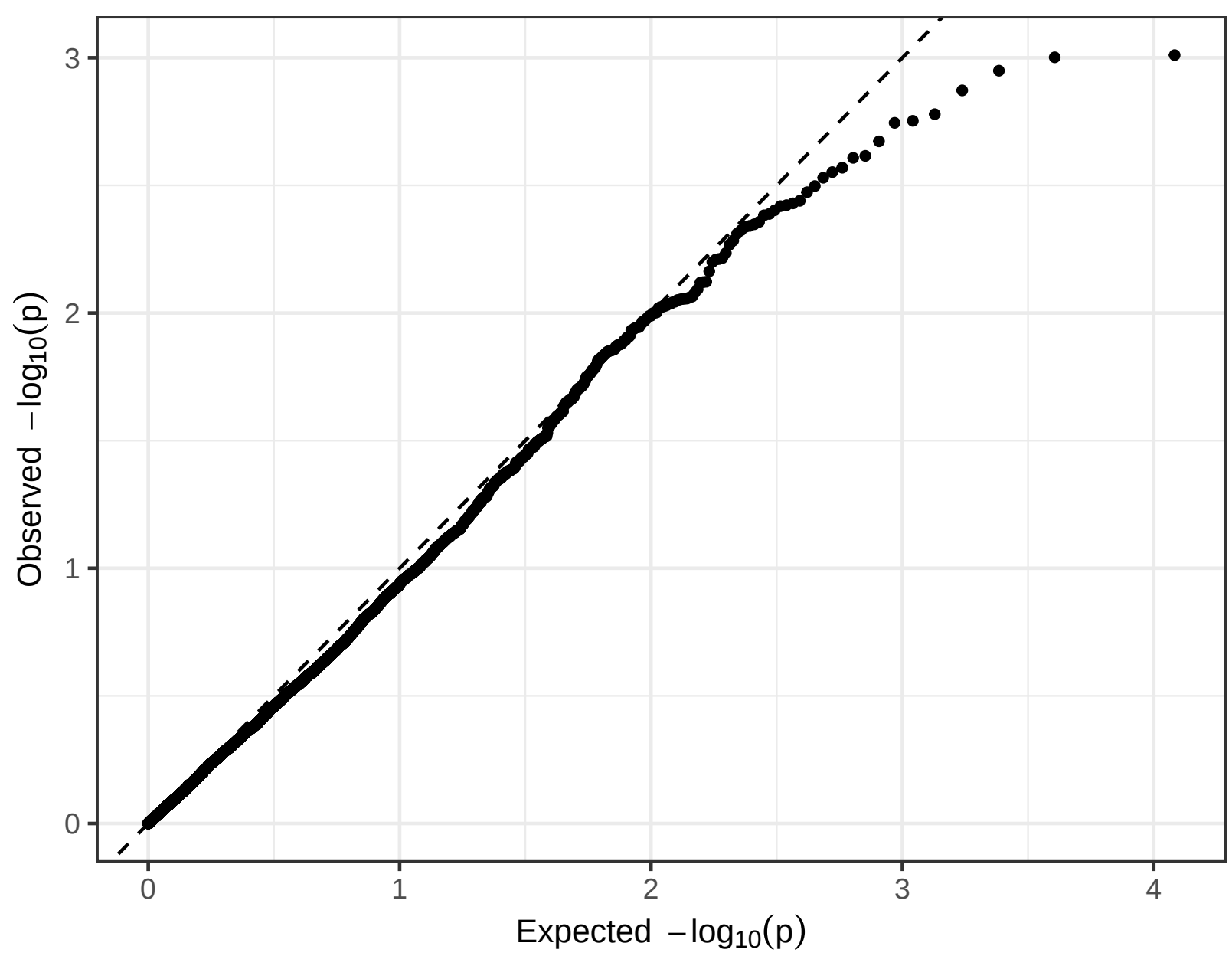

Faecalimonas

Cecum | Brain | . = 0.977

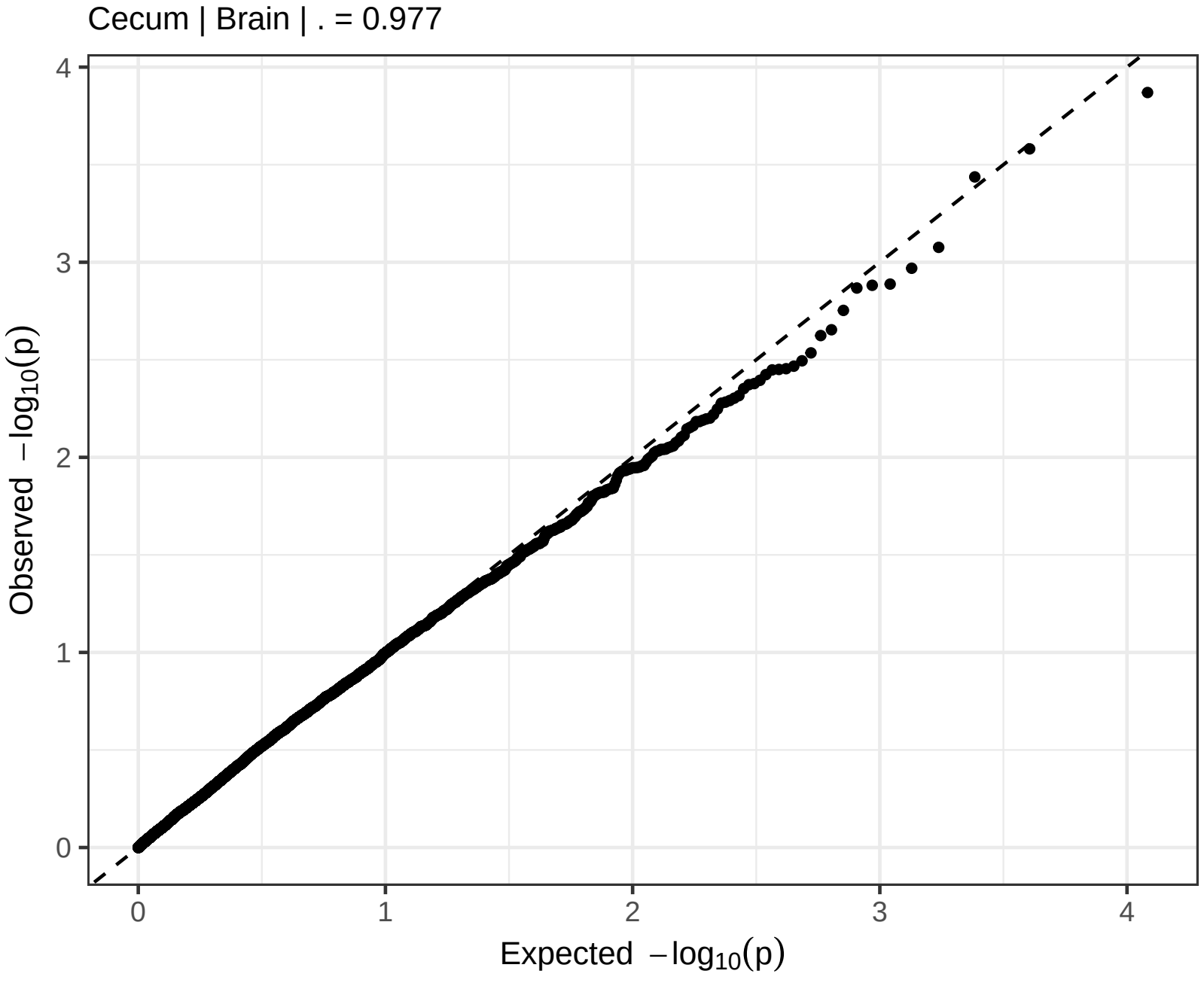

Falcatimonas

Cecum | Brain | . = 0.852

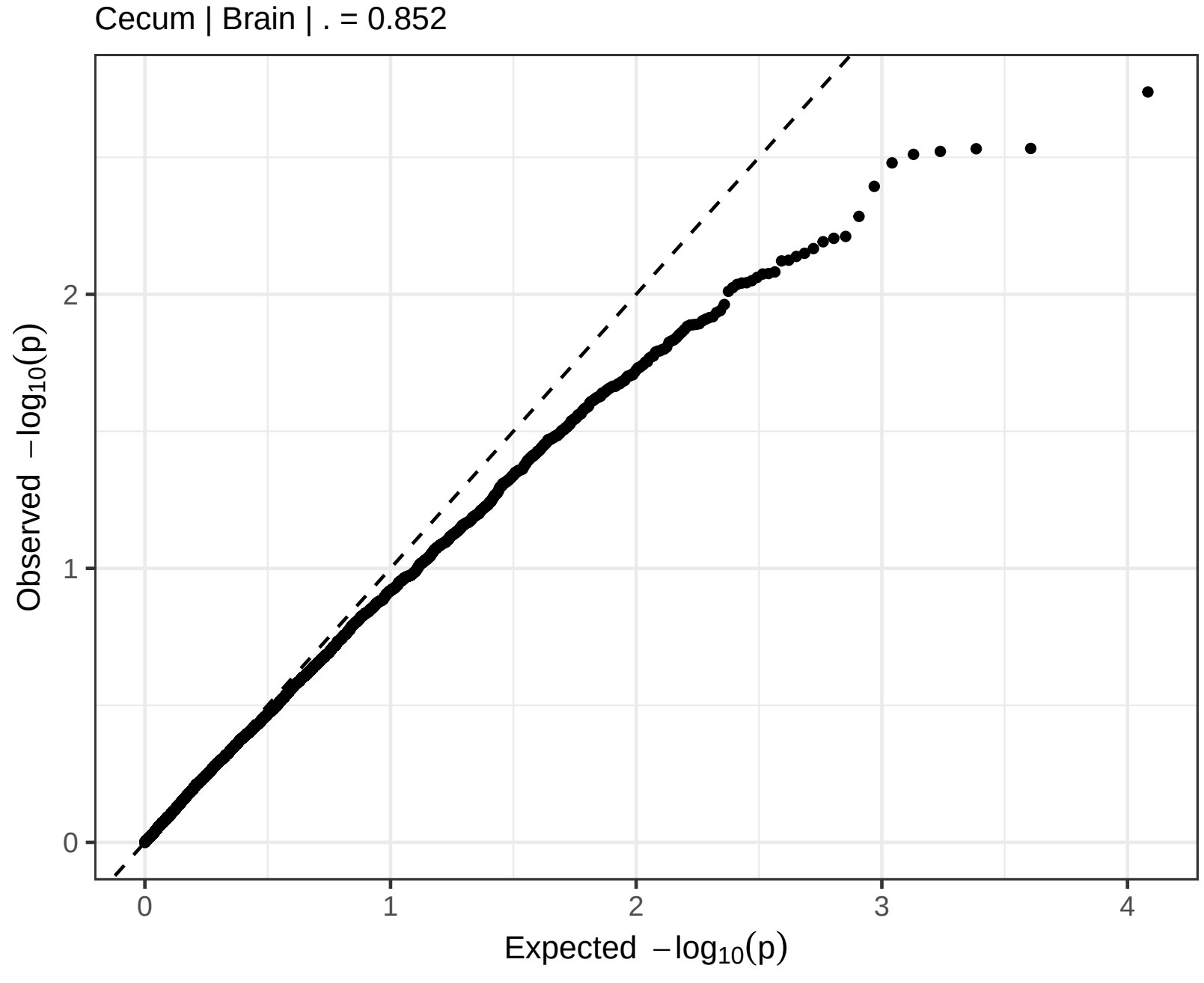

Family_XIII_AD3011_group

Cecum | Brain | . = 0.898

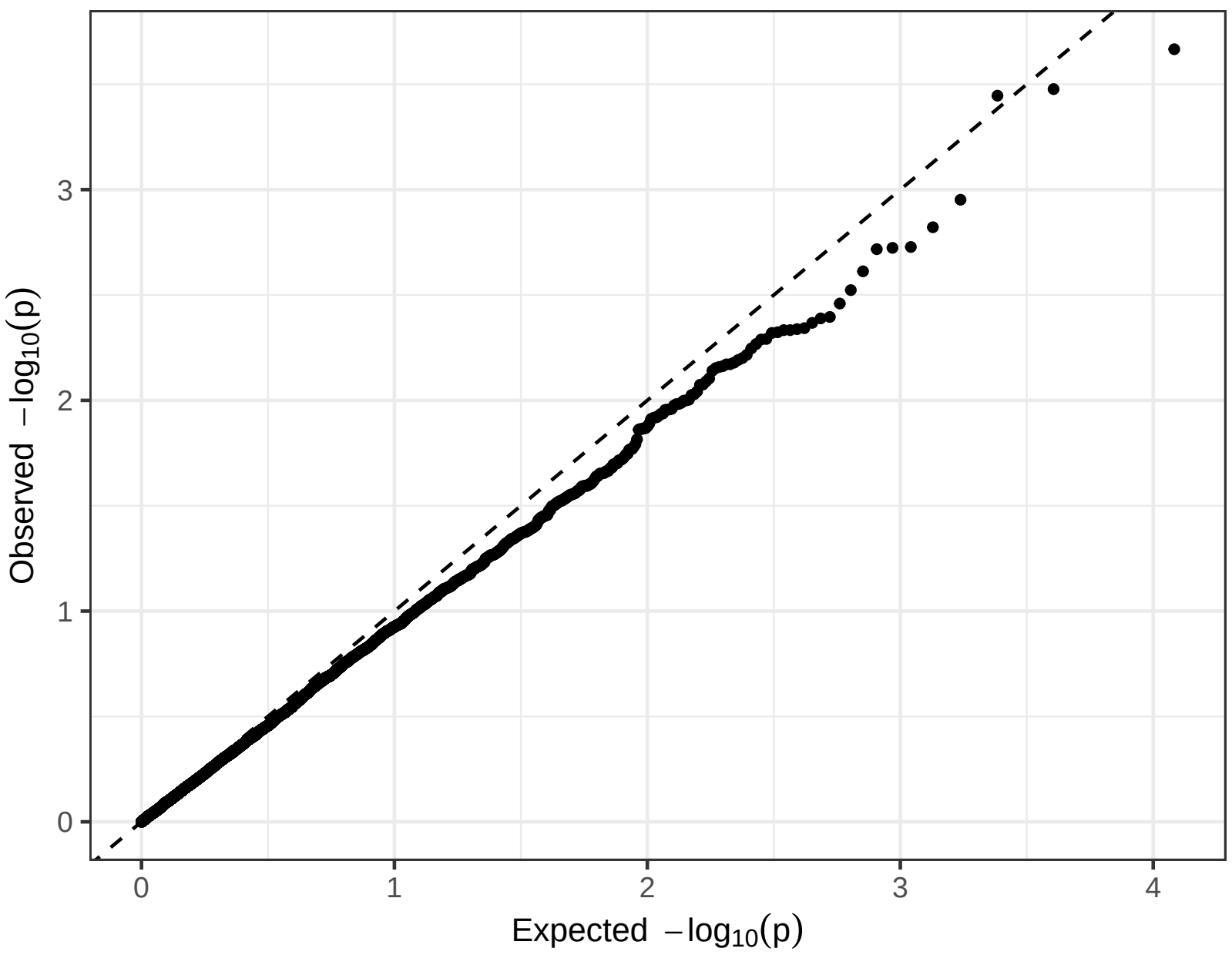

Family_XIII_UCG.001

Cecum | Brain | . = 1.006

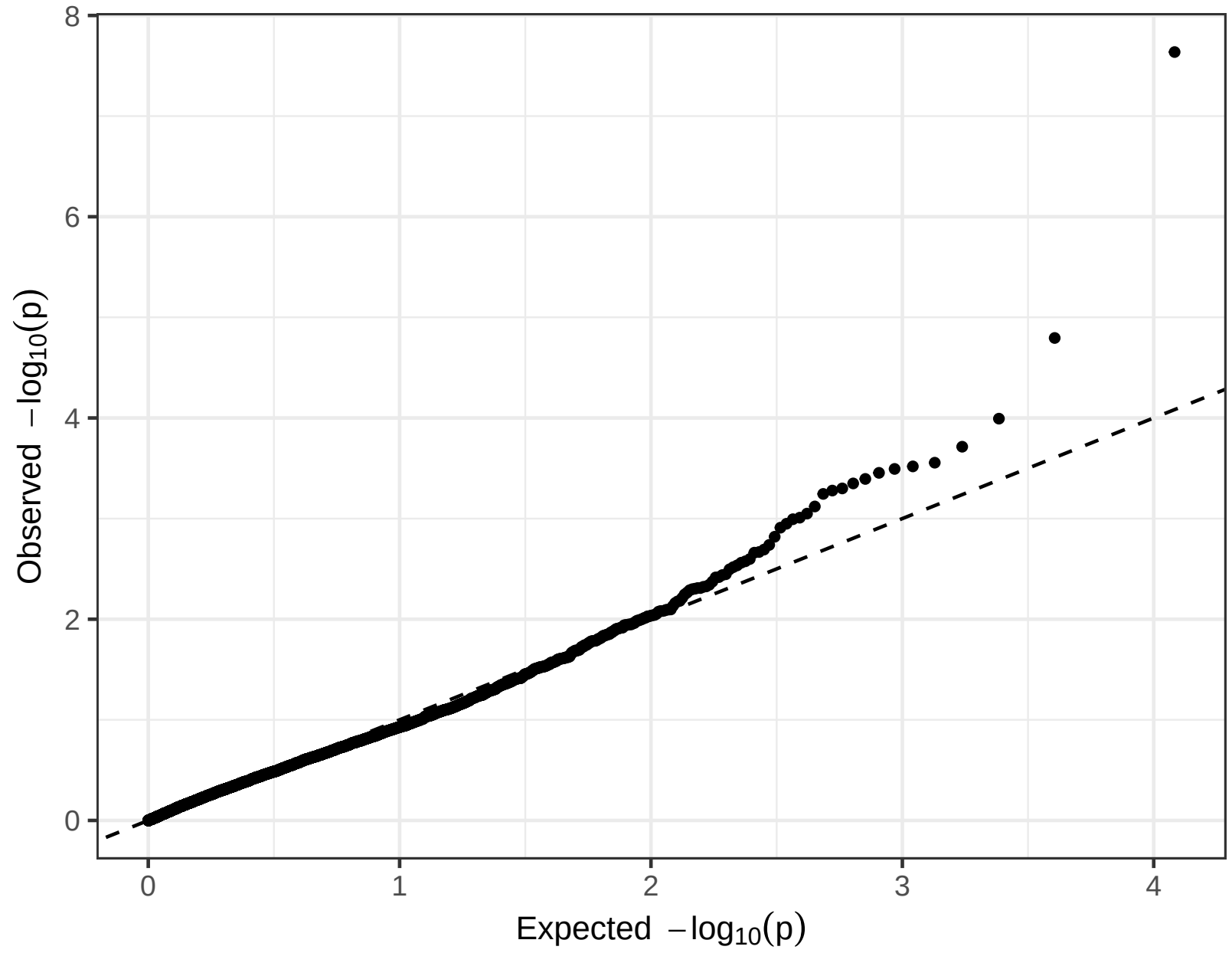

FD2005

Cecum | Brain | . = 1

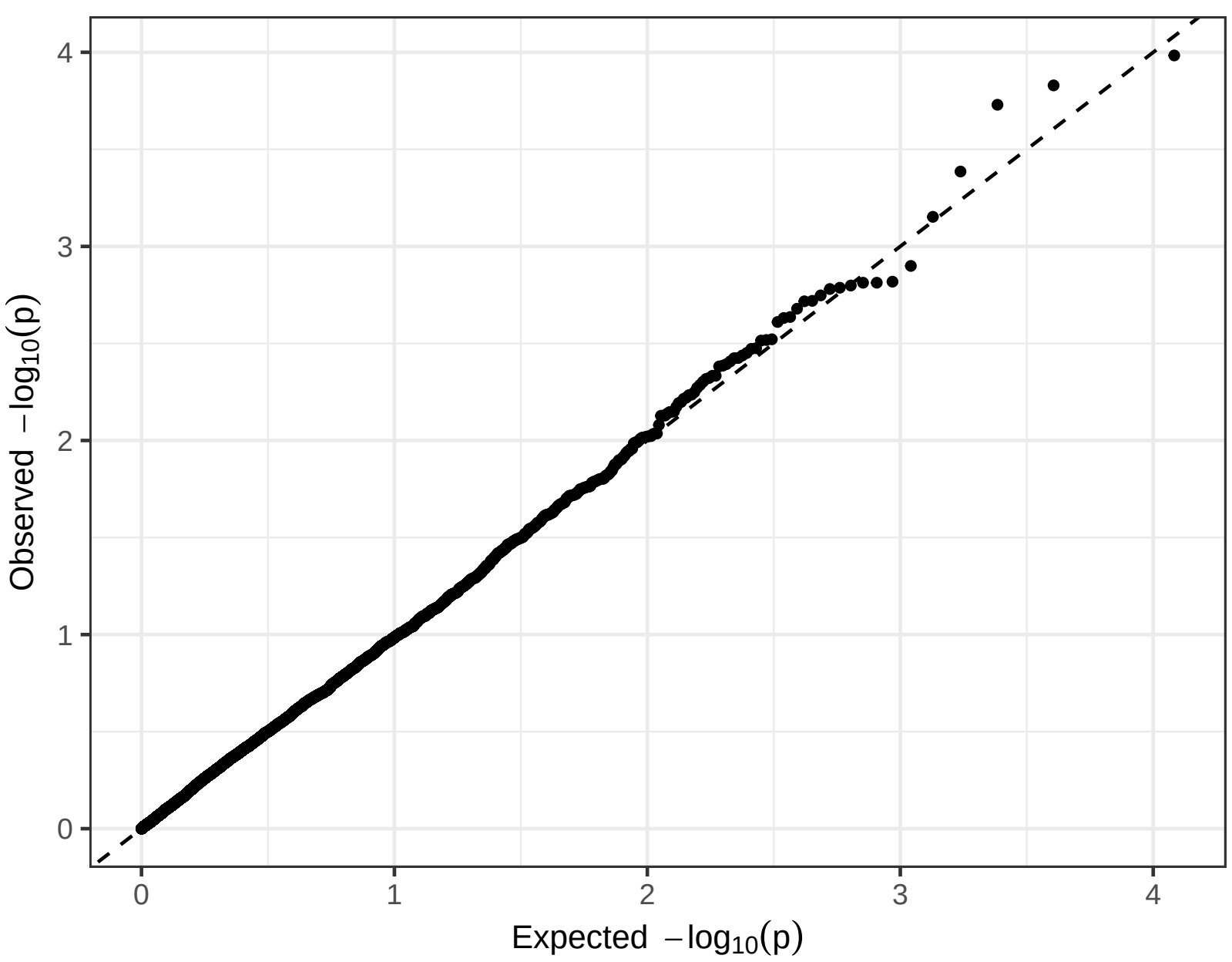

Fibrobacter

Cecum | Brain | . = 0.844

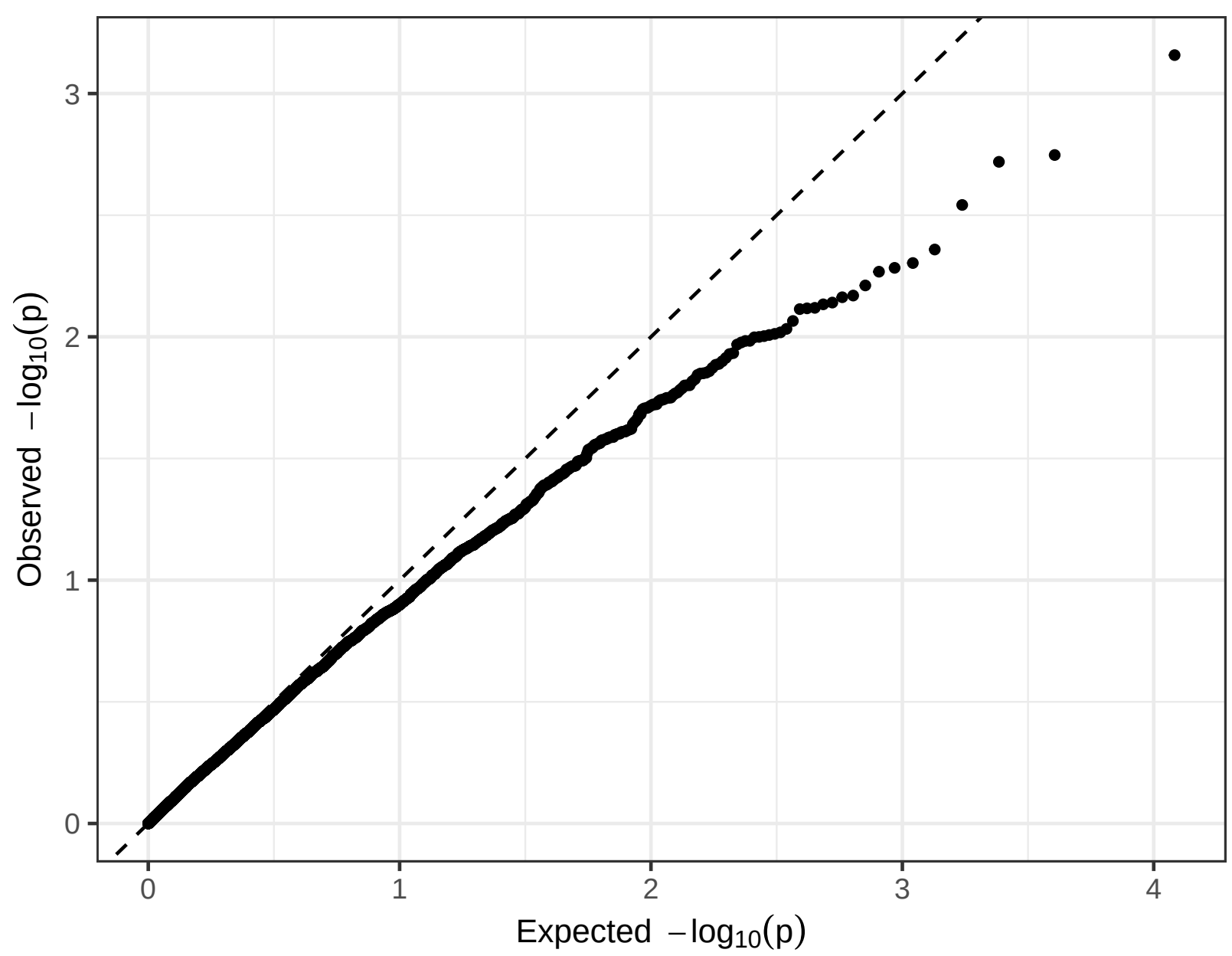

Fournierella

Cecum | Brain | . = 0.906

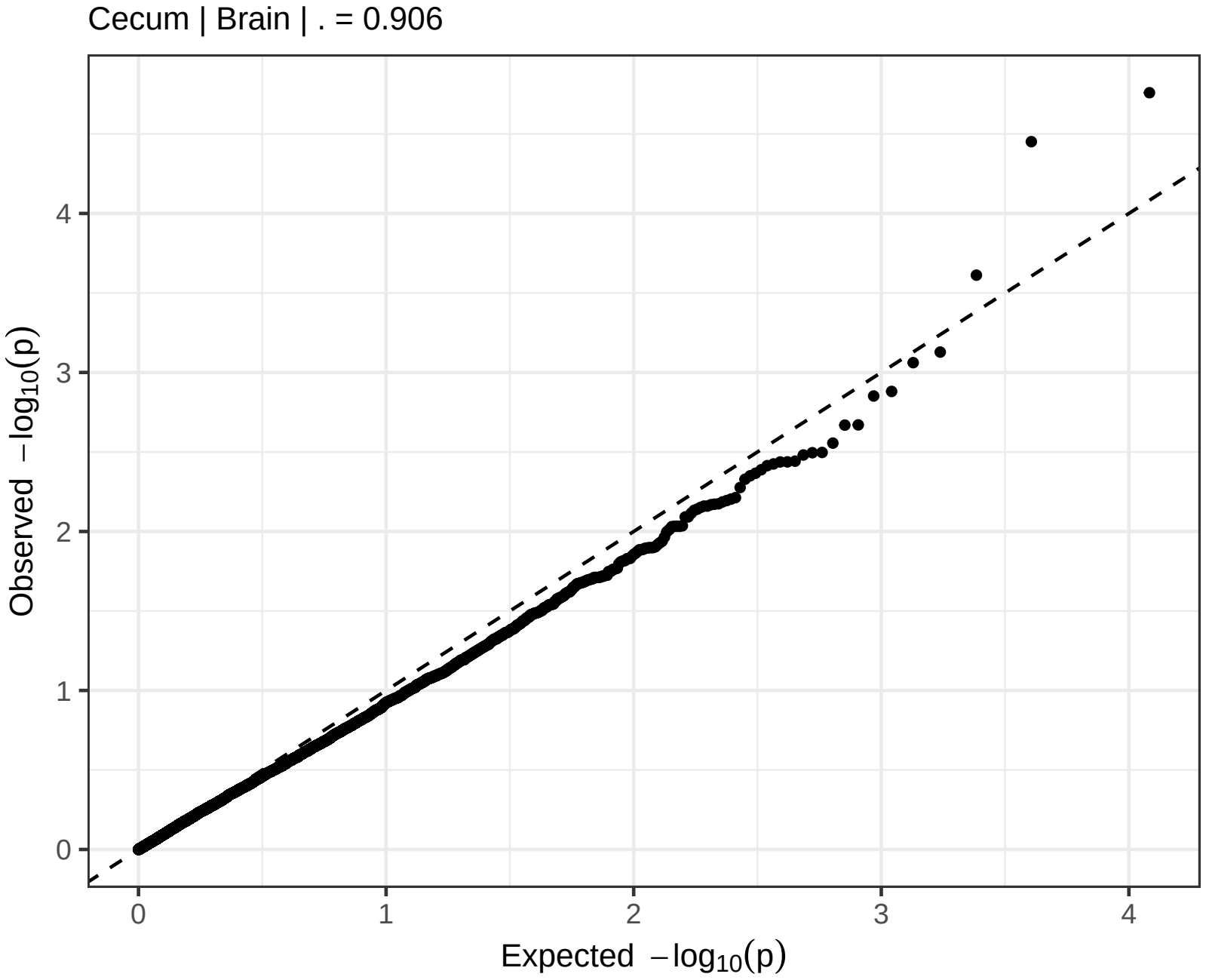

Frisingicoccus

Cecum | Brain | . = 0.99

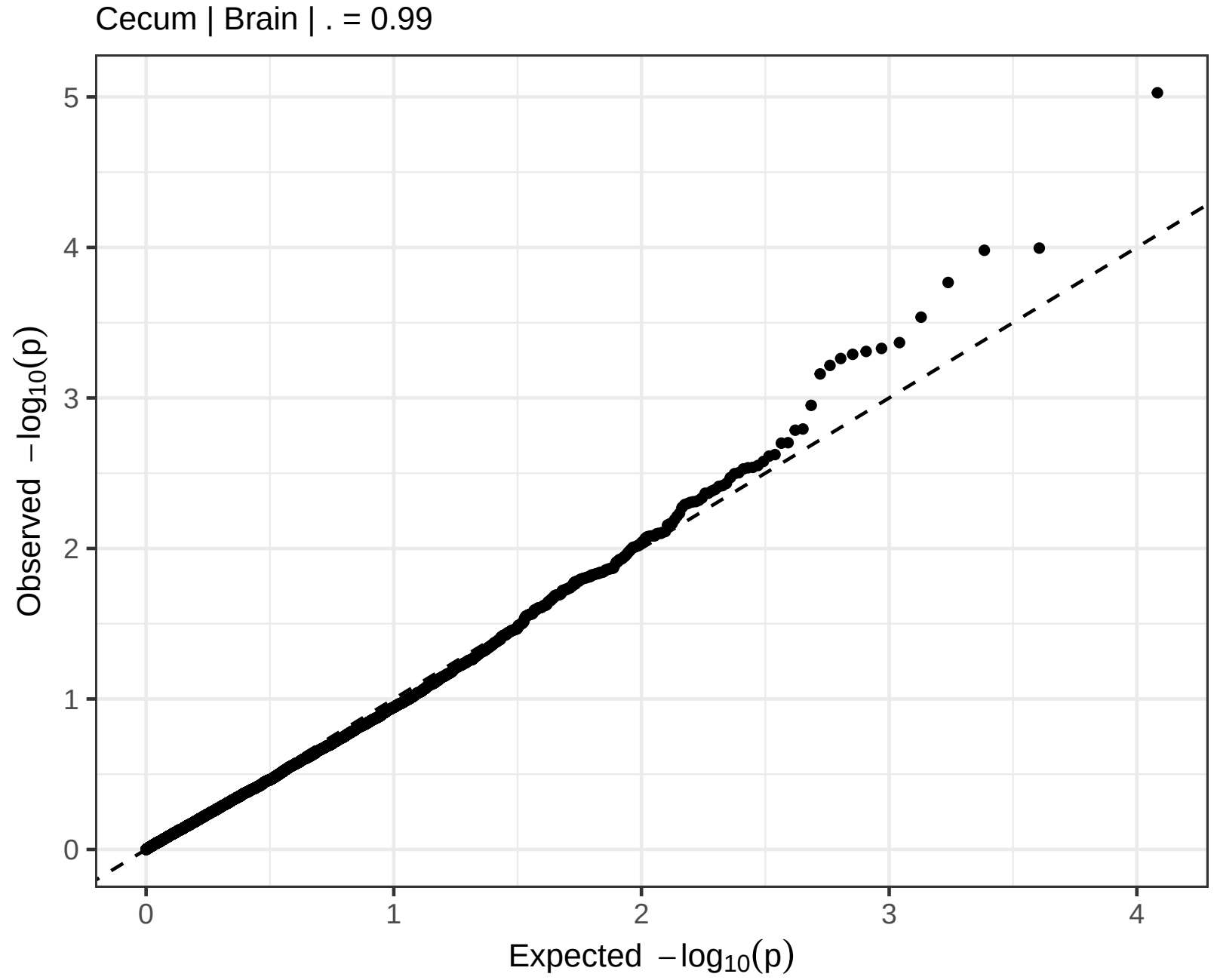

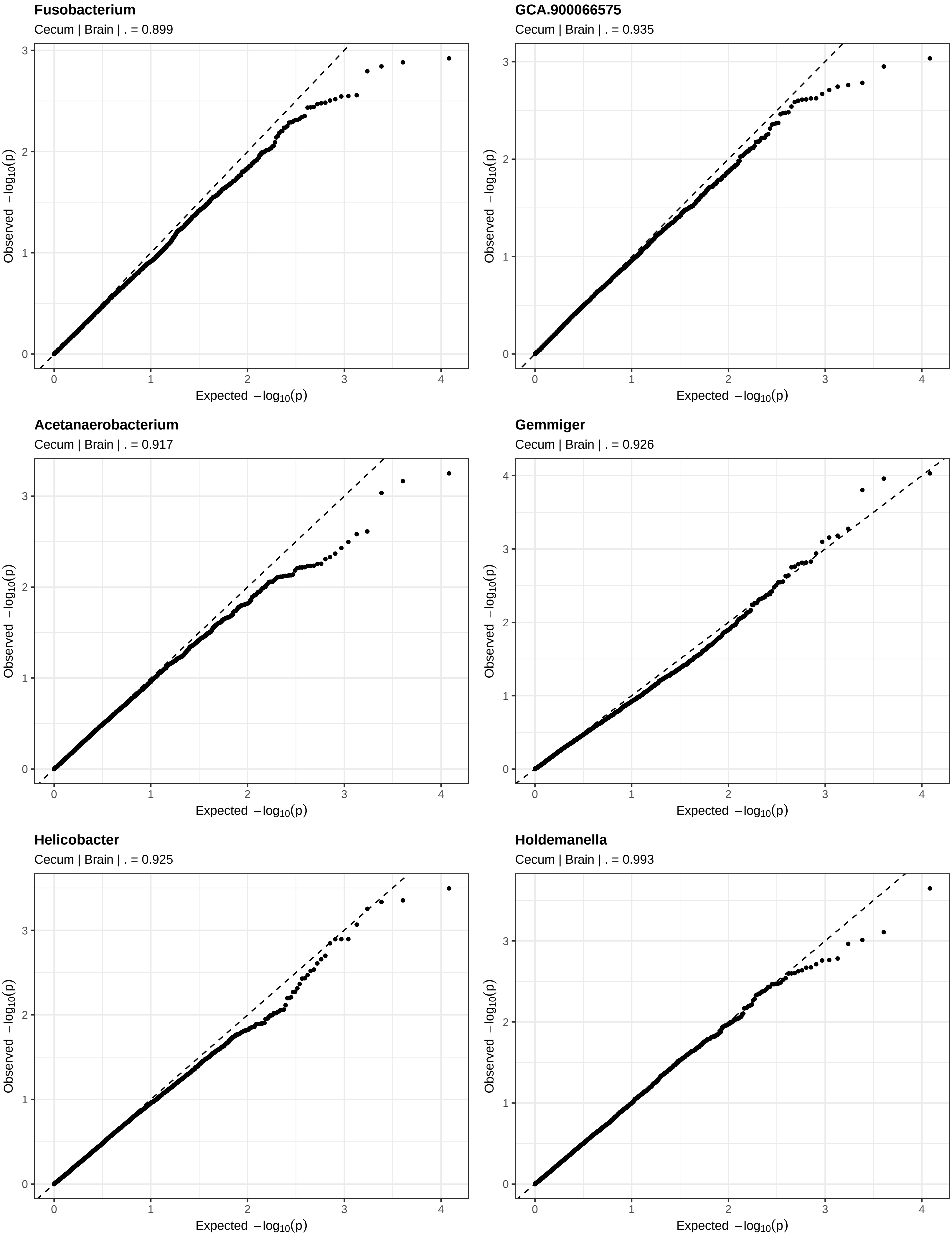

Fusobacterium
Cecum | Brain | . = 0.899
GCA.900066575
Cecum | Brain | . = 0.935
Acetanaerobacterium
Cecum | Brain | . = 0.917
Gemmiger
Cecum | Brain | . = 0.926
Helicobacter
Cecum | Brain | . = 0.925
Holdemanella
Cecum | Brain | . = 0.993
Observed $-\log_{10}(p)$
Expected $-\log_{10}(p)$

horsej.a03

Cecum | Brain | . = 0.865

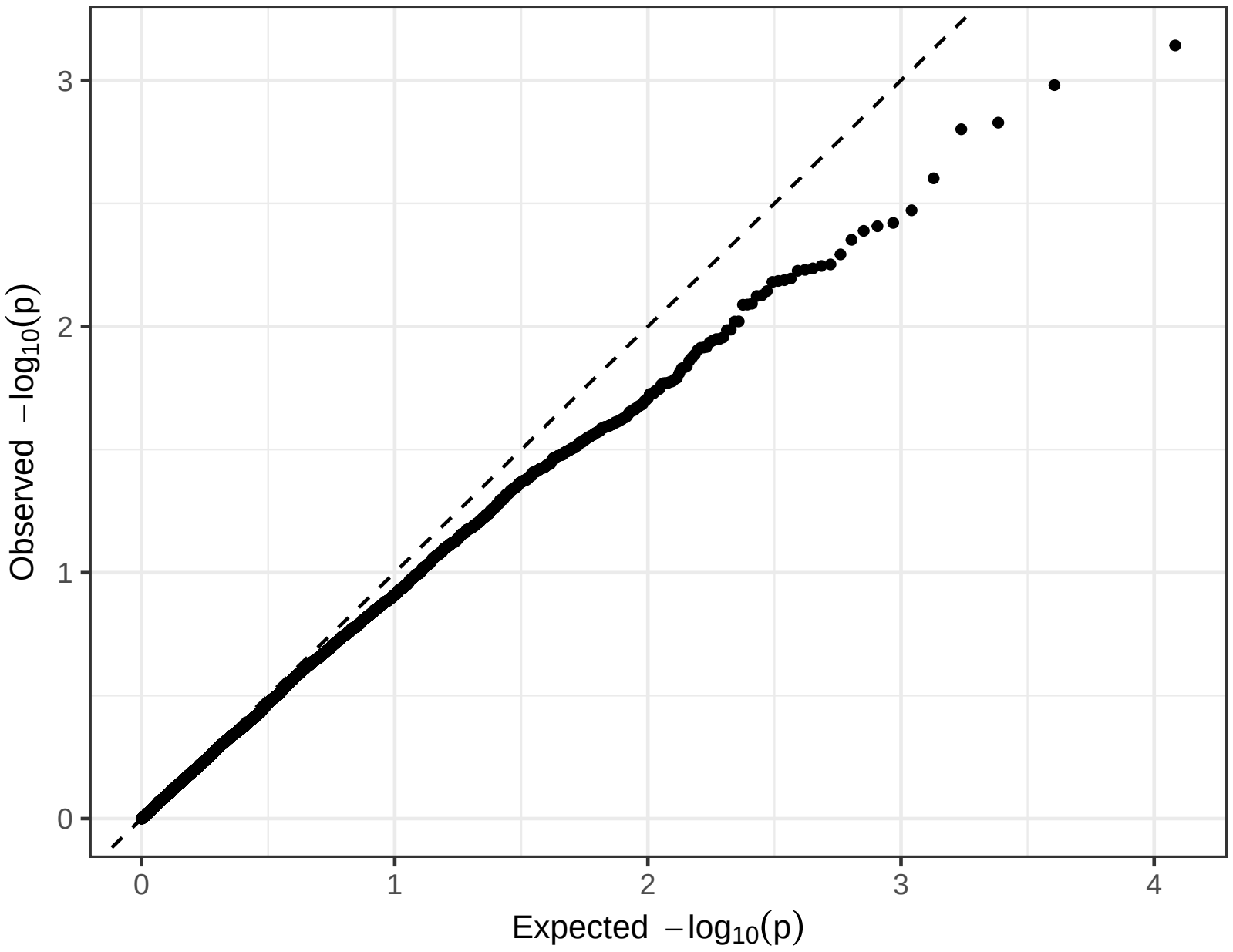

HT002

Cecum | Brain | . = 0.957

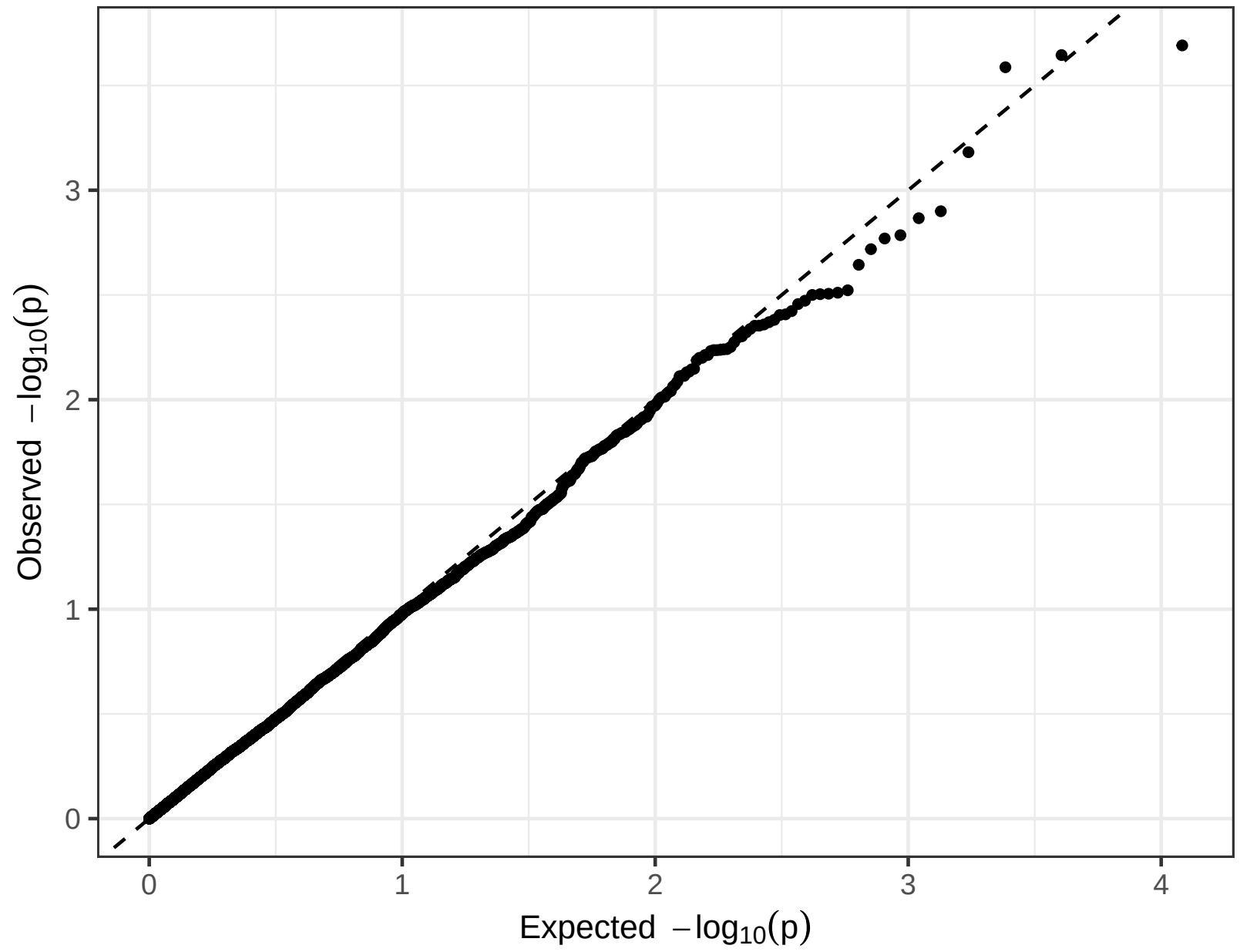

Hydrogenoanaerobacterium

Cecum | Brain | . = 0.965

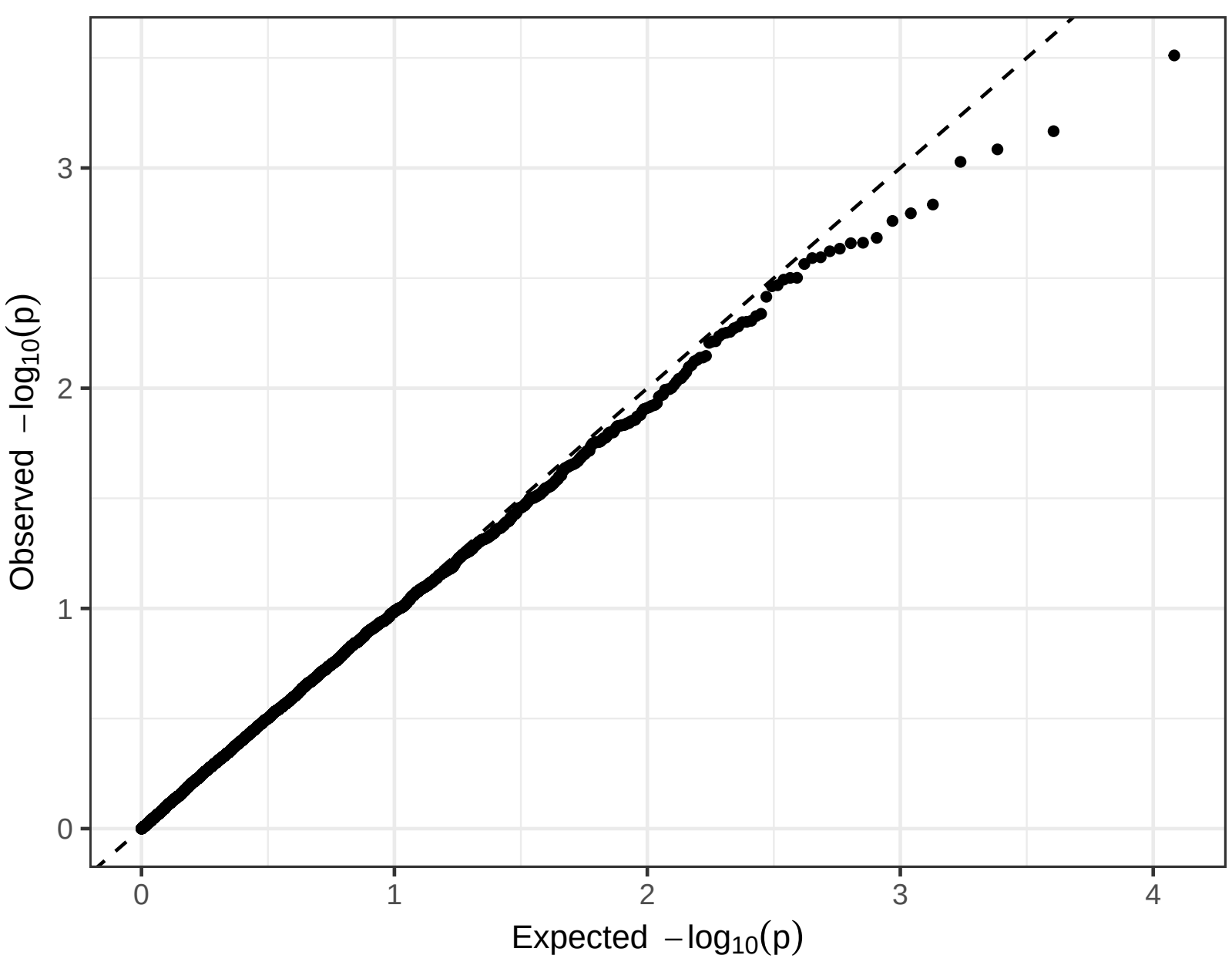

Hydromonas

Cecum | Brain | . = 0.975

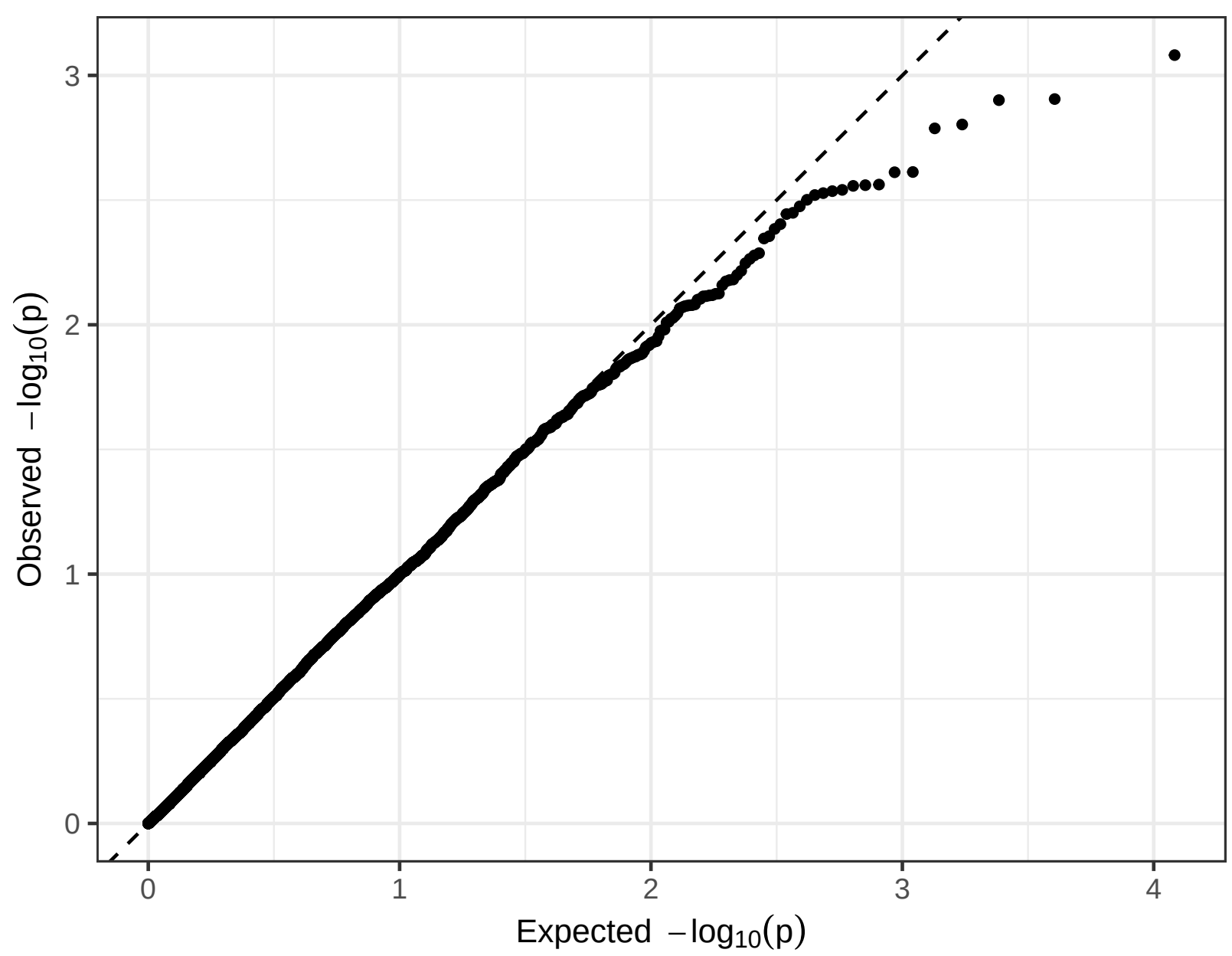

Incertae_Sedis

Cecum | Brain | . = 0.905

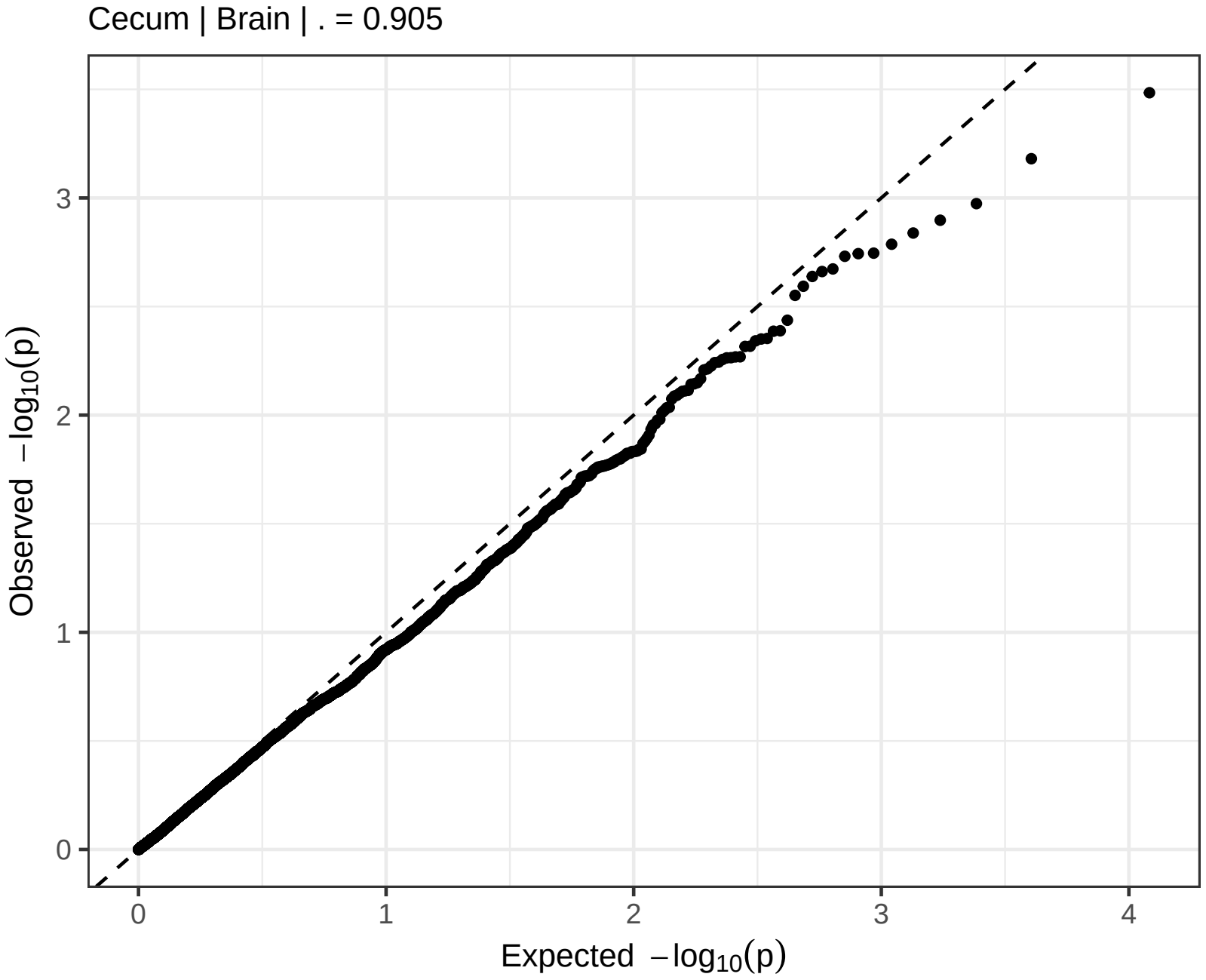

Intestinibacter

Cecum | Brain | . = 0.884

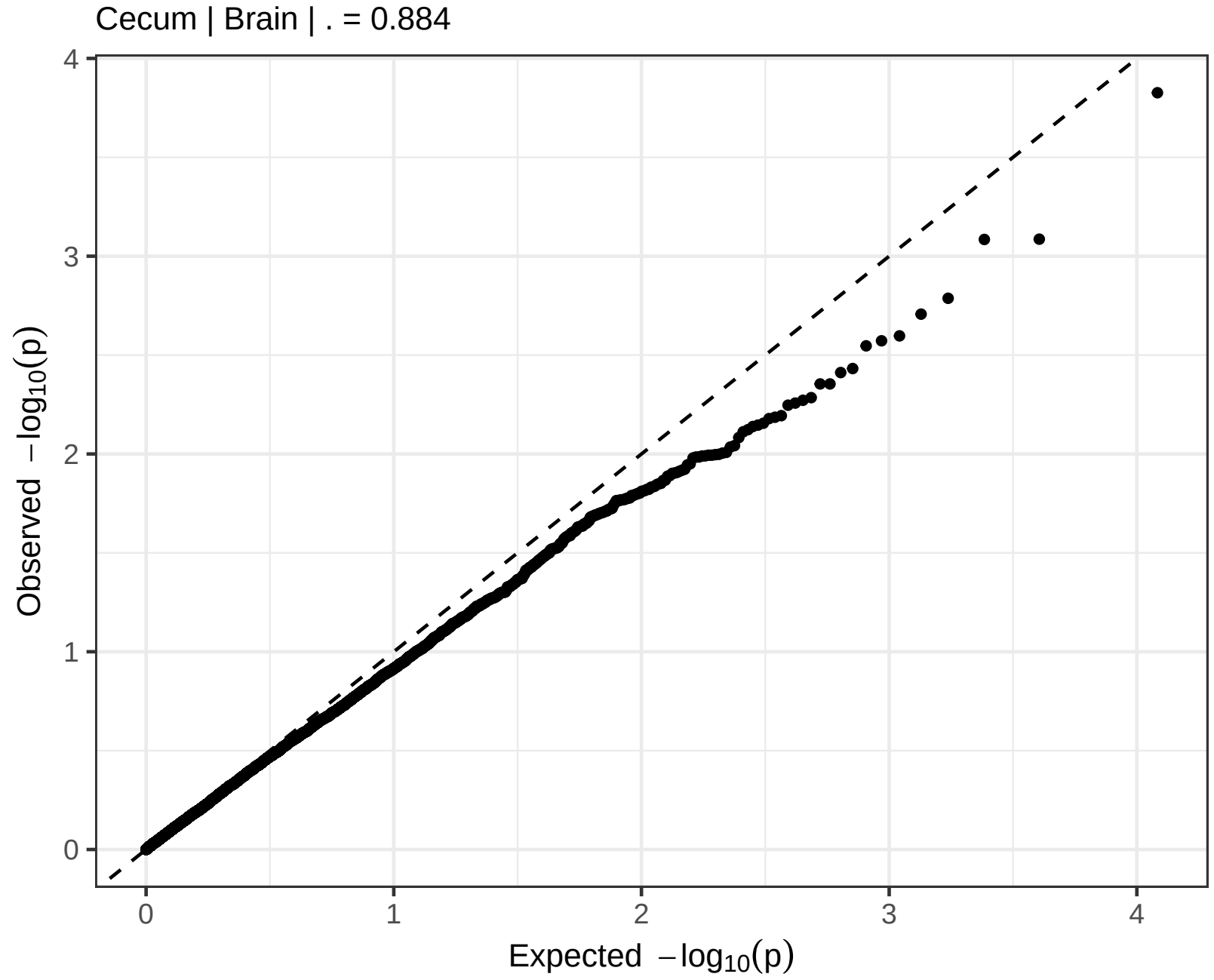

Intestinimonas

Cecum | Brain | . = 0.919

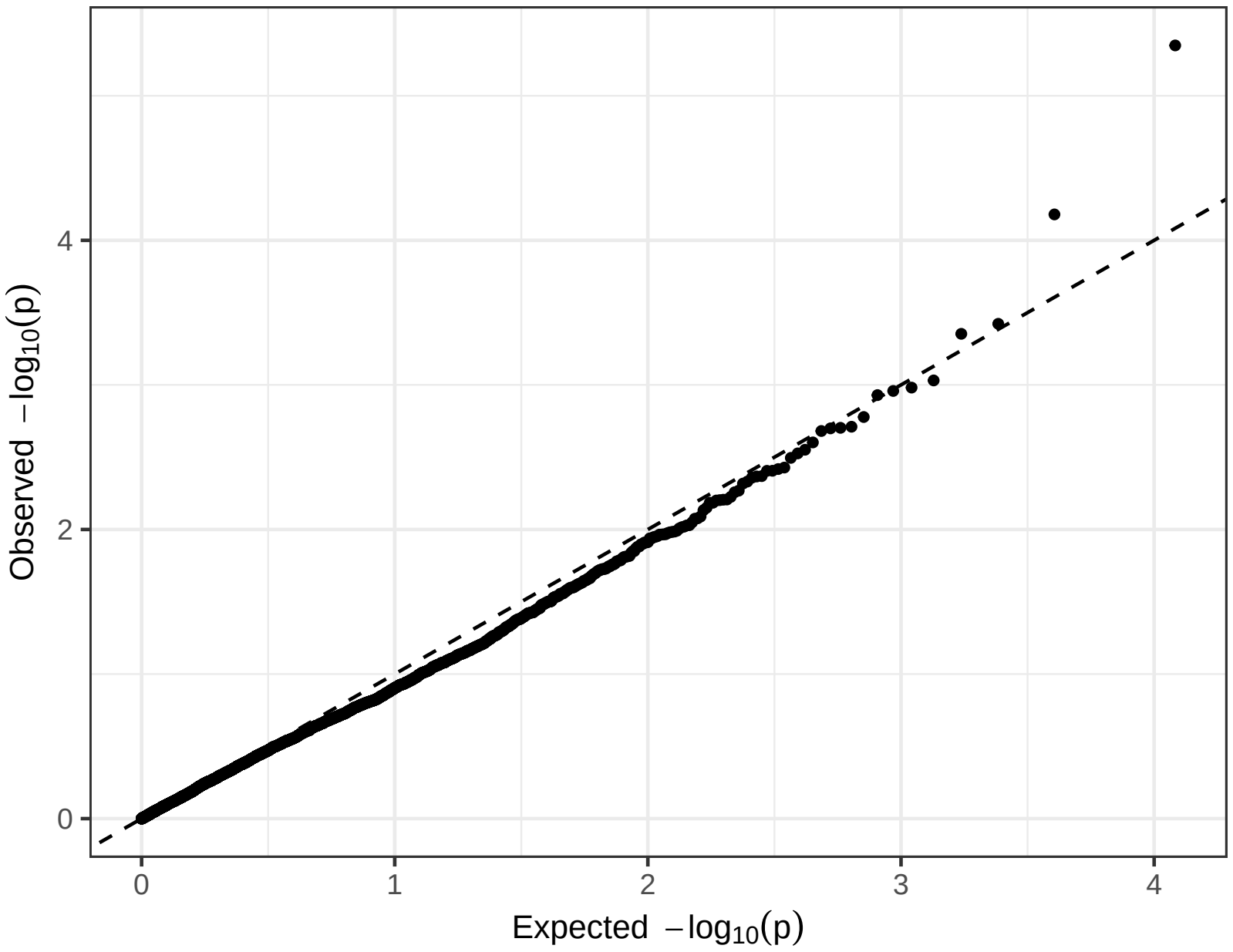

Acetitomaculum

Cecum | Brain | . = 0.928

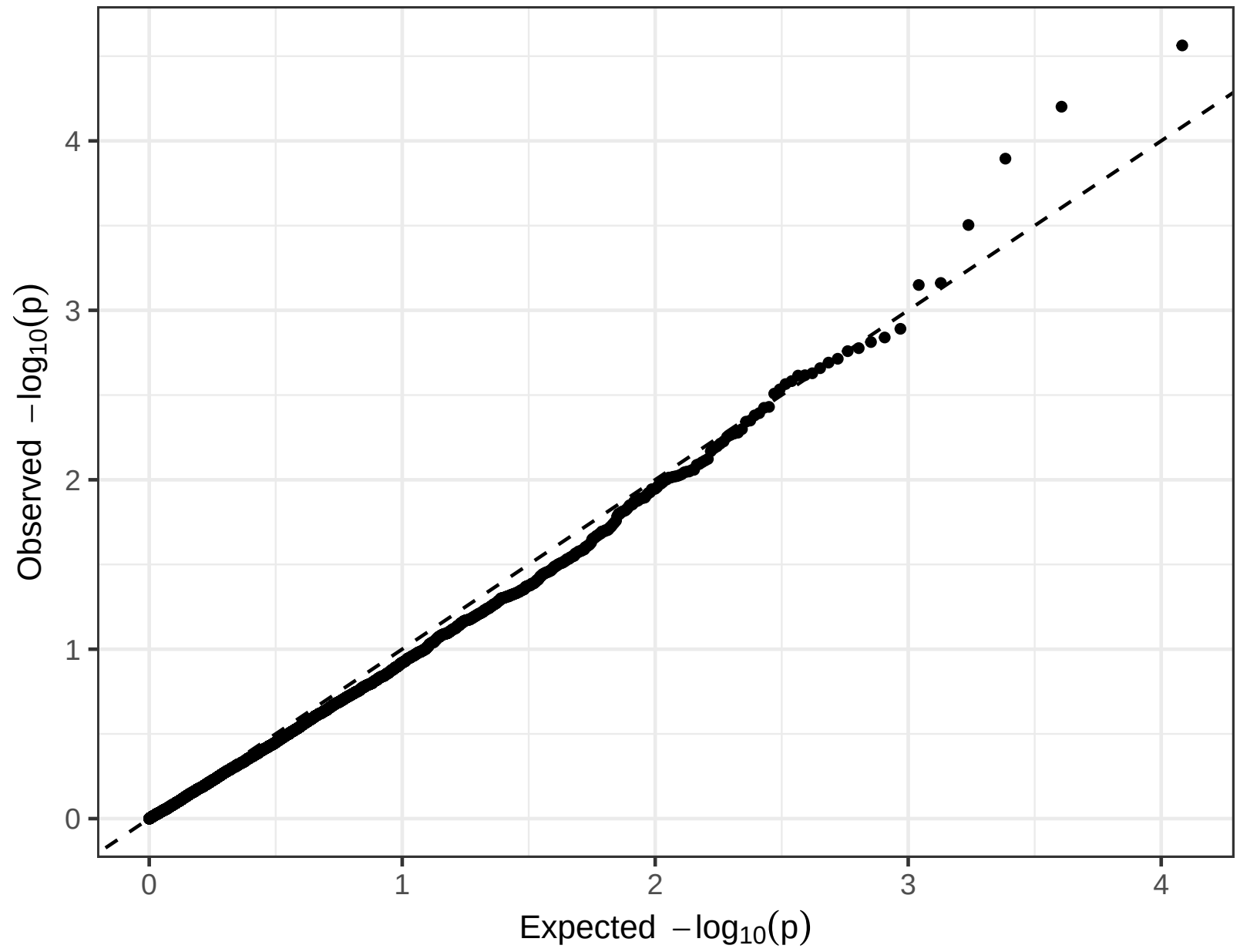

Kineothrix

Cecum | Brain | . = 0.89

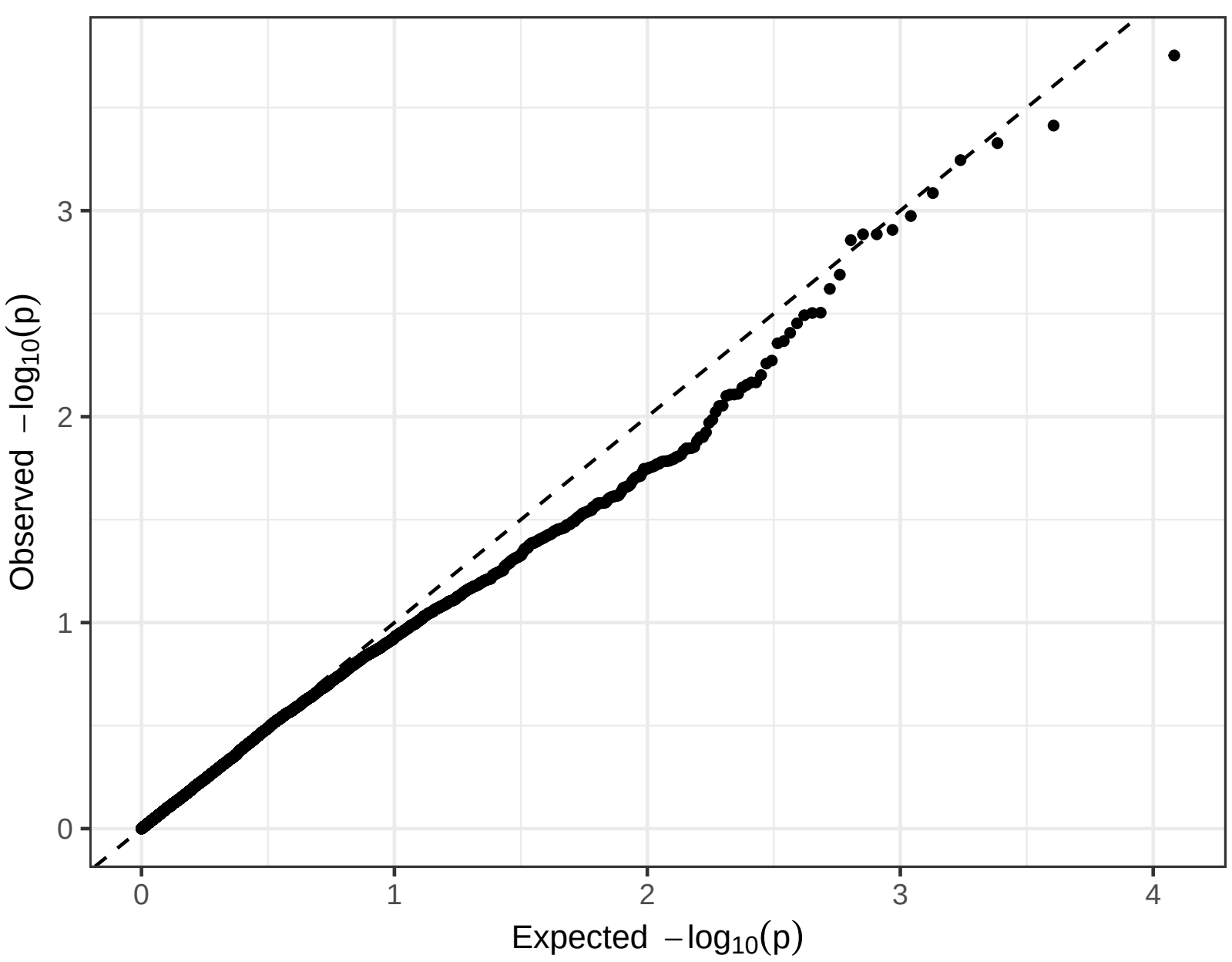

Lachnoclostridium

Cecum | Brain | . = 0.95

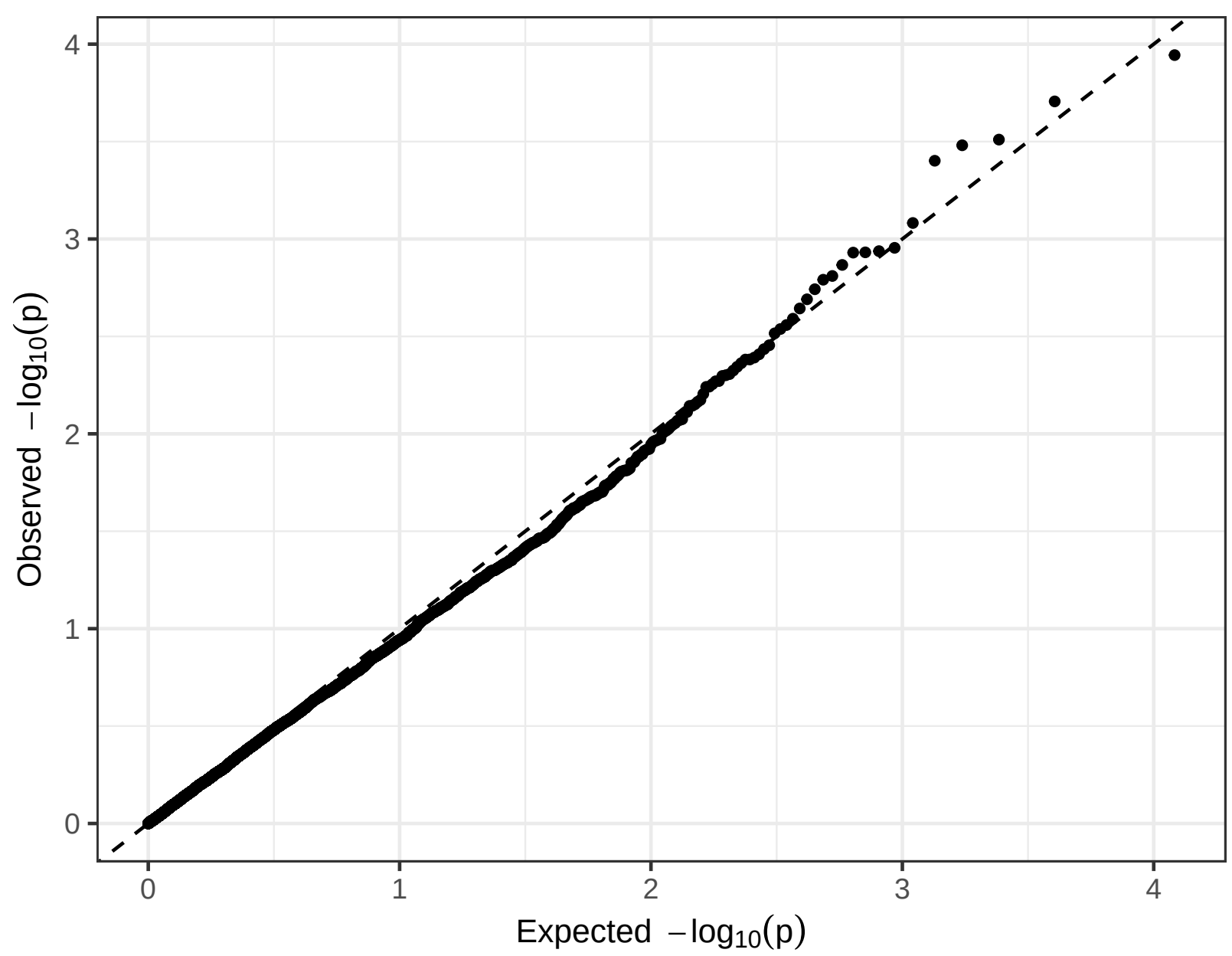

Lachnospira

Cecum | Brain | . = 0.905

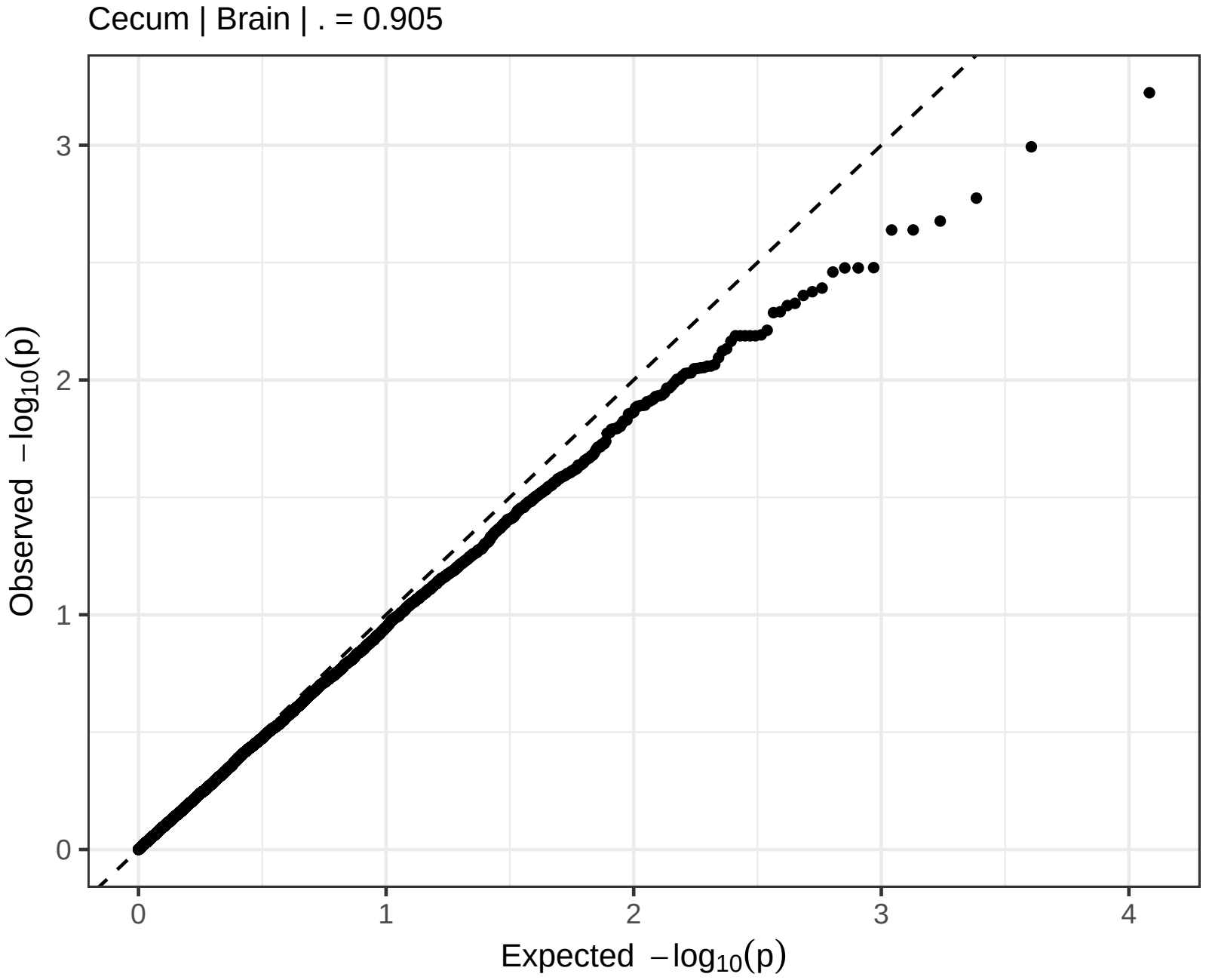

Lachnospiraceae_AC2044_group

Cecum | Brain | . = 0.952

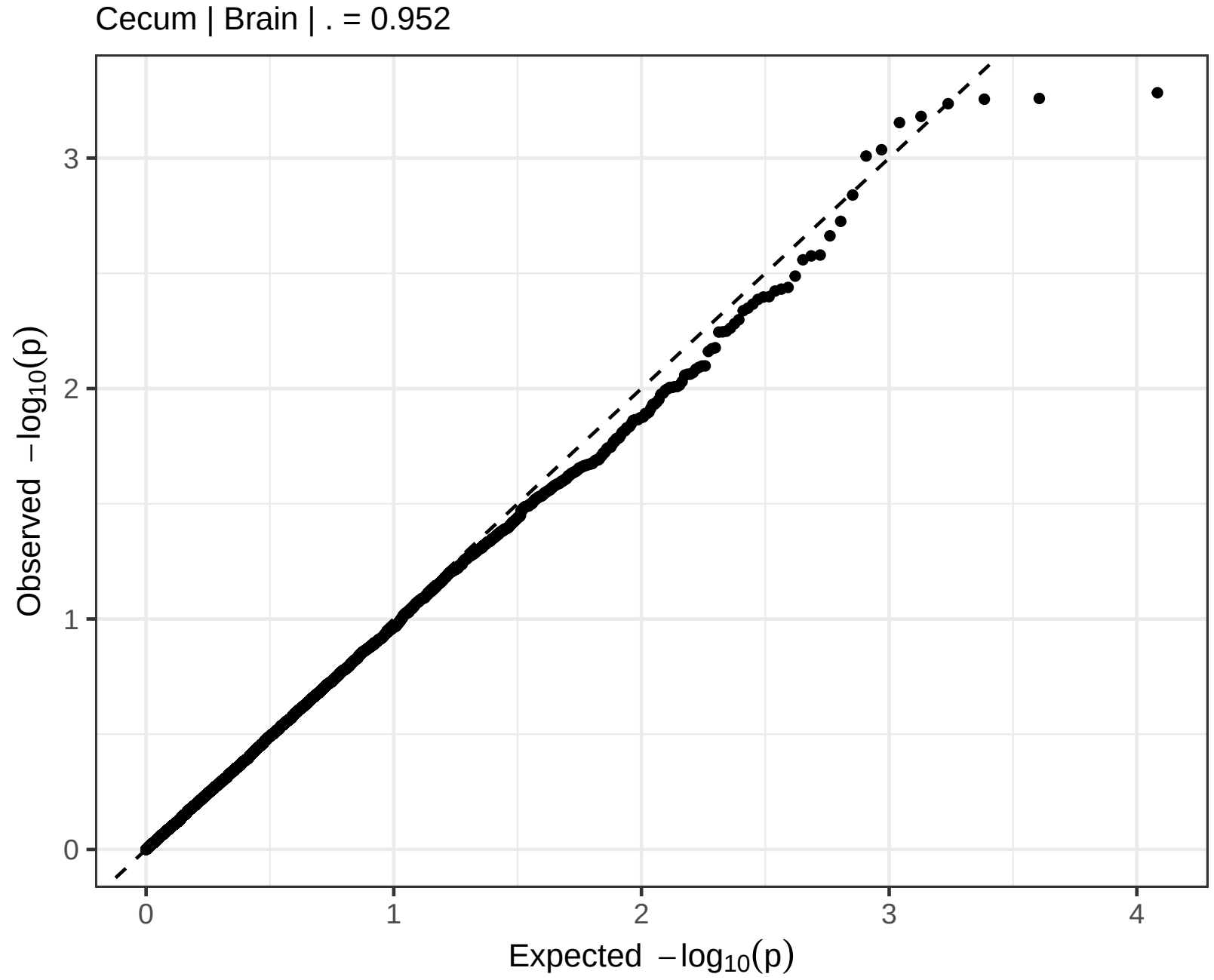

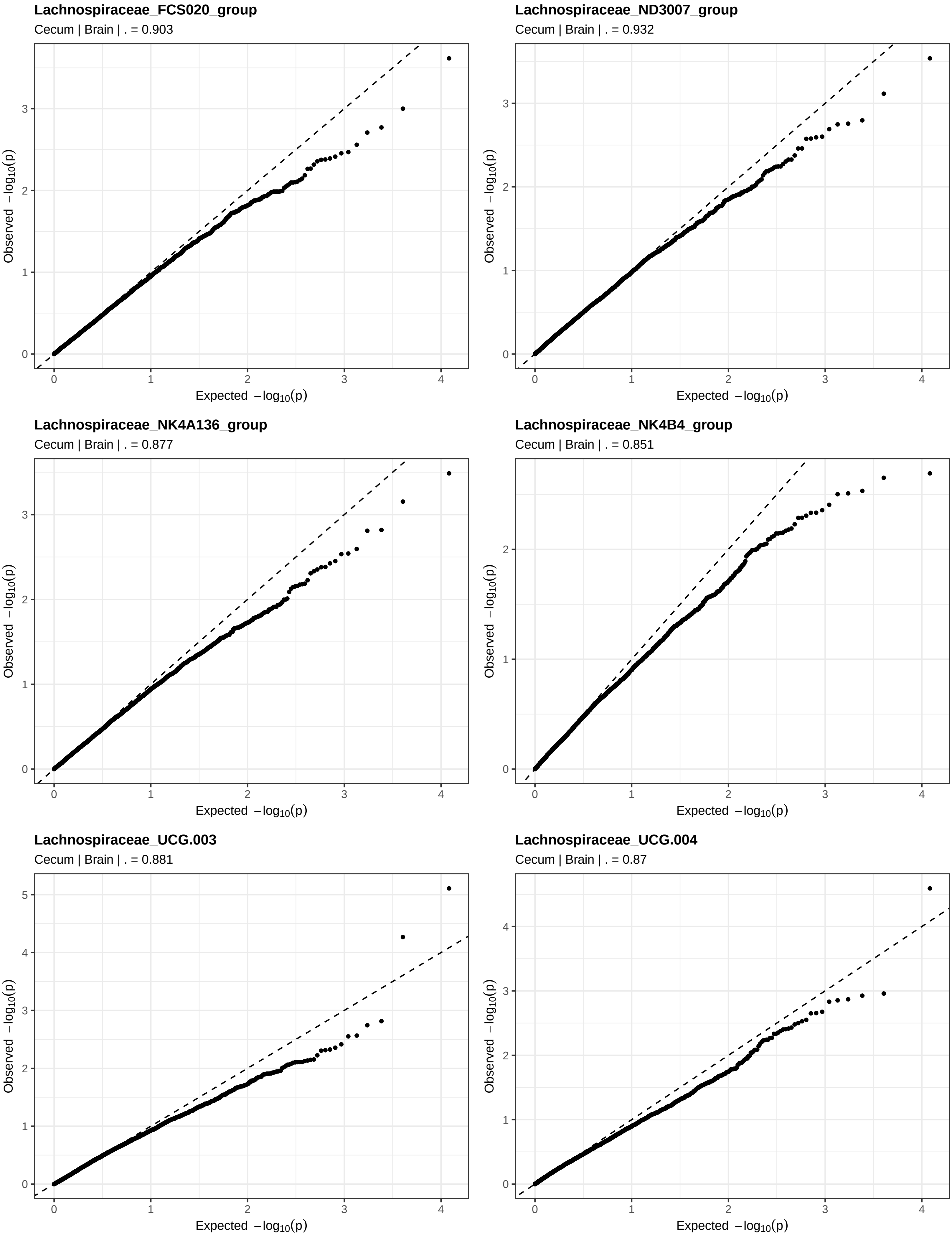
Lachnospiraceae_FCS020_group
Cecum | Brain | . = 0.903
Lachnospiraceae_ND3007_group
Cecum | Brain | . = 0.932
Lachnospiraceae_NK4A136_group
Cecum | Brain | . = 0.877
Lachnospiraceae_NK4B4_group
Cecum | Brain | . = 0.851
Lachnospiraceae_UCG.003
Cecum | Brain | . = 0.881
Lachnospiraceae_UCG.004
Cecum | Brain | . = 0.87
Observed $-\log_{10}(p)$
Expected $-\log_{10}(p)$

Acutalibacter

Cecum | Brain | . = 0.911

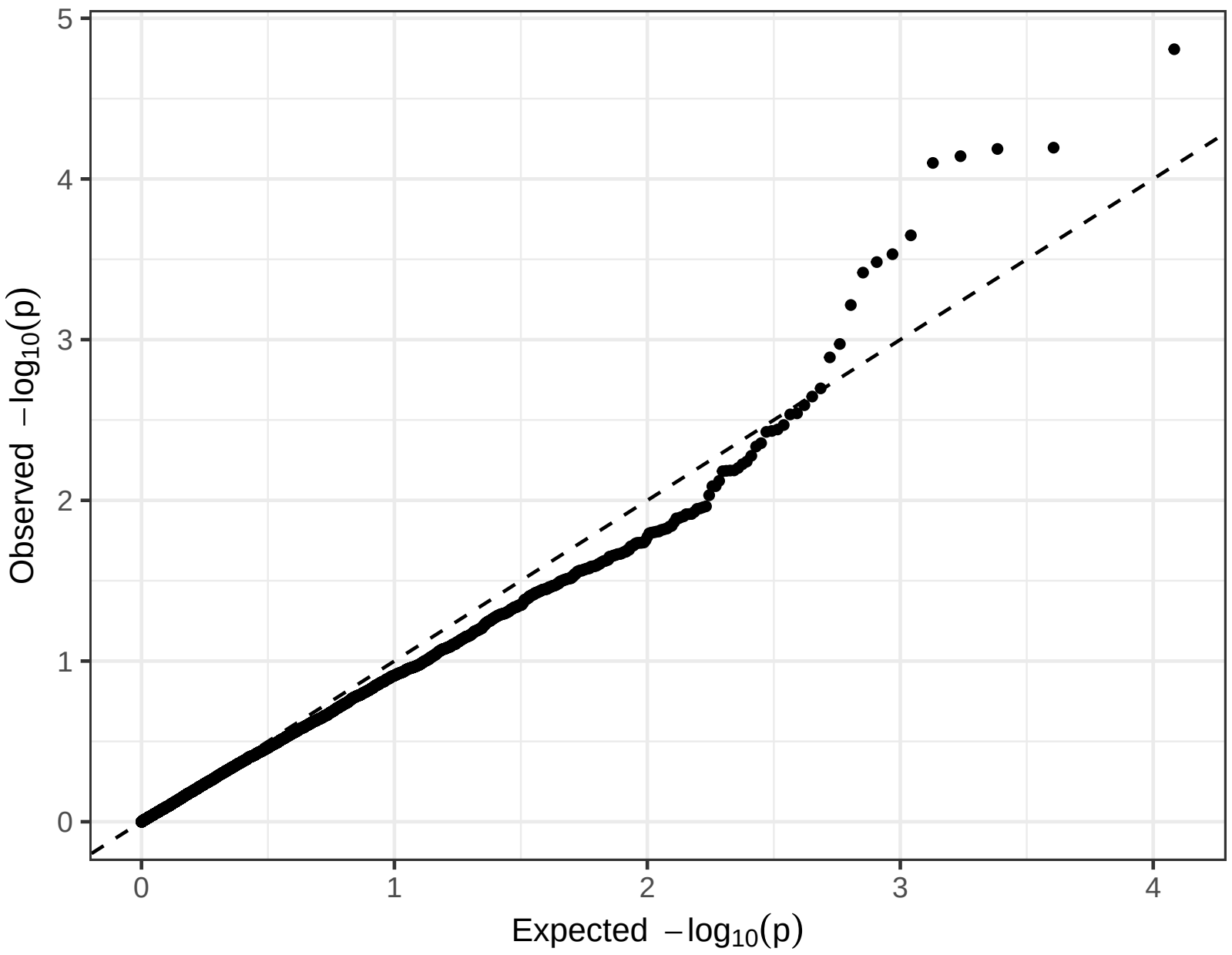

Lachnospiraceae_UCG.007

Cecum | Brain | . = 0.938

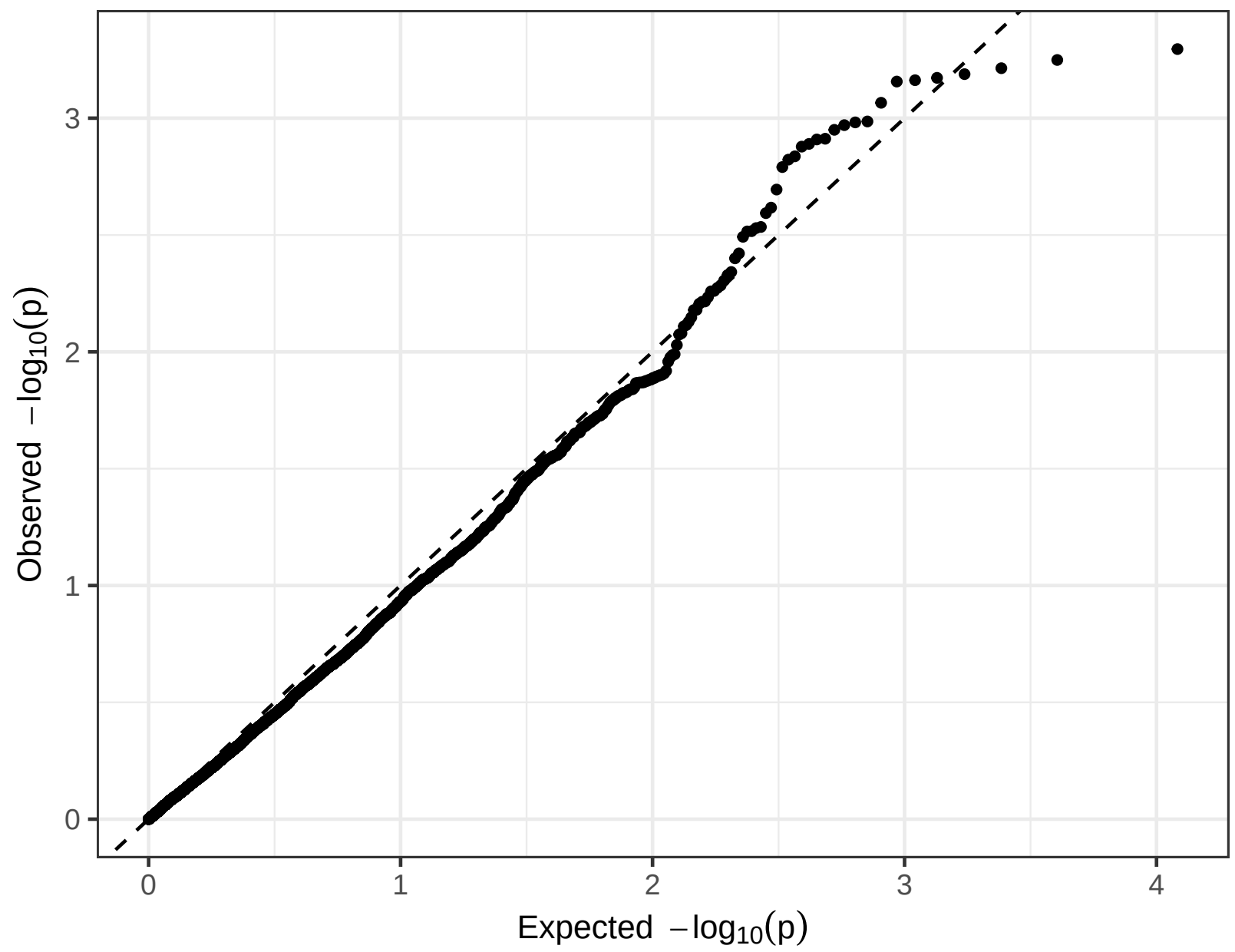

Lachnospiraceae_UCG.008

Cecum | Brain | . = 0.898

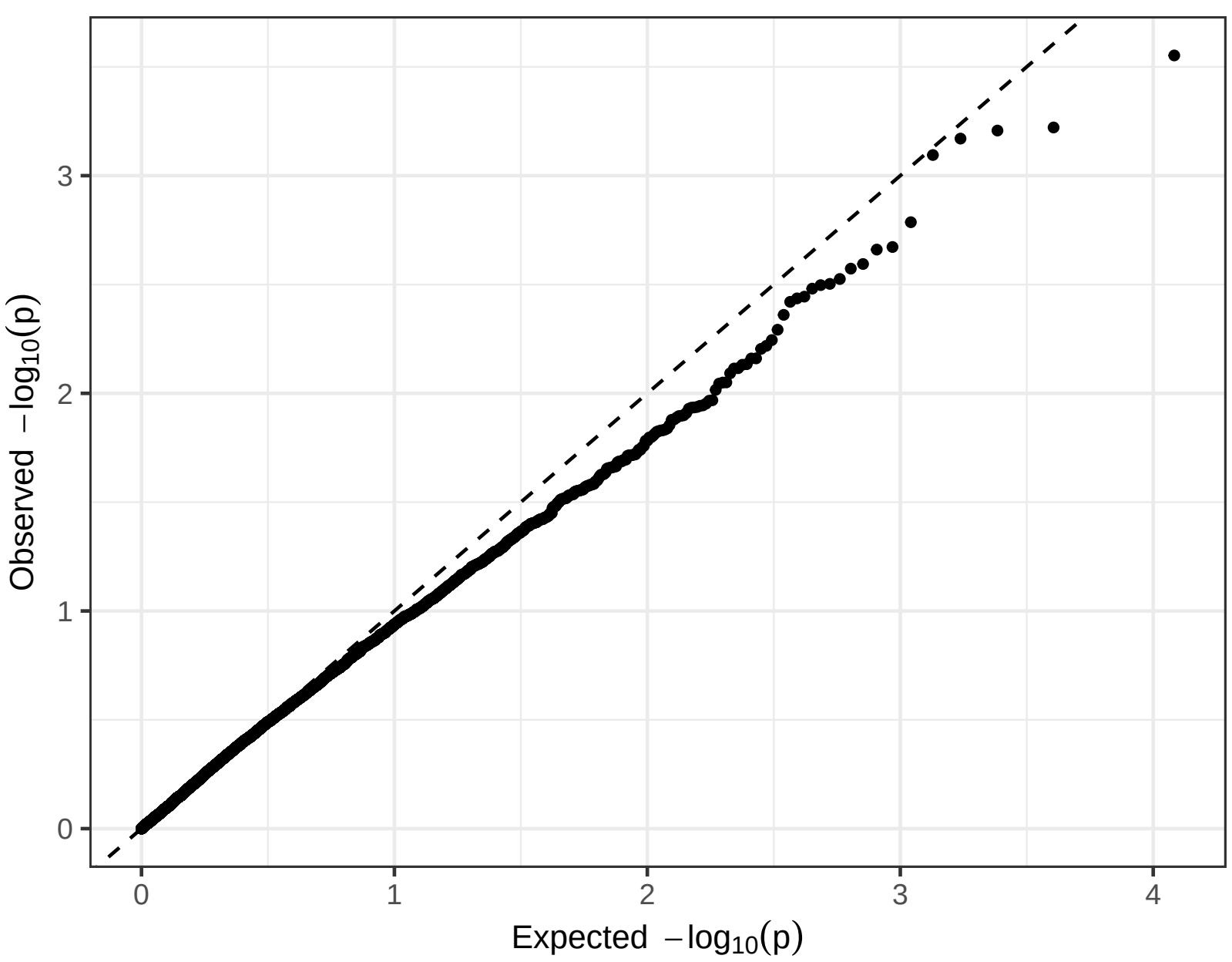

Lachnospiraceae_UCG.009

Cecum | Brain | . = 0.972

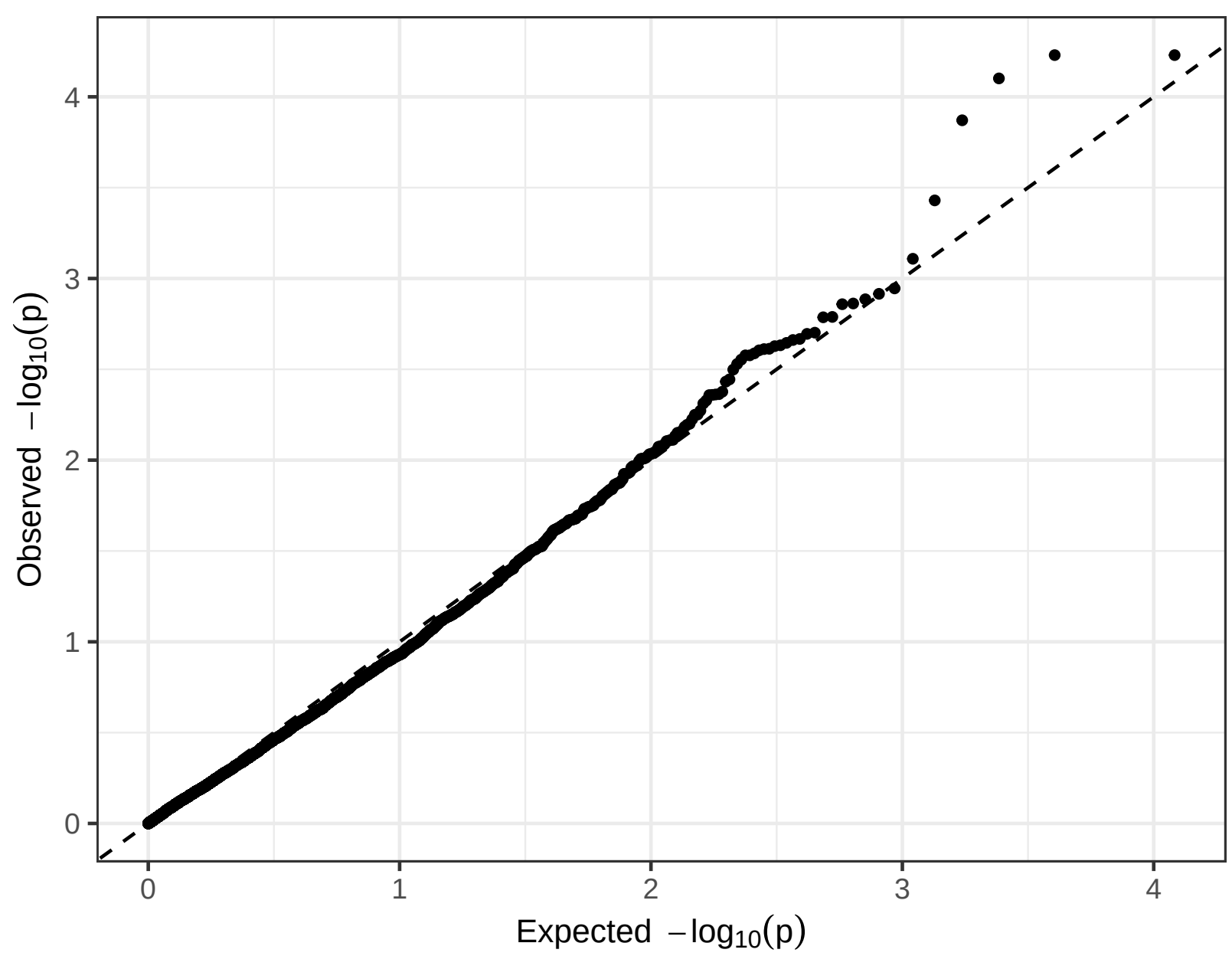

Lachnospiraceae_UCG.010

Cecum | Brain | . = 0.938

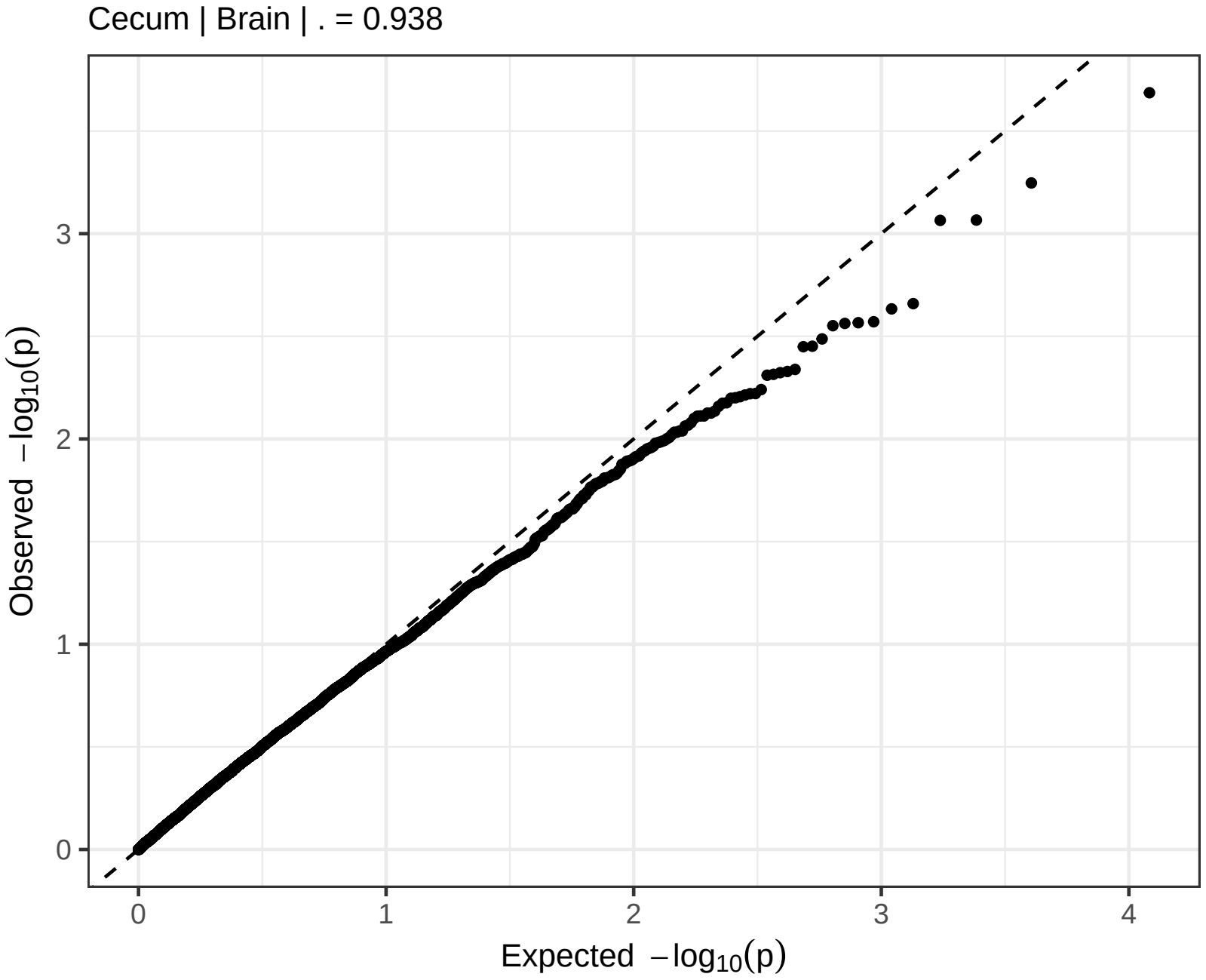

Lachnospiraceae_XPB1014_group

Cecum | Brain | . = 0.986

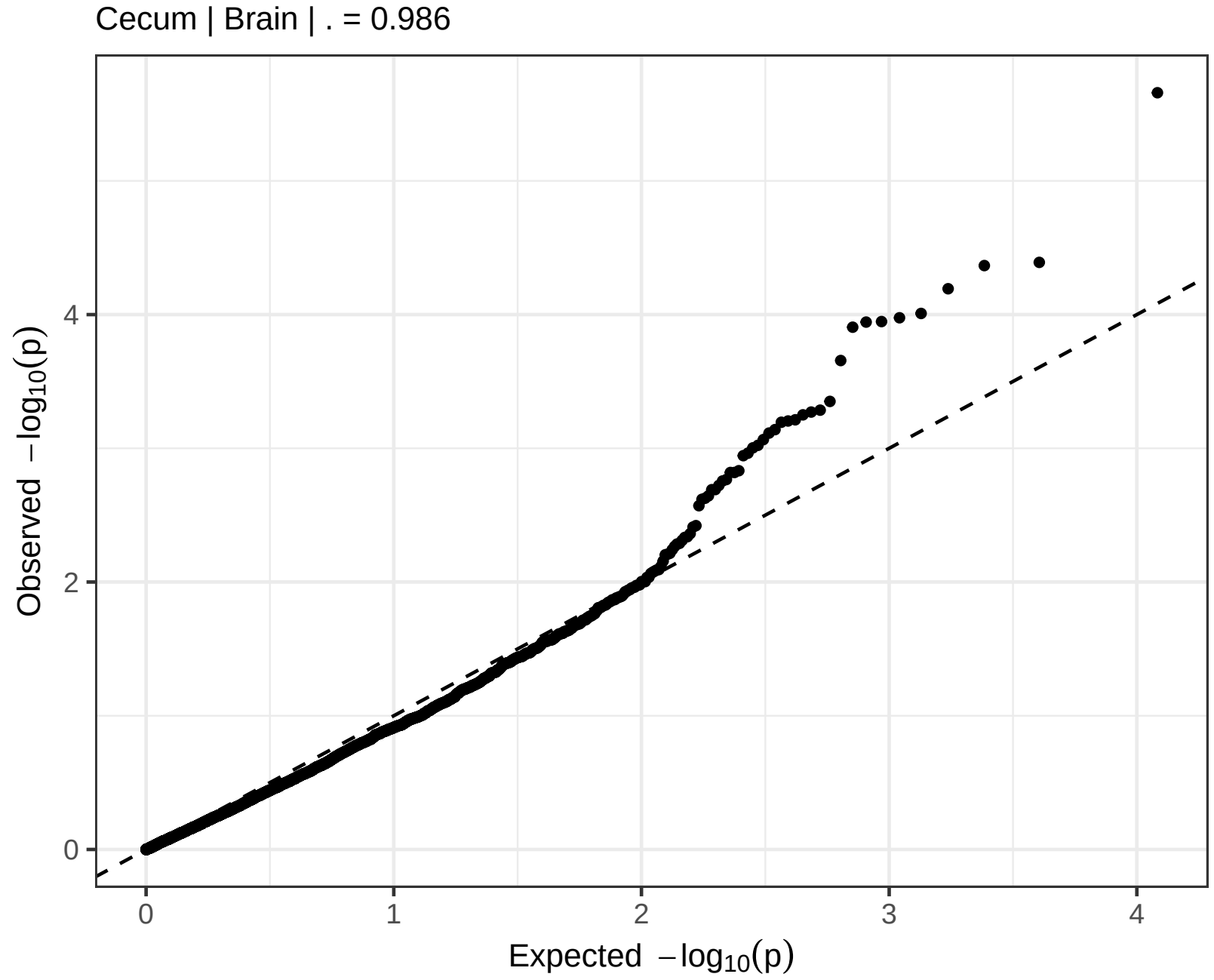

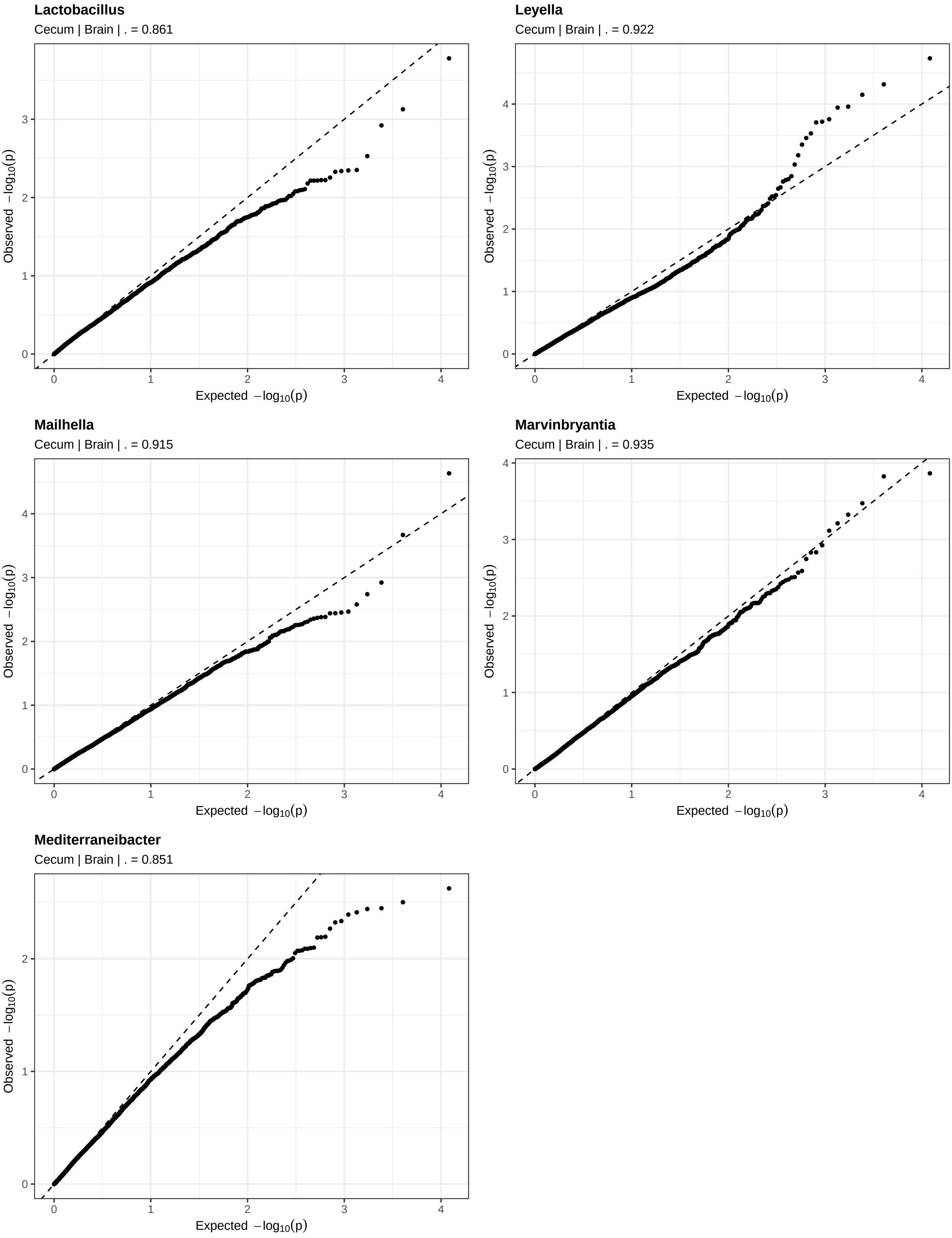

Lactobacillus
Cecum | Brain | . = 0.861
Observed $-\log_{10}(p)$
Expected $-\log_{10}(p)$
Leyella
Cecum | Brain | . = 0.922
Observed $-\log_{10}(p)$
Expected $-\log_{10}(p)$
Mailhella
Cecum | Brain | . = 0.915
Observed $-\log_{10}(p)$
Expected $-\log_{10}(p)$
Marvinbryantia
Cecum | Brain | . = 0.935
Observed $-\log_{10}(p)$
Expected $-\log_{10}(p)$
Mediterraneibacter
Cecum | Brain | . = 0.851
Observed $-\log_{10}(p)$
Expected $-\log_{10}(p)$

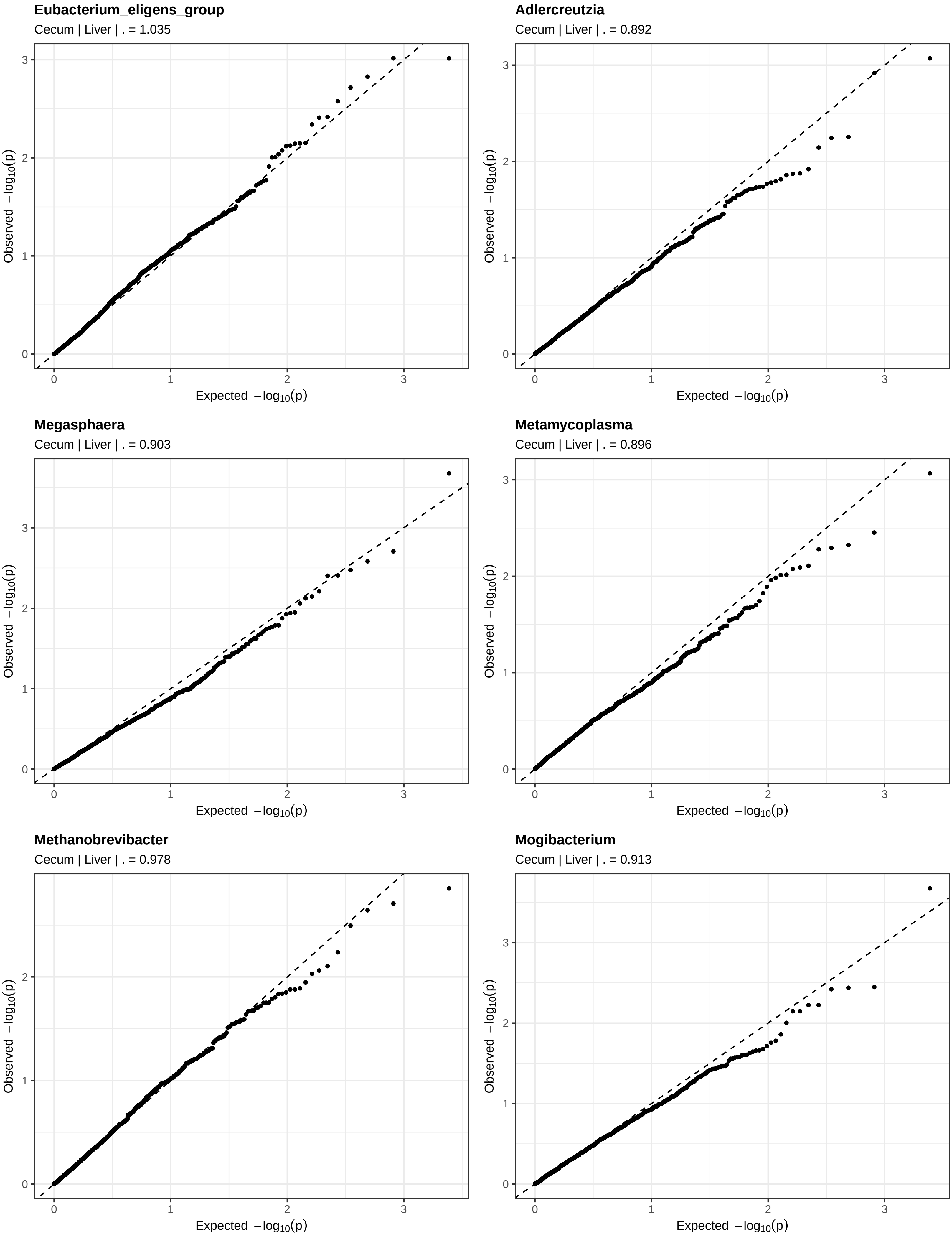
Eubacterium_eligens_group
Cecum | Liver | . = 1.035
Adlercreutzia
Cecum | Liver | . = 0.892
Megasphaera
Cecum | Liver | . = 0.903
Metamycoplasma
Cecum | Liver | . = 0.896
Methanobrevibacter
Cecum | Liver | . = 0.978
Mogibacterium
Cecum | Liver | . = 0.913
Observed $-\log_{10}(p)$
Expected $-\log_{10}(p)$

Monoglobus

Cecum | Liver | . = 1.117

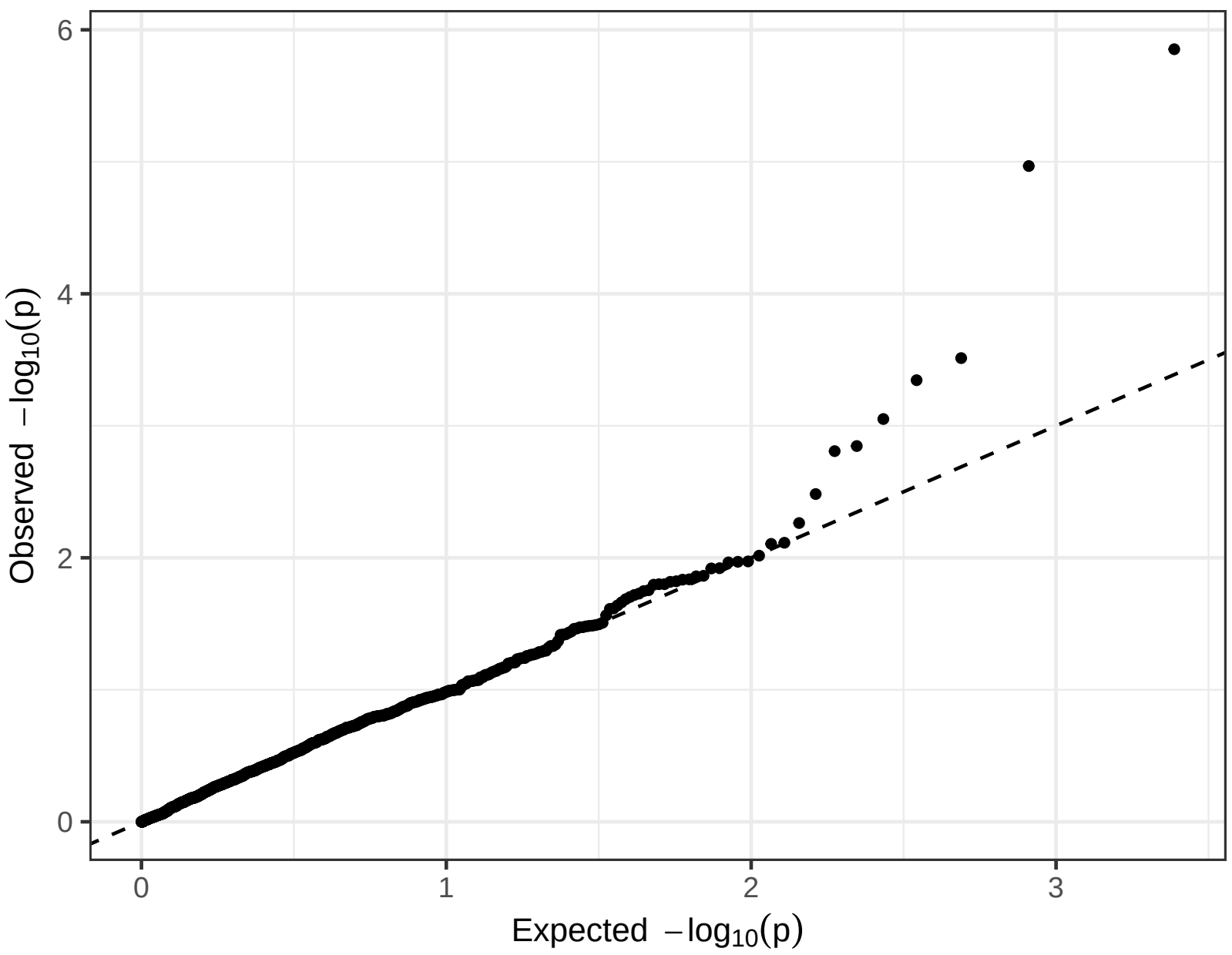

Negativibacillus

Cecum | Liver | . = 0.954

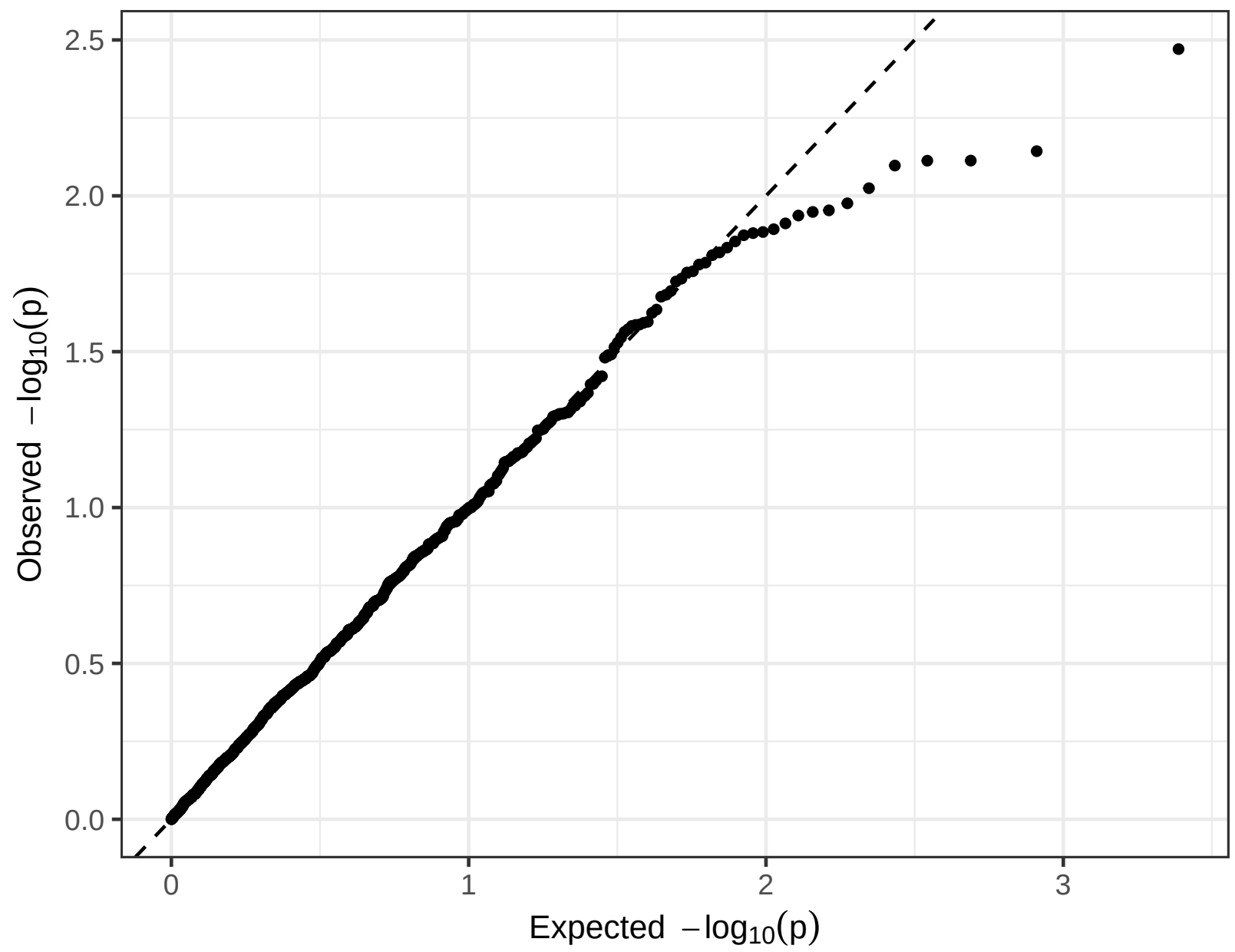

NK4A214_group

Cecum | Liver | . = 0.864

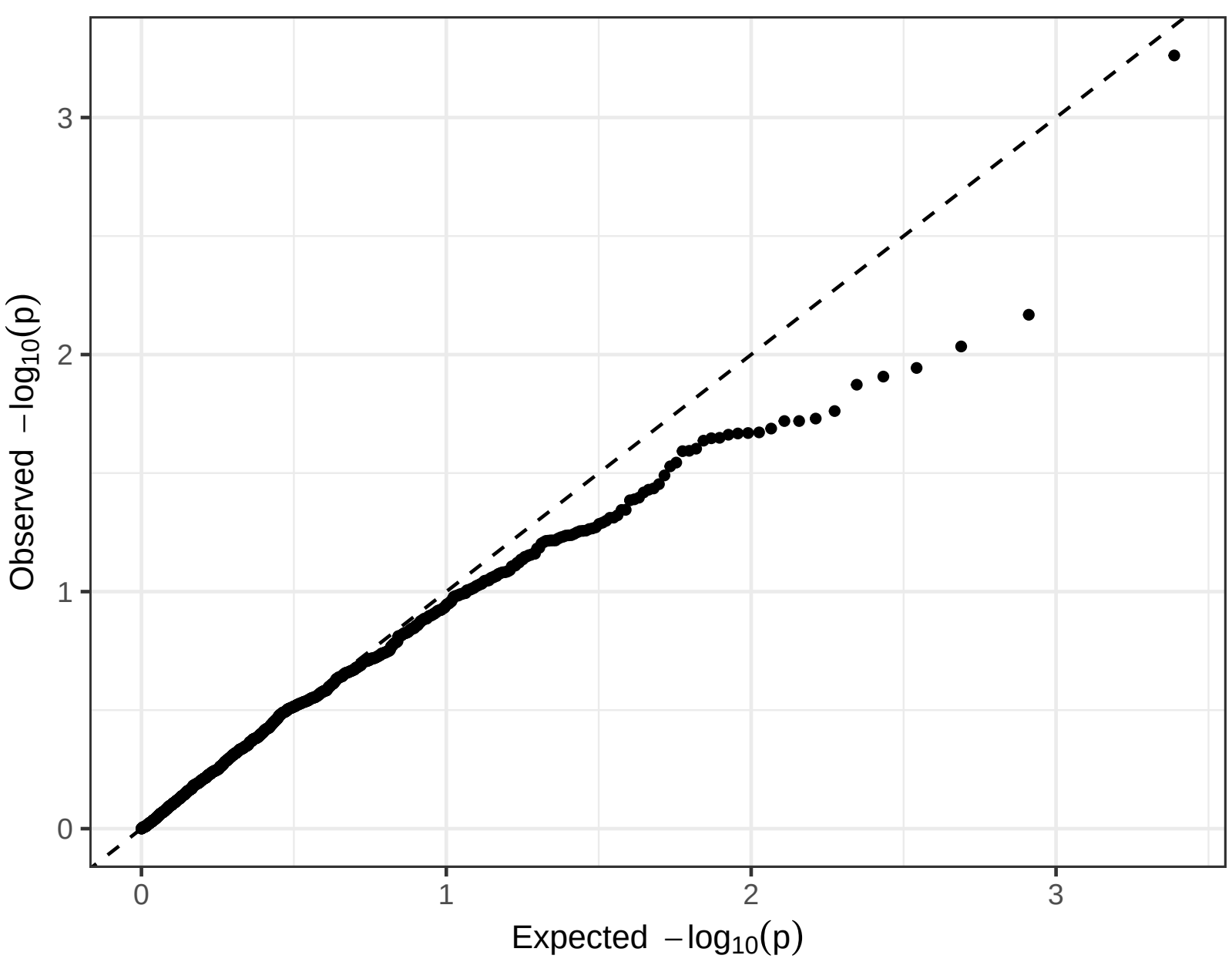

Oribacterium

Cecum | Liver | . = 0.863

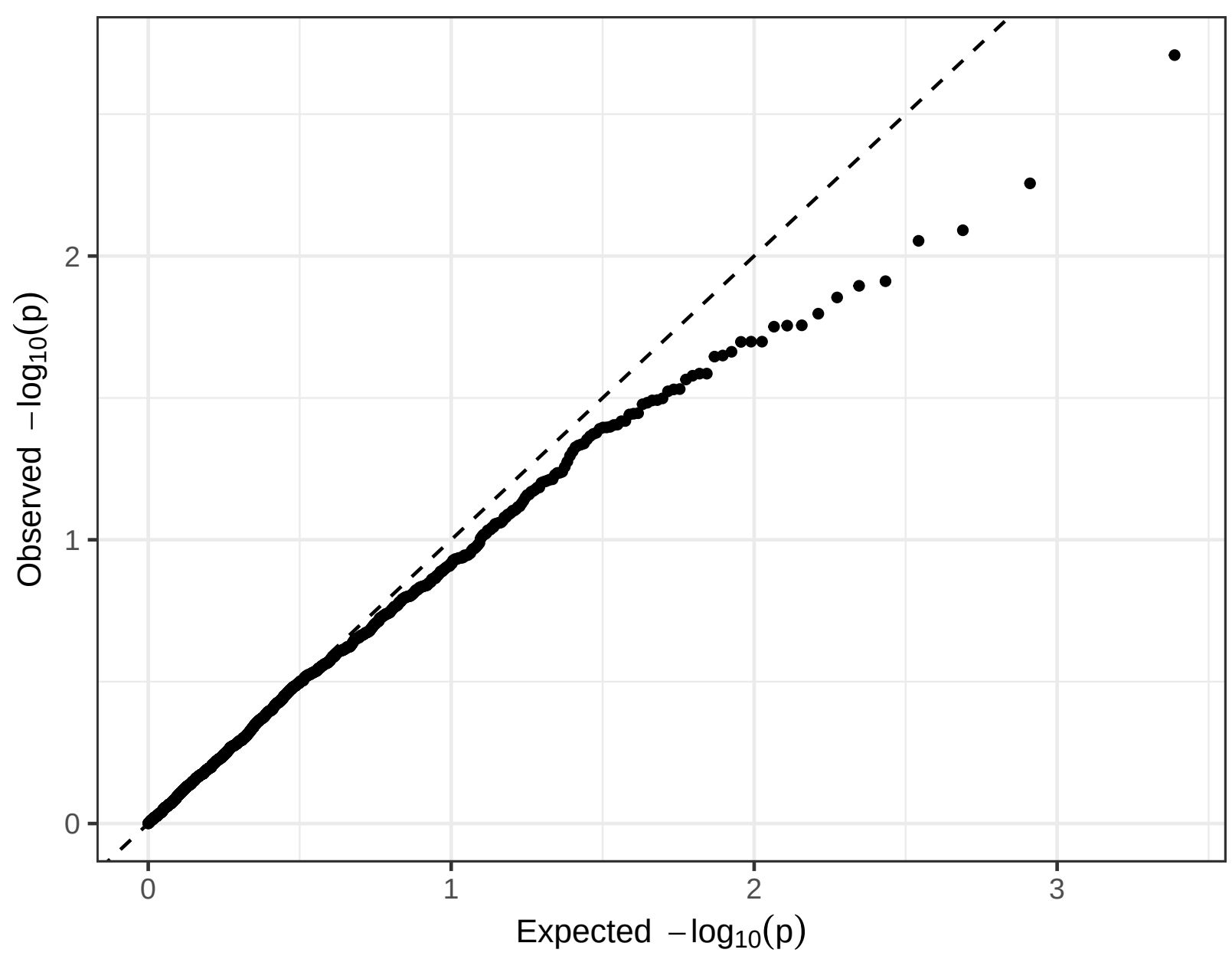

Oscillibacter

Cecum | Liver | . = 0.86

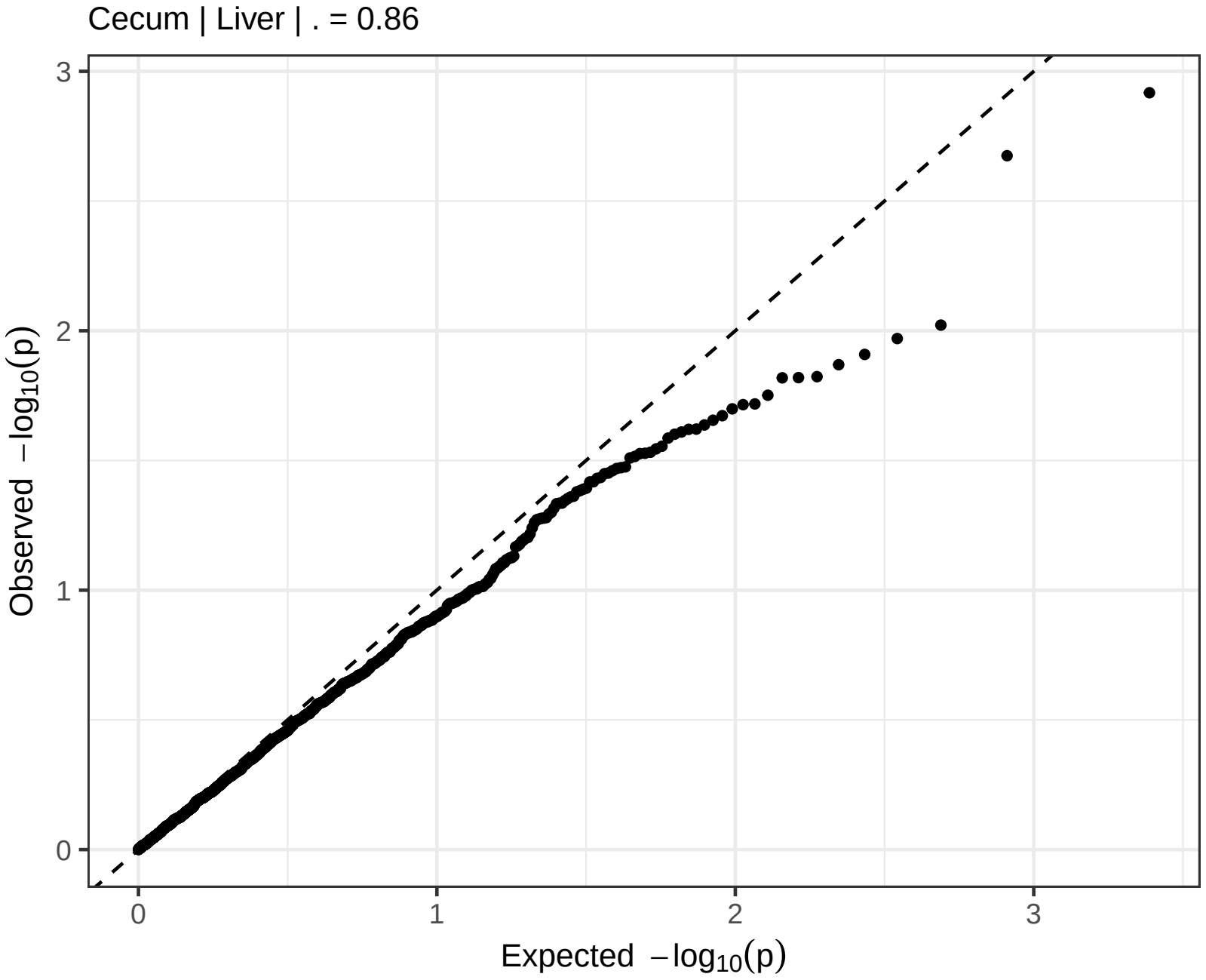

Oscillospira

Cecum | Liver | . = 0.977

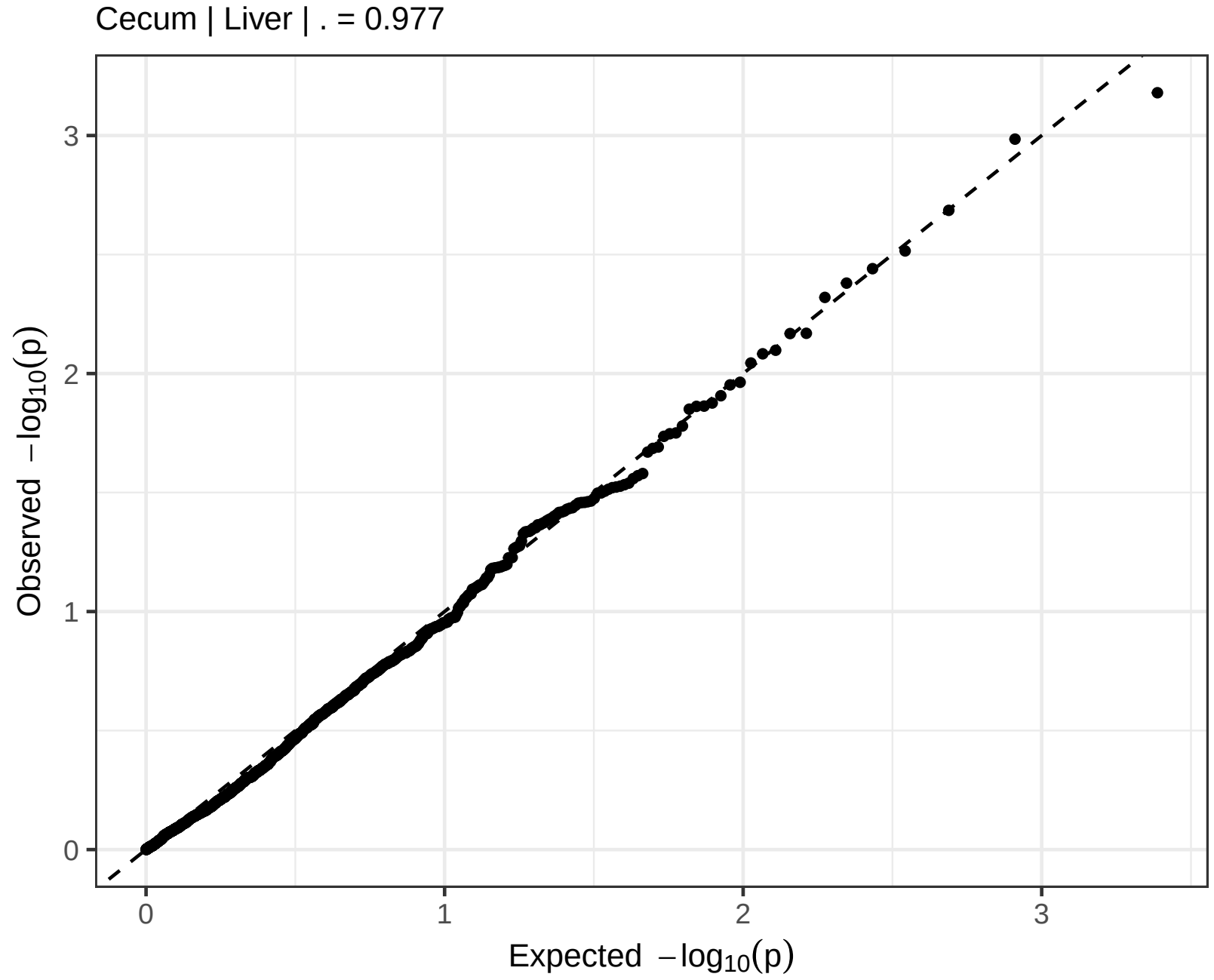

Agathobacter

Cecum | Liver | . = 0.999

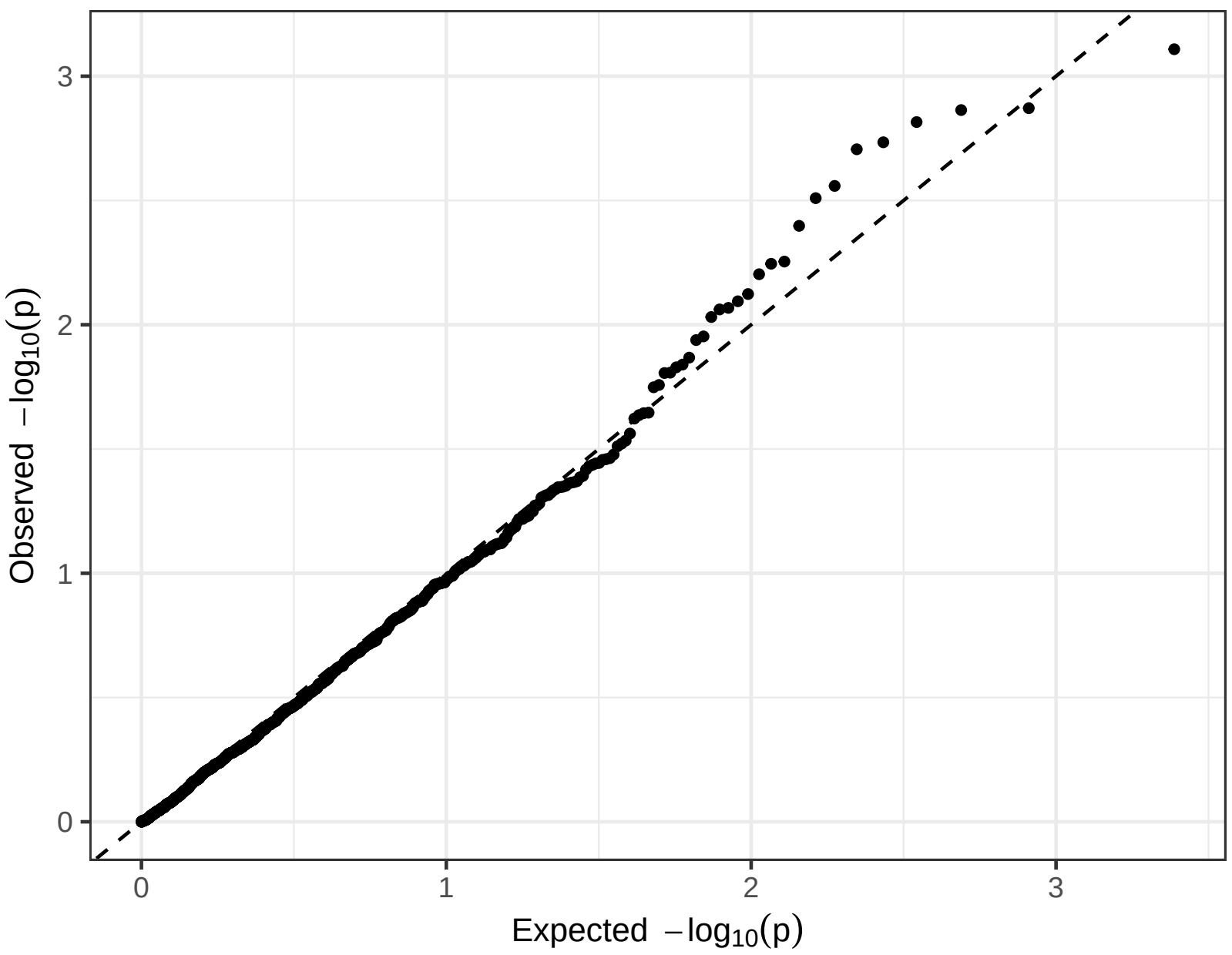

Oxalobacter

Cecum | Liver | . = 0.864

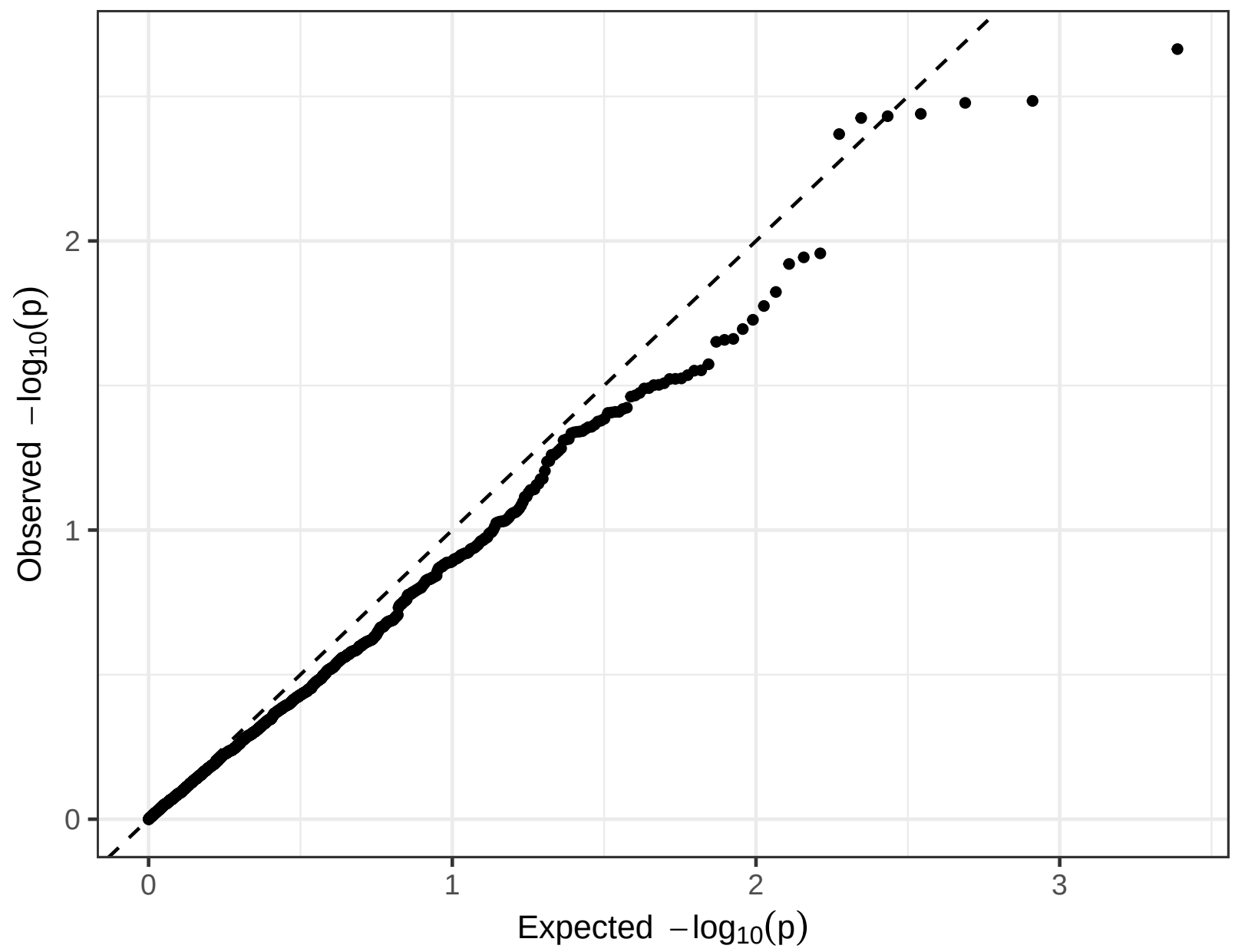

p.1088.a5_gut_group

Cecum | Liver | . = 0.889

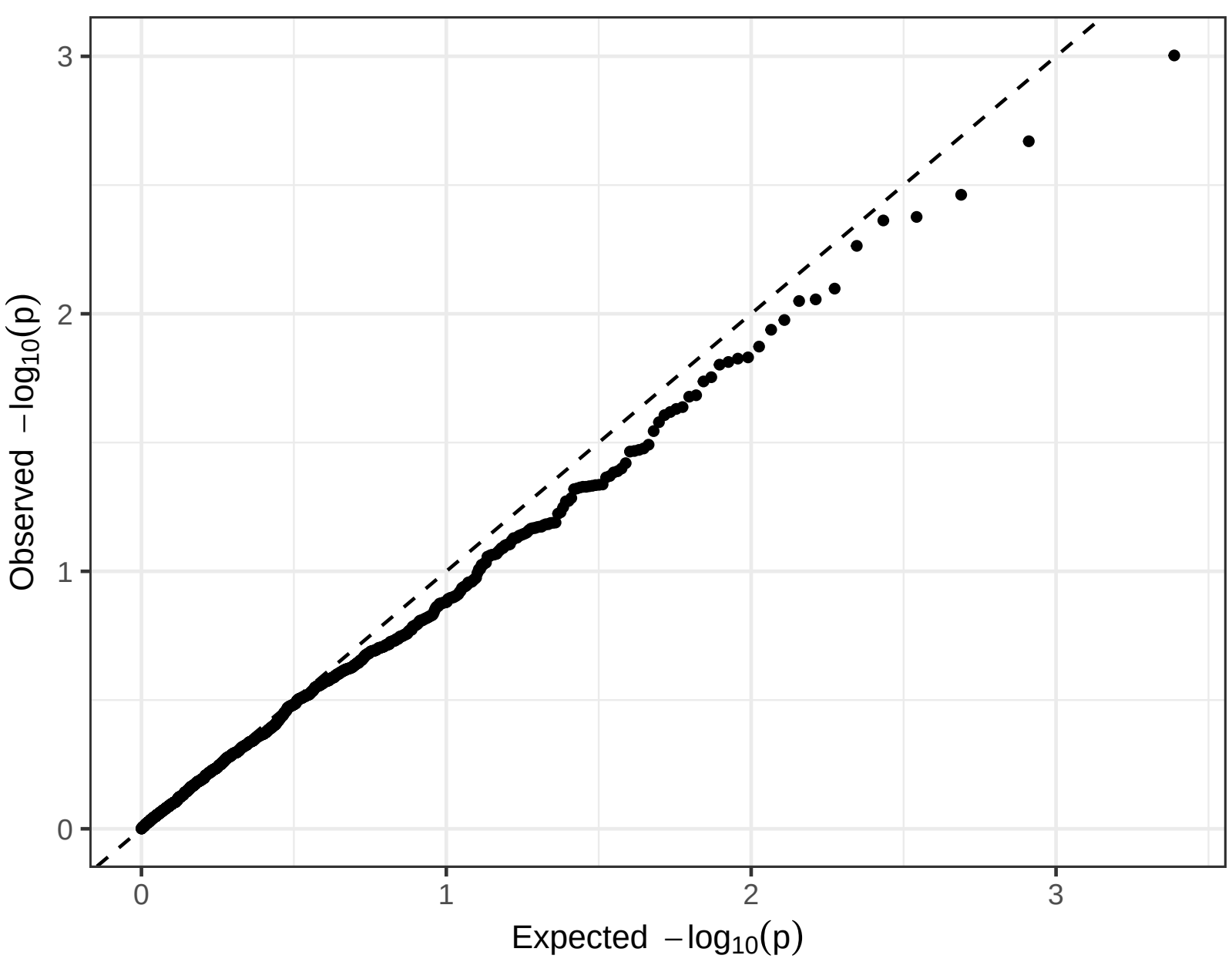

Paludicola

Cecum | Liver | . = 0.956

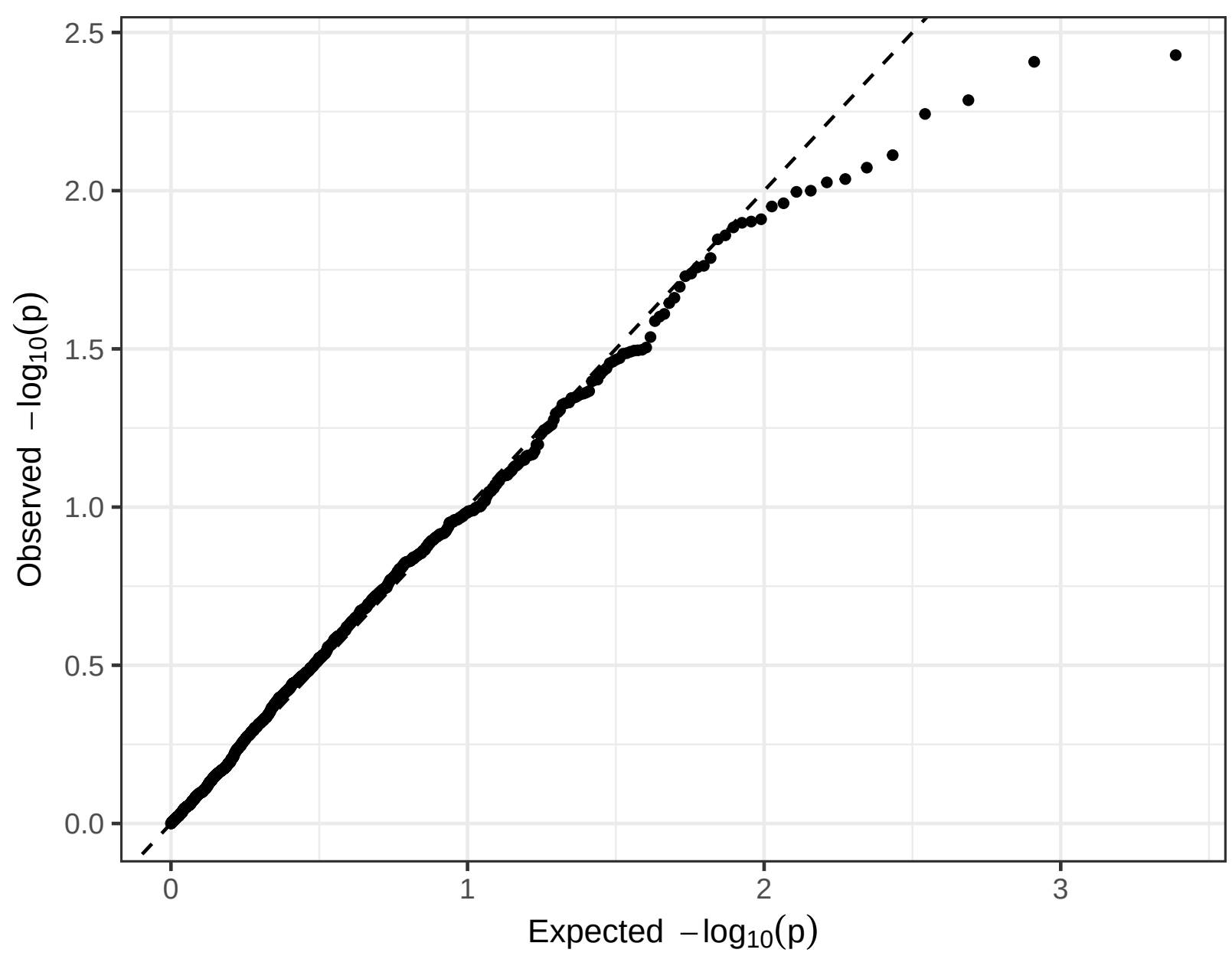

Parabacteroides

Cecum | Liver | . = 0.844

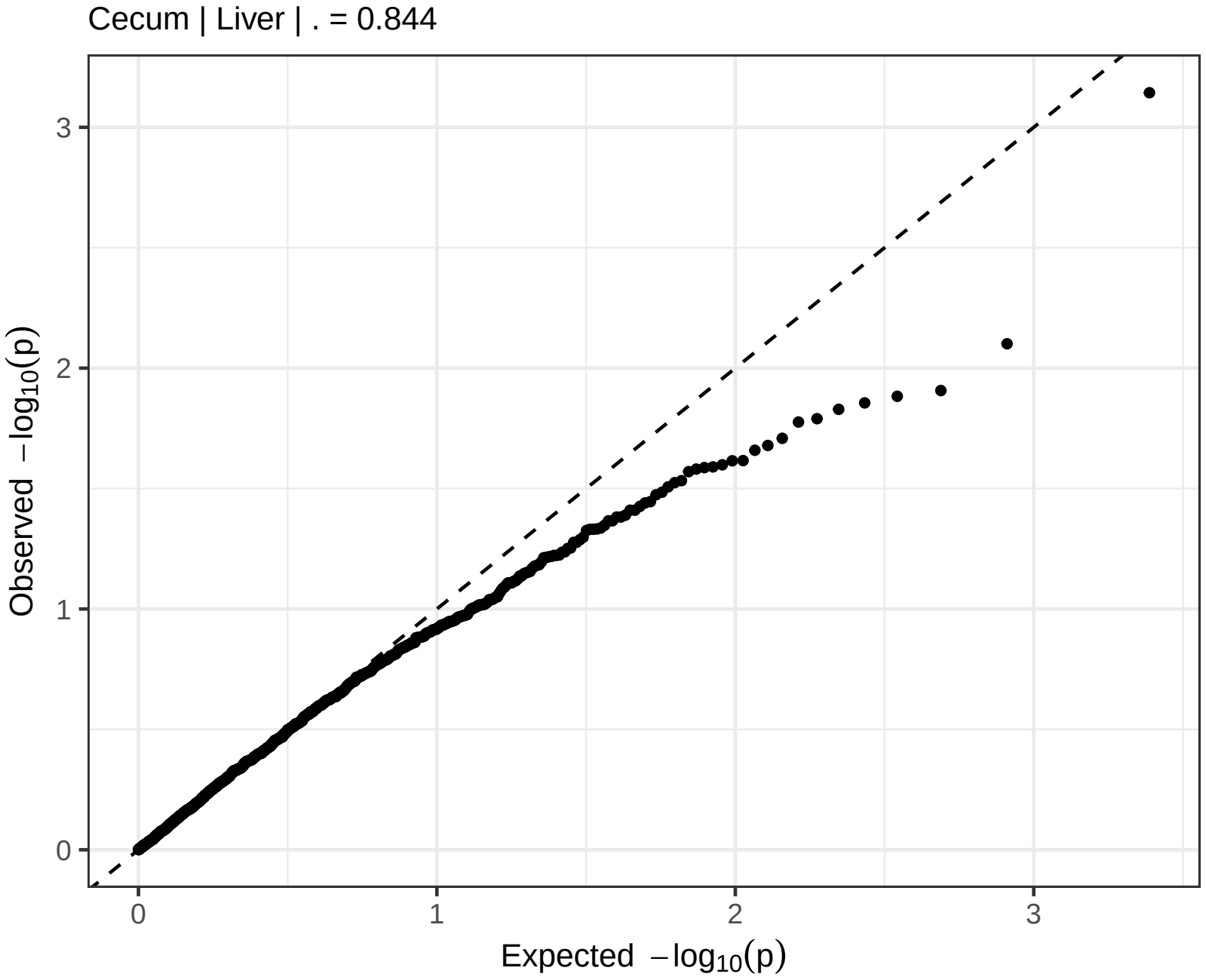

Parasutterella

Cecum | Liver | . = 0.987

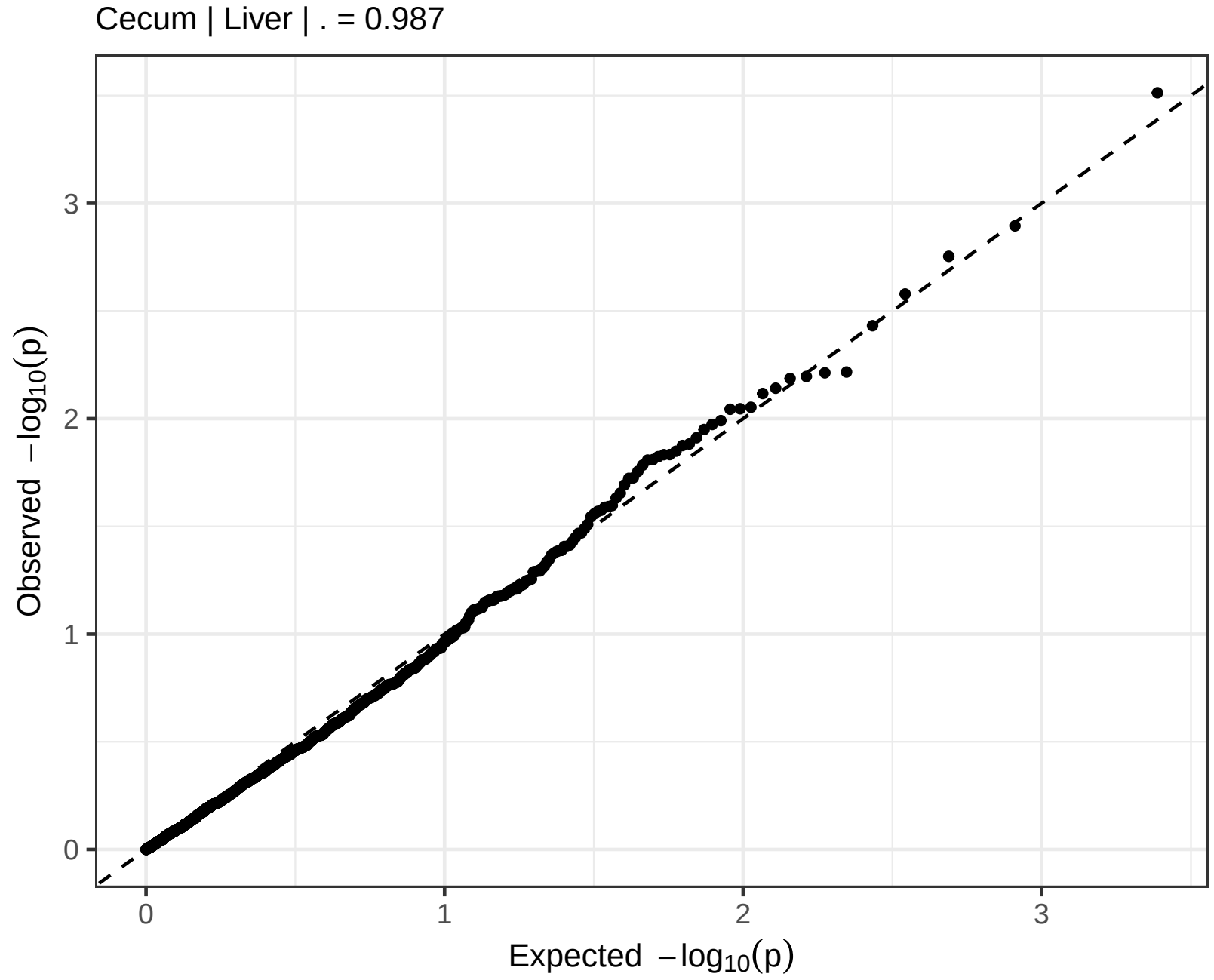

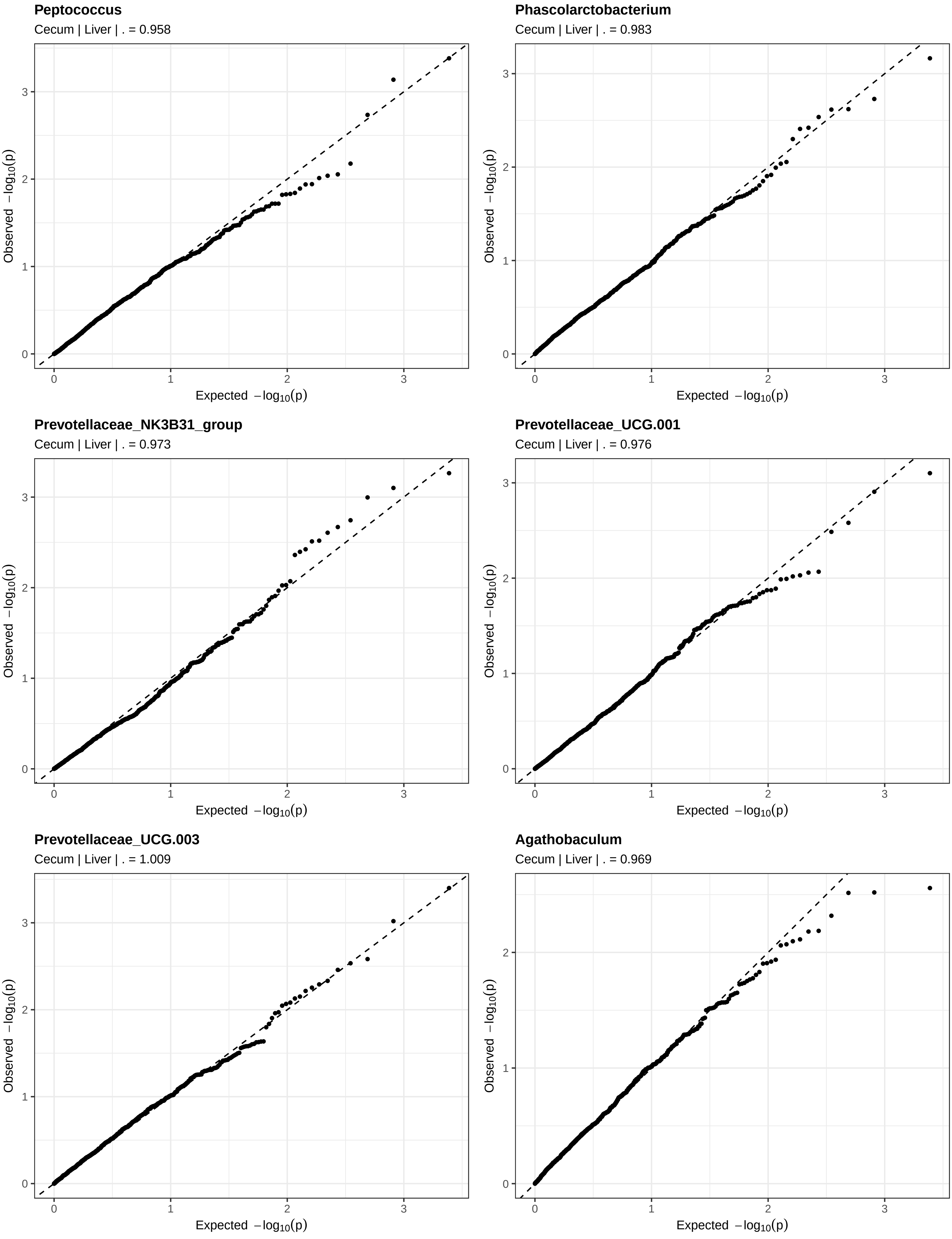
Peptococcus
Cecum | Liver | . = 0.958
Phascolarctobacterium
Cecum | Liver | . = 0.983
Prevotellaceae_NK3B31_group
Cecum | Liver | . = 0.973
Prevotellaceae_UCG.001
Cecum | Liver | . = 0.976
Prevotellaceae_UCG.003
Cecum | Liver | . = 1.009
Agathobaculum
Cecum | Liver | . = 0.969
Observed −log10(p)
Expected −log10(p)

Prevotellaceae_UCG.004

Cecum | Liver | . = 1.139

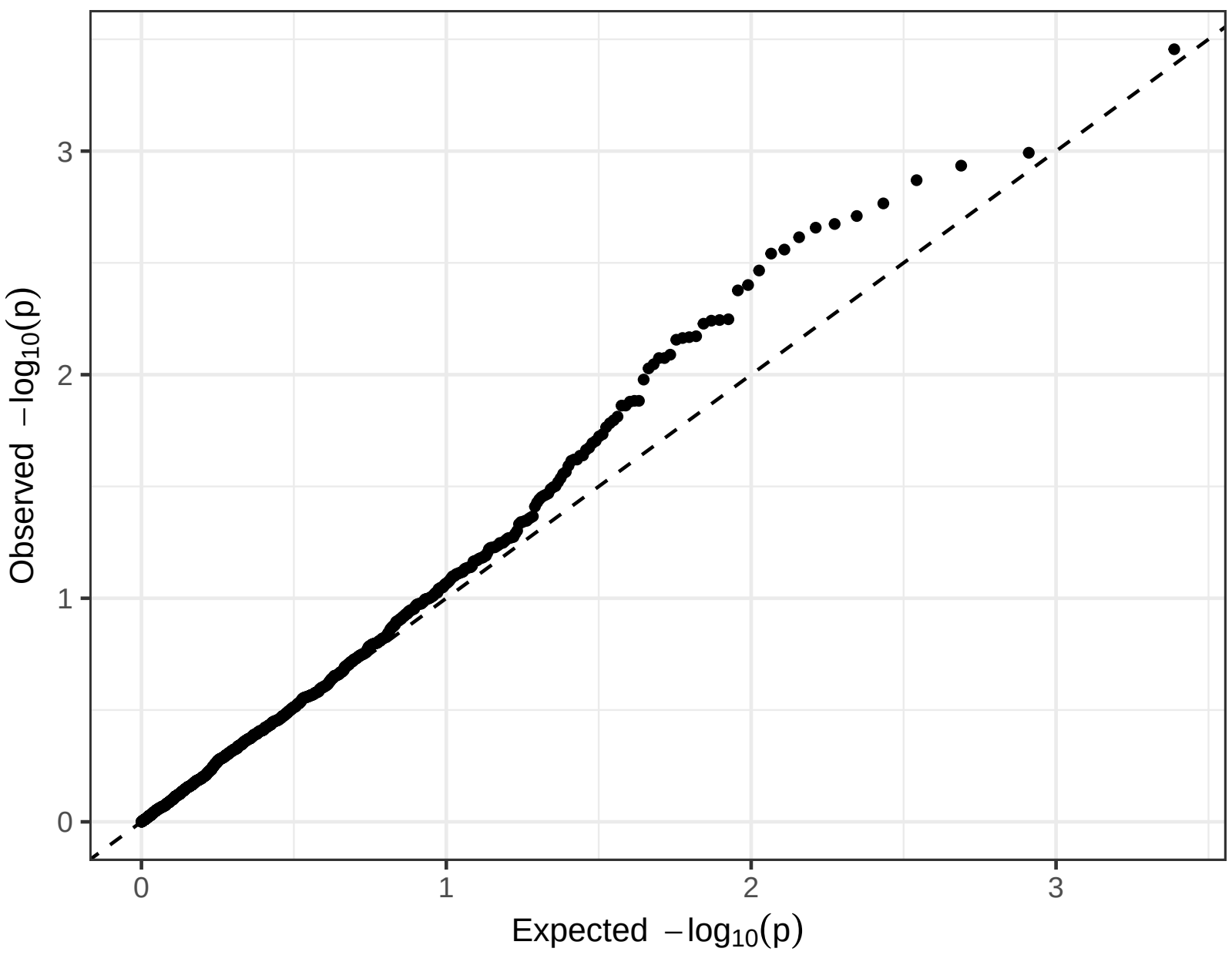

Pygmaiobacter

Cecum | Liver | . = 0.859

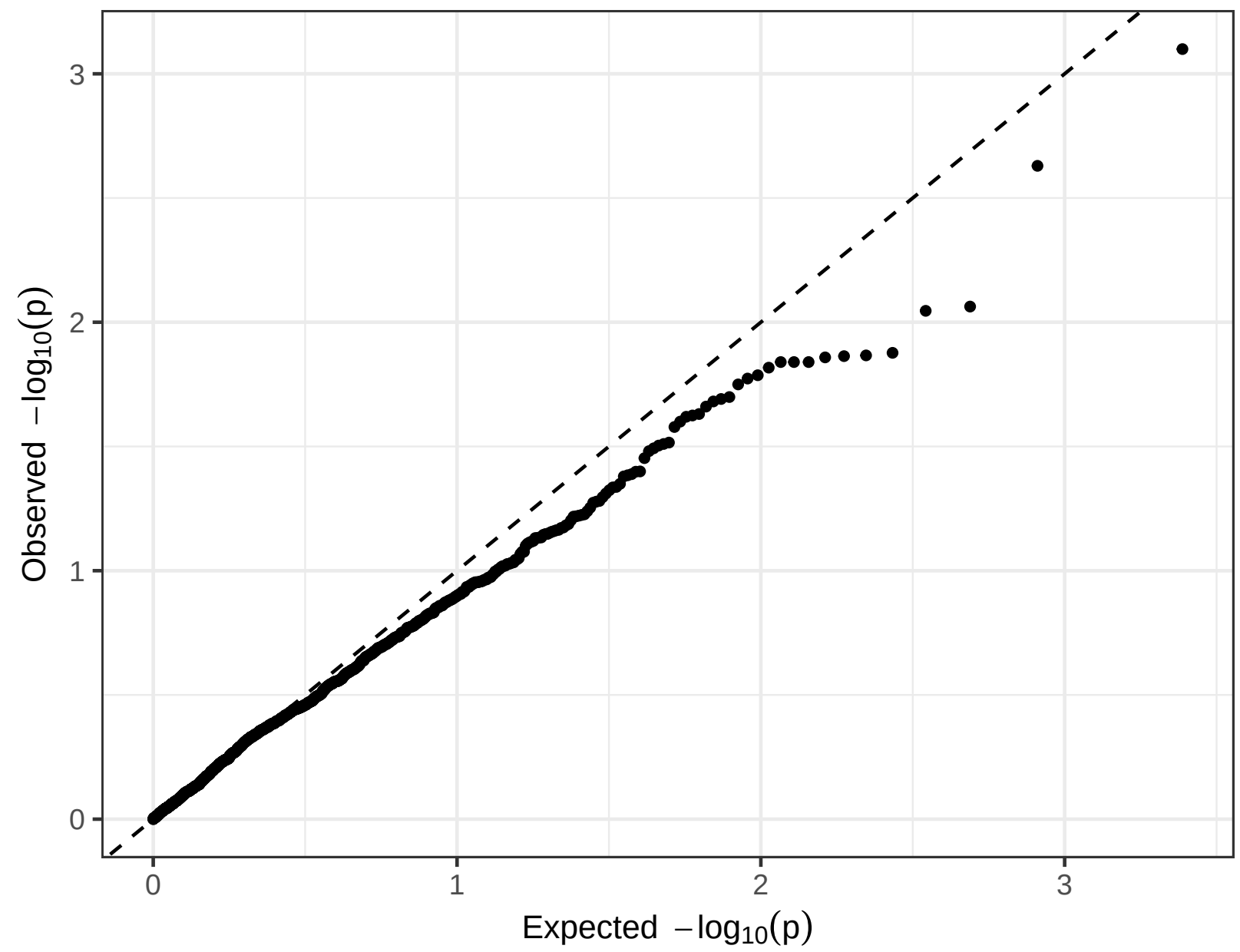

Pyramidobacter

Cecum | Liver | . = 0.875

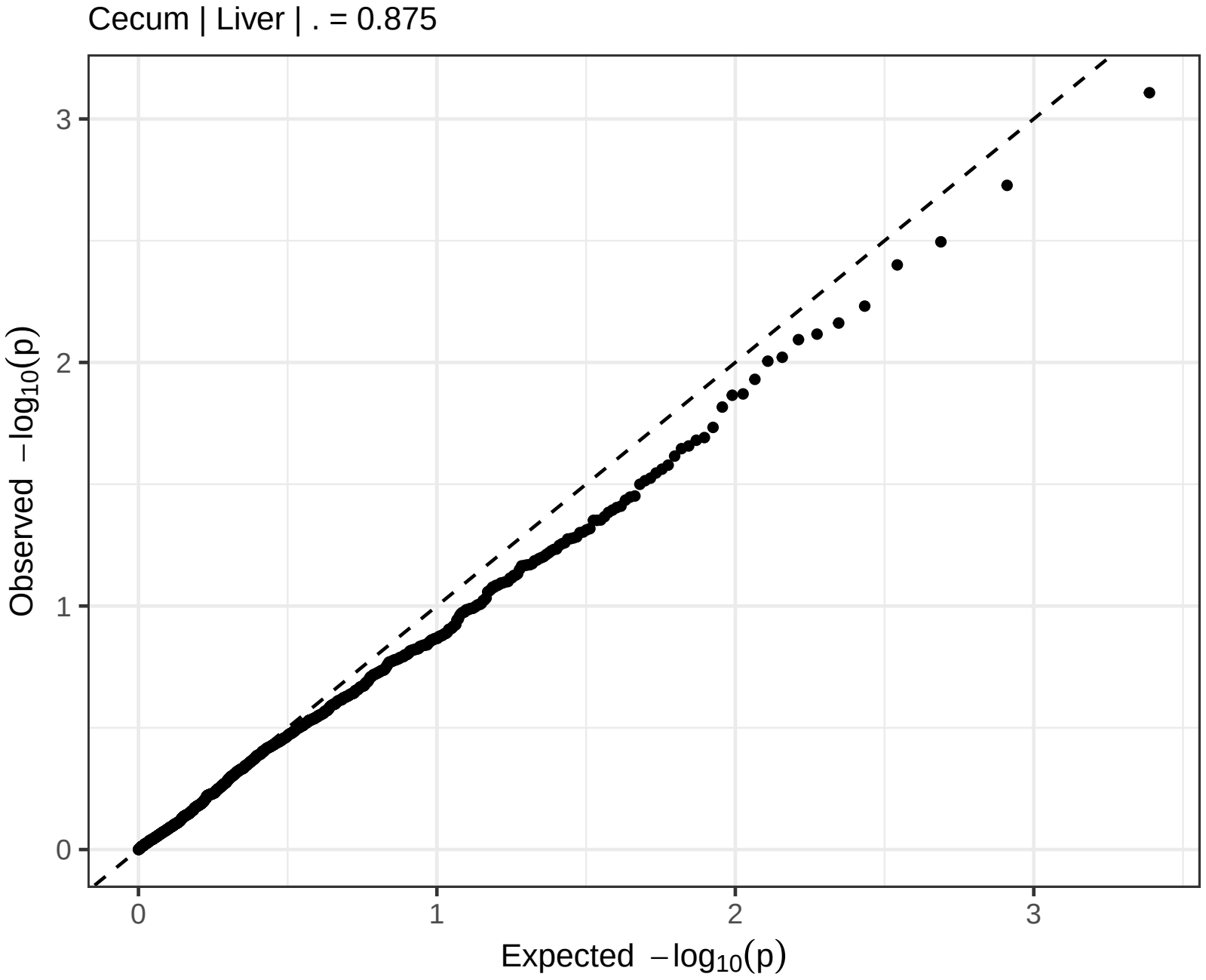

Quinella

Cecum | Liver | . = 0.898

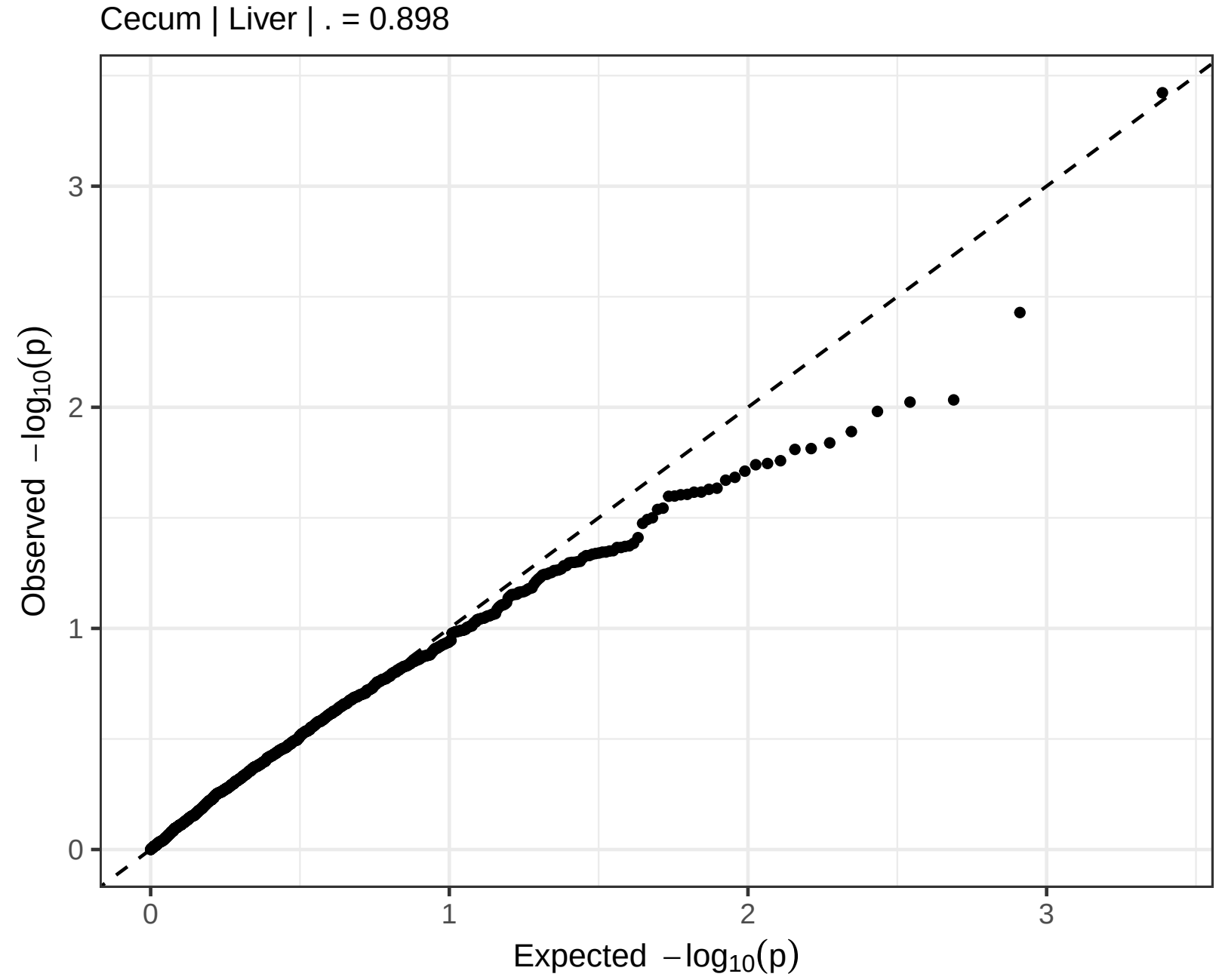

Rikenellaceae_RC9_gut_group

Cecum | Liver | . = 0.923

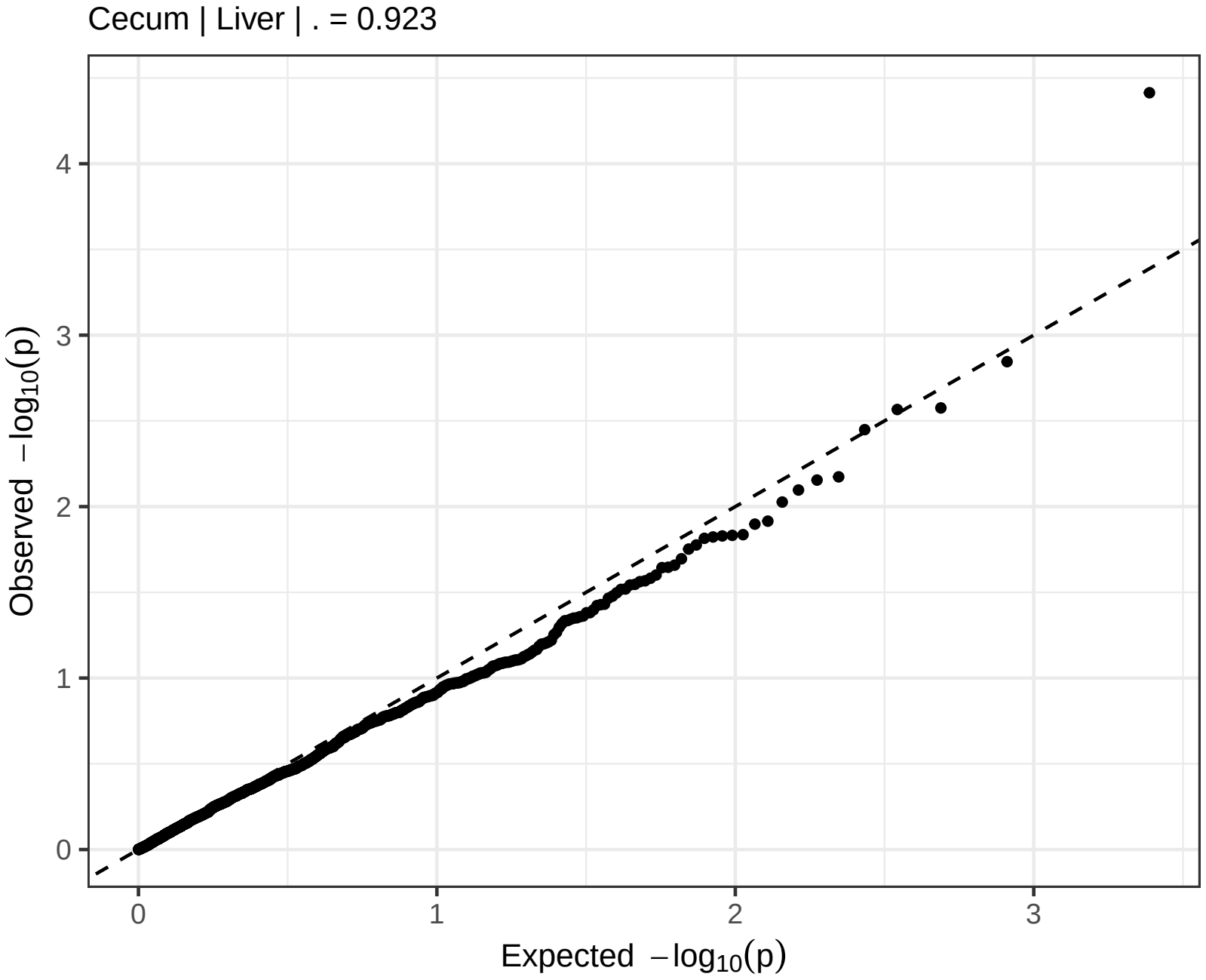

Romboutsia

Cecum | Liver | . = 0.889

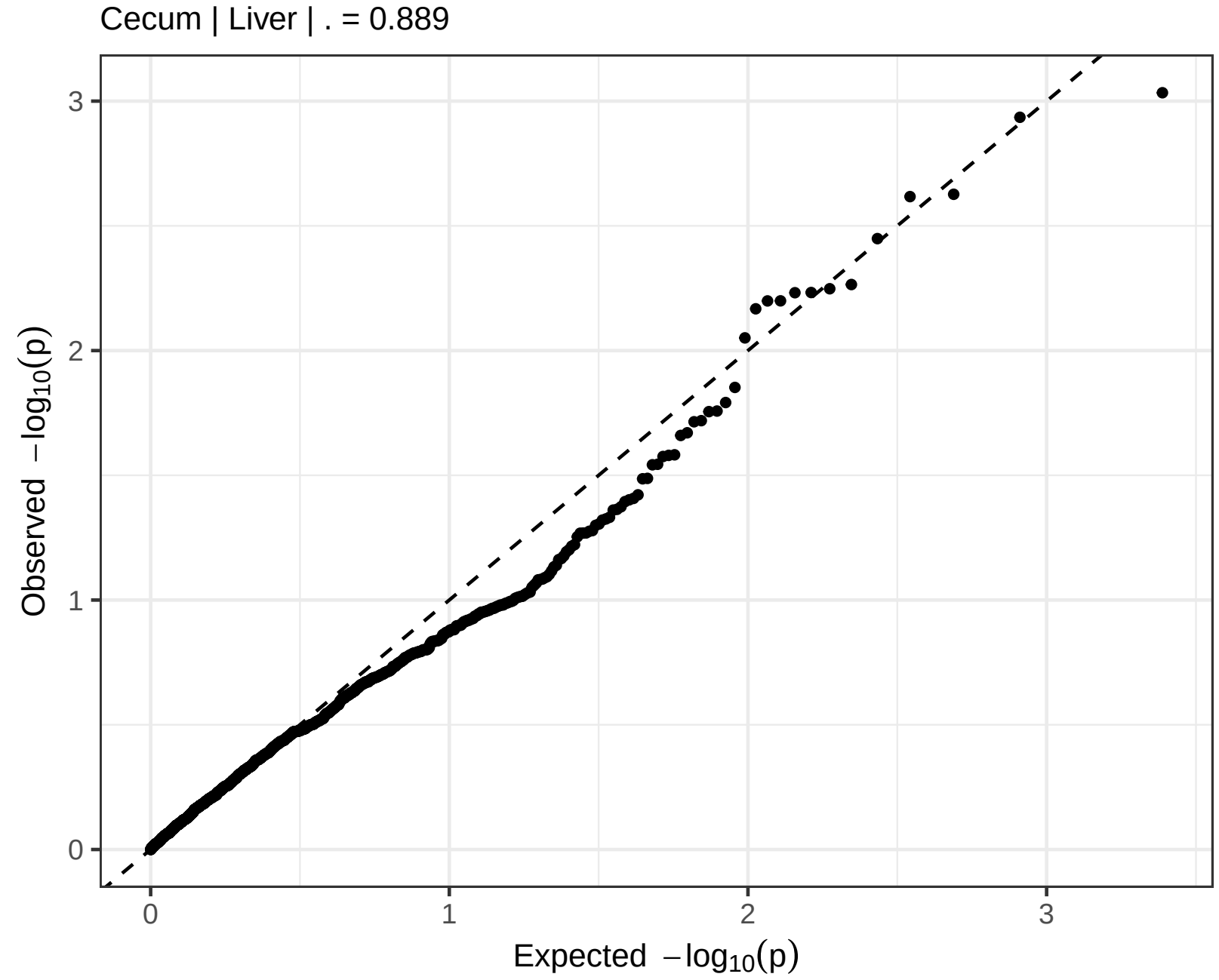

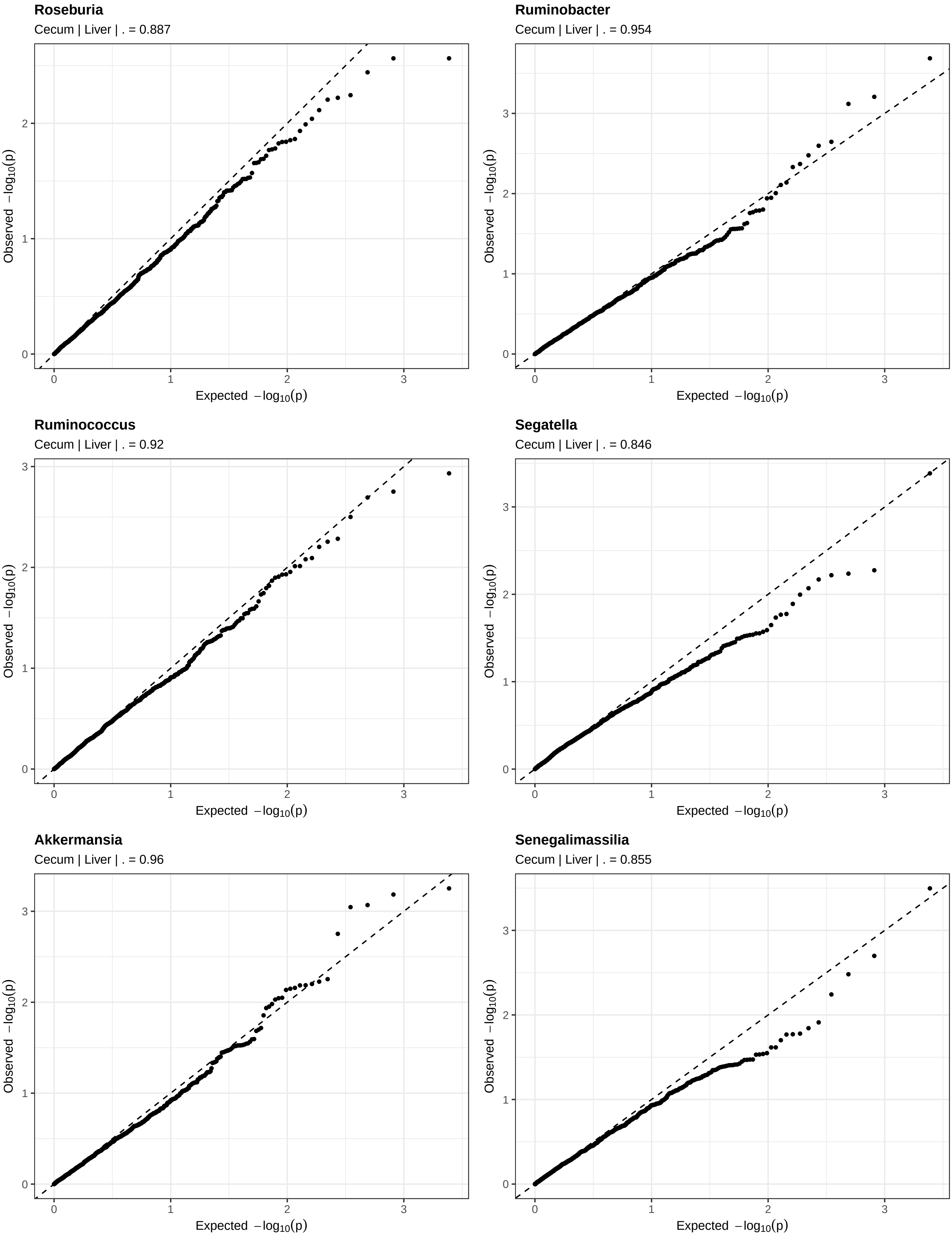
Roseburia
Cecum | Liver | . = 0.887
Ruminobacter
Cecum | Liver | . = 0.954
Ruminococcus
Cecum | Liver | . = 0.92
Segatella
Cecum | Liver | . = 0.846
Akkermansia
Cecum | Liver | . = 0.96
Senegalimassilia
Cecum | Liver | . = 0.855
Observed $-\log_{10}(p)$
Expected $-\log_{10}(p)$

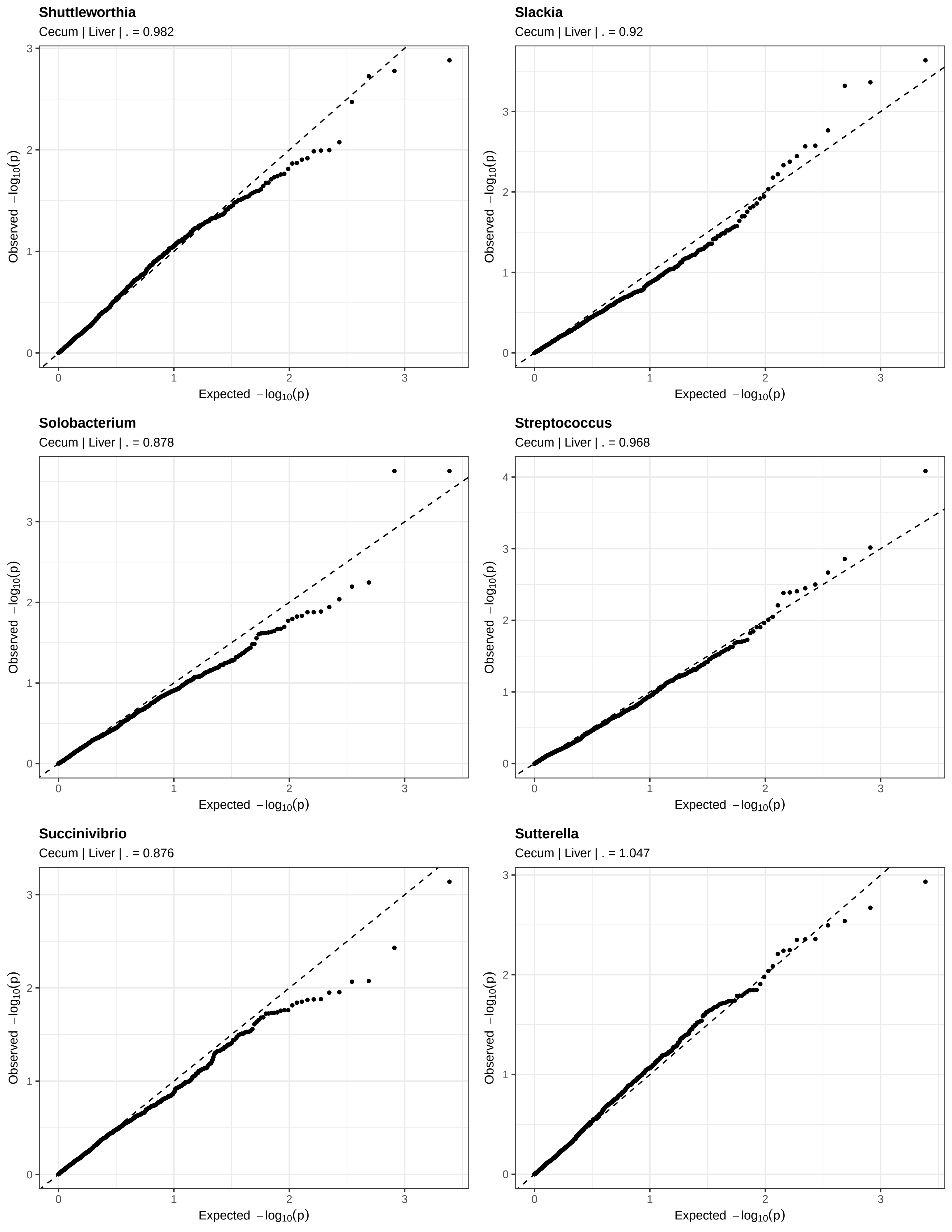

Shuttleworthia
Cecum | Liver | . = 0.982
Slackia
Cecum | Liver | . = 0.92
Solobacterium
Cecum | Liver | . = 0.878
Streptococcus
Cecum | Liver | . = 0.968
Succinivibrio
Cecum | Liver | . = 0.876
Sutterella
Cecum | Liver | . = 1.047
Observed $-\log_{10}(p)$
Expected $-\log_{10}(p)$

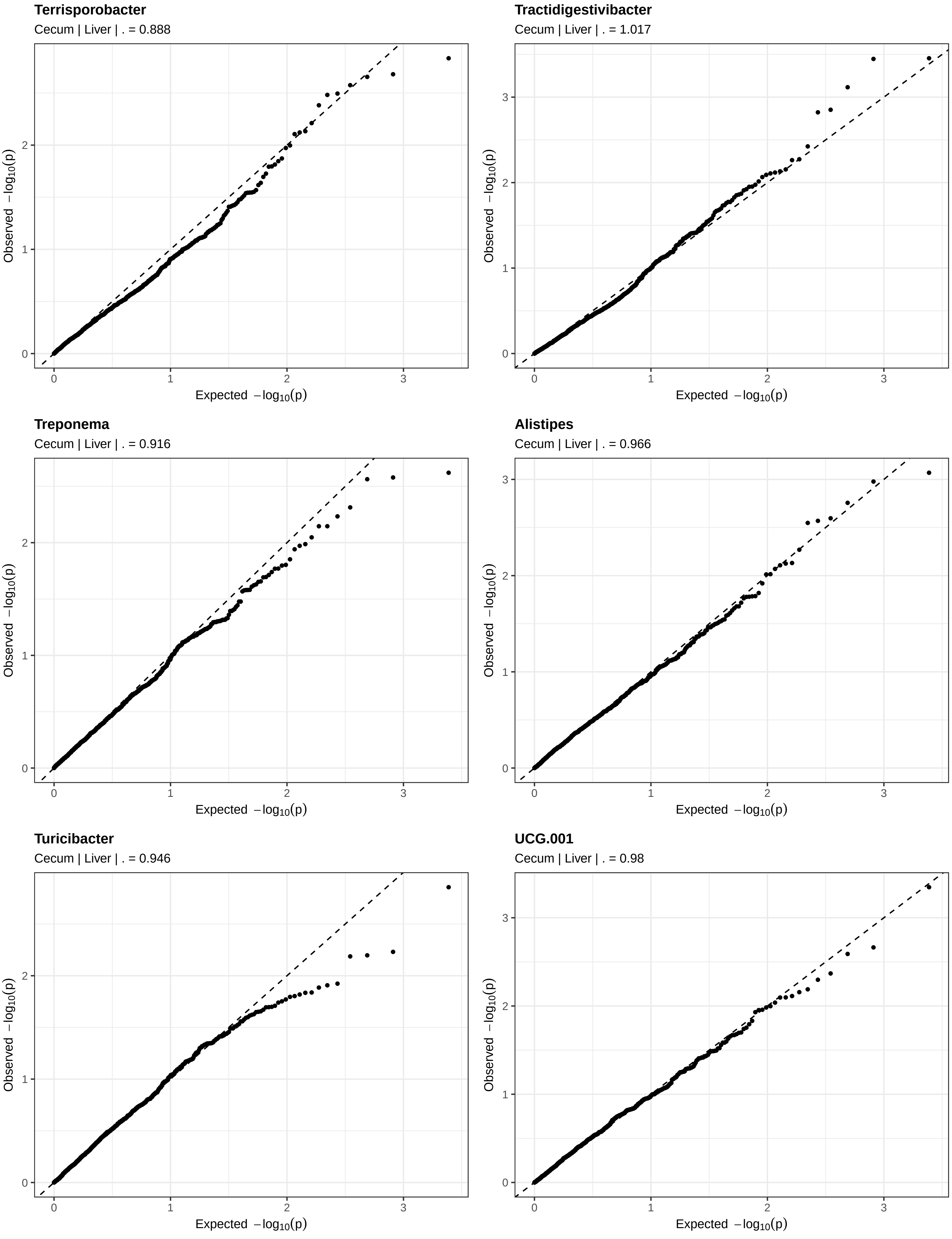
Terrisporobacter
Cecum | Liver | . = 0.888
Tractidigestivibacter
Cecum | Liver | . = 1.017
Treponema
Cecum | Liver | . = 0.916
Alistipes
Cecum | Liver | . = 0.966
Turicibacter
Cecum | Liver | . = 0.946
UCG.001
Cecum | Liver | . = 0.98
Observed $-\log_{10}(p)$
Expected $-\log_{10}(p)$
0
1
2
3

UCG.002

Cecum | Liver | . = 1.069

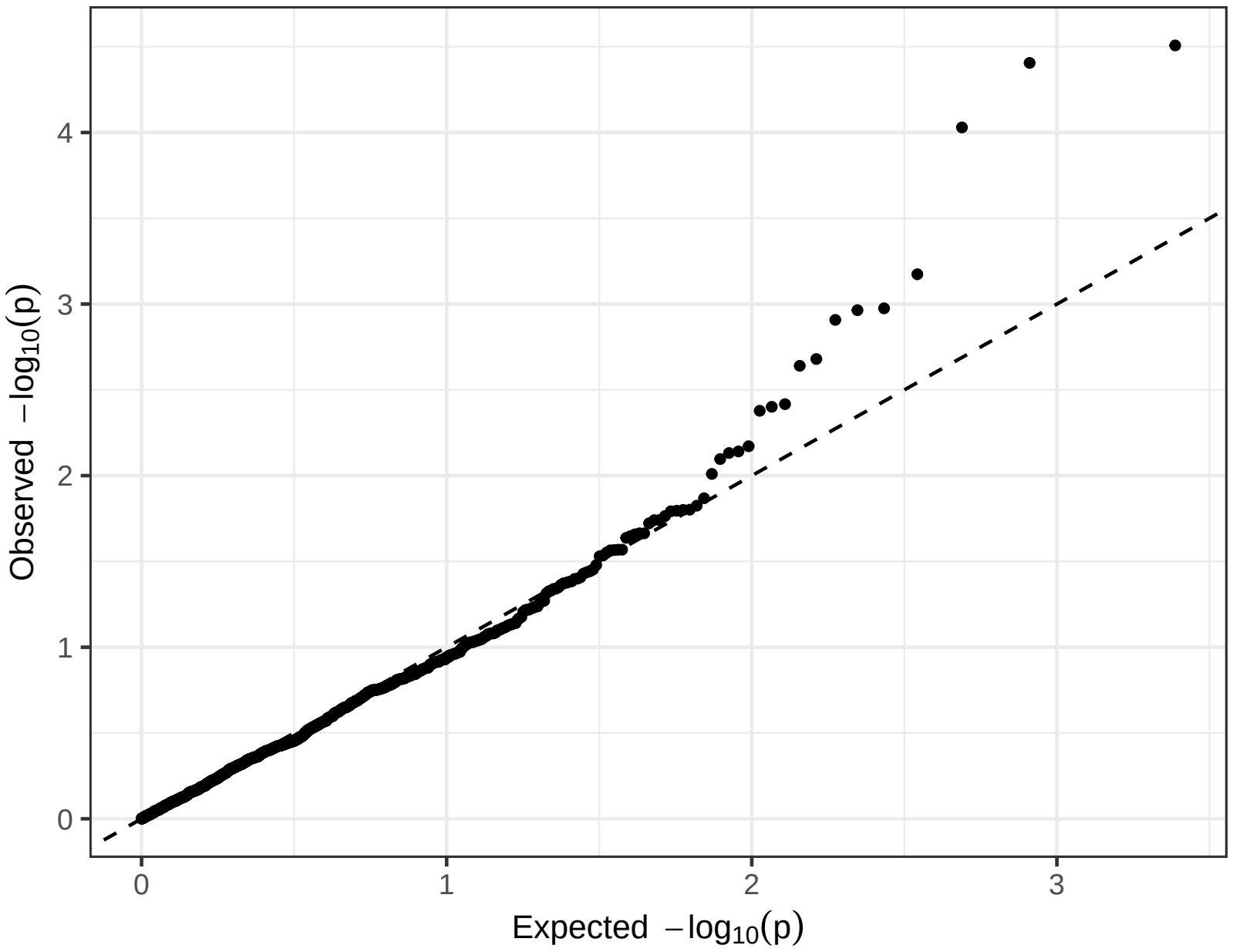

UCG.004

Cecum | Liver | . = 0.994

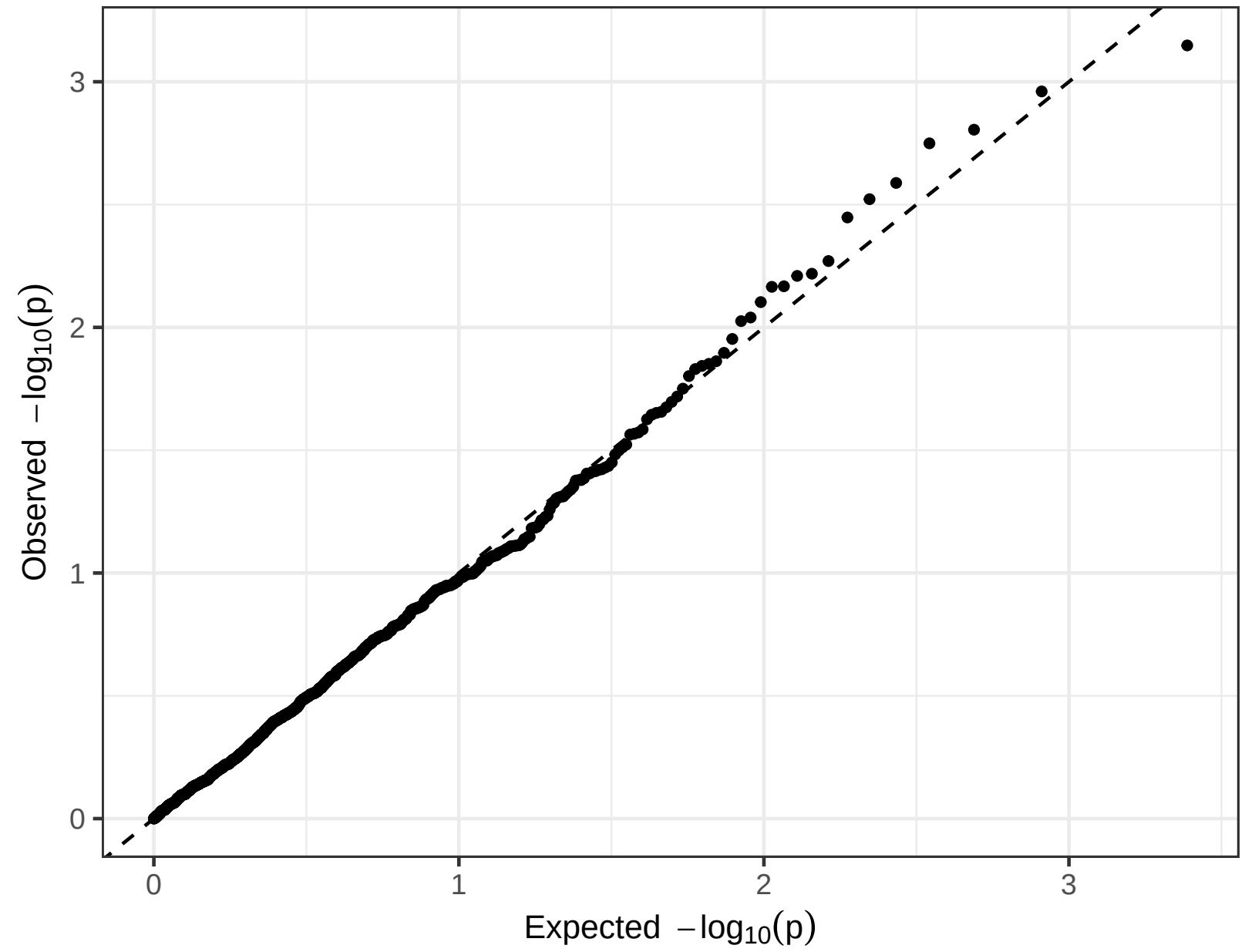

UCG.005

Cecum | Liver | . = 0.968

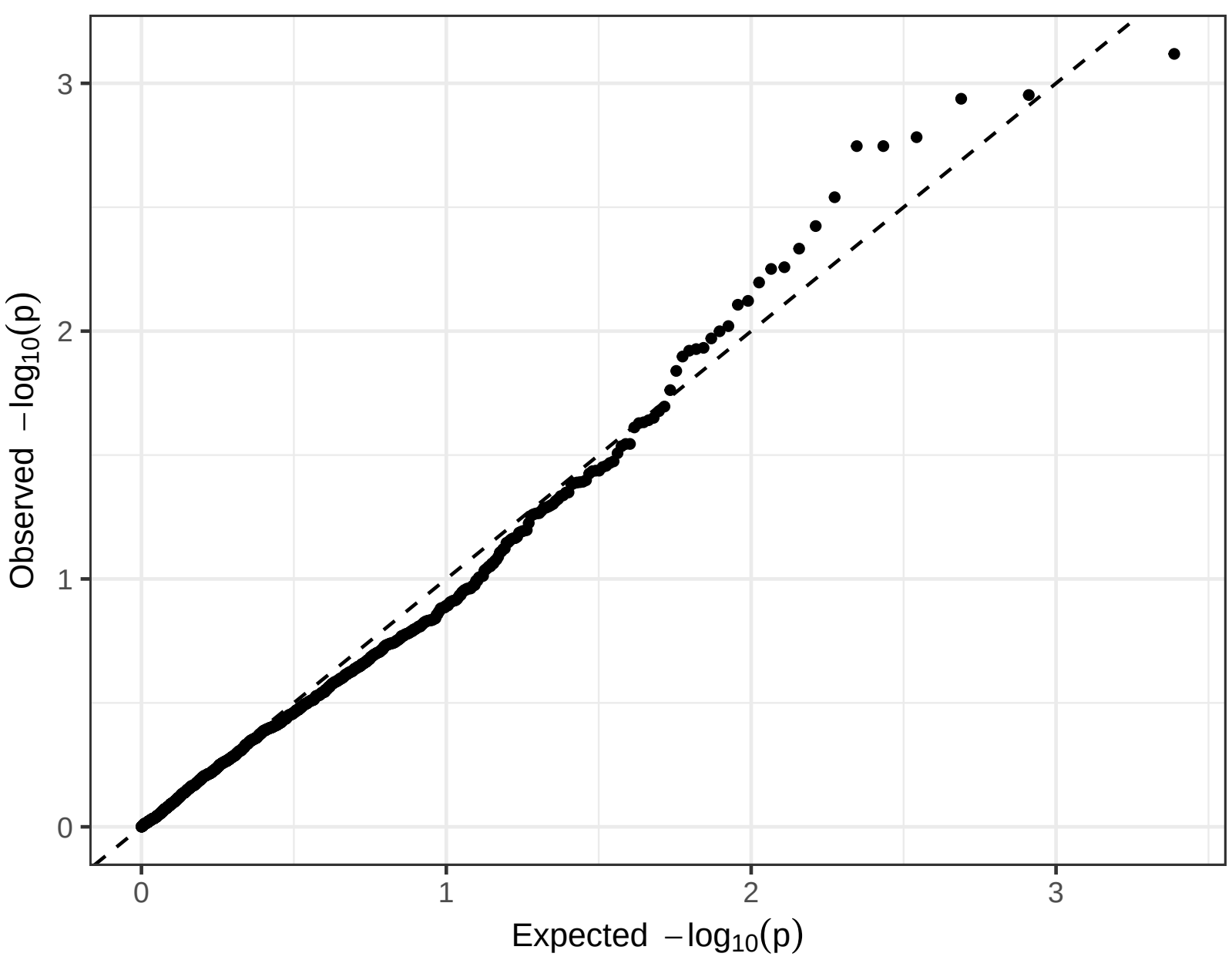

UCG.008

Cecum | Liver | . = 0.89

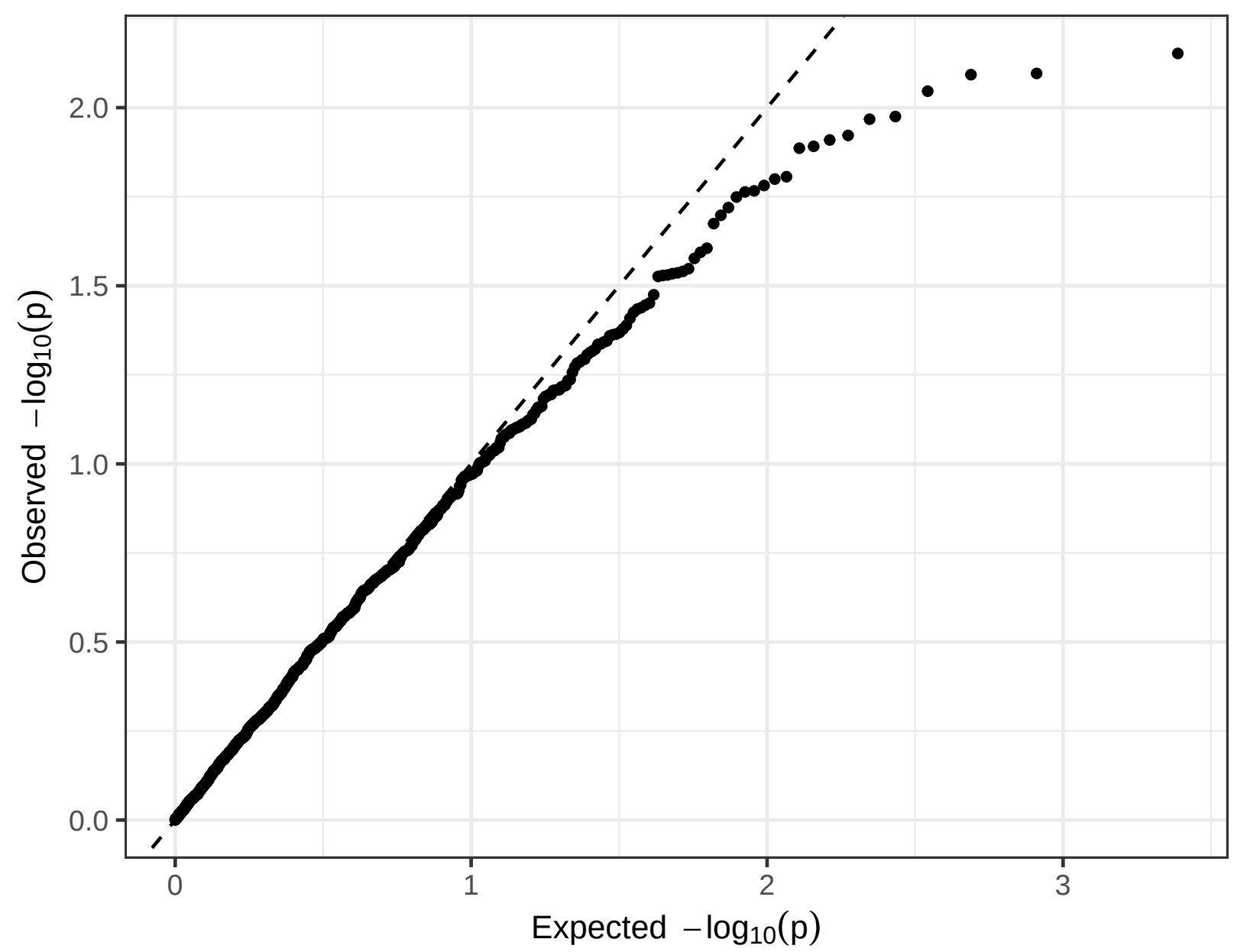

UCG.009

Cecum | Liver | . = 0.974

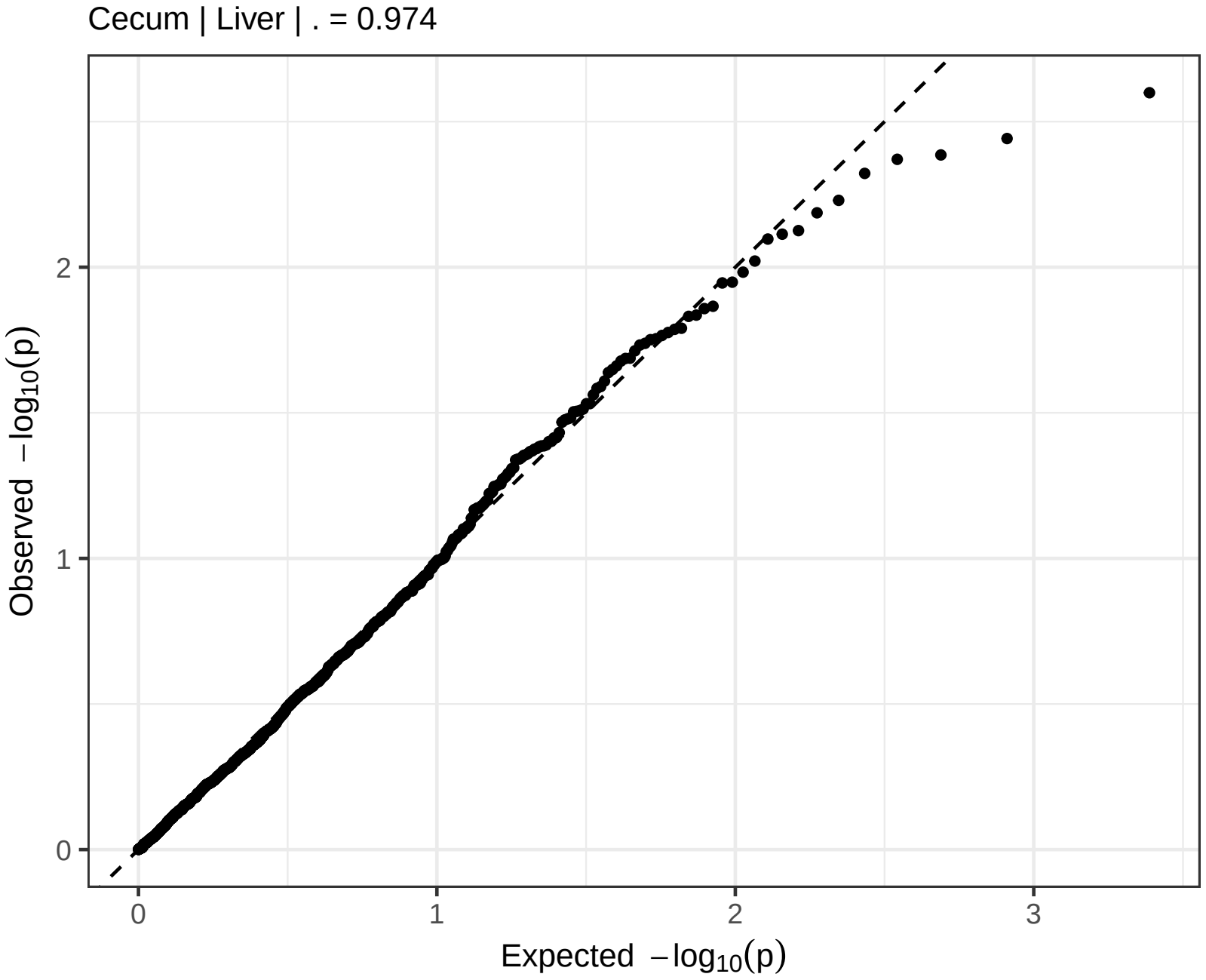

Xylanibacter

Cecum | Liver | . = 0.933

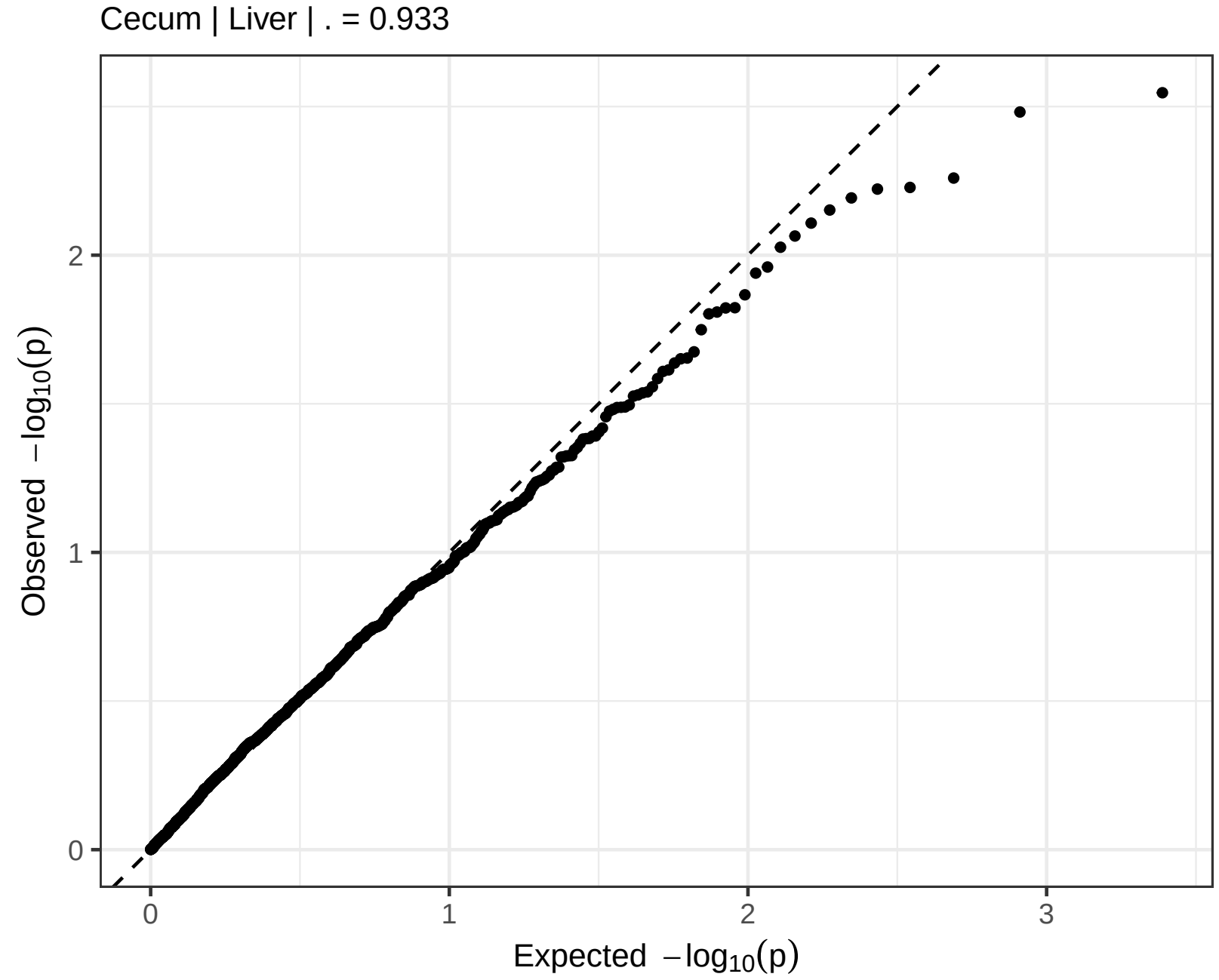

Z20

Cecum | Liver | . = 1.133

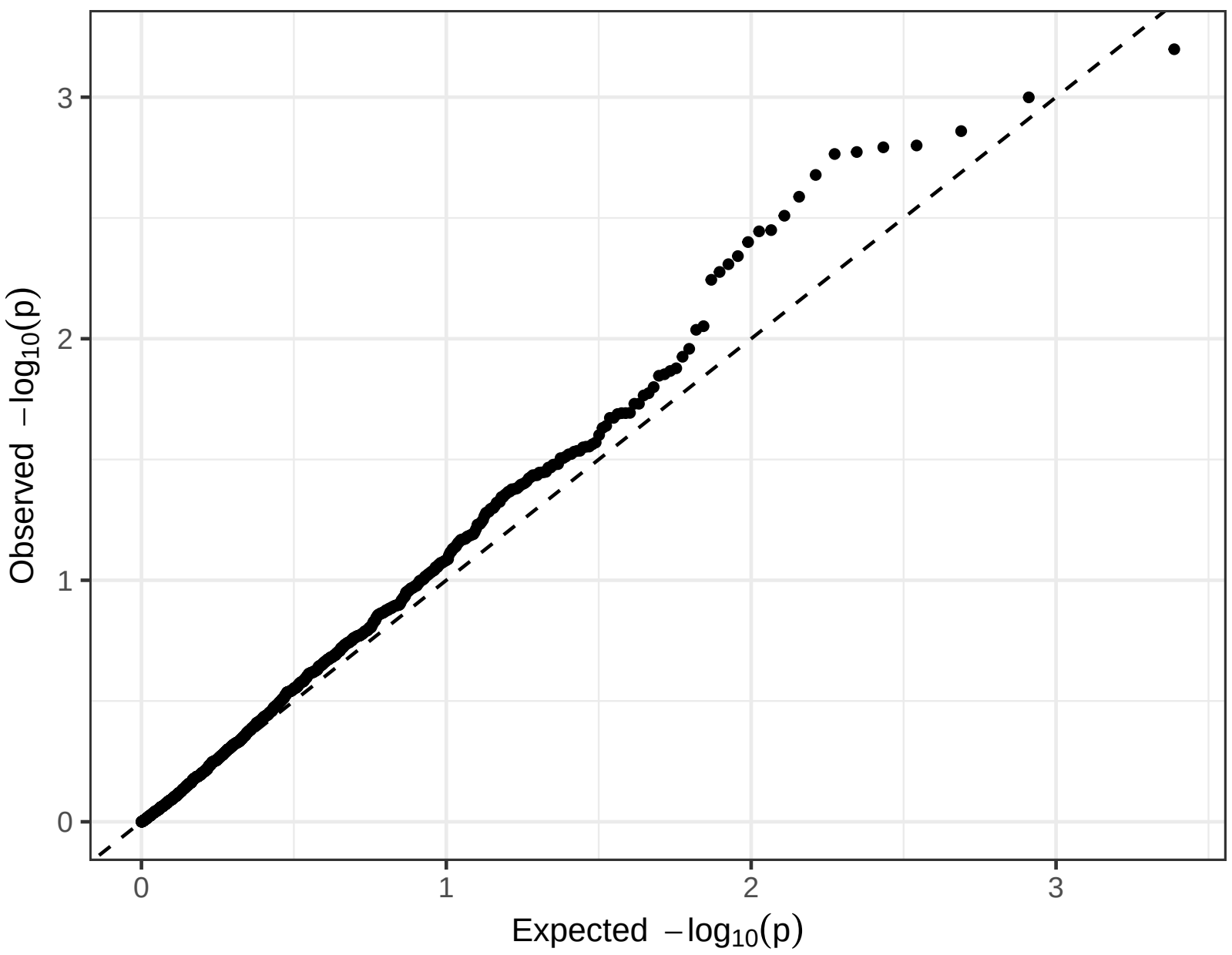

Zag_111

Cecum | Liver | . = 0.979

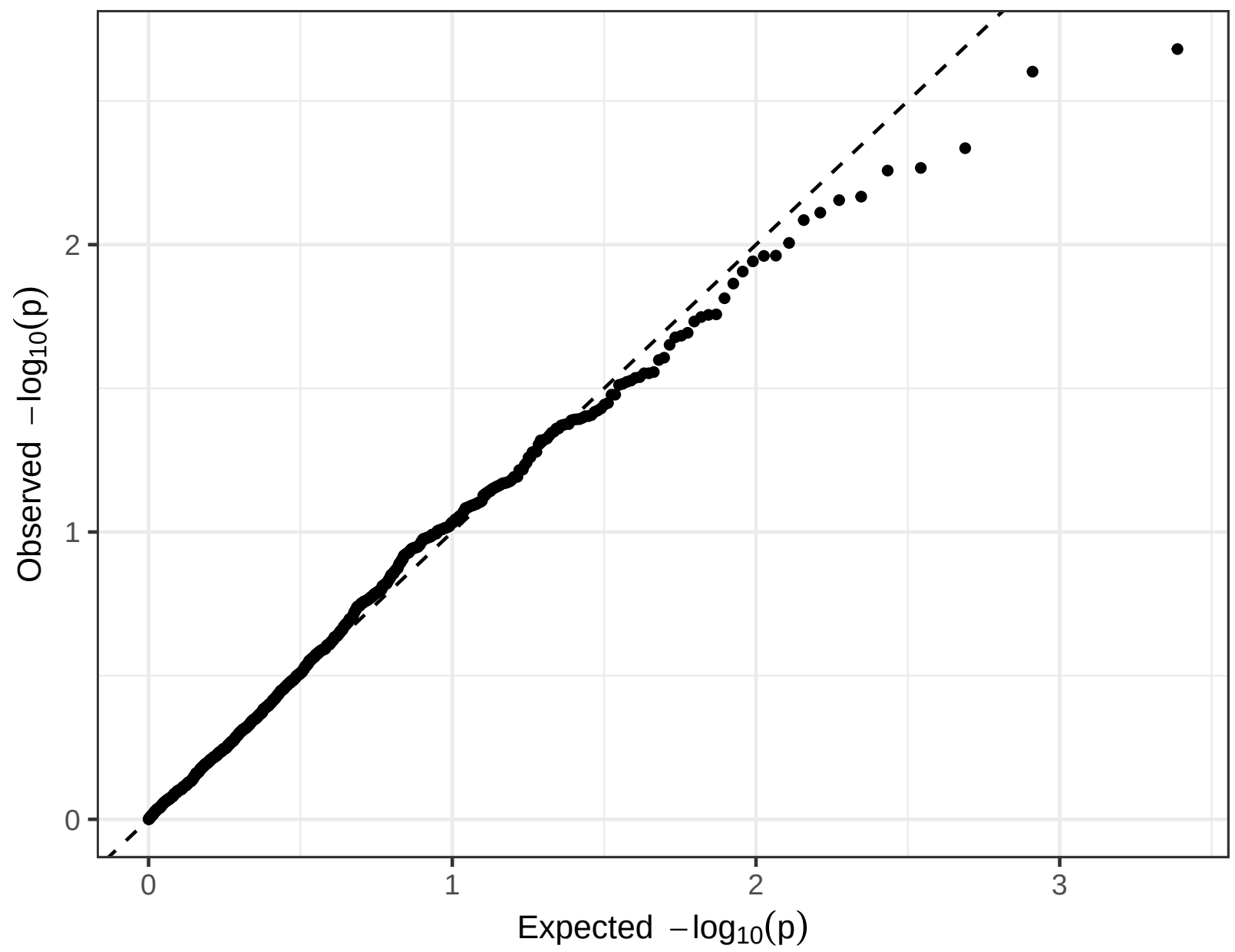

Alloprevotella

Cecum | Liver | . = 0.991

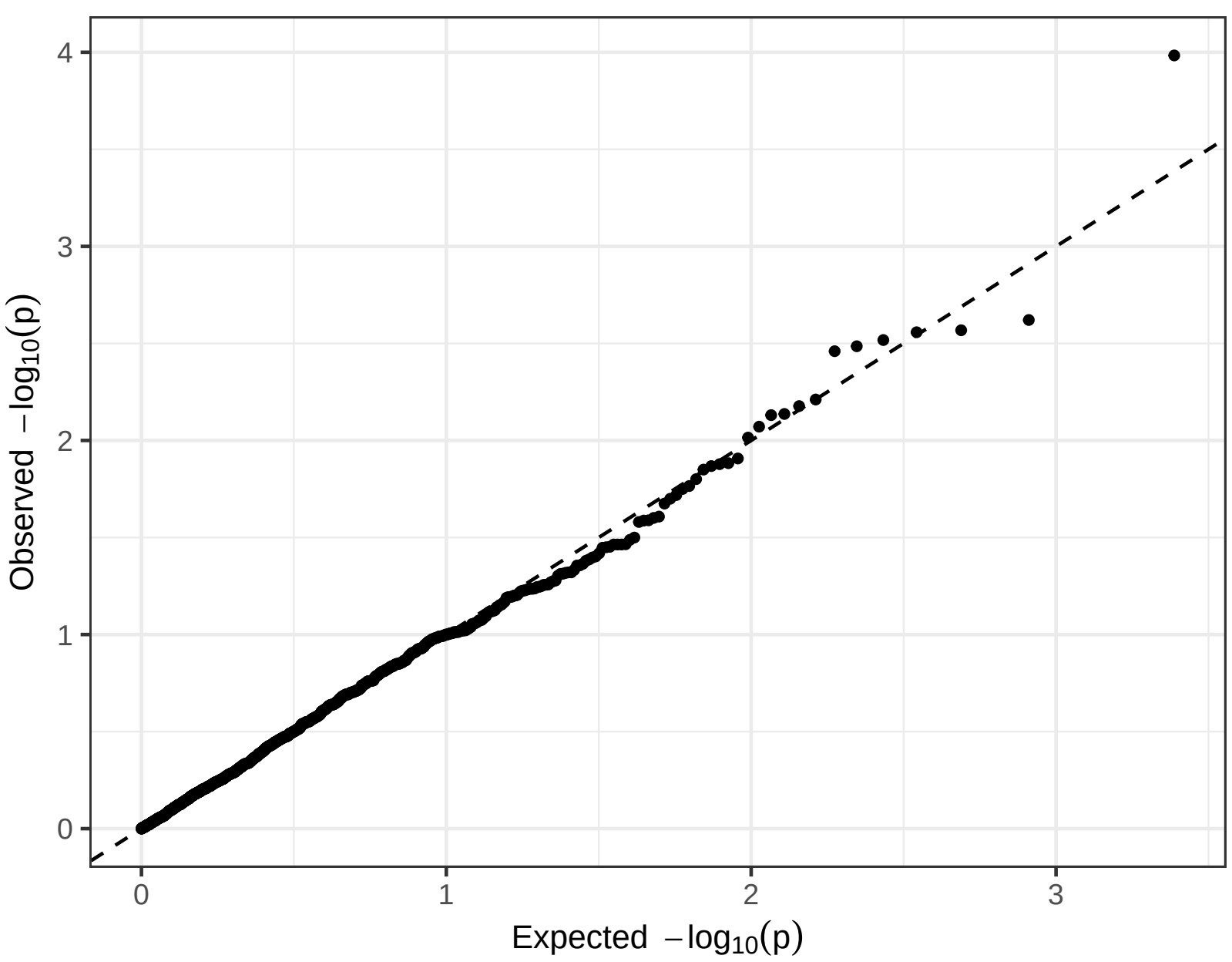

Aminipila

Cecum | Liver | . = 0.859

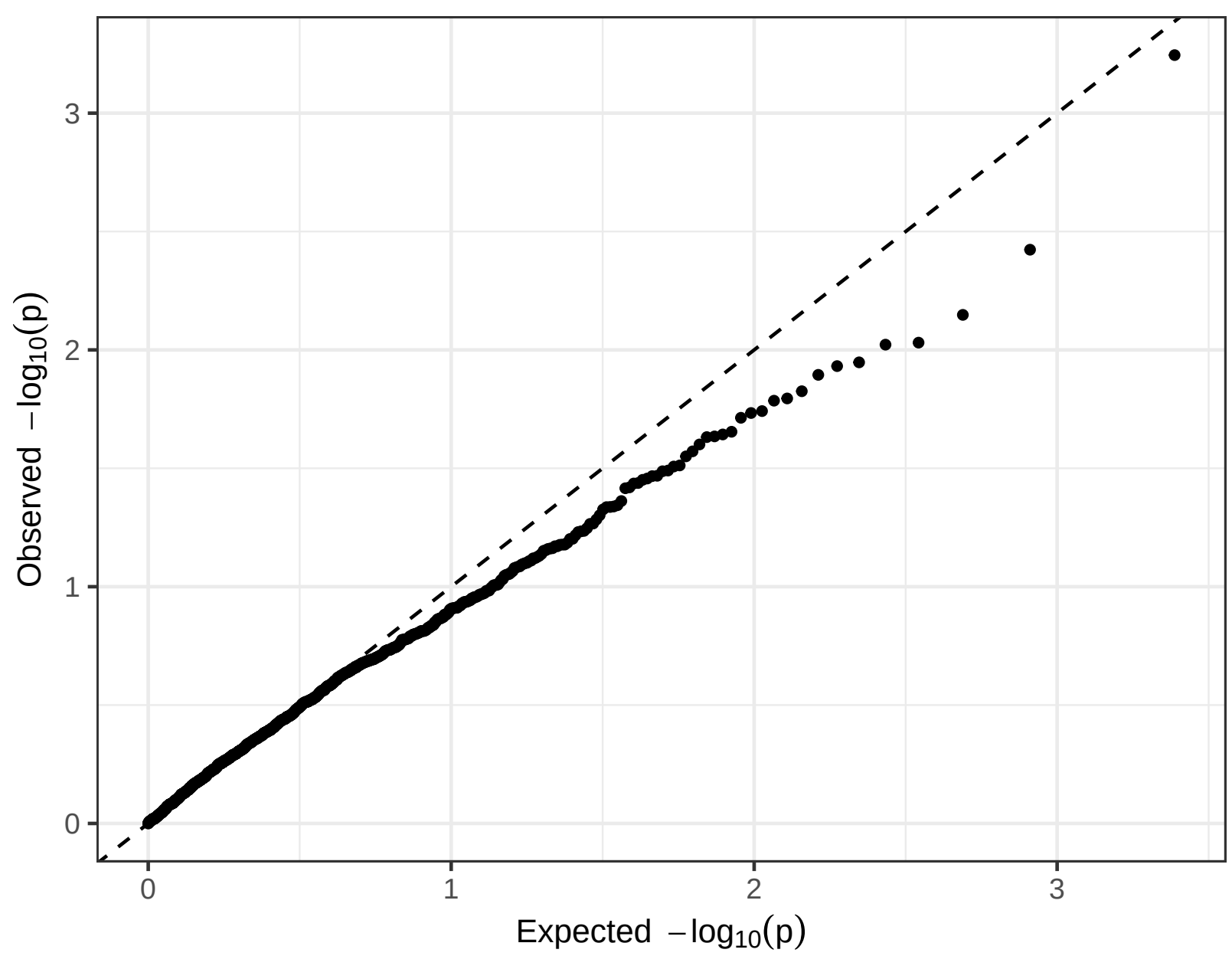

Anaerobiospirillum

Cecum | Liver | . = 0.996

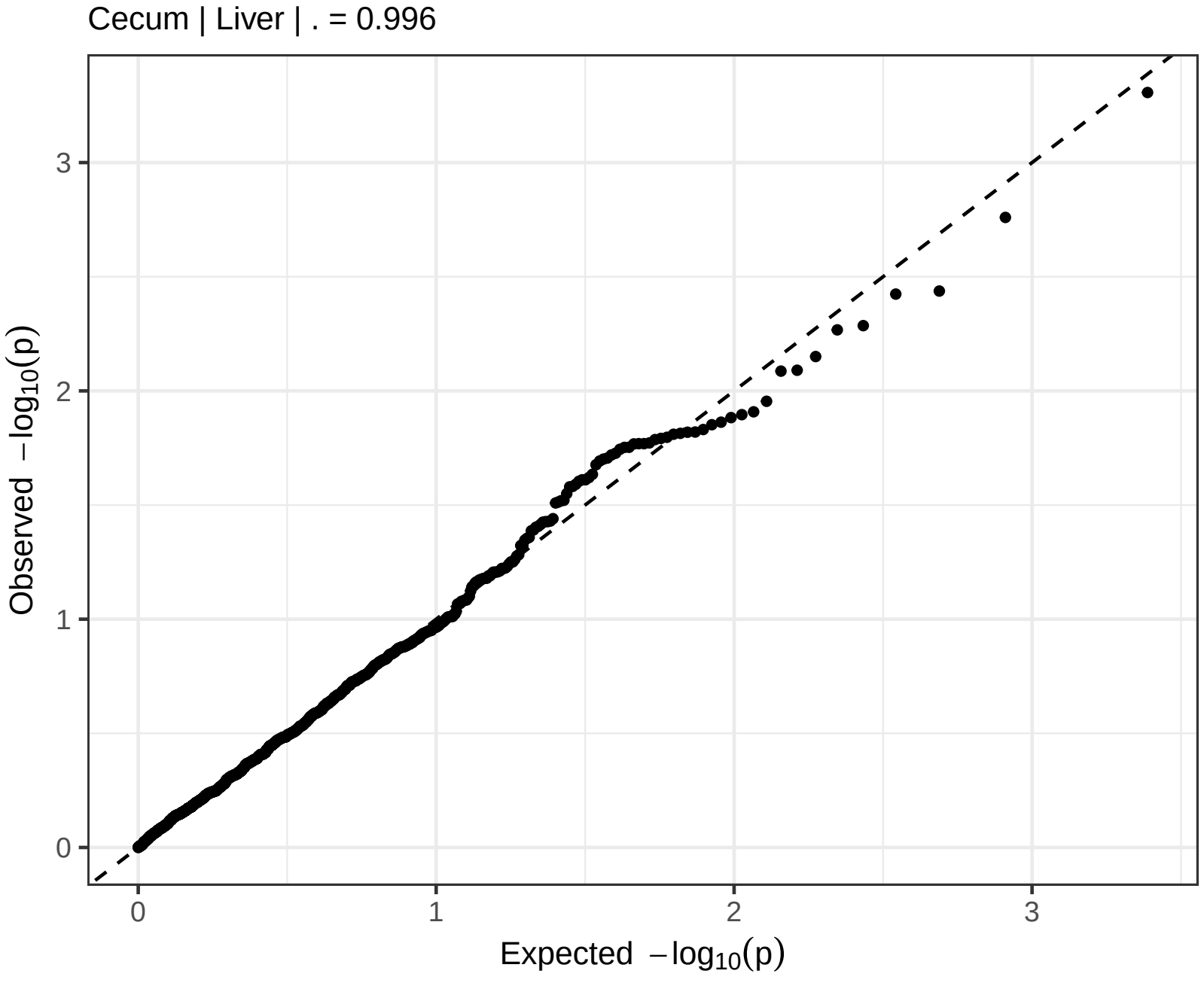

Anaerobutyricum

Cecum | Liver | . = 0.836

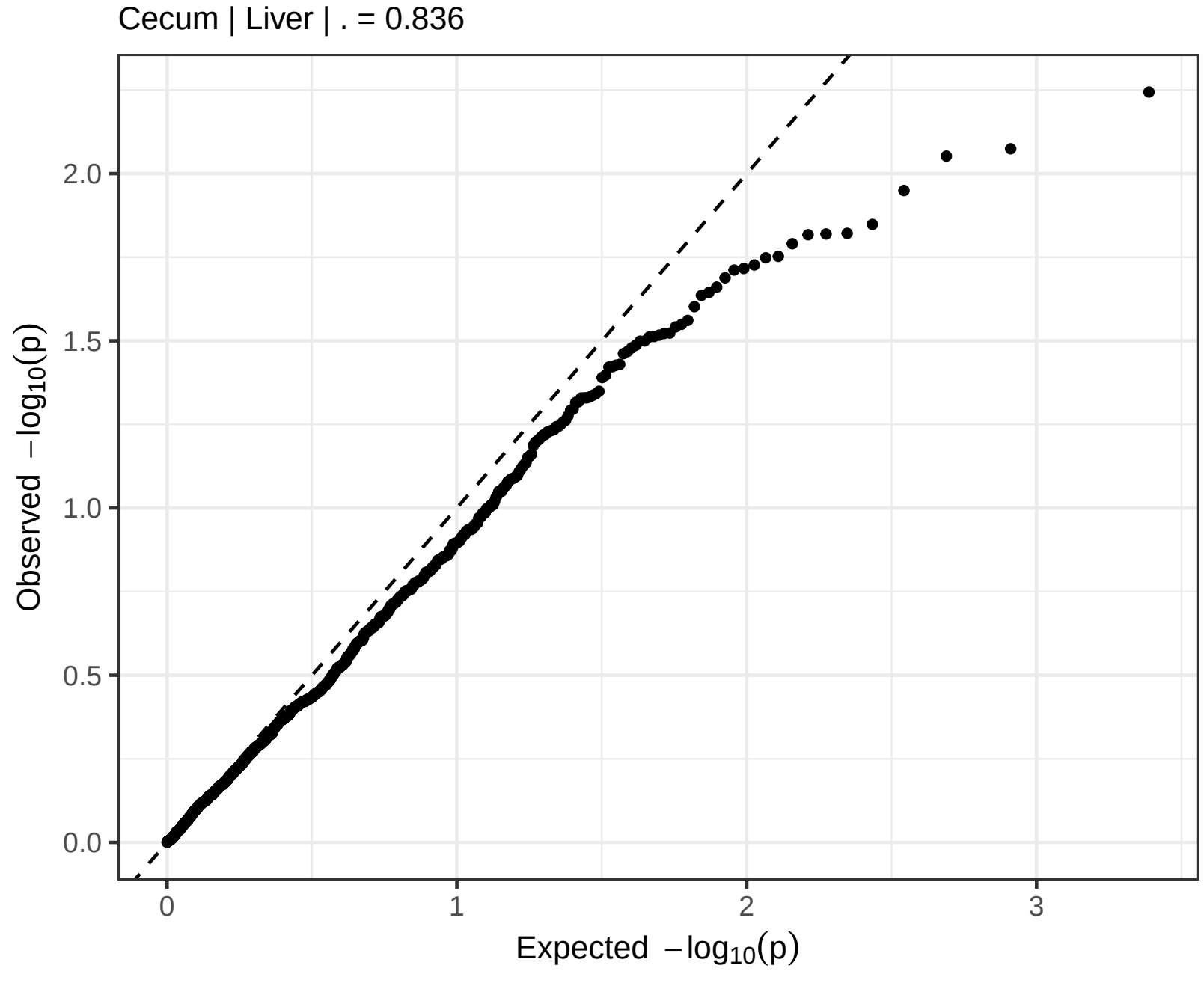

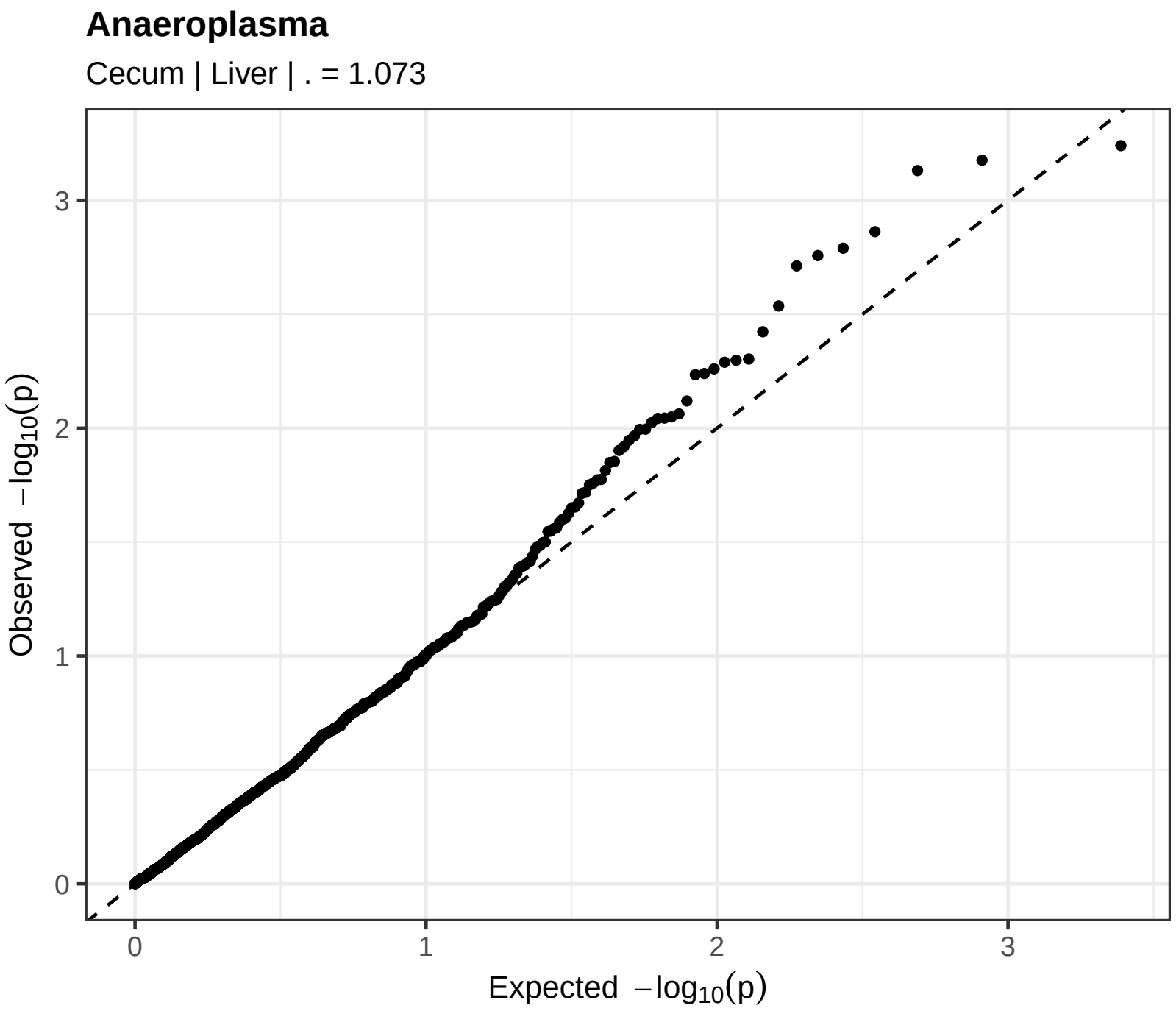
Anaeroplasma
Cecum | Liver | . = 1.073
Observed −log10(p)
Expected −log10(p)

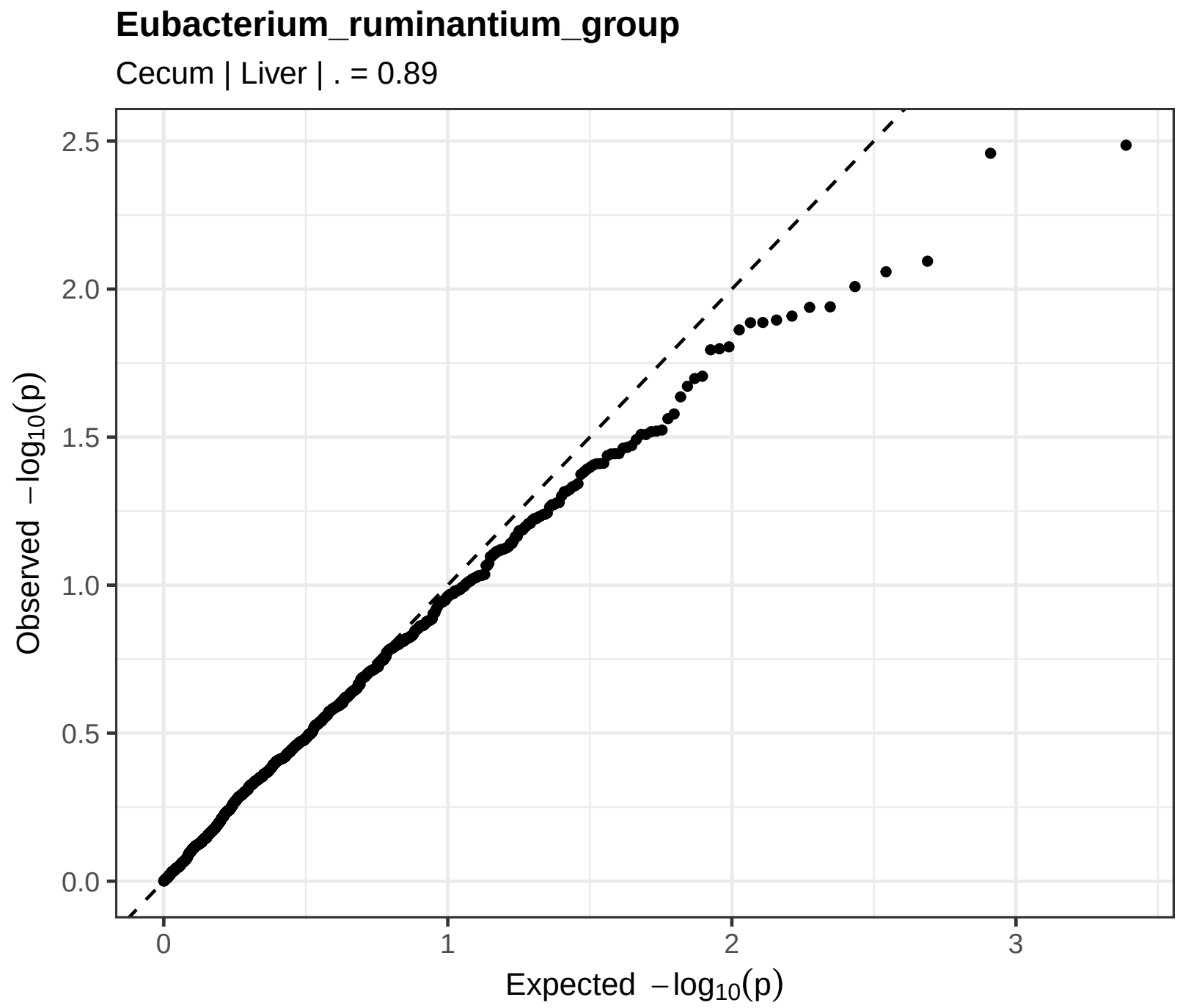
Eubacterium_ruminantium_group
Cecum | Liver | . = 0.89
Observed −log10(p)
Expected −log10(p)

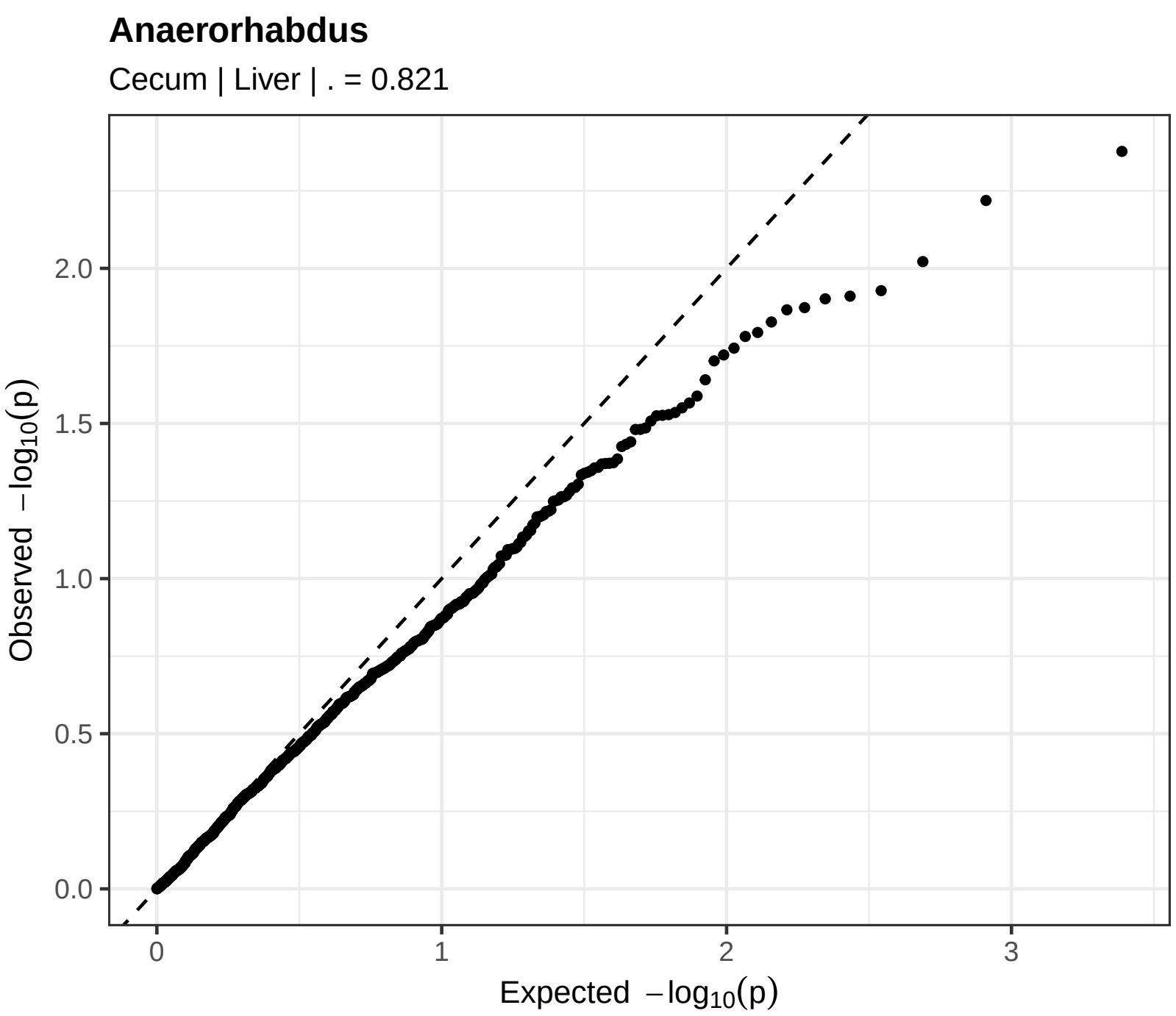
Anaerorhabdus
Cecum | Liver | . = 0.821
Observed −log10(p)
Expected −log10(p)

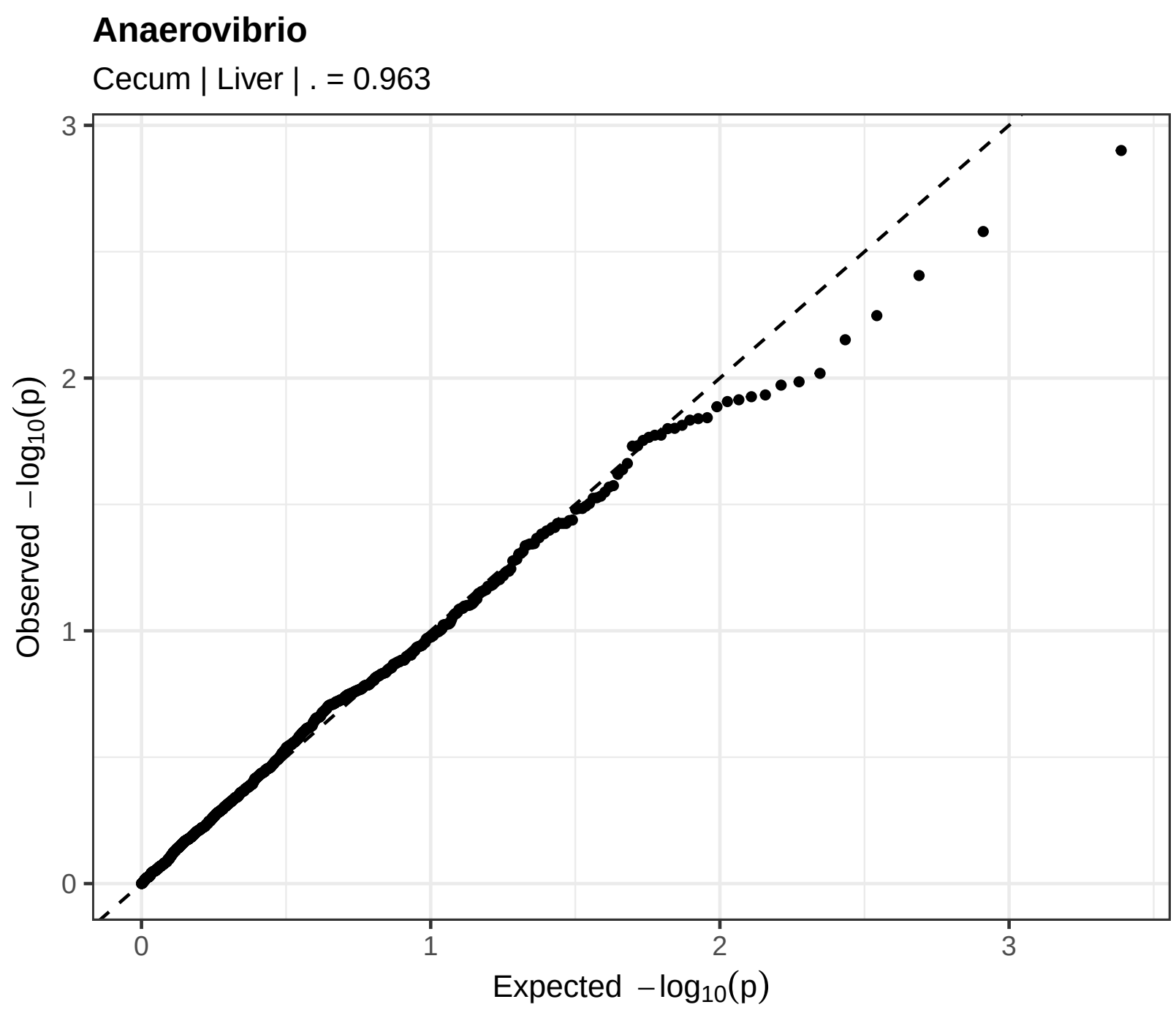
Anaerovibrio
Cecum | Liver | . = 0.963
Observed −log10(p)
Expected −log10(p)

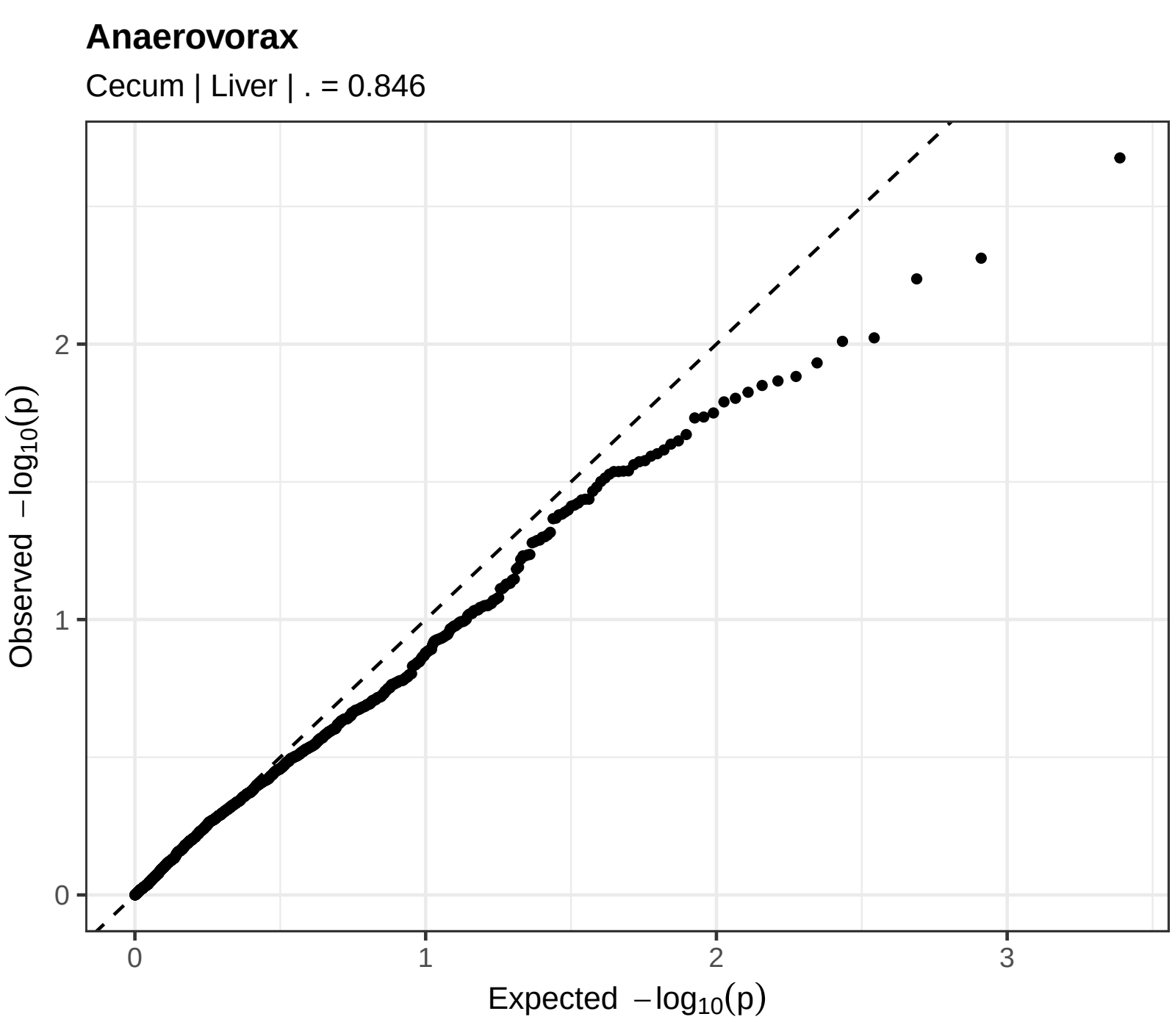
Anaerovorax
Cecum | Liver | . = 0.846
Observed −log10(p)
Expected −log10(p)

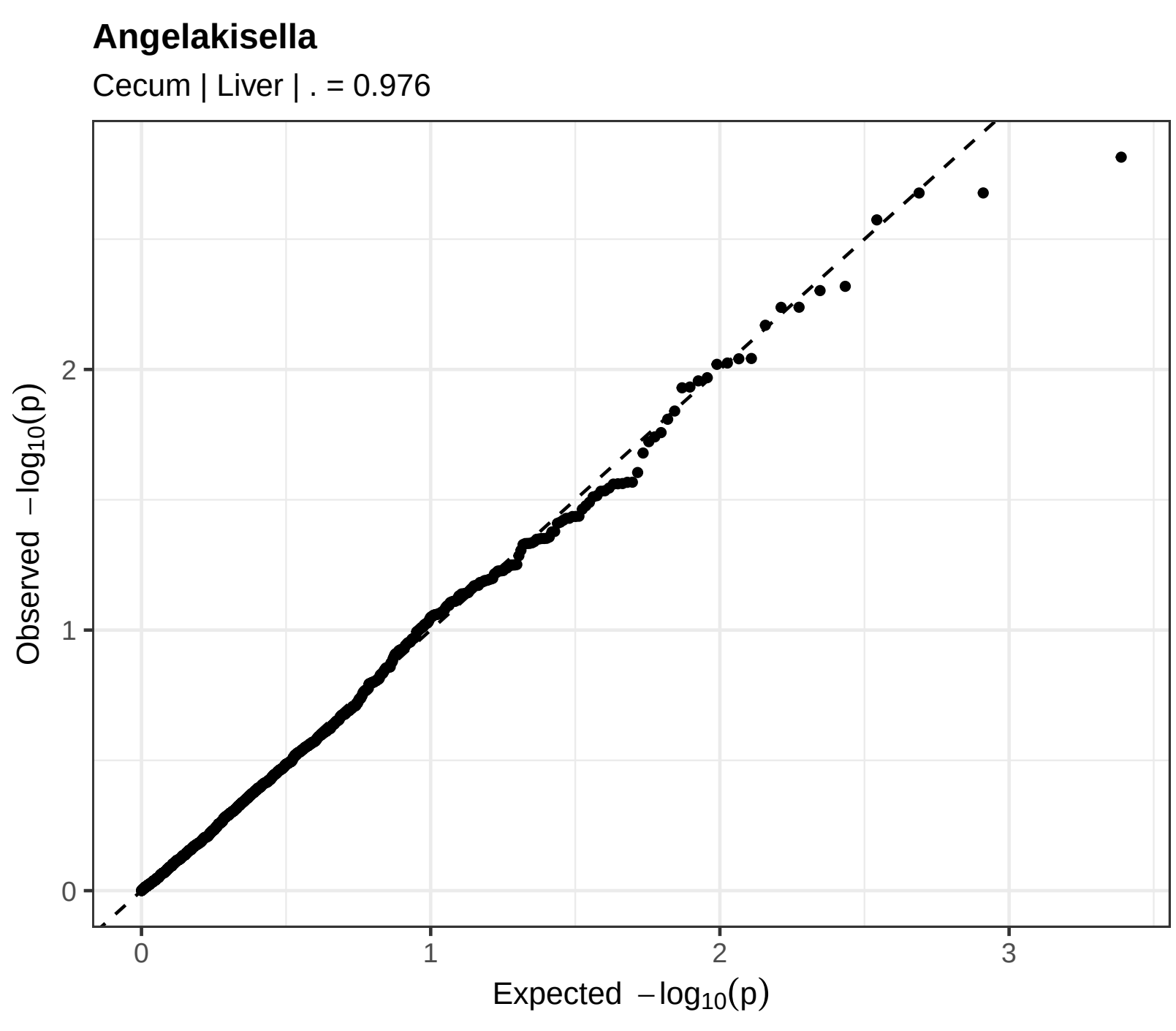
Angelakisella
Cecum | Liver | . = 0.976
Observed −log10(p)
Expected −log10(p)

Bacteroides

Cecum | Liver | . = 1.013

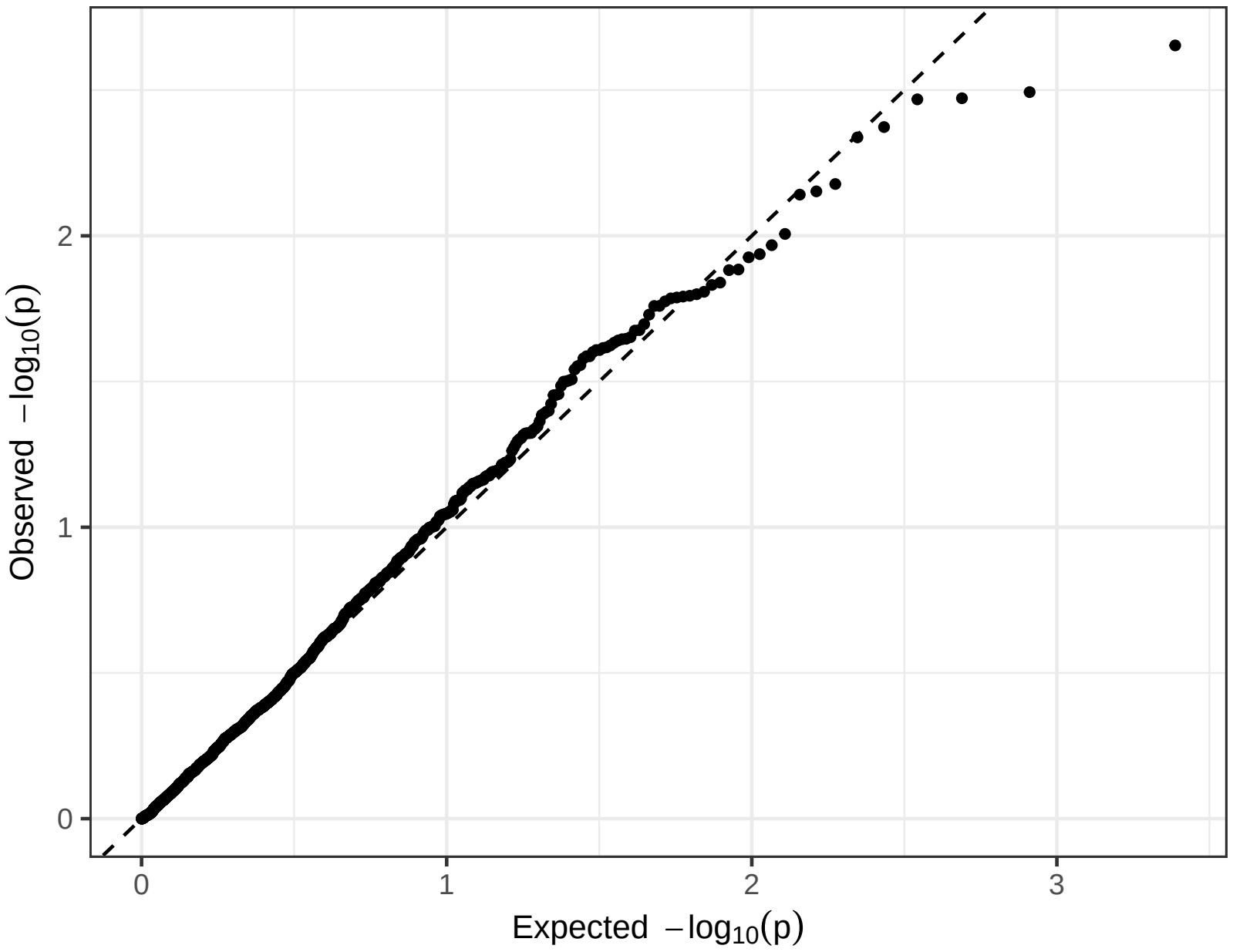

Blautia

Cecum | Liver | . = 0.889

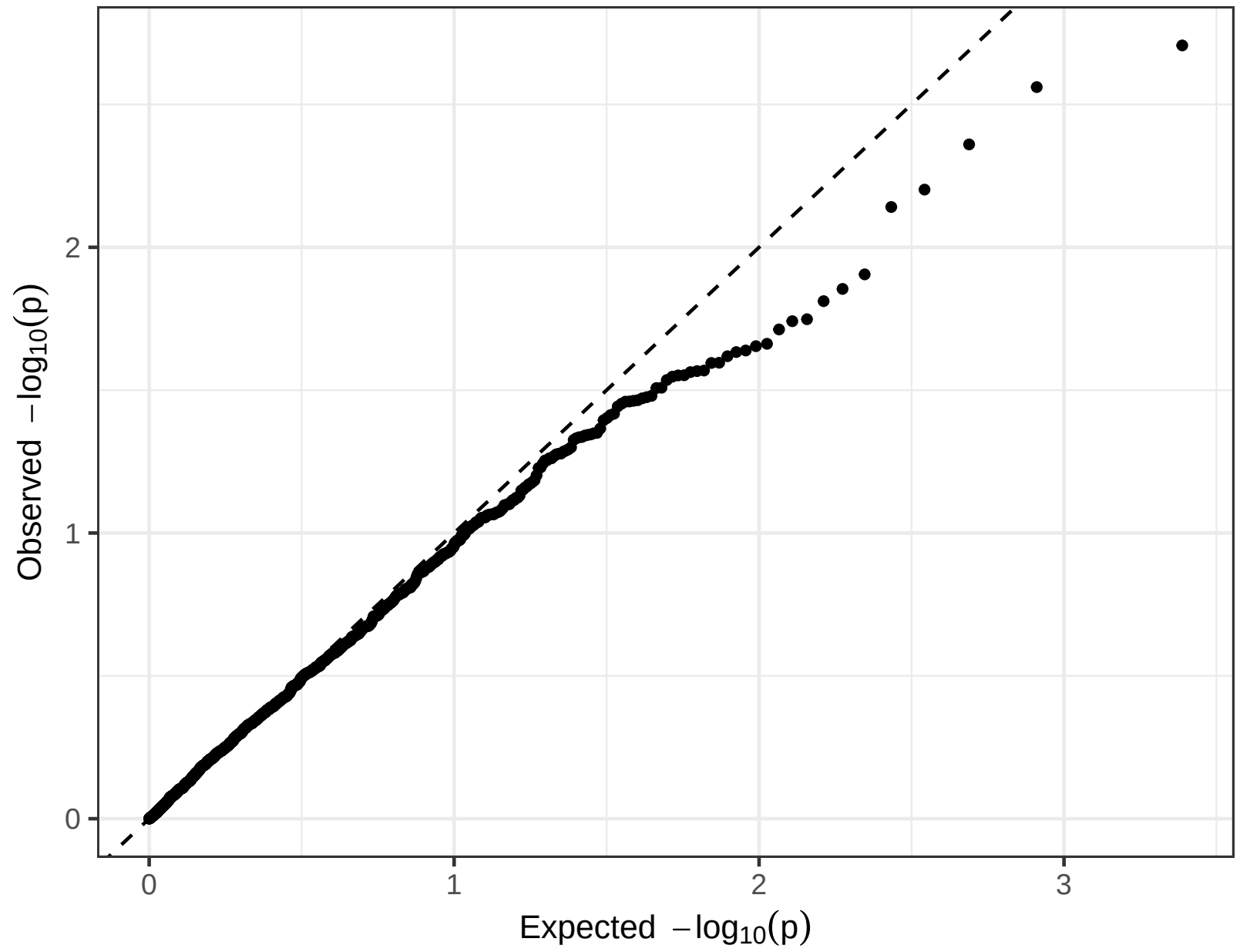

Breznakia

Cecum | Liver | . = 0.91

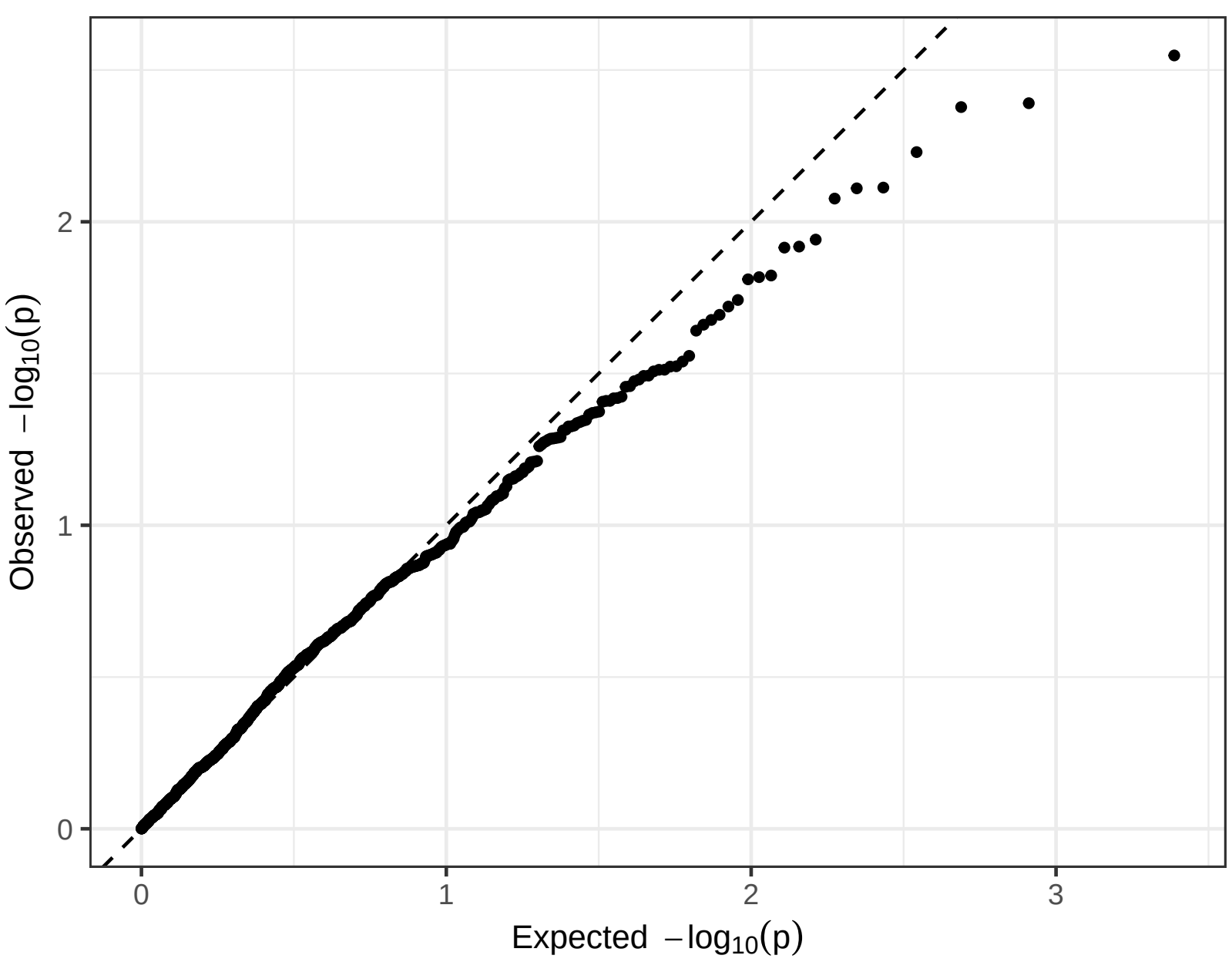

Butyribacter

Cecum | Liver | . = 0.841

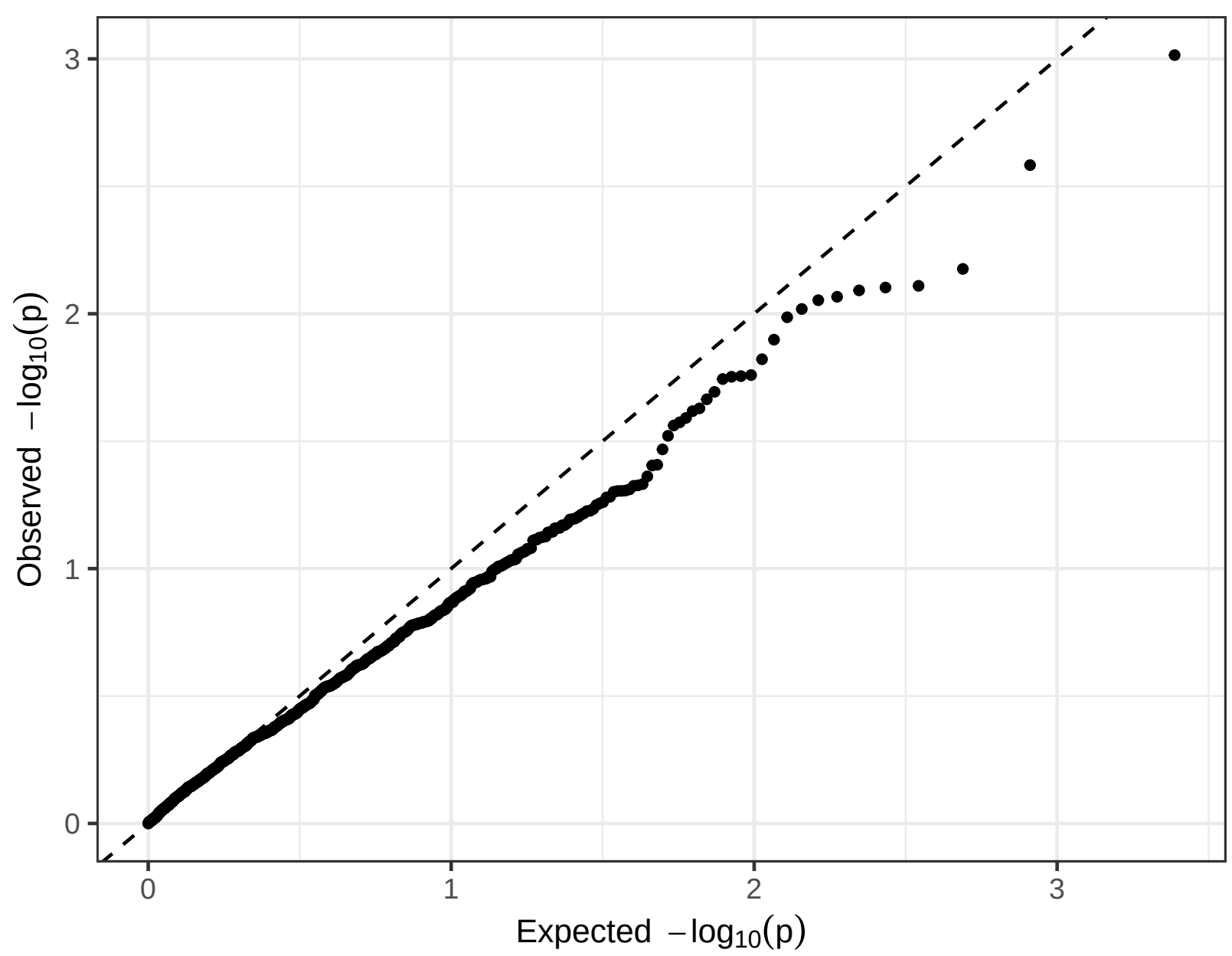

CAG.196

Cecum | Liver | . = 0.943

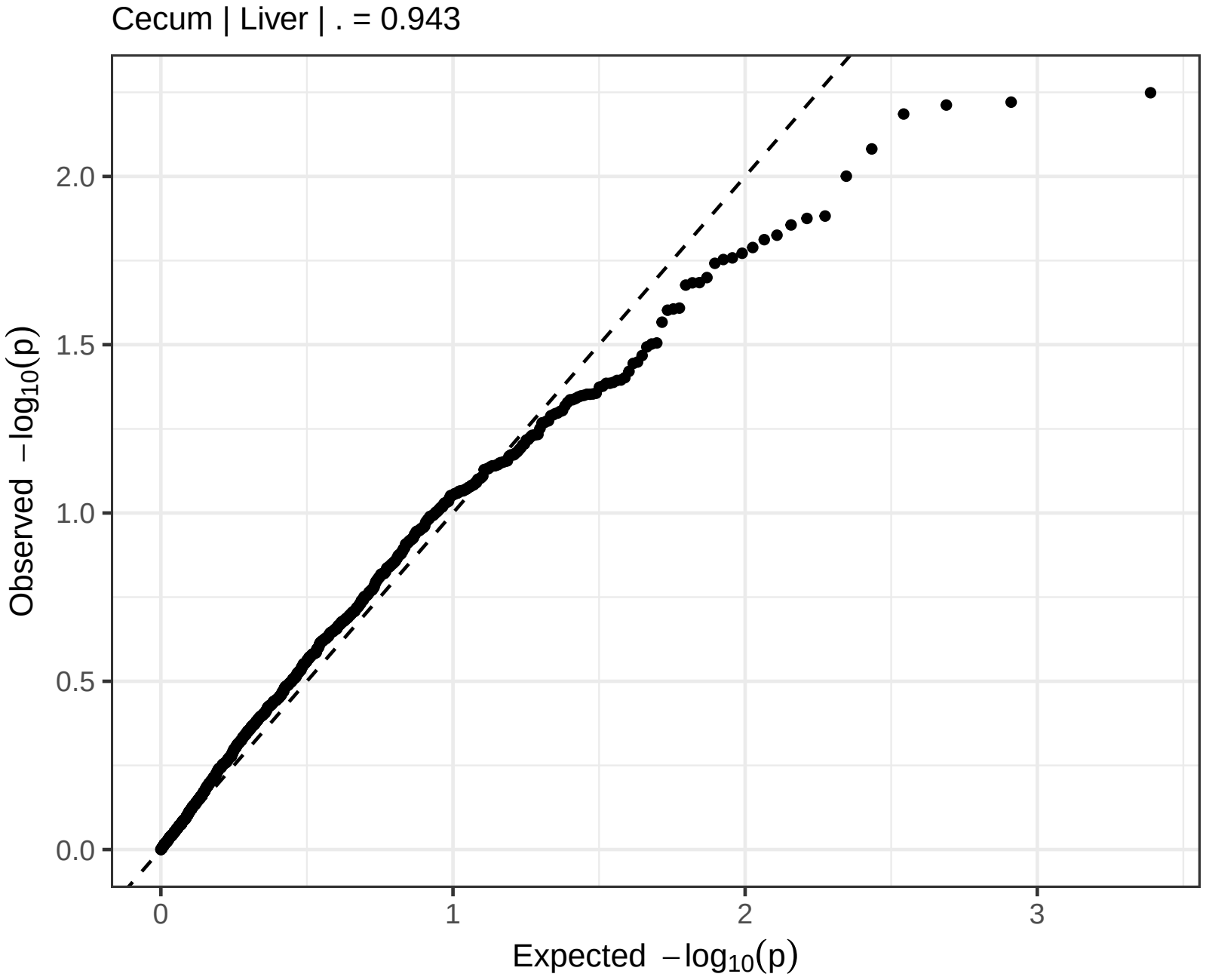

CAG.56

Cecum | Liver | . = 0.94

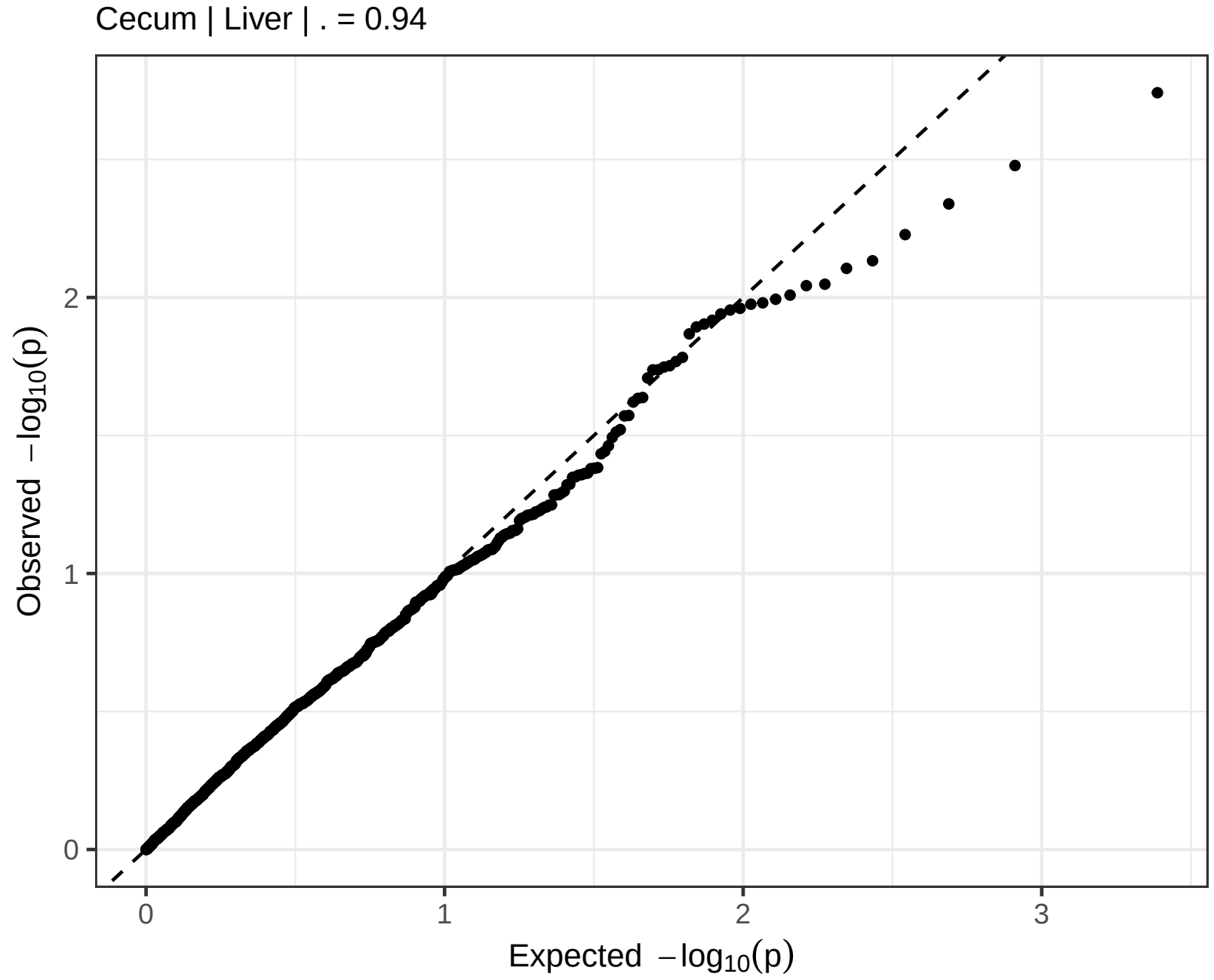

Eubacterium_siraeum_group

Cecum | Liver | . = 0.92

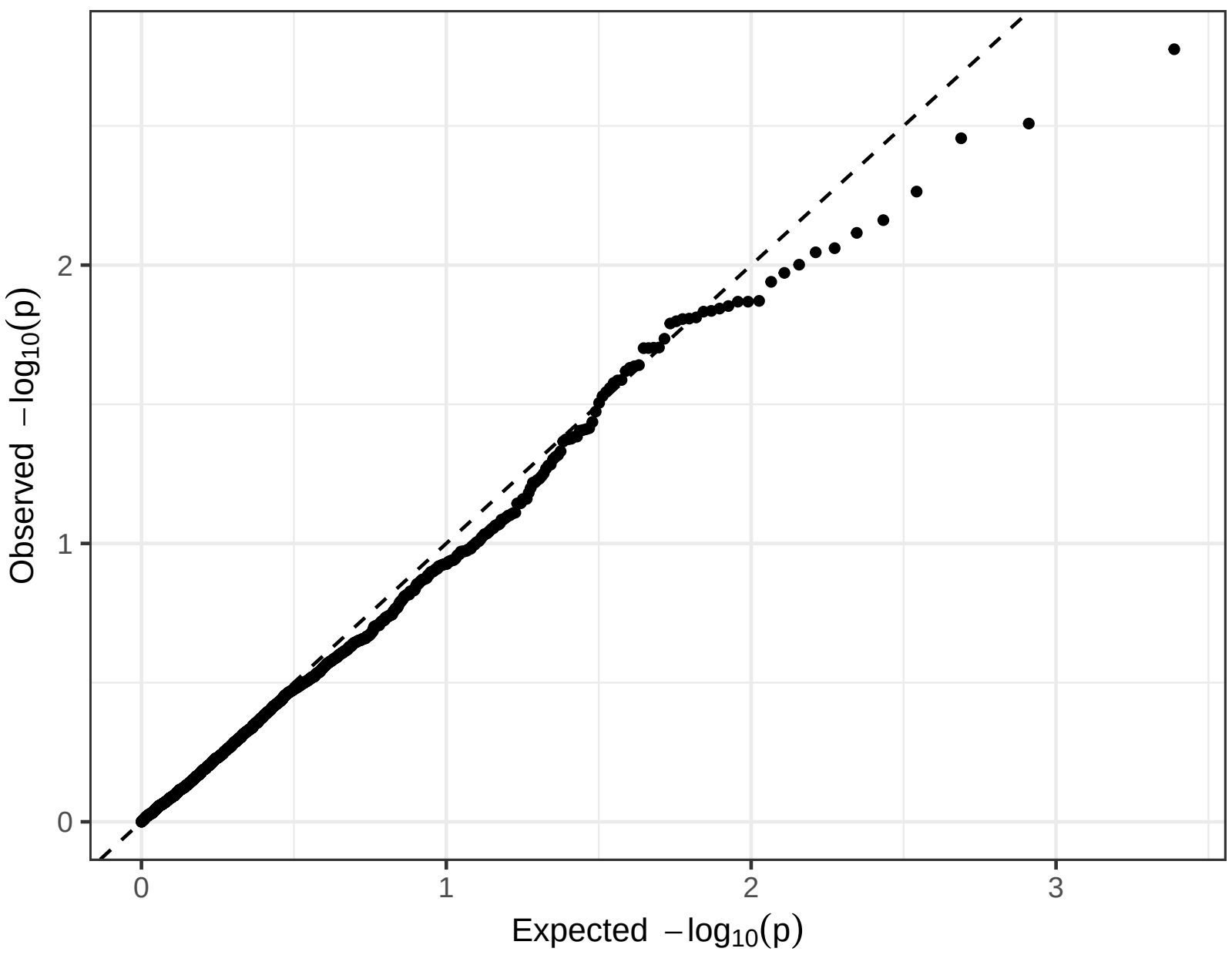

CAG.873

Cecum | Liver | . = 0.912

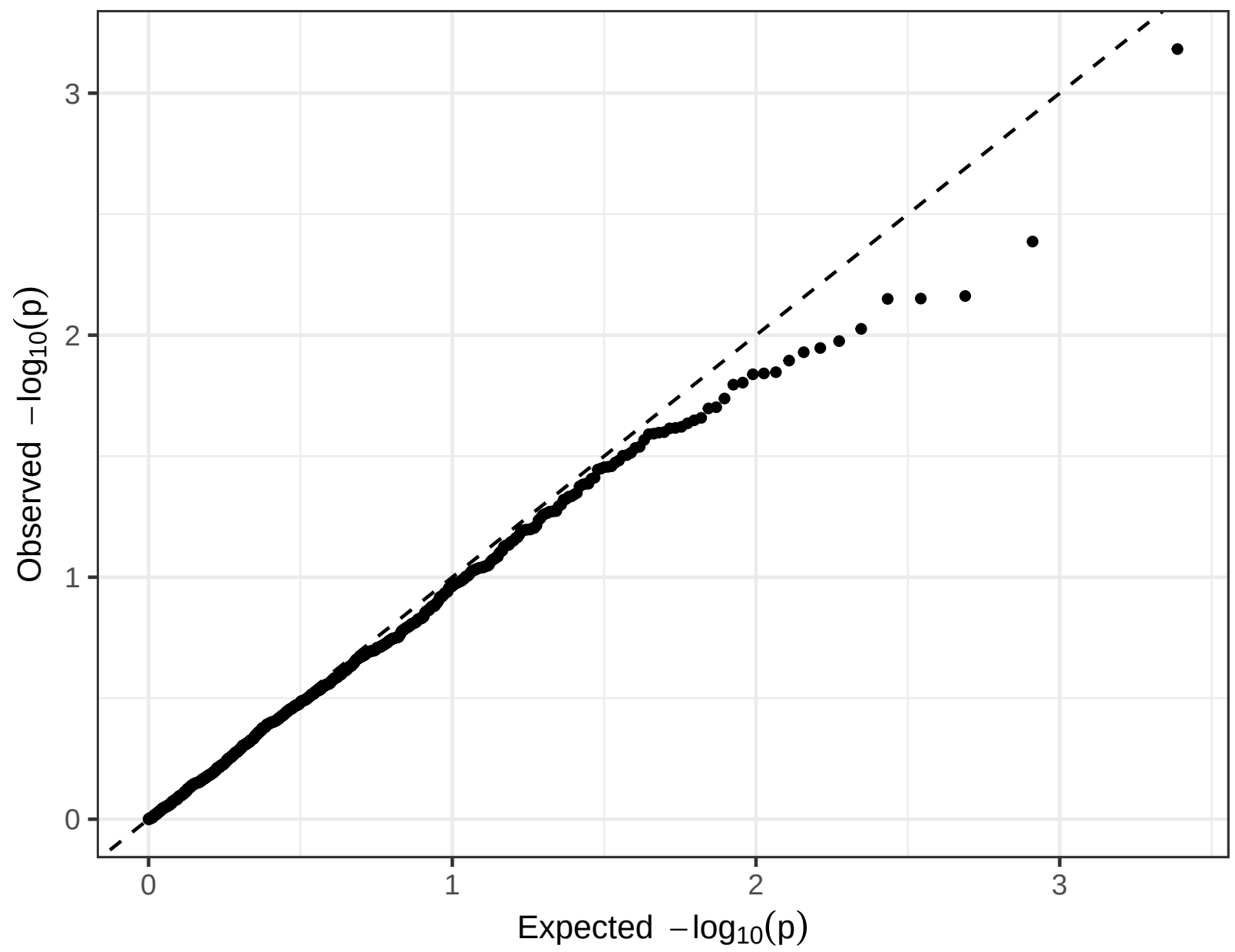

Caldicoprobacter

Cecum | Liver | . = 0.84

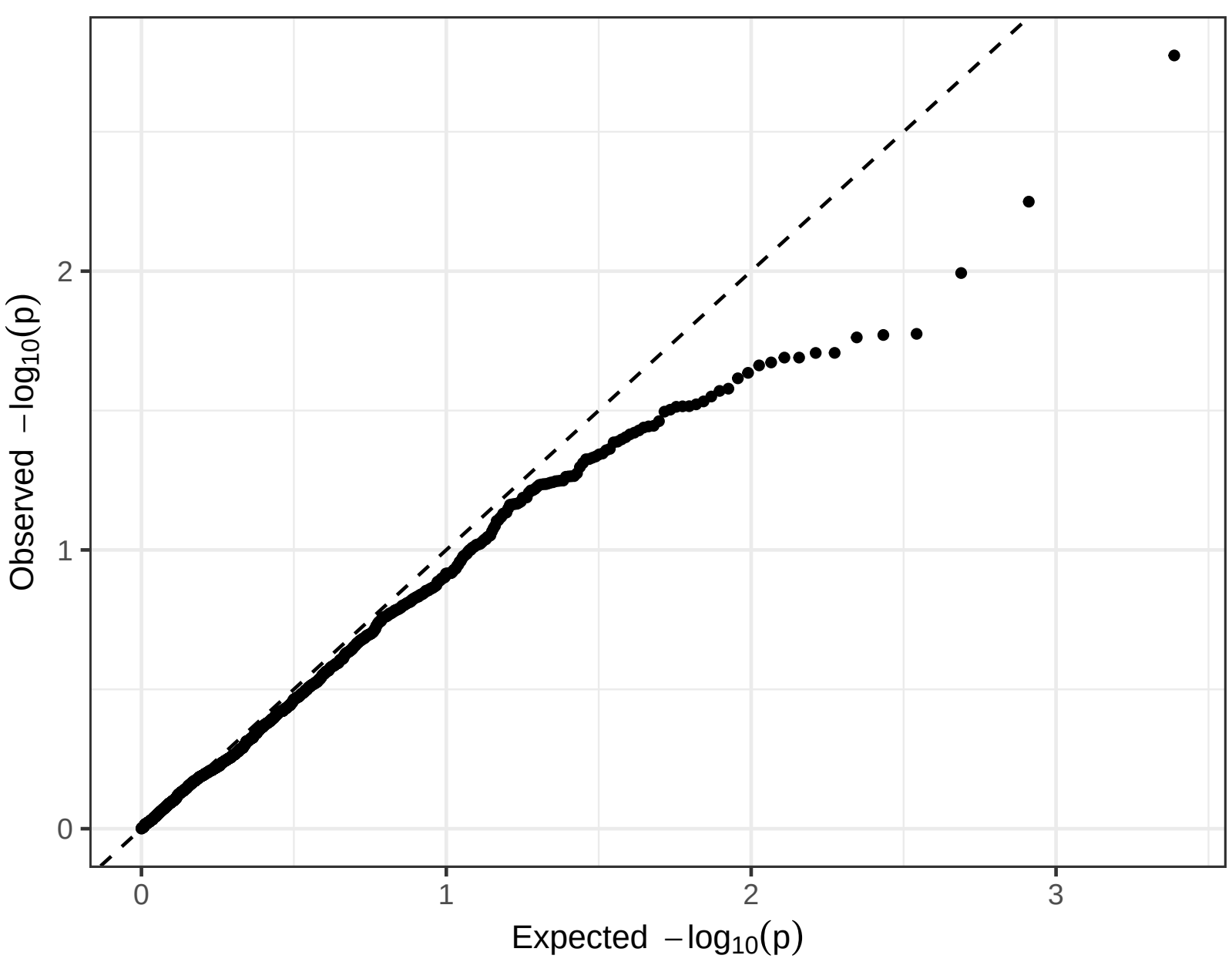

Campylobacter

Cecum | Liver | . = 1.02

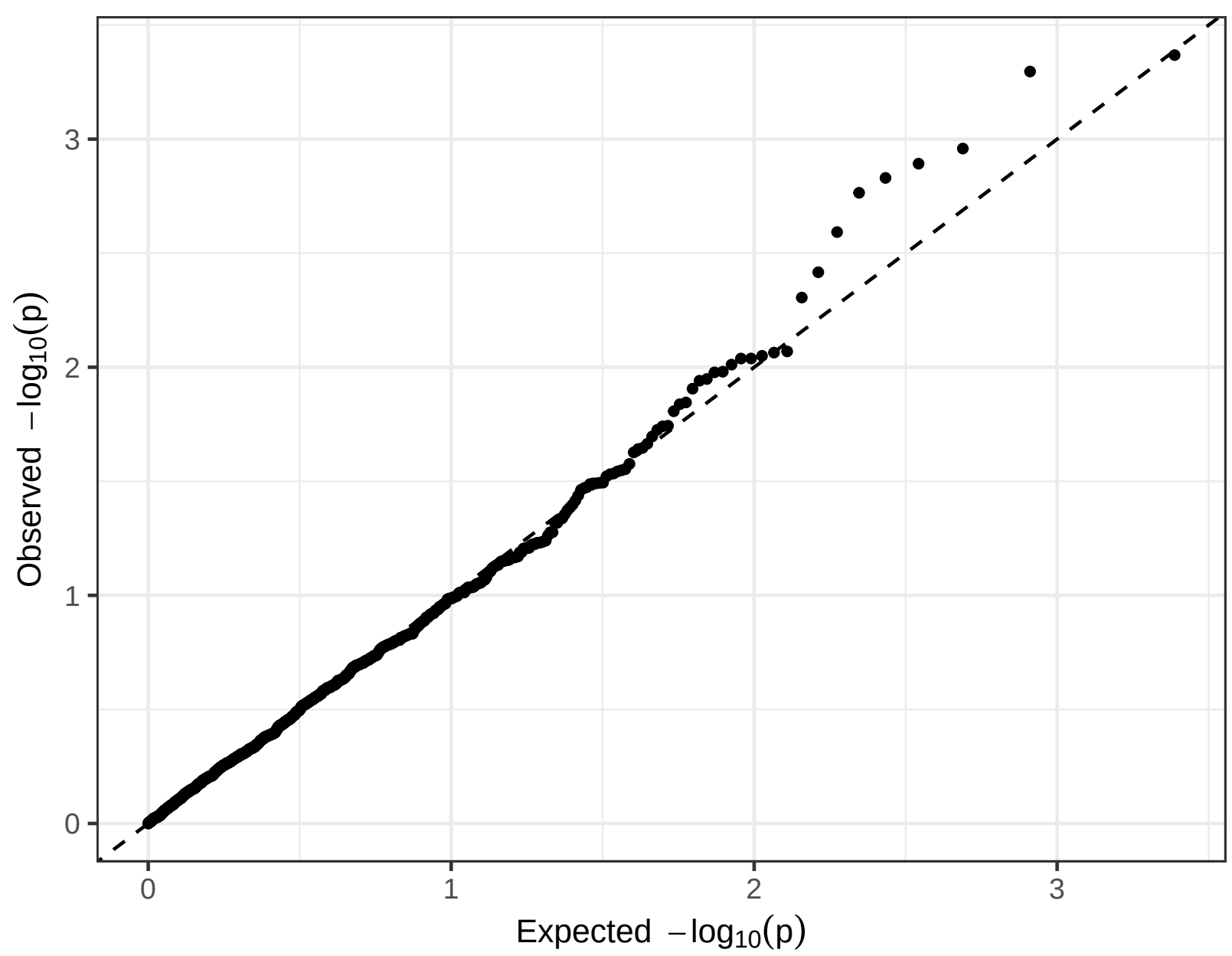

Candidatus_Methanomethylophilus

Cecum | Liver | . = 0.946

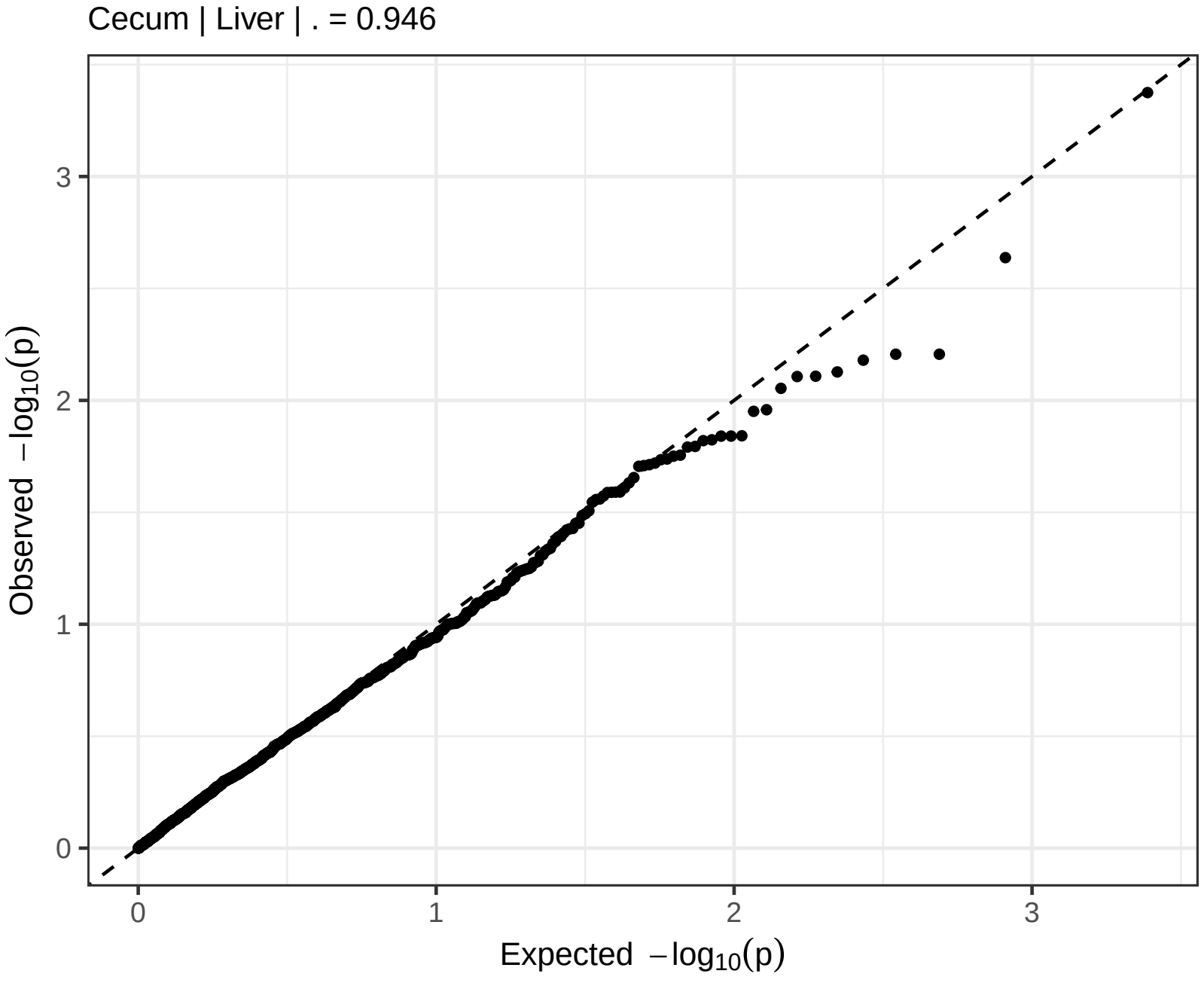

Candidatus_Methanoplasma

Cecum | Liver | . = 1.056

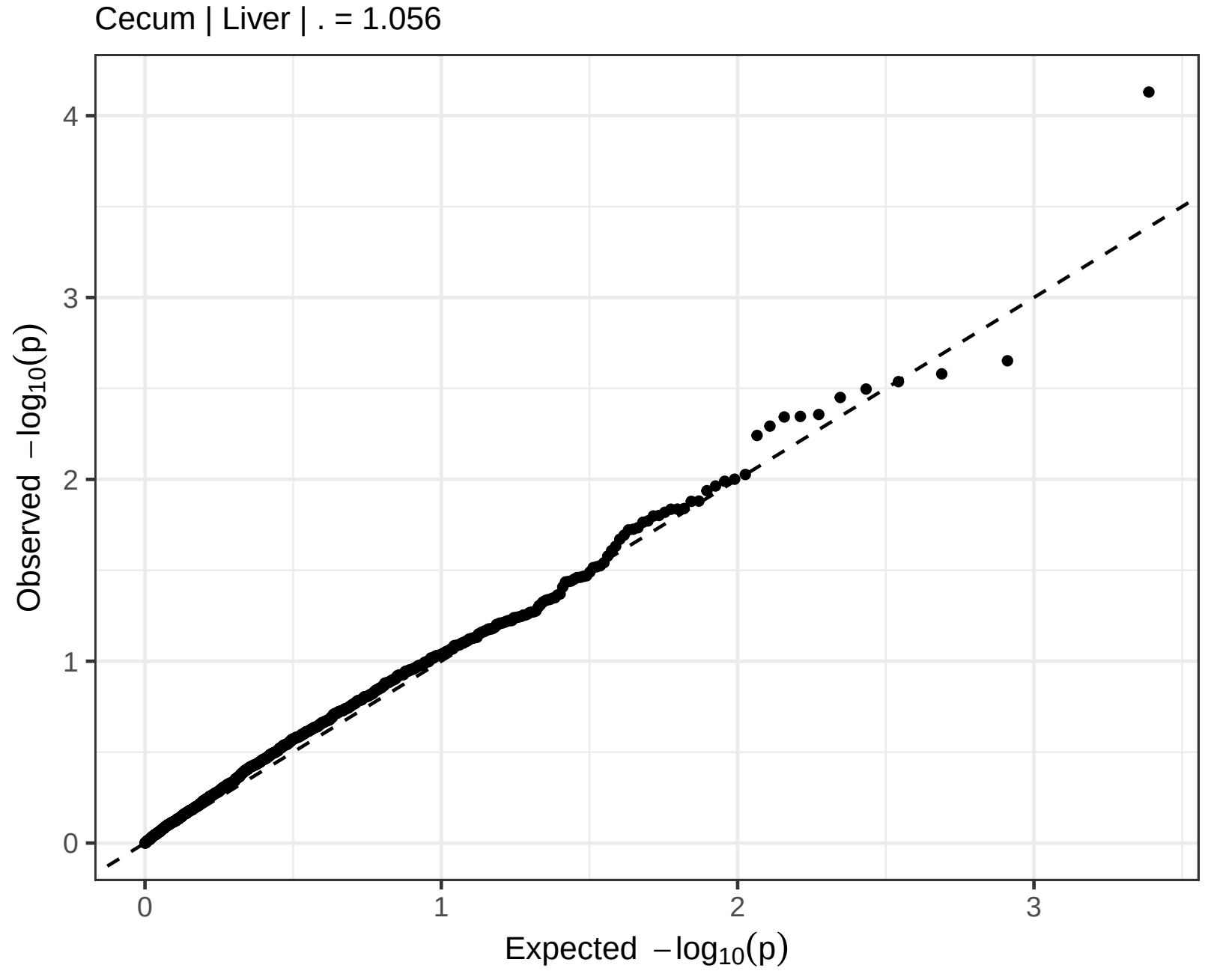

Candidatus_Saccharimonas

Cecum | Liver | . = 0.858

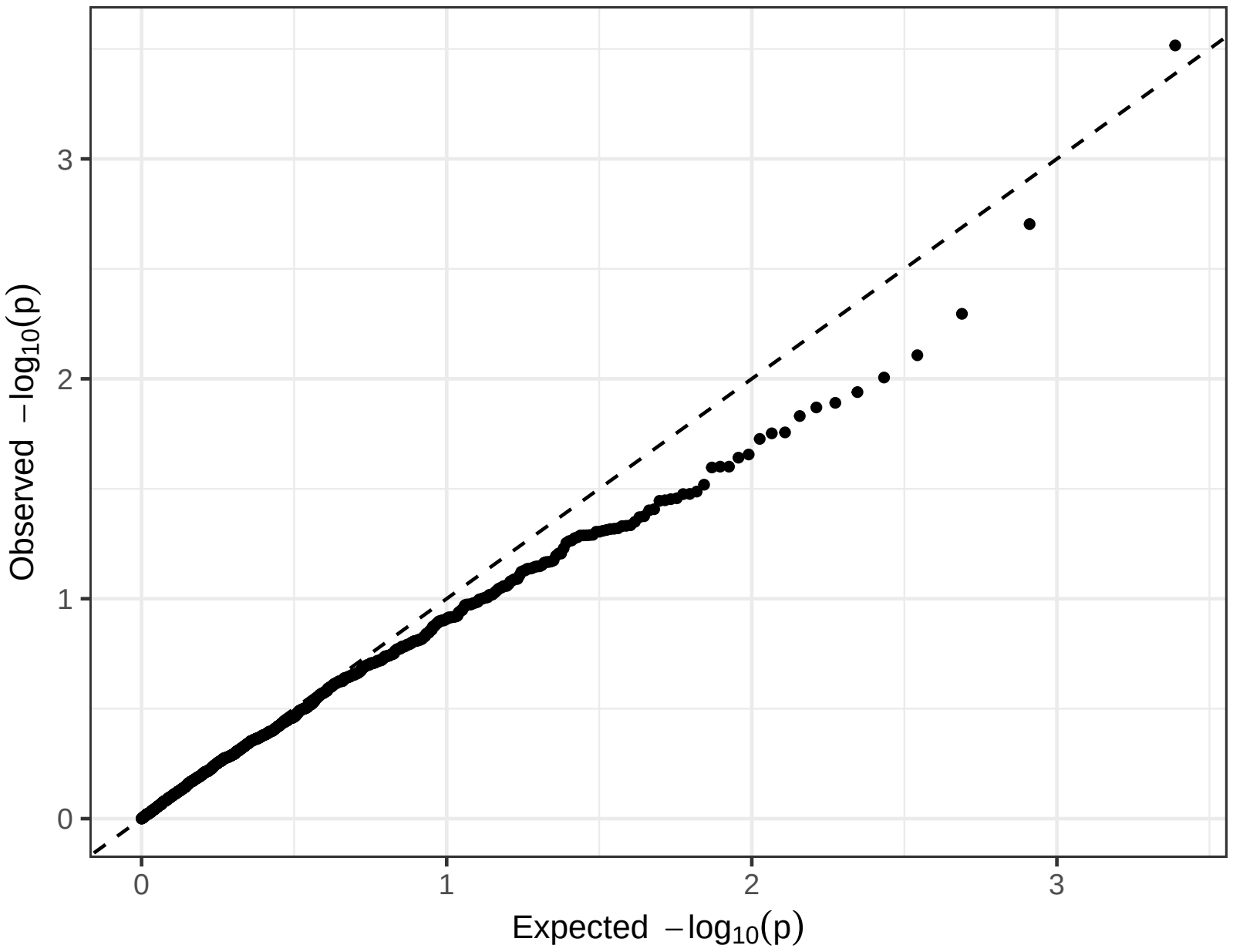

Candidatus_Soleaferrea

Cecum | Liver | . = 0.97

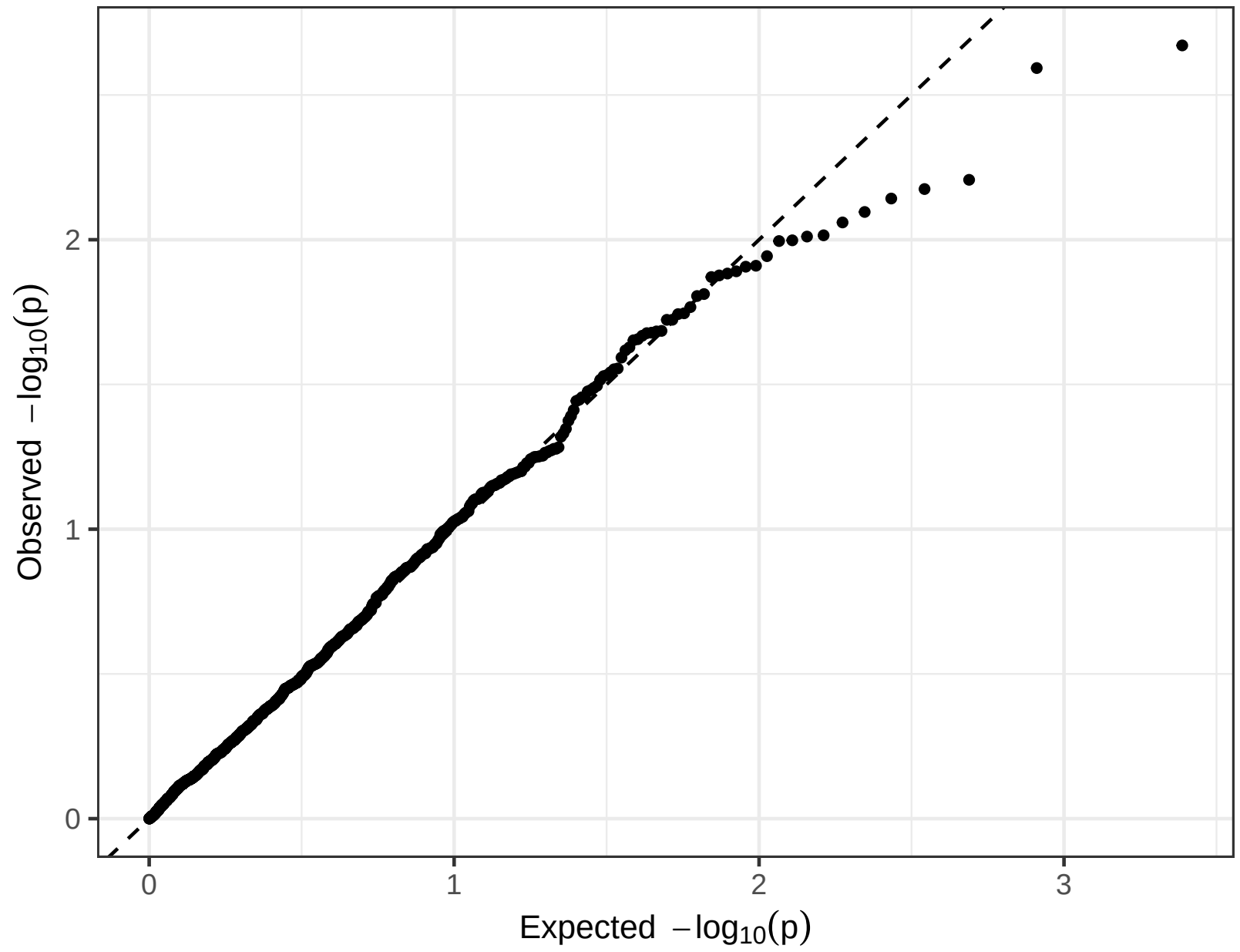

Caproiciproducens

Cecum | Liver | . = 0.952

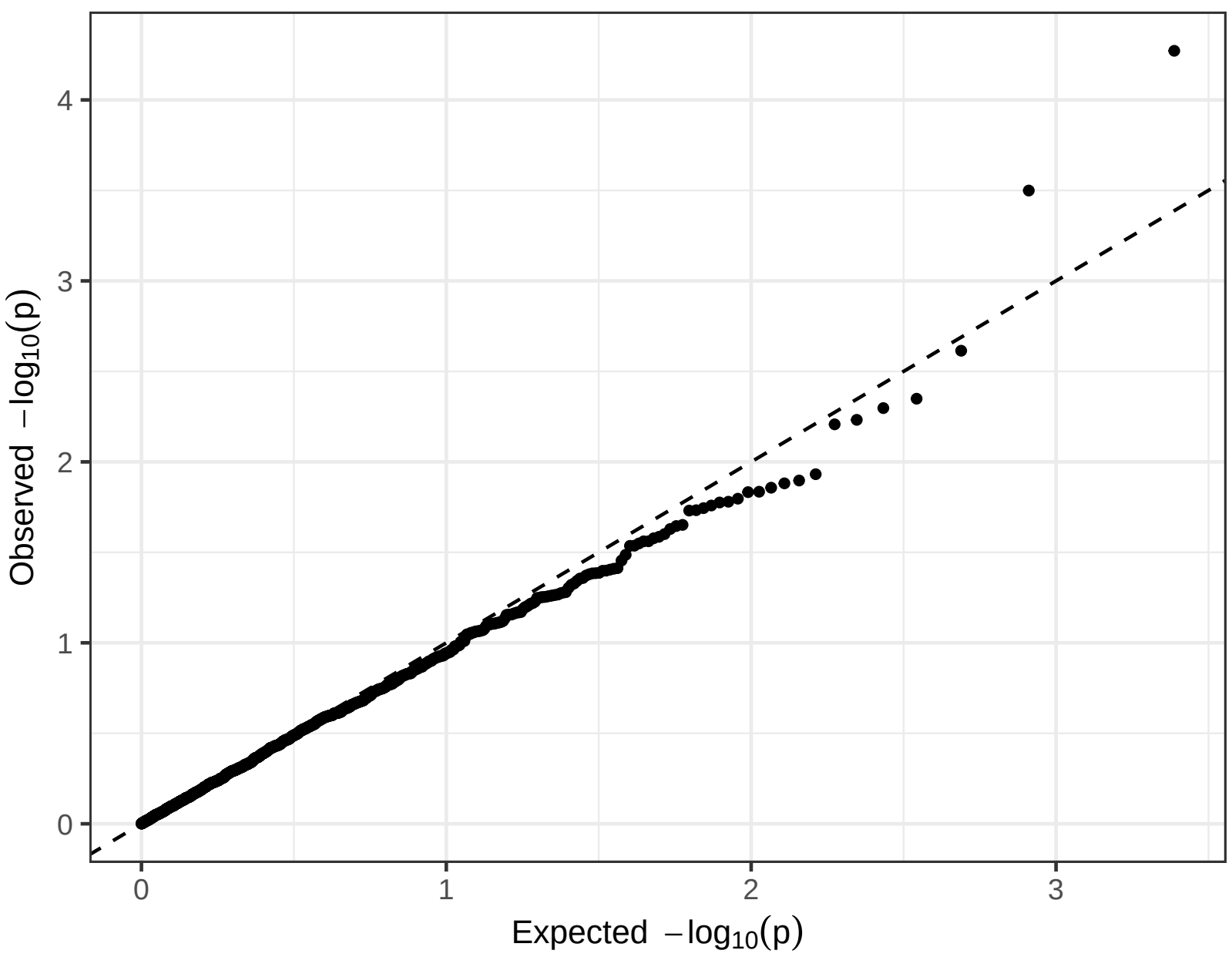

Catenibacterium

Cecum | Liver | . = 0.901

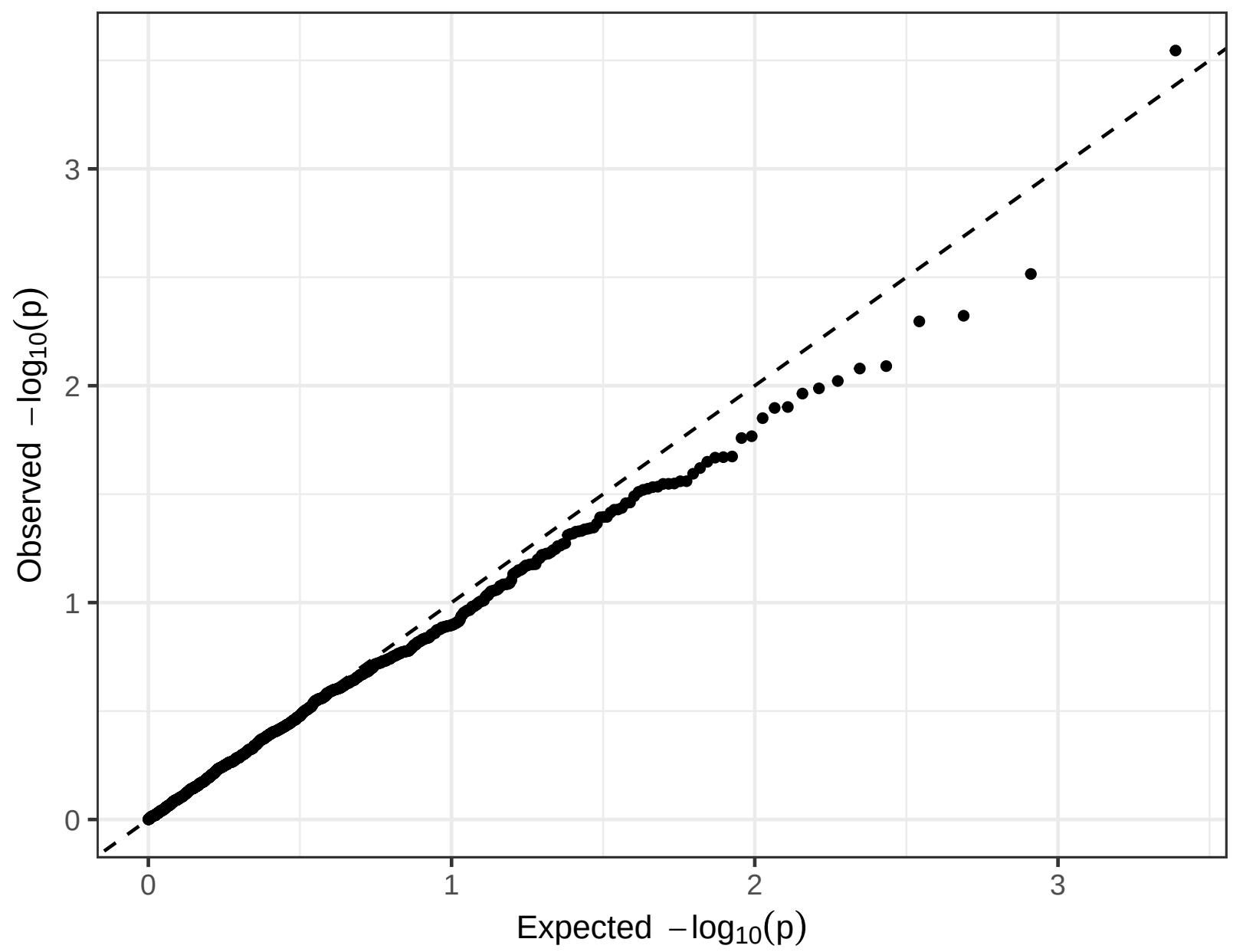

Catenisphaera

Cecum | Liver | . = 0.983

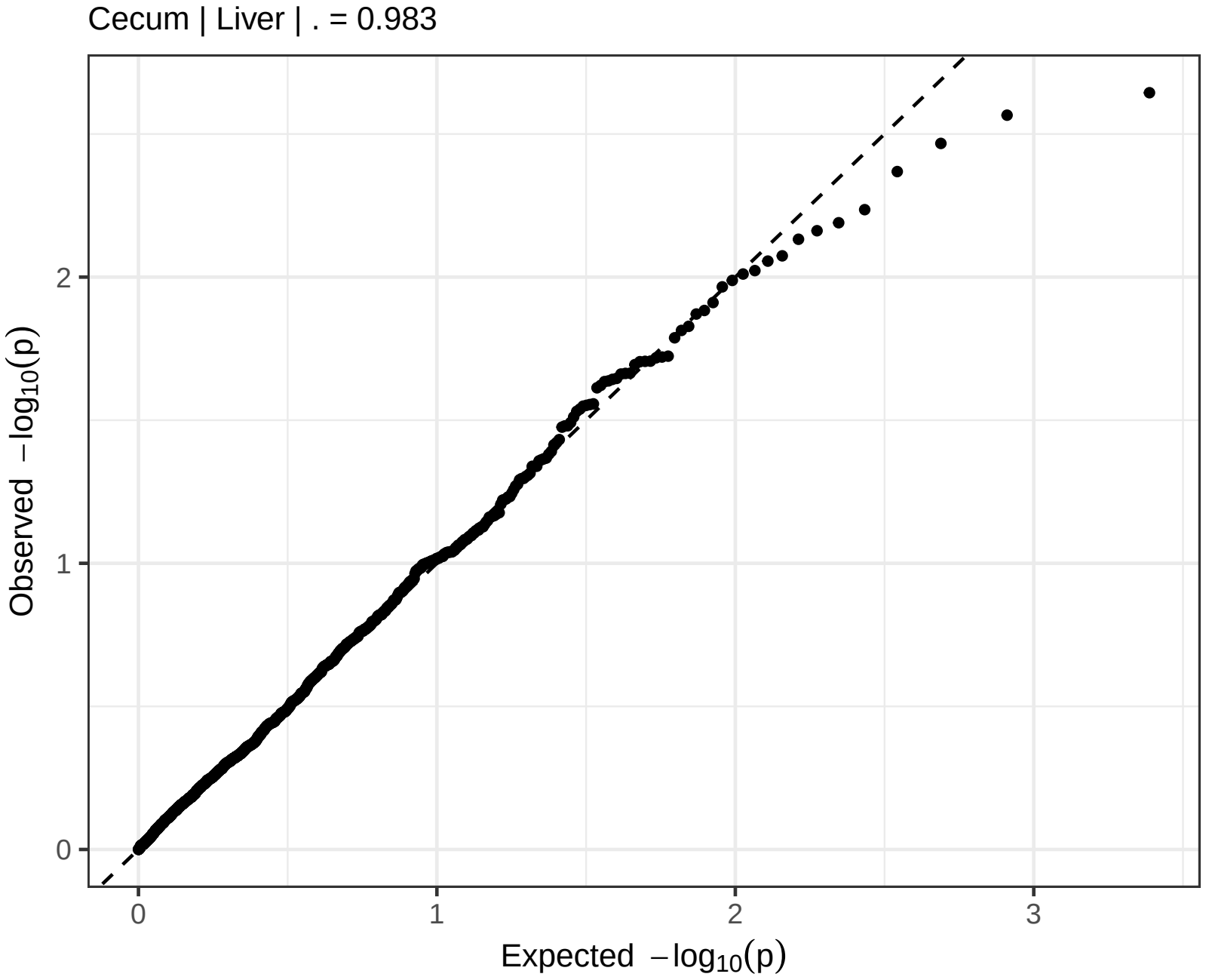

Eubacterium_xylanophilum_group

Cecum | Liver | . = 0.919

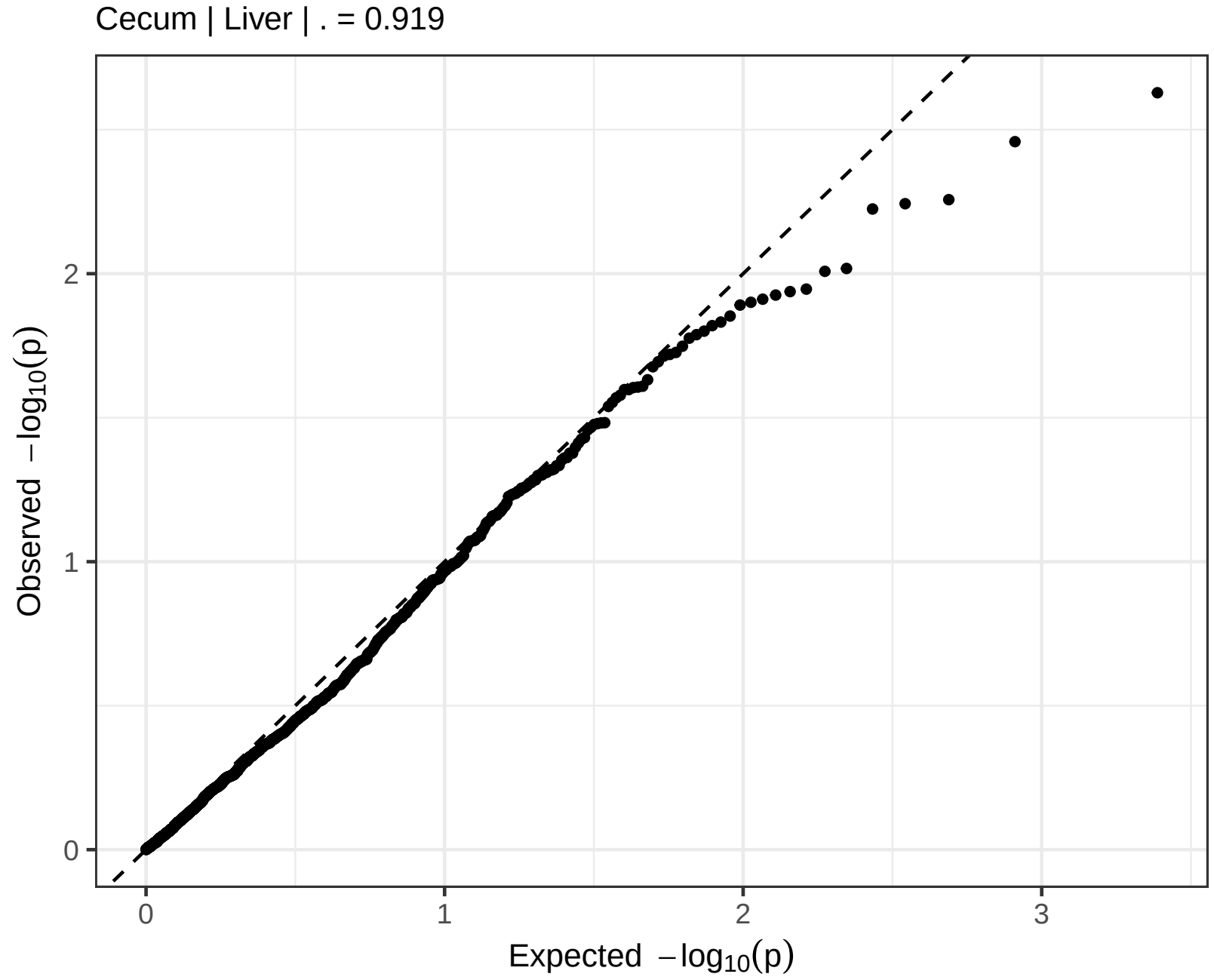

Cellulosilyticum

Cecum | Liver | . = 0.886

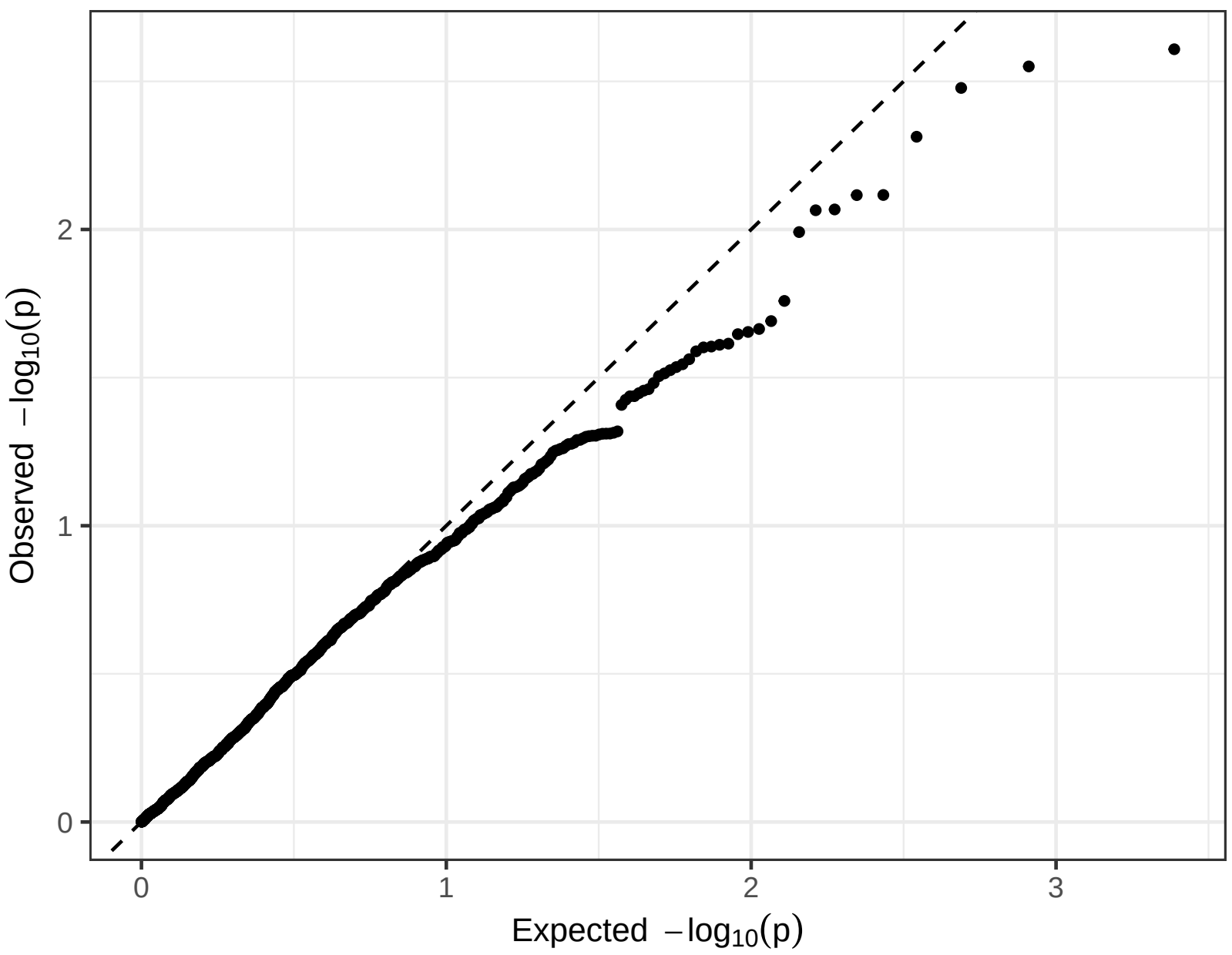

Christensenellaceae_R.7_group

Cecum | Liver | . = 1.032

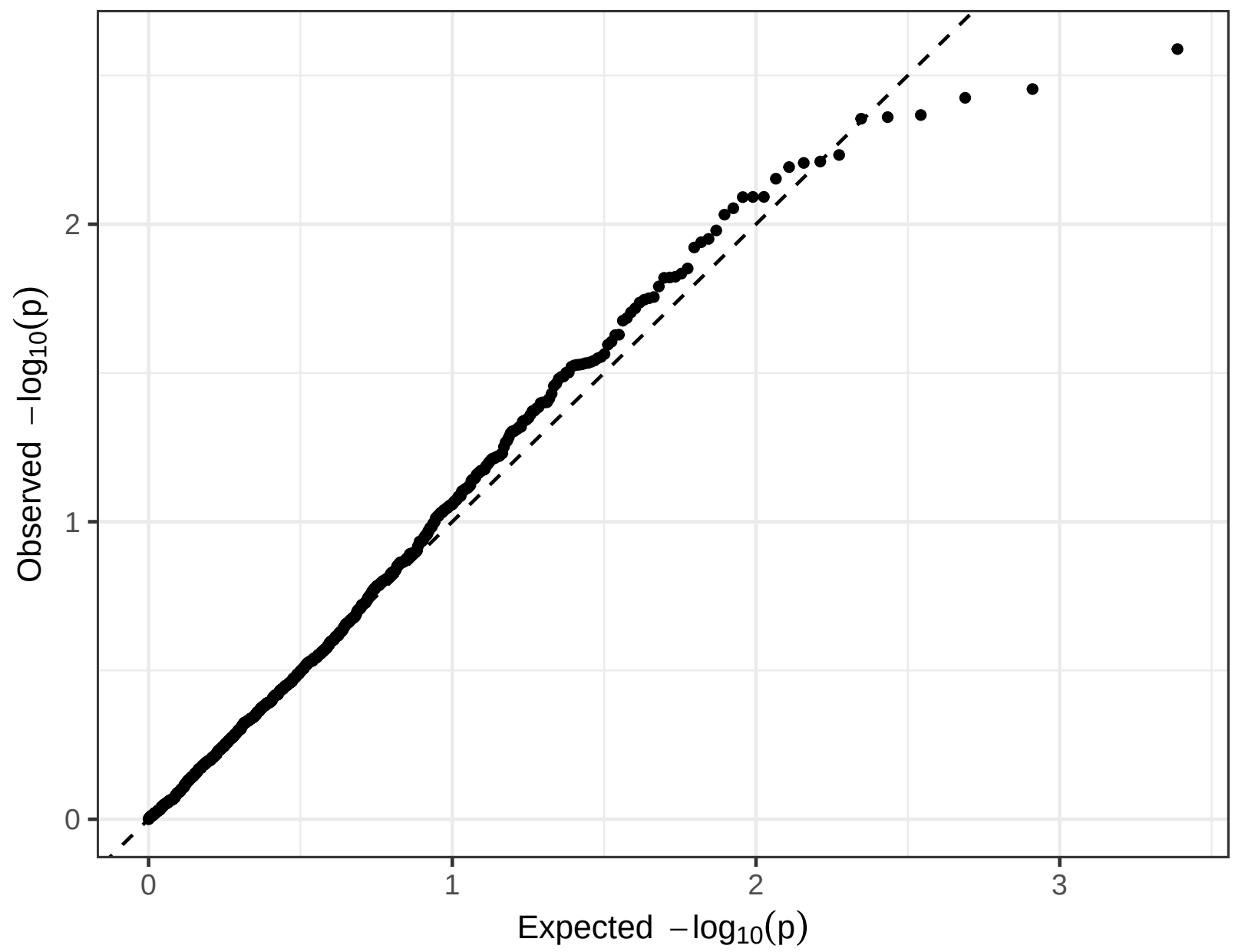

Cloacibacillus

Cecum | Liver | . = 0.897

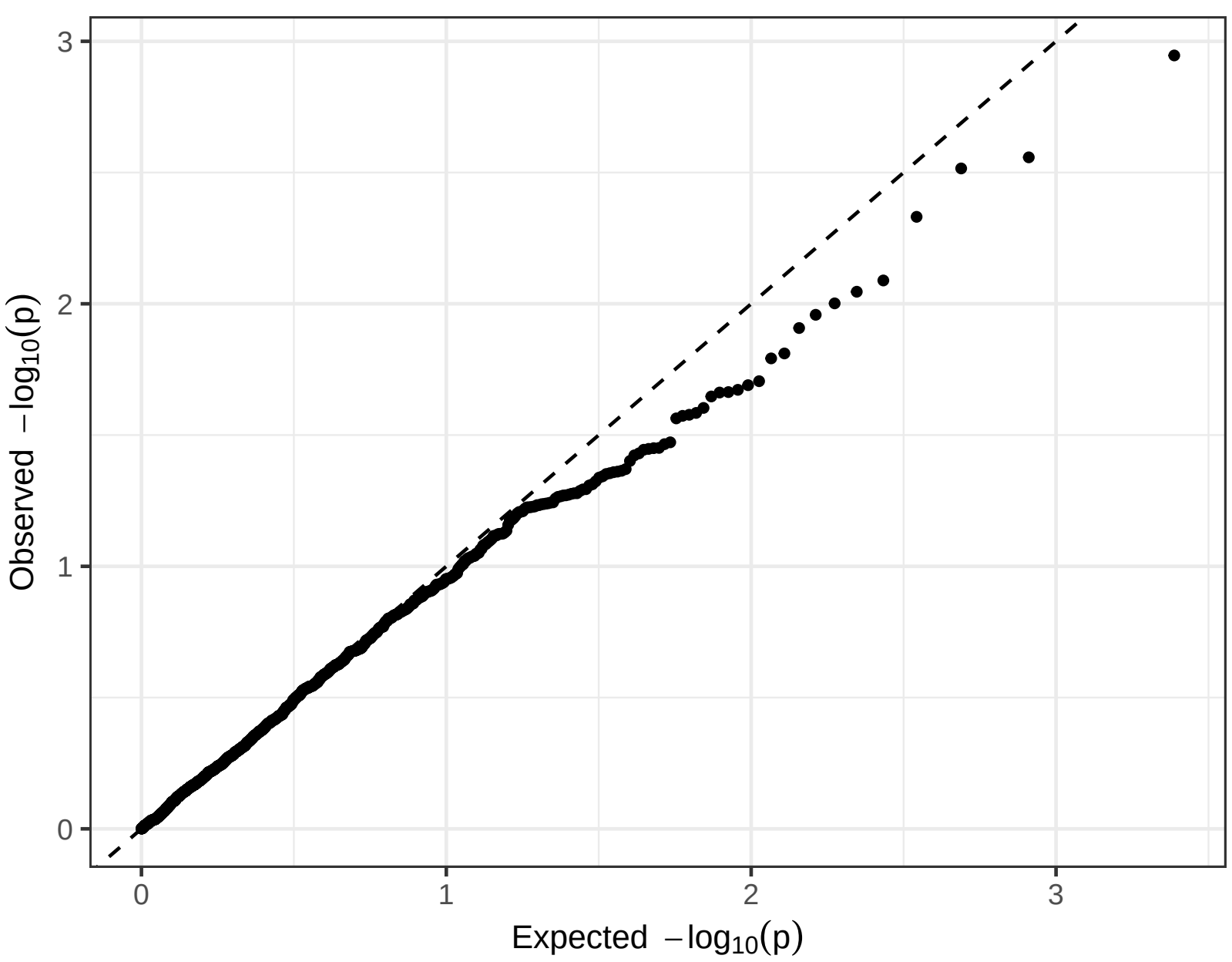

Clostridium

Cecum | Liver | . = 0.895

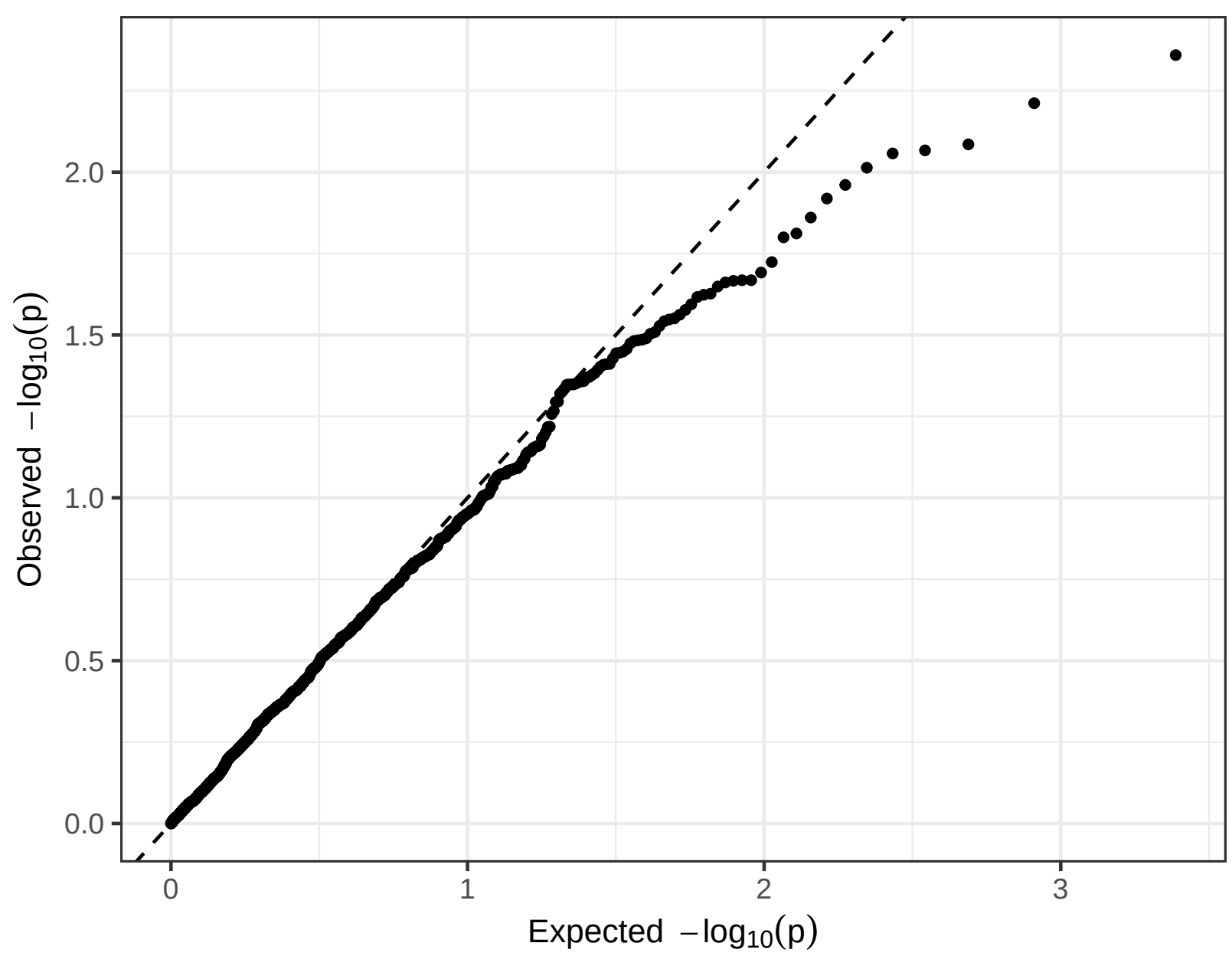

Colidextribacter

Cecum | Liver | . = 0.96

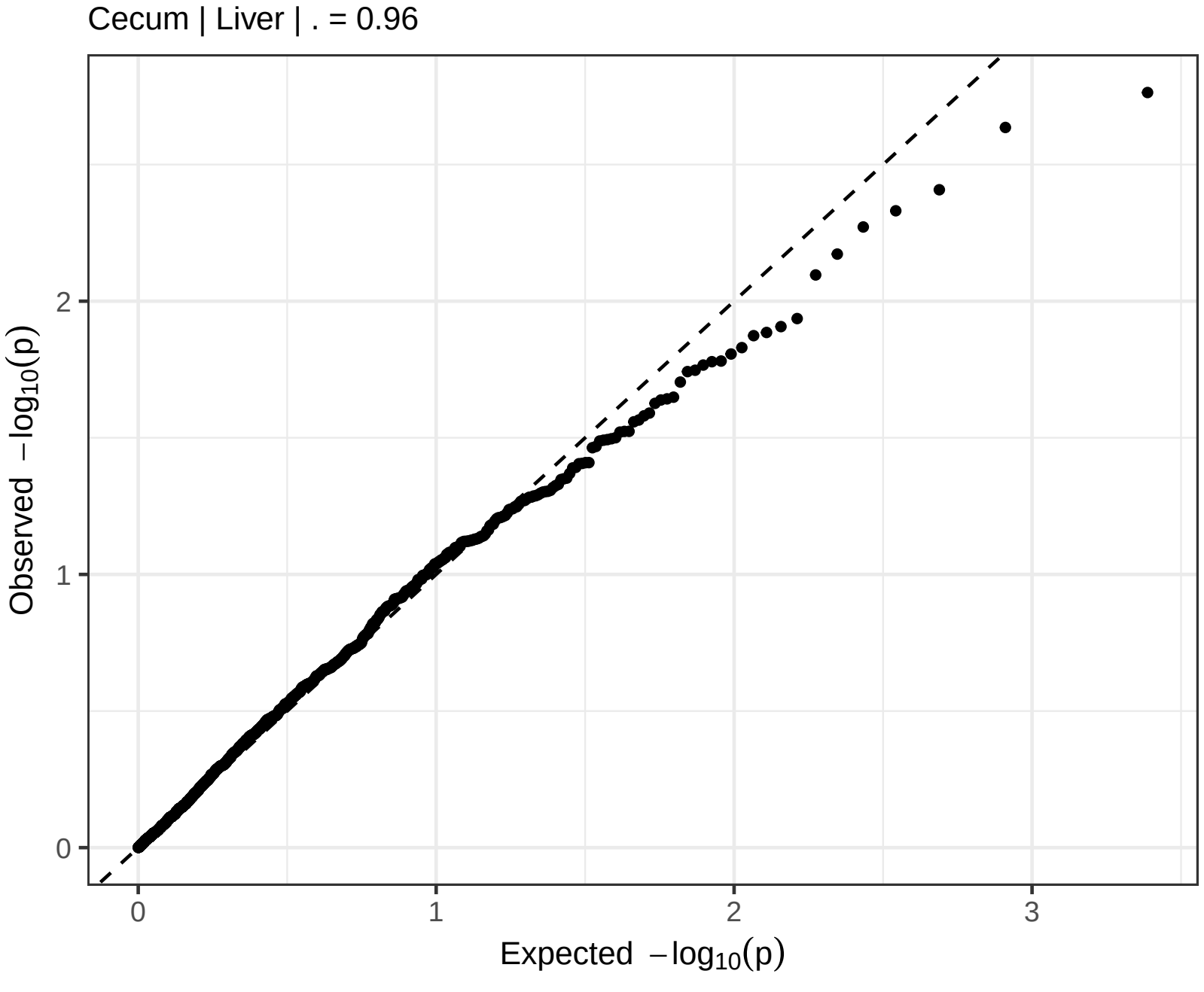

Collinsella

Cecum | Liver | . = 1.011

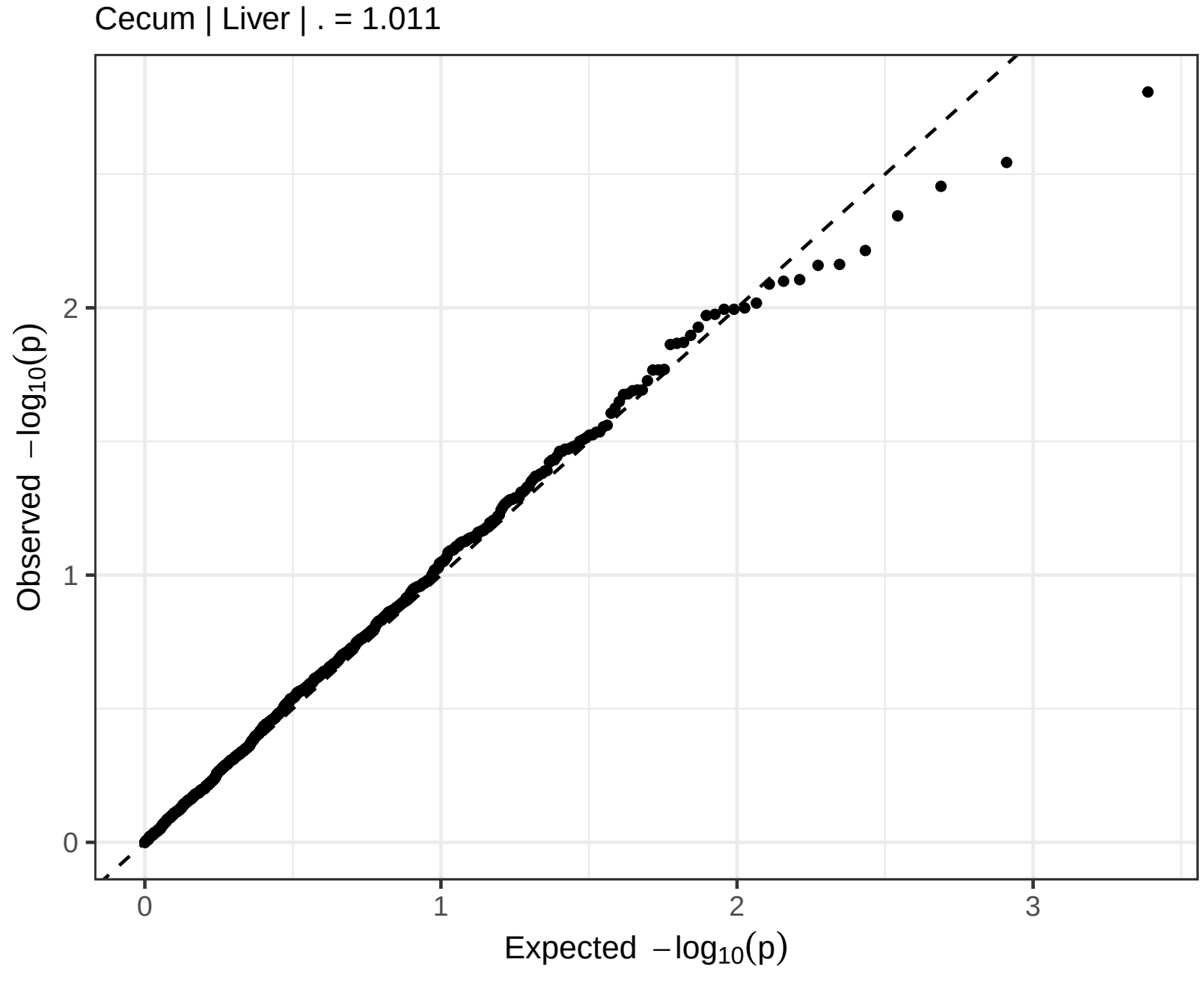

Coprococcus

Cecum | Liver | . = 1.092

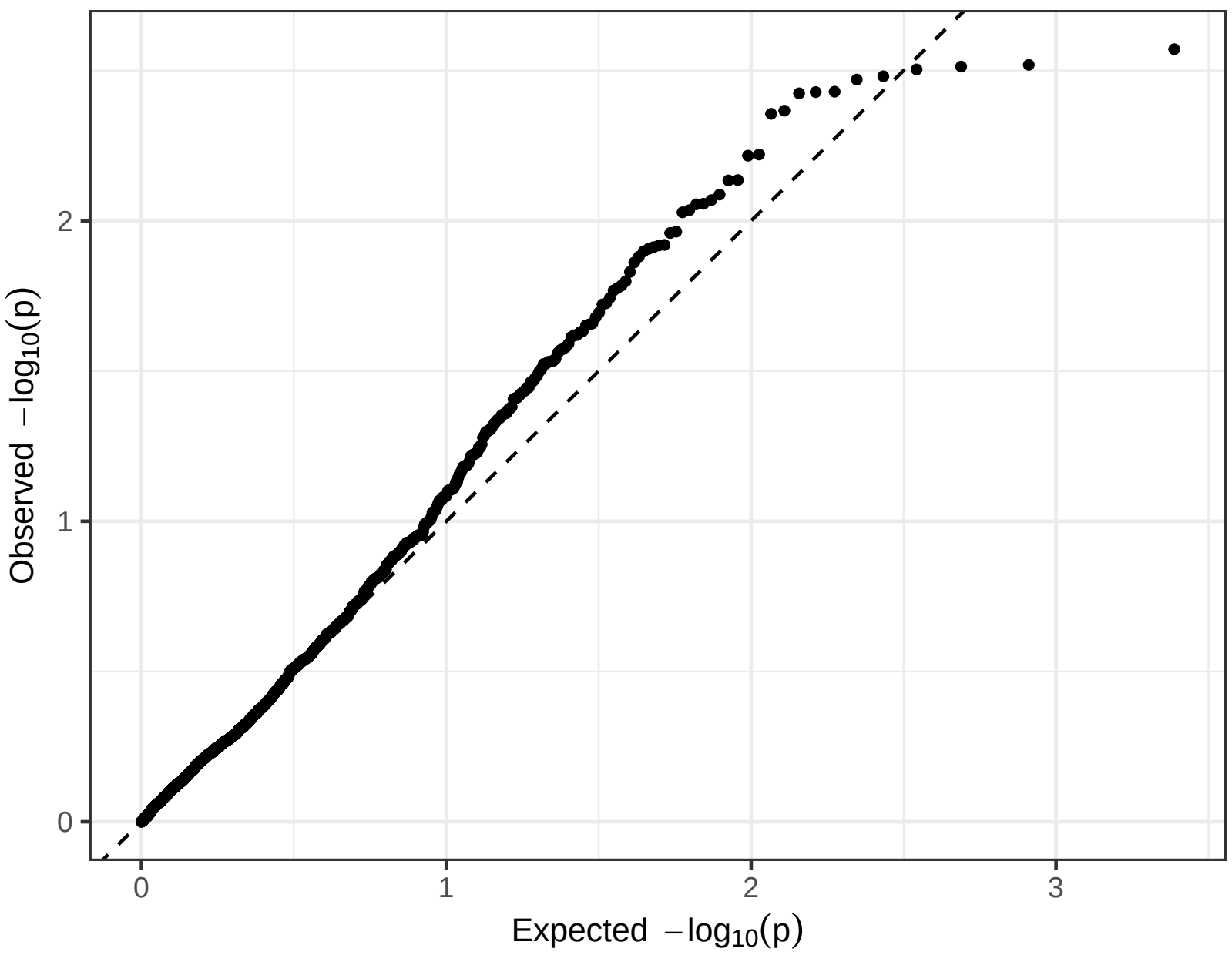

Defluviitaleaceae_UCG.011

Cecum | Liver | . = 0.89

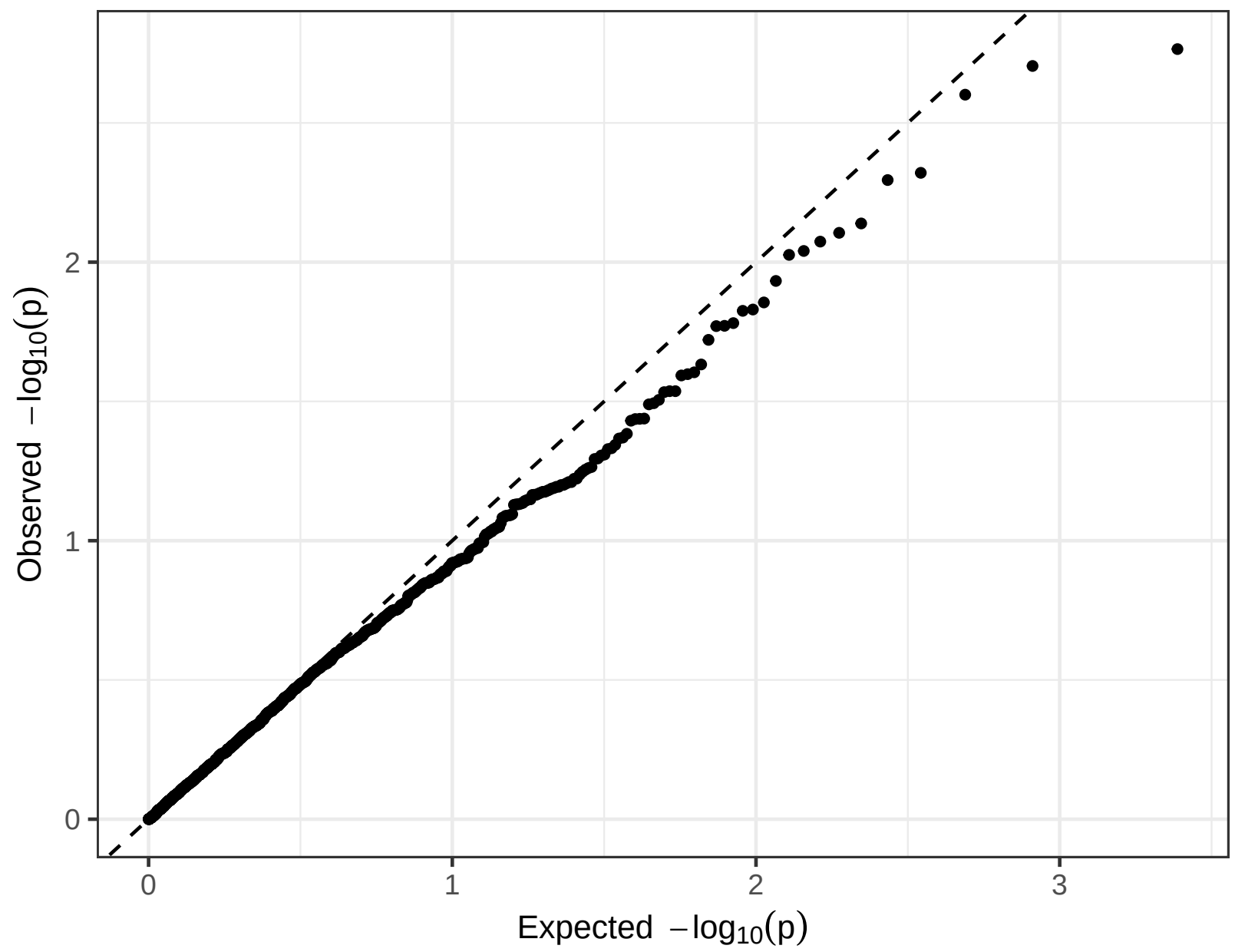

Desulfovibrio

Cecum | Liver | . = 0.909

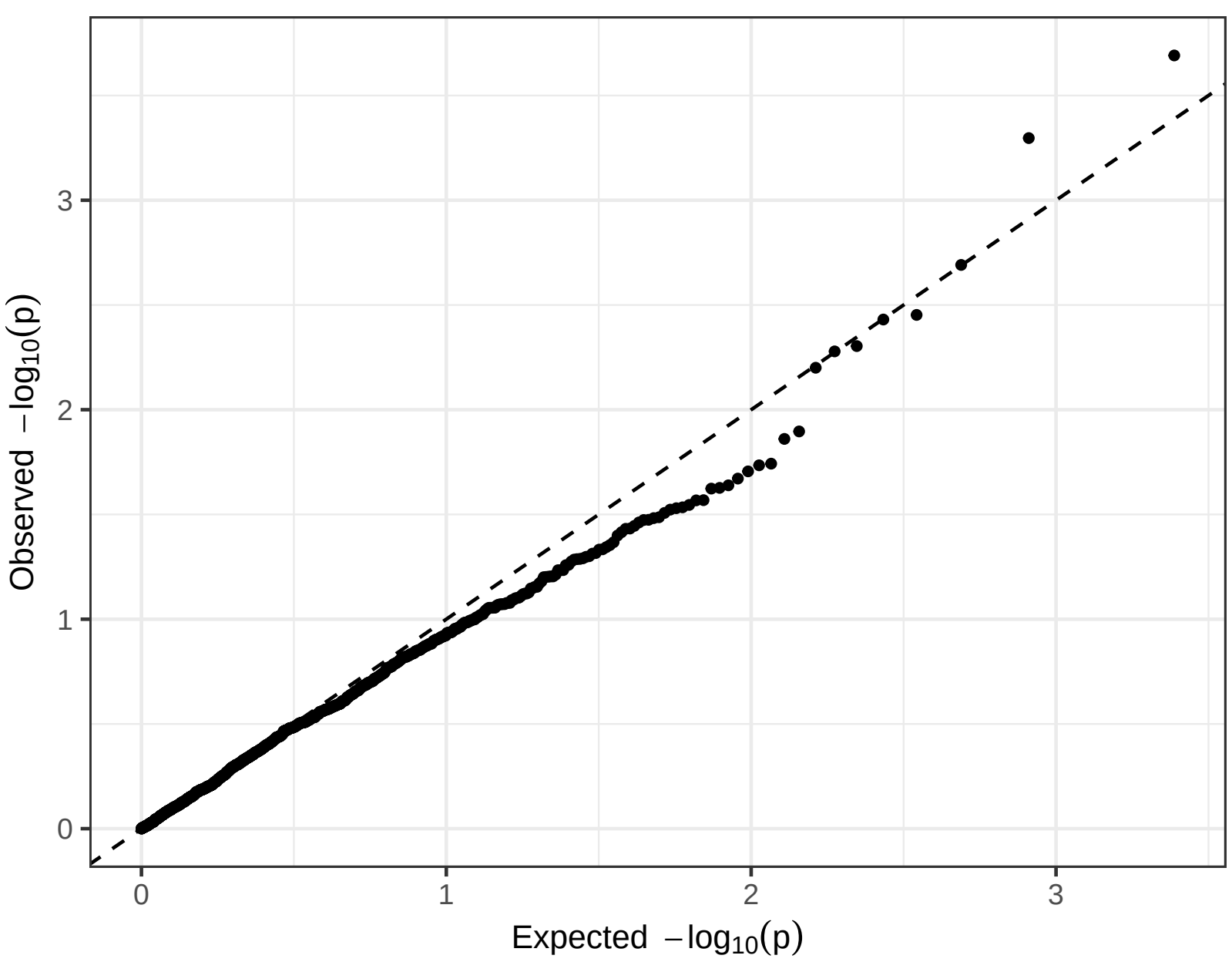

dgA.11_gut_group

Cecum | Liver | . = 1.018

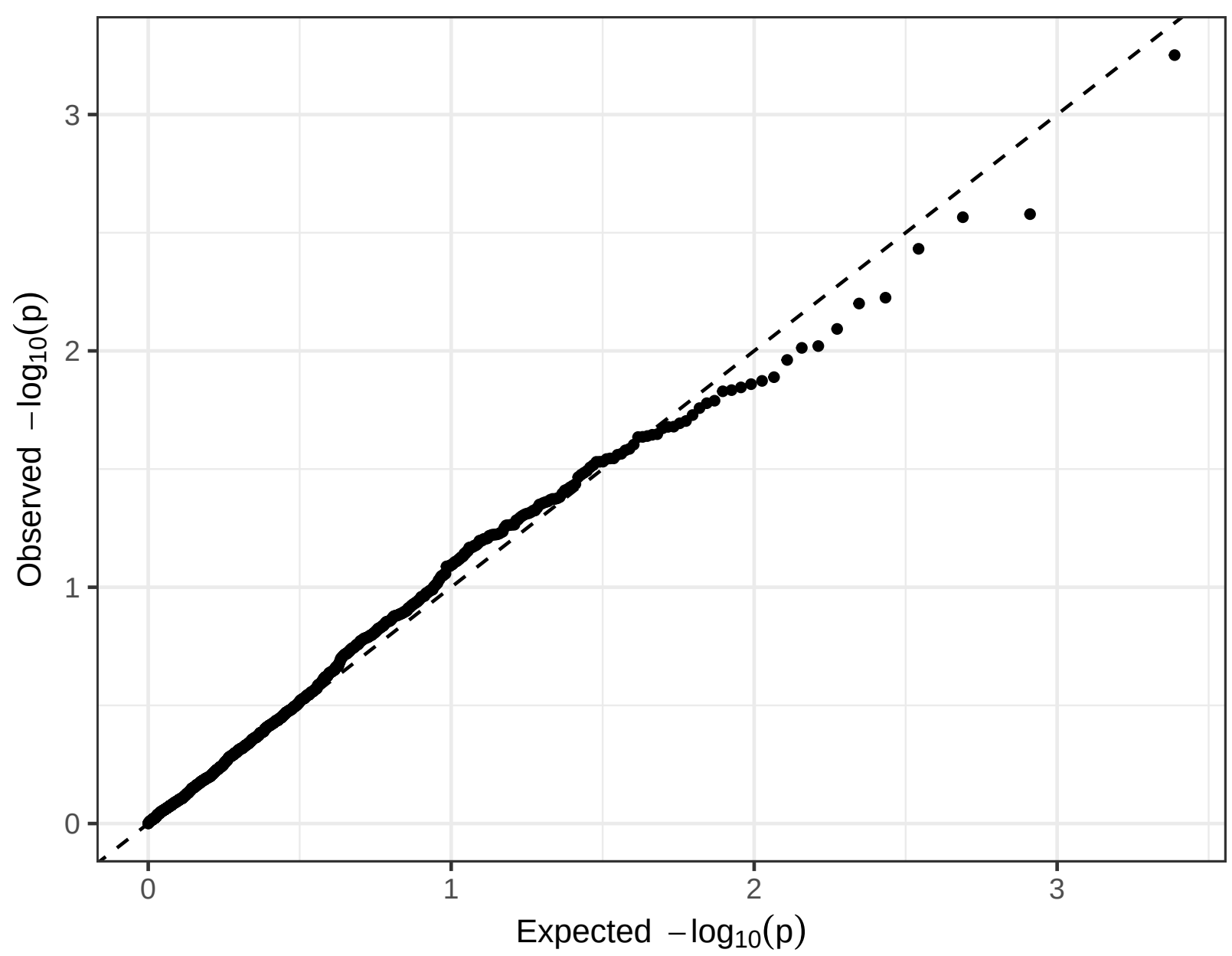

Ruminococcus_gauvreauii_group

Cecum | Liver | . = 0.885

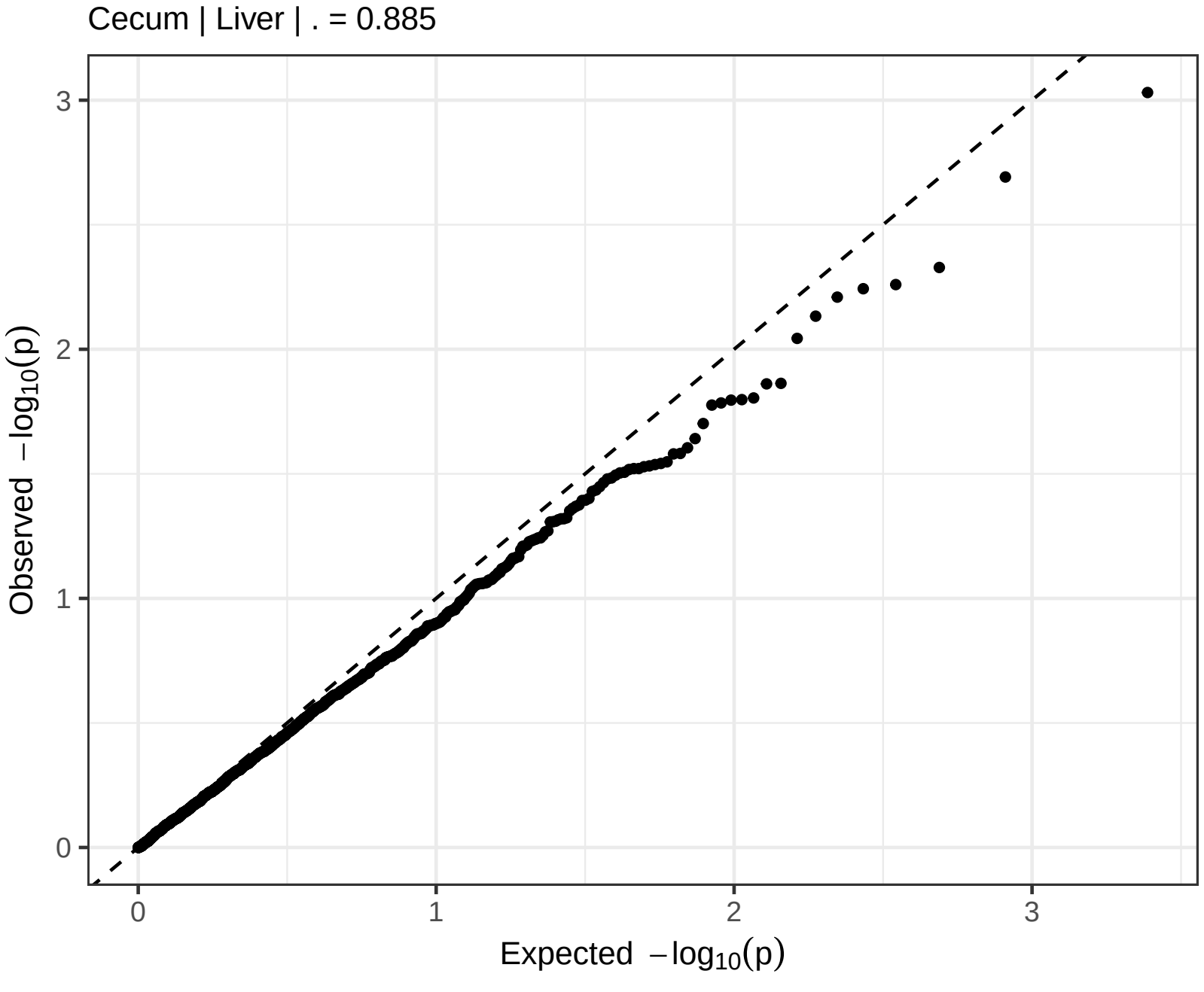

Dorea

Cecum | Liver | . = 1.072

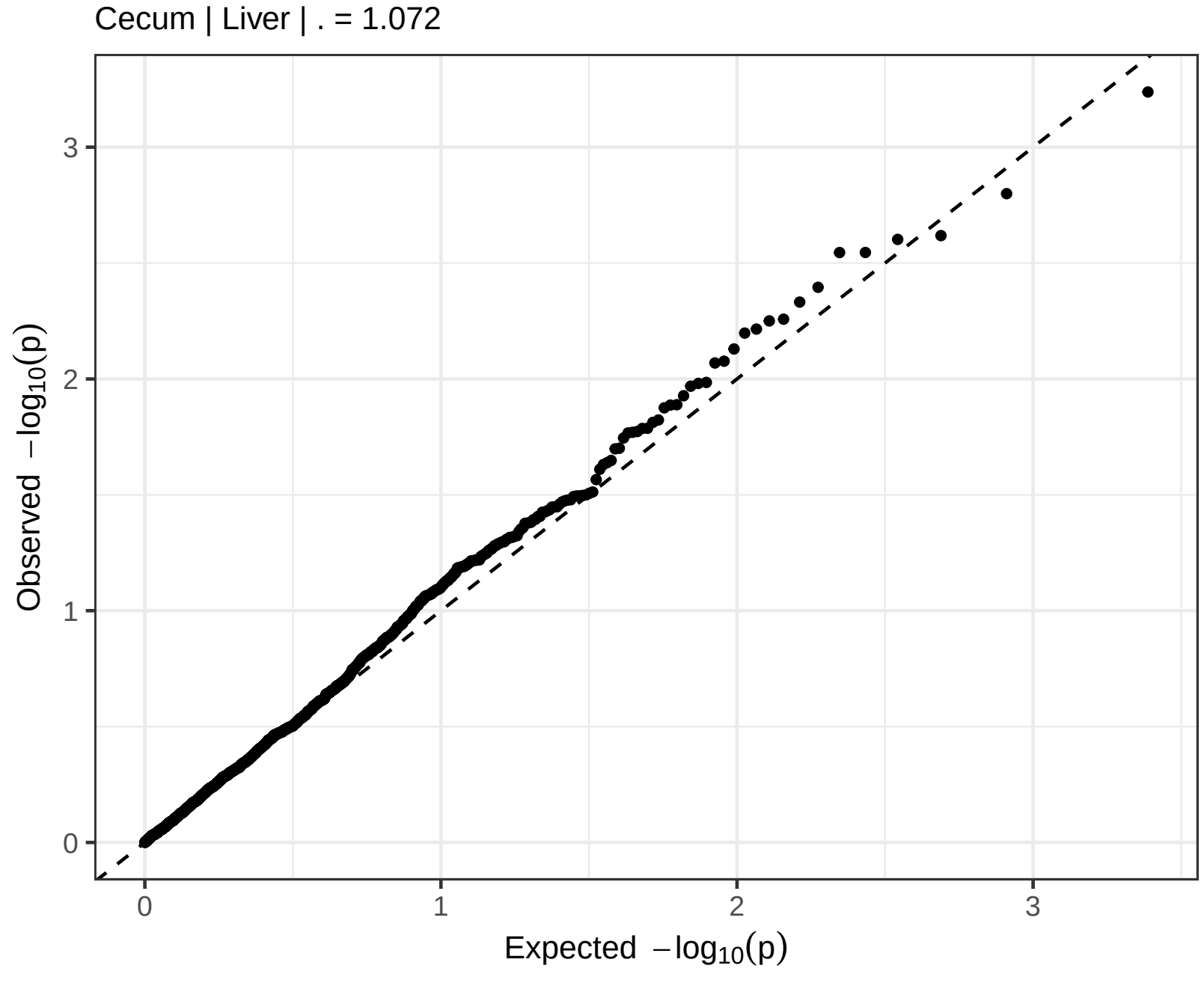

Eisenbergiella

Cecum | Liver | . = 1.065

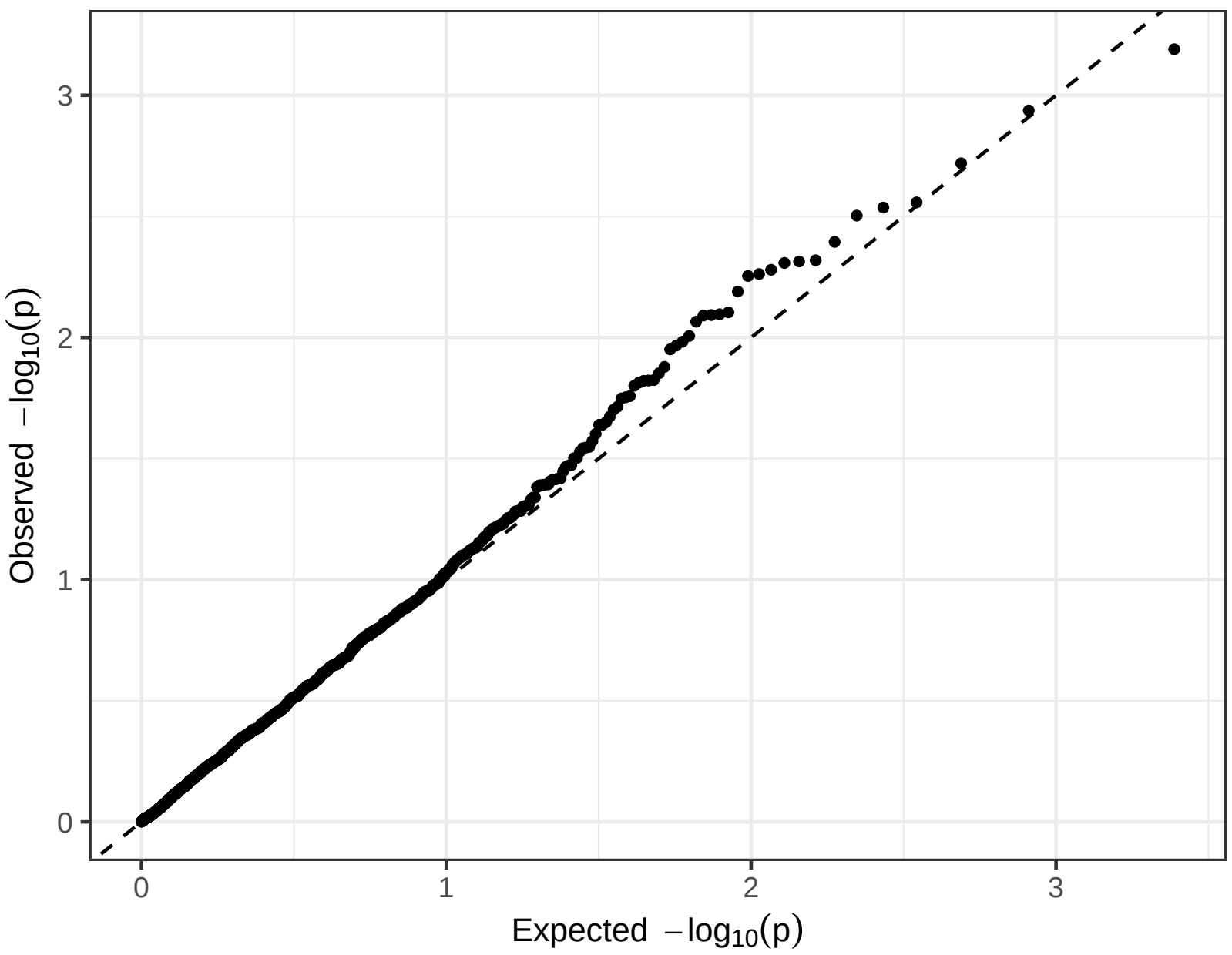

Elusimicrobium

Cecum | Liver | . = 0.873

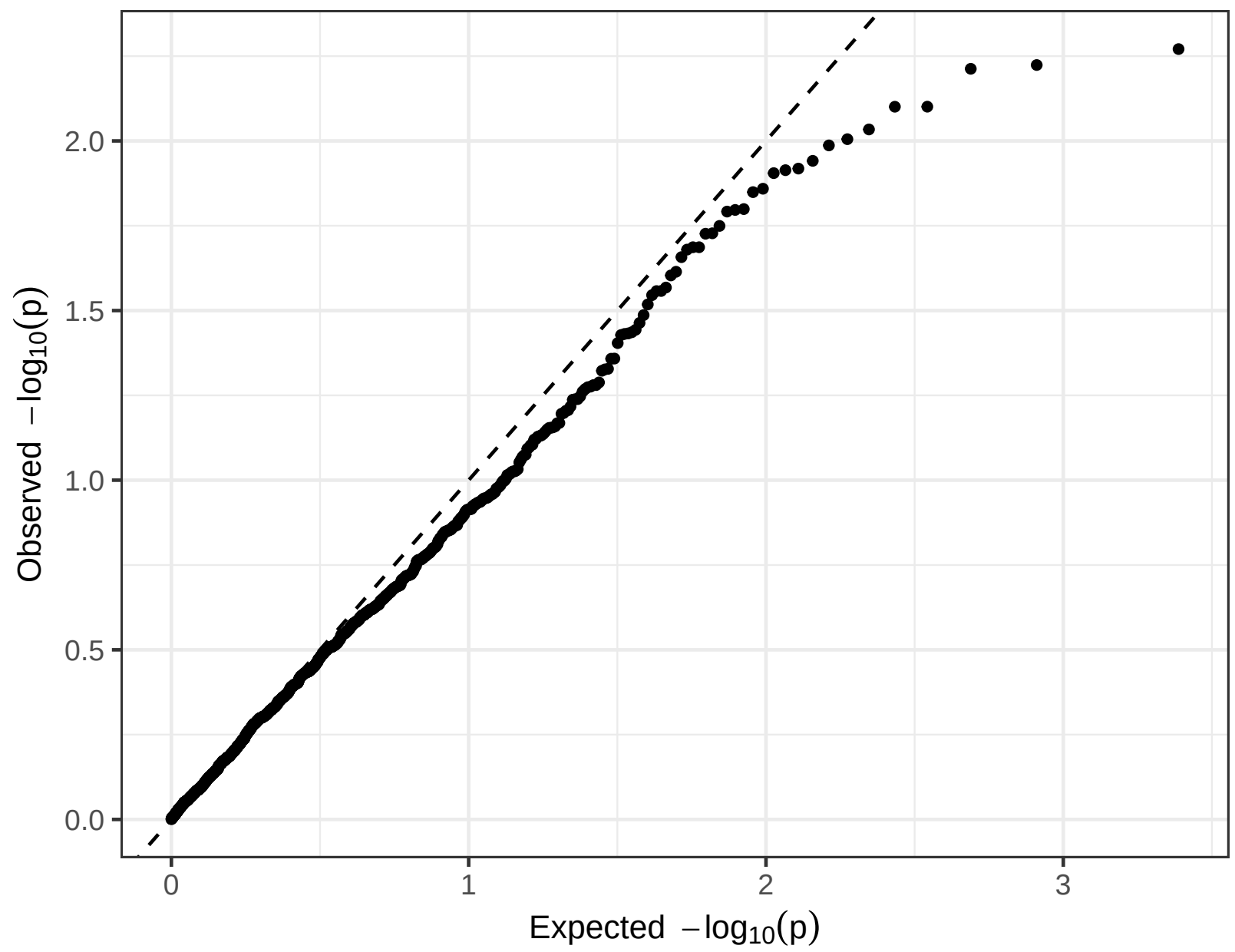

Enterocloster

Cecum | Liver | . = 1.079

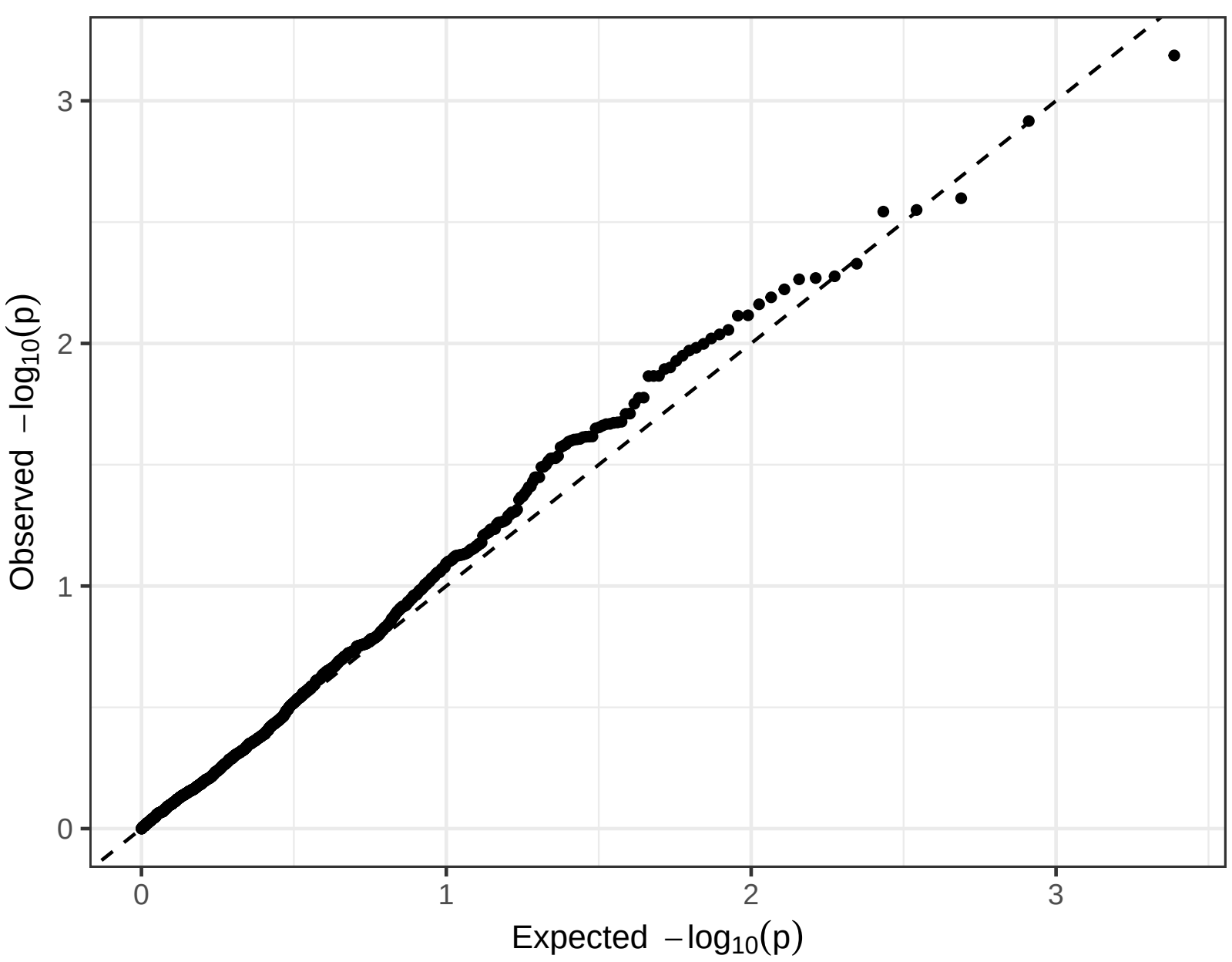

Epulopiscium

Cecum | Liver | . = 0.921

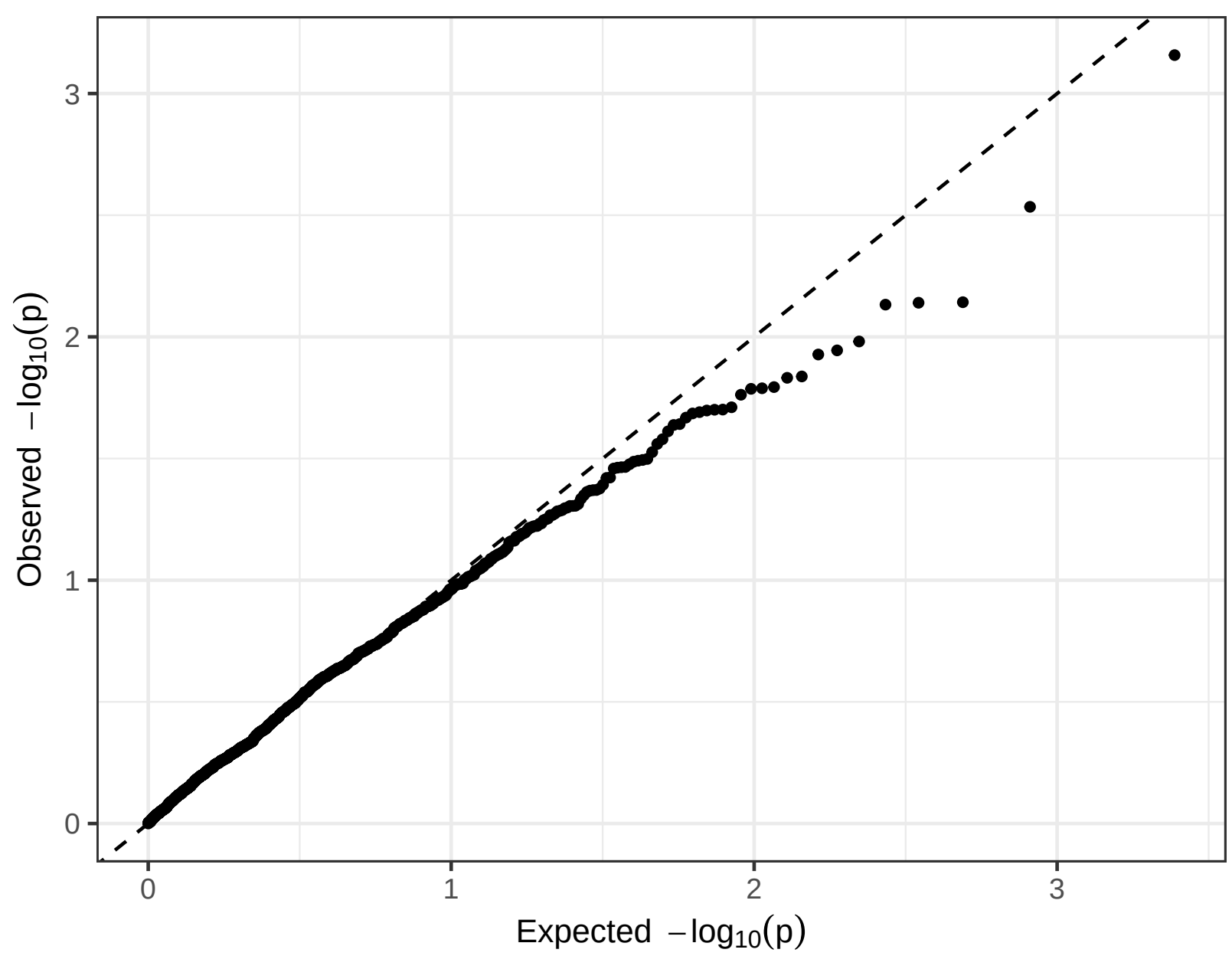

Erysipelotrichaceae_UCG.003

Cecum | Liver | . = 0.902

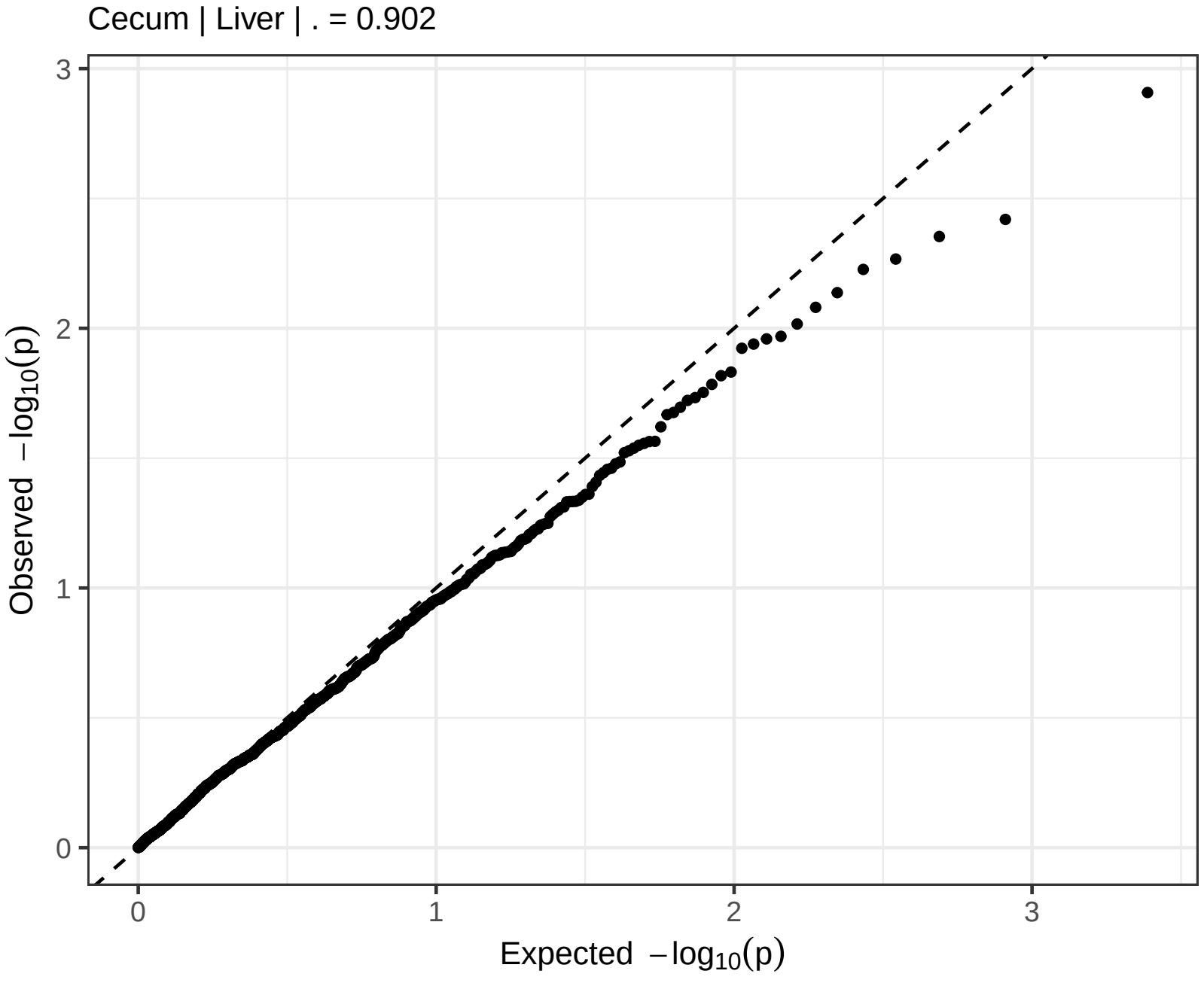

Escherichia.Shigella

Cecum | Liver | . = 0.98

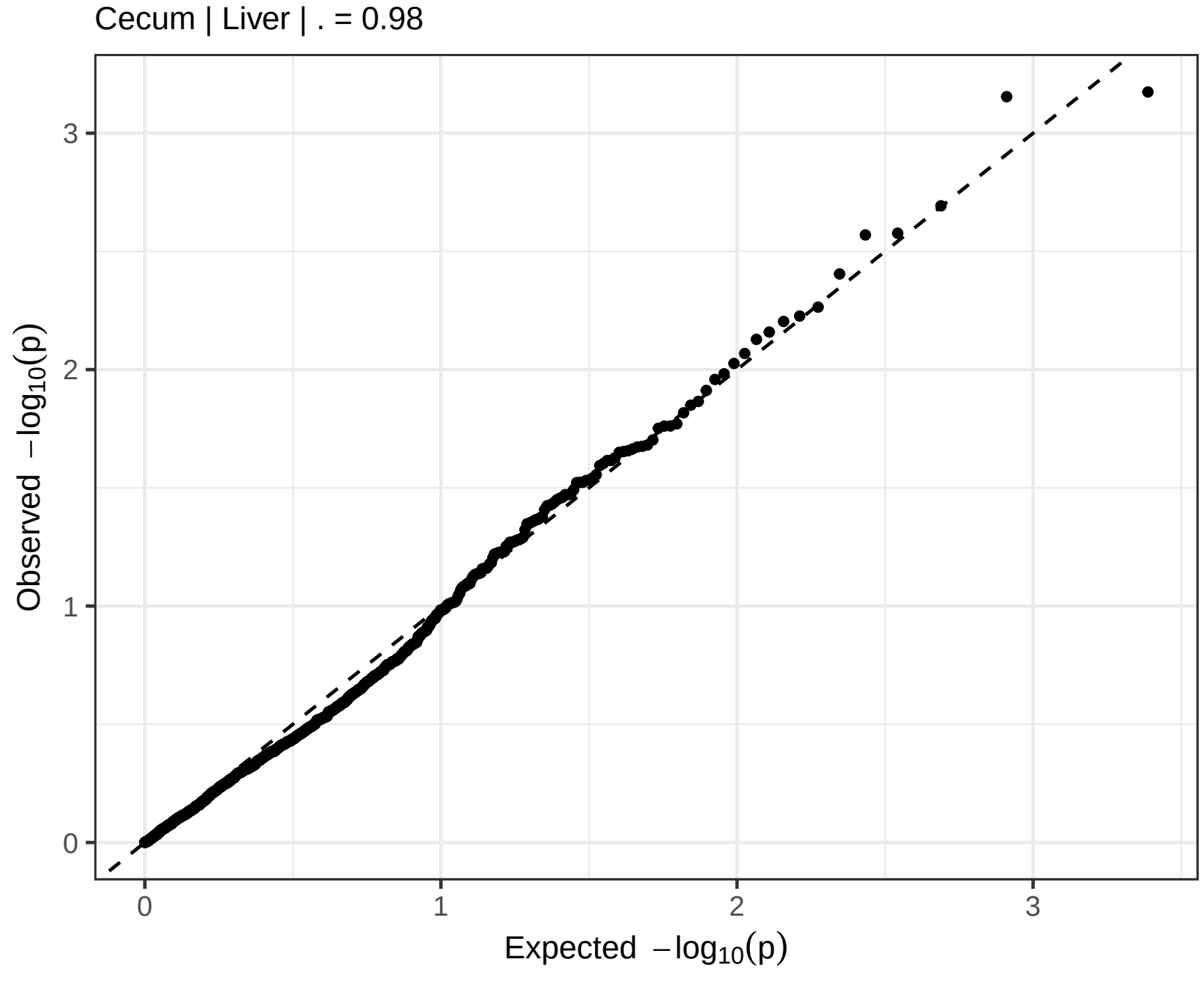

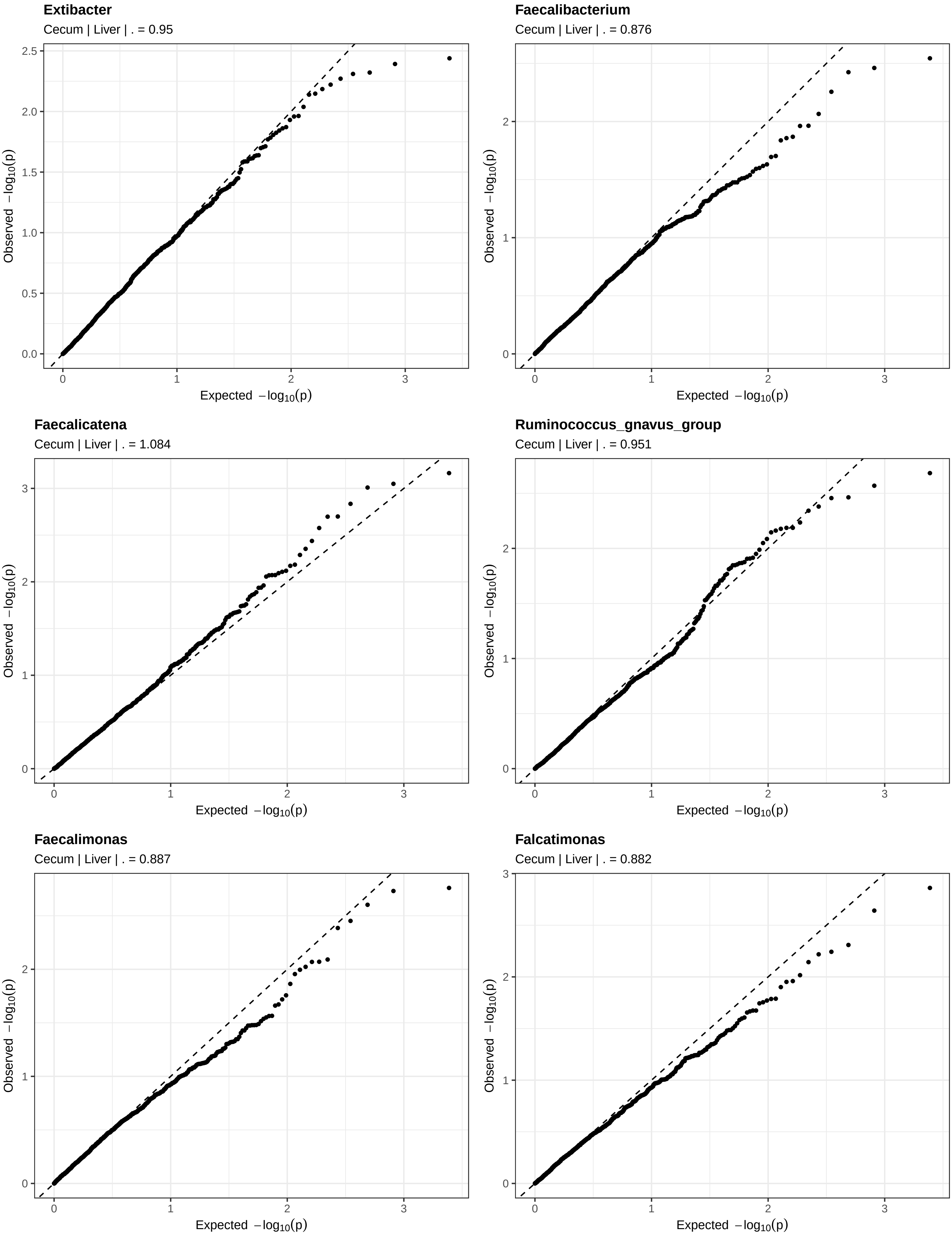
Extibacter
Cecum | Liver | . = 0.95
Faecalibacterium
Cecum | Liver | . = 0.876
Faecalicatena
Cecum | Liver | . = 1.084
Ruminococcus_gnavus_group
Cecum | Liver | . = 0.951
Faecalimonas
Cecum | Liver | . = 0.887
Falcatimonas
Cecum | Liver | . = 0.882
Observed $-\log_{10}(p)$
Expected $-\log_{10}(p)$

Family_XIII_AD3011_group

Cecum | Liver | . = 0.942

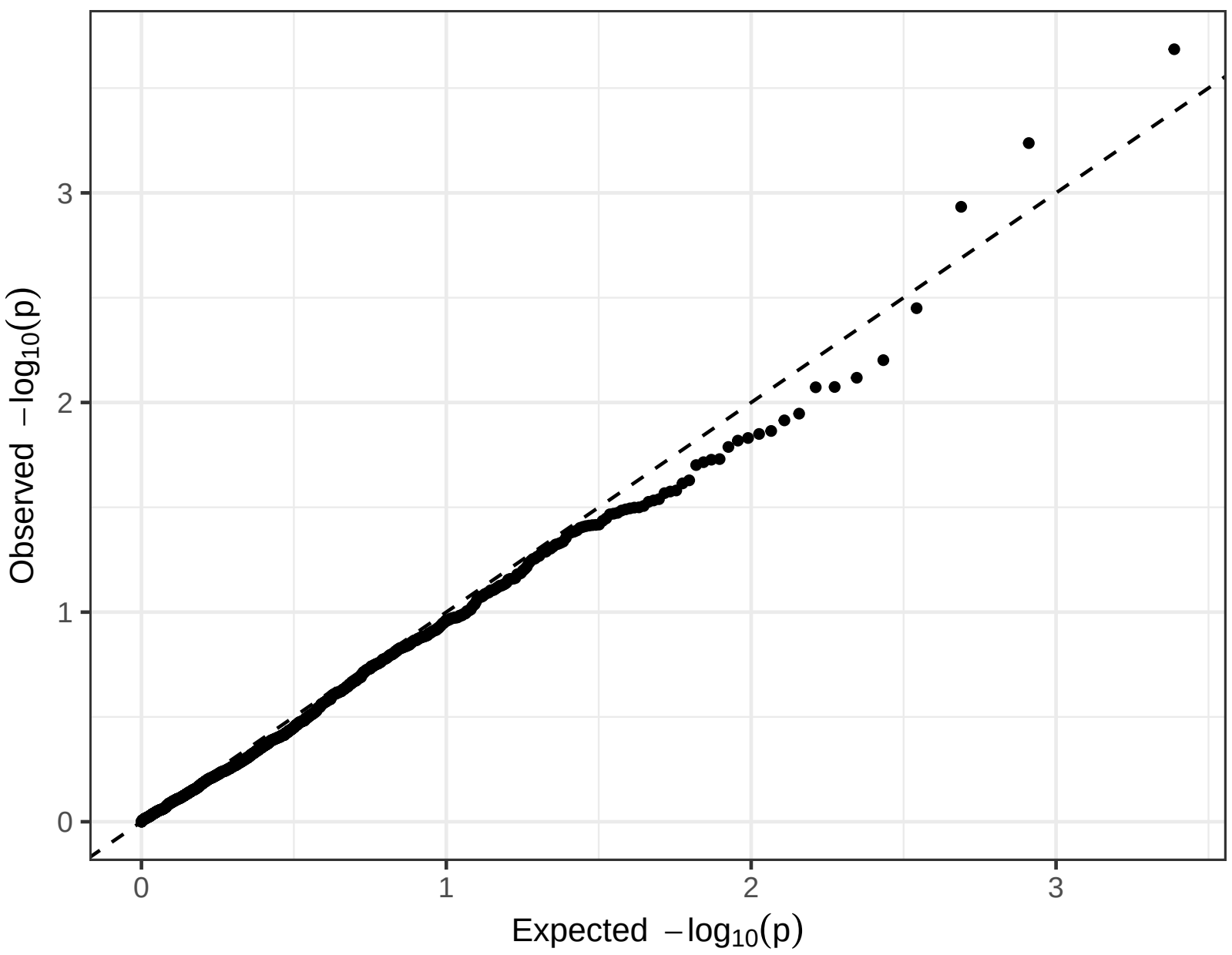

Family_XIII_UCG.001

Cecum | Liver | . = 1.06

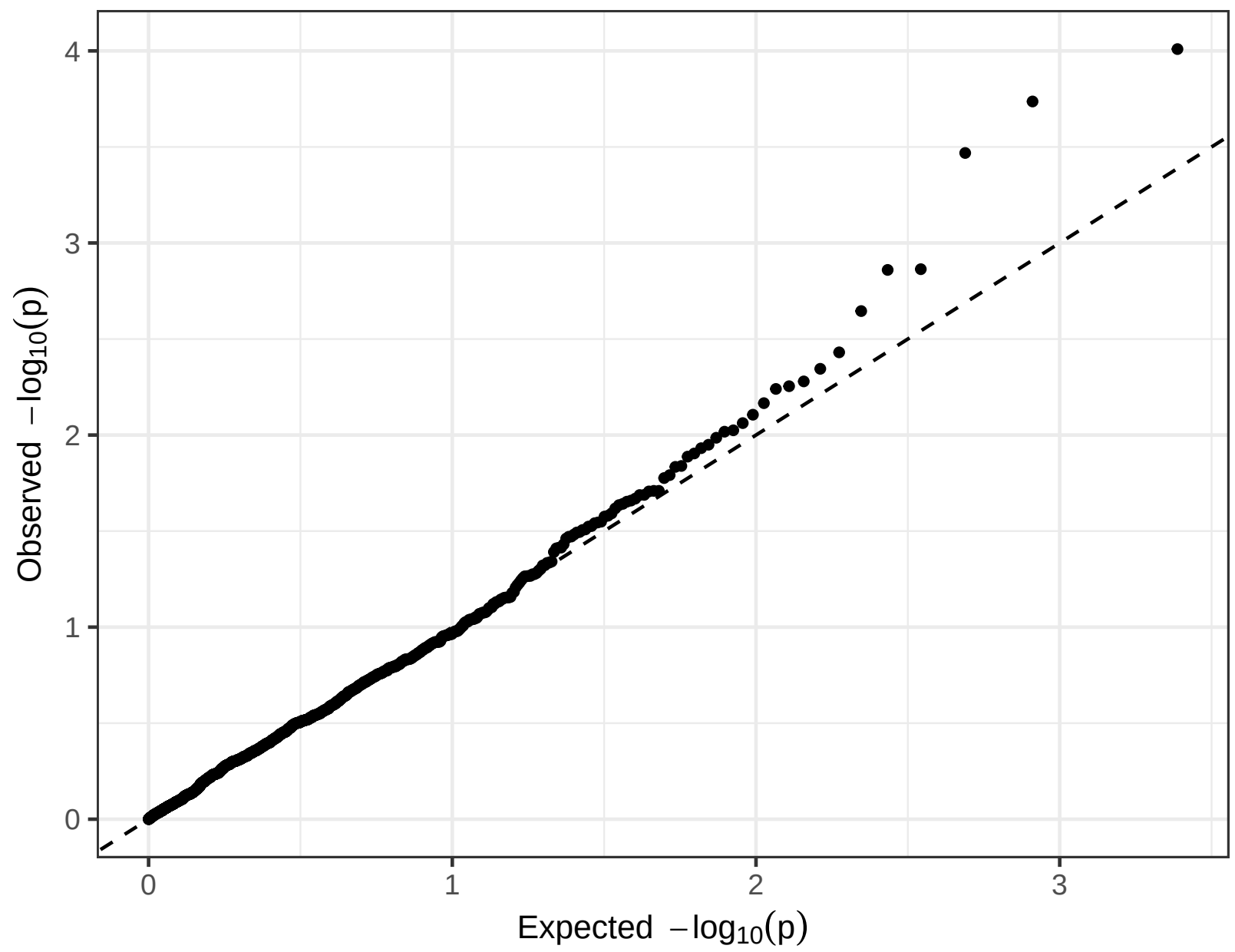

FD2005

Cecum | Liver | . = 0.971

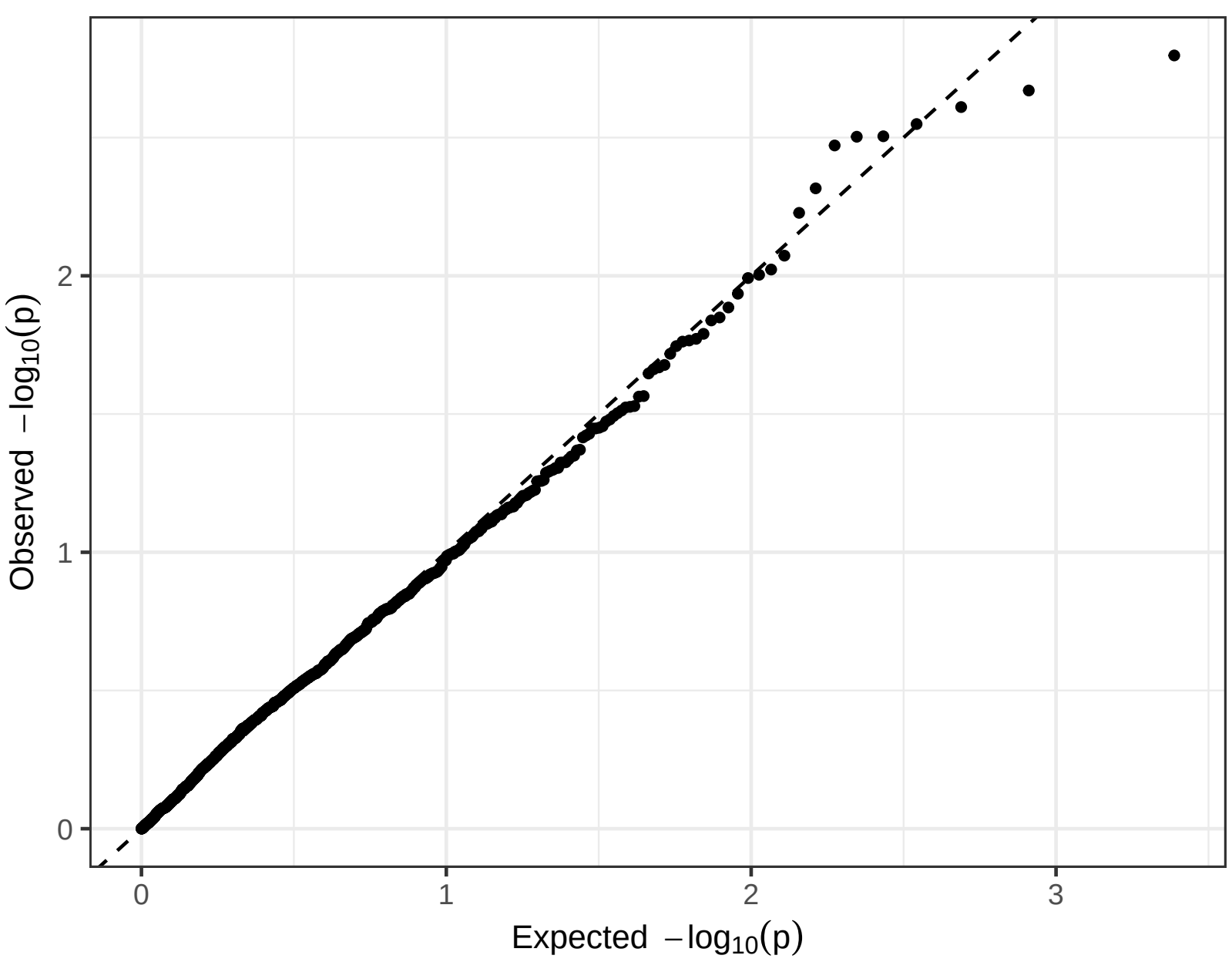

Fibrobacter

Cecum | Liver | . = 0.903

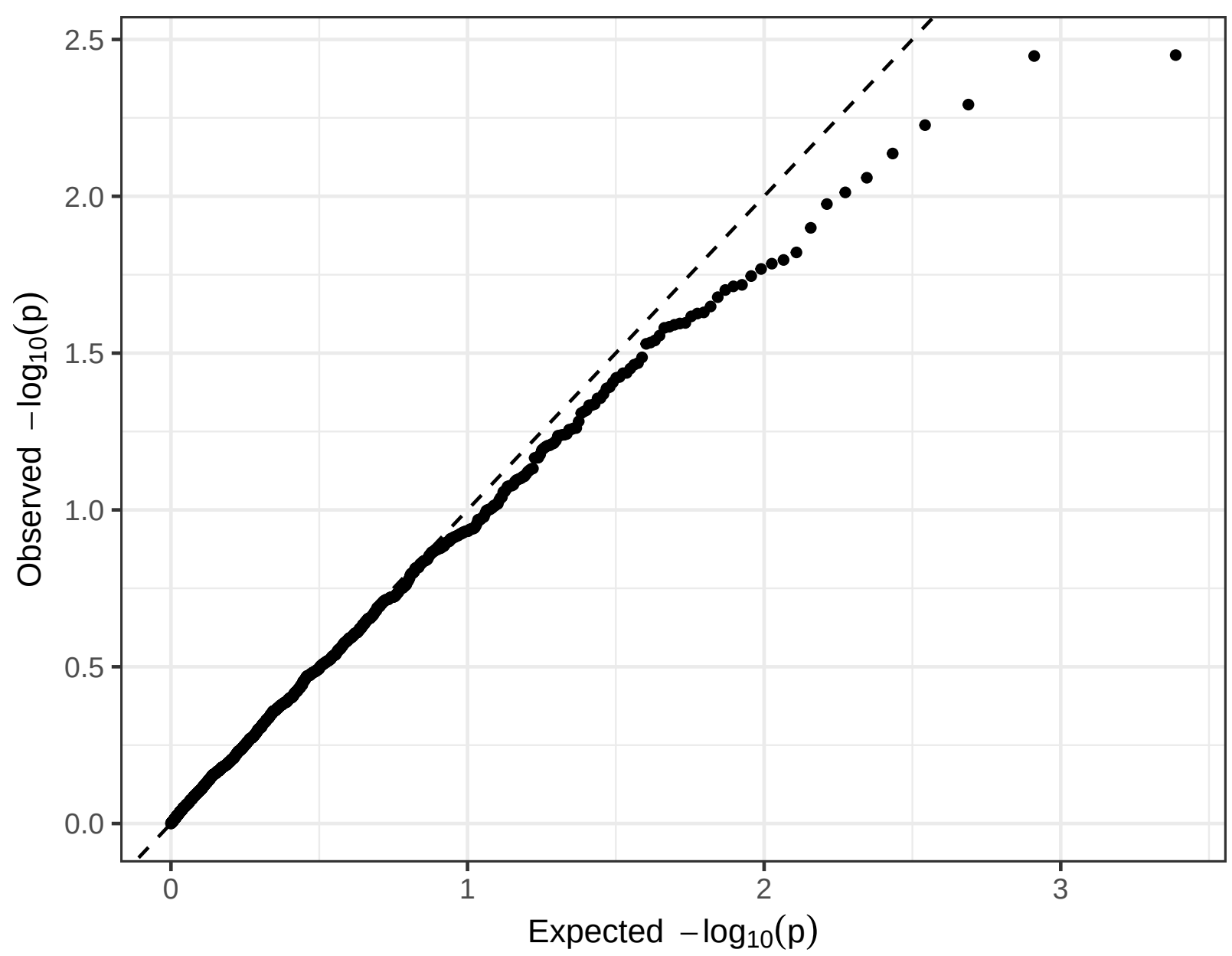

Fournierella

Cecum | Liver | . = 0.9

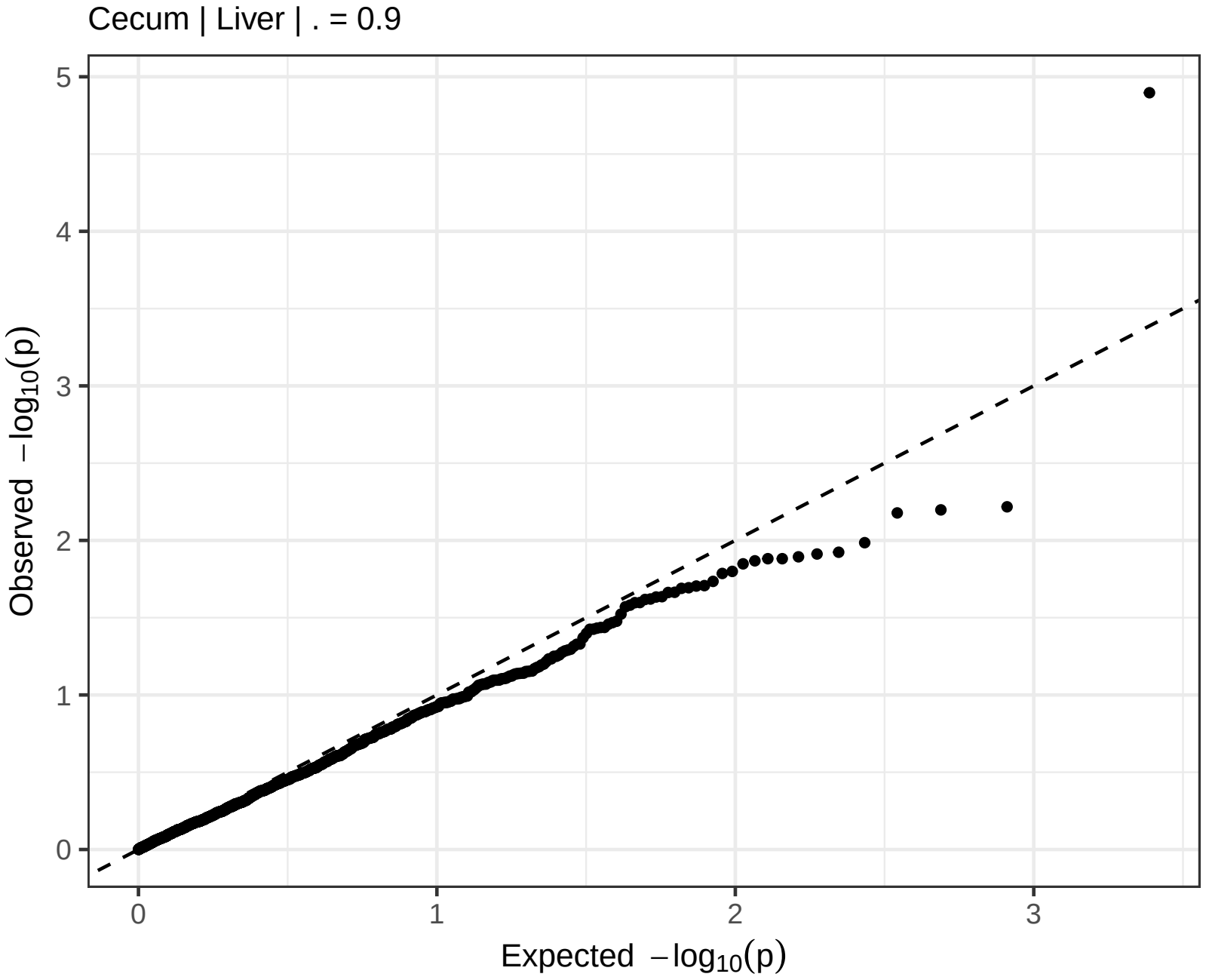

Frisingicoccus

Cecum | Liver | . = 0.856

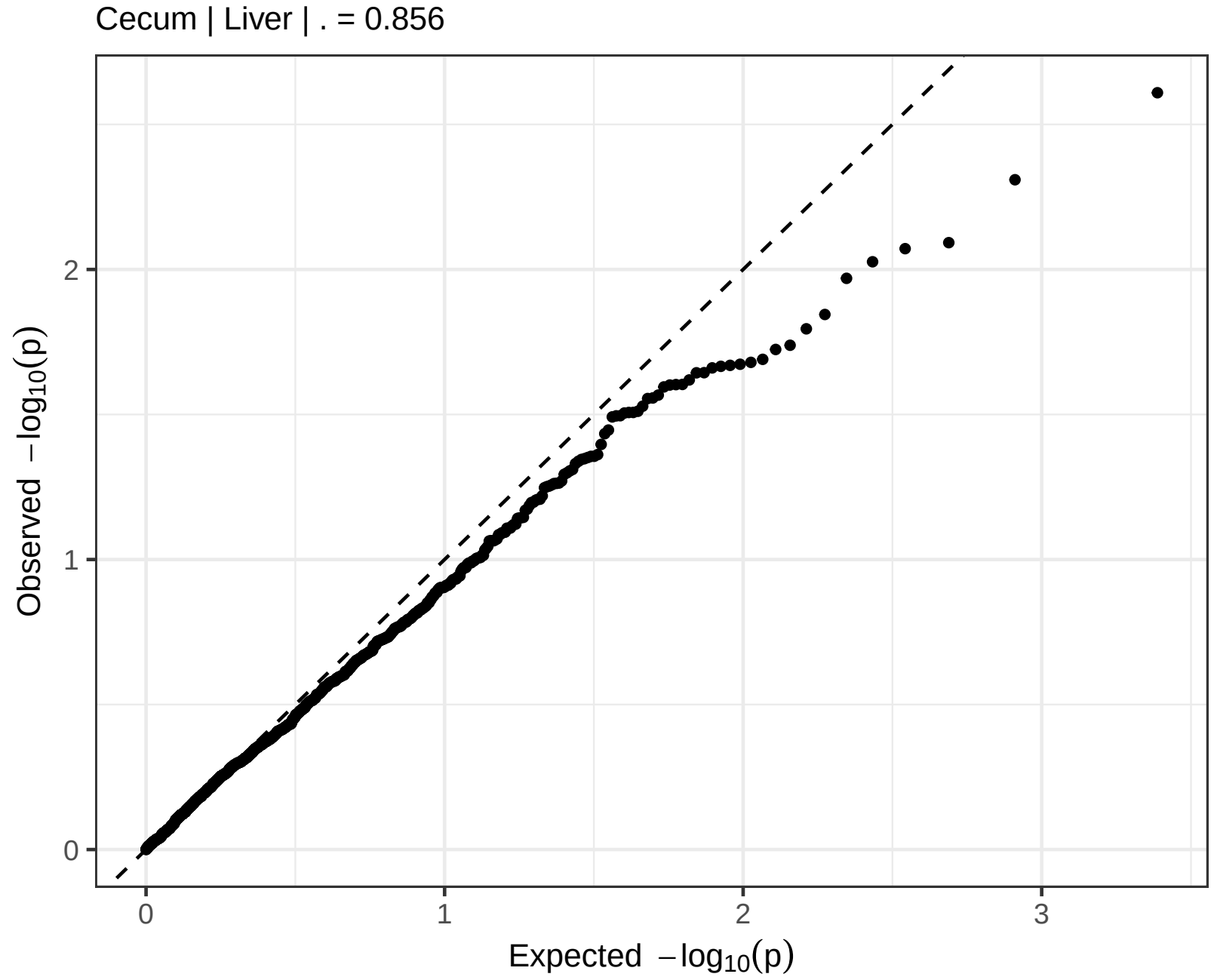

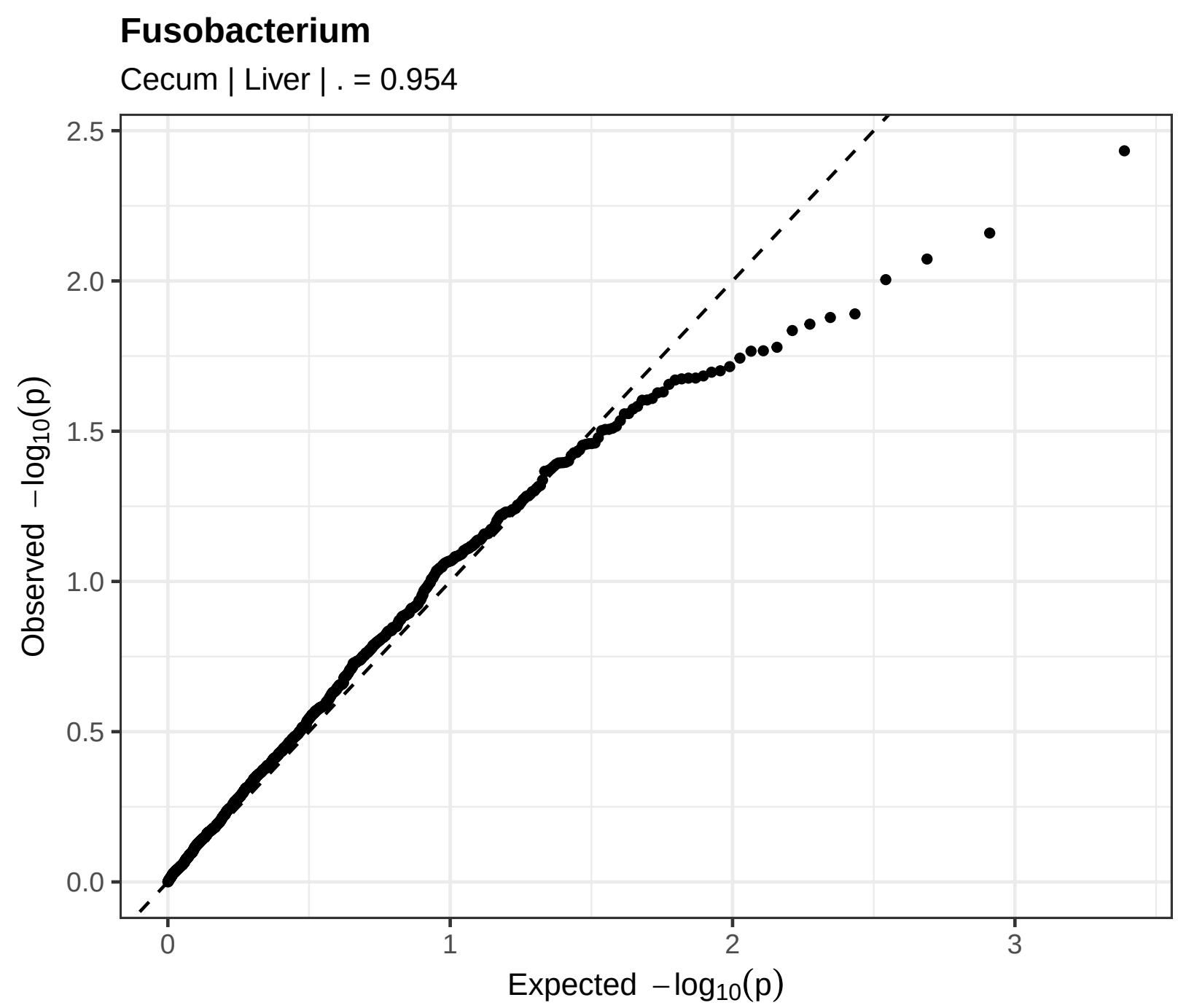
Fusobacterium
Cecum | Liver | . = 0.954
Observed $-\log_{10}(p)$
Expected $-\log_{10}(p)$

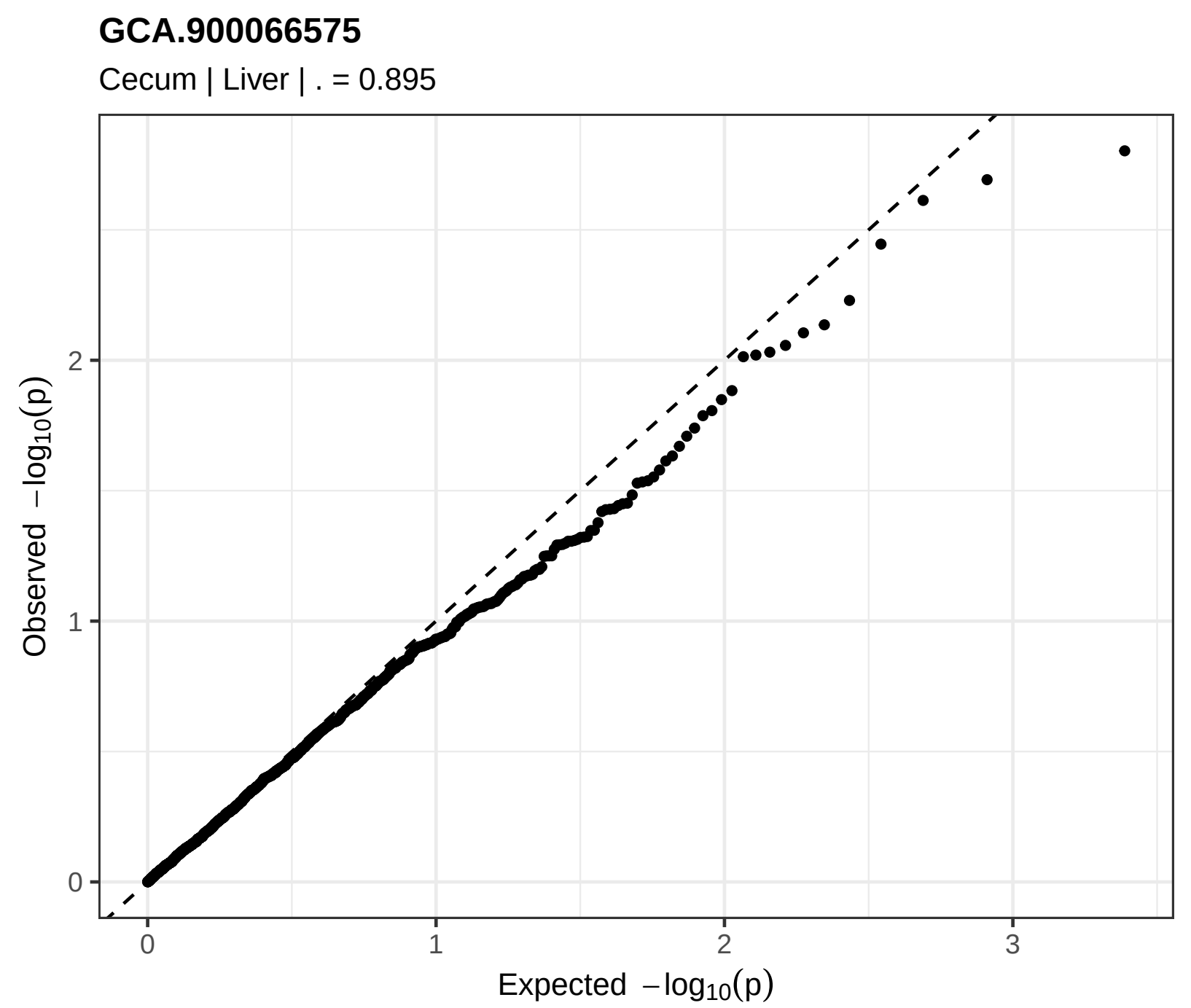
GCA.900066575
Cecum | Liver | . = 0.895
Observed $-\log_{10}(p)$
Expected $-\log_{10}(p)$

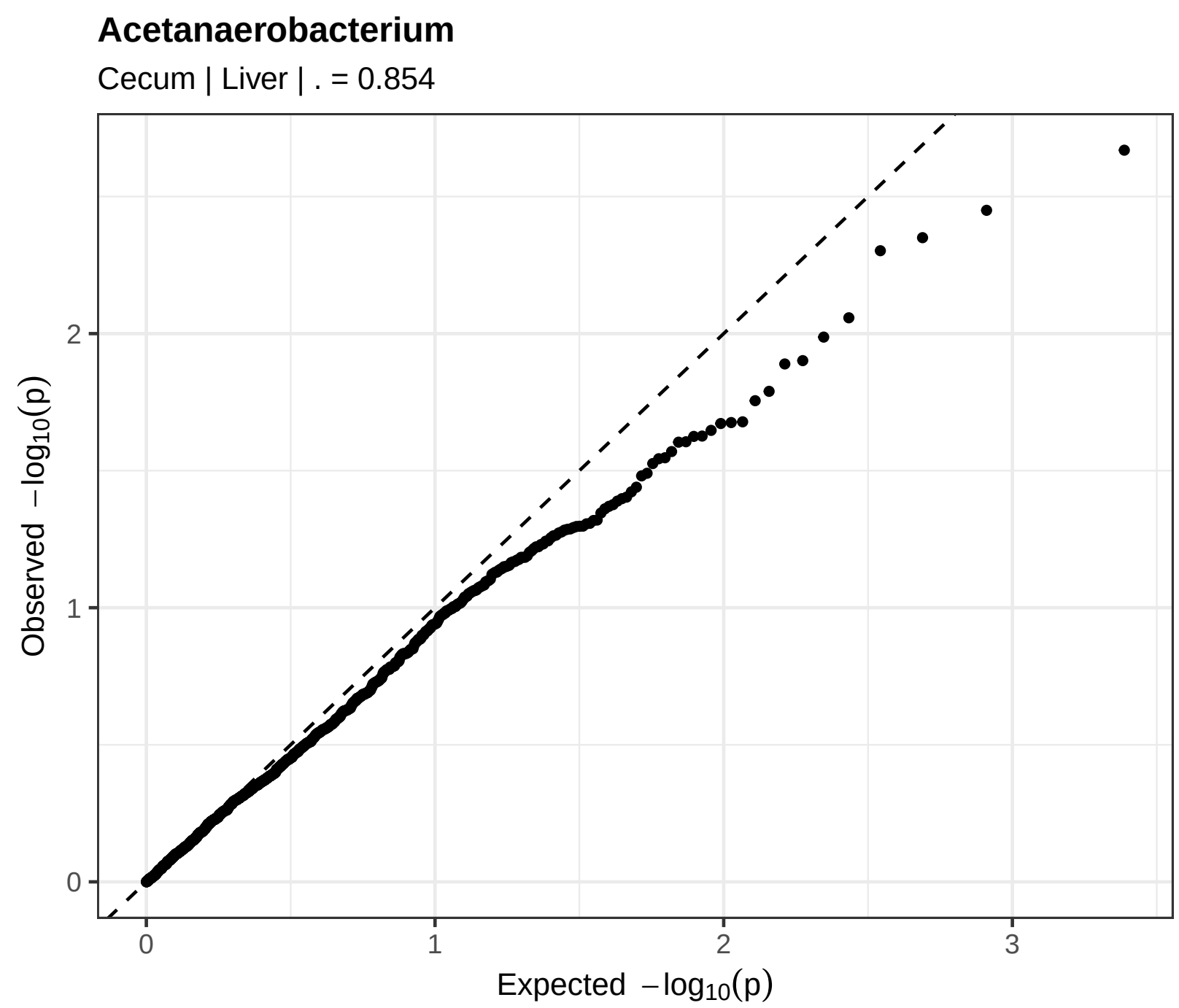
Acetanaerobacterium
Cecum | Liver | . = 0.854
Observed $-\log_{10}(p)$
Expected $-\log_{10}(p)$

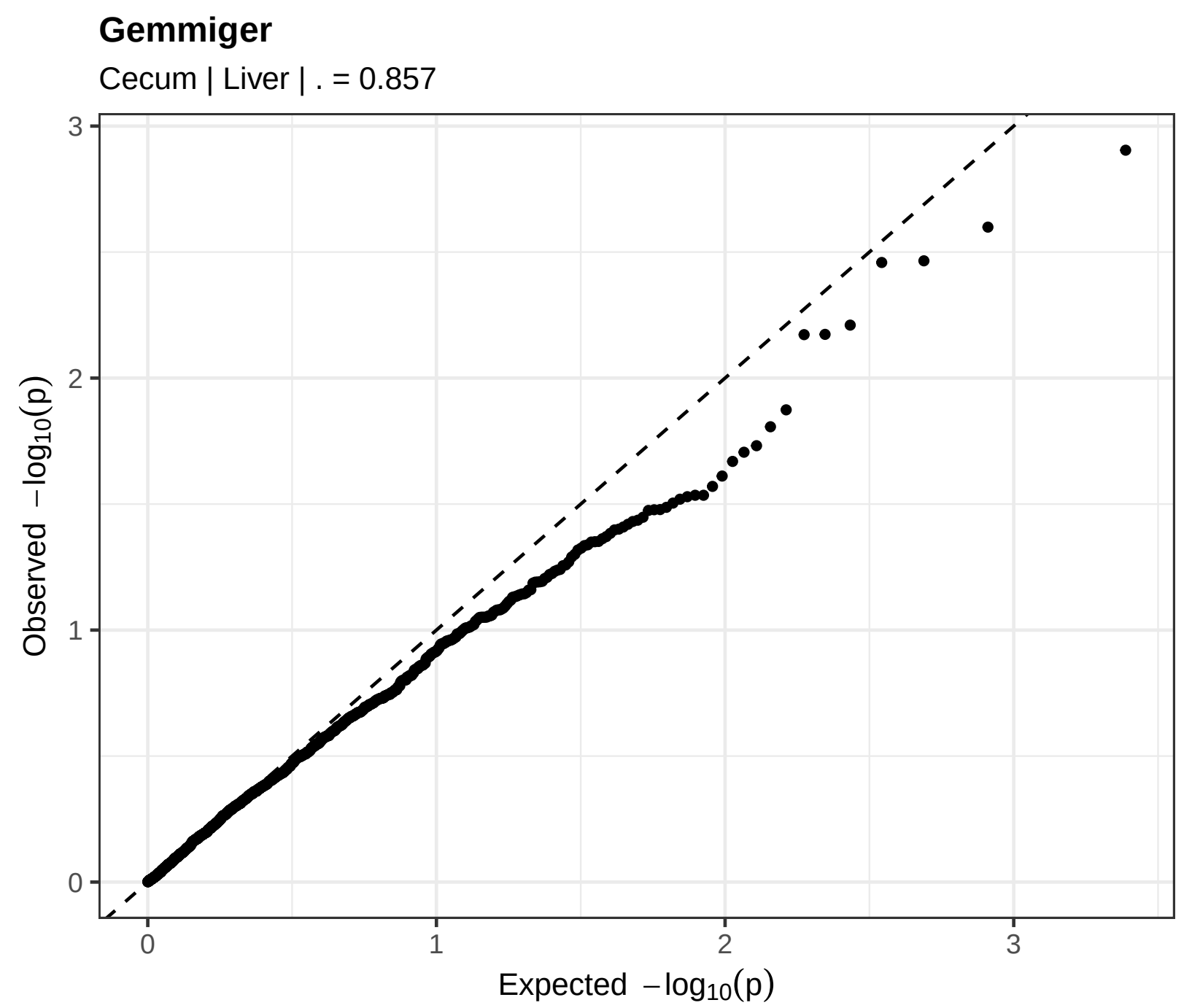
Gemmiger
Cecum | Liver | . = 0.857
Observed $-\log_{10}(p)$
Expected $-\log_{10}(p)$

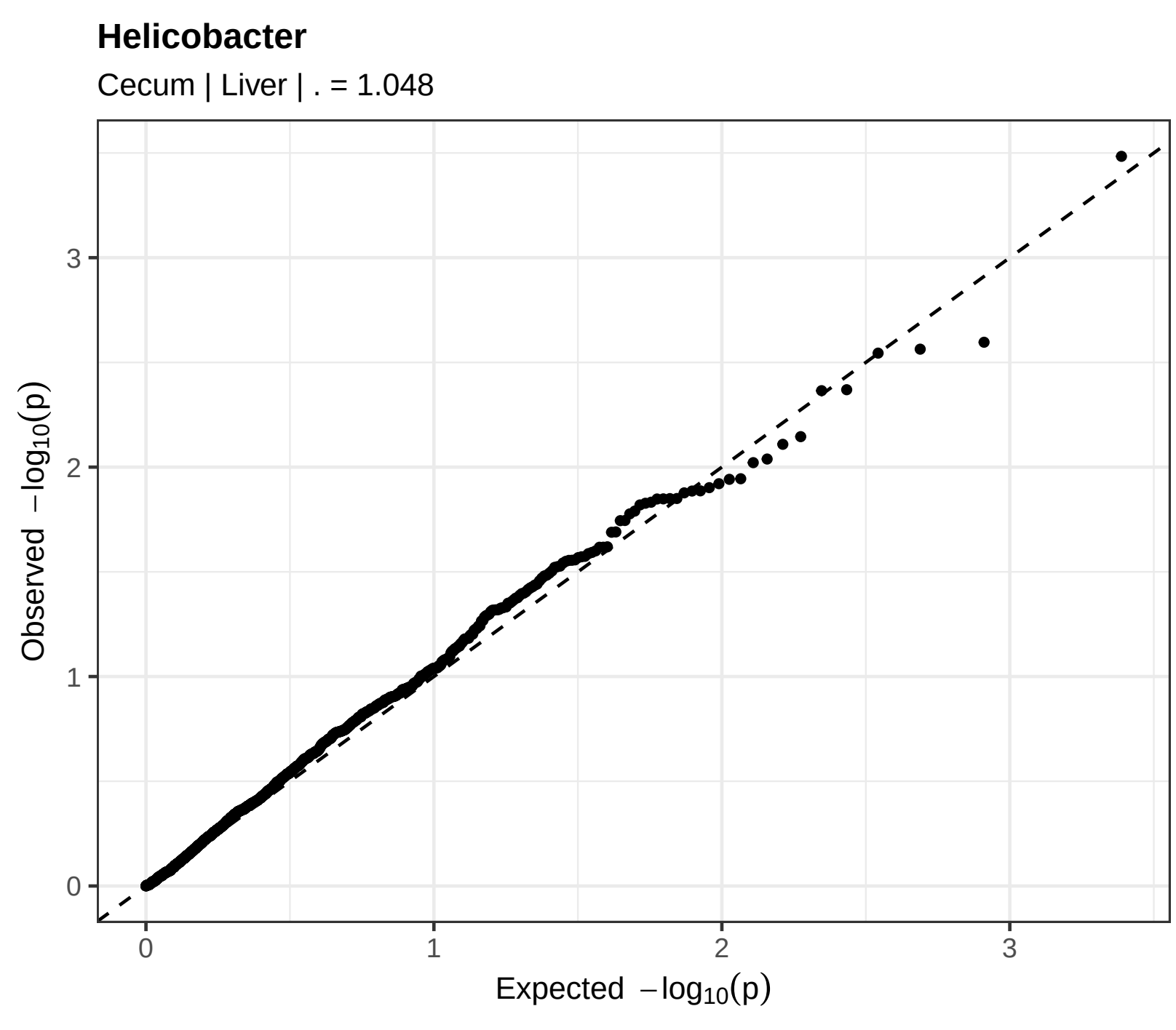
Helicobacter
Cecum | Liver | . = 1.048
Observed $-\log_{10}(p)$
Expected $-\log_{10}(p)$

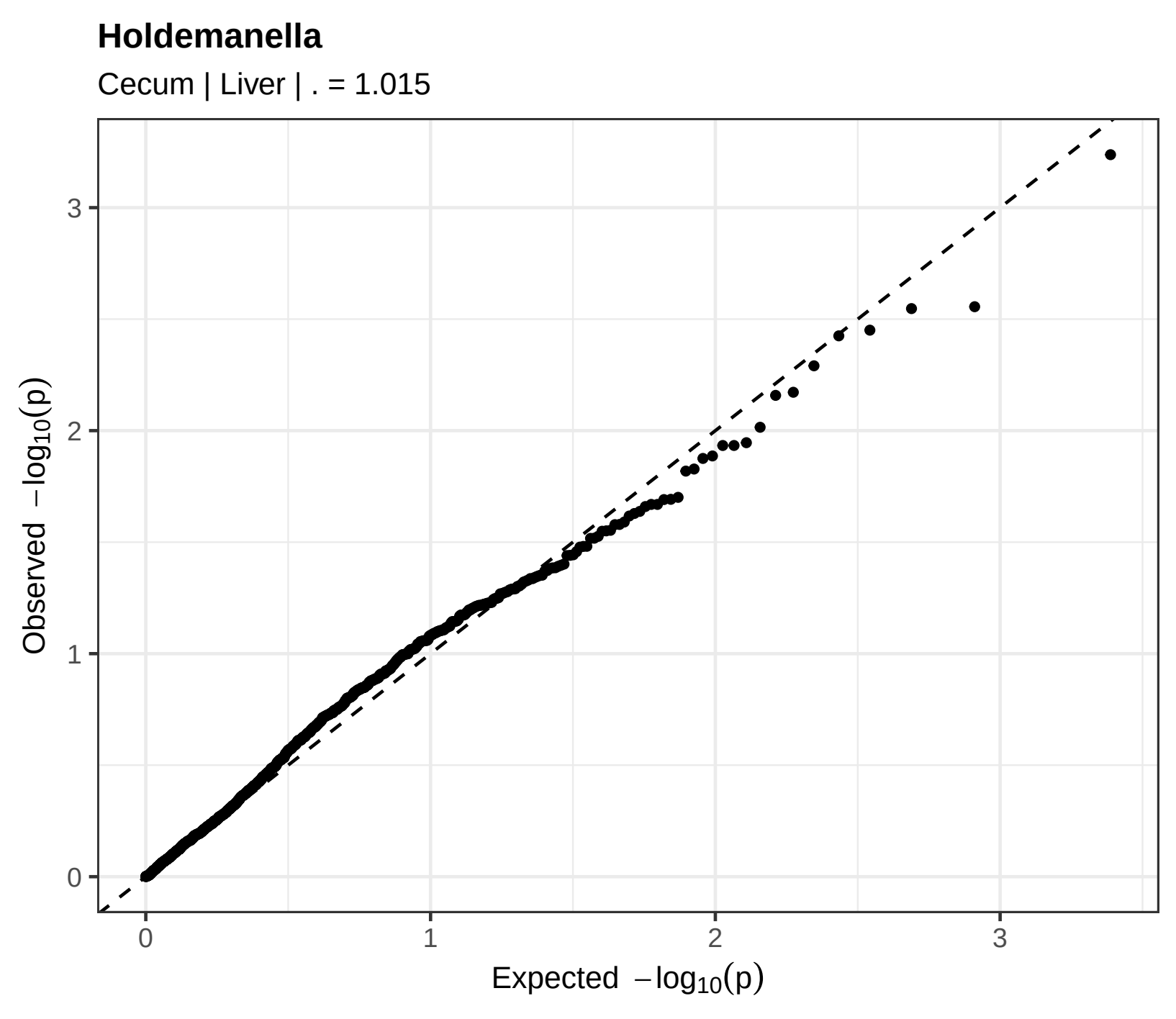
Holdemanella
Cecum | Liver | . = 1.015
Observed $-\log_{10}(p)$
Expected $-\log_{10}(p)$

horsej.a03

Cecum | Liver | . = 0.913

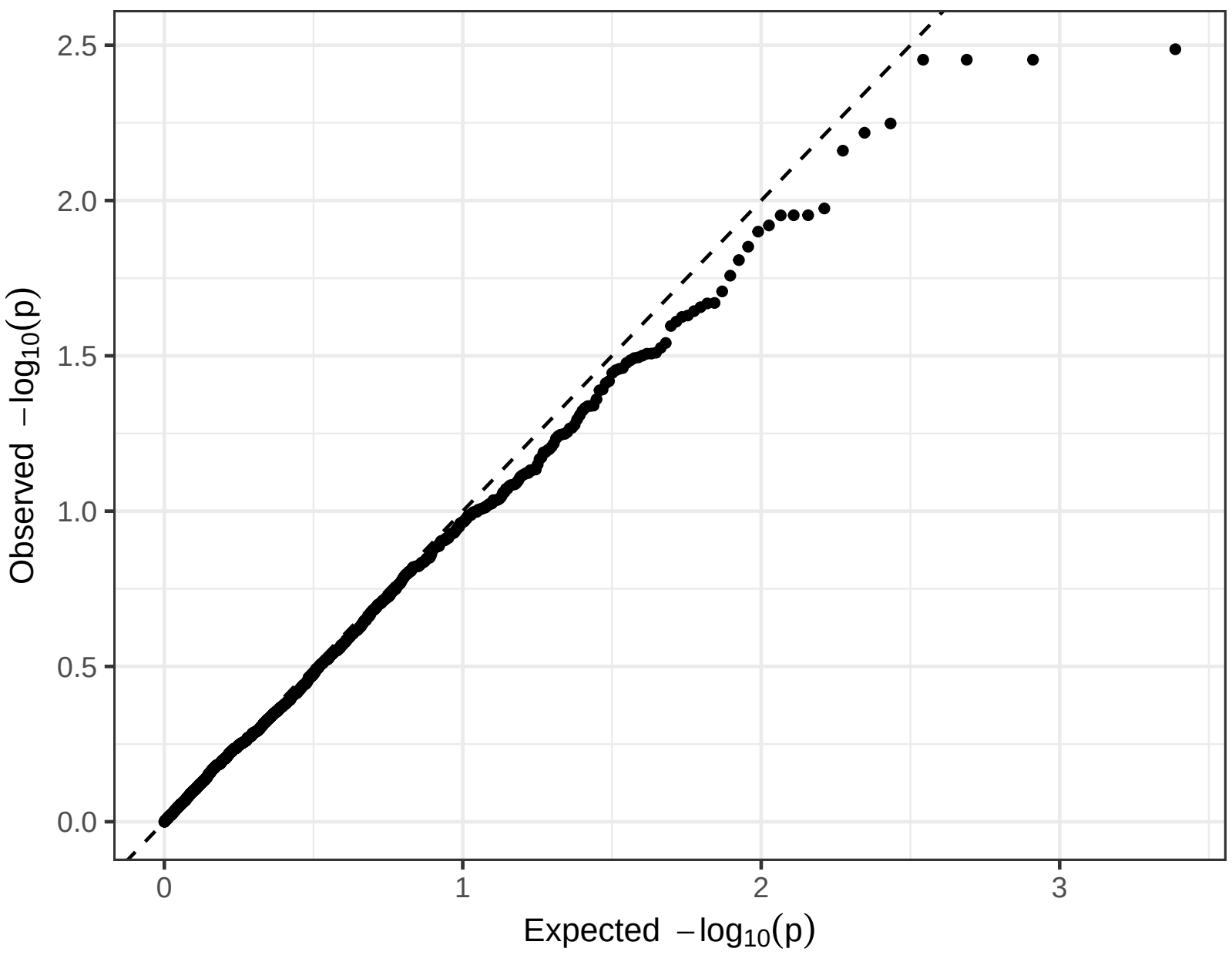

HT002

Cecum | Liver | . = 0.977

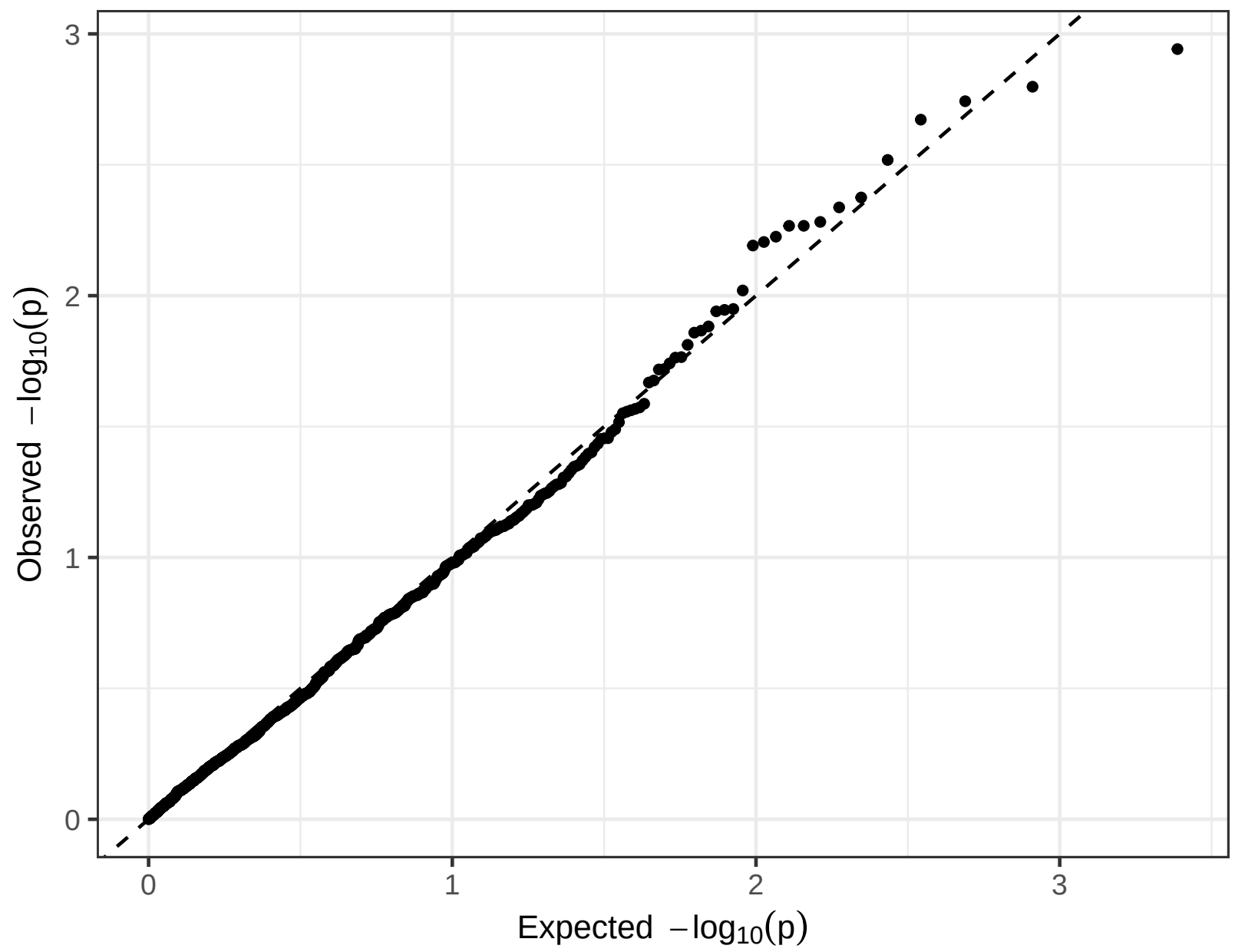

Hydrogenoanaerobacterium

Cecum | Liver | . = 0.964

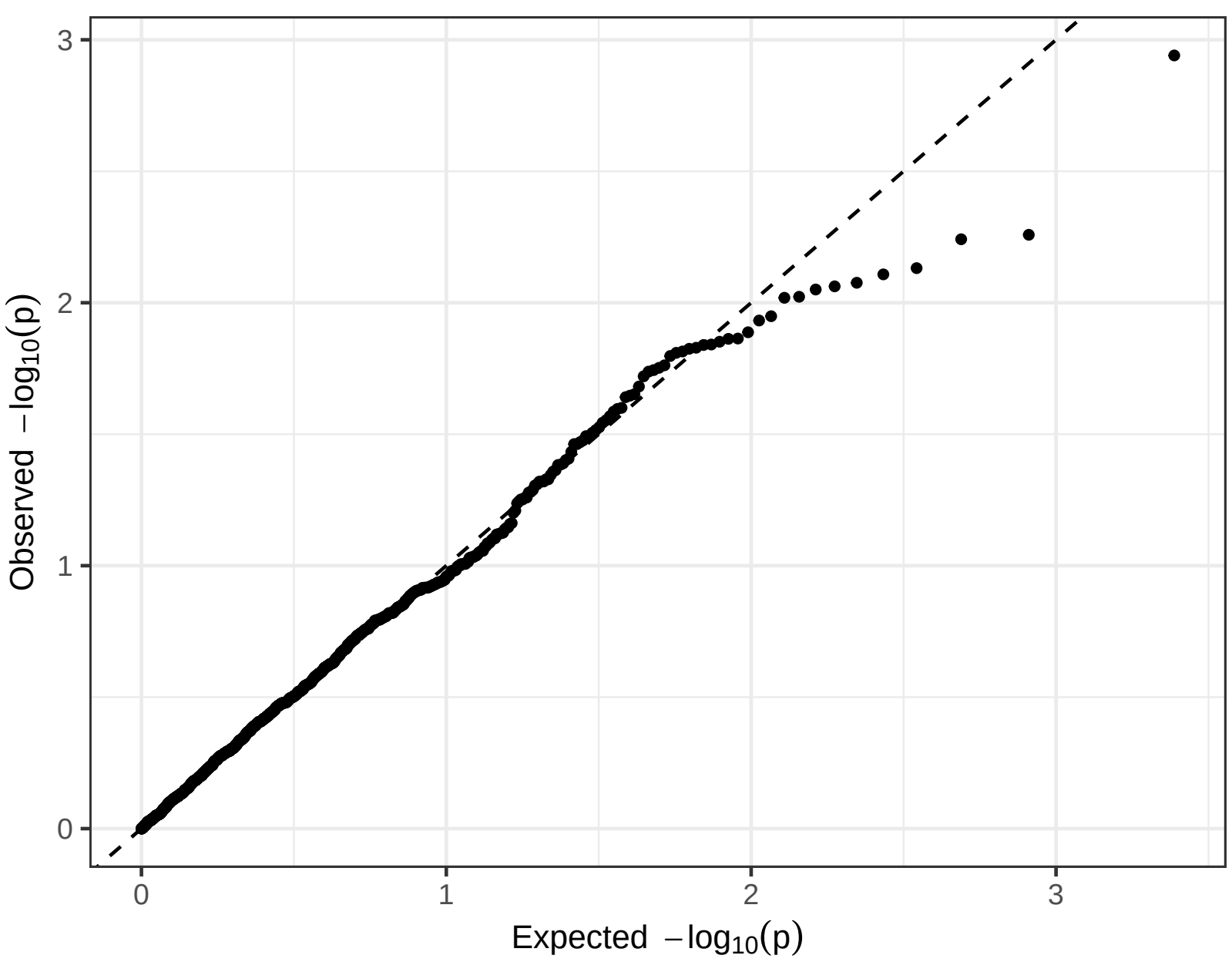

Hydromonas

Cecum | Liver | . = 0.895

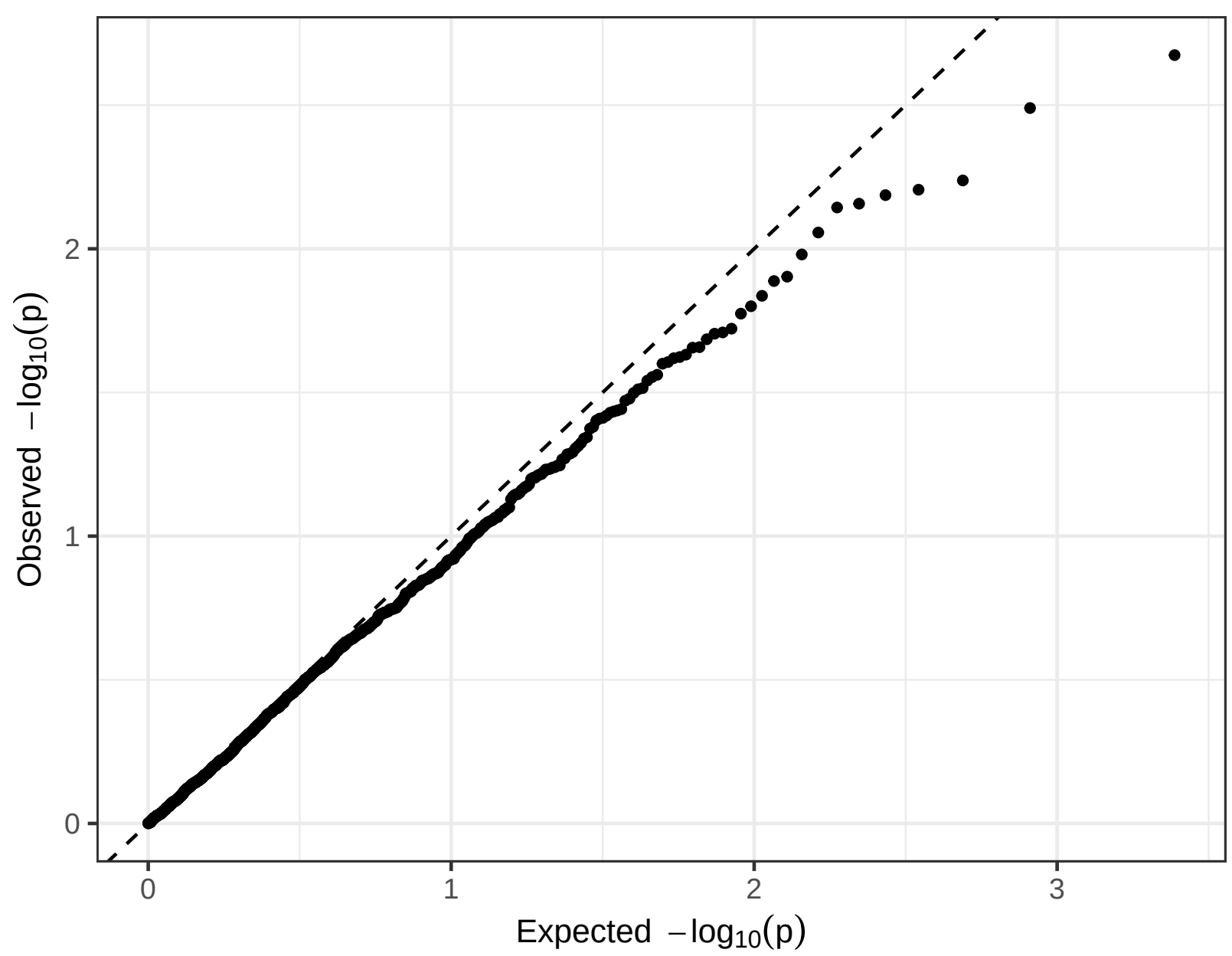

Incertae_Sedis

Cecum | Liver | . = 0.905

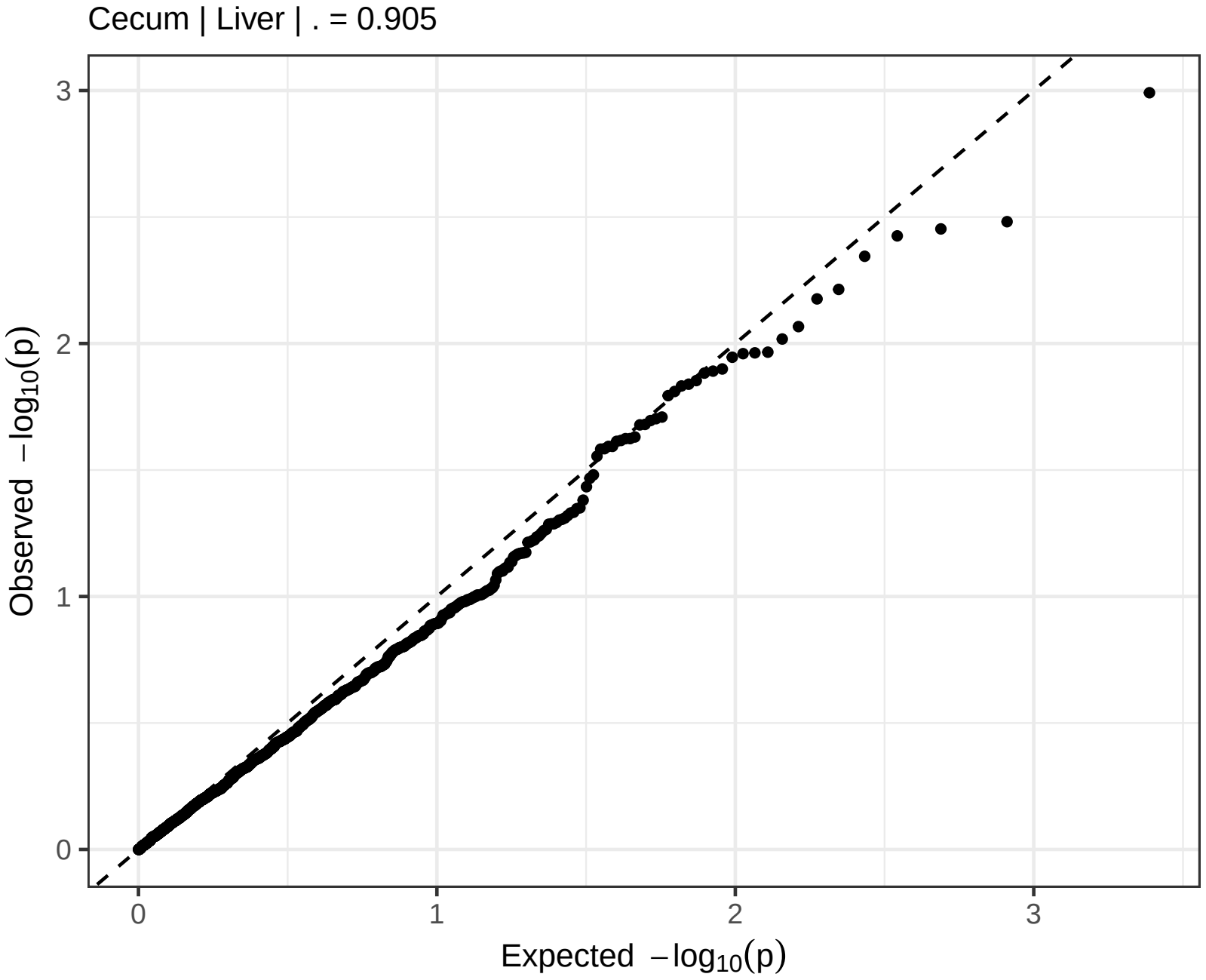

Intestinibacter

Cecum | Liver | . = 0.895

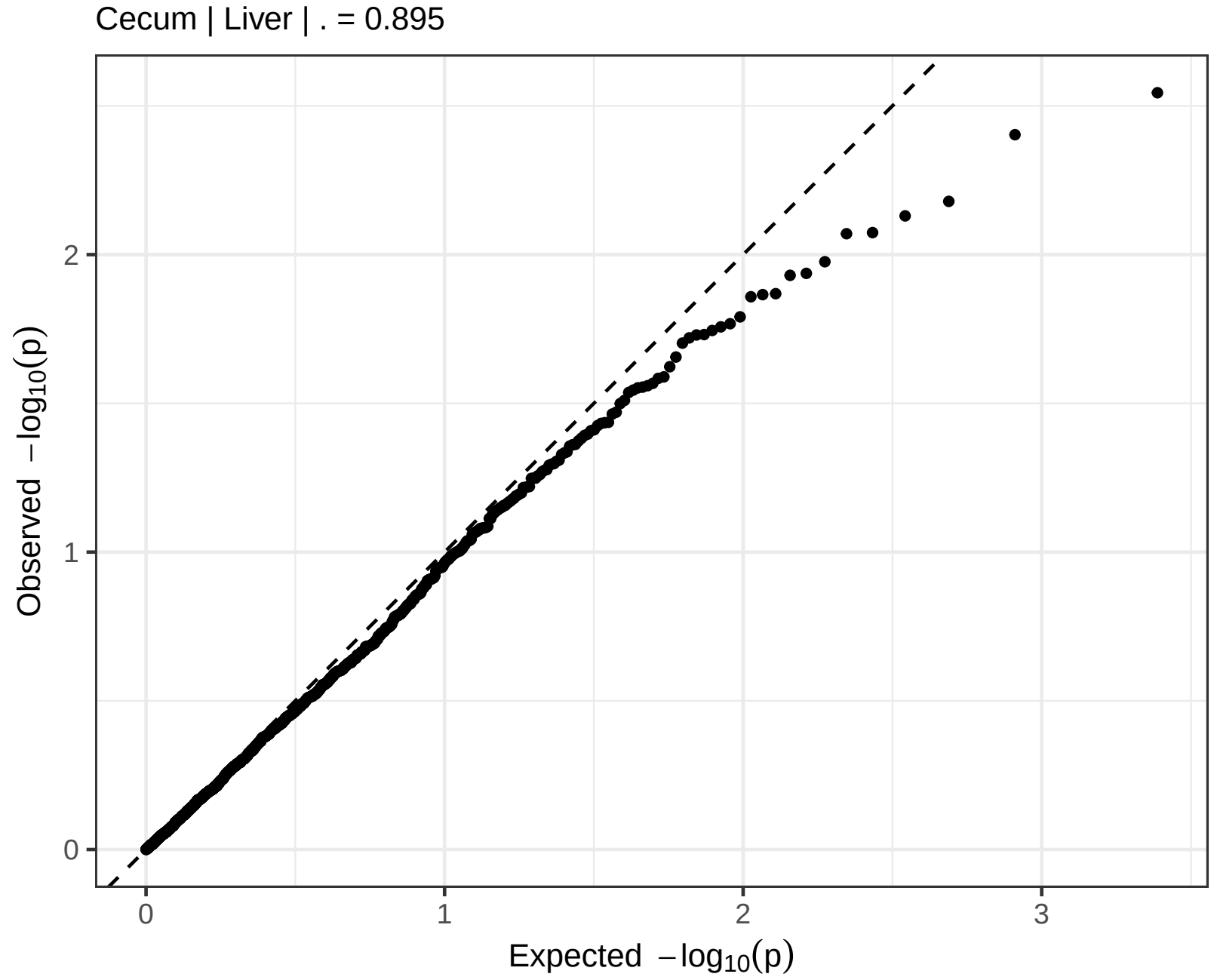

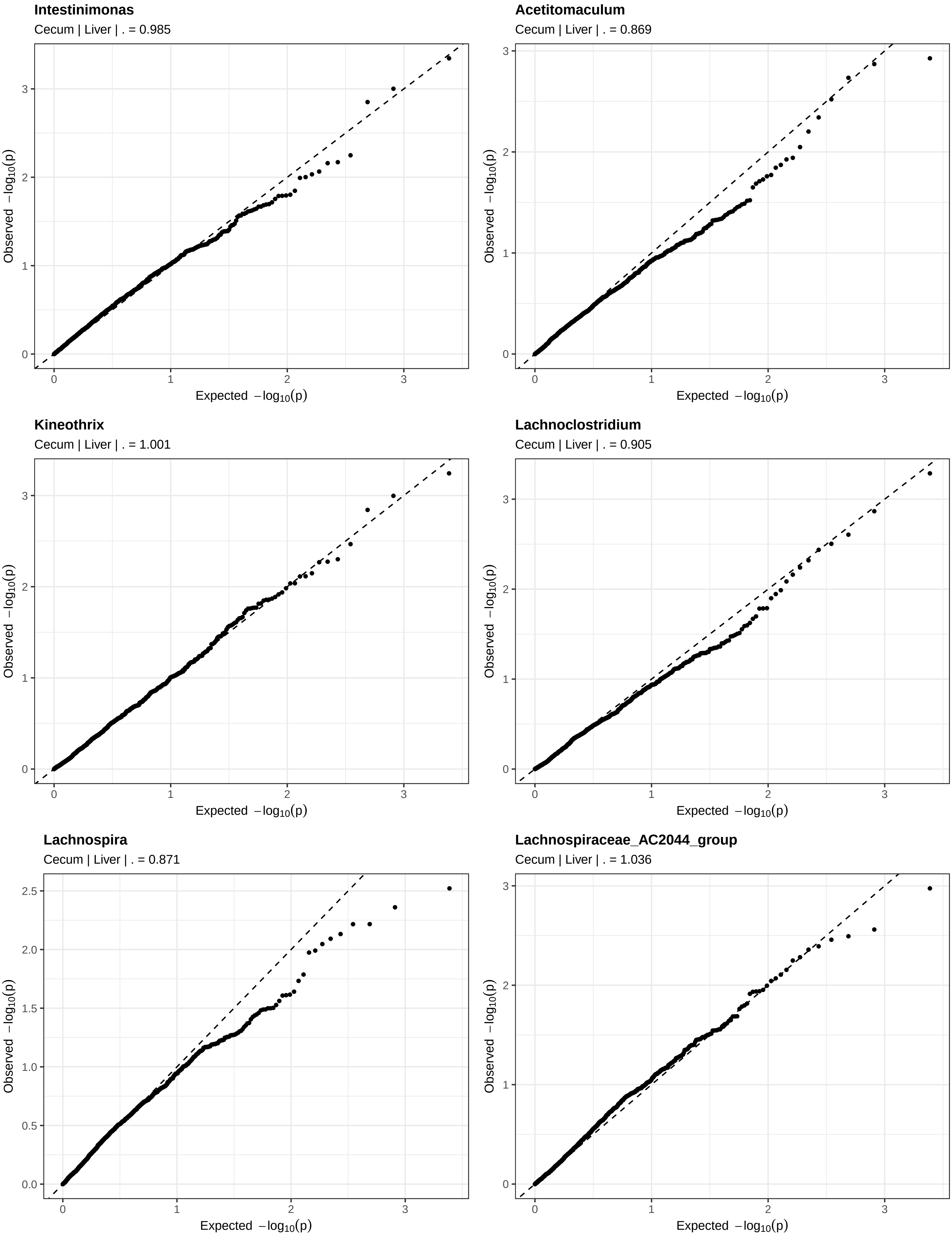
Intestinimonas
Cecum | Liver | . = 0.985
Acetitomaculum
Cecum | Liver | . = 0.869
Kineothrix
Cecum | Liver | . = 1.001
Lachnoclostridium
Cecum | Liver | . = 0.905
Lachnospira
Cecum | Liver | . = 0.871
Lachnospiraceae_AC2044_group
Cecum | Liver | . = 1.036
Observed $-\log_{10}(p)$
Expected $-\log_{10}(p)$

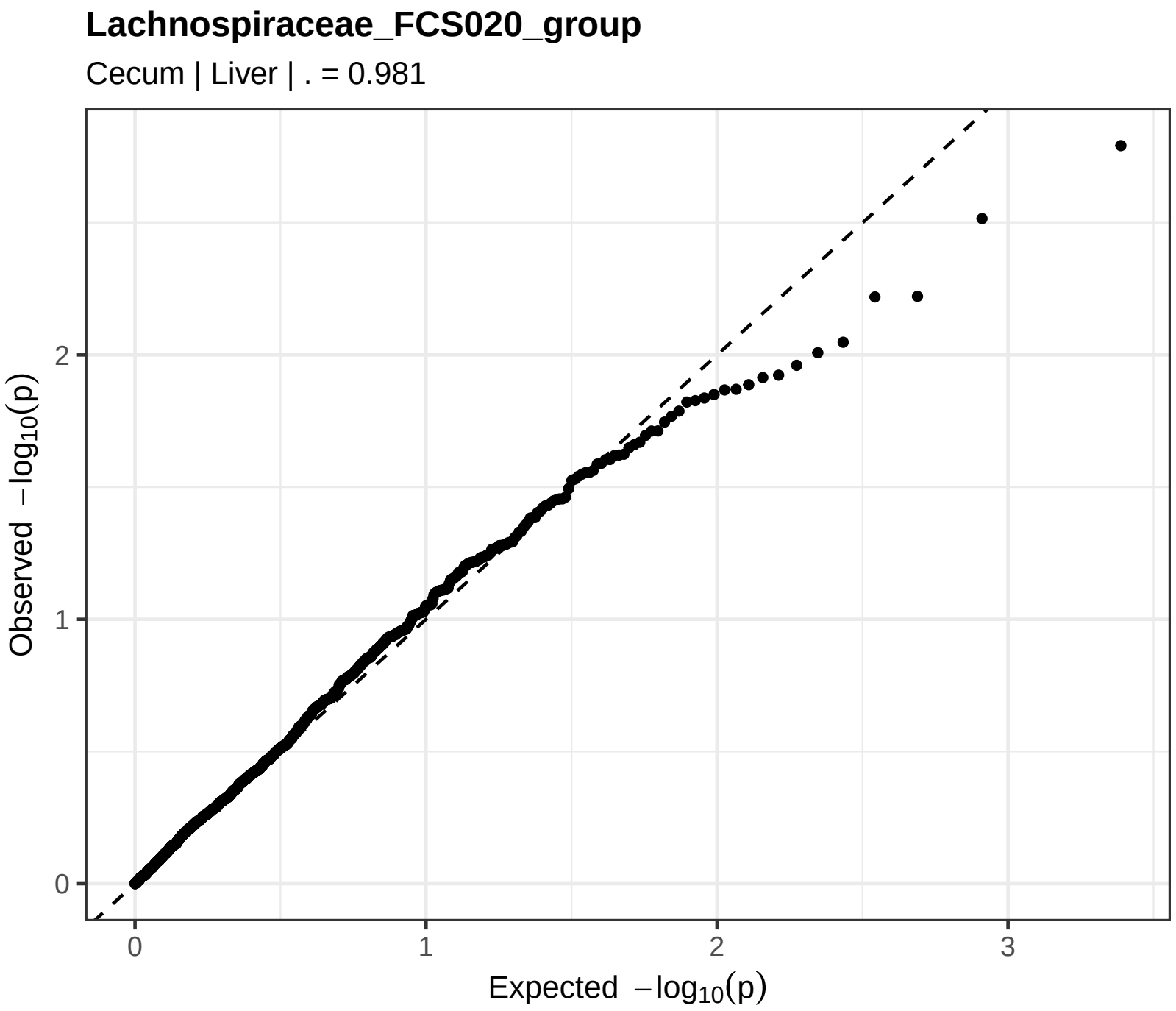
Lachnospiraceae_FCS020_group
Cecum | Liver | . = 0.981
Observed $-\log_{10}(p)$
Expected $-\log_{10}(p)$

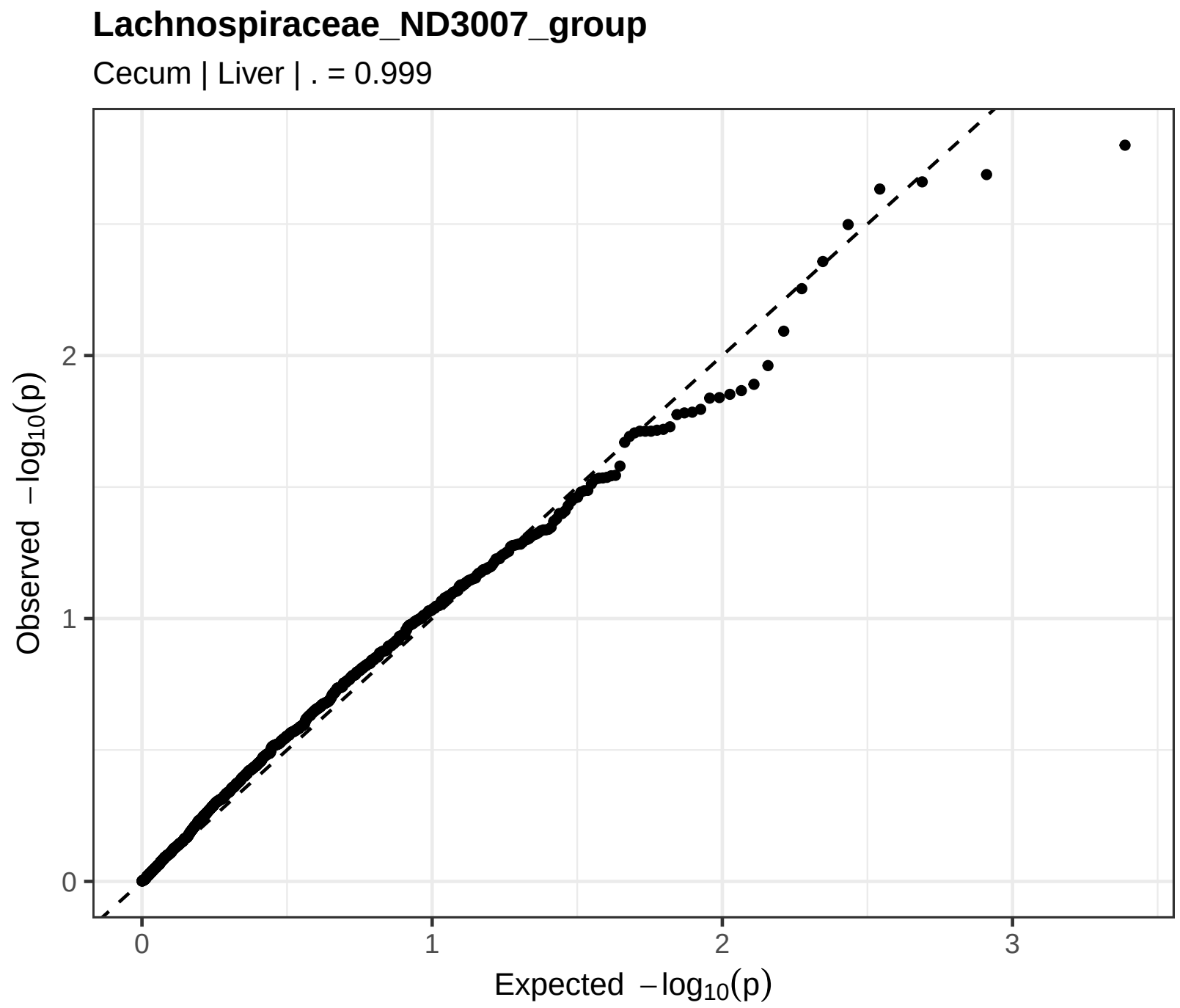
Lachnospiraceae_ND3007_group
Cecum | Liver | . = 0.999
Observed $-\log_{10}(p)$
Expected $-\log_{10}(p)$

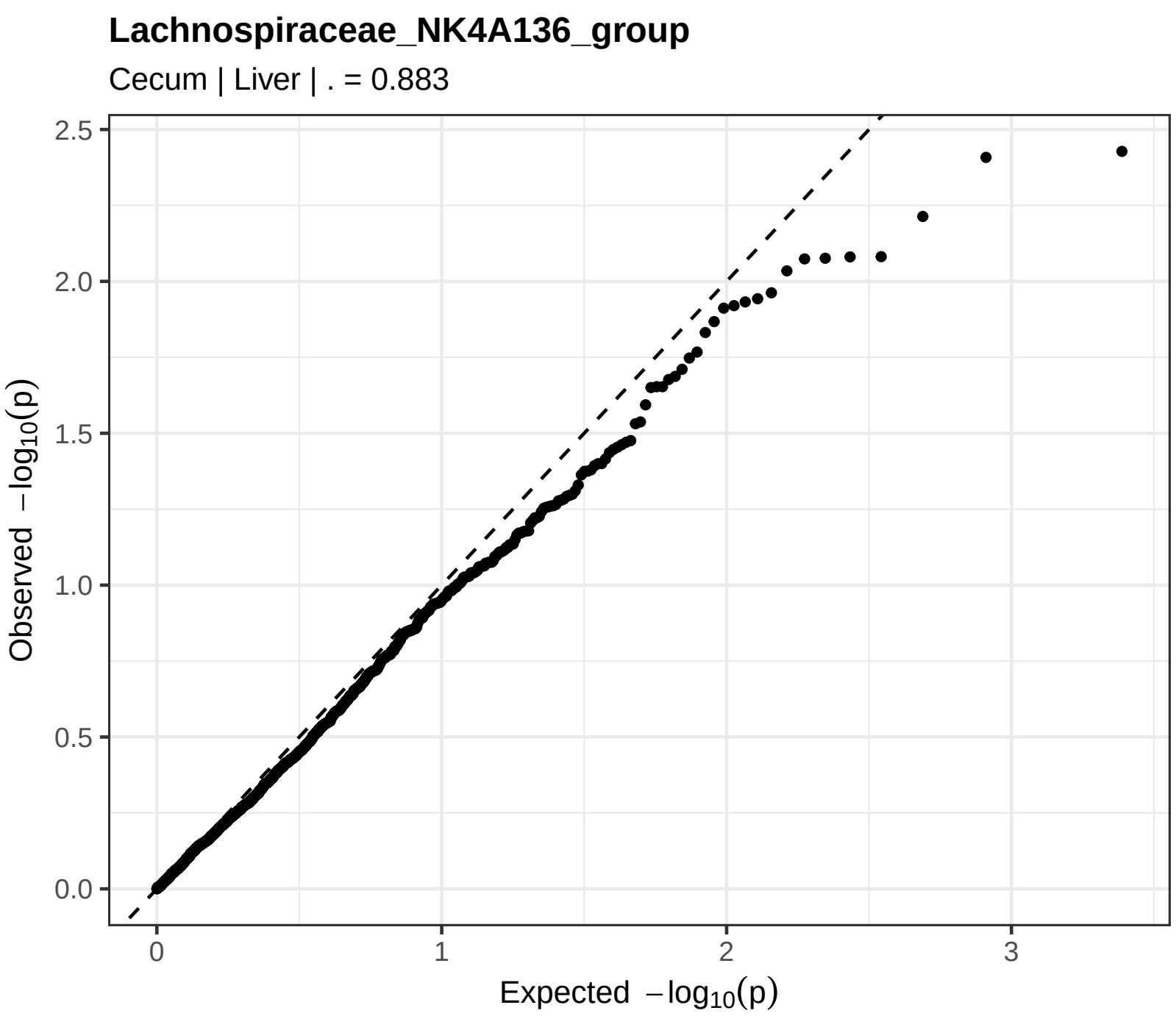
Lachnospiraceae_NK4A136_group
Cecum | Liver | . = 0.883
Observed $-\log_{10}(p)$
Expected $-\log_{10}(p)$

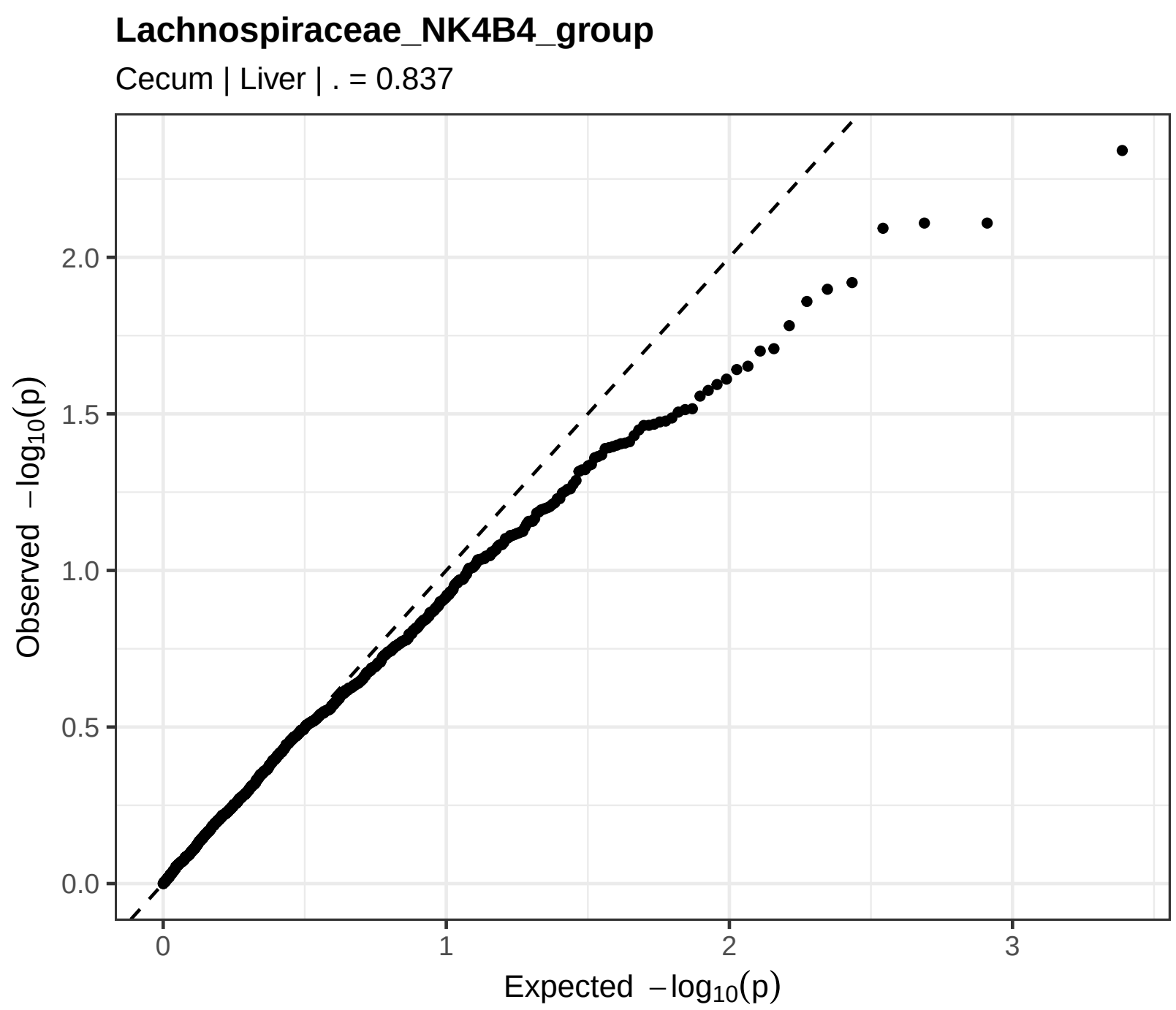
Lachnospiraceae_NK4B4_group
Cecum | Liver | . = 0.837
Observed $-\log_{10}(p)$
Expected $-\log_{10}(p)$

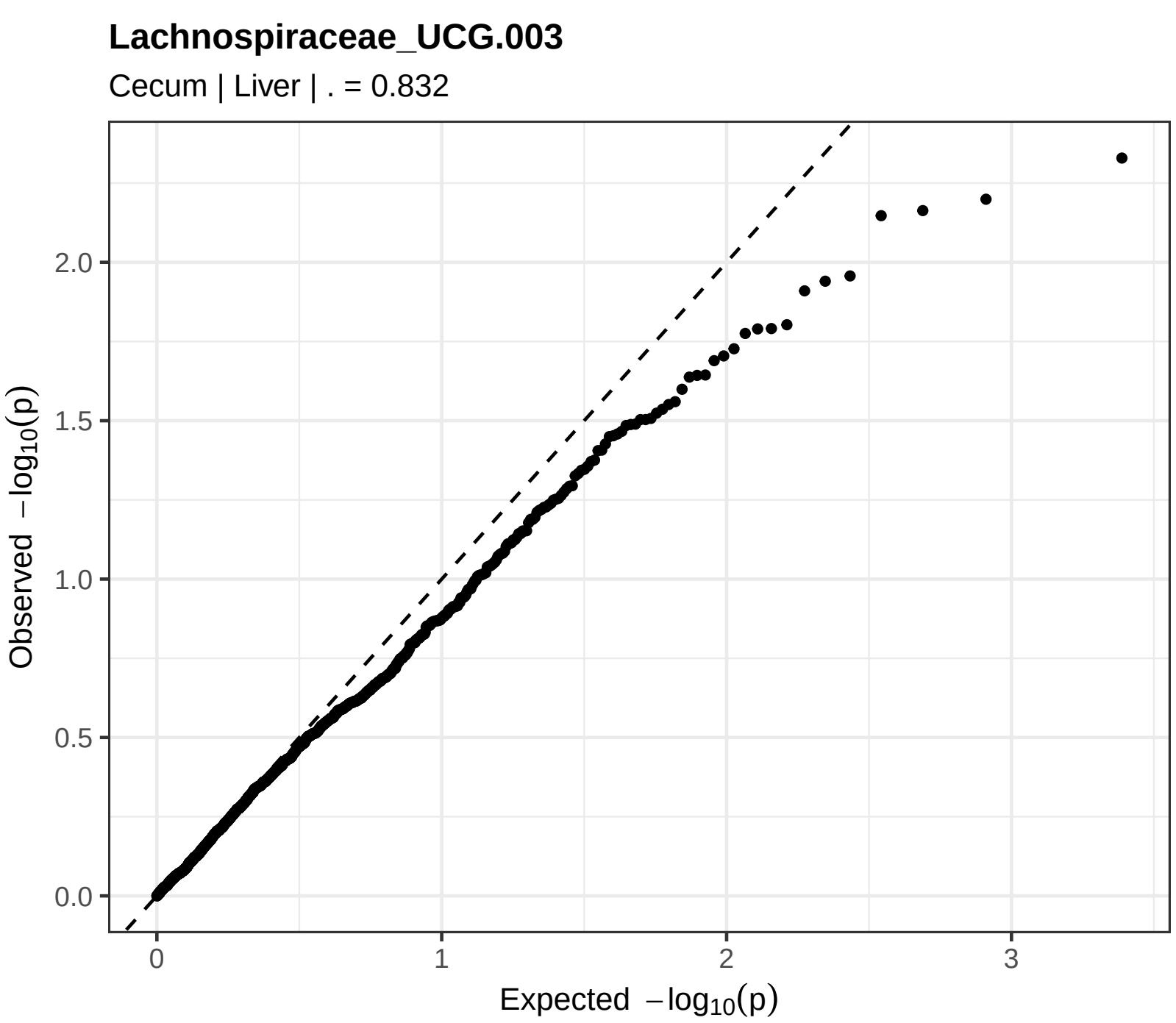
Lachnospiraceae_UCG.003
Cecum | Liver | . = 0.832
Observed $-\log_{10}(p)$
Expected $-\log_{10}(p)$

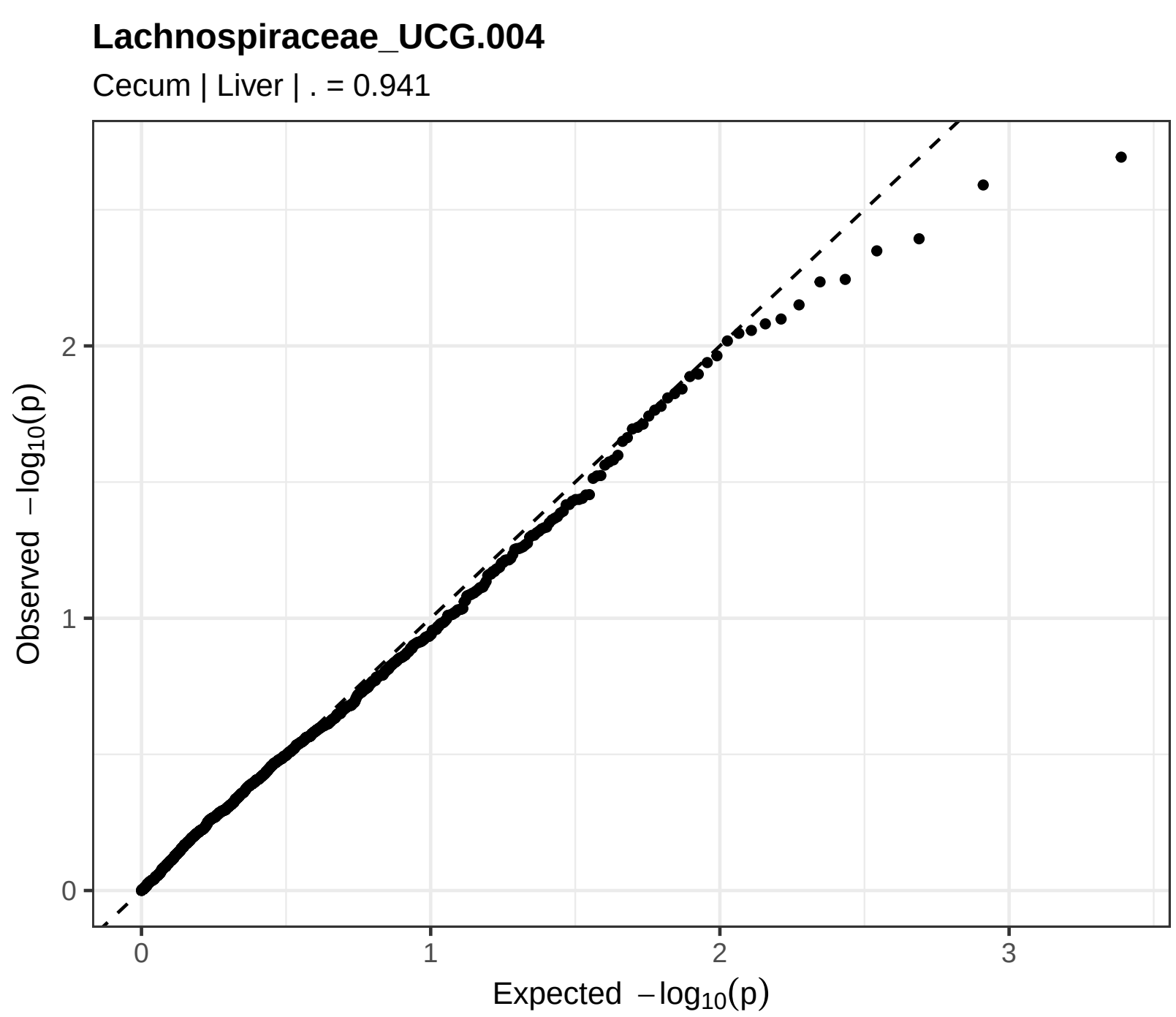
Lachnospiraceae_UCG.004
Cecum | Liver | . = 0.941
Observed $-\log_{10}(p)$
Expected $-\log_{10}(p)$

Acutalibacter

Cecum | Liver | . = 0.878

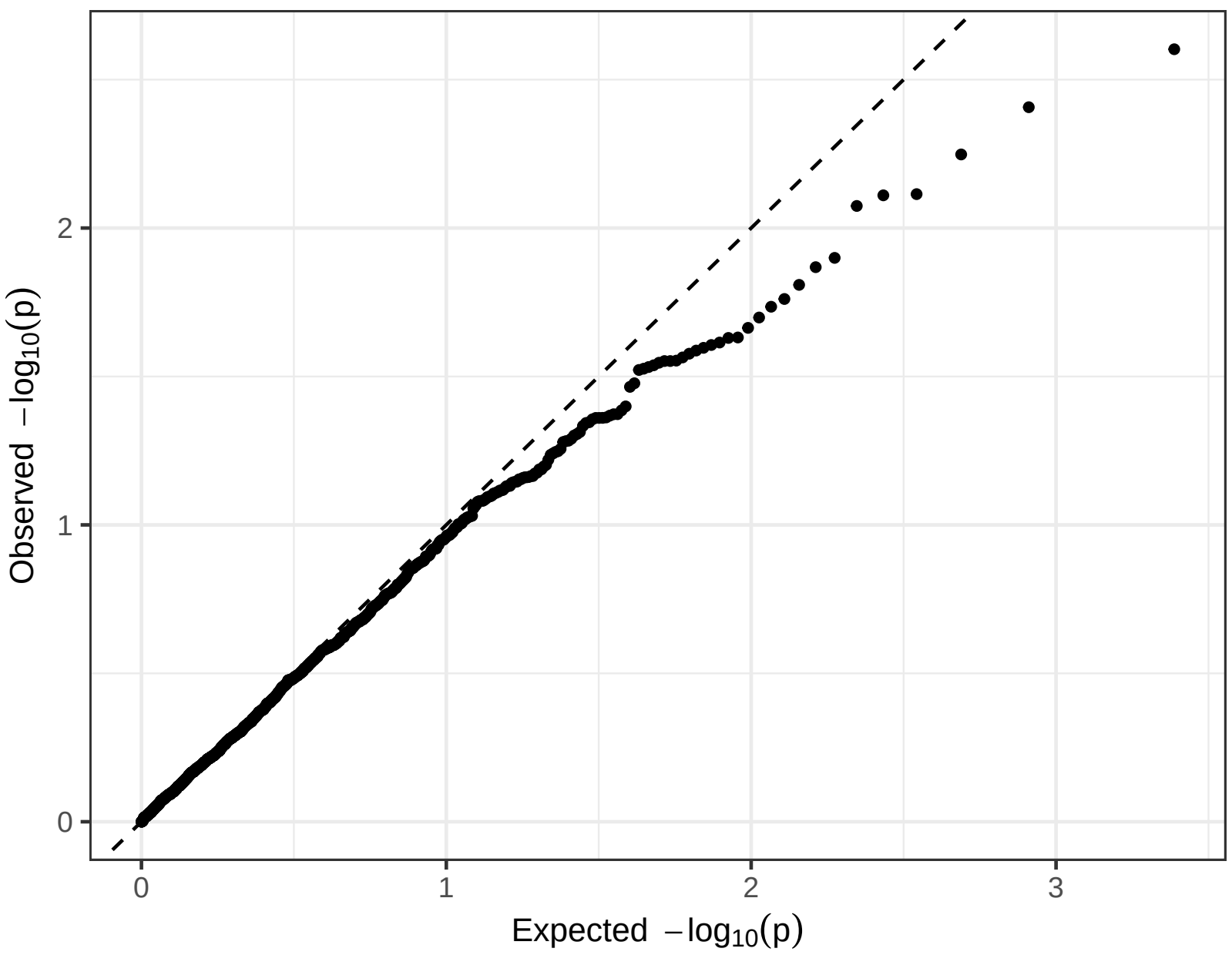

Lachnospiraceae_UCG.007

Cecum | Liver | . = 0.905

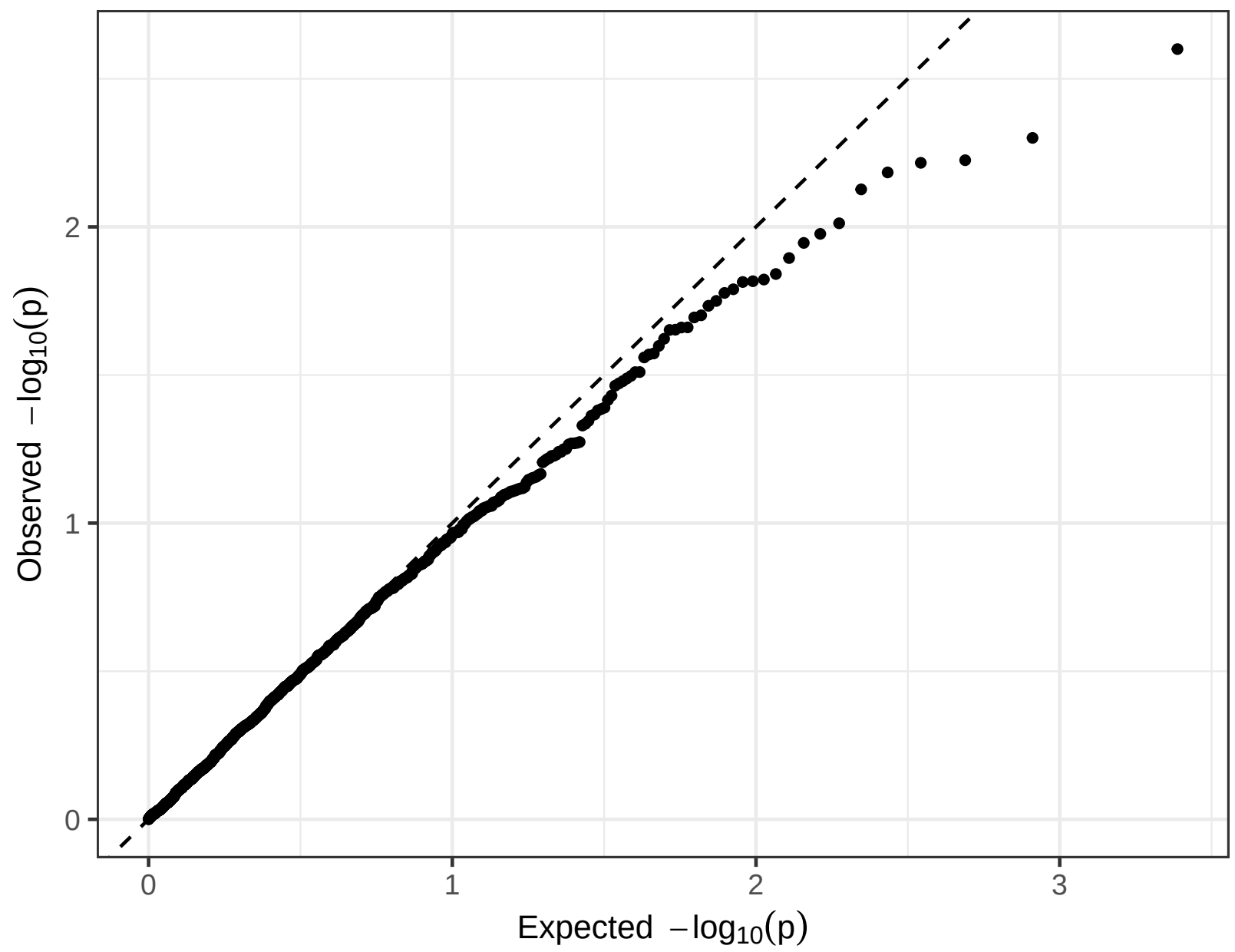

Lachnospiraceae_UCG.008

Cecum | Liver | . = 0.915

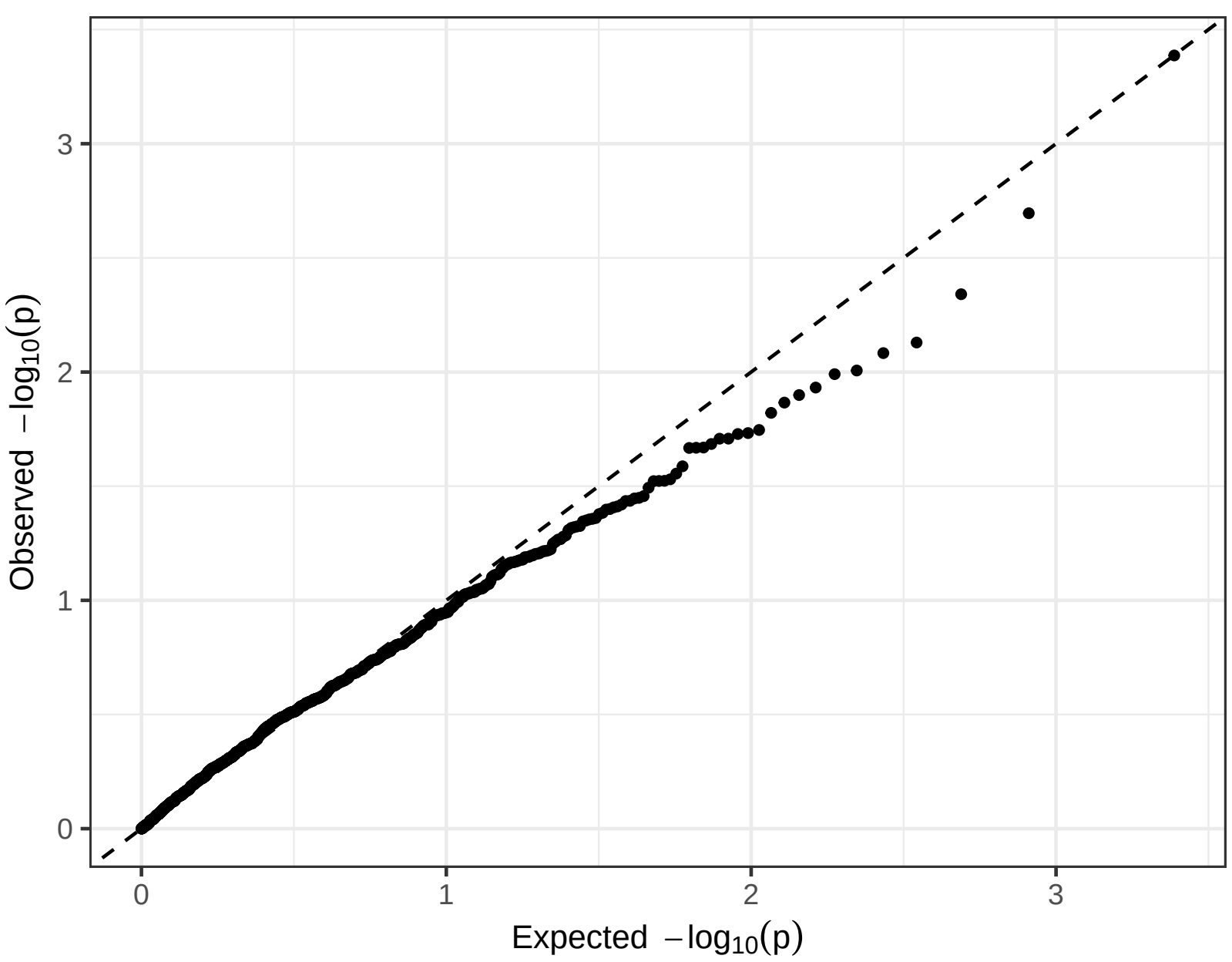

Lachnospiraceae_UCG.009

Cecum | Liver | . = 0.911

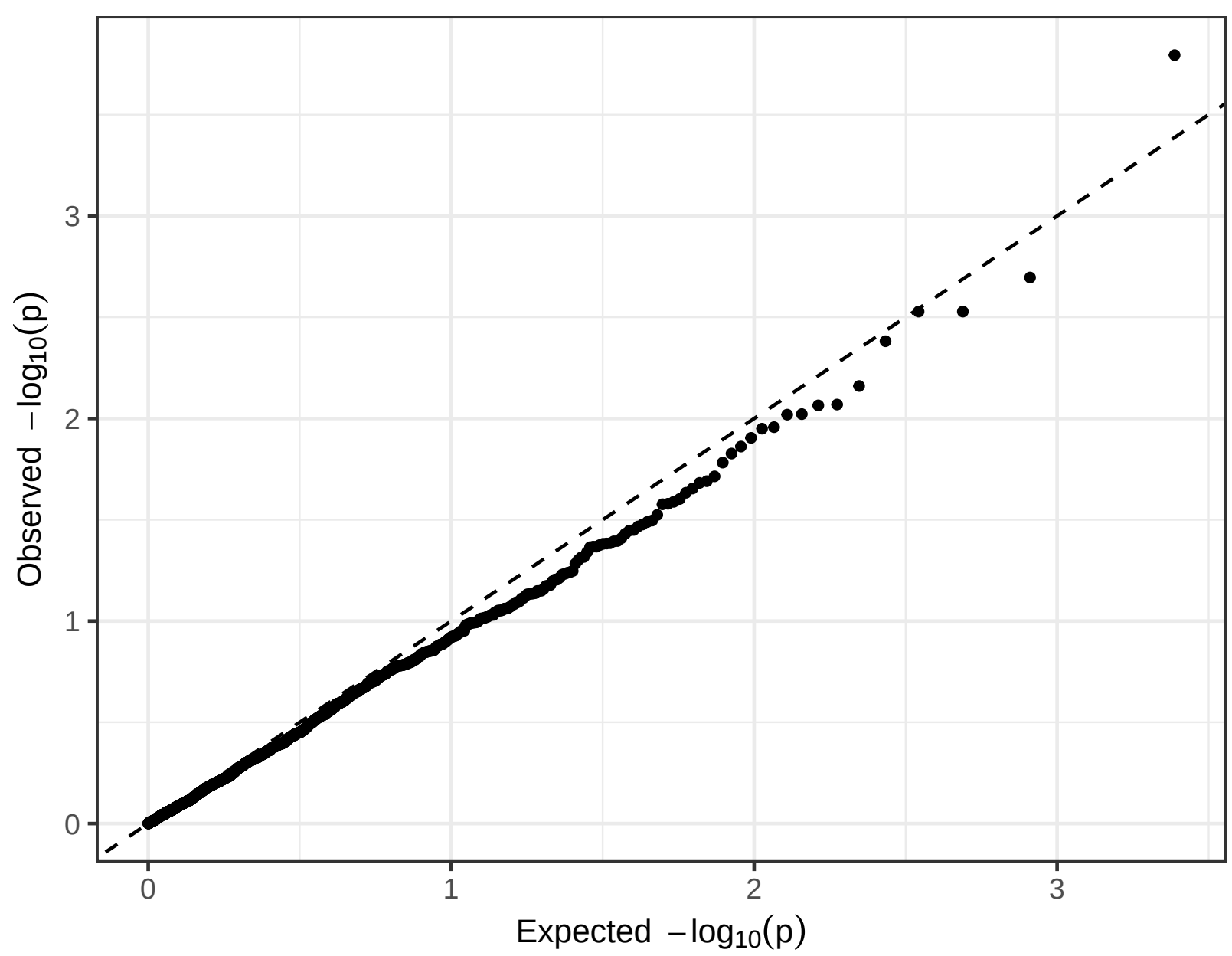

Lachnospiraceae_UCG.010

Cecum | Liver | . = 0.934

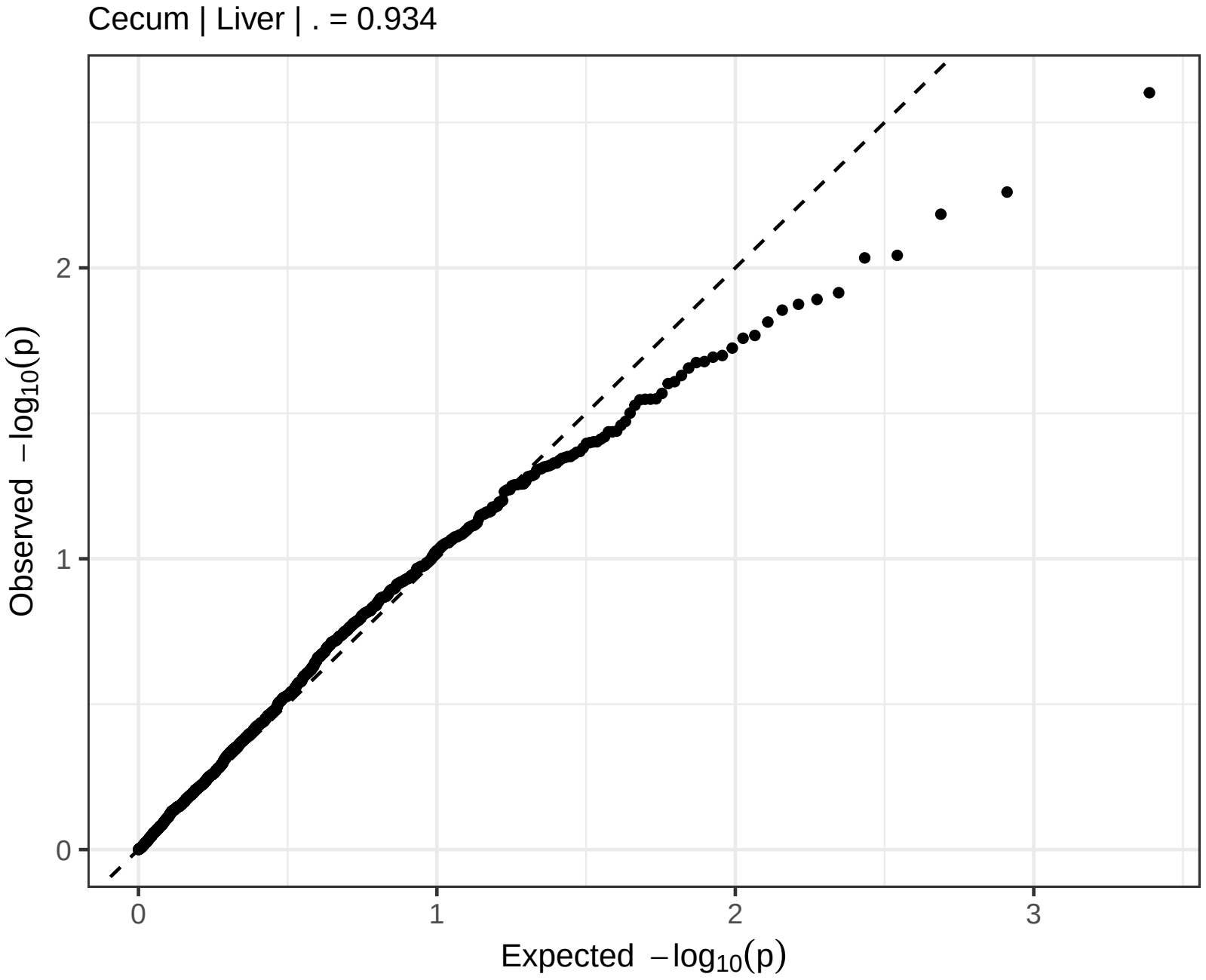

Lachnospiraceae_XPB1014_group

Cecum | Liver | . = 0.888

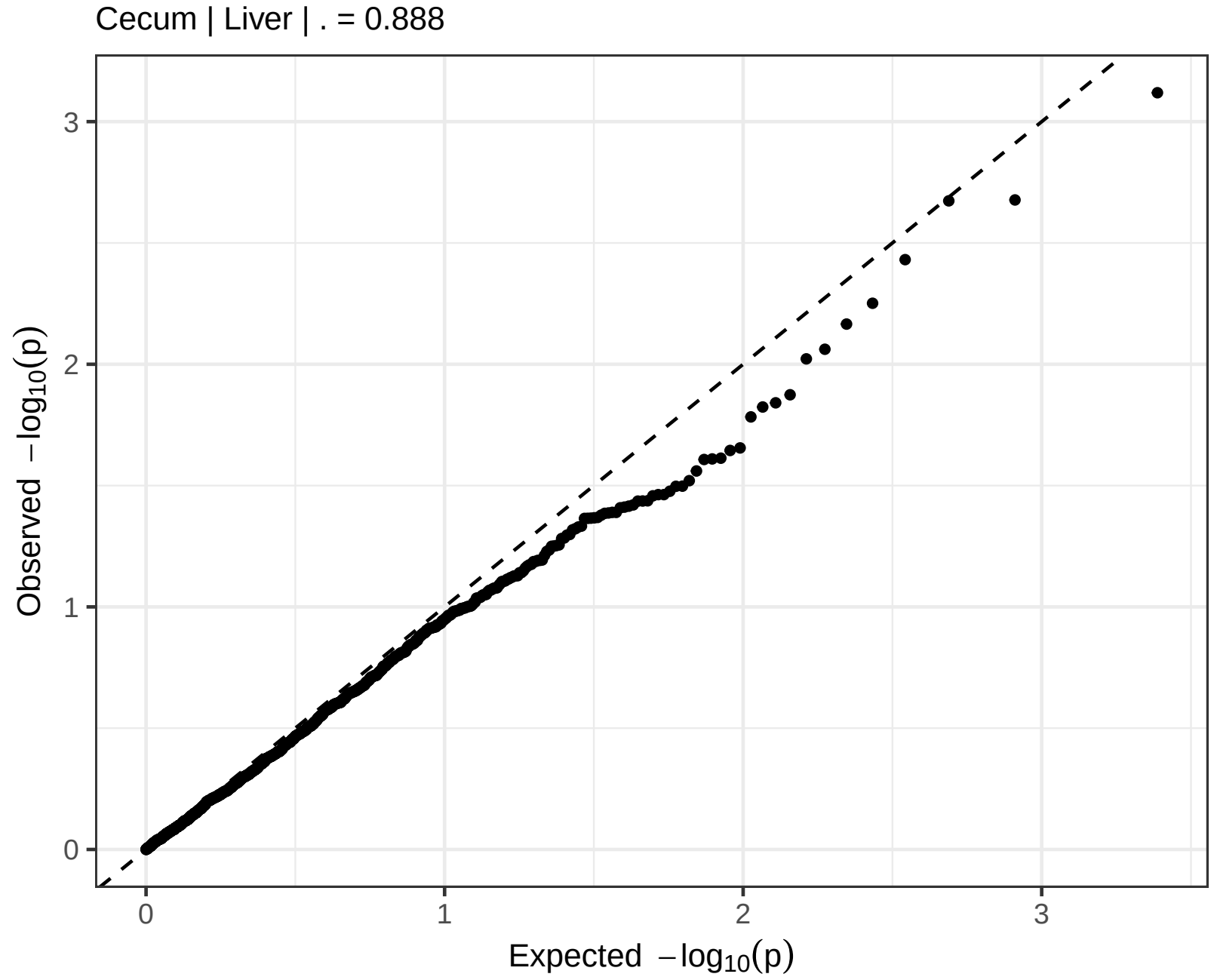

Lactobacillus

Cecum | Liver | . = 0.899

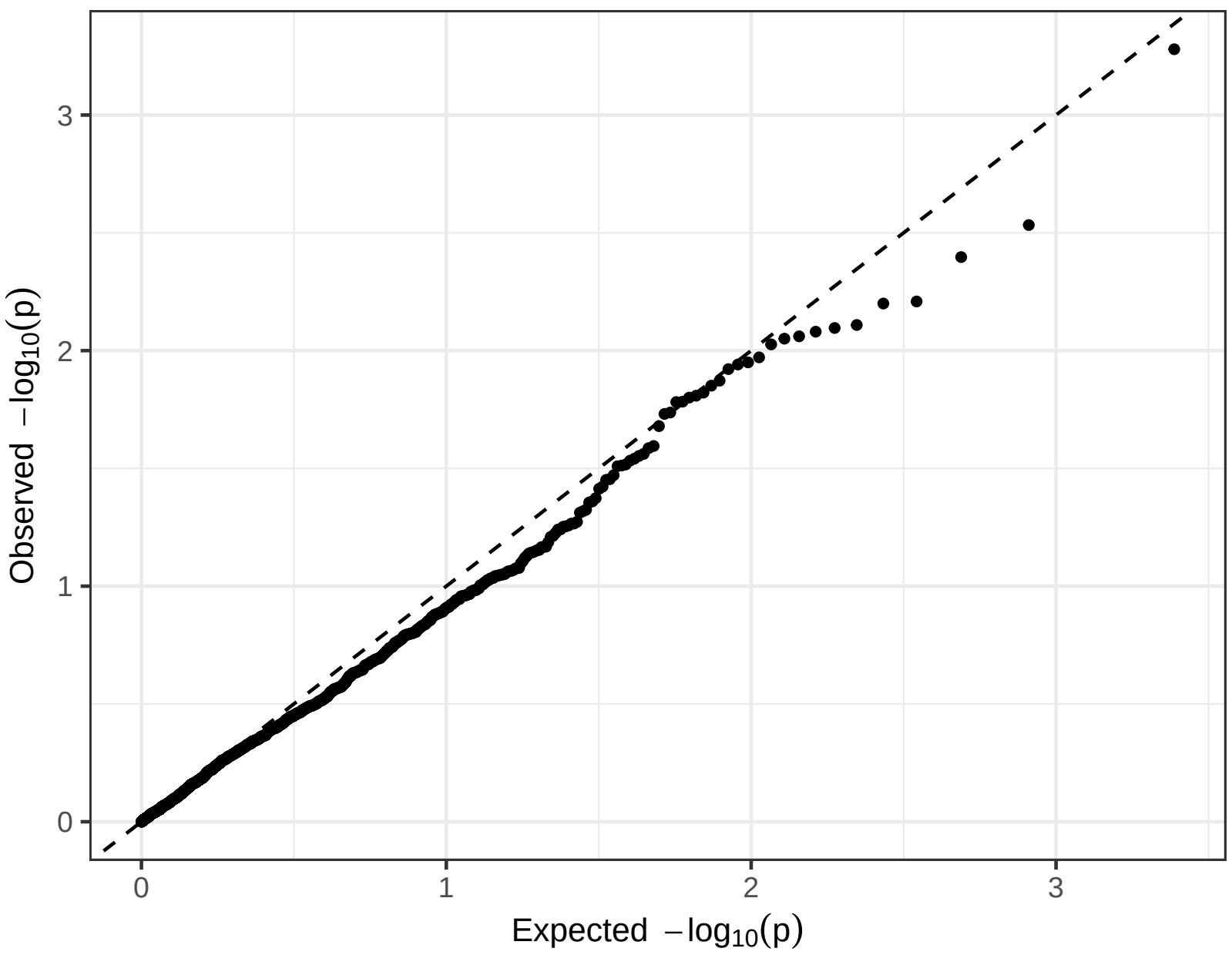

Leyella

Cecum | Liver | . = 0.882

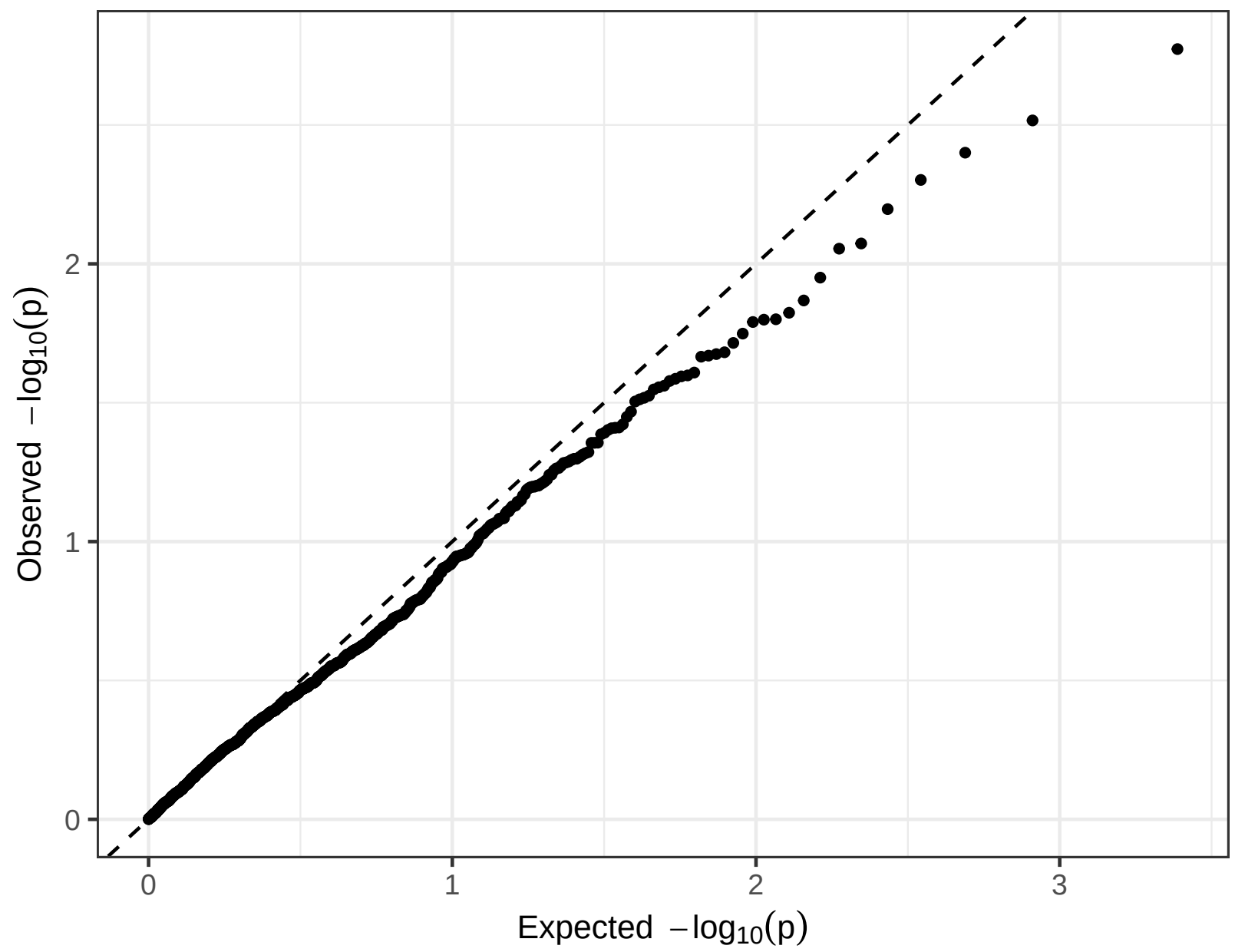

Mailhella

Cecum | Liver | . = 0.936

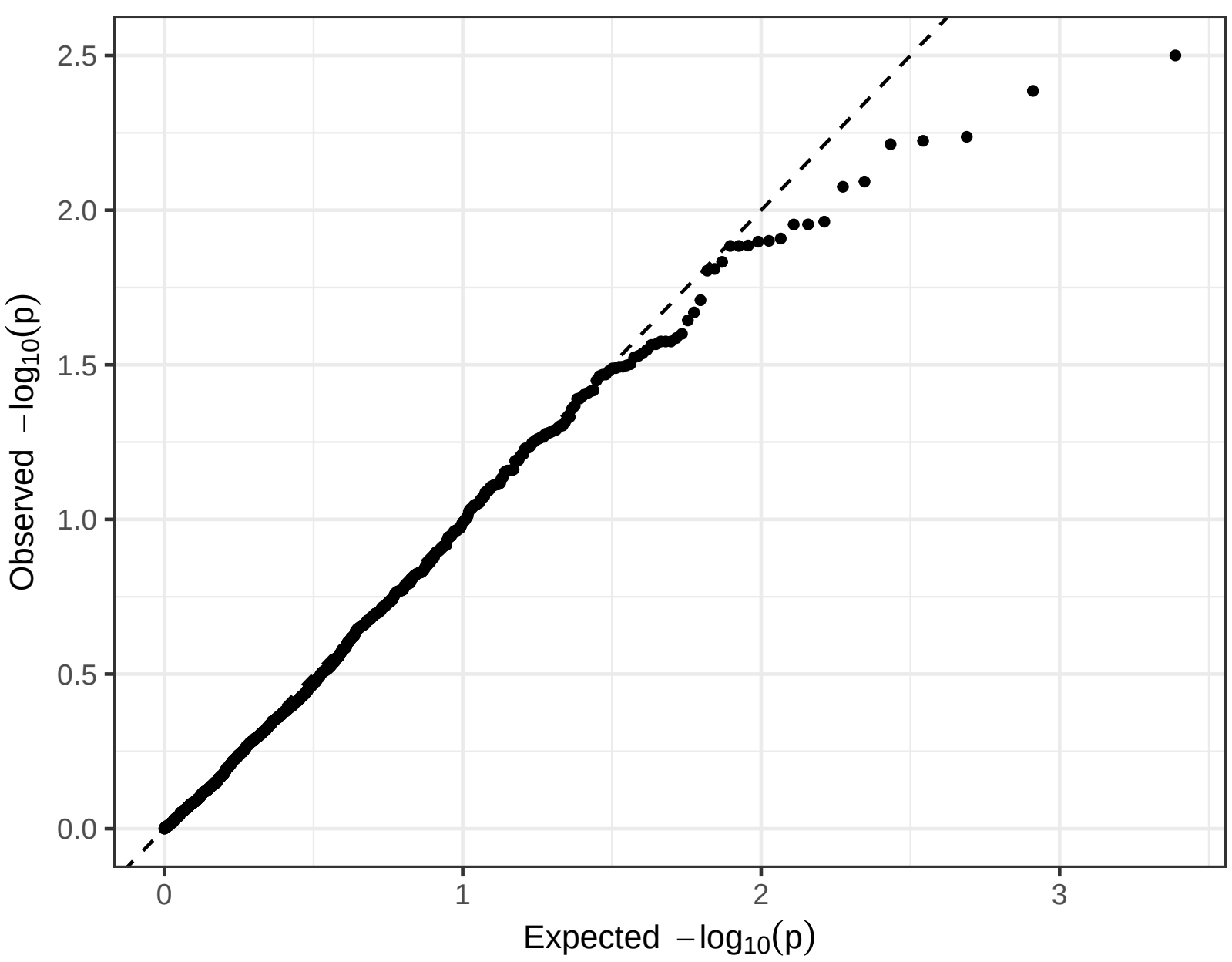

Marvinbryantia

Cecum | Liver | . = 0.989

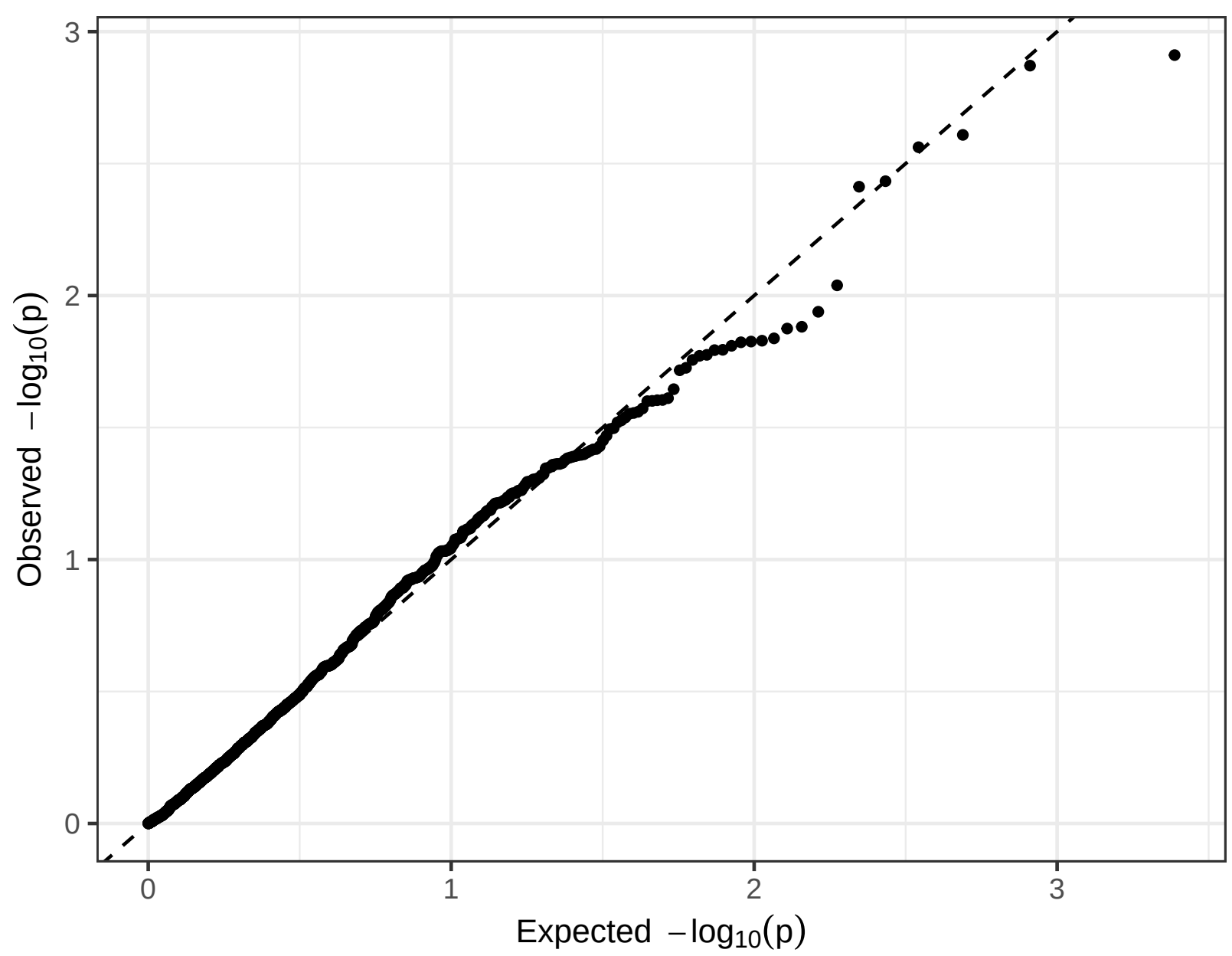

Mediterraneibacter

Cecum | Liver | . = 0.89

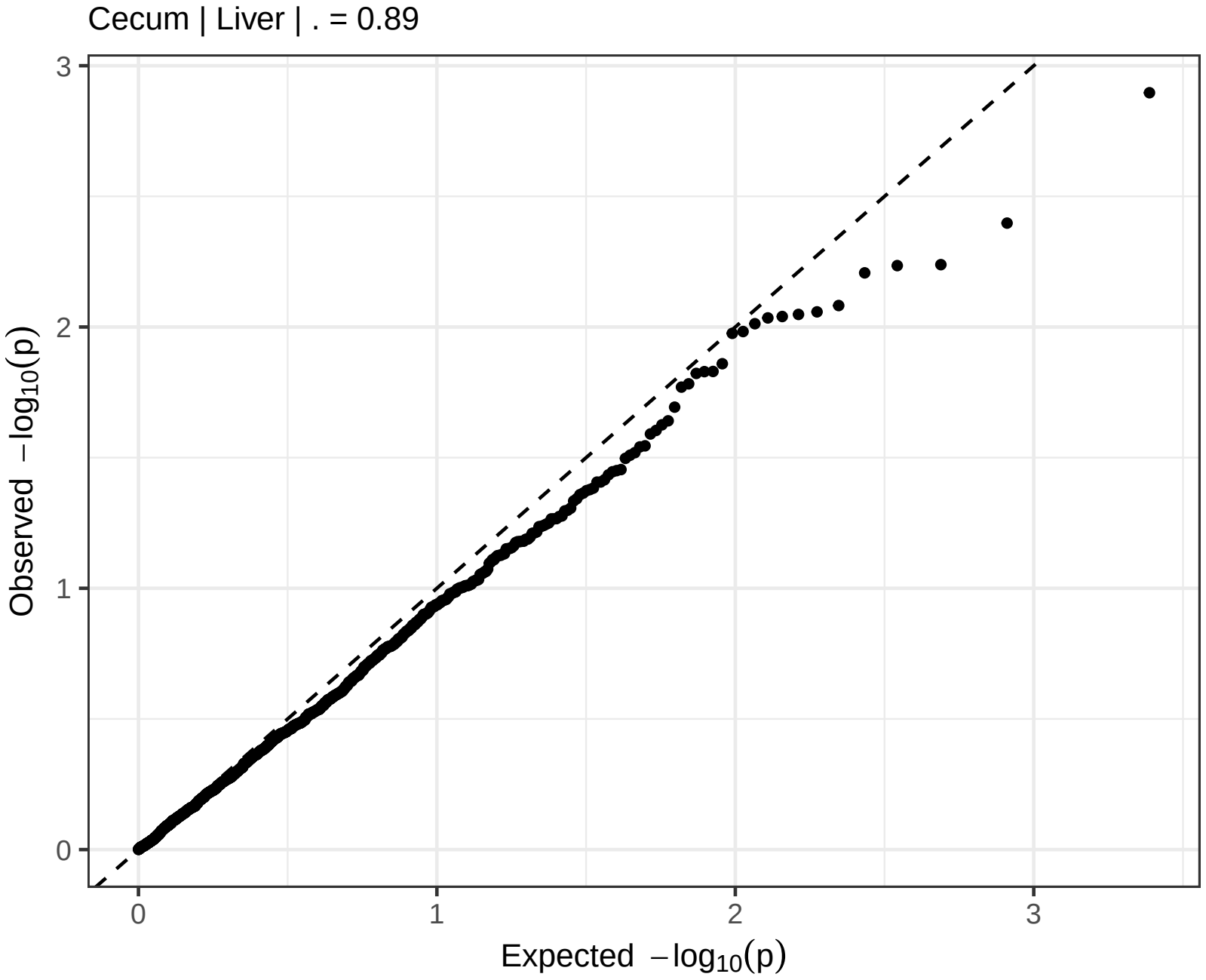

Supplement: Supplementary file 1 [file 10142_2026_2009_MOESM1_ESM.pdf]
