# Supplementary material for: Associations of host gene expression in the brain, liver, muscle with fecal and cecal microbiota composition in pigs
Source: Funct Integr Genomics. 2026 Aug 15;26(1):226. doi: 10.1007/s10142-026-02009-5 (PMC13477493; doi:10.1007/s10142-026-02009-5)
Supplement: Supplementary file 2 [file 10142_2026_2009_MOESM2_ESM.pdf]

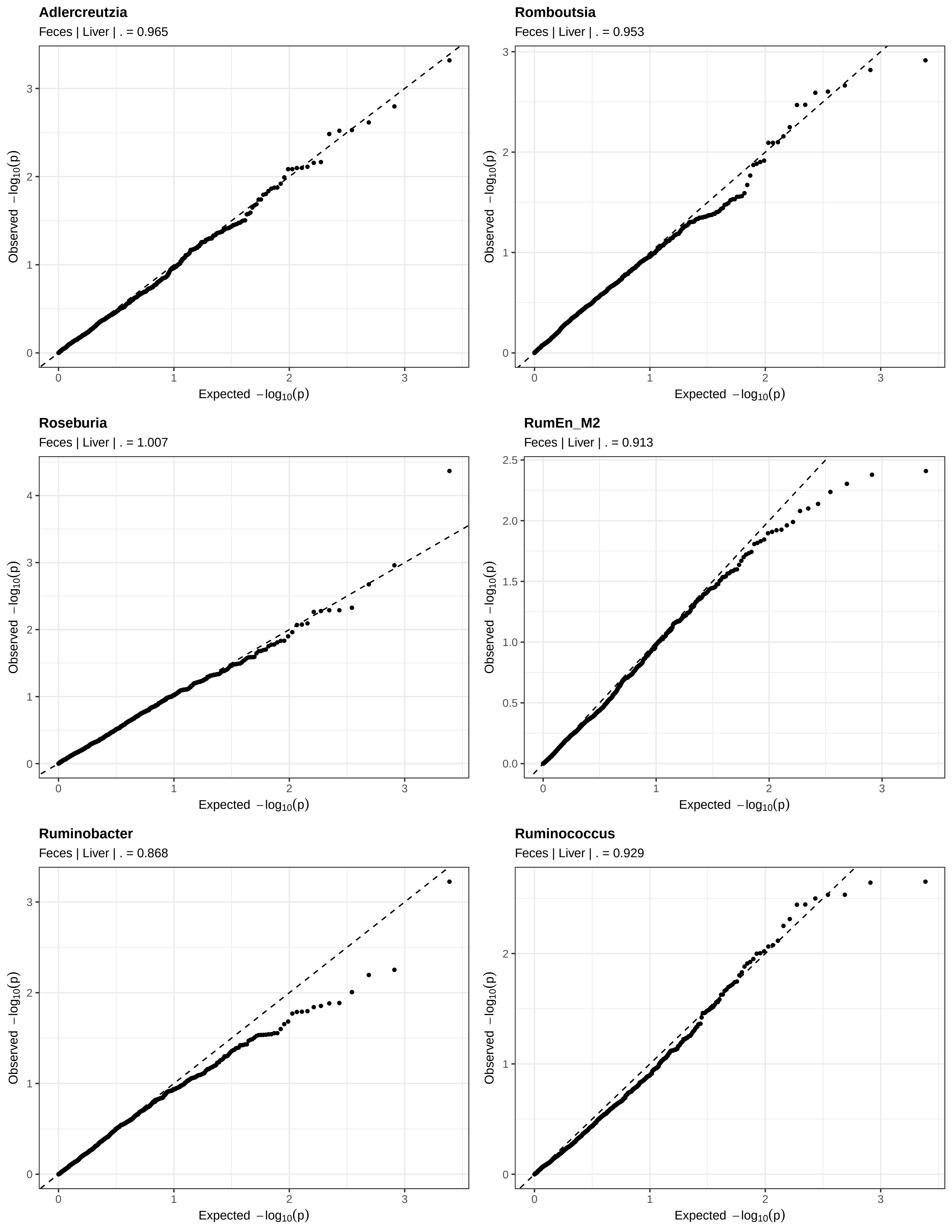
Adlercreutzia
Feces | Liver | . = 0.965
Romboutsia
Feces | Liver | . = 0.953
Roseburia
Feces | Liver | . = 1.007
RumEn_M2
Feces | Liver | . = 0.913
Ruminobacter
Feces | Liver | . = 0.868
Ruminococcus
Feces | Liver | . = 0.929
Observed $-\log_{10}(p)$
Expected $-\log_{10}(p)$

Schwartzia

Feces | Liver | . = 0.904

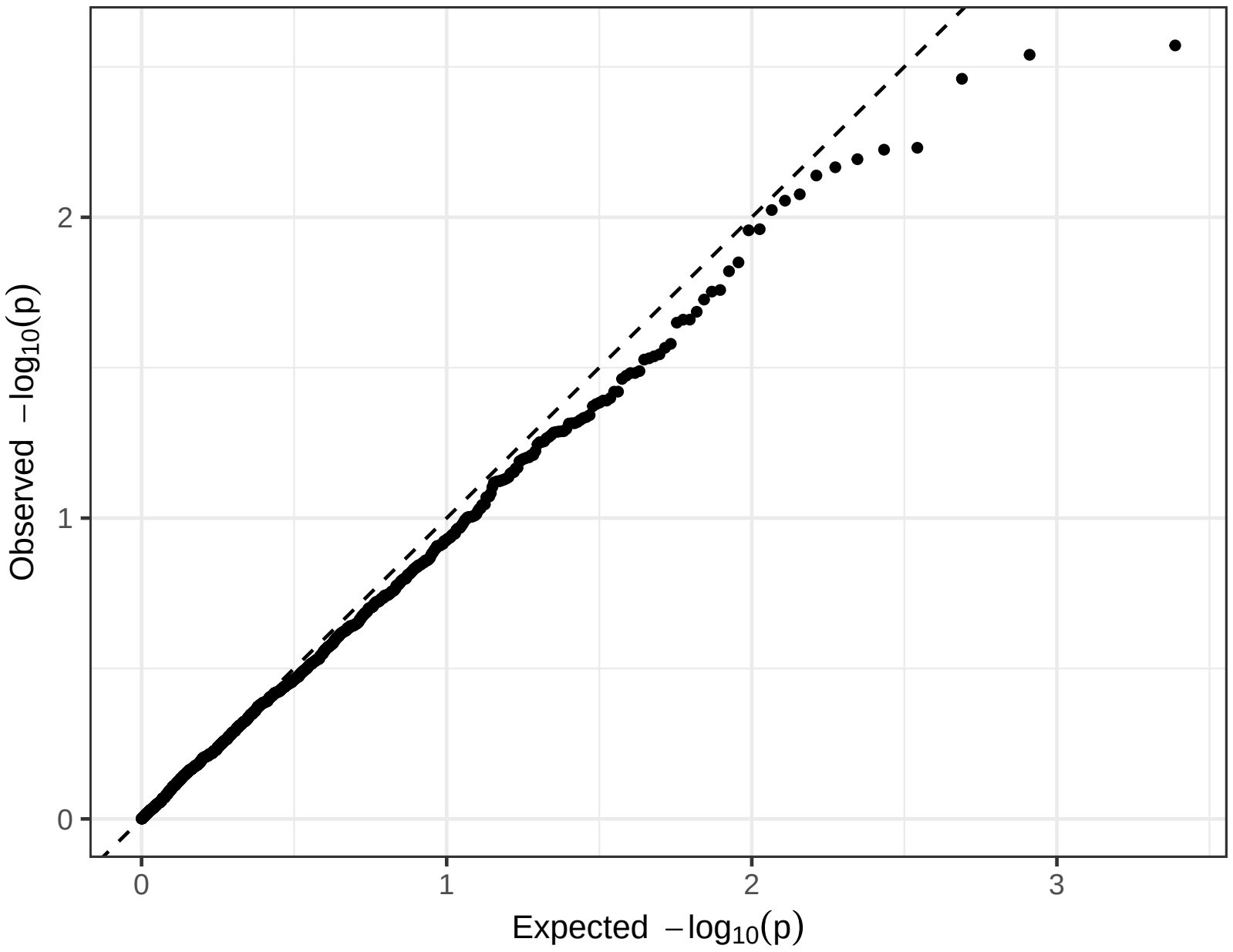

Segatella

Feces | Liver | . = 0.869

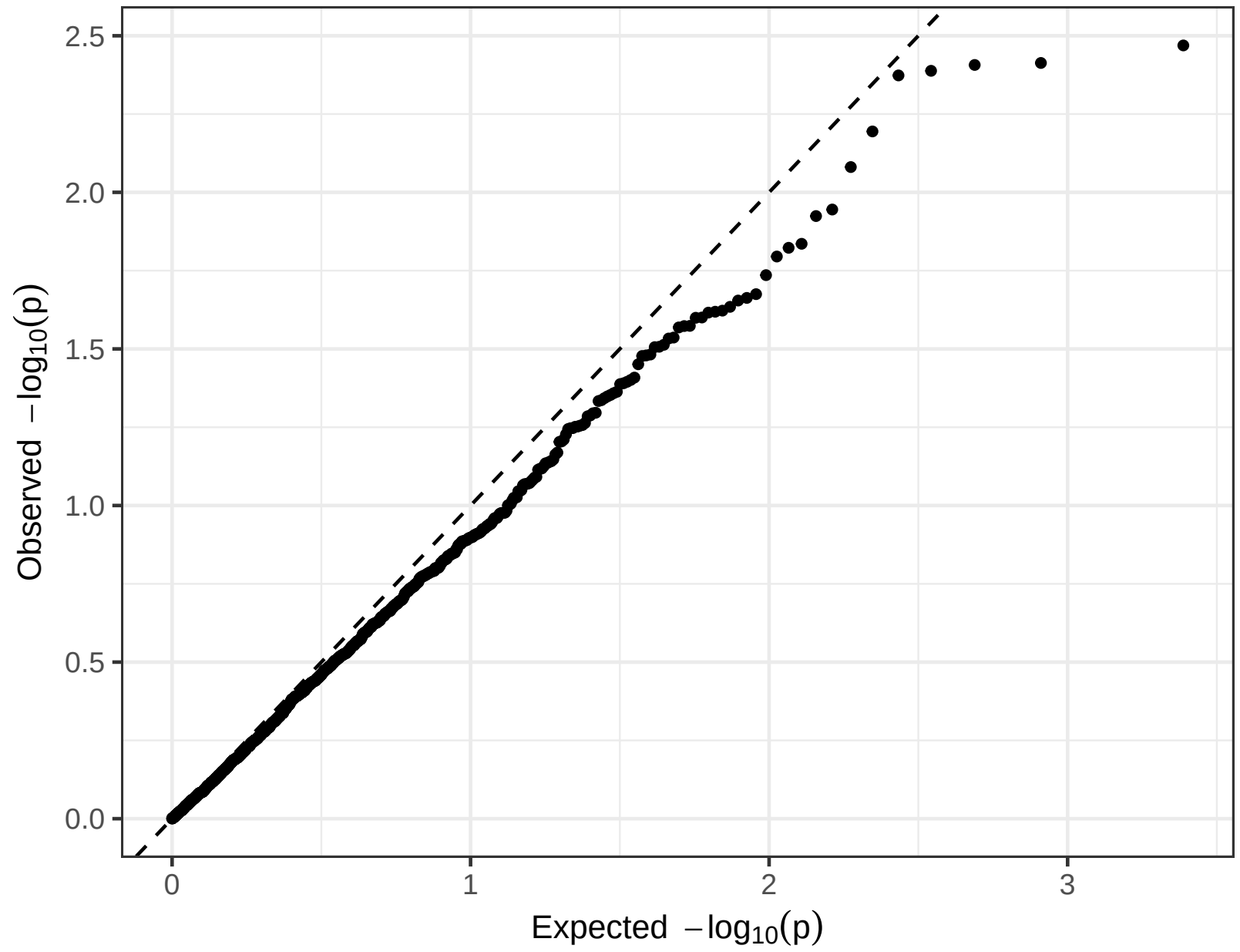

Selenomonas

Feces | Liver | . = 0.984

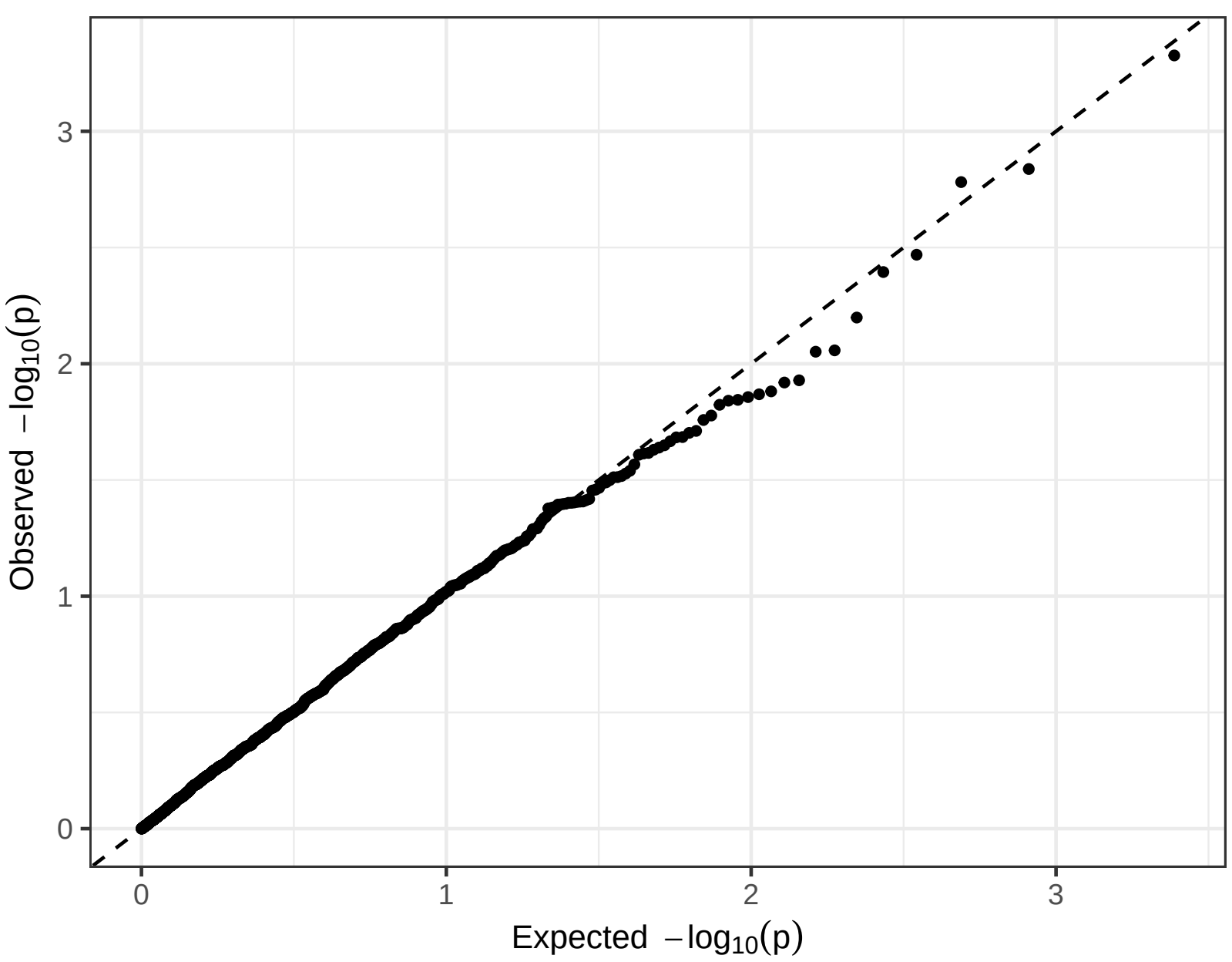

Senegalimassilia

Feces | Liver | . = 0.878

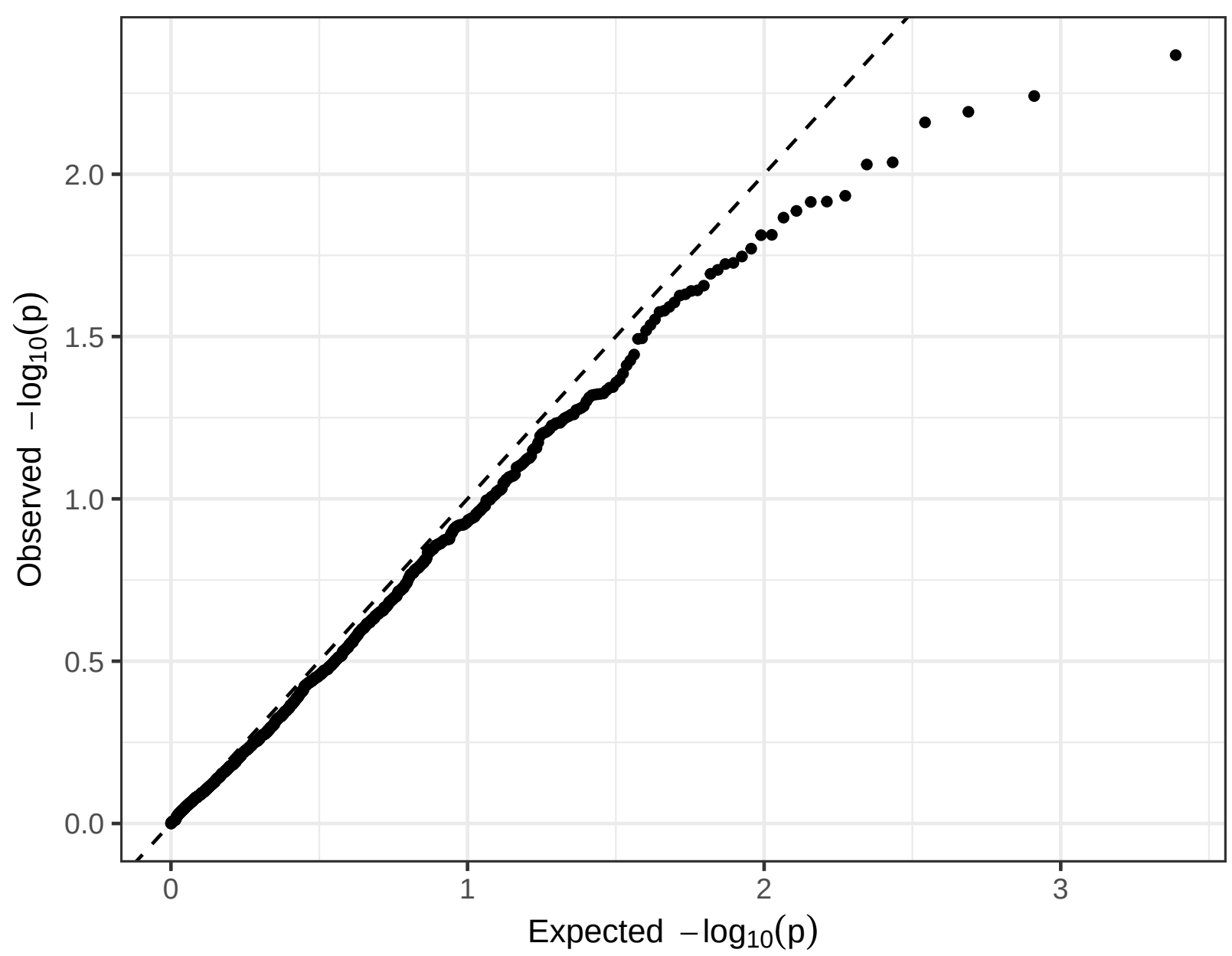

Shuttleworthia

Feces | Liver | . = 0.958

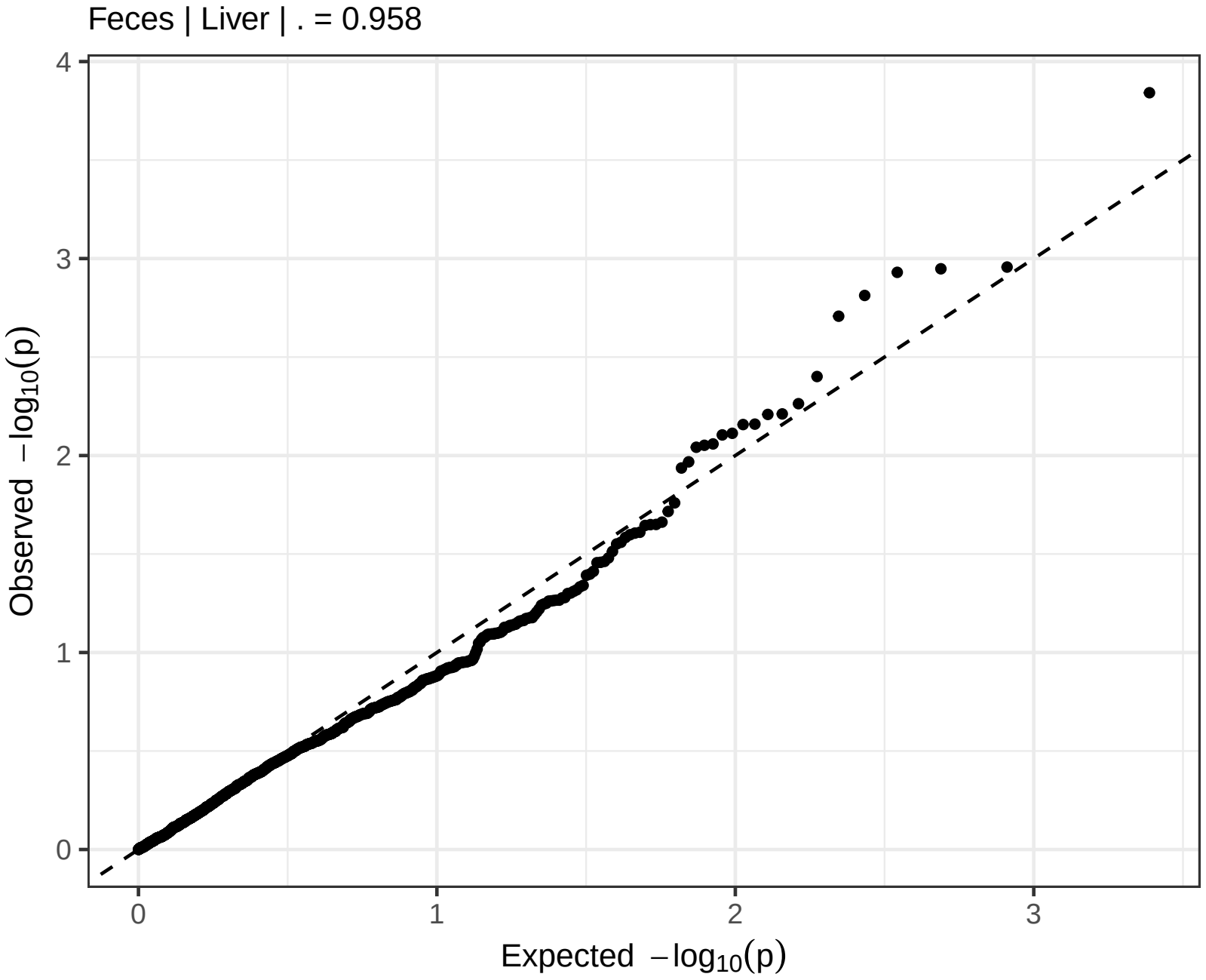

Agathobacter

Feces | Liver | . = 1.006

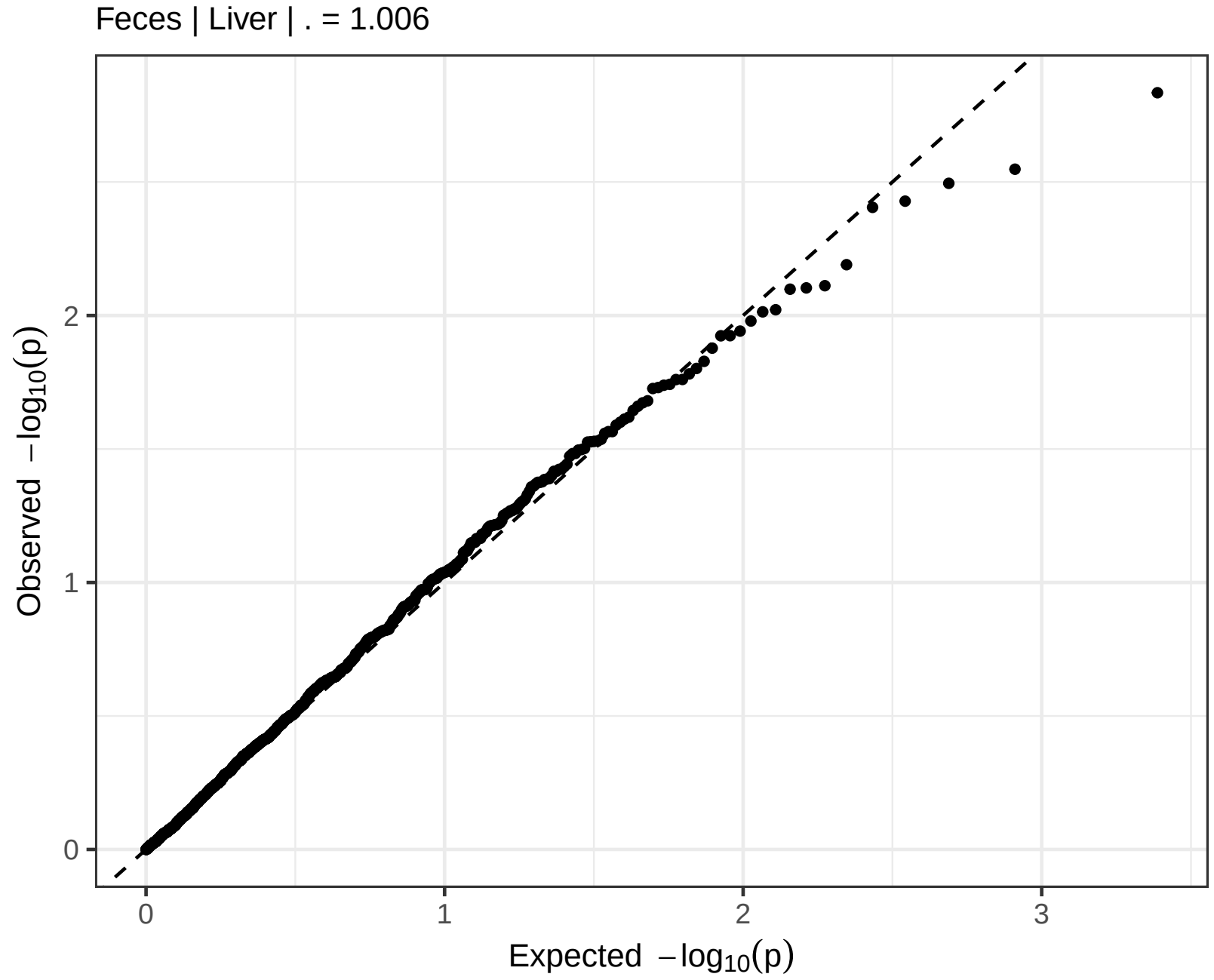

Slackia

Feces | Liver | . = 1.039

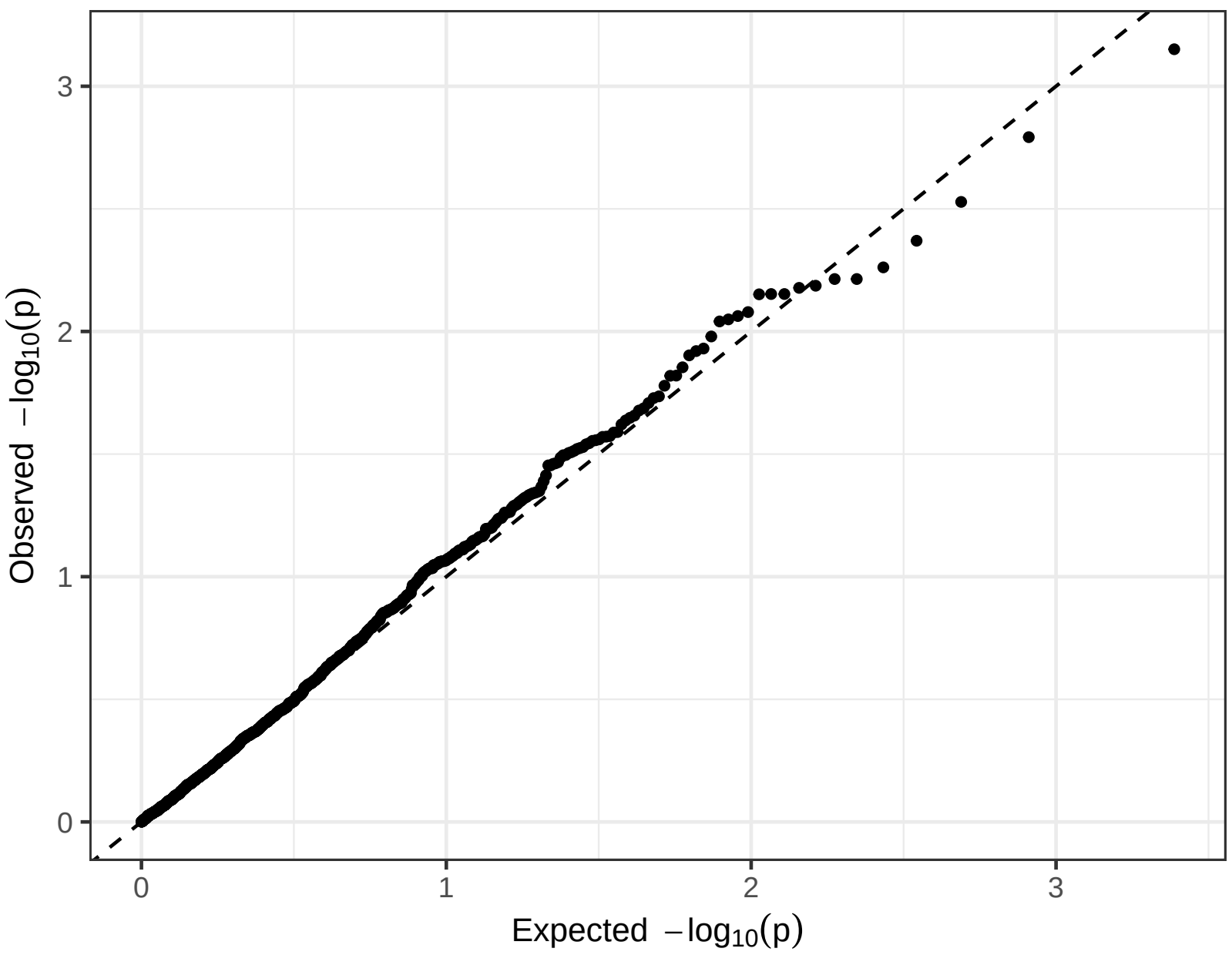

Solobacterium

Feces | Liver | . = 0.902

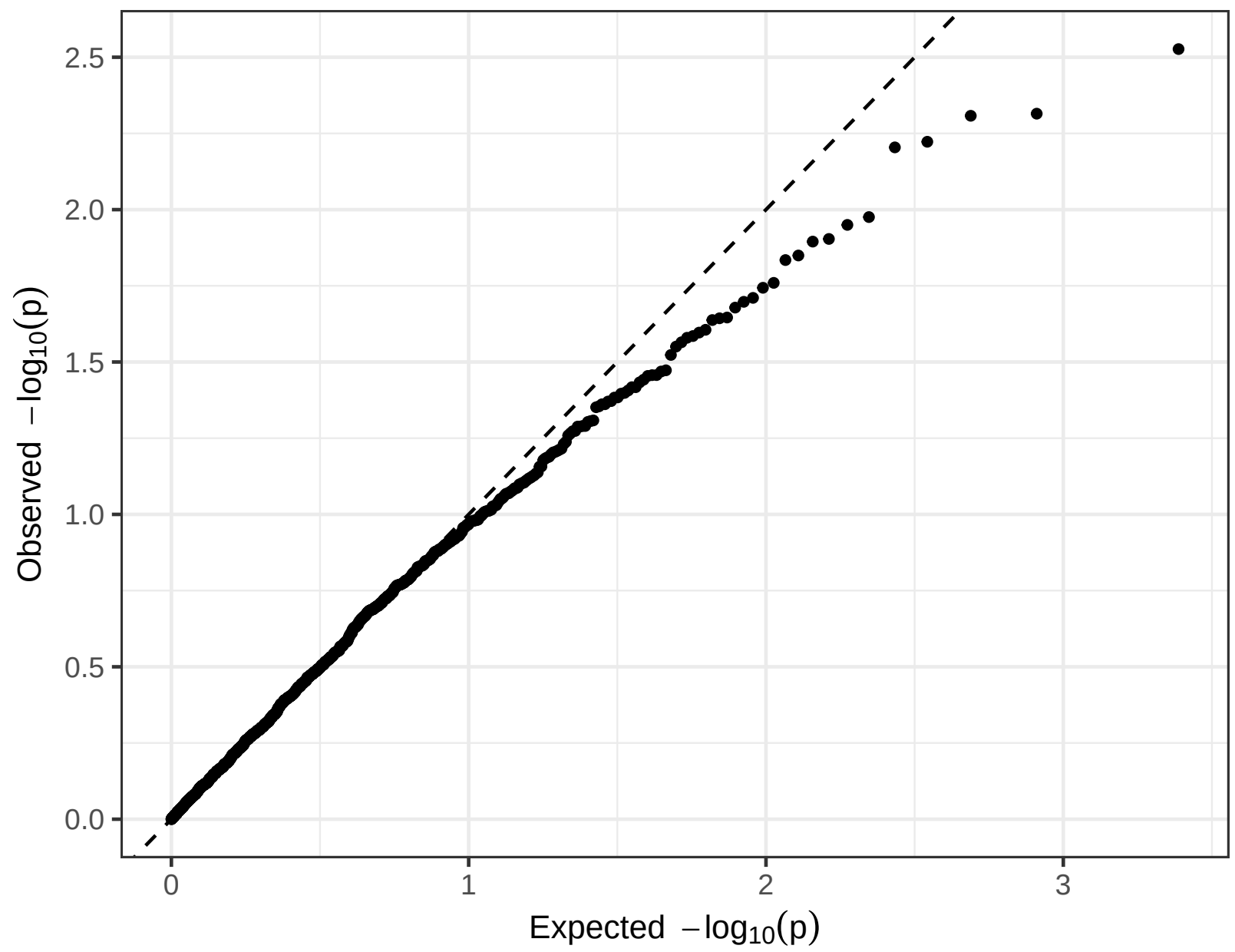

Streptococcus

Feces | Liver | . = 0.969

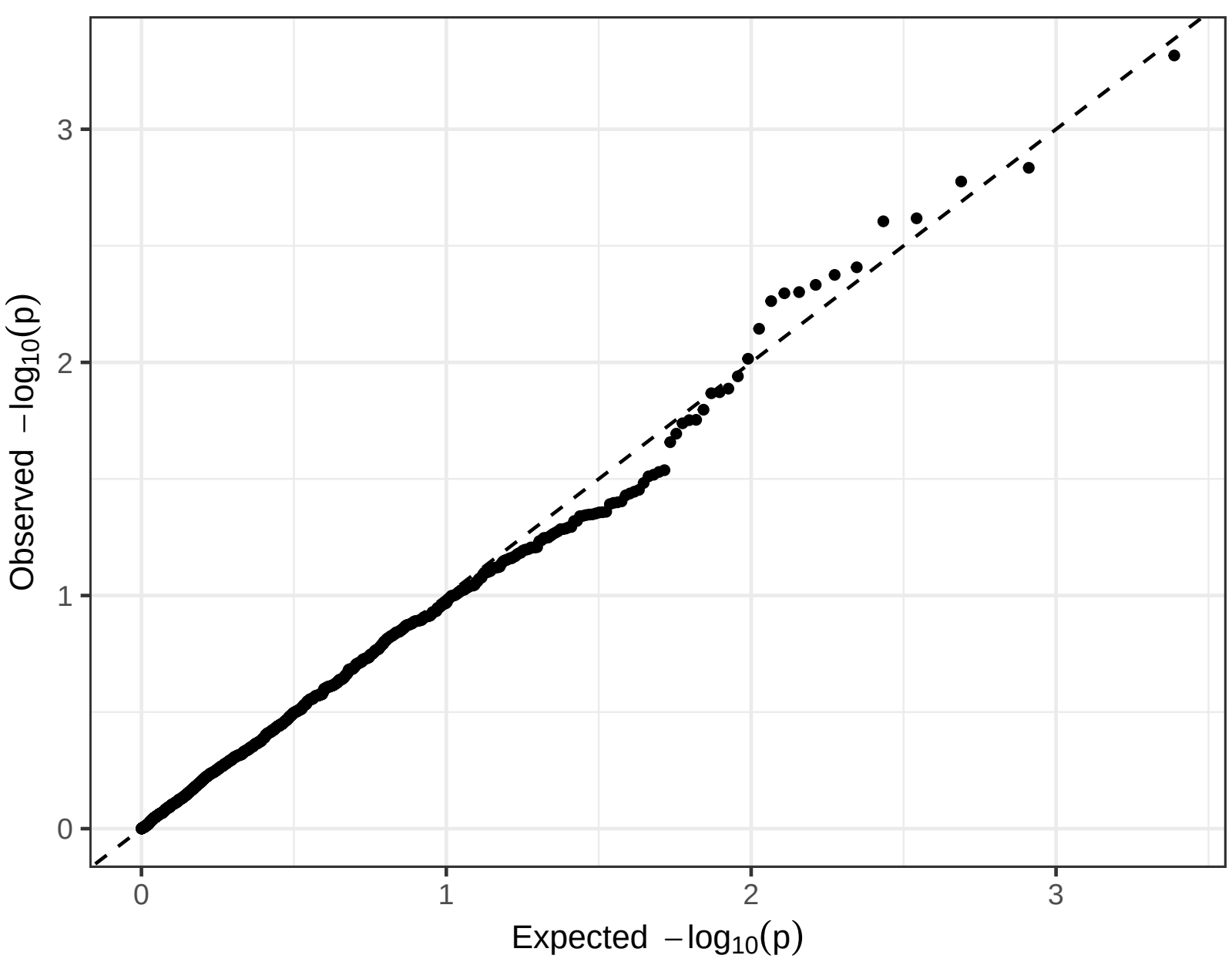

Succinivibrio

Feces | Liver | . = 1.052

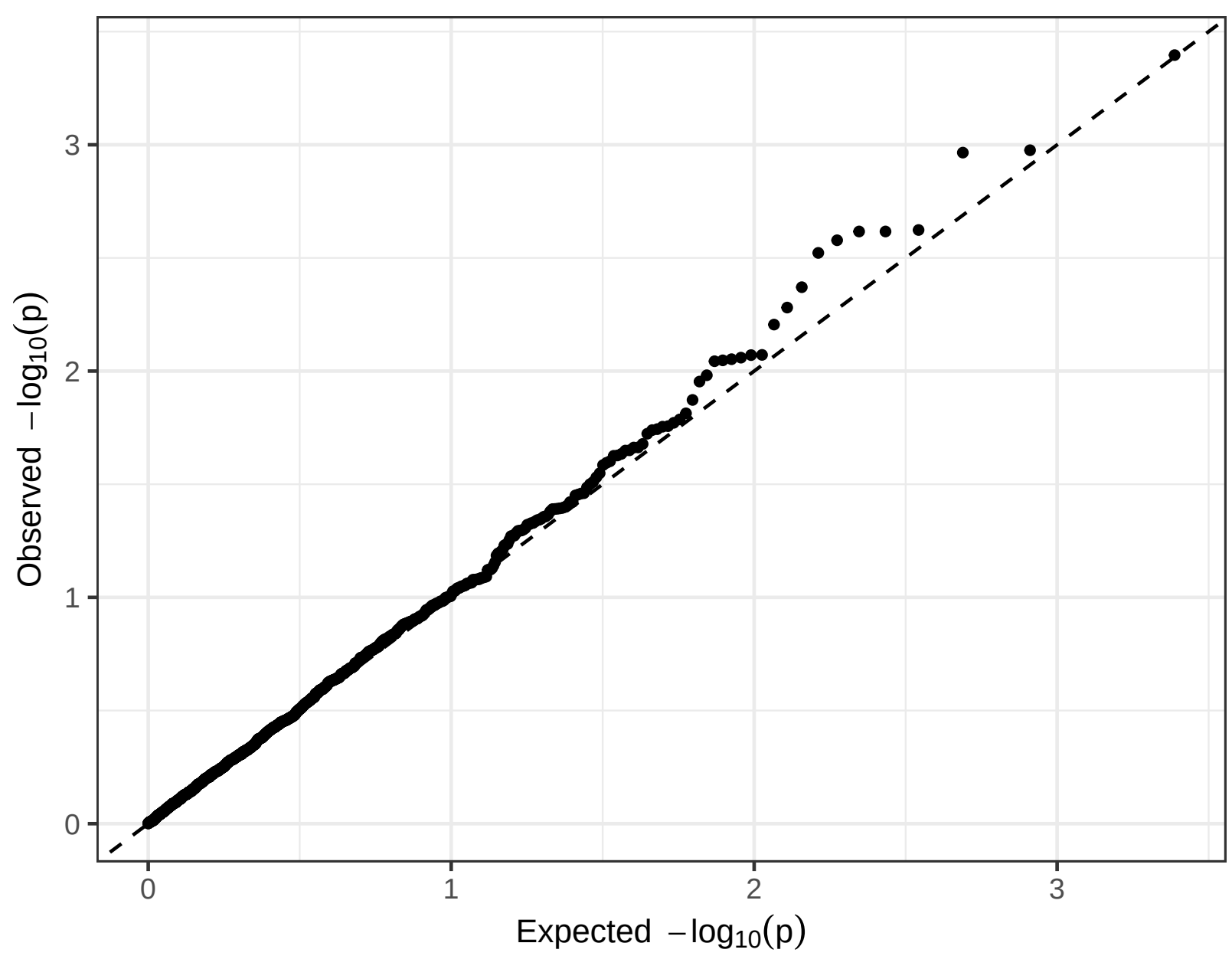

Succinivibrionaceae_UCG.001

Feces | Liver | . = 0.864

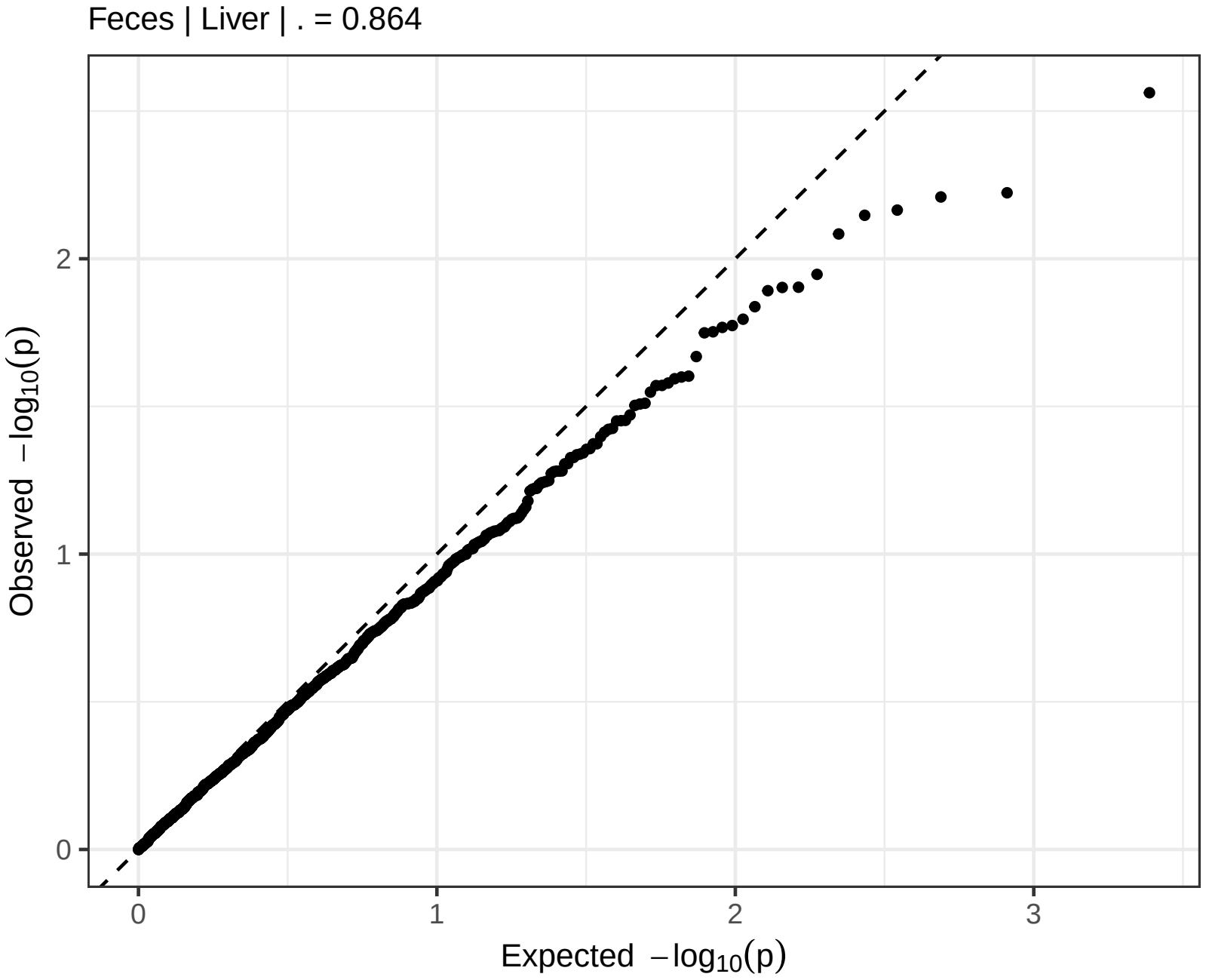

Sutterella

Feces | Liver | . = 0.9

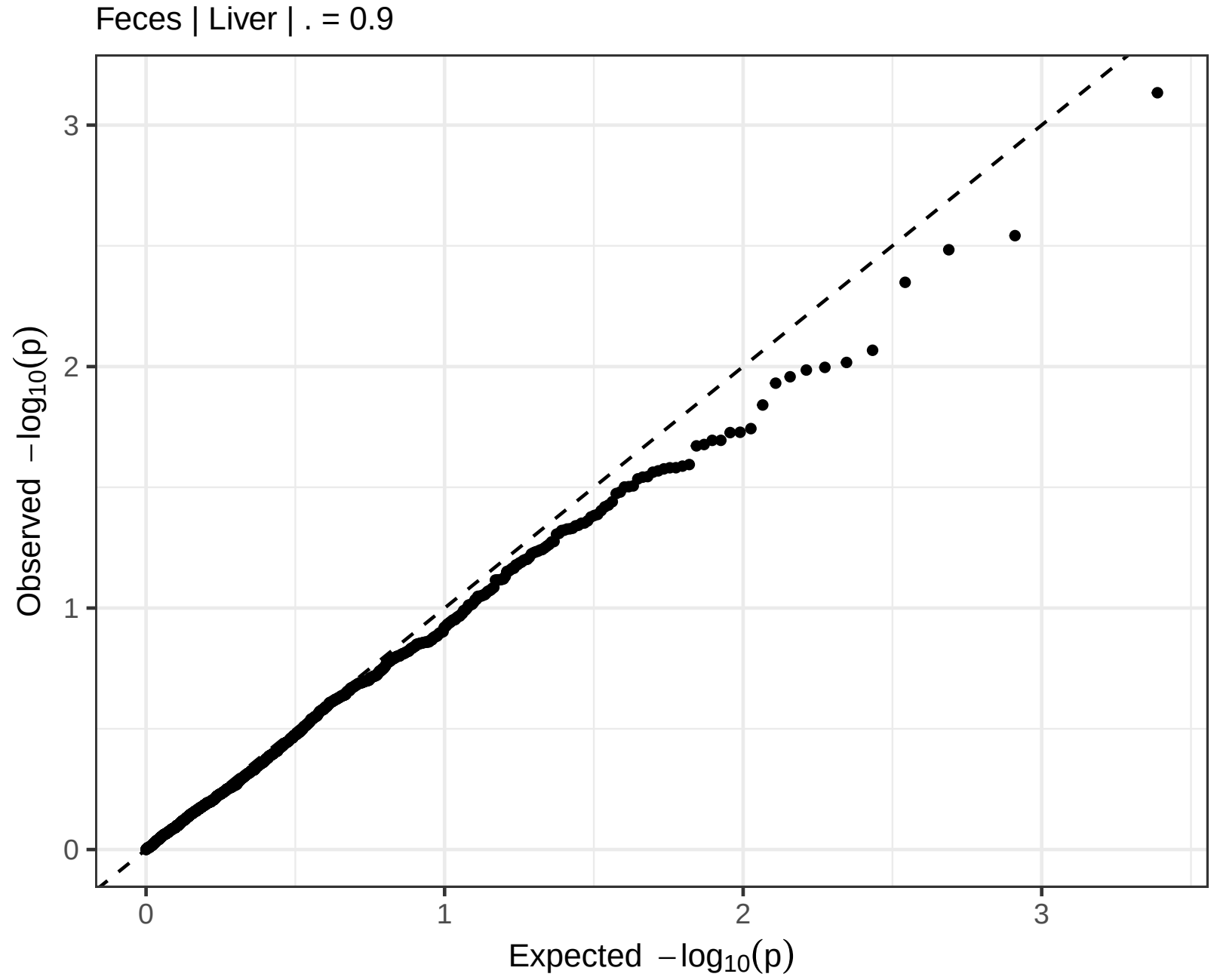

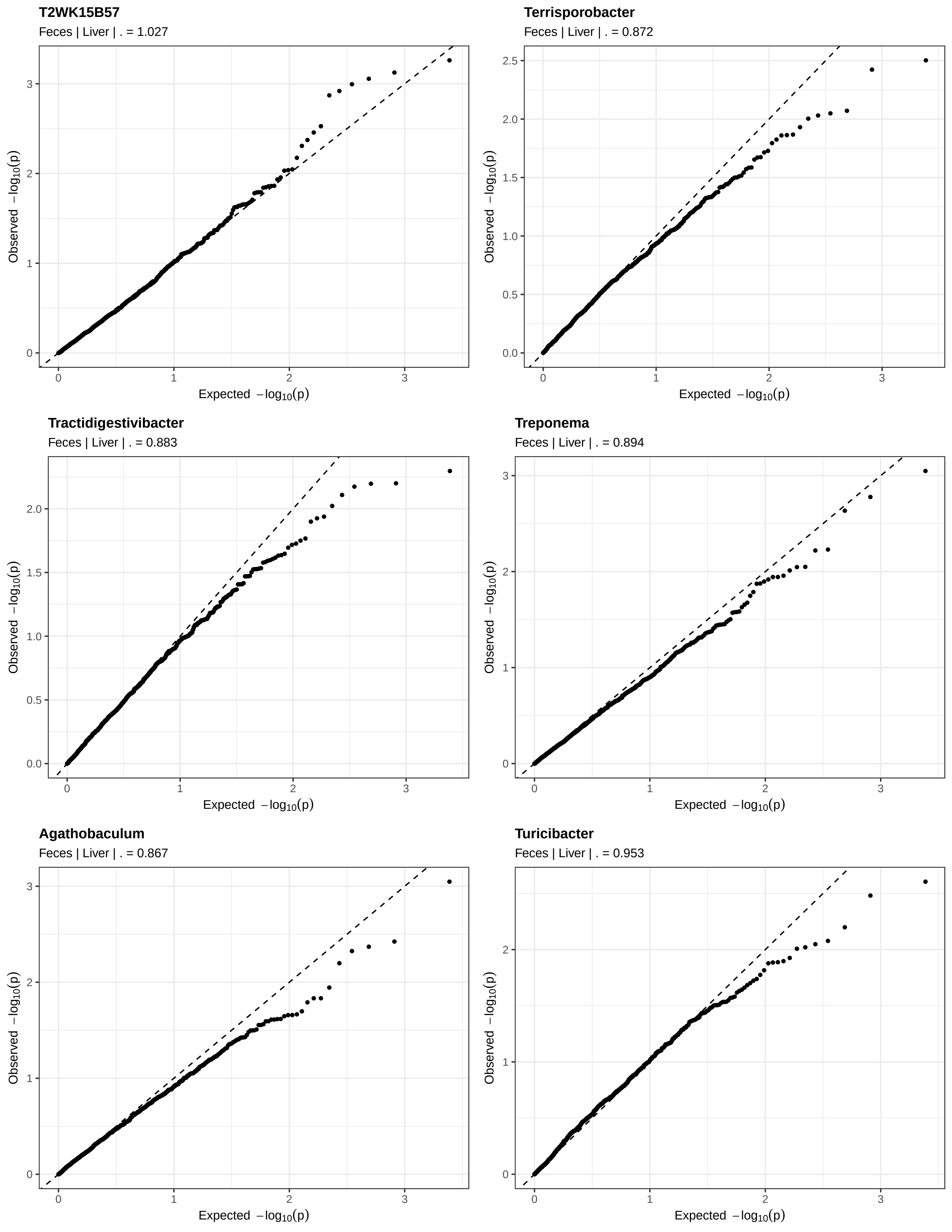
T2WK15B57
Feces | Liver | . = 1.027
Terrisporobacter
Feces | Liver | . = 0.872
Tractidigestivibacter
Feces | Liver | . = 0.883
Treponema
Feces | Liver | . = 0.894
Agathobaculum
Feces | Liver | . = 0.867
Turicibacter
Feces | Liver | . = 0.953
Observed $-\log_{10}(p)$
Expected $-\log_{10}(p)$

UCG.001

Feces | Liver | . = 0.929

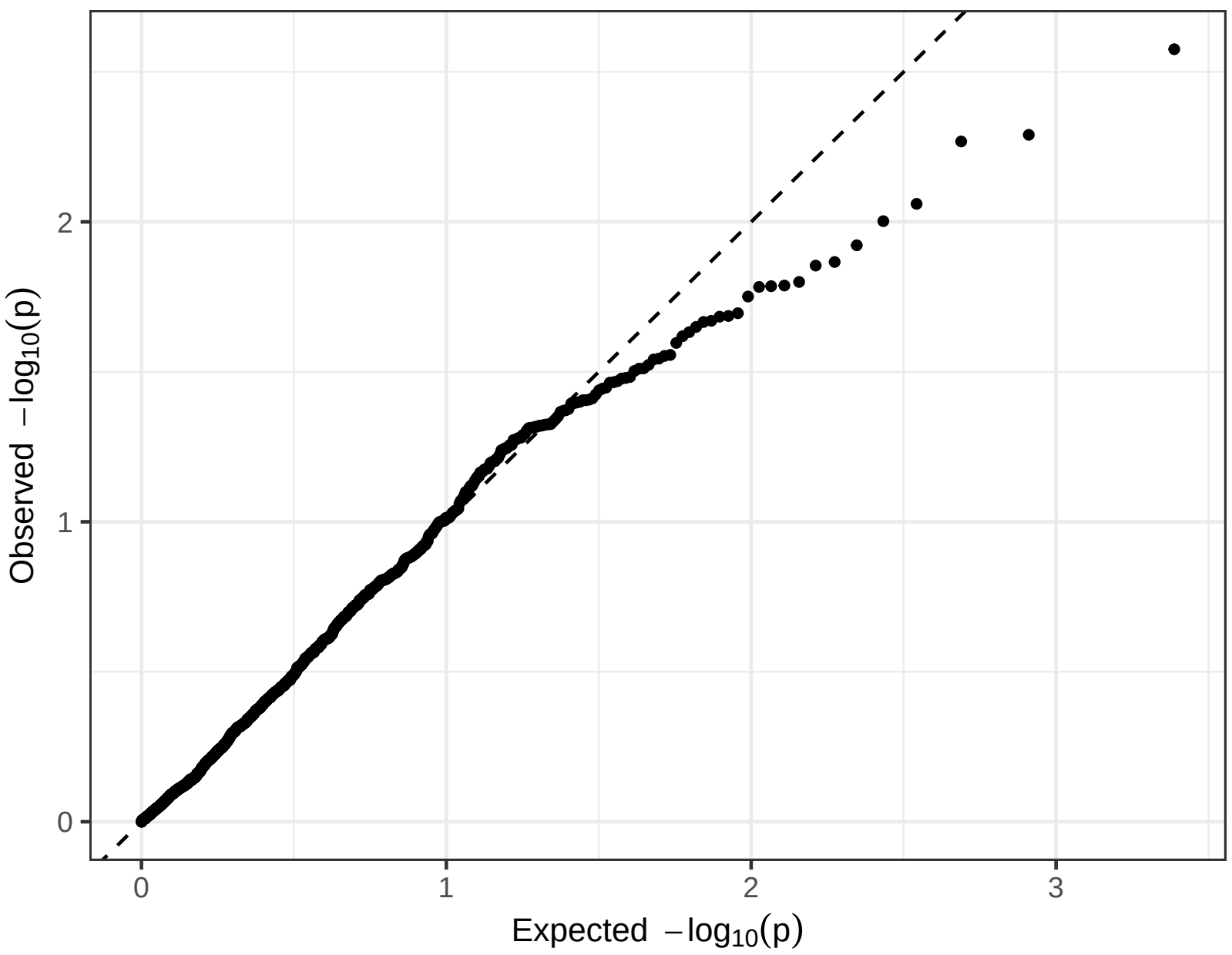

UCG.002

Feces | Liver | . = 0.871

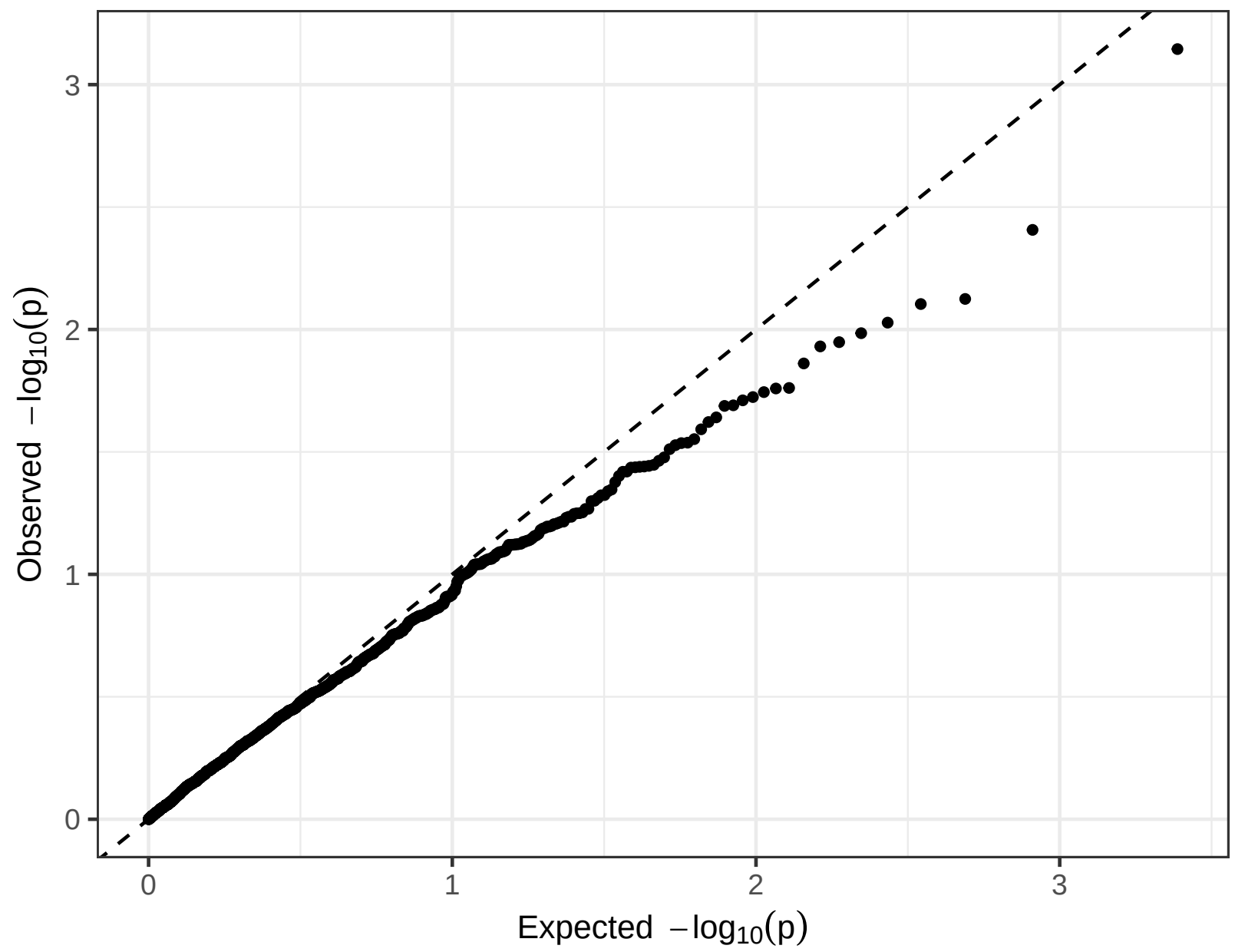

UCG.004

Feces | Liver | . = 0.974

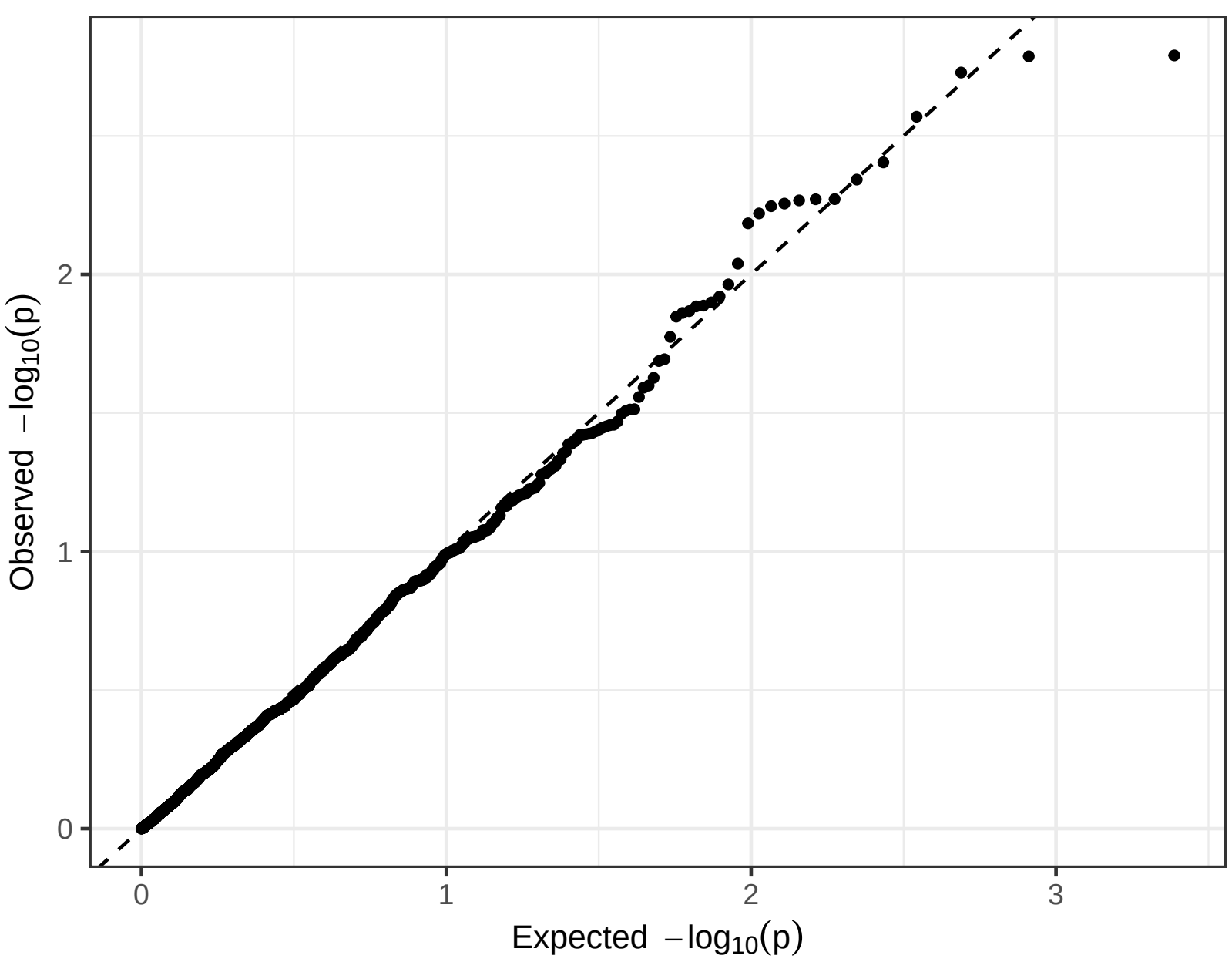

UCG.005

Feces | Liver | . = 1.14

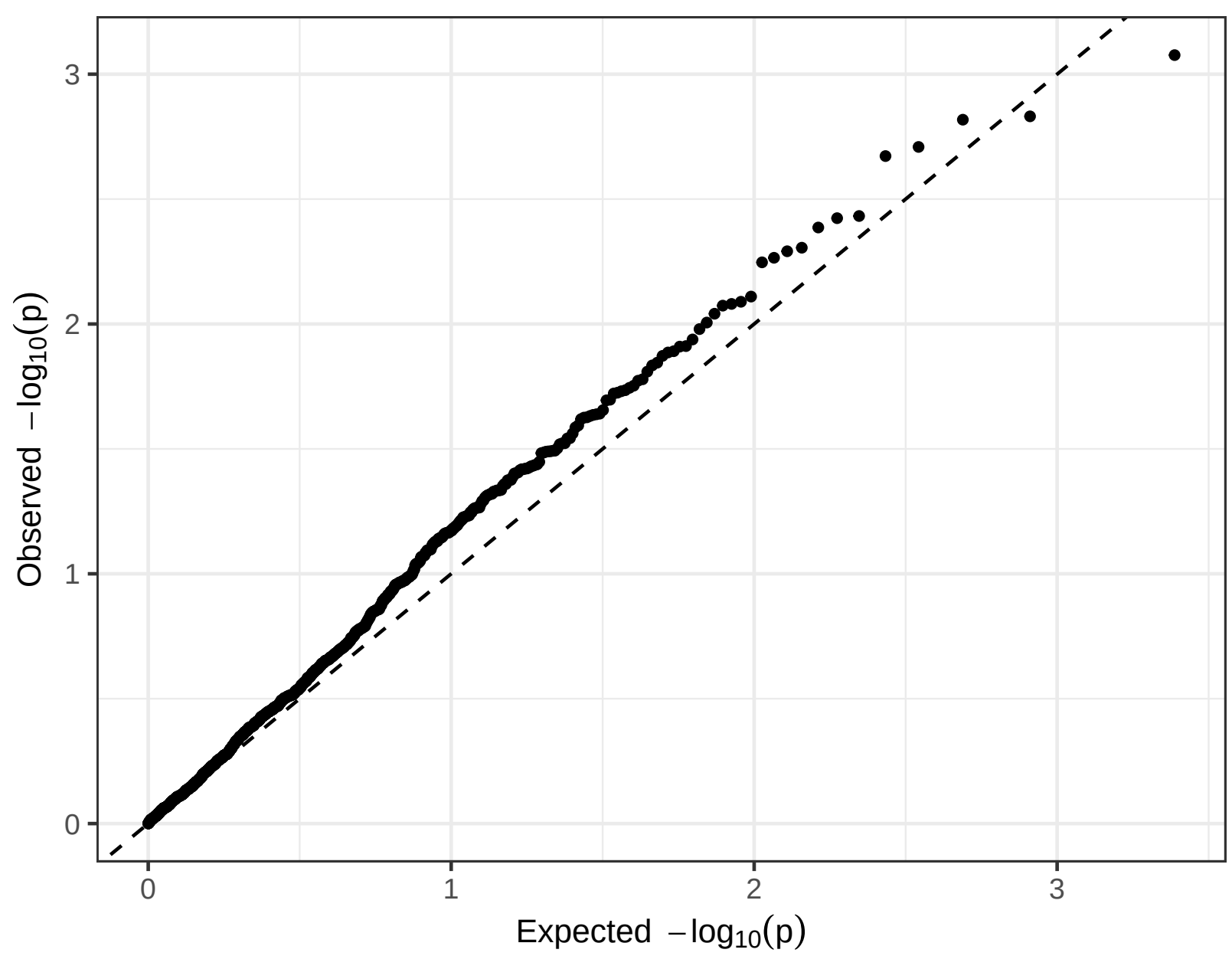

UCG.008

Feces | Liver | . = 0.896

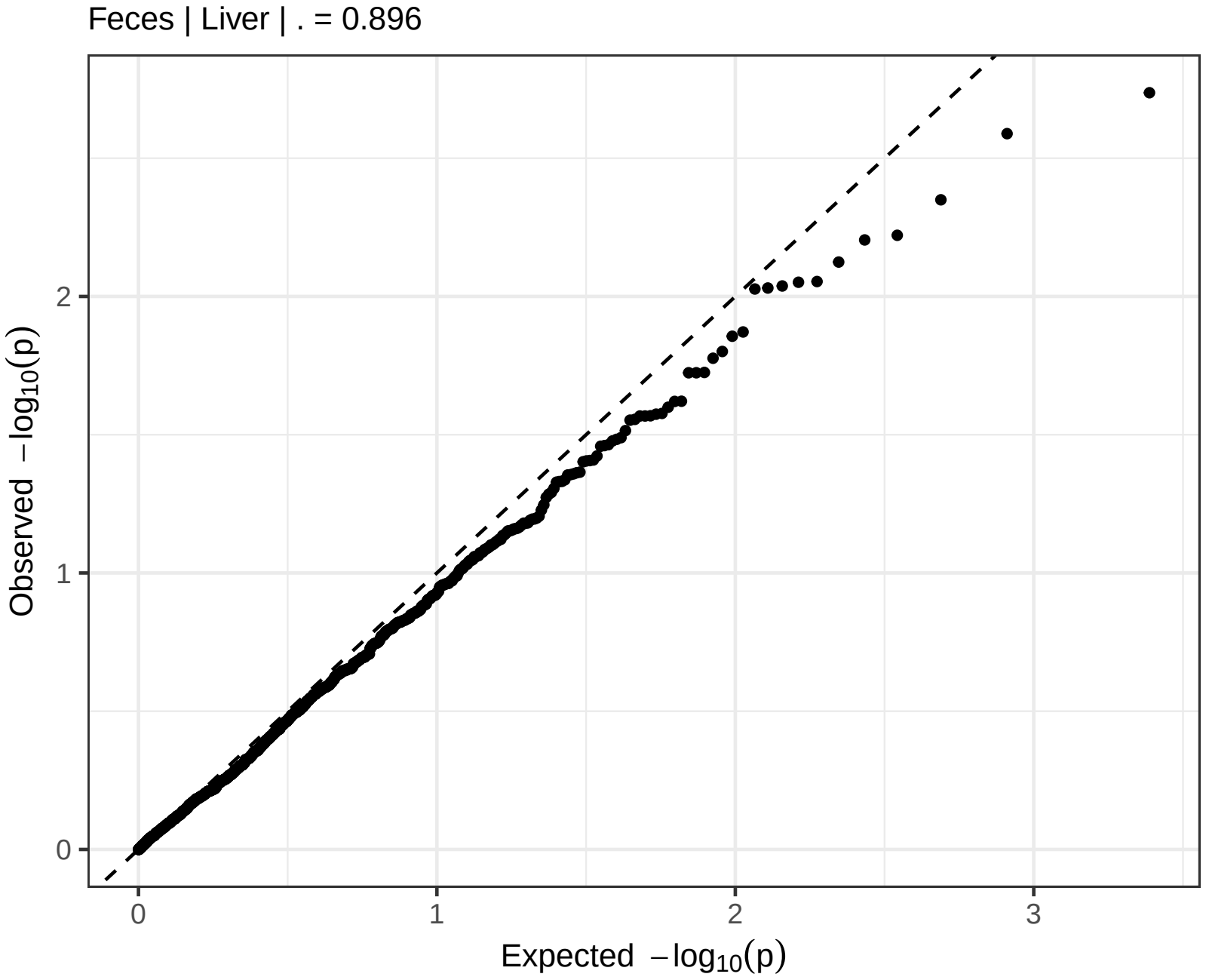

UCG.009

Feces | Liver | . = 0.856

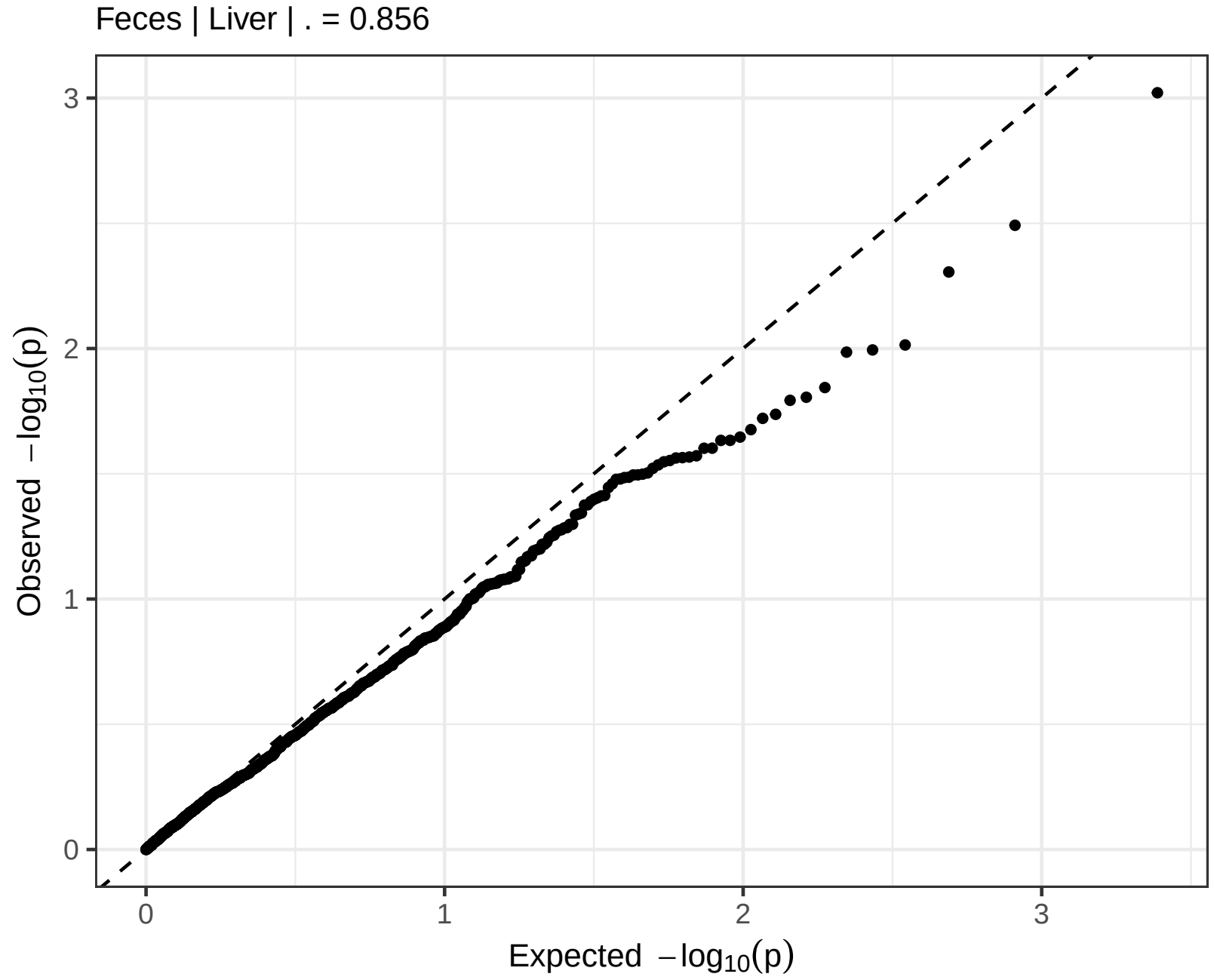

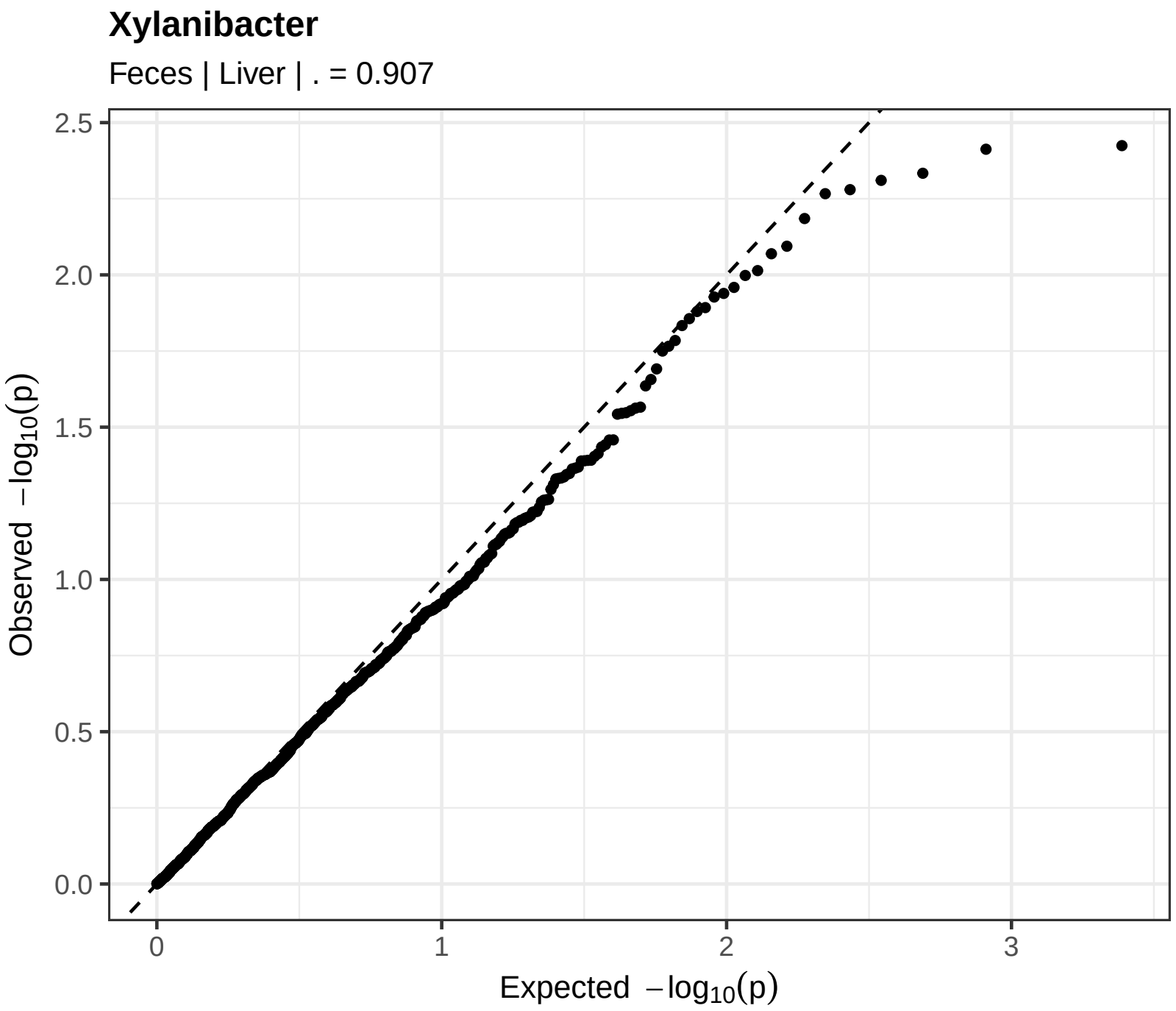
Xylanibacter
Feces | Liver | . = 0.907
Observed $-\log_{10}(p)$
Expected $-\log_{10}(p)$

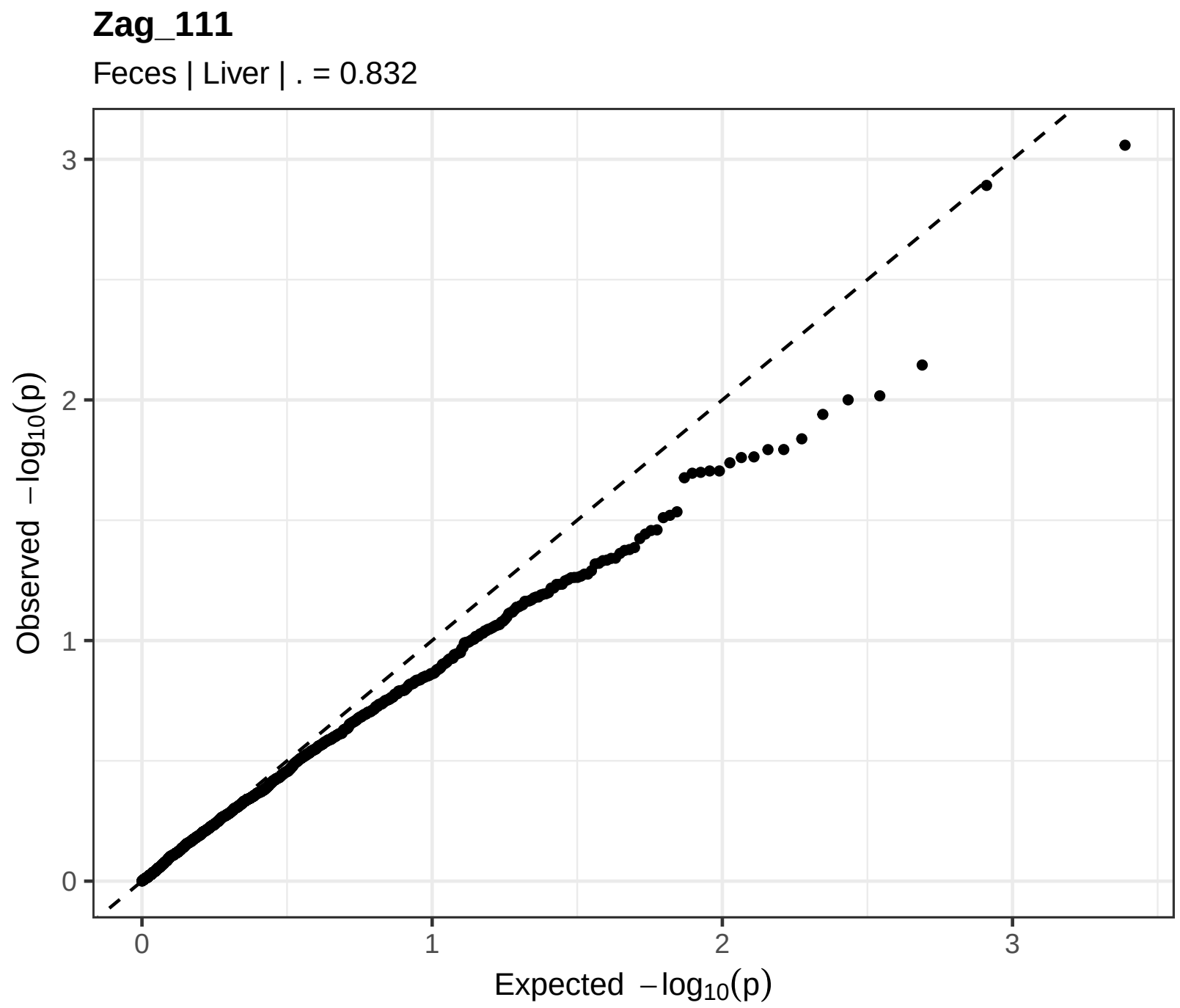
Zag_111
Feces | Liver | . = 0.832
Observed $-\log_{10}(p)$
Expected $-\log_{10}(p)$

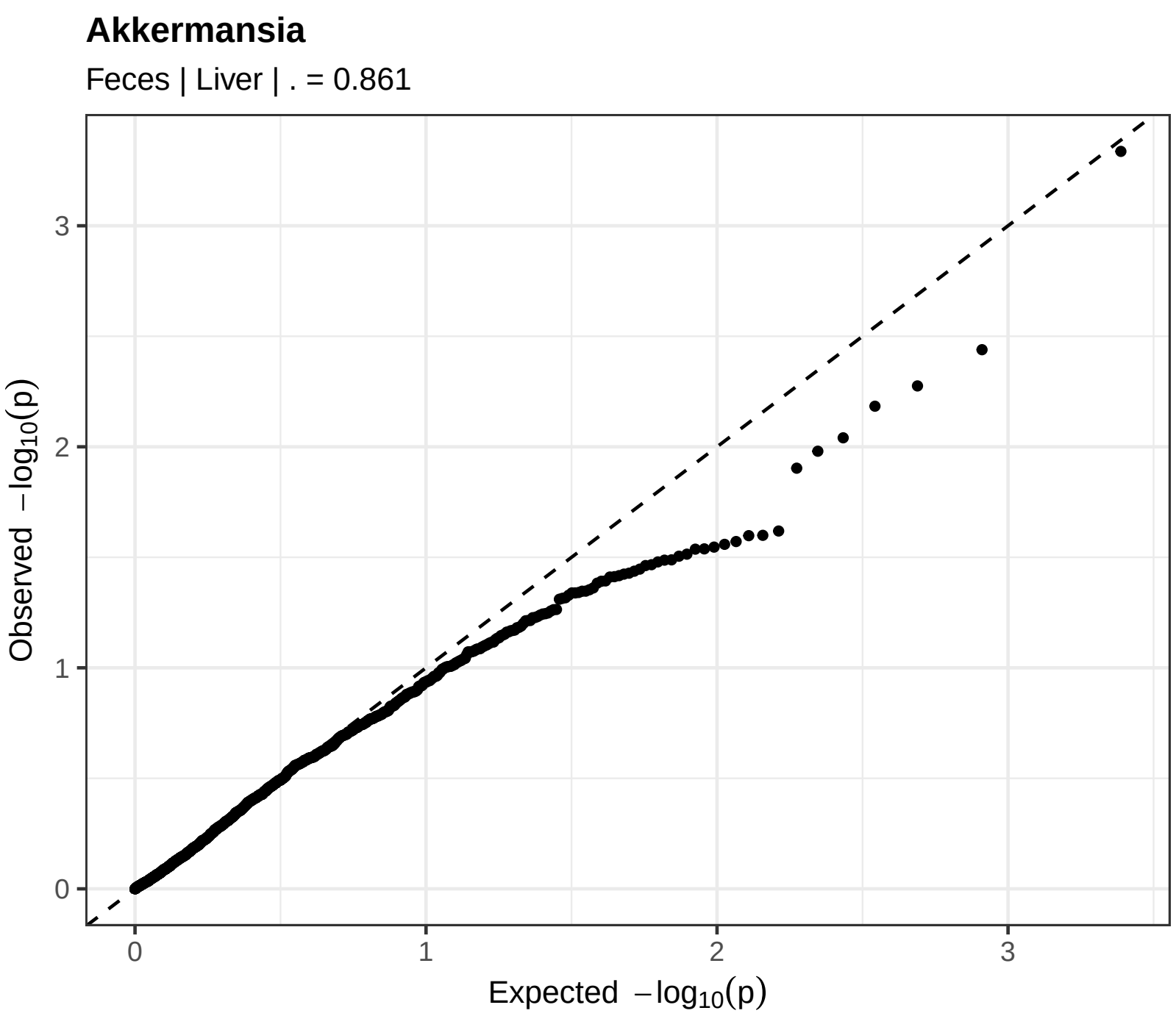
Akkermansia
Feces | Liver | . = 0.861
Observed $-\log_{10}(p)$
Expected $-\log_{10}(p)$

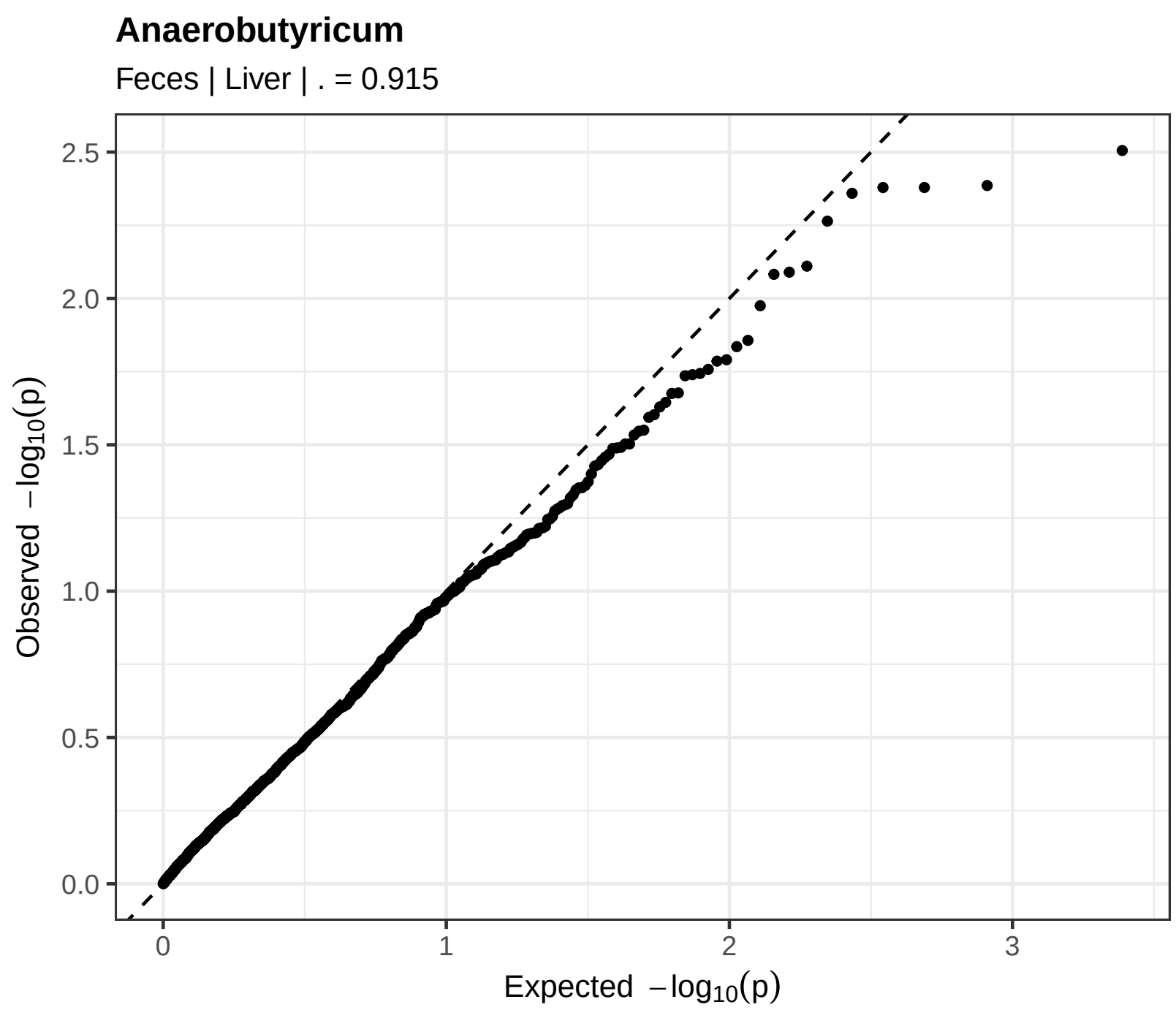
Anaerobutyricum
Feces | Liver | . = 0.915
Observed $-\log_{10}(p)$
Expected $-\log_{10}(p)$

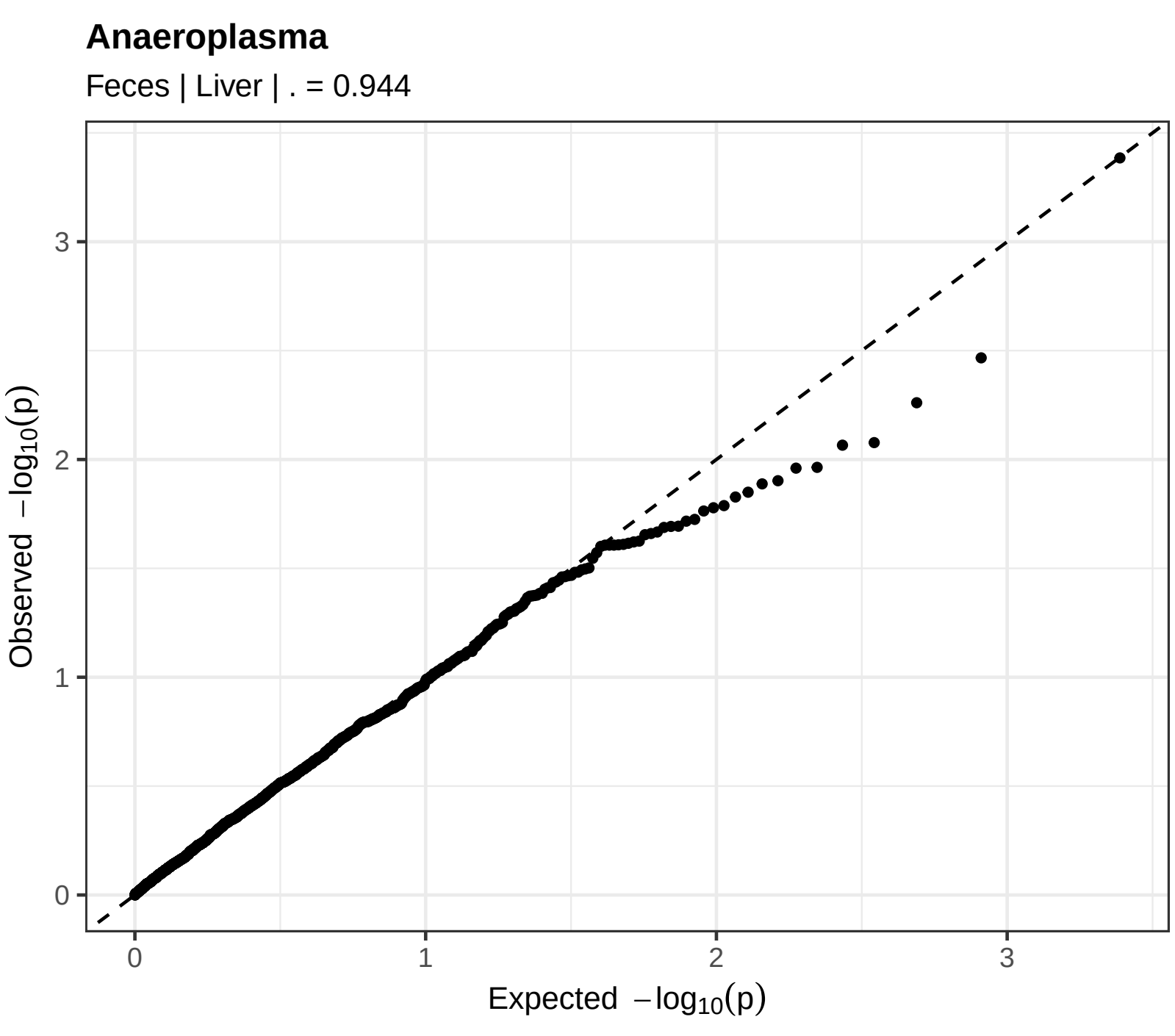
Anaeroplasma
Feces | Liver | . = 0.944
Observed $-\log_{10}(p)$
Expected $-\log_{10}(p)$

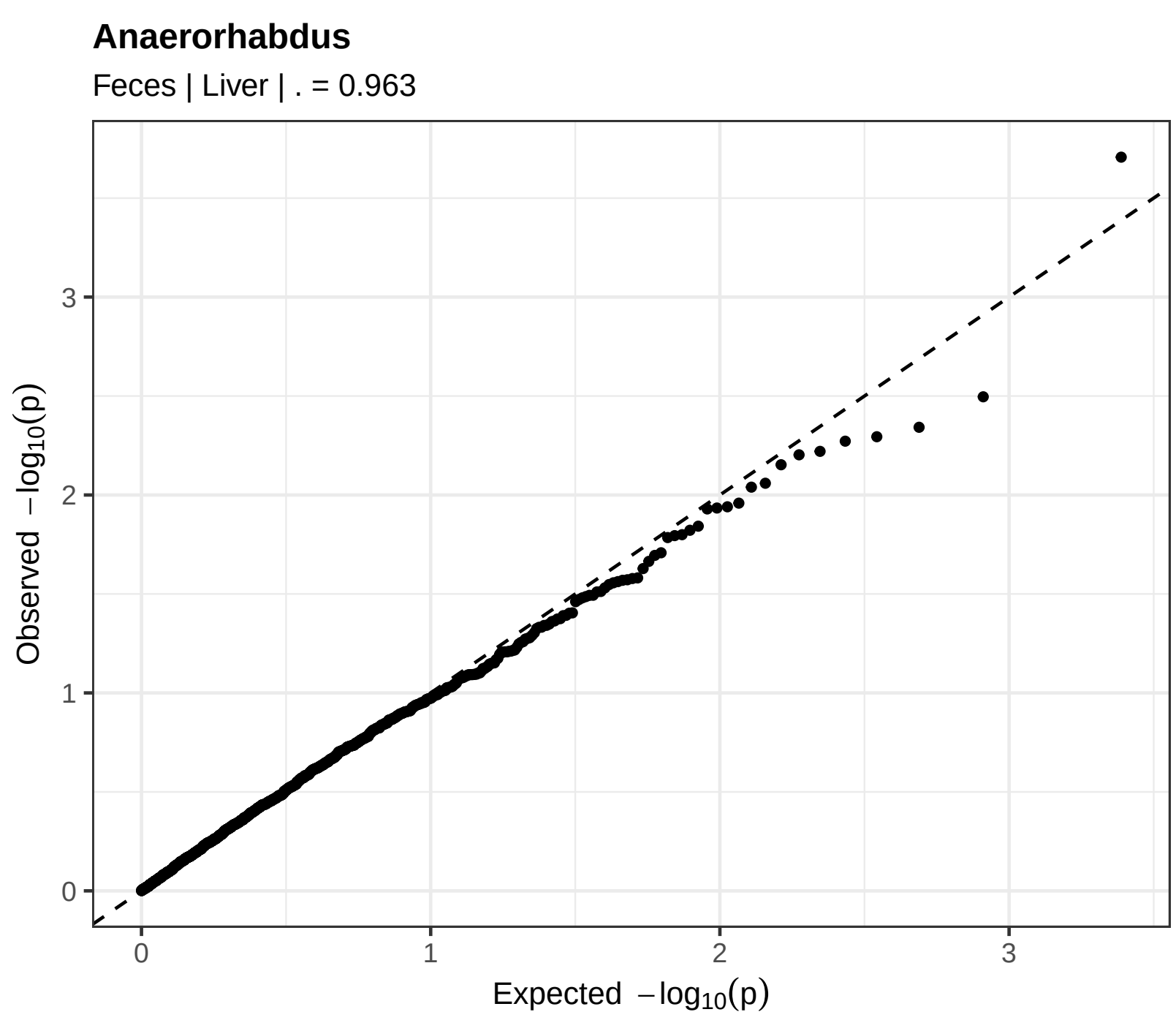
Anaerorhabdus
Feces | Liver | . = 0.963
Observed $-\log_{10}(p)$
Expected $-\log_{10}(p)$

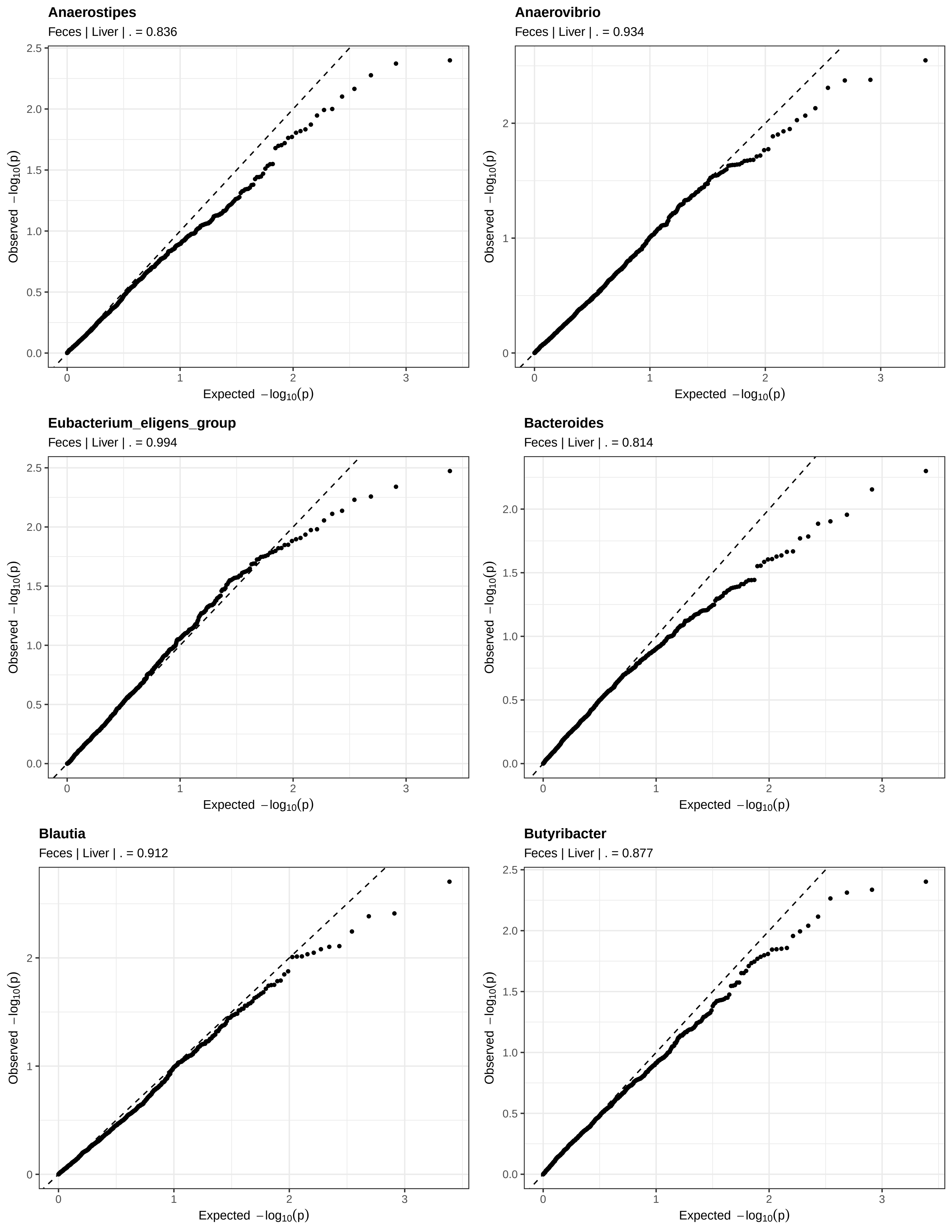
Anaerostipes
Feces | Liver | . = 0.836
Anaerovibrio
Feces | Liver | . = 0.934
Eubacterium_eligens_group
Feces | Liver | . = 0.994
Bacteroides
Feces | Liver | . = 0.814
Blautia
Feces | Liver | . = 0.912
Butyribacter
Feces | Liver | . = 0.877
Observed $-\log_{10}(p)$
Expected $-\log_{10}(p)$

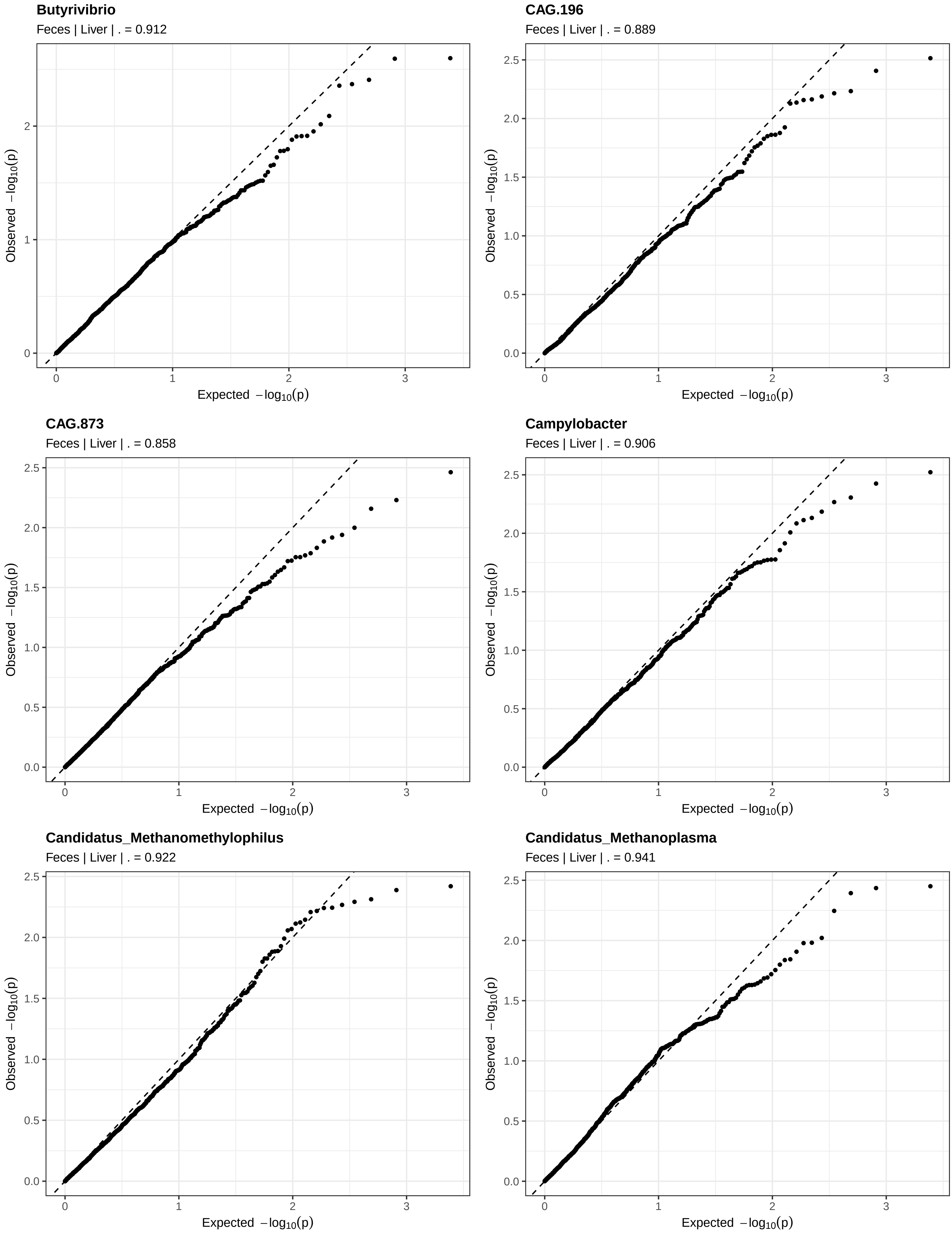
Butyrivibrio
Feces | Liver | . = 0.912
CAG.196
Feces | Liver | . = 0.889
CAG.873
Feces | Liver | . = 0.858
Campylobacter
Feces | Liver | . = 0.906
Candidatus_Methanomethylophilus
Feces | Liver | . = 0.922
Candidatus_Methanoplasma
Feces | Liver | . = 0.941
Observed −log10(p)
Expected −log10(p)

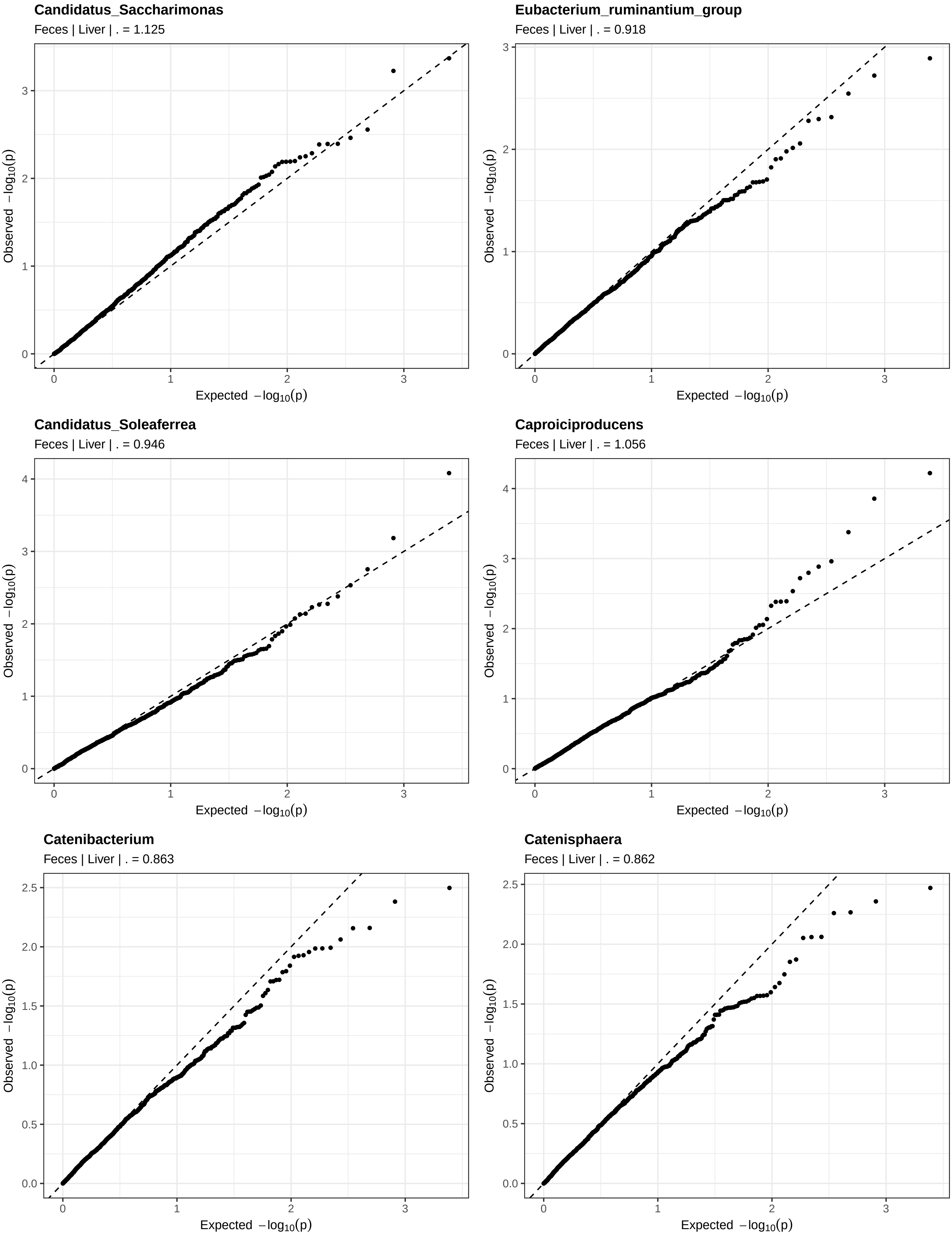

Candidatus_Saccharimonas
Feces | Liver | . = 1.125
Eubacterium_ruminantium_group
Feces | Liver | . = 0.918
Candidatus_Soleaferrea
Feces | Liver | . = 0.946
Caproiciproducens
Feces | Liver | . = 1.056
Catenibacterium
Feces | Liver | . = 0.863
Catenisphaera
Feces | Liver | . = 0.862
Observed $-\log_{10}(p)$
Expected $-\log_{10}(p)$

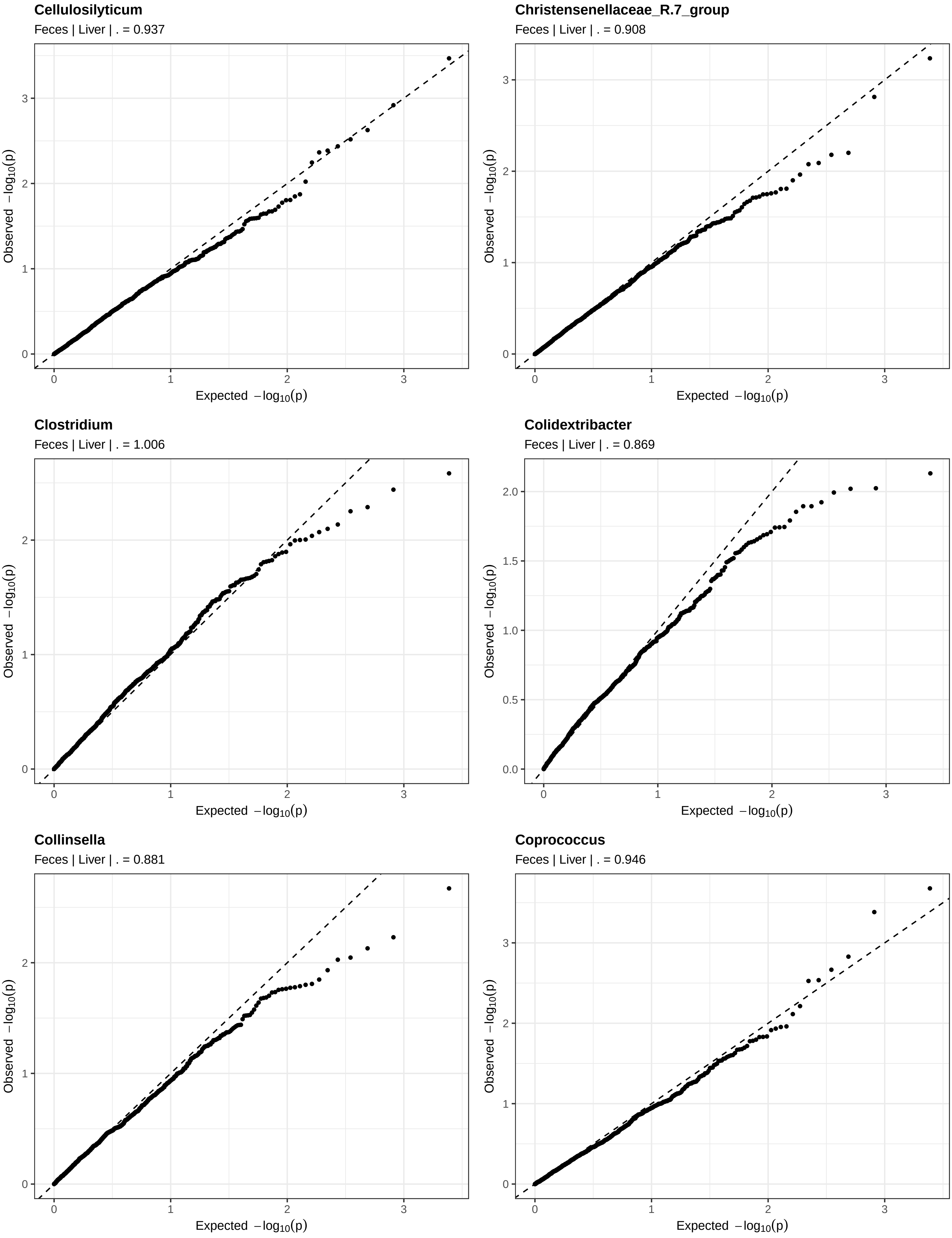
Cellulosilyticum
Feces | Liver | . = 0.937
Christensenellaceae_R.7_group
Feces | Liver | . = 0.908
Clostridium
Feces | Liver | . = 1.006
Colidextribacter
Feces | Liver | . = 0.869
Collinsella
Feces | Liver | . = 0.881
Coprococcus
Feces | Liver | . = 0.946
Observed −log10(p)
Expected −log10(p)

Eubacterium_siraeum_group

Feces | Liver | . = 0.919

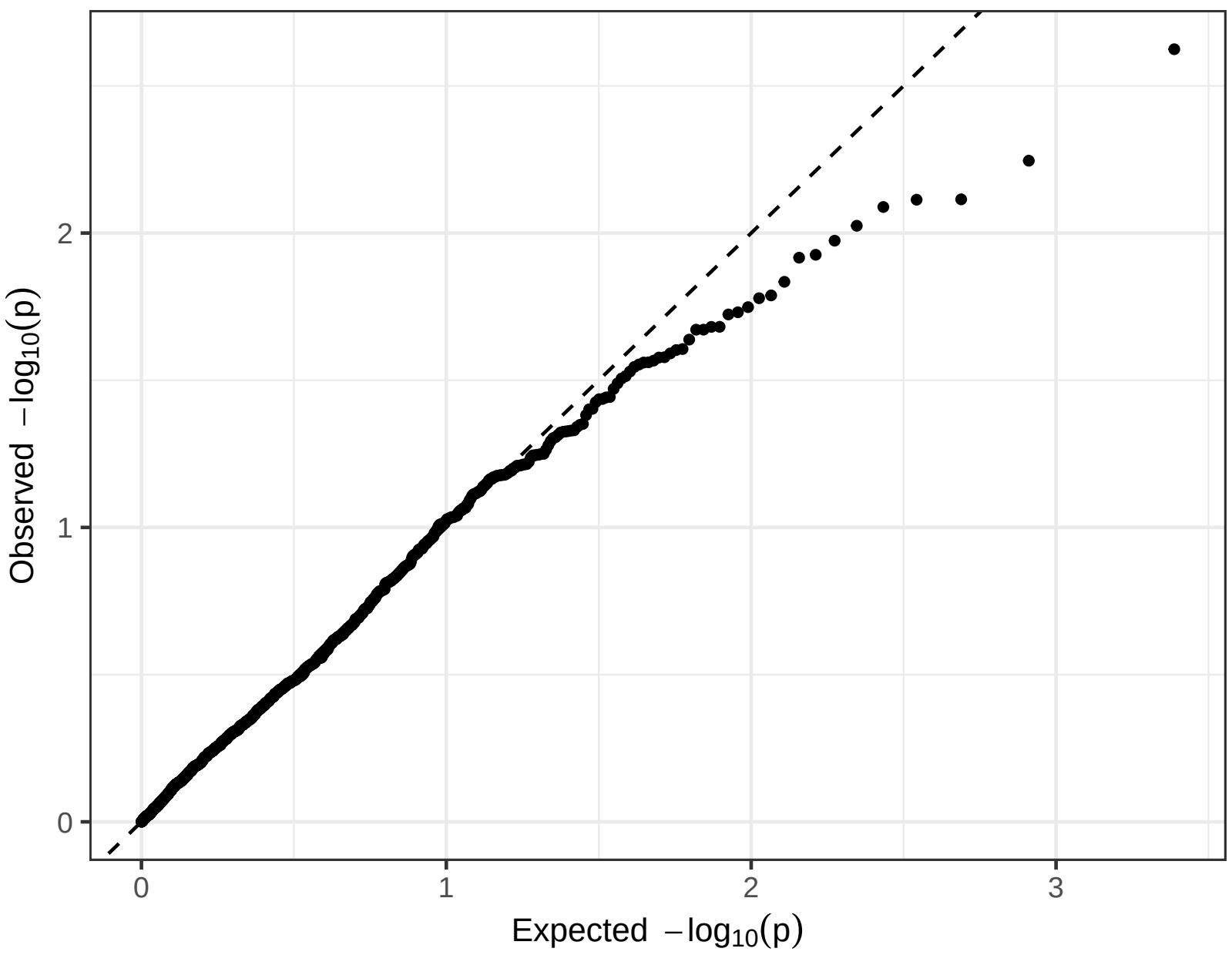

Desulfovibrio

Feces | Liver | . = 0.862

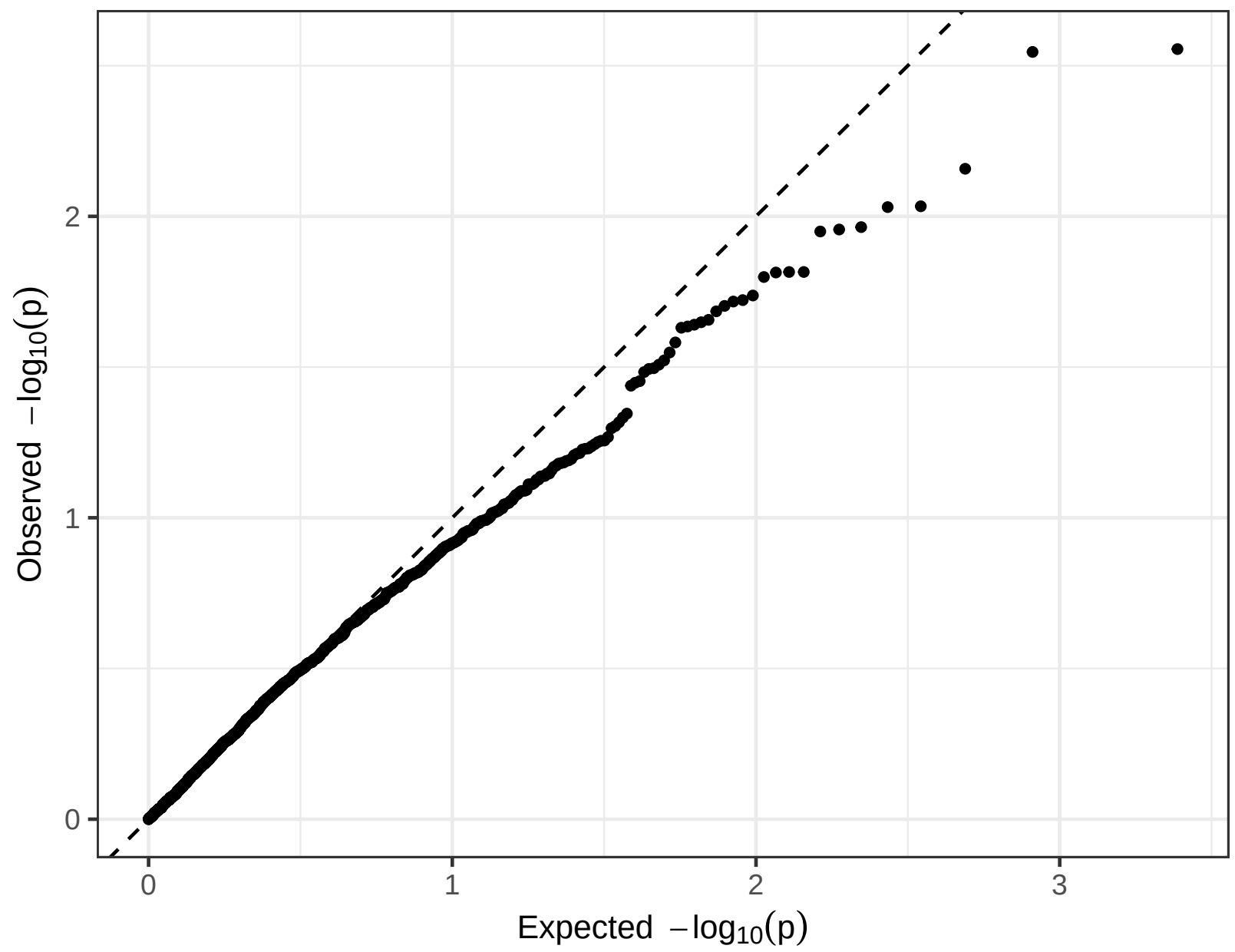

dgA.11_gut_group

Feces | Liver | . = 0.834

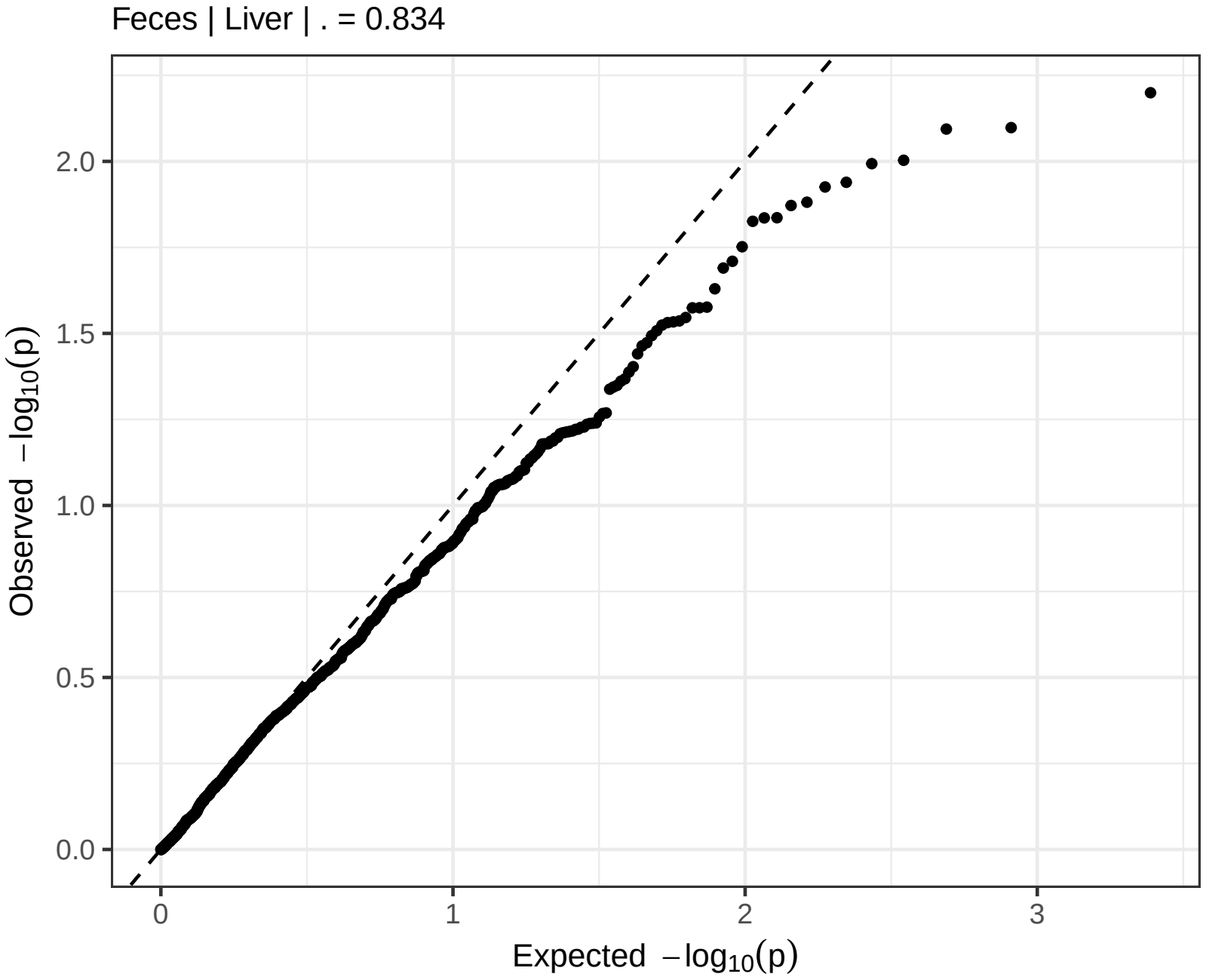

Dorea

Feces | Liver | . = 0.888

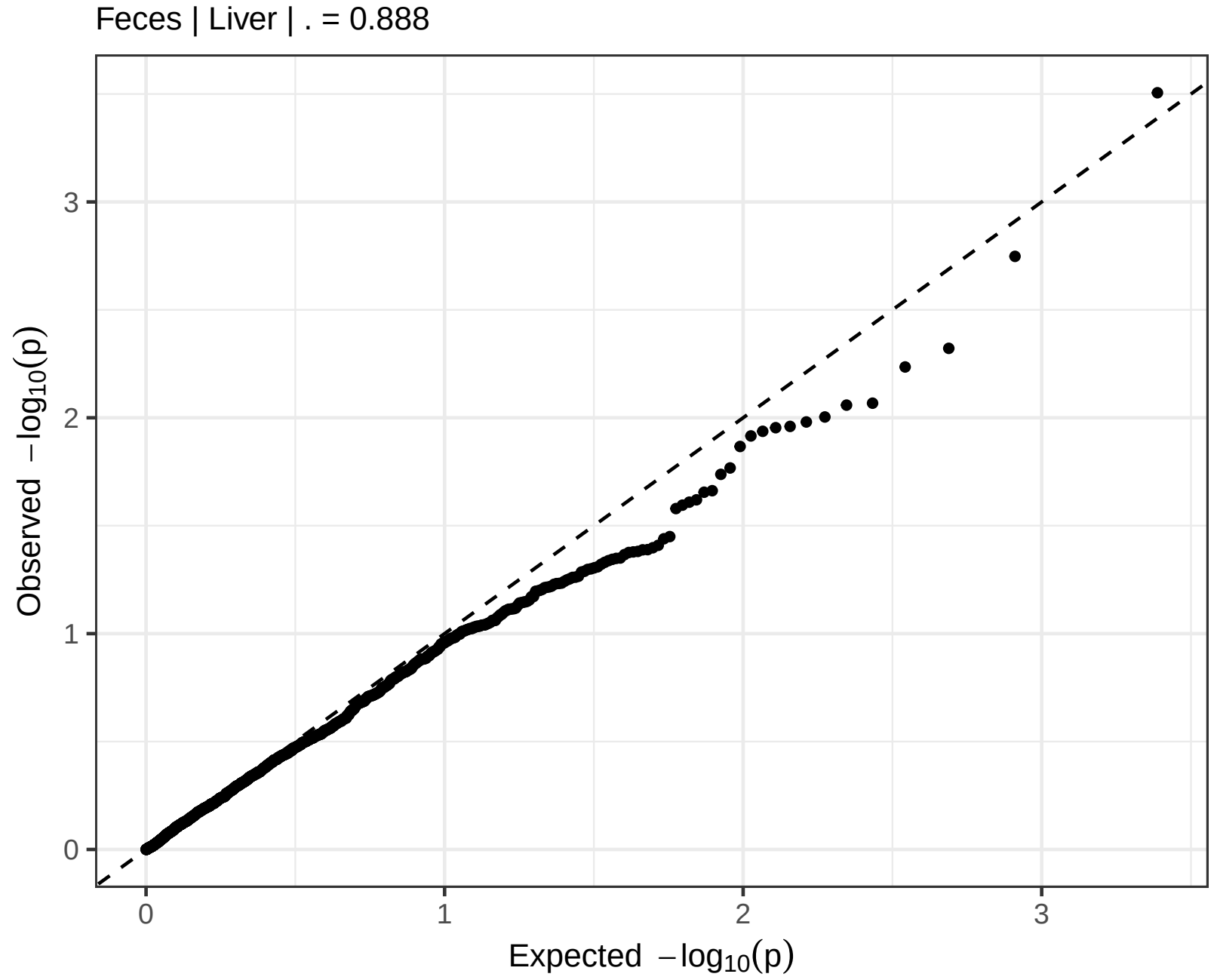

Erysipelotrichaceae_UCG.003

Feces | Liver | . = 0.976

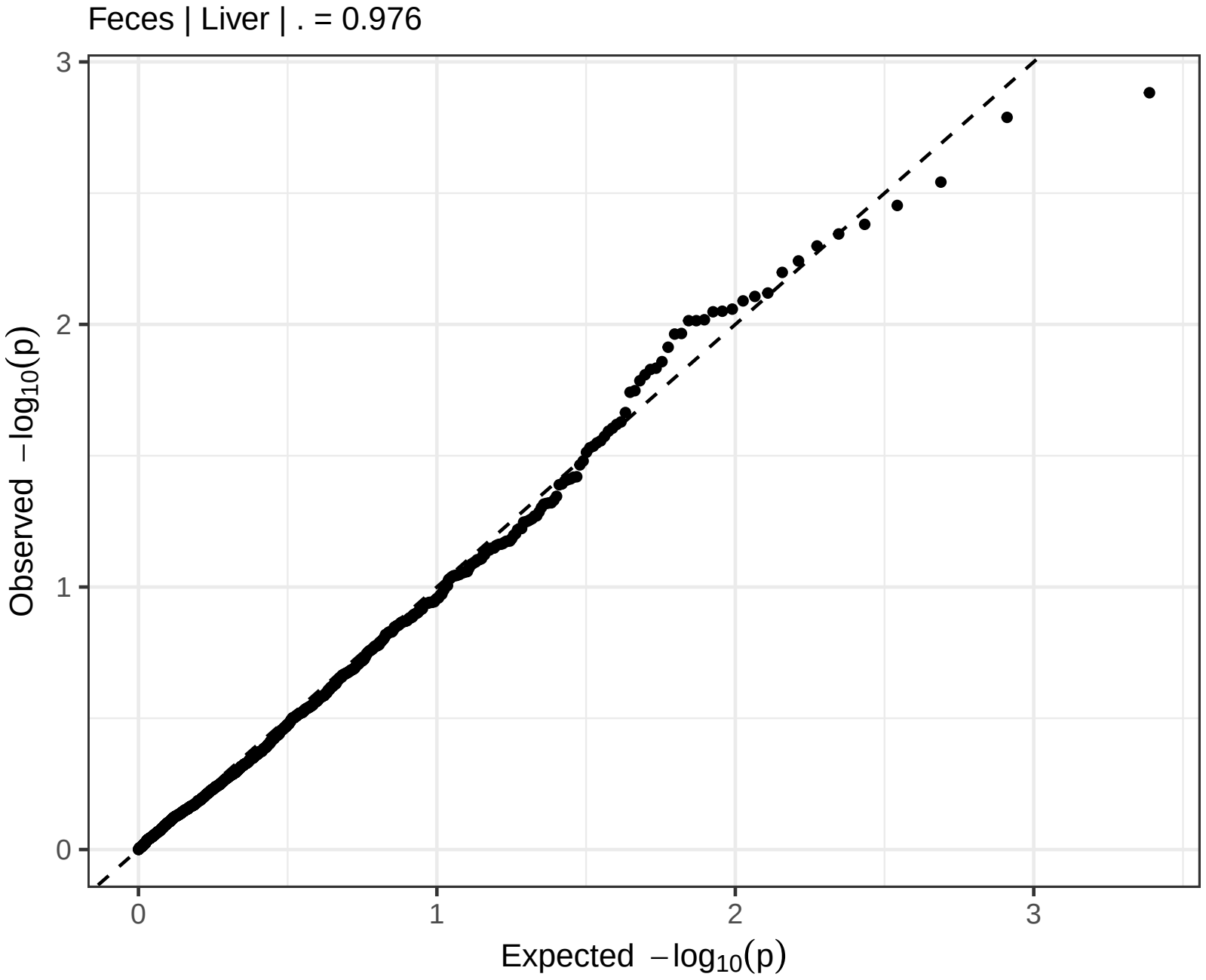

Erysipelotrichaceae_UCG.006

Feces | Liver | . = 0.857

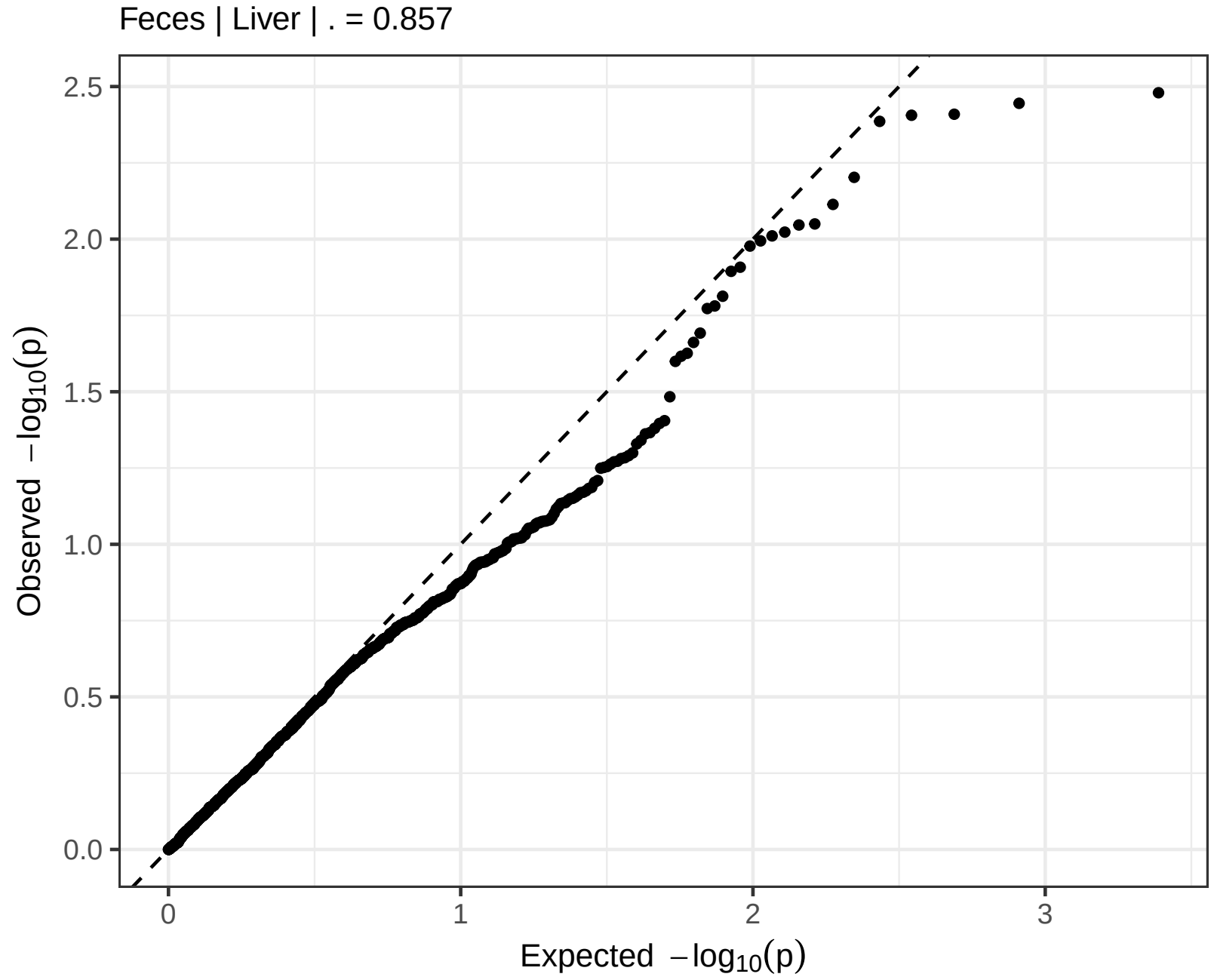

Escherichia.Shigella

Feces | Liver | . = 0.893

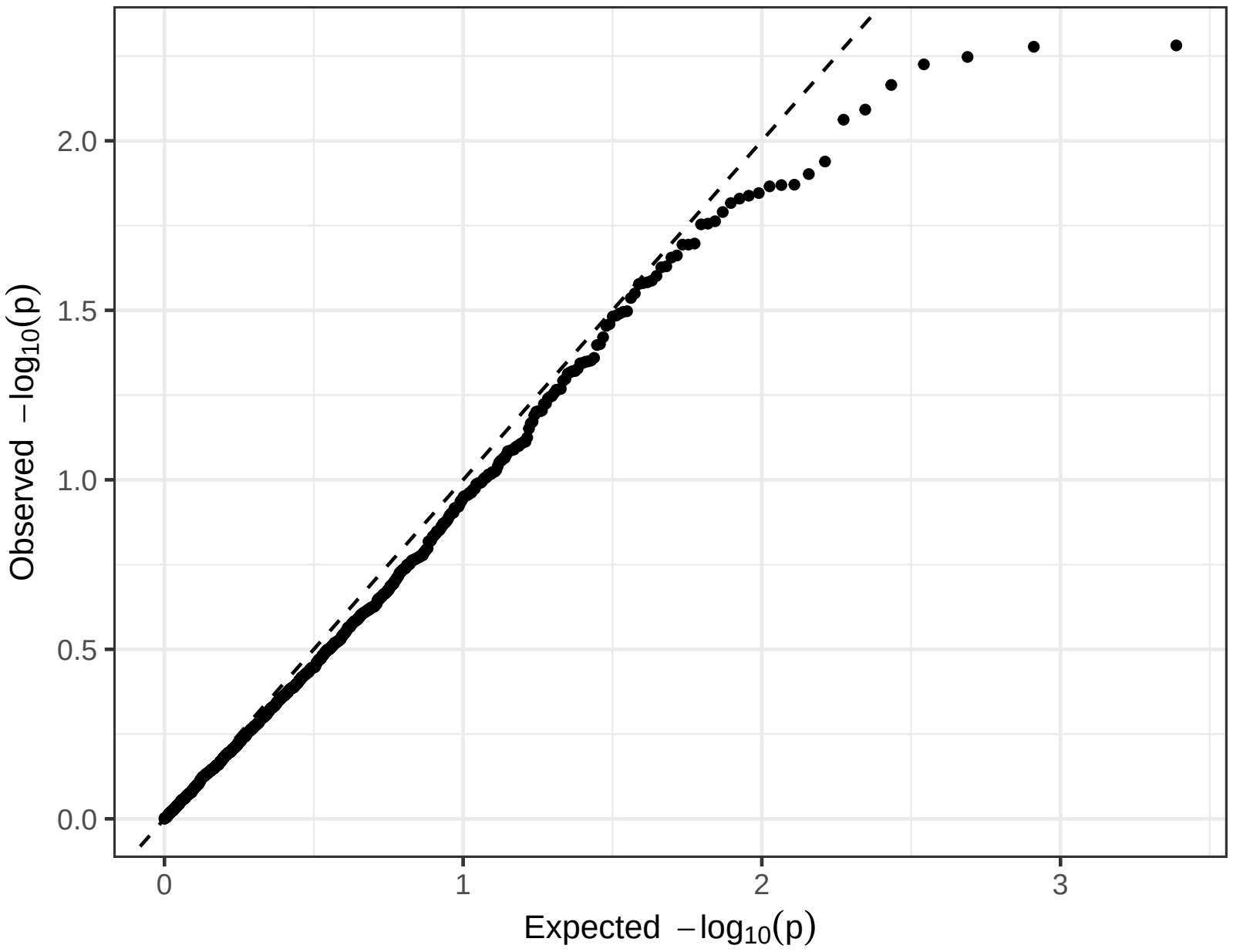

Extibacter

Feces | Liver | . = 0.917

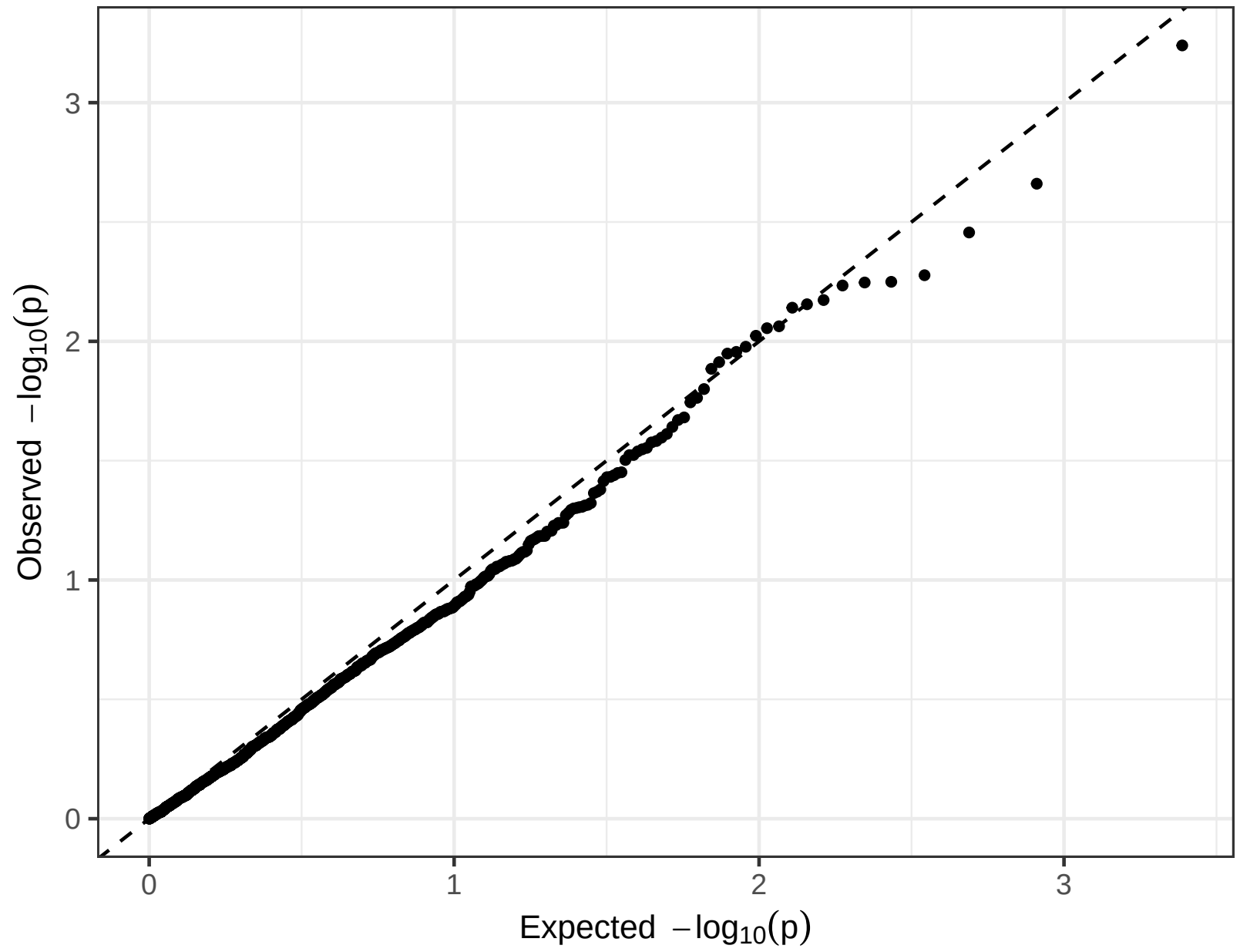

Faecalibacterium

Feces | Liver | . = 0.943

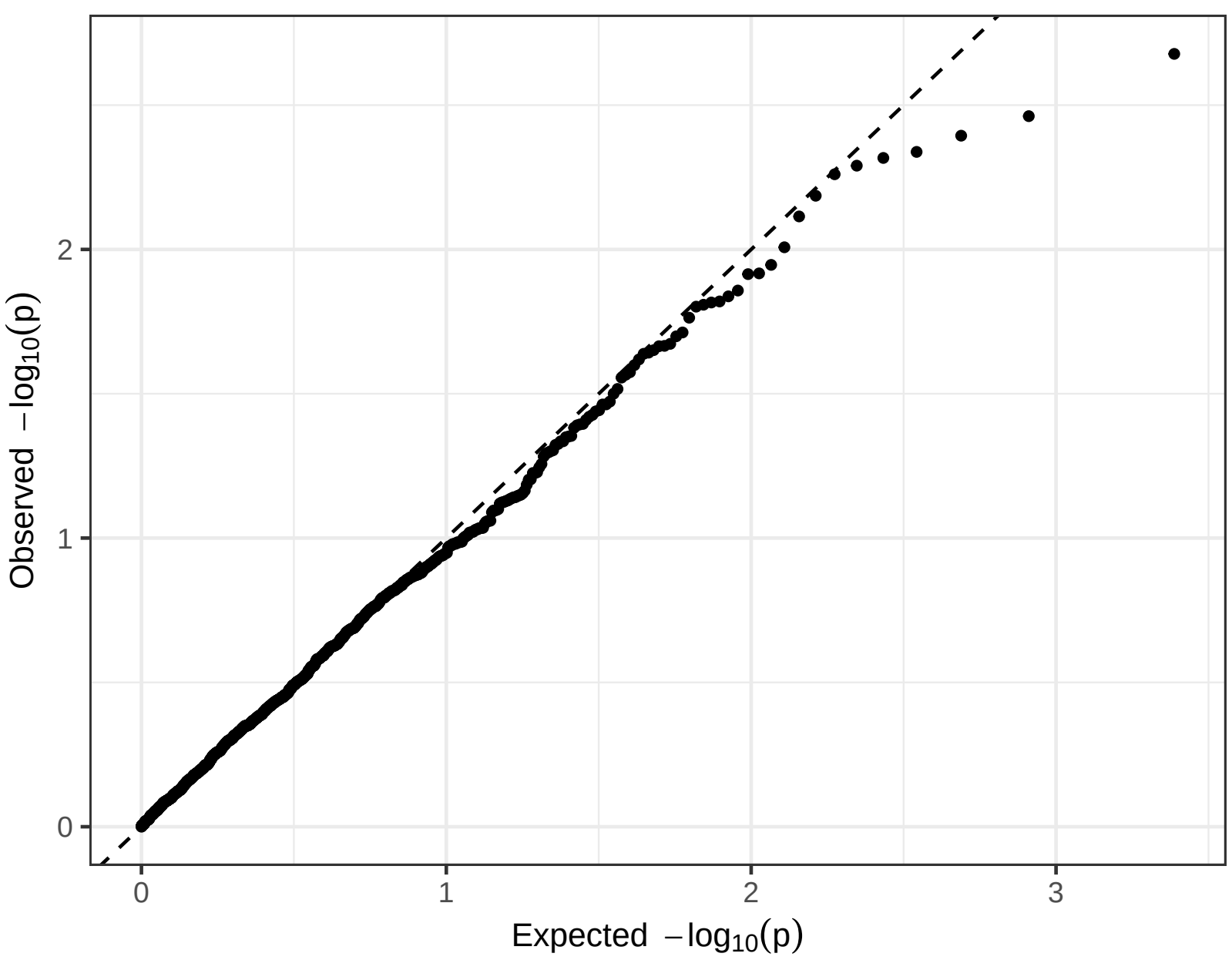

Faecalicatena

Feces | Liver | . = 0.982

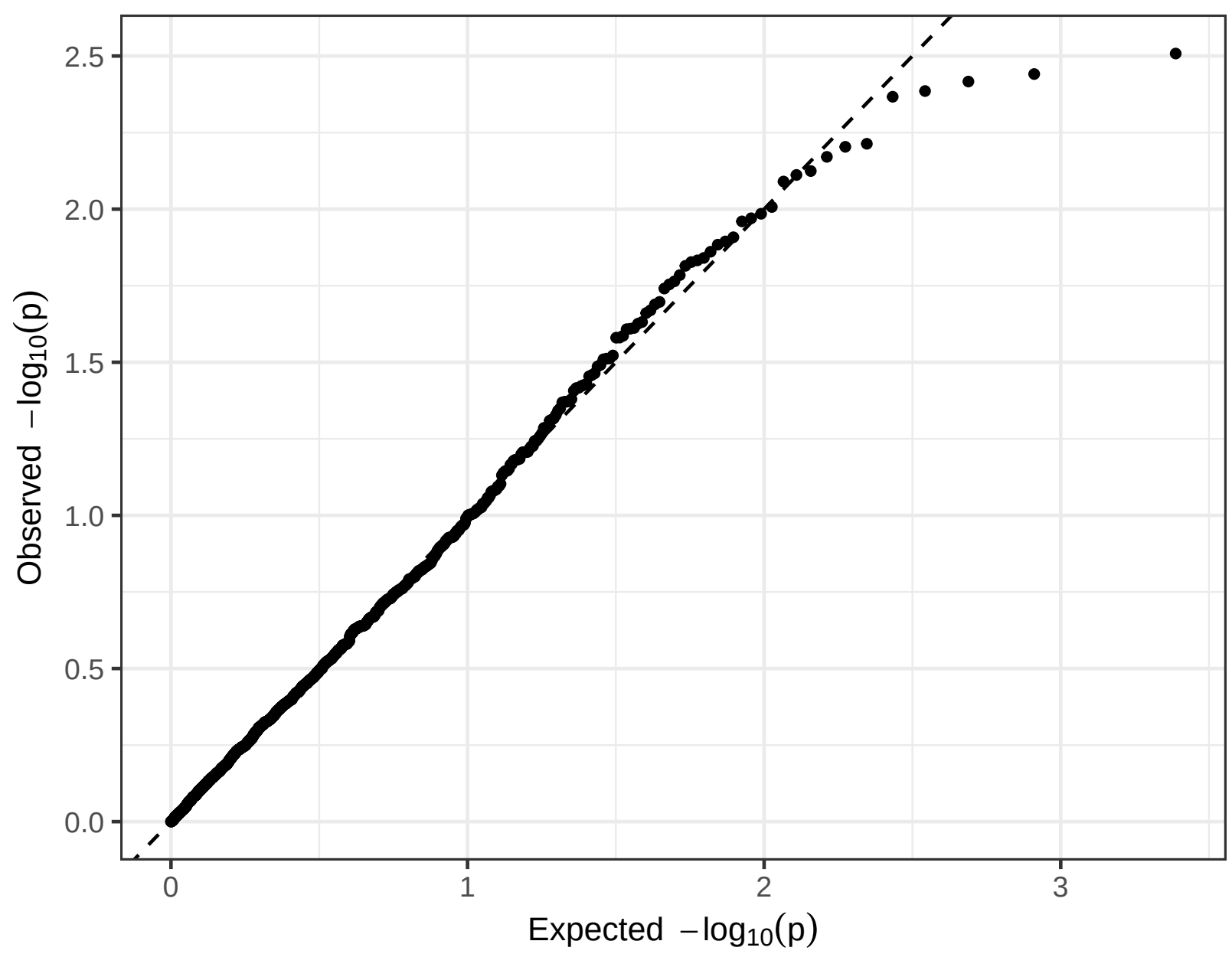

Family_XIII_AD3011_group

Feces | Liver | . = 0.891

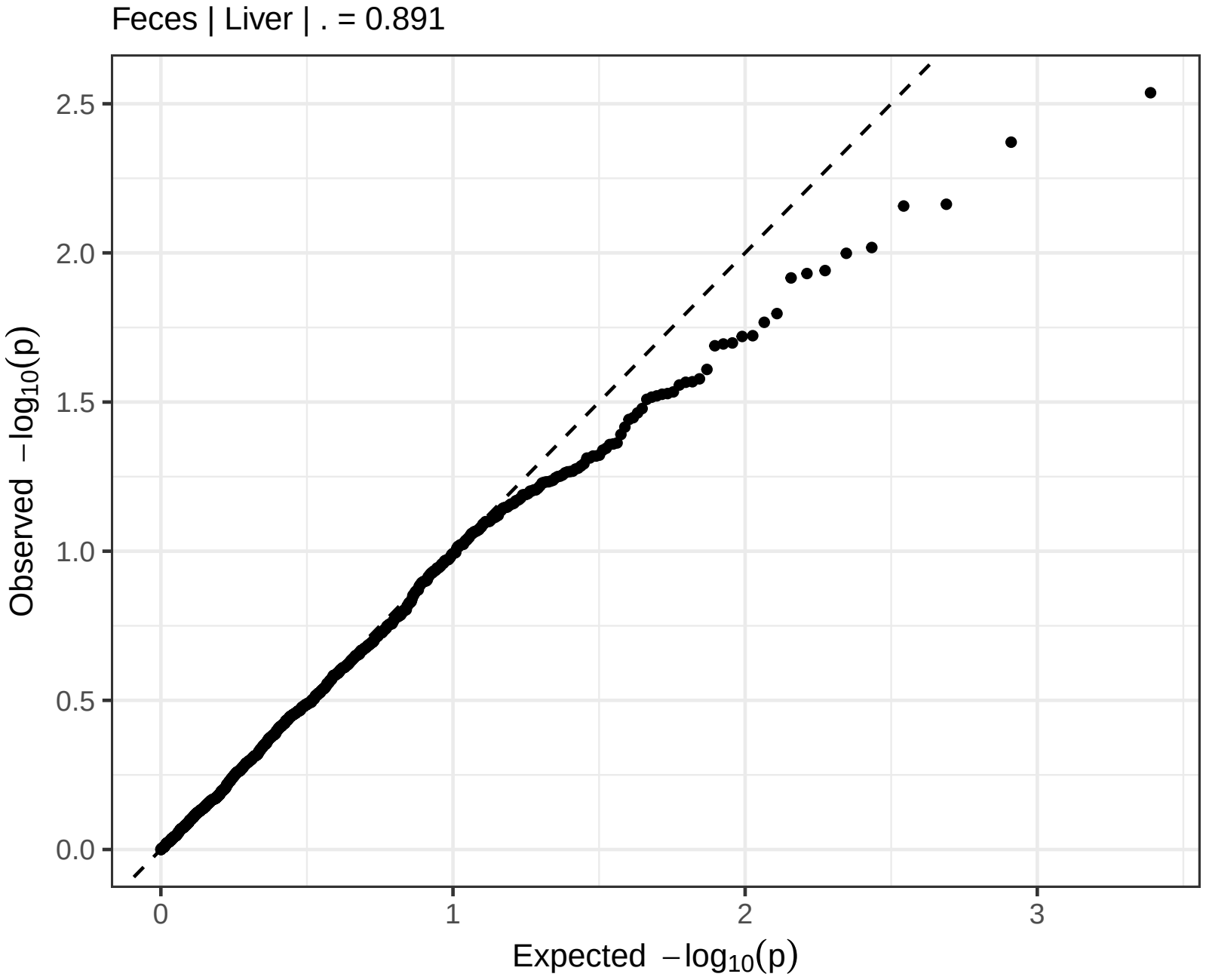

Eubacterium_xylanophilum_group

Feces | Liver | . = 1.1

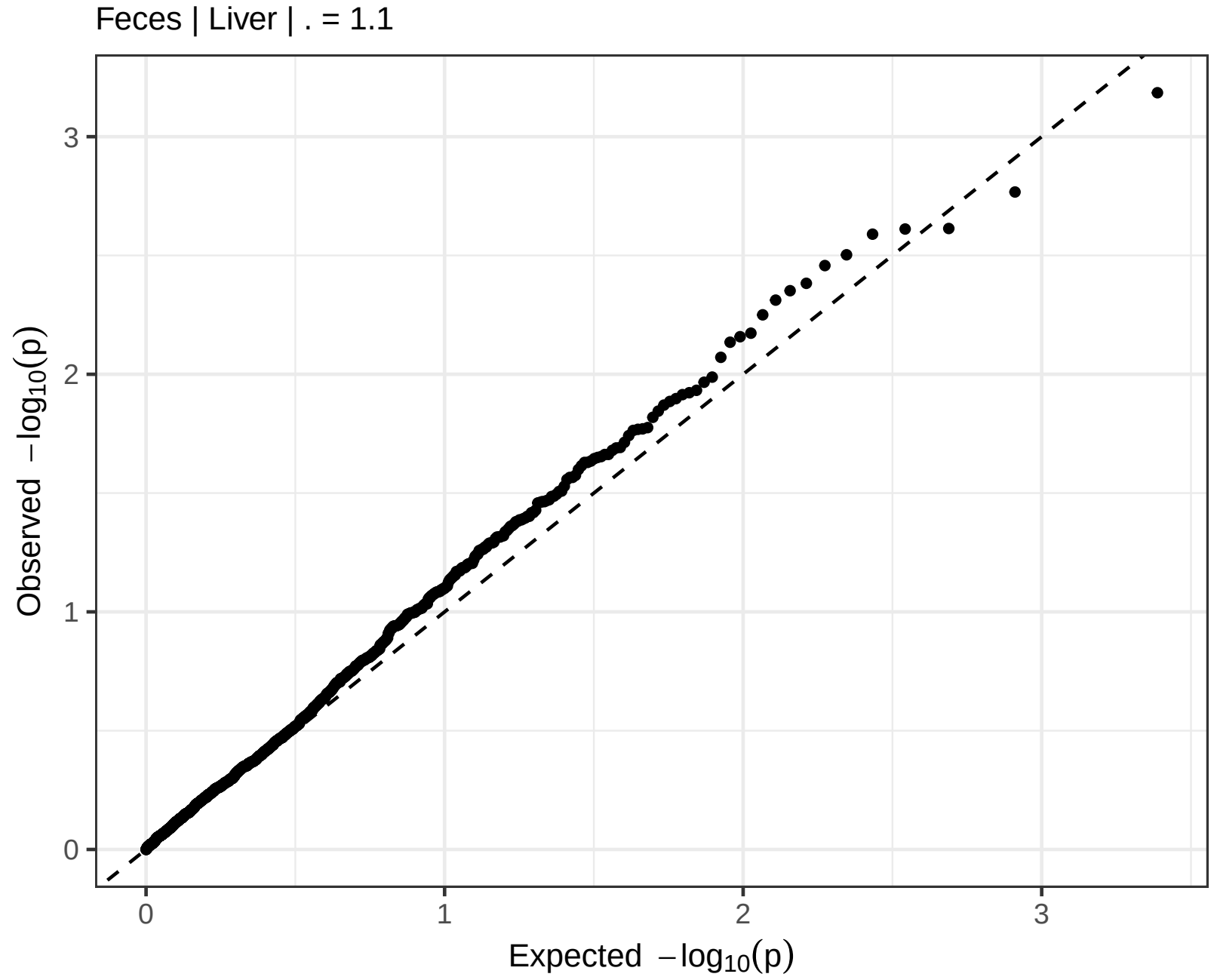

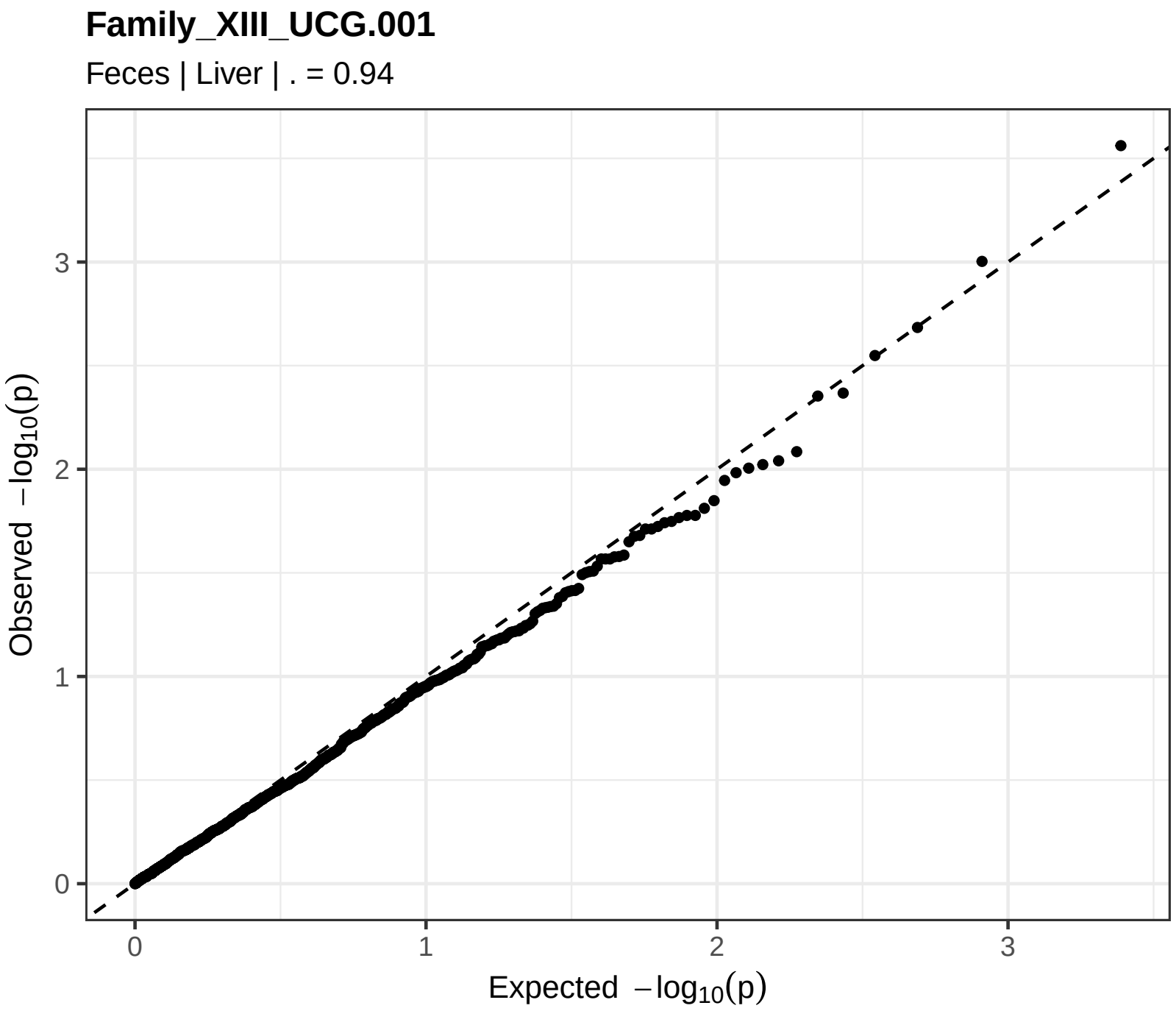
Family_XIII_UCG.001
Feces | Liver | . = 0.94
Observed $-\log_{10}(p)$
Expected $-\log_{10}(p)$

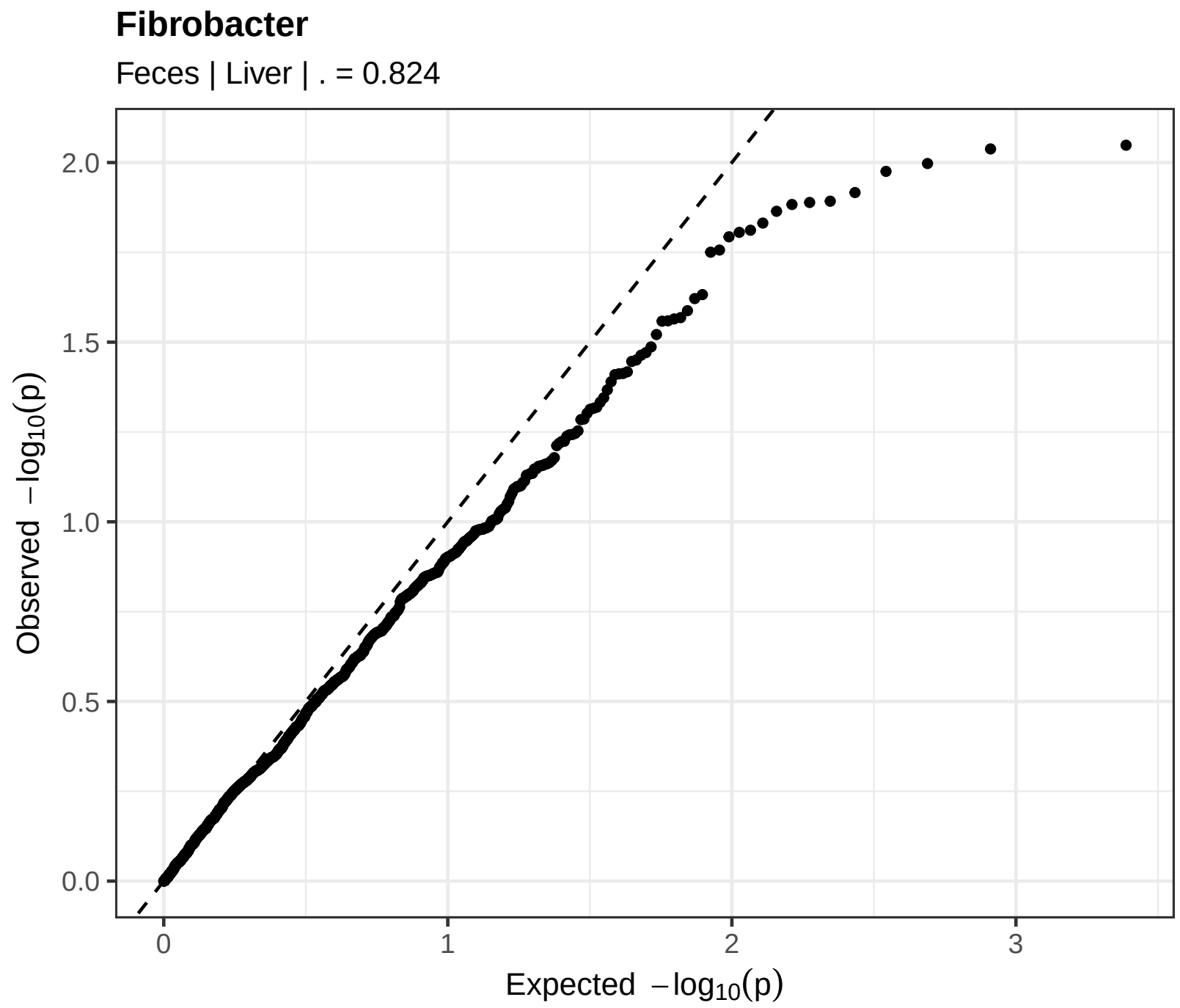
Fibrobacter
Feces | Liver | . = 0.824
Observed $-\log_{10}(p)$
Expected $-\log_{10}(p)$

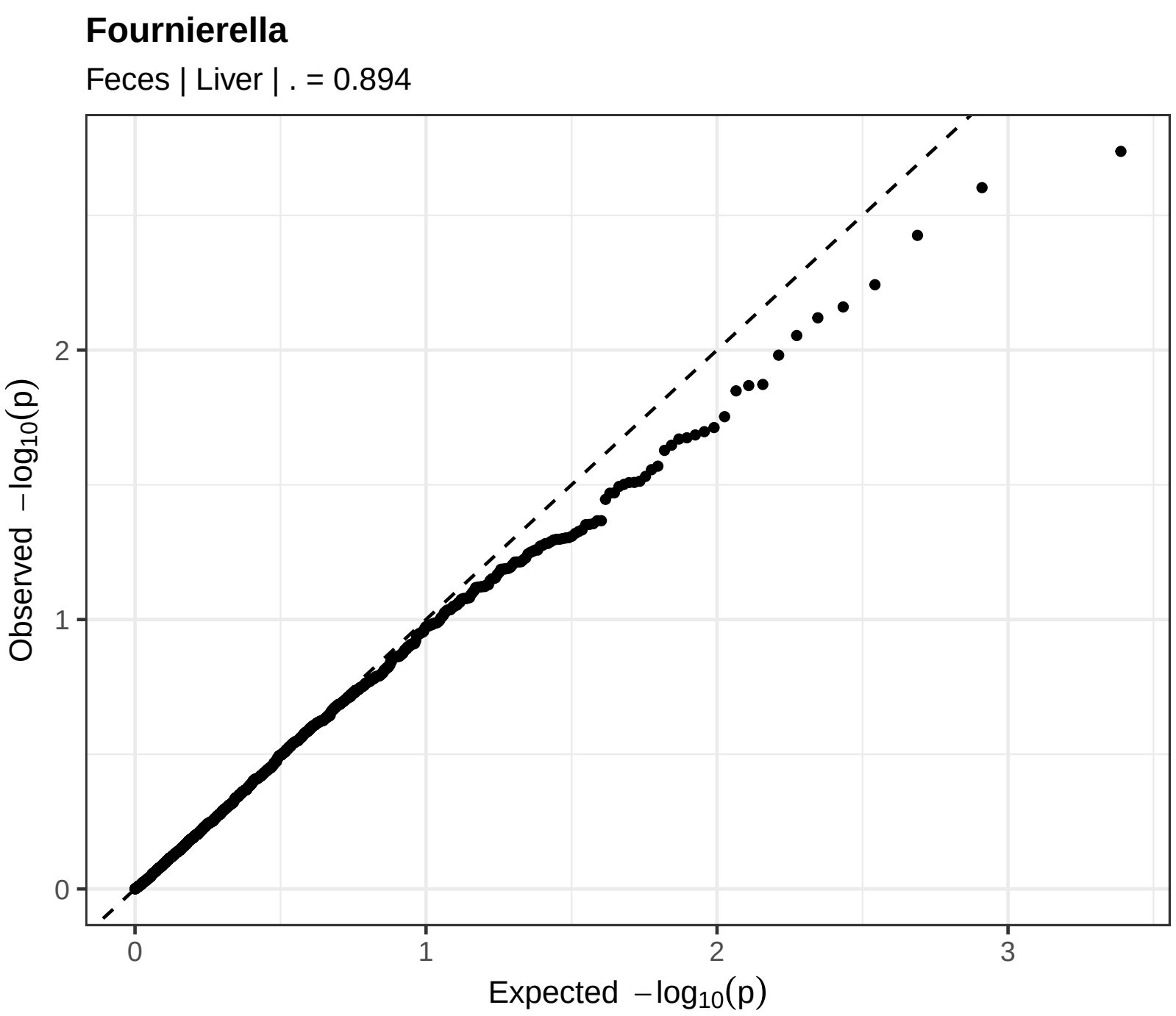
Fournierella
Feces | Liver | . = 0.894
Observed $-\log_{10}(p)$
Expected $-\log_{10}(p)$

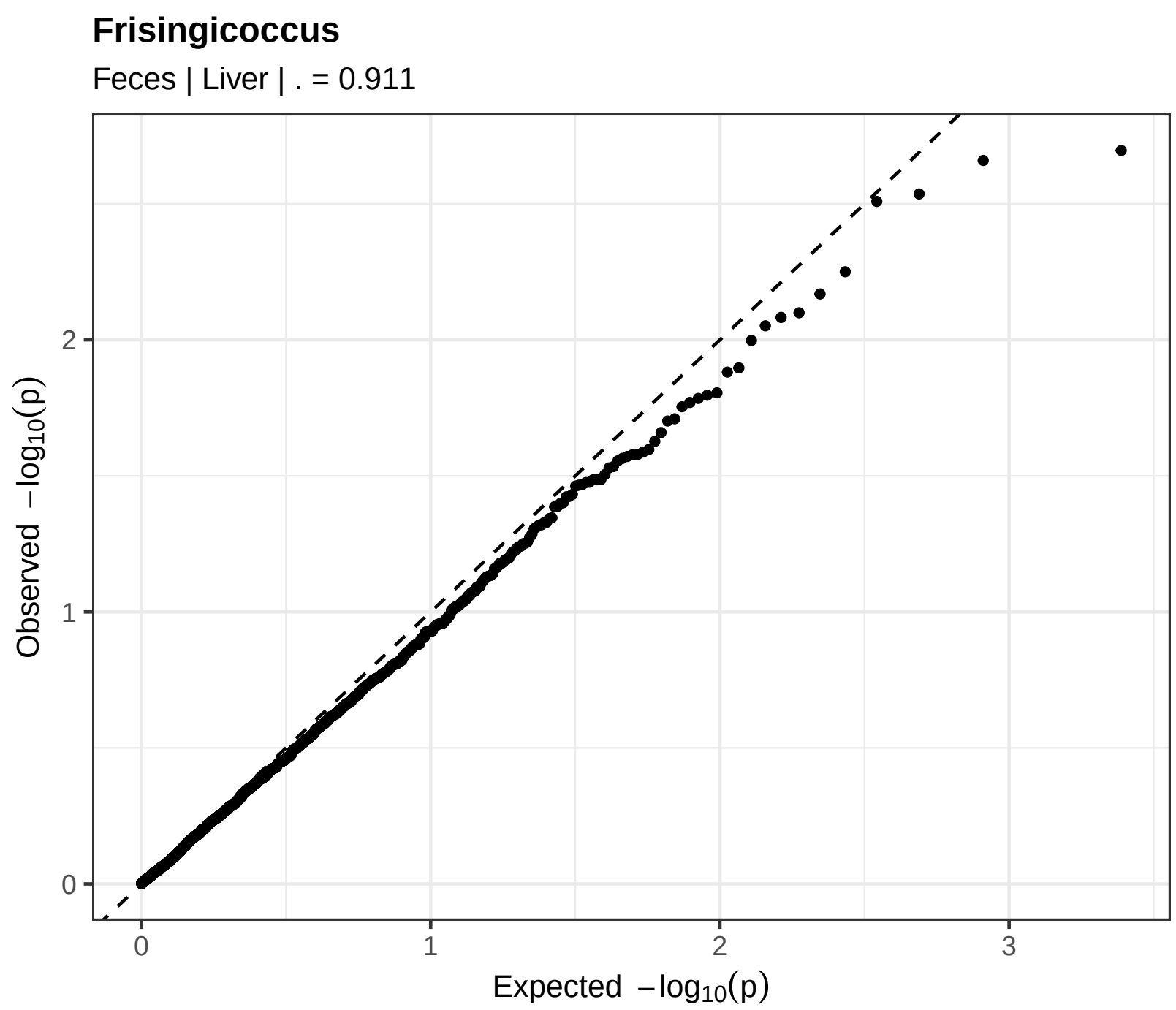
Frisingicoccus
Feces | Liver | . = 0.911
Observed $-\log_{10}(p)$
Expected $-\log_{10}(p)$

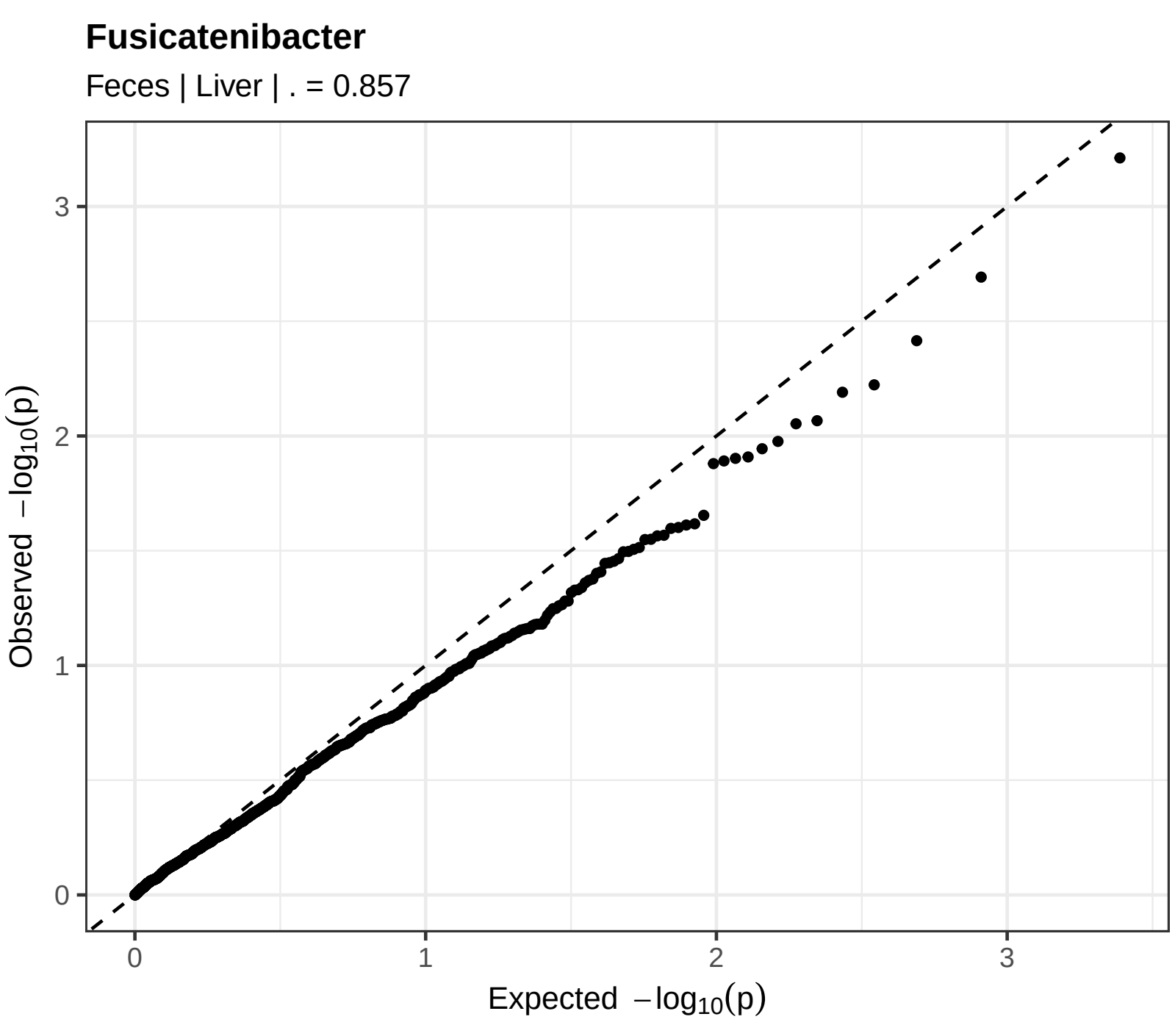
Fusicatenibacter
Feces | Liver | . = 0.857
Observed $-\log_{10}(p)$
Expected $-\log_{10}(p)$

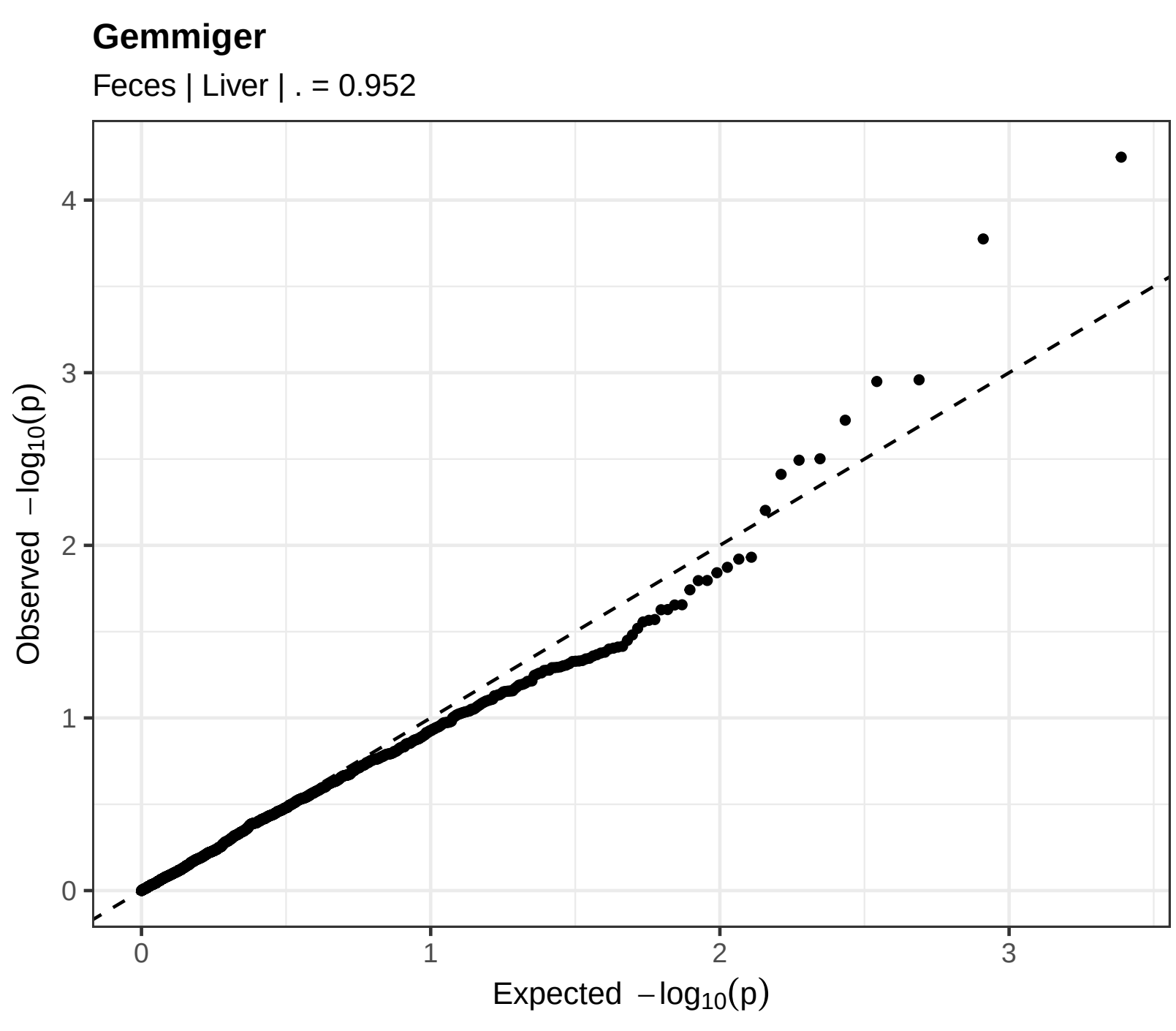
Gemmiger
Feces | Liver | . = 0.952
Observed $-\log_{10}(p)$
Expected $-\log_{10}(p)$

Helicobacter

Feces | Liver | . = 1.063

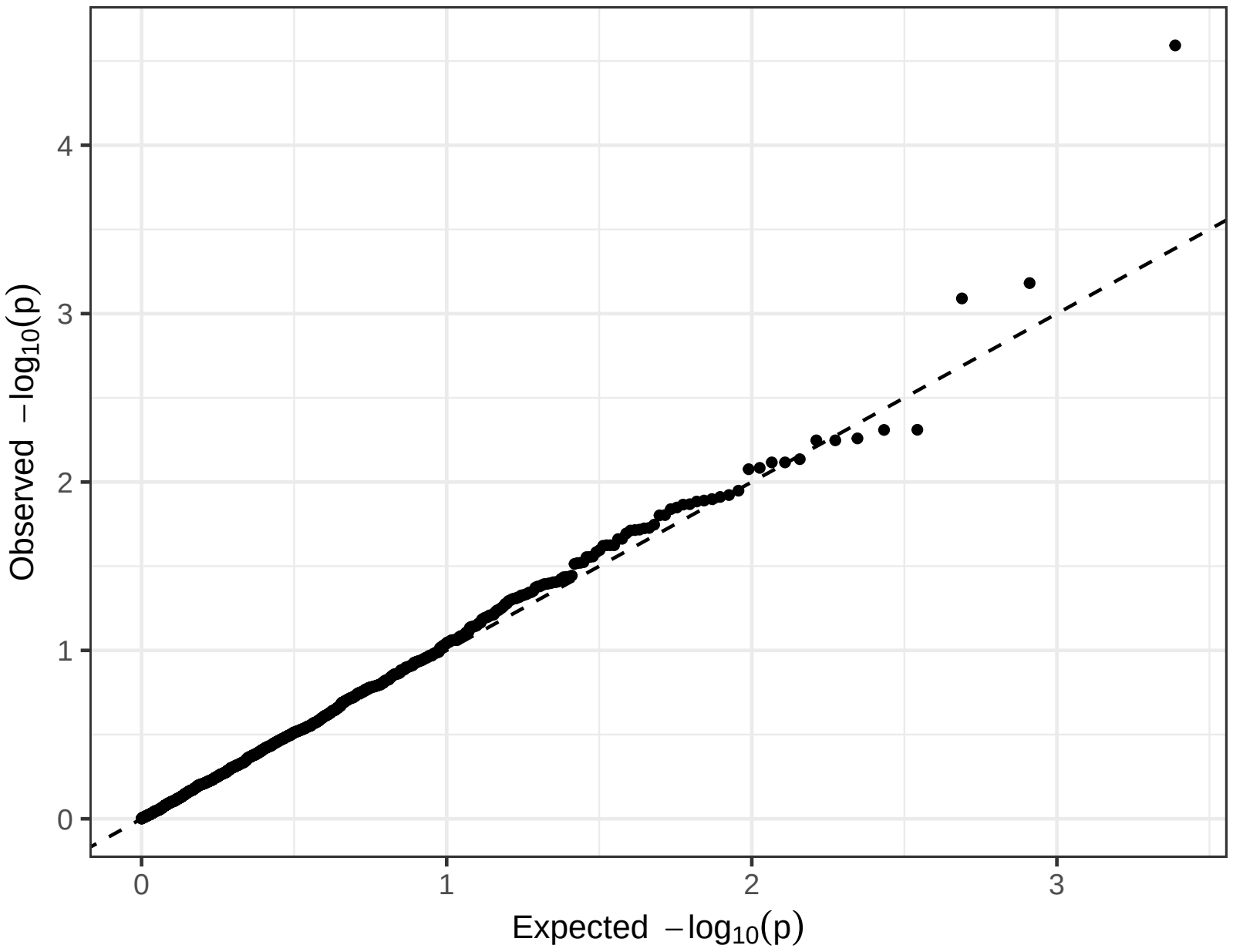

Holdemanella

Feces | Liver | . = 0.868

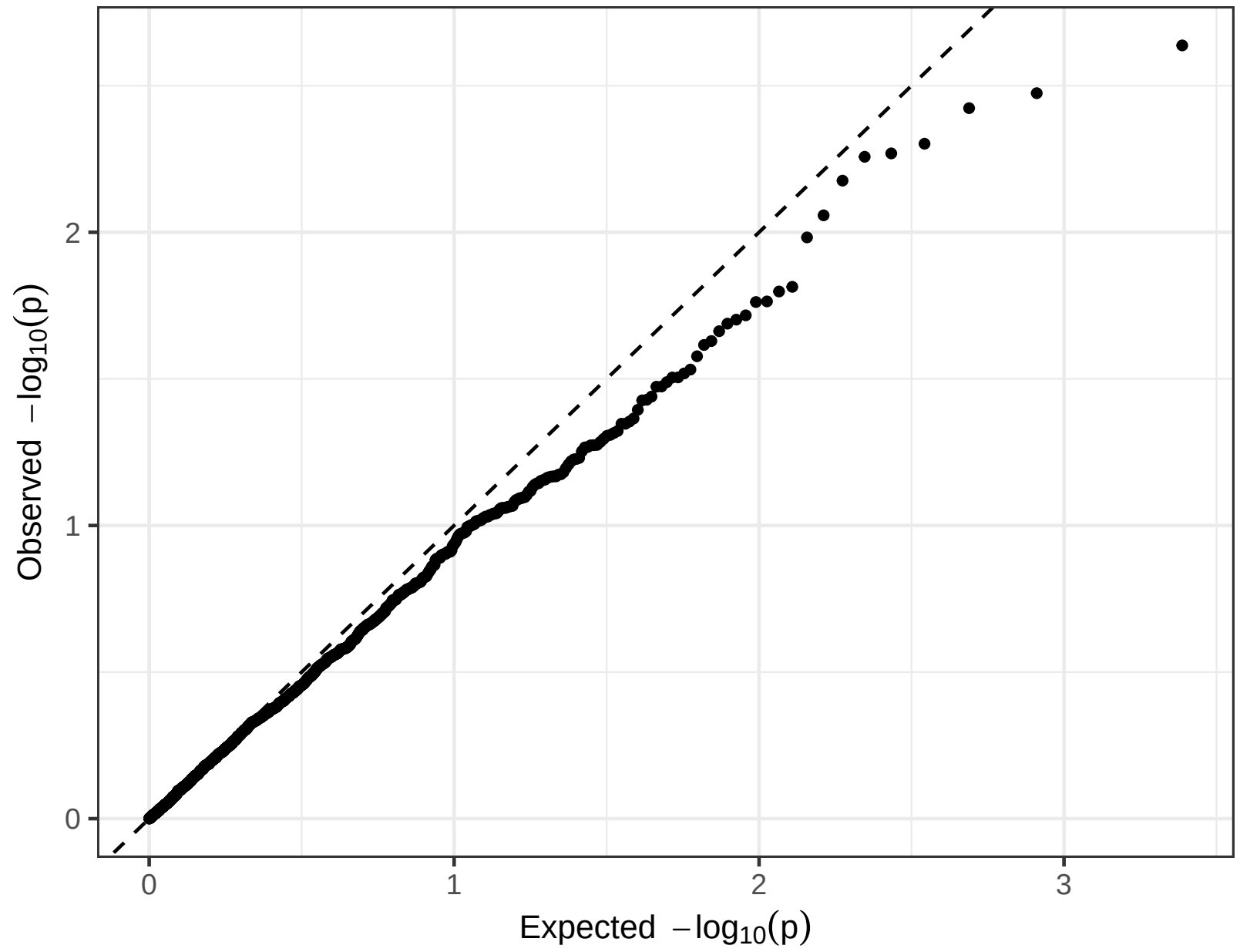

horsej.a03

Feces | Liver | . = 0.921

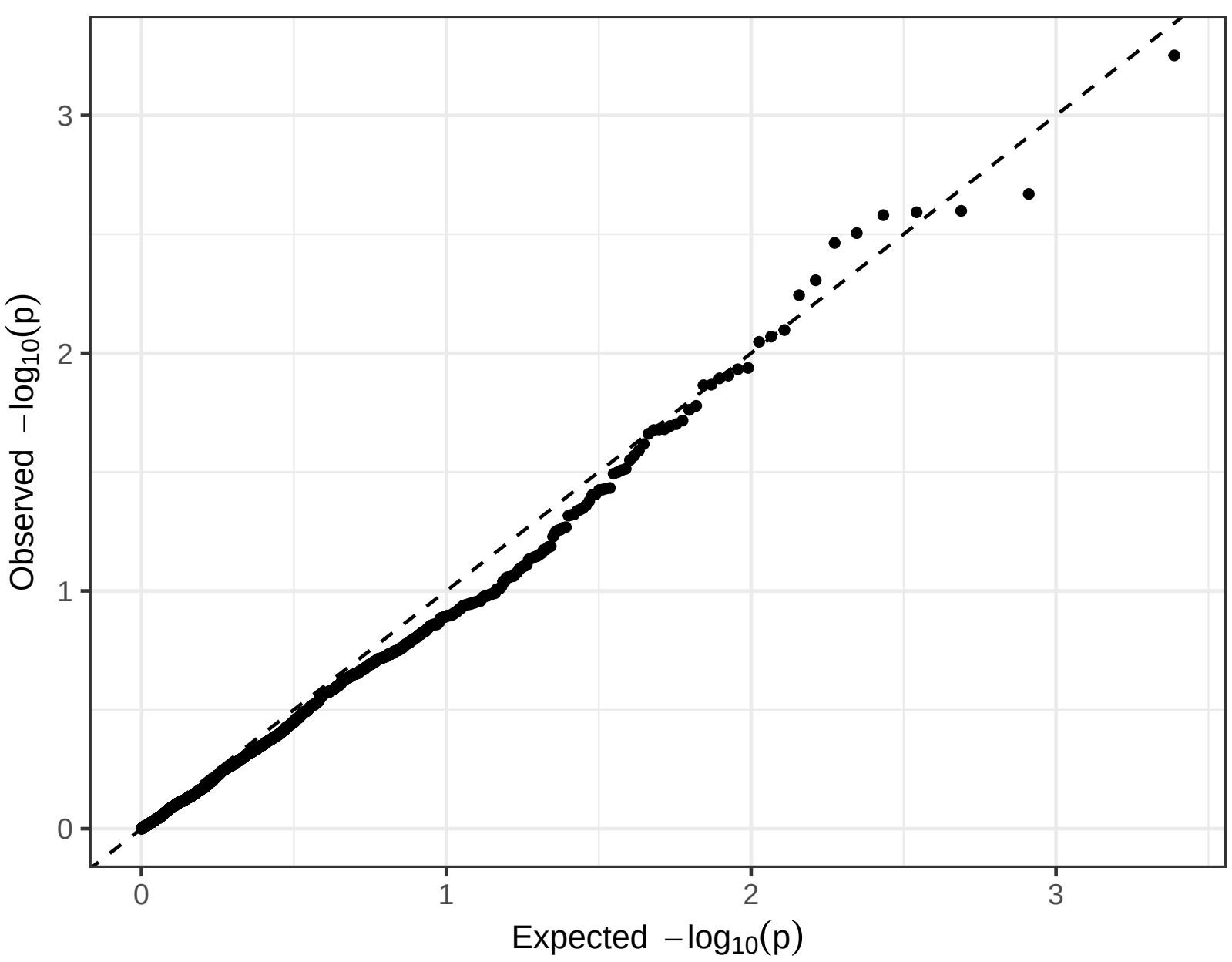

HT002

Feces | Liver | . = 0.939

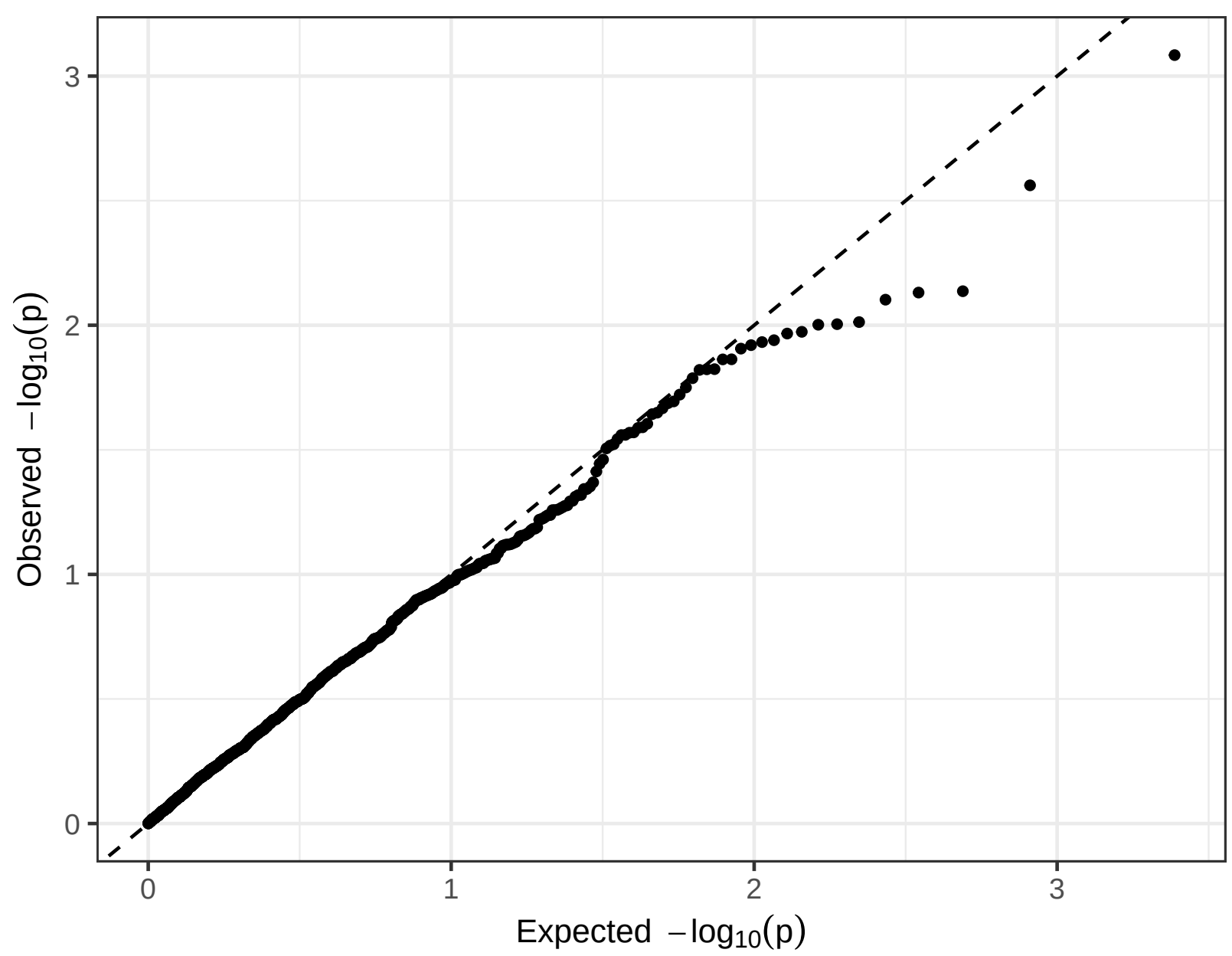

Ruminococcus_gauvreauii_group

Feces | Liver | . = 1.116

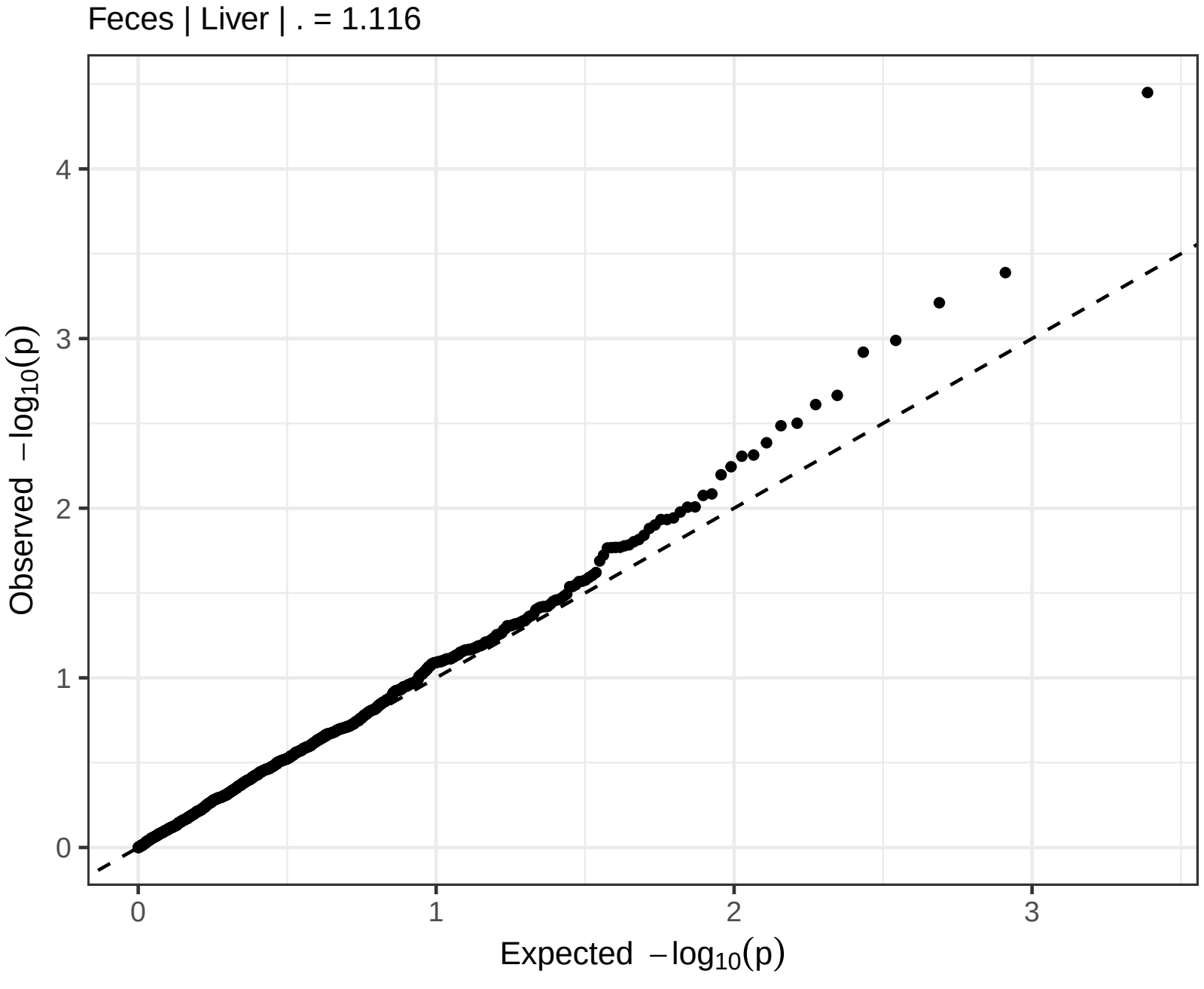

Incertae_Sedis

Feces | Liver | . = 0.905

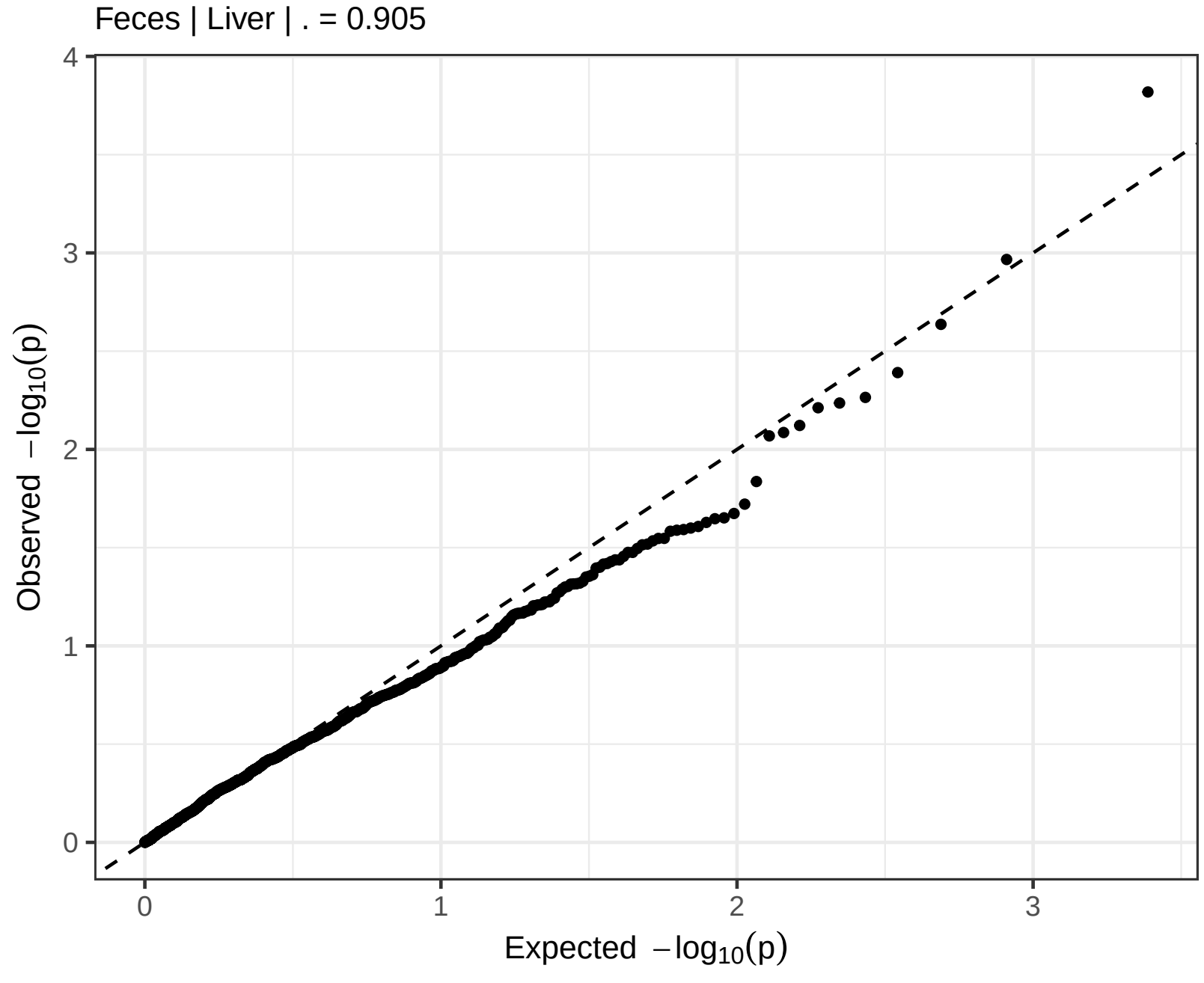

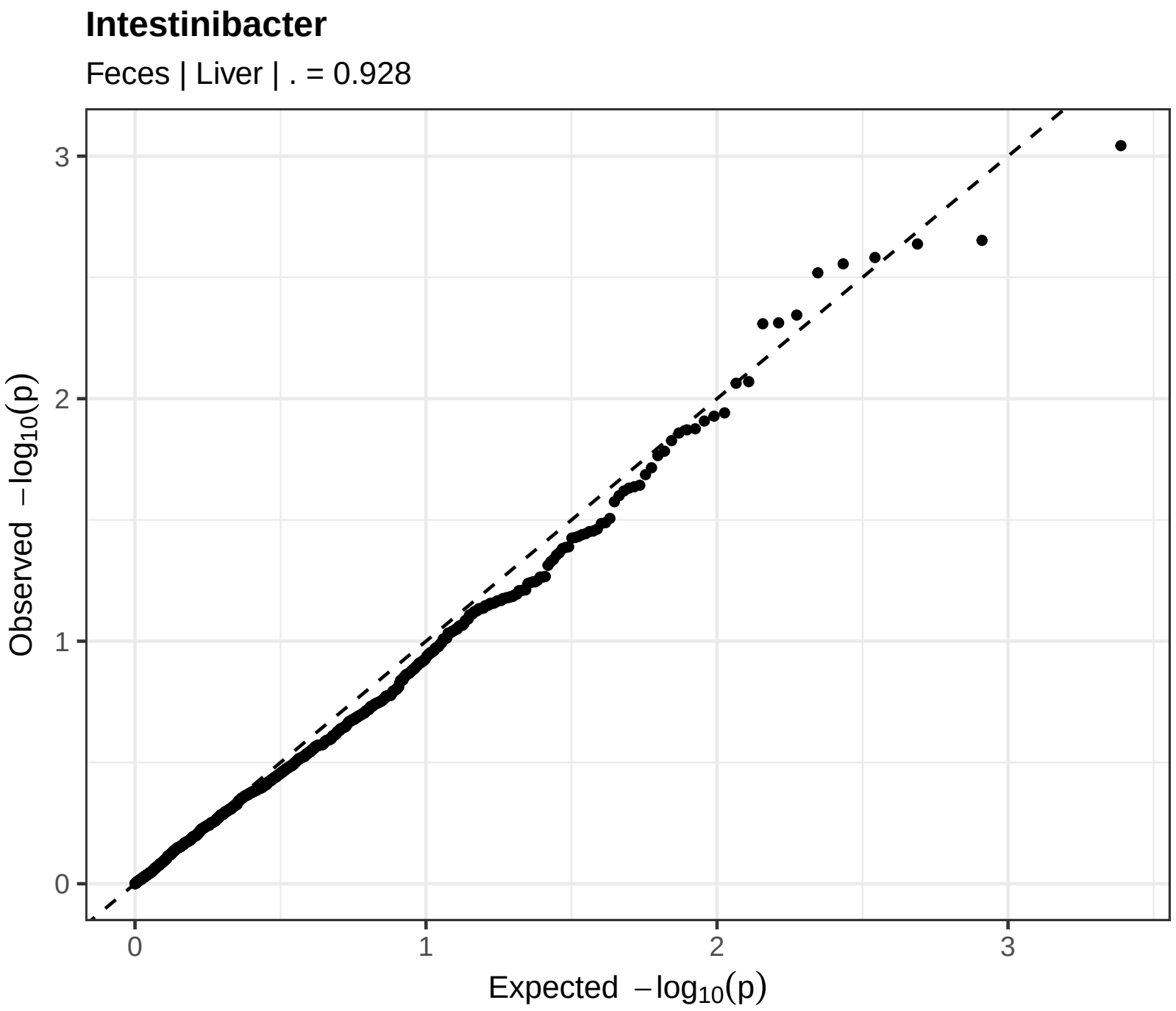
Intestinibacter
Feces | Liver | . = 0.928
Observed $-\log_{10}(p)$
Expected $-\log_{10}(p)$

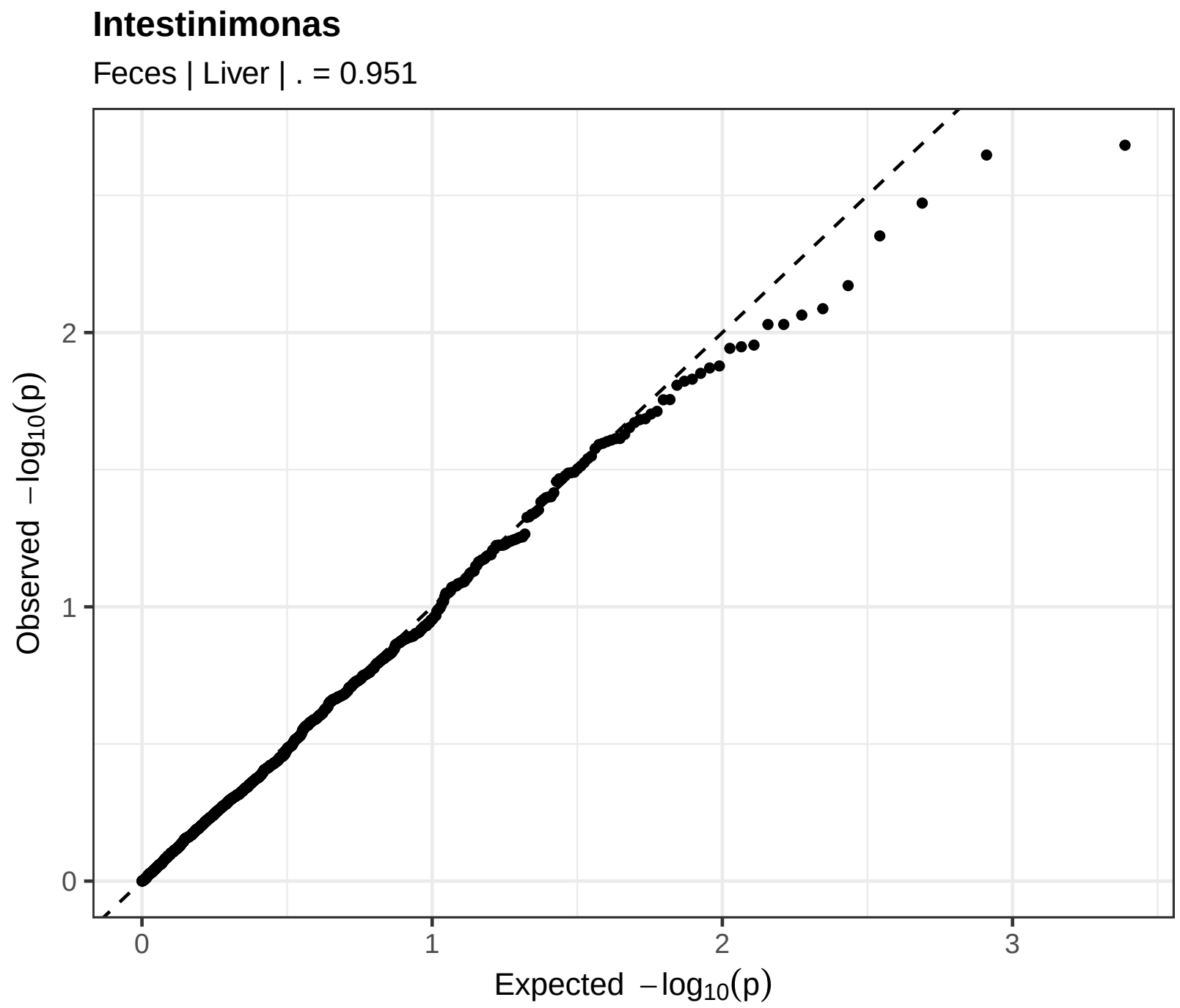
Intestinimonas
Feces | Liver | . = 0.951
Observed $-\log_{10}(p)$
Expected $-\log_{10}(p)$

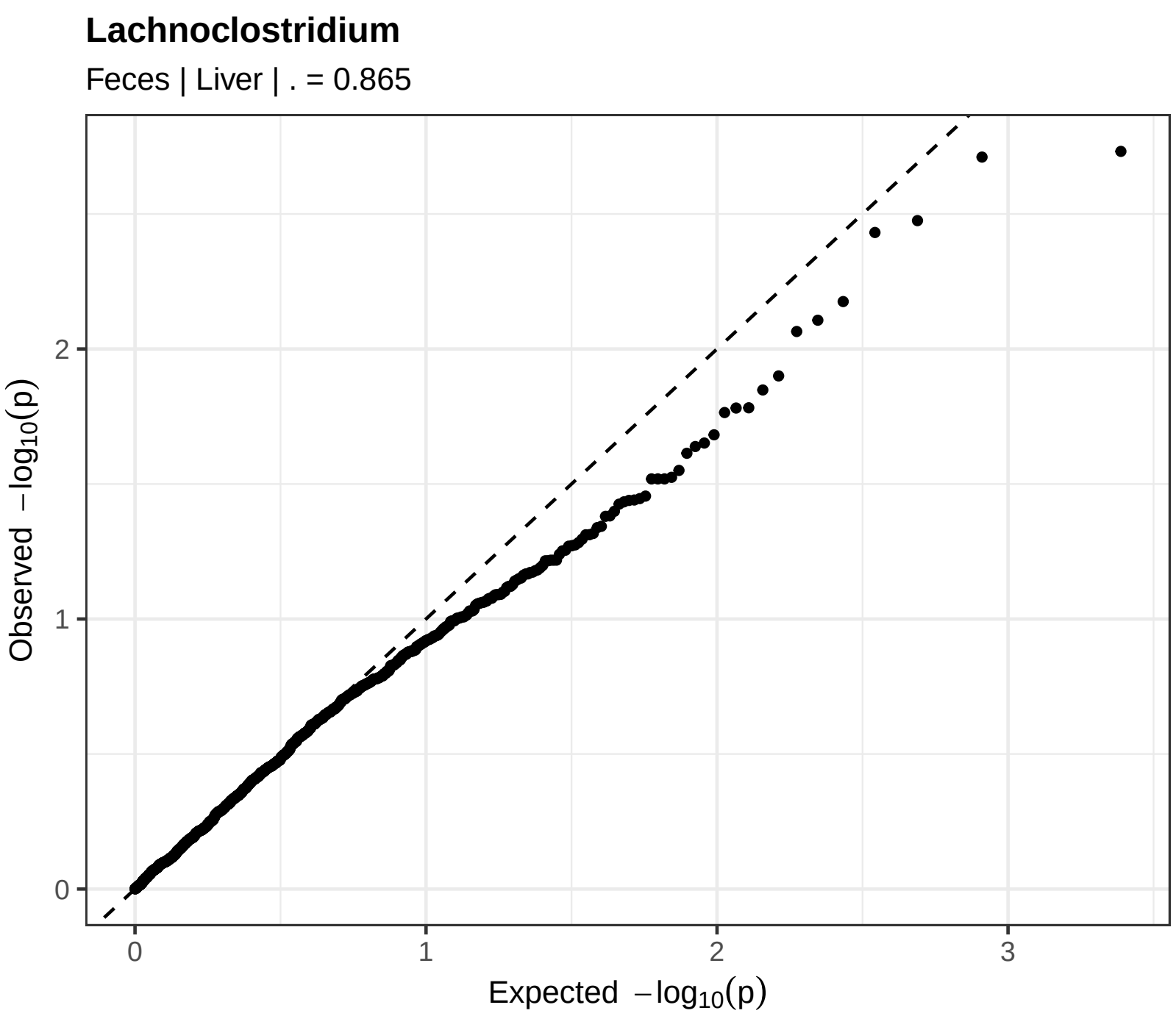
Lachnoclostridium
Feces | Liver | . = 0.865
Observed $-\log_{10}(p)$
Expected $-\log_{10}(p)$

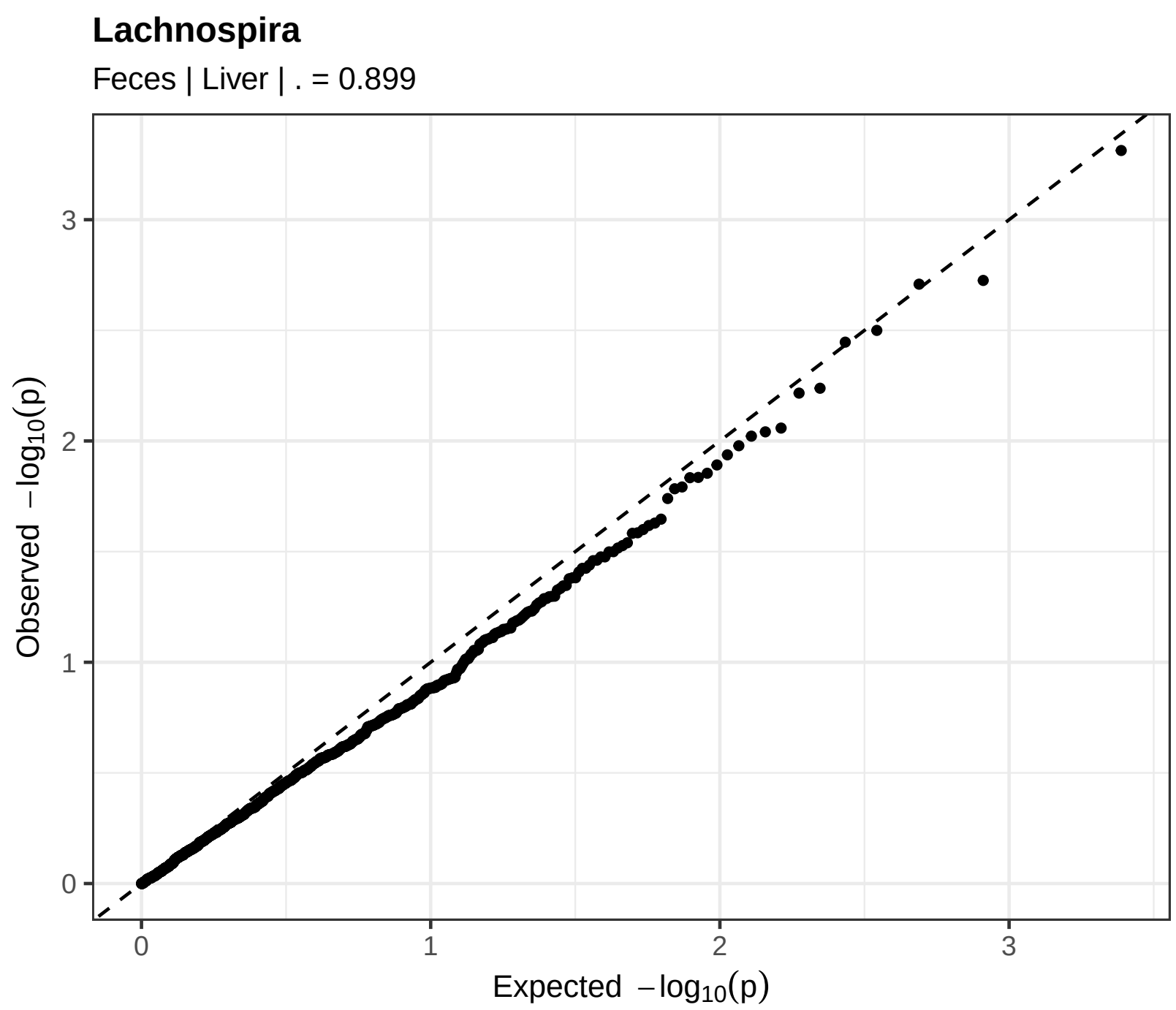
Lachnospira
Feces | Liver | . = 0.899
Observed $-\log_{10}(p)$
Expected $-\log_{10}(p)$

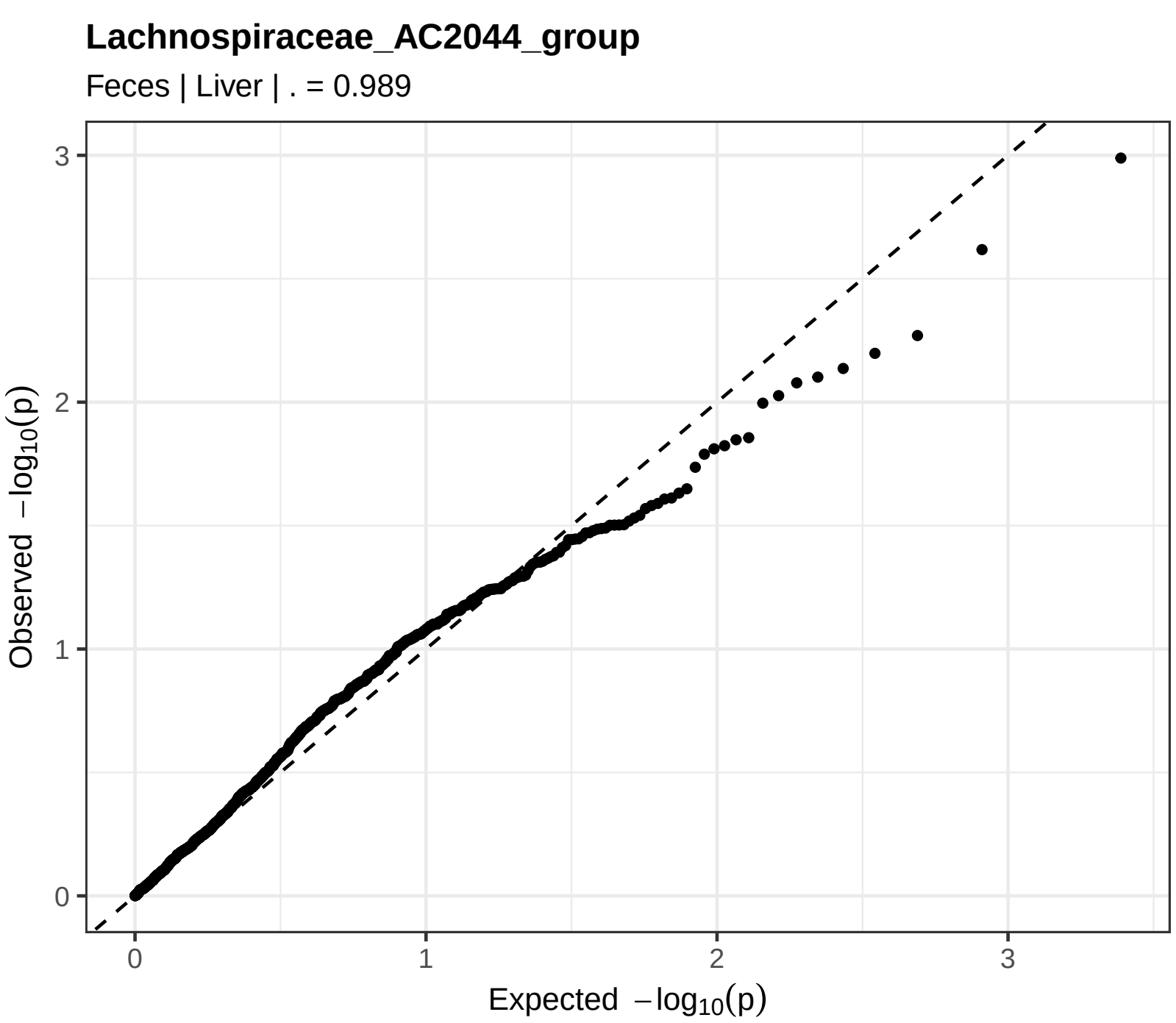
Lachnospiraceae_AC2044_group
Feces | Liver | . = 0.989
Observed $-\log_{10}(p)$
Expected $-\log_{10}(p)$

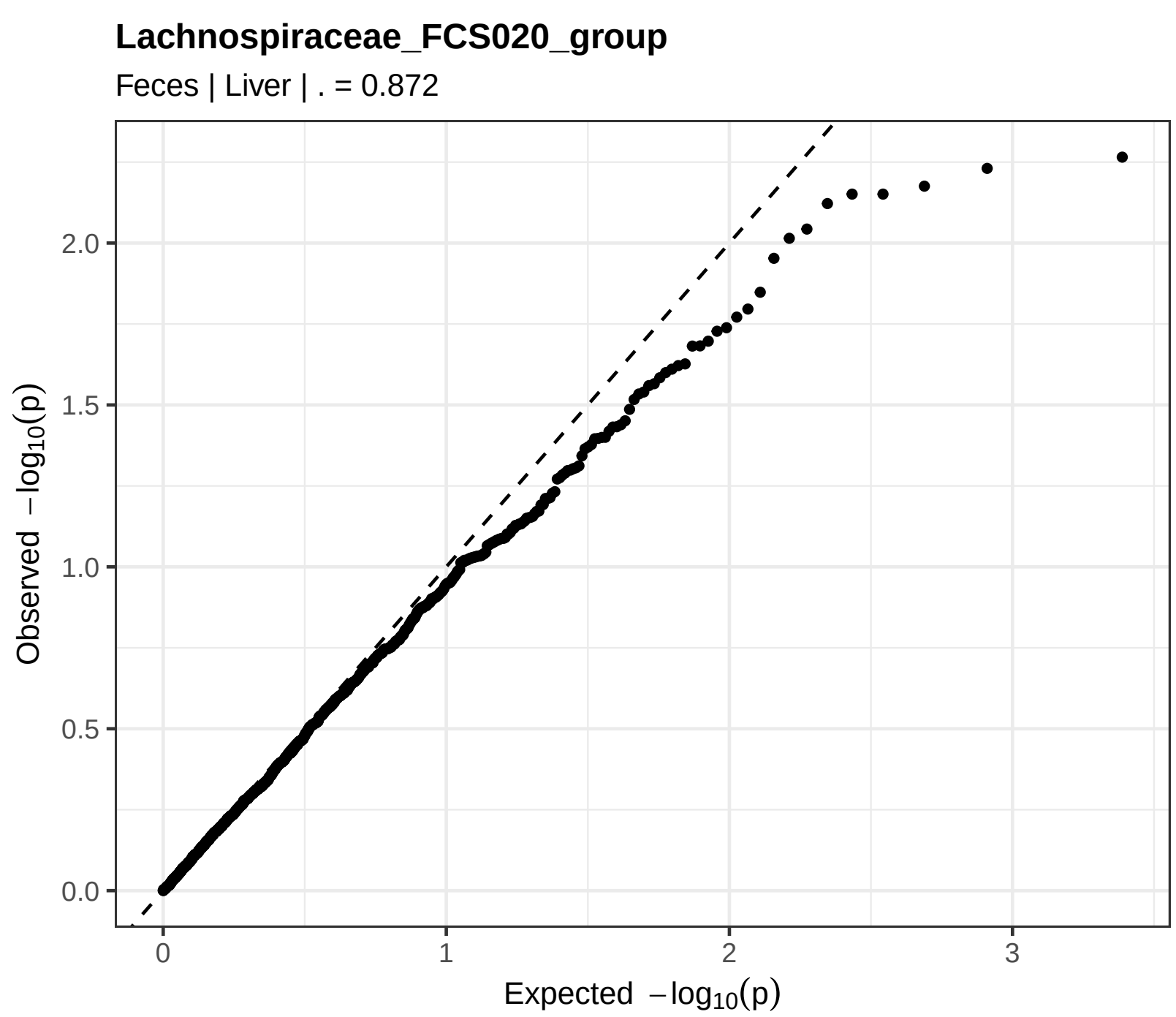
Lachnospiraceae_FCS020_group
Feces | Liver | . = 0.872
Observed $-\log_{10}(p)$
Expected $-\log_{10}(p)$

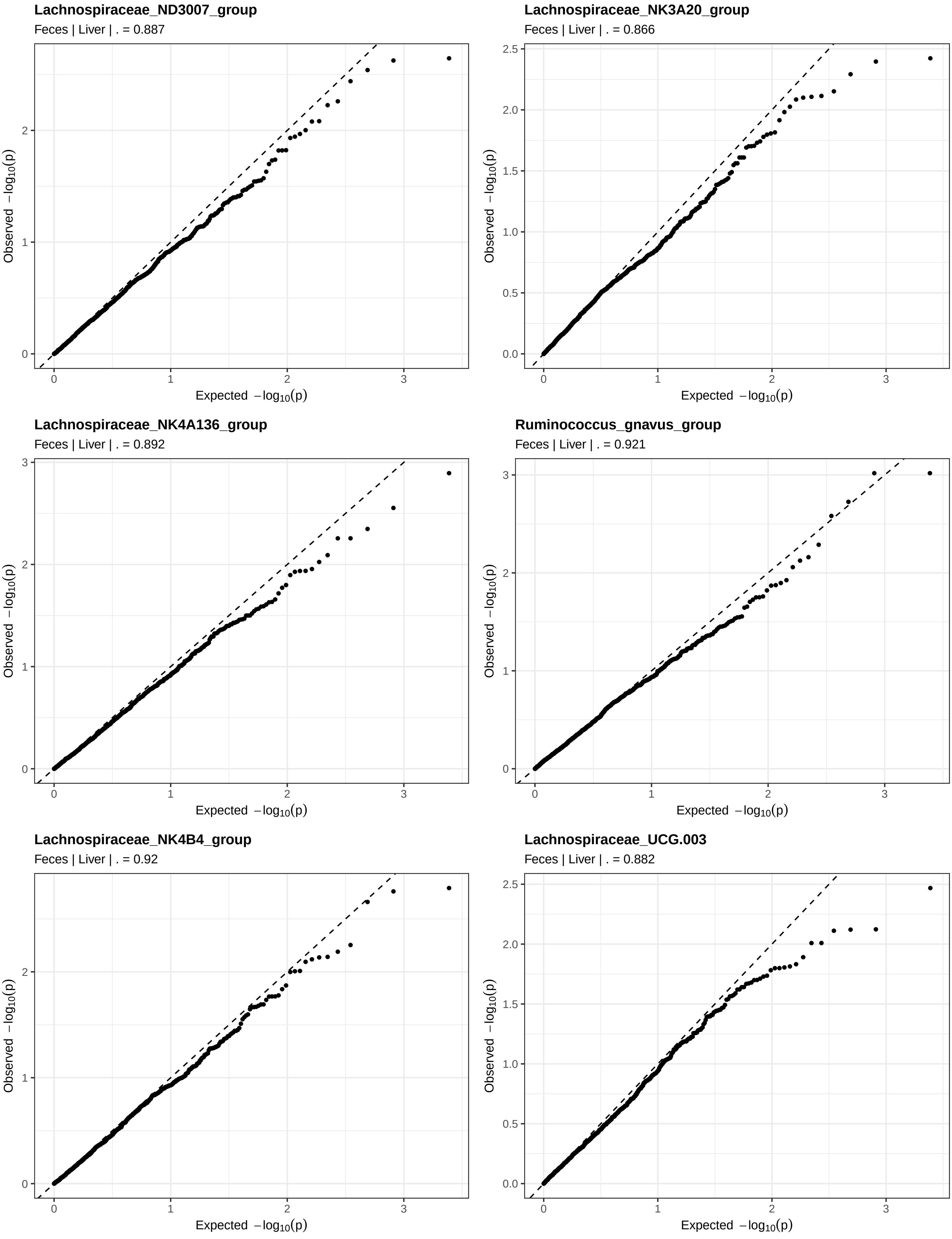

Lachnospiraceae_ND3007_group
Feces | Liver | . = 0.887
Lachnospiraceae_NK3A20_group
Feces | Liver | . = 0.866
Lachnospiraceae_NK4A136_group
Feces | Liver | . = 0.892
Ruminococcus_gnavus_group
Feces | Liver | . = 0.921
Lachnospiraceae_NK4B4_group
Feces | Liver | . = 0.92
Lachnospiraceae_UCG.003
Feces | Liver | . = 0.882
Observed −log10(p)
Expected −log10(p)

Lachnospiraceae_UCG.004

Feces | Liver | . = 0.998

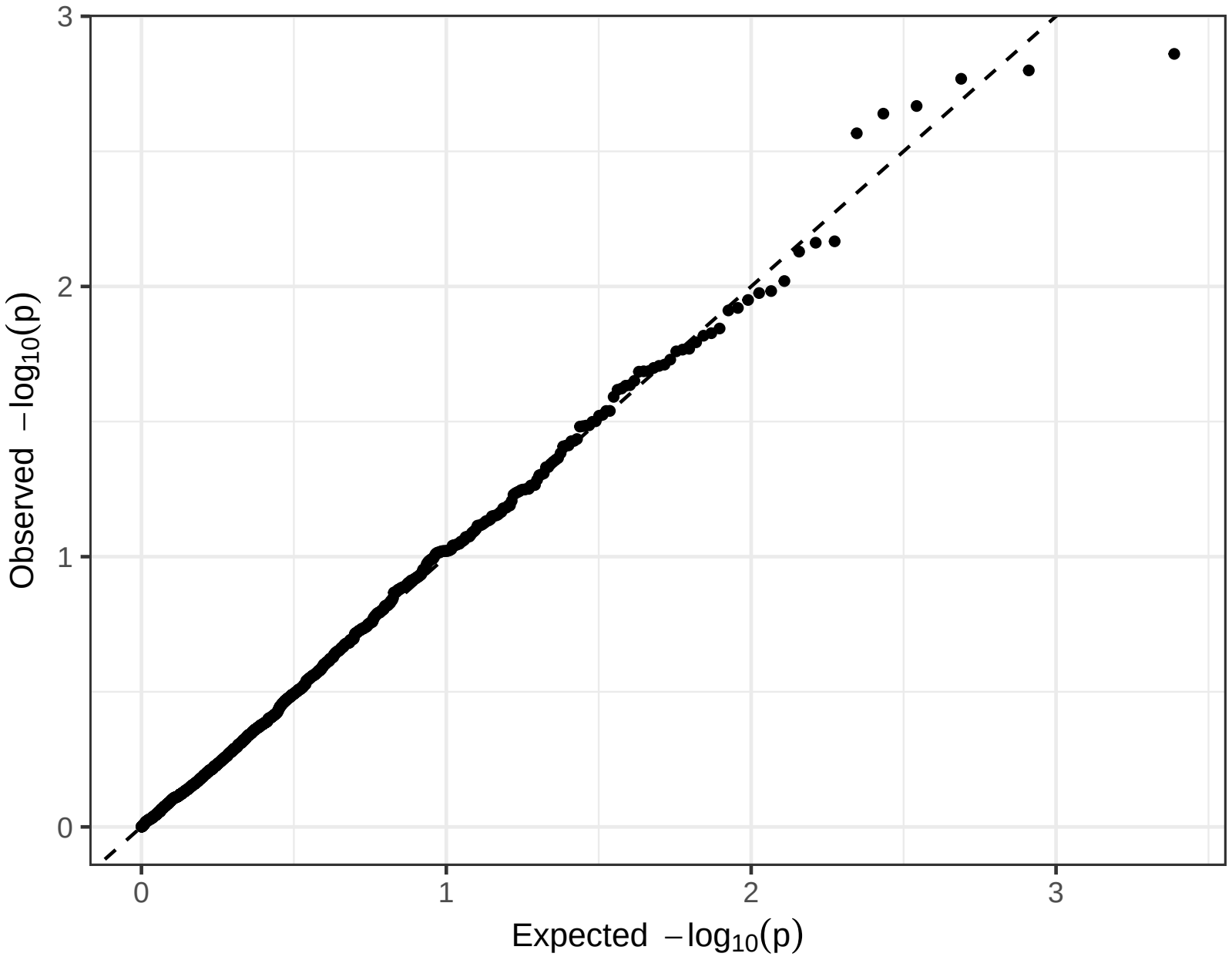

Lachnospiraceae_UCG.007

Feces | Liver | . = 0.877

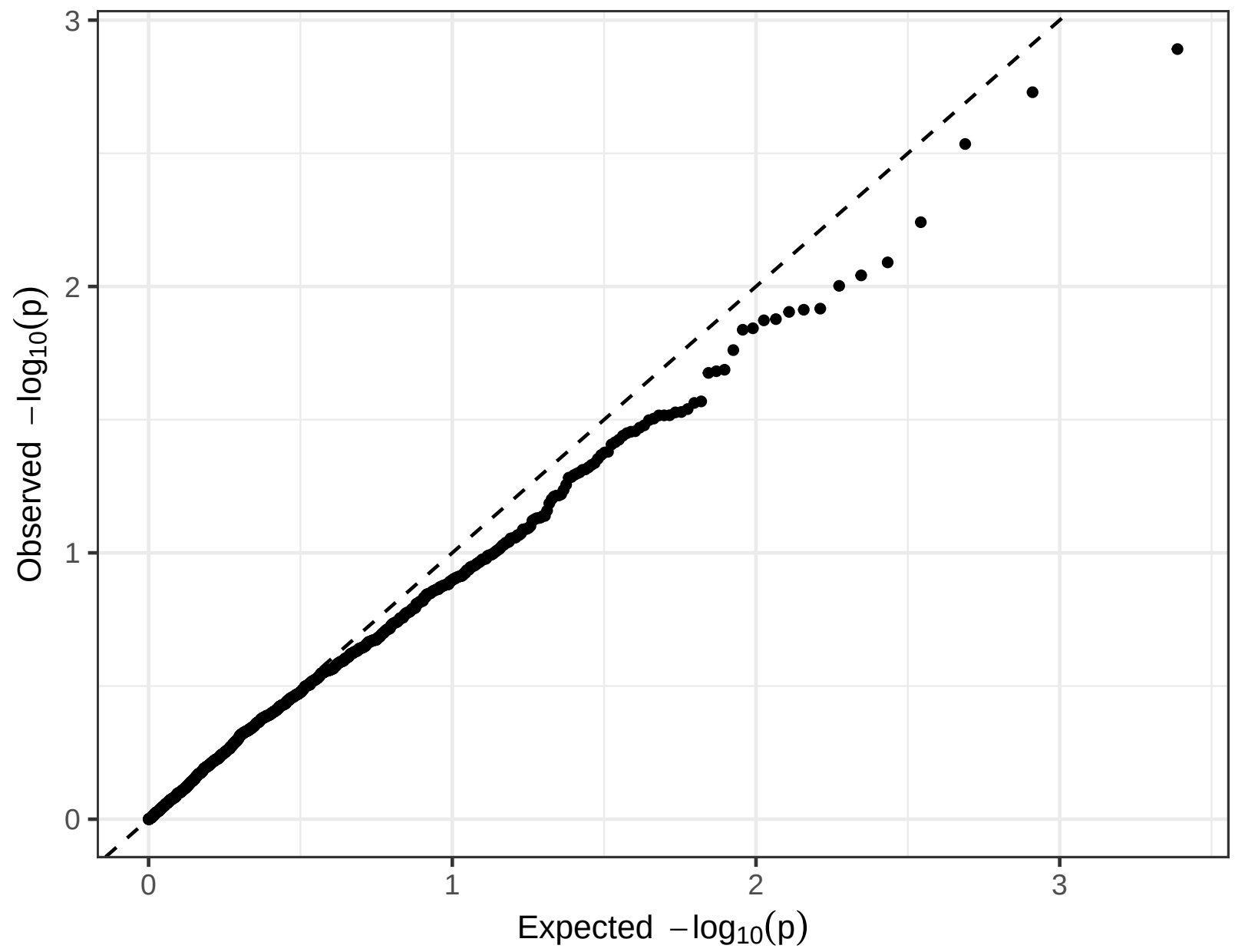

Lachnospiraceae_UCG.009

Feces | Liver | . = 0.879

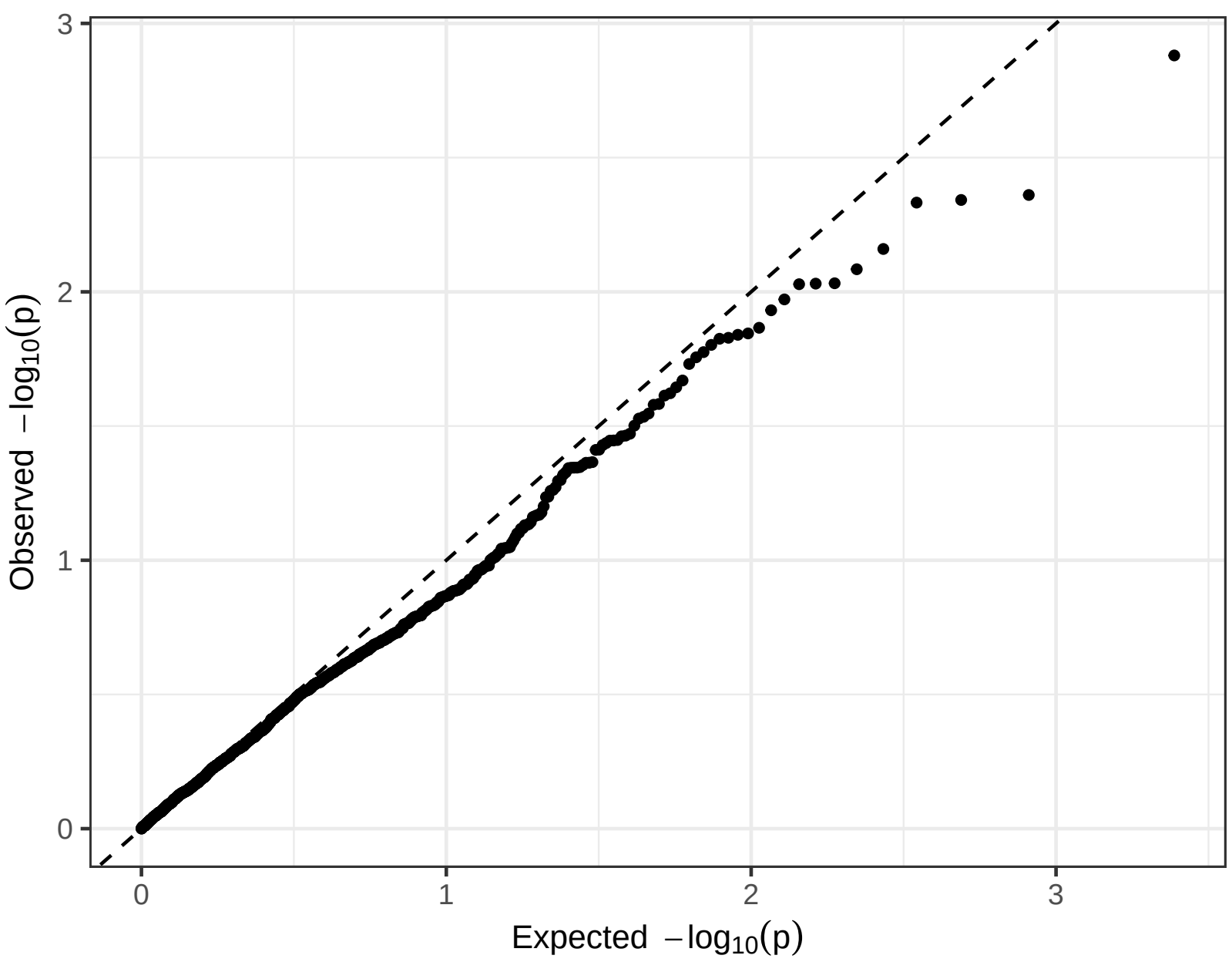

Lachnospiraceae_UCG.010

Feces | Liver | . = 0.881

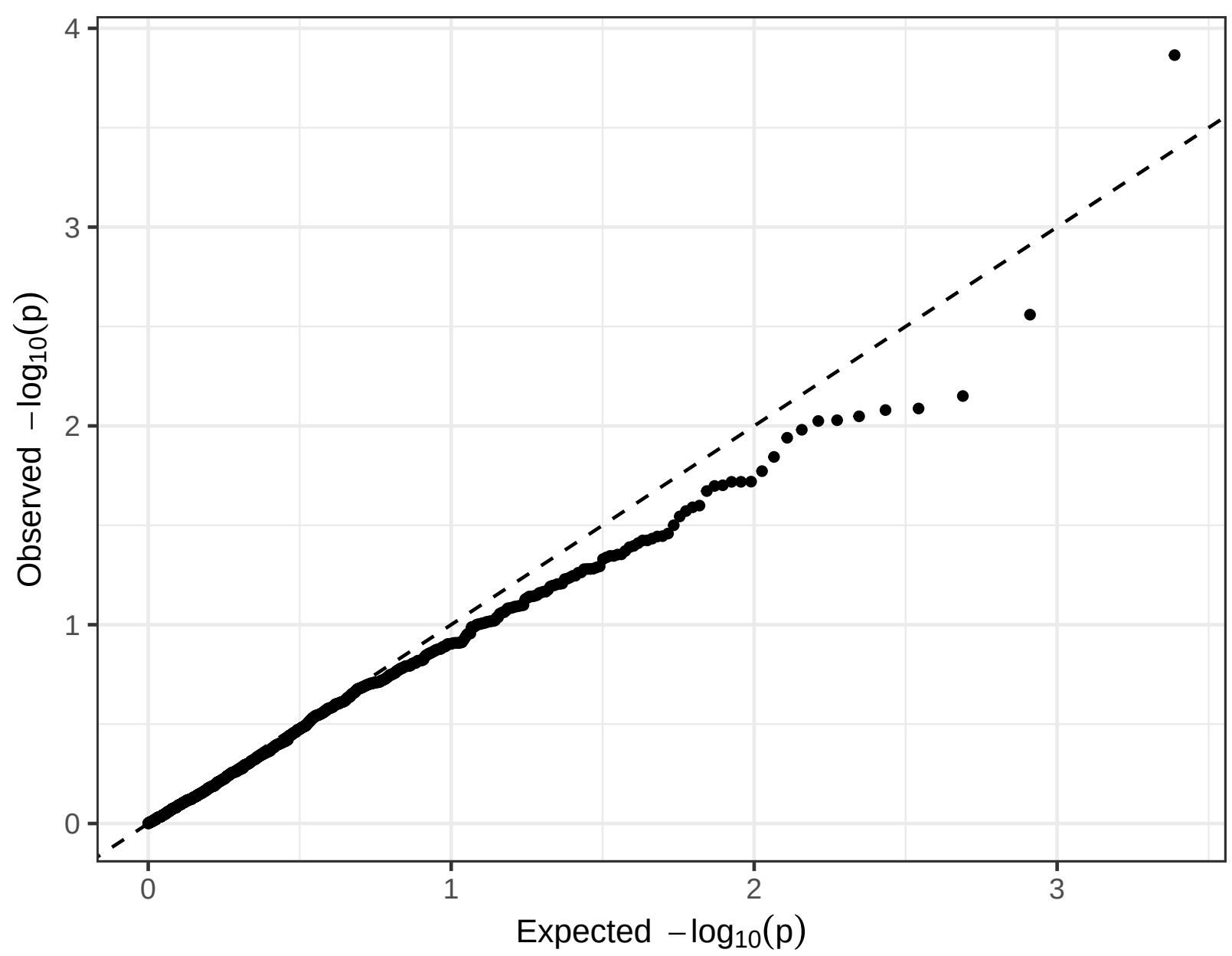

Lachnospiraceae_XPB1014_group

Feces | Liver | . = 0.975

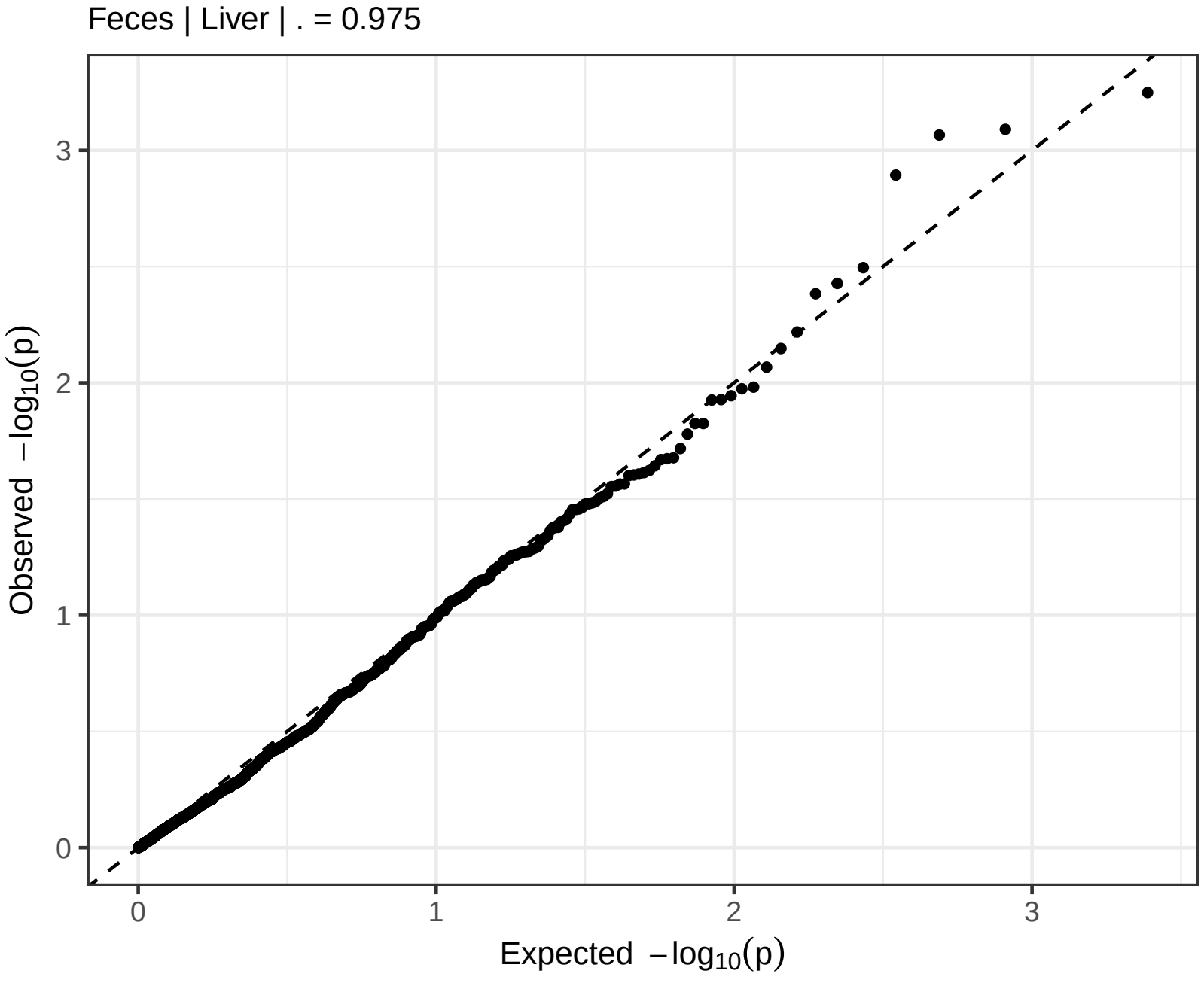

Lactobacillus

Feces | Liver | . = 1.001

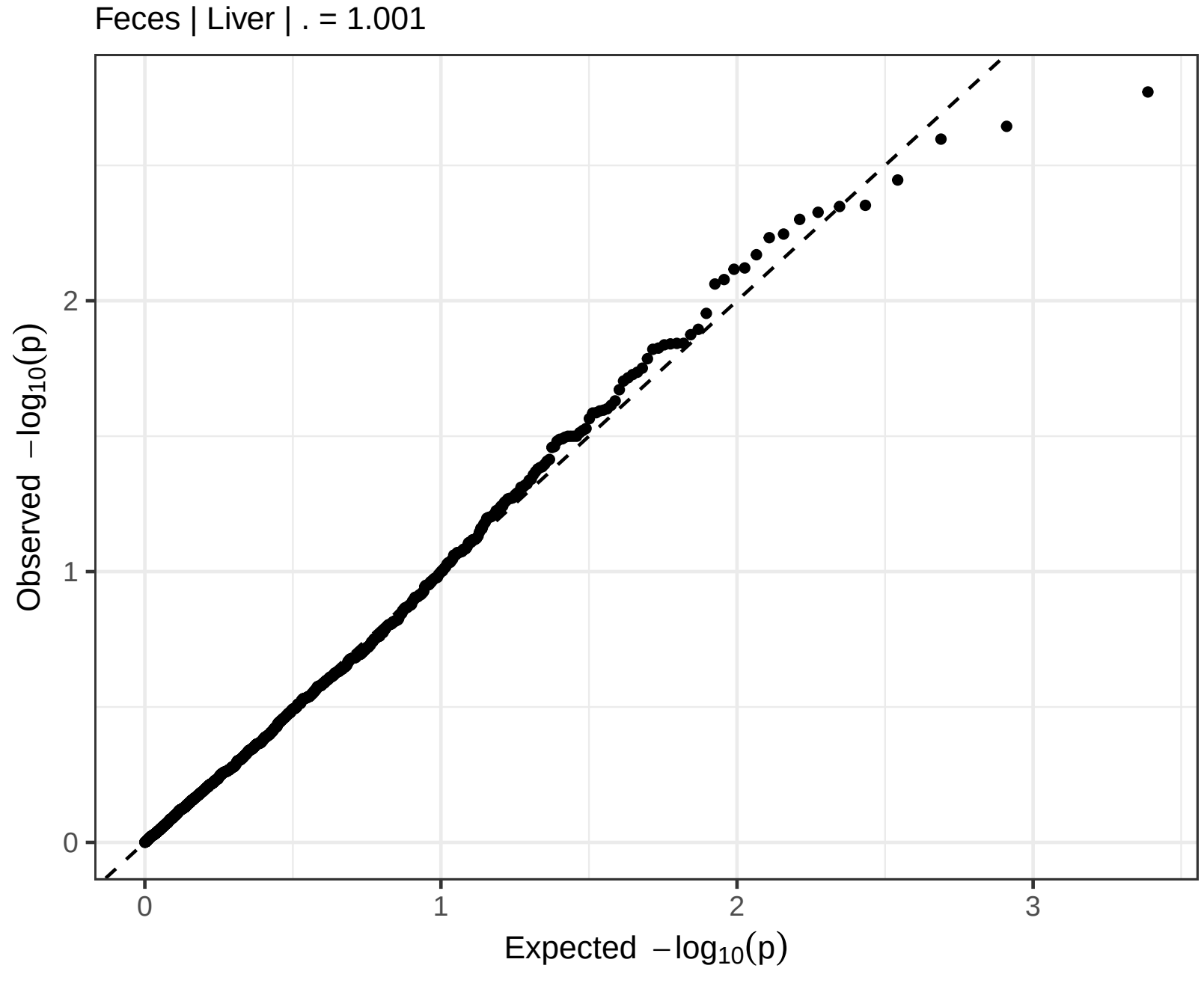

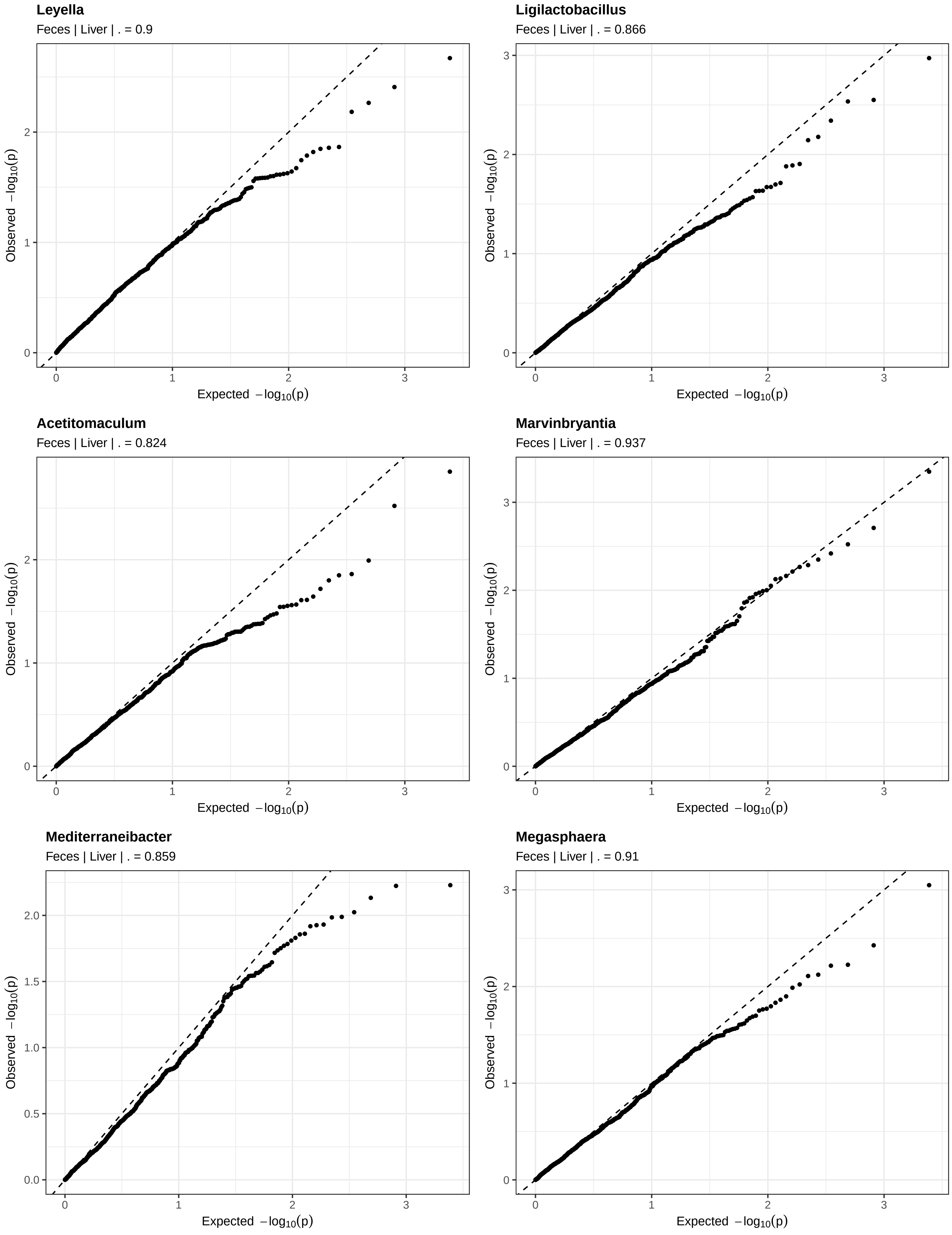
Leyella
Feces | Liver | . = 0.9
Ligilactobacillus
Feces | Liver | . = 0.866
Acetitomaculum
Feces | Liver | . = 0.824
Marvinbryantia
Feces | Liver | . = 0.937
Mediterraneibacter
Feces | Liver | . = 0.859
Megasphaera
Feces | Liver | . = 0.91
Observed −log10(p)
Expected −log10(p)

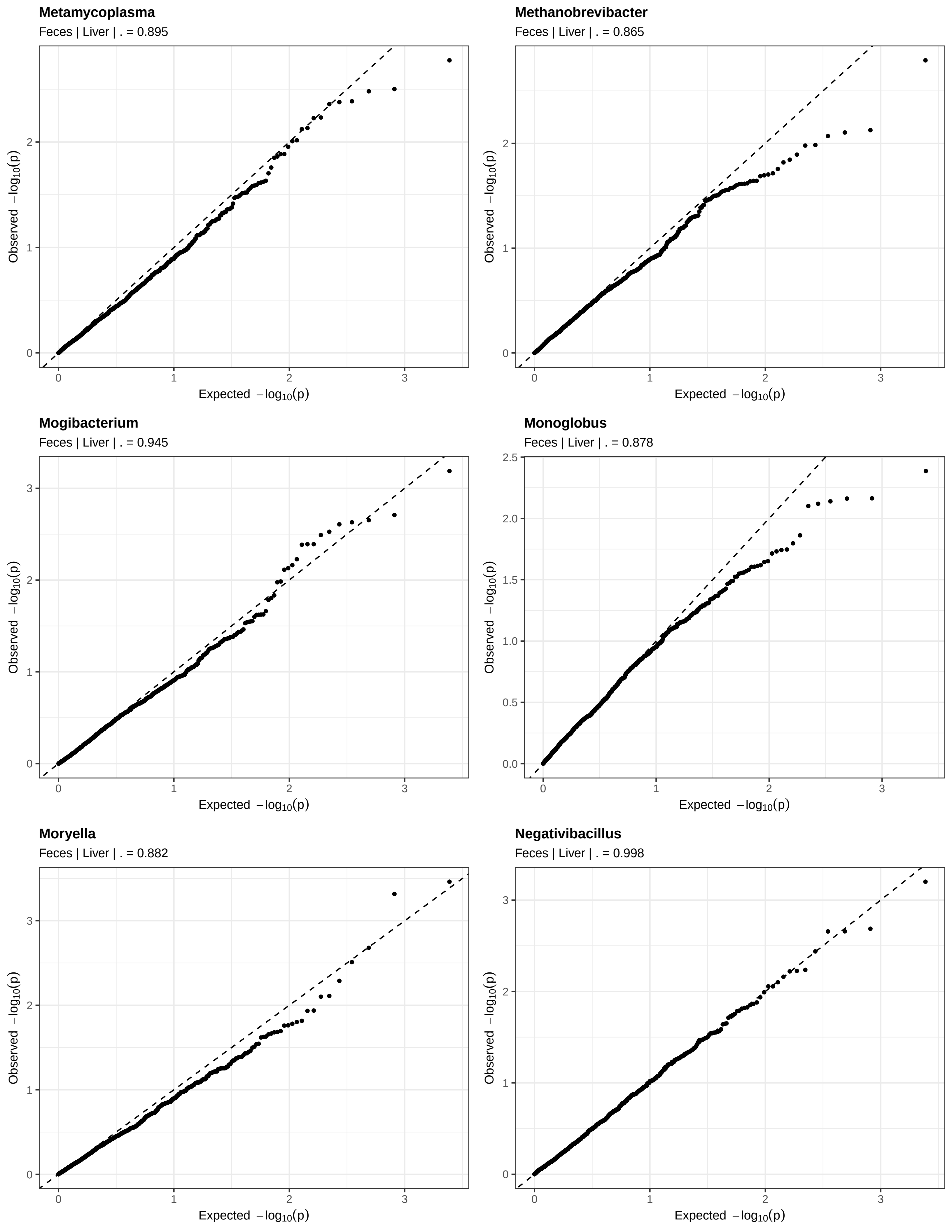

Metamycoplasma
Feces | Liver | . = 0.895
Methanobrevibacter
Feces | Liver | . = 0.865
Mogibacterium
Feces | Liver | . = 0.945
Monoglobus
Feces | Liver | . = 0.878
Moryella
Feces | Liver | . = 0.882
Negativibacillus
Feces | Liver | . = 0.998
Expected $-\log_{10}(p)$
Observed $-\log_{10}(p)$

NK4A214_group

Feces | Liver | . = 0.873

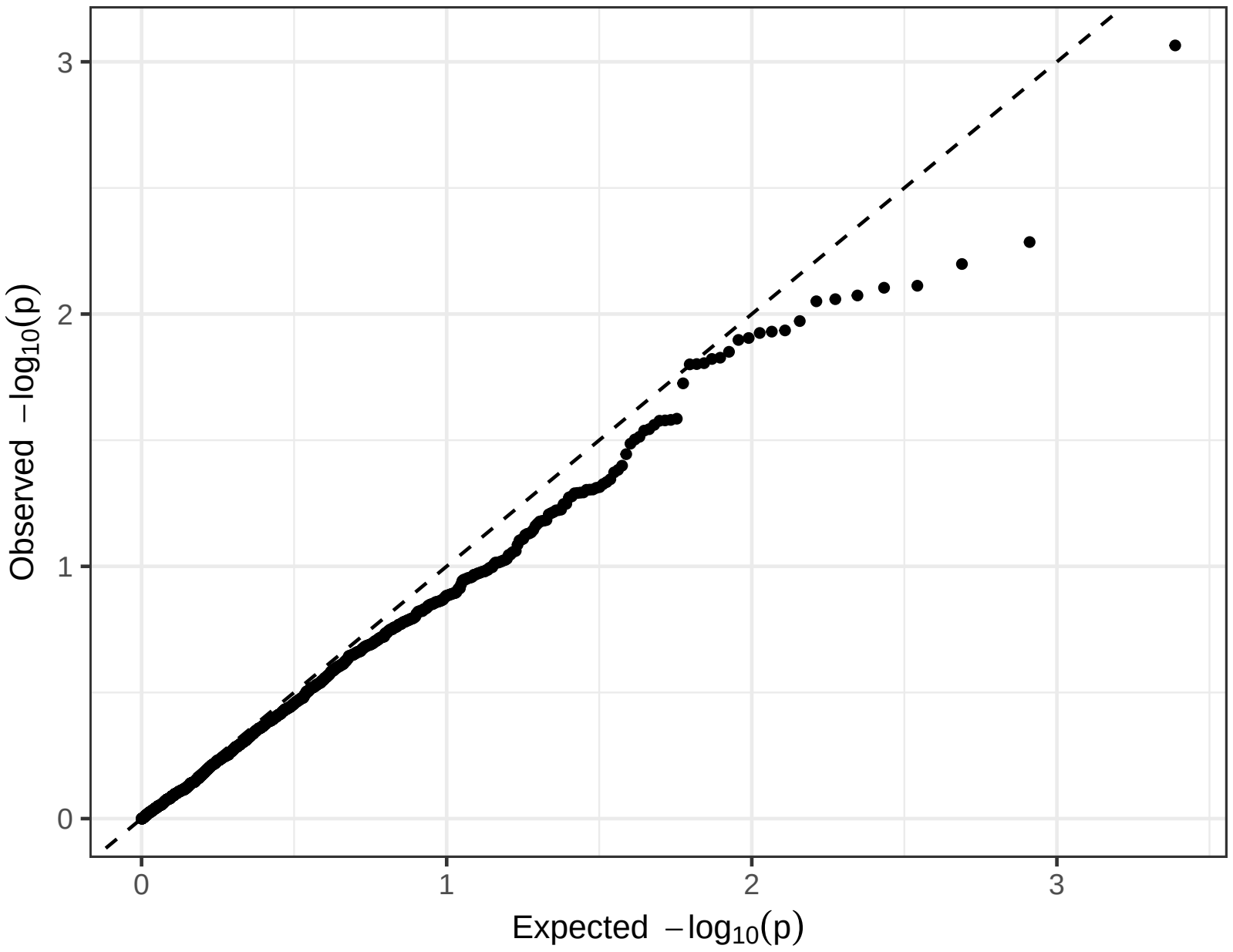

Acidaminococcus

Feces | Liver | . = 0.932

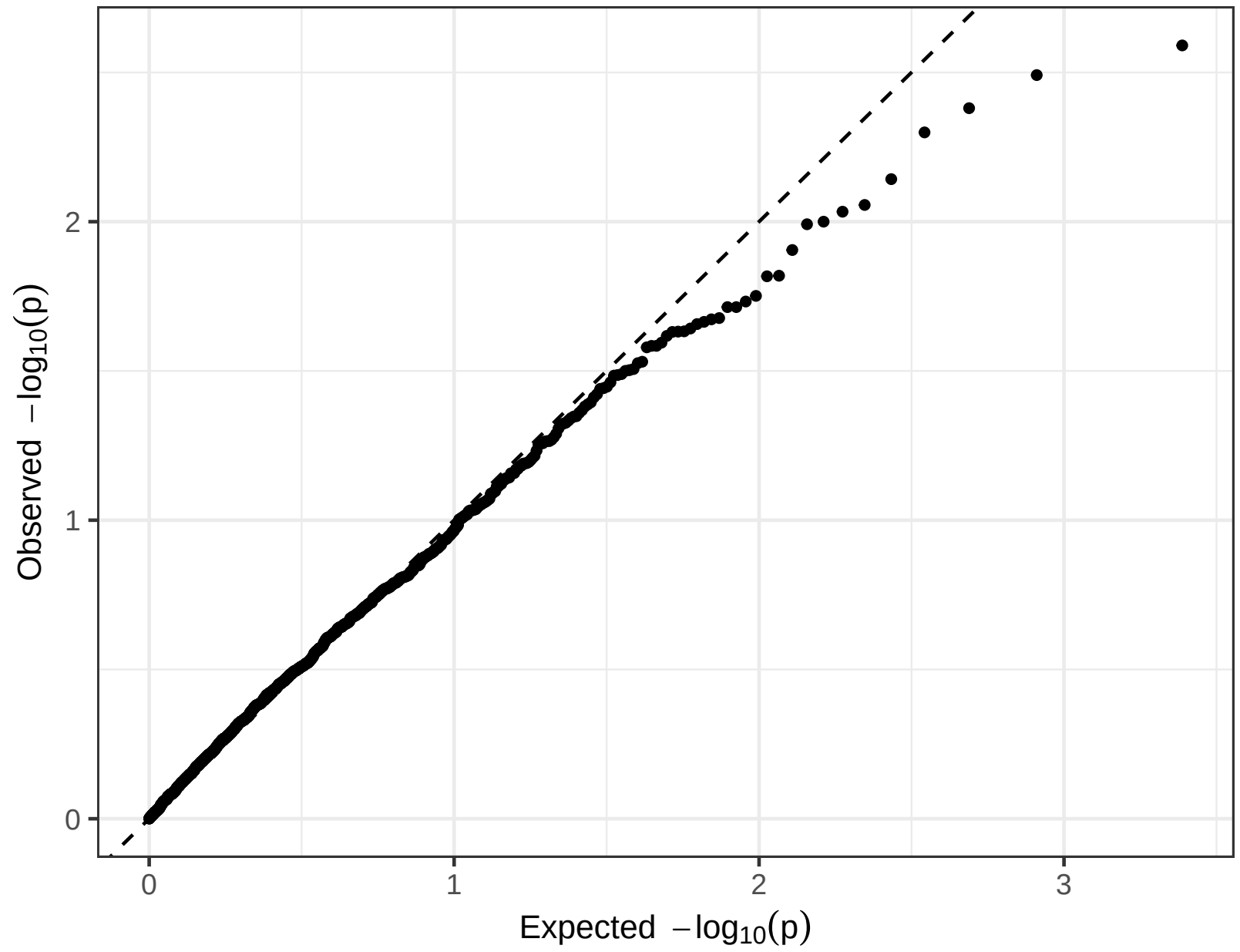

Oribacterium

Feces | Liver | . = 0.938

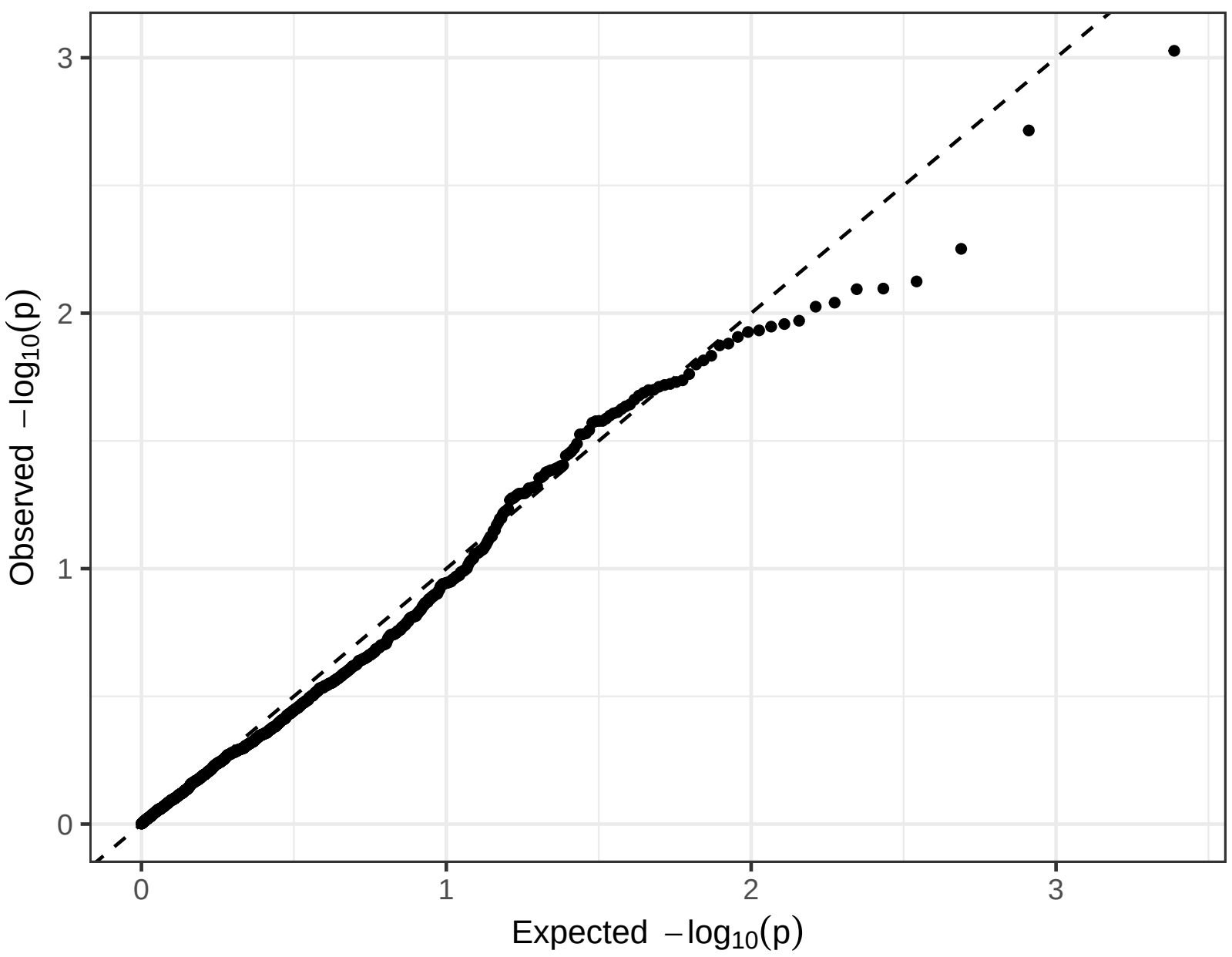

Oscillibacter

Feces | Liver | . = 0.96

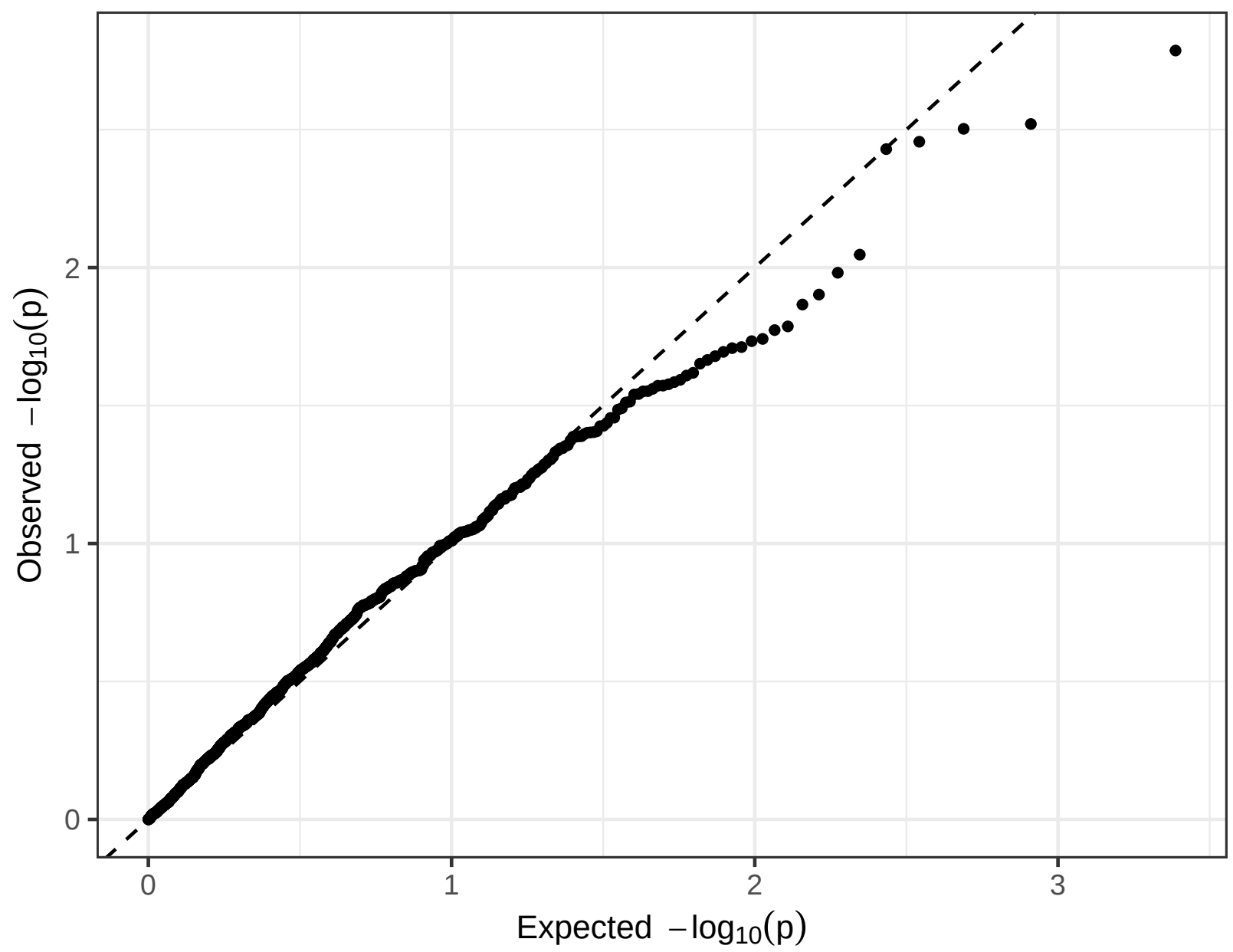

Oscillospira

Feces | Liver | . = 0.933

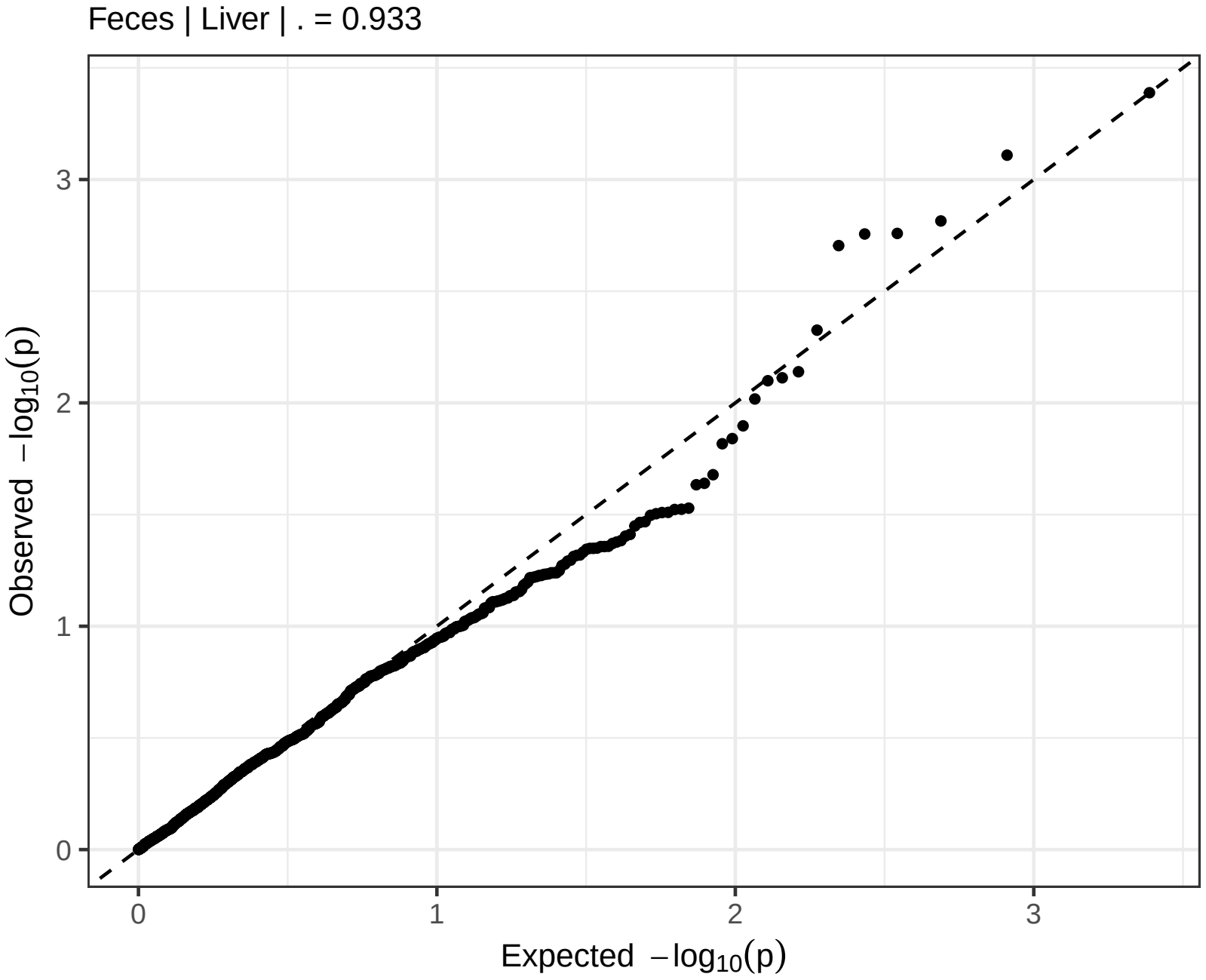

Oxalobacter

Feces | Liver | . = 0.911

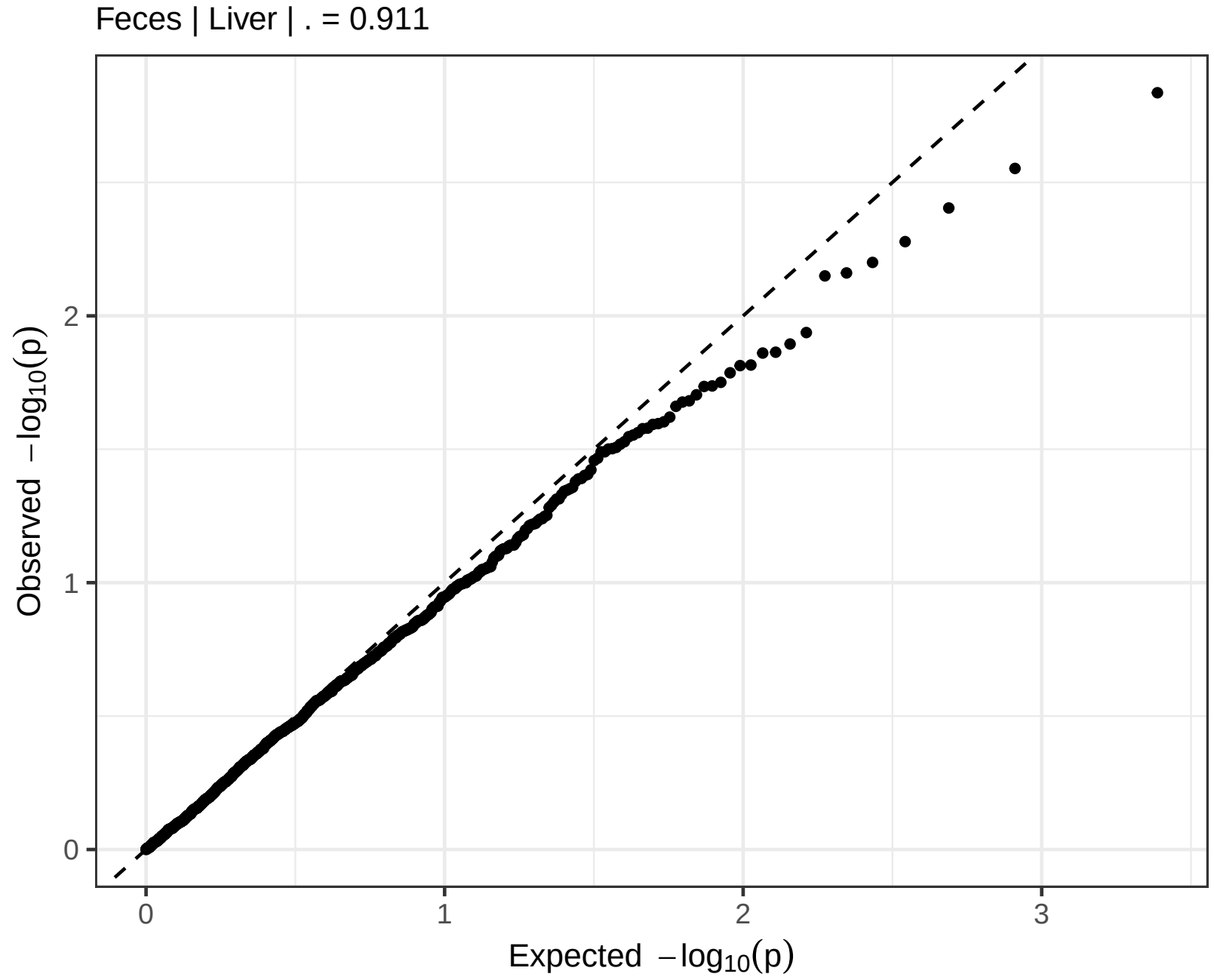

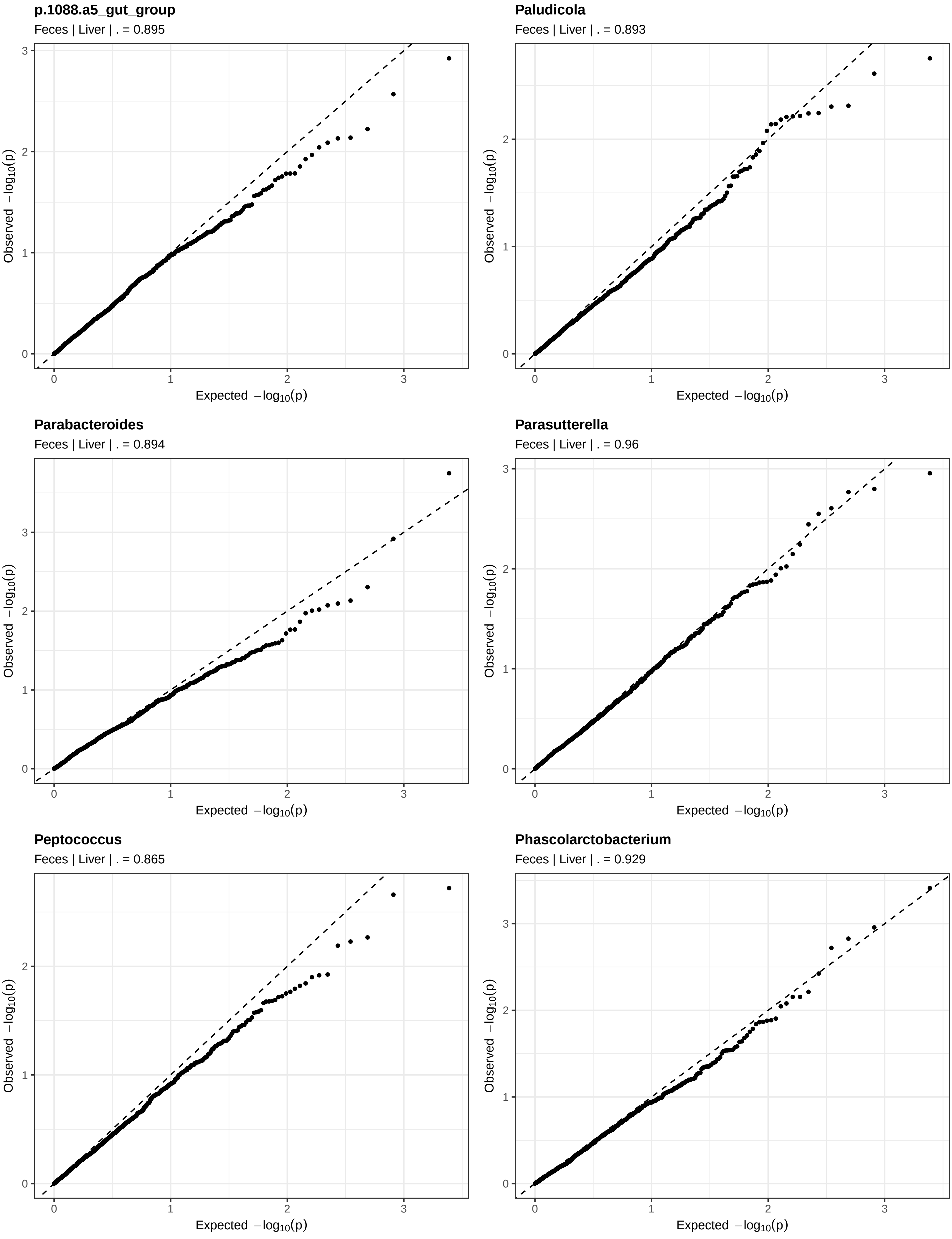

p.1088.a5_gut_group
Feces | Liver | . = 0.895
Paludicola
Feces | Liver | . = 0.893
Parabacteroides
Feces | Liver | . = 0.894
Parasutterella
Feces | Liver | . = 0.96
Peptococcus
Feces | Liver | . = 0.865
Phascolarctobacterium
Feces | Liver | . = 0.929
Observed $-\log_{10}(p)$
Expected $-\log_{10}(p)$
0
1
2
3

Bacteroides_pectinophilus_group

Feces | Muscle | . = 0.798

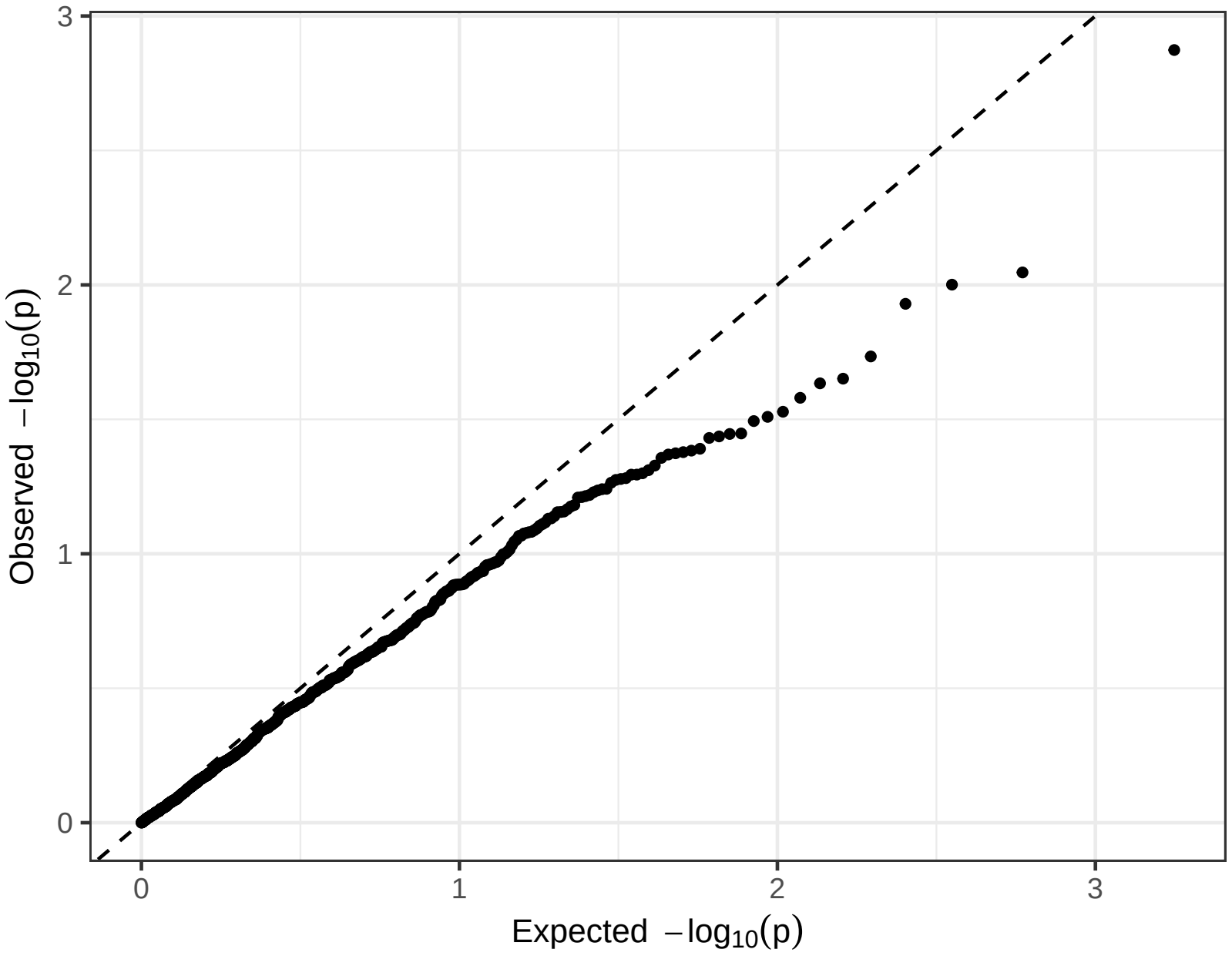

Acutalibacter

Feces | Muscle | . = 1.053

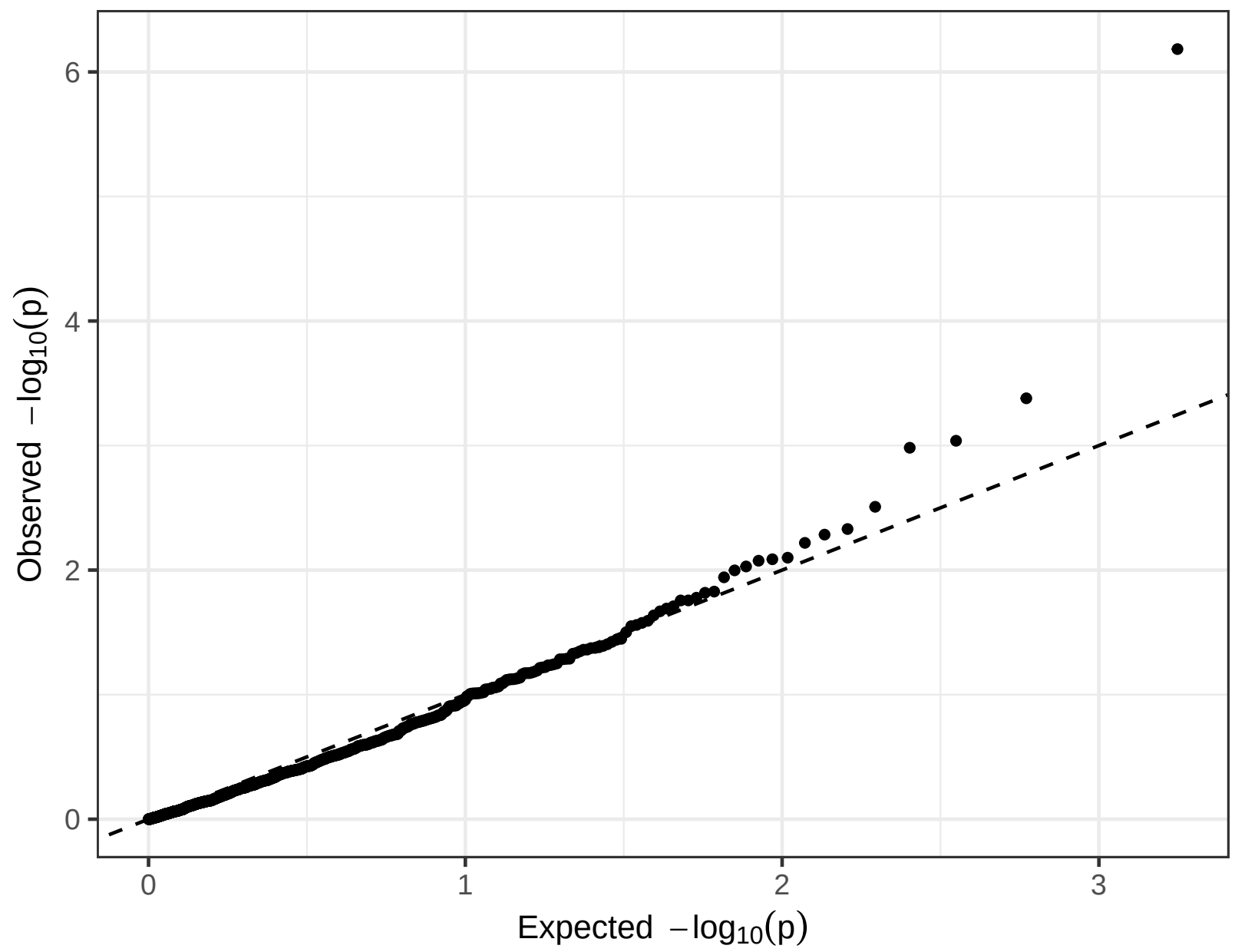

possible_genus_Sk018

Feces | Muscle | . = 1.031

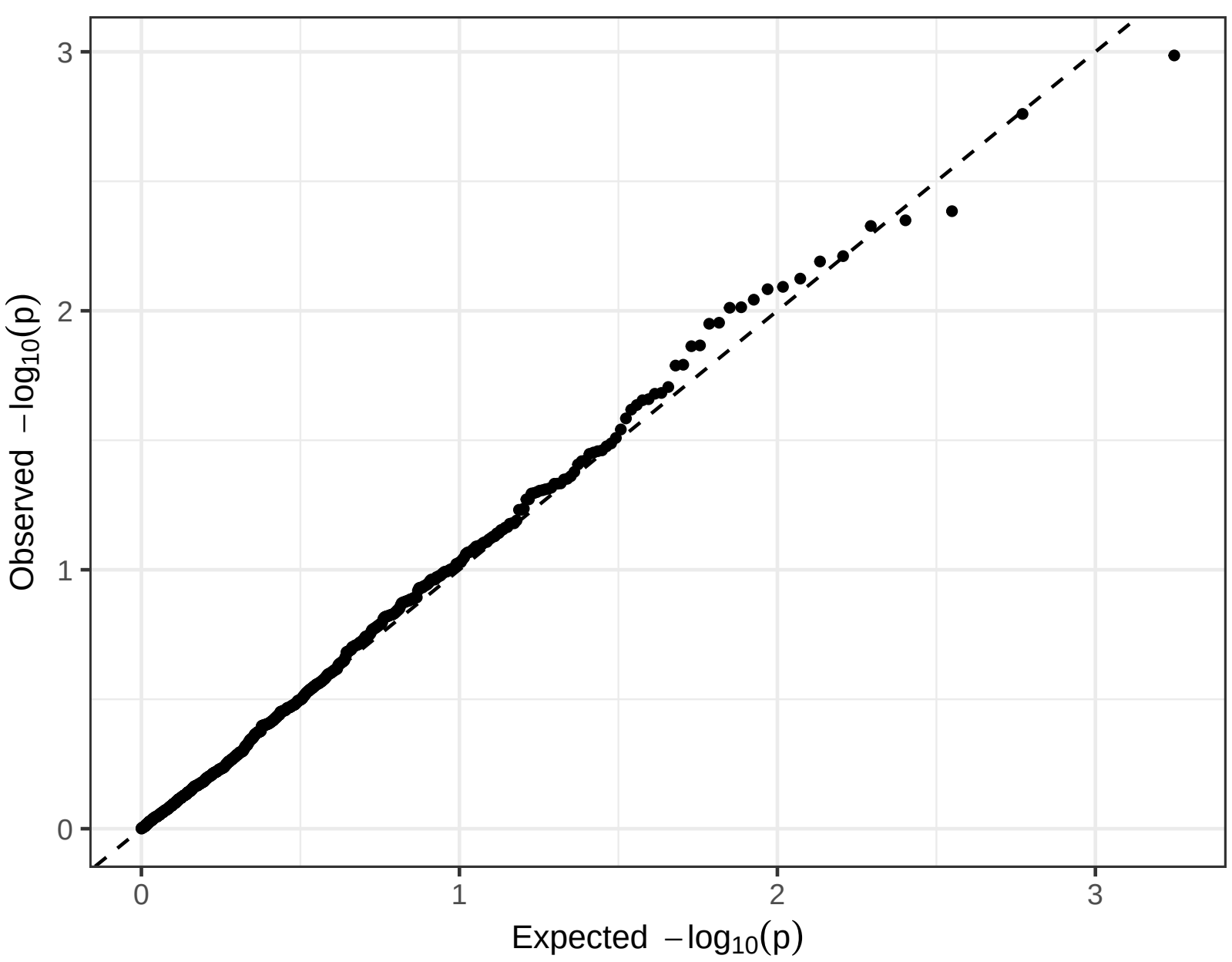

Prevotellaceae_NK3B31_group

Feces | Muscle | . = 0.846

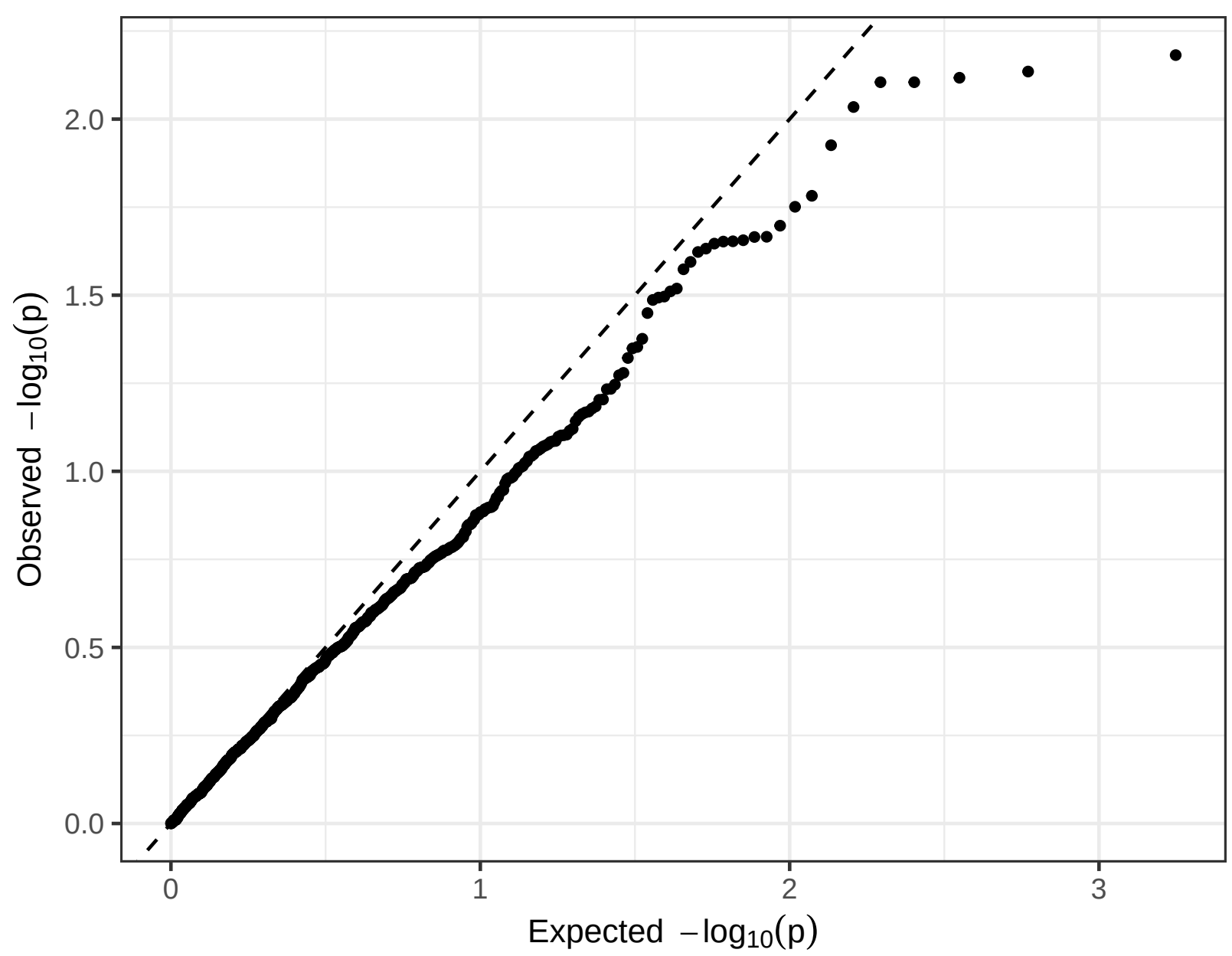

Prevotellaceae_UCG.001

Feces | Muscle | . = 0.83

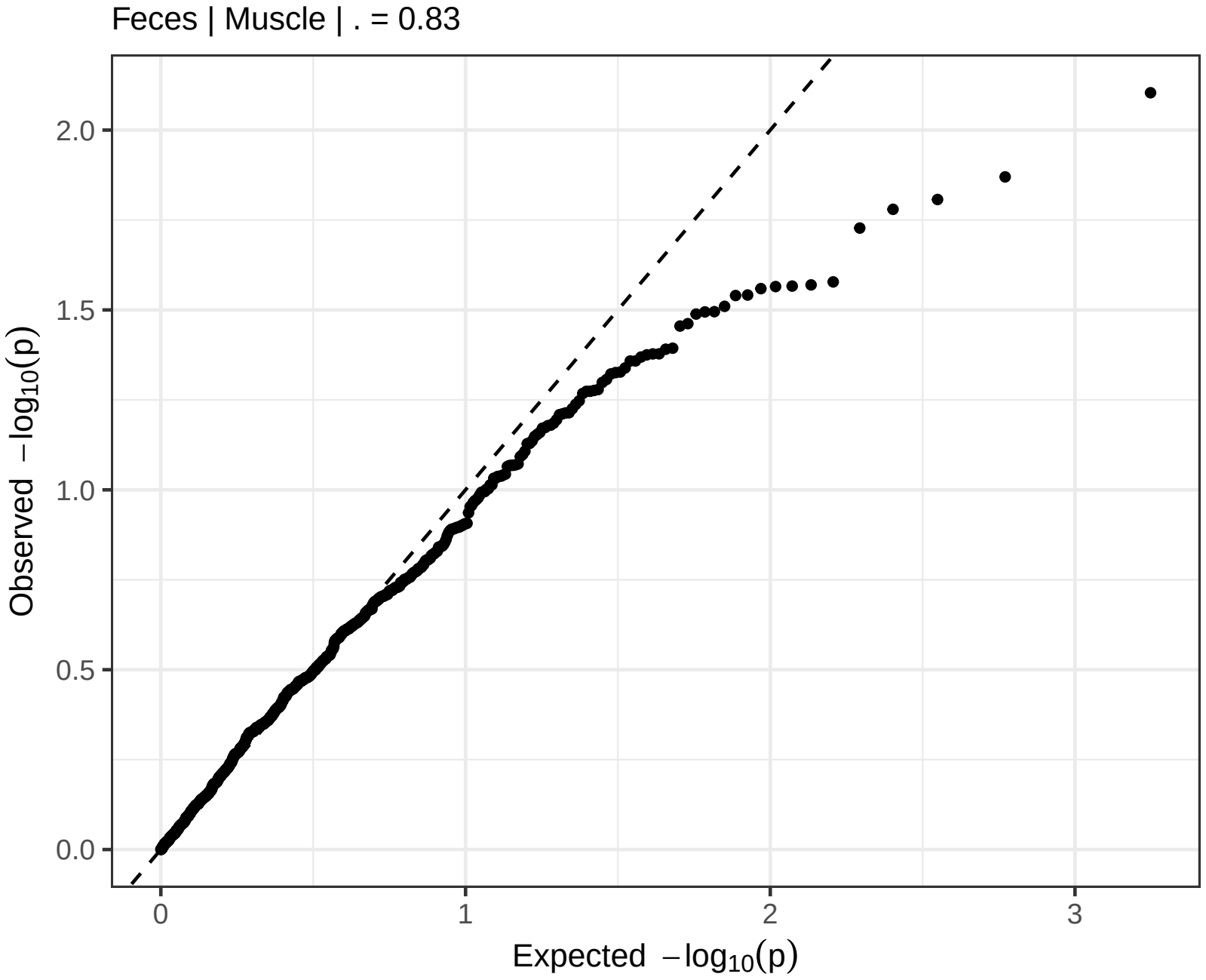

Prevotellaceae_UCG.003

Feces | Muscle | . = 0.917

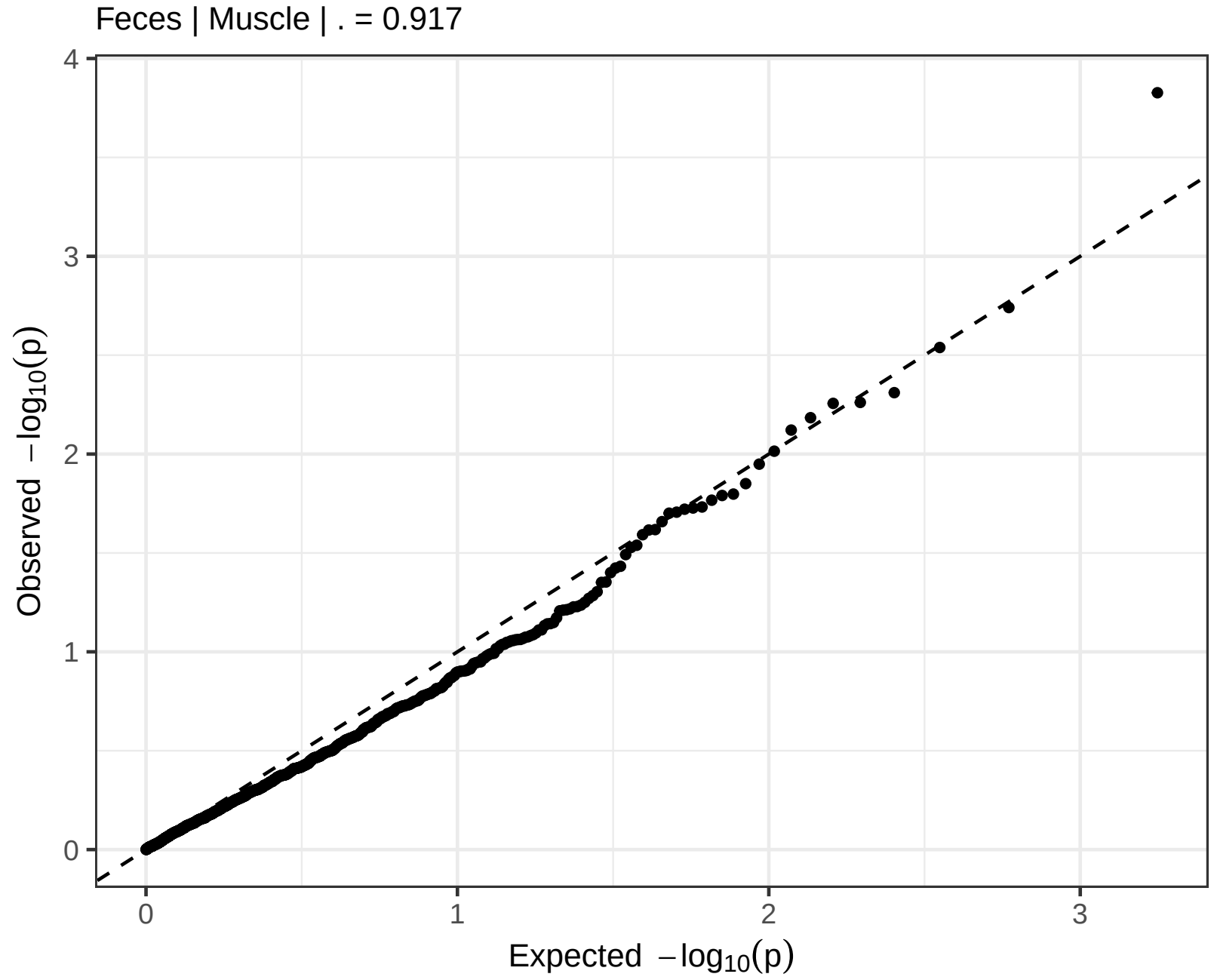

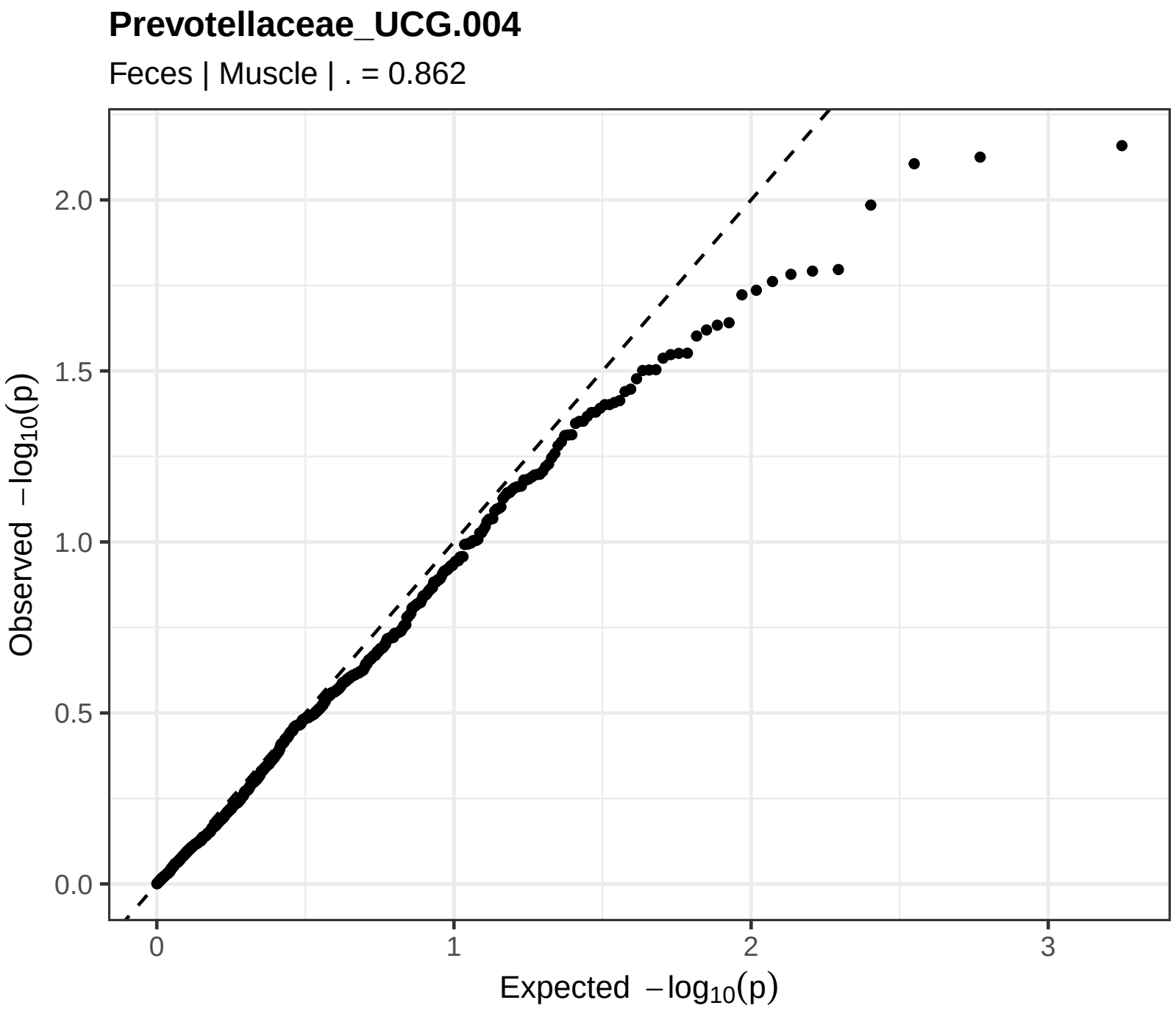
Prevotellaceae_UCG.004
Feces | Muscle | . = 0.862
Observed $-\log_{10}(p)$
Expected $-\log_{10}(p)$
0.0
0.5
1.0
1.5
2.0
0
1
2
3

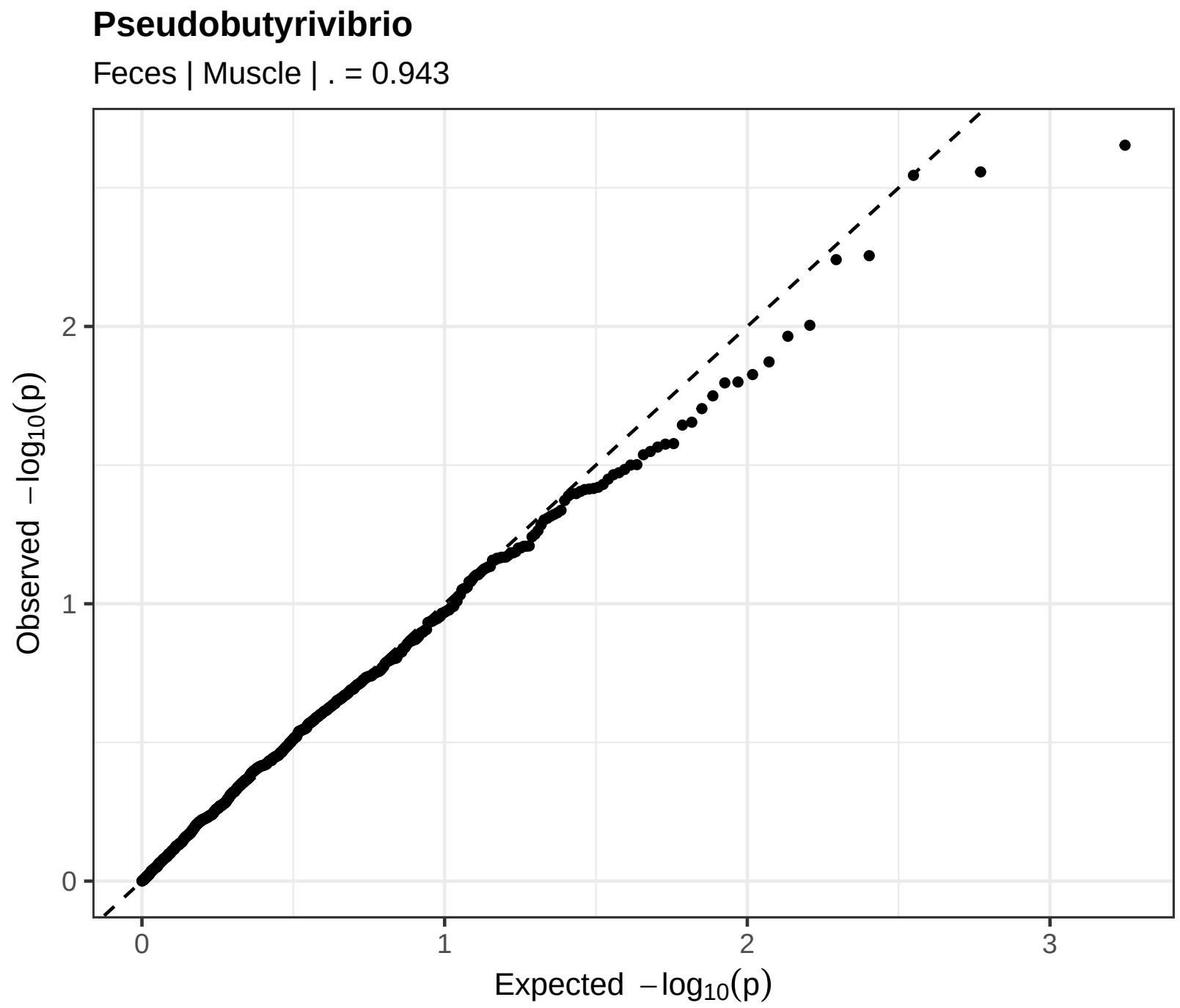
Pseudobutyrivibrio
Feces | Muscle | . = 0.943
Observed $-\log_{10}(p)$
Expected $-\log_{10}(p)$
0
1
2
0
1
2
3

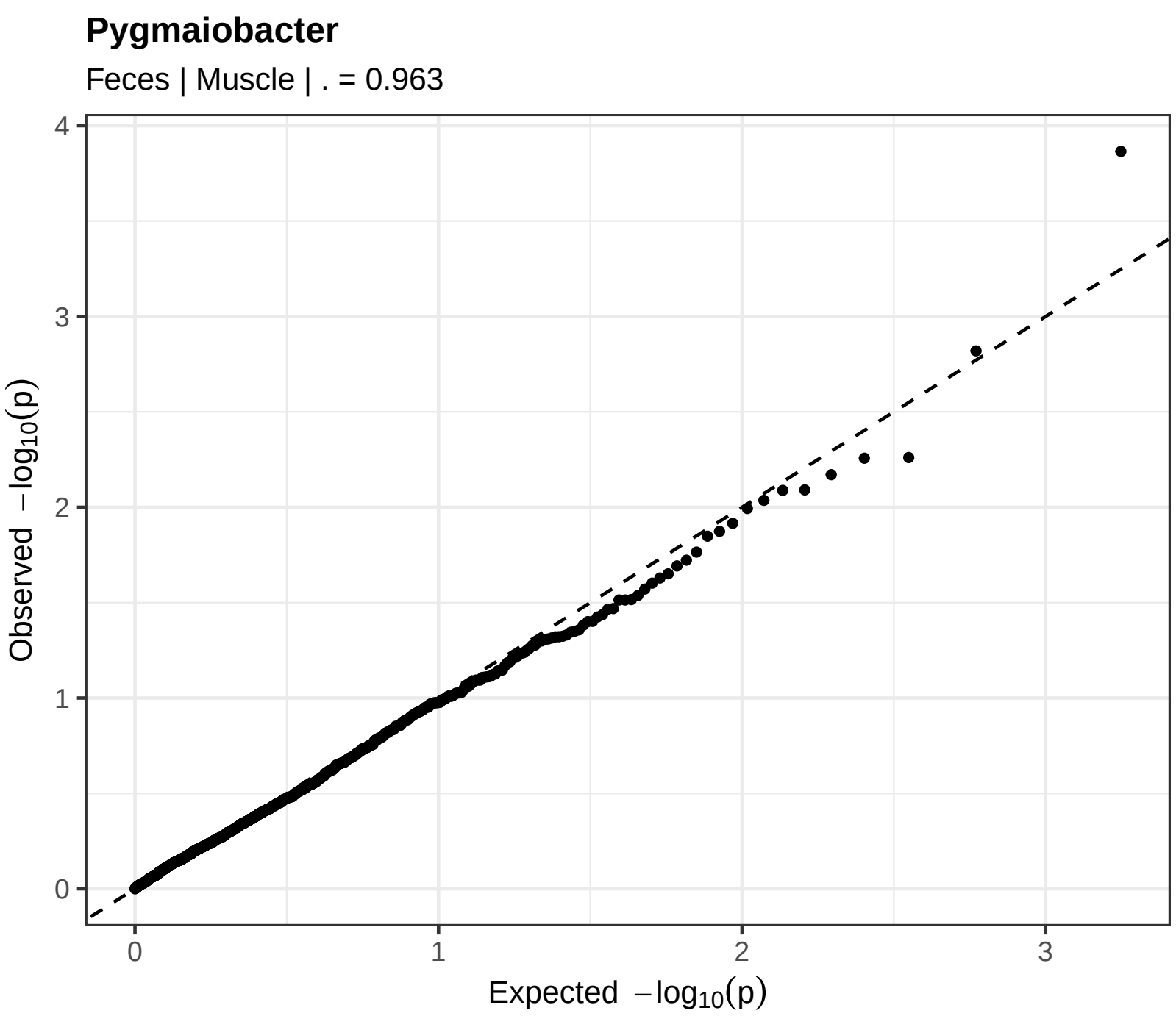
Pygmaiobacter
Feces | Muscle | . = 0.963
Observed $-\log_{10}(p)$
Expected $-\log_{10}(p)$
0
1
2
3
4
0
1
2
3

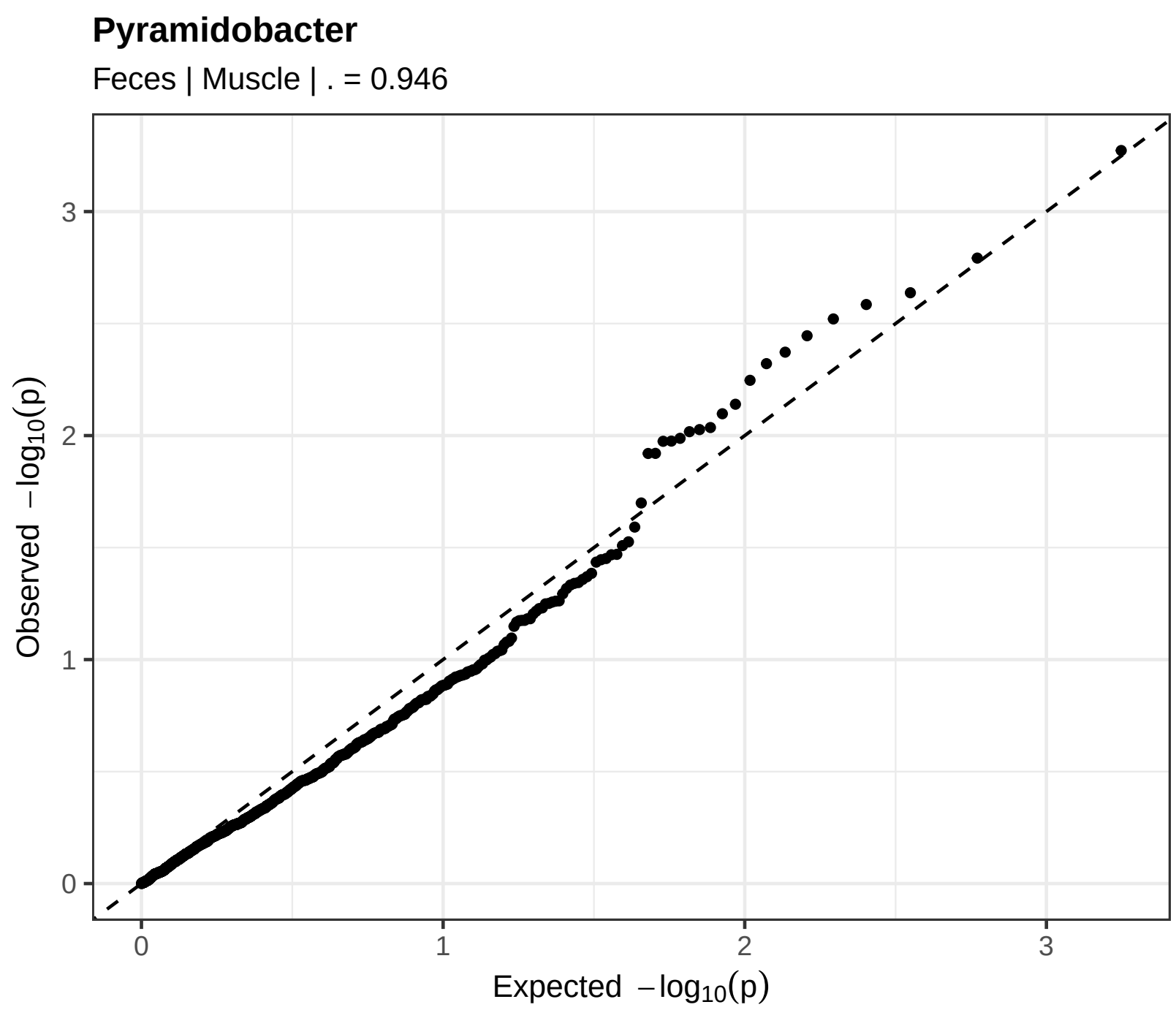
Pyramidobacter
Feces | Muscle | . = 0.946
Observed $-\log_{10}(p)$
Expected $-\log_{10}(p)$
0
1
2
3
0
1
2
3

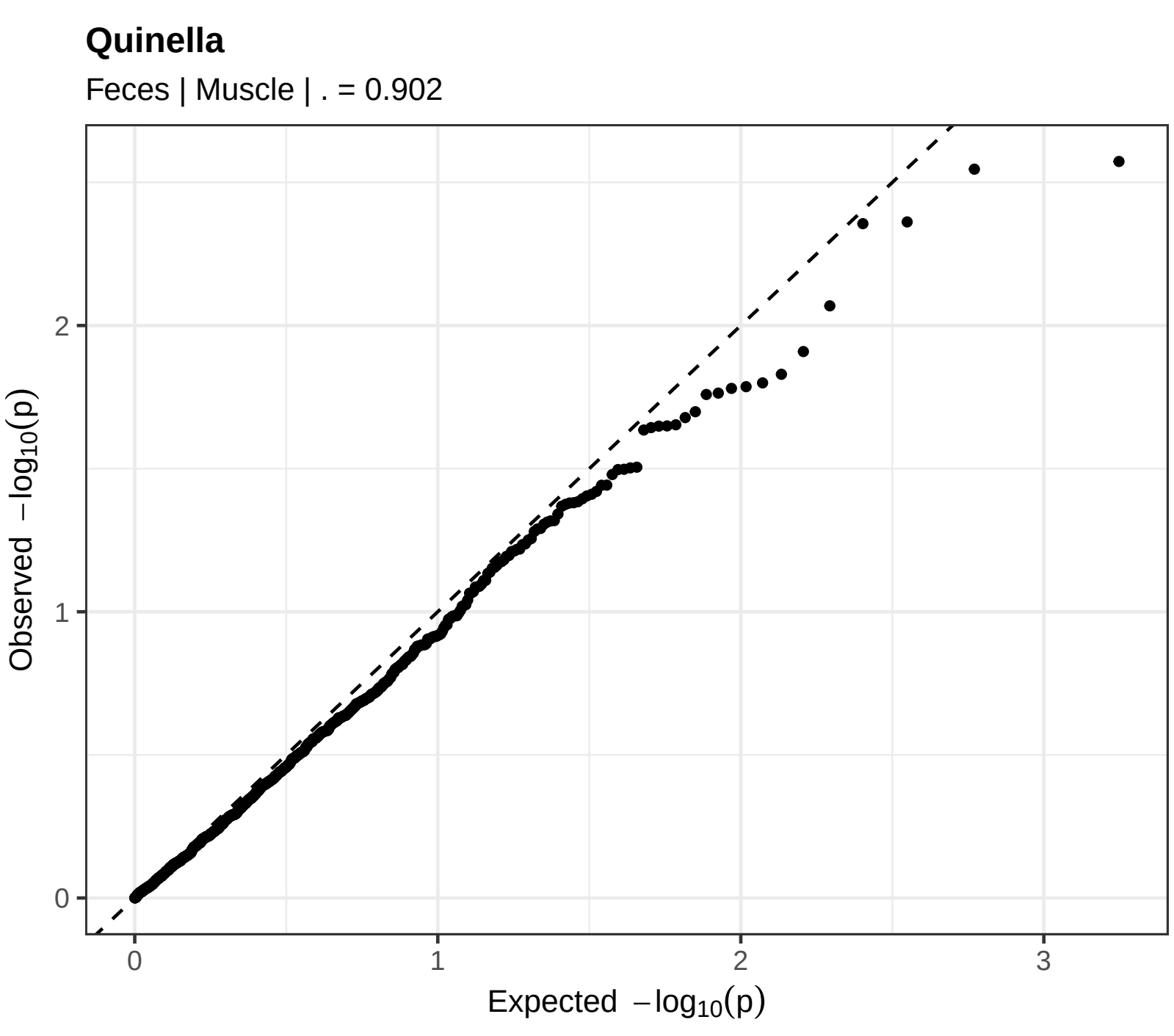
Quinella
Feces | Muscle | . = 0.902
Observed $-\log_{10}(p)$
Expected $-\log_{10}(p)$
0
1
2
0
1
2
3

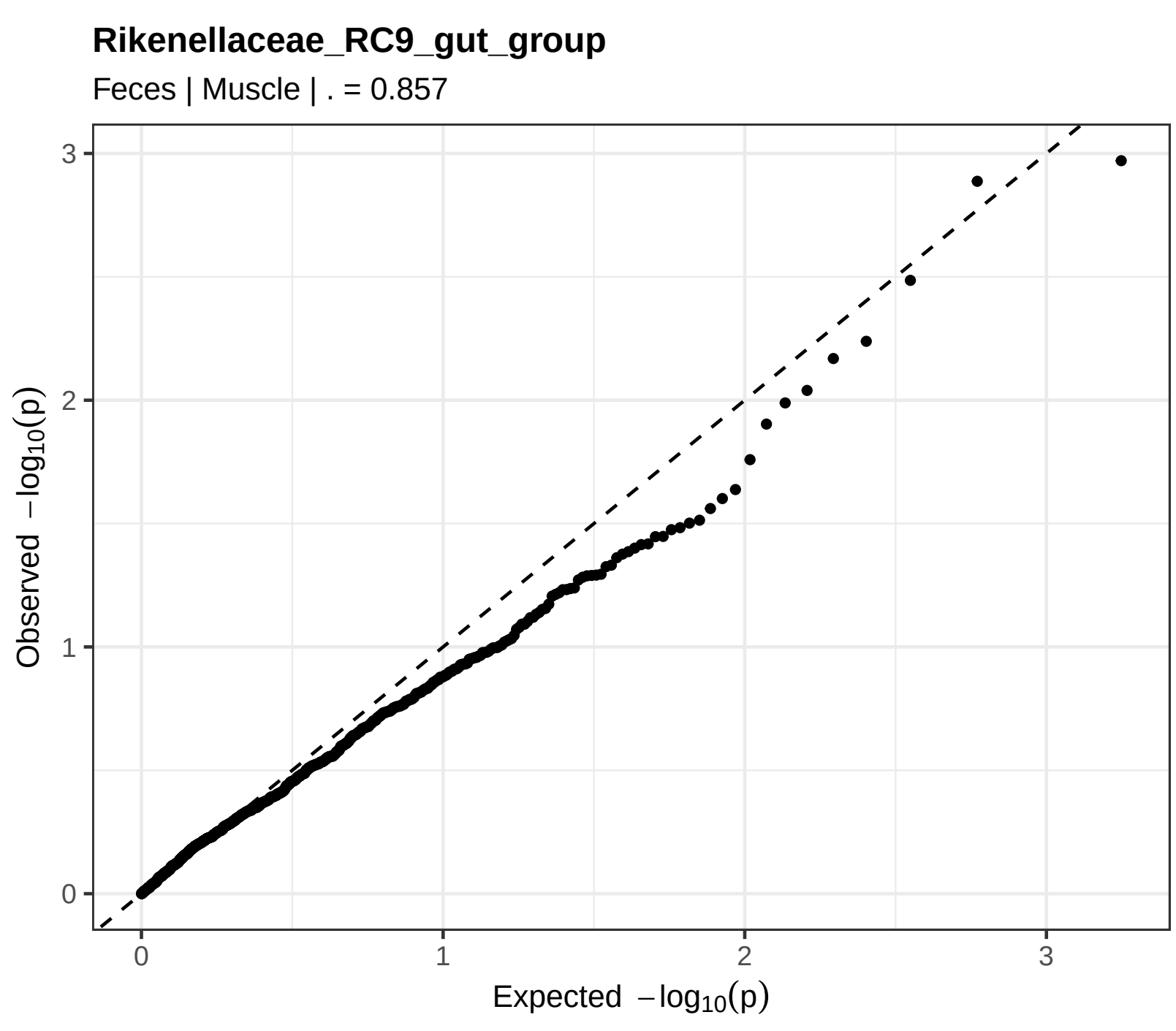
Rikenellaceae_RC9_gut_group
Feces | Muscle | . = 0.857
Observed $-\log_{10}(p)$
Expected $-\log_{10}(p)$
0
1
2
3
0
1
2
3

Adlercreutzia

Feces | Muscle | . = 1.05

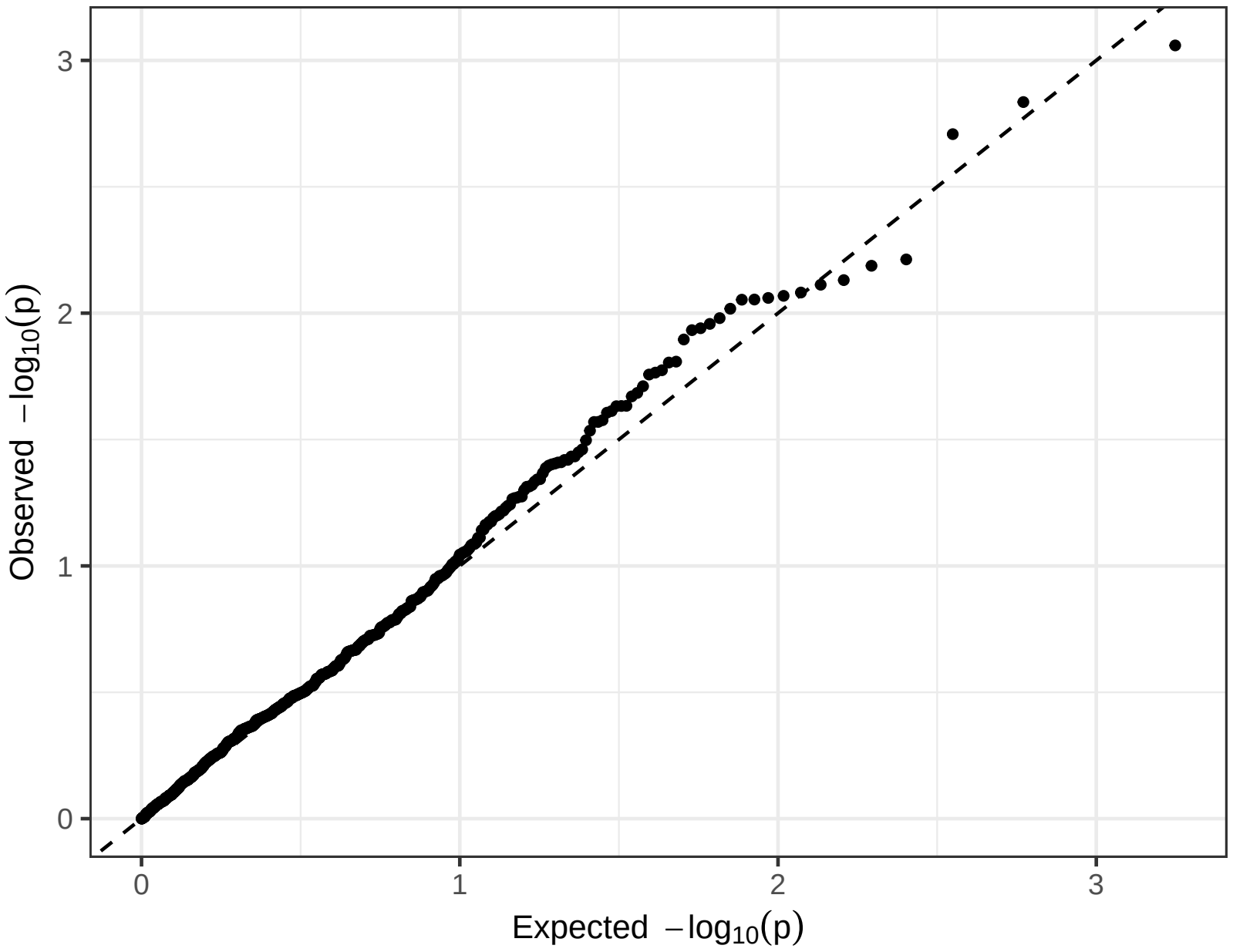

Romboutsia

Feces | Muscle | . = 1.019

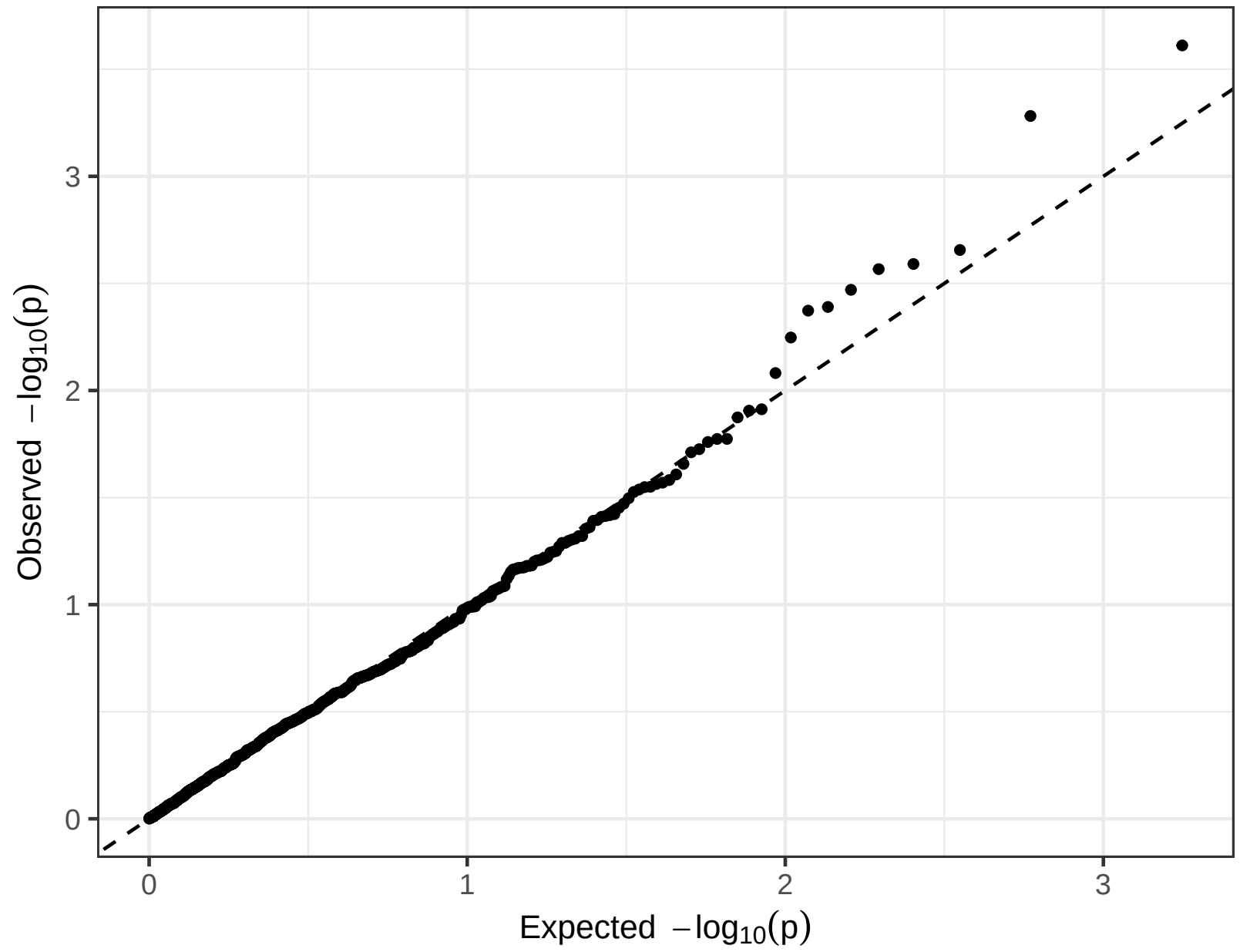

Roseburia

Feces | Muscle | . = 1.132

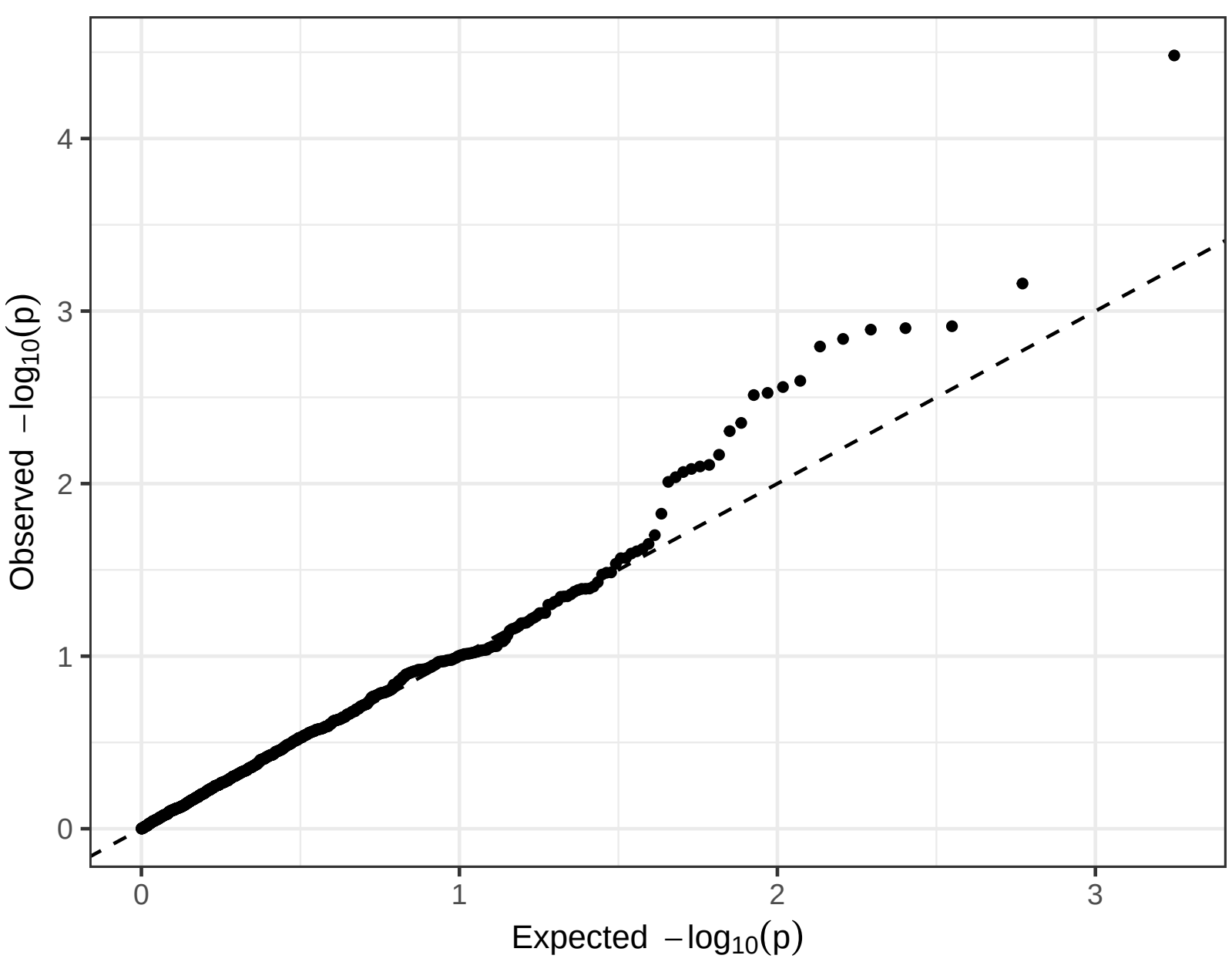

RumEn_M2

Feces | Muscle | . = 0.972

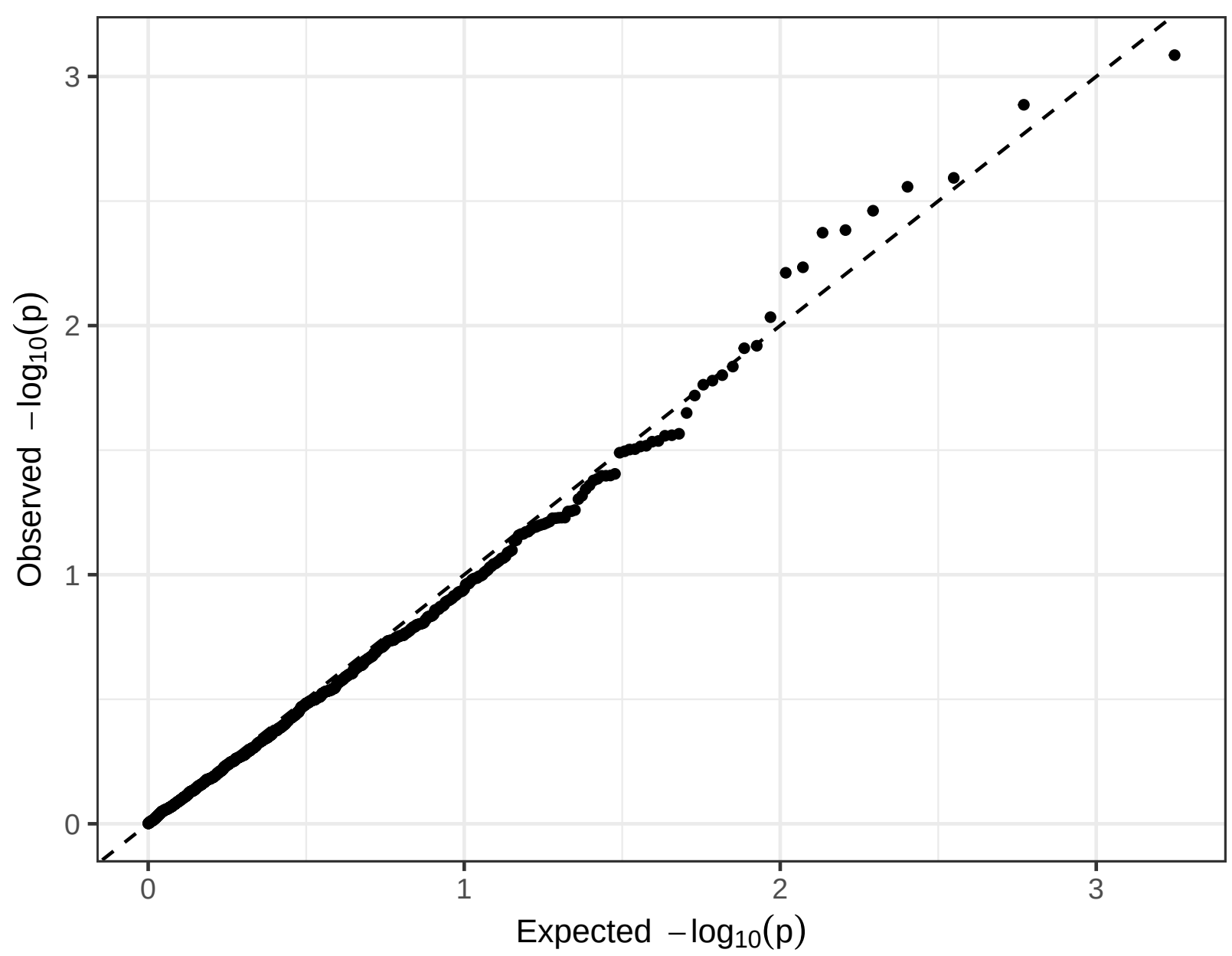

Ruminobacter

Feces | Muscle | . = 0.869

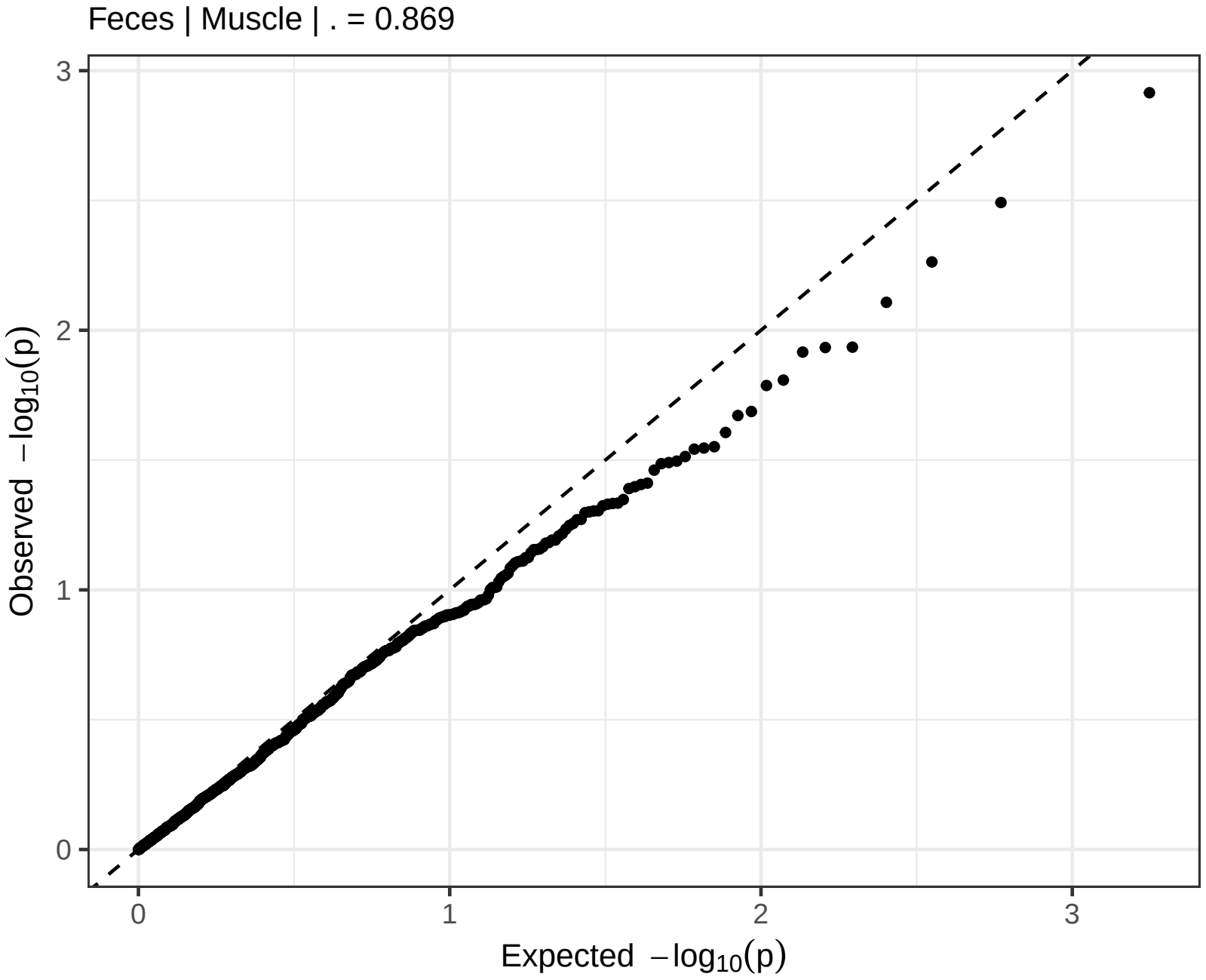

Ruminococcus

Feces | Muscle | . = 0.912

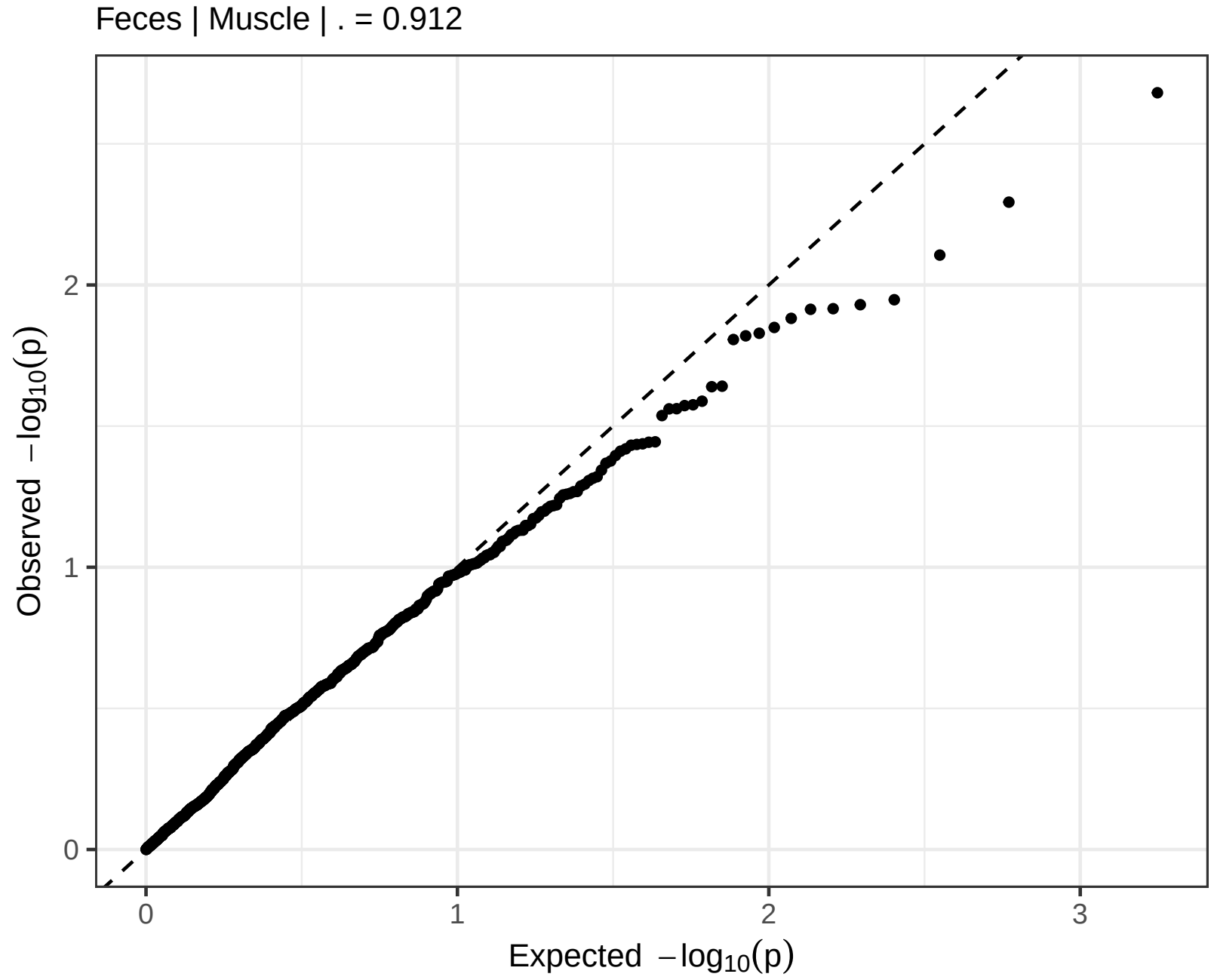

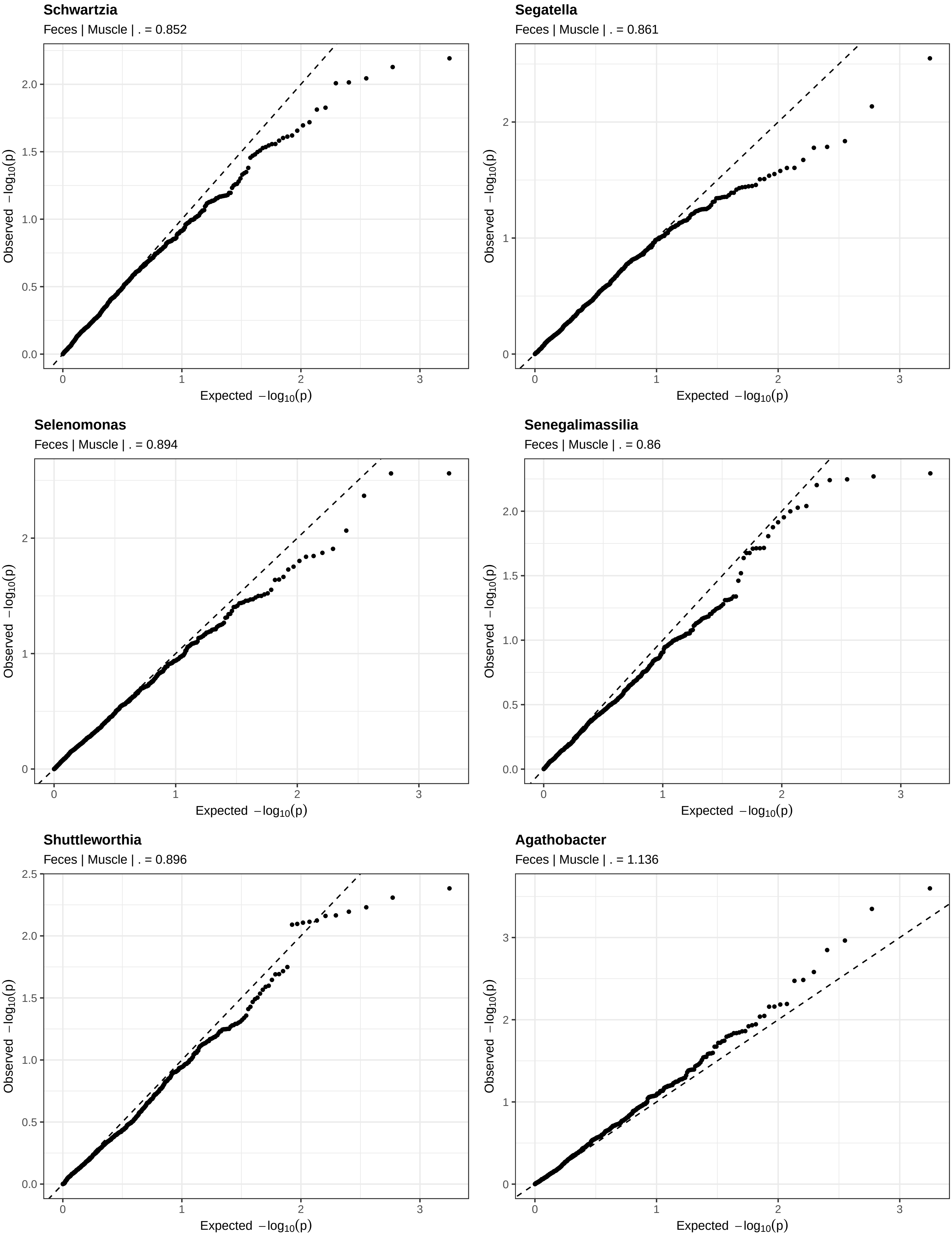
Schwartzia
Feces | Muscle | . = 0.852
Segatella
Feces | Muscle | . = 0.861
Selenomonas
Feces | Muscle | . = 0.894
Senegalimassilia
Feces | Muscle | . = 0.86
Shuttleworthia
Feces | Muscle | . = 0.896
Agathobacter
Feces | Muscle | . = 1.136
Observed −log10(p)
Expected −log10(p)

Slackia

Feces | Muscle | . = 1.022

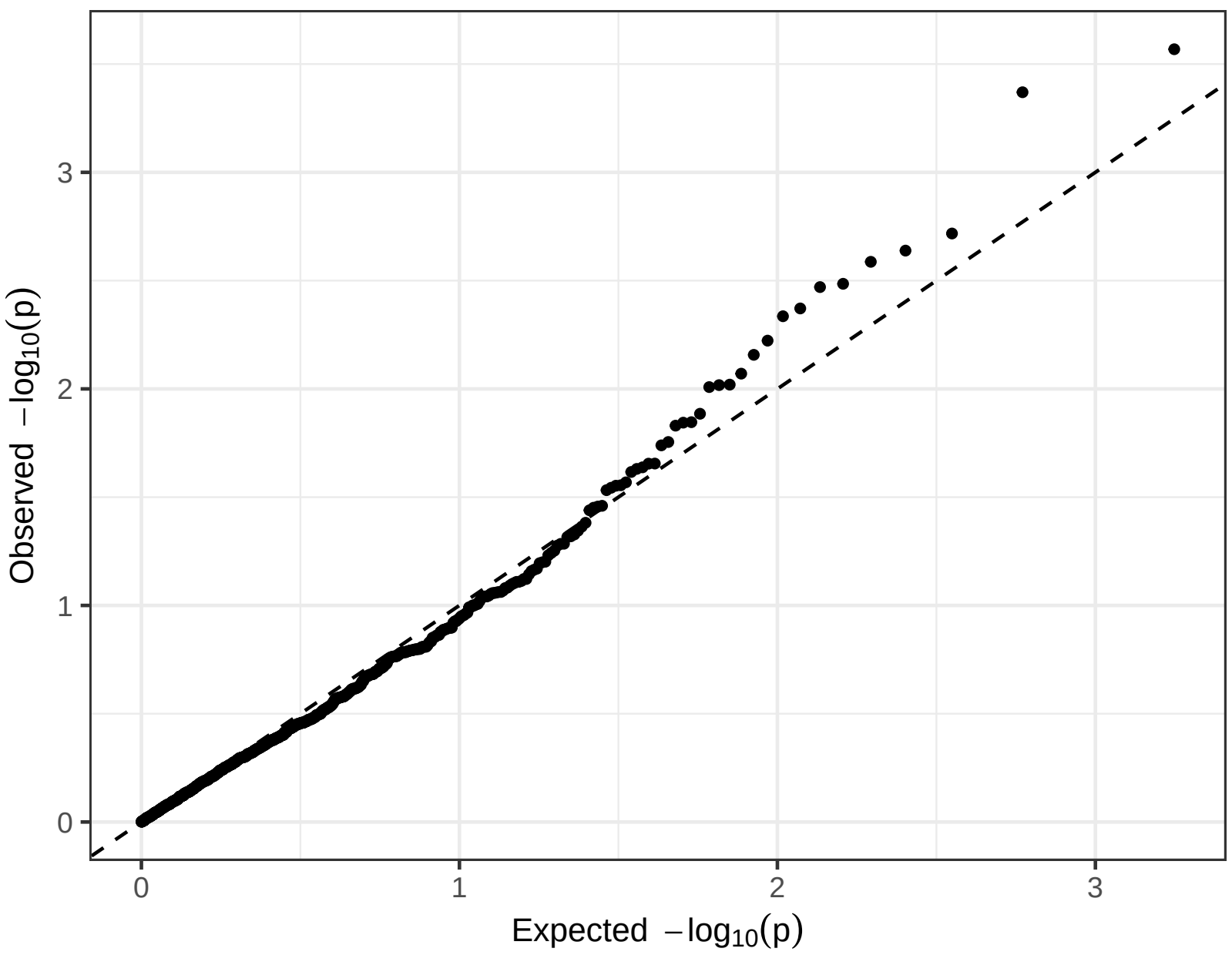

Solobacterium

Feces | Muscle | . = 0.834

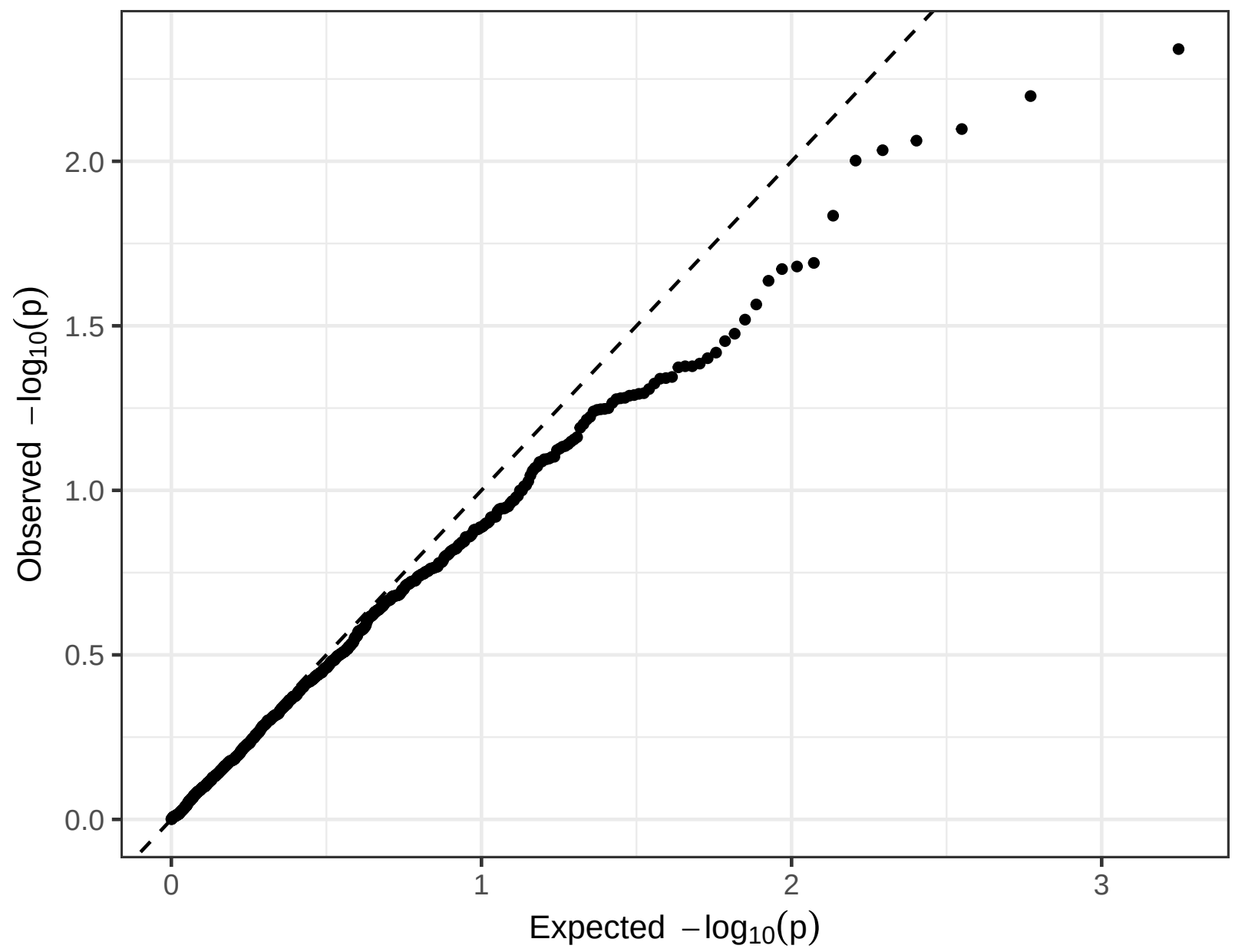

Streptococcus

Feces | Muscle | . = 1.003

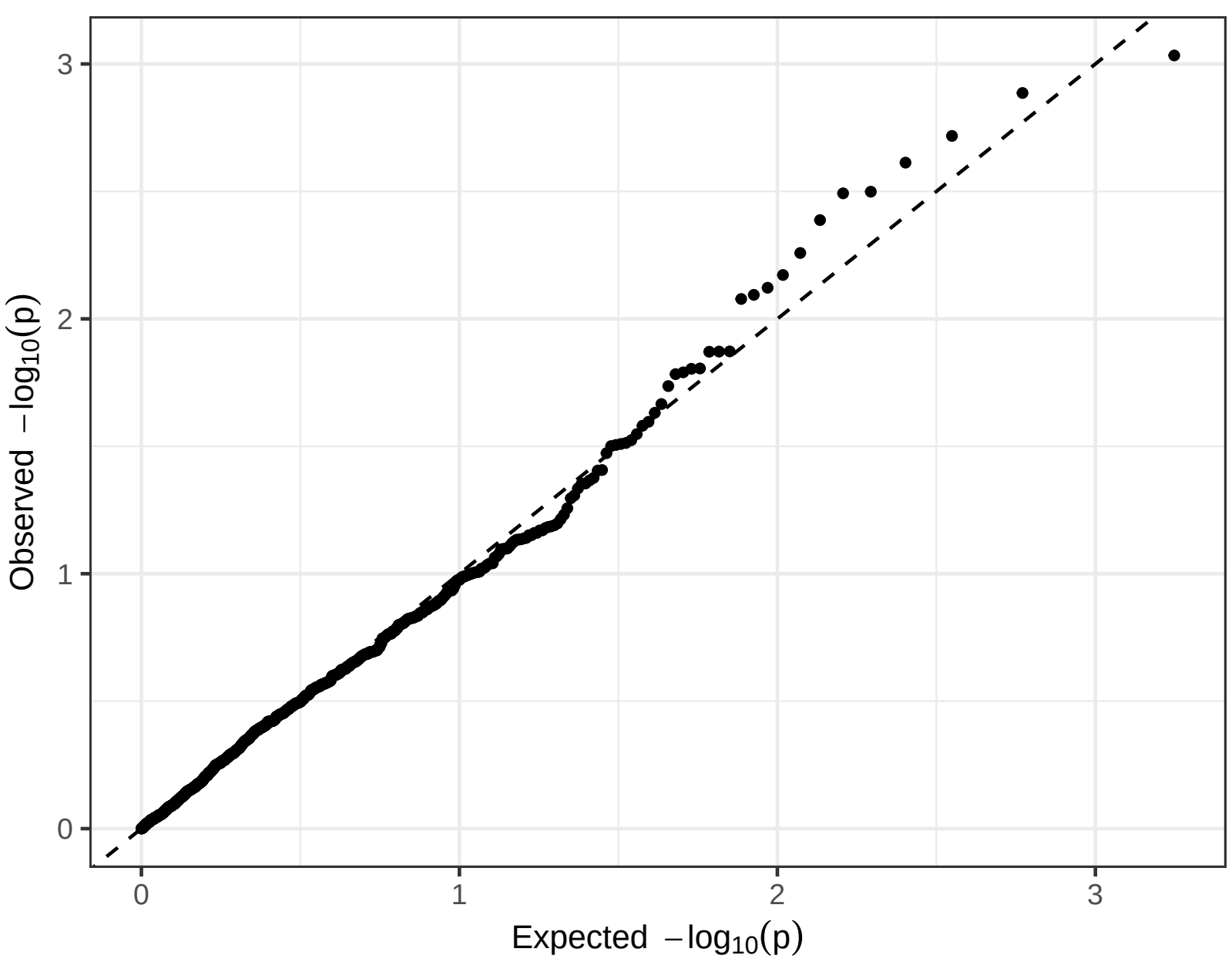

Succinivibrio

Feces | Muscle | . = 0.902

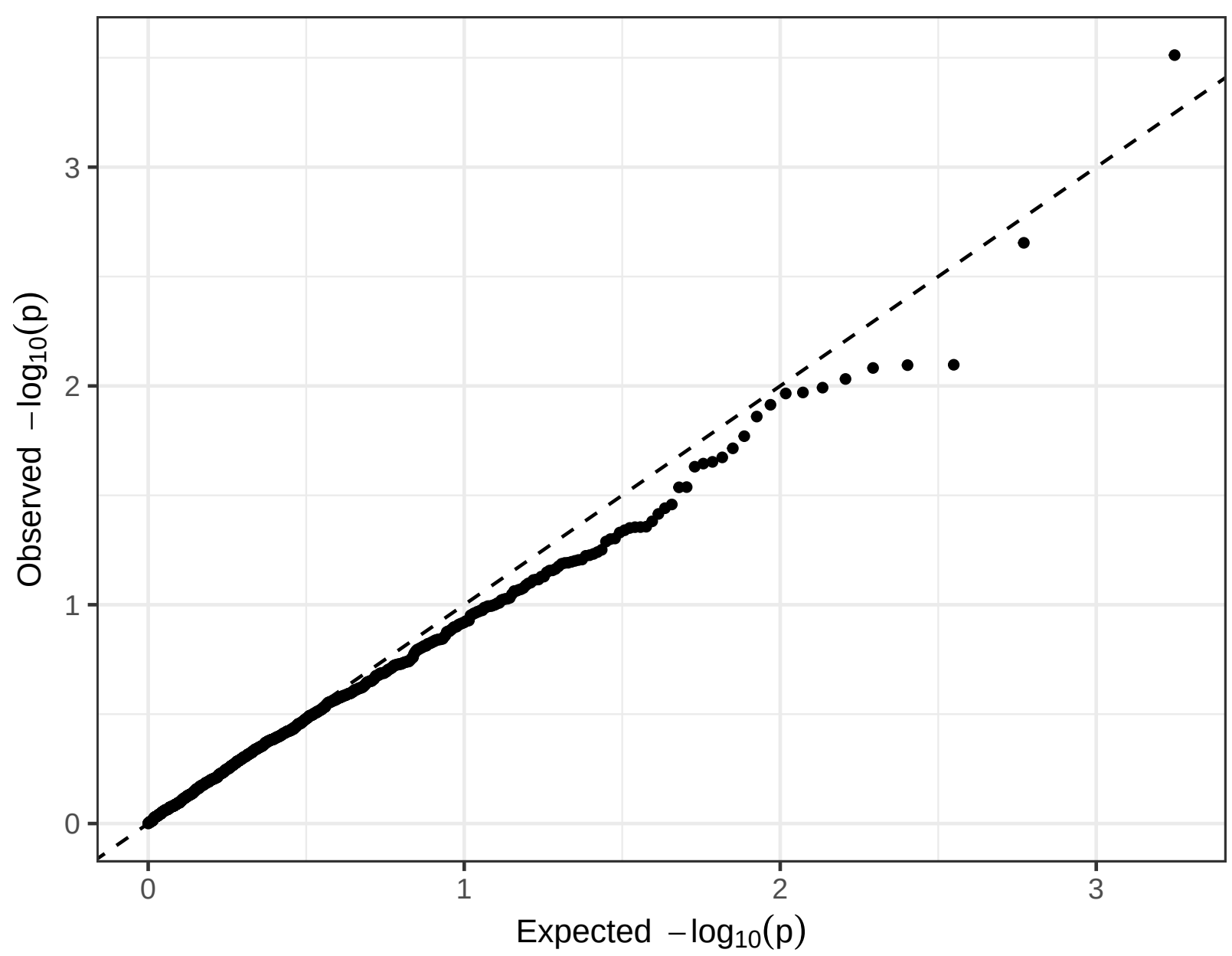

Succinivibrionaceae_UCG.001

Feces | Muscle | . = 0.831

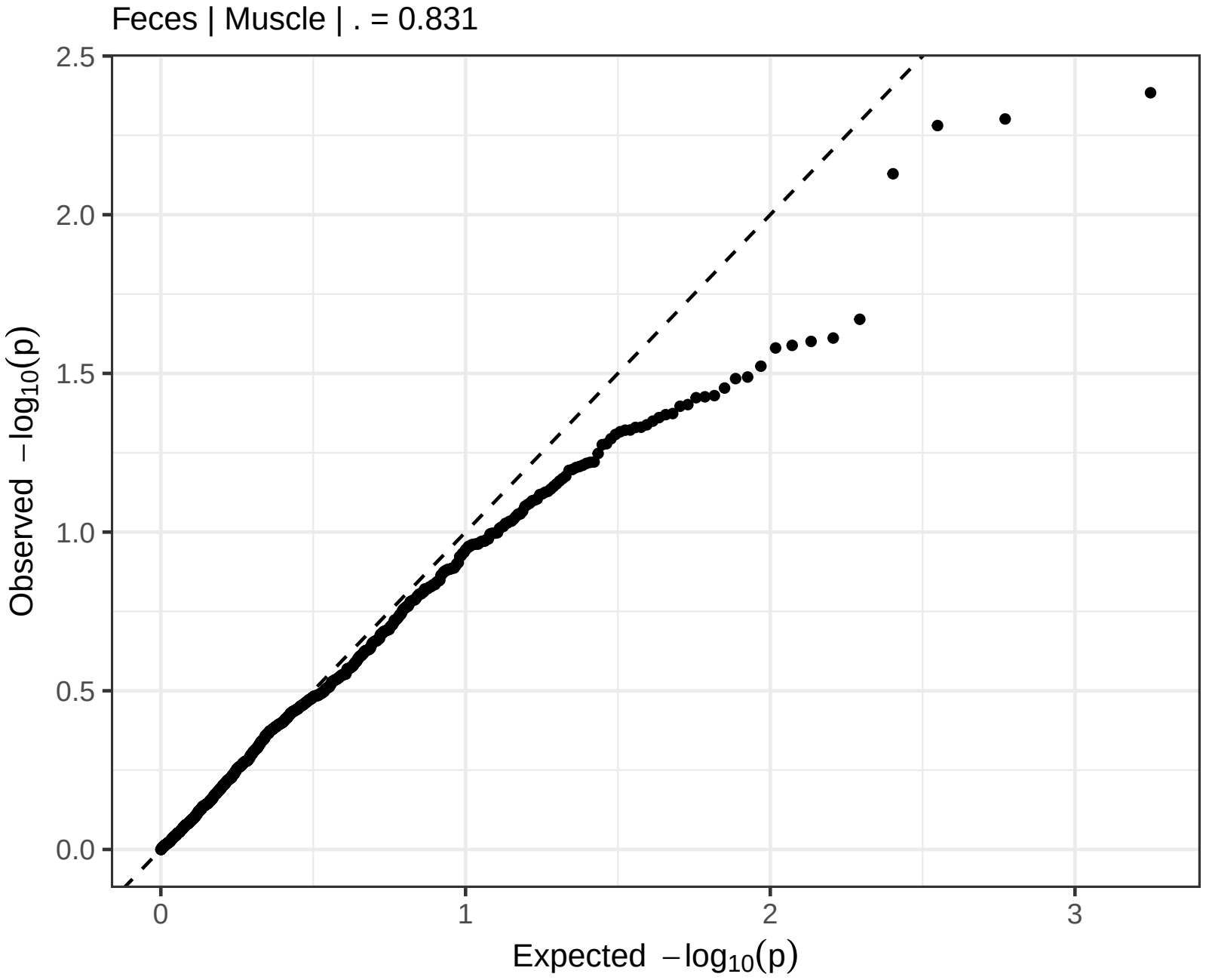

Sutterella

Feces | Muscle | . = 0.925

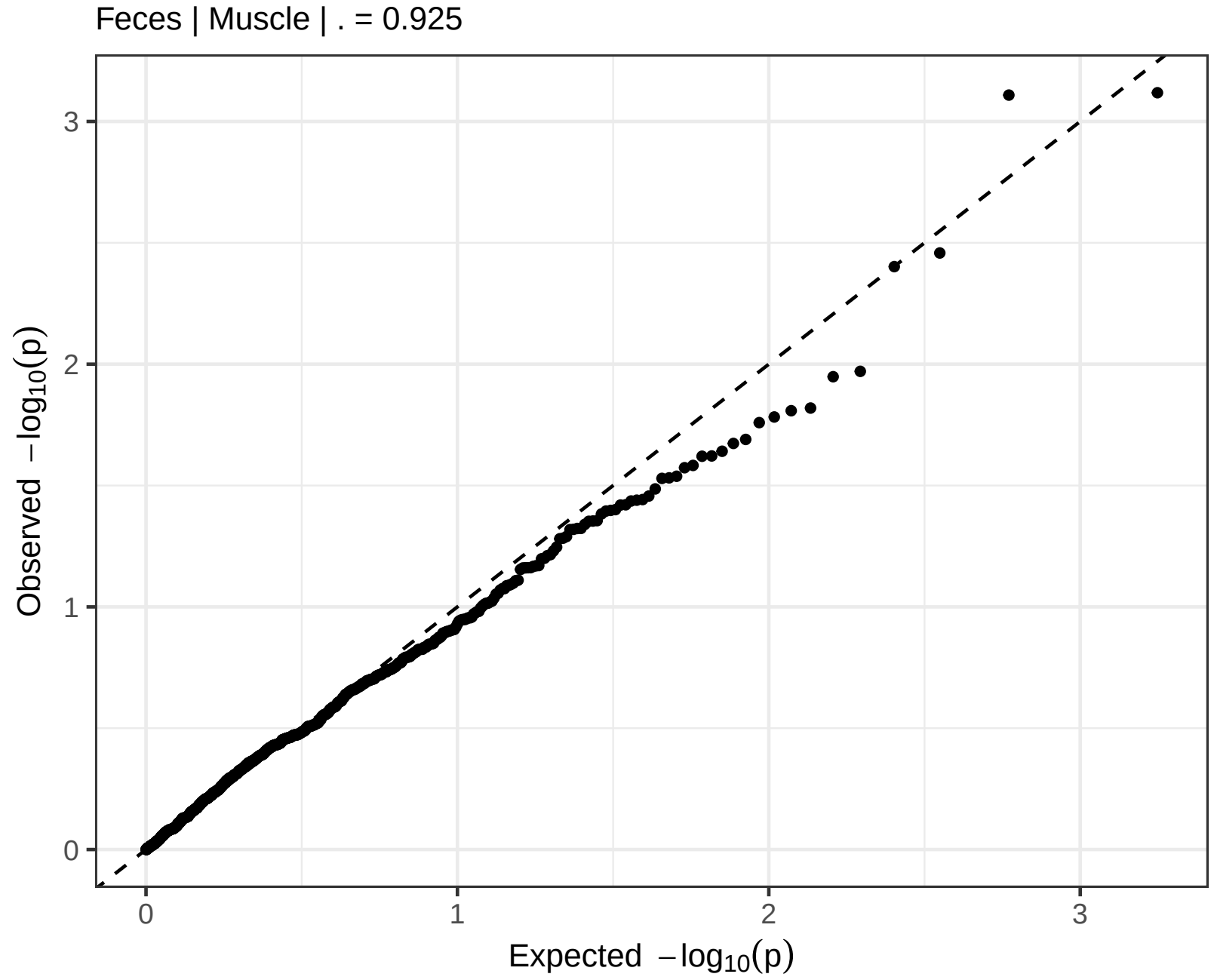

T2WK15B57

Feces | Muscle | . = 1.02

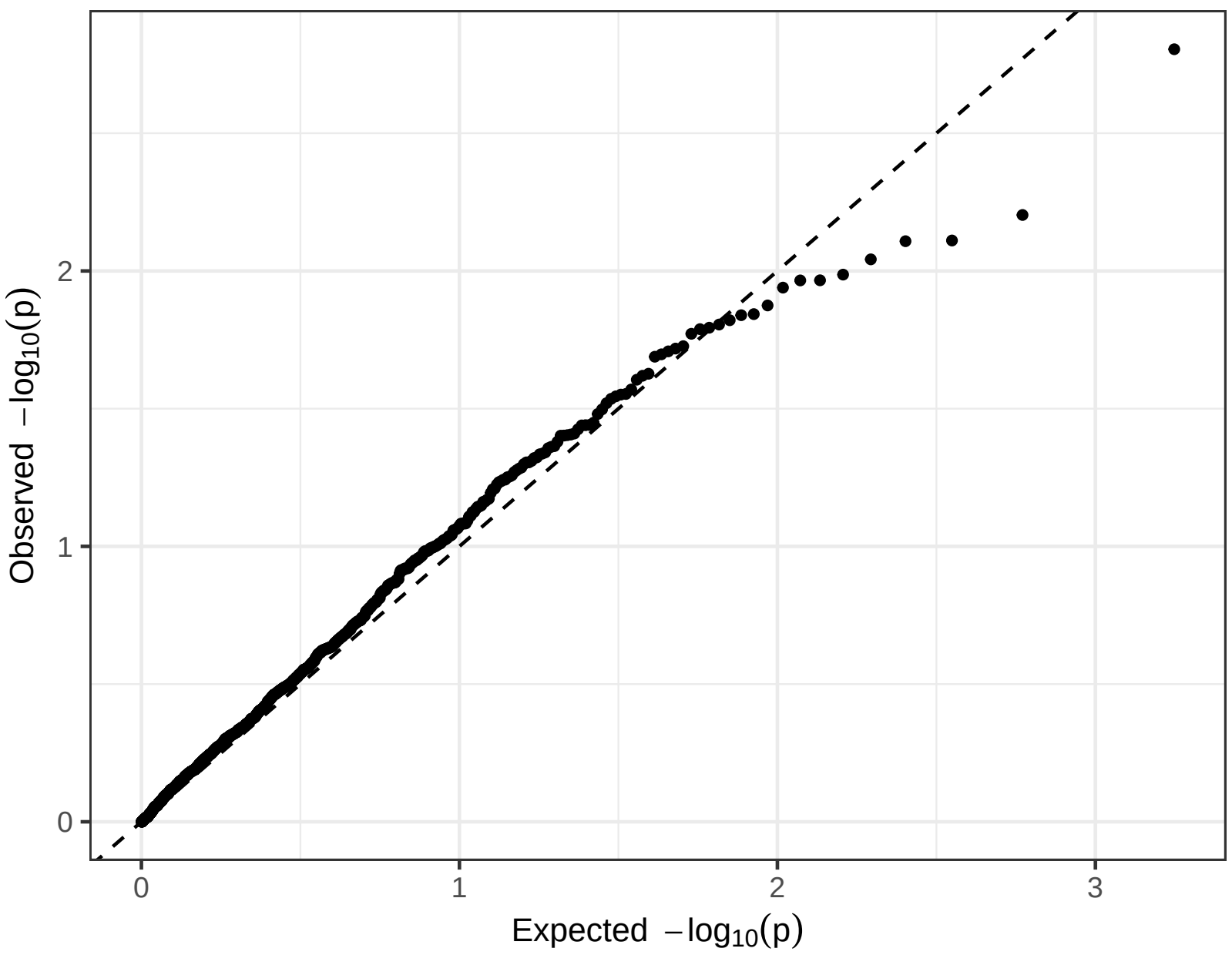

Terrisporobacter

Feces | Muscle | . = 1

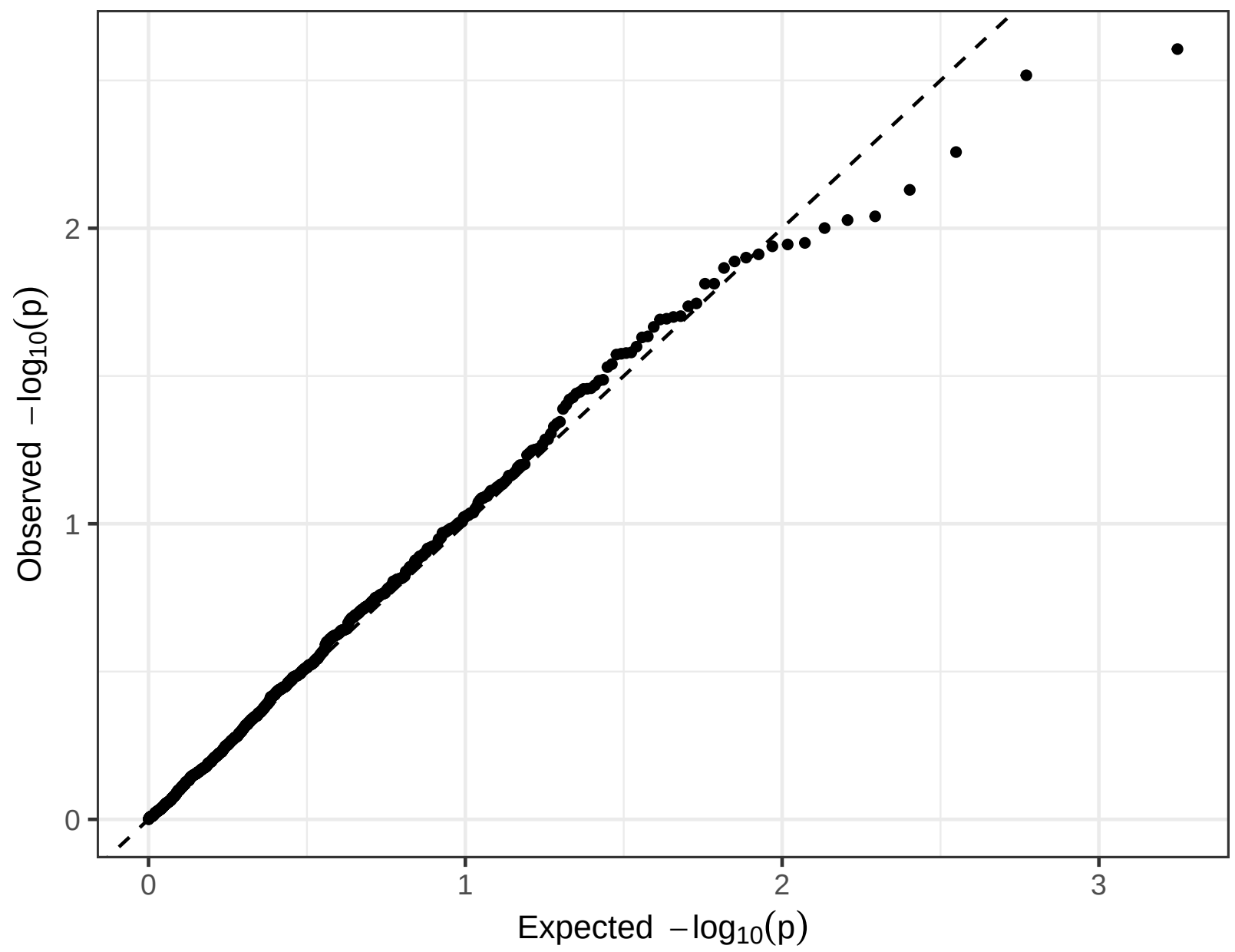

Tractidigestivibacter

Feces | Muscle | . = 0.87

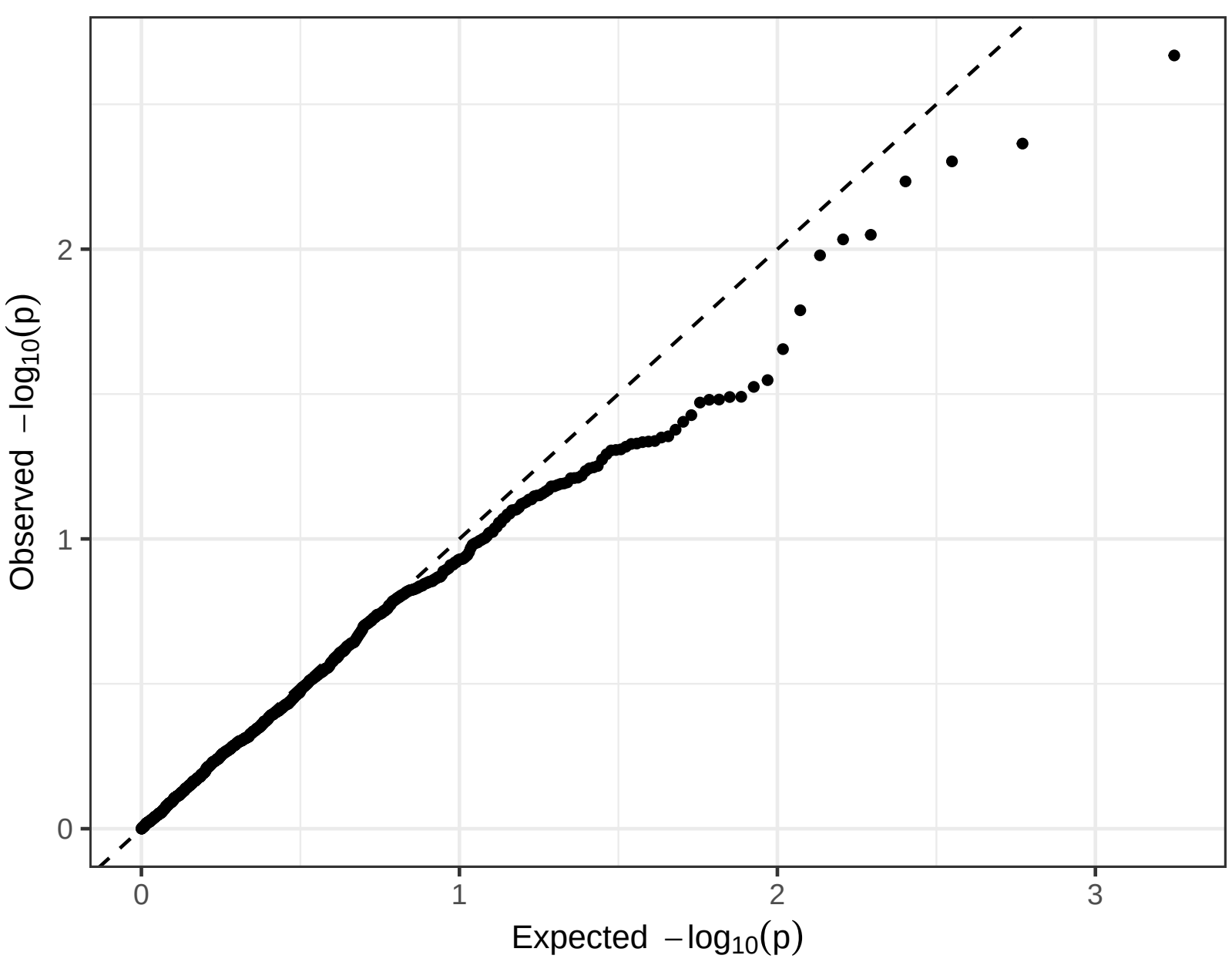

Treponema

Feces | Muscle | . = 0.804

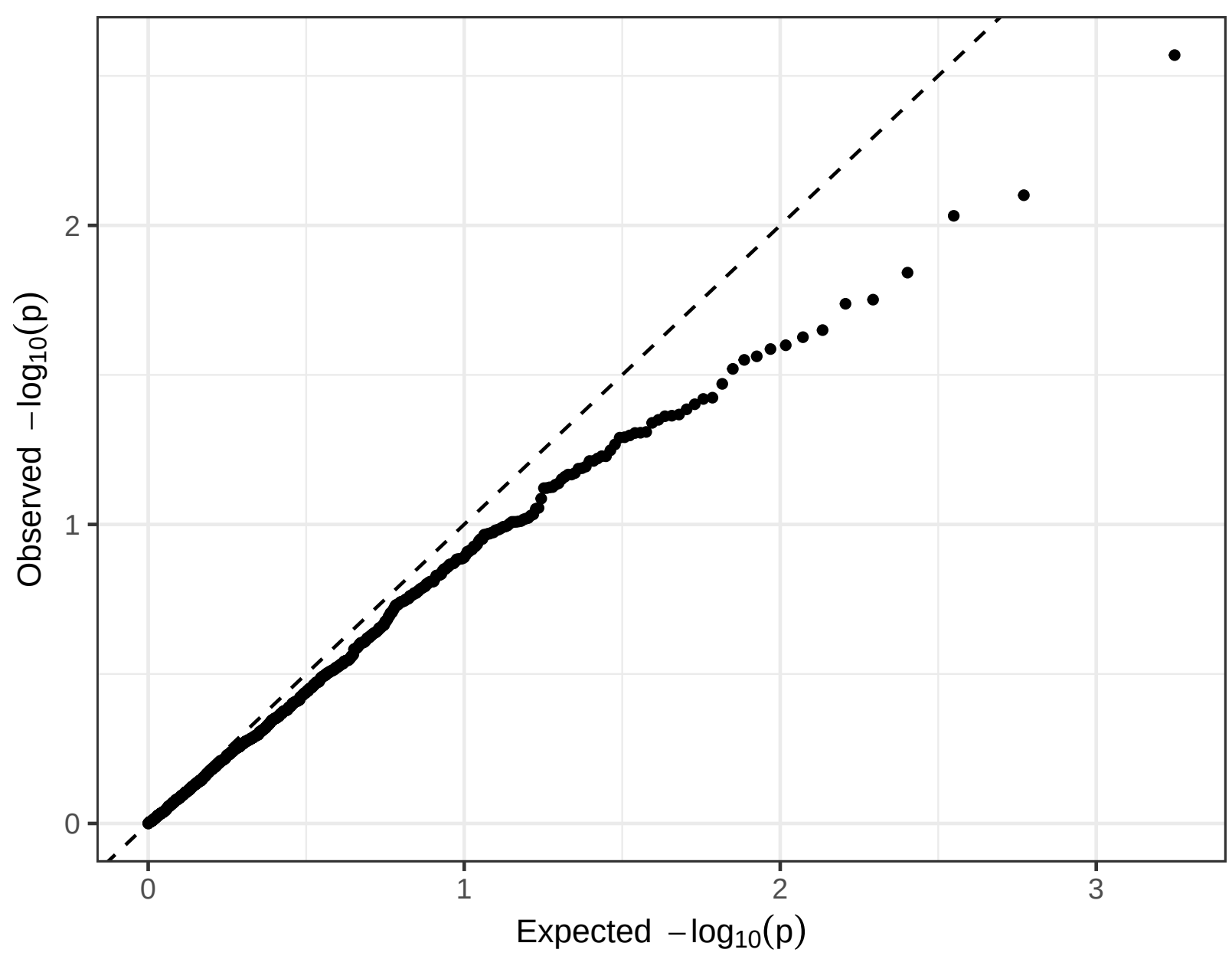

Agathobaculum

Feces | Muscle | . = 0.808

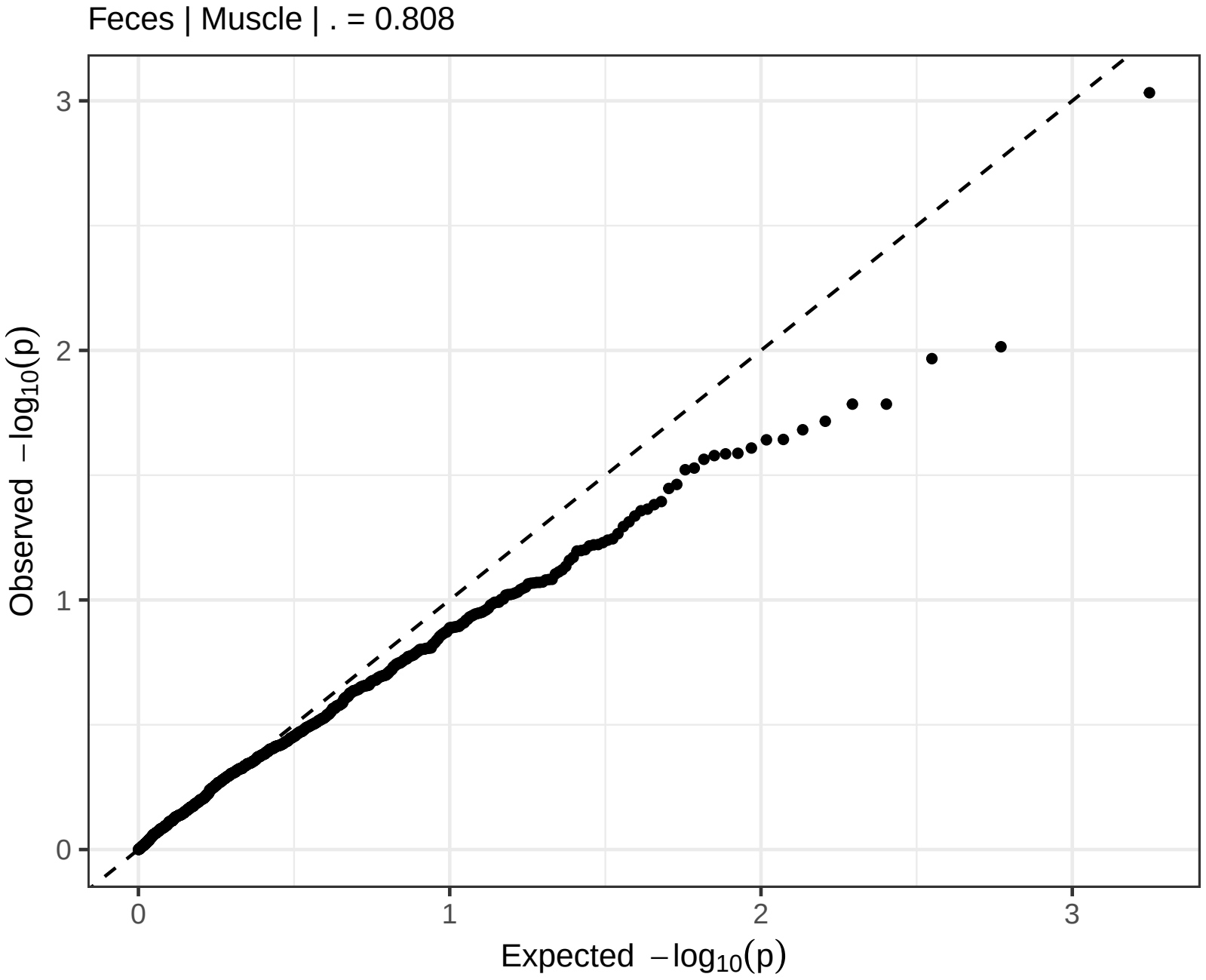

Turicibacter

Feces | Muscle | . = 1.066

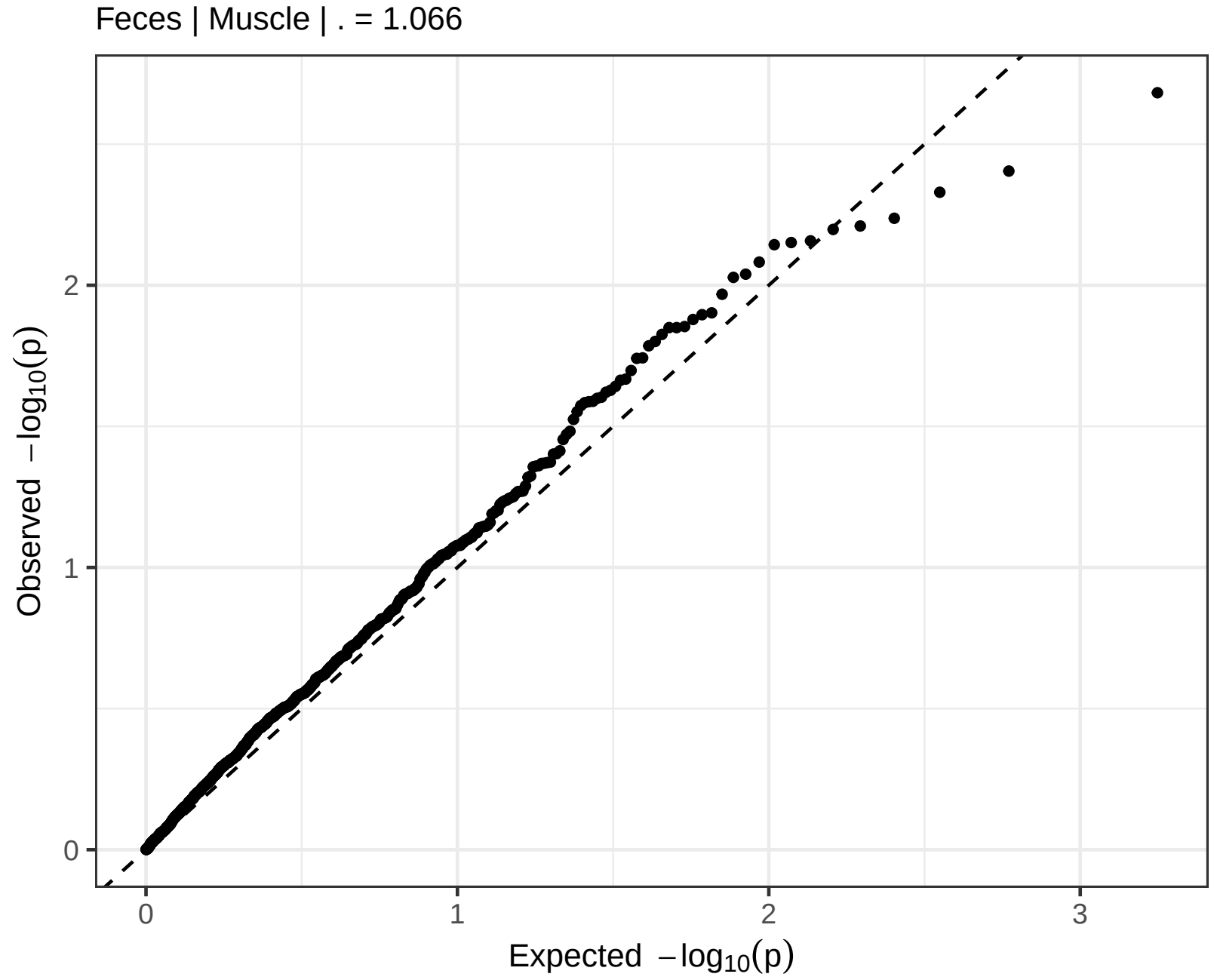

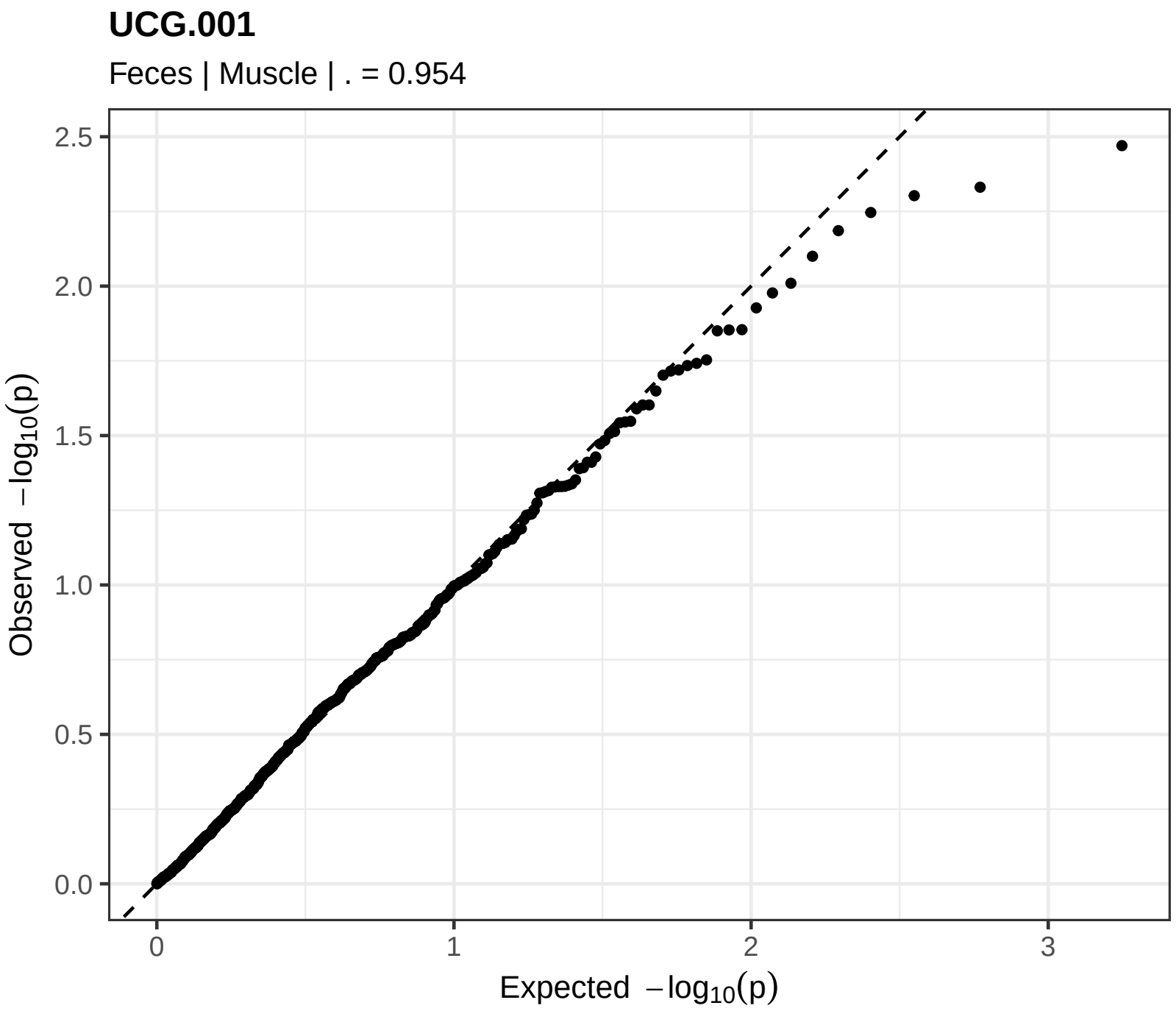
UCG.001
Feces | Muscle | . = 0.954
Observed −log10(p)
Expected −log10(p)

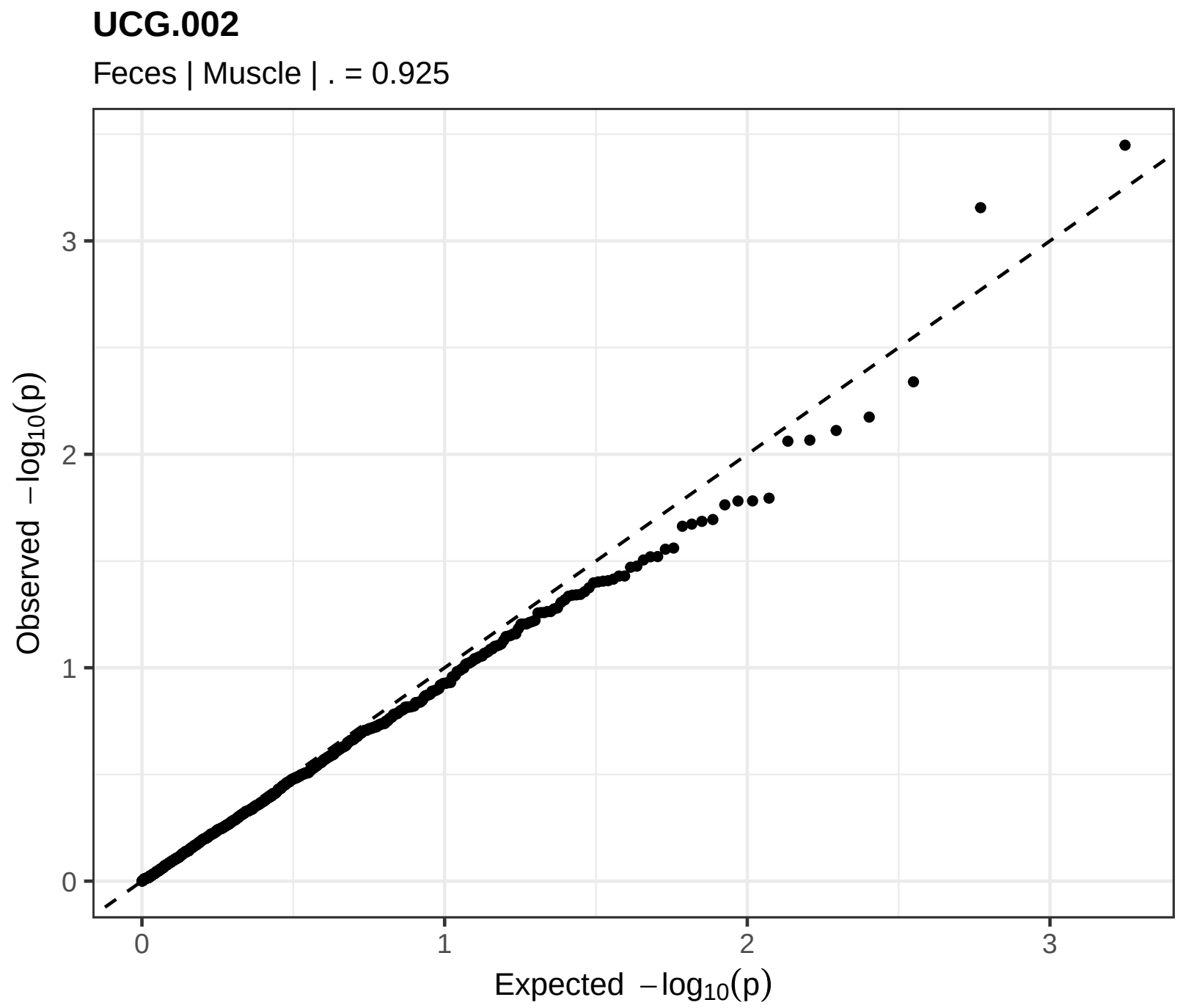
UCG.002
Feces | Muscle | . = 0.925
Observed −log10(p)
Expected −log10(p)

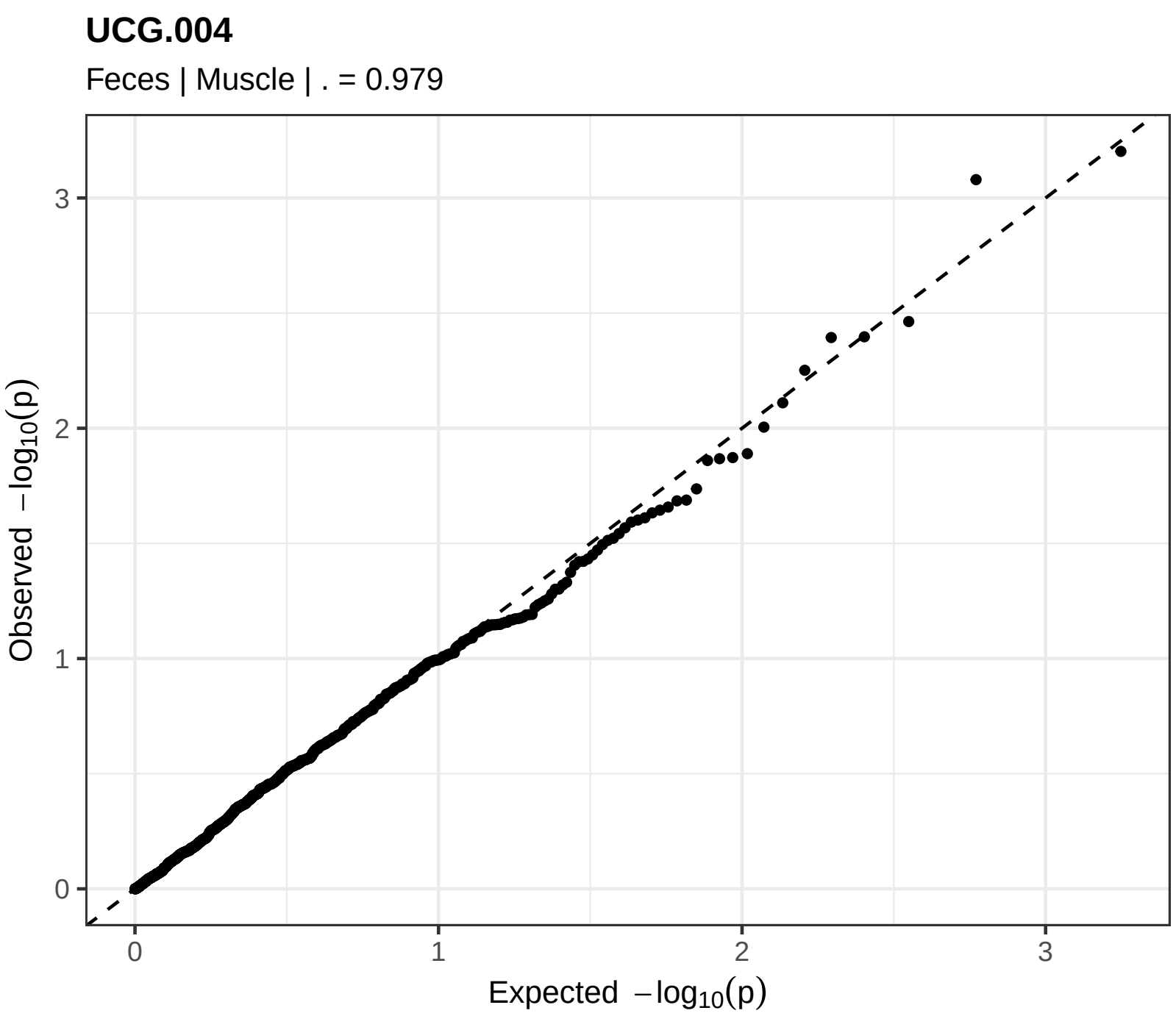
UCG.004
Feces | Muscle | . = 0.979
Observed −log10(p)
Expected −log10(p)

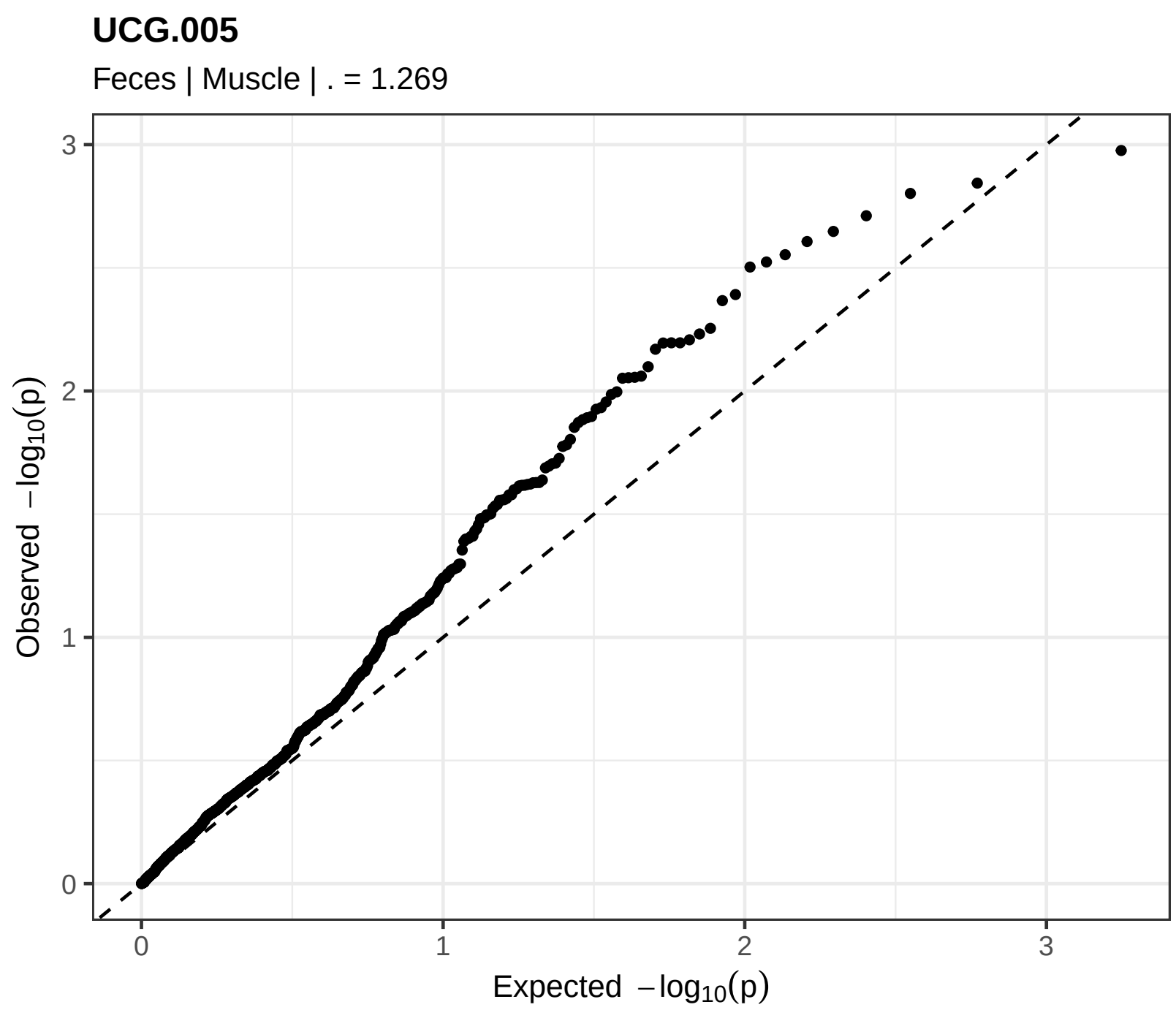
UCG.005
Feces | Muscle | . = 1.269
Observed −log10(p)
Expected −log10(p)

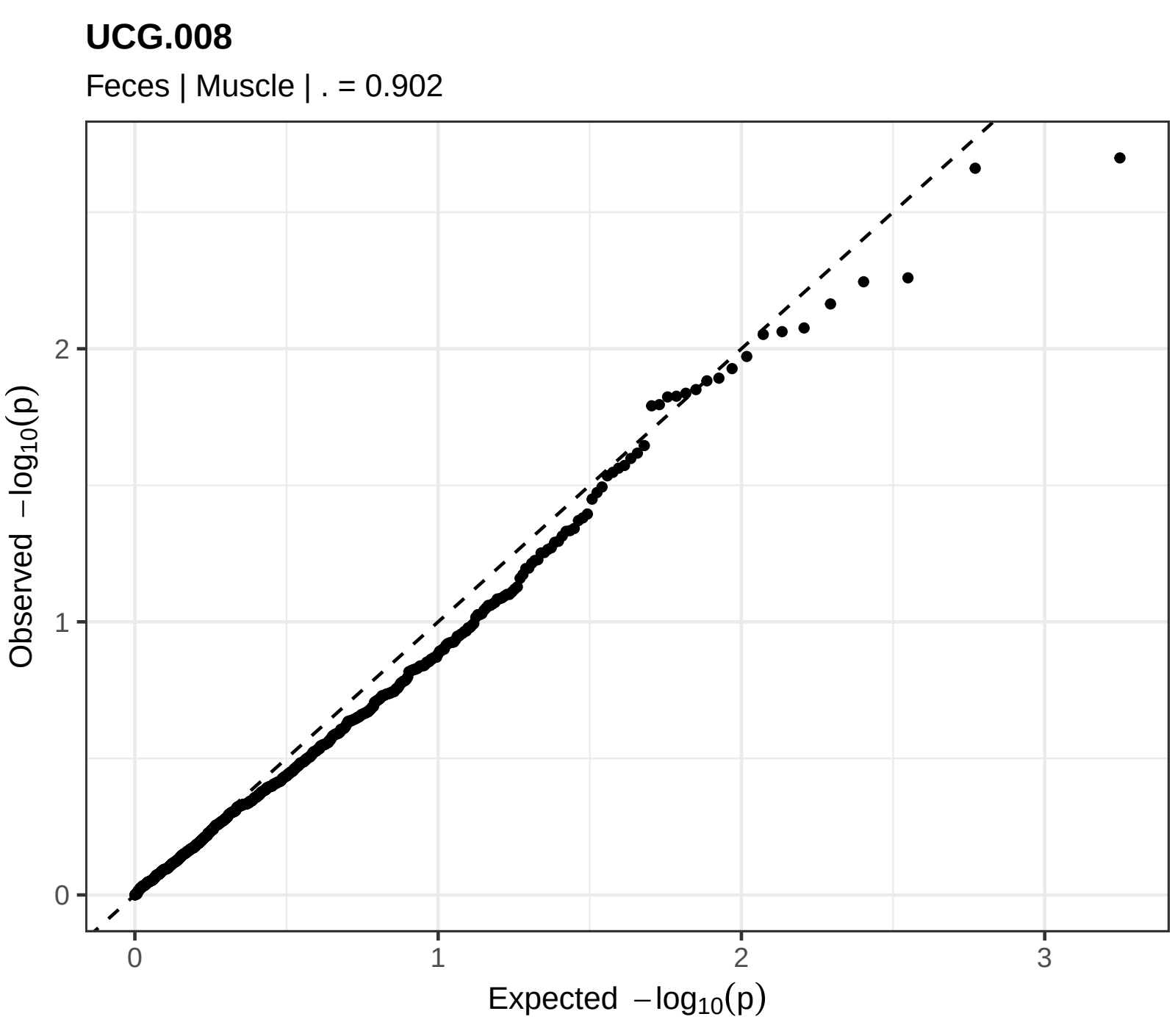
UCG.008
Feces | Muscle | . = 0.902
Observed −log10(p)
Expected −log10(p)

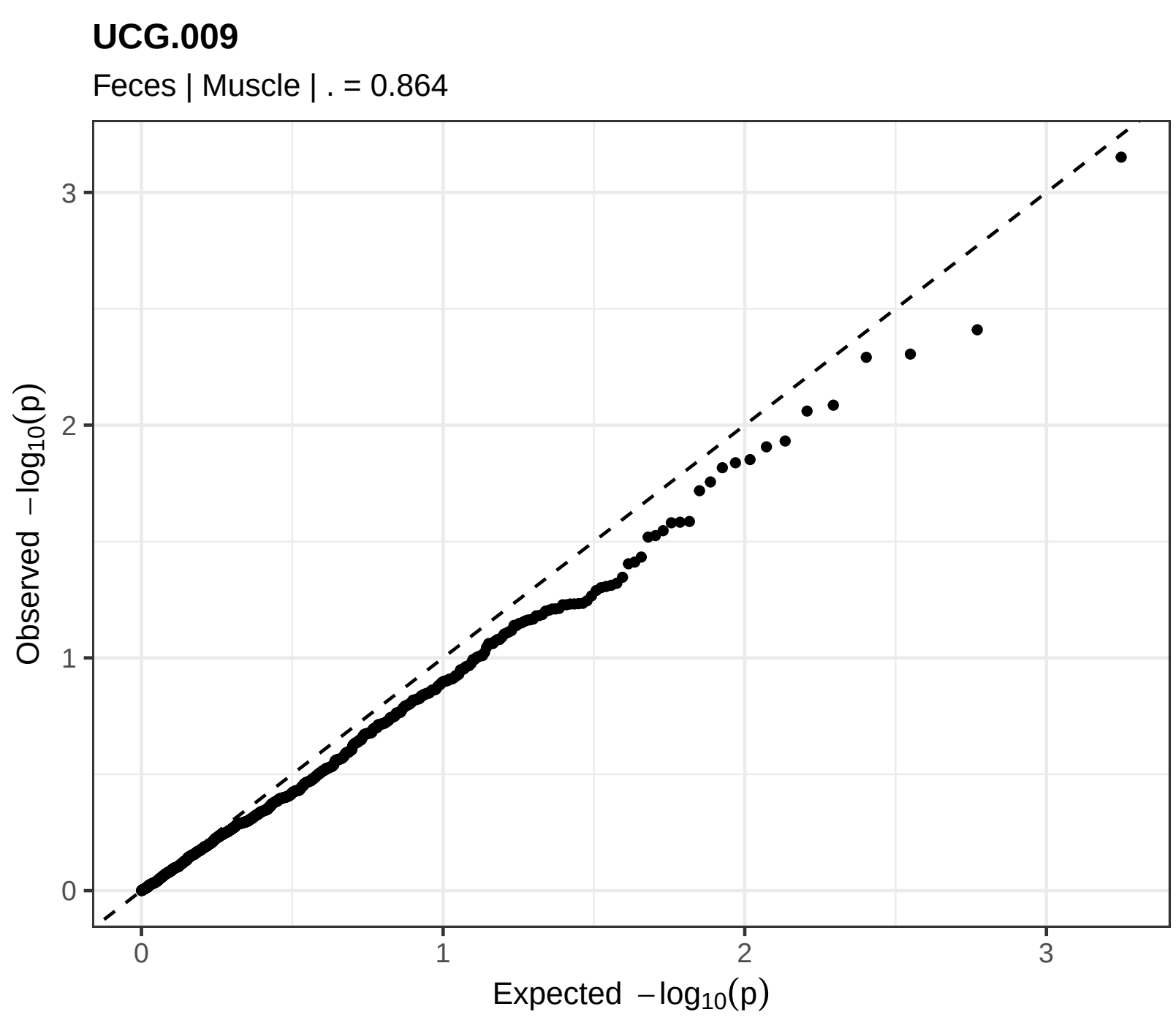
UCG.009
Feces | Muscle | . = 0.864
Observed −log10(p)
Expected −log10(p)

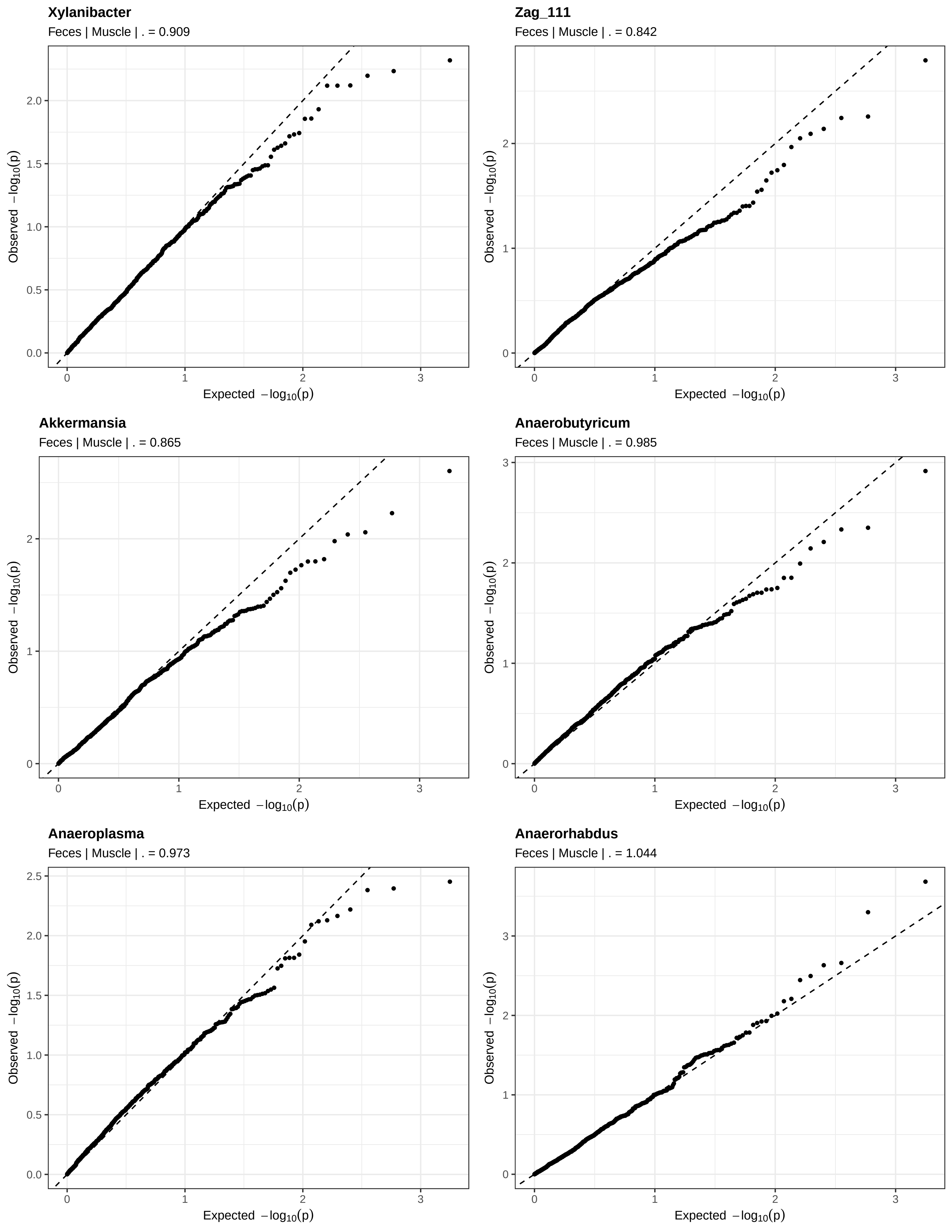
Xylanibacter
Feces | Muscle | . = 0.909
Zag_111
Feces | Muscle | . = 0.842
Akkermansia
Feces | Muscle | . = 0.865
Anaerobutyricum
Feces | Muscle | . = 0.985
Anaeroplasma
Feces | Muscle | . = 0.973
Anaerorhabdus
Feces | Muscle | . = 1.044
Observed $-\log_{10}(p)$
Expected $-\log_{10}(p)$

Anaerostipes

Feces | Muscle | . = 0.946

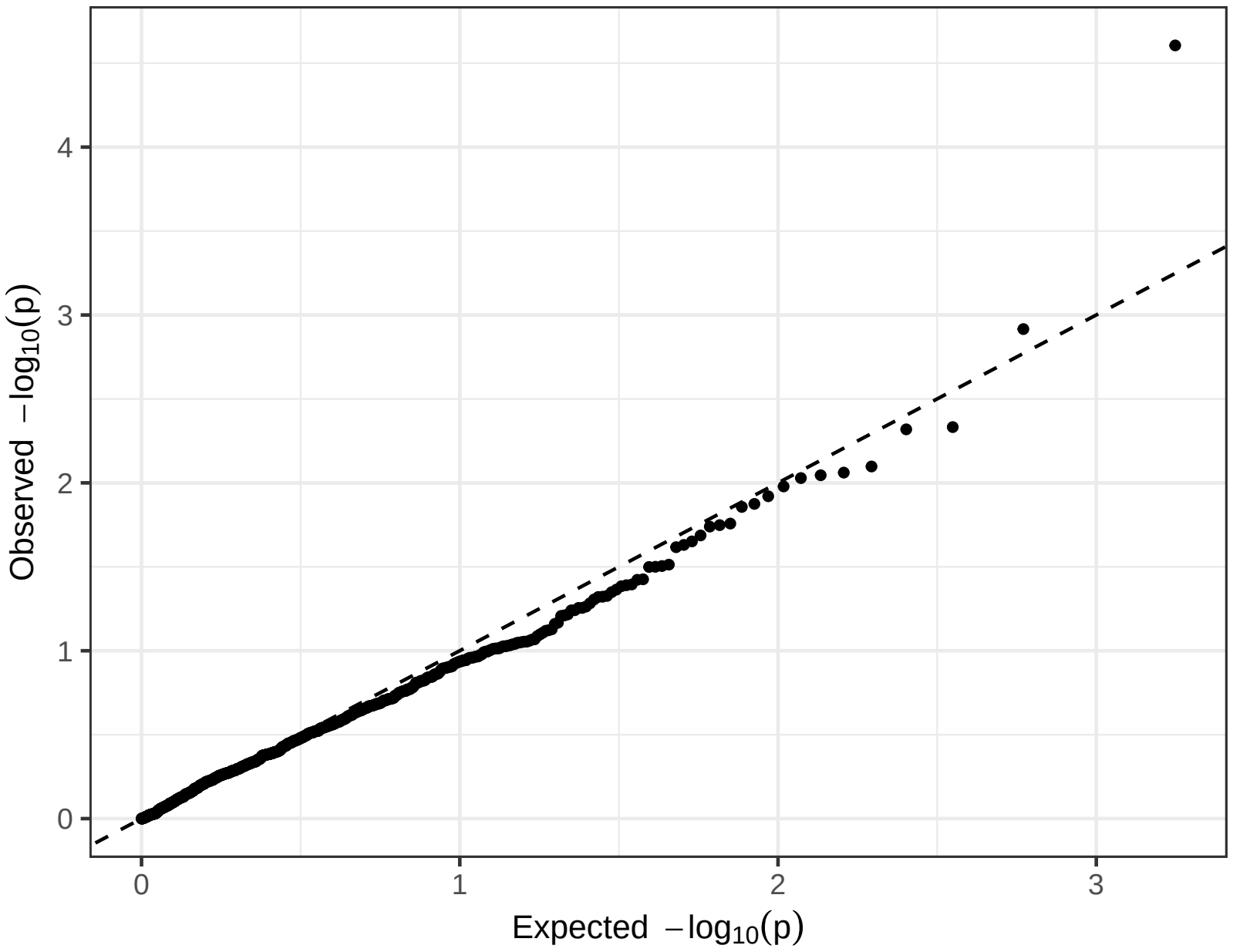

Anaerovibrio

Feces | Muscle | . = 1.001

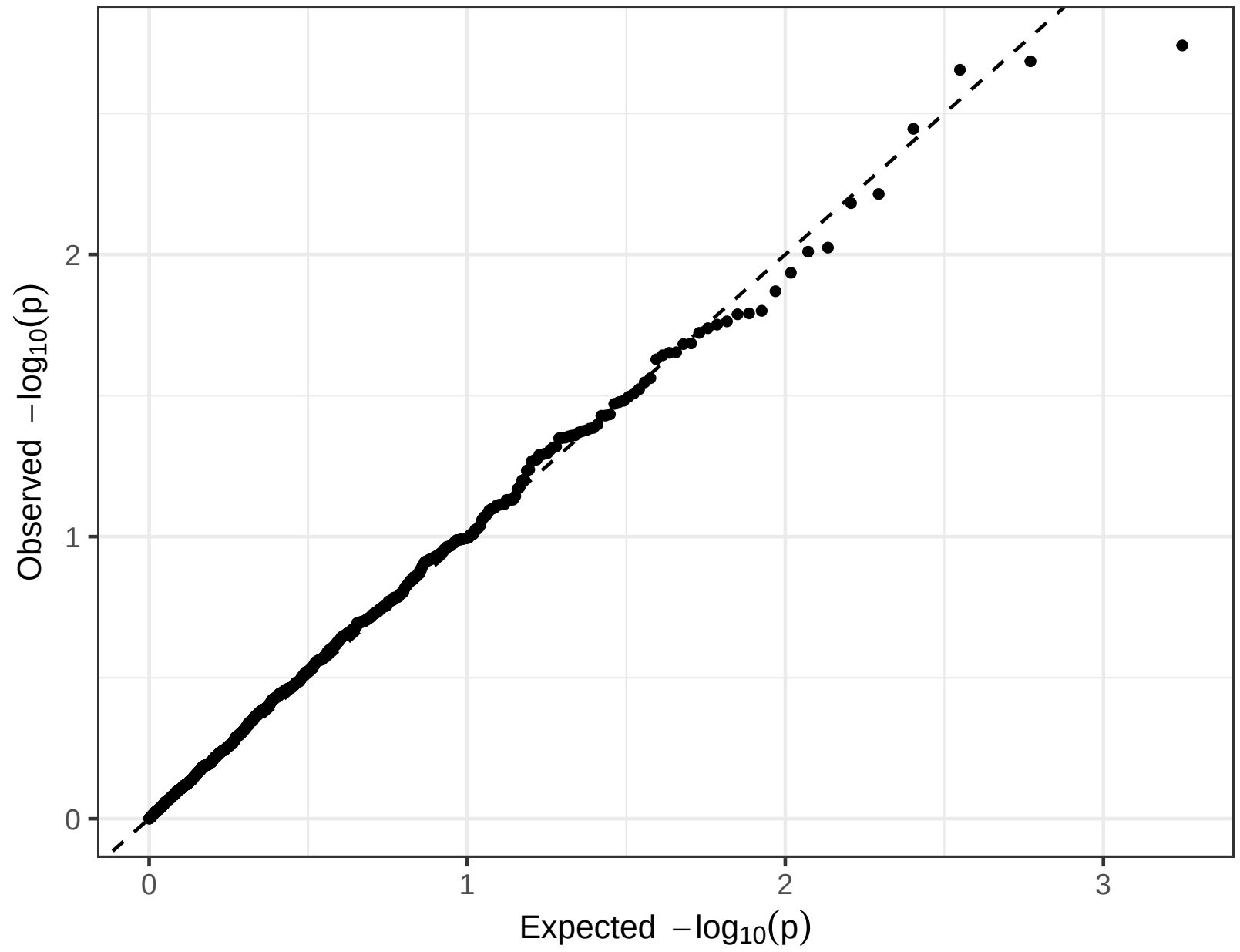

Eubacterium_eligens_group

Feces | Muscle | . = 0.974

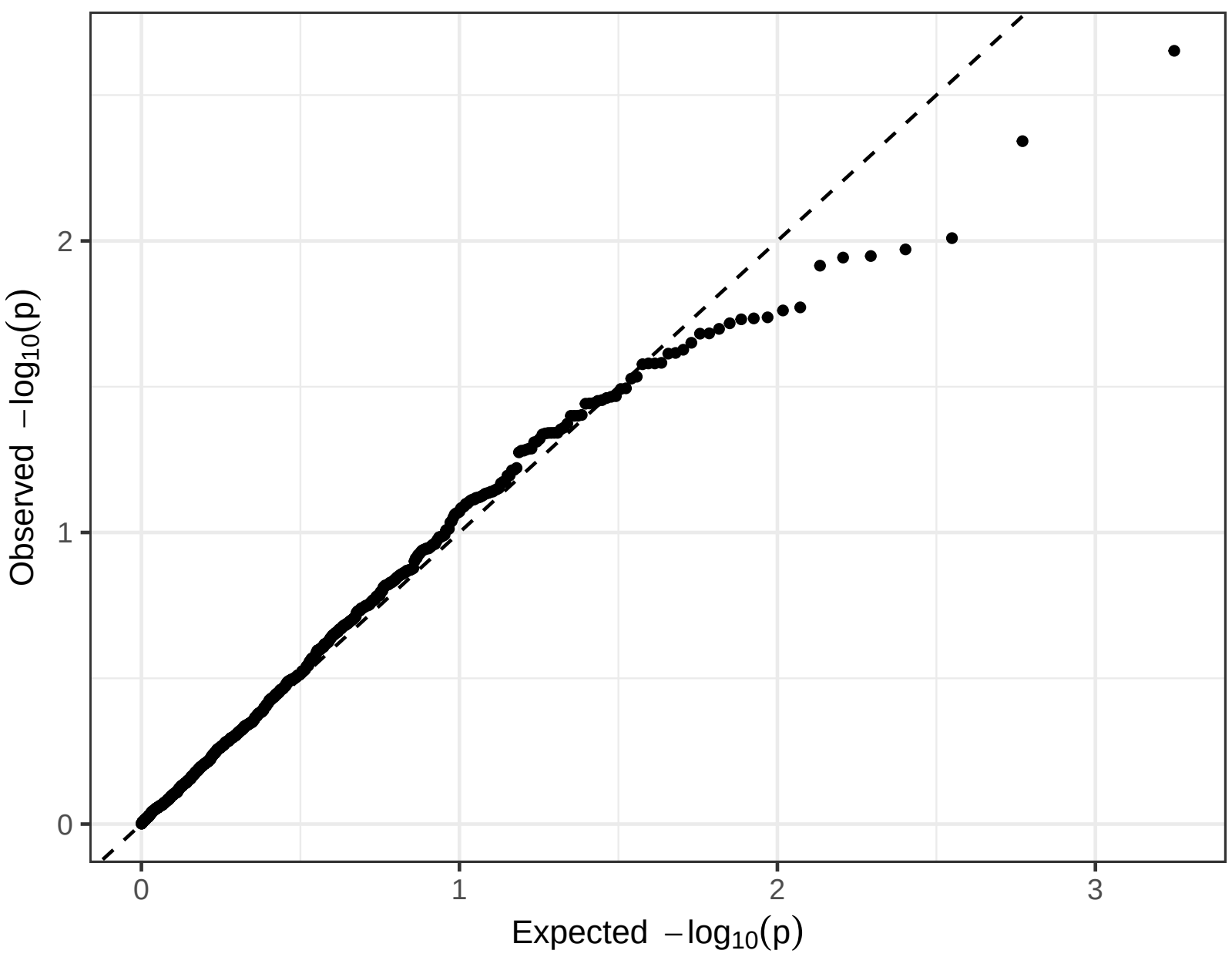

Bacteroides

Feces | Muscle | . = 0.854

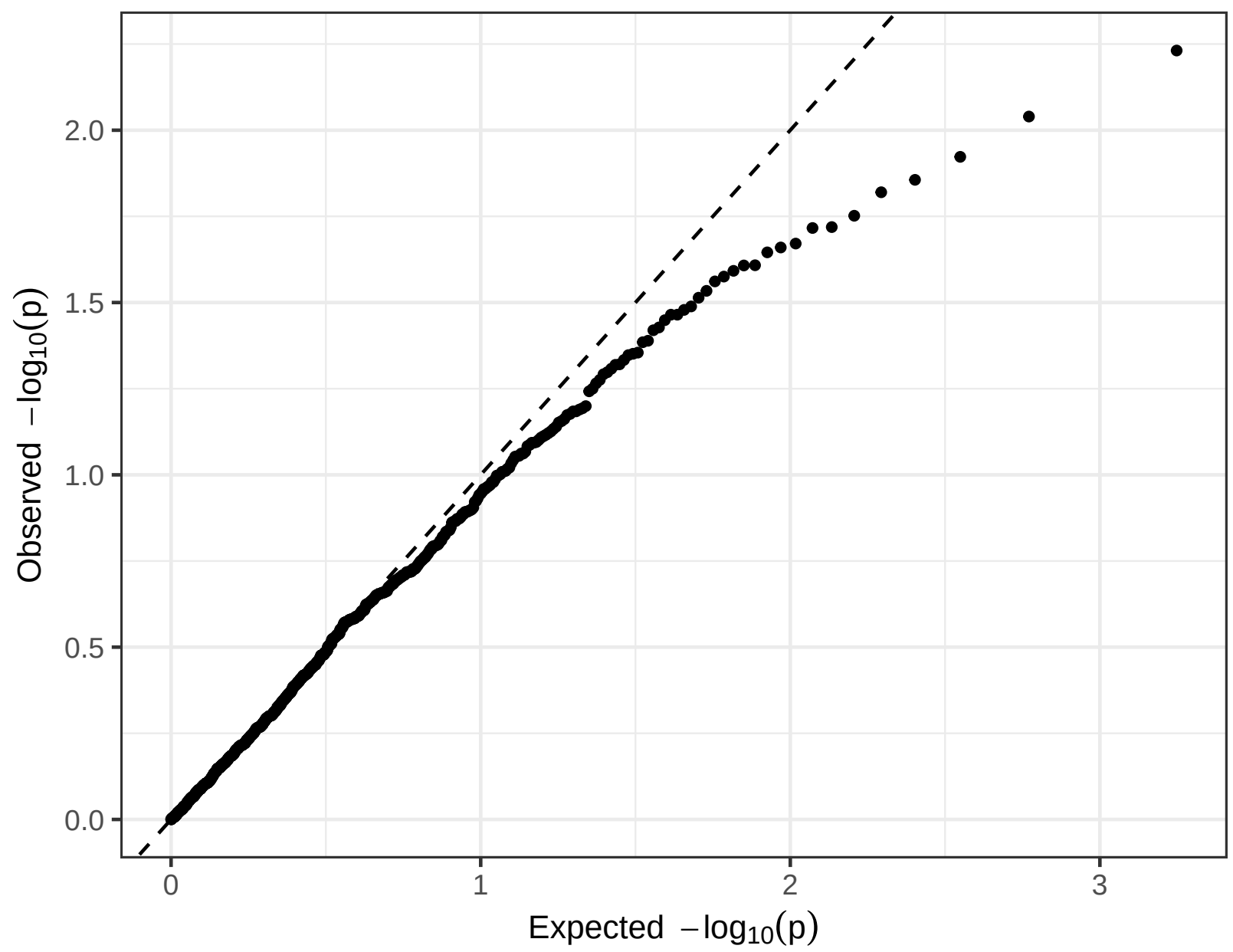

Blautia

Feces | Muscle | . = 1.022

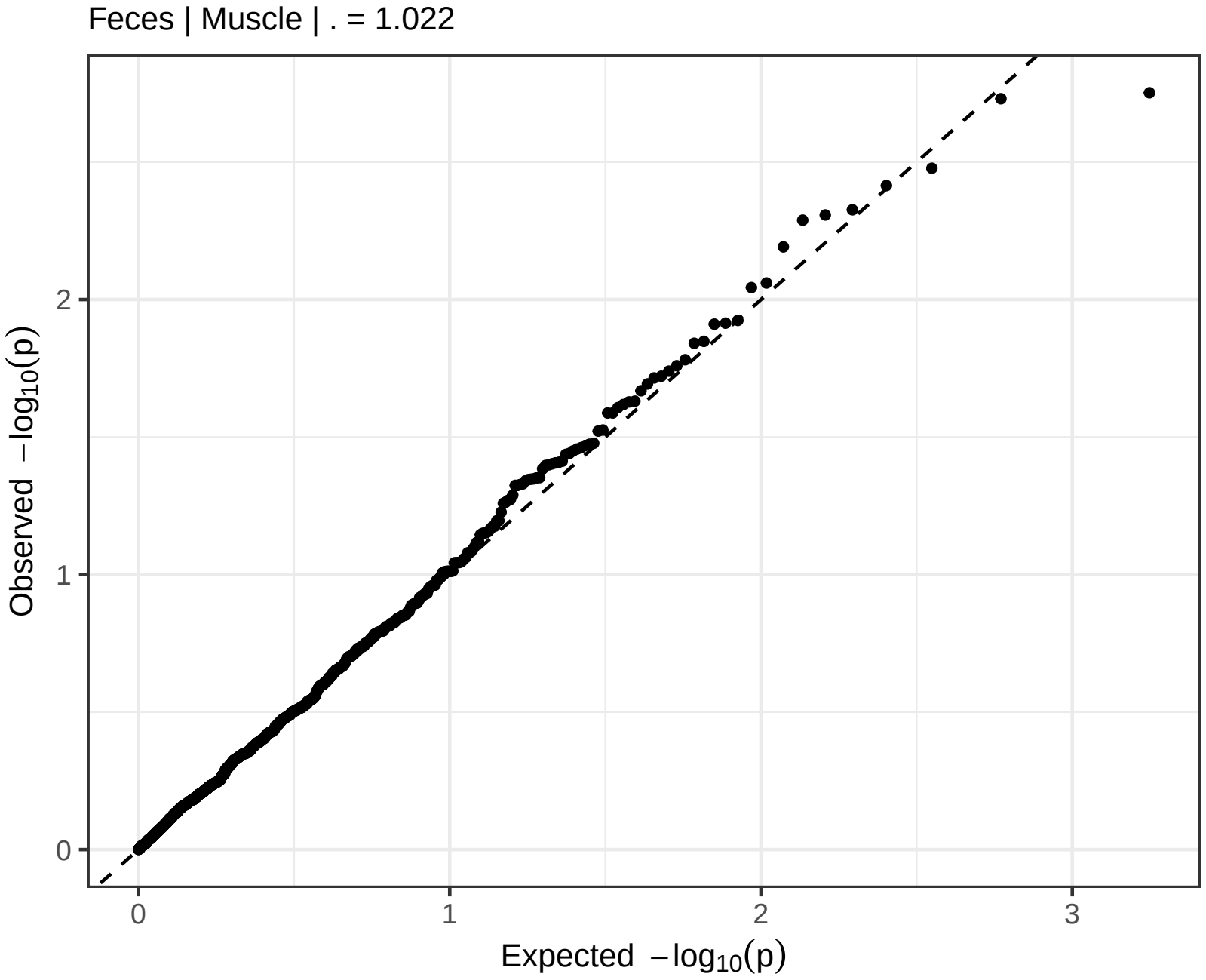

Butyribacter

Feces | Muscle | . = 0.909

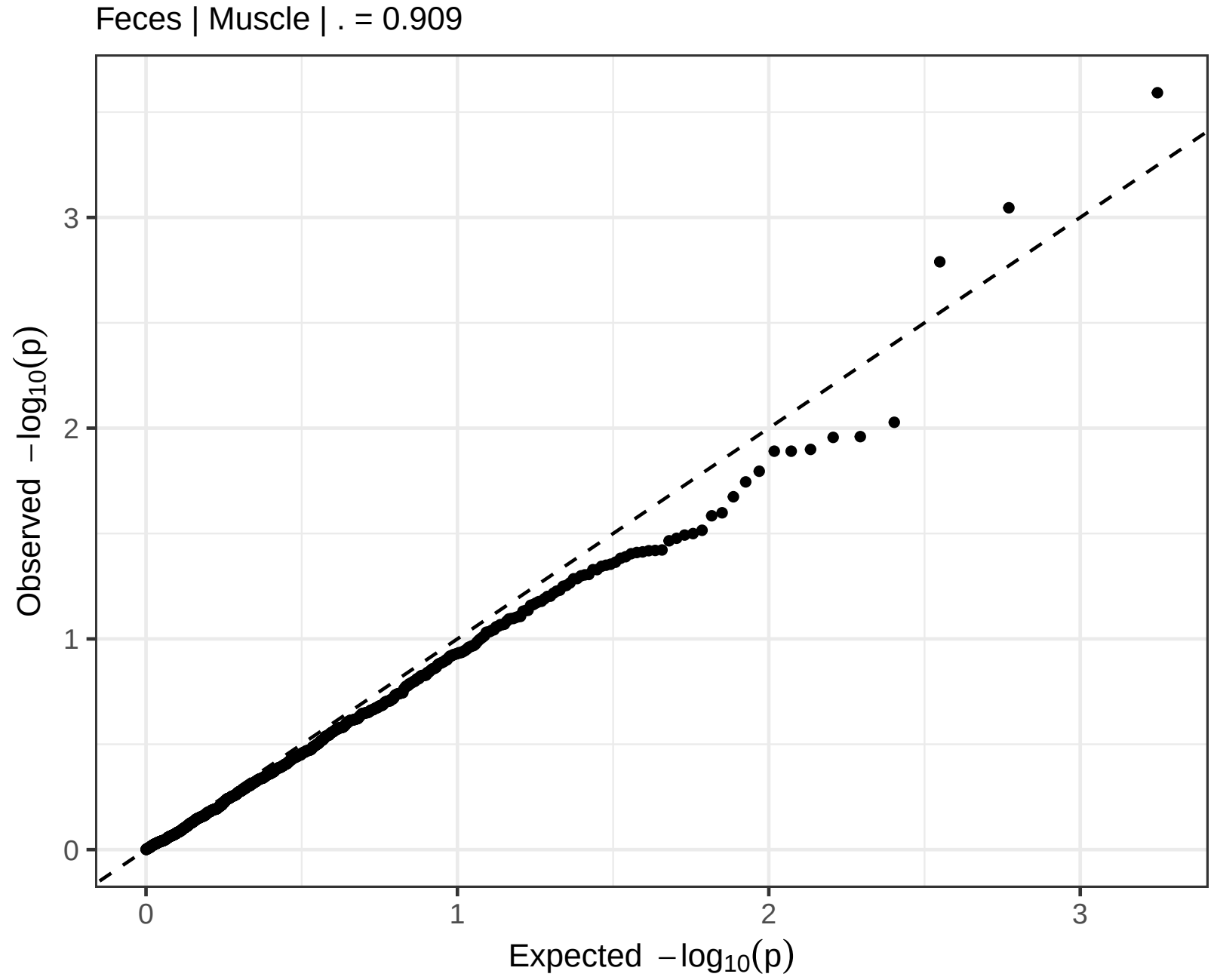

Butyrivibrio

Feces | Muscle | . = 0.833

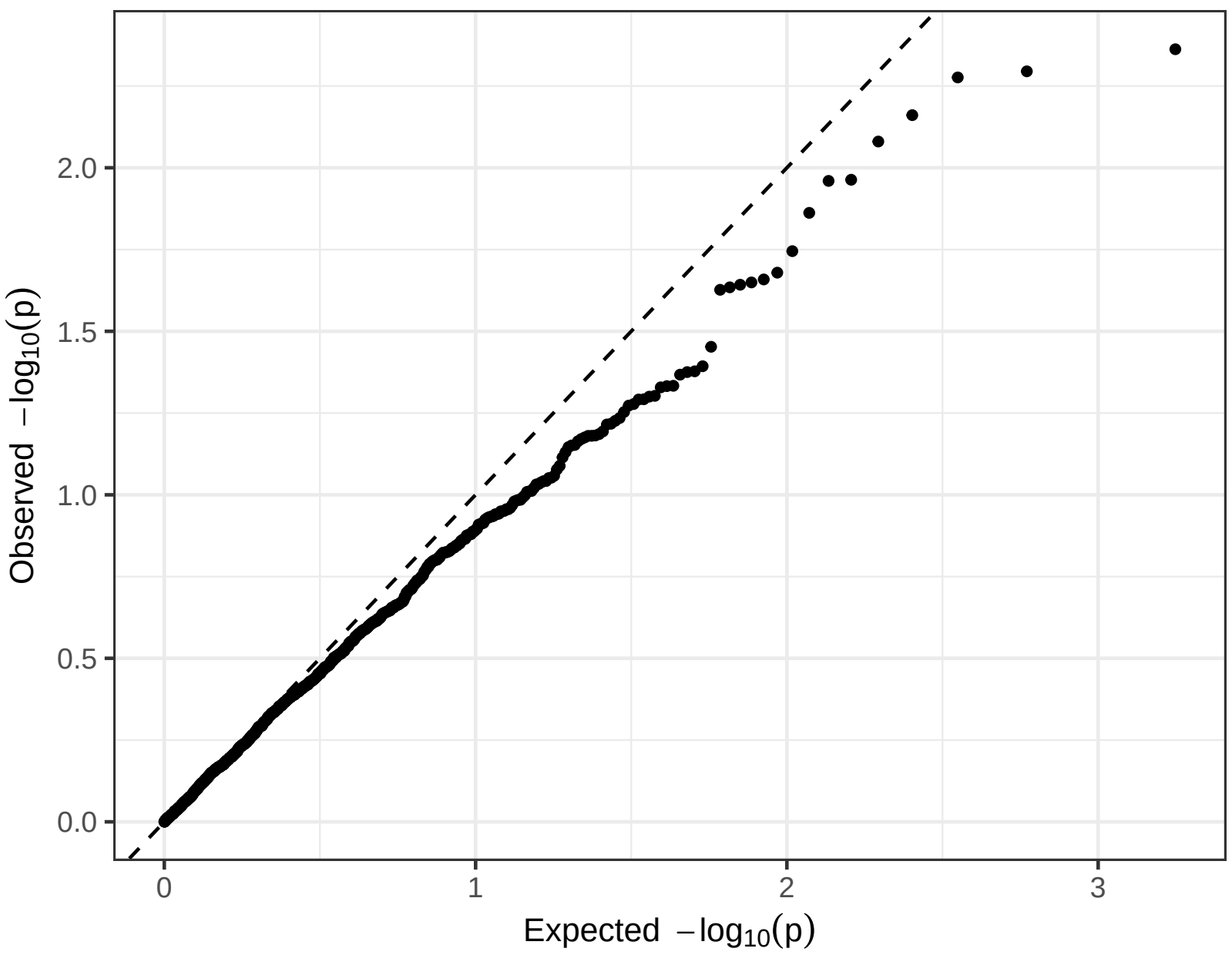

CAG.196

Feces | Muscle | . = 0.781

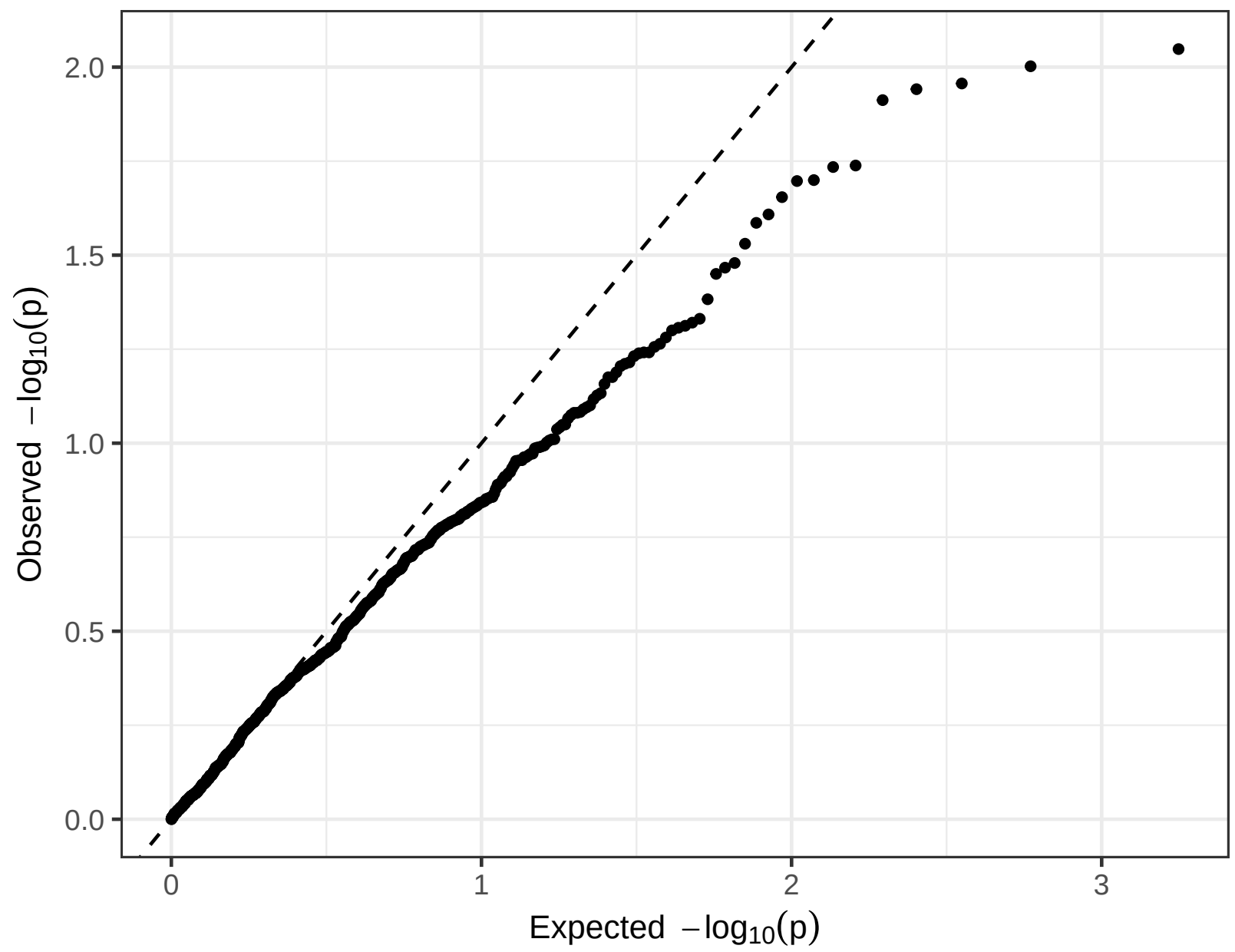

CAG.873

Feces | Muscle | . = 0.907

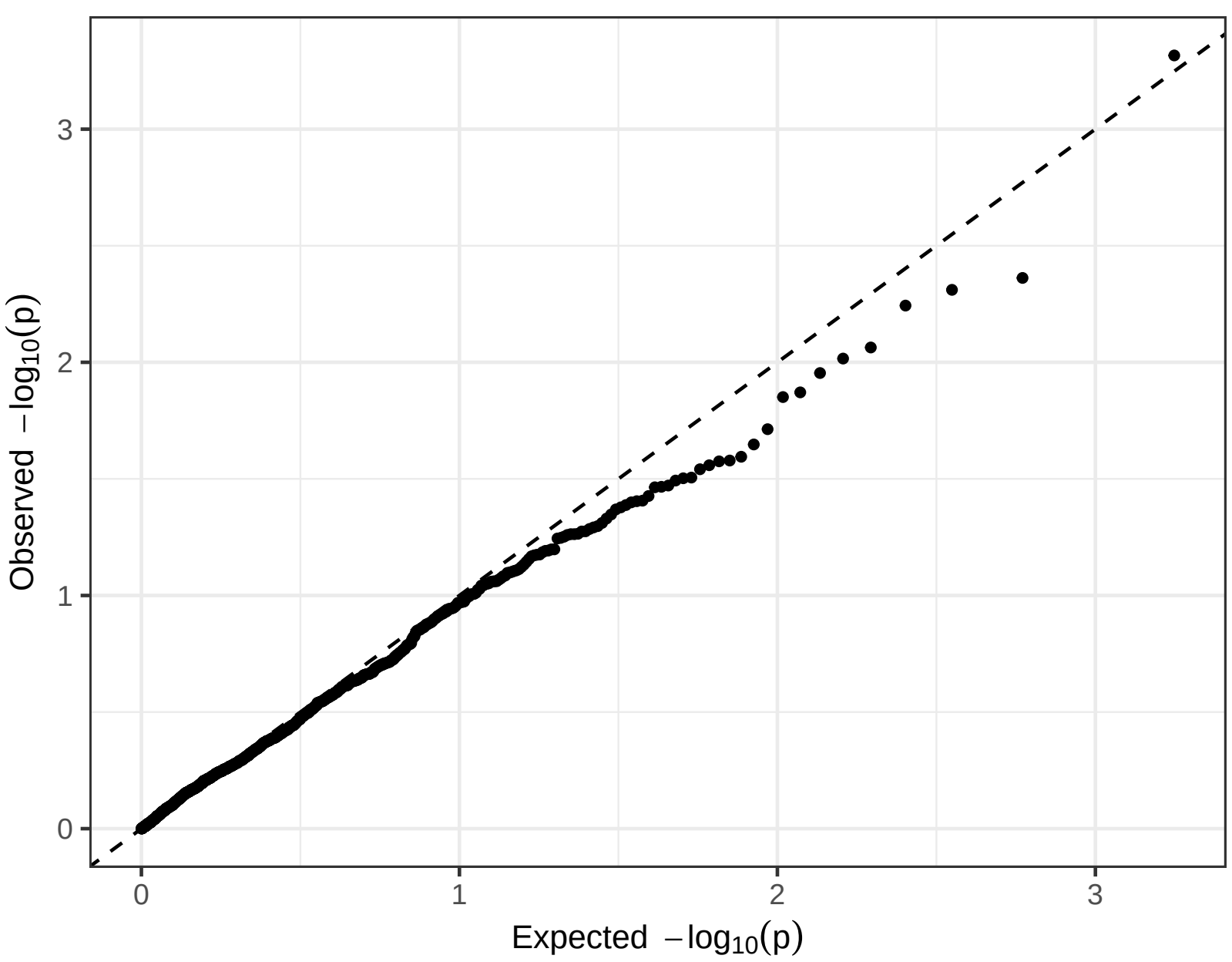

Campylobacter

Feces | Muscle | . = 0.918

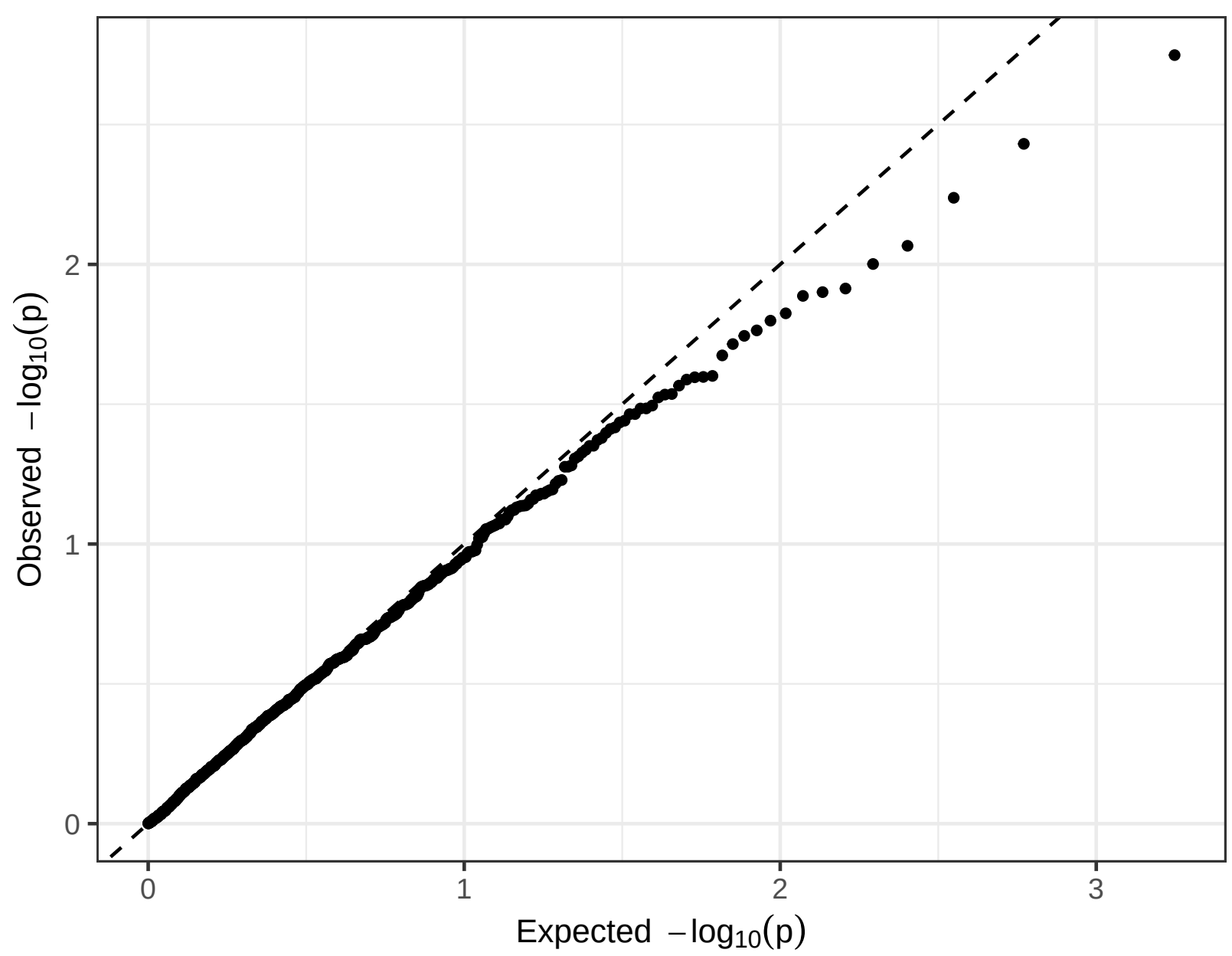

Candidatus_Methanomethylophilus

Feces | Muscle | . = 1.054

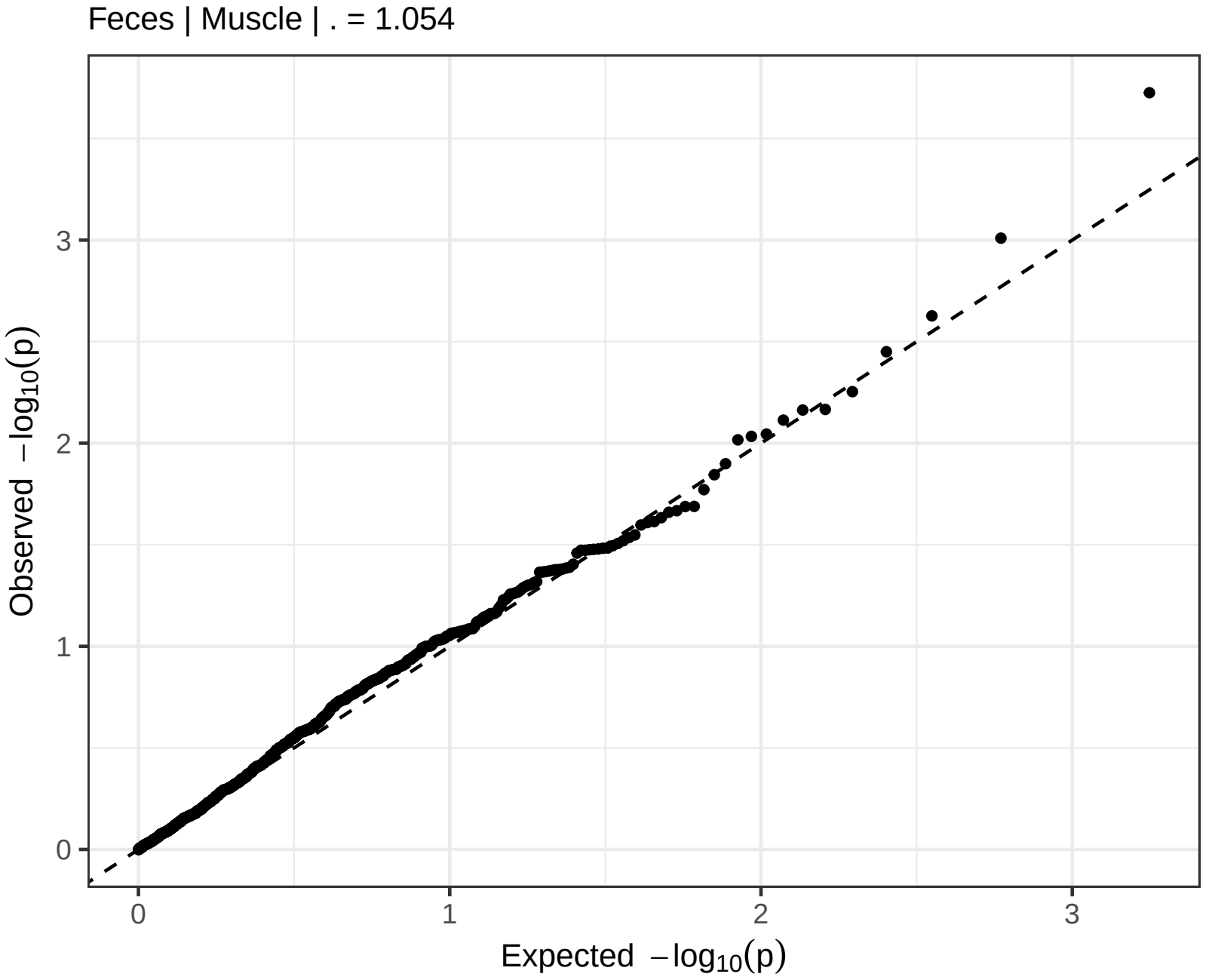

Candidatus_Methanoplasma

Feces | Muscle | . = 1.012

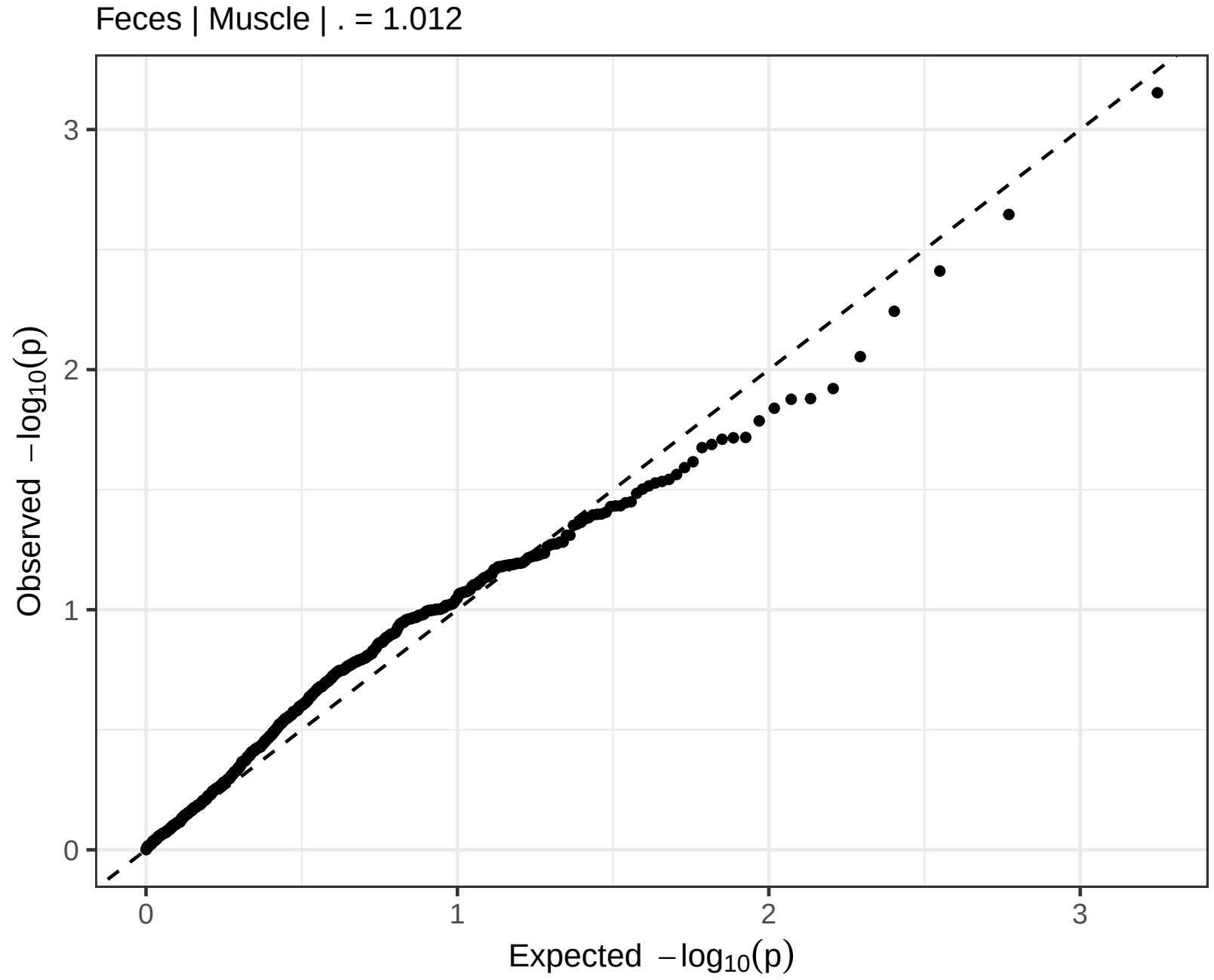

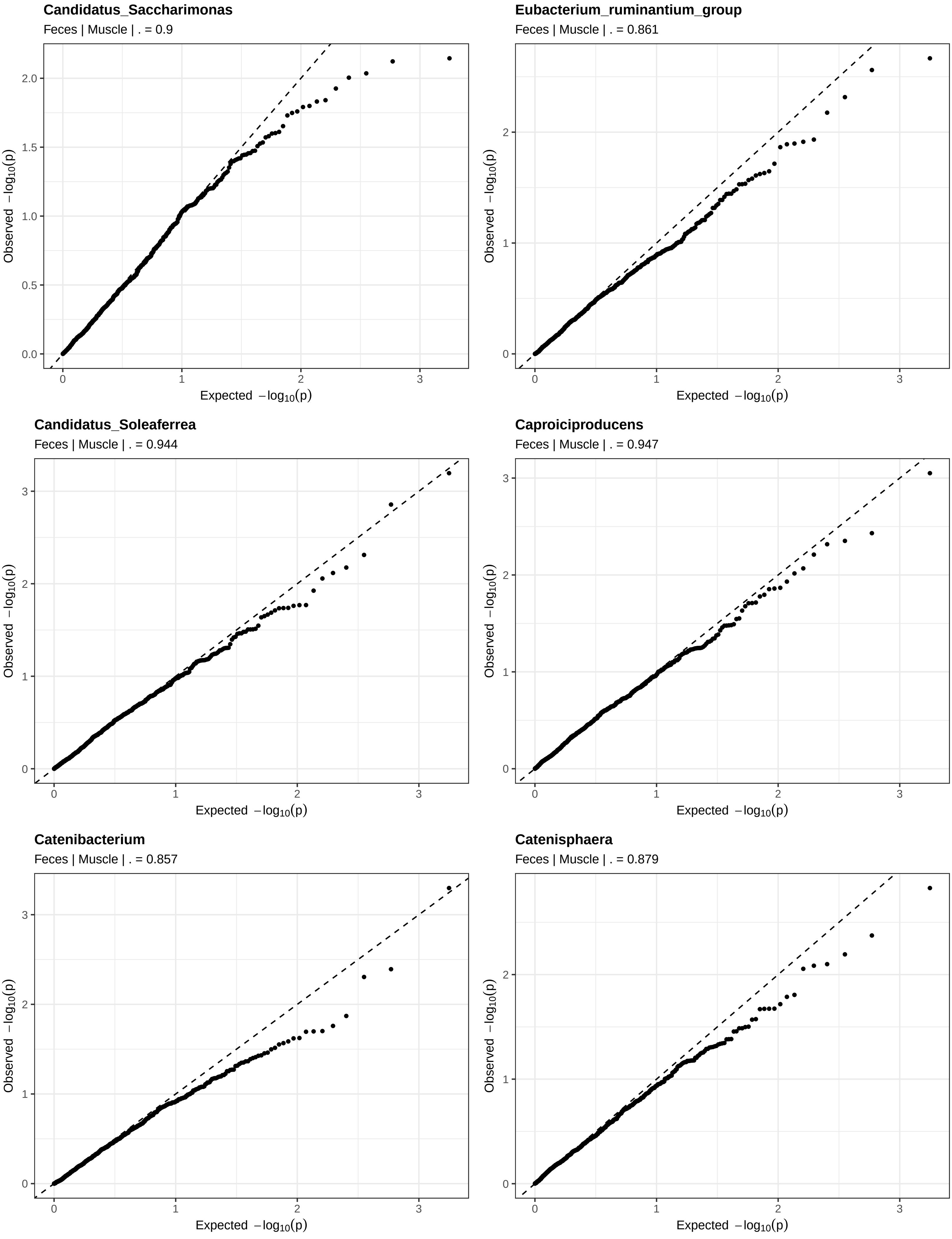
Candidatus_Saccharimonas
Feces | Muscle | . = 0.9
Eubacterium_ruminantium_group
Feces | Muscle | . = 0.861
Candidatus_Soleaferrea
Feces | Muscle | . = 0.944
Caproiciproducens
Feces | Muscle | . = 0.947
Catenibacterium
Feces | Muscle | . = 0.857
Catenisphaera
Feces | Muscle | . = 0.879
Observed −log10(p)
Expected −log10(p)

Cellulosilyticum

Feces | Muscle | . = 0.843

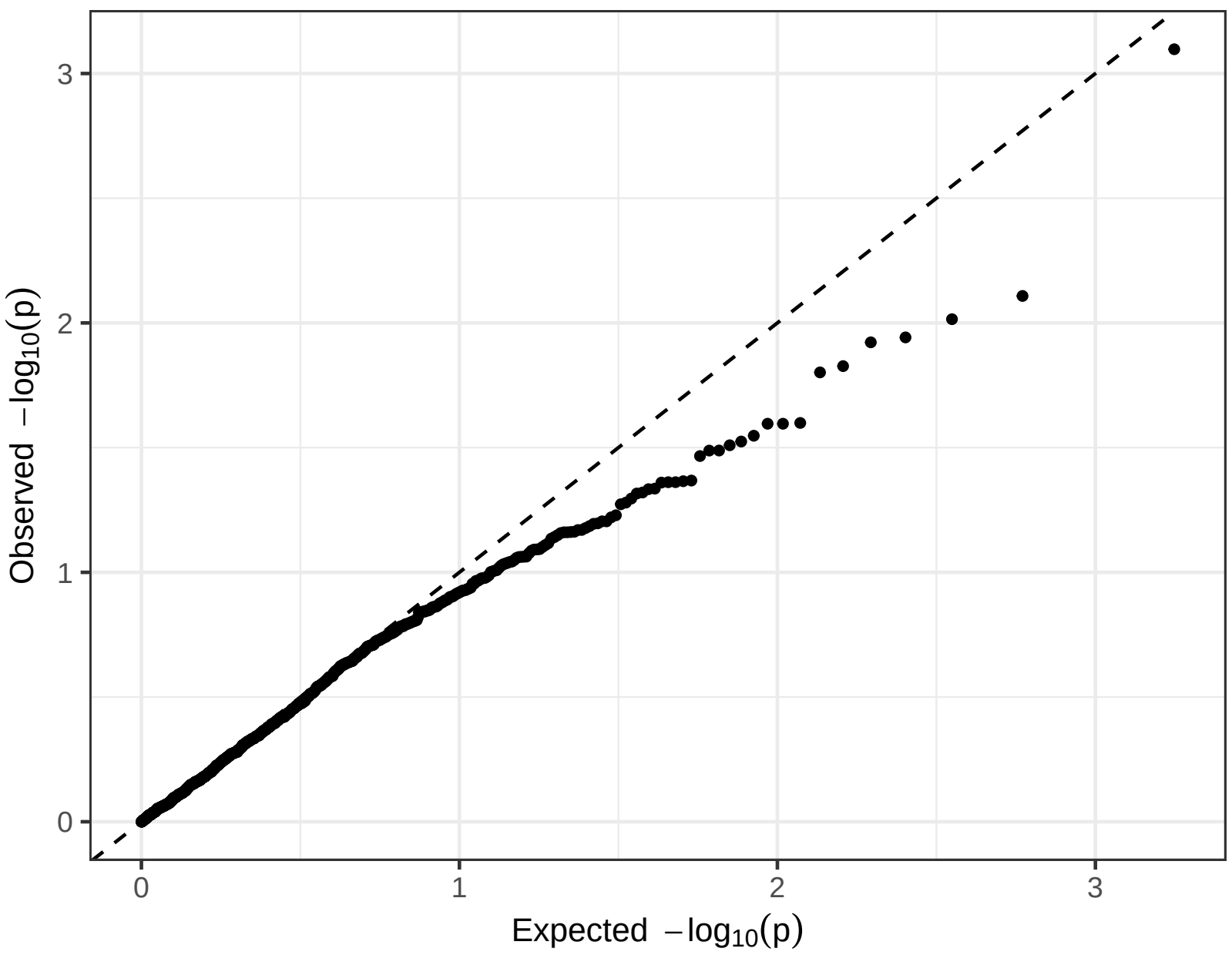

Christensenellaceae_R.7_group

Feces | Muscle | . = 0.953

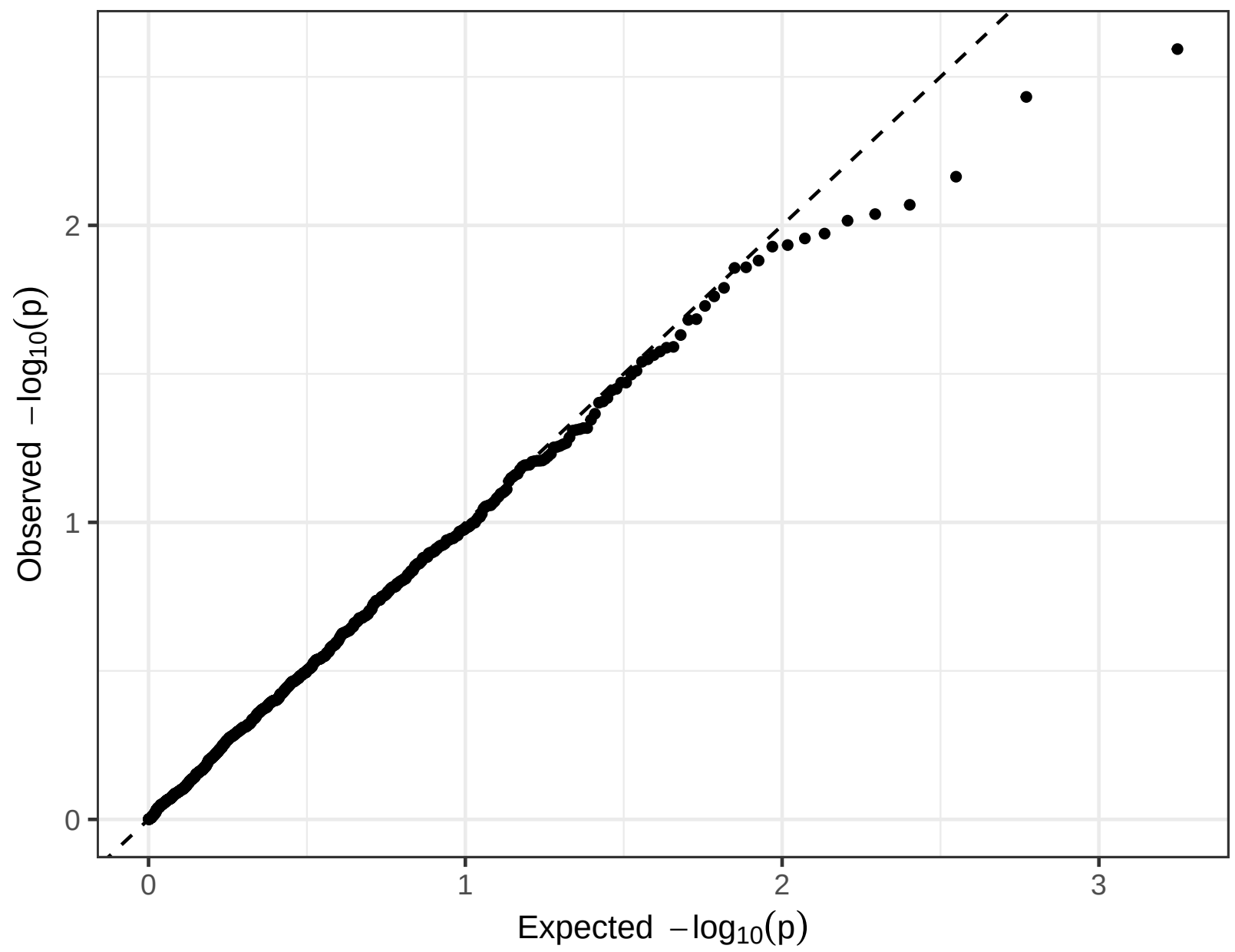

Clostridium

Feces | Muscle | . = 1.017

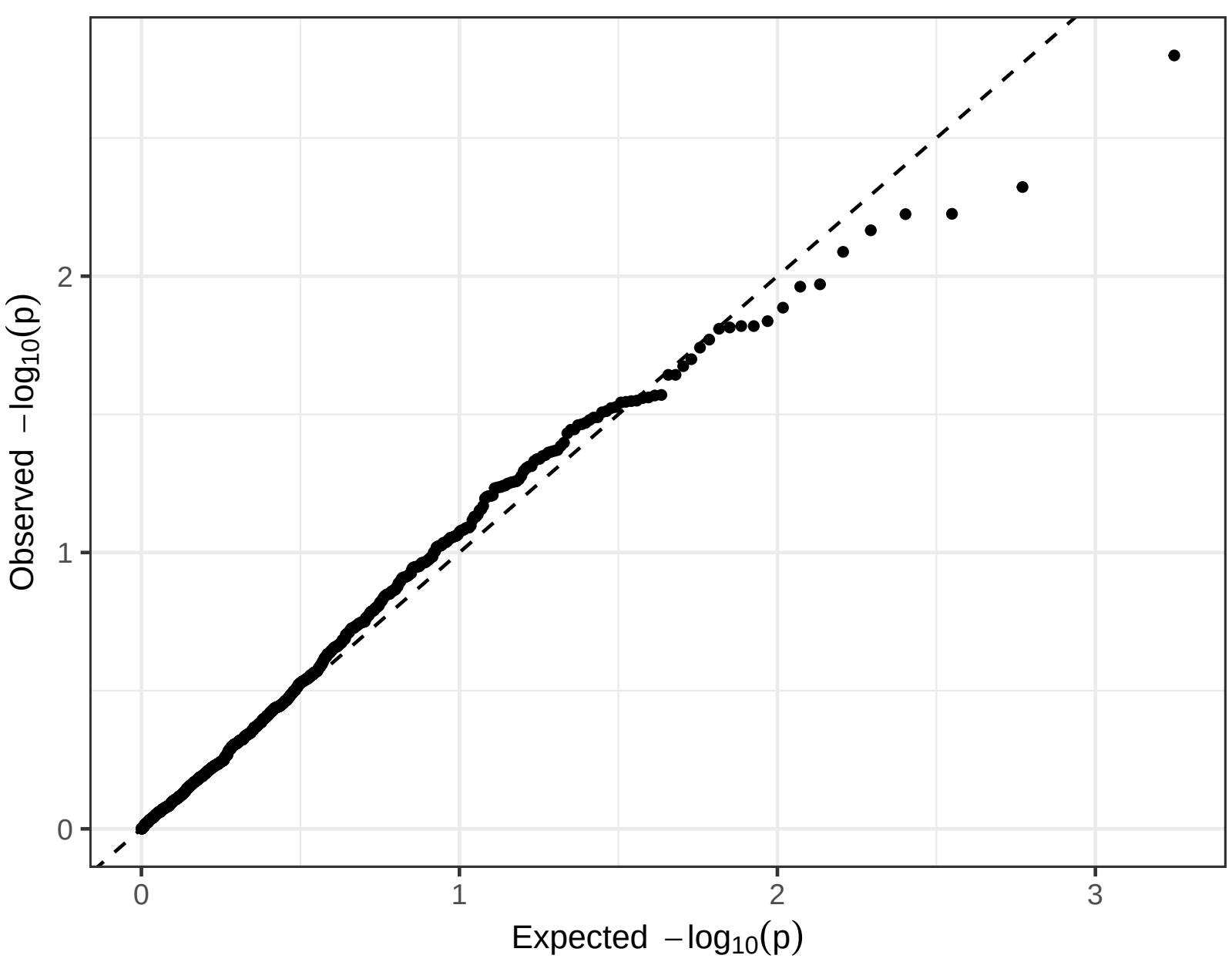

Colidextribacter

Feces | Muscle | . = 1.037

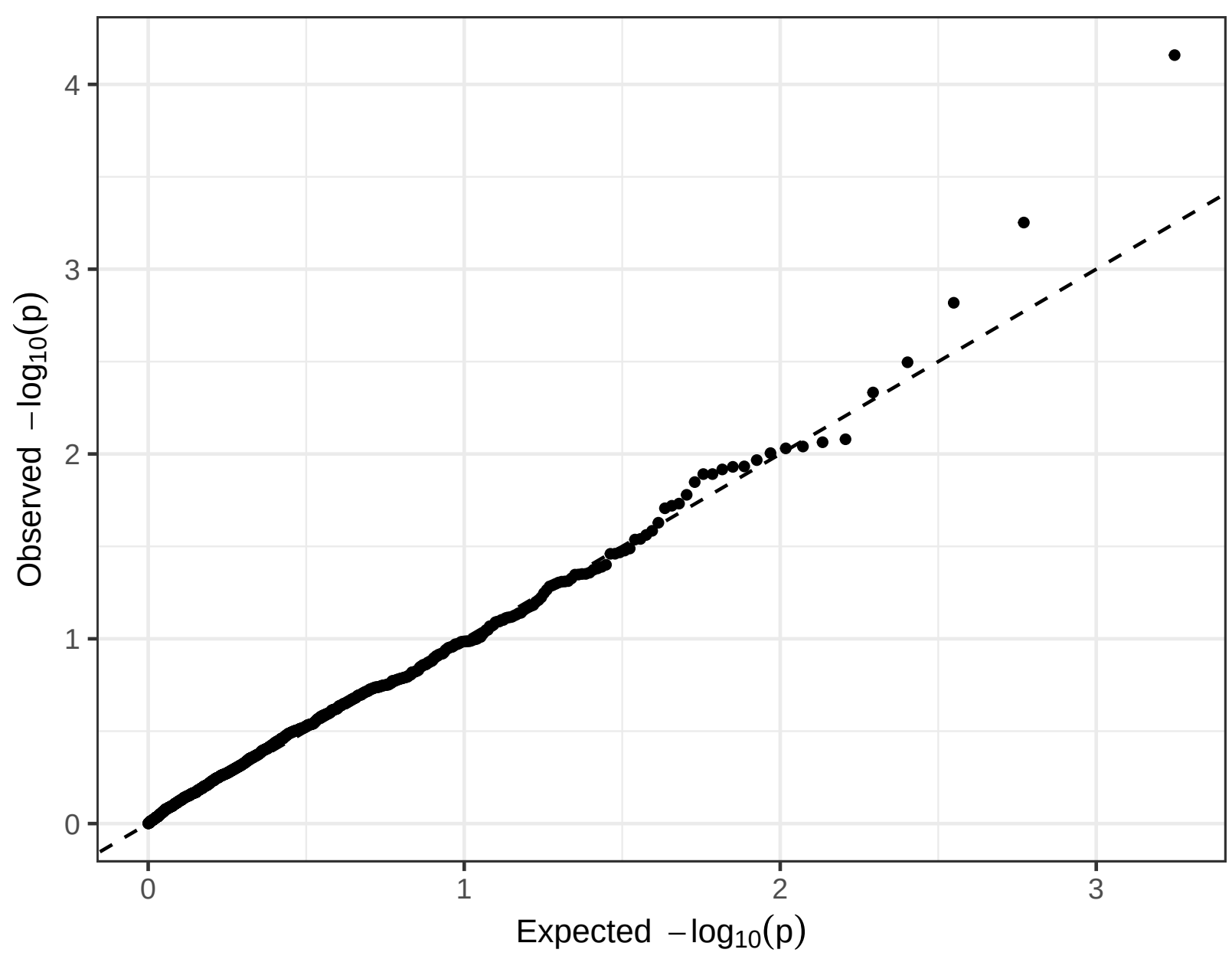

Collinsella

Feces | Muscle | . = 0.917

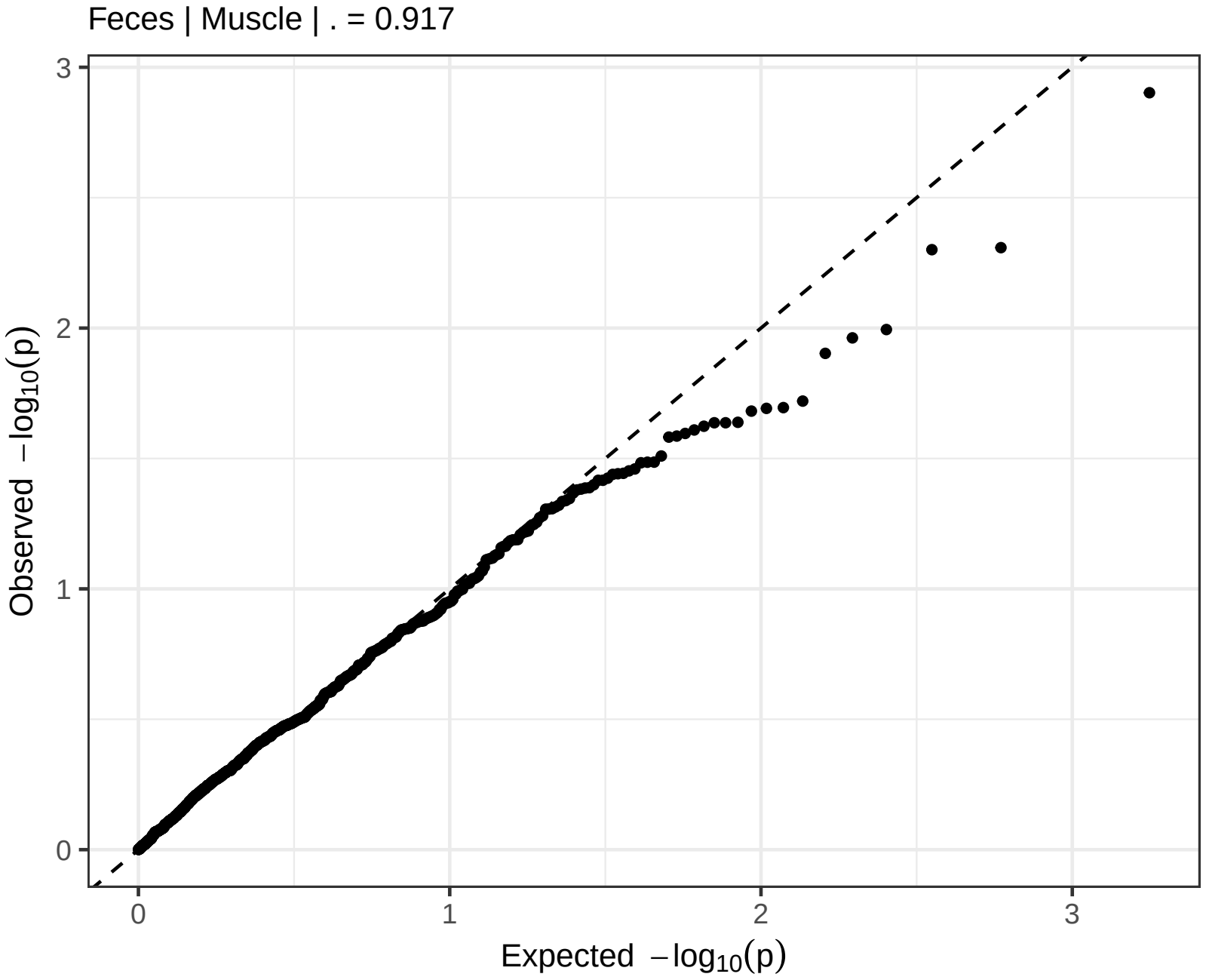

Coprococcus

Feces | Muscle | . = 0.914

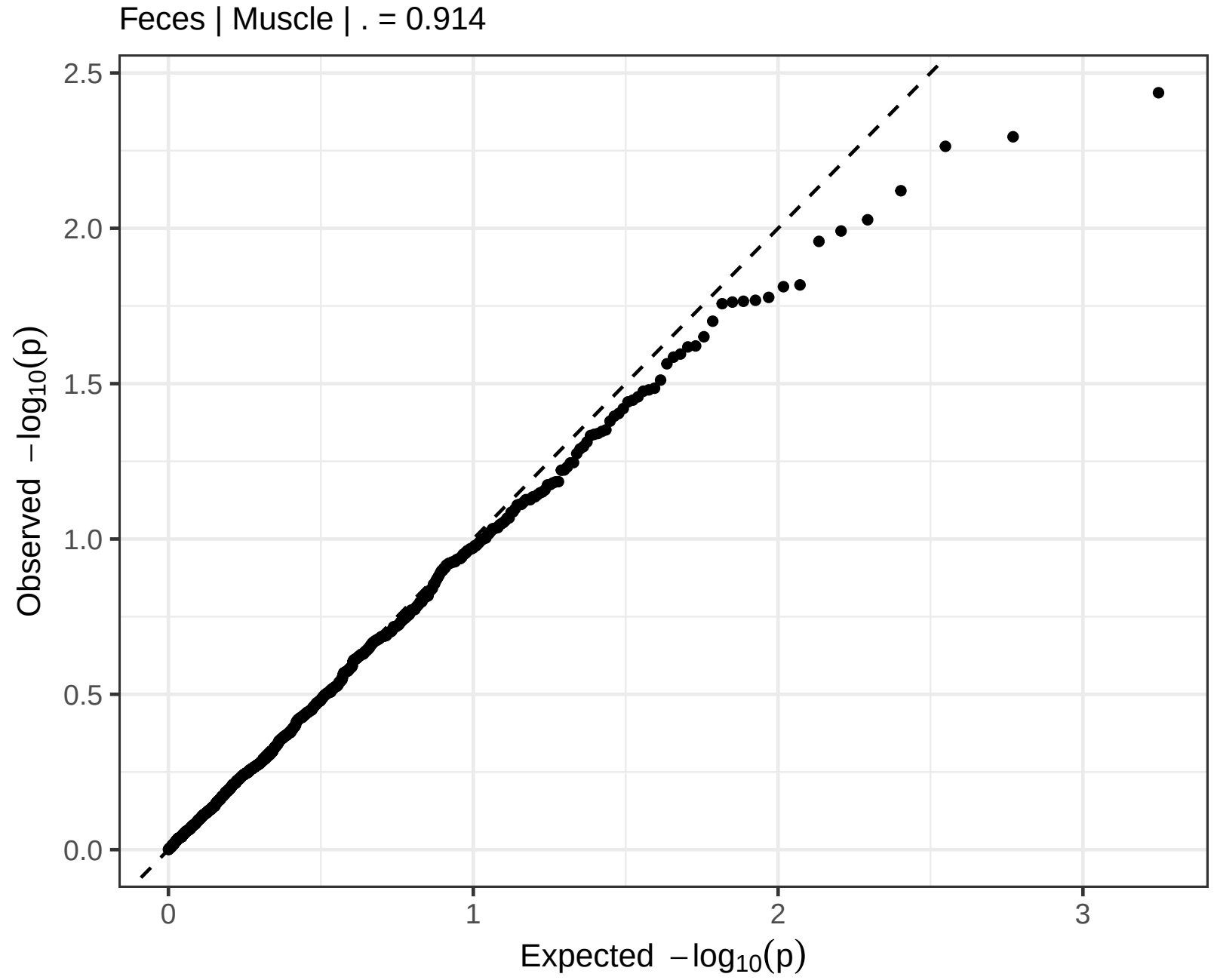

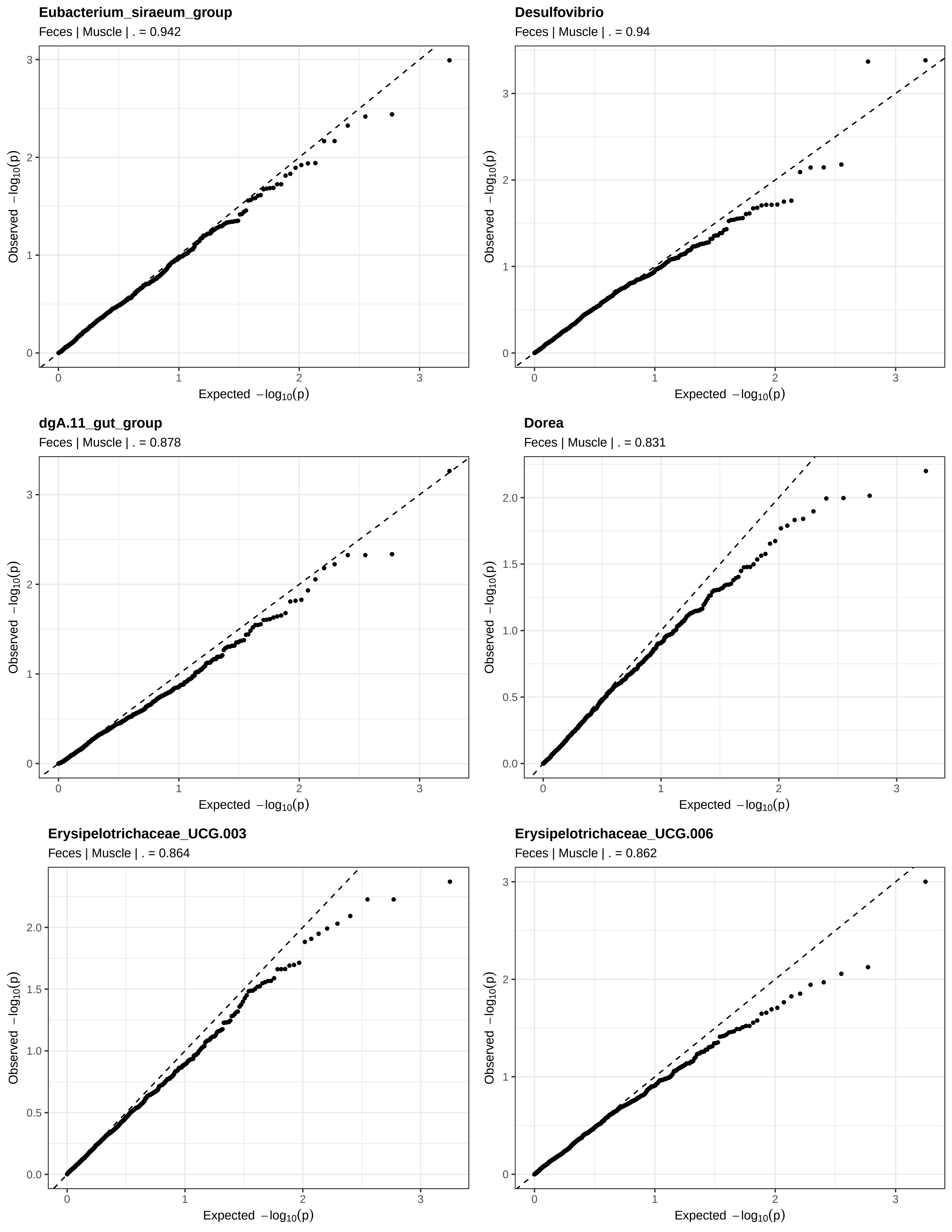
Eubacterium_siraeum_group
Feces | Muscle | . = 0.942
Desulfovibrio
Feces | Muscle | . = 0.94
dgA.11_gut_group
Feces | Muscle | . = 0.878
Dorea
Feces | Muscle | . = 0.831
Erysipelotrichaceae_UCG.003
Feces | Muscle | . = 0.864
Erysipelotrichaceae_UCG.006
Feces | Muscle | . = 0.862
Observed −log10(p)
Expected −log10(p)

Escherichia.Shigella

Feces | Muscle | . = 0.852

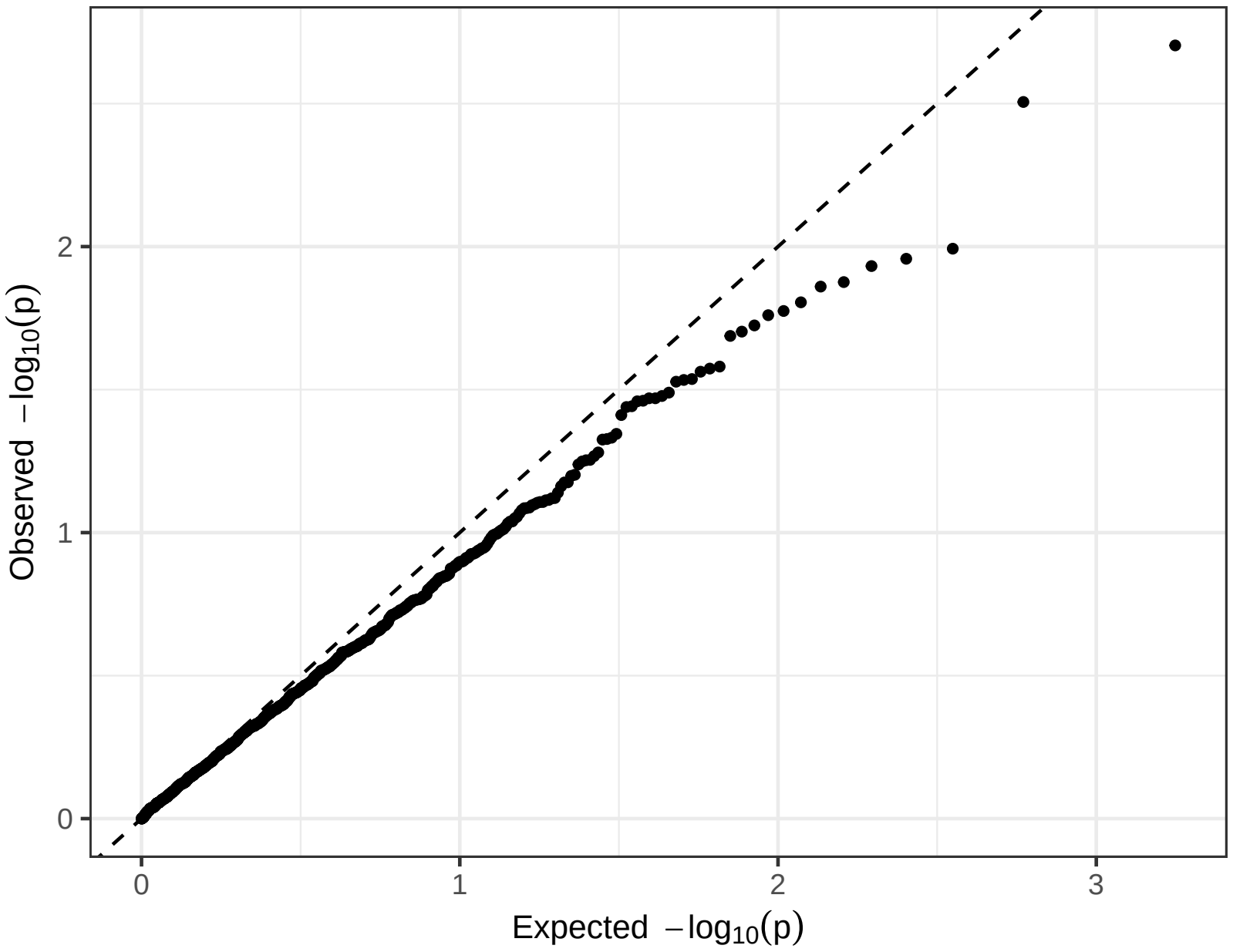

Extibacter

Feces | Muscle | . = 0.84

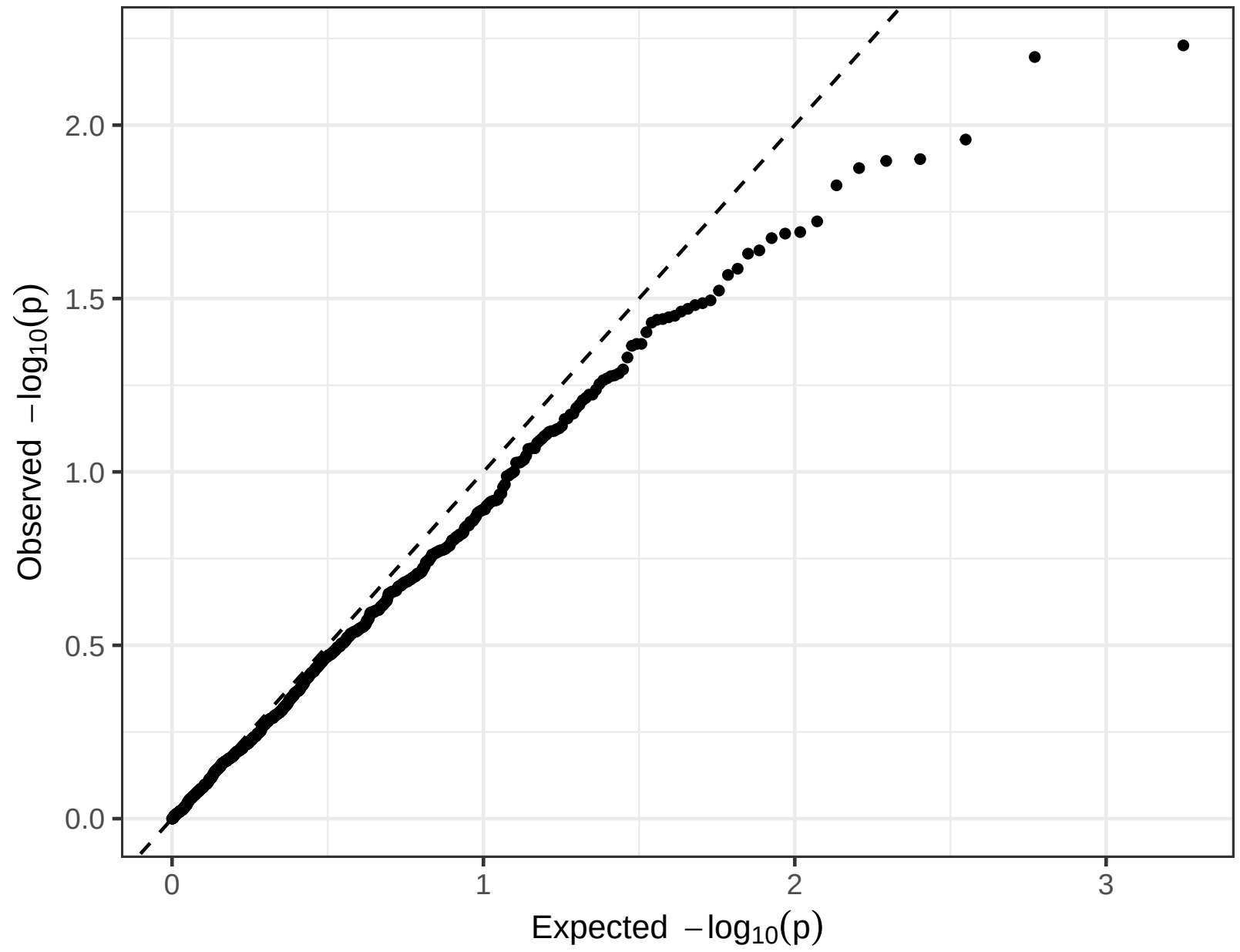

Faecalibacterium

Feces | Muscle | . = 1.18

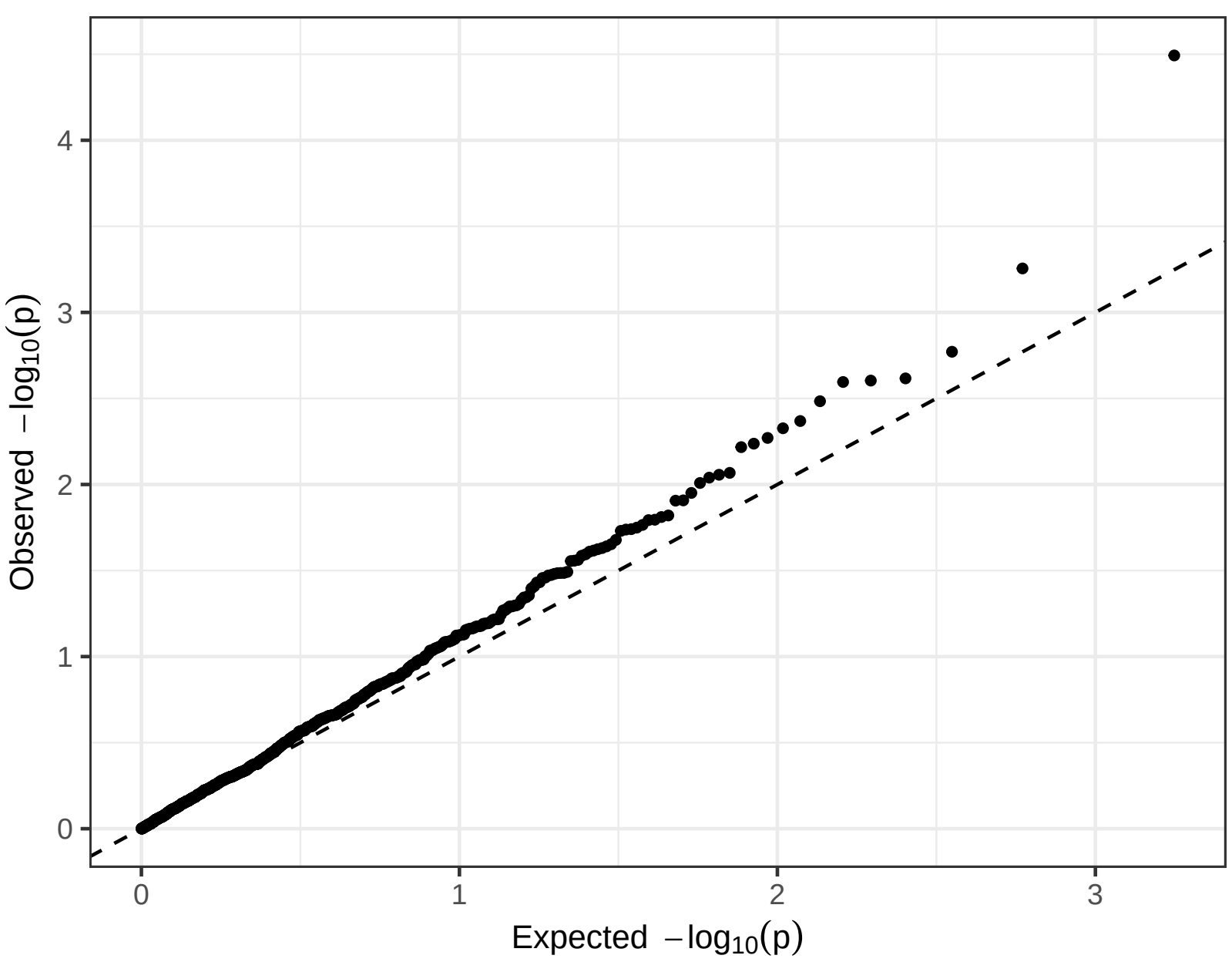

Faecalicatena

Feces | Muscle | . = 1.04

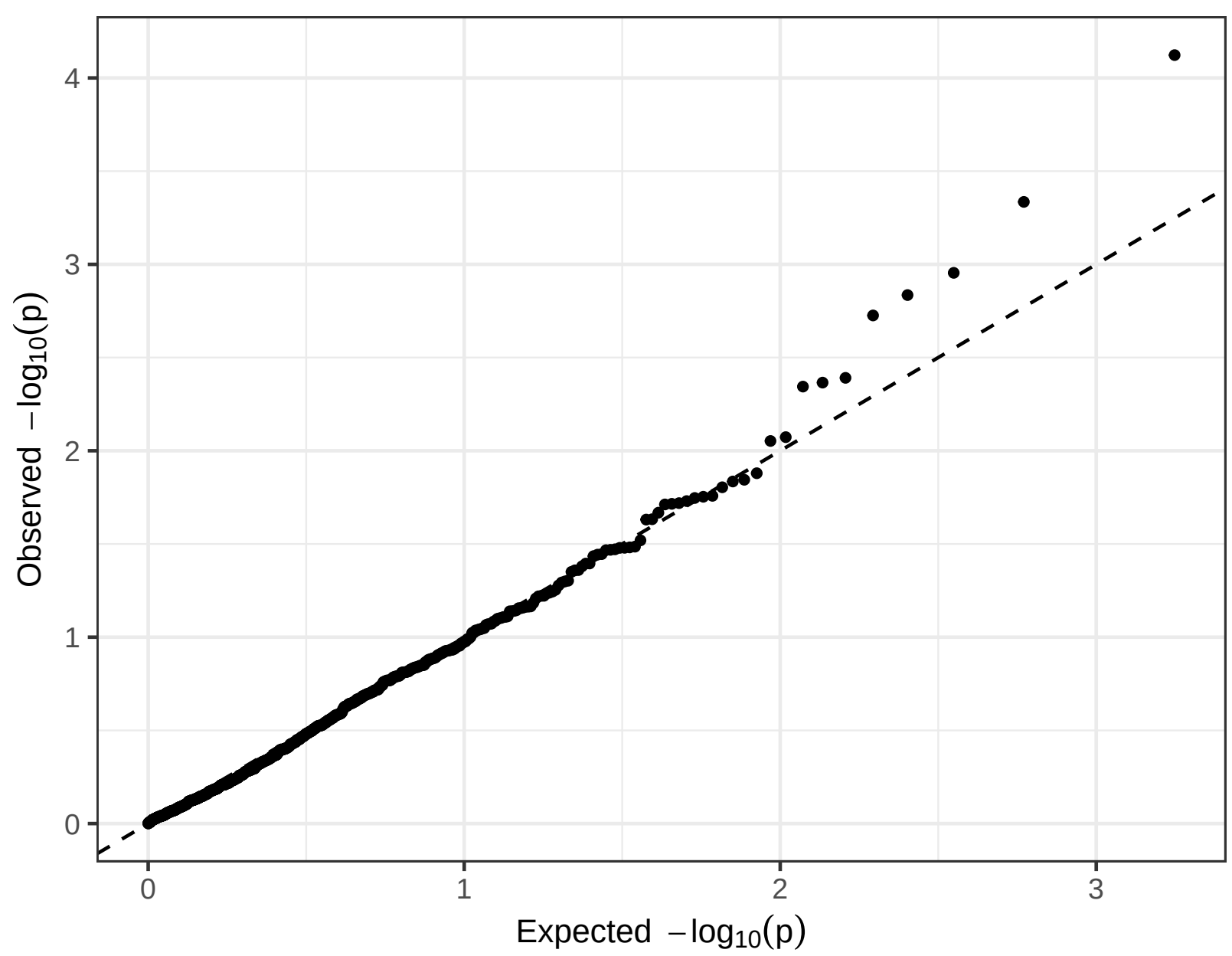

Family_XIII_AD3011_group

Feces | Muscle | . = 0.926

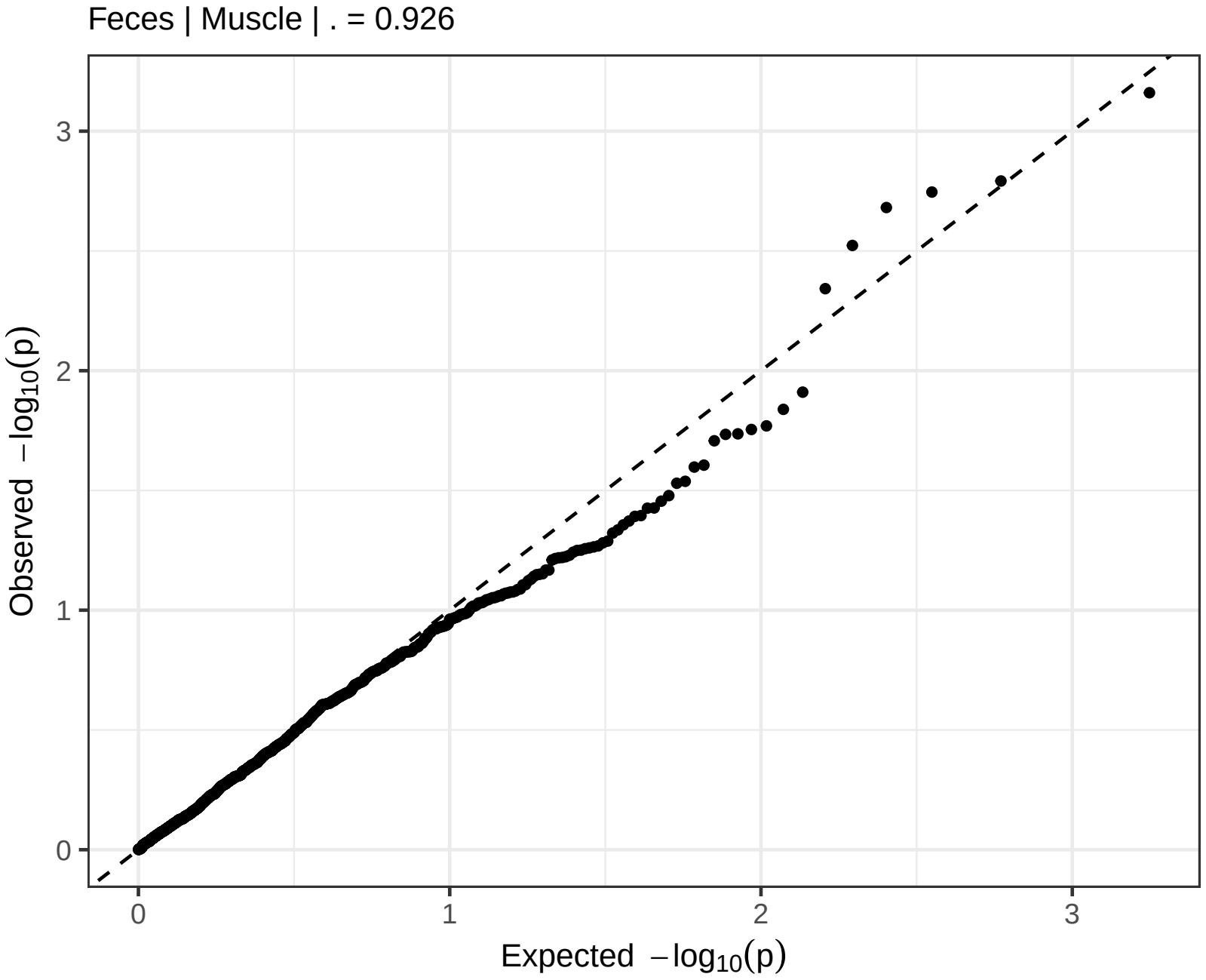

Eubacterium_xylanophilum_group

Feces | Muscle | . = 1.083

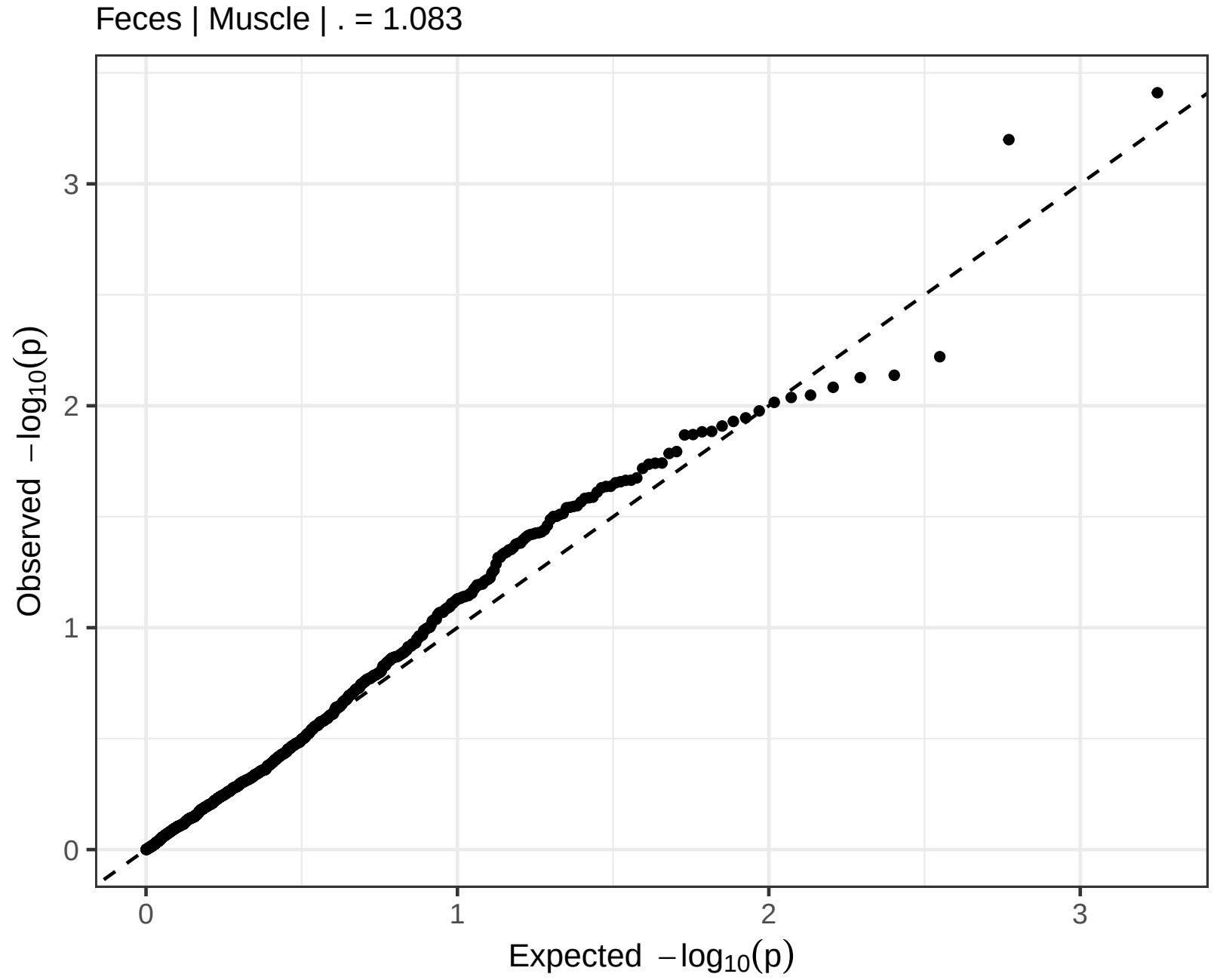

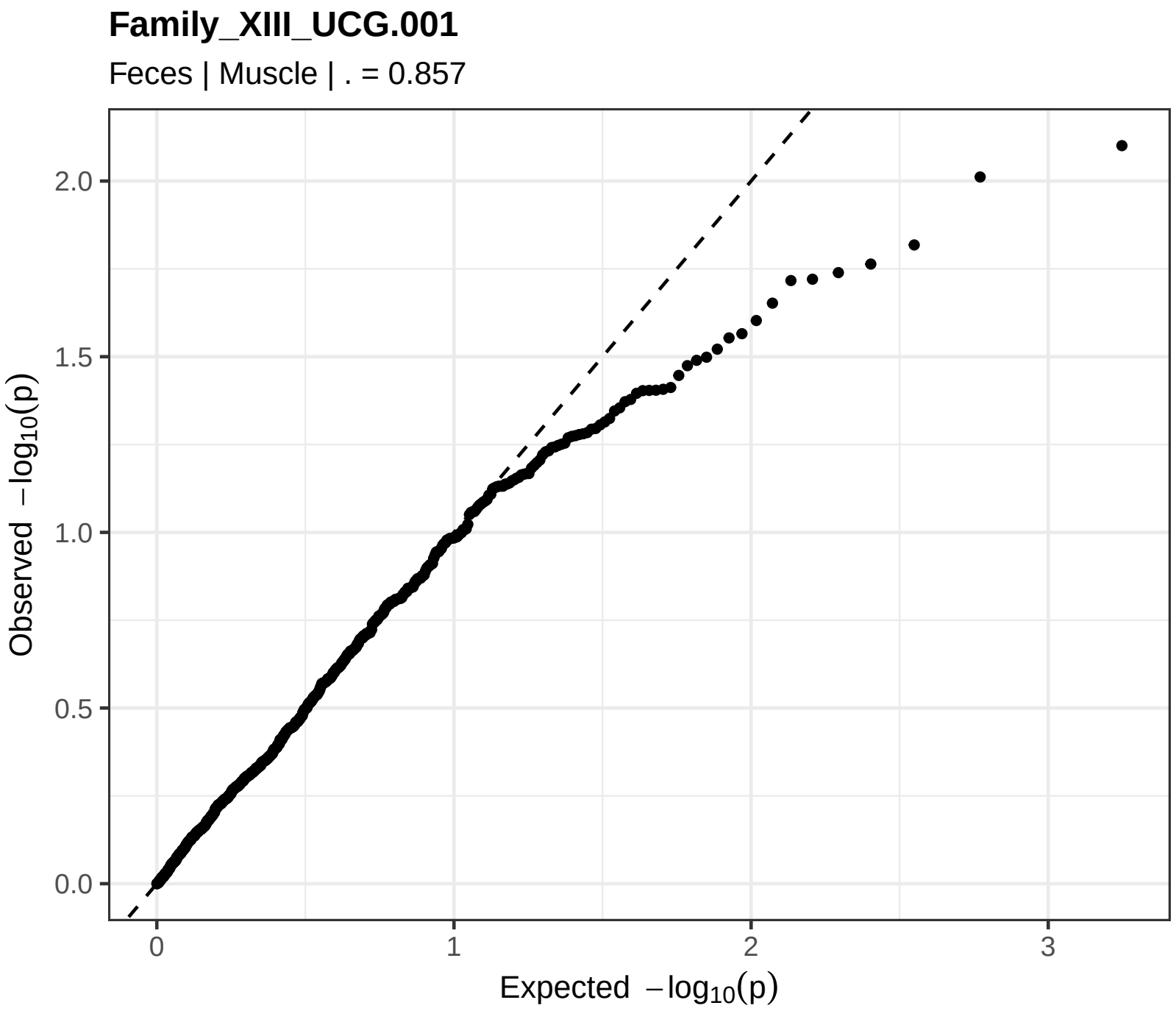
Family_XIII_UCG.001
Feces | Muscle | . = 0.857
Observed −log10(p)
Expected −log10(p)

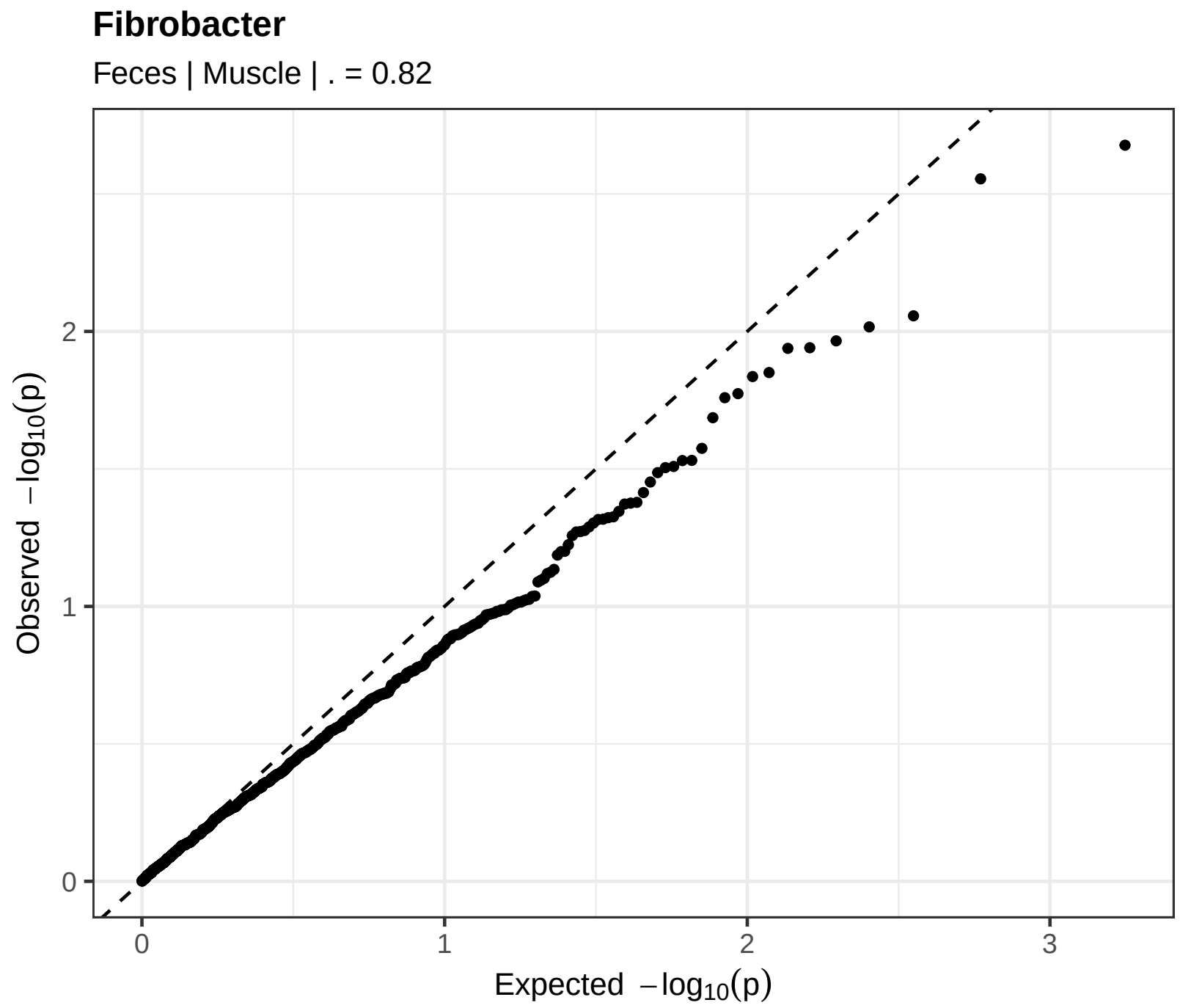
Fibrobacter
Feces | Muscle | . = 0.82
Observed −log10(p)
Expected −log10(p)

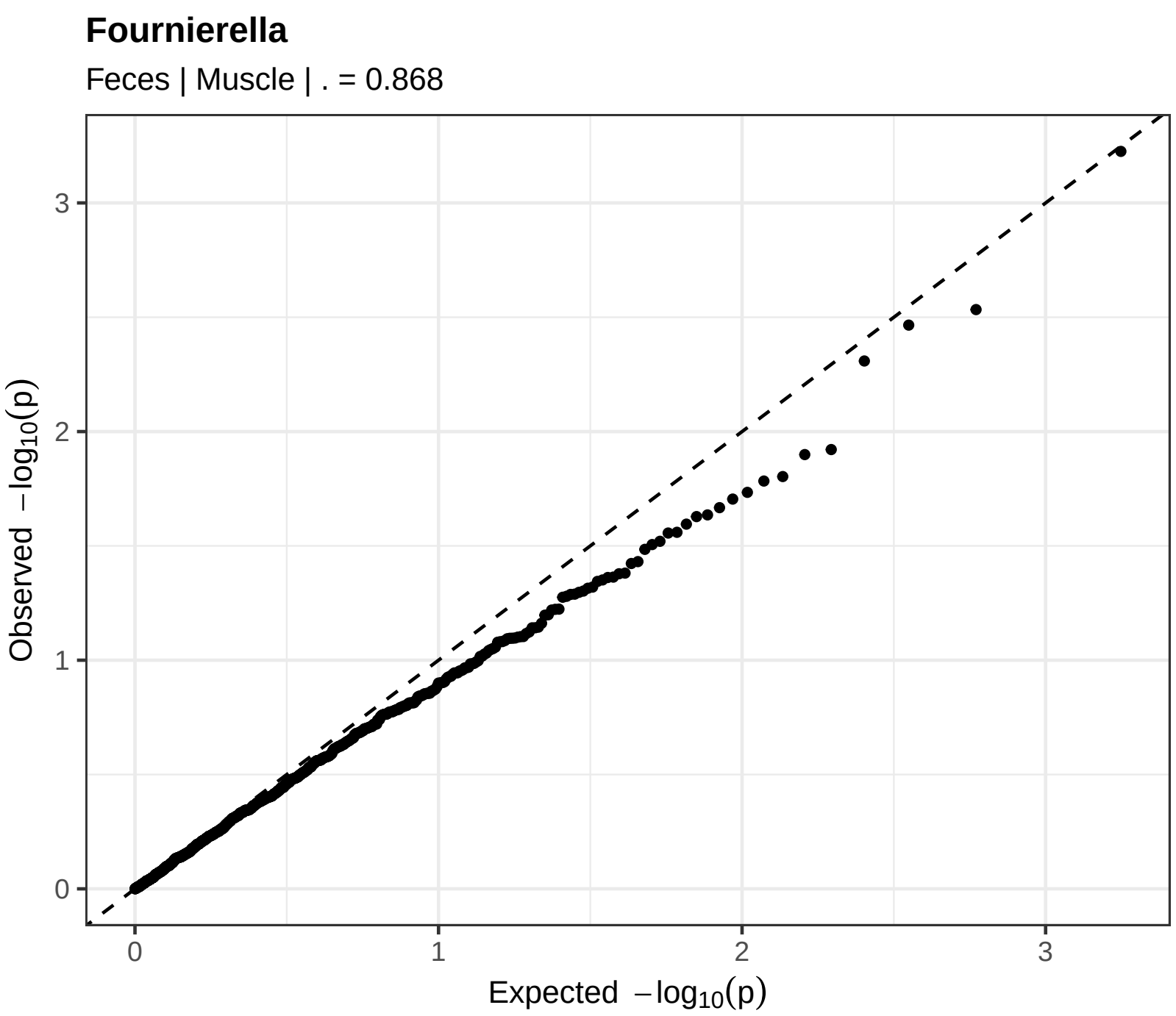
Fournierella
Feces | Muscle | . = 0.868
Observed −log10(p)
Expected −log10(p)

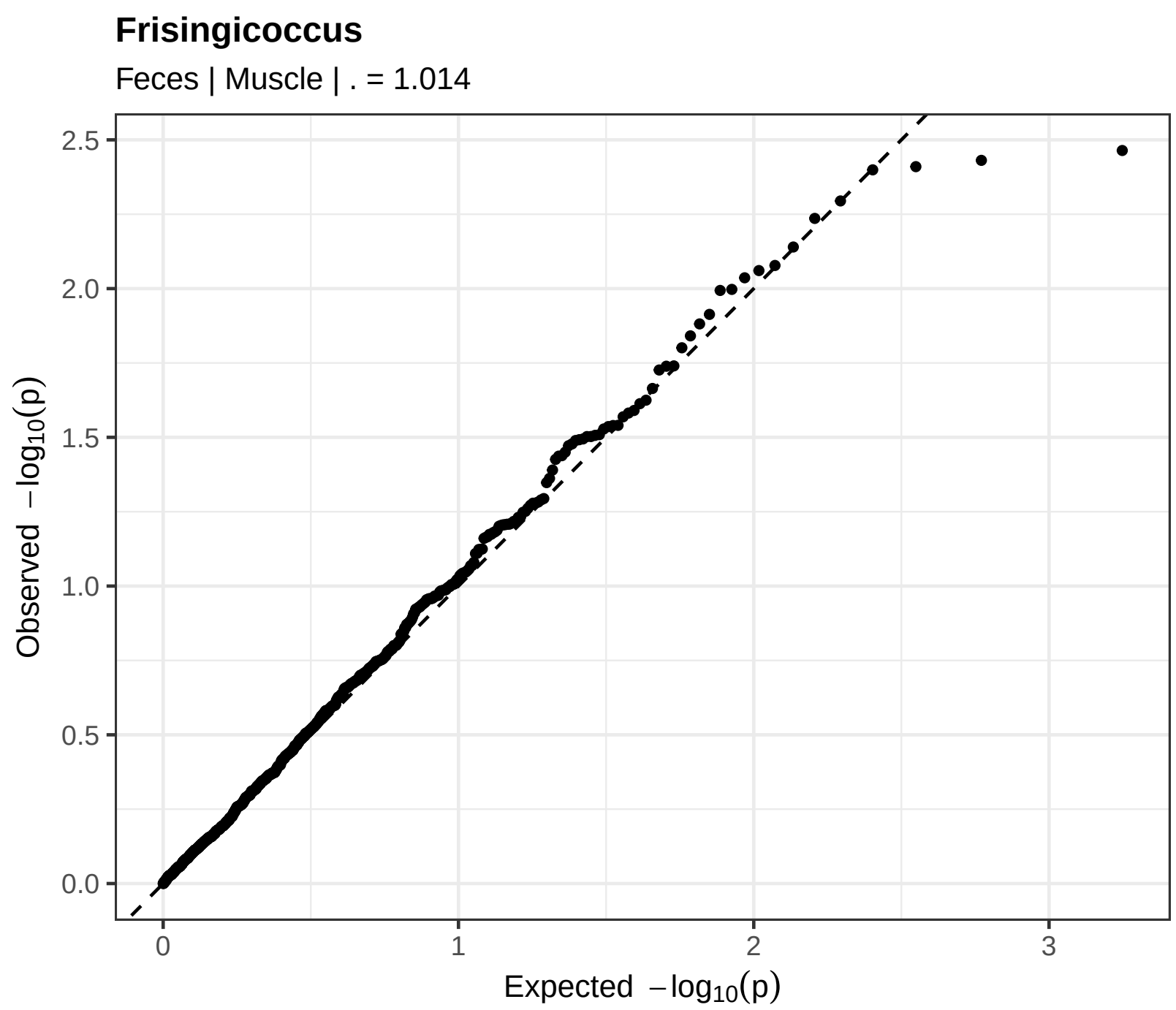
Frisingicoccus
Feces | Muscle | . = 1.014
Observed −log10(p)
Expected −log10(p)

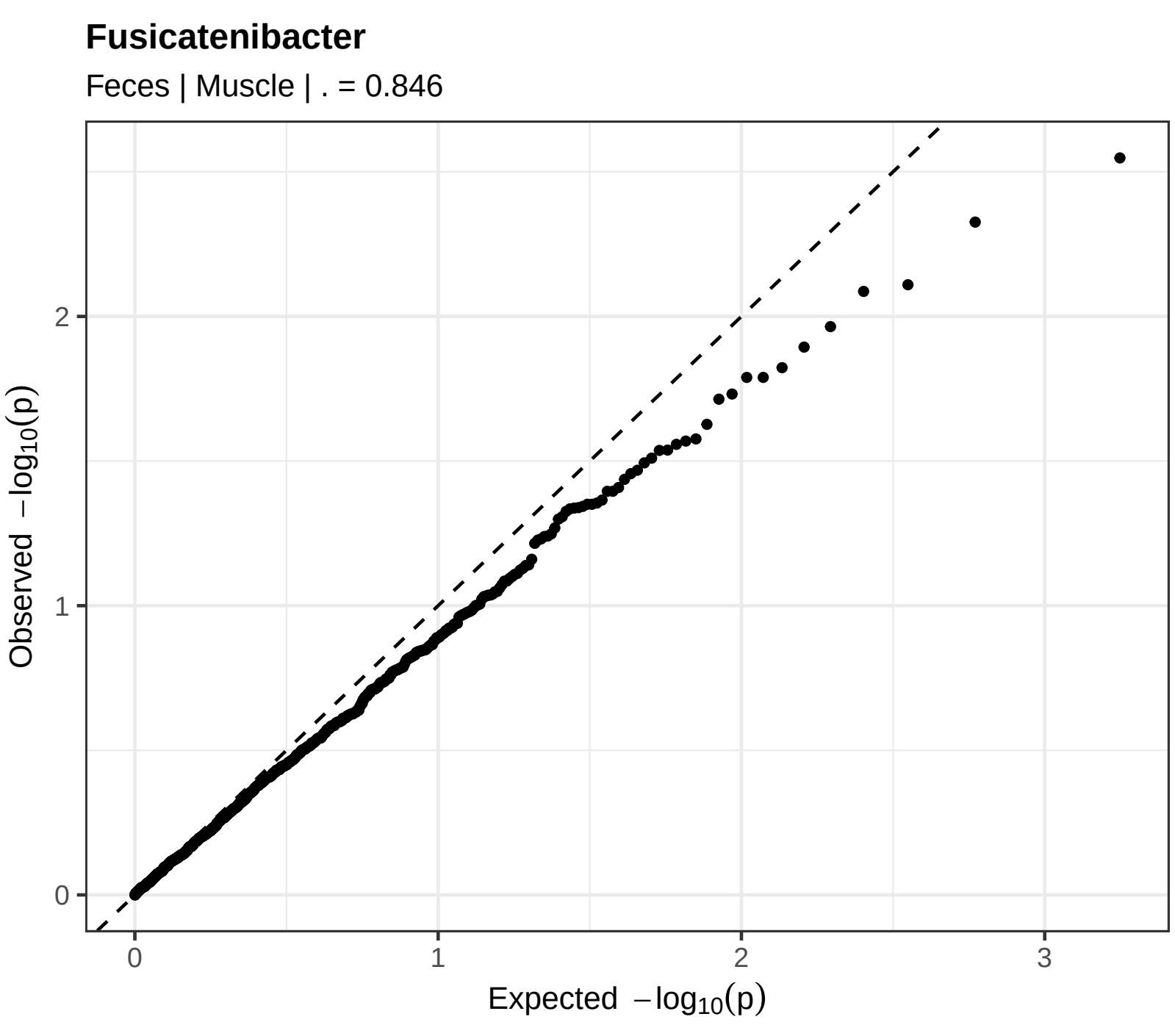
Fusicatenibacter
Feces | Muscle | . = 0.846
Observed −log10(p)
Expected −log10(p)

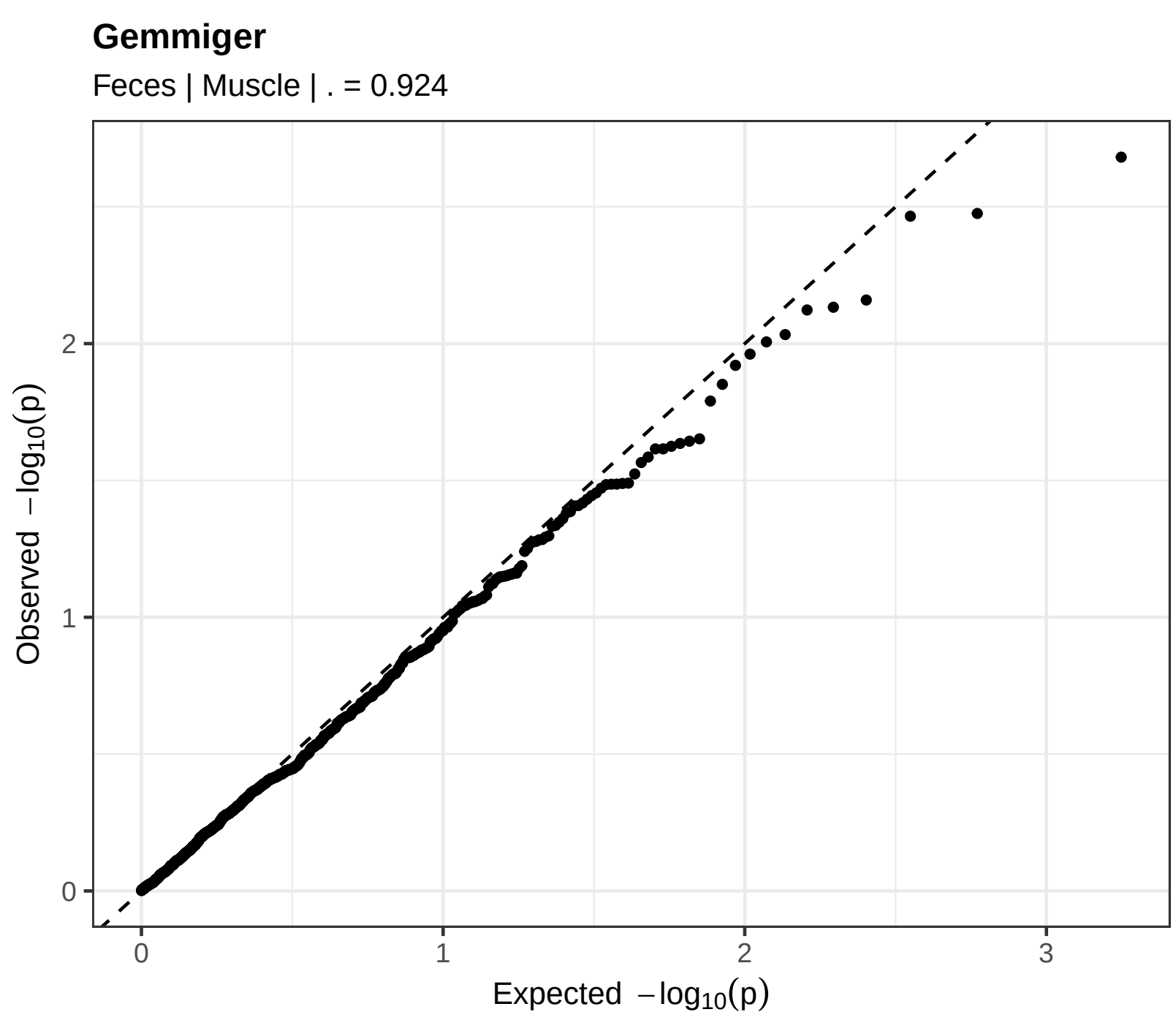
Gemmiger
Feces | Muscle | . = 0.924
Observed −log10(p)
Expected −log10(p)

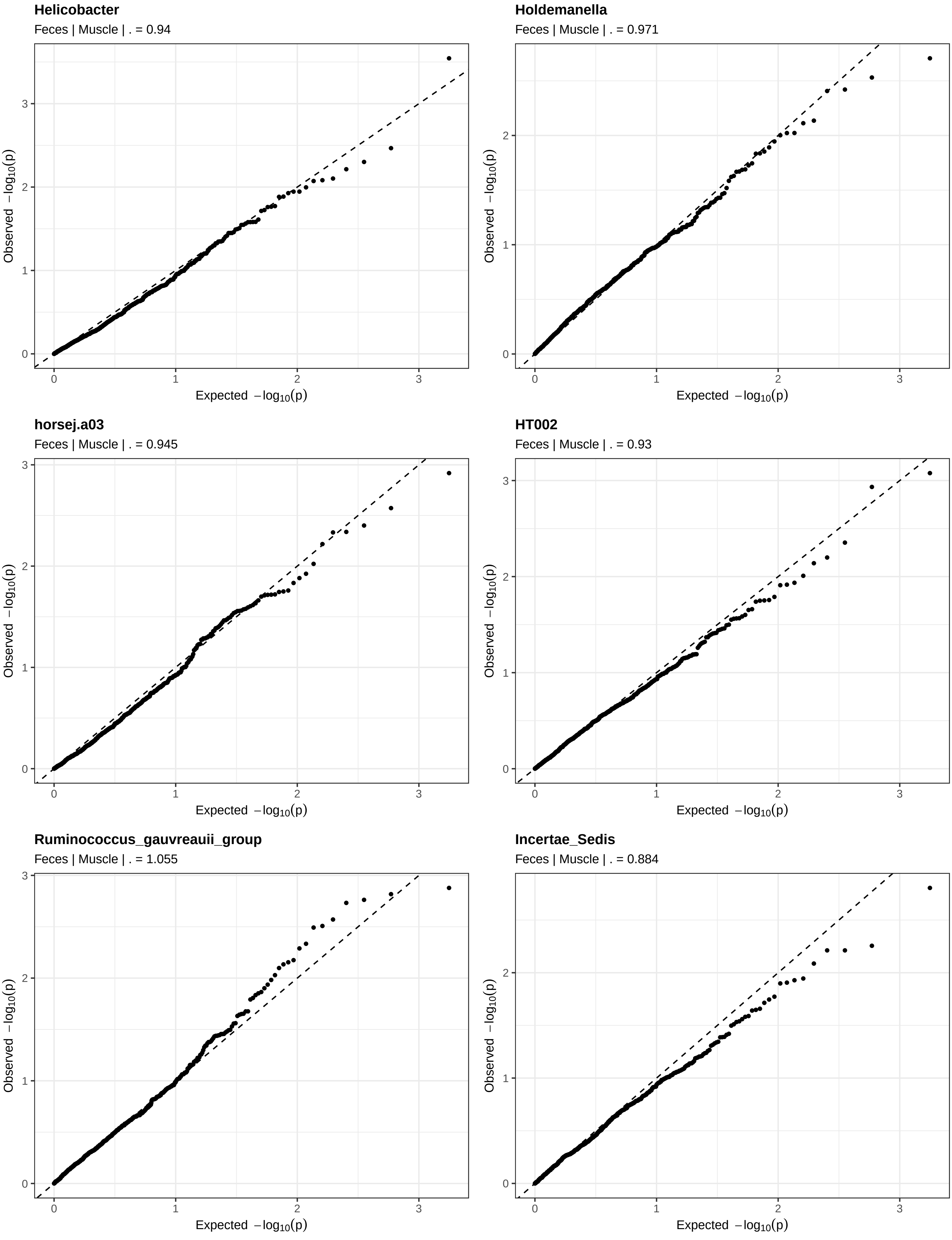

Helicobacter
Feces | Muscle | . = 0.94
Holdemanella
Feces | Muscle | . = 0.971
horsej.a03
Feces | Muscle | . = 0.945
HT002
Feces | Muscle | . = 0.93
Ruminococcus_gauvreauii_group
Feces | Muscle | . = 1.055
Incertae_Sedis
Feces | Muscle | . = 0.884
Observed $-\log_{10}(p)$
Expected $-\log_{10}(p)$
0
1
2
3

Intestinibacter

Feces | Muscle | . = 0.924

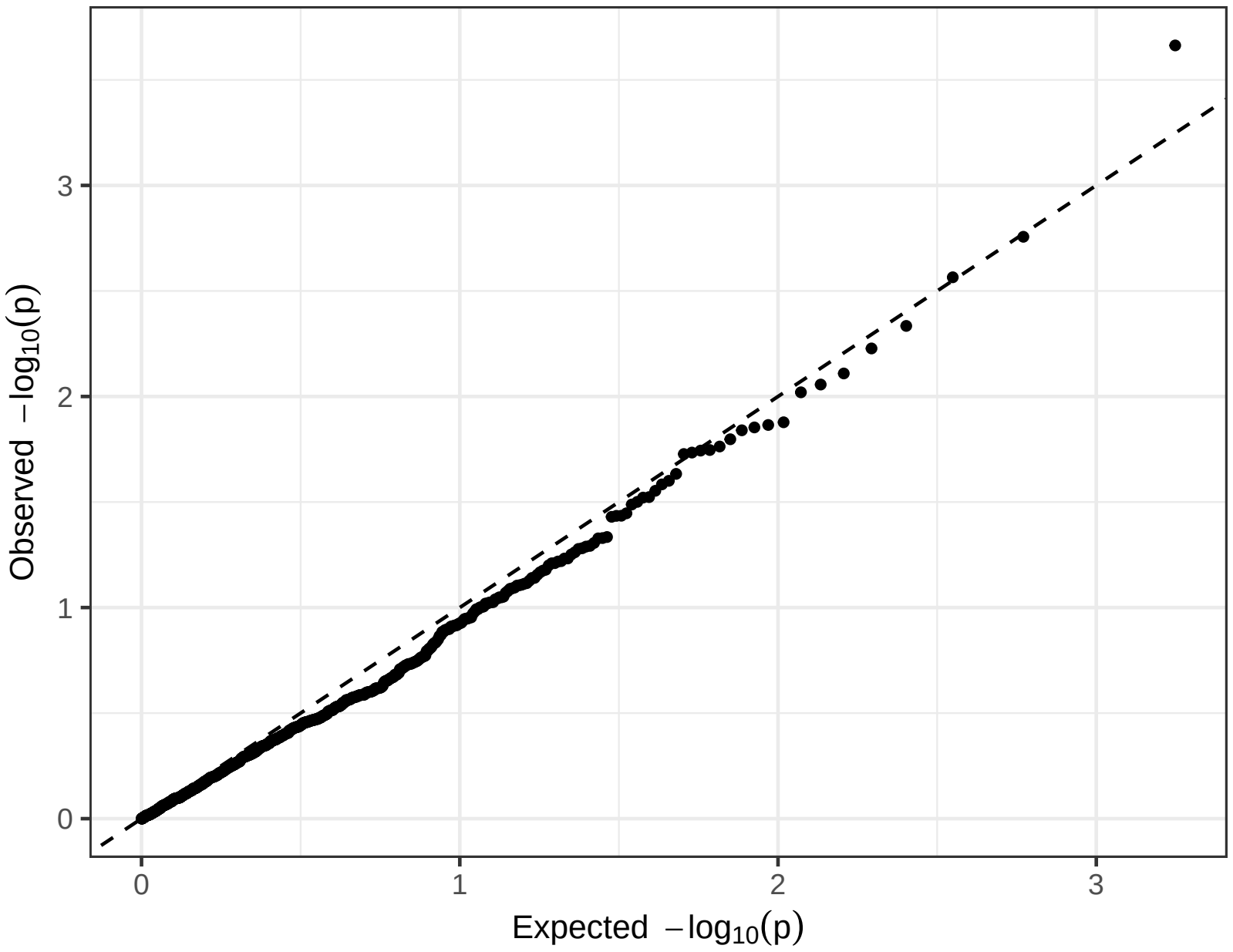

Intestinimonas

Feces | Muscle | . = 0.842

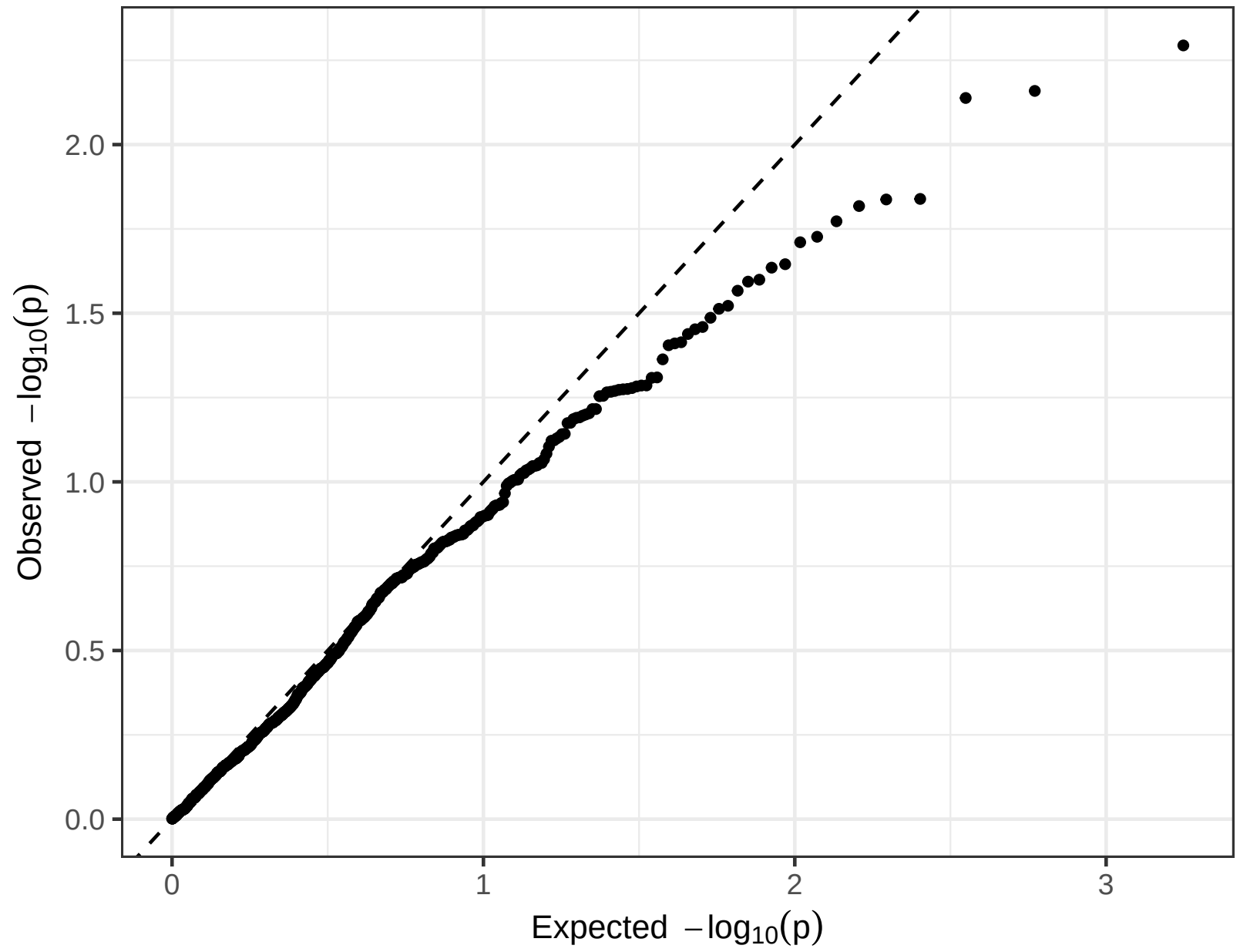

Lachnoclostridium

Feces | Muscle | . = 0.945

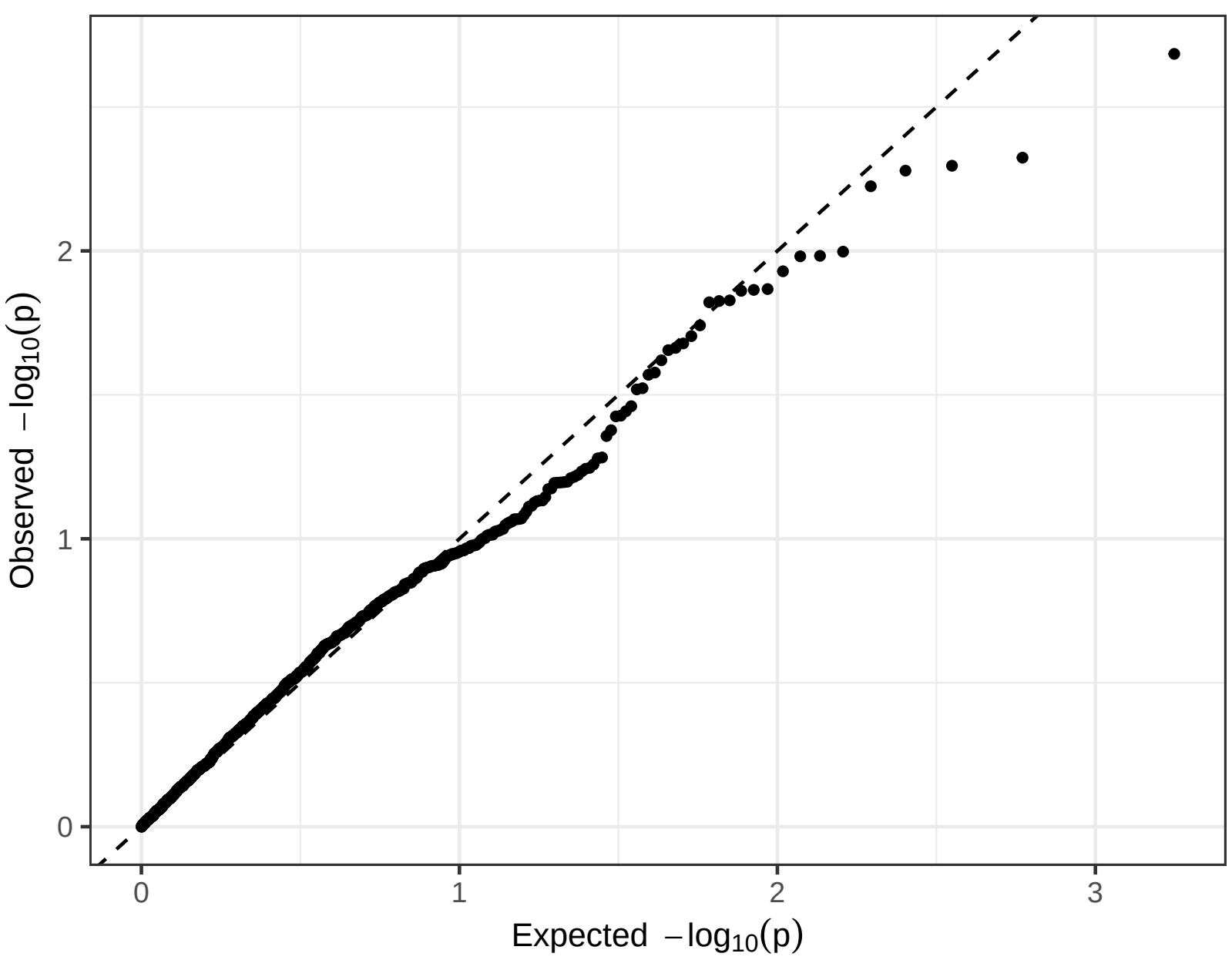

Lachnospira

Feces | Muscle | . = 0.913

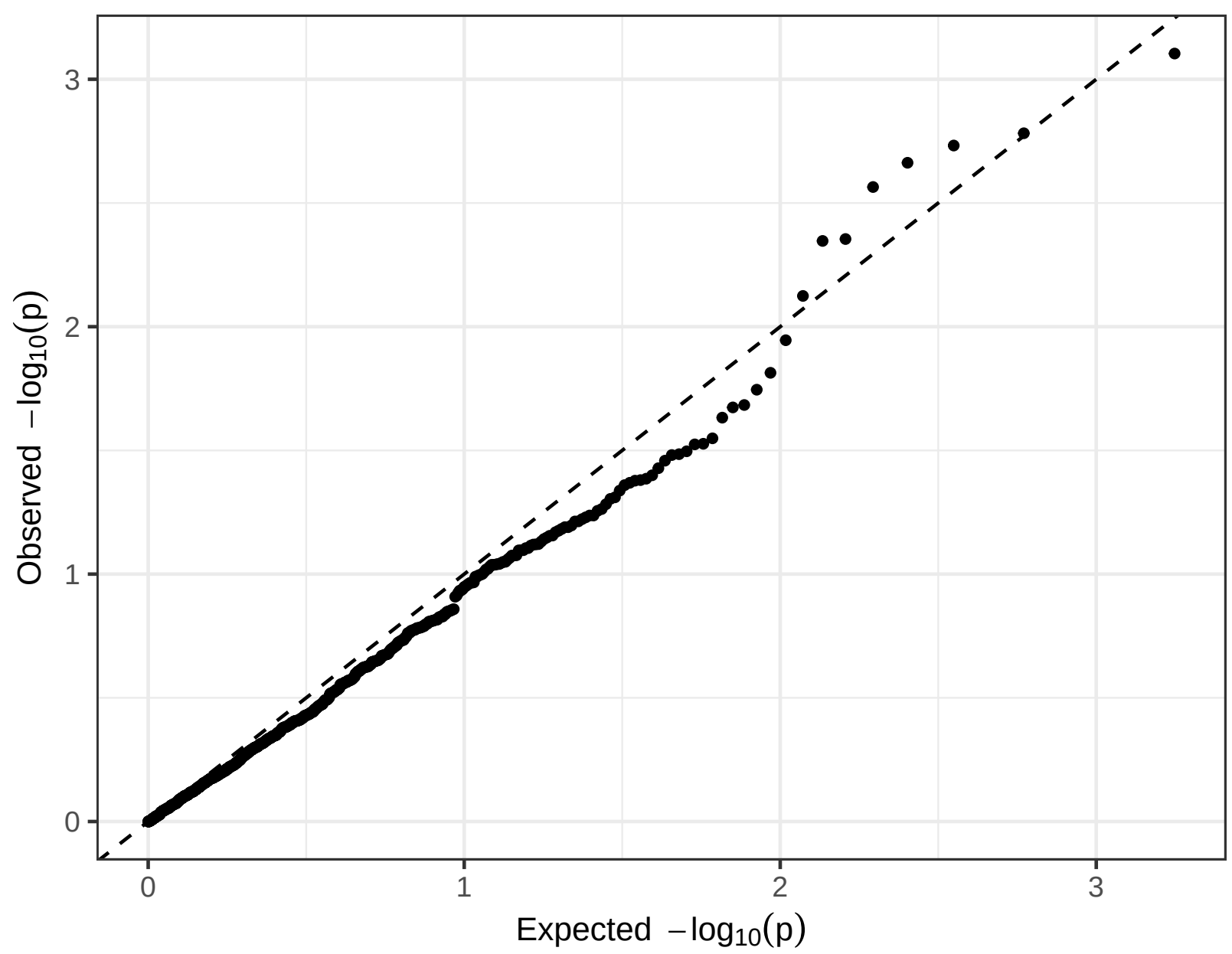

Lachnospiraceae_AC2044_group

Feces | Muscle | . = 1.01

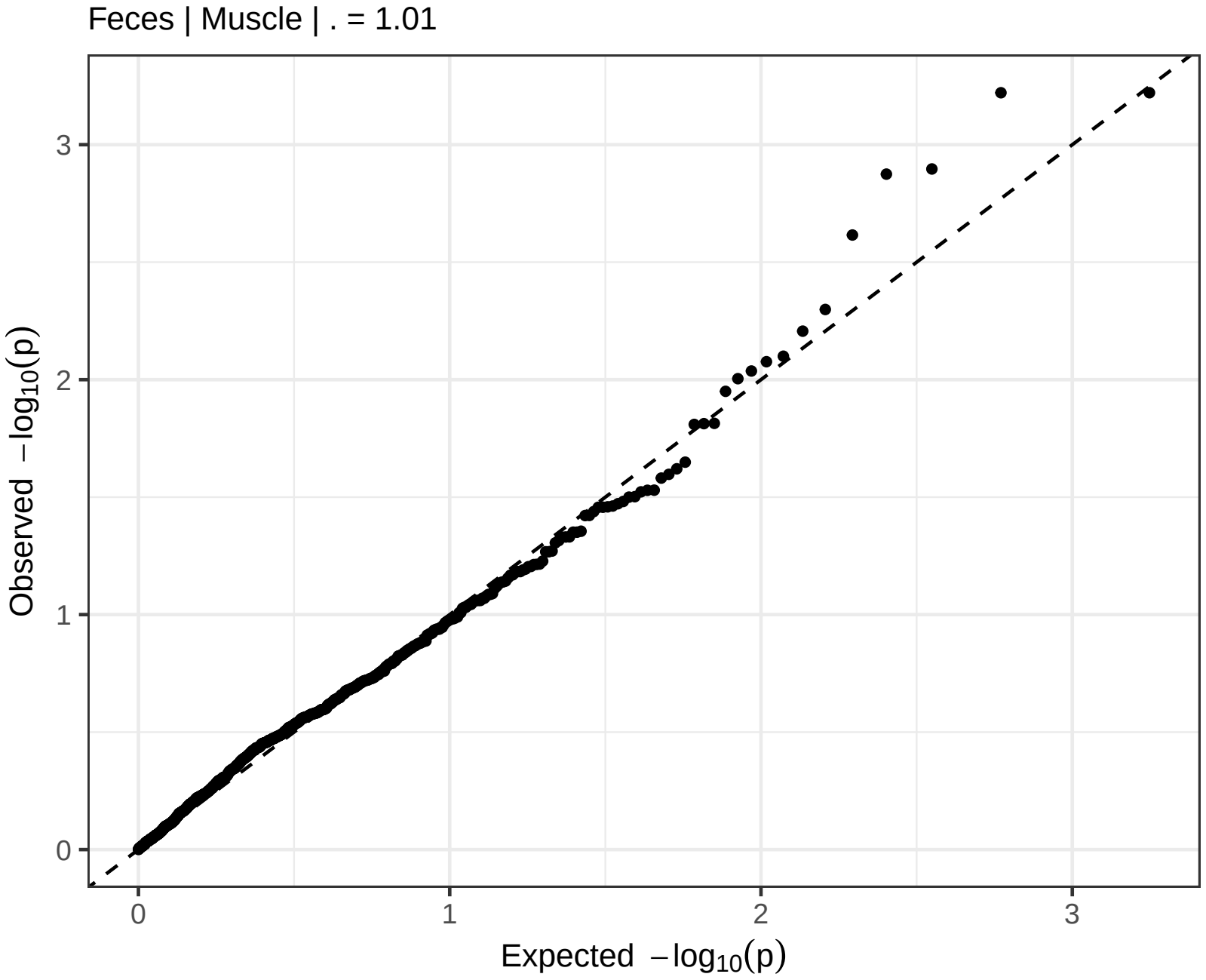

Lachnospiraceae_FCS020_group

Feces | Muscle | . = 0.928

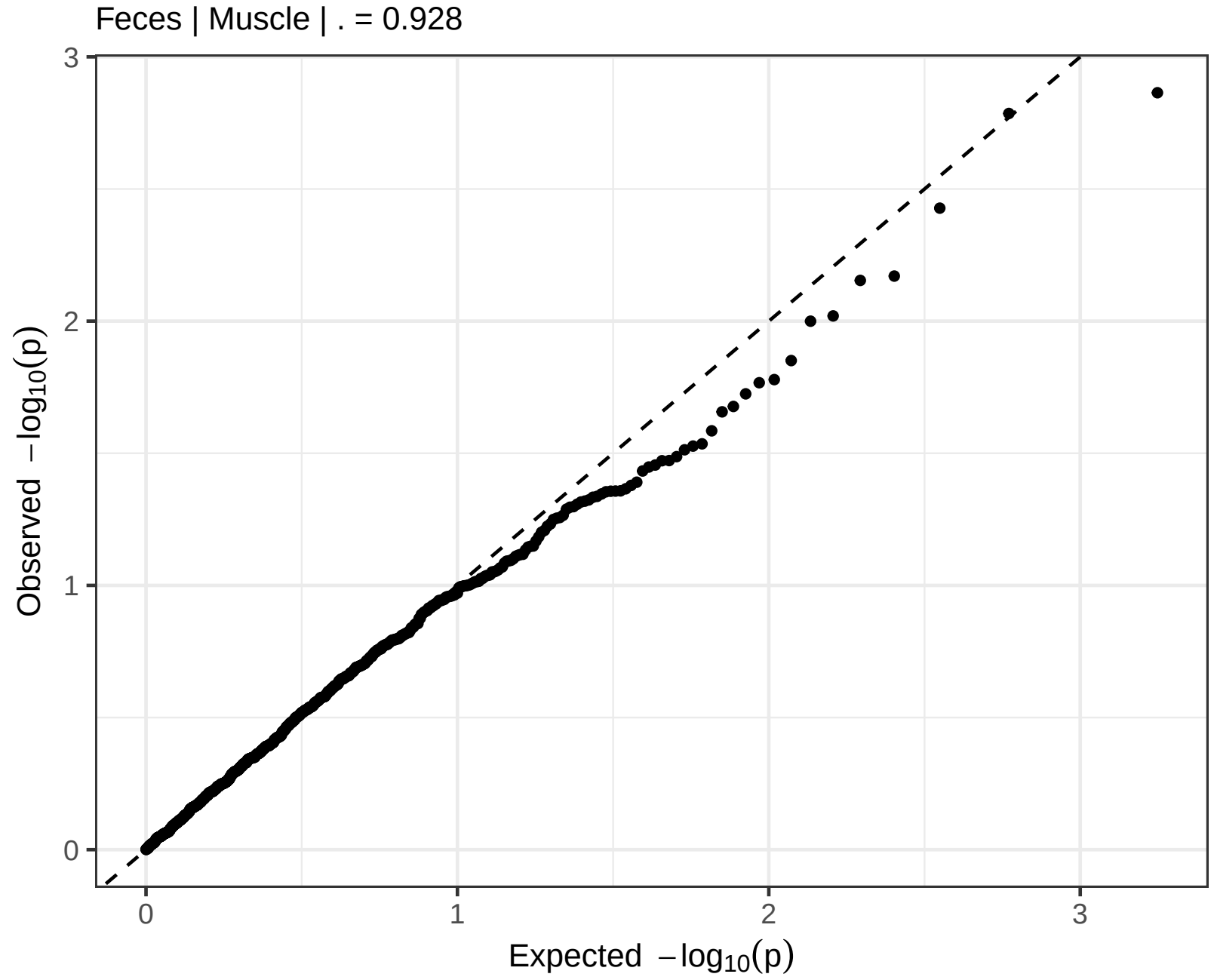

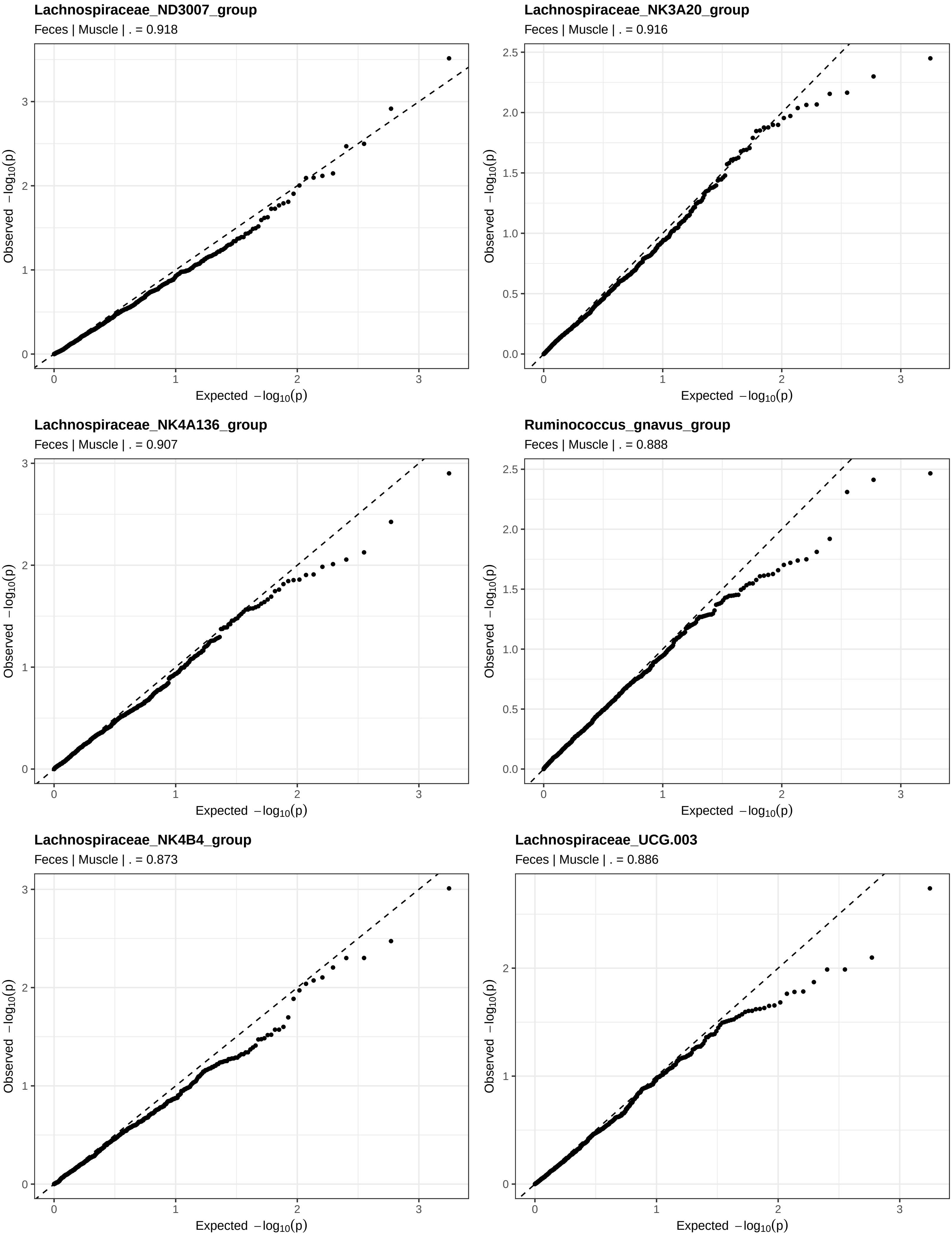

Lachnospiraceae_ND3007_group
Feces | Muscle | . = 0.918
Lachnospiraceae_NK3A20_group
Feces | Muscle | . = 0.916
Lachnospiraceae_NK4A136_group
Feces | Muscle | . = 0.907
Ruminococcus_gnavus_group
Feces | Muscle | . = 0.888
Lachnospiraceae_NK4B4_group
Feces | Muscle | . = 0.873
Lachnospiraceae_UCG.003
Feces | Muscle | . = 0.886
Observed $-\log_{10}(p)$
Expected $-\log_{10}(p)$

Lachnospiraceae_UCG.004

Feces | Muscle | . = 1.199

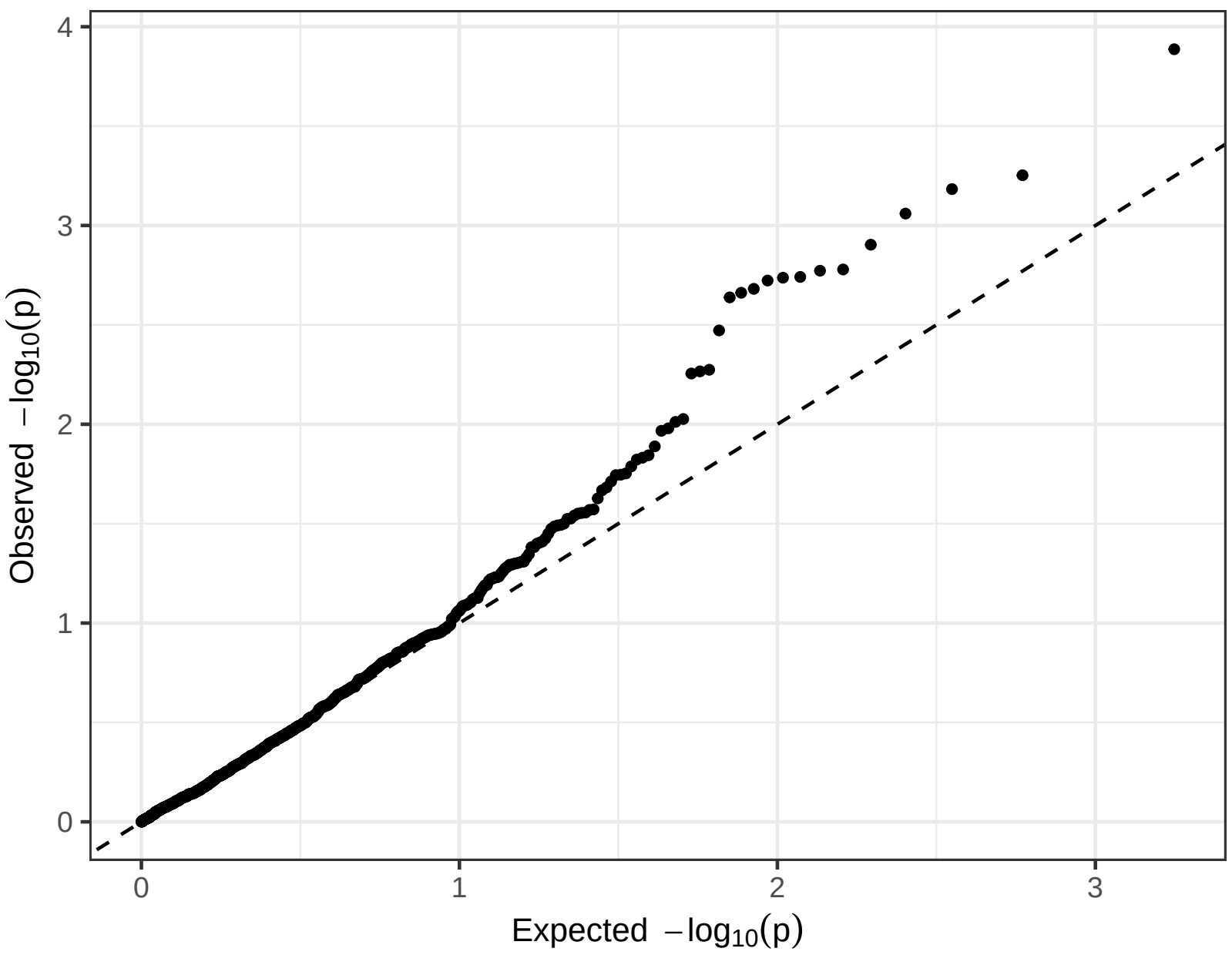

Lachnospiraceae_UCG.007

Feces | Muscle | . = 0.916

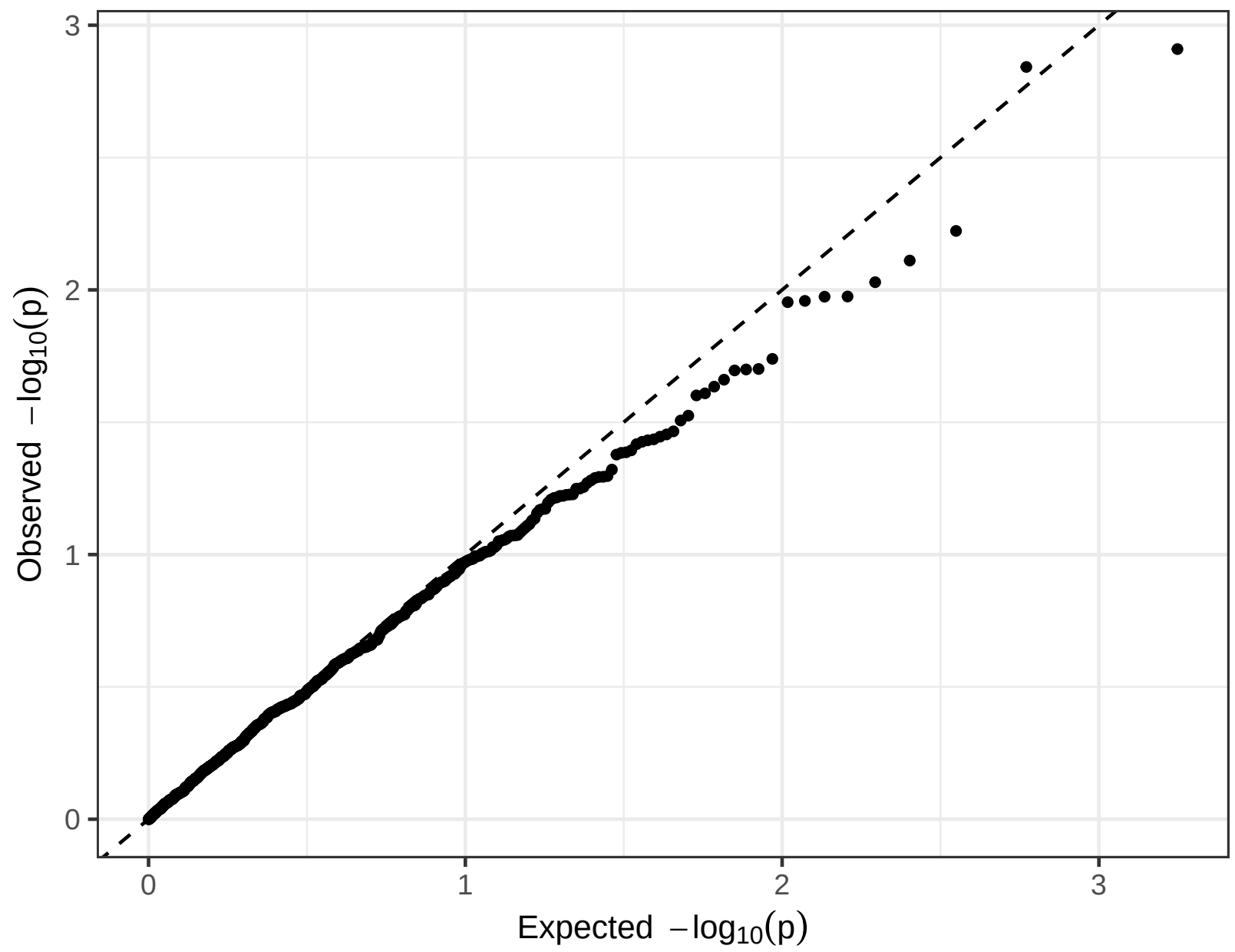

Lachnospiraceae_UCG.009

Feces | Muscle | . = 0.914

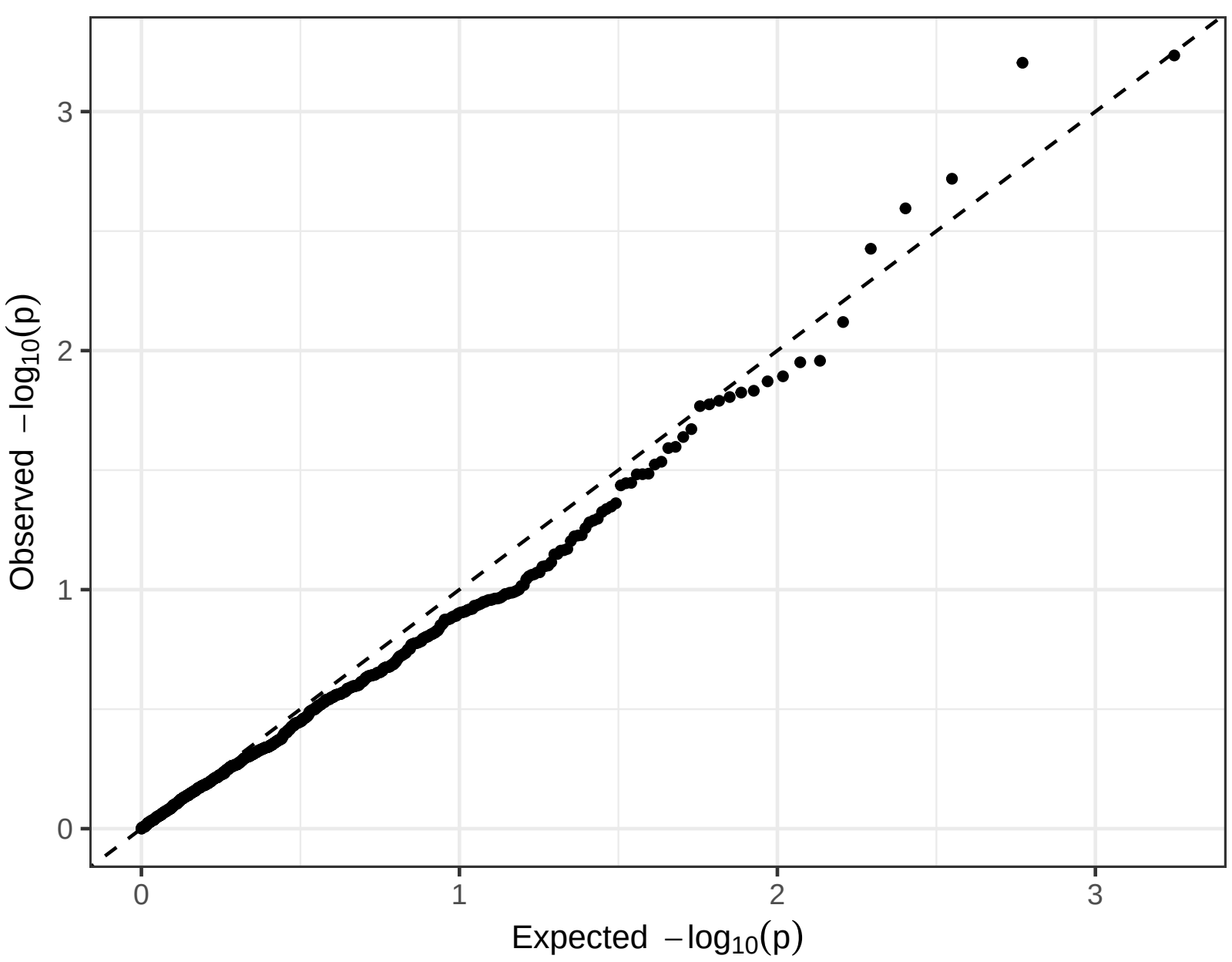

Lachnospiraceae_UCG.010

Feces | Muscle | . = 0.919

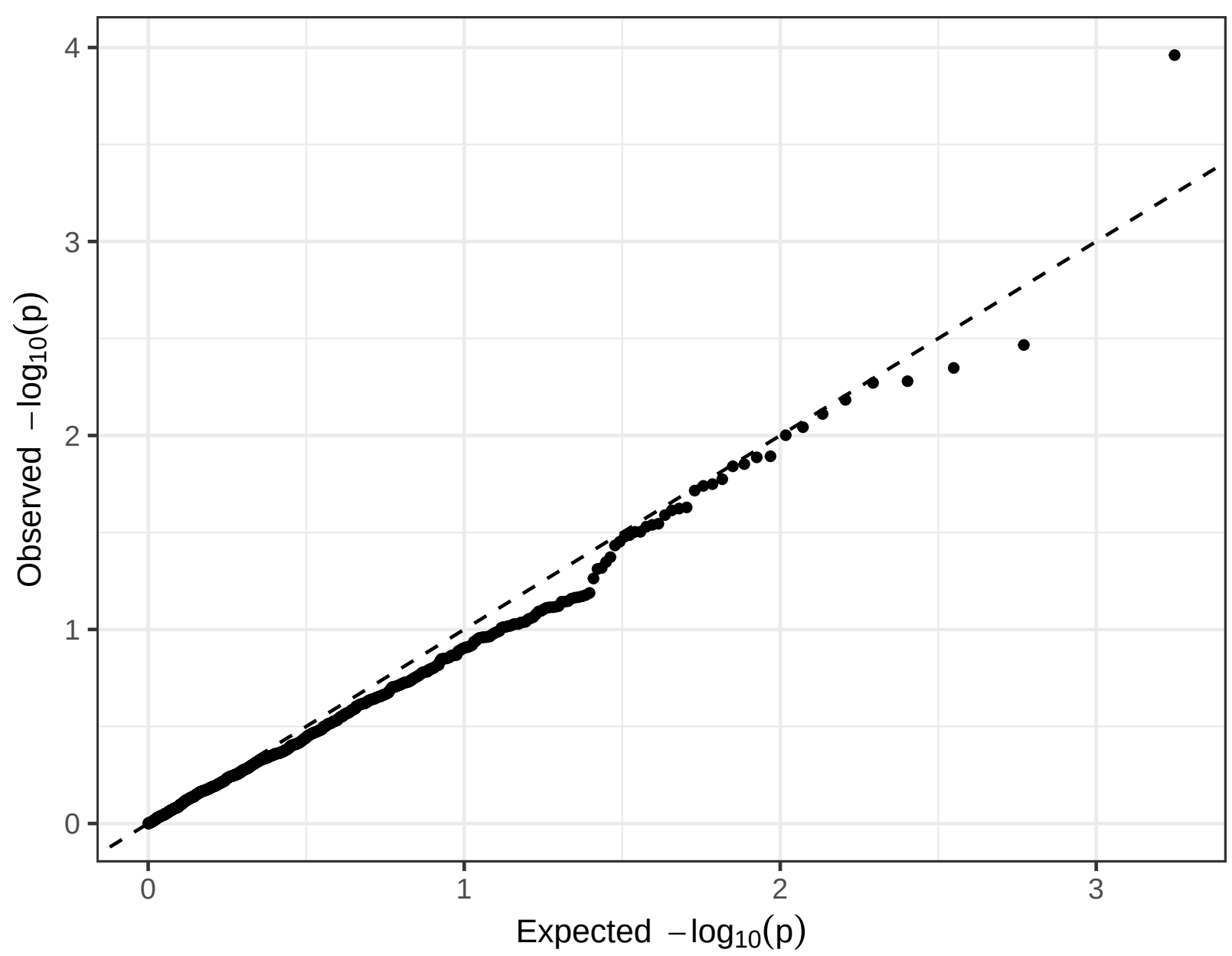

Lachnospiraceae_XPB1014_group

Feces | Muscle | . = 0.942

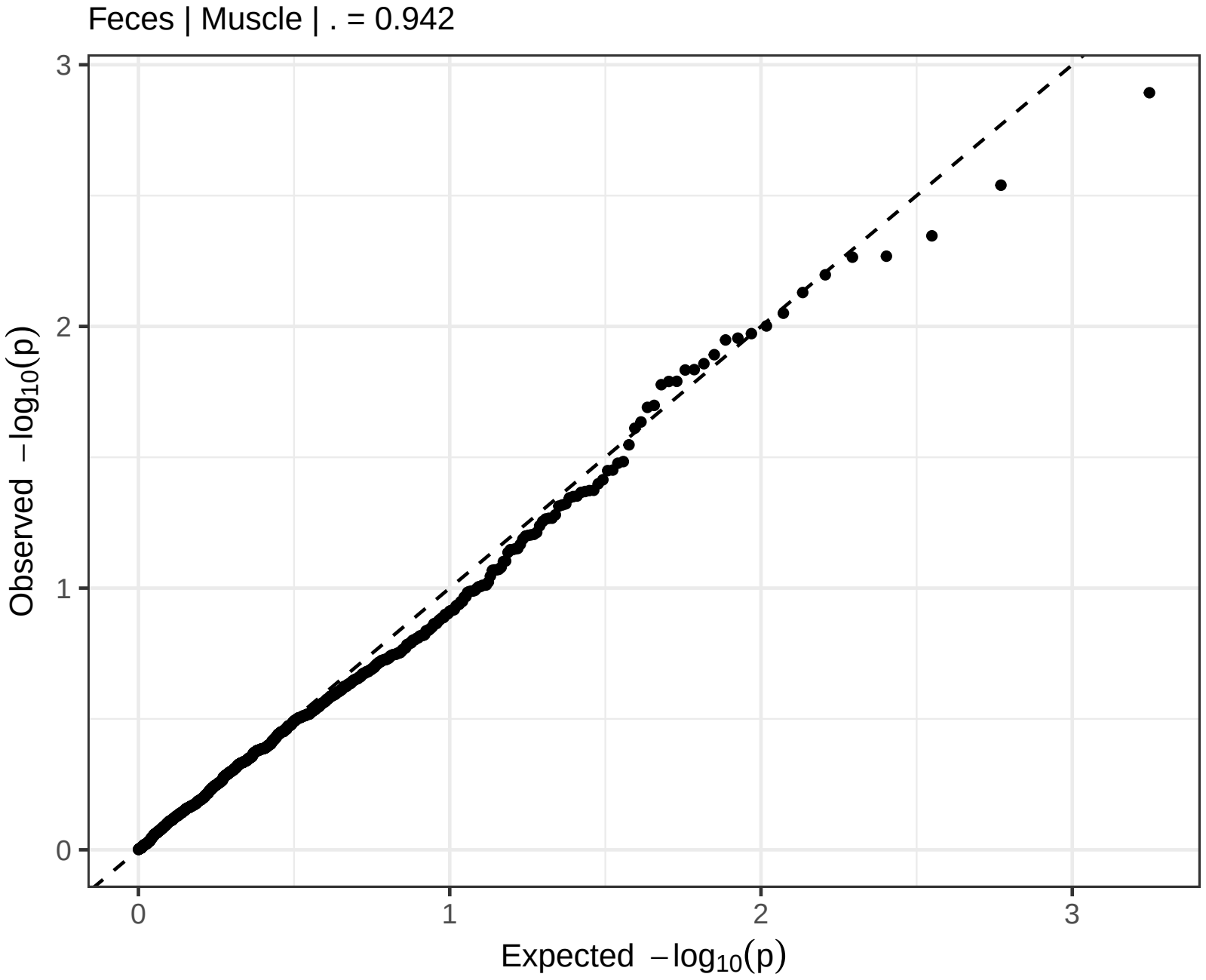

Lactobacillus

Feces | Muscle | . = 0.991

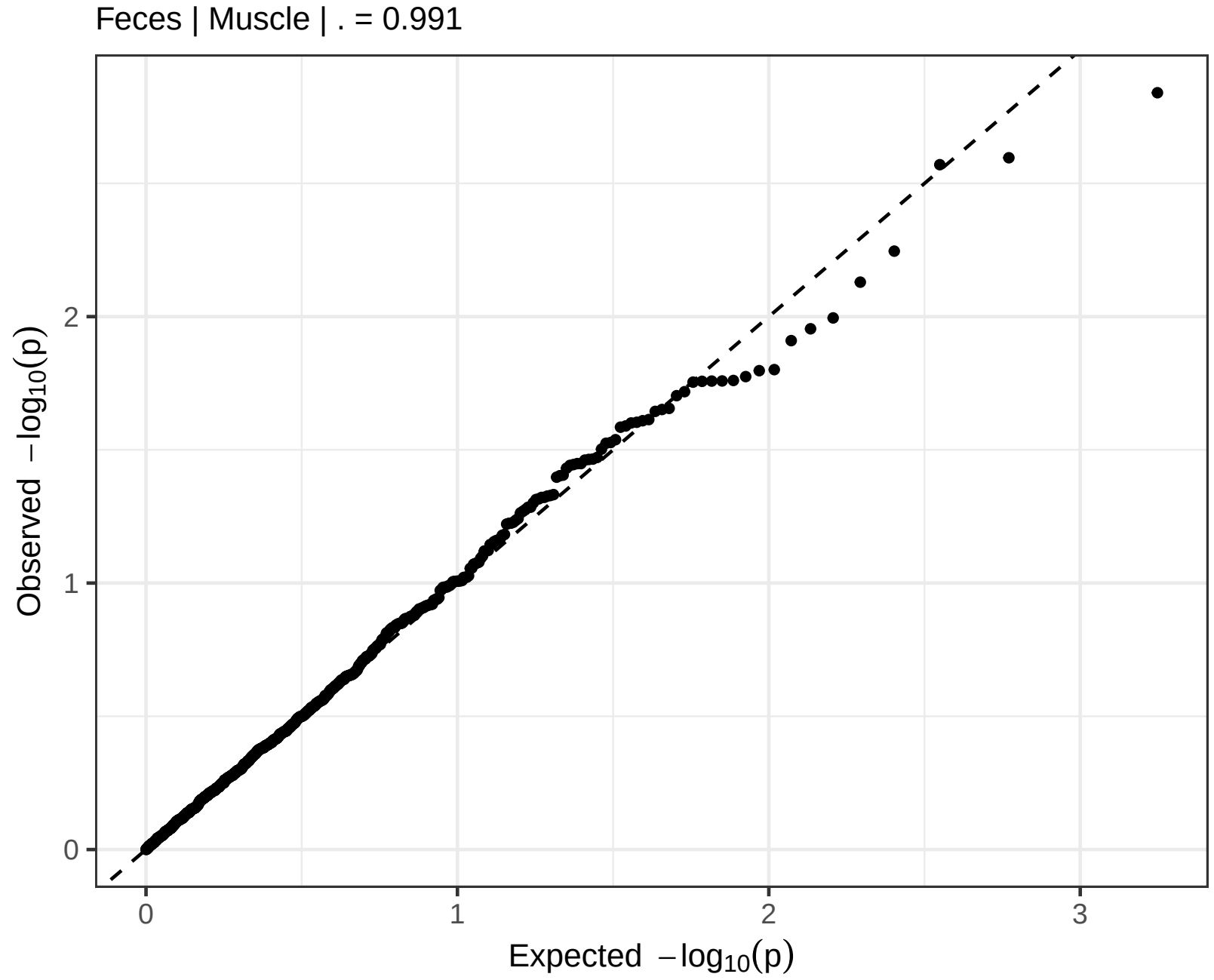

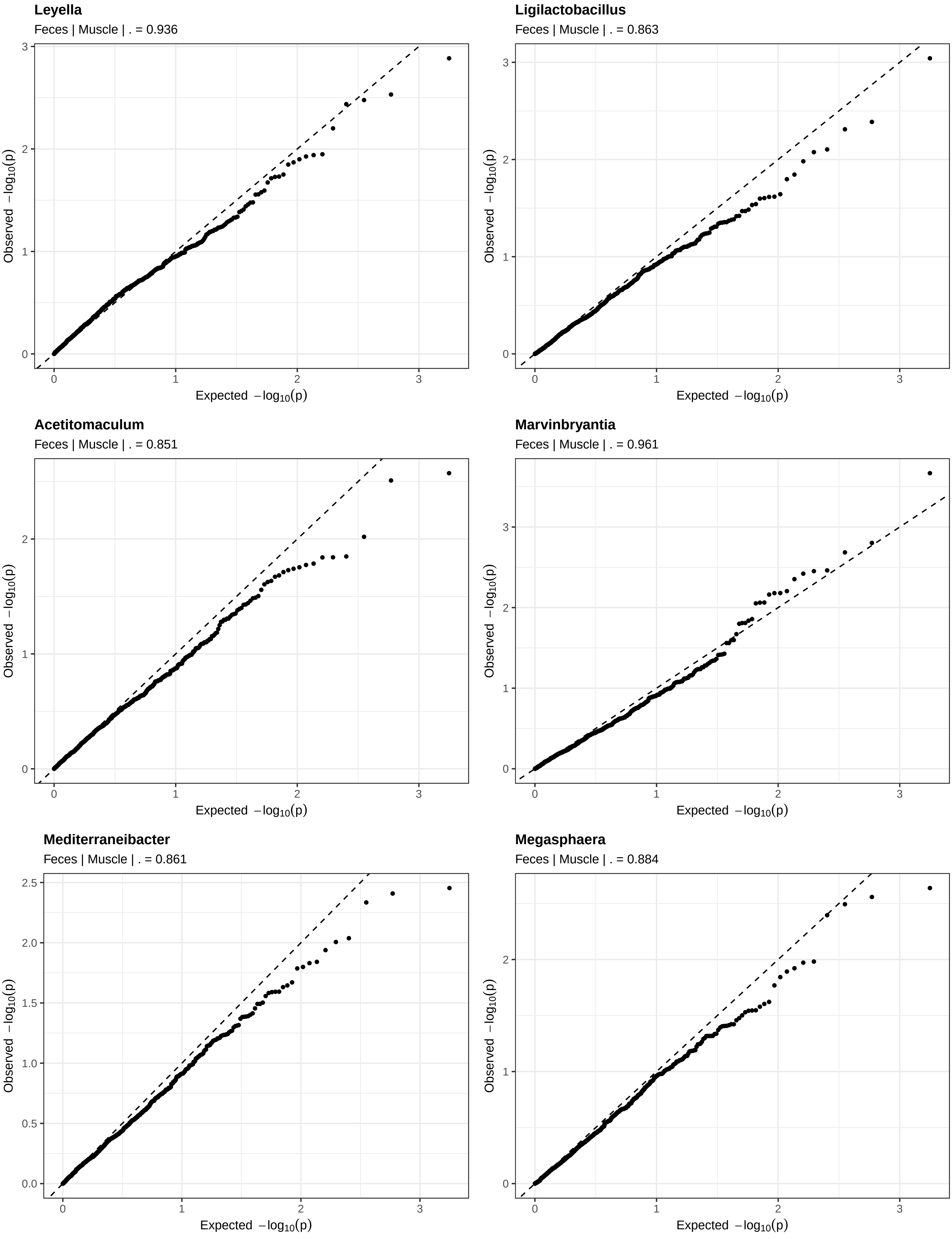
Leyella
Feces | Muscle | . = 0.936
Ligilactobacillus
Feces | Muscle | . = 0.863
Acetitomaculum
Feces | Muscle | . = 0.851
Marvinbryantia
Feces | Muscle | . = 0.961
Mediterraneibacter
Feces | Muscle | . = 0.861
Megasphaera
Feces | Muscle | . = 0.884
Observed $-\log_{10}(p)$
Expected $-\log_{10}(p)$

Metamycoplasma

Feces | Muscle | . = 0.92

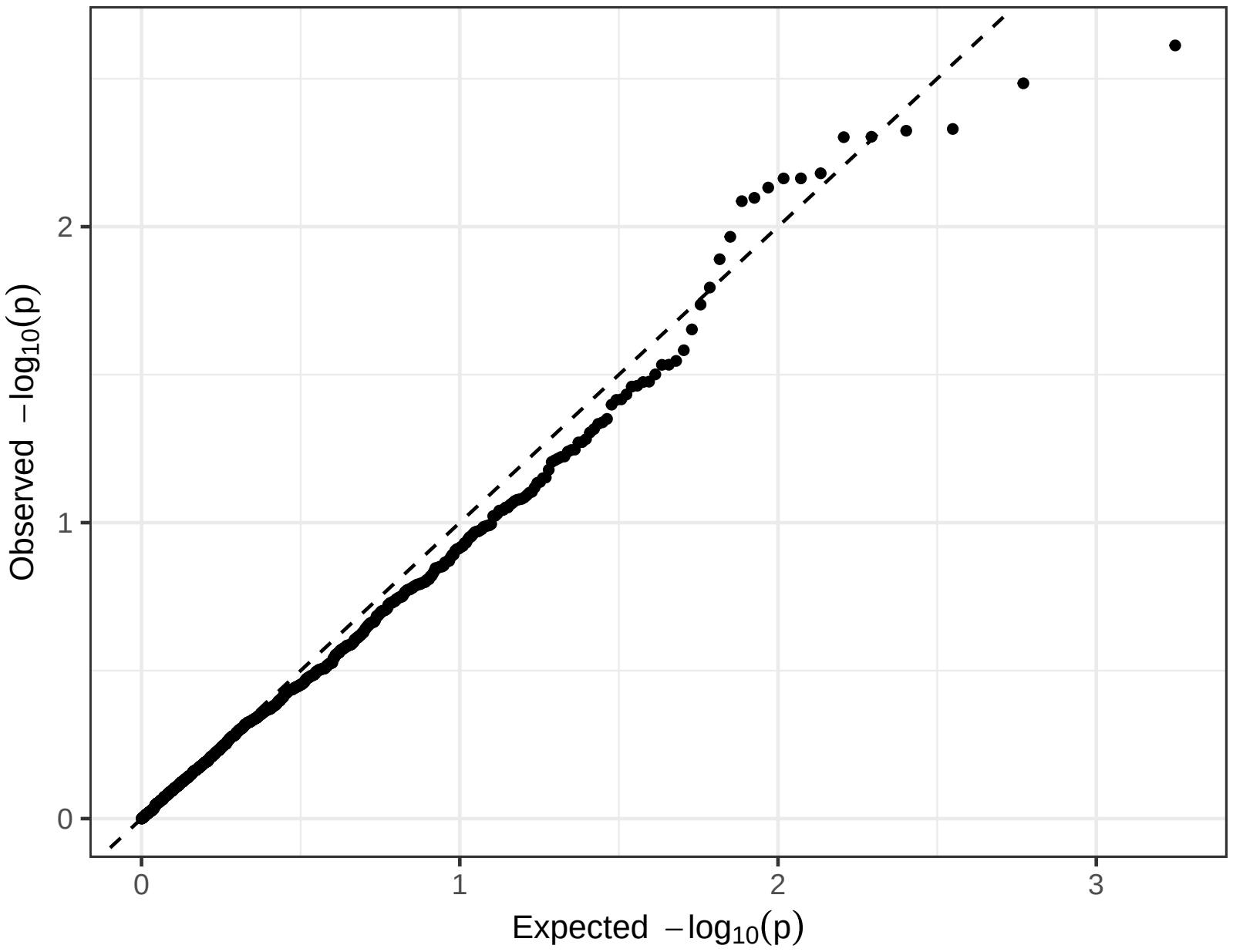

Methanobrevibacter

Feces | Muscle | . = 0.851

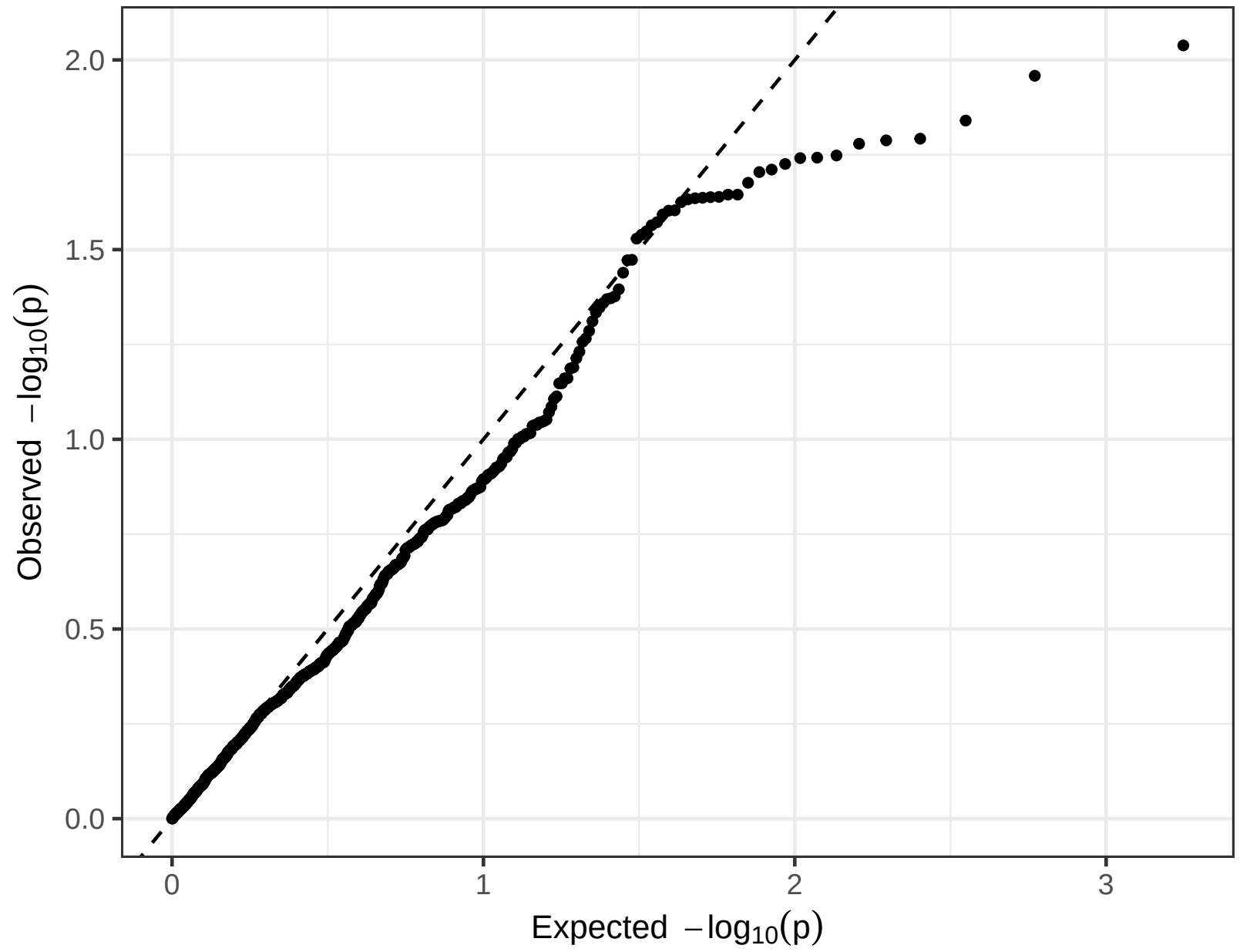

Mogibacterium

Feces | Muscle | . = 0.9

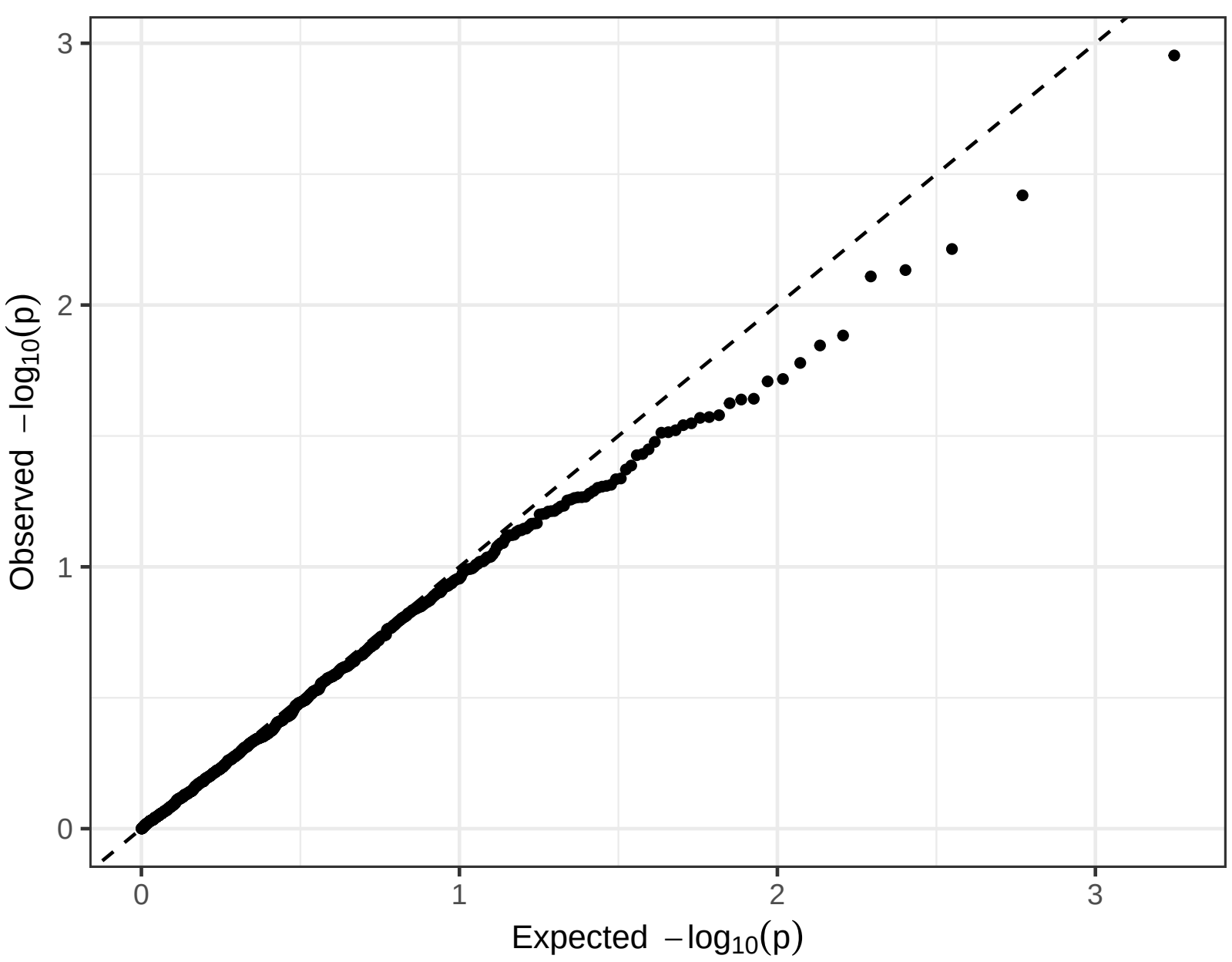

Monoglobus

Feces | Muscle | . = 0.874

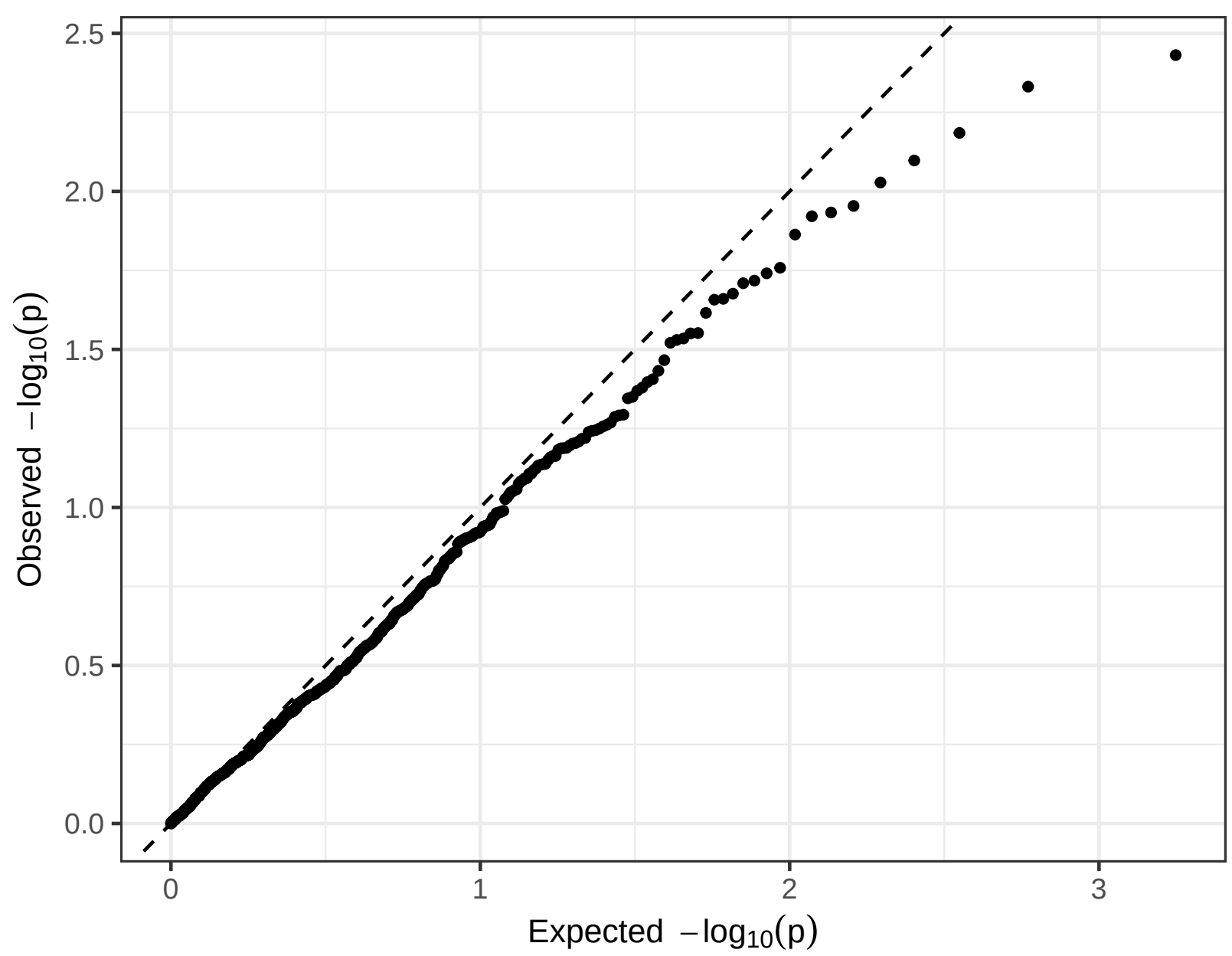

Moryella

Feces | Muscle | . = 0.92

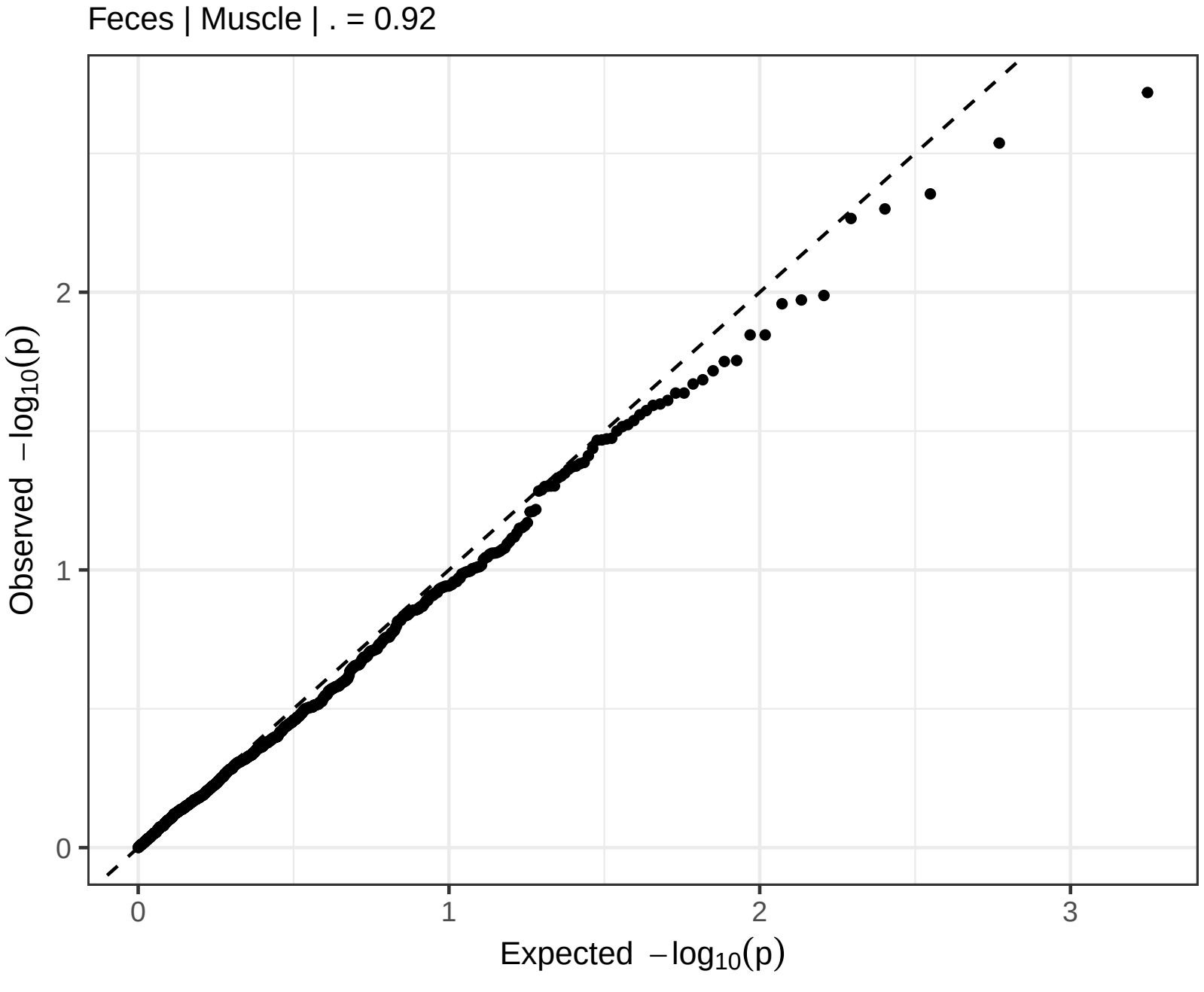

Negativibacillus

Feces | Muscle | . = 0.981

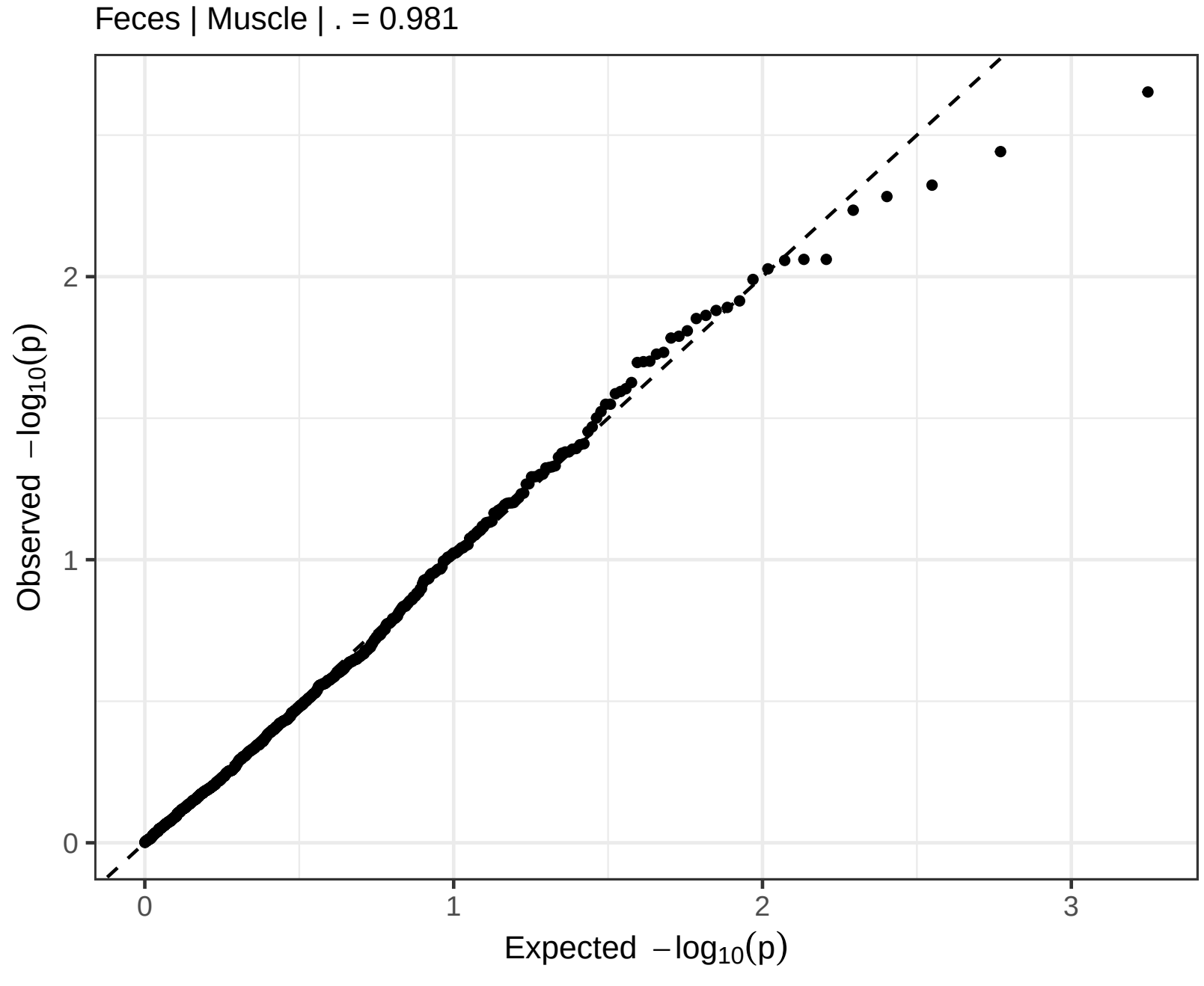

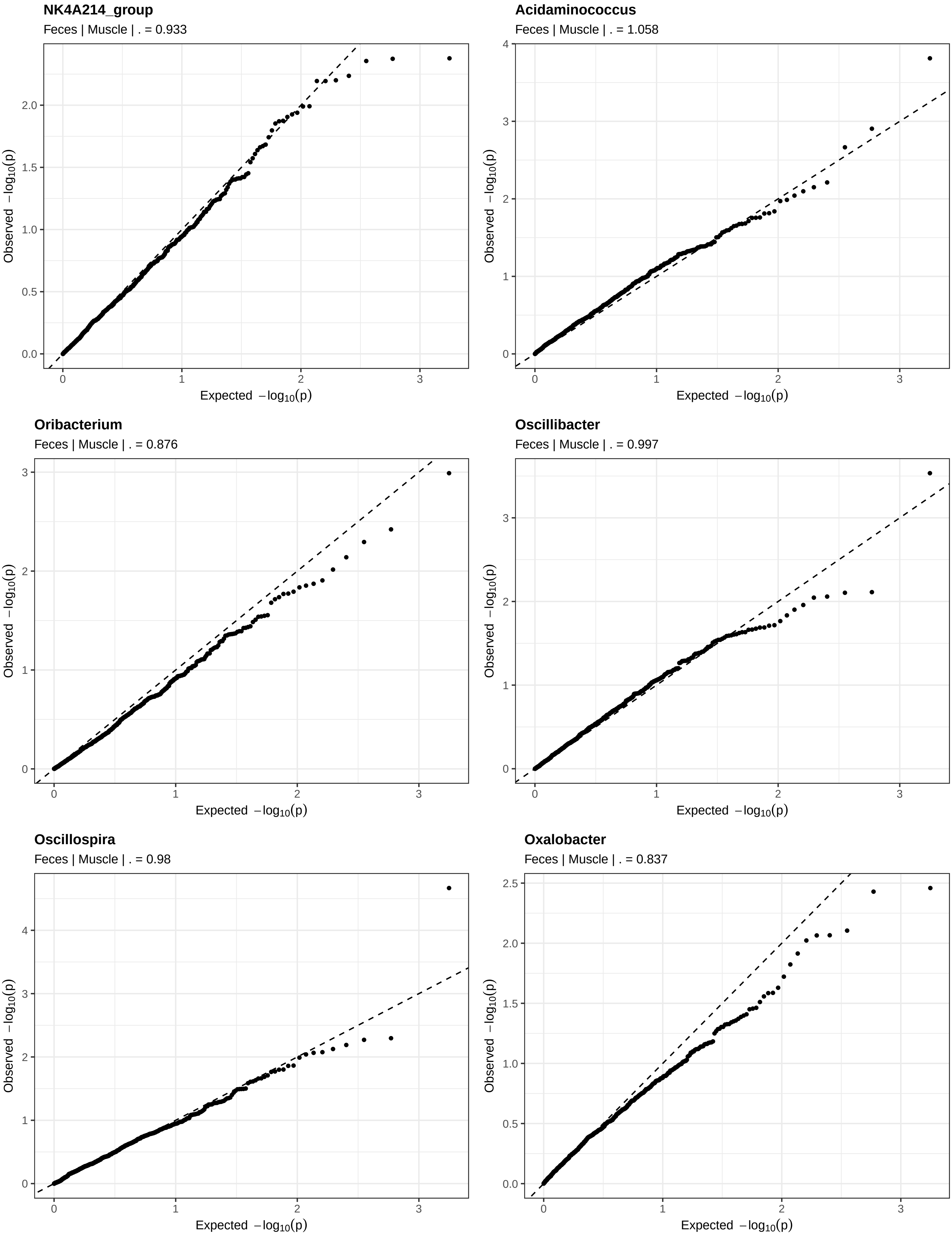
NK4A214_group
Feces | Muscle | . = 0.933
Acidaminococcus
Feces | Muscle | . = 1.058
Oribacterium
Feces | Muscle | . = 0.876
Oscillibacter
Feces | Muscle | . = 0.997
Oscillospira
Feces | Muscle | . = 0.98
Oxalobacter
Feces | Muscle | . = 0.837
Observed $-\log_{10}(p)$
Expected $-\log_{10}(p)$

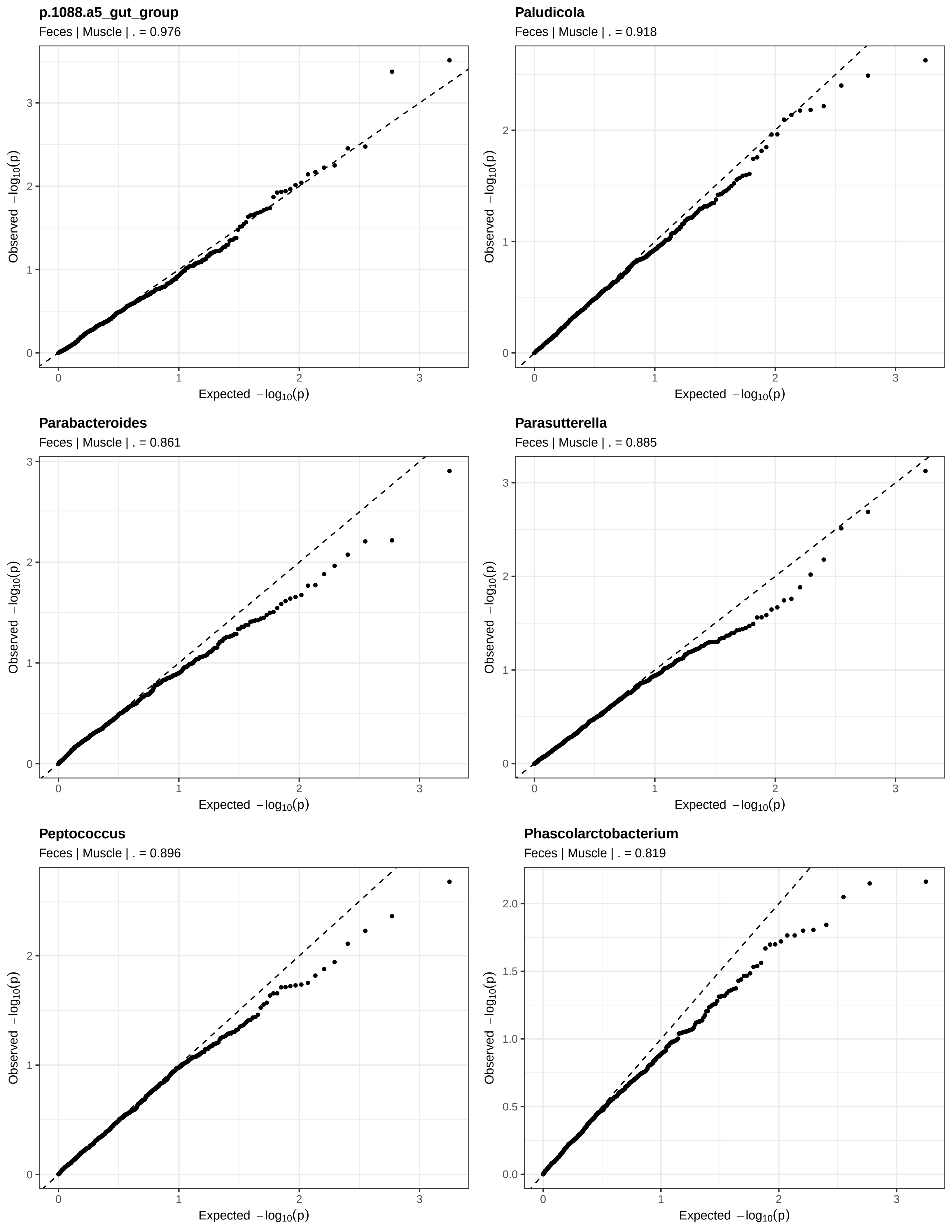

p.1088.a5_gut_group
Feces | Muscle | . = 0.976
Paludicola
Feces | Muscle | . = 0.918
Parabacteroides
Feces | Muscle | . = 0.861
Parasutterella
Feces | Muscle | . = 0.885
Peptococcus
Feces | Muscle | . = 0.896
Phascolarctobacterium
Feces | Muscle | . = 0.819
Observed $-\log_{10}(p)$
Expected $-\log_{10}(p)$
