# Supplementary material for: Associations of host gene expression in the brain, liver, muscle with fecal and cecal microbiota composition in pigs
Source: Funct Integr Genomics. 2026 Aug 15;26(1):226. doi: 10.1007/s10142-026-02009-5 (PMC13477493; doi:10.1007/s10142-026-02009-5)

Eubacterium_eligens_group

Cecum | Muscle | . = 1.161

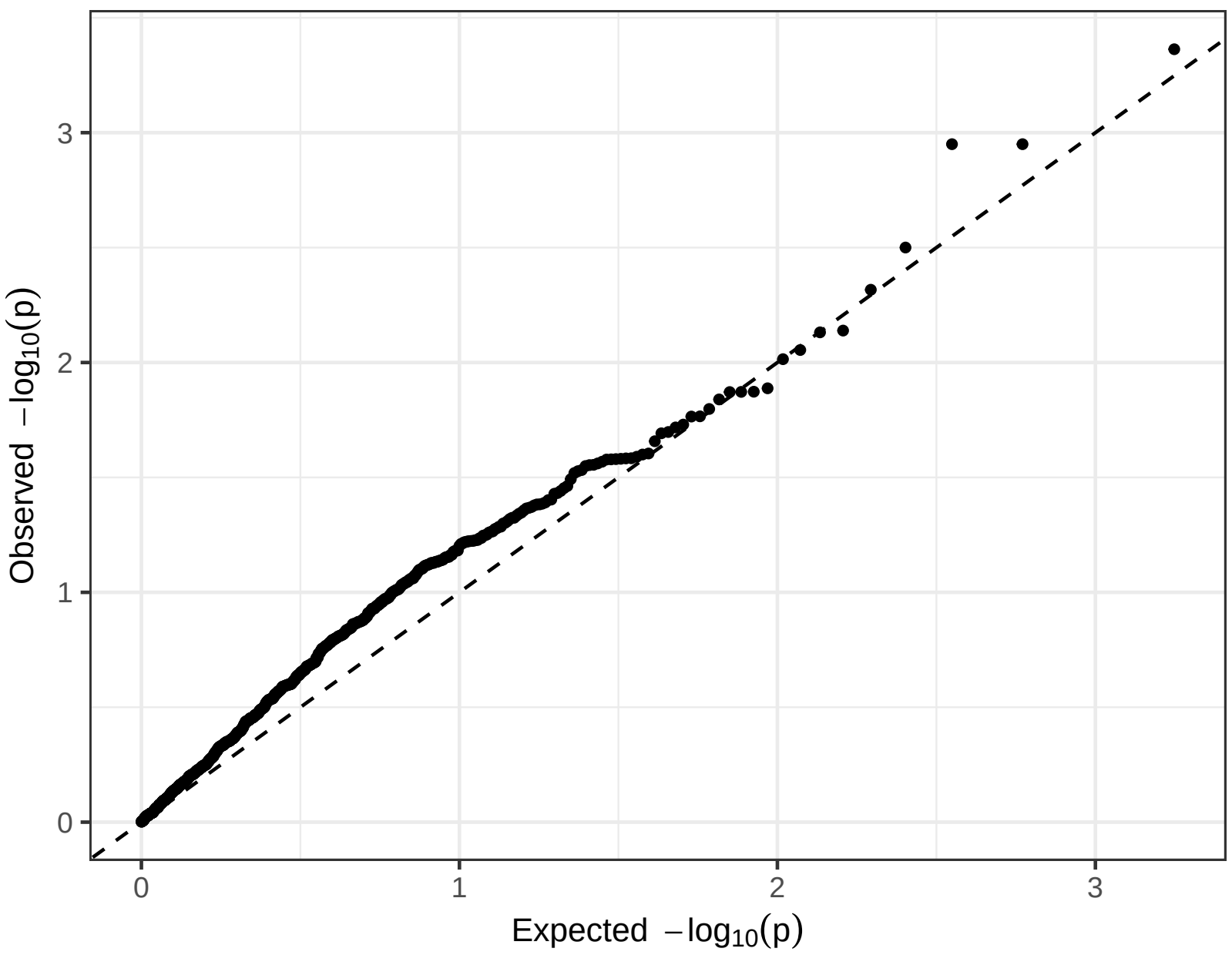

Adlercreutzia

Cecum | Muscle | . = 0.944

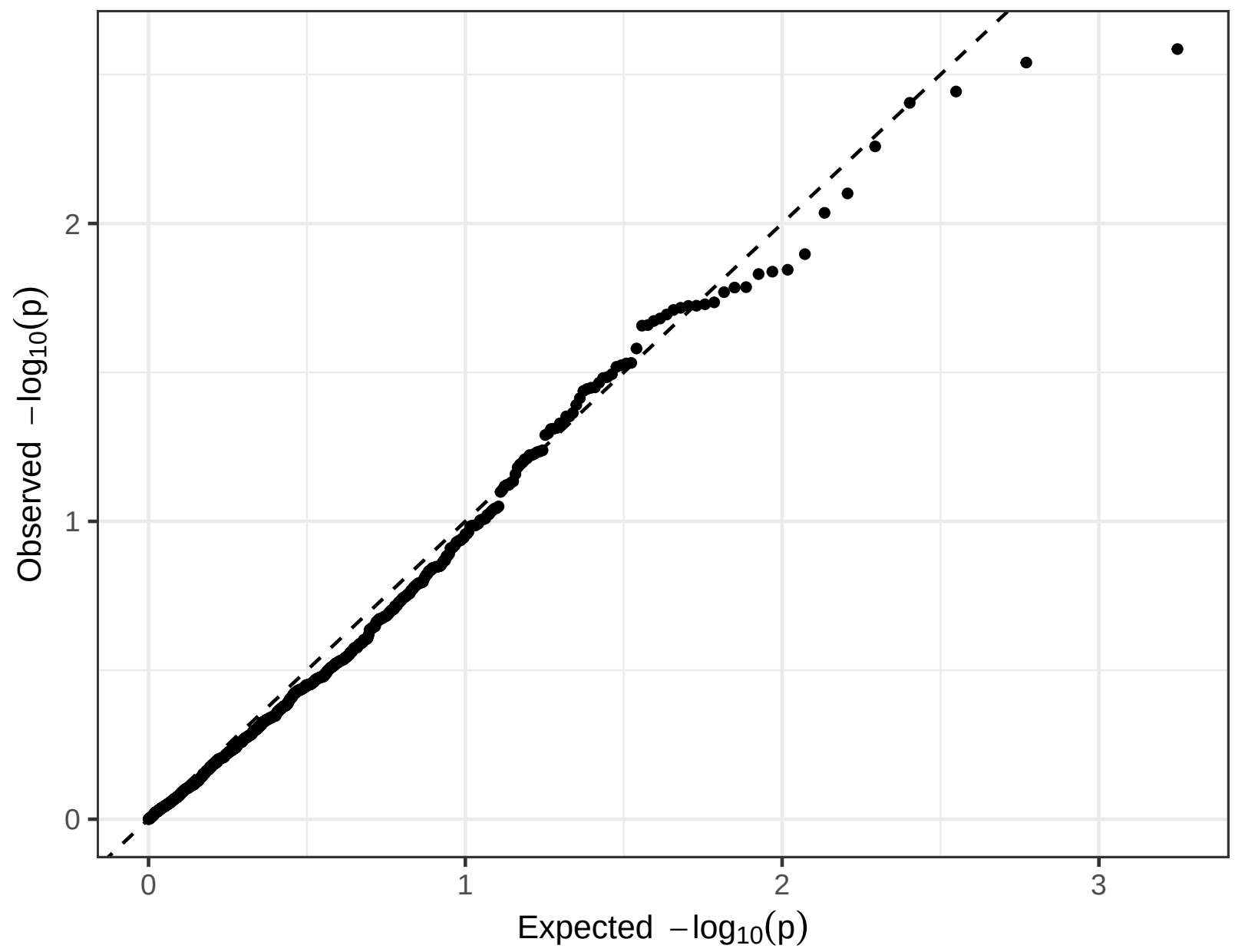

Megasphaera

Cecum | Muscle | . = 0.869

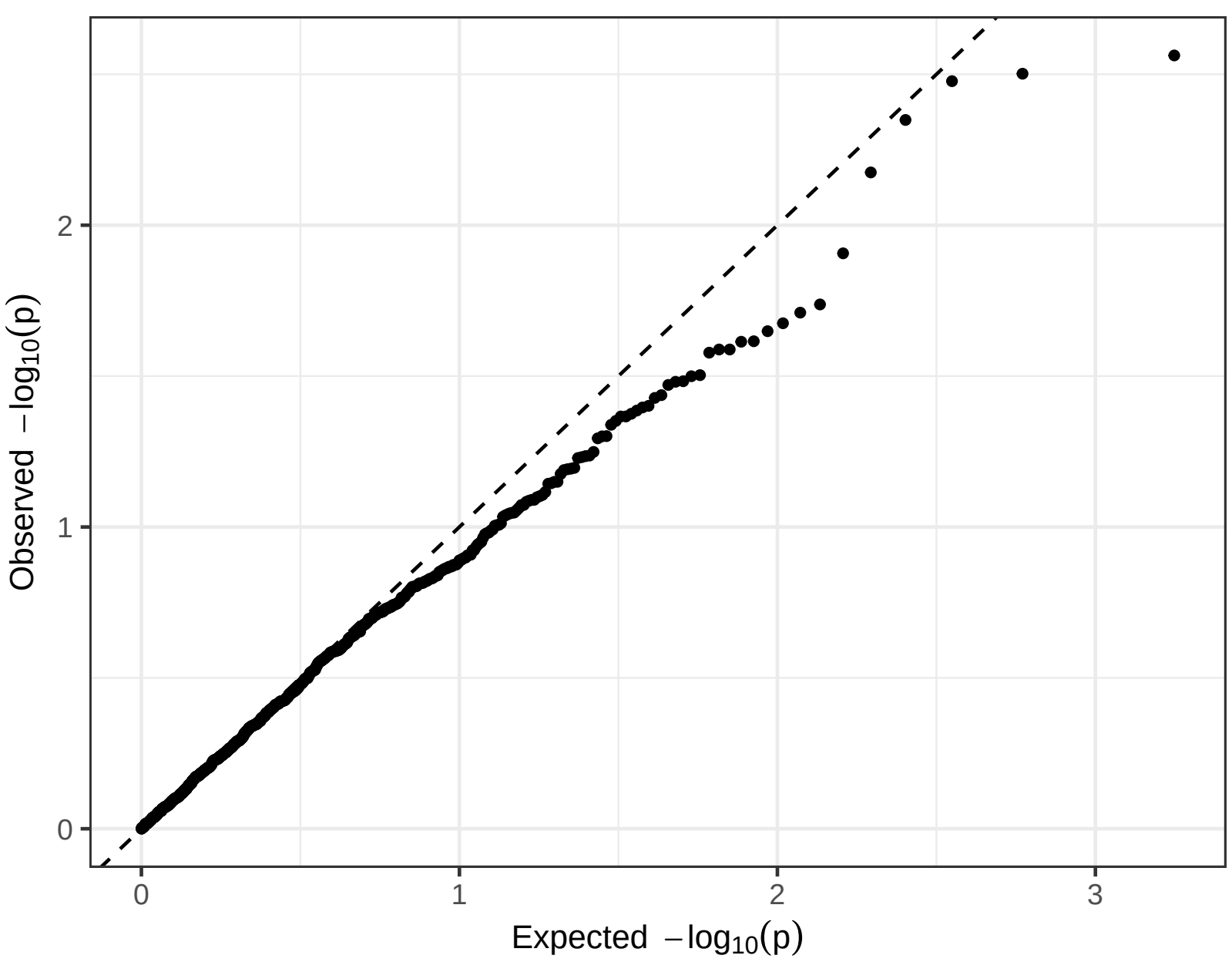

Metamycoplasma

Cecum | Muscle | . = 0.852

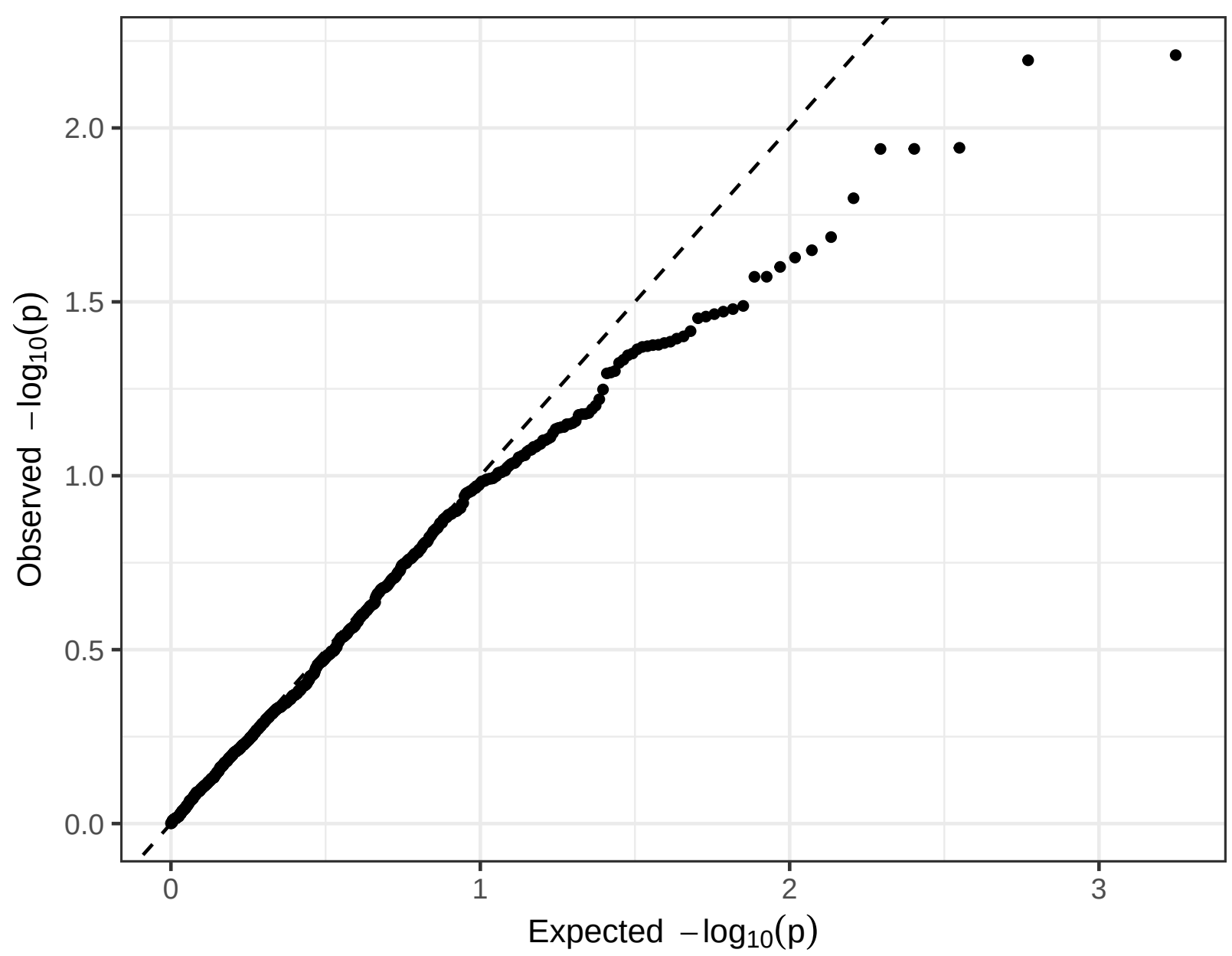

Methanobrevibacter

Cecum | Muscle | . = 0.994

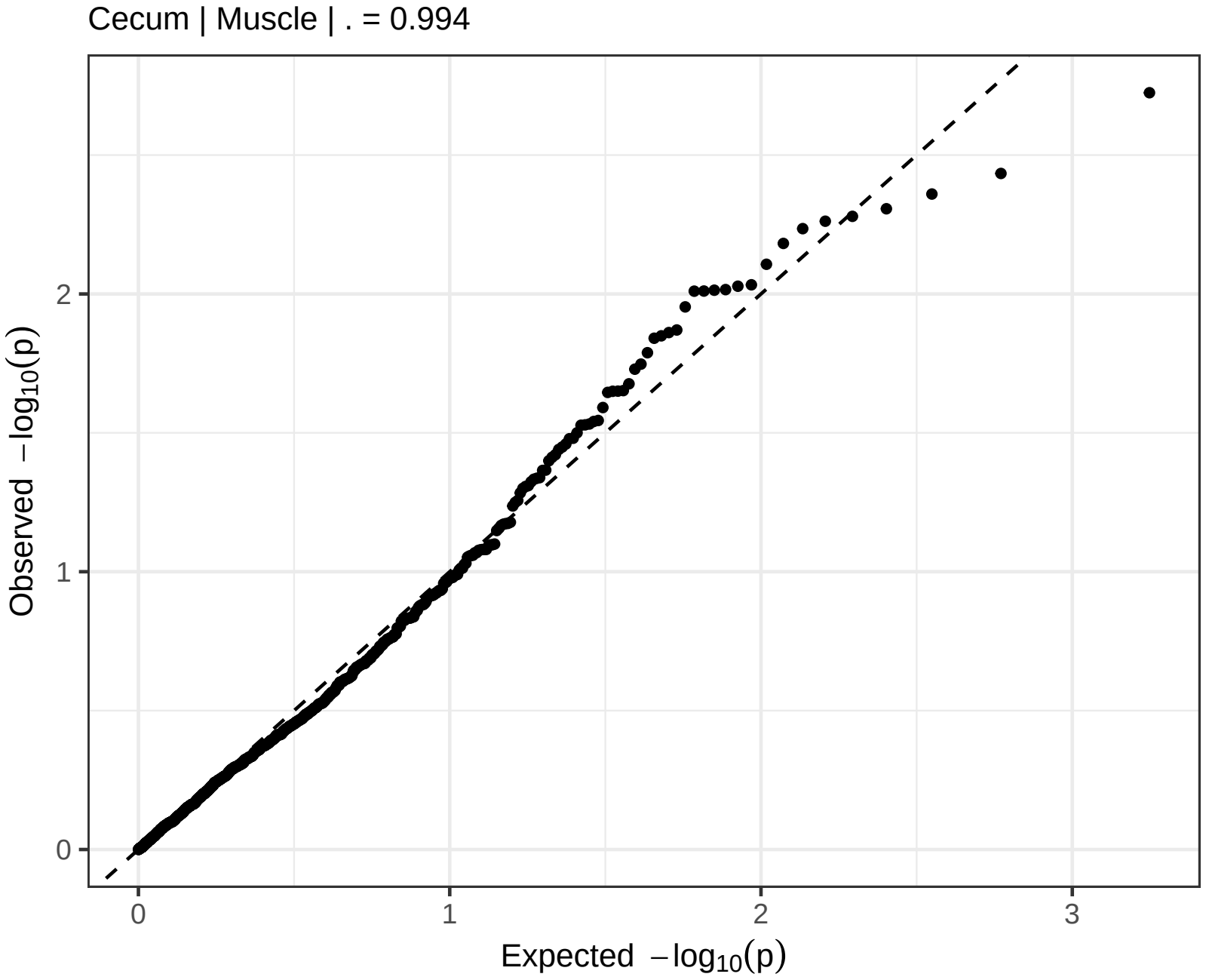

Mogibacterium

Cecum | Muscle | . = 0.943

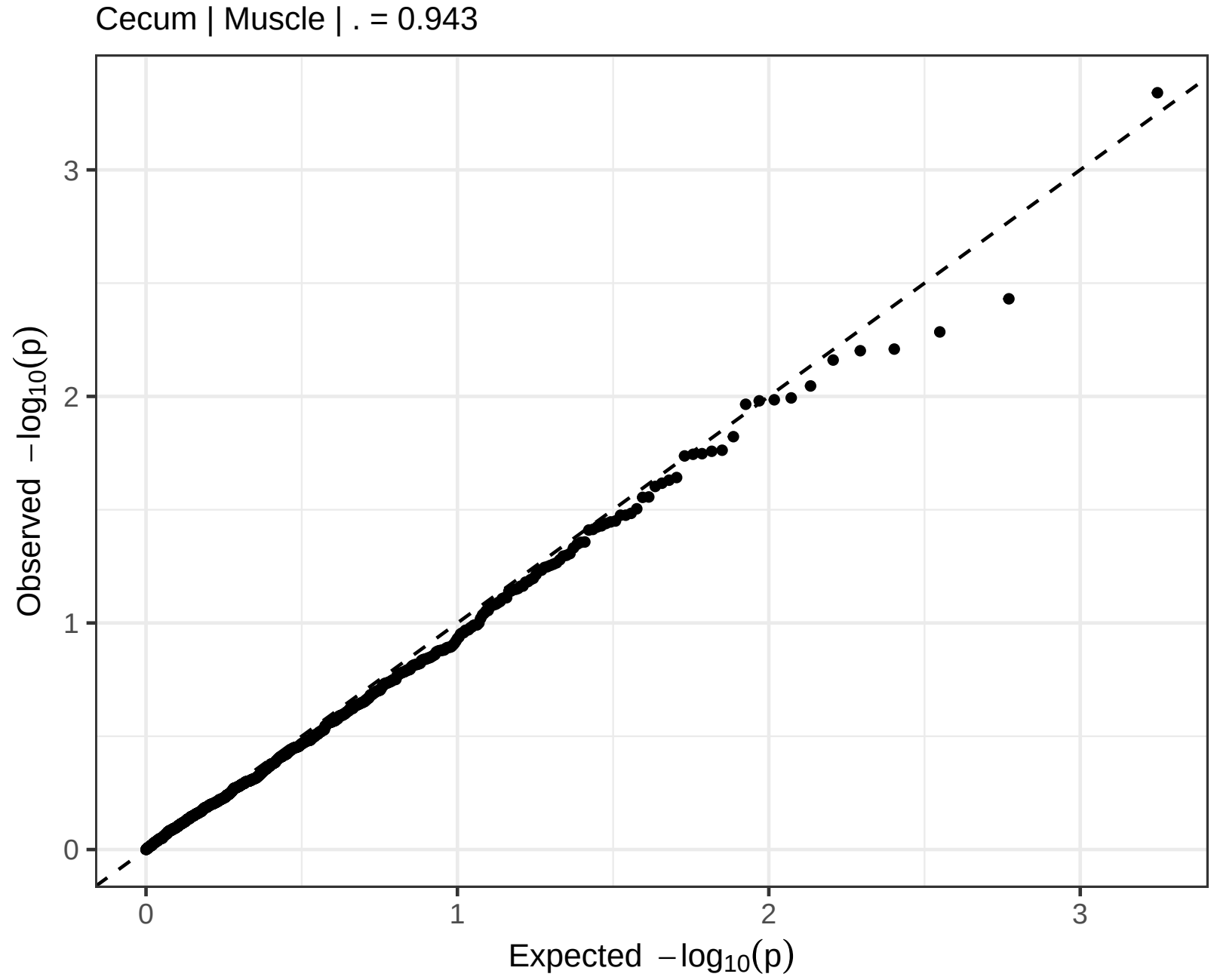

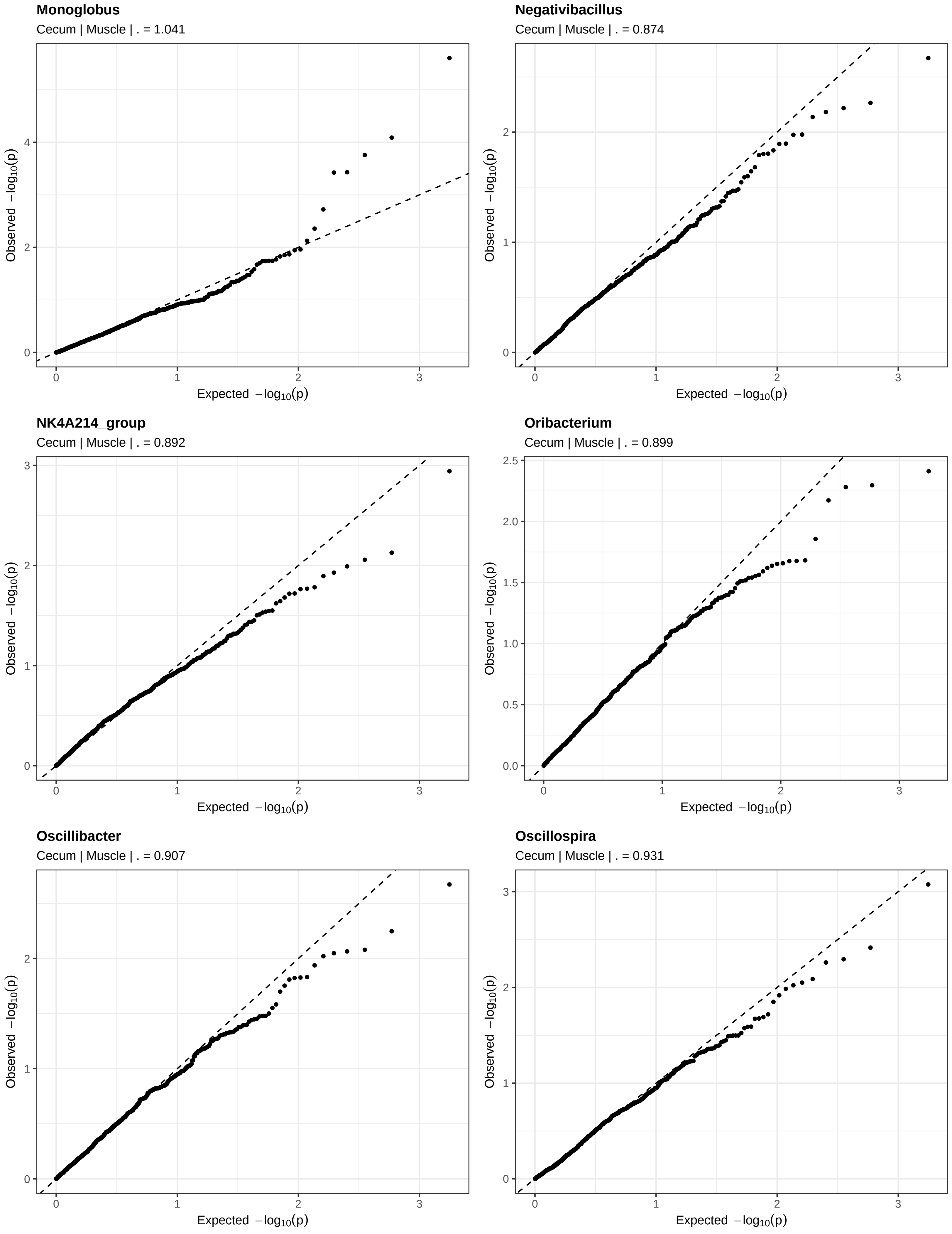
Monoglobus
Cecum | Muscle | . = 1.041
Negativibacillus
Cecum | Muscle | . = 0.874
NK4A214_group
Cecum | Muscle | . = 0.892
Oribacterium
Cecum | Muscle | . = 0.899
Oscillibacter
Cecum | Muscle | . = 0.907
Oscillospira
Cecum | Muscle | . = 0.931
Observed $-\log_{10}(p)$
Expected $-\log_{10}(p)$

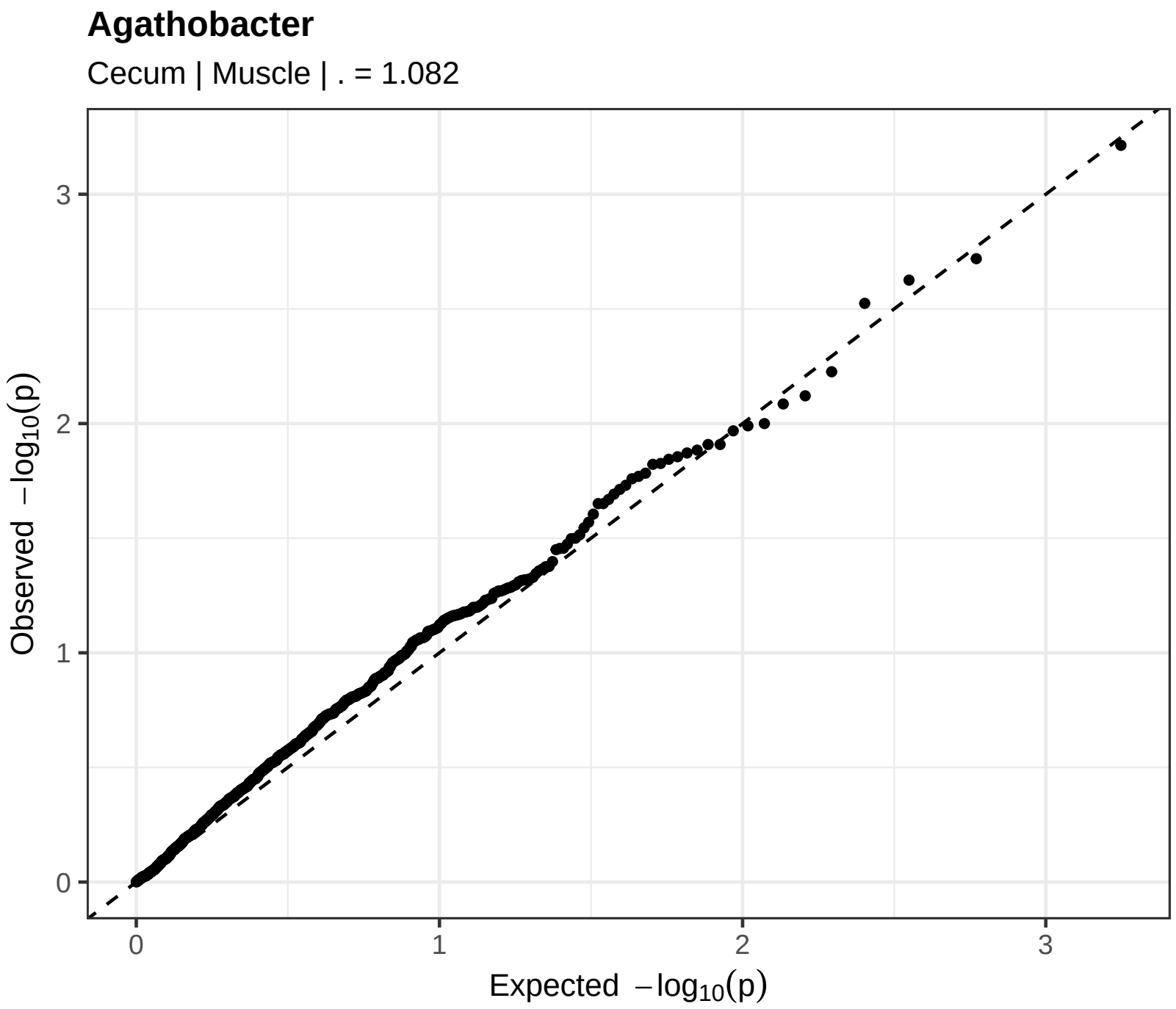
Agathobacter
Cecum | Muscle | . = 1.082
Observed −log10(p)
Expected −log10(p)

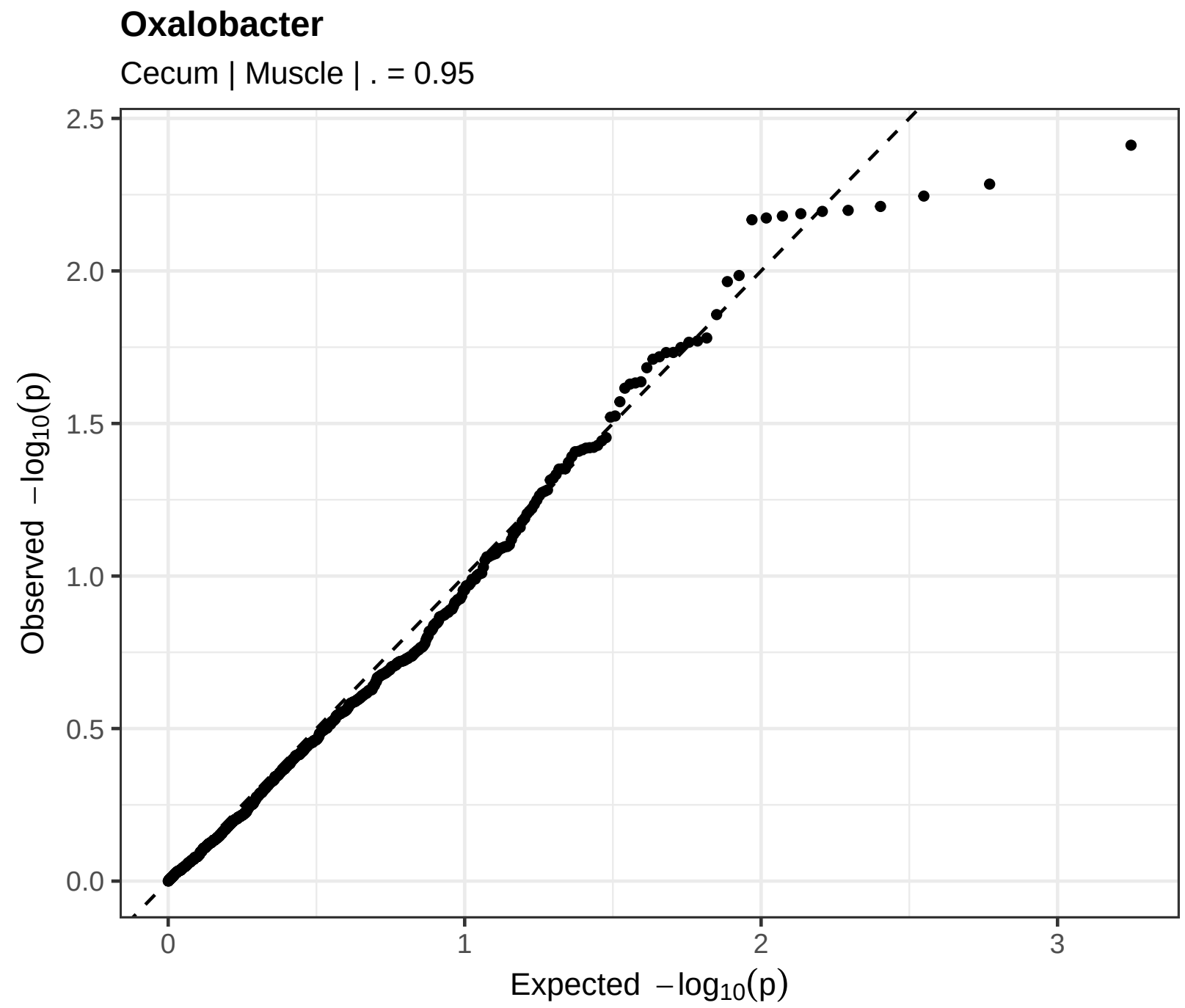
Oxalobacter
Cecum | Muscle | . = 0.95
Observed −log10(p)
Expected −log10(p)

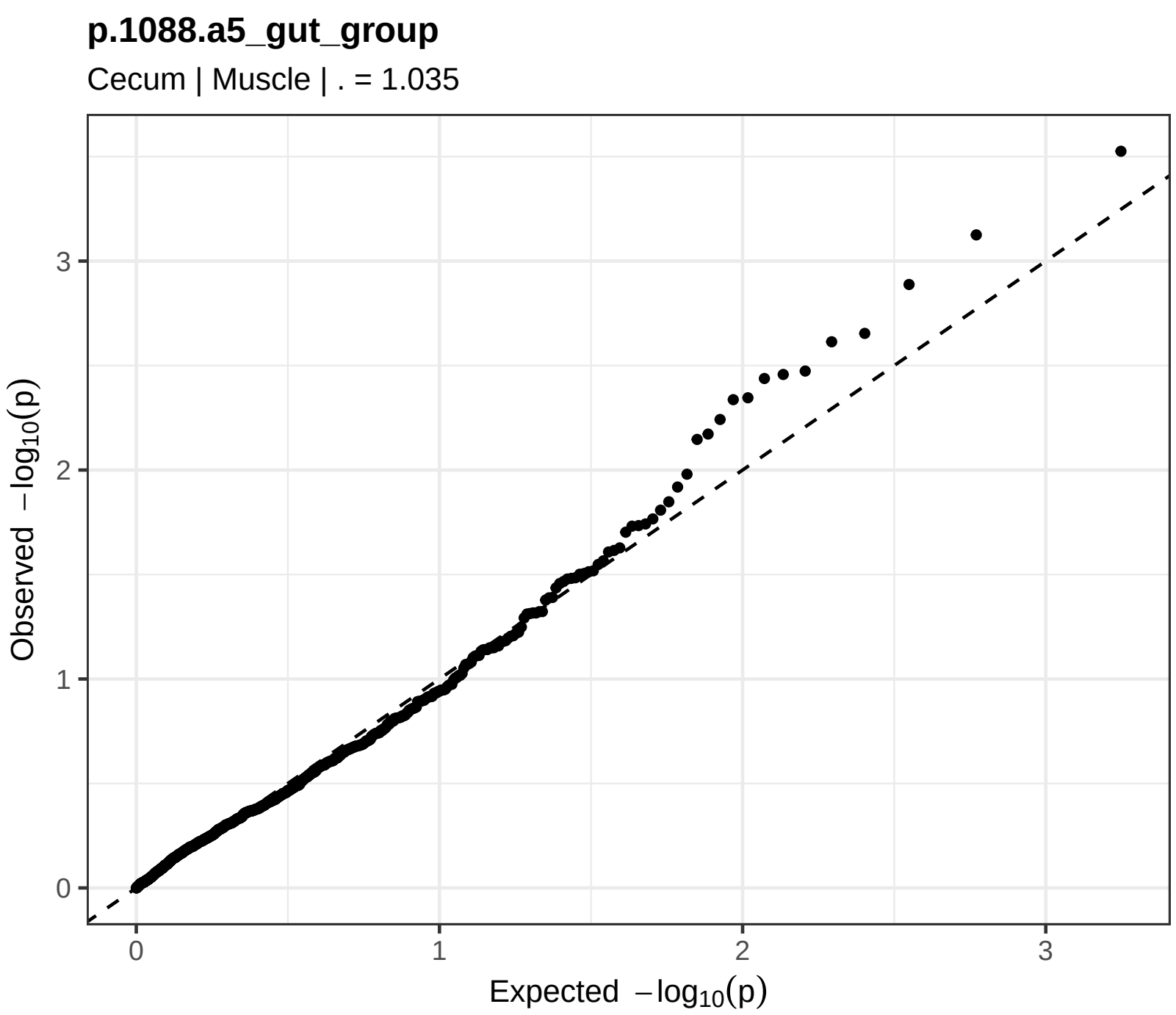
p.1088.a5_gut_group
Cecum | Muscle | . = 1.035
Observed −log10(p)
Expected −log10(p)

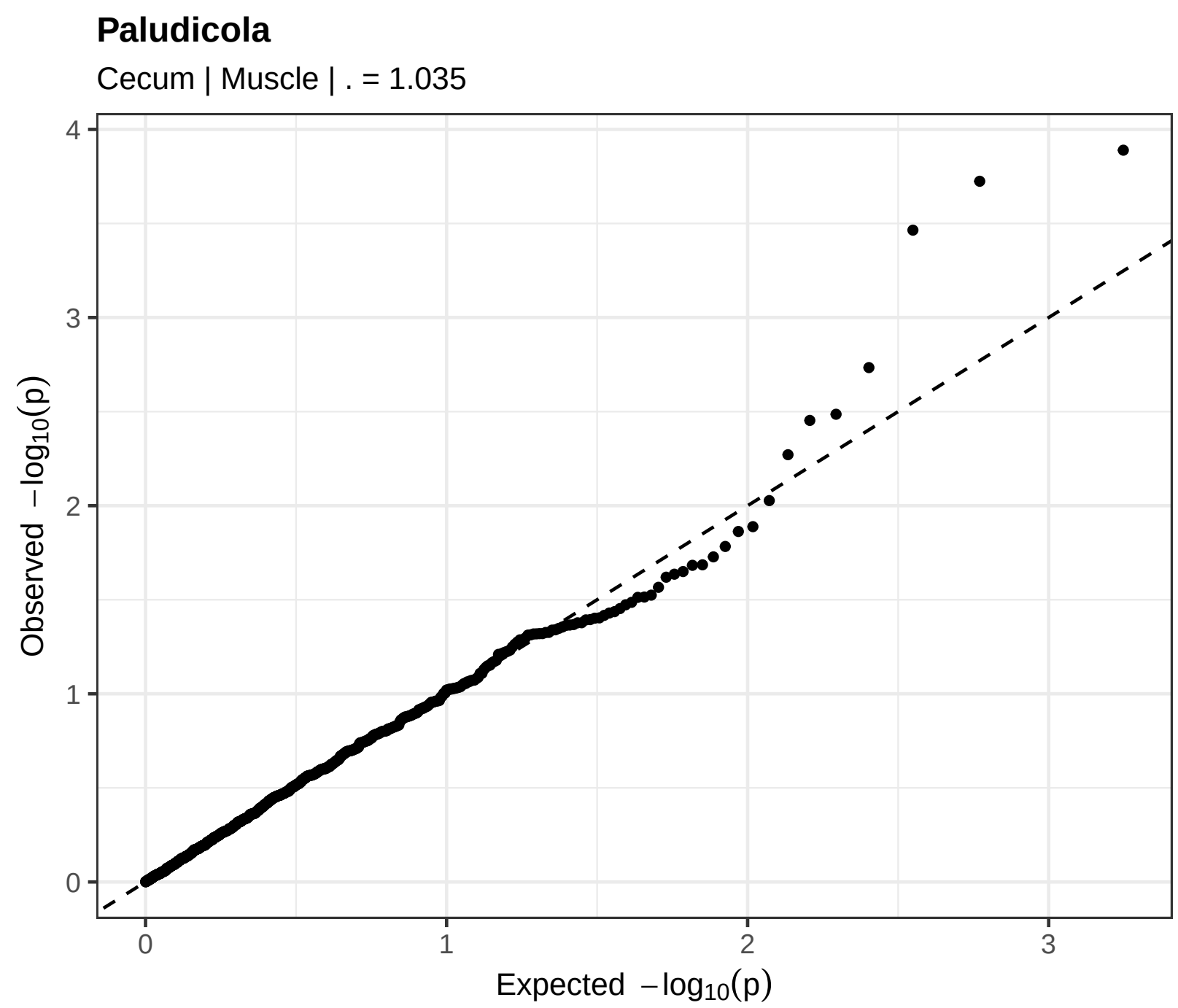
Paludicola
Cecum | Muscle | . = 1.035
Observed −log10(p)
Expected −log10(p)

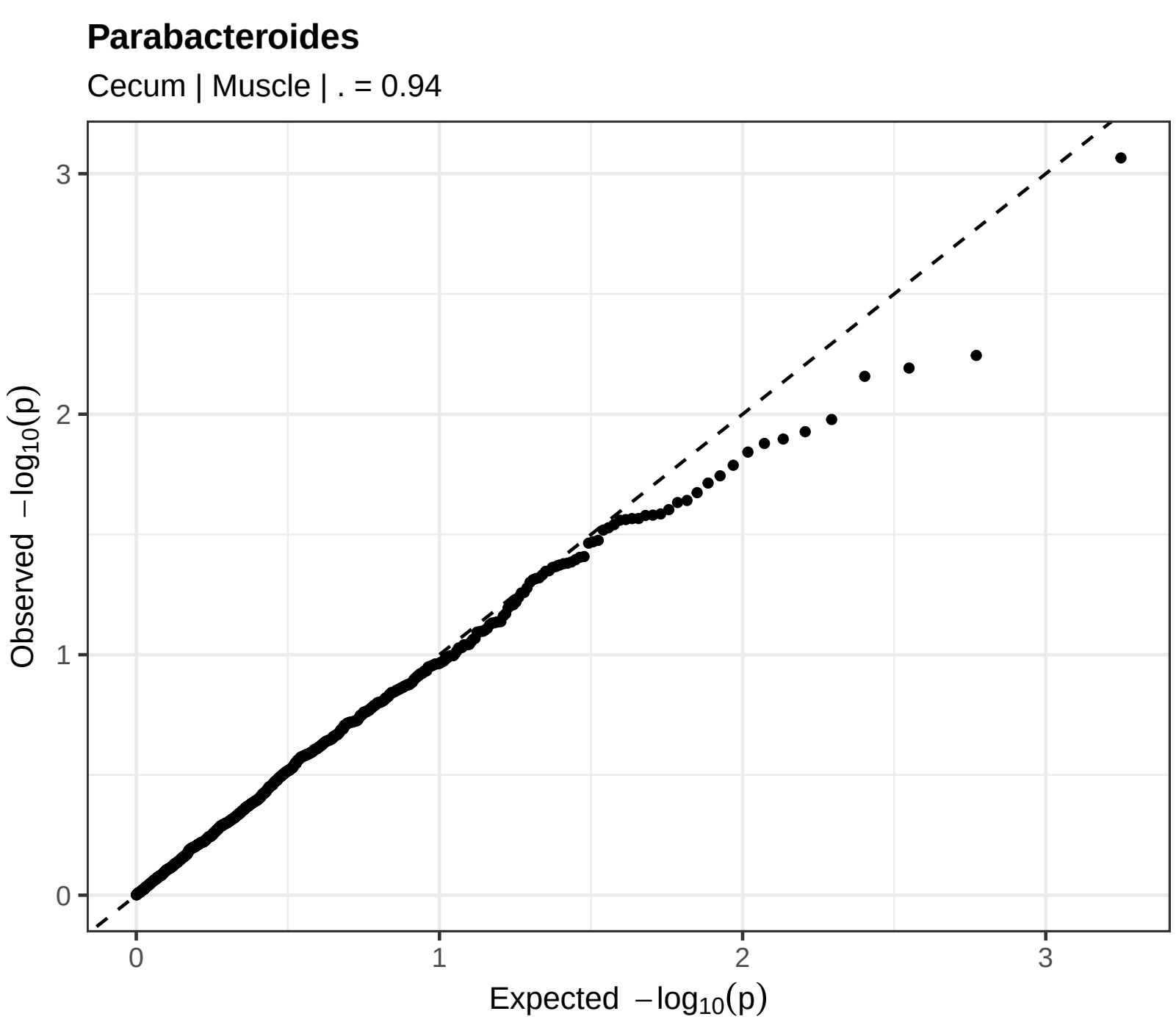
Parabacteroides
Cecum | Muscle | . = 0.94
Observed −log10(p)
Expected −log10(p)

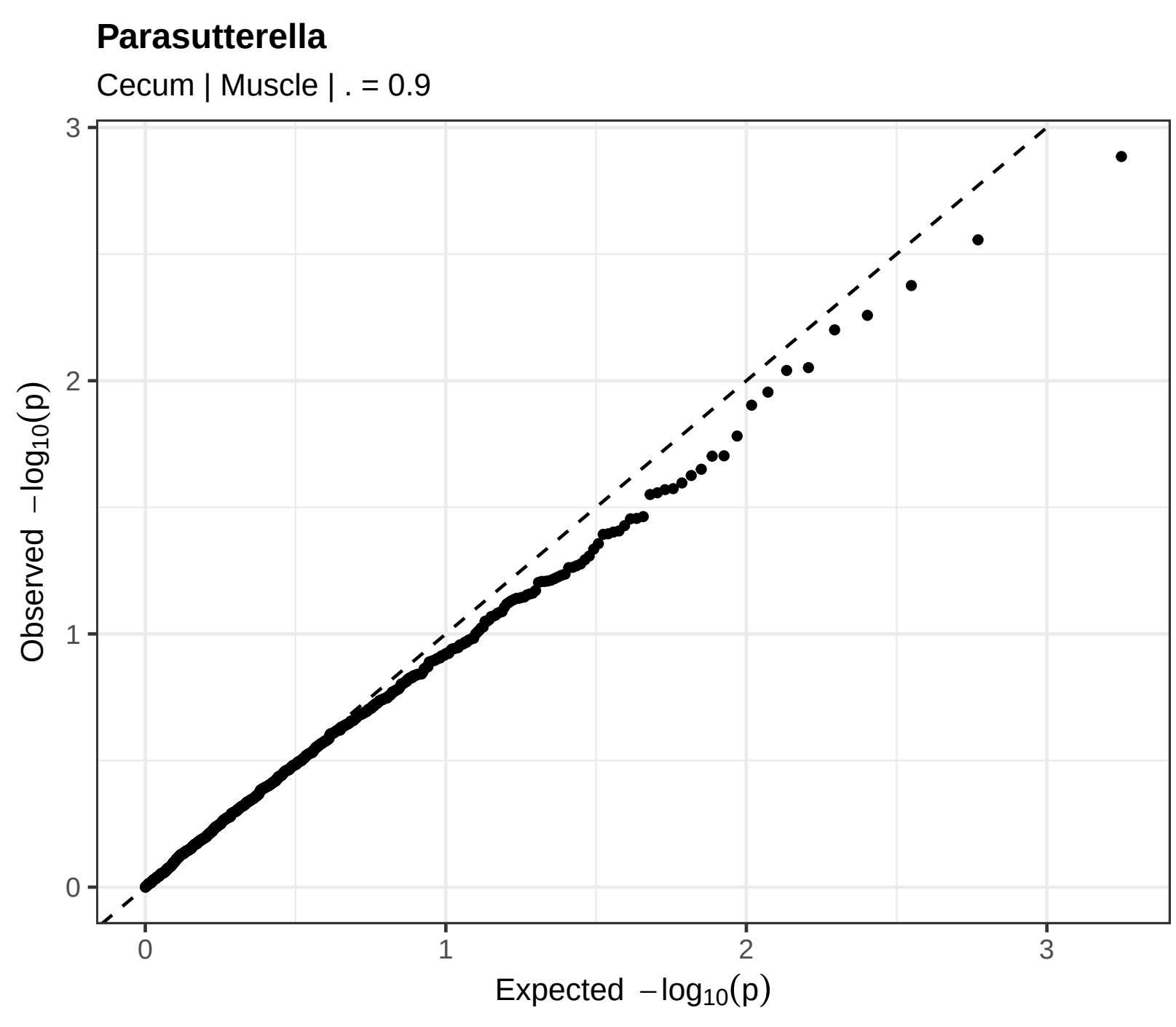
Parasutterella
Cecum | Muscle | . = 0.9
Observed −log10(p)
Expected −log10(p)

Peptococcus

Cecum | Muscle | . = 1.055

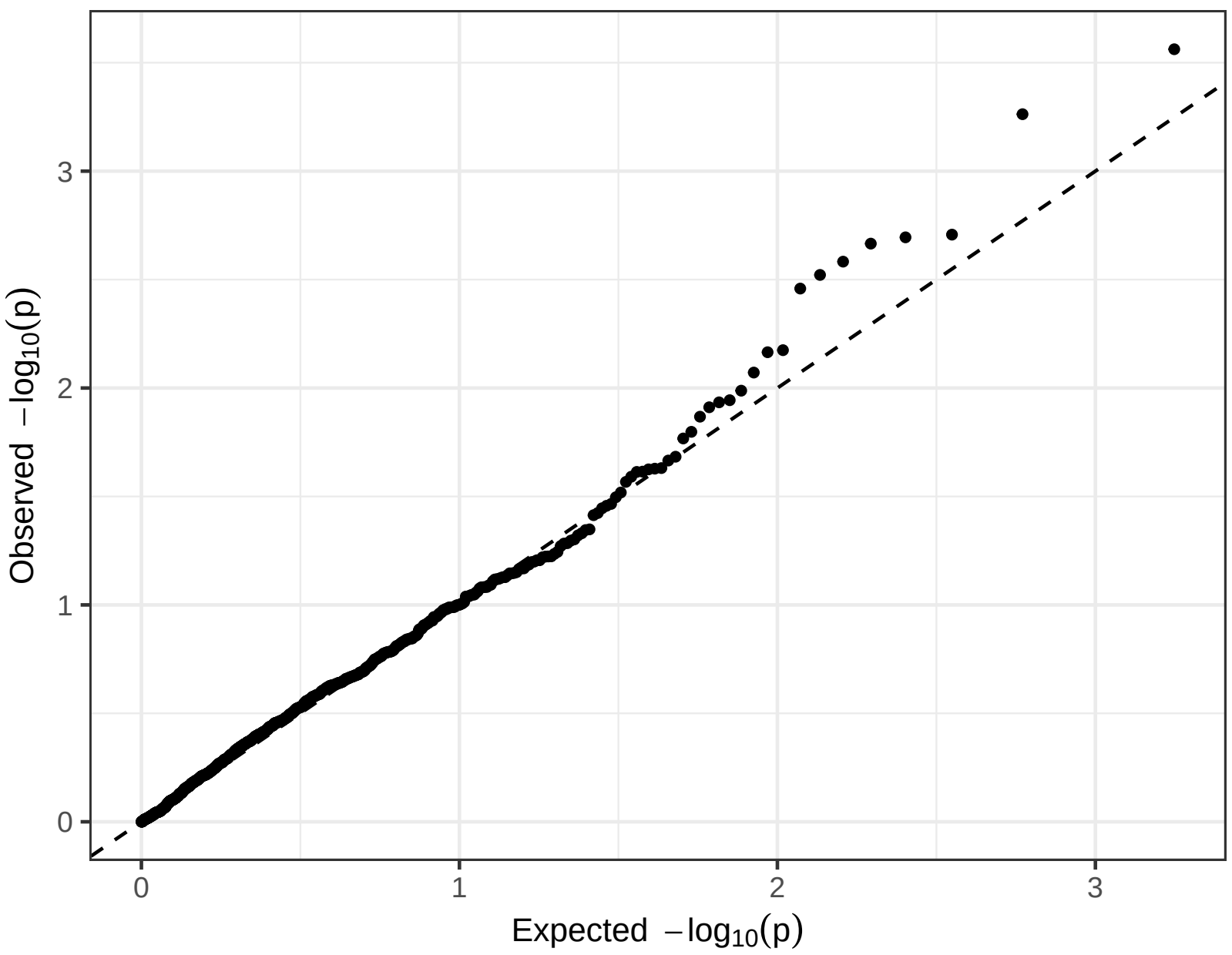

Phascolarctobacterium

Cecum | Muscle | . = 1.074

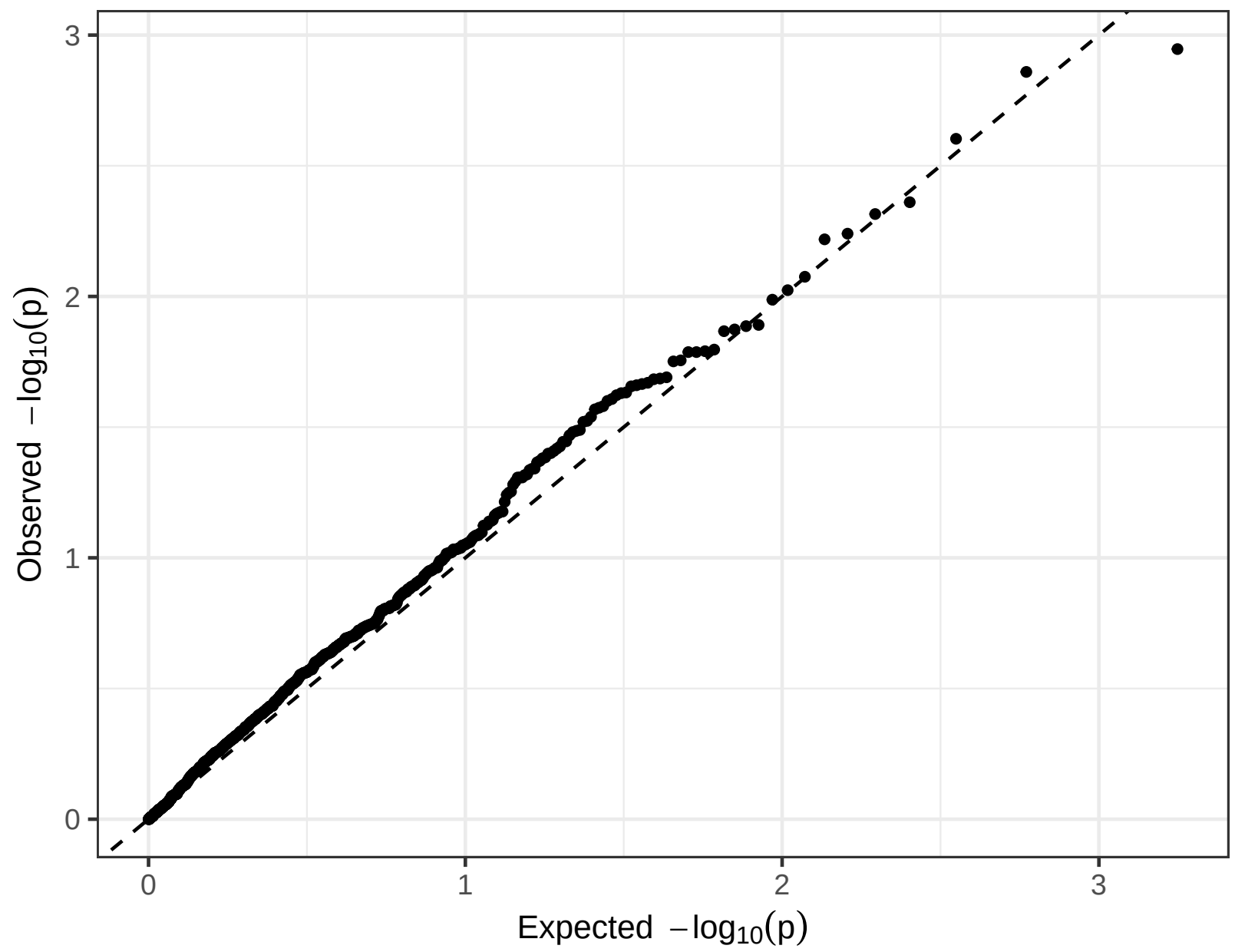

Prevotellaceae_NK3B31_group

Cecum | Muscle | . = 0.894

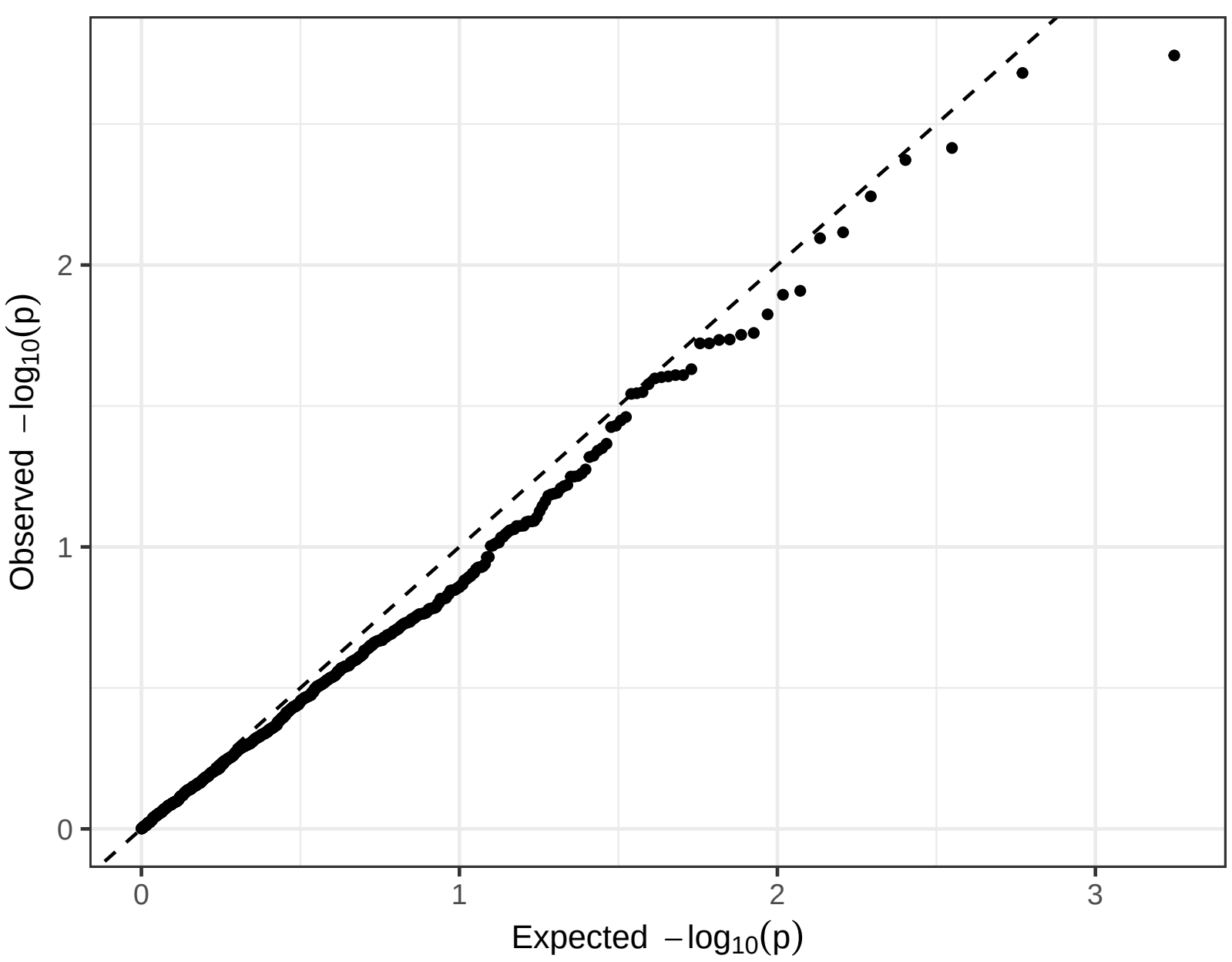

Prevotellaceae_UCG.001

Cecum | Muscle | . = 0.993

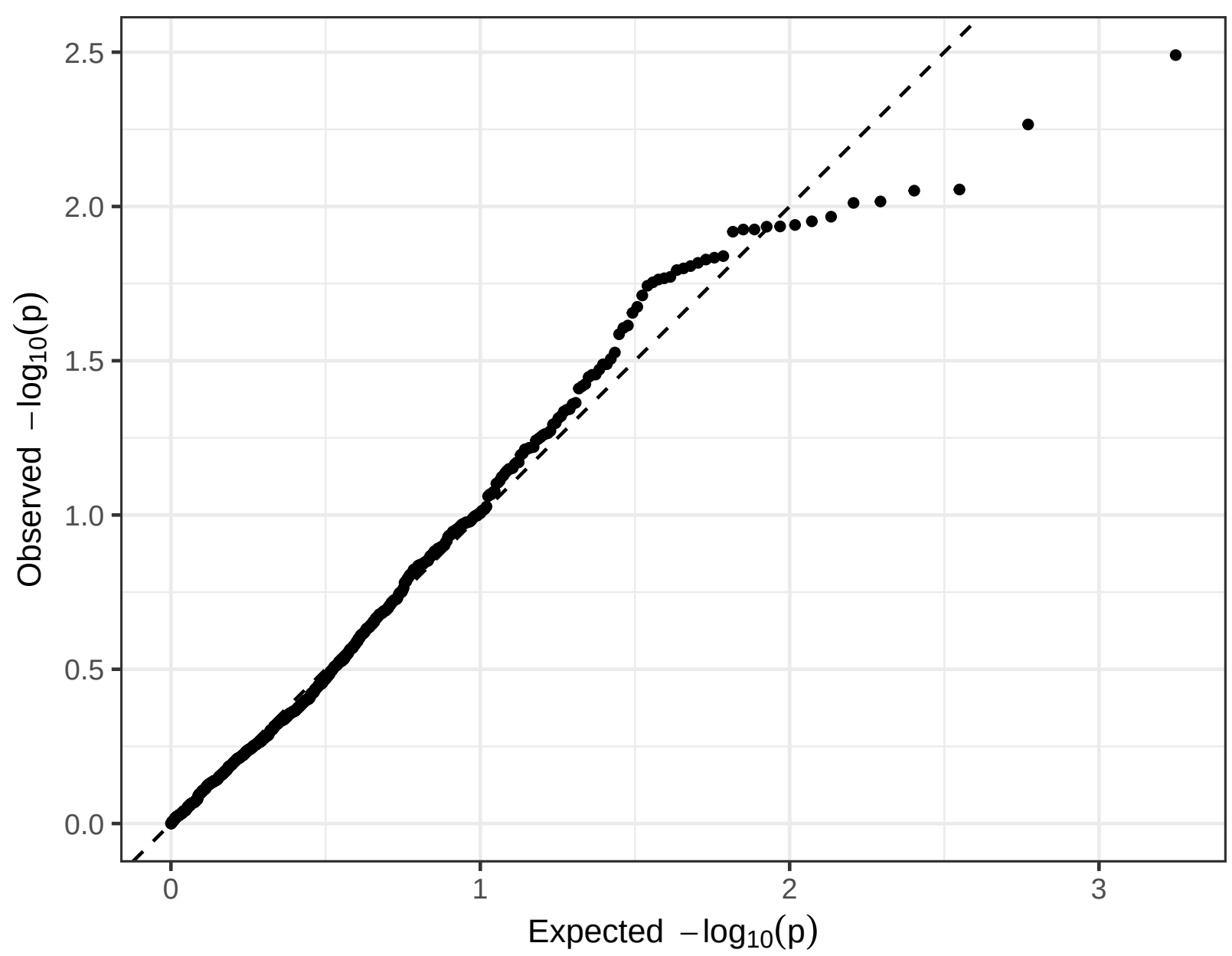

Prevotellaceae_UCG.003

Cecum | Muscle | . = 0.989

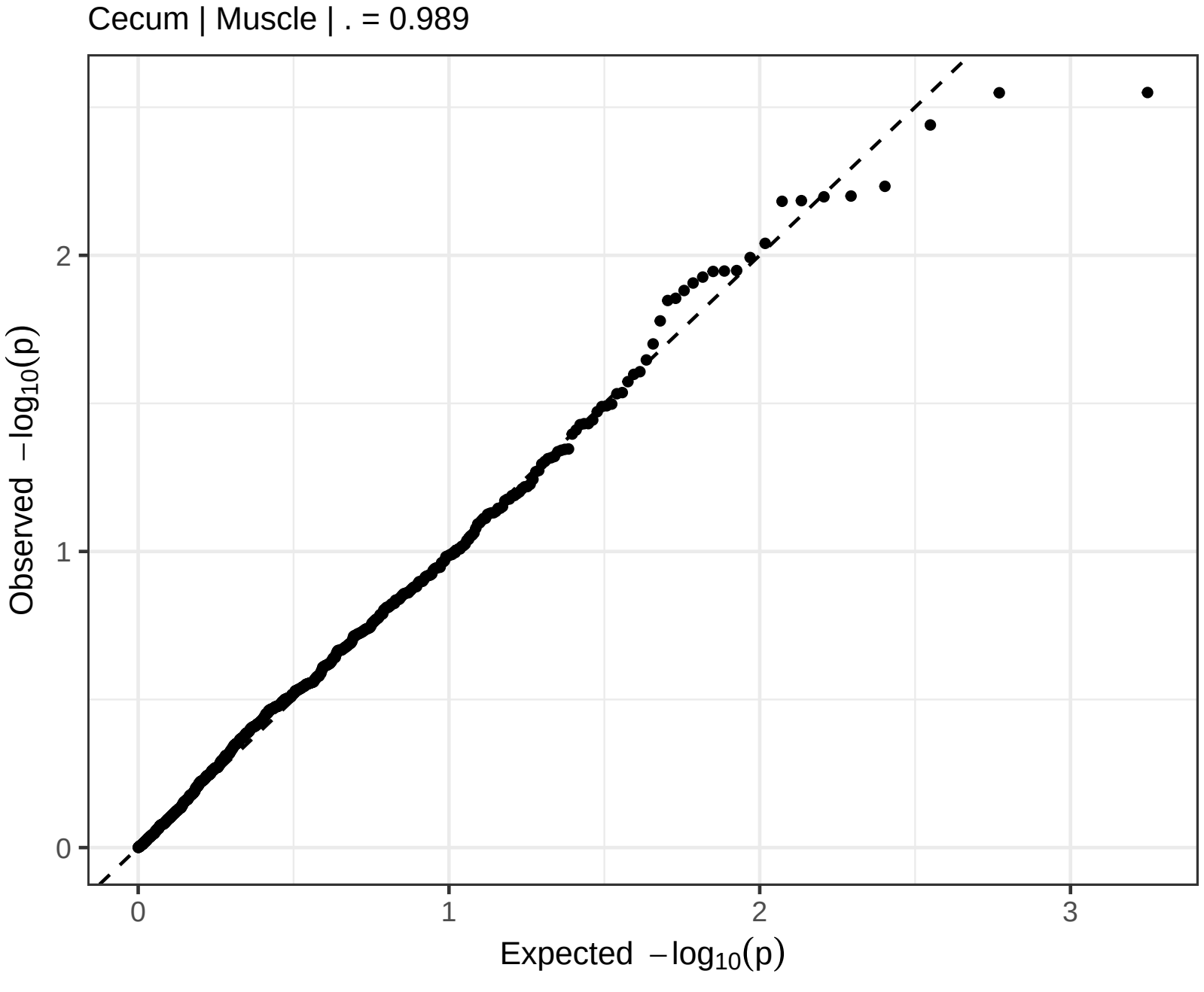

Agathobaculum

Cecum | Muscle | . = 0.917

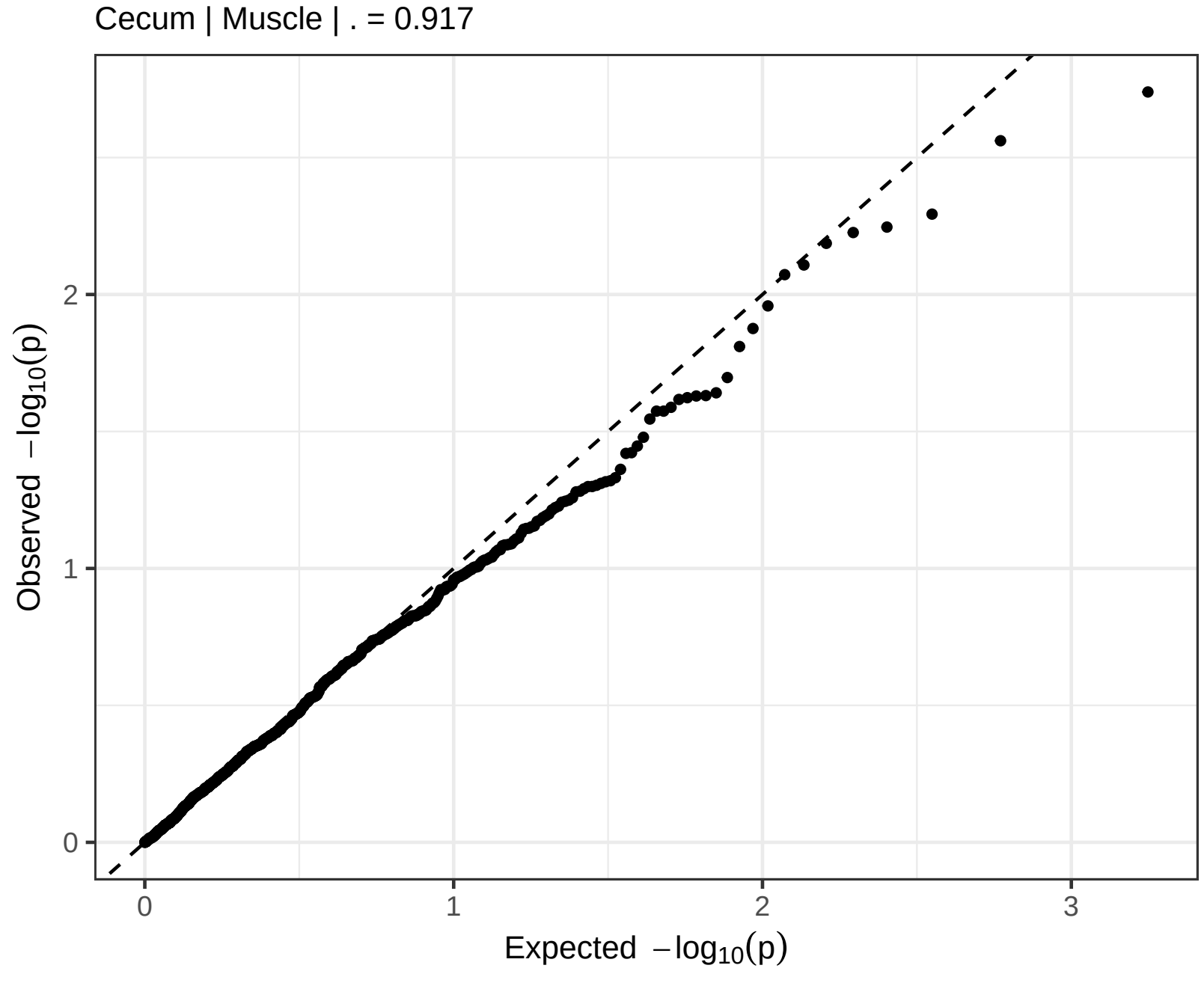

Prevotellaceae_UCG.004

Cecum | Muscle | . = 1.153

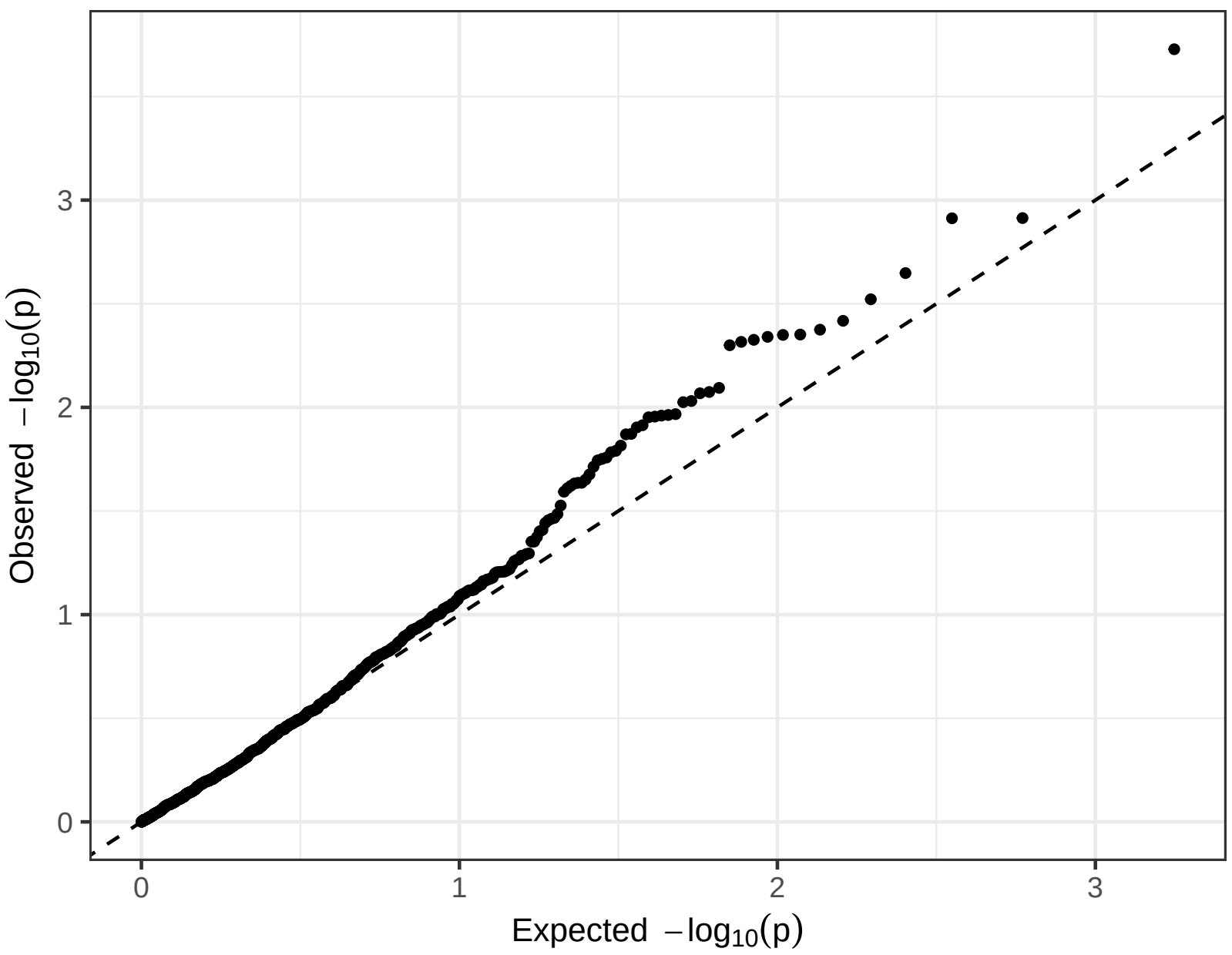

Pygmaiobacter

Cecum | Muscle | . = 0.908

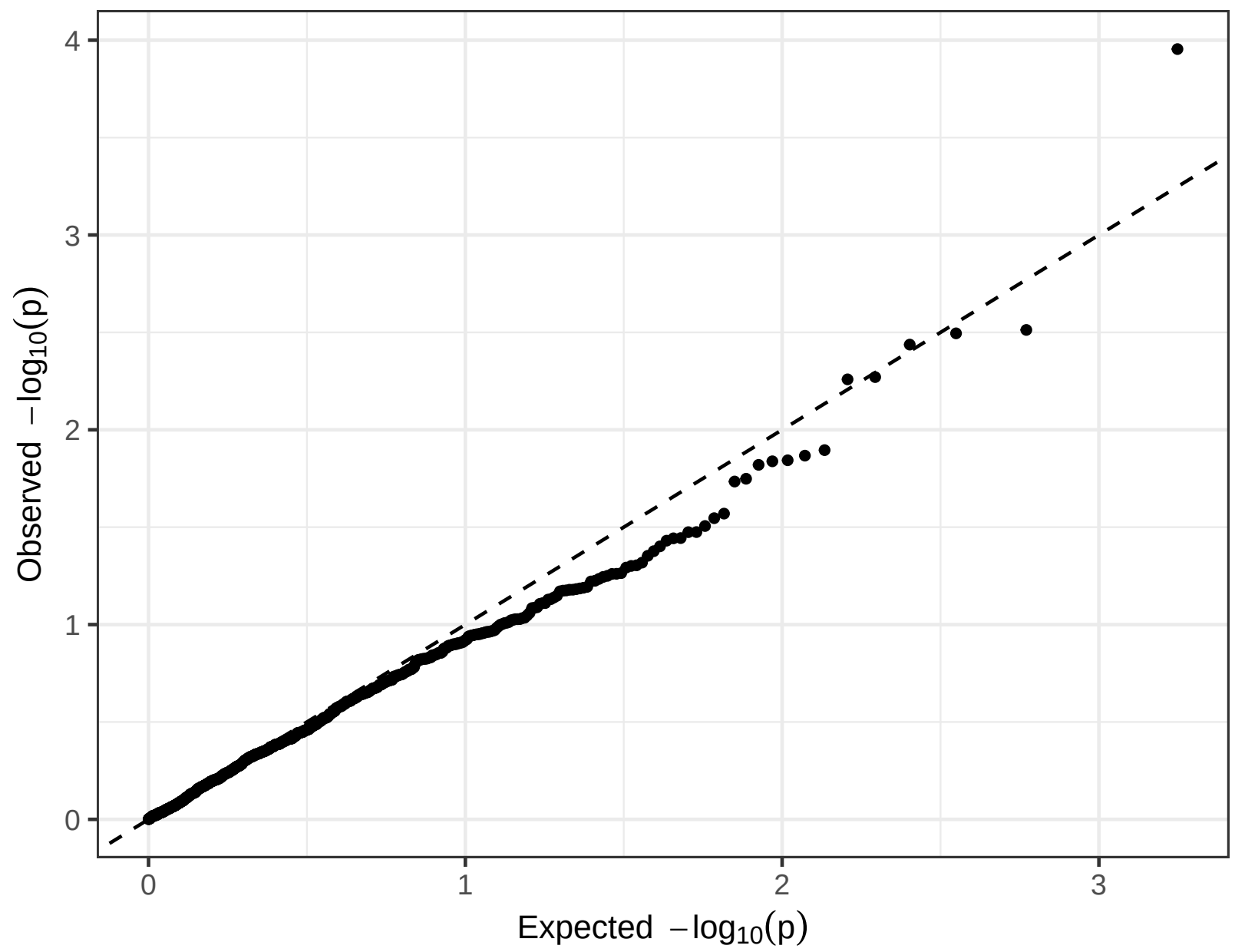

Pyramidobacter

Cecum | Muscle | . = 0.854

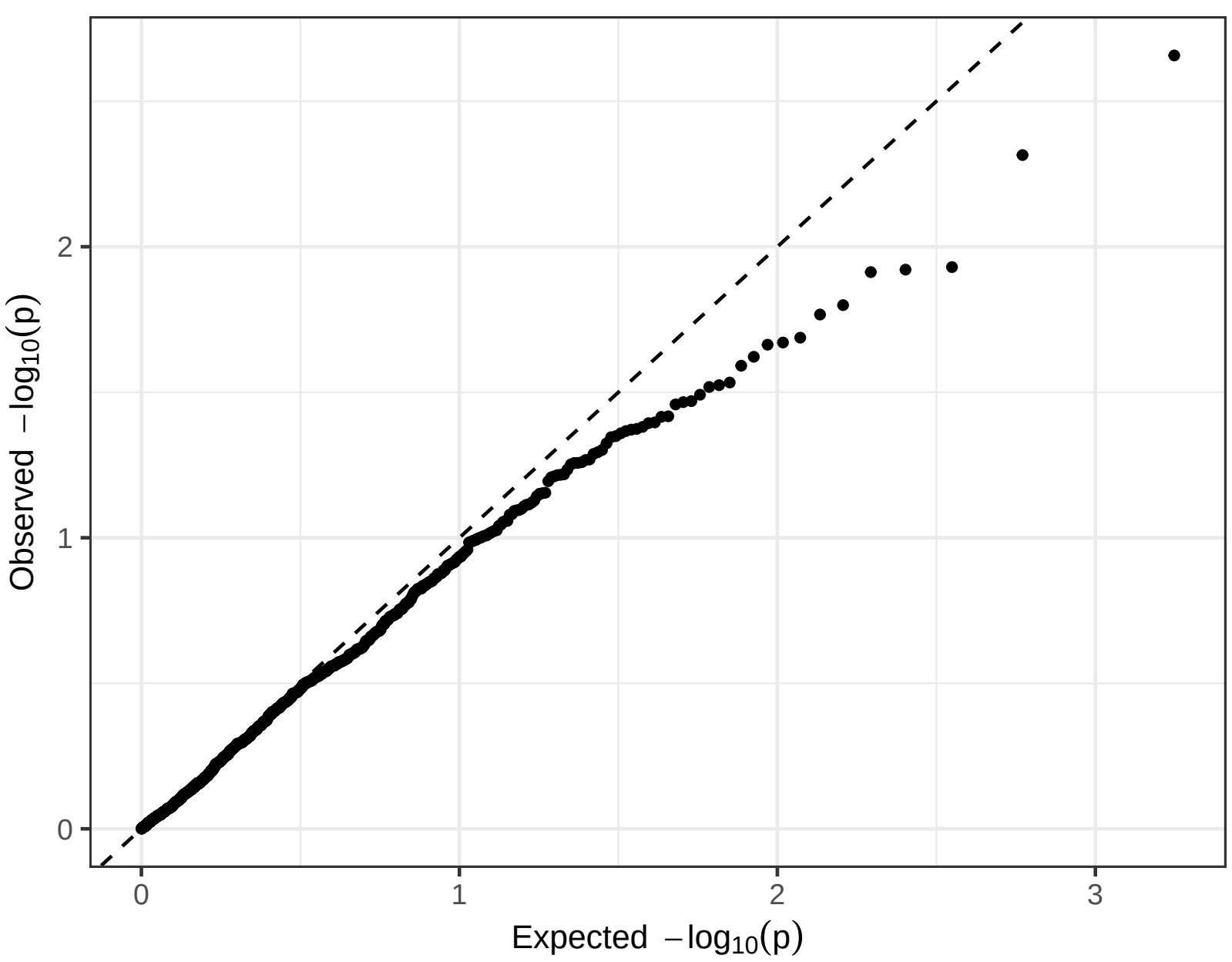

Quinella

Cecum | Muscle | . = 1.005

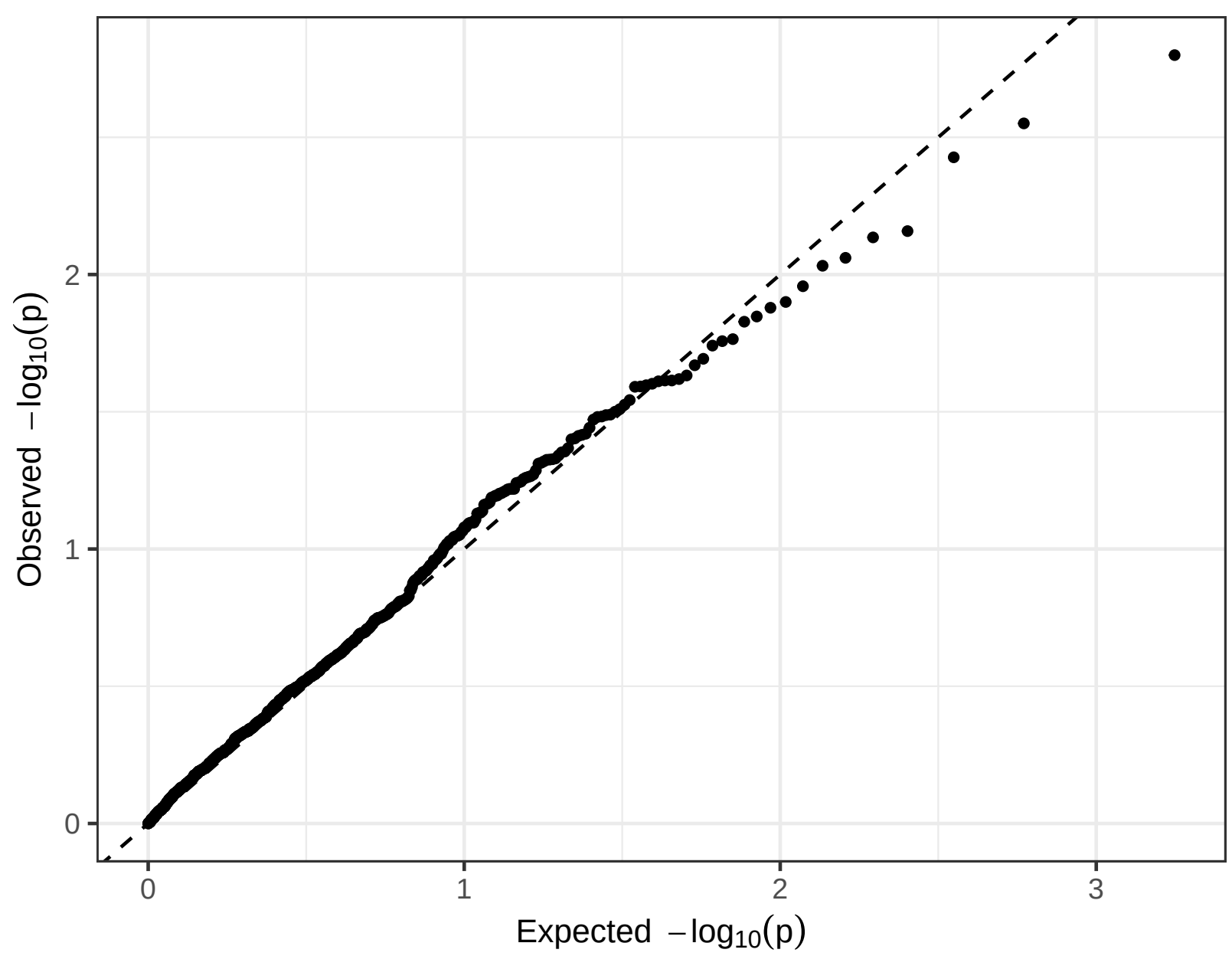

Rikenellaceae_RC9_gut_group

Cecum | Muscle | . = 0.951

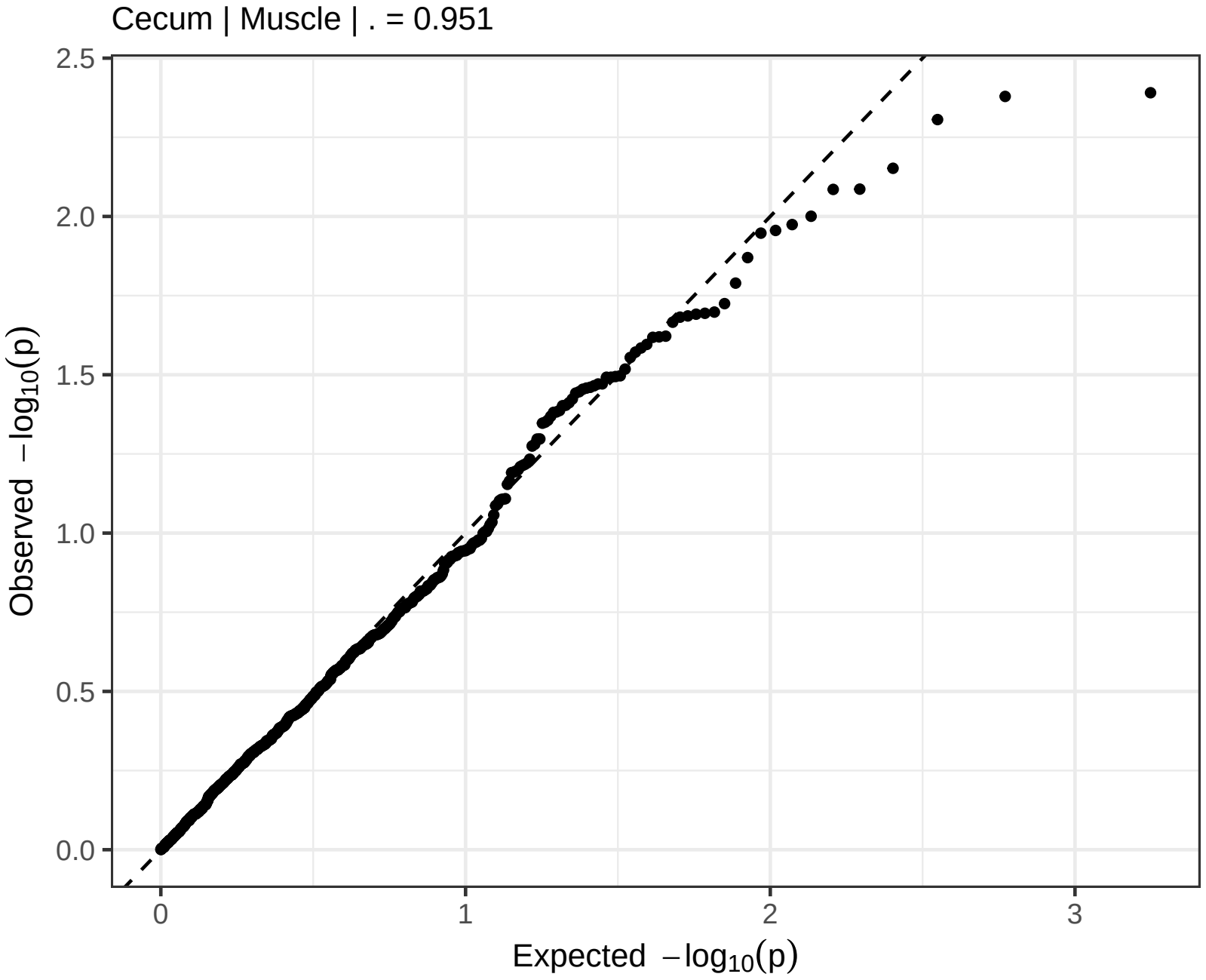

Romboutsia

Cecum | Muscle | . = 0.77

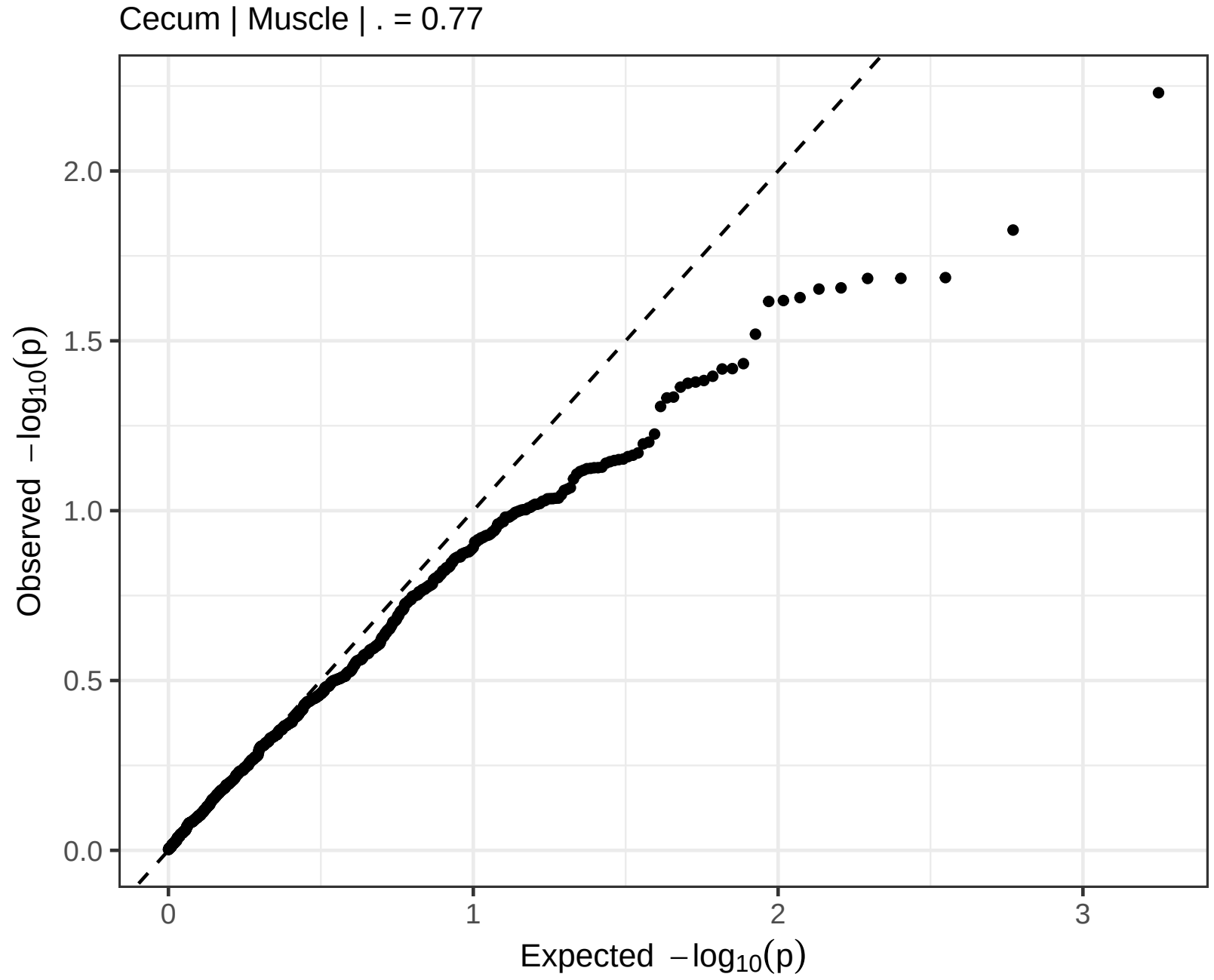

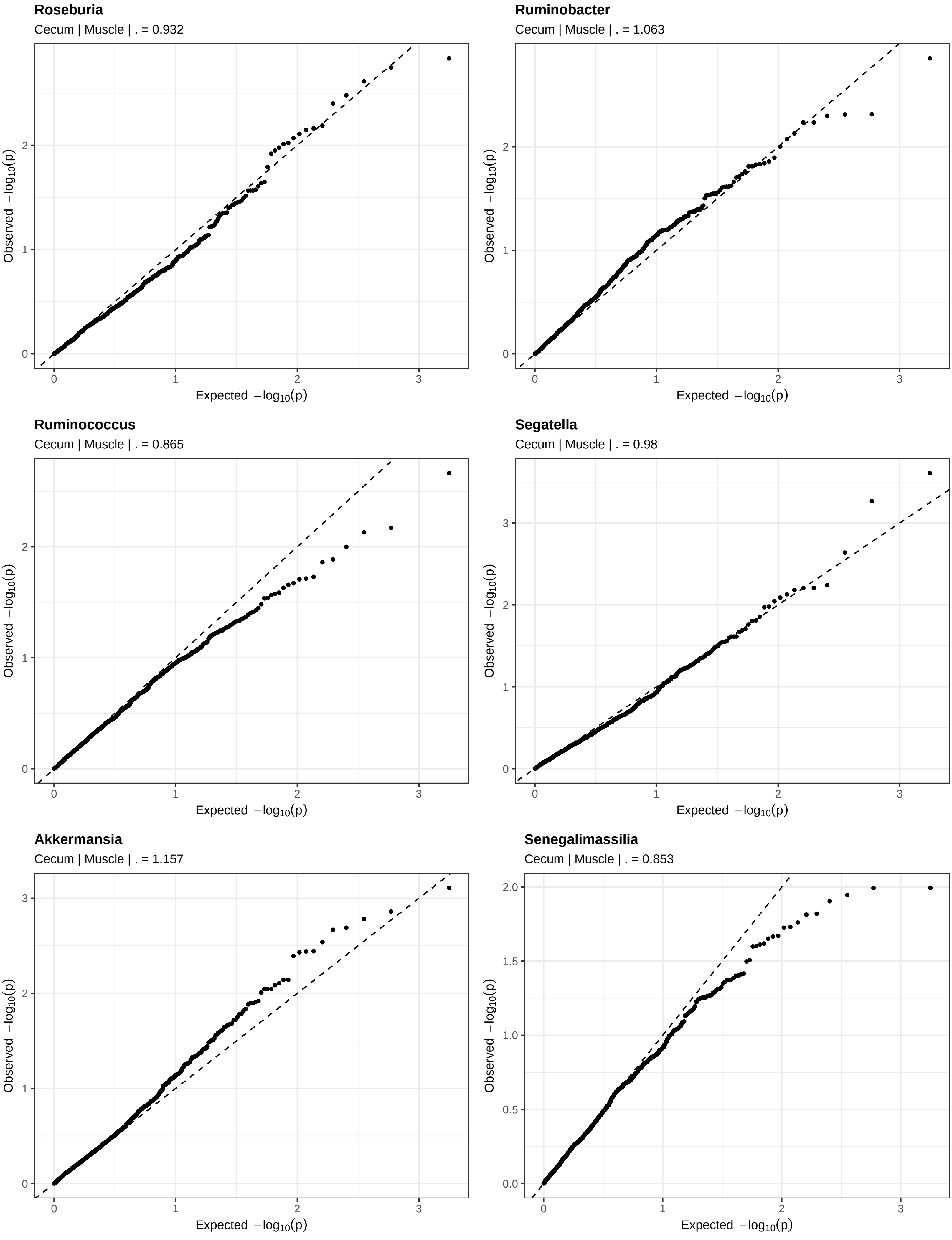
Roseburia
Cecum | Muscle | . = 0.932
Ruminobacter
Cecum | Muscle | . = 1.063
Ruminococcus
Cecum | Muscle | . = 0.865
Segatella
Cecum | Muscle | . = 0.98
Akkermansia
Cecum | Muscle | . = 1.157
Senegalimassilia
Cecum | Muscle | . = 0.853
Observed $-\log_{10}(p)$
Expected $-\log_{10}(p)$

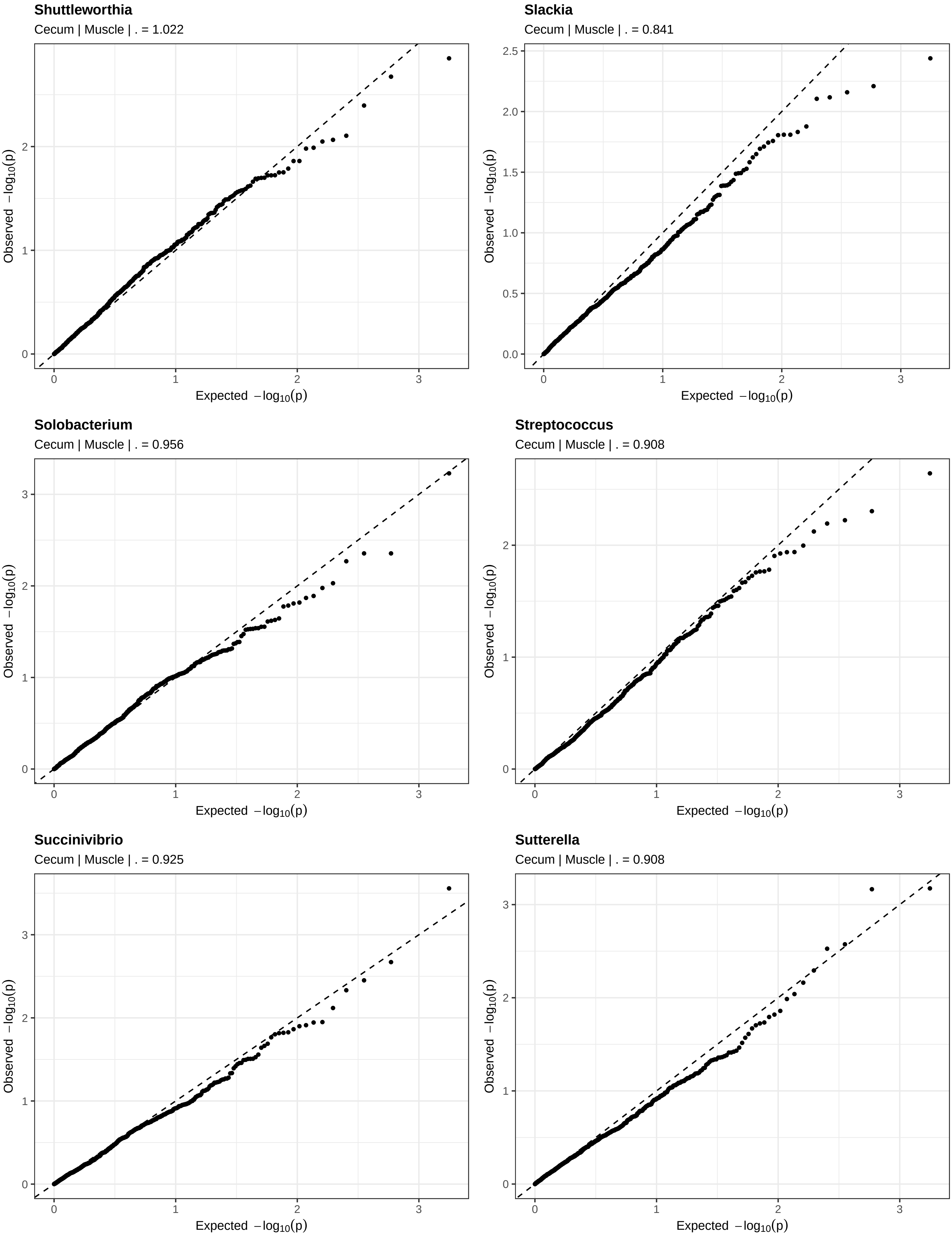

Shuttleworthia
Cecum | Muscle | . = 1.022
Slackia
Cecum | Muscle | . = 0.841
Solobacterium
Cecum | Muscle | . = 0.956
Streptococcus
Cecum | Muscle | . = 0.908
Succinivibrio
Cecum | Muscle | . = 0.925
Sutterella
Cecum | Muscle | . = 0.908
Observed −log10(p)
Expected −log10(p)
0
1
2
3
0.0
0.5
1.0
1.5
2.0
2.5

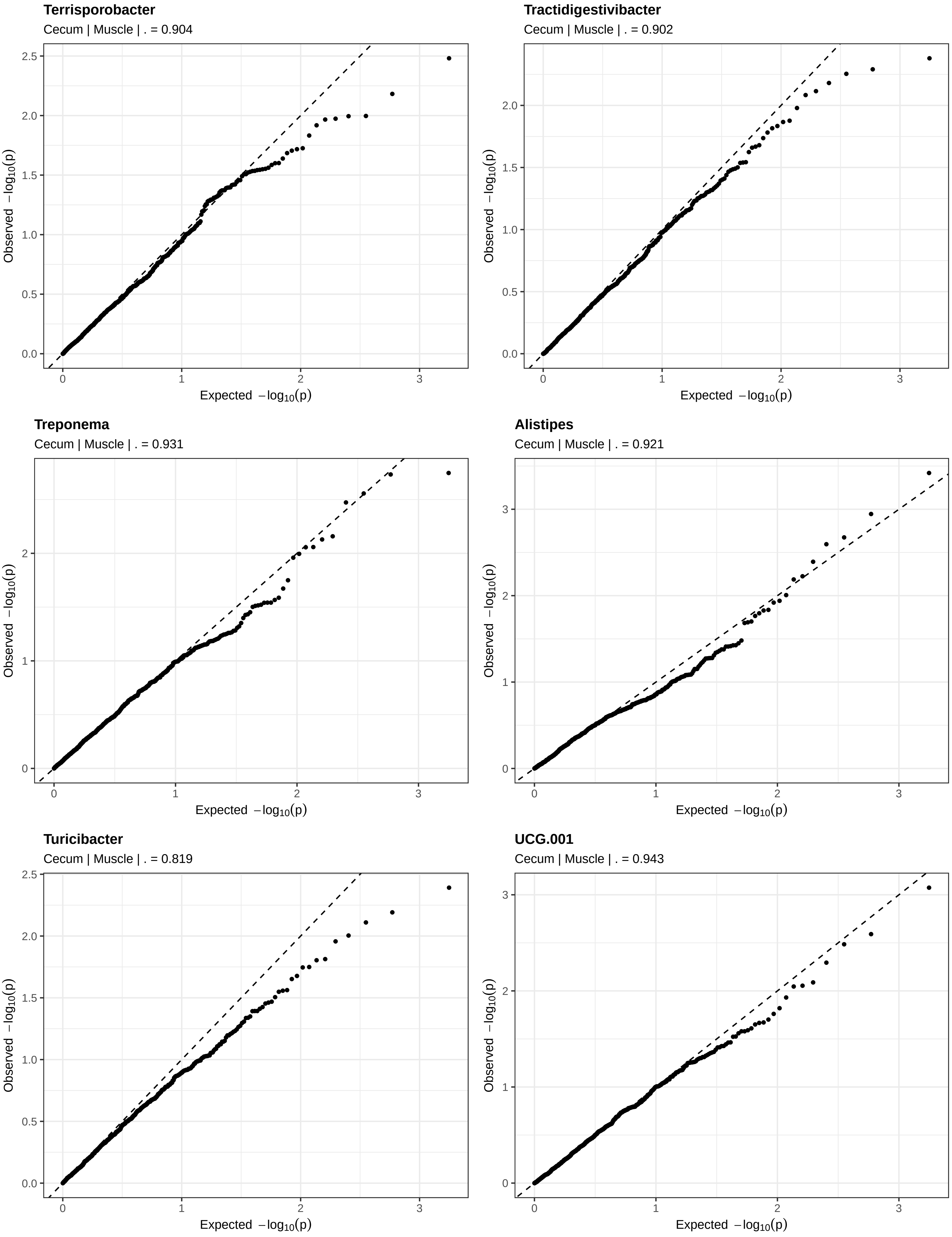
Terrisporobacter
Cecum | Muscle | . = 0.904
Tractidigestivibacter
Cecum | Muscle | . = 0.902
Treponema
Cecum | Muscle | . = 0.931
Alistipes
Cecum | Muscle | . = 0.921
Turicibacter
Cecum | Muscle | . = 0.819
UCG.001
Cecum | Muscle | . = 0.943
Observed $-\log_{10}(p)$
Expected $-\log_{10}(p)$

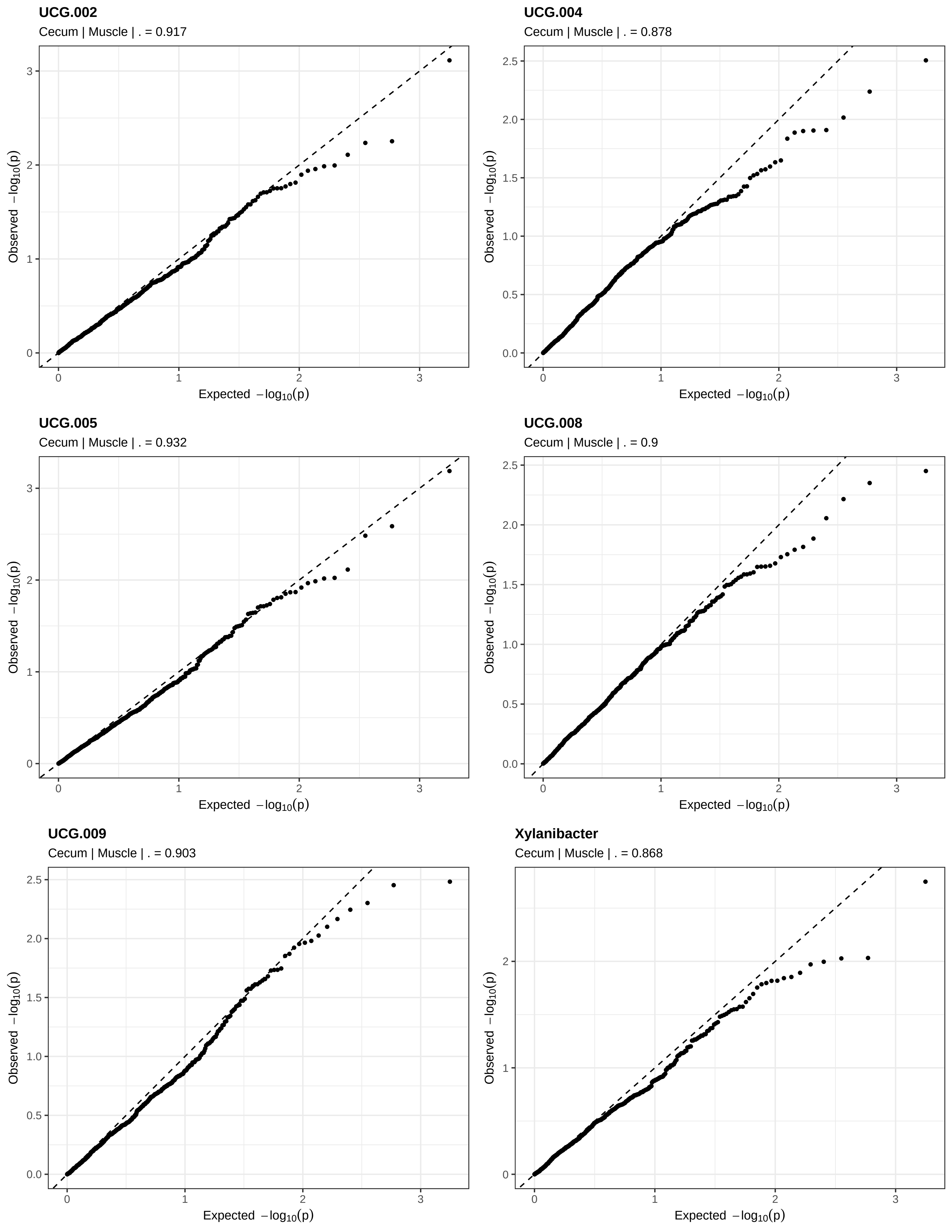

UCG.002
Cecum | Muscle | . = 0.917
UCG.004
Cecum | Muscle | . = 0.878
UCG.005
Cecum | Muscle | . = 0.932
UCG.008
Cecum | Muscle | . = 0.9
UCG.009
Cecum | Muscle | . = 0.903
Xylanibacter
Cecum | Muscle | . = 0.868
Observed $-\log_{10}(p)$
Expected $-\log_{10}(p)$

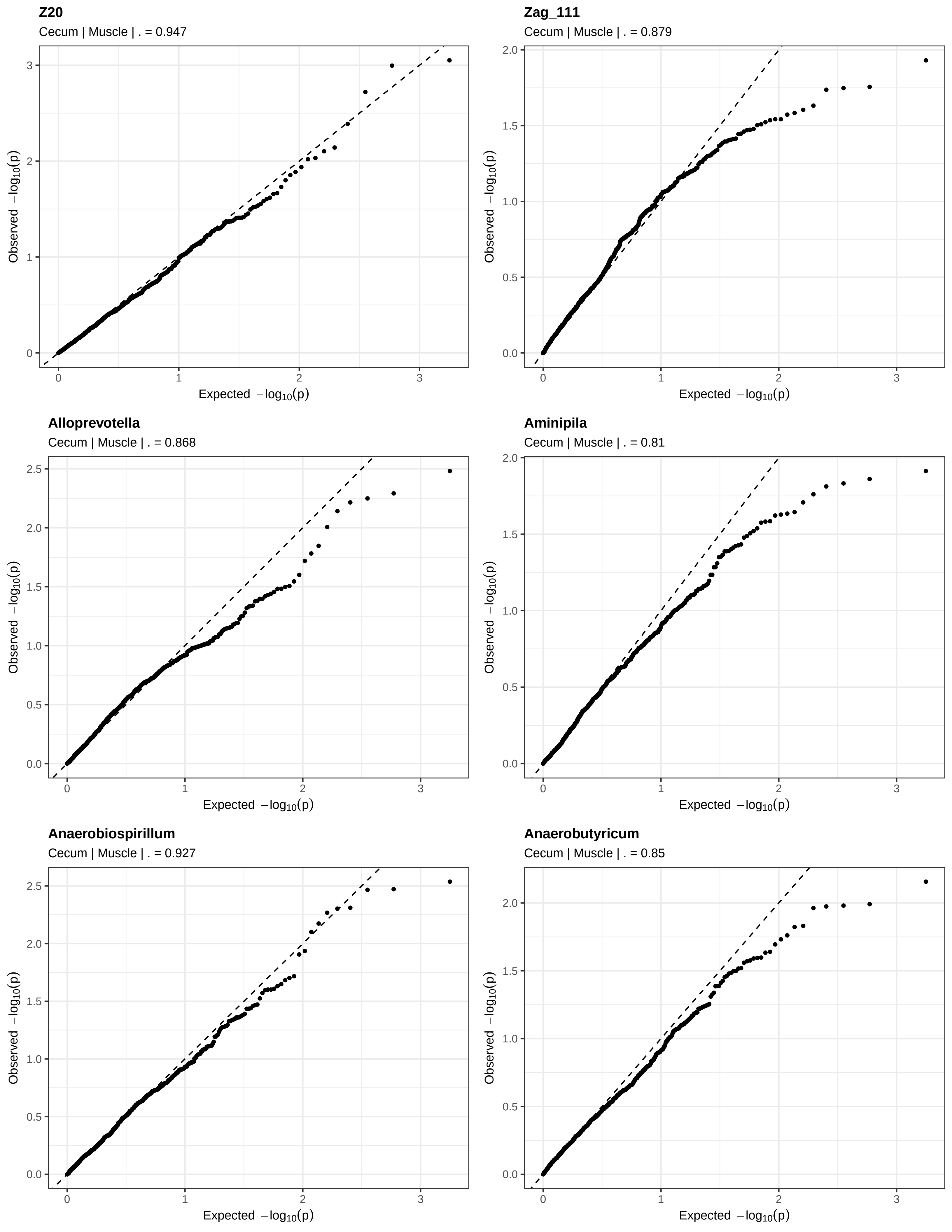
Z20
Cecum | Muscle | . = 0.947
Observed −log10(p)
Expected −log10(p)
Zag_111
Cecum | Muscle | . = 0.879
Observed −log10(p)
Expected −log10(p)
Alloprevotella
Cecum | Muscle | . = 0.868
Observed −log10(p)
Expected −log10(p)
Aminipila
Cecum | Muscle | . = 0.81
Observed −log10(p)
Expected −log10(p)
Anaerobiospirillum
Cecum | Muscle | . = 0.927
Observed −log10(p)
Expected −log10(p)
Anaerobutyricum
Cecum | Muscle | . = 0.85
Observed −log10(p)
Expected −log10(p)

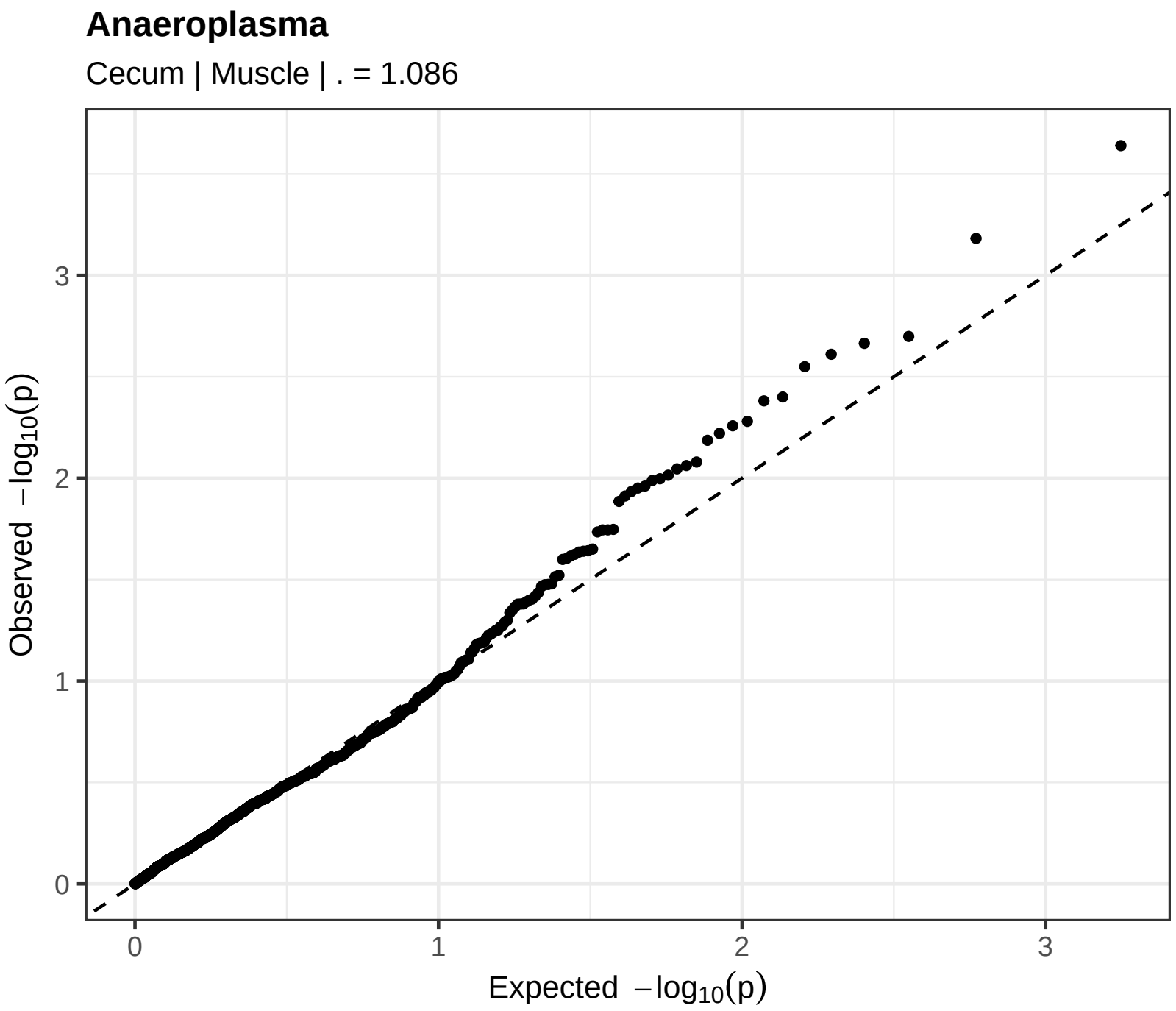
Anaeroplasma
Cecum | Muscle | . = 1.086
Observed −log10(p)
Expected −log10(p)

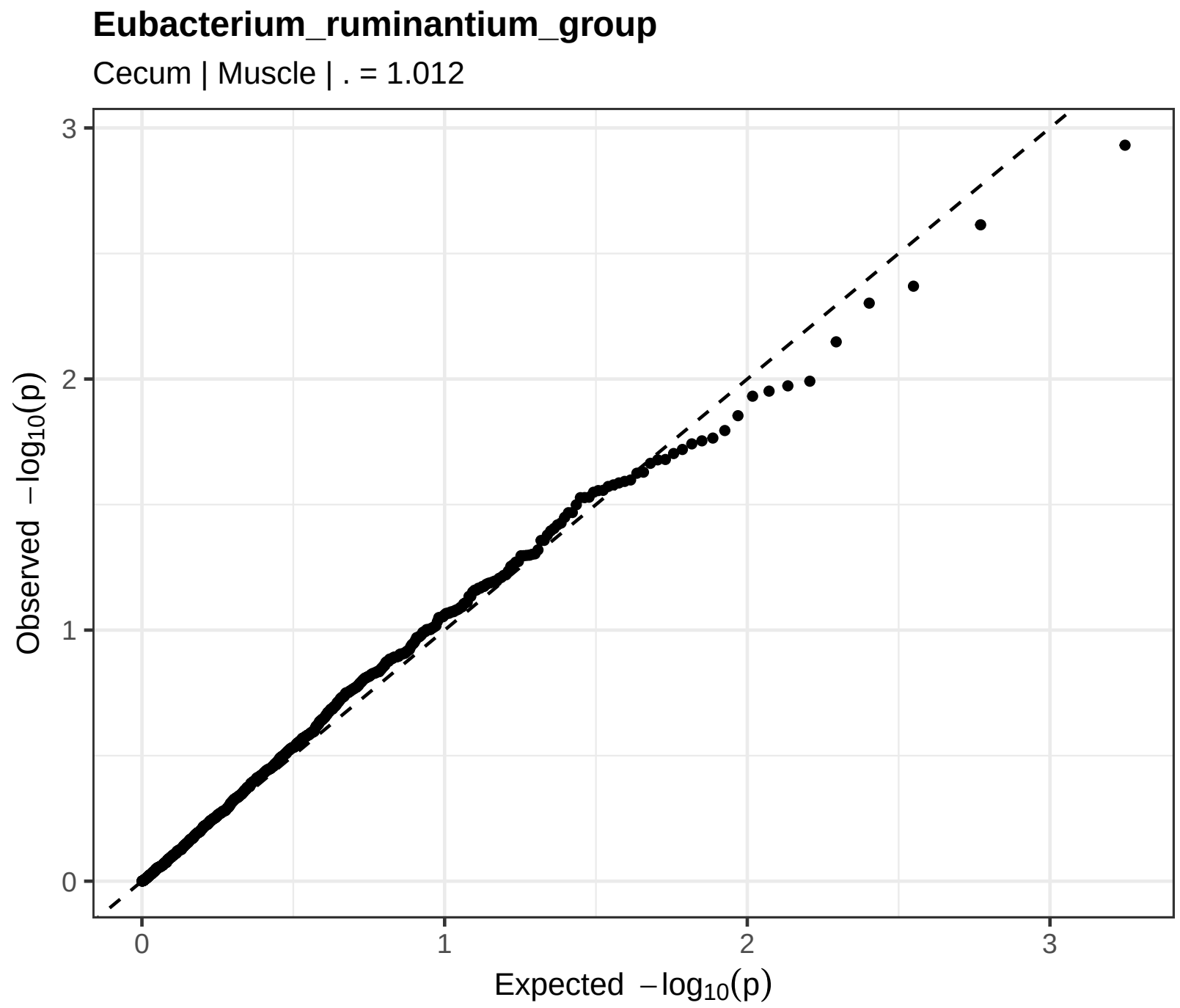
Eubacterium_ruminantium_group
Cecum | Muscle | . = 1.012
Observed −log10(p)
Expected −log10(p)

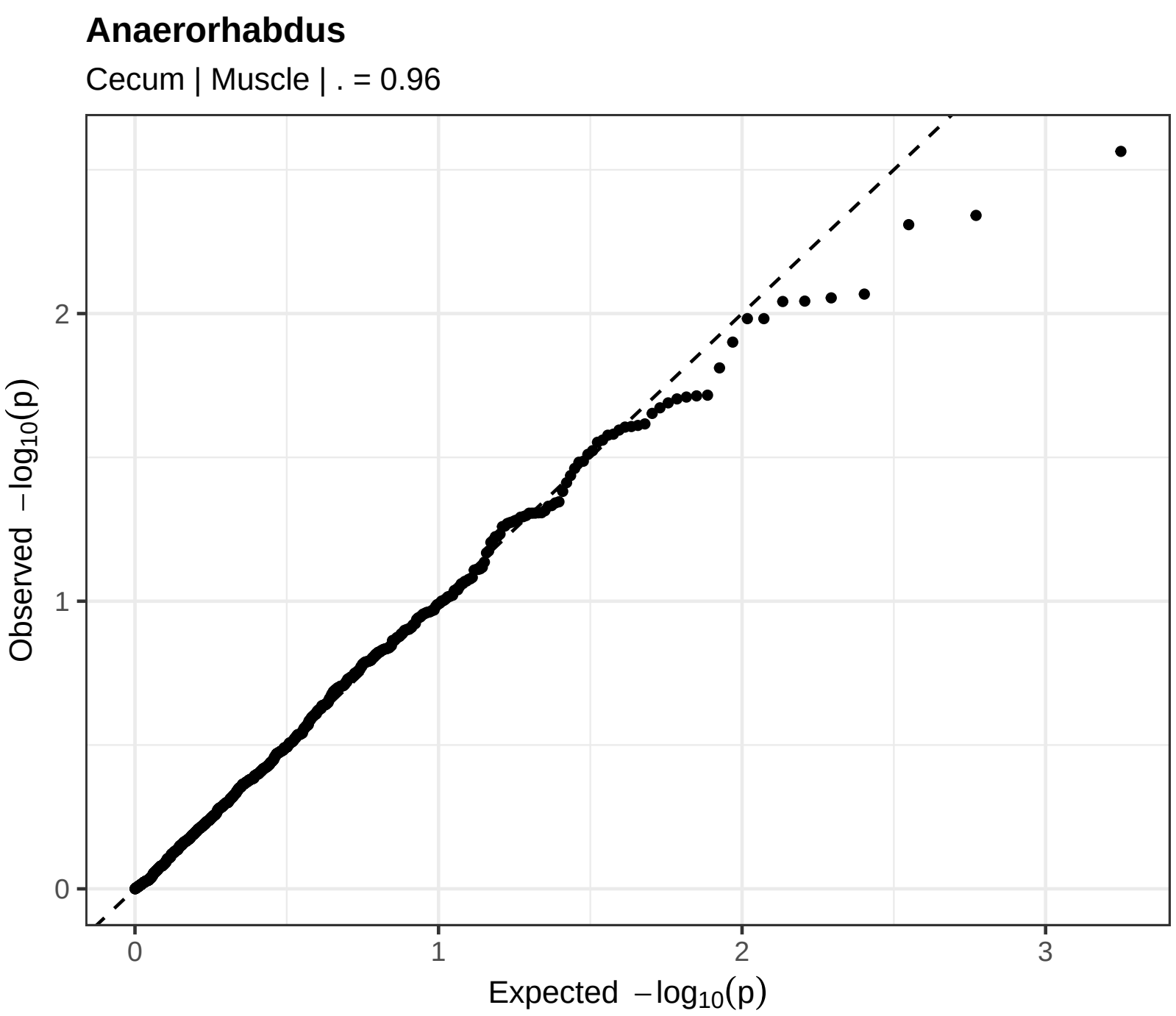
Anaerorhabdus
Cecum | Muscle | . = 0.96
Observed −log10(p)
Expected −log10(p)

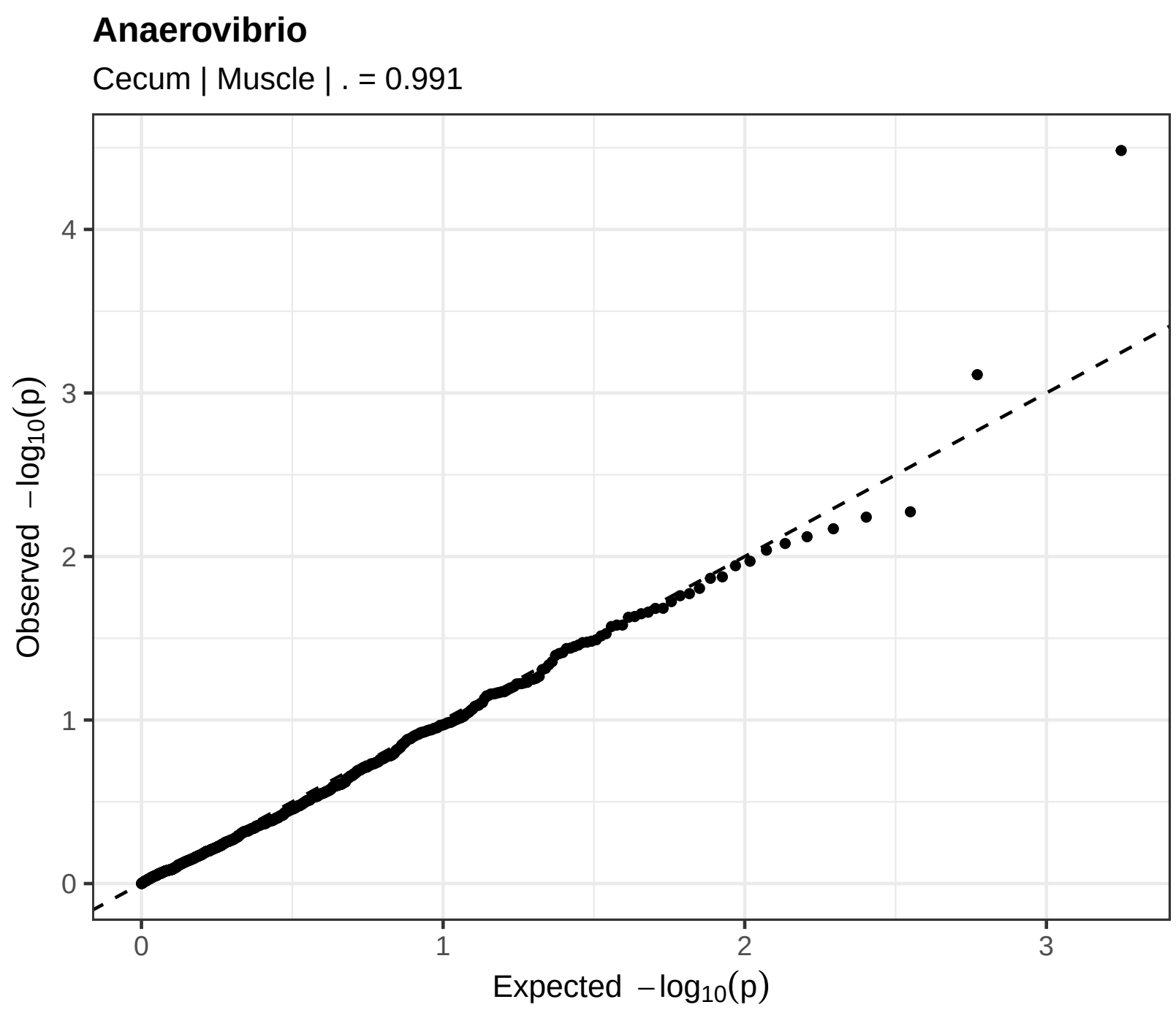
Anaerovibrio
Cecum | Muscle | . = 0.991
Observed −log10(p)
Expected −log10(p)

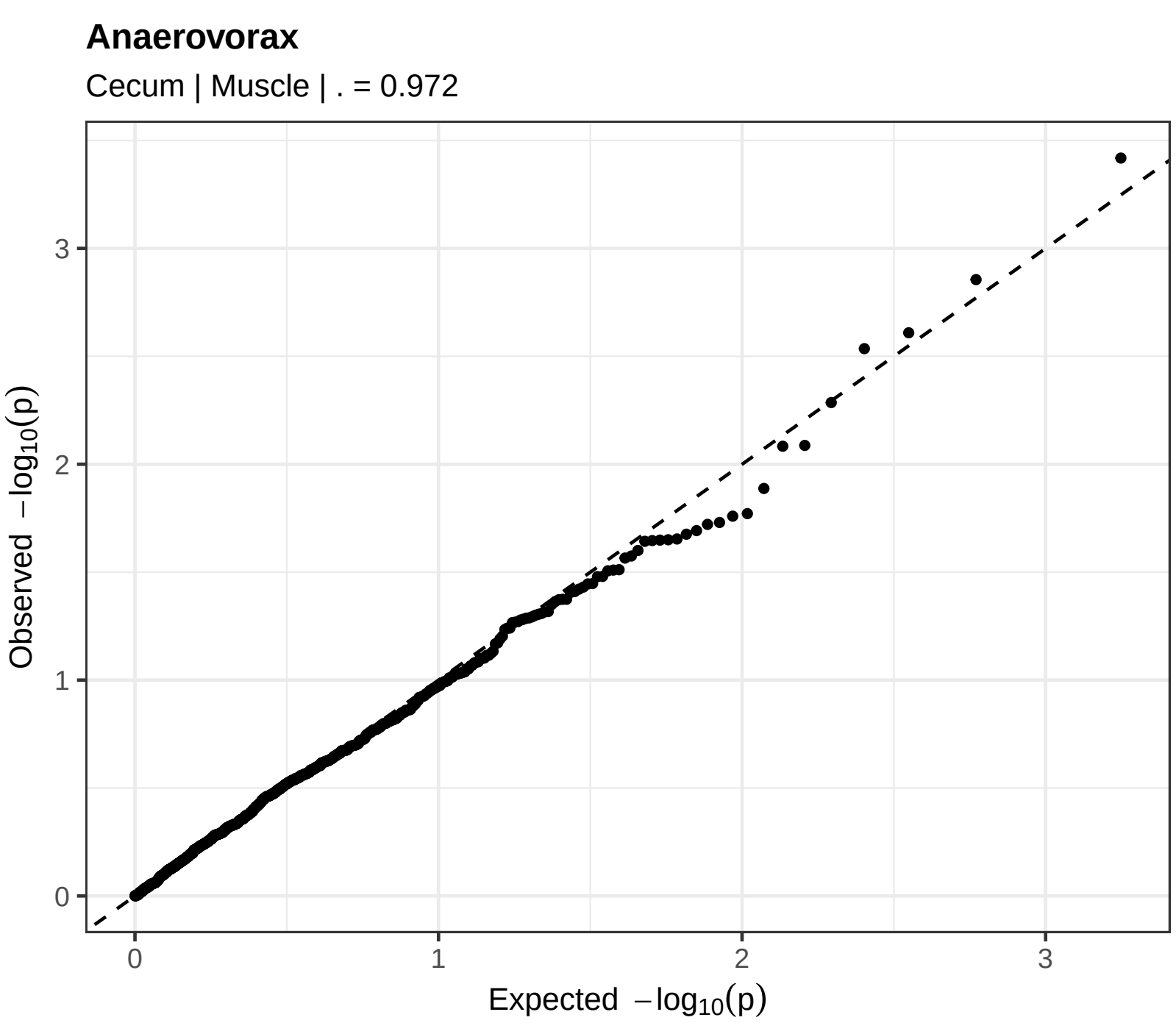
Anaerovorax
Cecum | Muscle | . = 0.972
Observed −log10(p)
Expected −log10(p)

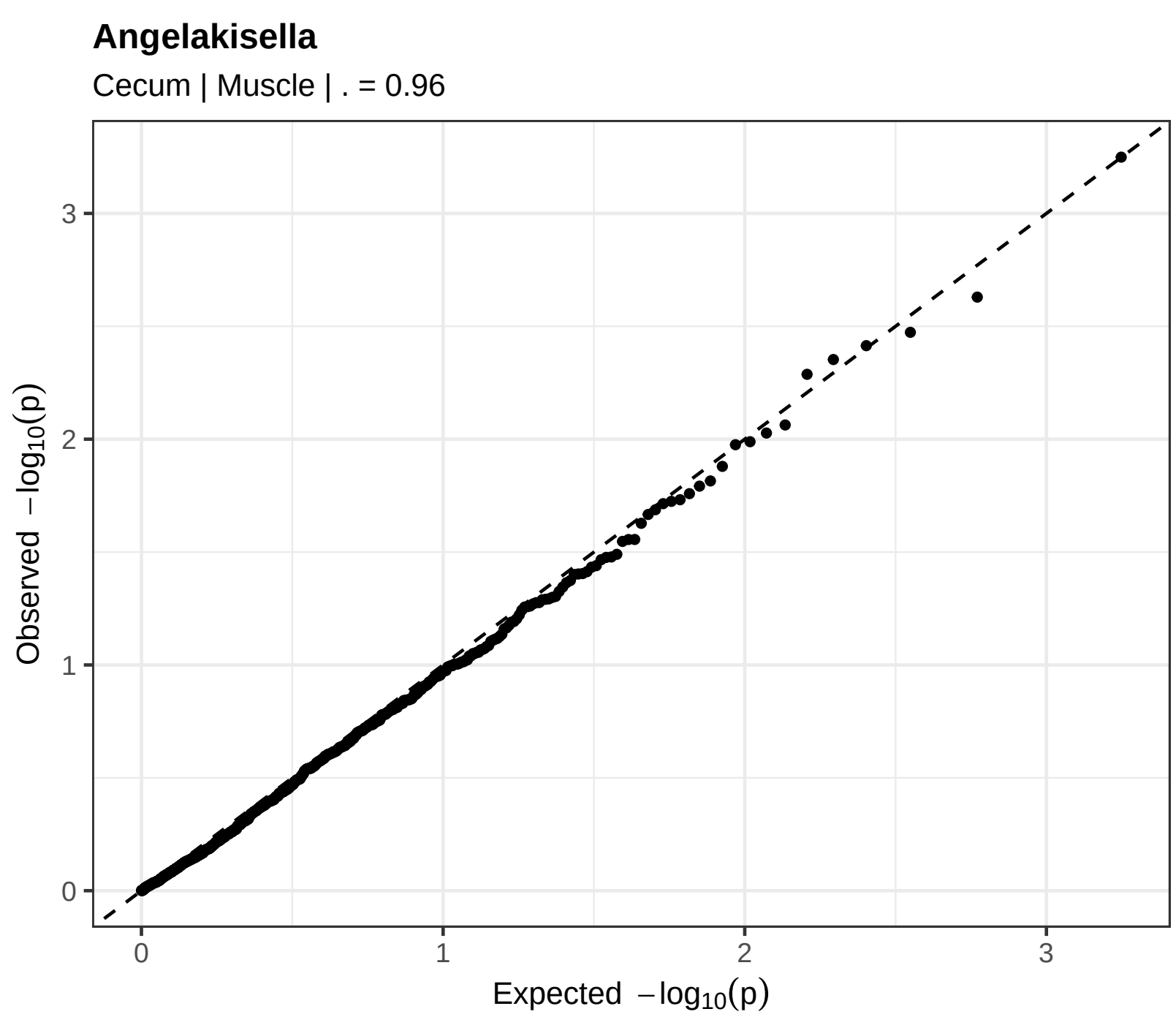
Angelakisella
Cecum | Muscle | . = 0.96
Observed −log10(p)
Expected −log10(p)

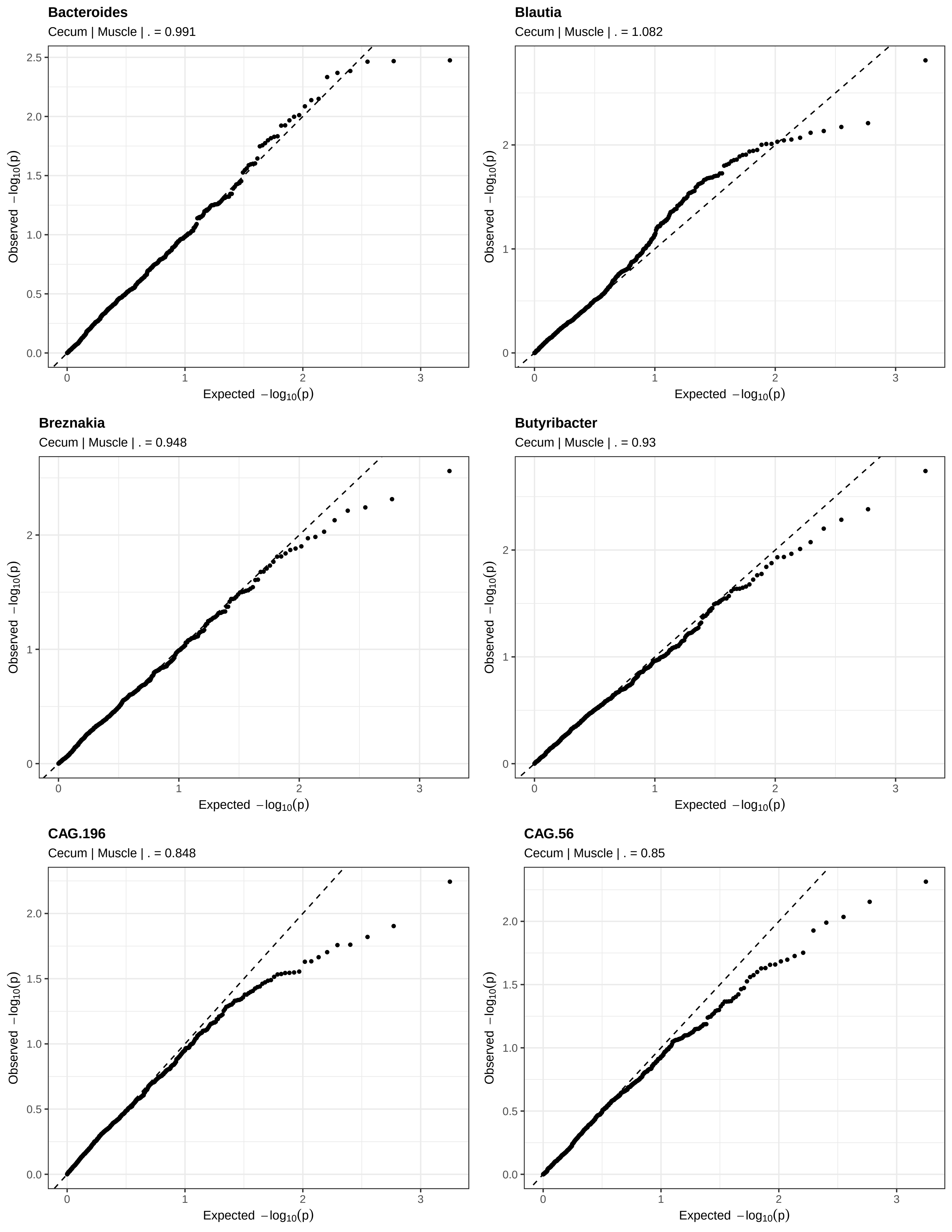
Bacteroides
Cecum | Muscle | . = 0.991
Blautia
Cecum | Muscle | . = 1.082
Breznakia
Cecum | Muscle | . = 0.948
Butyribacter
Cecum | Muscle | . = 0.93
CAG.196
Cecum | Muscle | . = 0.848
CAG.56
Cecum | Muscle | . = 0.85
Observed $-\log_{10}(p)$
Expected $-\log_{10}(p)$

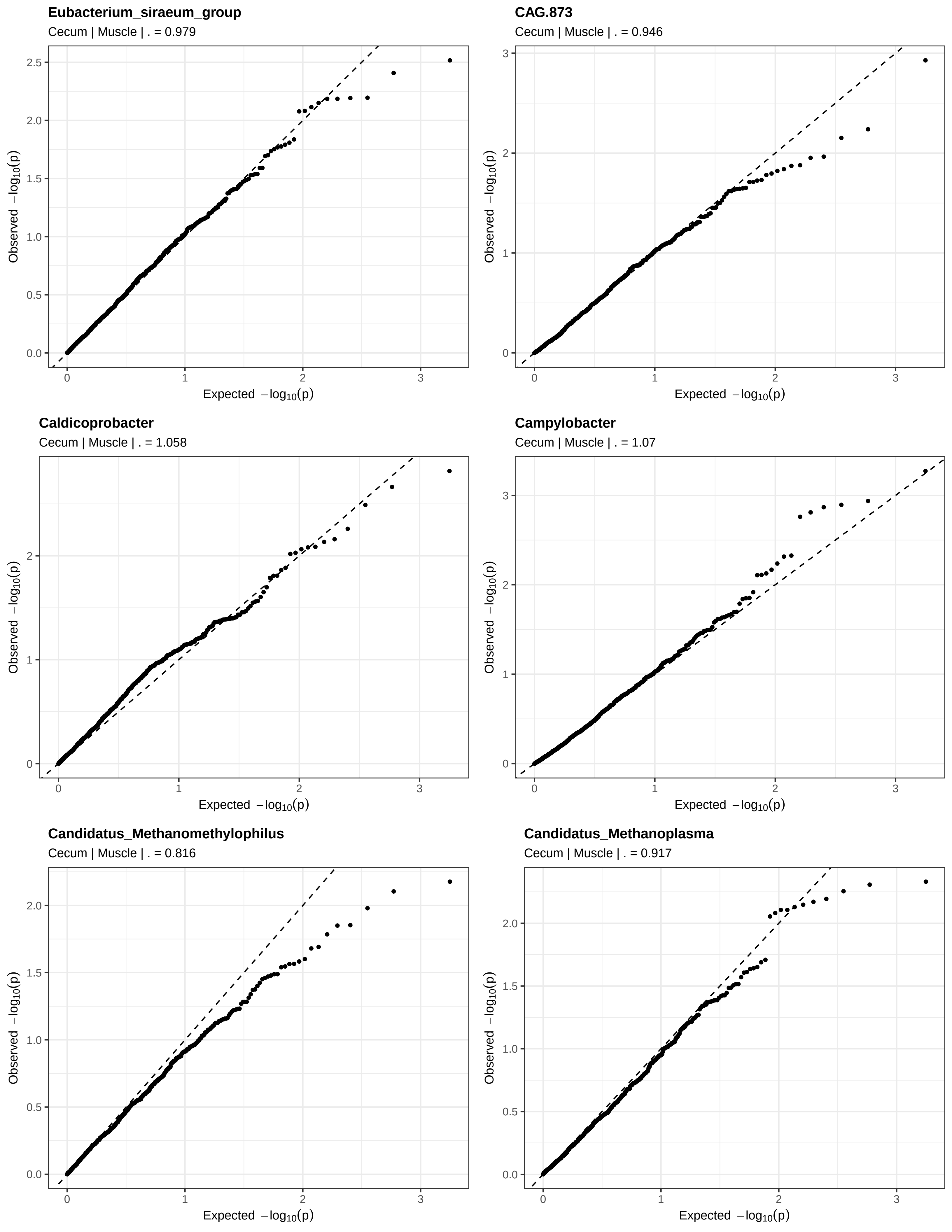
Eubacterium_siraeum_group
Cecum | Muscle | . = 0.979
Observed −log10(p)
Expected −log10(p)
CAG.873
Cecum | Muscle | . = 0.946
Observed −log10(p)
Expected −log10(p)
Caldicoprobacter
Cecum | Muscle | . = 1.058
Observed −log10(p)
Expected −log10(p)
Campylobacter
Cecum | Muscle | . = 1.07
Observed −log10(p)
Expected −log10(p)
Candidatus_Methanomethylophilus
Cecum | Muscle | . = 0.816
Observed −log10(p)
Expected −log10(p)
Candidatus_Methanoplasma
Cecum | Muscle | . = 0.917
Observed −log10(p)
Expected −log10(p)

Candidatus_Saccharimonas

Cecum | Muscle | . = 0.875

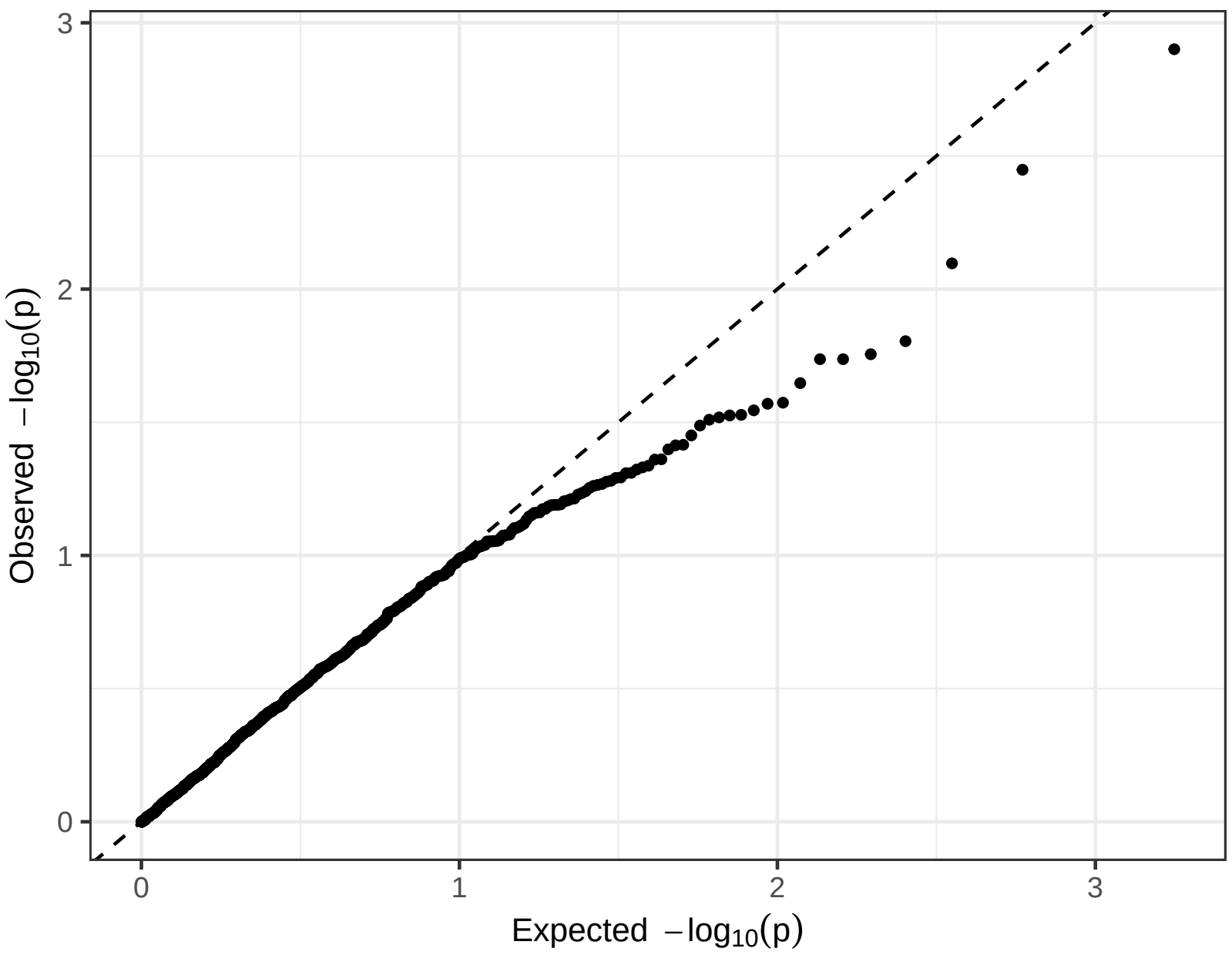

Candidatus_Soleaferrea

Cecum | Muscle | . = 0.918

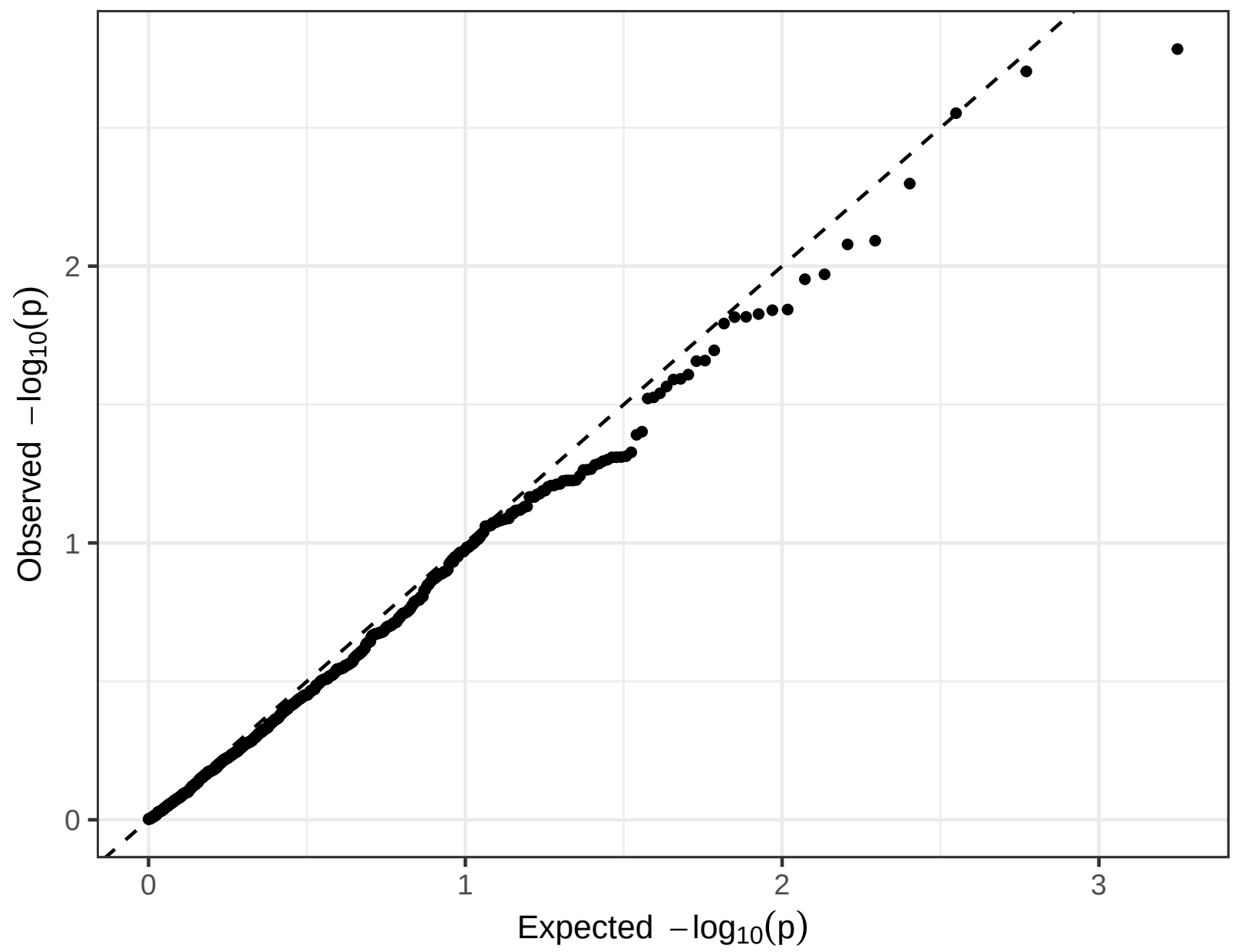

Caproiciproducens

Cecum | Muscle | . = 1.186

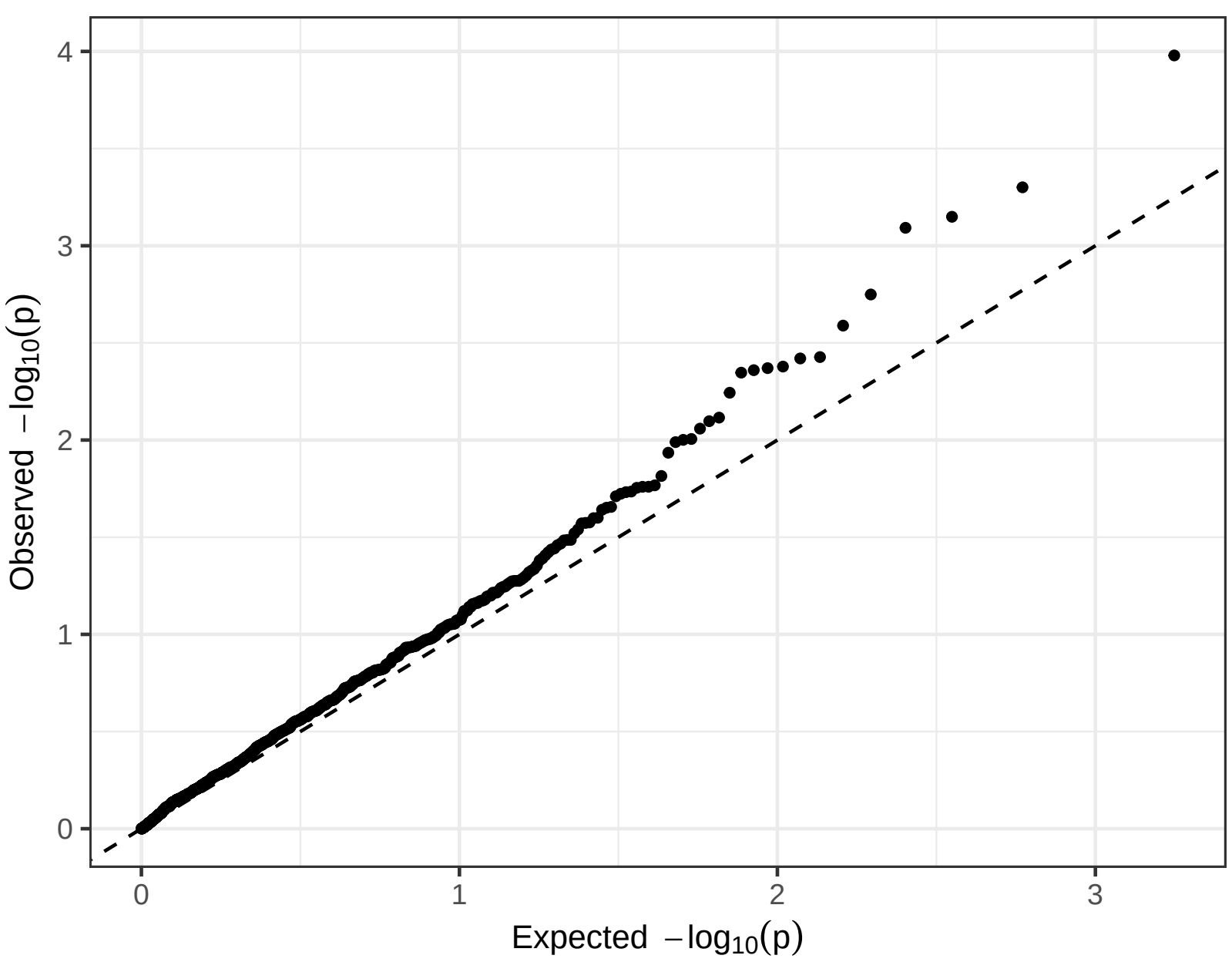

Catenibacterium

Cecum | Muscle | . = 1.077

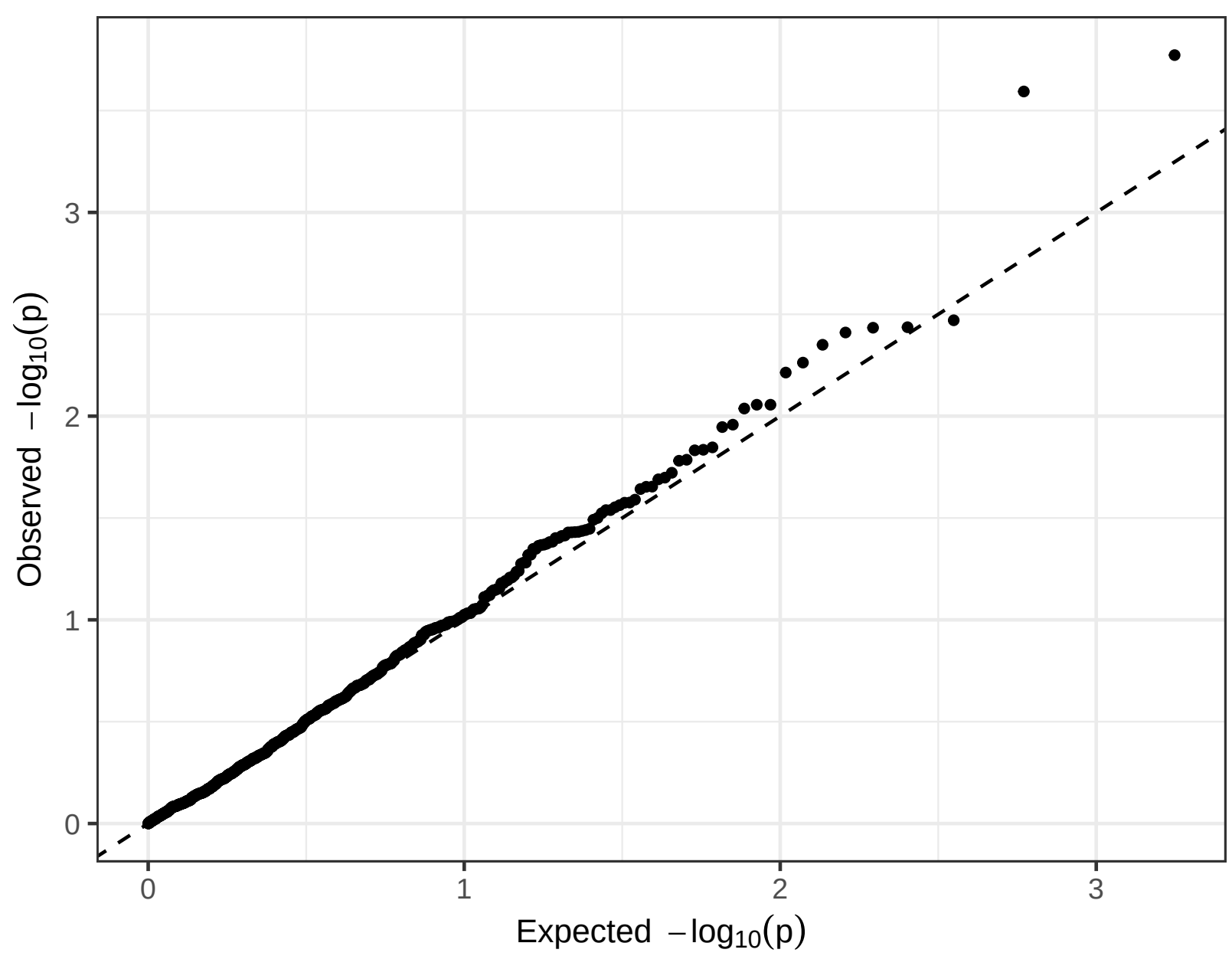

Catenisphaera

Cecum | Muscle | . = 0.913

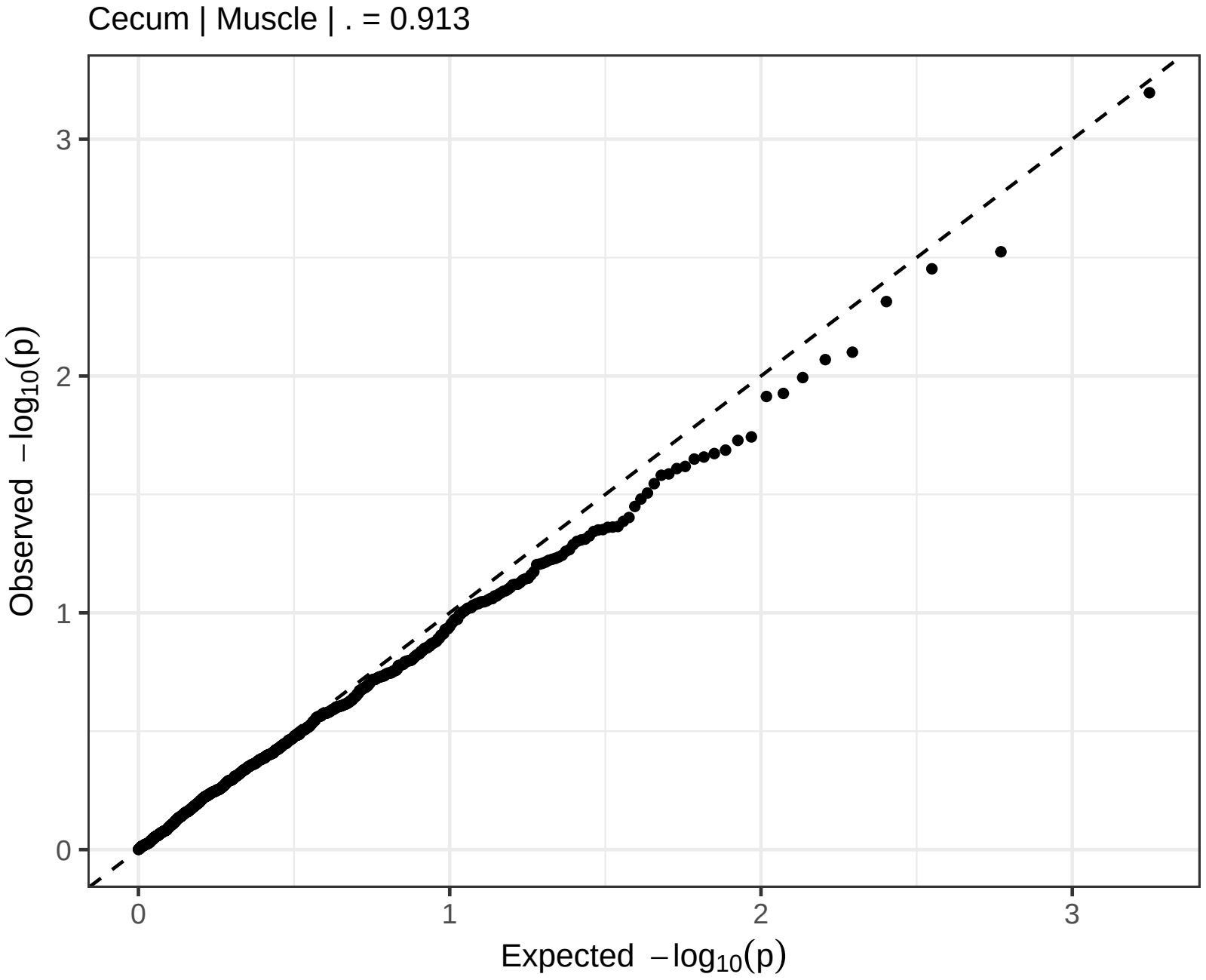

Eubacterium_xylanophilum_group

Cecum | Muscle | . = 0.969

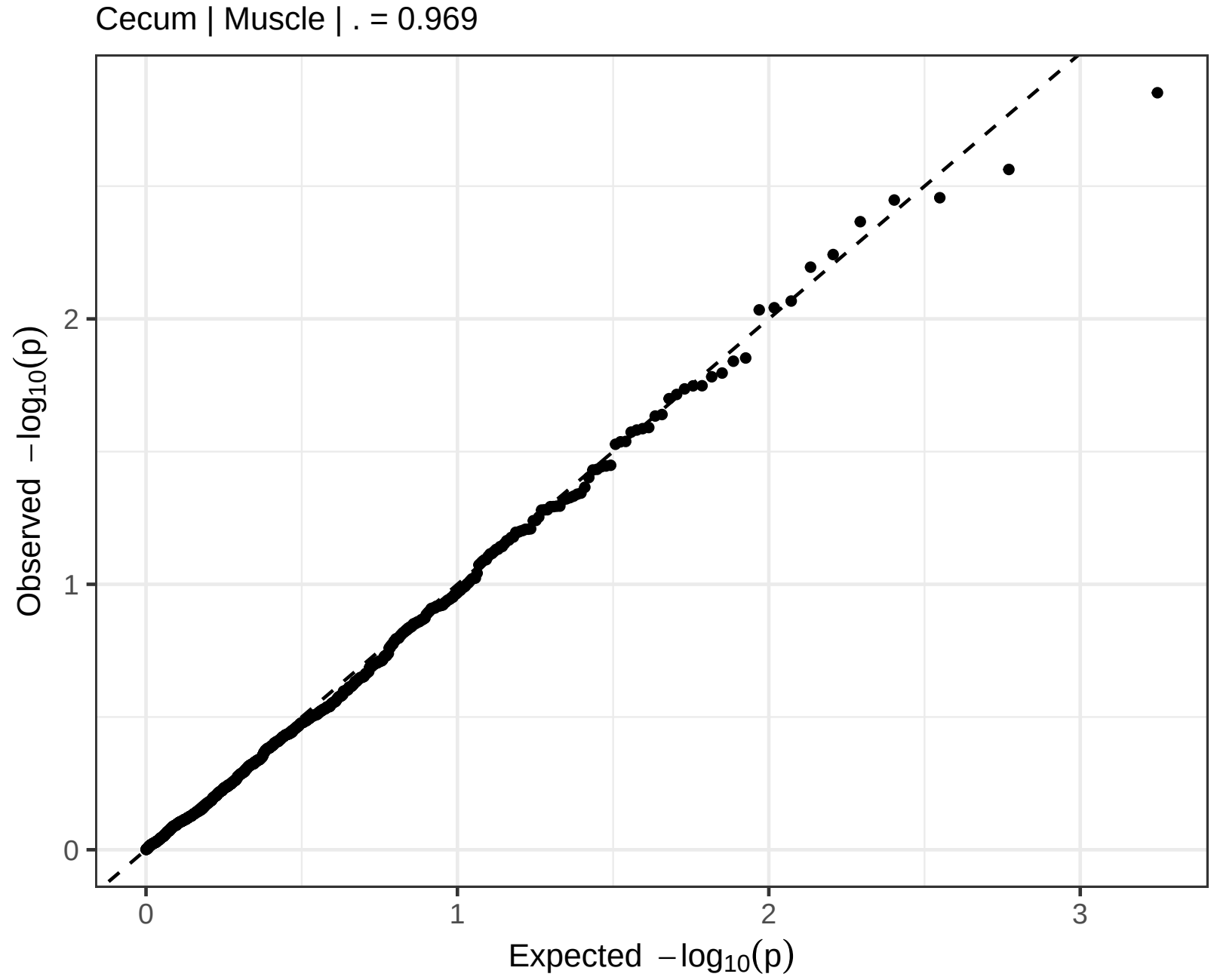

Cellulosilyticum

Cecum | Muscle | . = 0.973

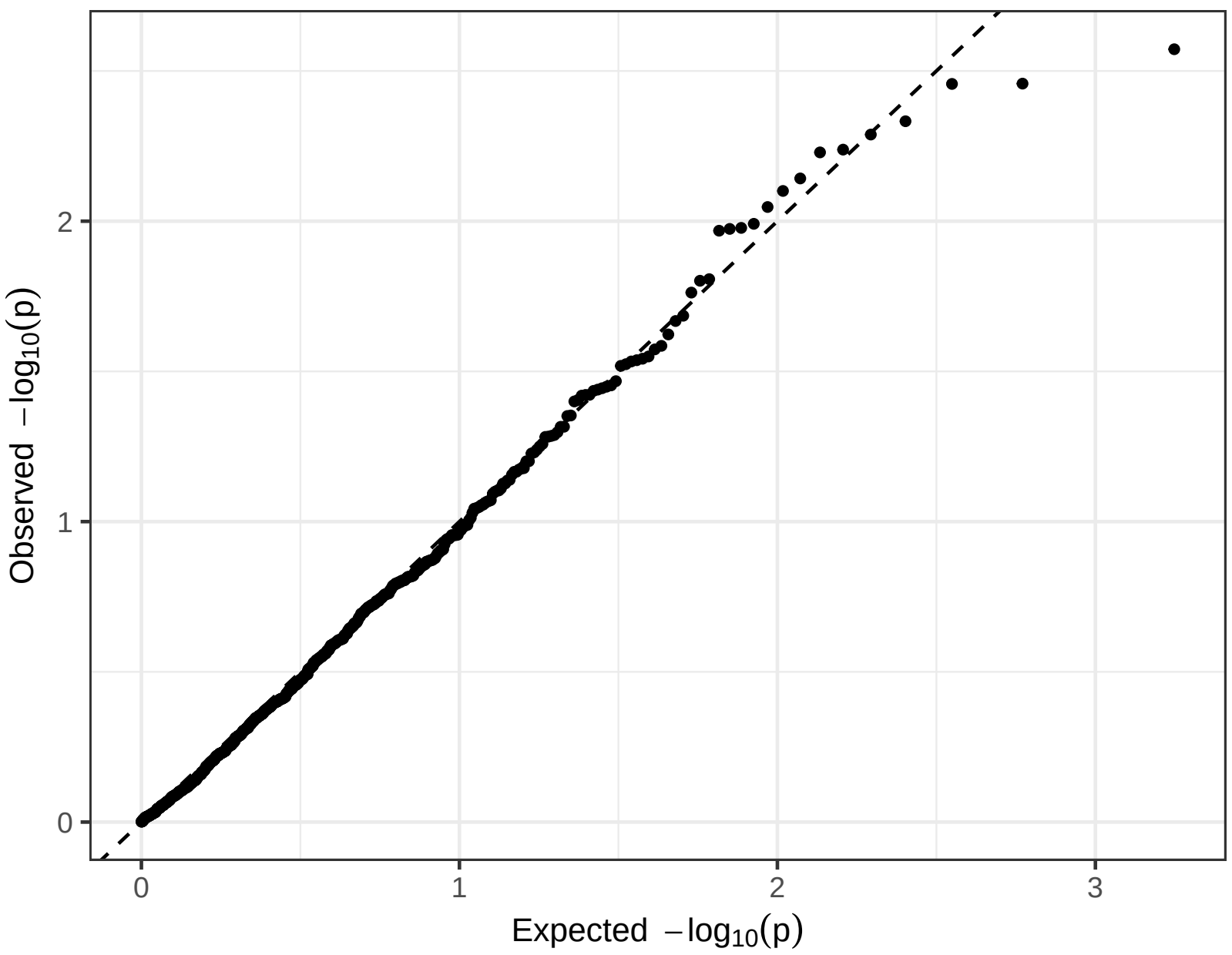

Christensenellaceae_R.7_group

Cecum | Muscle | . = 0.987

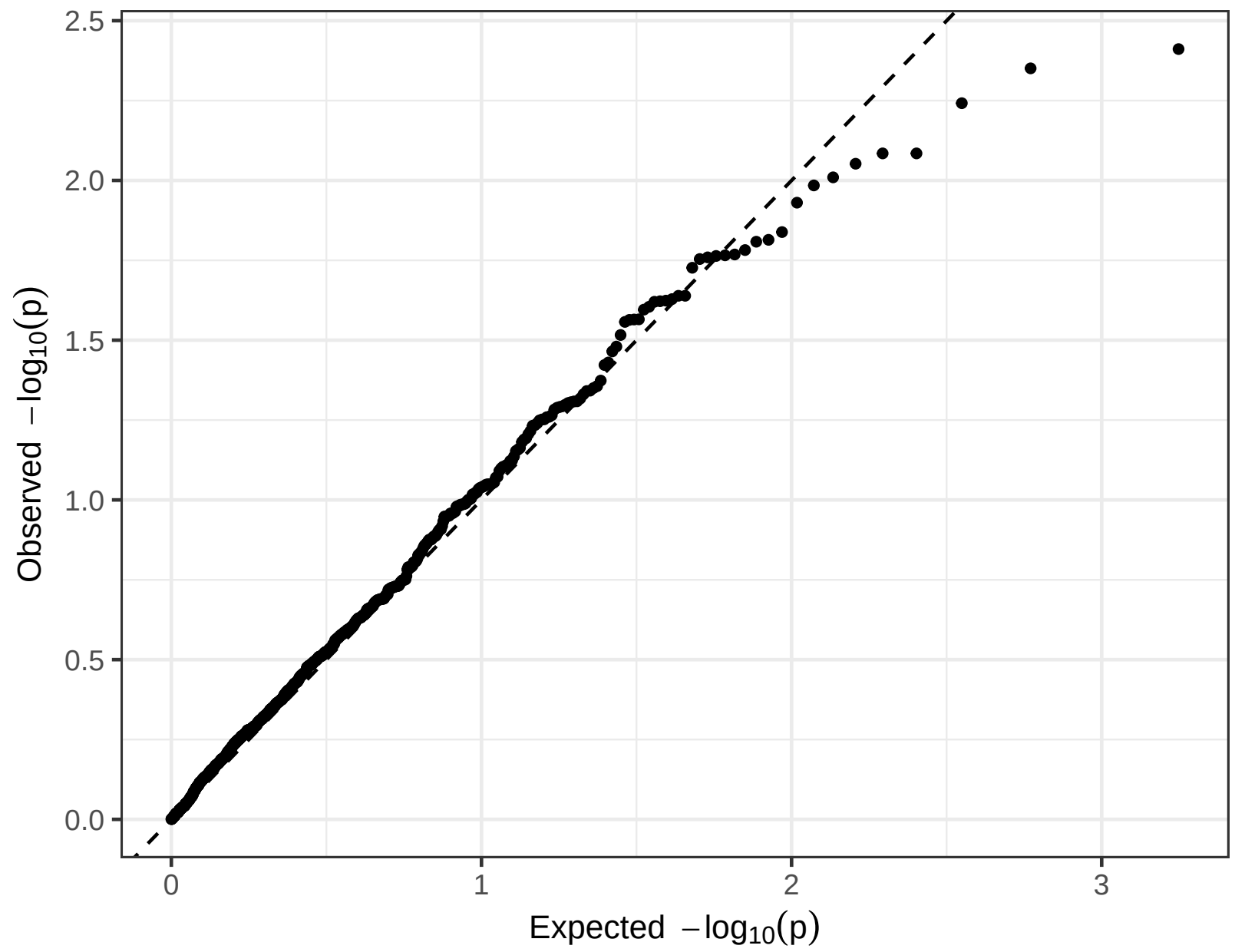

Cloacibacillus

Cecum | Muscle | . = 1.032

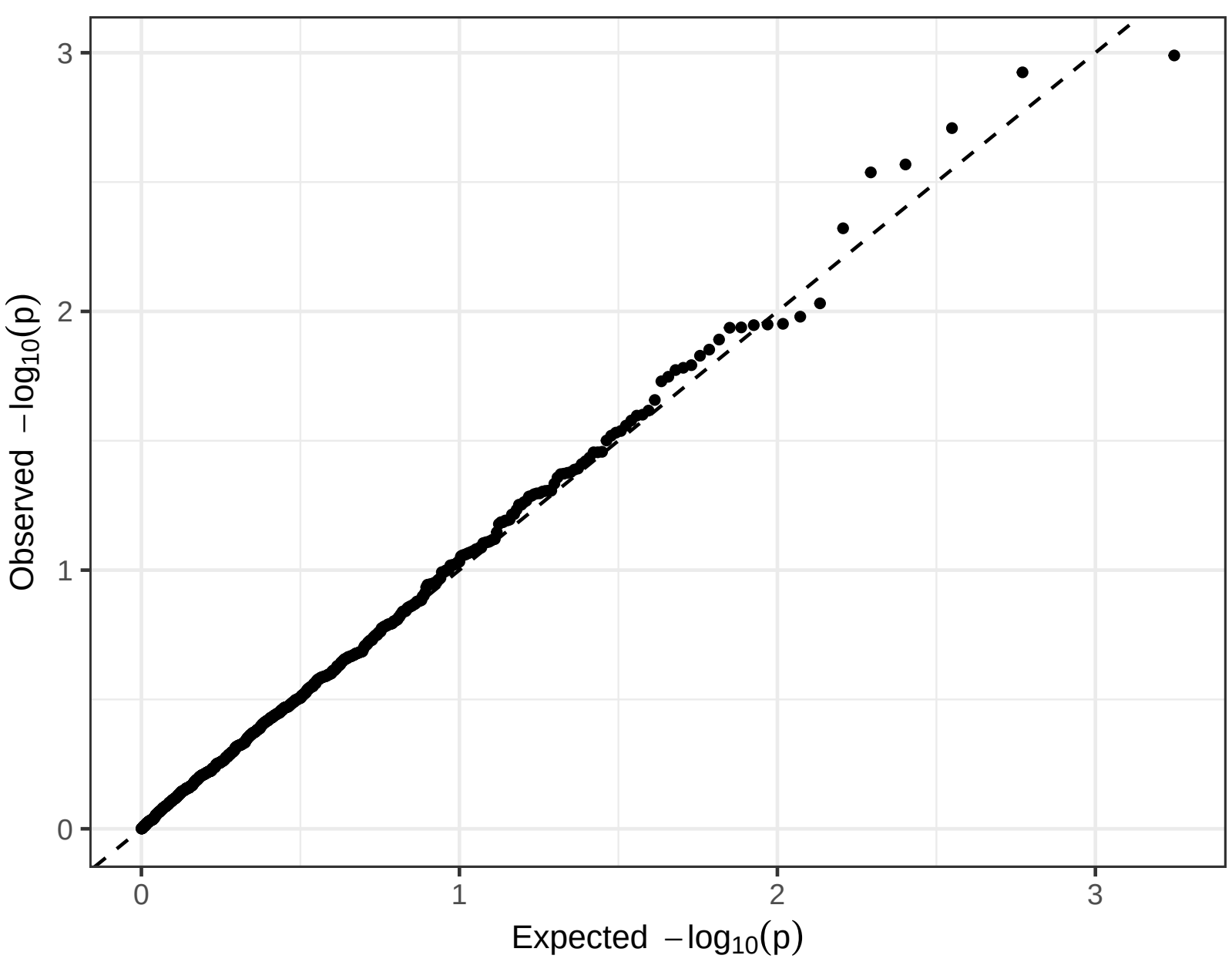

Clostridium

Cecum | Muscle | . = 0.823

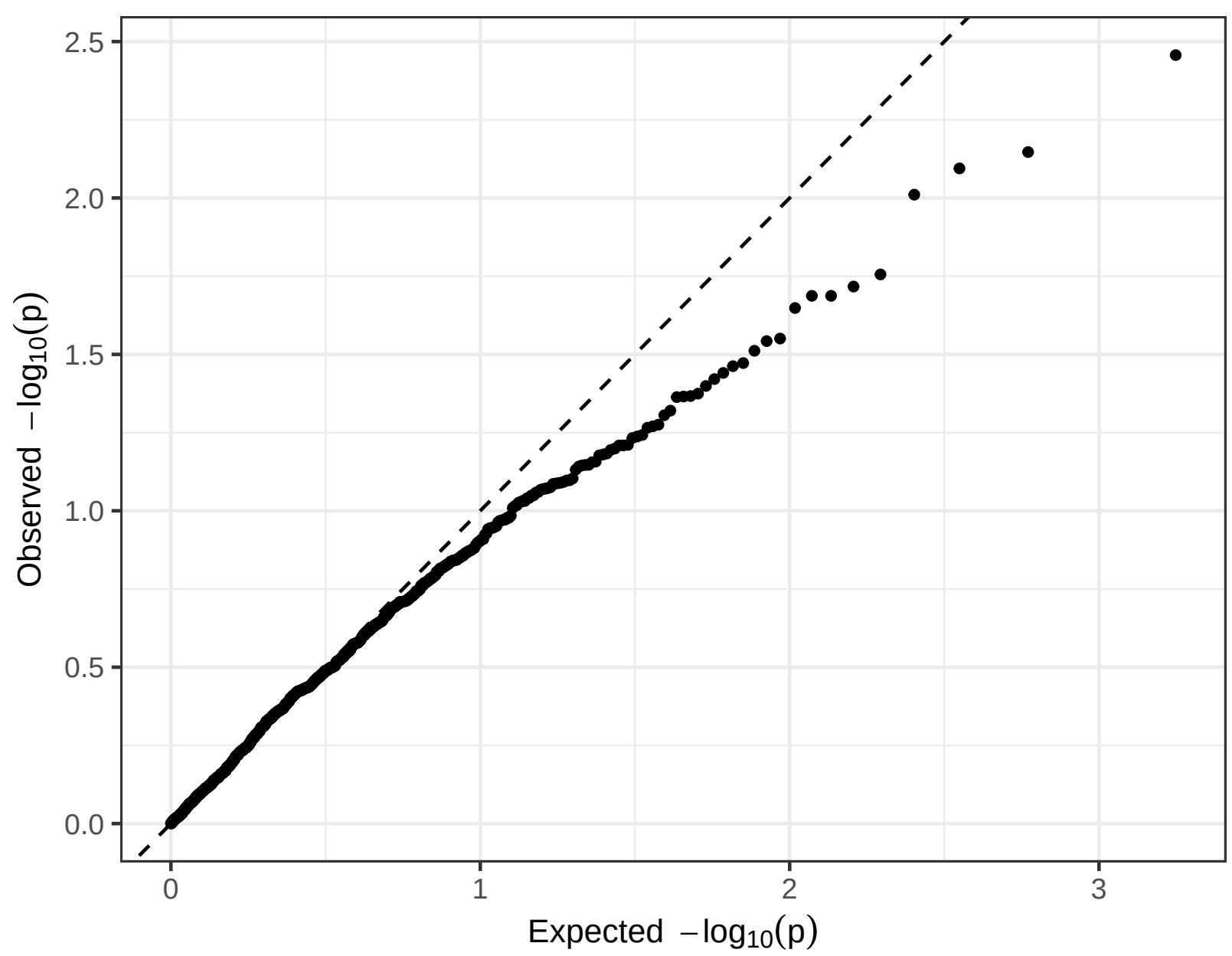

Colidextribacter

Cecum | Muscle | . = 0.827

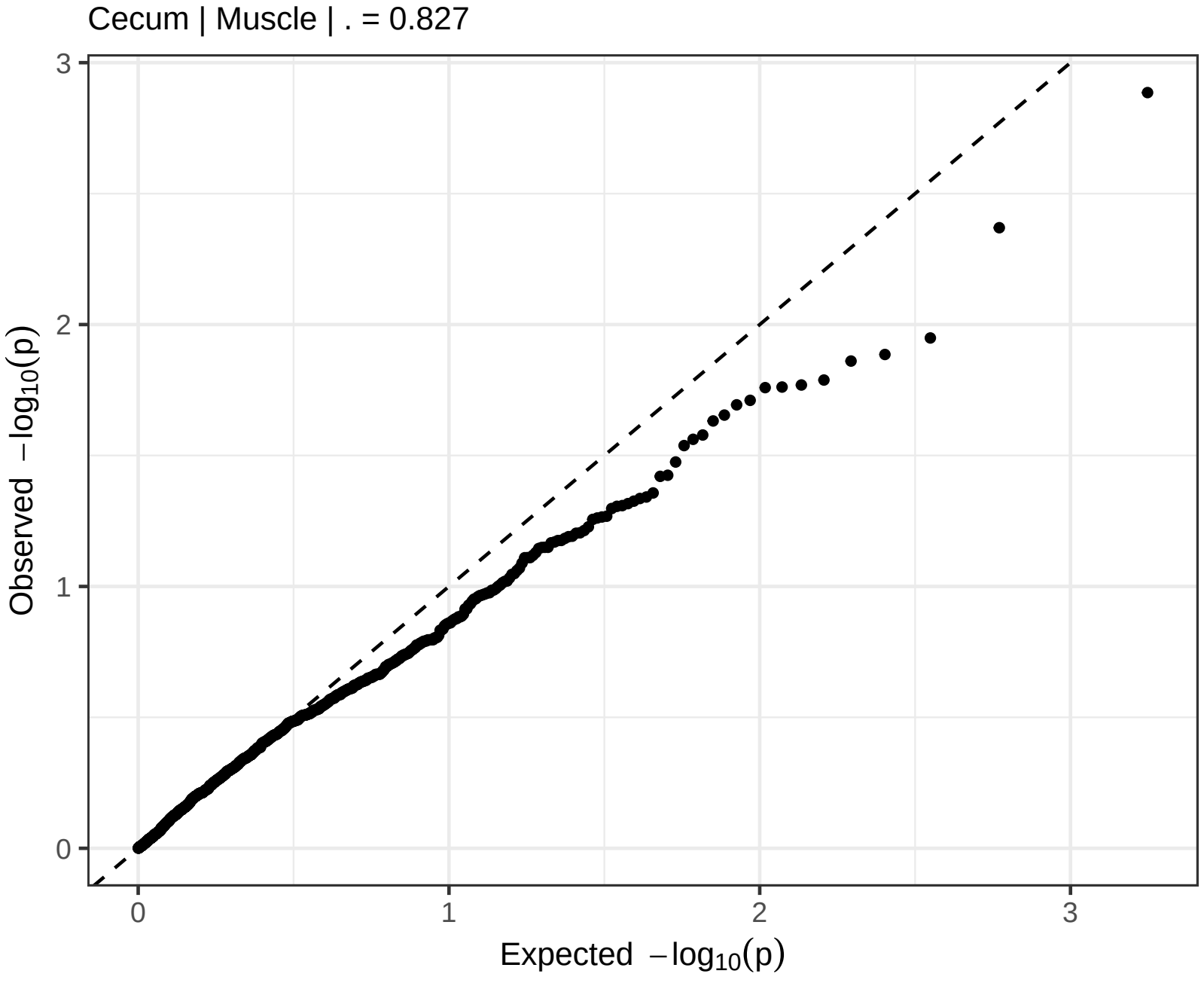

Collinsella

Cecum | Muscle | . = 1.036

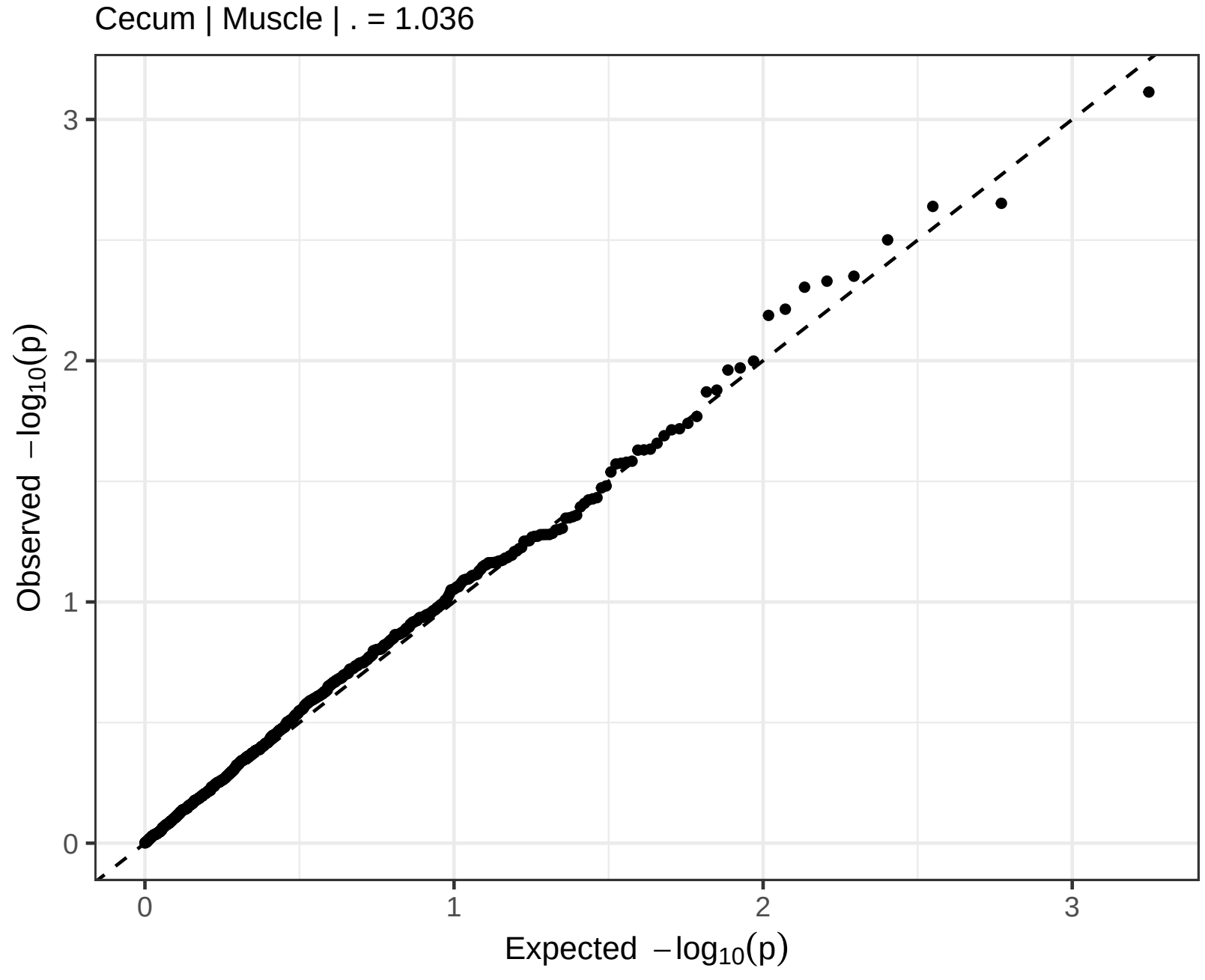

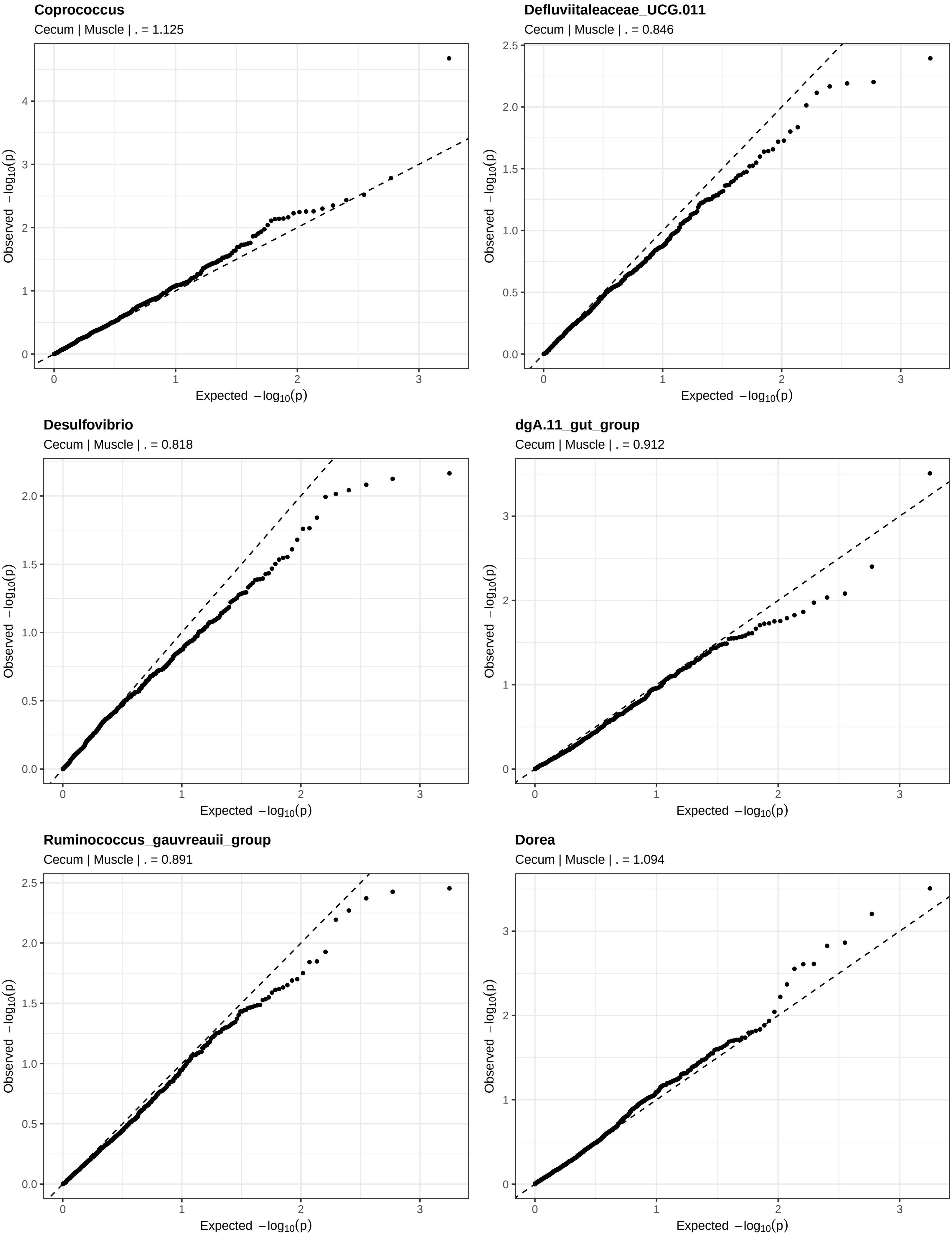

Coprococcus
Cecum | Muscle | . = 1.125
Defluviitaleaceae_UCG.011
Cecum | Muscle | . = 0.846
Desulfovibrio
Cecum | Muscle | . = 0.818
dgA.11_gut_group
Cecum | Muscle | . = 0.912
Ruminococcus_gauvreauii_group
Cecum | Muscle | . = 0.891
Dorea
Cecum | Muscle | . = 1.094
Observed $-\log_{10}(p)$
Expected $-\log_{10}(p)$

Eisenbergiella

Cecum | Muscle | . = 1.003

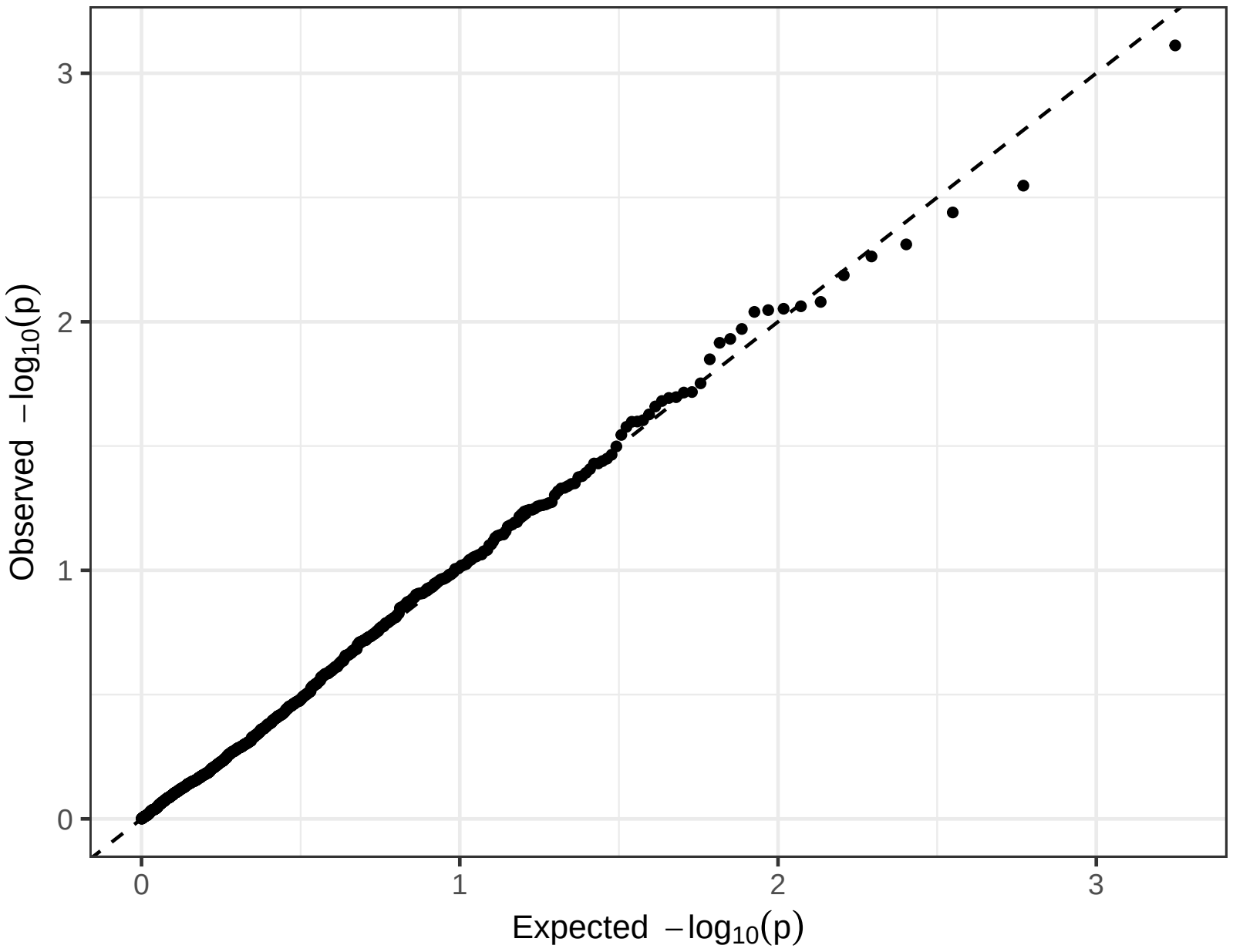

Elusimicrobium

Cecum | Muscle | . = 0.929

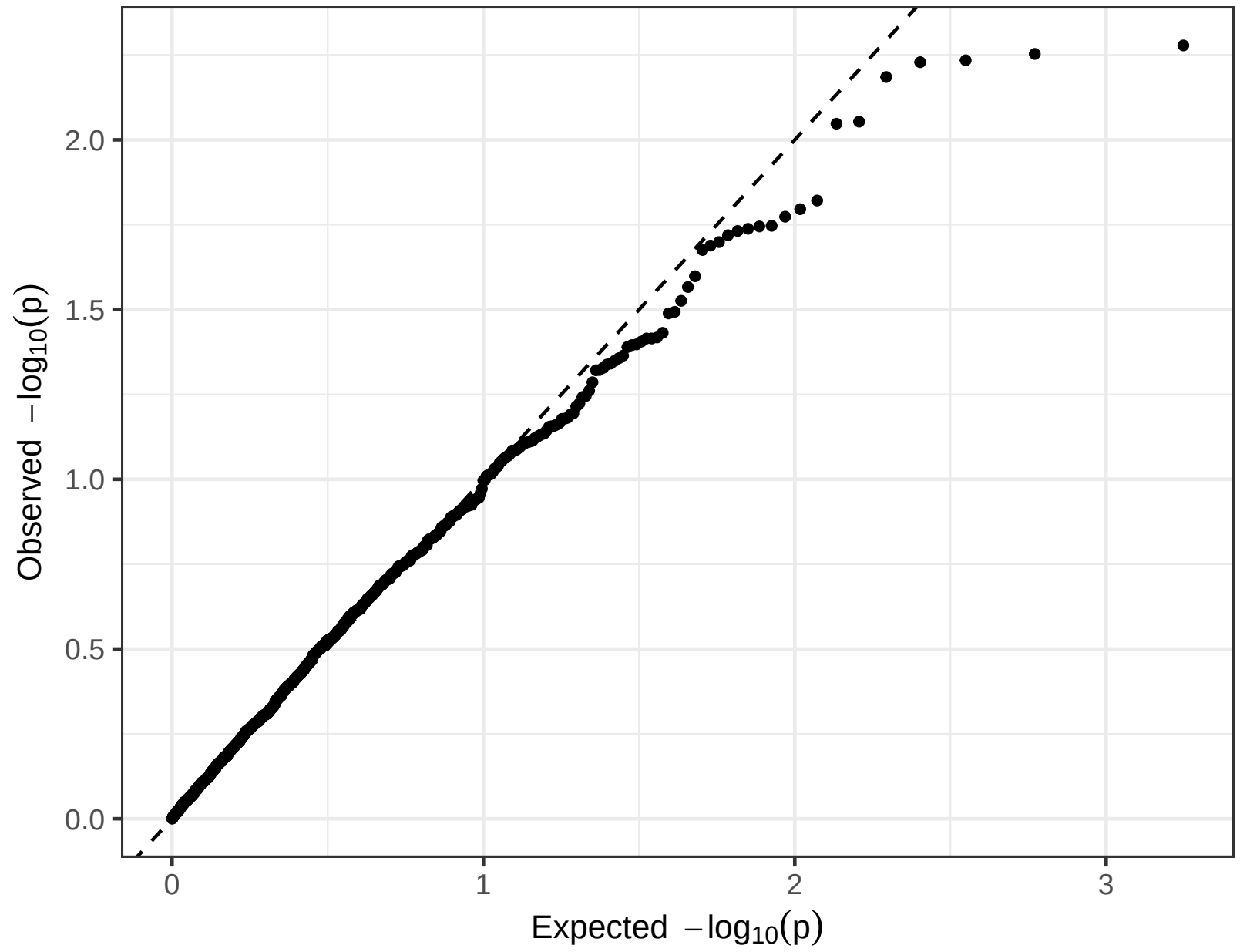

Enterocloster

Cecum | Muscle | . = 1.141

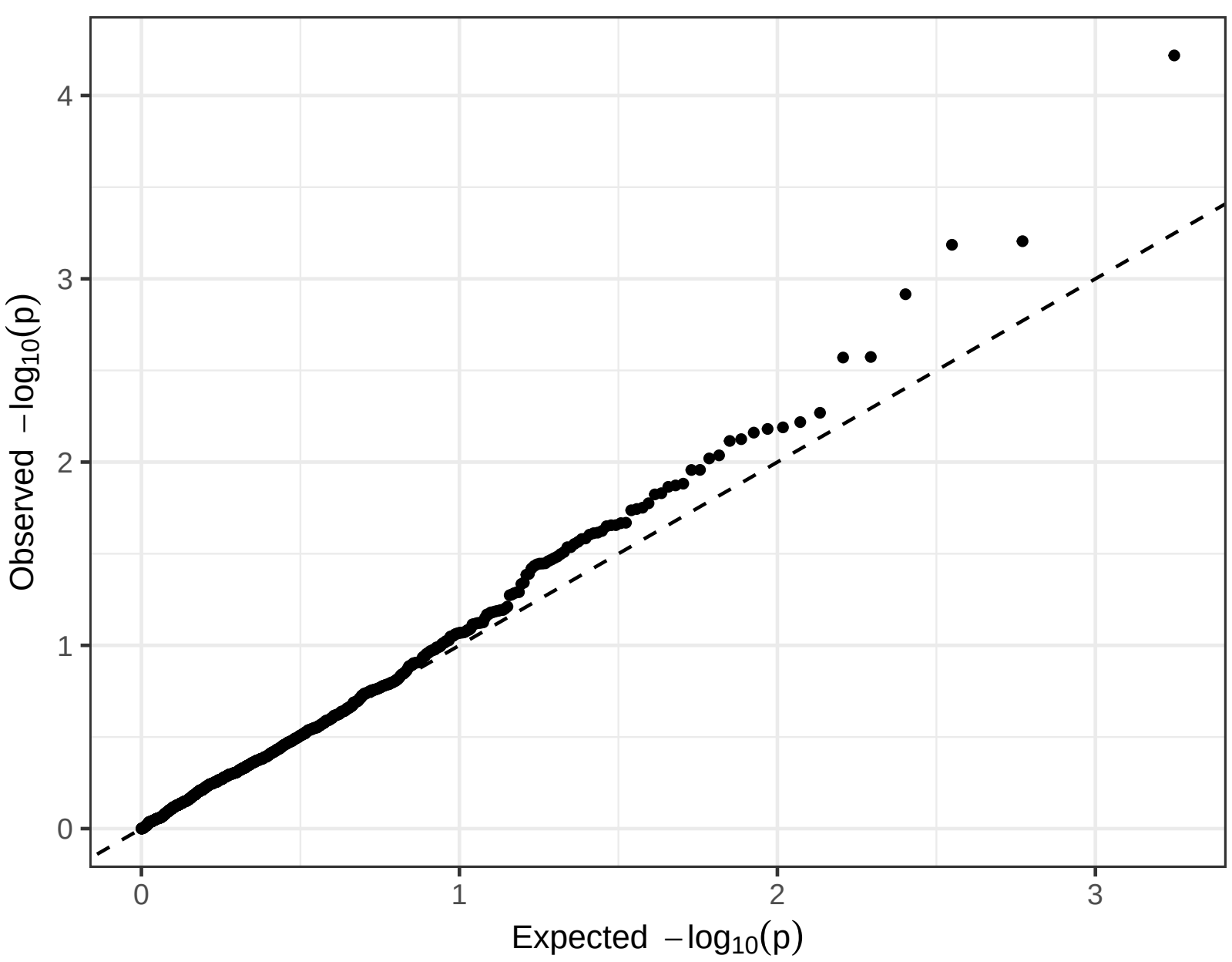

Epulopiscium

Cecum | Muscle | . = 0.855

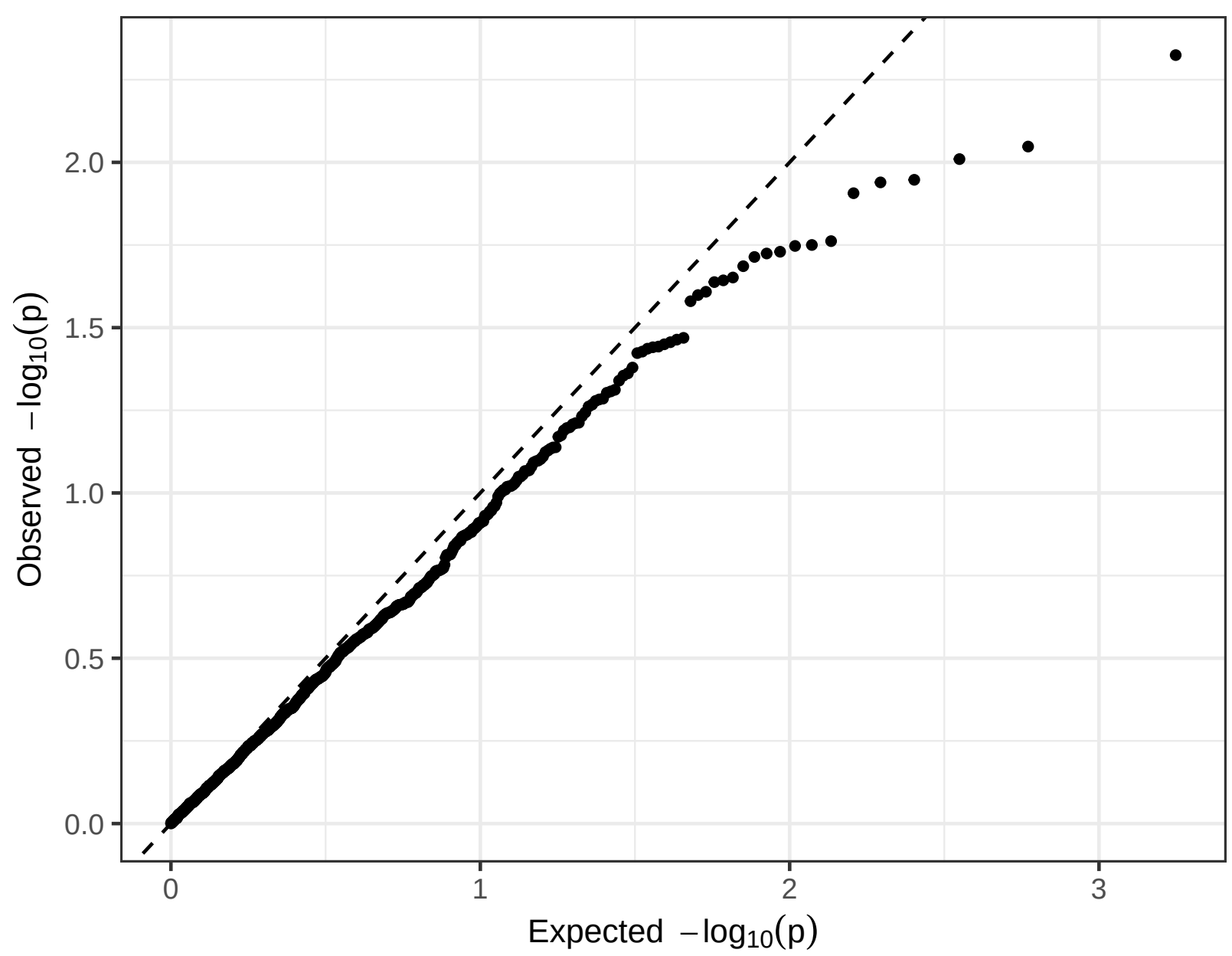

Erysipelotrichaceae_UCG.003

Cecum | Muscle | . = 0.985

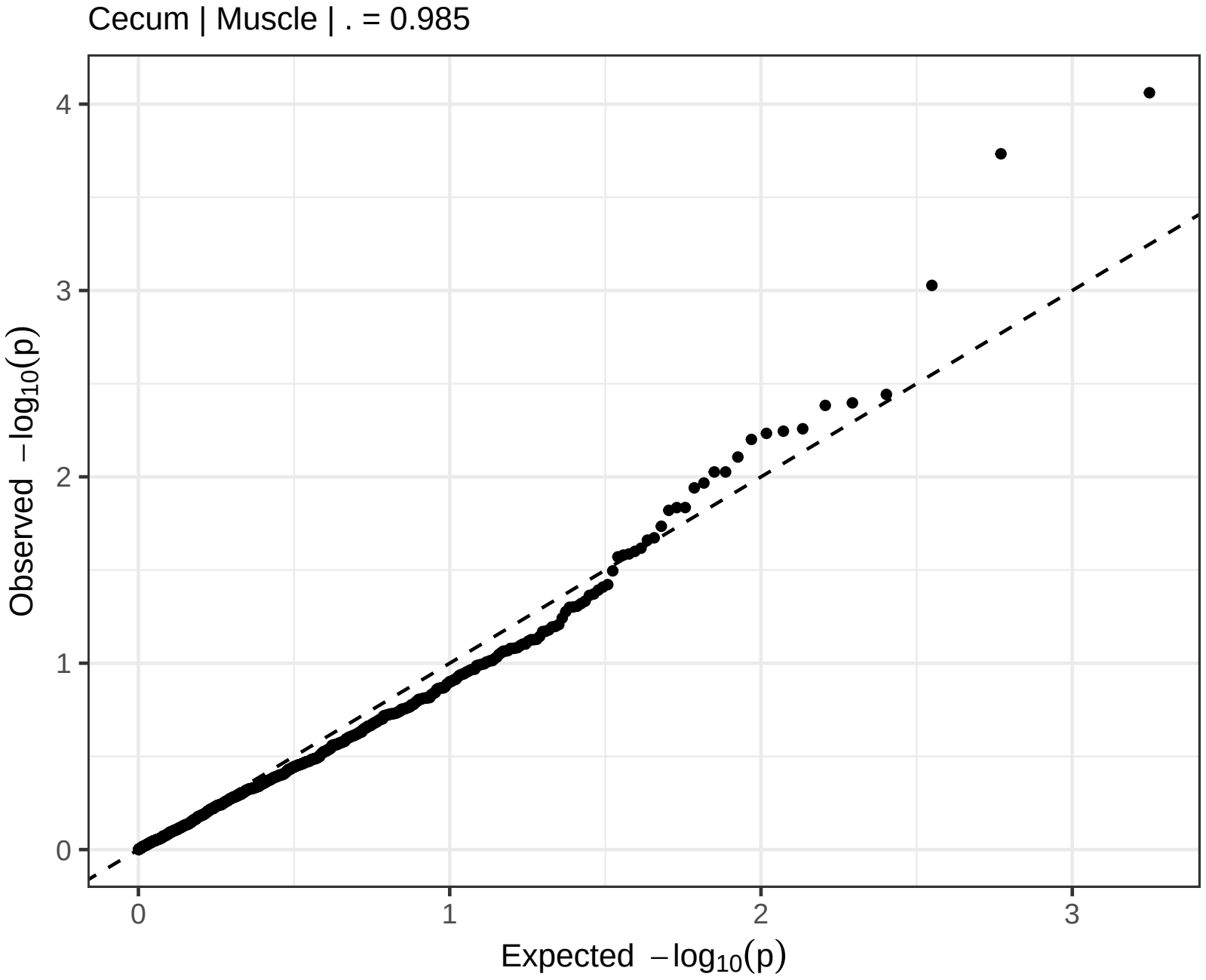

Escherichia.Shigella

Cecum | Muscle | . = 0.895

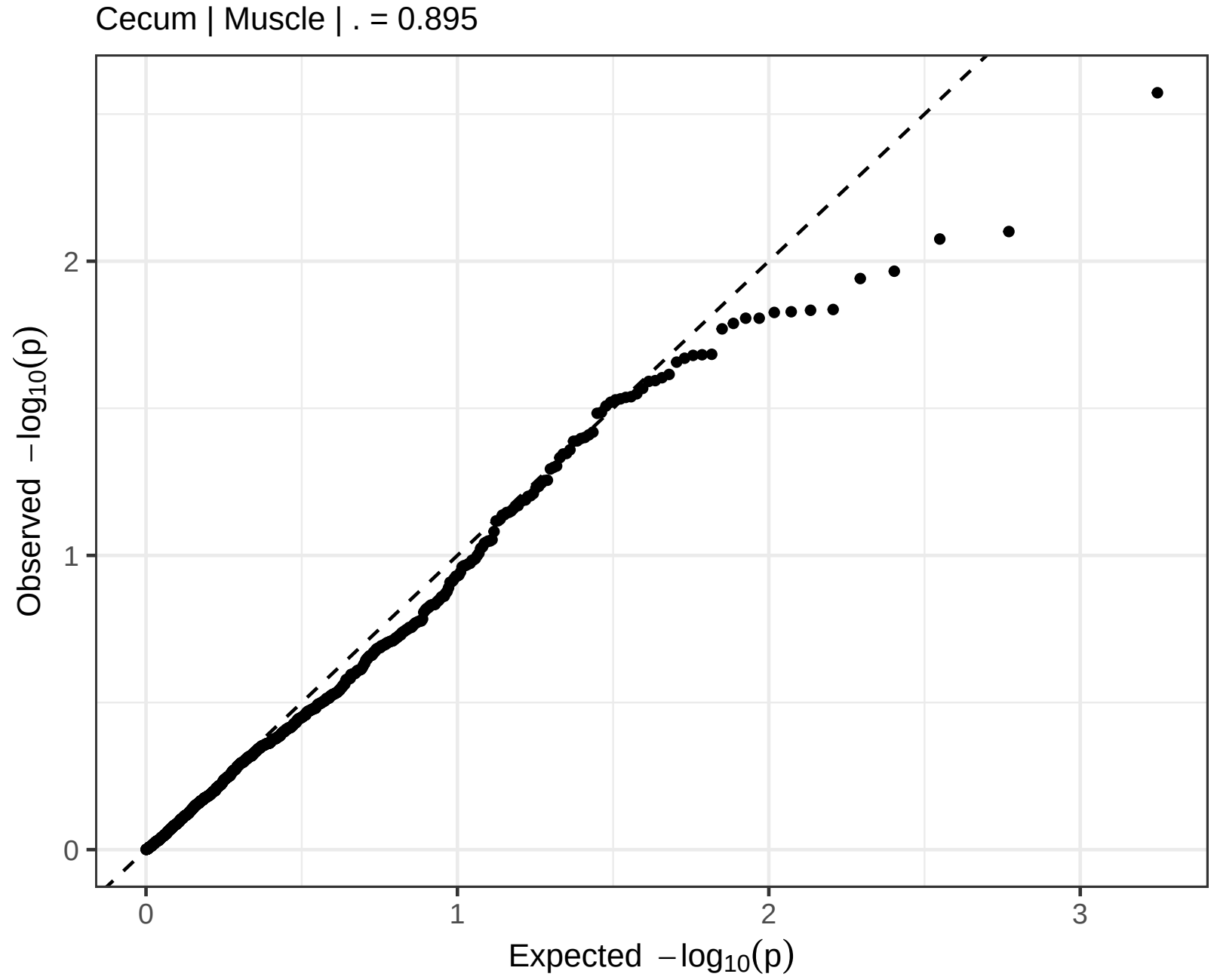

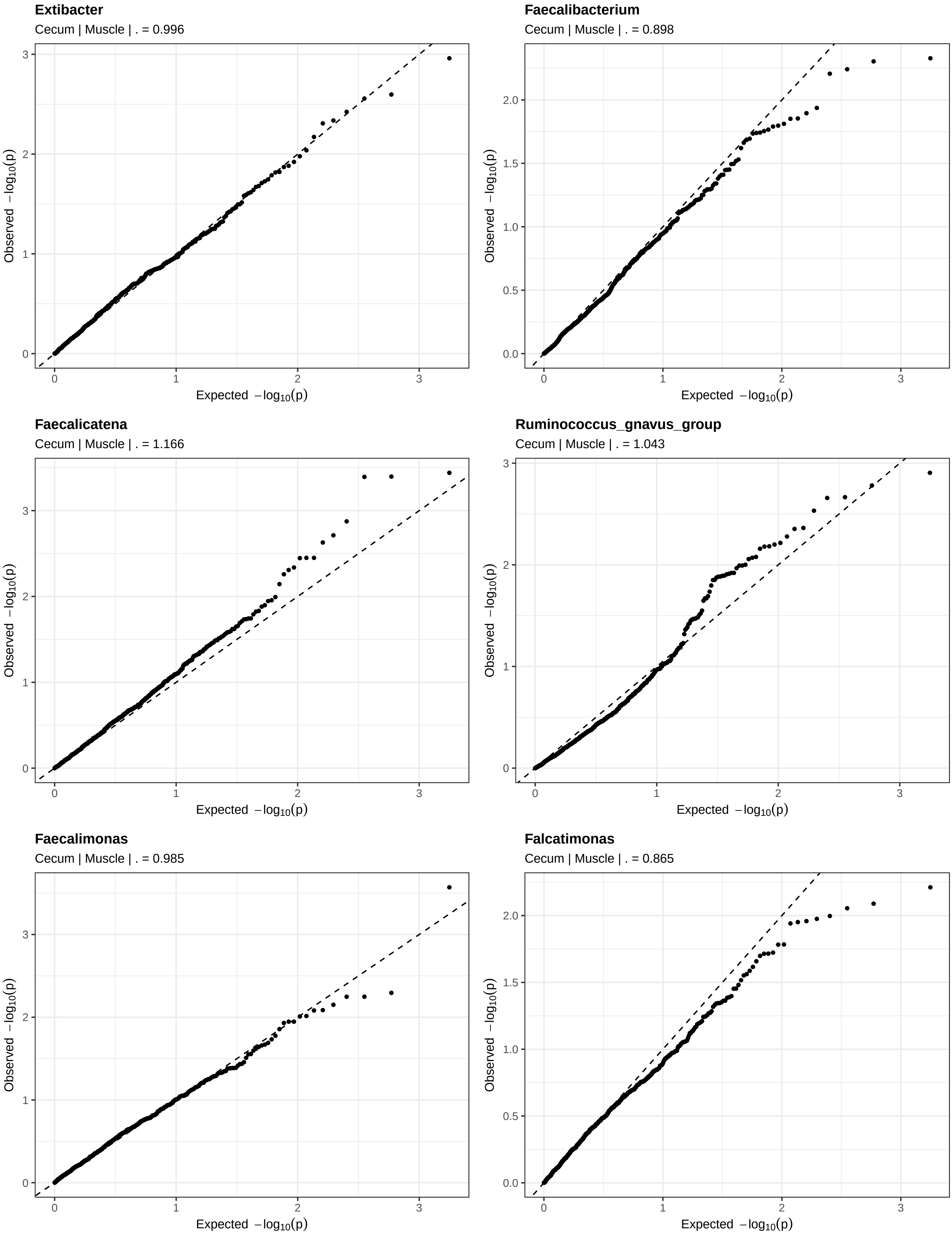
Extibacter
Cecum | Muscle | . = 0.996
Faecalibacterium
Cecum | Muscle | . = 0.898
Faecalicatena
Cecum | Muscle | . = 1.166
Ruminococcus_gnavus_group
Cecum | Muscle | . = 1.043
Faecalimonas
Cecum | Muscle | . = 0.985
Falcatimonas
Cecum | Muscle | . = 0.865
Observed $-\log_{10}(p)$
Expected $-\log_{10}(p)$

Family_XIII_AD3011_group

Cecum | Muscle | . = 0.874

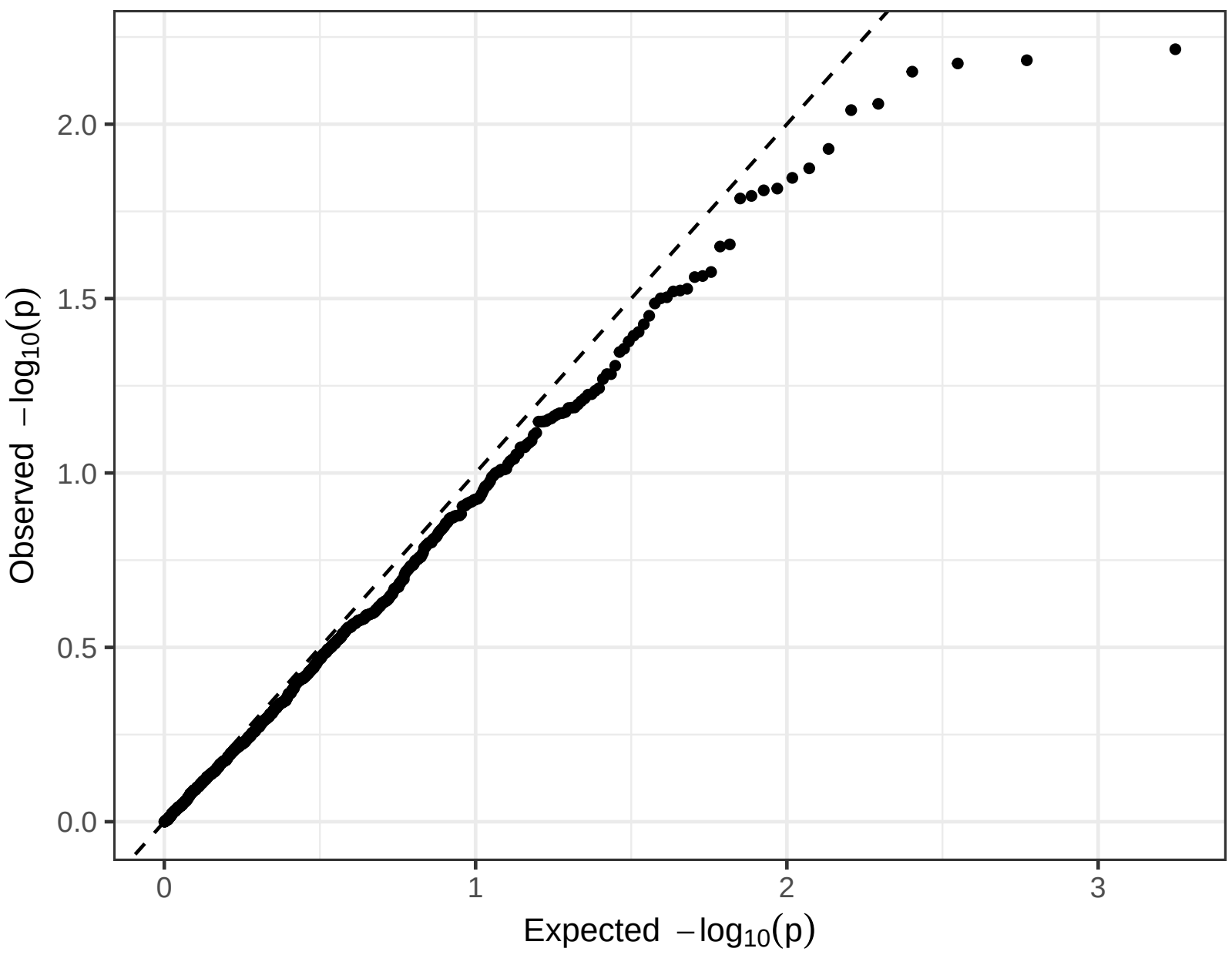

Family_XIII_UCG.001

Cecum | Muscle | . = 0.997

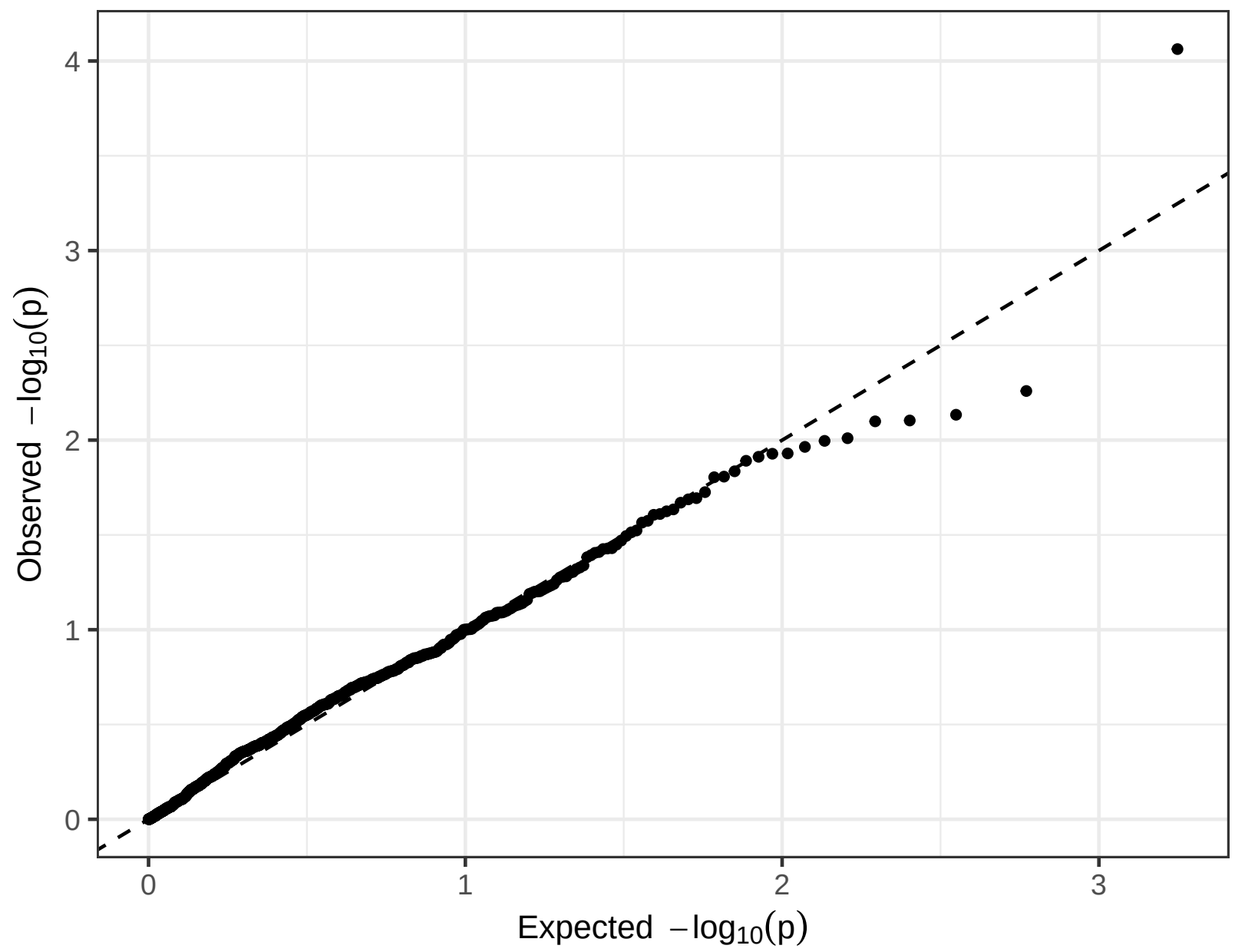

FD2005

Cecum | Muscle | . = 0.953

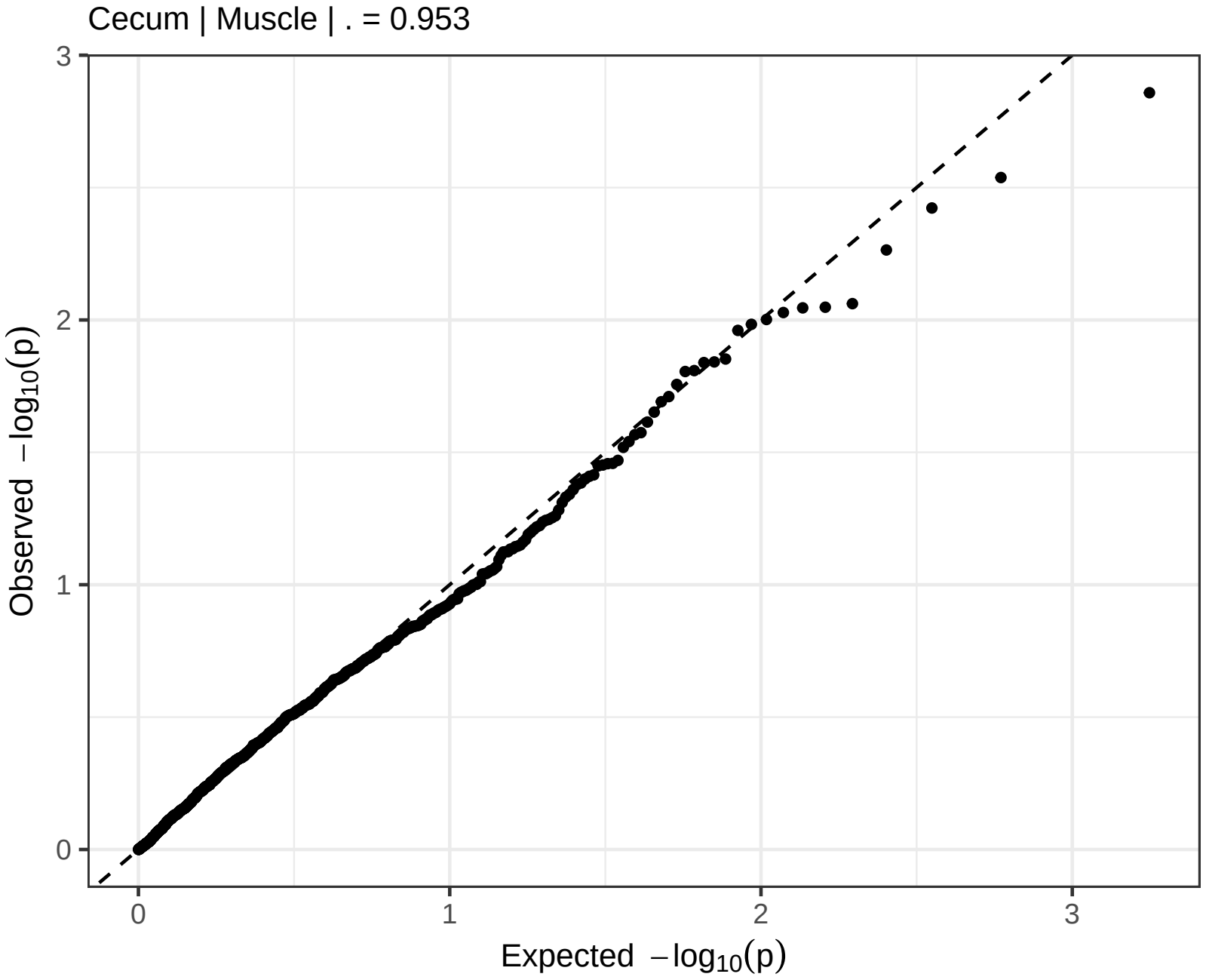

Fibrobacter

Cecum | Muscle | . = 0.876

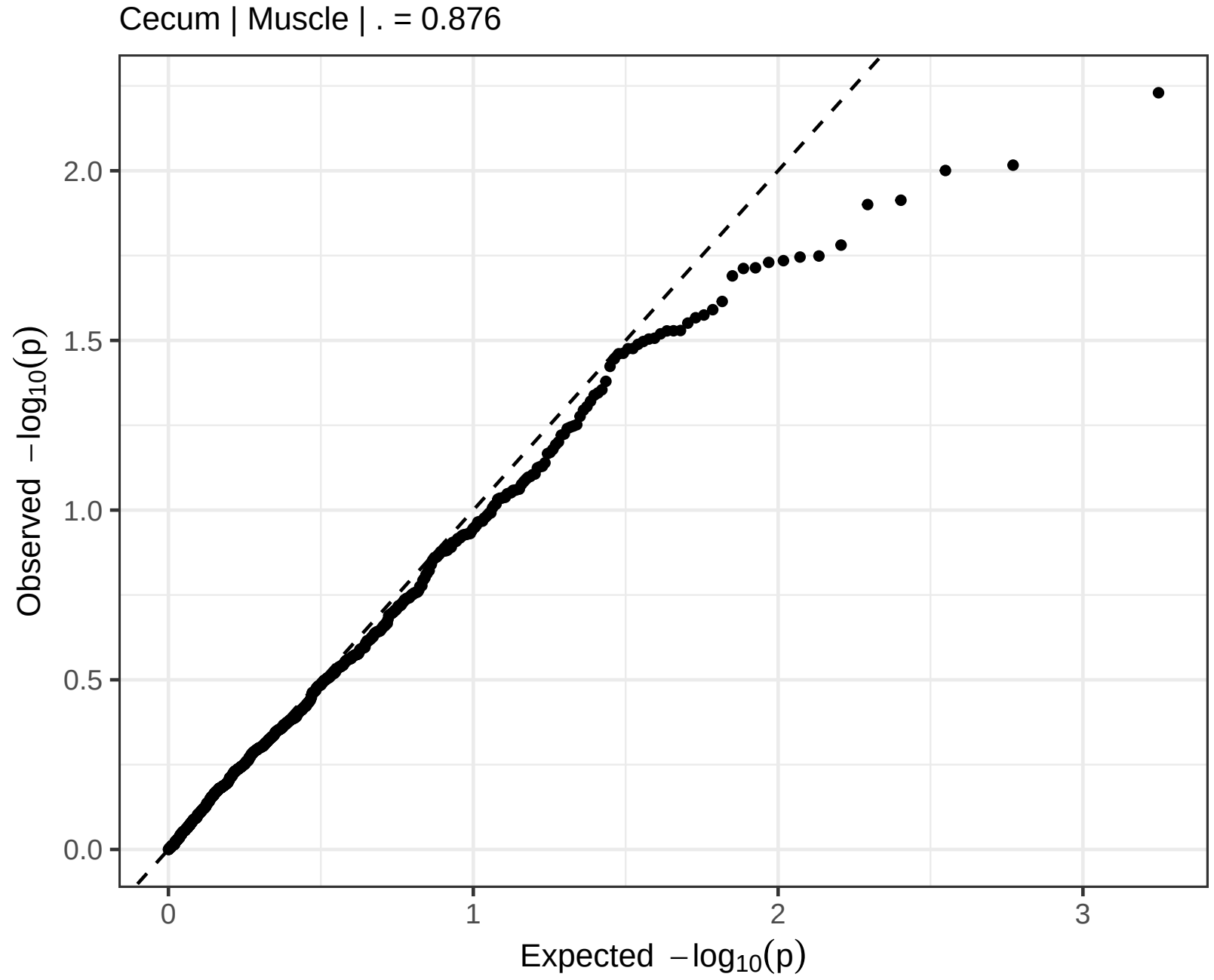

Fournierella

Cecum | Muscle | . = 0.9

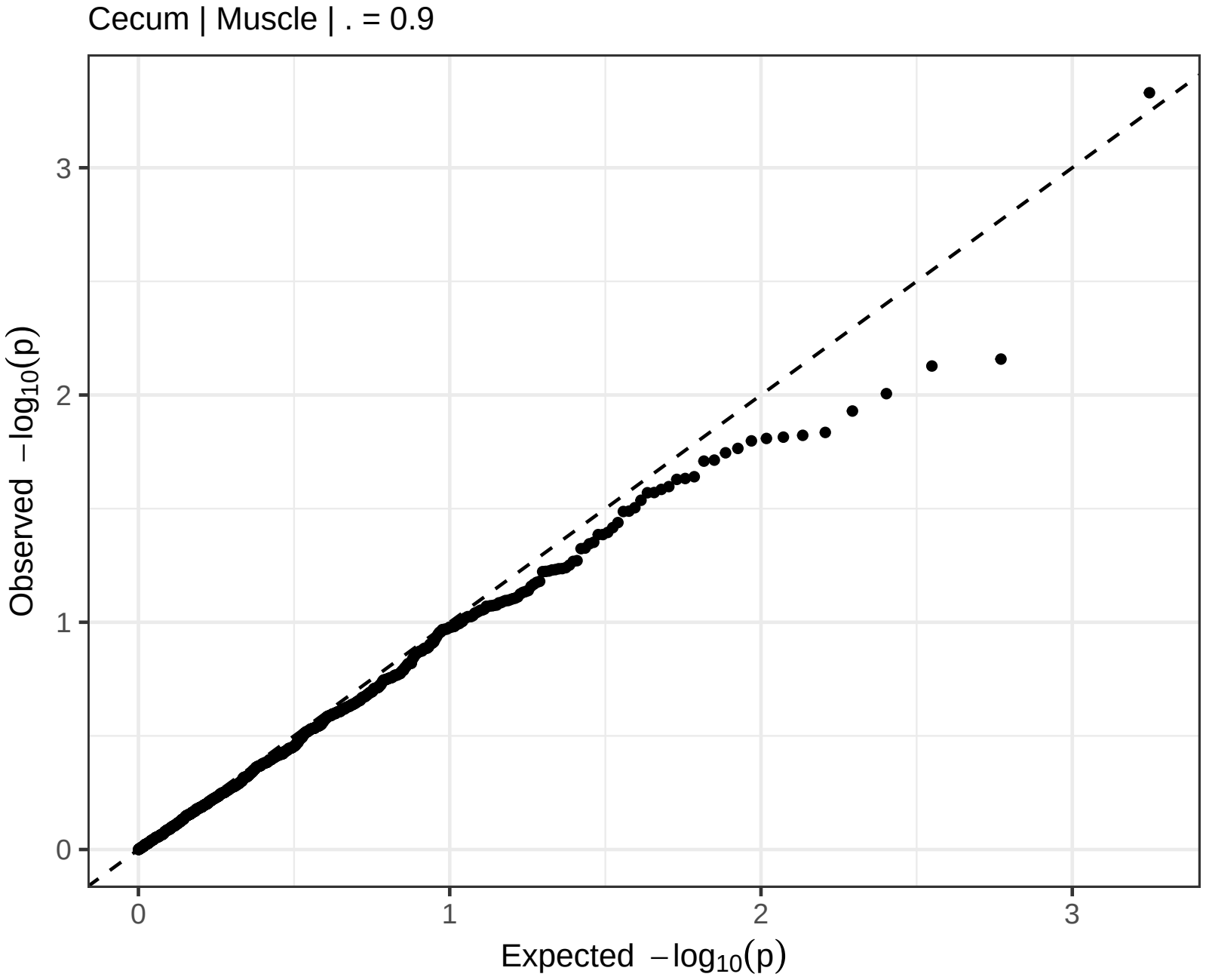

Frisingicoccus

Cecum | Muscle | . = 0.904

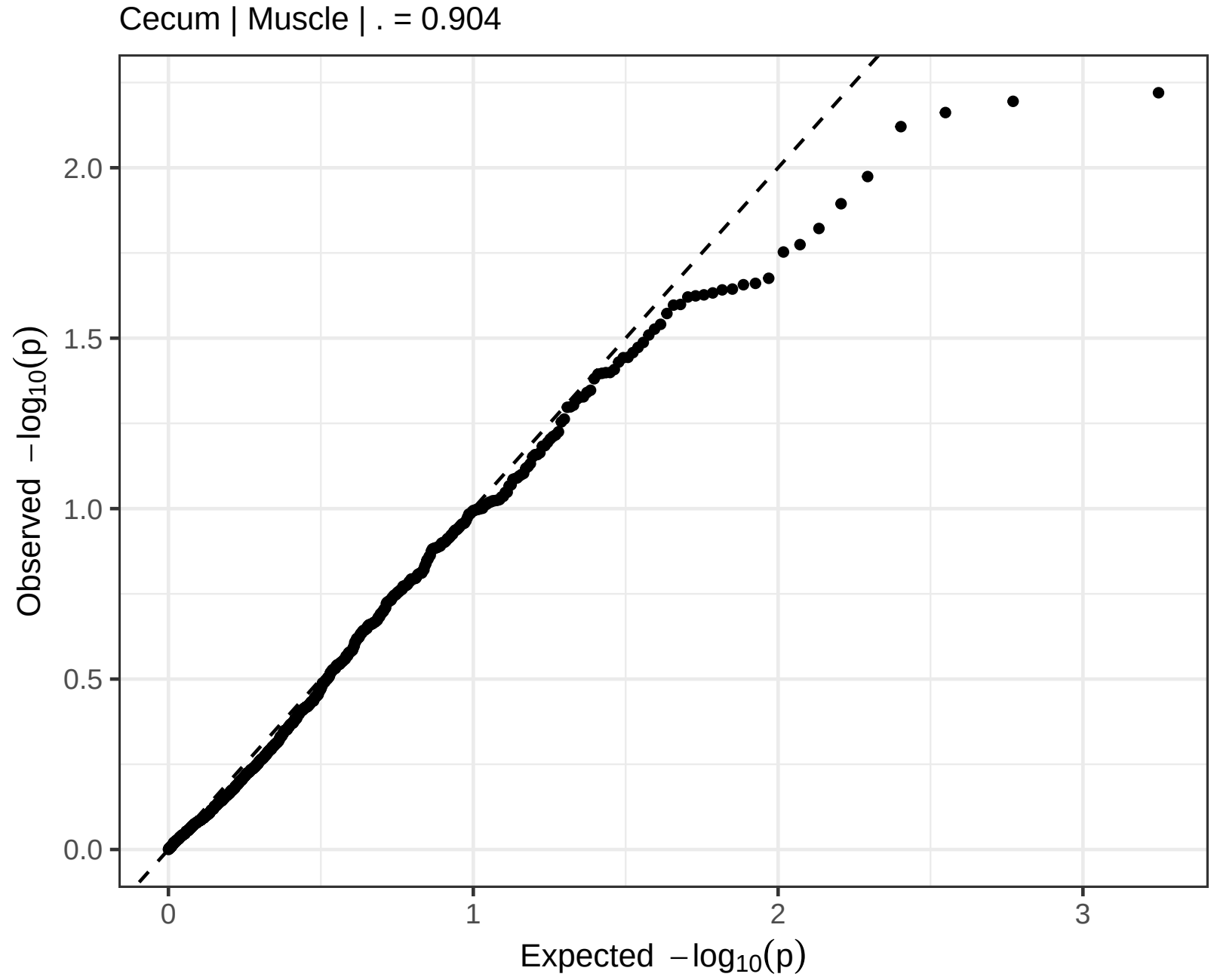

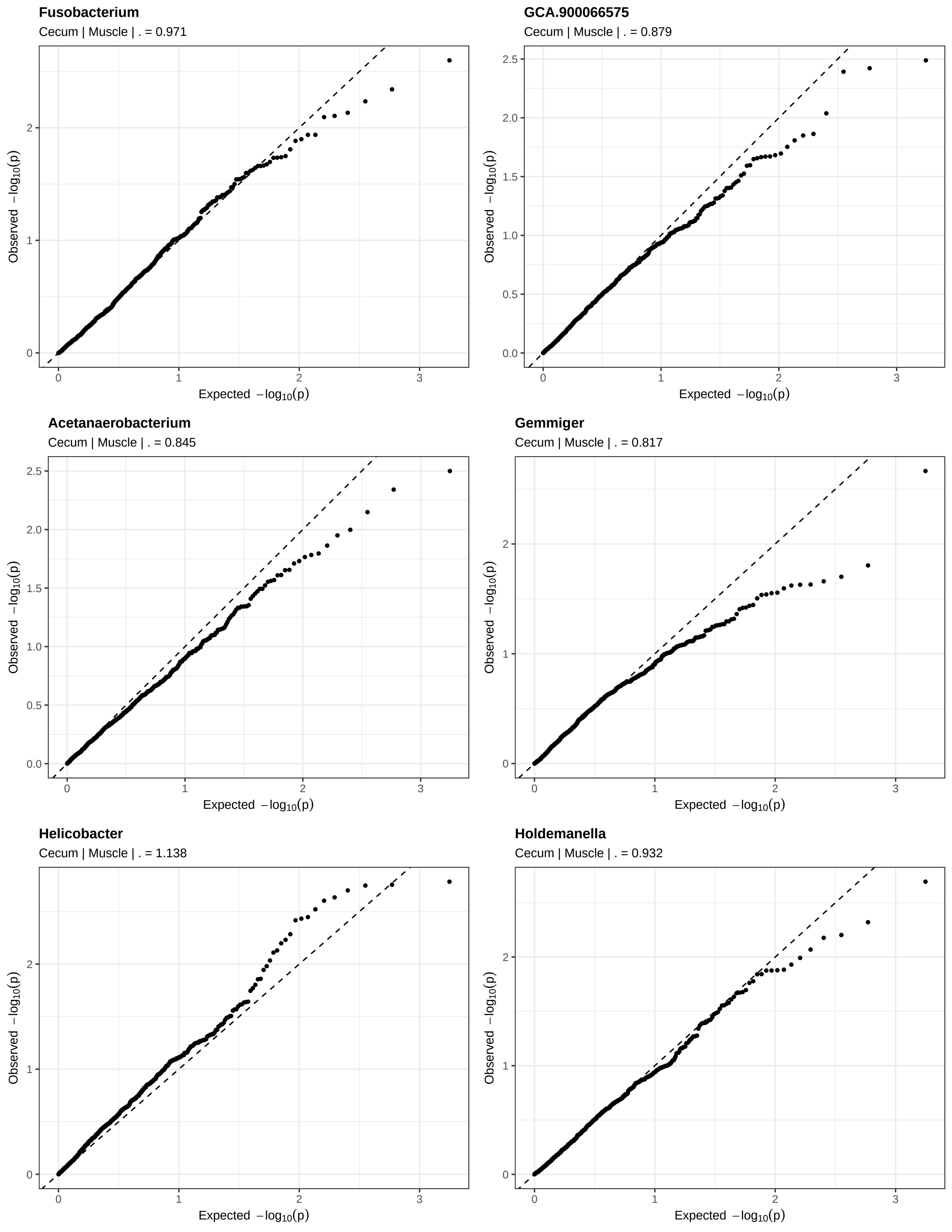
Fusobacterium
Cecum | Muscle | . = 0.971
GCA.900066575
Cecum | Muscle | . = 0.879
Acetanaerobacterium
Cecum | Muscle | . = 0.845
Gemmiger
Cecum | Muscle | . = 0.817
Helicobacter
Cecum | Muscle | . = 1.138
Holdemanella
Cecum | Muscle | . = 0.932
Observed $-\log_{10}(p)$
Expected $-\log_{10}(p)$

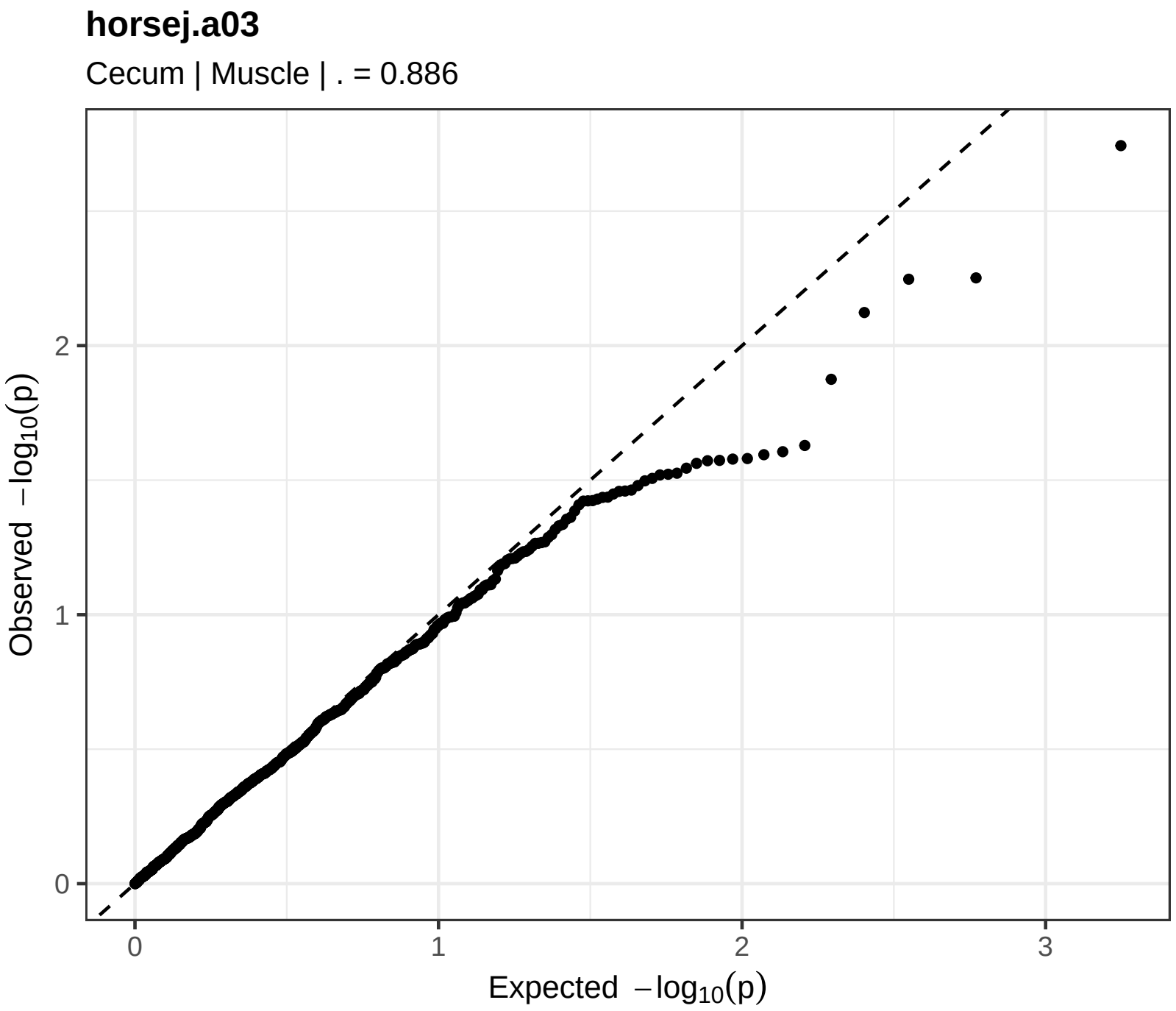
horsej.a03
Cecum | Muscle | . = 0.886
Observed −log10(p)
Expected −log10(p)
0
1
2
3

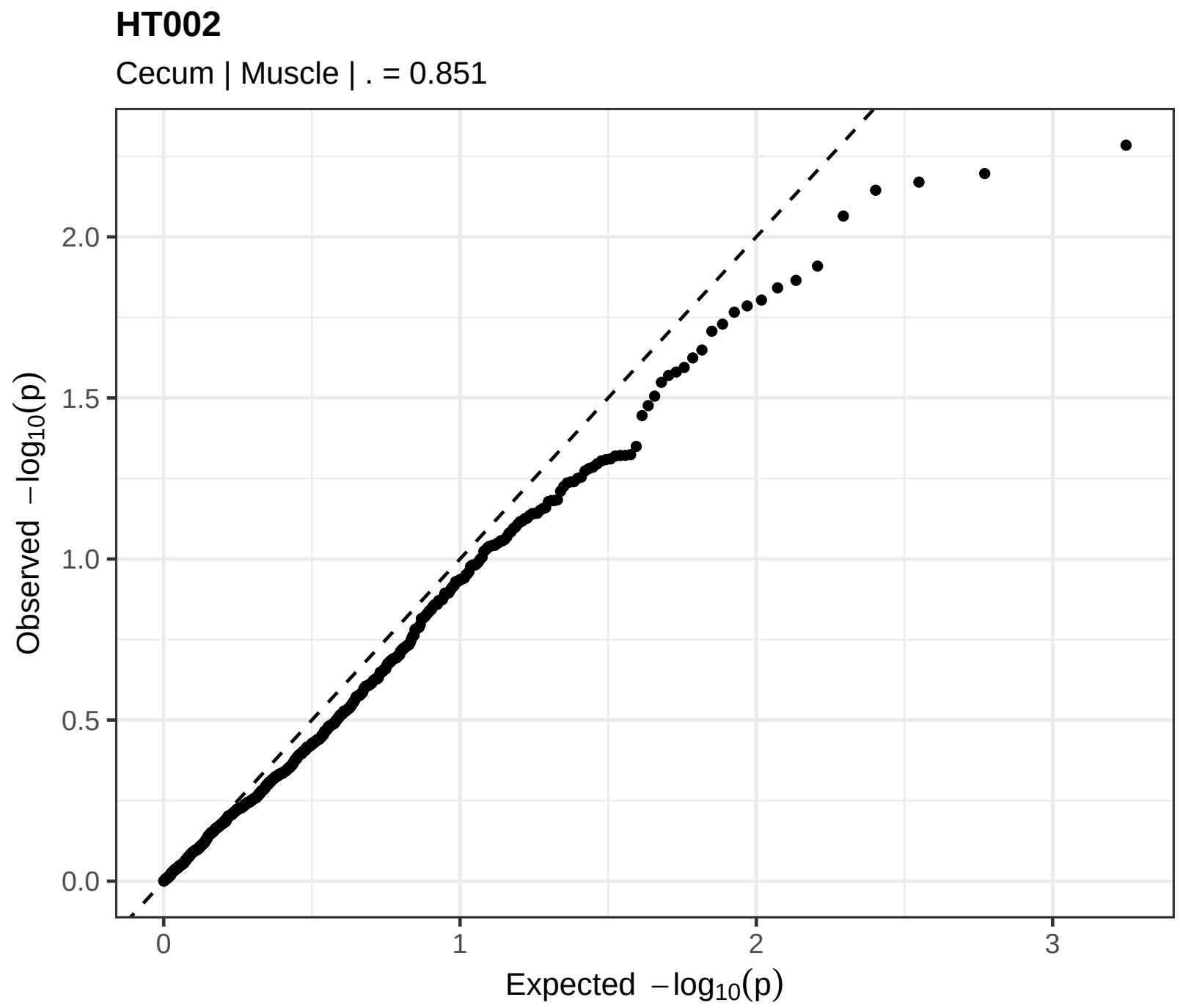
HT002
Cecum | Muscle | . = 0.851
Observed −log10(p)
Expected −log10(p)
0.0
0.5
1.0
1.5
2.0
0
1
2
3

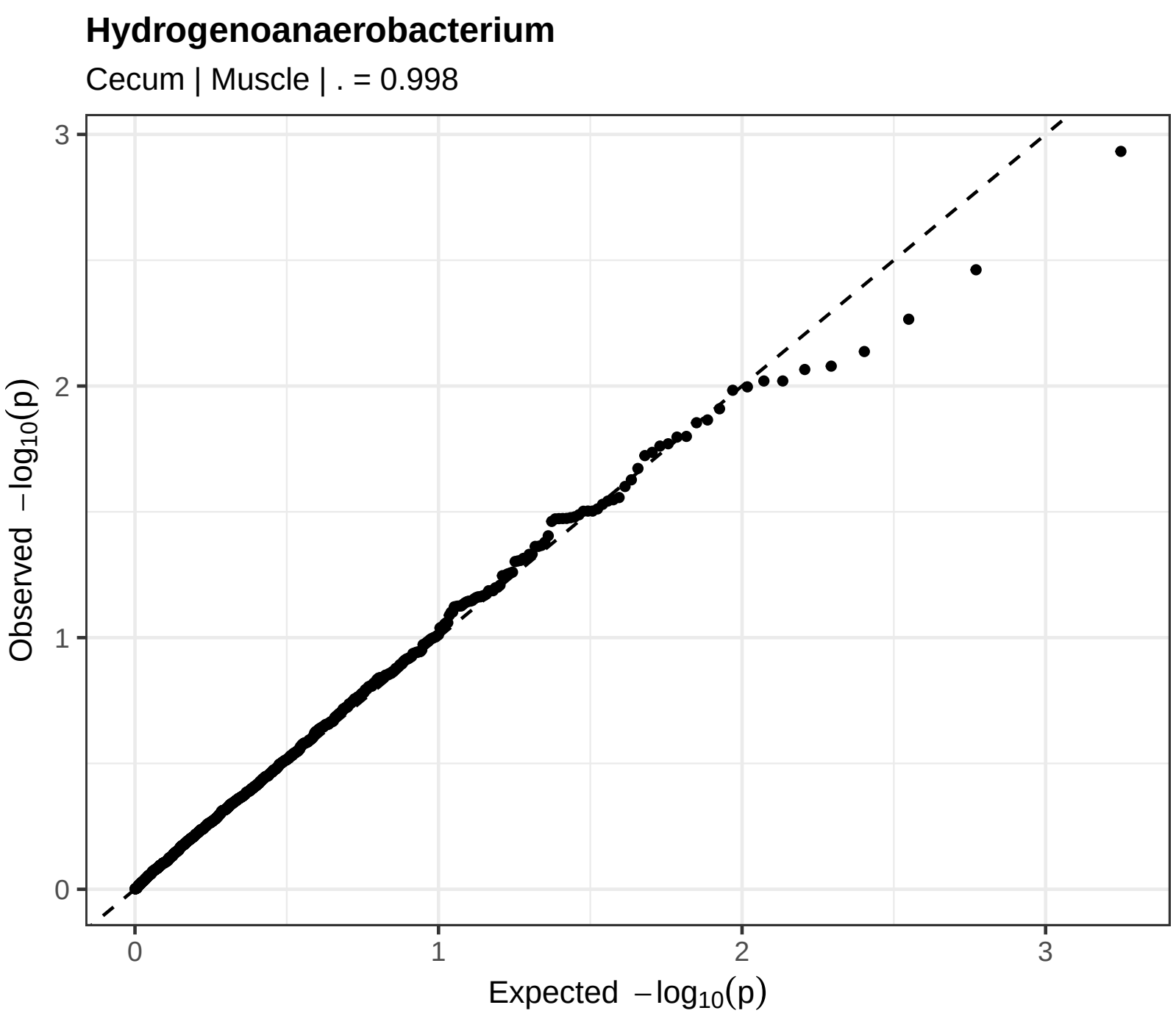
Hydrogenoanaerobacterium
Cecum | Muscle | . = 0.998
Observed −log10(p)
Expected −log10(p)
0
1
2
3

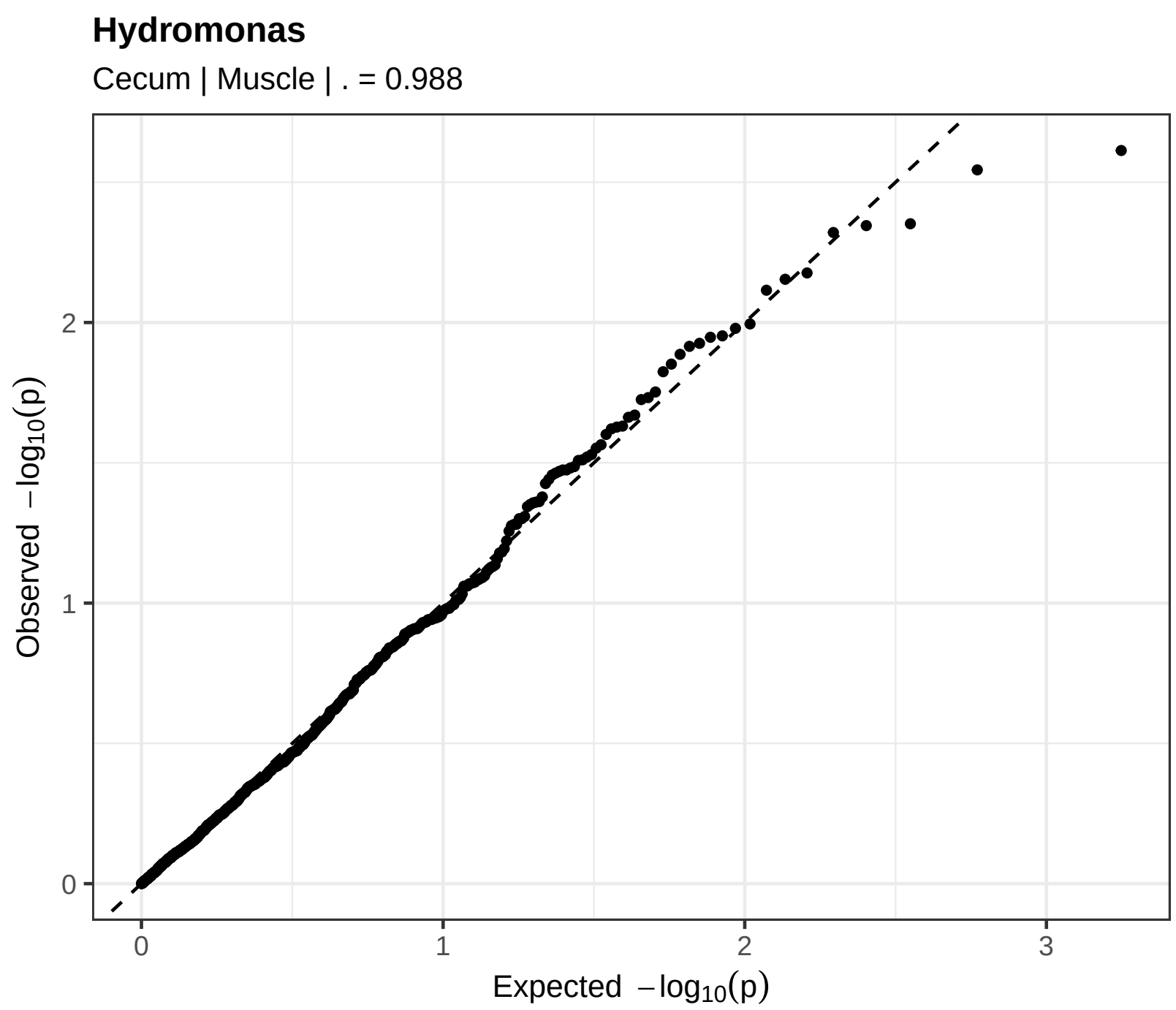
Hydromonas
Cecum | Muscle | . = 0.988
Observed −log10(p)
Expected −log10(p)
0
1
2
3

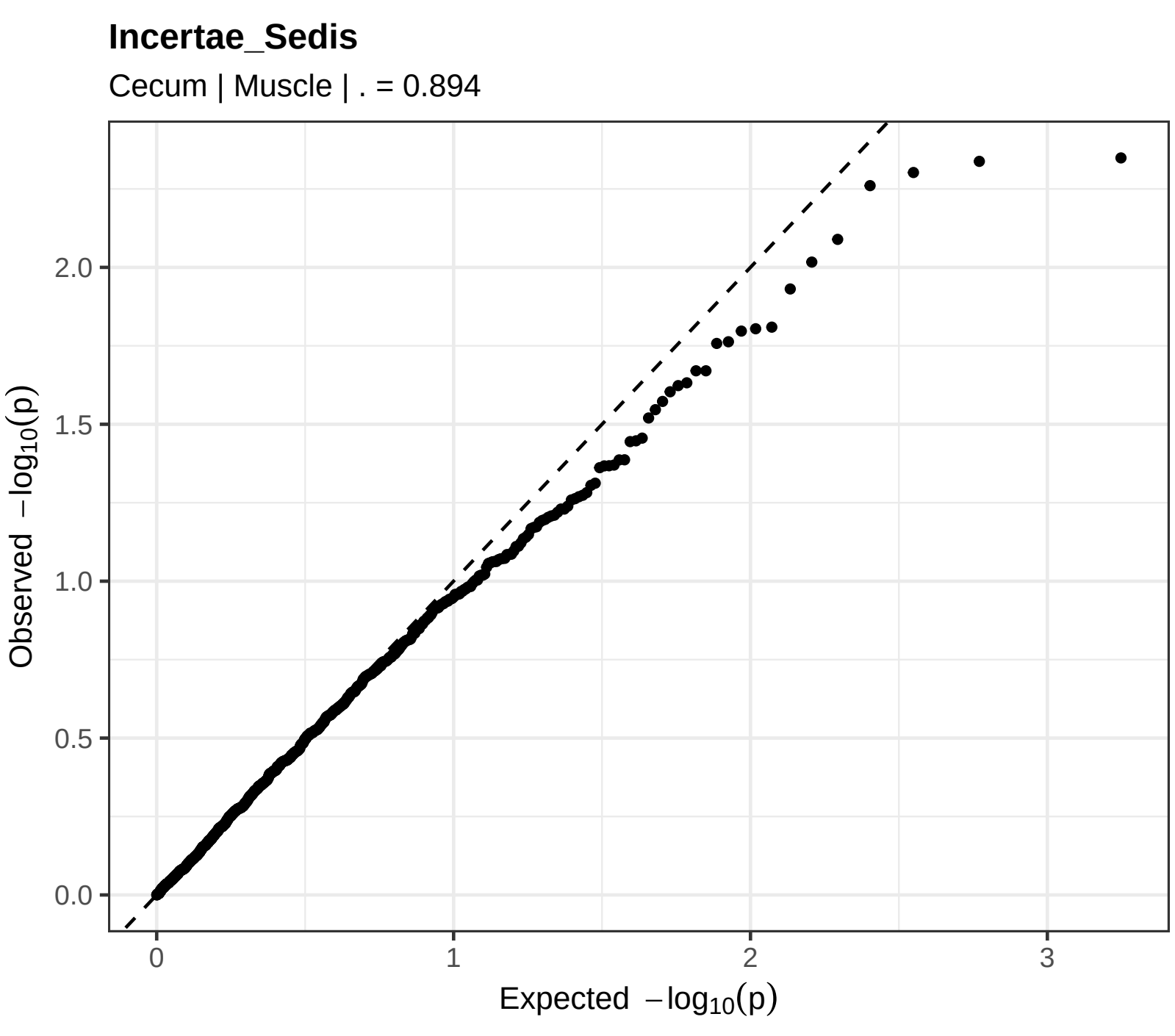
Incertae_Sedis
Cecum | Muscle | . = 0.894
Observed −log10(p)
Expected −log10(p)
0.0
0.5
1.0
1.5
2.0
0
1
2
3

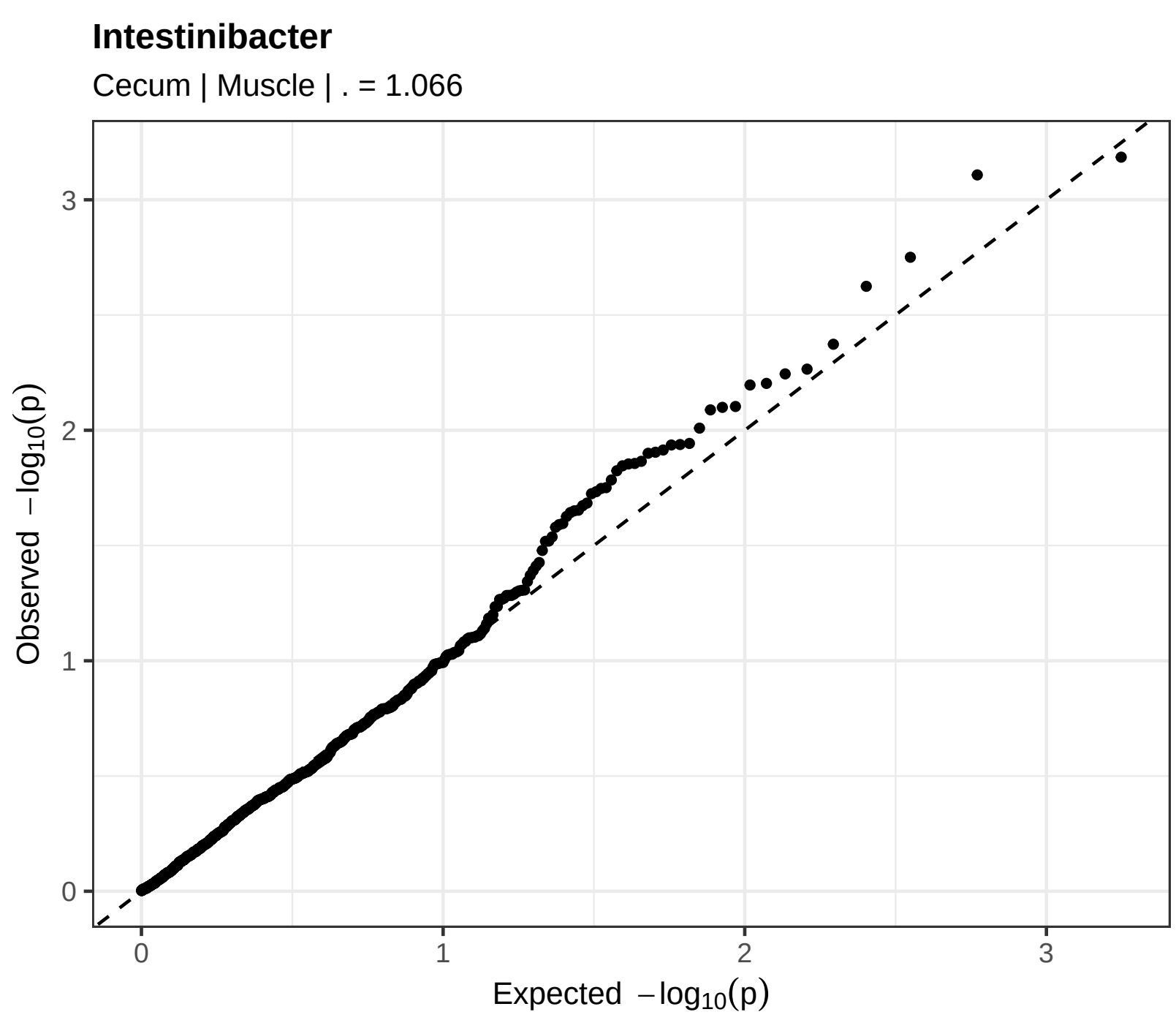
Intestinibacter
Cecum | Muscle | . = 1.066
Observed −log10(p)
Expected −log10(p)
0
1
2
3

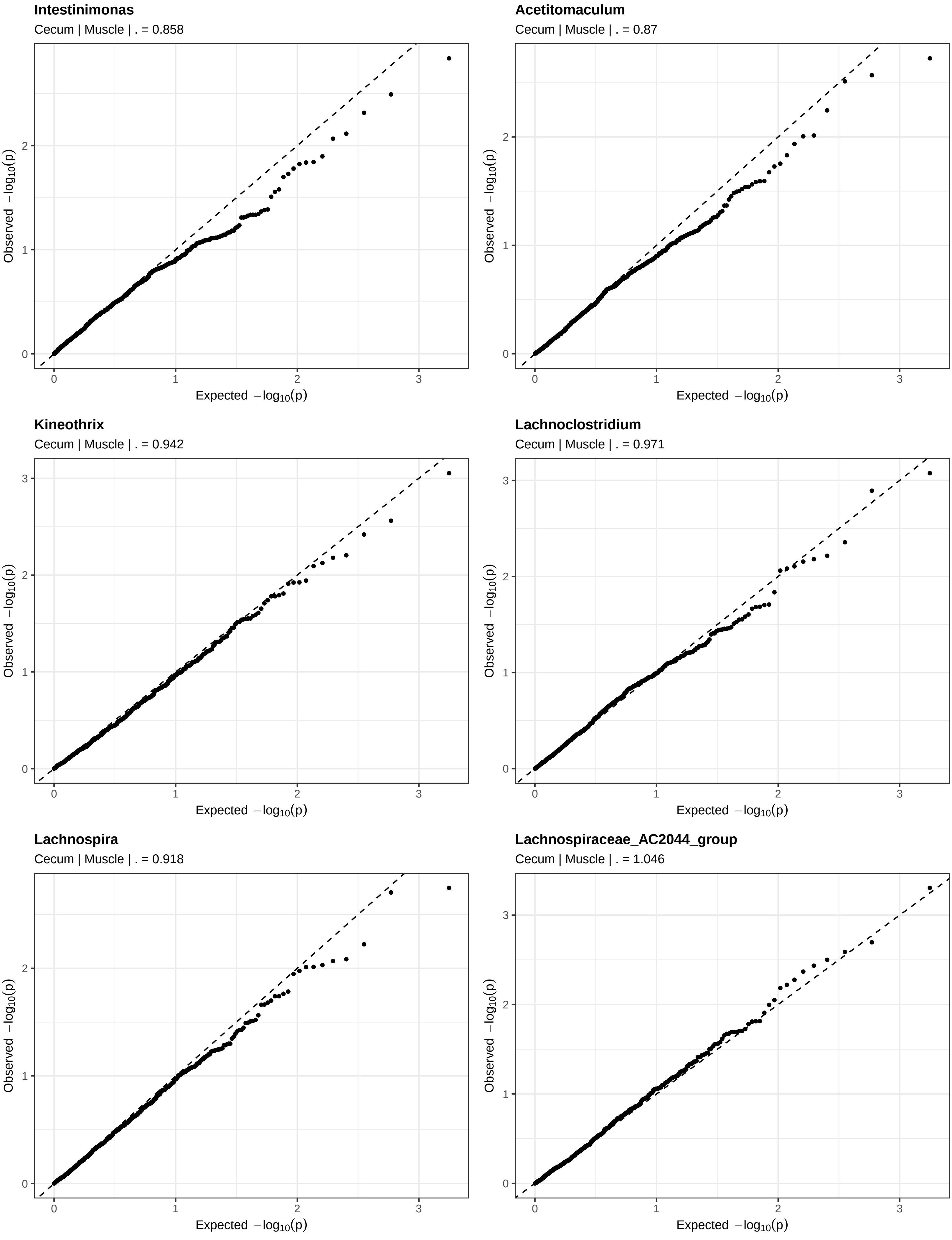
Intestinimonas
Cecum | Muscle | . = 0.858
Acetitomaculum
Cecum | Muscle | . = 0.87
Kineothrix
Cecum | Muscle | . = 0.942
Lachnoclostridium
Cecum | Muscle | . = 0.971
Lachnospira
Cecum | Muscle | . = 0.918
Lachnospiraceae_AC2044_group
Cecum | Muscle | . = 1.046
Observed $-\log_{10}(p)$
Expected $-\log_{10}(p)$

Lachnospiraceae_FCS020_group

Cecum | Muscle | . = 0.876

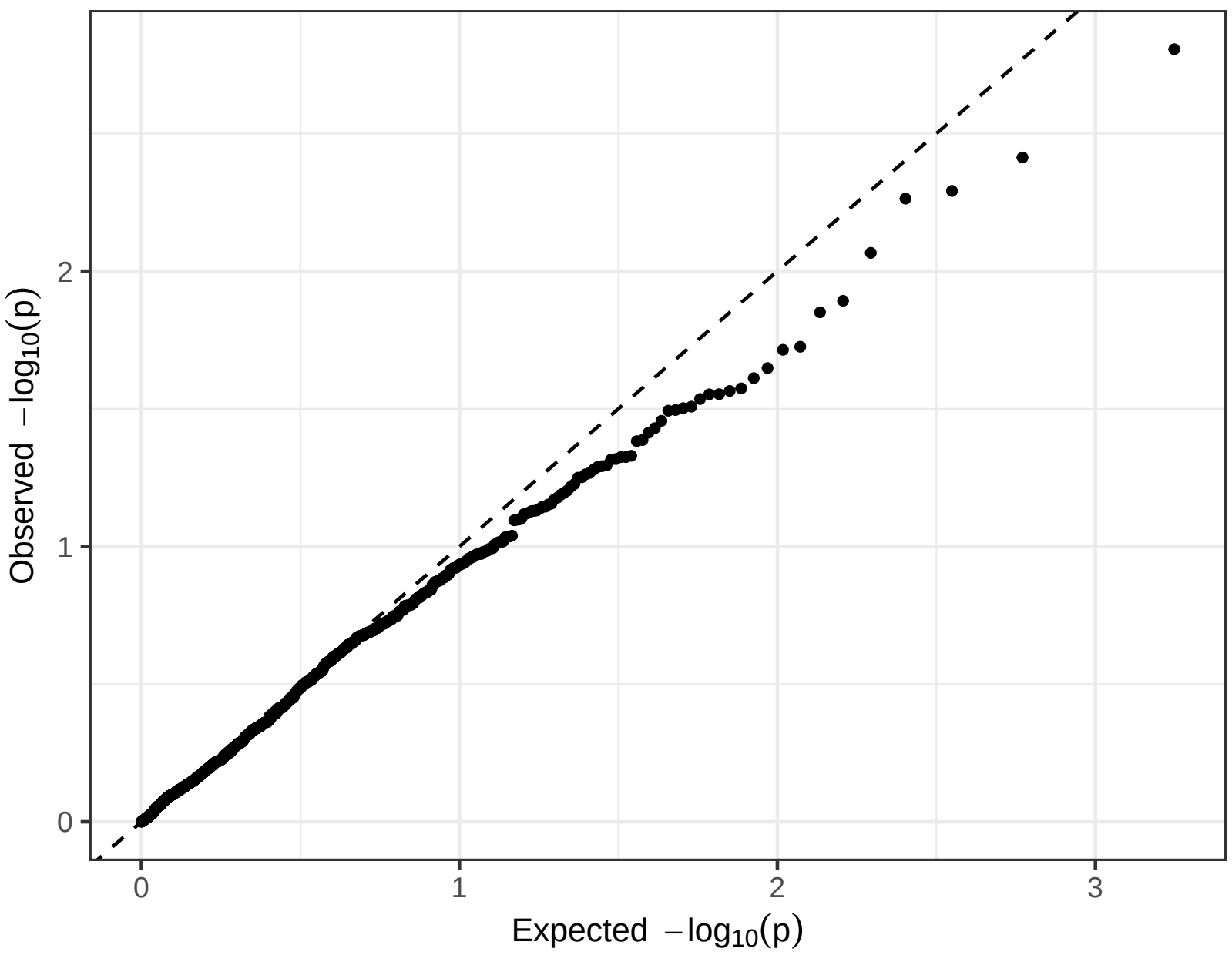

Lachnospiraceae_ND3007_group

Cecum | Muscle | . = 0.935

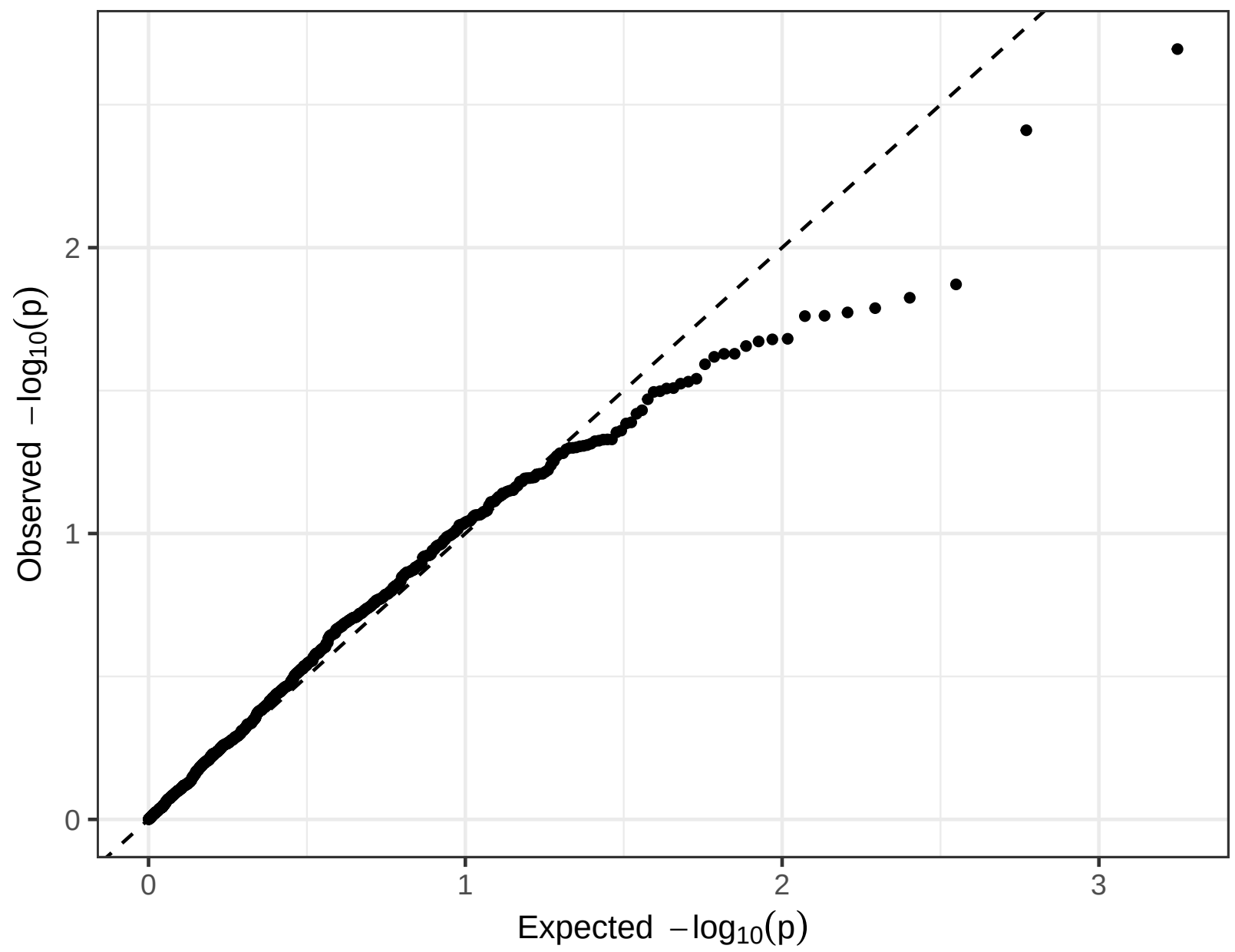

Lachnospiraceae_NK4A136_group

Cecum | Muscle | . = 0.875

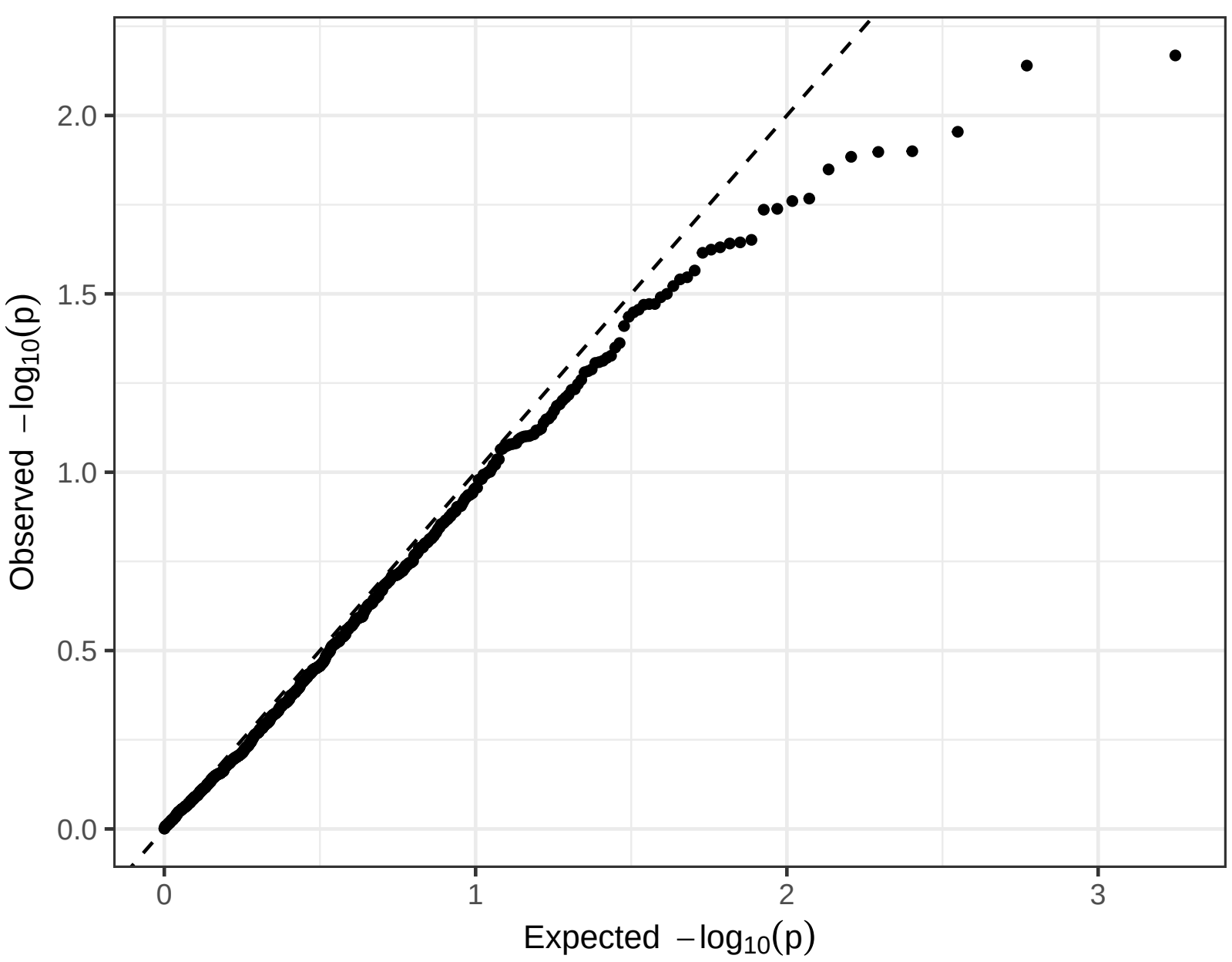

Lachnospiraceae_NK4B4_group

Cecum | Muscle | . = 0.966

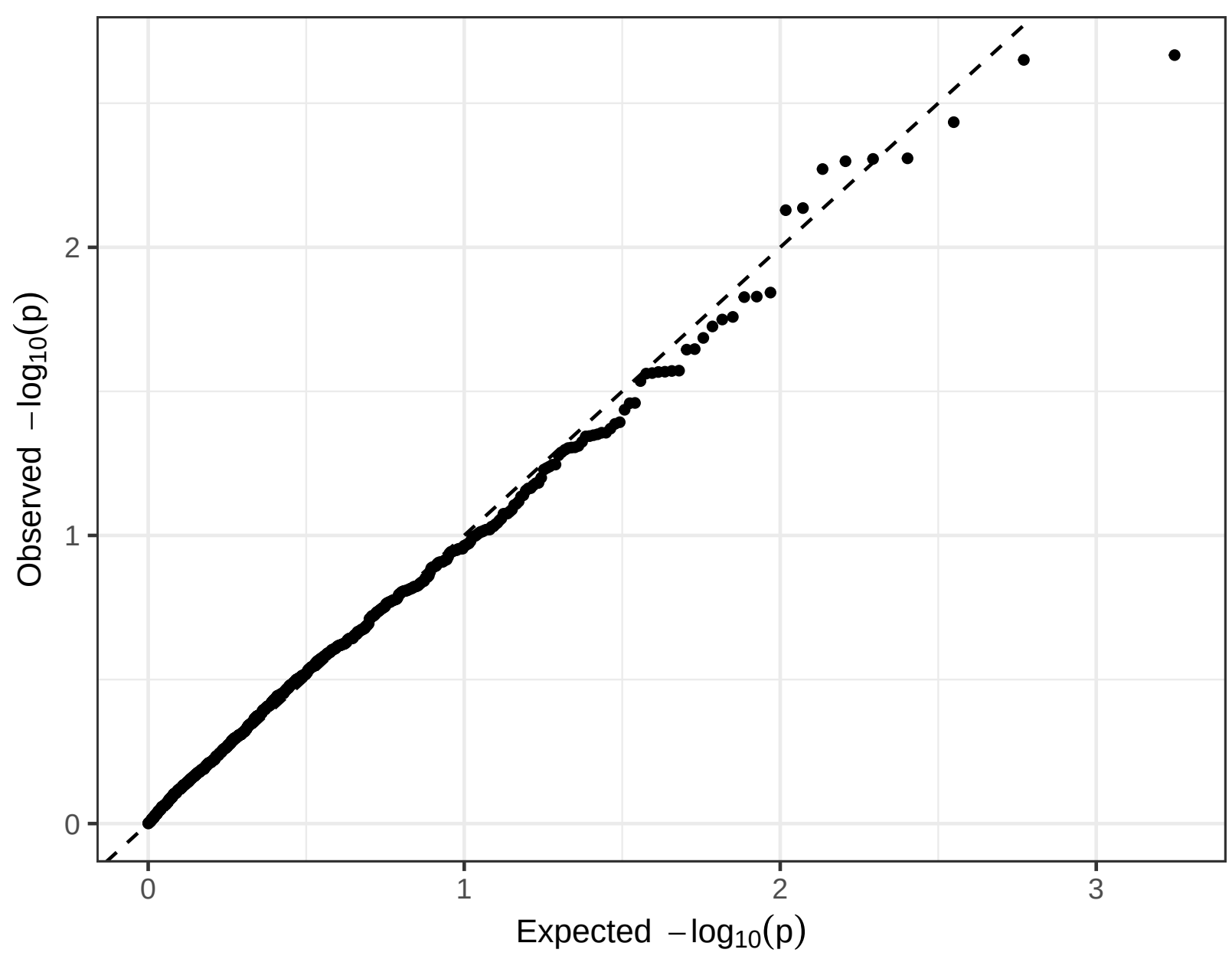

Lachnospiraceae_UCG.003

Cecum | Muscle | . = 0.837

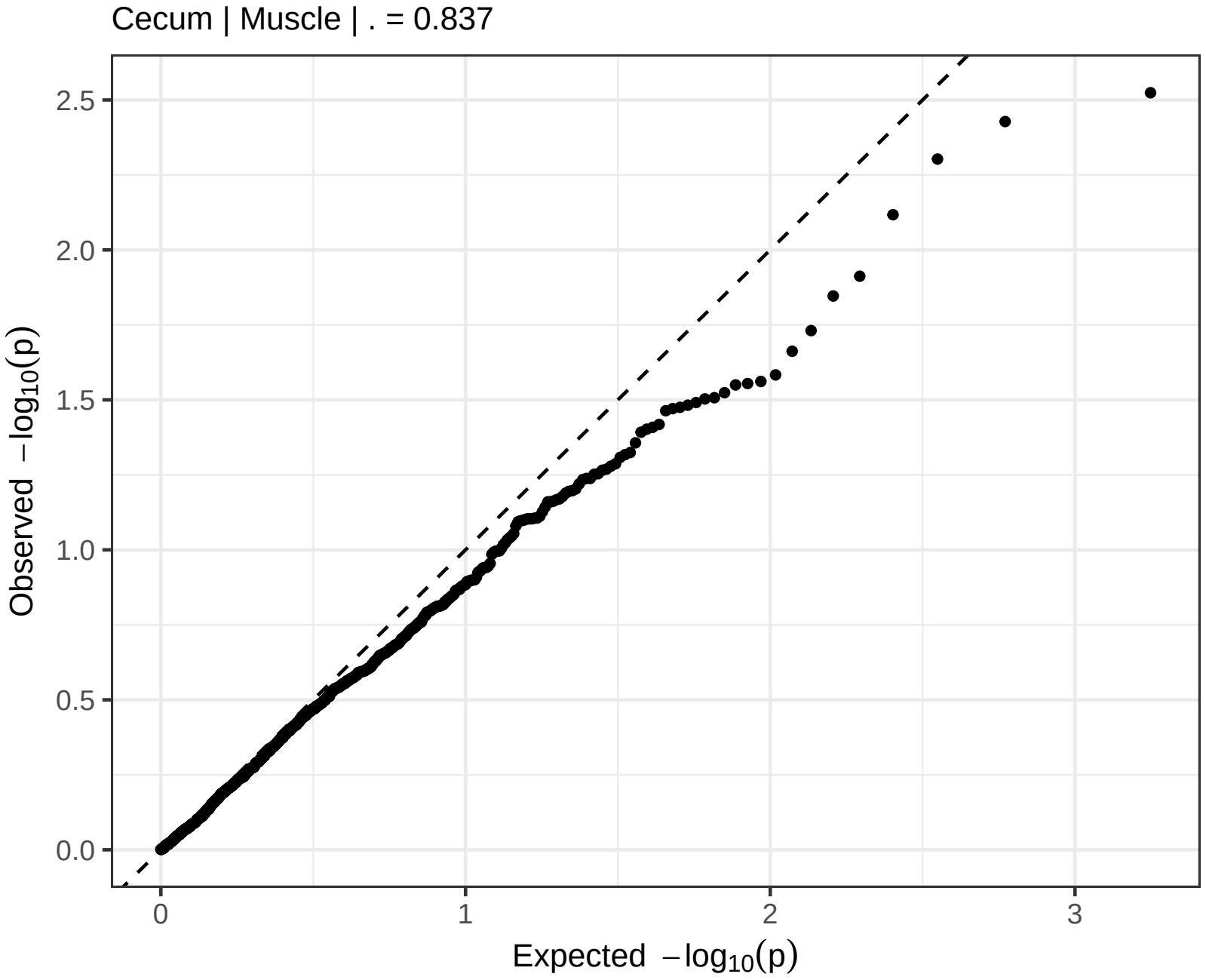

Lachnospiraceae_UCG.004

Cecum | Muscle | . = 0.85

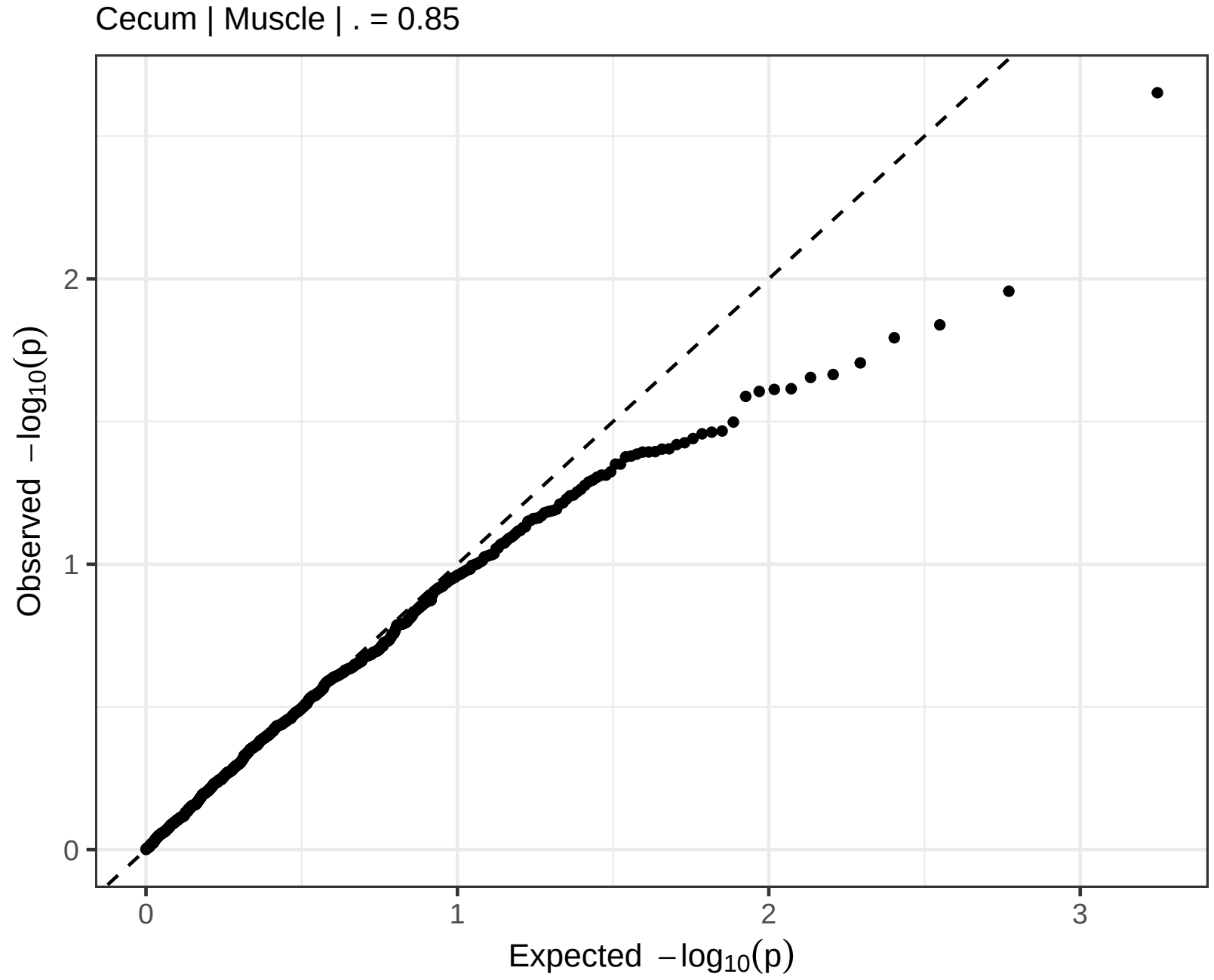

Acutalibacter

Cecum | Muscle | . = 0.82

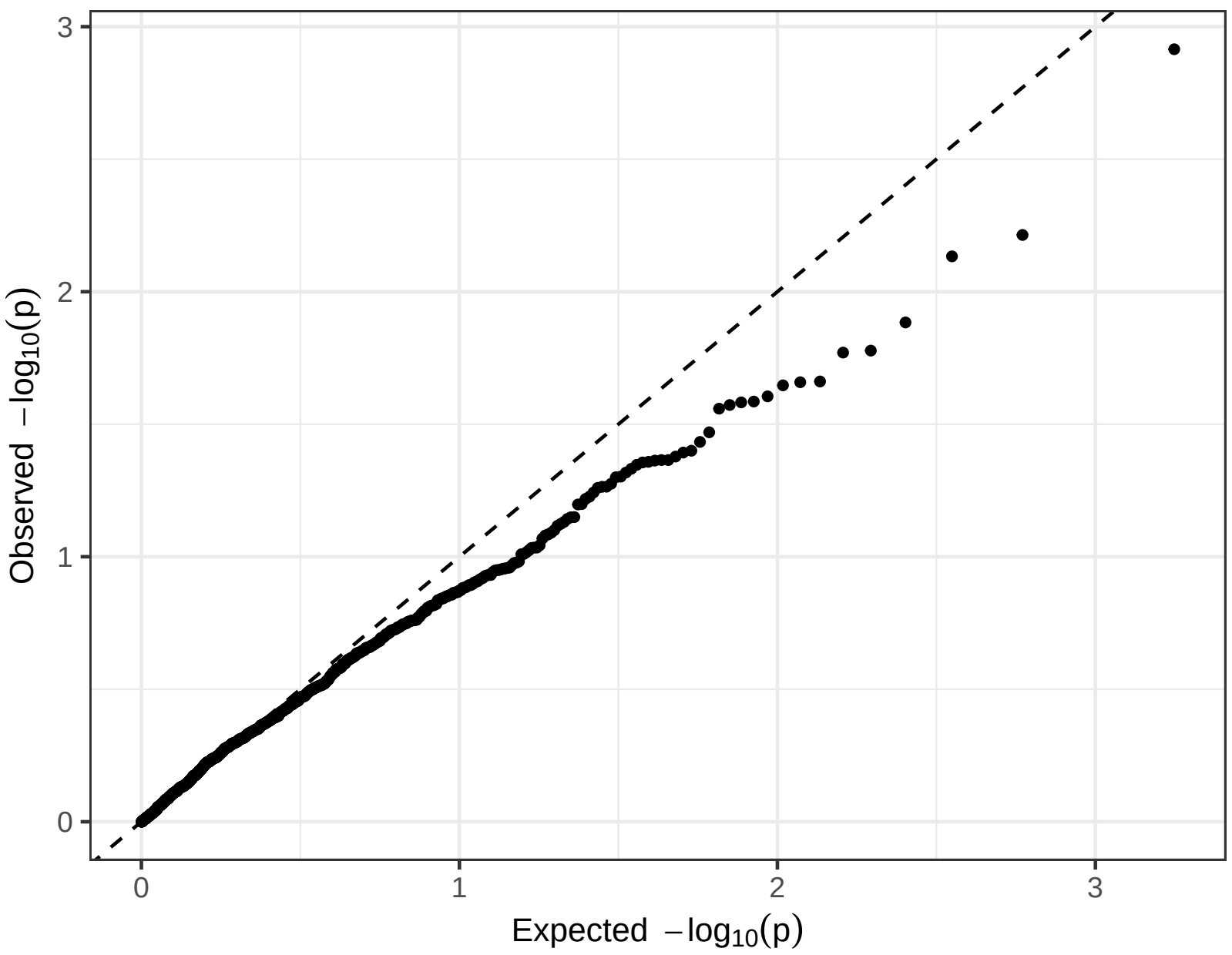

Lachnospiraceae_UCG.007

Cecum | Muscle | . = 0.915

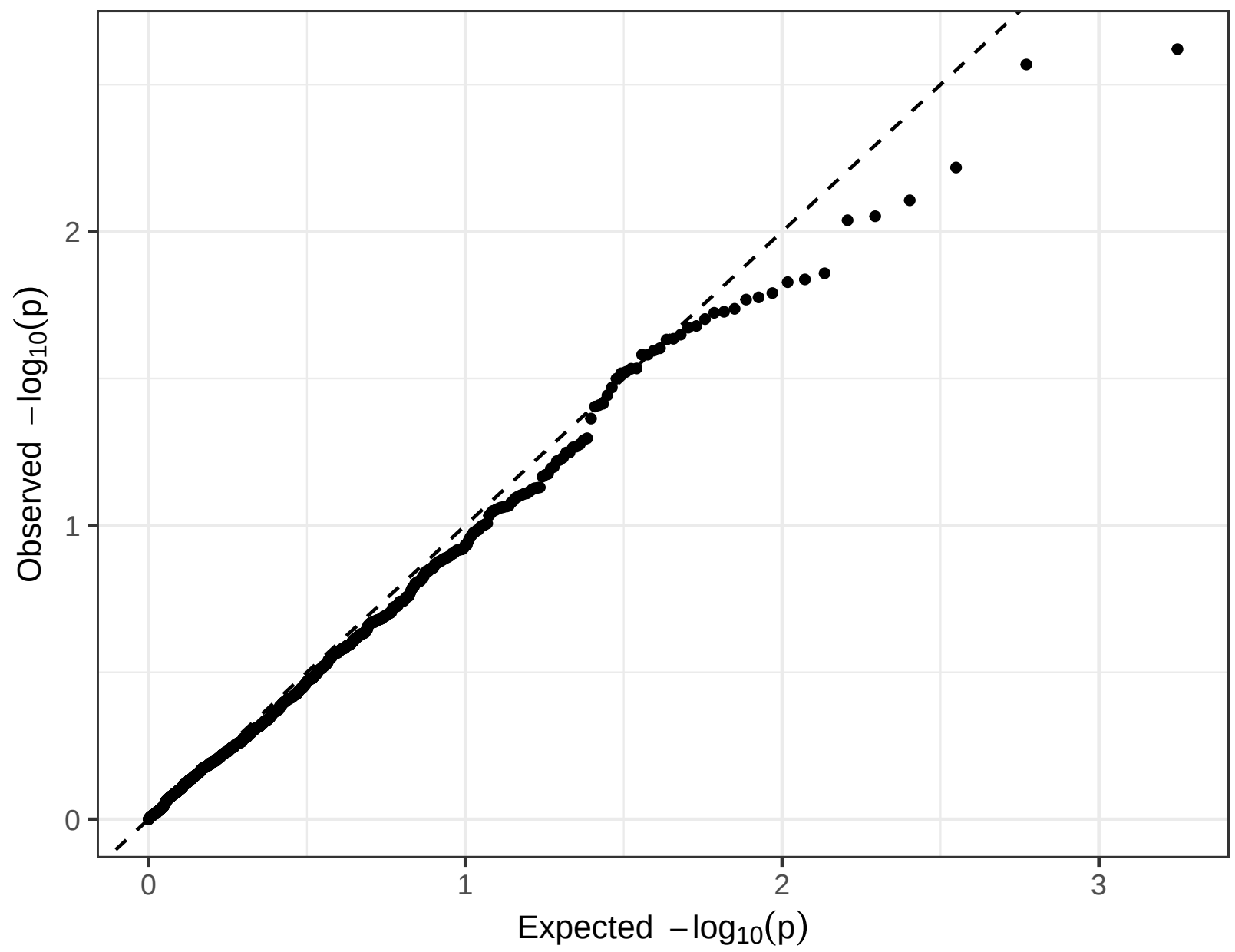

Lachnospiraceae_UCG.008

Cecum | Muscle | . = 0.946

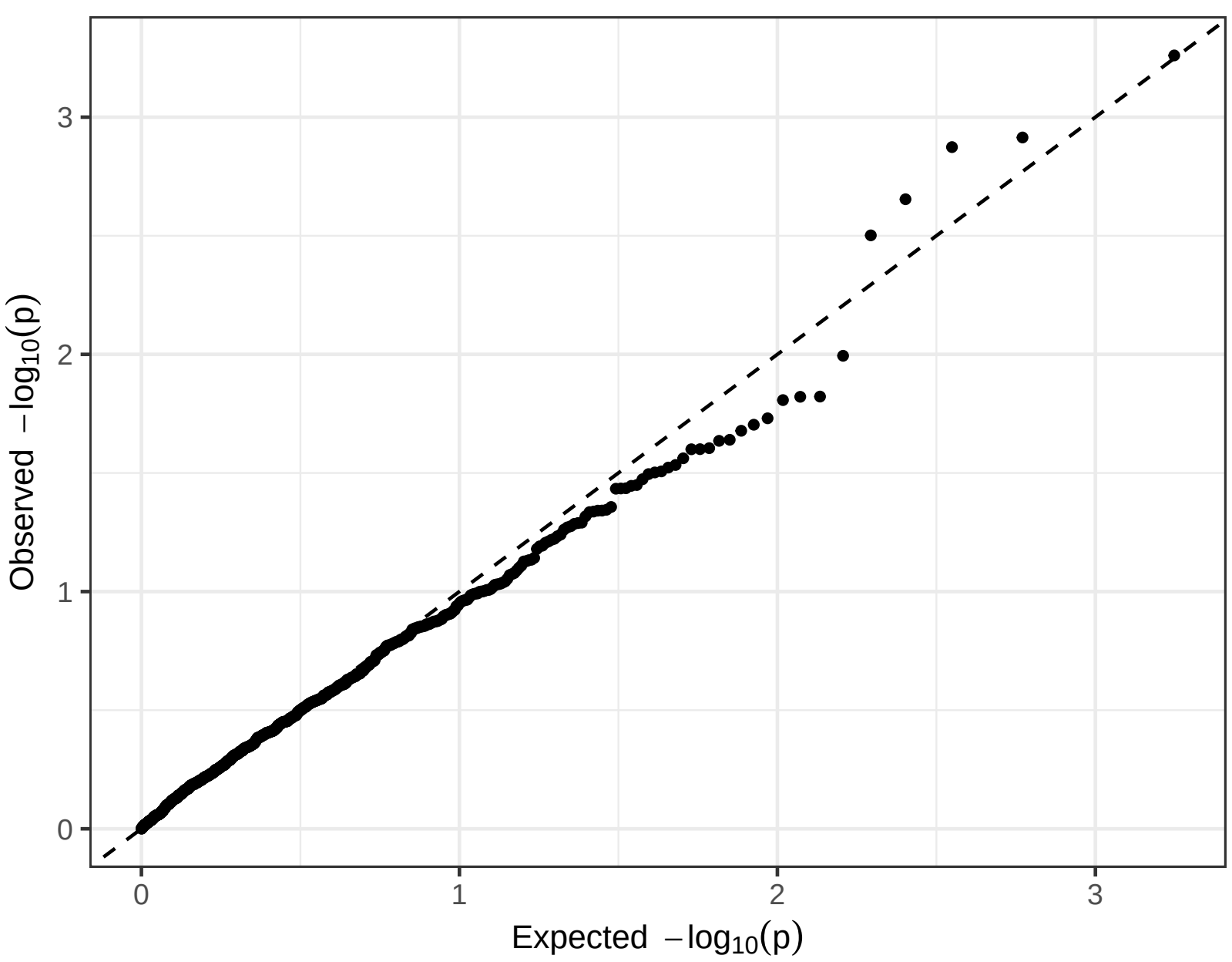

Lachnospiraceae_UCG.009

Cecum | Muscle | . = 0.966

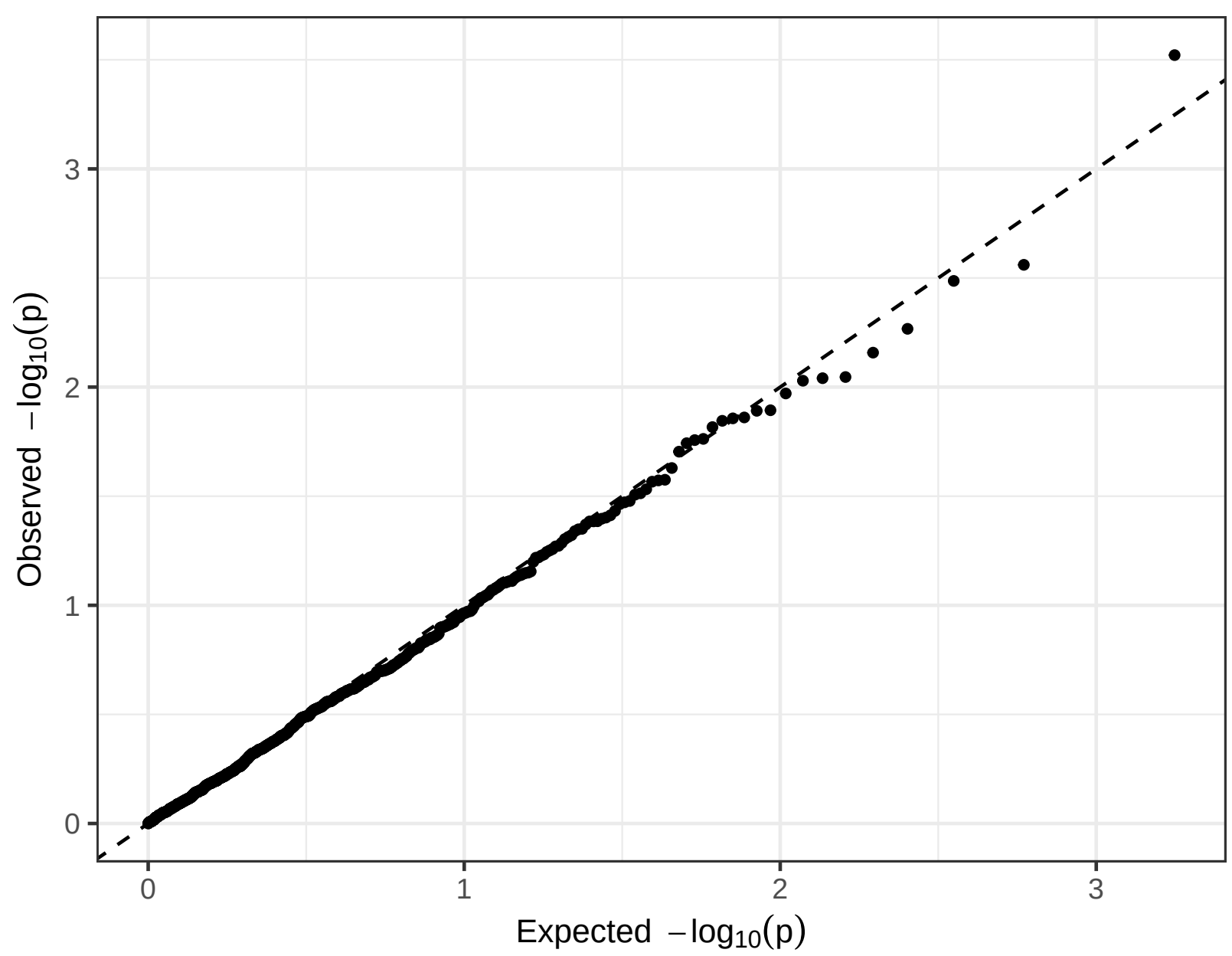

Lachnospiraceae_UCG.010

Cecum | Muscle | . = 0.902

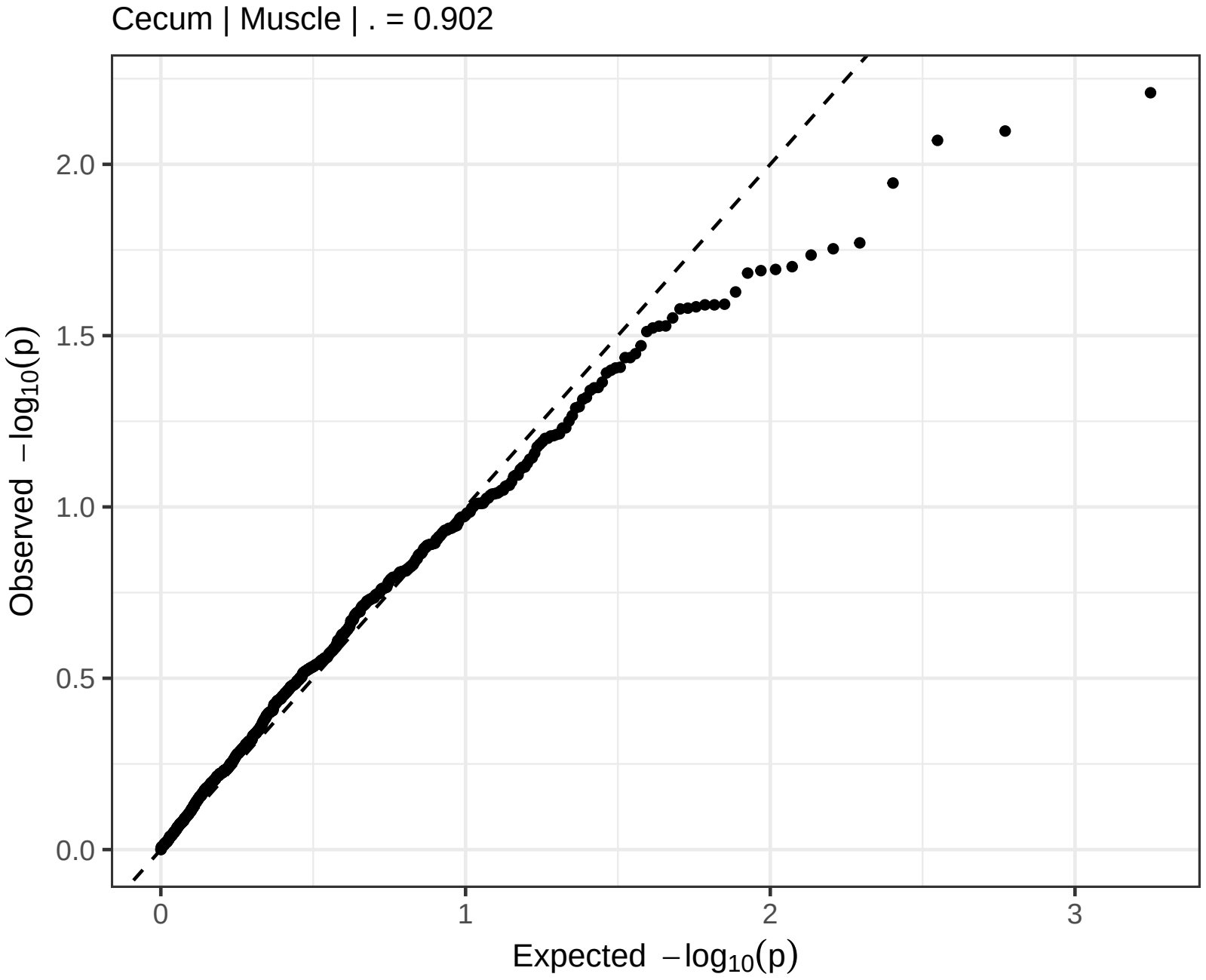

Lachnospiraceae_XPB1014_group

Cecum | Muscle | . = 0.872

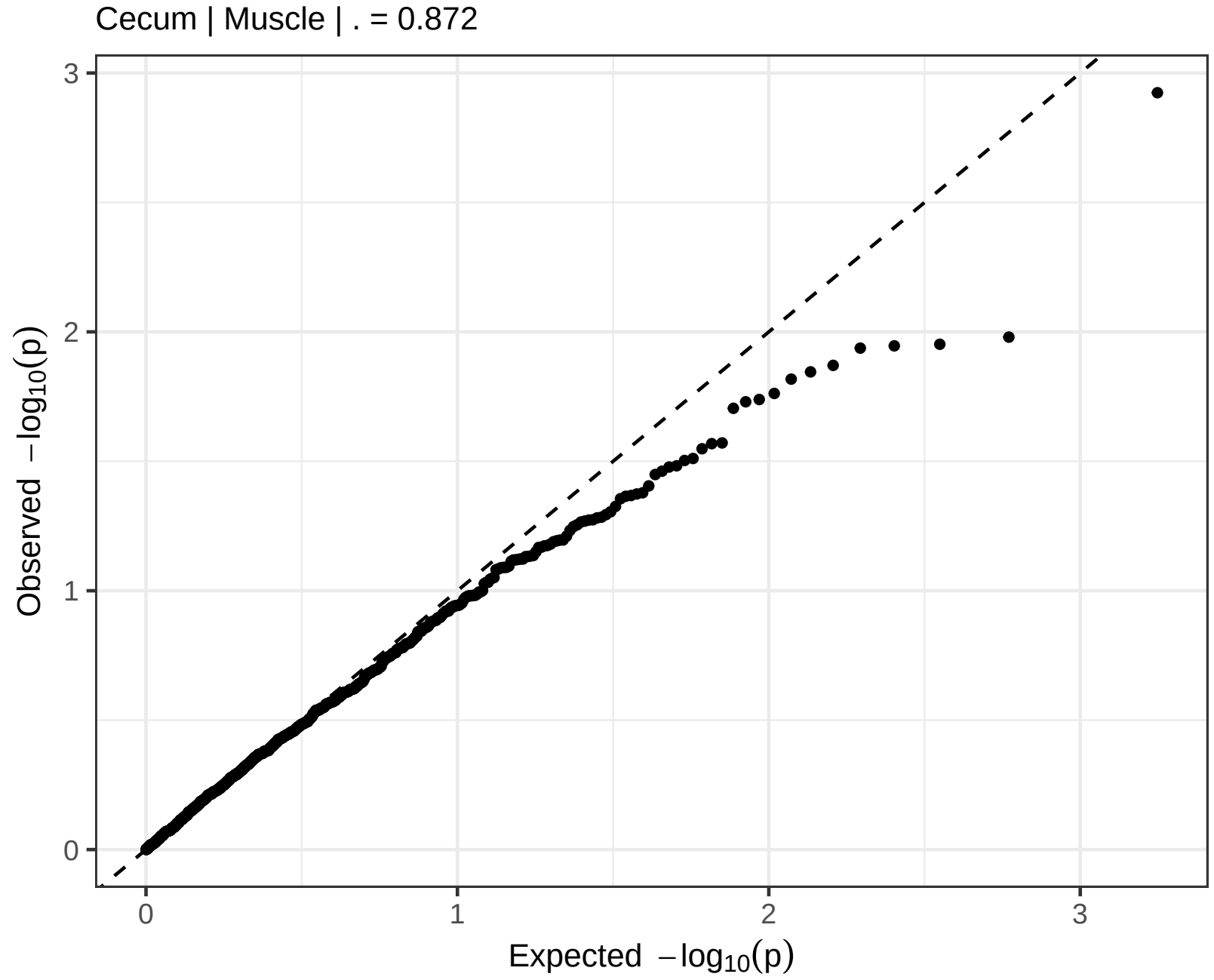

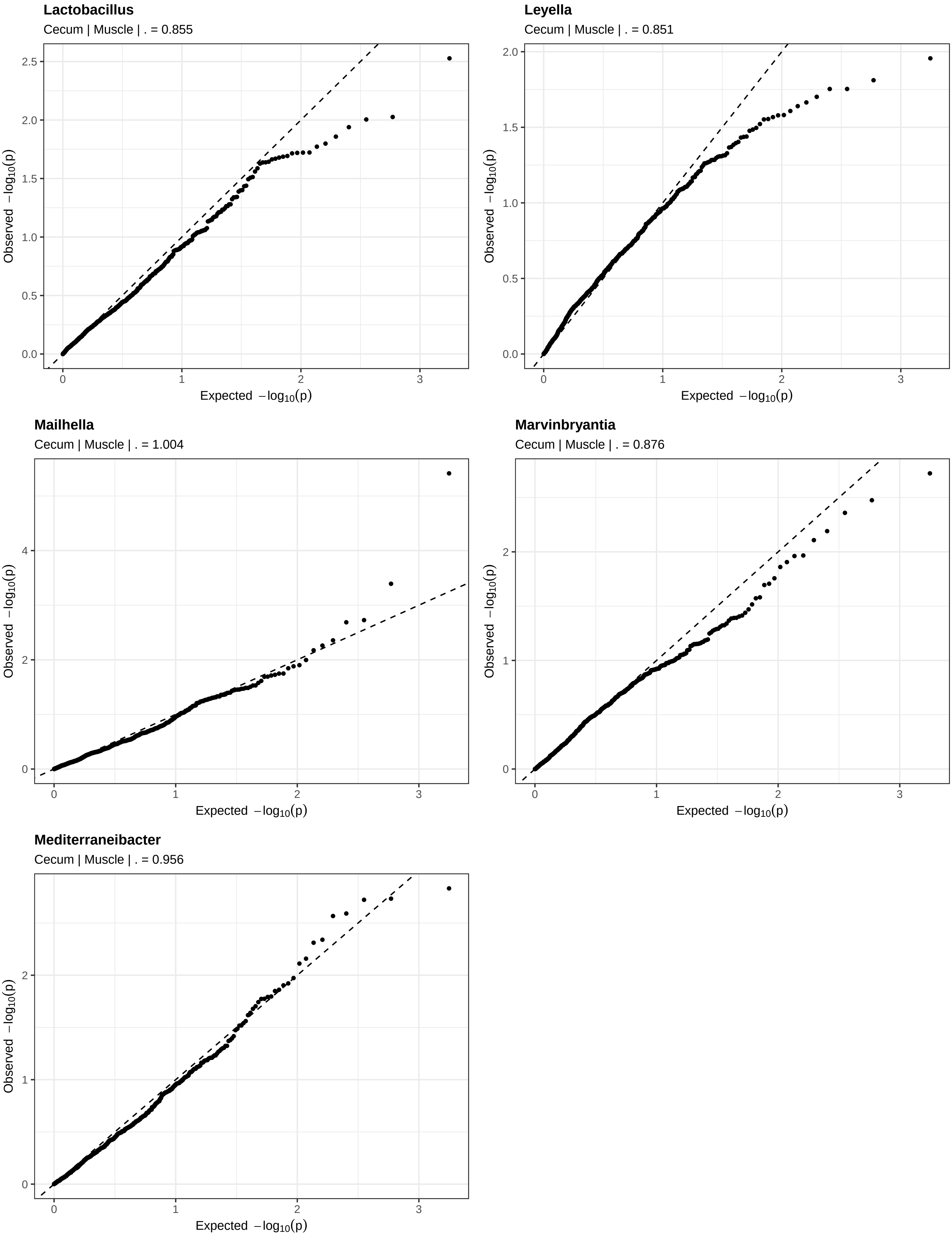

Lactobacillus
Cecum | Muscle | . = 0.855
Observed $-\log_{10}(p)$
Expected $-\log_{10}(p)$
Leyella
Cecum | Muscle | . = 0.851
Observed $-\log_{10}(p)$
Expected $-\log_{10}(p)$
Mailhella
Cecum | Muscle | . = 1.004
Observed $-\log_{10}(p)$
Expected $-\log_{10}(p)$
Marvinbryantia
Cecum | Muscle | . = 0.876
Observed $-\log_{10}(p)$
Expected $-\log_{10}(p)$
Mediterraneibacter
Cecum | Muscle | . = 0.956
Observed $-\log_{10}(p)$
Expected $-\log_{10}(p)$

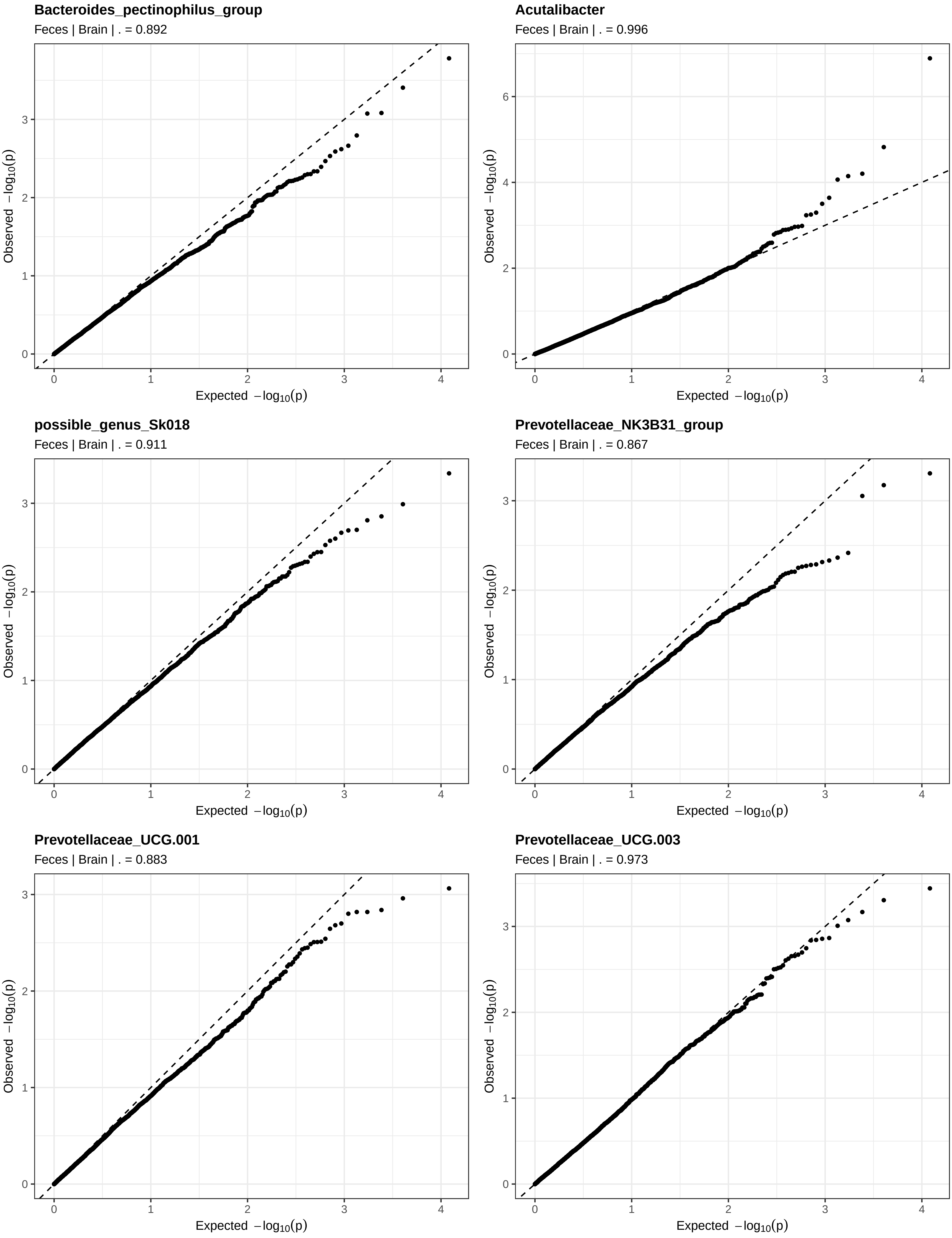
Bacteroides_pectinophilus_group
Feces | Brain | . = 0.892
Acutalibacter
Feces | Brain | . = 0.996
possible_genus_Sk018
Feces | Brain | . = 0.911
Prevotellaceae_NK3B31_group
Feces | Brain | . = 0.867
Prevotellaceae_UCG.001
Feces | Brain | . = 0.883
Prevotellaceae_UCG.003
Feces | Brain | . = 0.973
Observed $-\log_{10}(p)$
Expected $-\log_{10}(p)$

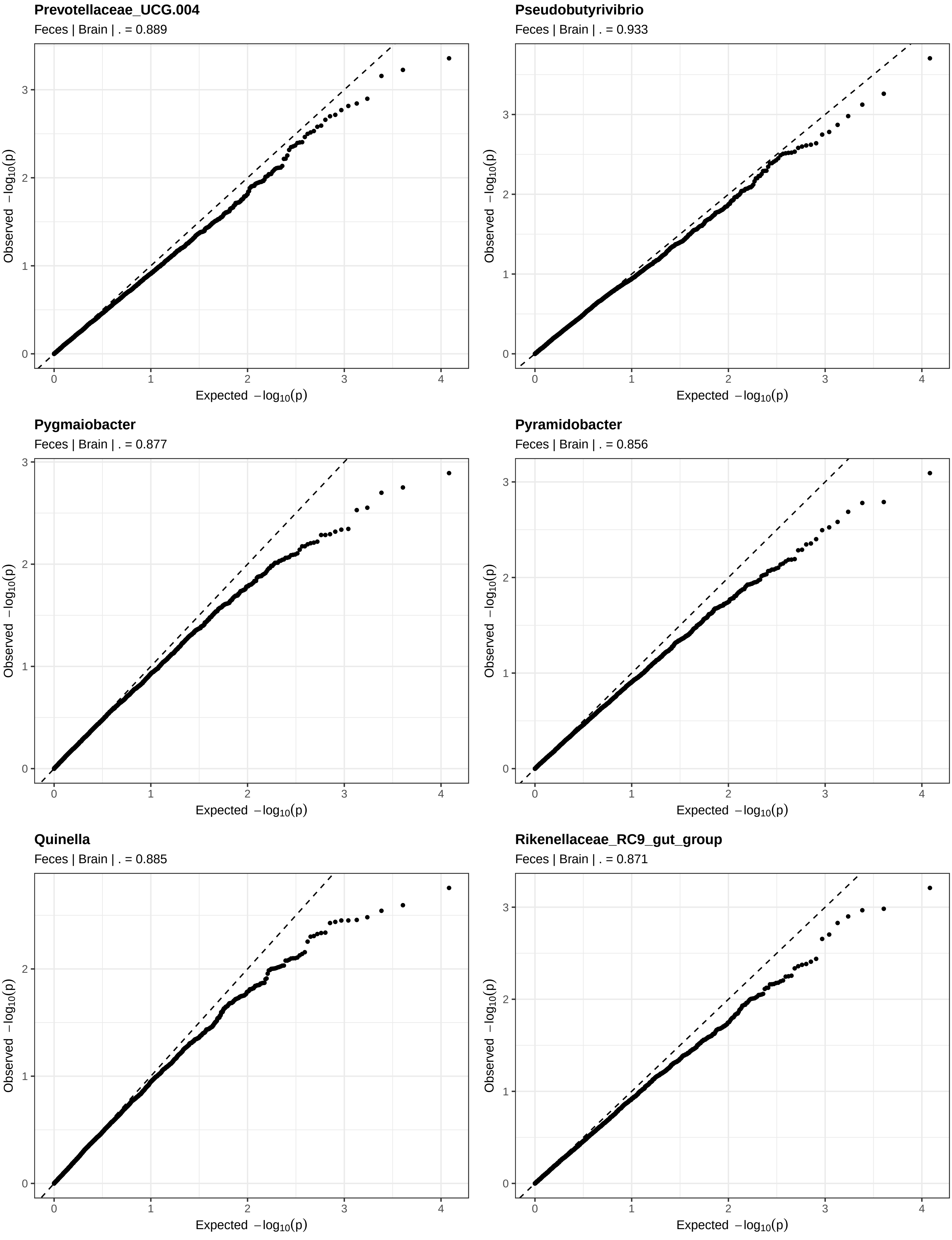

Prevotellaceae_UCG.004
Feces | Brain | . = 0.889
Pseudobutyrivibrio
Feces | Brain | . = 0.933
Pygmaiobacter
Feces | Brain | . = 0.877
Pyramidobacter
Feces | Brain | . = 0.856
Quinella
Feces | Brain | . = 0.885
Rikenellaceae_RC9_gut_group
Feces | Brain | . = 0.871
Observed $-\log_{10}(p)$
Expected $-\log_{10}(p)$
0
1
2
3
4

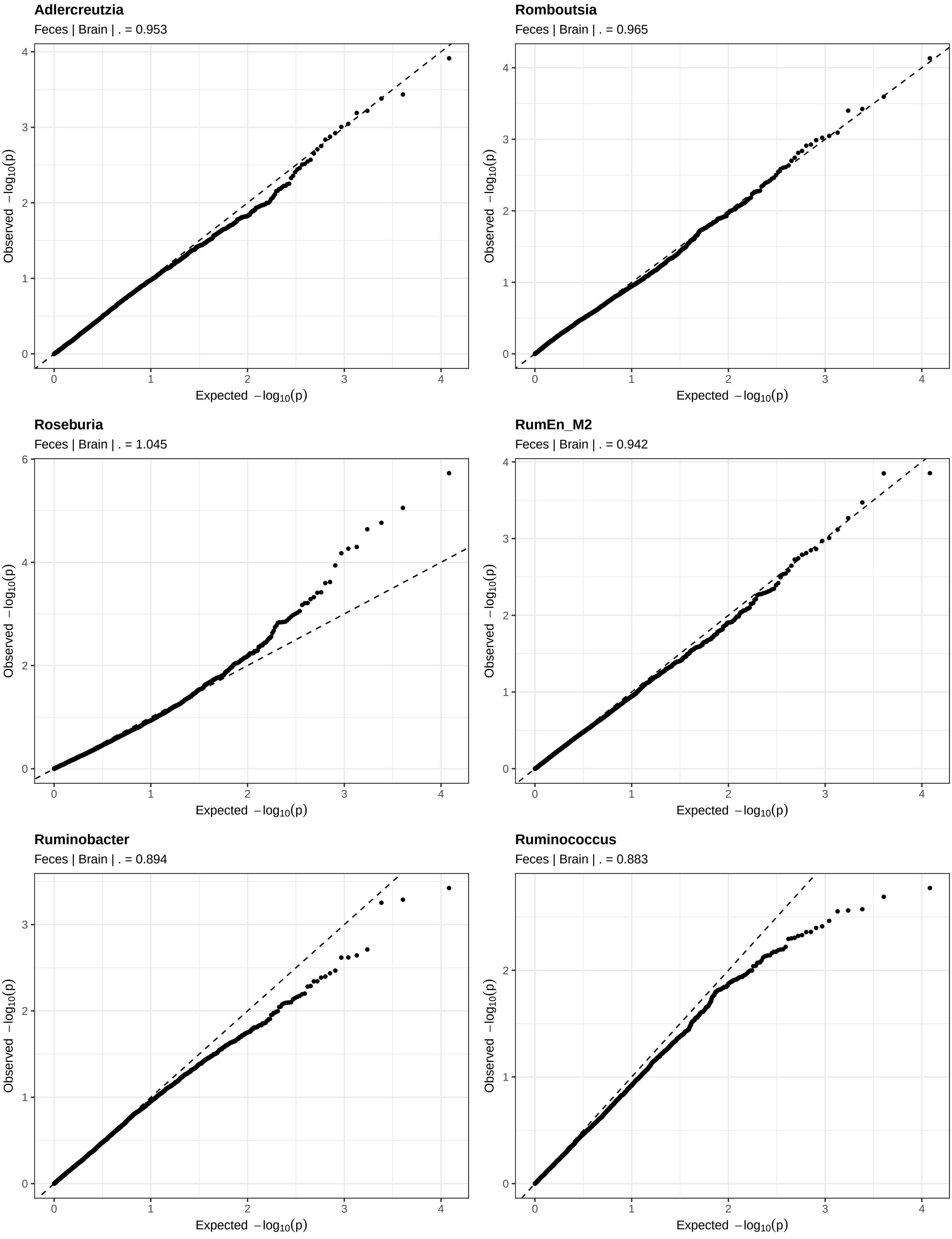
Adlercreutzia
Feces | Brain | . = 0.953
Romboutsia
Feces | Brain | . = 0.965
Roseburia
Feces | Brain | . = 1.045
RumEn_M2
Feces | Brain | . = 0.942
Ruminobacter
Feces | Brain | . = 0.894
Ruminococcus
Feces | Brain | . = 0.883
Observed $-\log_{10}(p)$
Expected $-\log_{10}(p)$

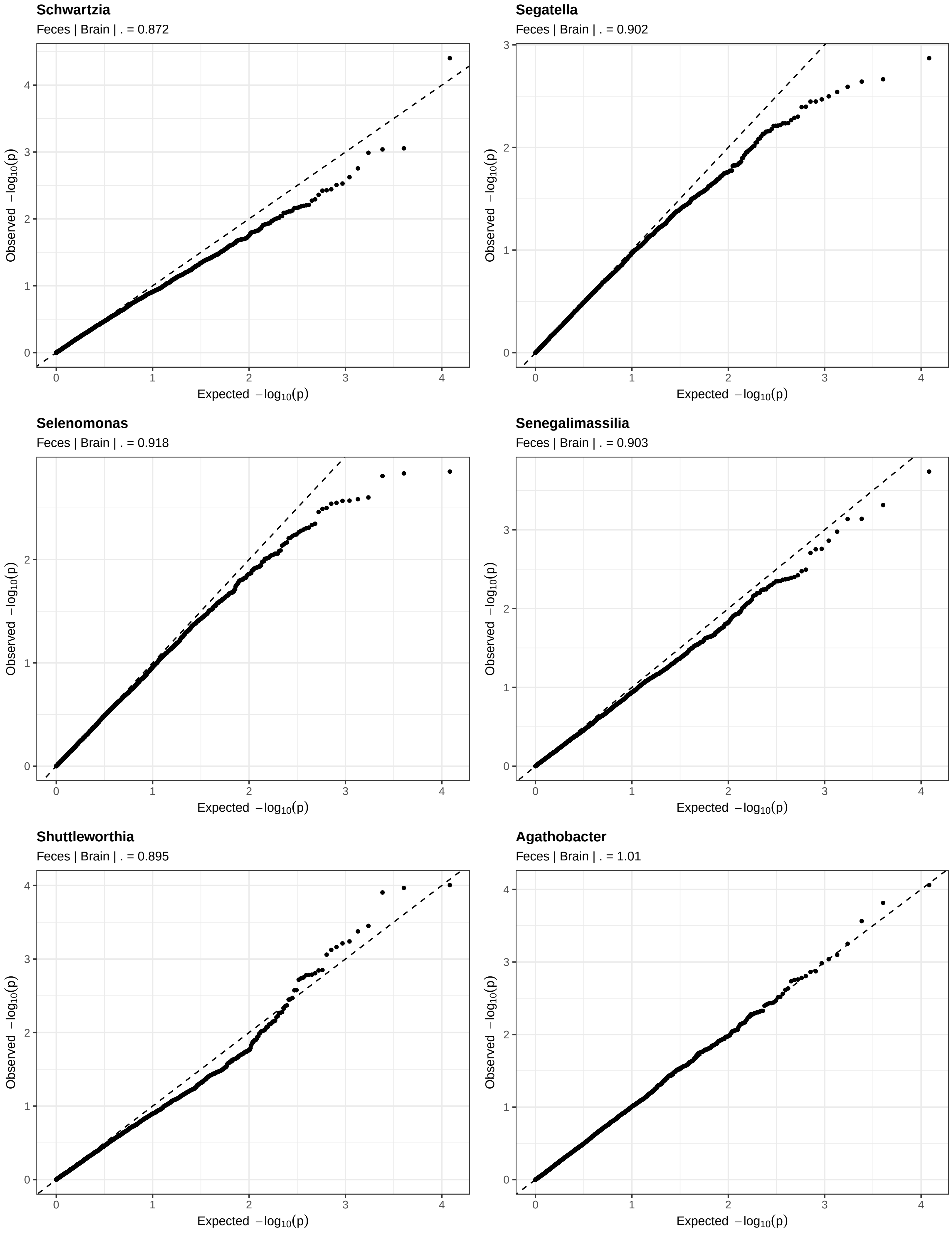
Schwartzia
Feces | Brain | . = 0.872
Segatella
Feces | Brain | . = 0.902
Selenomonas
Feces | Brain | . = 0.918
Senegalimassilia
Feces | Brain | . = 0.903
Shuttleworthia
Feces | Brain | . = 0.895
Agathobacter
Feces | Brain | . = 1.01
Observed −log10(p)
Expected −log10(p)

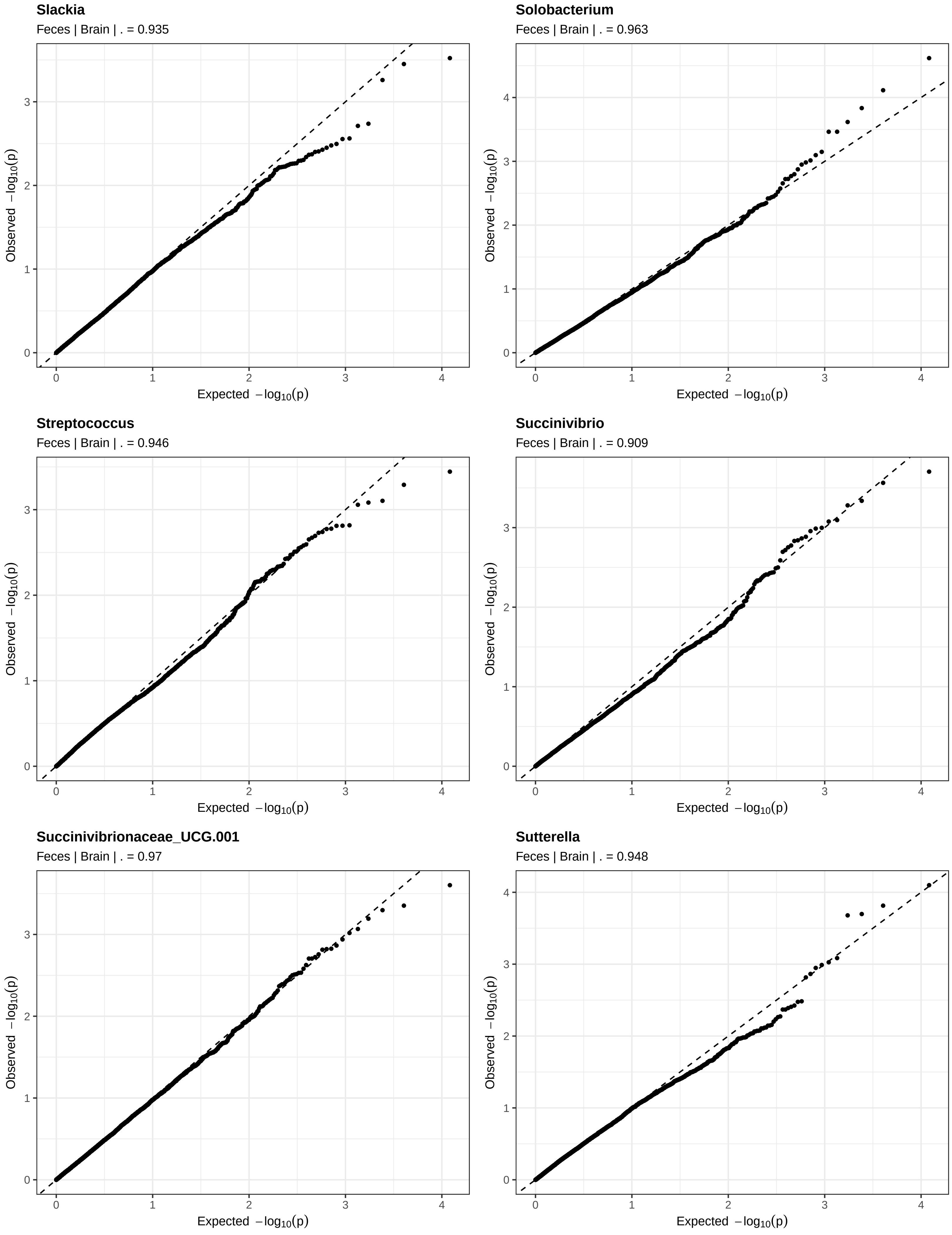
Slackia
Feces | Brain | . = 0.935
Observed $-\log_{10}(p)$
Expected $-\log_{10}(p)$
Solobacterium
Feces | Brain | . = 0.963
Observed $-\log_{10}(p)$
Expected $-\log_{10}(p)$
Streptococcus
Feces | Brain | . = 0.946
Observed $-\log_{10}(p)$
Expected $-\log_{10}(p)$
Succinivibrio
Feces | Brain | . = 0.909
Observed $-\log_{10}(p)$
Expected $-\log_{10}(p)$
Succinivibrionaceae_UCG.001
Feces | Brain | . = 0.97
Observed $-\log_{10}(p)$
Expected $-\log_{10}(p)$
Sutterella
Feces | Brain | . = 0.948
Observed $-\log_{10}(p)$
Expected $-\log_{10}(p)$

T2WK15B57

Feces | Brain | . = 1.054

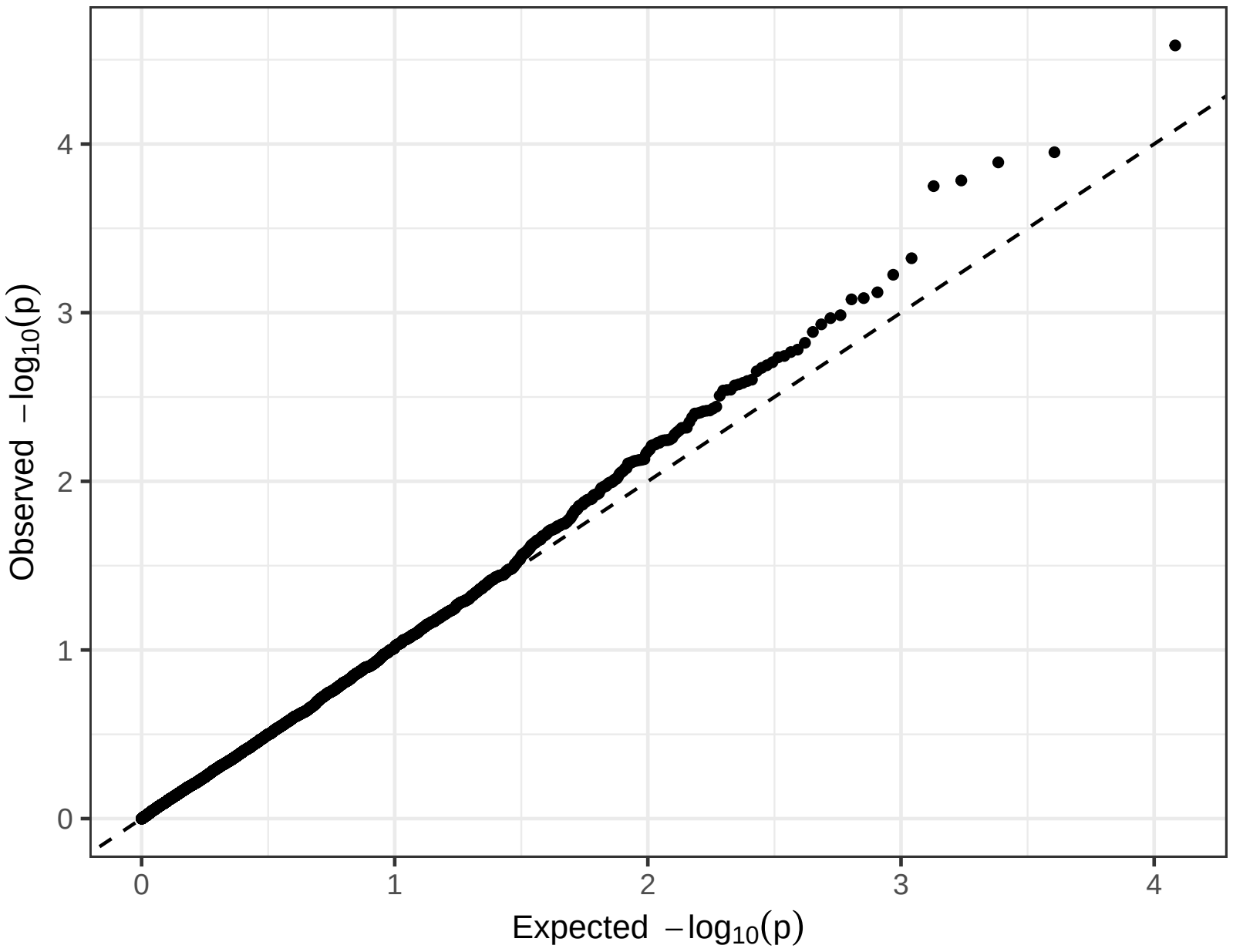

Terrisporobacter

Feces | Brain | . = 0.965

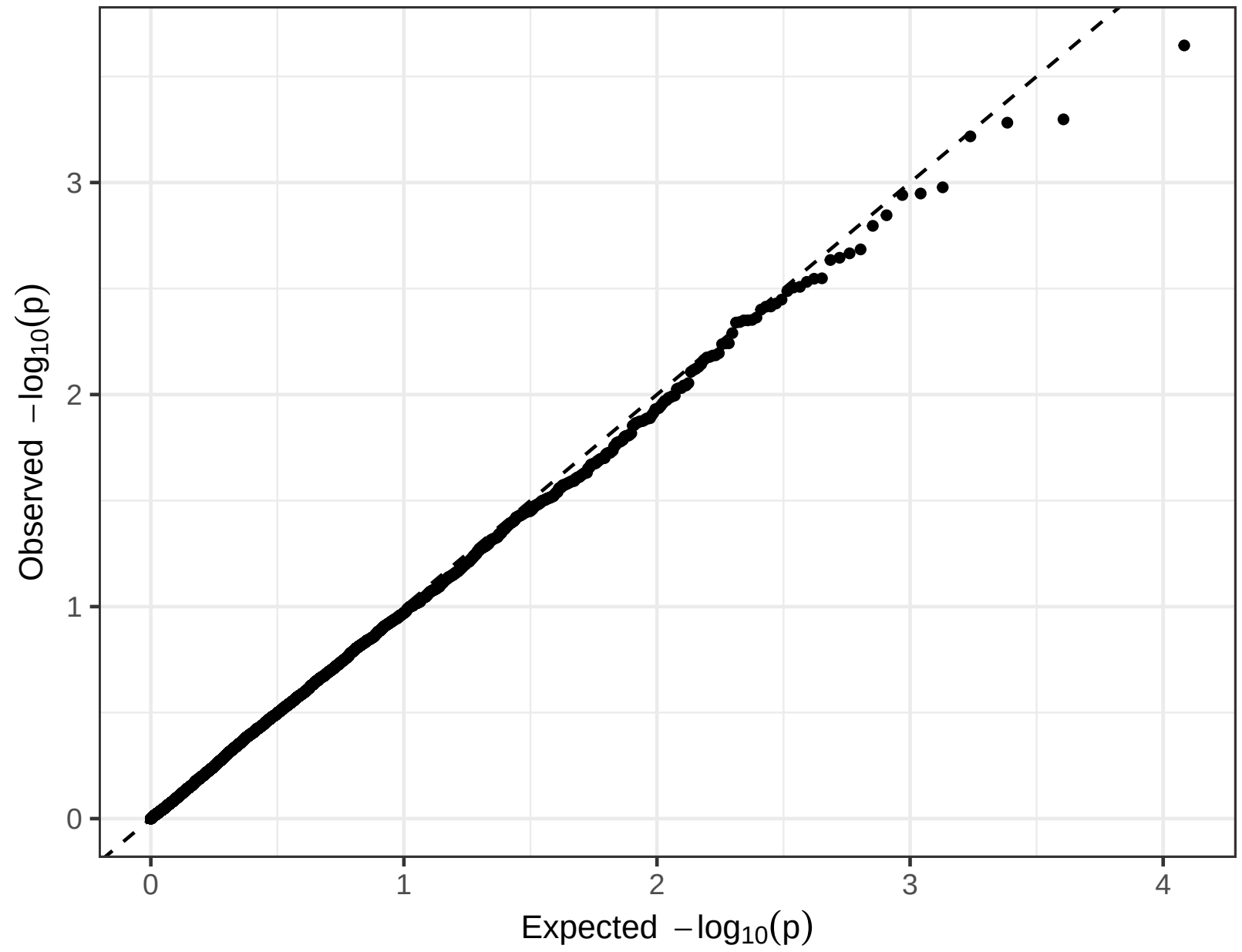

Tractidigestivibacter

Feces | Brain | . = 0.87

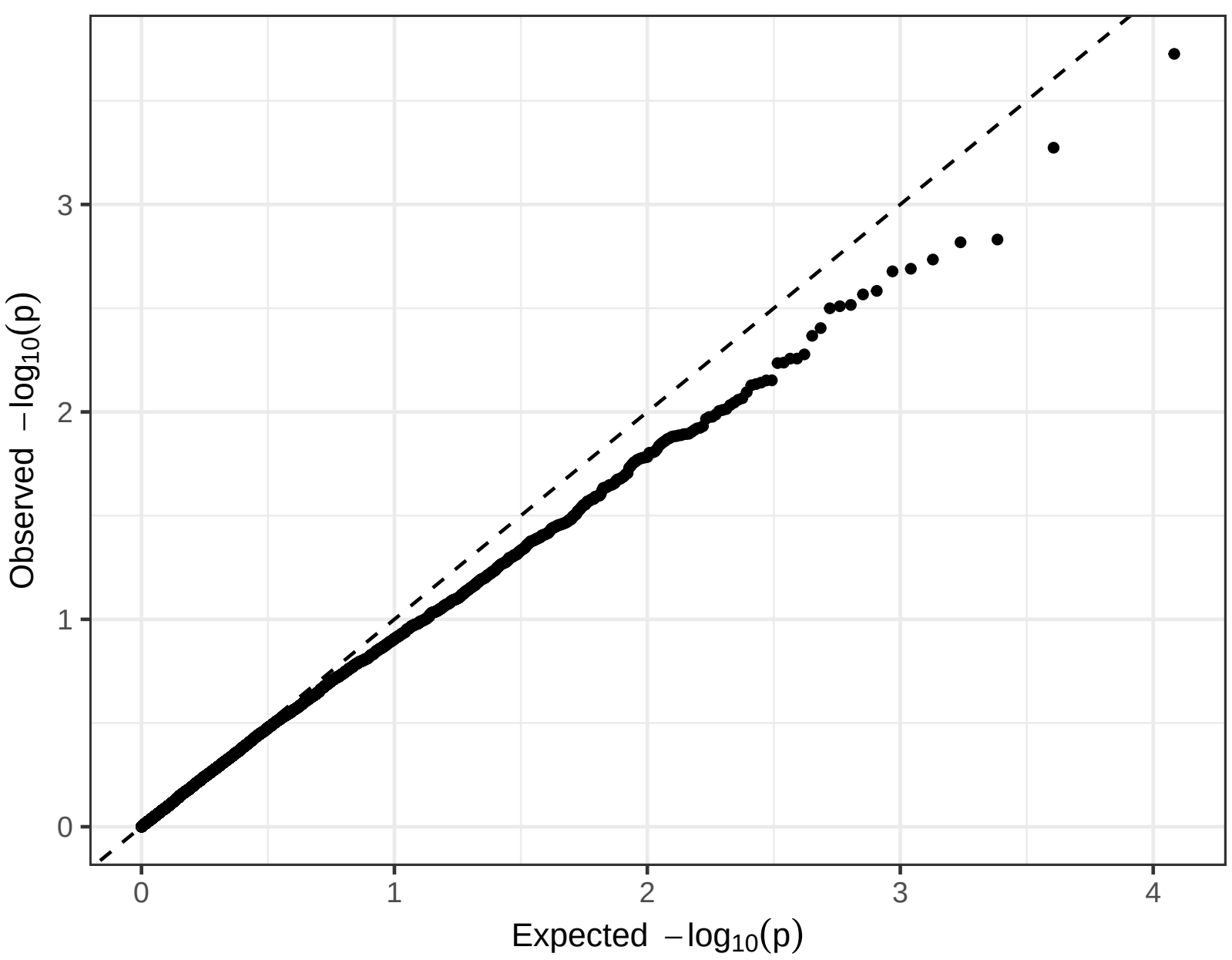

Treponema

Feces | Brain | . = 0.882

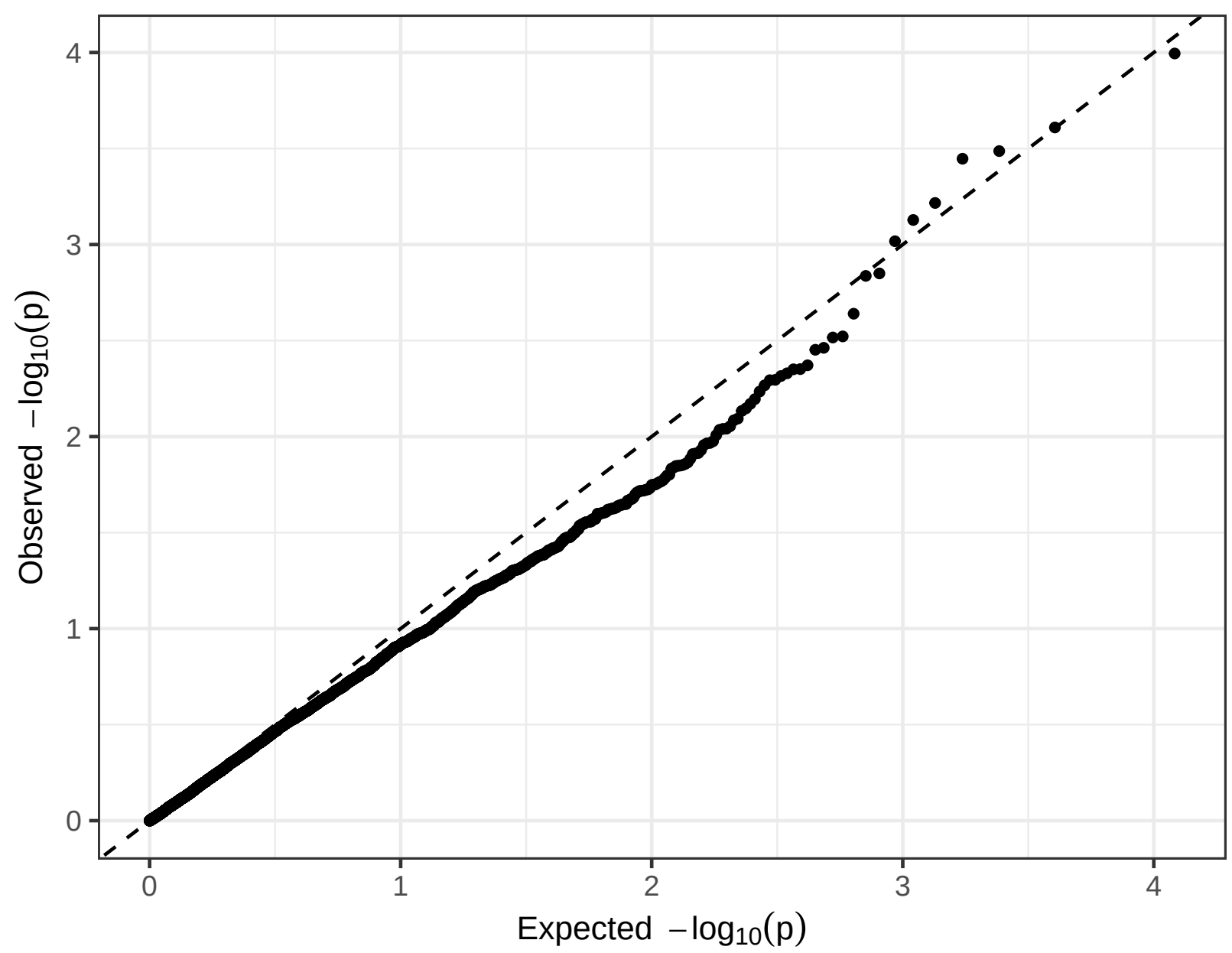

Agathobaculum

Feces | Brain | . = 0.87

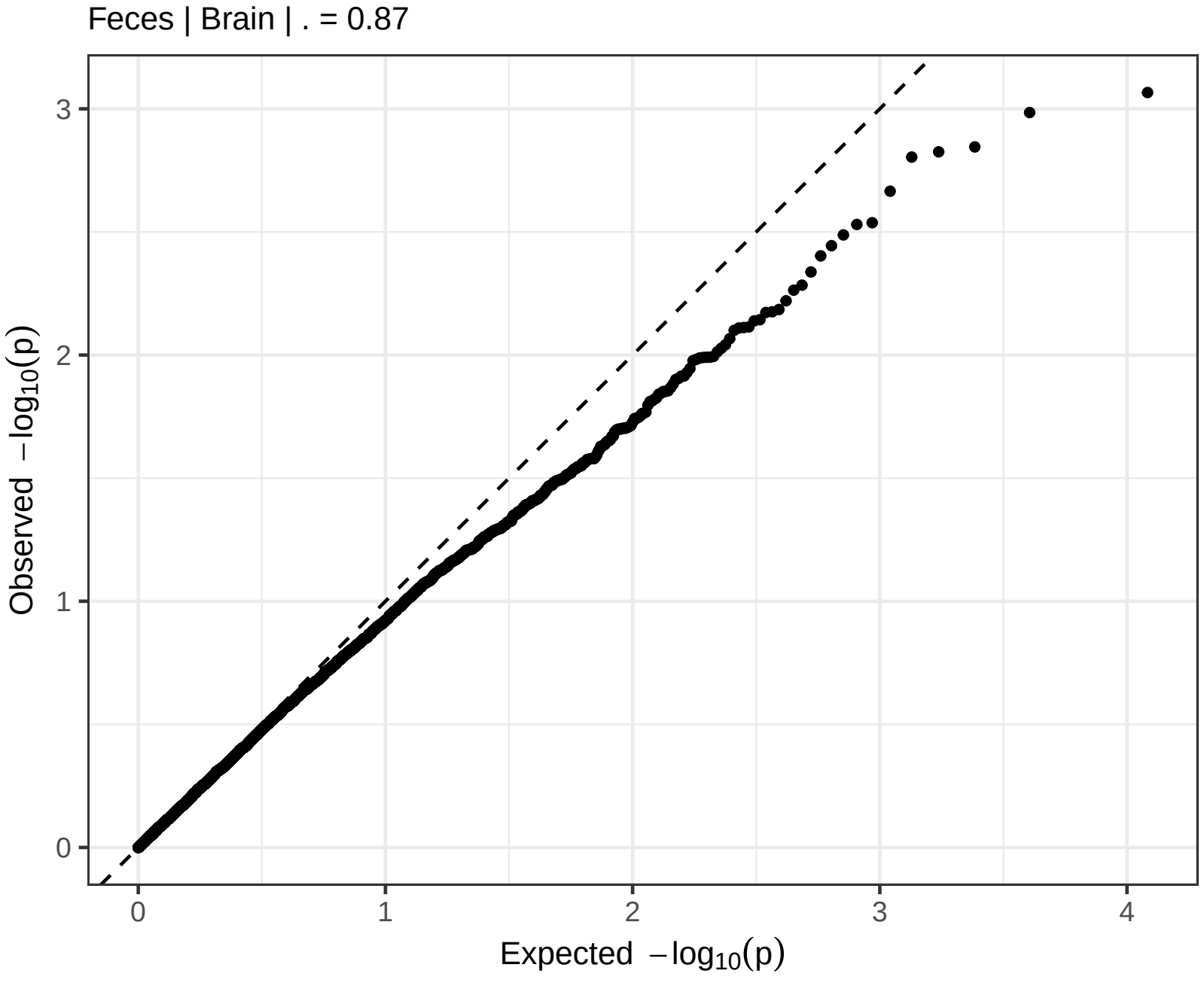

Turicibacter

Feces | Brain | . = 1.03

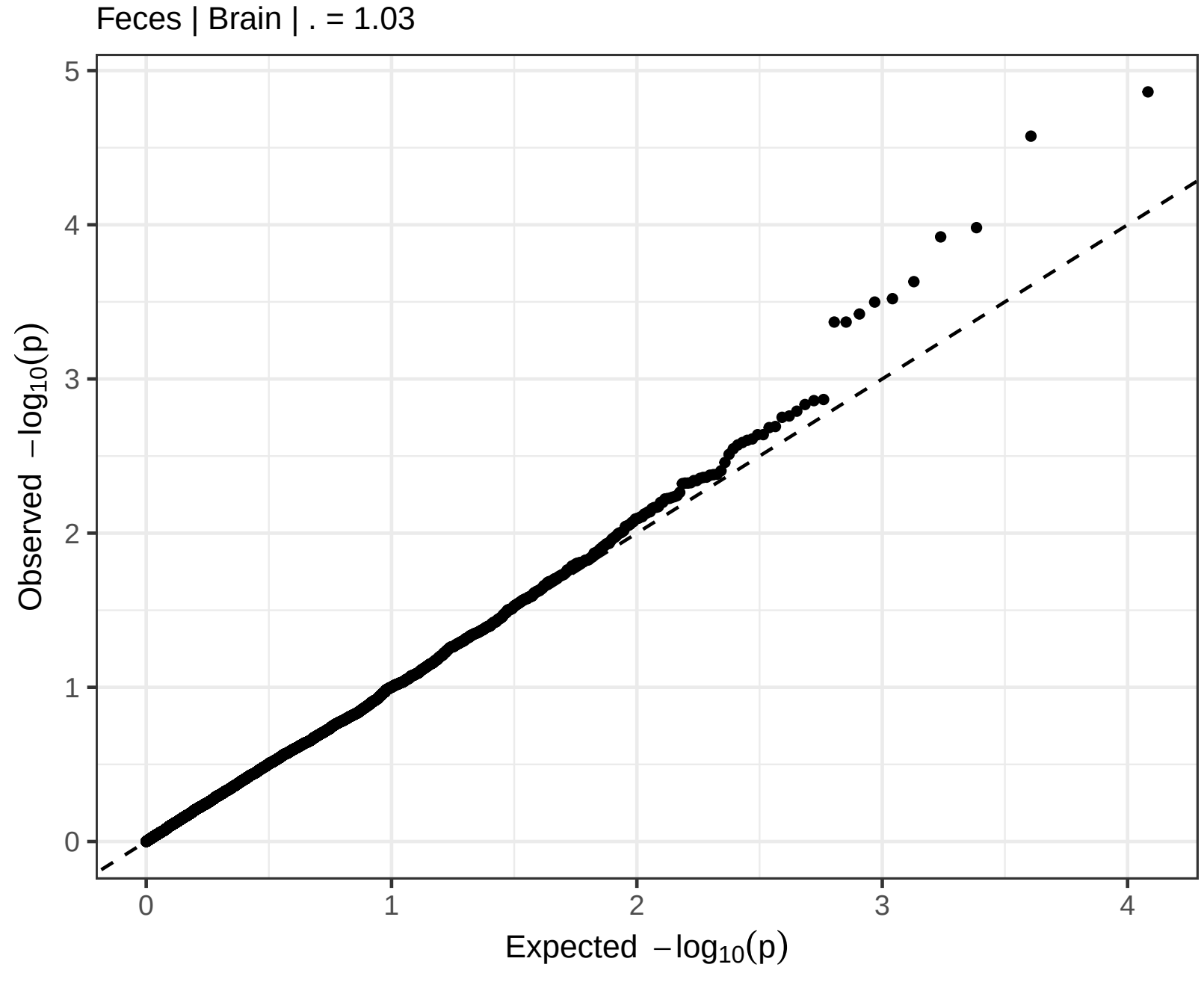

UCG.001

Feces | Brain | . = 0.862

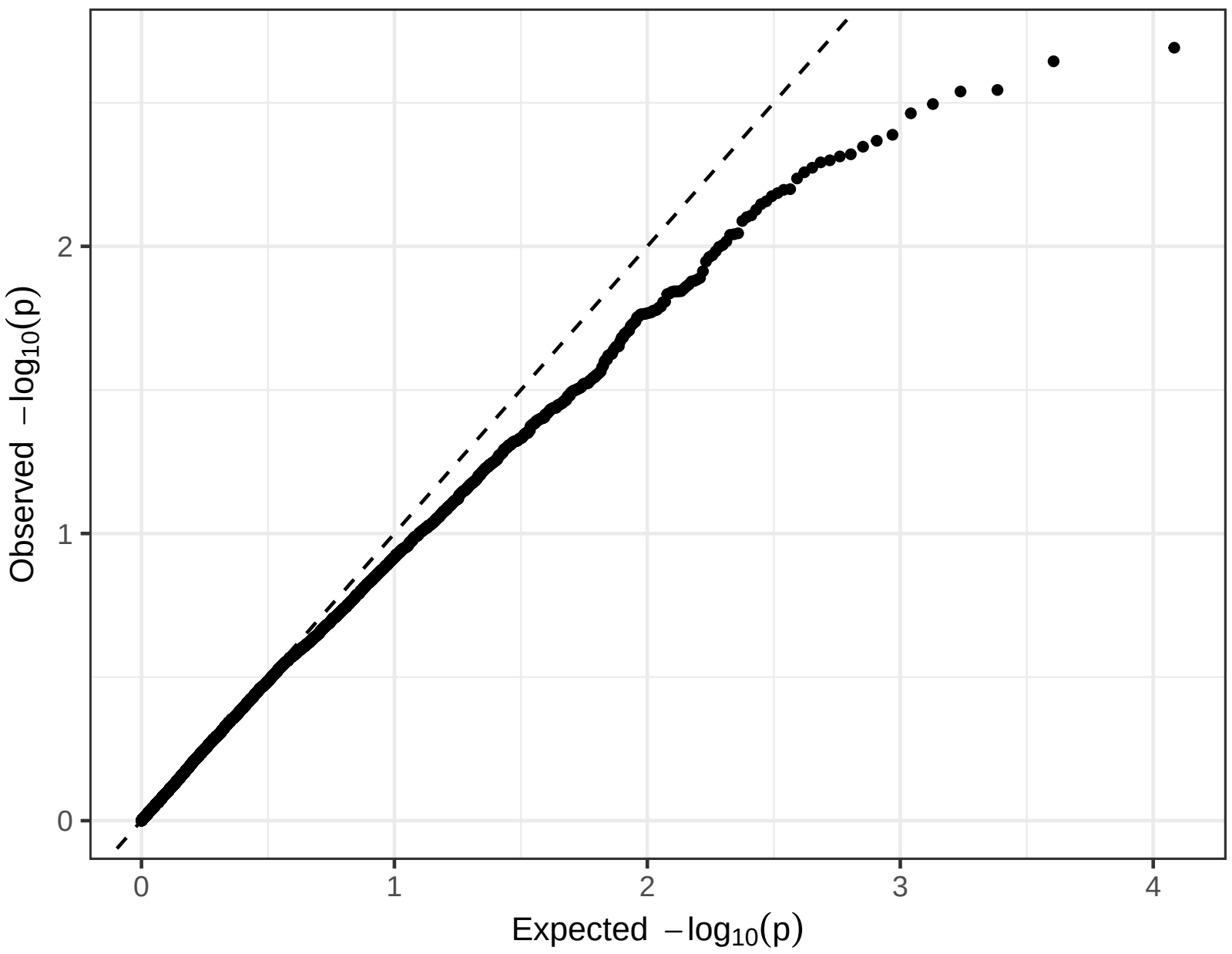

UCG.002

Feces | Brain | . = 0.919

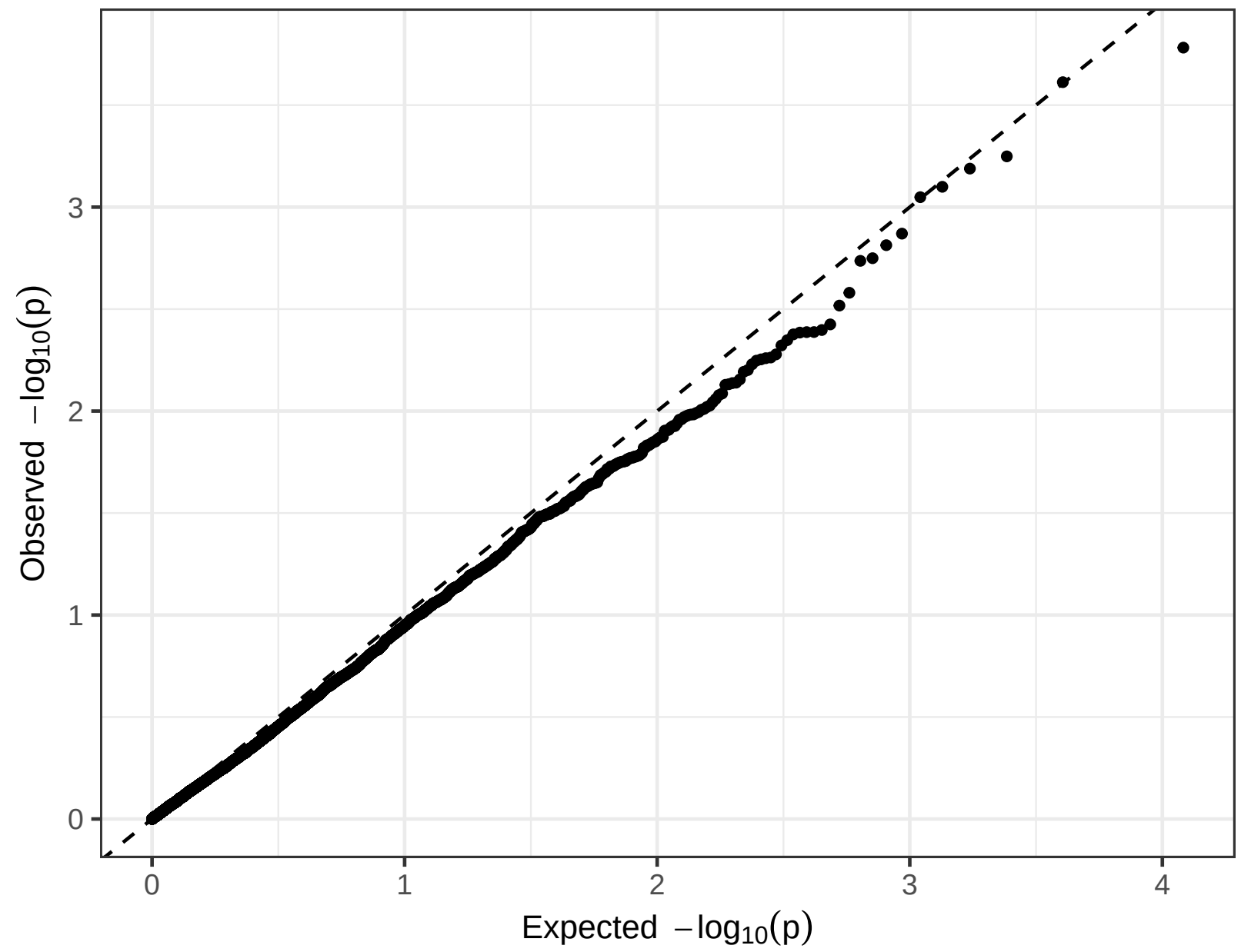

UCG.004

Feces | Brain | . = 0.994

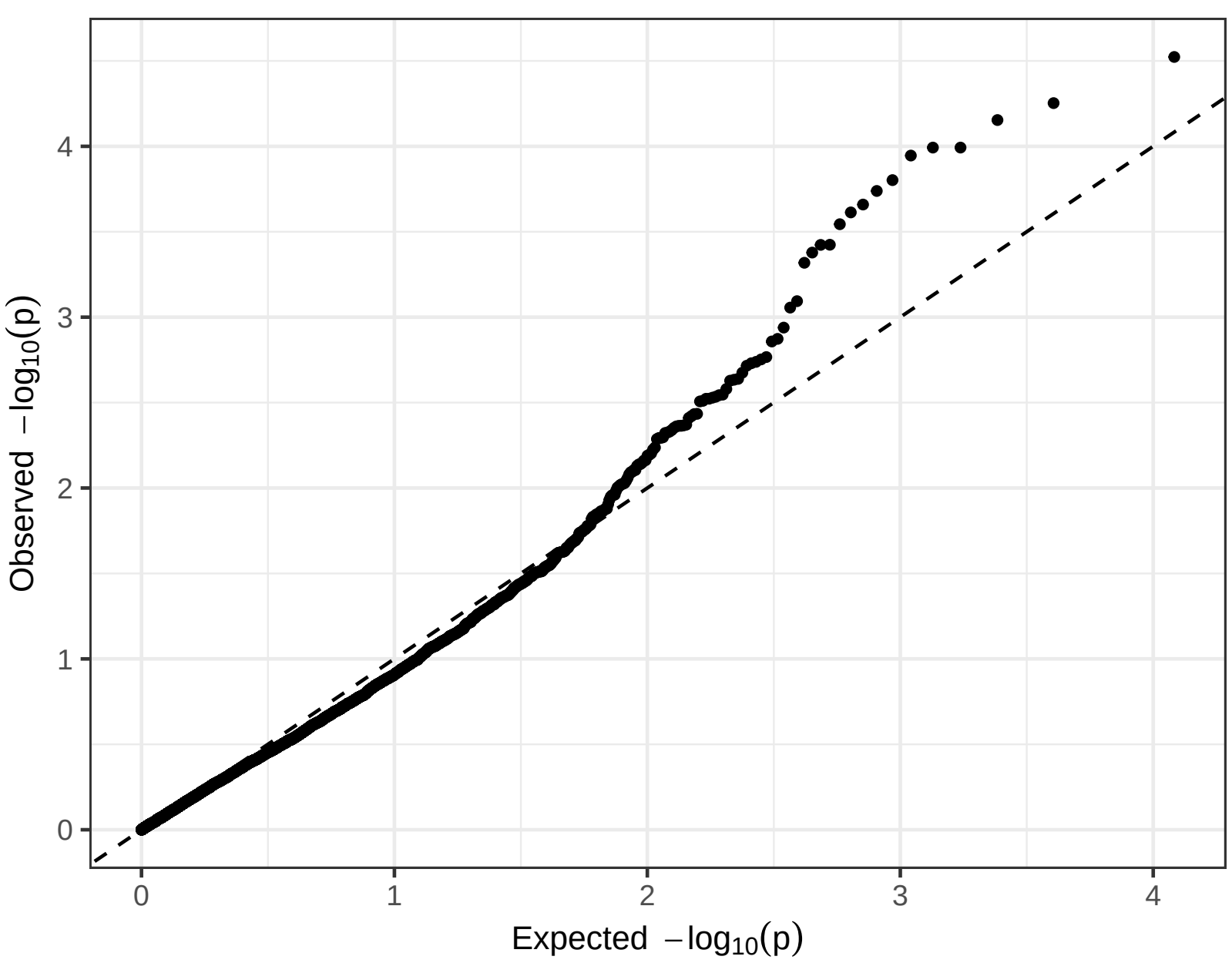

UCG.005

Feces | Brain | . = 1.067

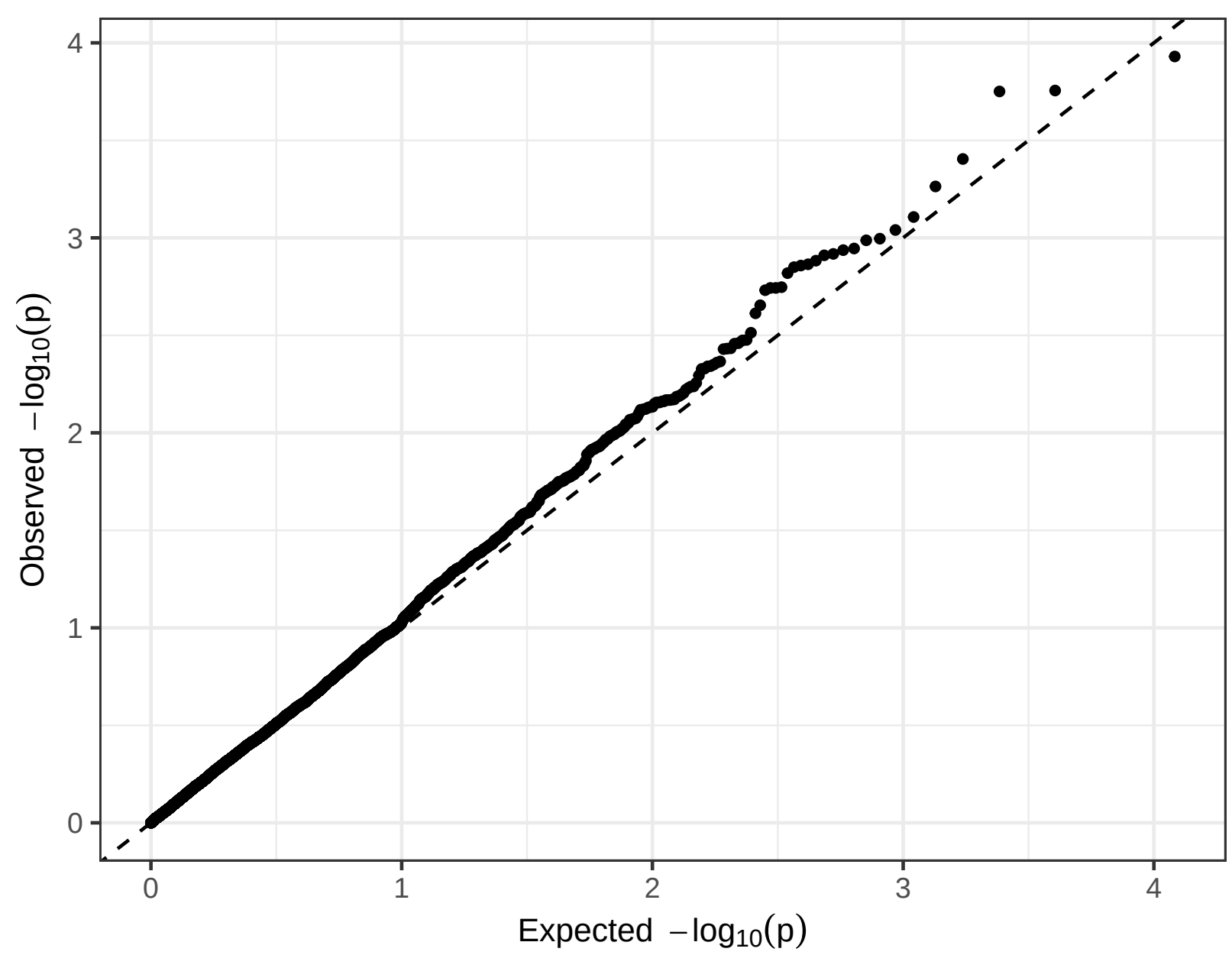

UCG.008

Feces | Brain | . = 0.944

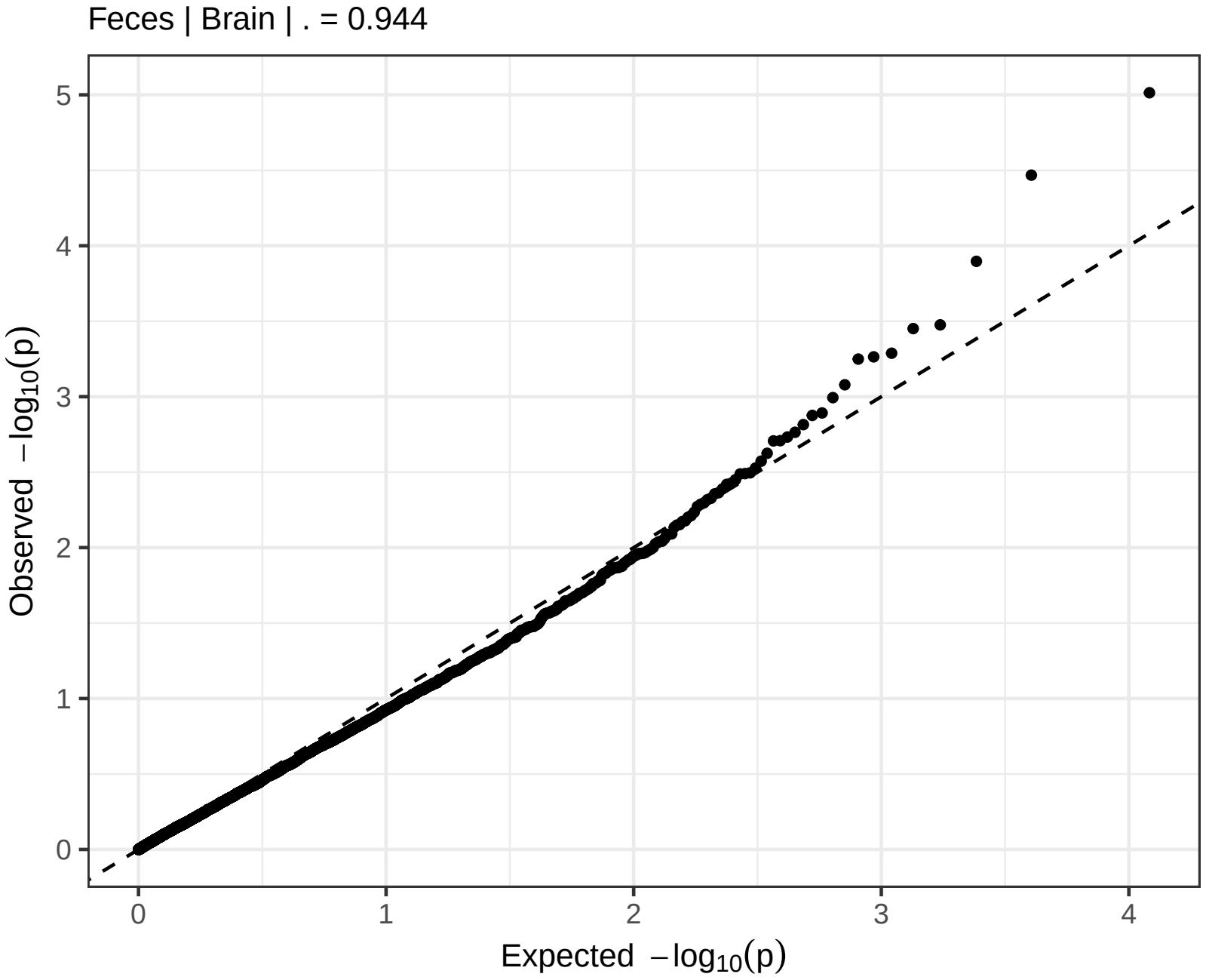

UCG.009

Feces | Brain | . = 0.905

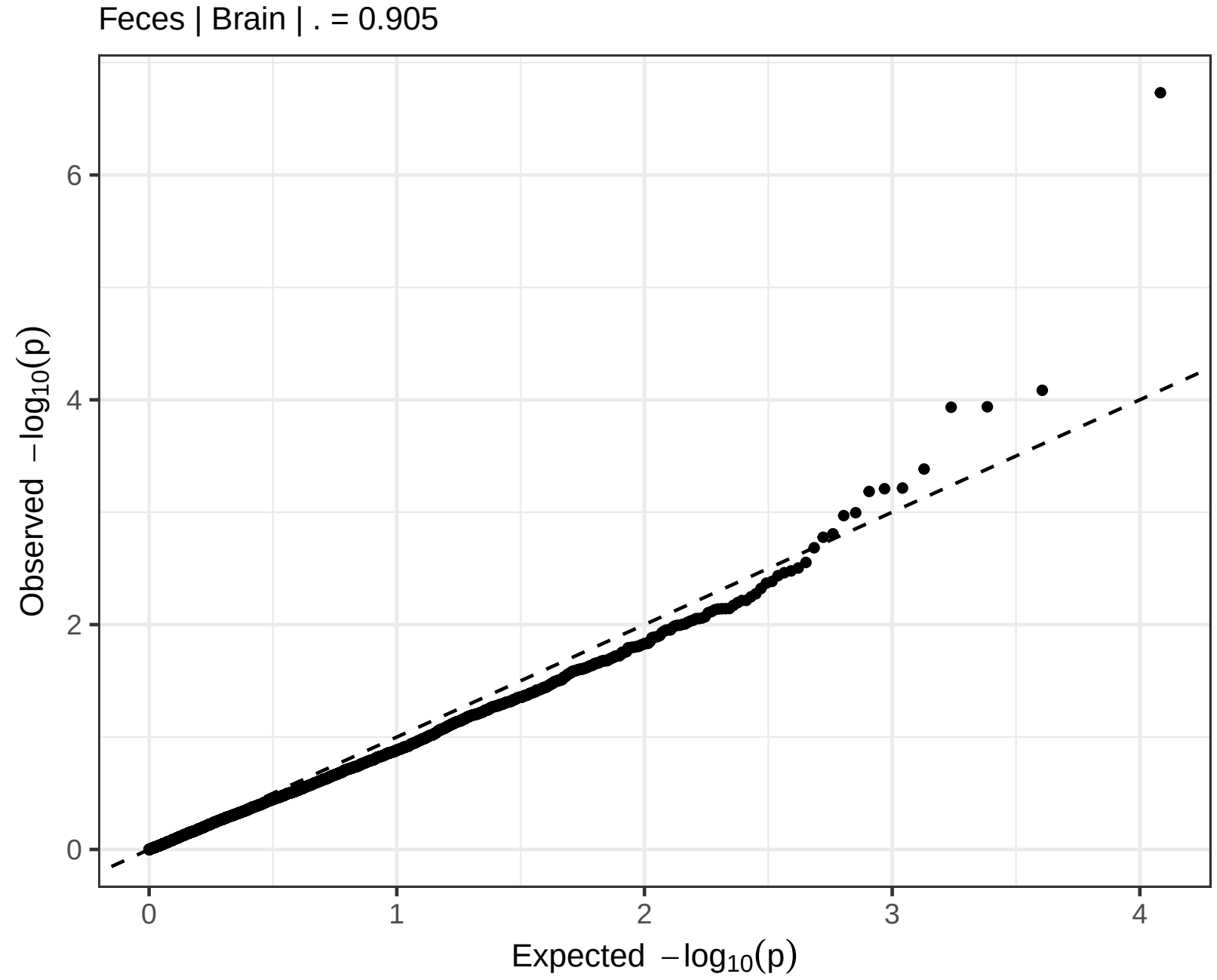

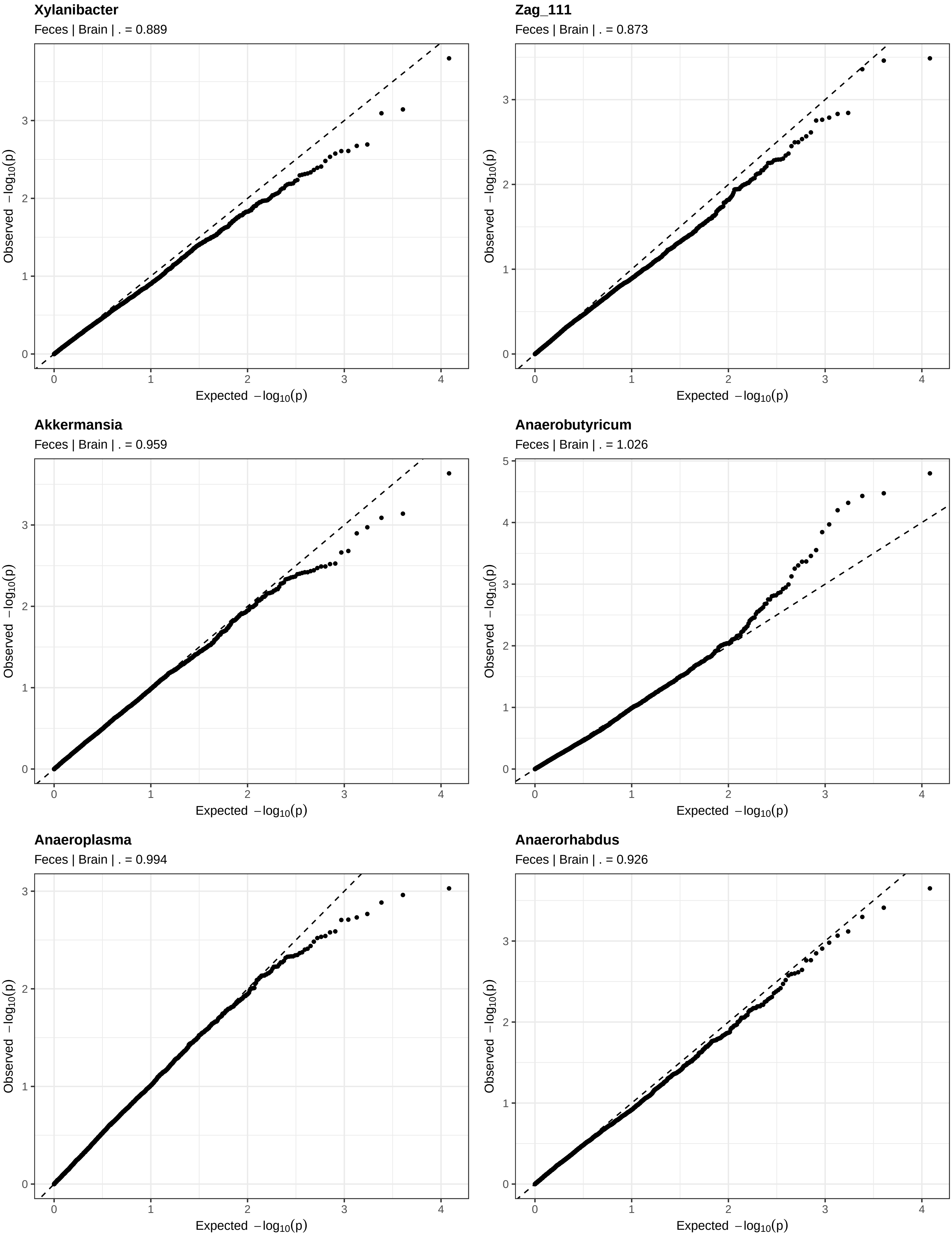

Xylanibacter
Feces | Brain | . = 0.889
Zag_111
Feces | Brain | . = 0.873
Akkermansia
Feces | Brain | . = 0.959
Anaerobutyricum
Feces | Brain | . = 1.026
Anaeroplasma
Feces | Brain | . = 0.994
Anaerorhabdus
Feces | Brain | . = 0.926
Observed $-\log_{10}(p)$
Expected $-\log_{10}(p)$

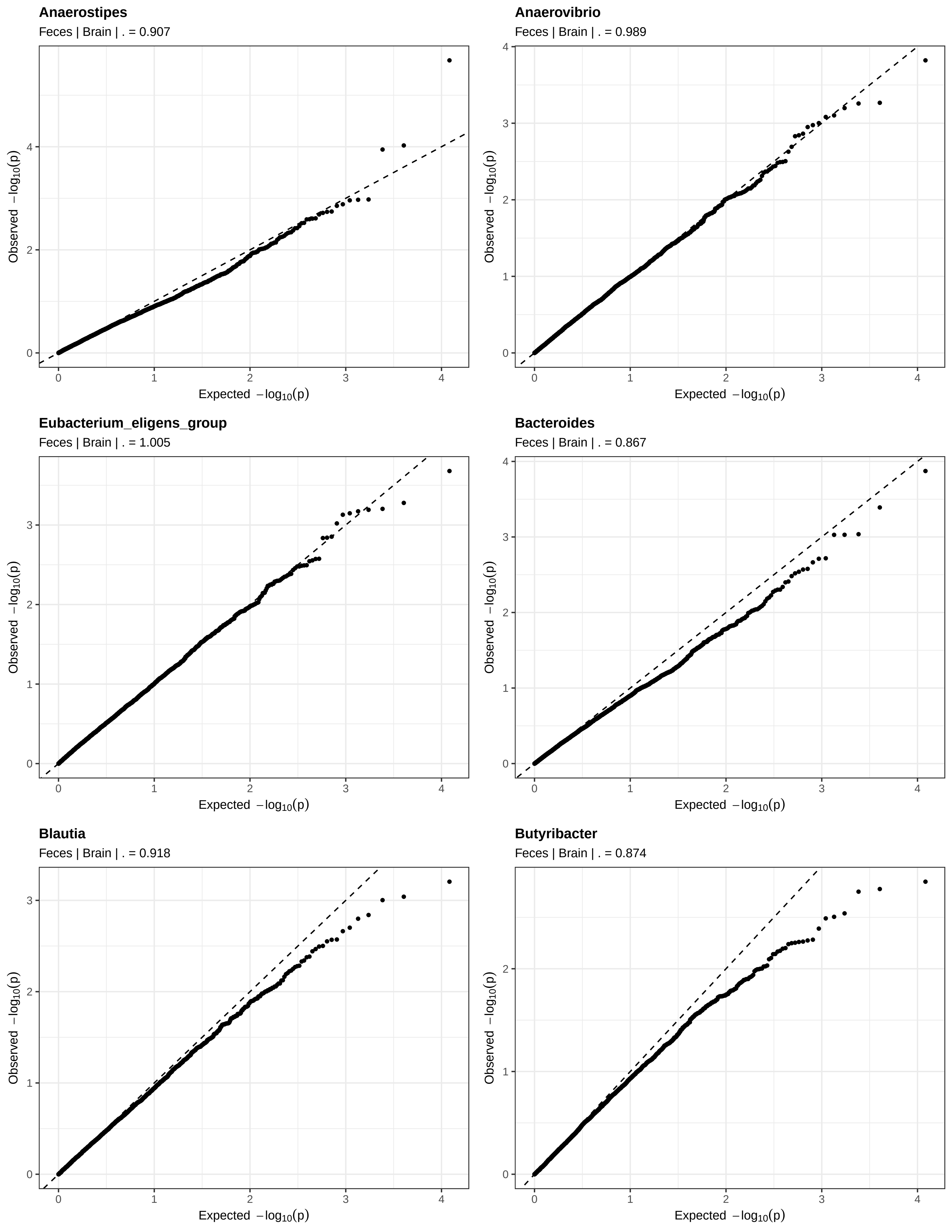

Anaerostipes
Feces | Brain | . = 0.907
Anaerovibrio
Feces | Brain | . = 0.989
Eubacterium_eligens_group
Feces | Brain | . = 1.005
Bacteroides
Feces | Brain | . = 0.867
Blautia
Feces | Brain | . = 0.918
Butyribacter
Feces | Brain | . = 0.874
Observed $-\log_{10}(p)$
Expected $-\log_{10}(p)$

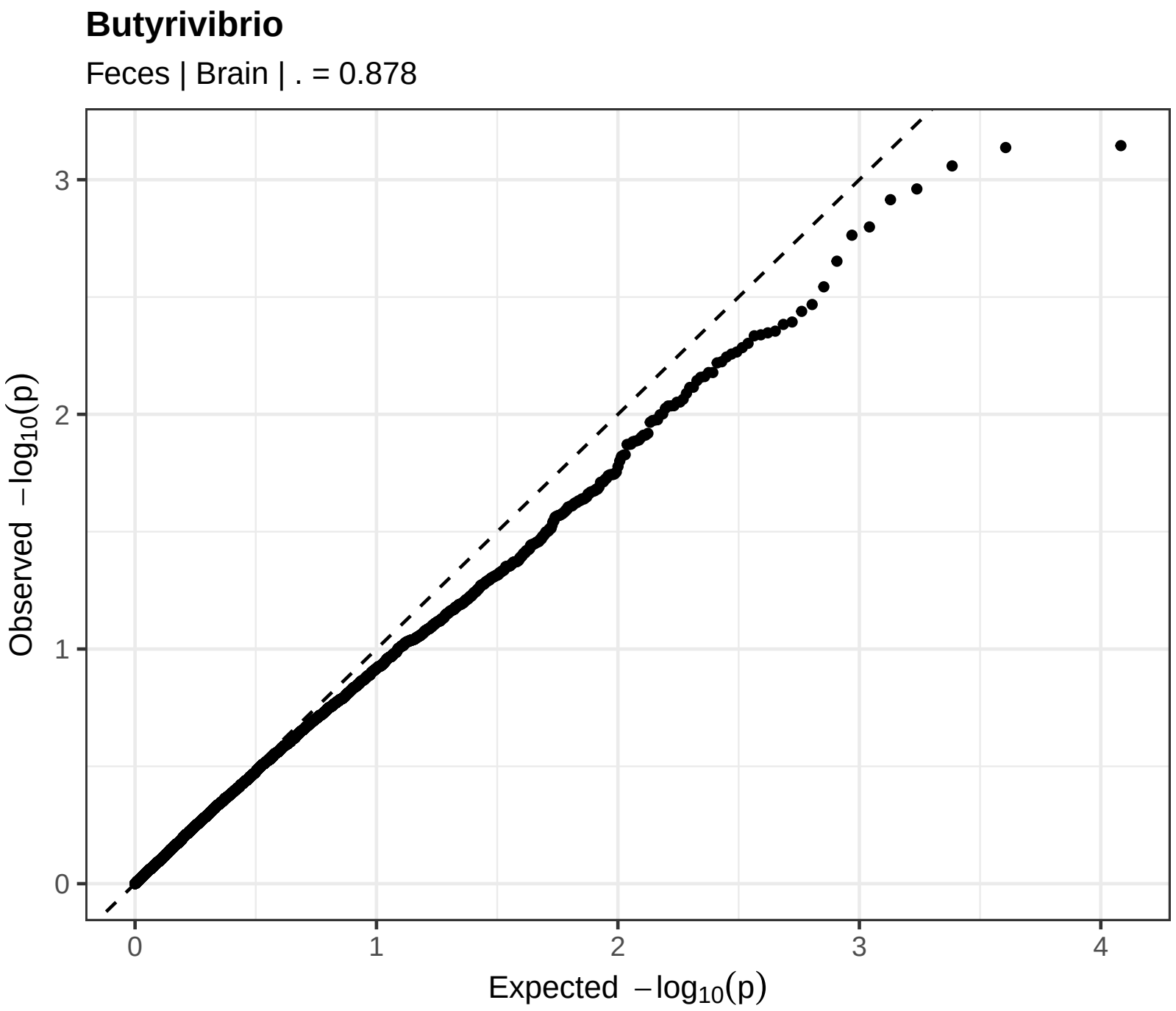
Butyrivibrio
Feces | Brain | . = 0.878
Observed $-\log_{10}(p)$
Expected $-\log_{10}(p)$

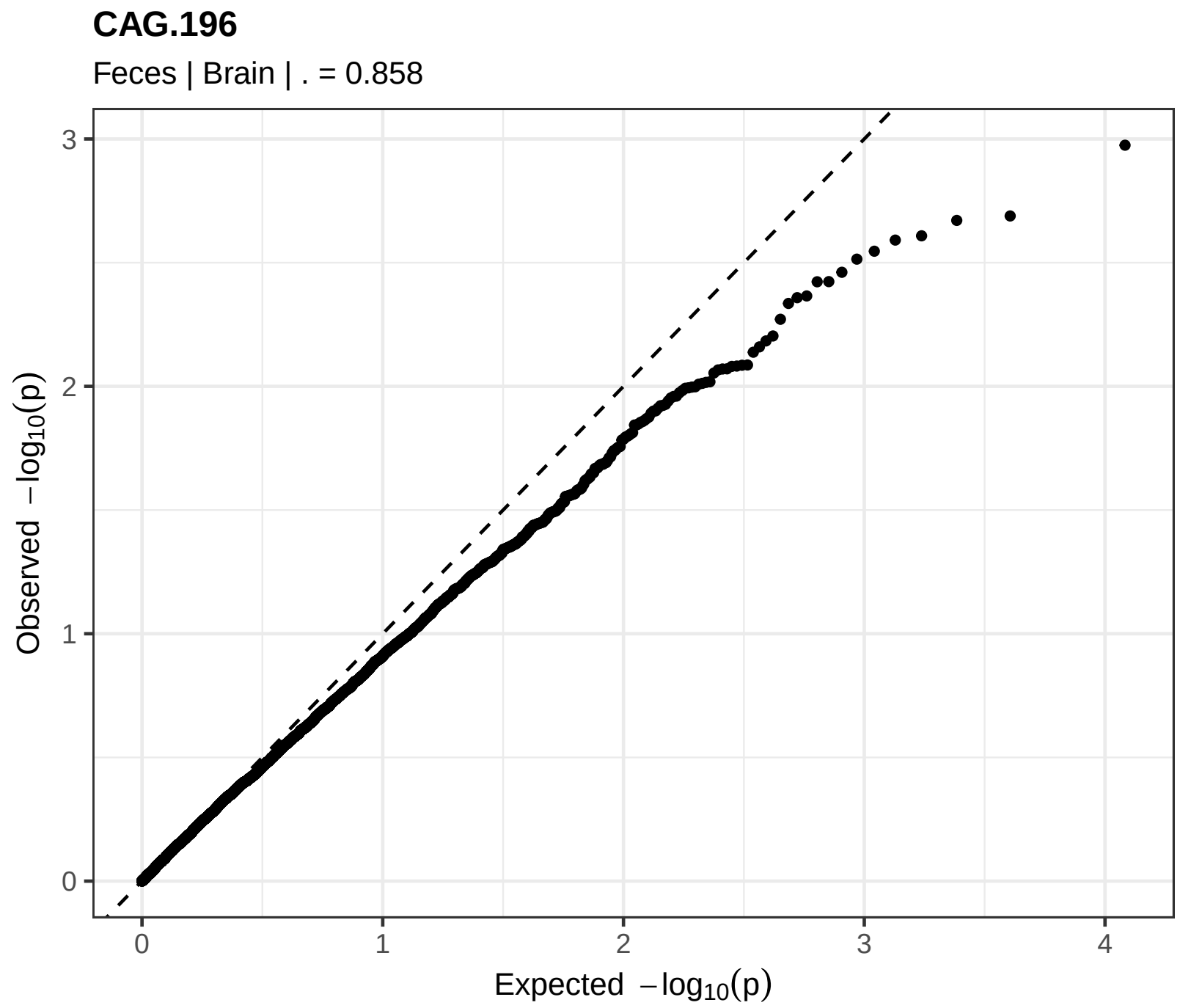
CAG.196
Feces | Brain | . = 0.858
Observed $-\log_{10}(p)$
Expected $-\log_{10}(p)$

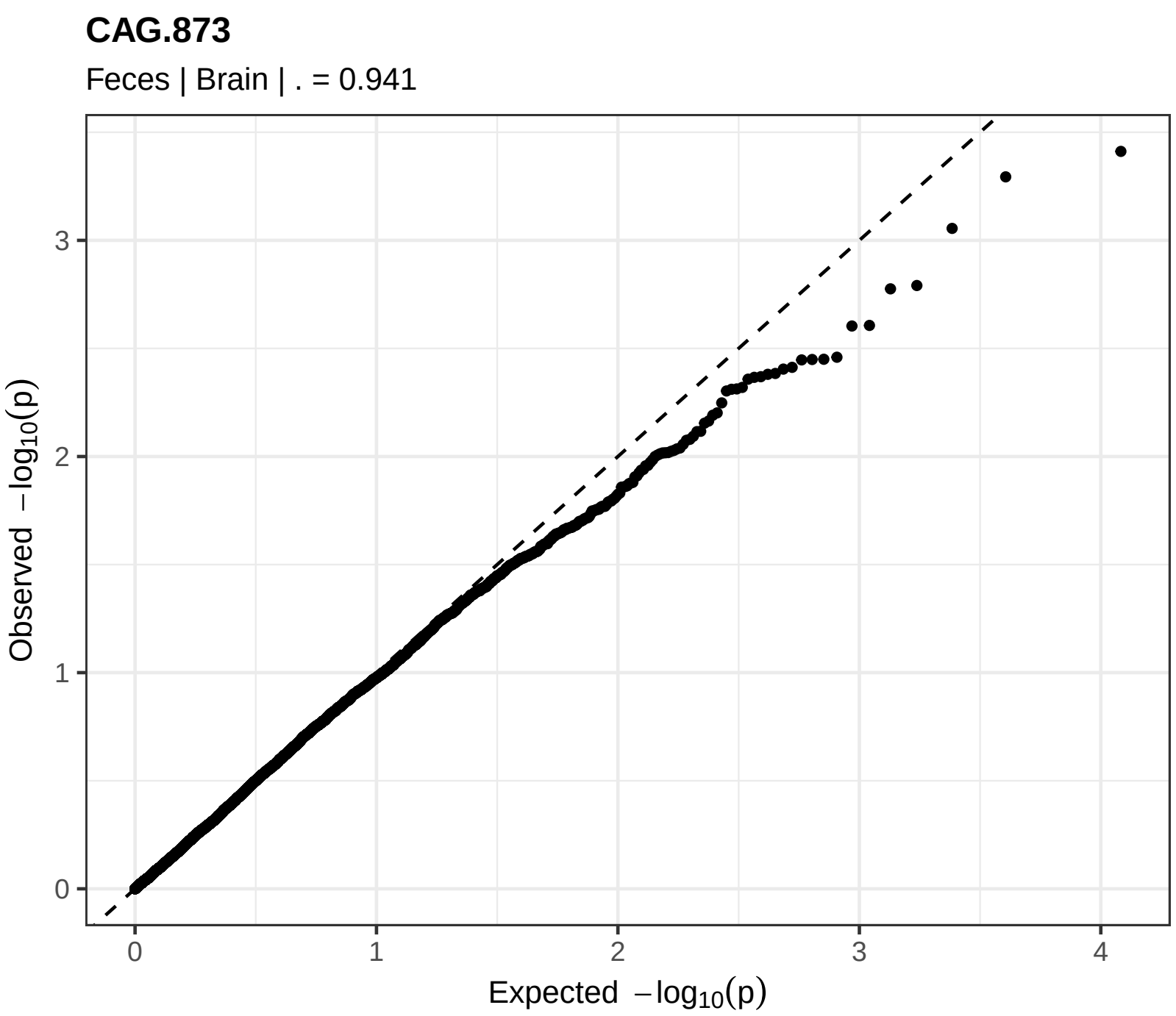
CAG.873
Feces | Brain | . = 0.941
Observed $-\log_{10}(p)$
Expected $-\log_{10}(p)$

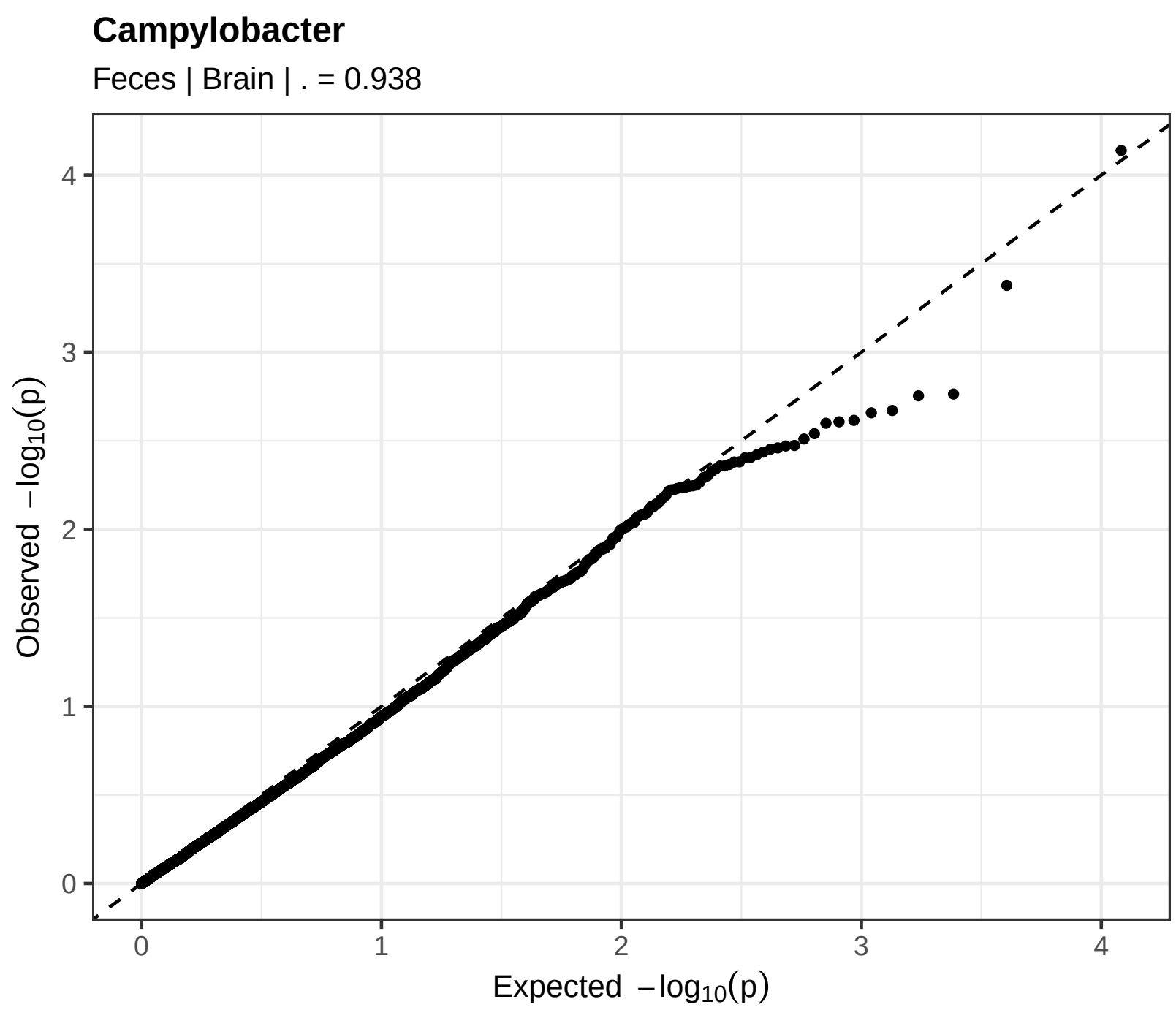
Campylobacter
Feces | Brain | . = 0.938
Observed $-\log_{10}(p)$
Expected $-\log_{10}(p)$

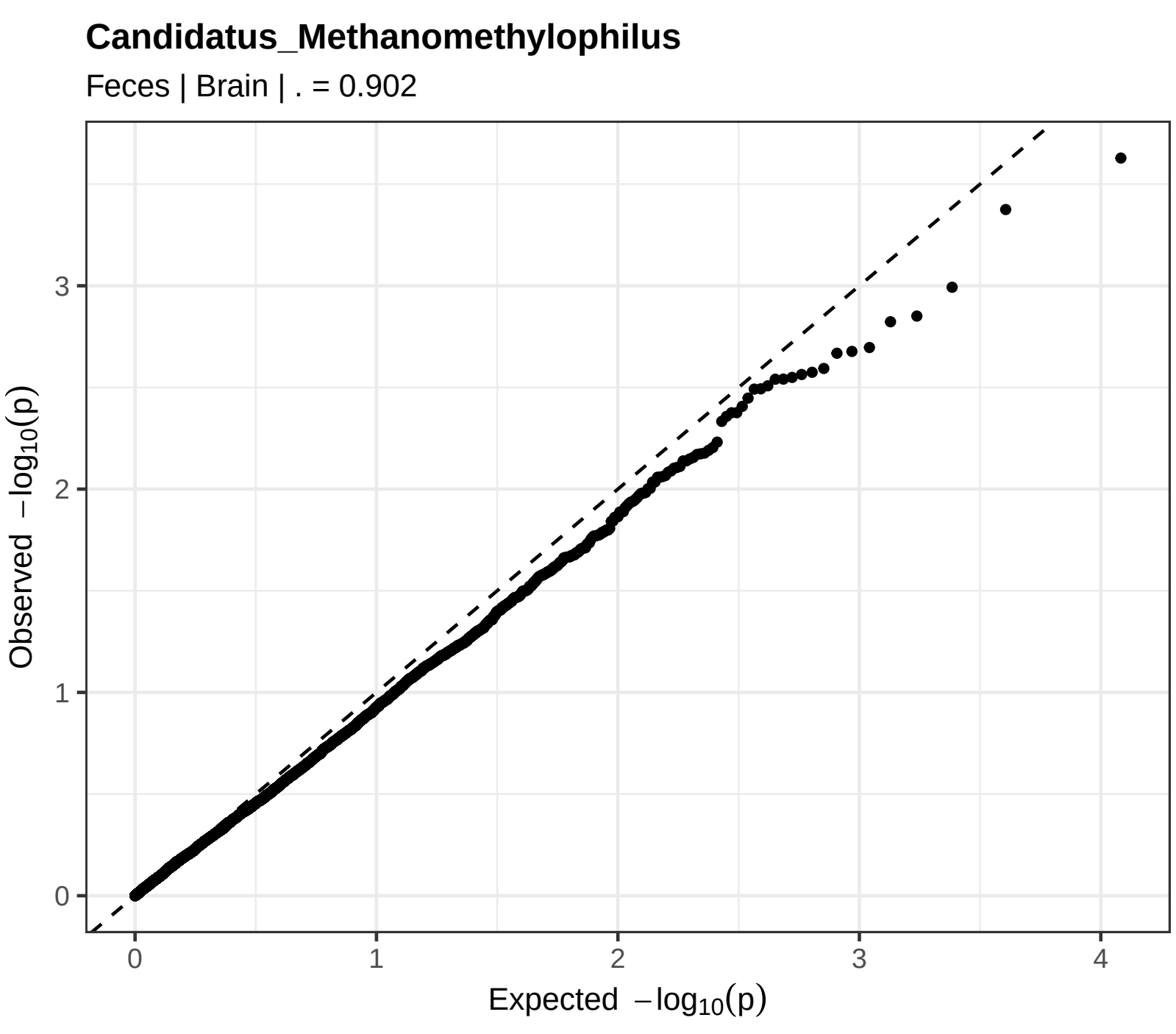
Candidatus_Methanomethylophilus
Feces | Brain | . = 0.902
Observed $-\log_{10}(p)$
Expected $-\log_{10}(p)$

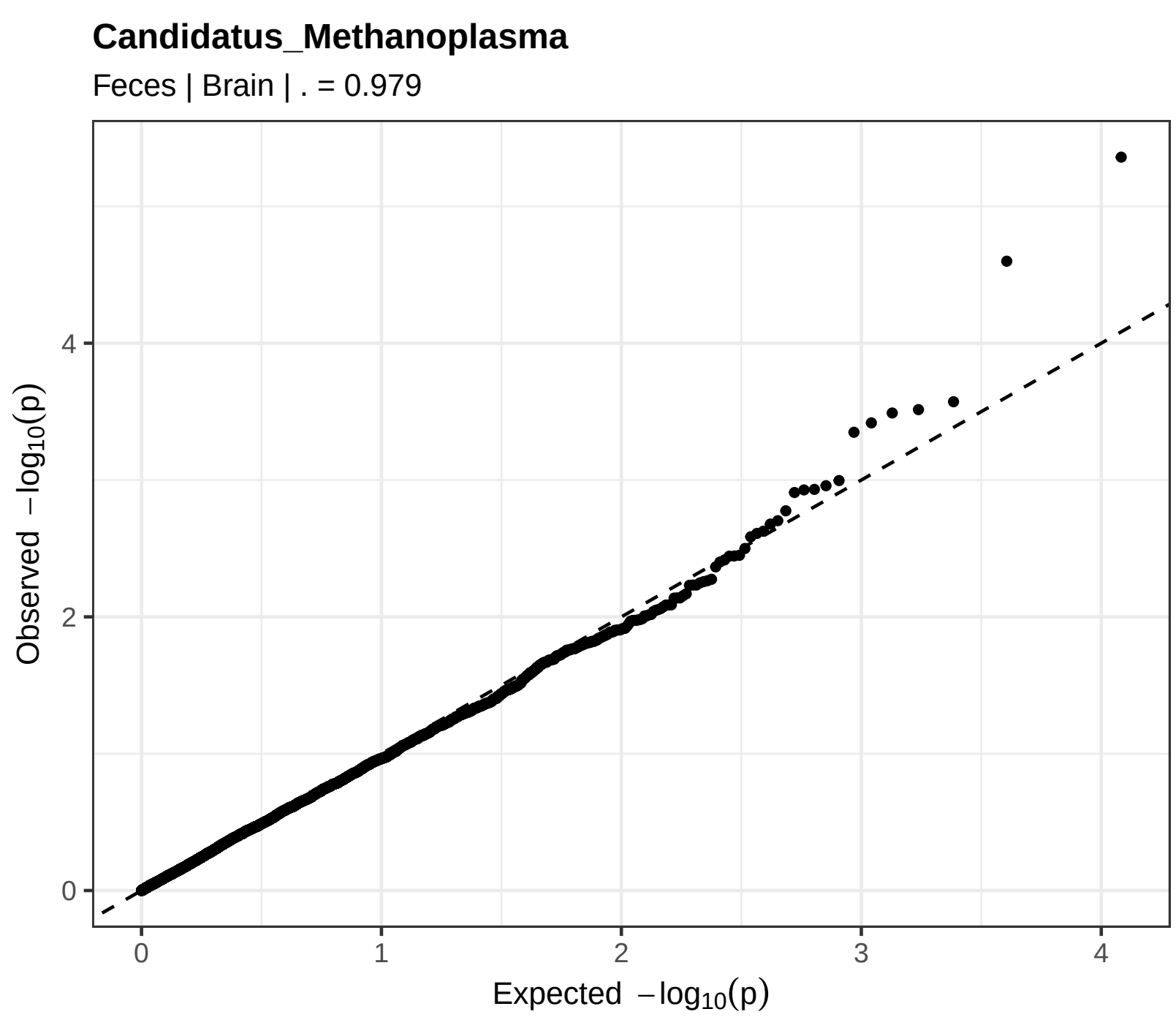
Candidatus_Methanoplasma
Feces | Brain | . = 0.979
Observed $-\log_{10}(p)$
Expected $-\log_{10}(p)$

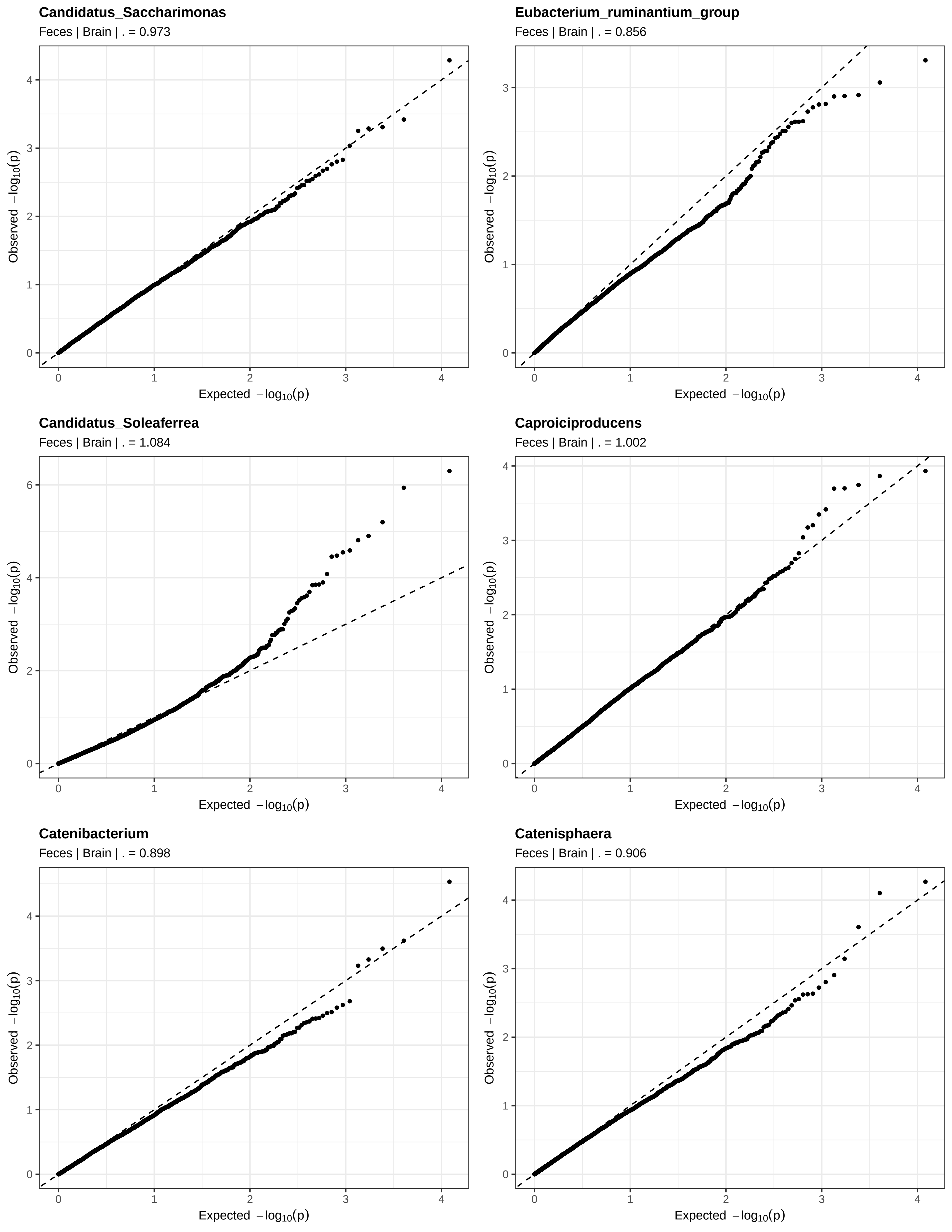
Candidatus_Saccharimonas
Feces | Brain | . = 0.973
Eubacterium_ruminantium_group
Feces | Brain | . = 0.856
Candidatus_Soleaferrea
Feces | Brain | . = 1.084
Caproiciproducens
Feces | Brain | . = 1.002
Catenibacterium
Feces | Brain | . = 0.898
Catenisphaera
Feces | Brain | . = 0.906
Observed $-\log_{10}(p)$
Expected $-\log_{10}(p)$
0
1
2
3
4
6

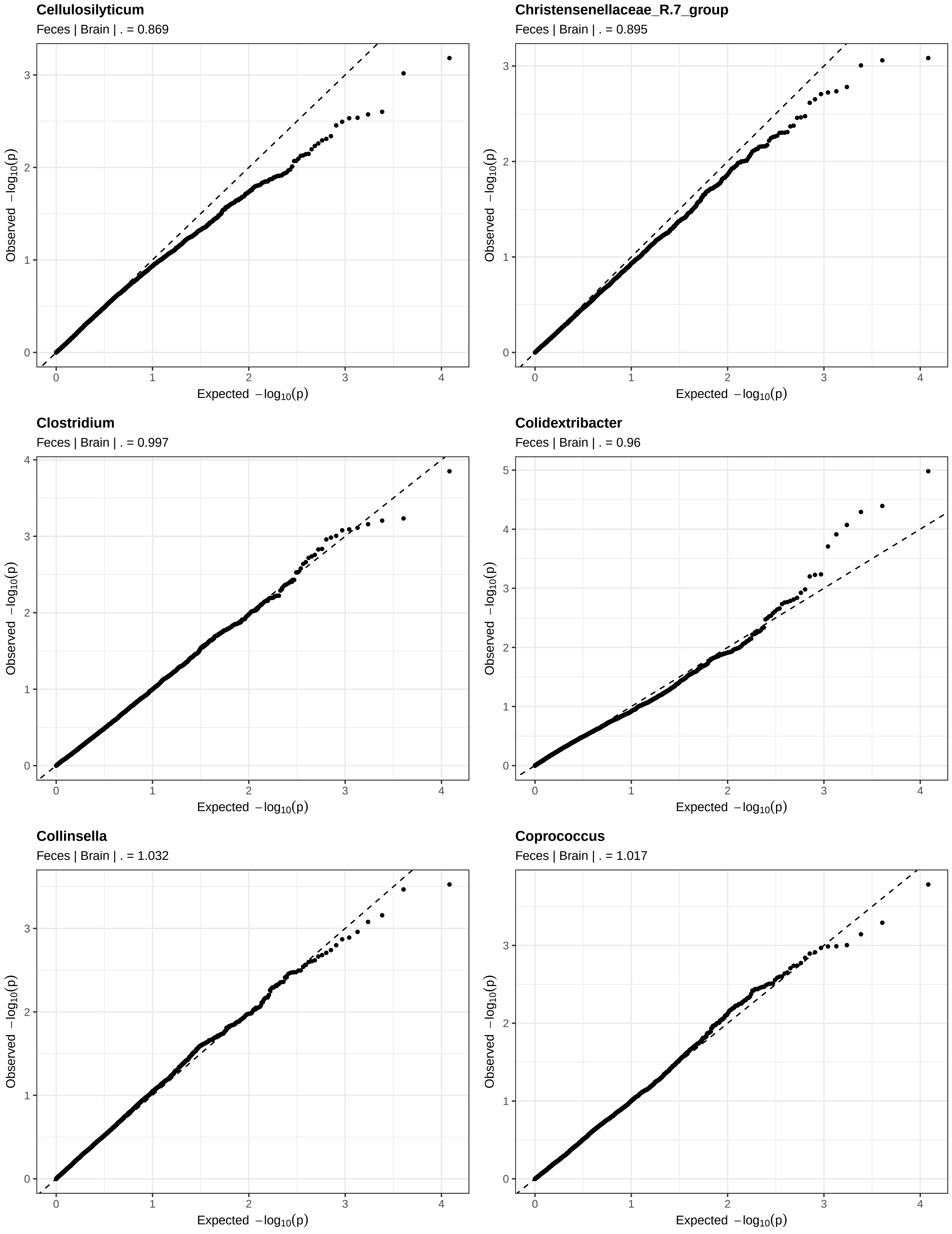
Cellulosilyticum
Feces | Brain | . = 0.869
Christensenellaceae_R.7_group
Feces | Brain | . = 0.895
Clostridium
Feces | Brain | . = 0.997
Colidextribacter
Feces | Brain | . = 0.96
Collinsella
Feces | Brain | . = 1.032
Coprococcus
Feces | Brain | . = 1.017
Observed −log10(p)
Expected −log10(p)

Eubacterium_siraeum_group

Feces | Brain | . = 1.042

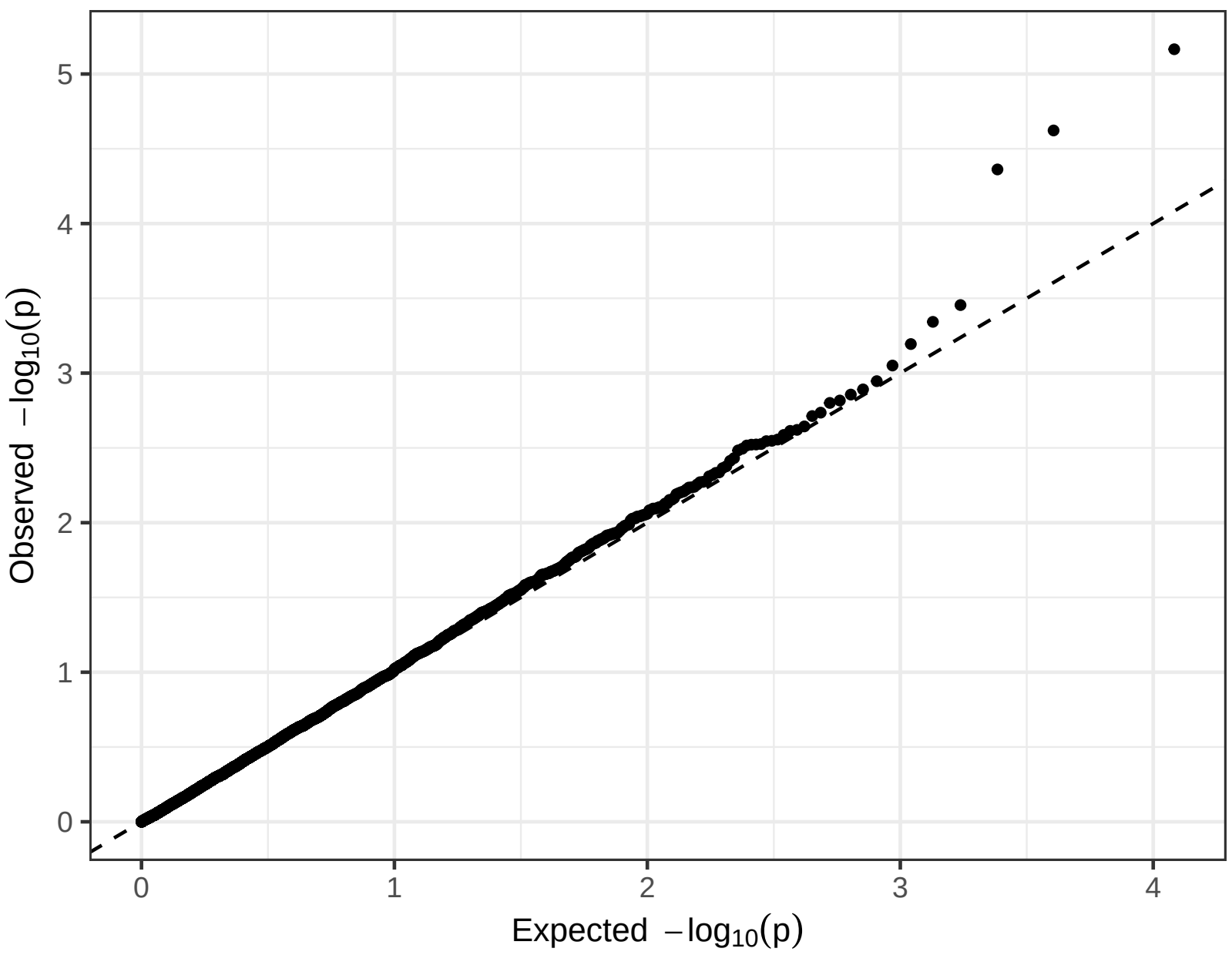

Desulfovibrio

Feces | Brain | . = 0.934

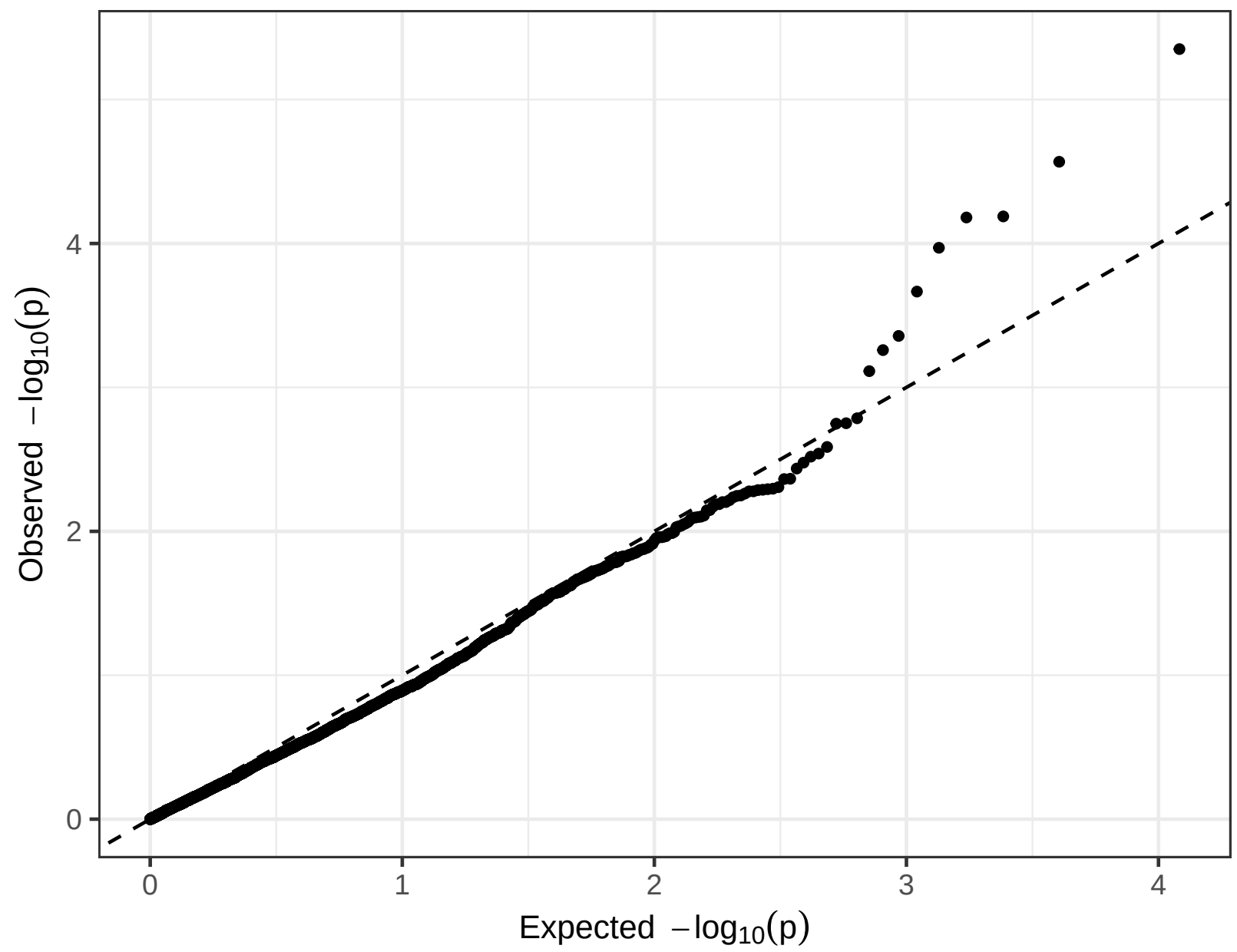

dgA.11_gut_group

Feces | Brain | . = 0.983

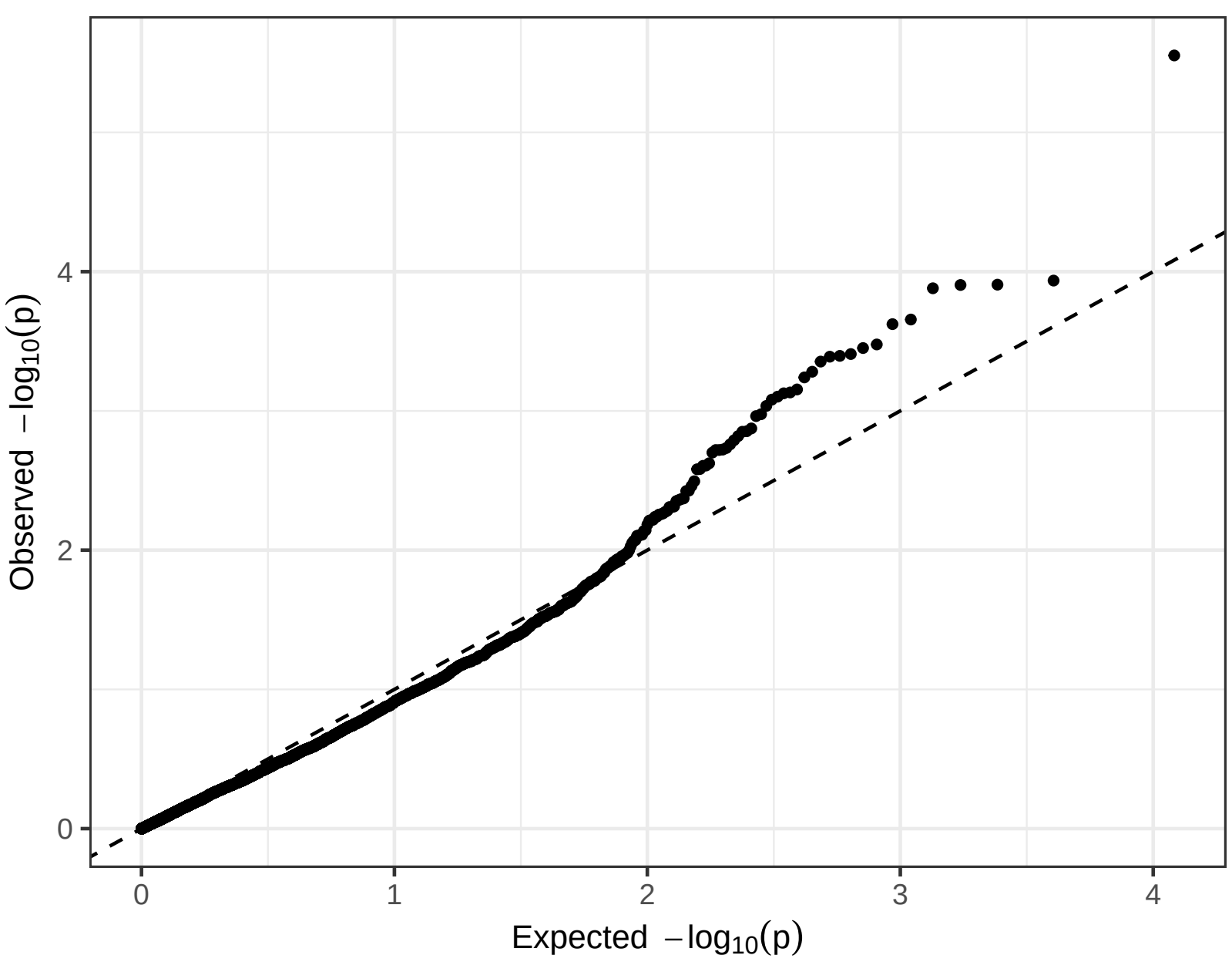

Dorea

Feces | Brain | . = 0.906

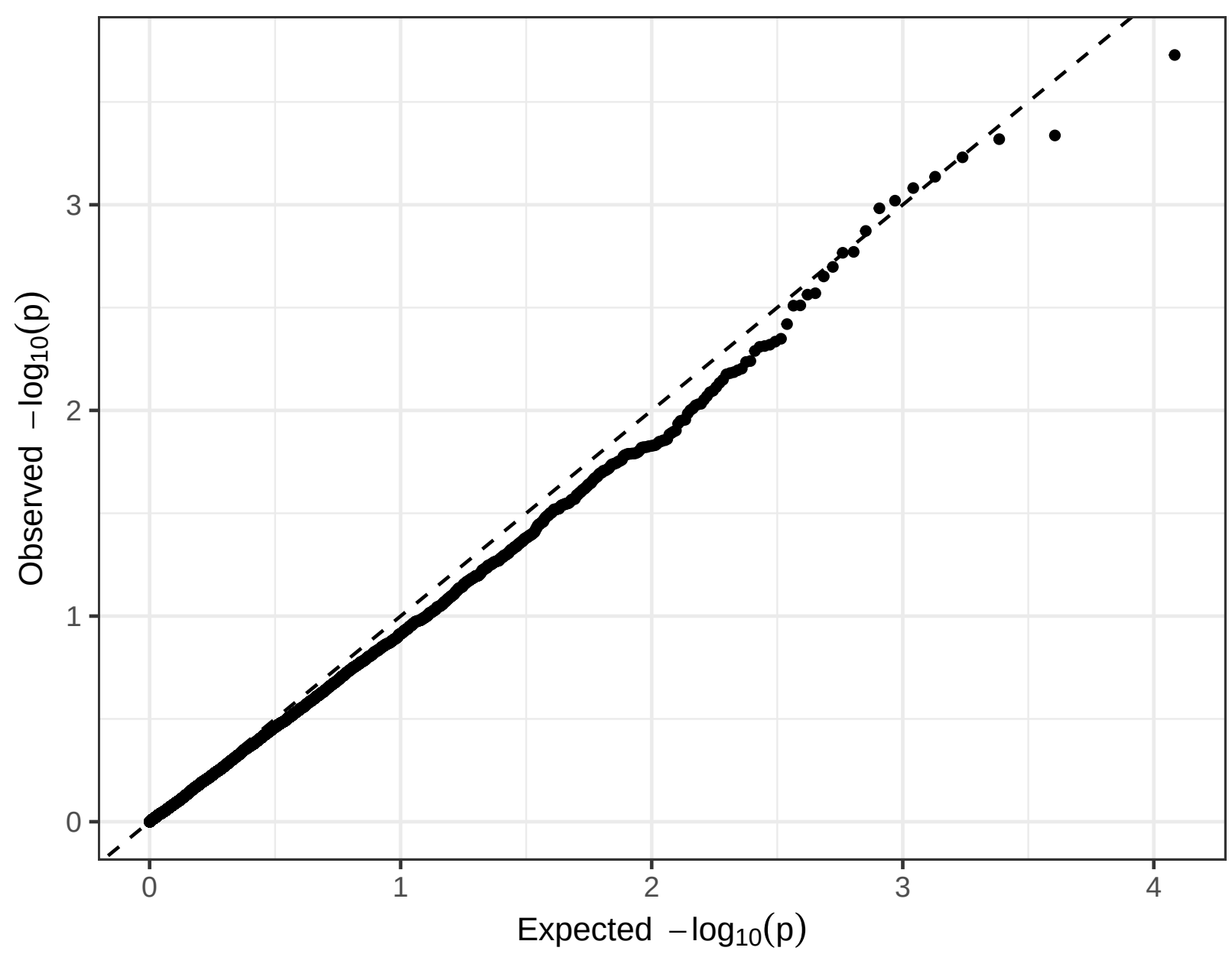

Erysipelotrichaceae_UCG.003

Feces | Brain | . = 0.921

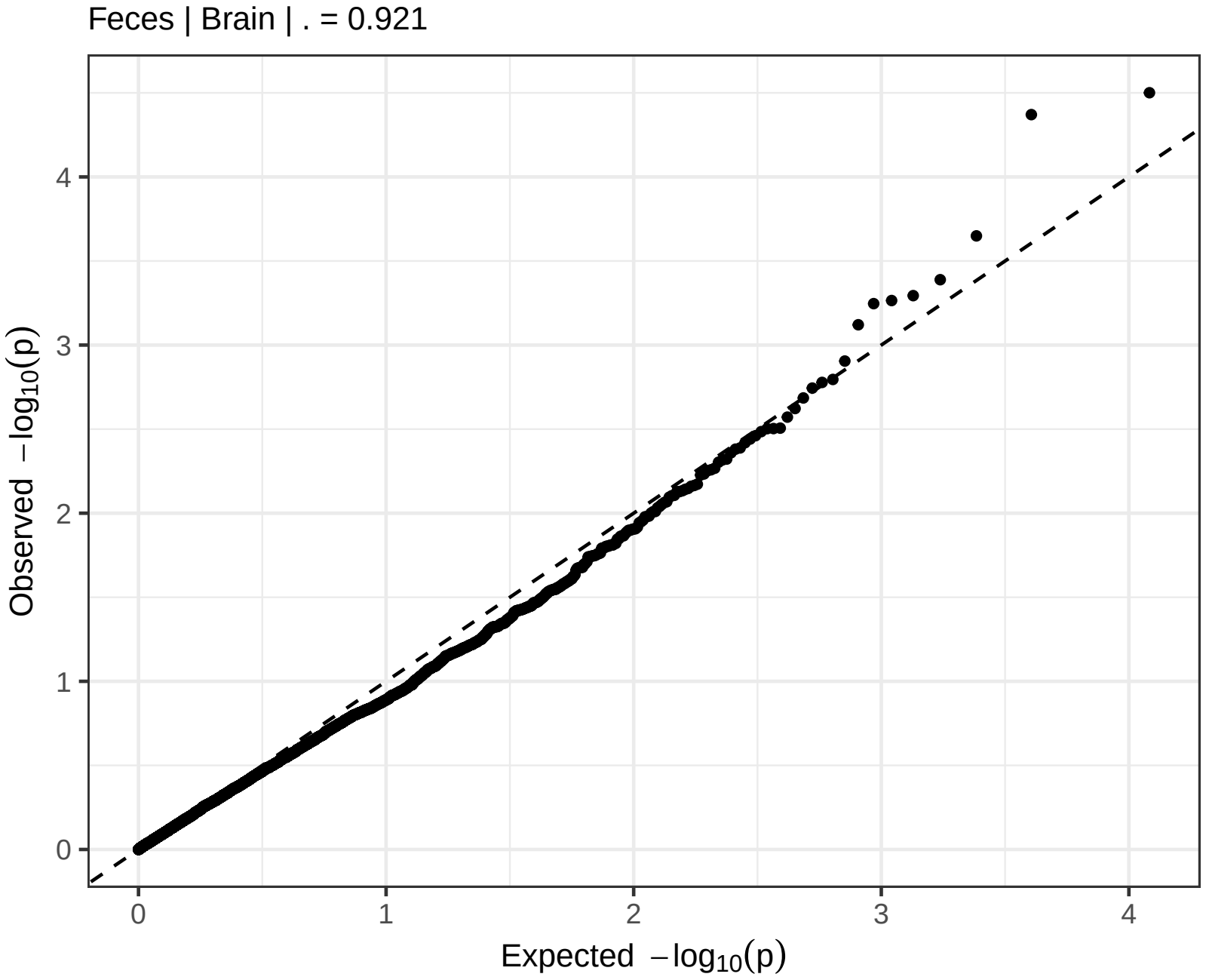

Erysipelotrichaceae_UCG.006

Feces | Brain | . = 0.876

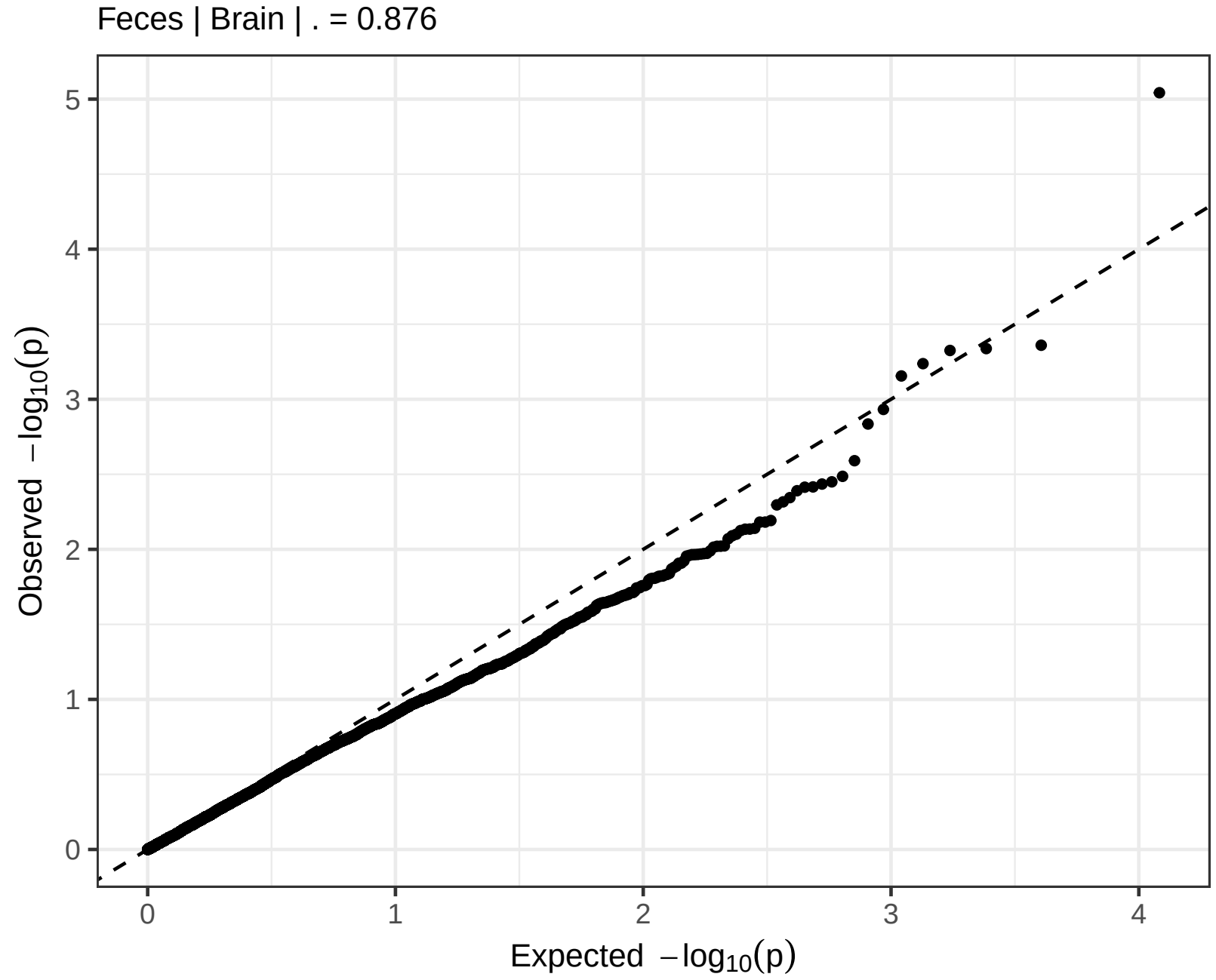

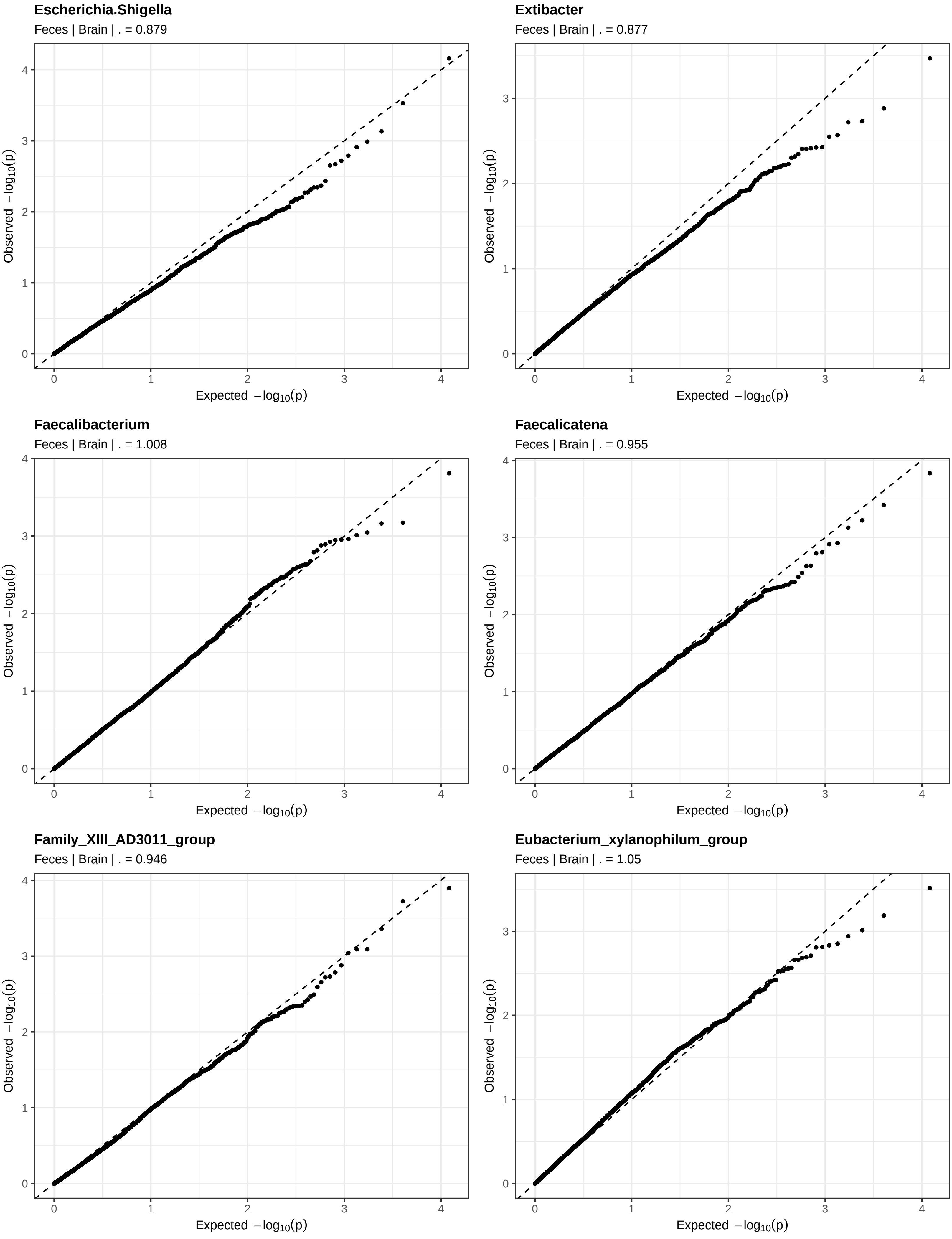
Escherichia.Shigella
Feces | Brain | . = 0.879
Extibacter
Feces | Brain | . = 0.877
Faecalibacterium
Feces | Brain | . = 1.008
Faecalicatena
Feces | Brain | . = 0.955
Family_XIII_AD3011_group
Feces | Brain | . = 0.946
Eubacterium_xylanophilum_group
Feces | Brain | . = 1.05
Observed $-\log_{10}(p)$
Expected $-\log_{10}(p)$
0
1
2
3
4

Family_XIII_UCG.001

Feces | Brain | . = 0.945

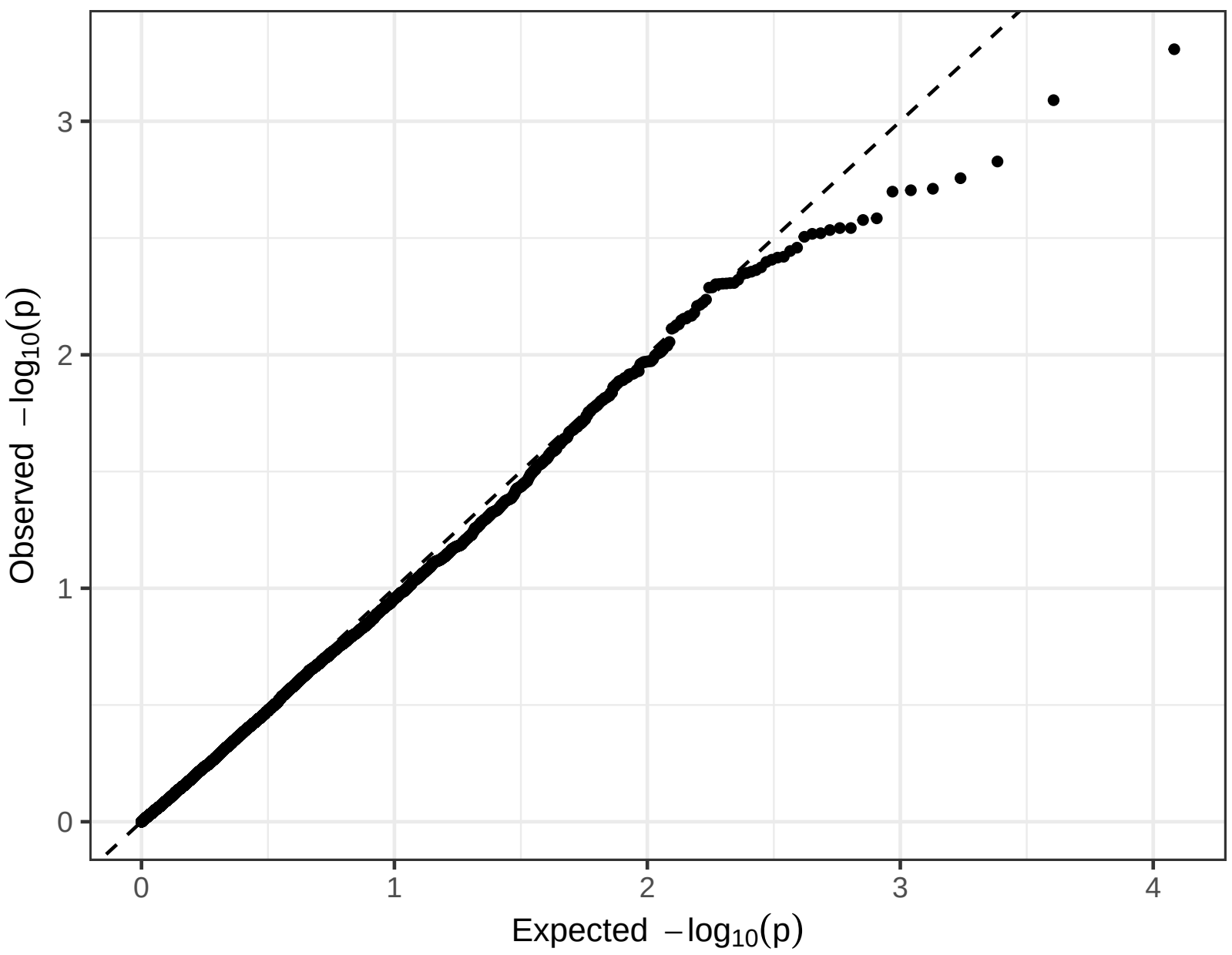

Fibrobacter

Feces | Brain | . = 0.911

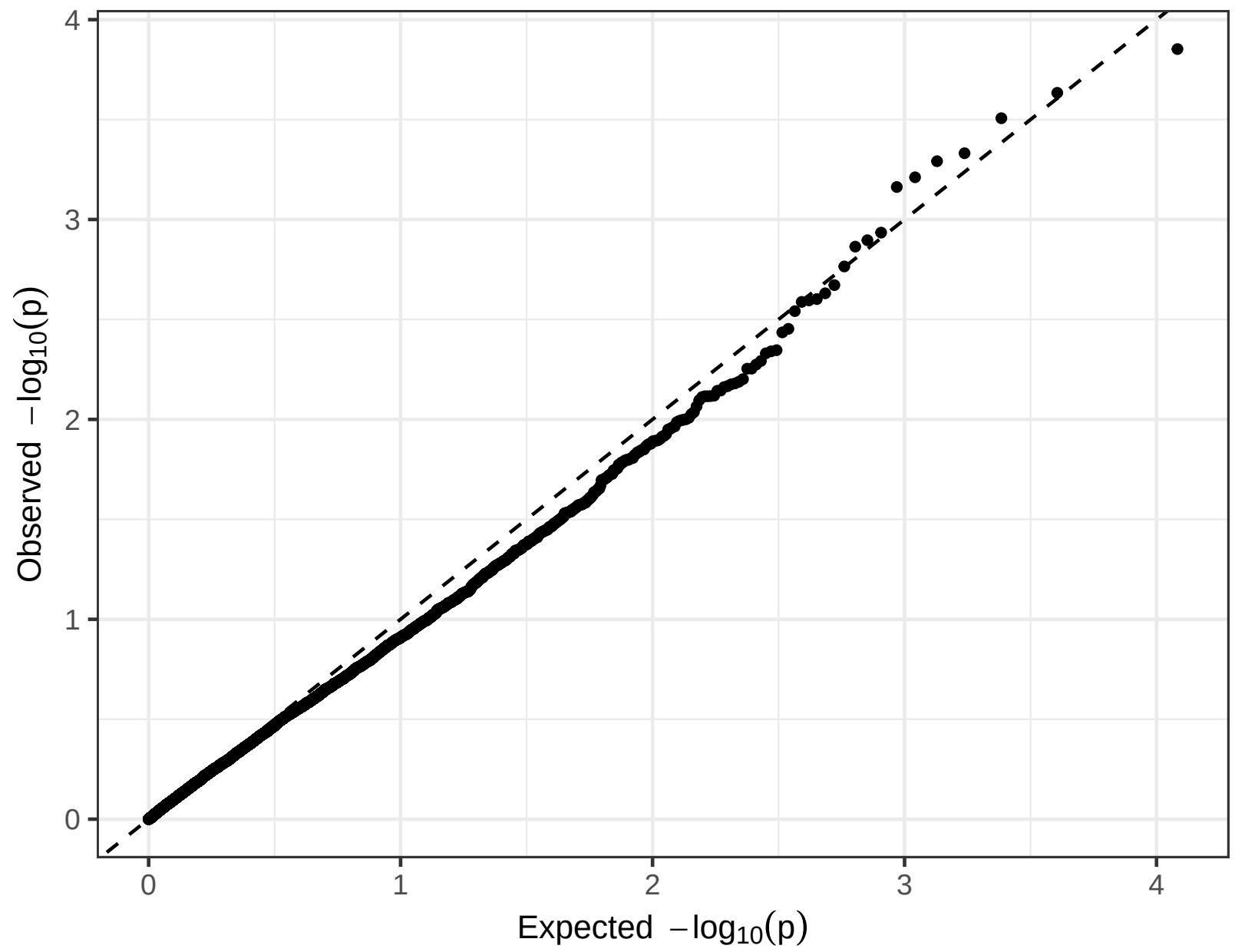

Fournierella

Feces | Brain | . = 0.98

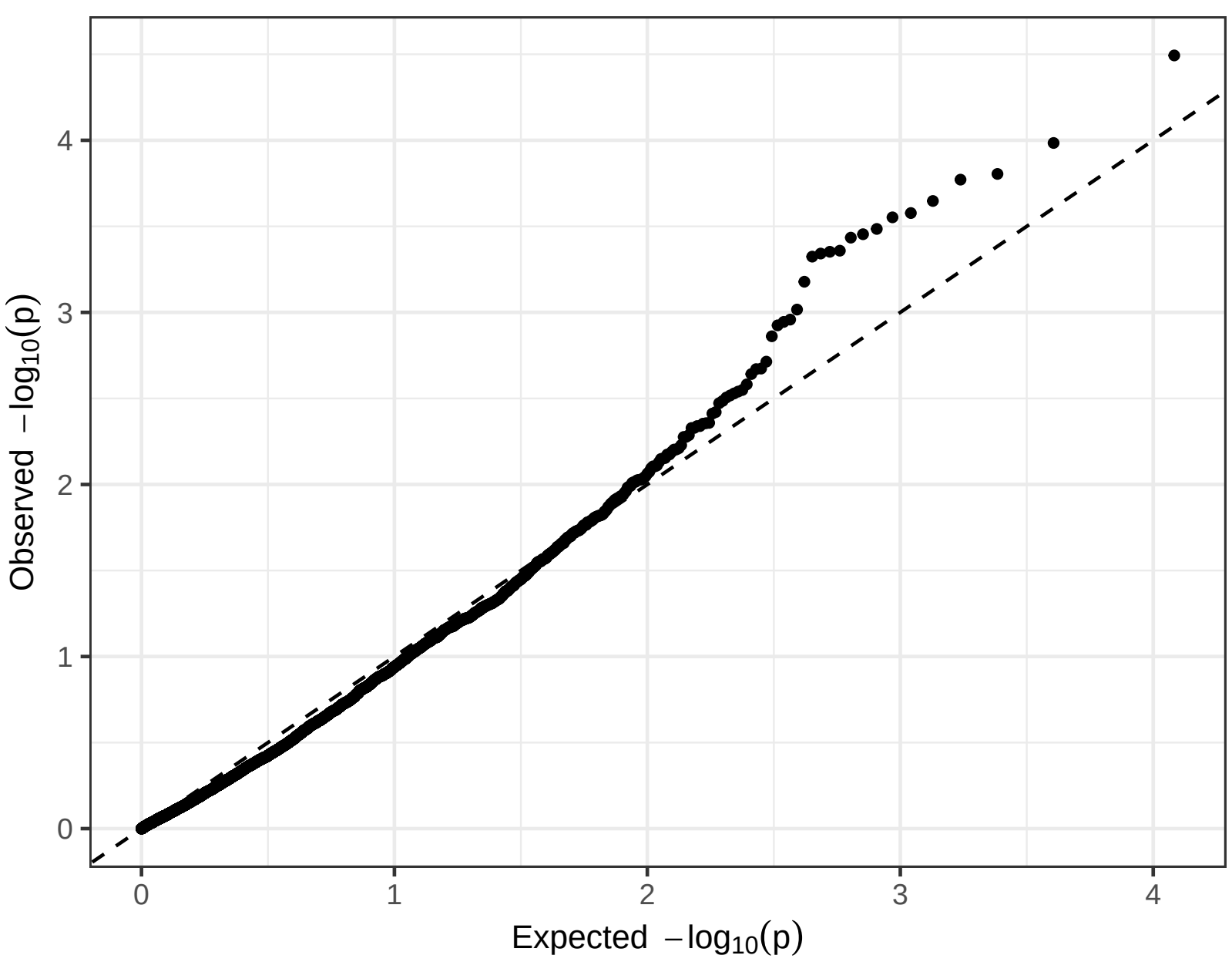

Frisingicoccus

Feces | Brain | . = 1.008

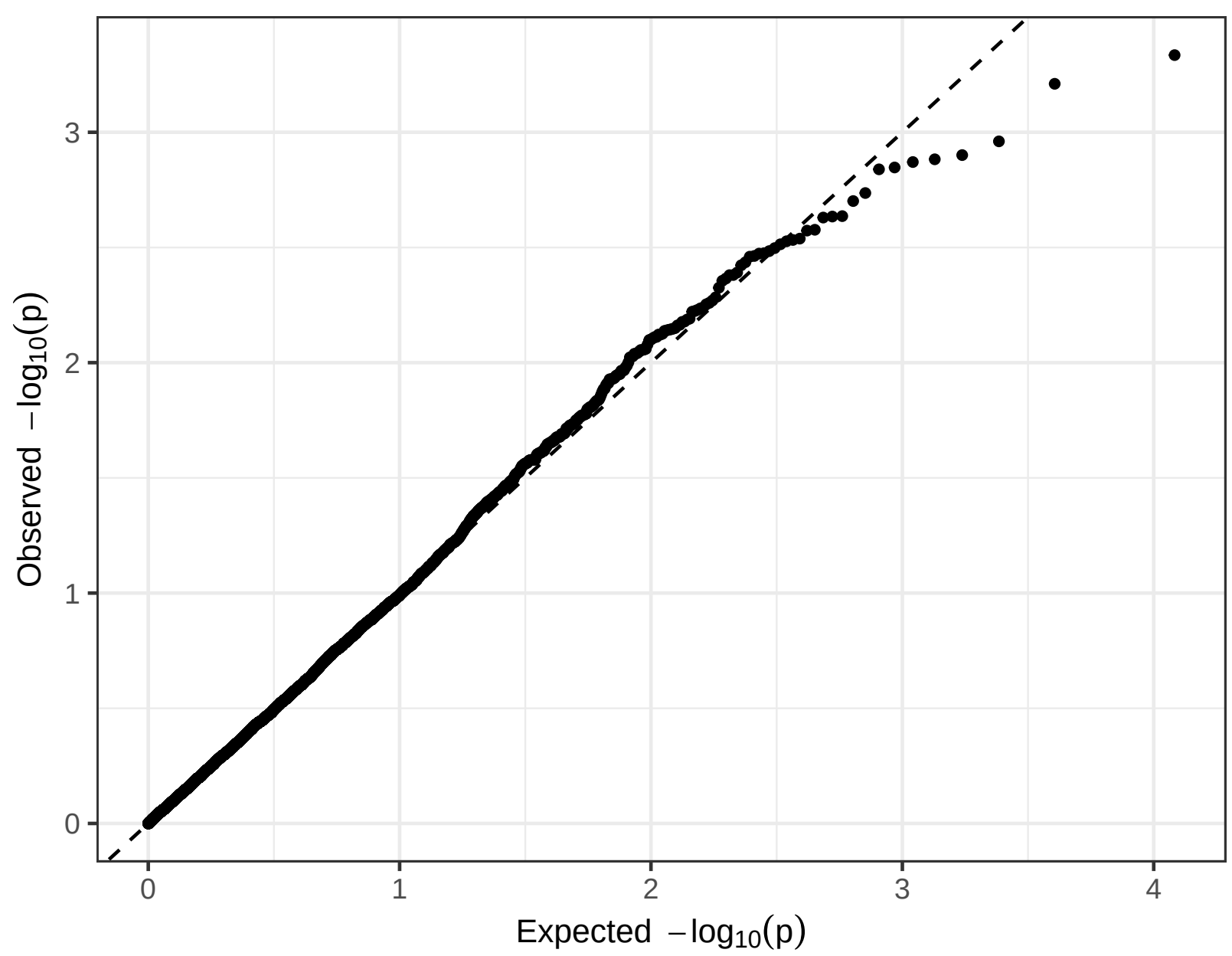

Fusicatenibacter

Feces | Brain | . = 0.846

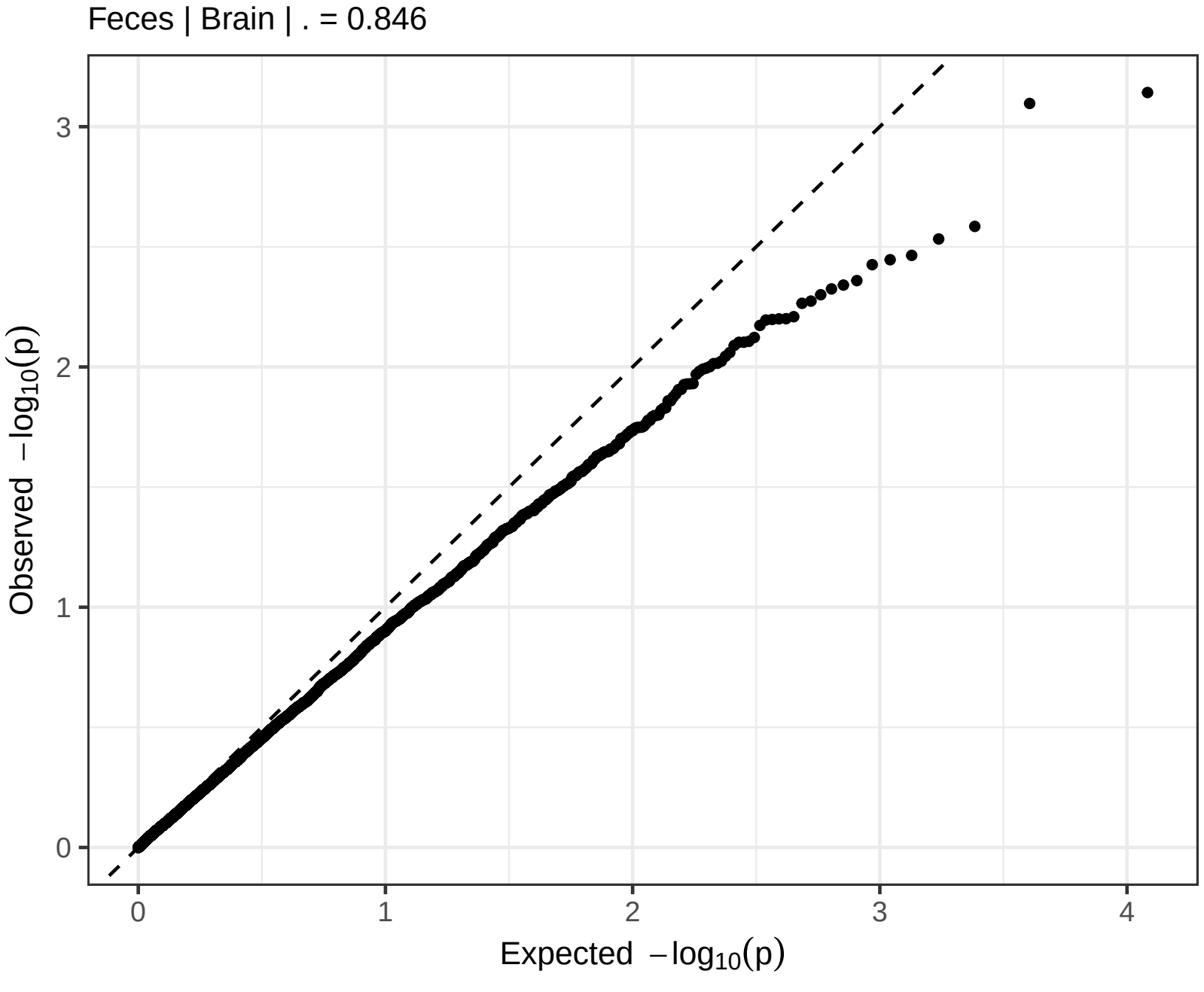

Gemmiger

Feces | Brain | . = 1.095

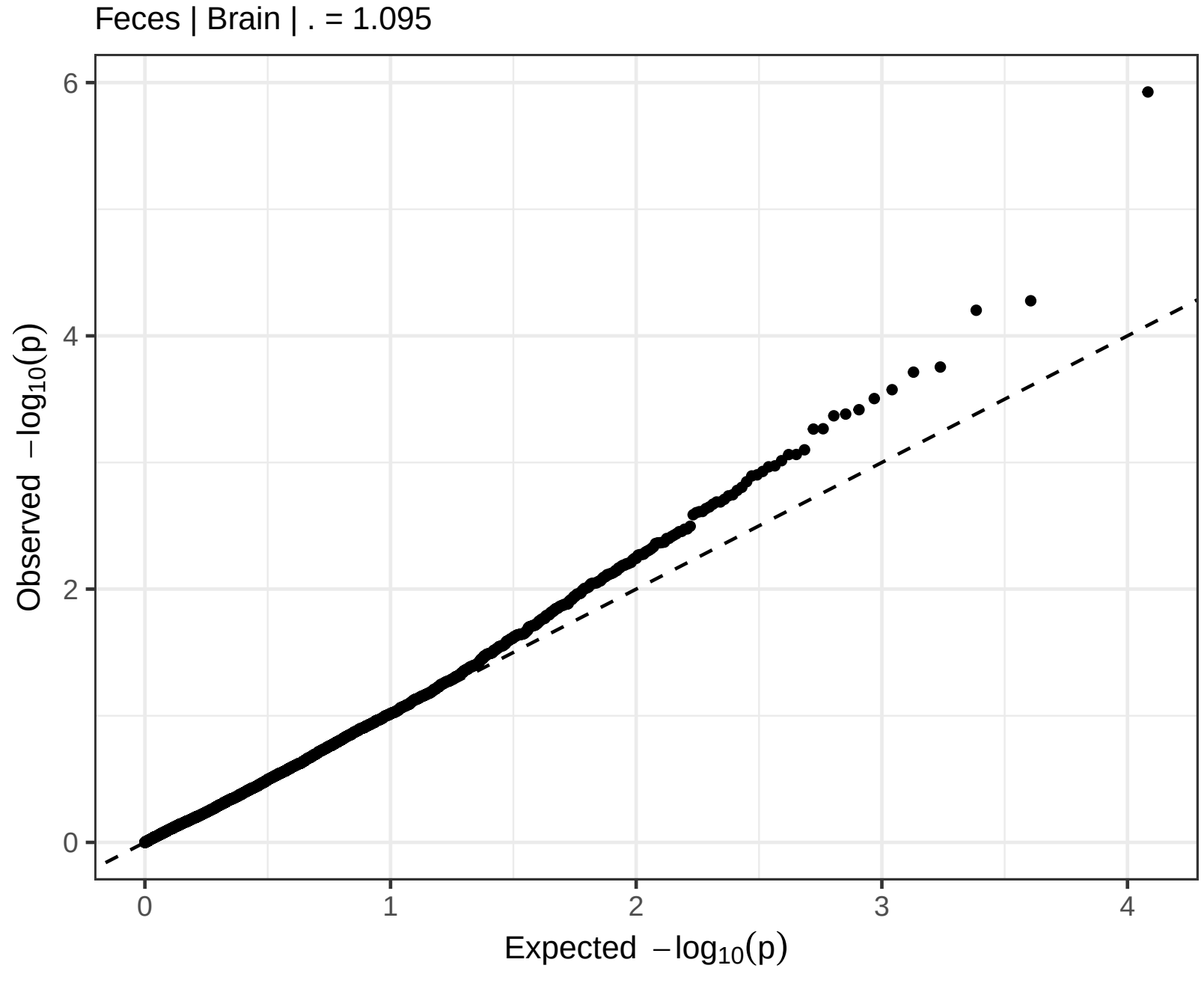

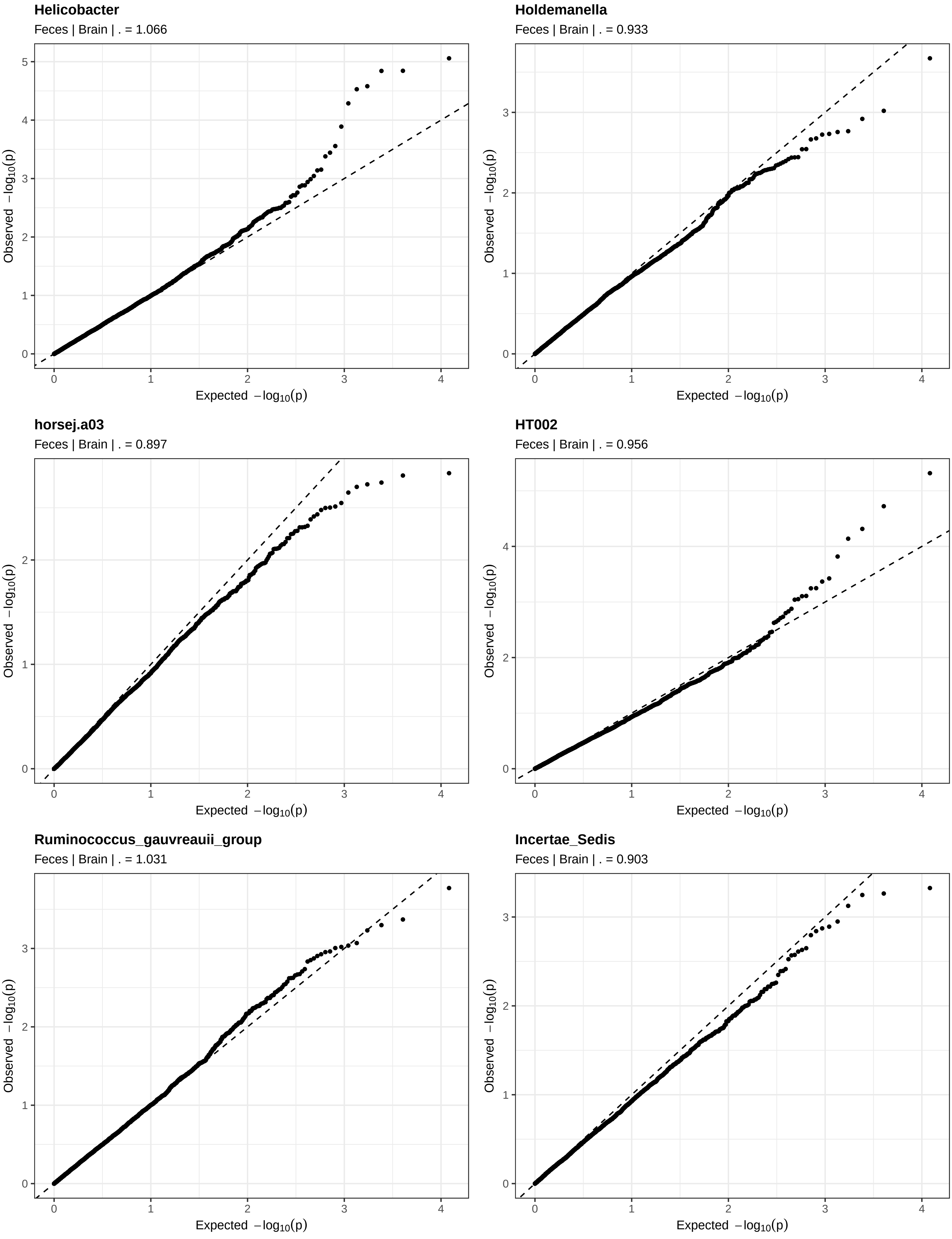

Helicobacter
Feces | Brain | . = 1.066
Holdemanella
Feces | Brain | . = 0.933
horsej.a03
Feces | Brain | . = 0.897
HT002
Feces | Brain | . = 0.956
Ruminococcus_gauvreauii_group
Feces | Brain | . = 1.031
Incertae_Sedis
Feces | Brain | . = 0.903
Observed −log10(p)
Expected −log10(p)

Intestinibacter

Feces | Brain | . = 0.923

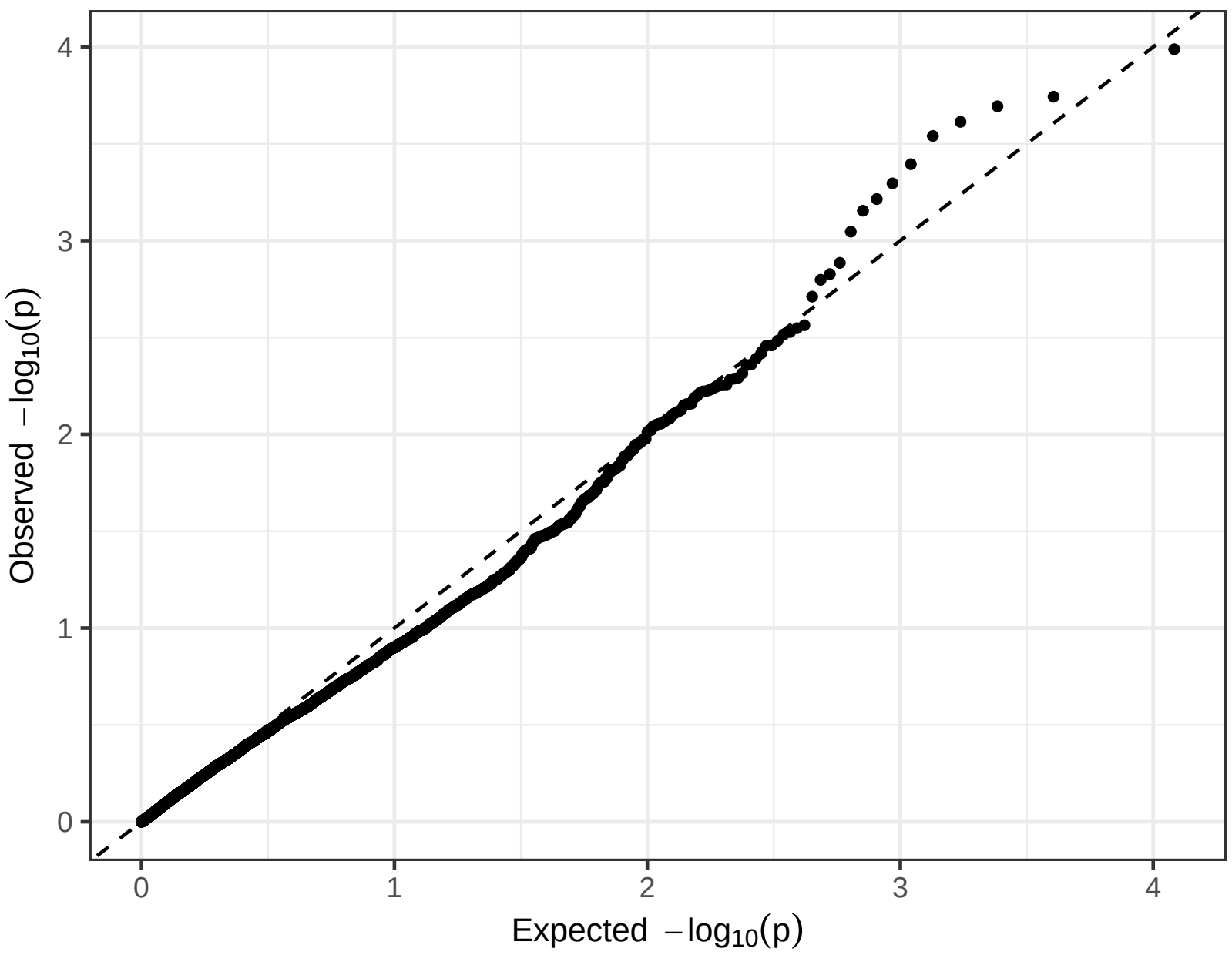

Intestinimonas

Feces | Brain | . = 0.884

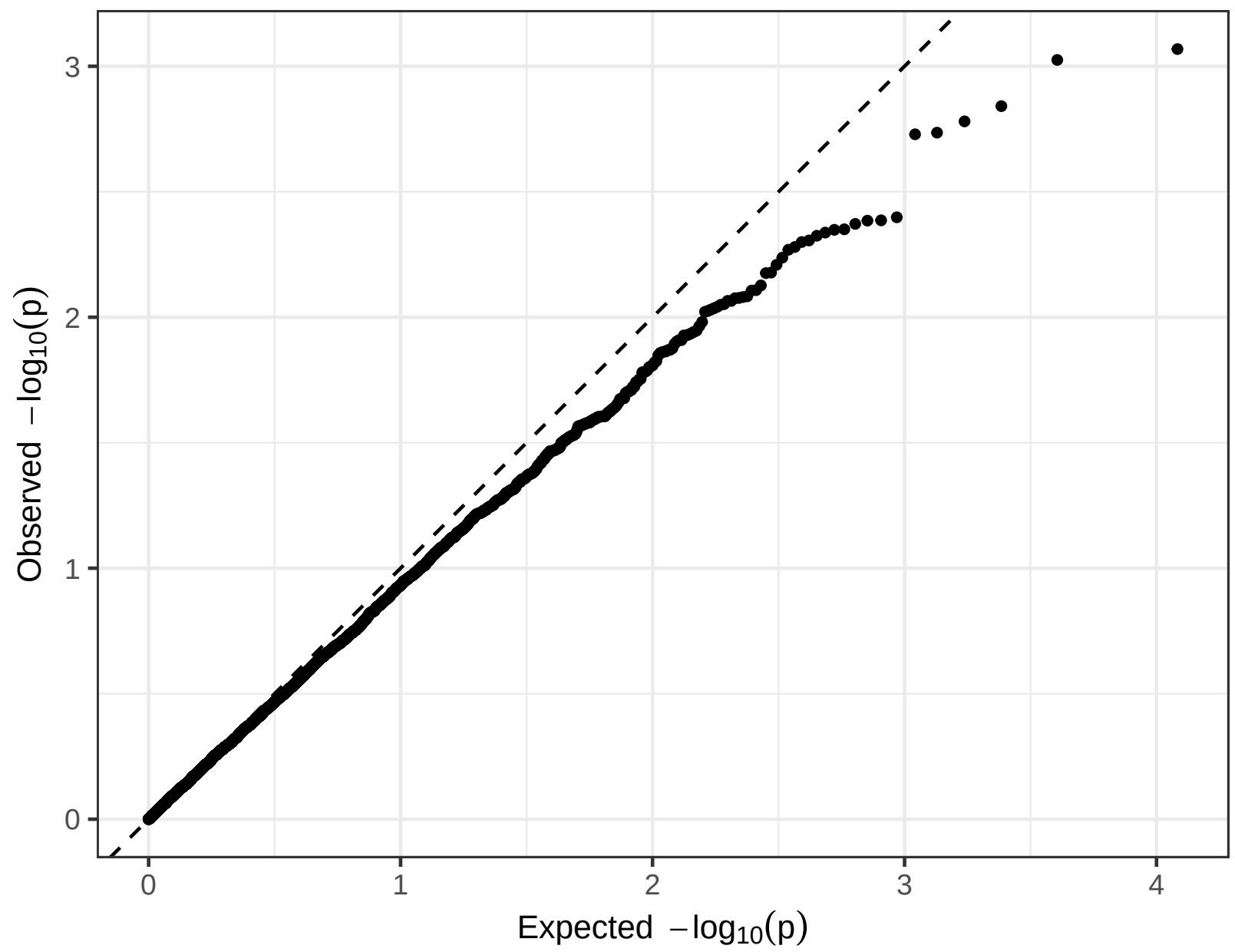

Lachnoclostridium

Feces | Brain | . = 0.957

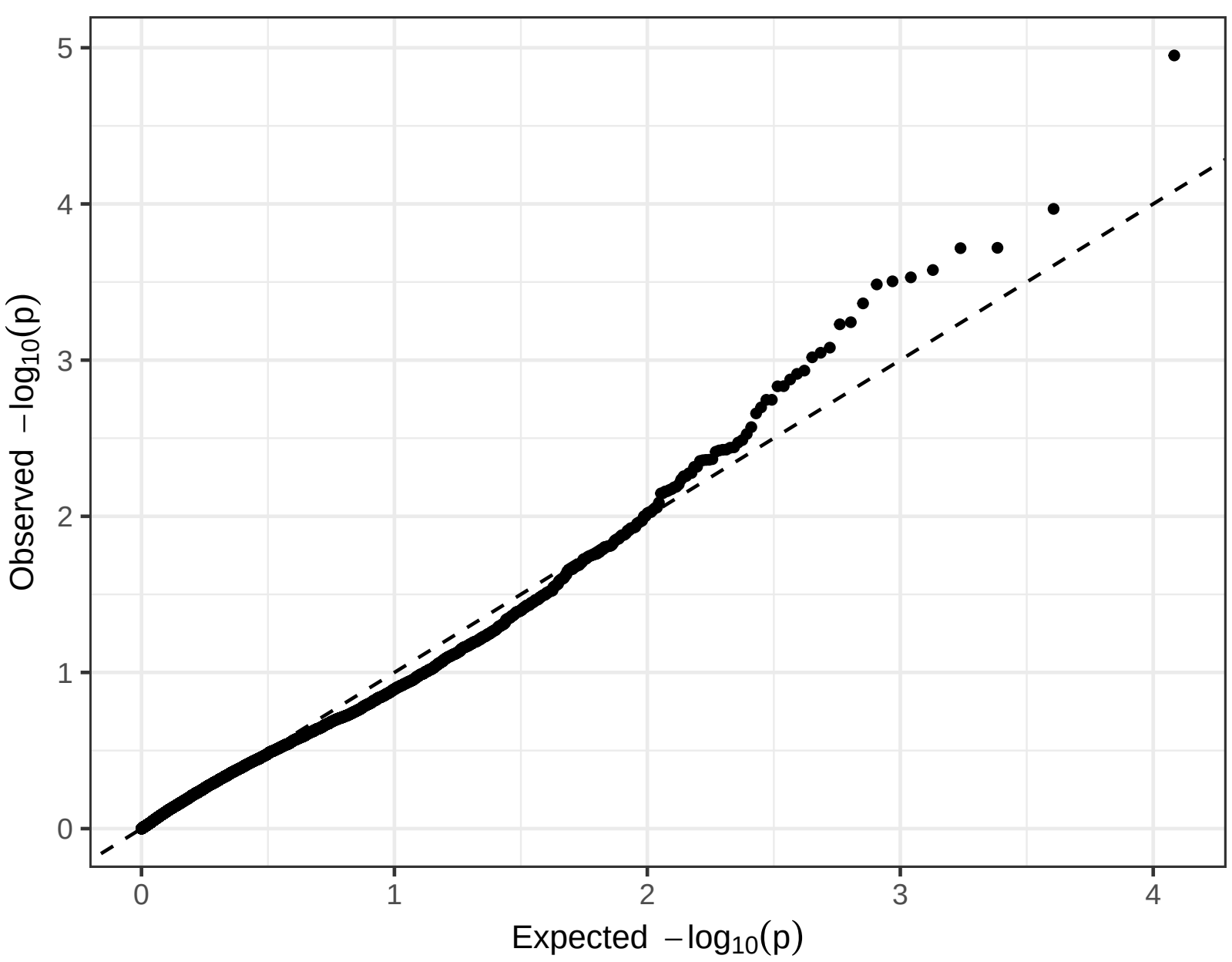

Lachnospira

Feces | Brain | . = 0.915

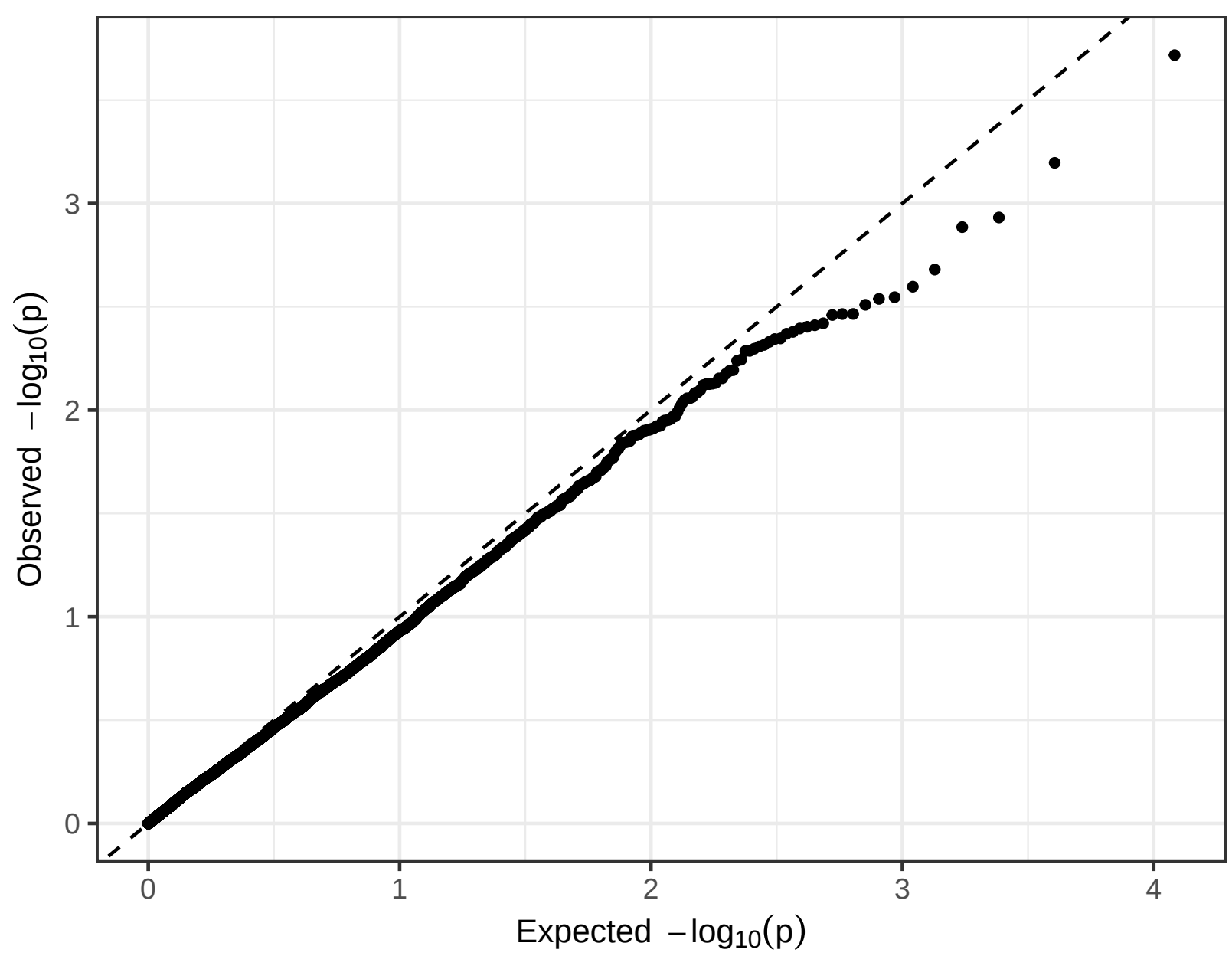

Lachnospiraceae_AC2044_group

Feces | Brain | . = 0.953

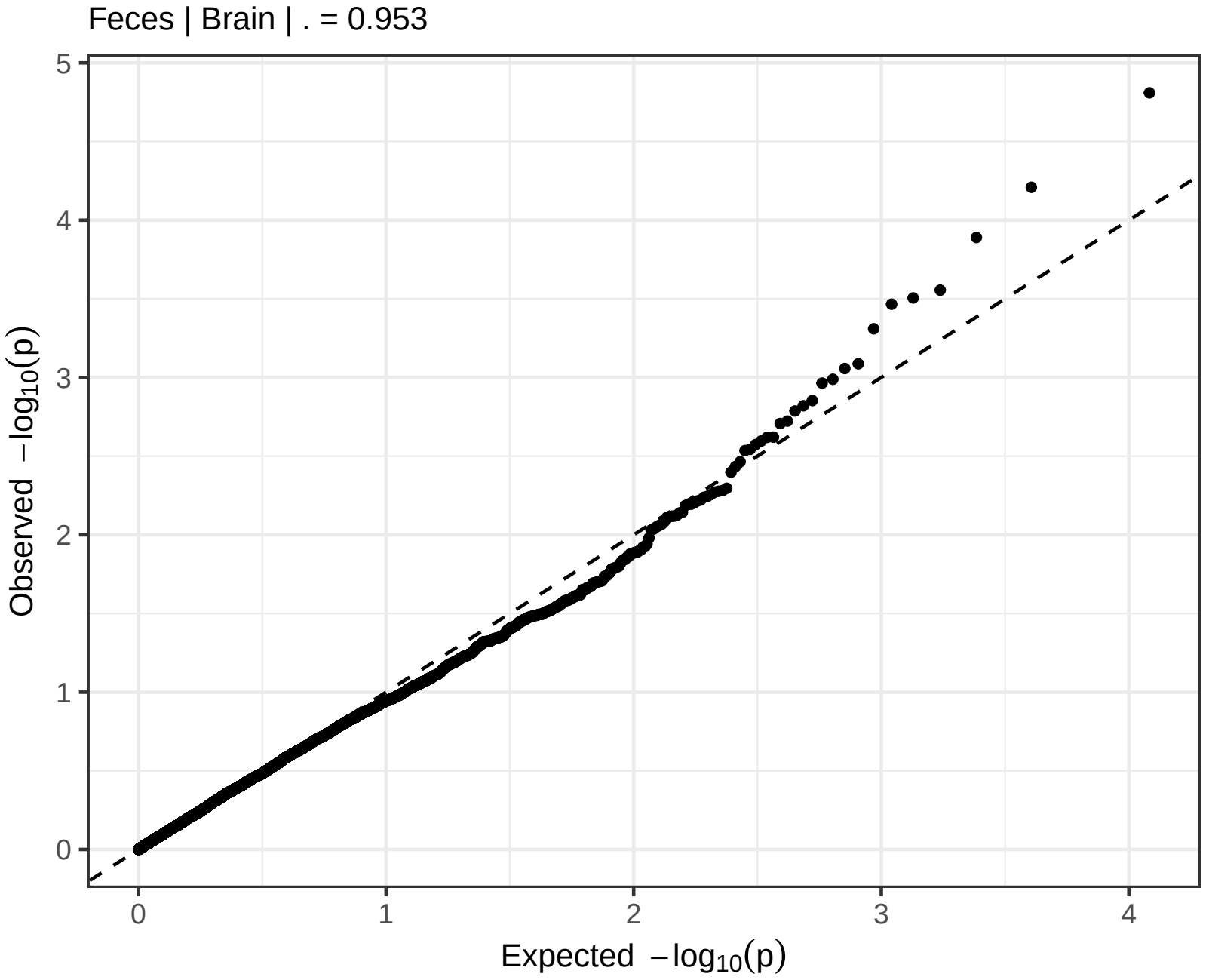

Lachnospiraceae_FCS020_group

Feces | Brain | . = 0.949

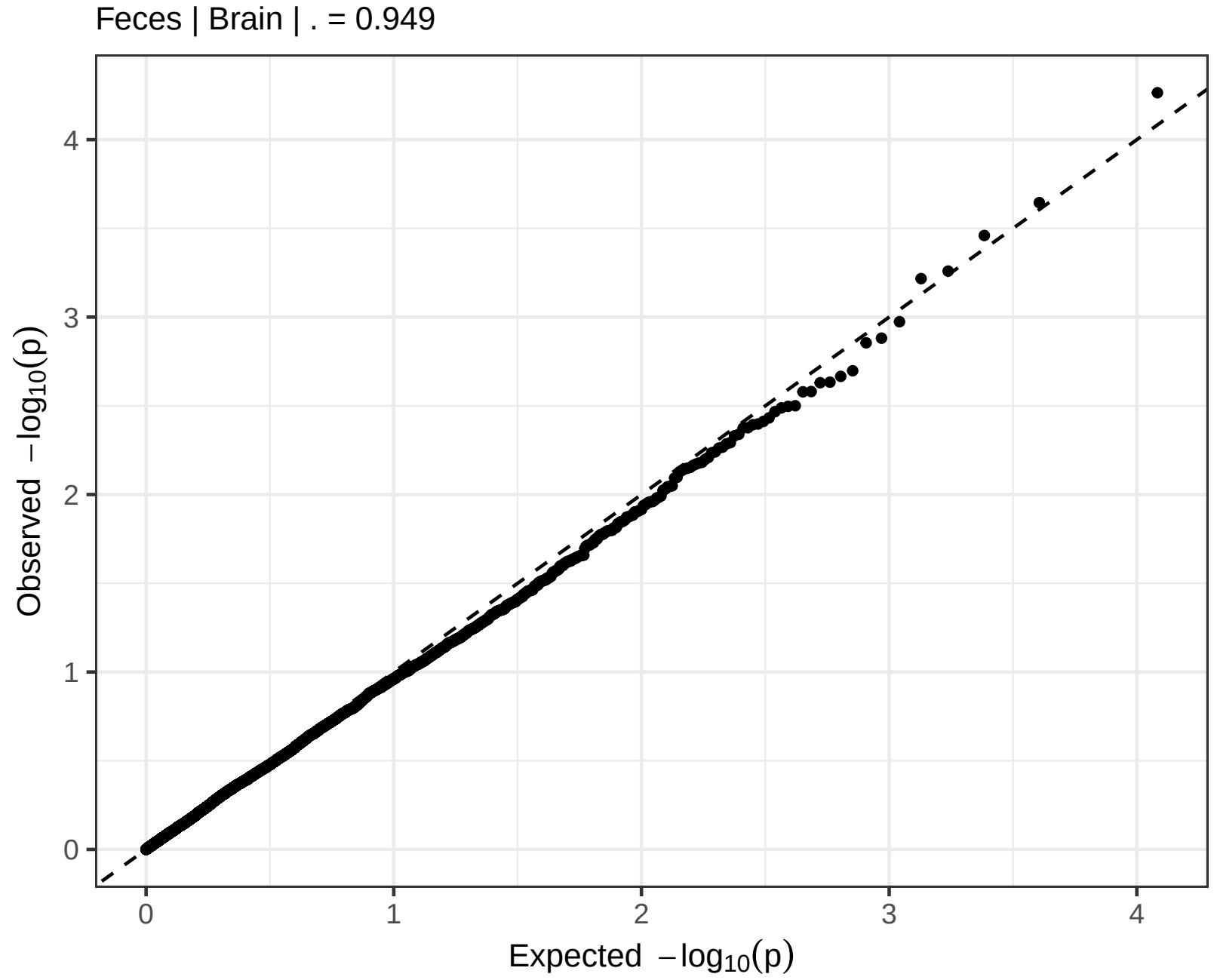

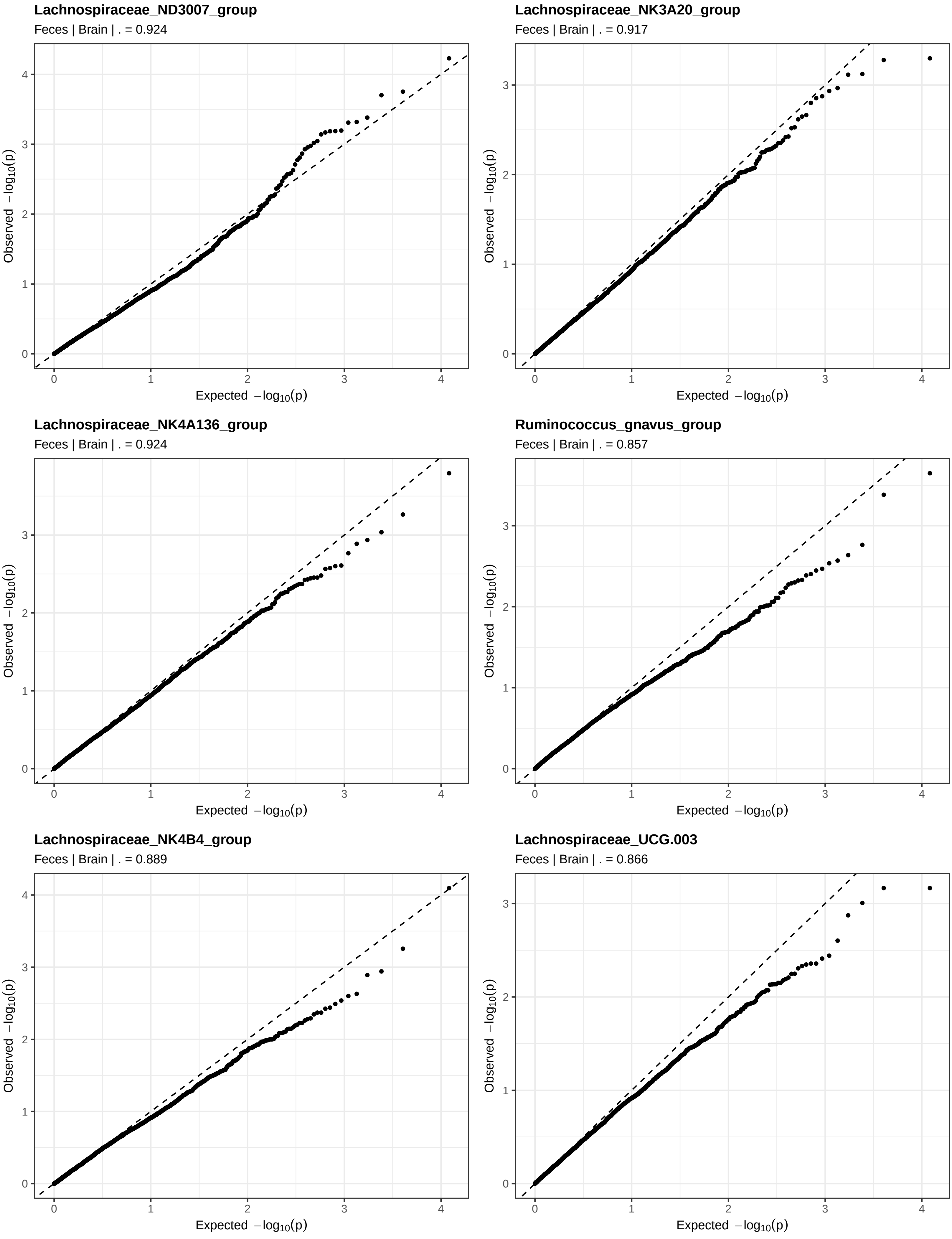

Lachnospiraceae_ND3007_group
Feces | Brain | . = 0.924
Lachnospiraceae_NK3A20_group
Feces | Brain | . = 0.917
Lachnospiraceae_NK4A136_group
Feces | Brain | . = 0.924
Ruminococcus_gnavus_group
Feces | Brain | . = 0.857
Lachnospiraceae_NK4B4_group
Feces | Brain | . = 0.889
Lachnospiraceae_UCG.003
Feces | Brain | . = 0.866
Observed $-\log_{10}(p)$
Expected $-\log_{10}(p)$

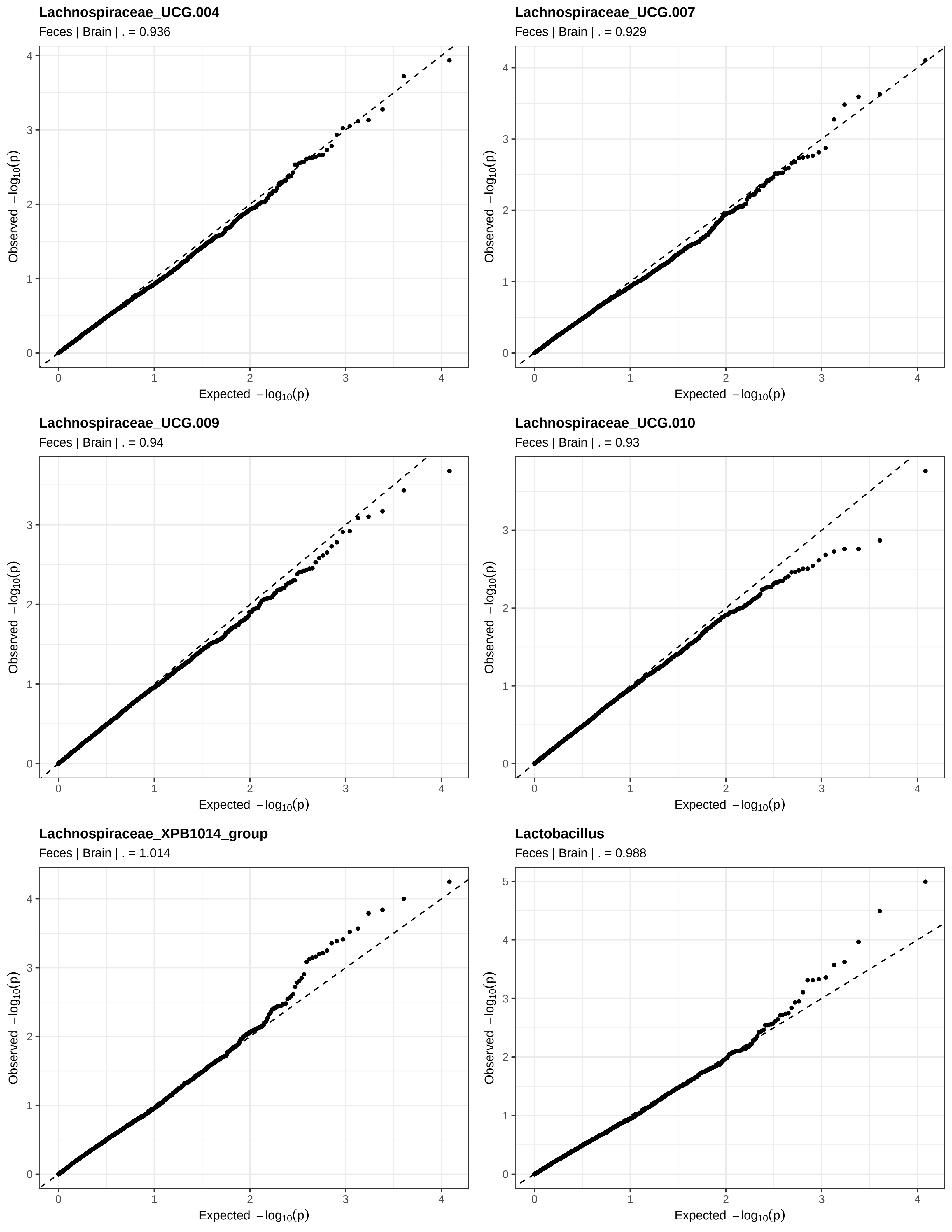

Lachnospiraceae_UCG.004
Feces | Brain | . = 0.936
Lachnospiraceae_UCG.007
Feces | Brain | . = 0.929
Lachnospiraceae_UCG.009
Feces | Brain | . = 0.94
Lachnospiraceae_UCG.010
Feces | Brain | . = 0.93
Lachnospiraceae_XPB1014_group
Feces | Brain | . = 1.014
Lactobacillus
Feces | Brain | . = 0.988
Observed $-\log_{10}(p)$
Expected $-\log_{10}(p)$

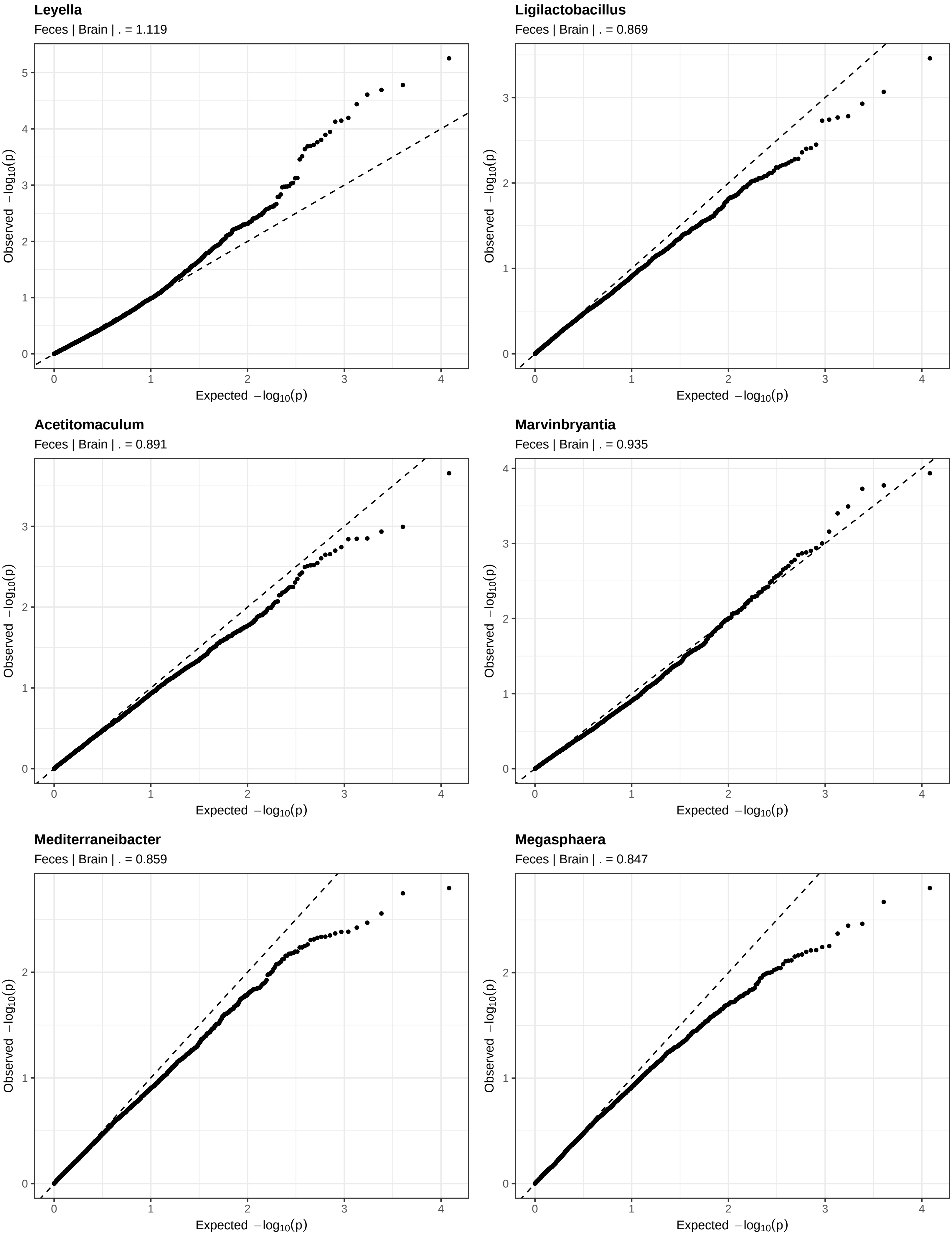
Leyella
Feces | Brain | . = 1.119
Ligilactobacillus
Feces | Brain | . = 0.869
Acetitomaculum
Feces | Brain | . = 0.891
Marvinbryantia
Feces | Brain | . = 0.935
Mediterraneibacter
Feces | Brain | . = 0.859
Megasphaera
Feces | Brain | . = 0.847
Observed $-\log_{10}(p)$
Expected $-\log_{10}(p)$

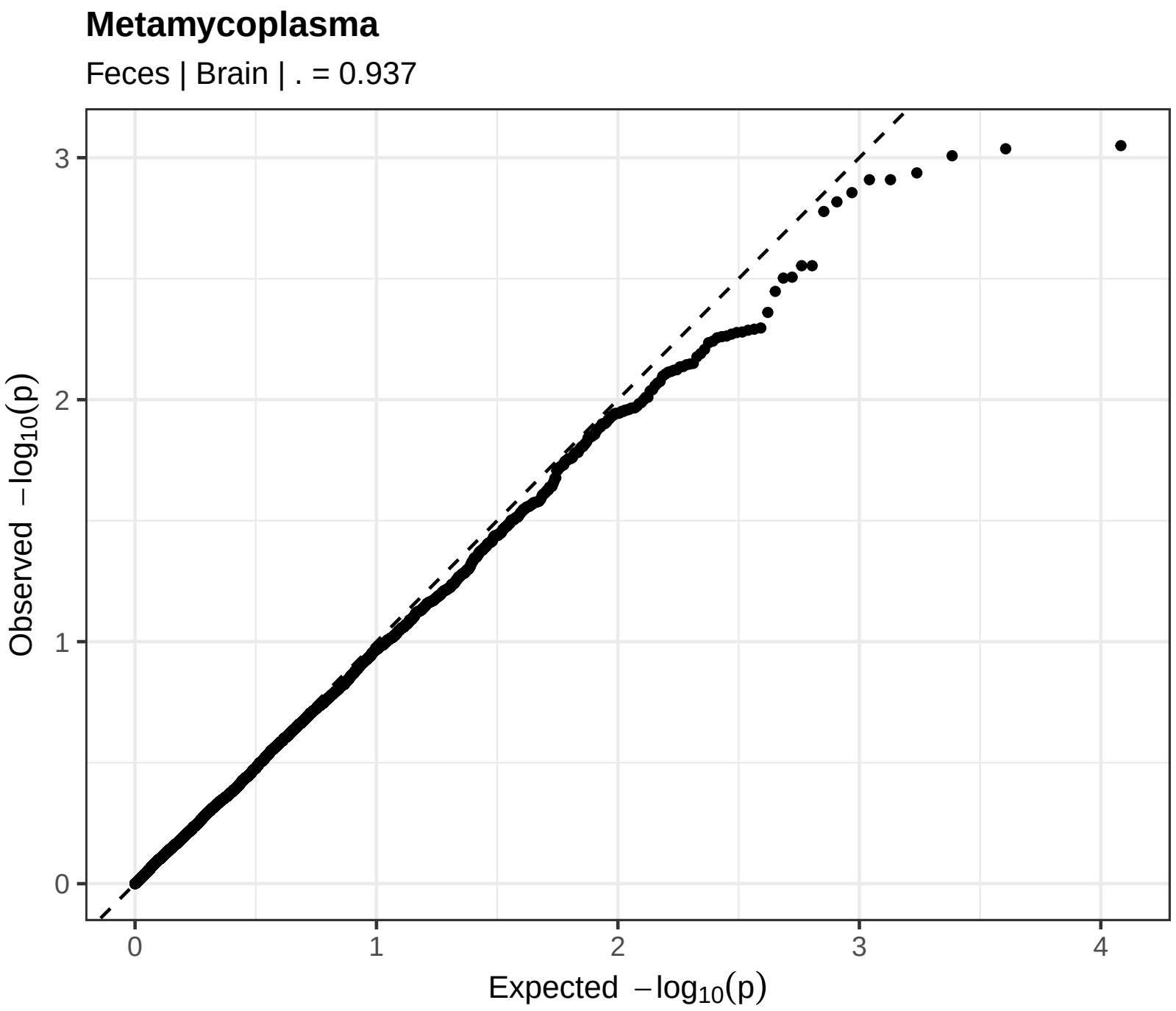
Metamycoplasma
Feces | Brain | . = 0.937
Observed −log10(p)
Expected −log10(p)

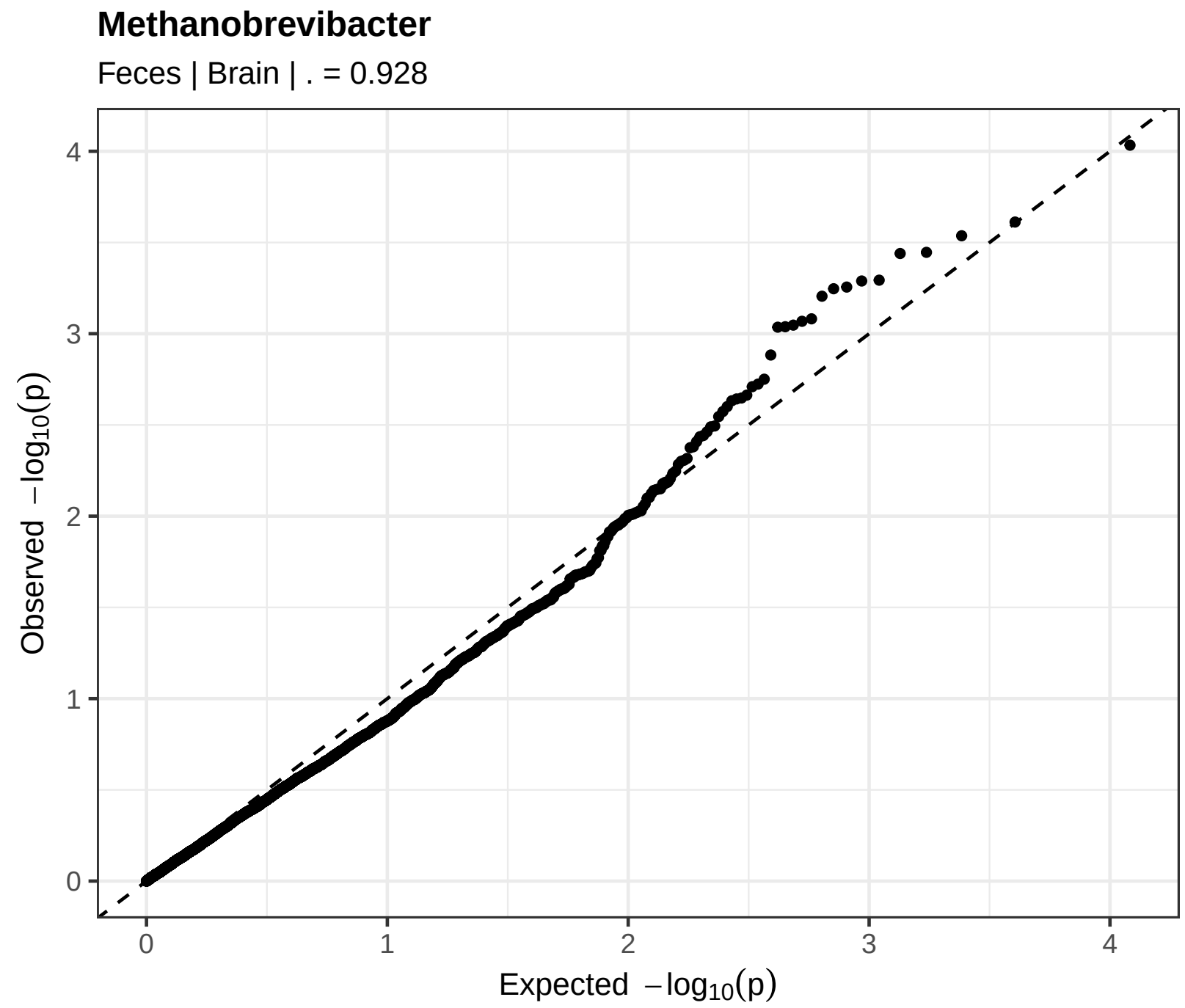
Methanobrevibacter
Feces | Brain | . = 0.928
Observed −log10(p)
Expected −log10(p)

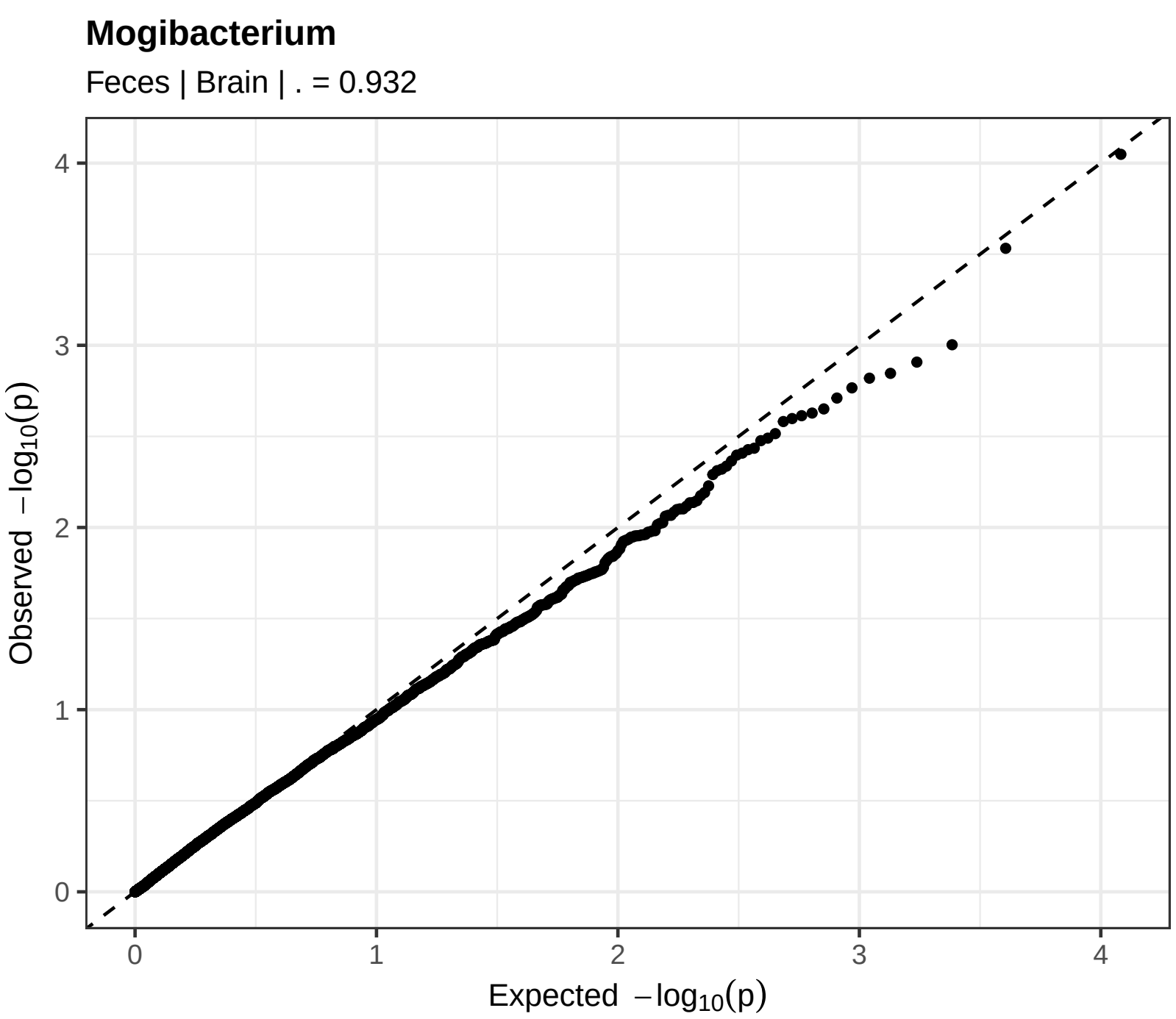
Mogibacterium
Feces | Brain | . = 0.932
Observed −log10(p)
Expected −log10(p)

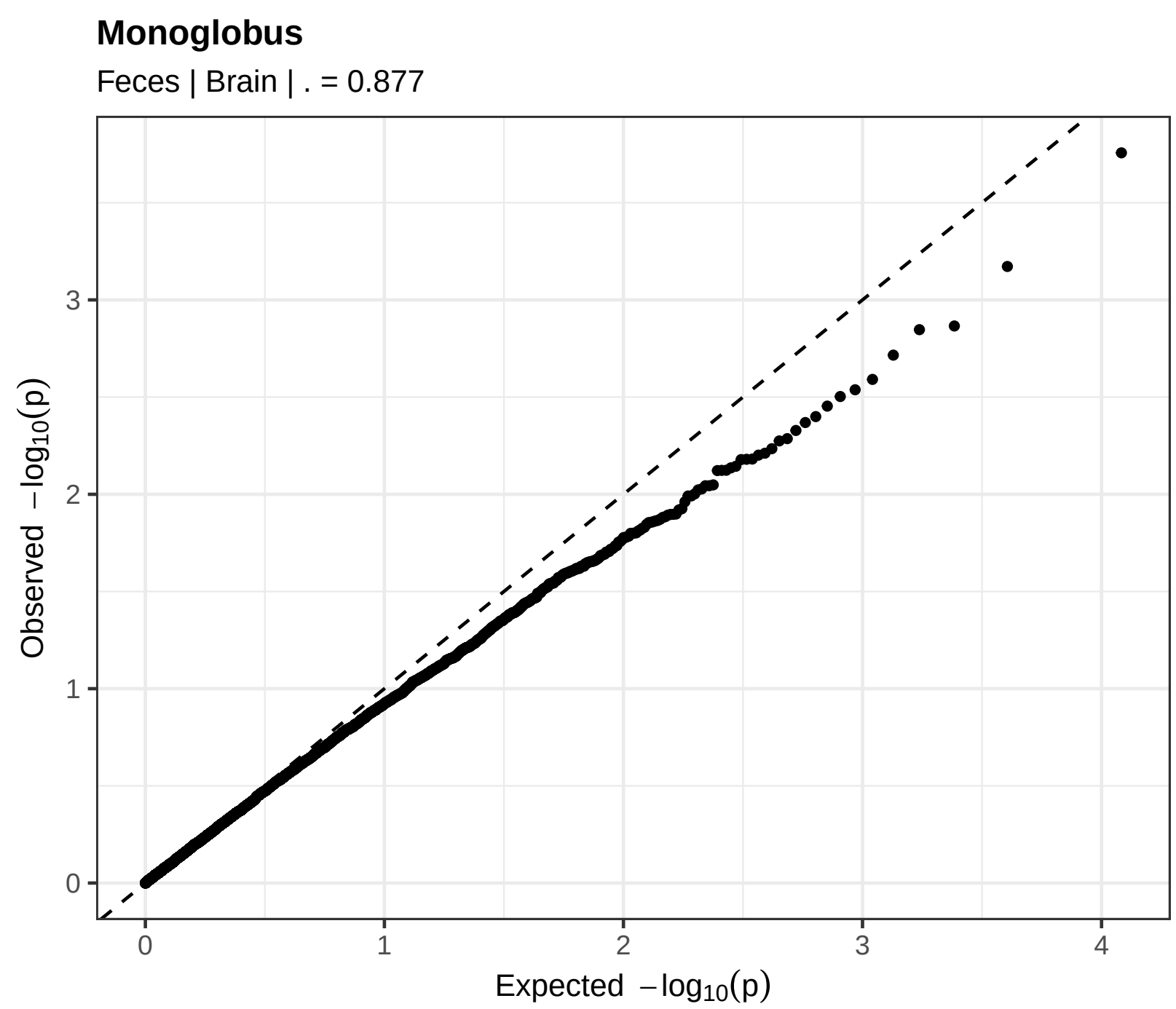
Monoglobus
Feces | Brain | . = 0.877
Observed −log10(p)
Expected −log10(p)

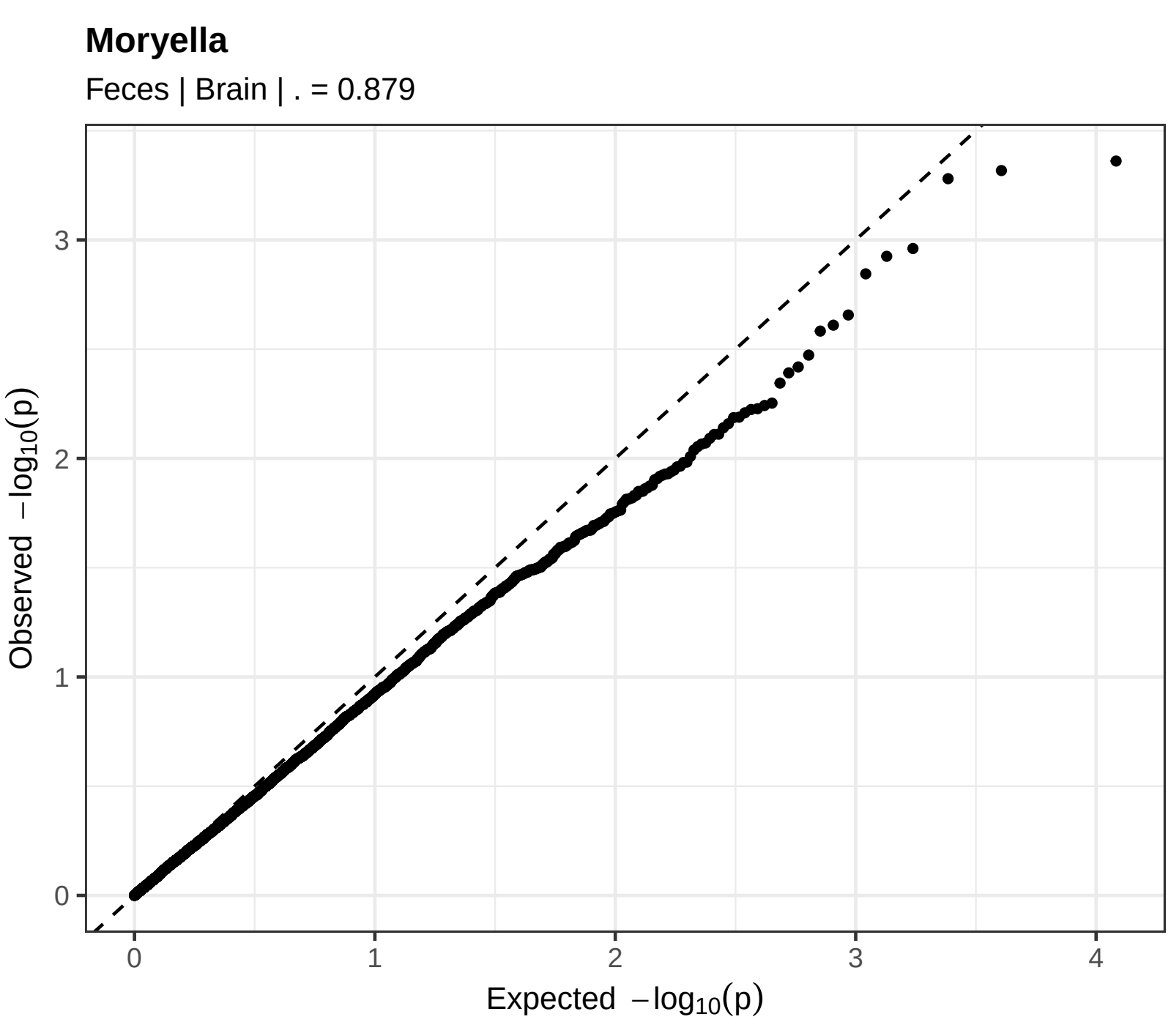
Moryella
Feces | Brain | . = 0.879
Observed −log10(p)
Expected −log10(p)

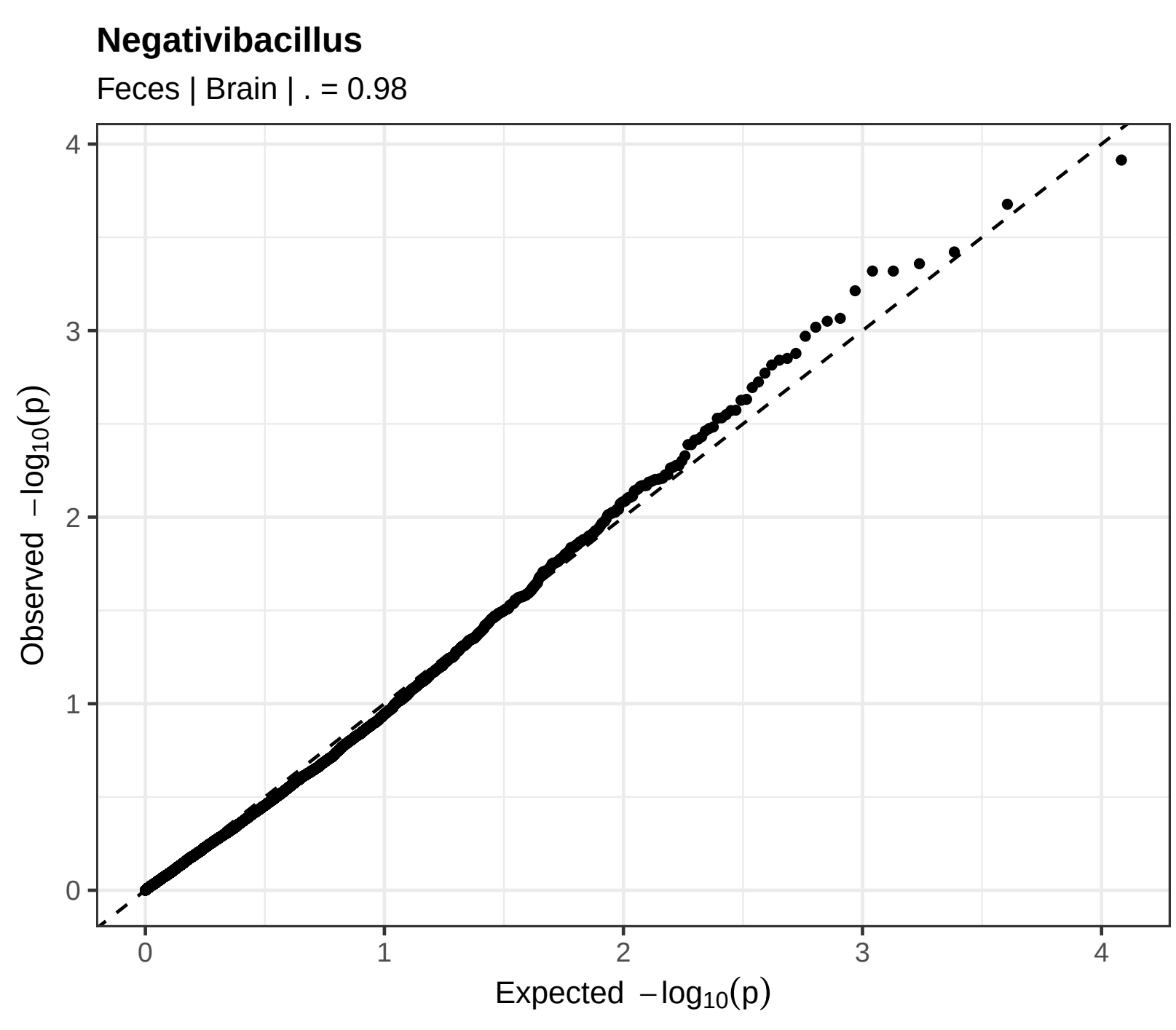
Negativibacillus
Feces | Brain | . = 0.98
Observed −log10(p)
Expected −log10(p)

NK4A214_group

Feces | Brain | . = 1.002

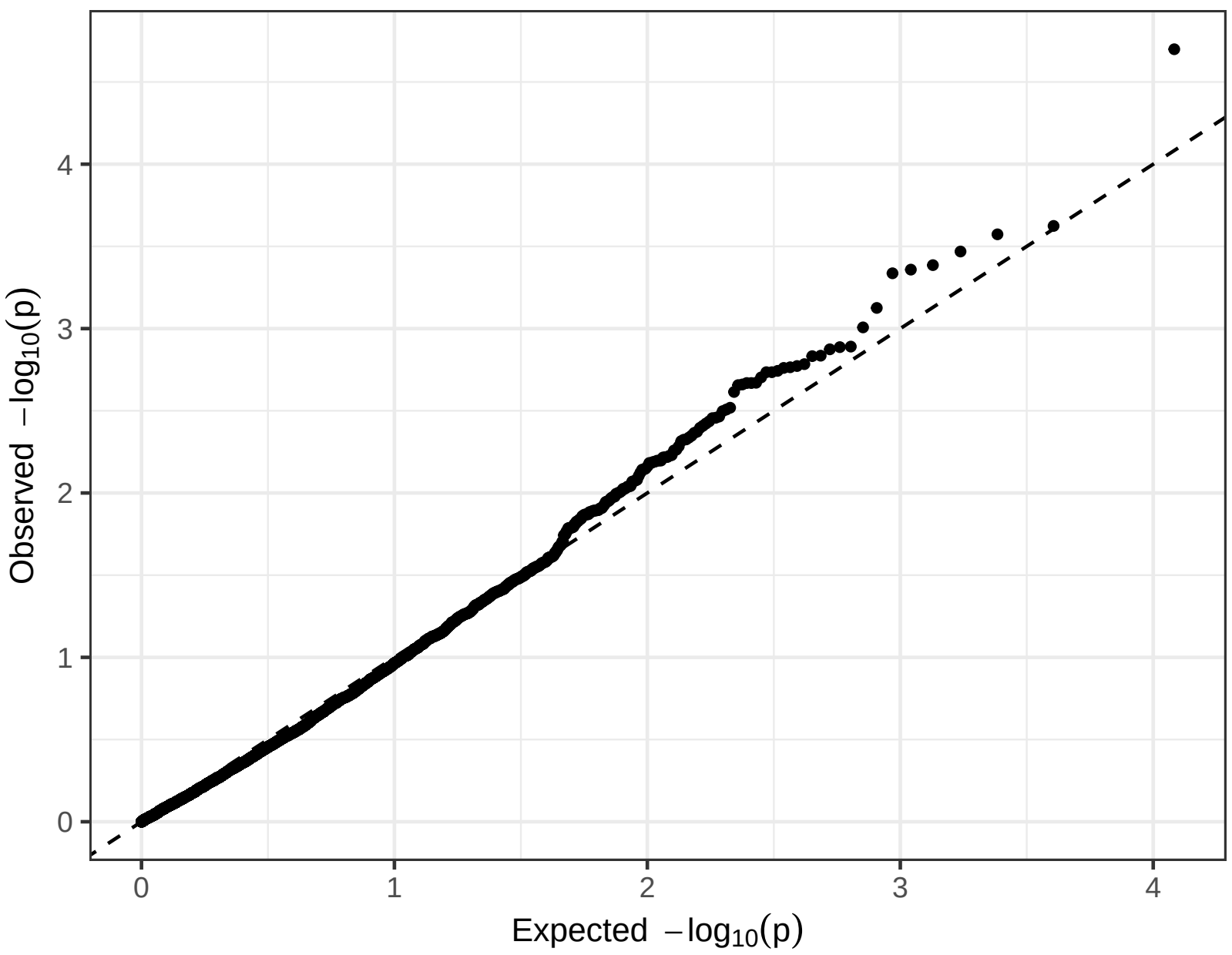

Acidaminococcus

Feces | Brain | . = 0.919

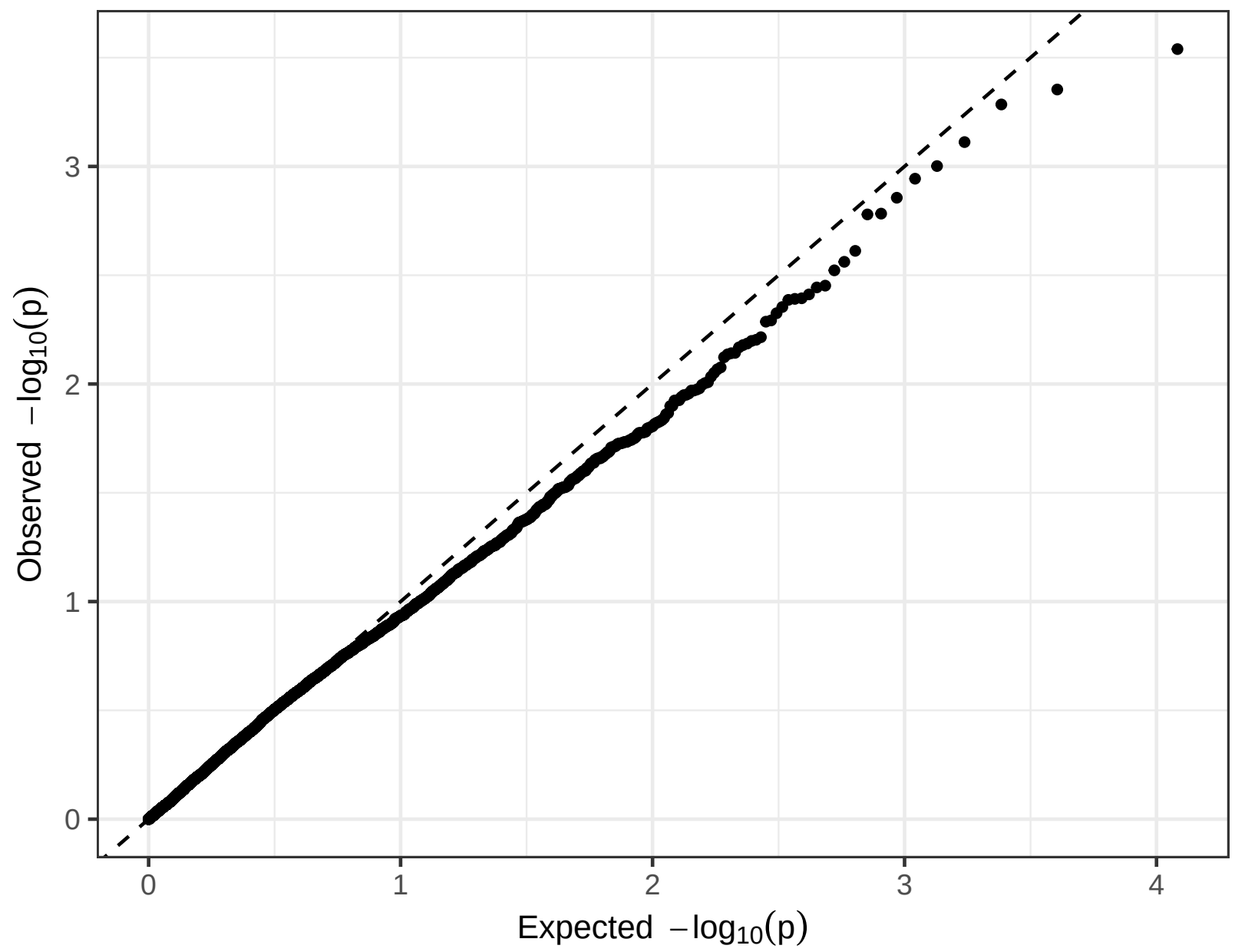

Oribacterium

Feces | Brain | . = 0.904

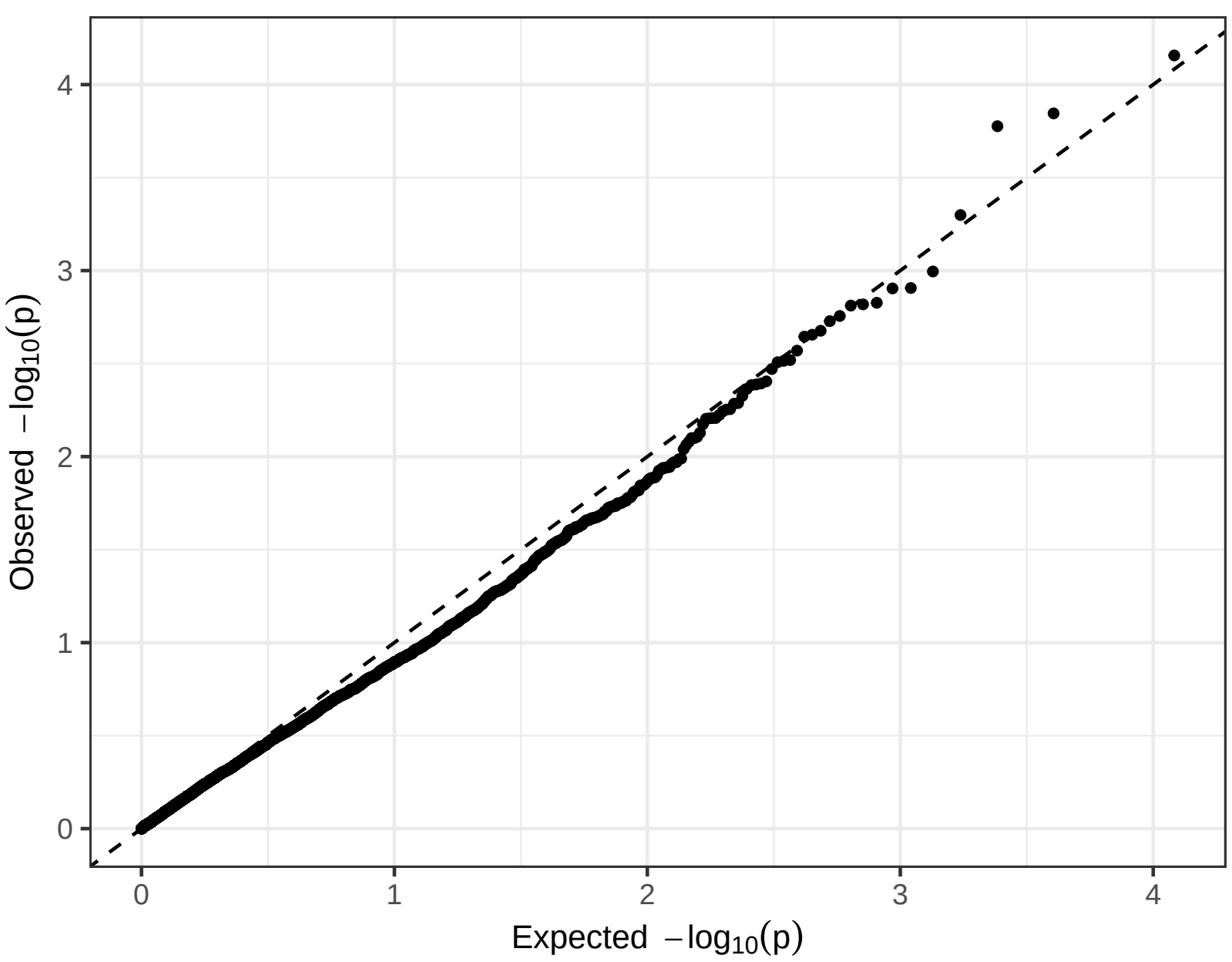

Oscillibacter

Feces | Brain | . = 1.041

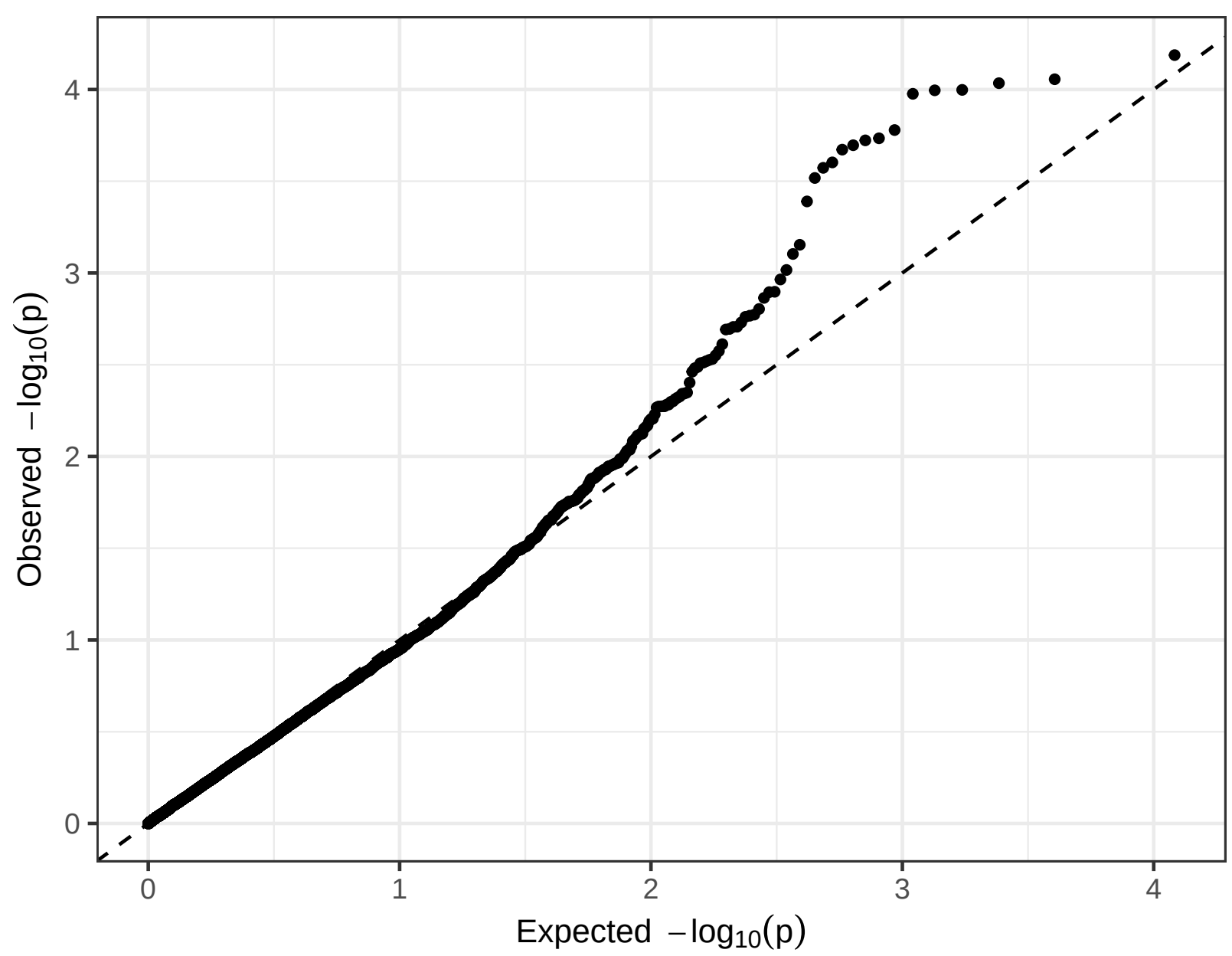

Oscillospira

Feces | Brain | . = 1.15

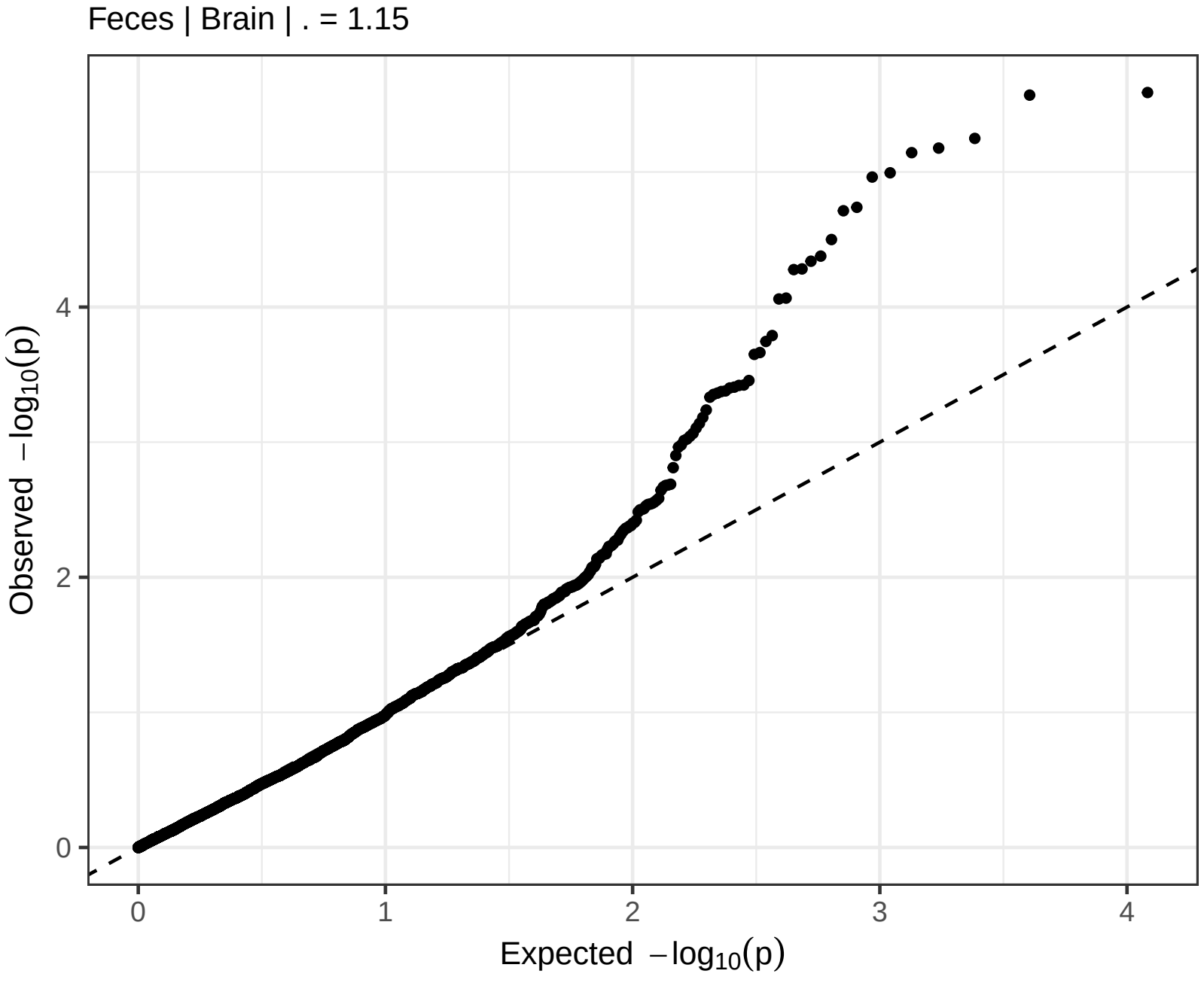

Oxalobacter

Feces | Brain | . = 0.884

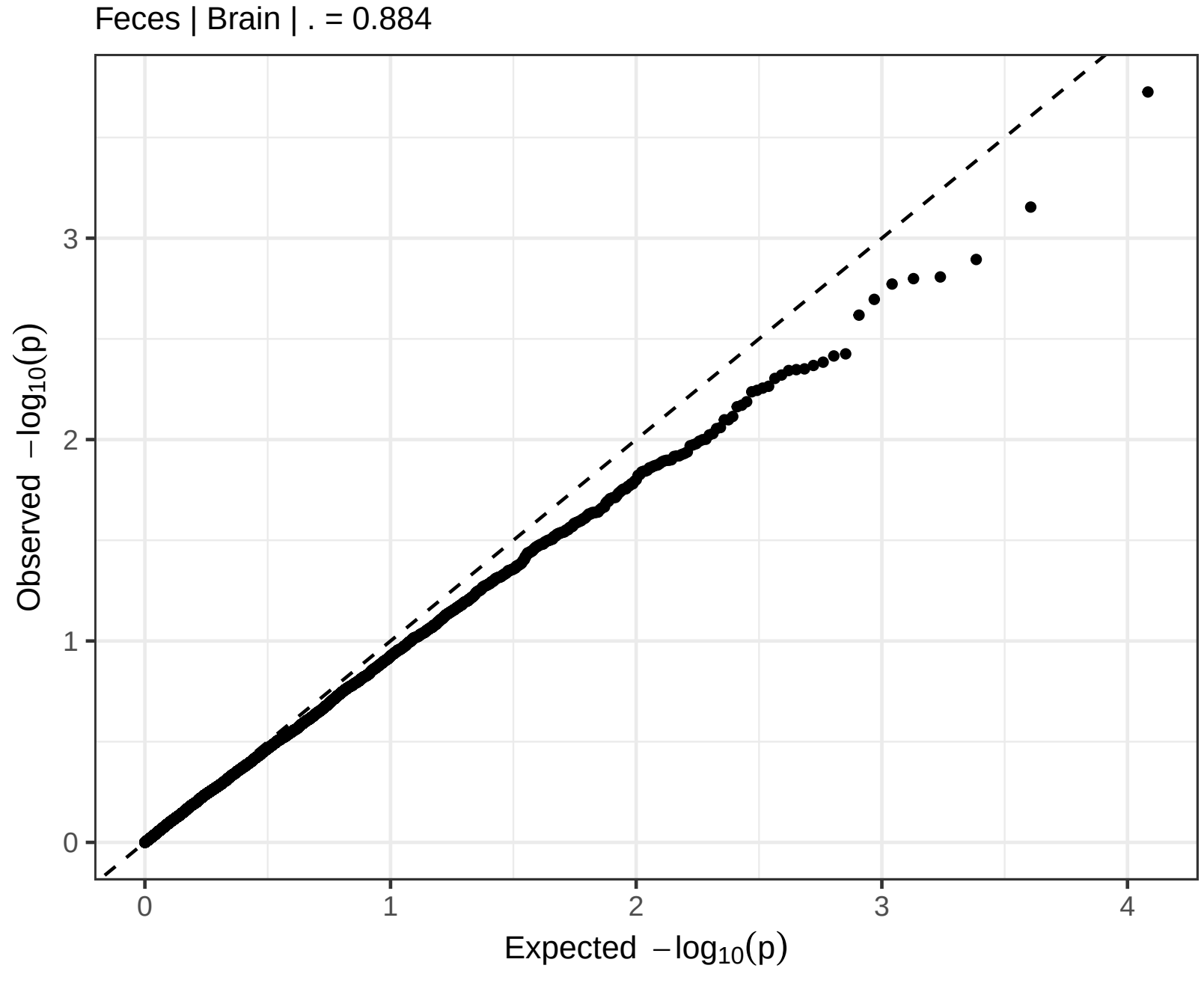

p.1088.a5_gut_group

Feces | Brain | . = 0.962

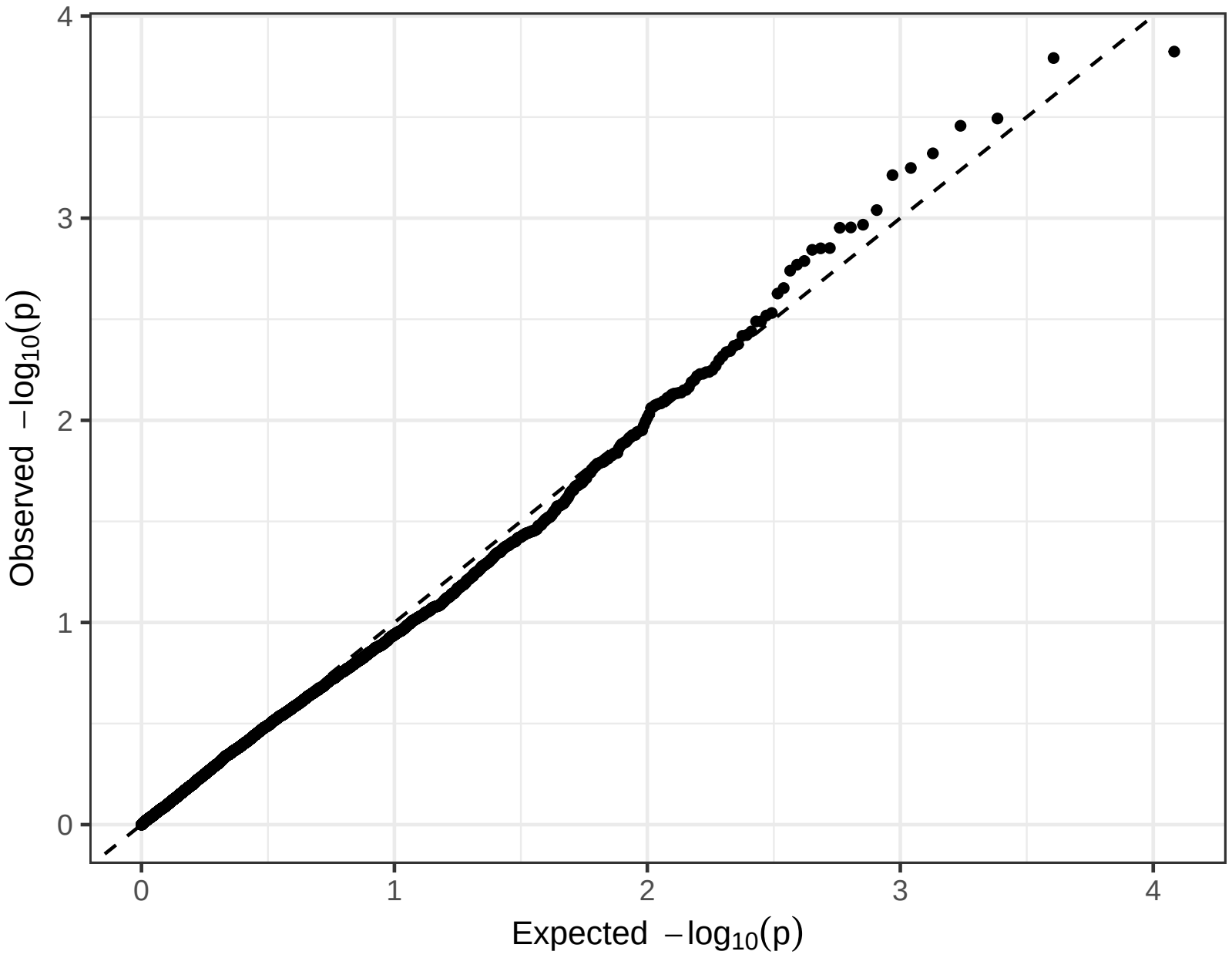

Paludicola

Feces | Brain | . = 0.907

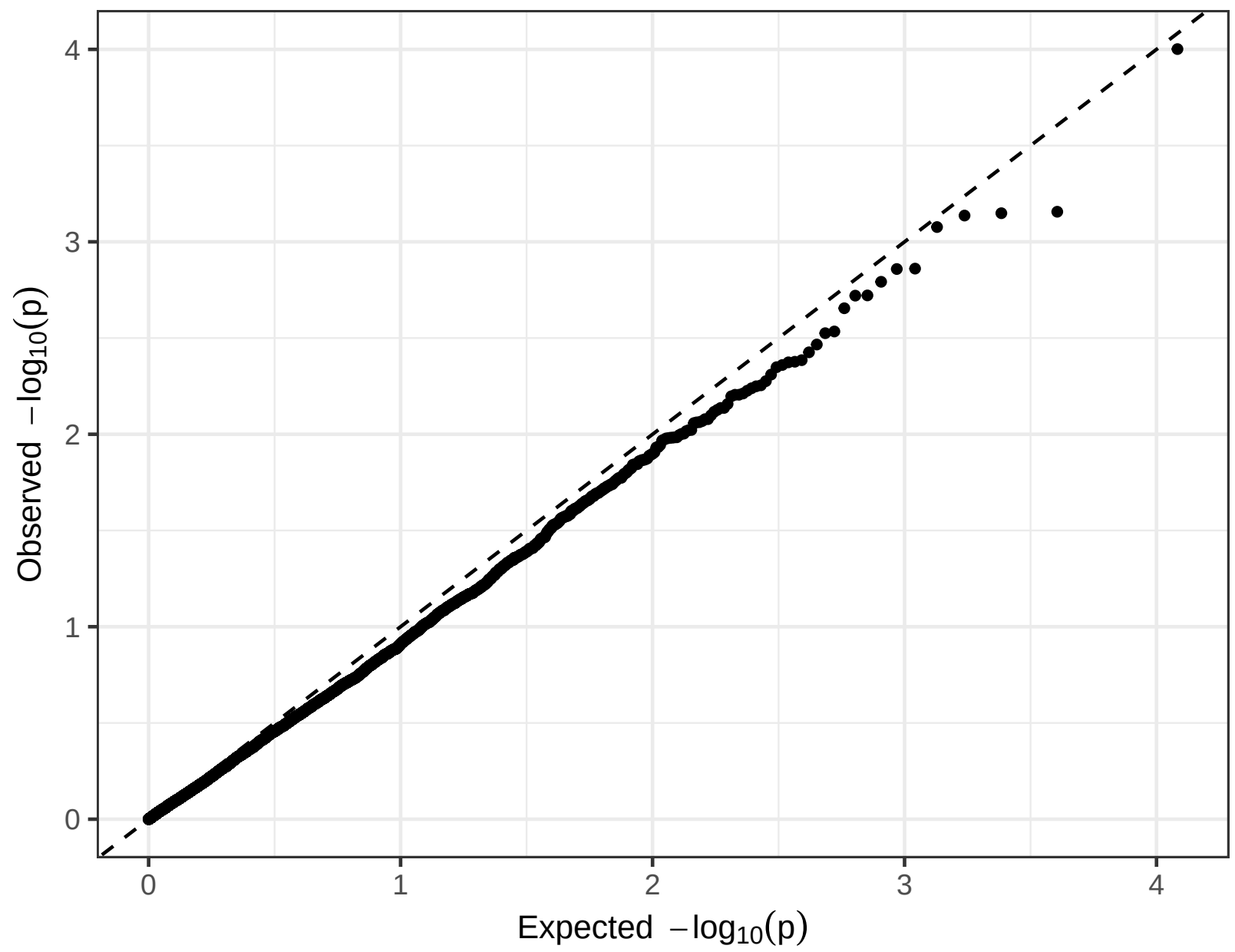

Parabacteroides

Feces | Brain | . = 0.942

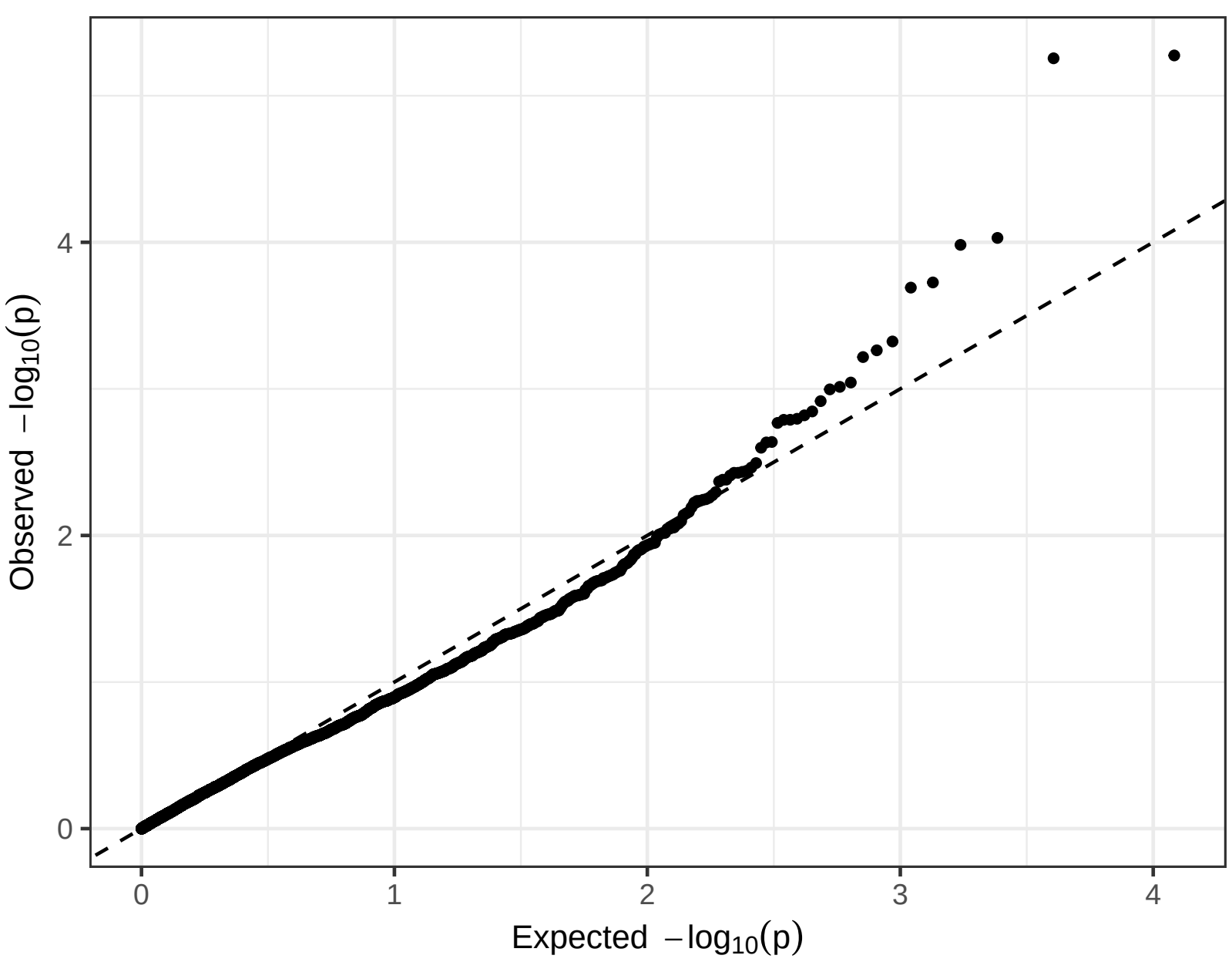

Parasutterella

Feces | Brain | . = 0.908

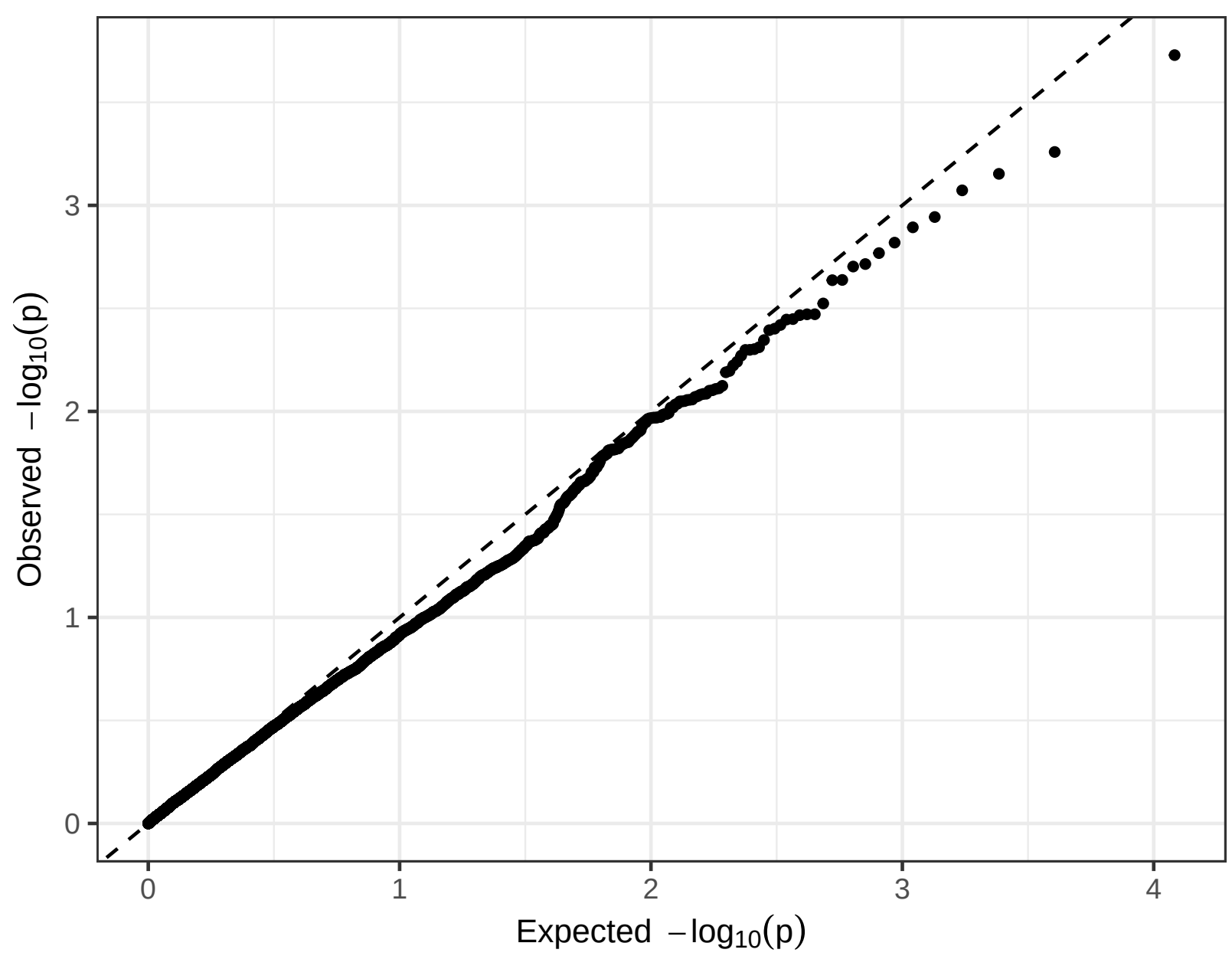

Peptococcus

Feces | Brain | . = 0.883

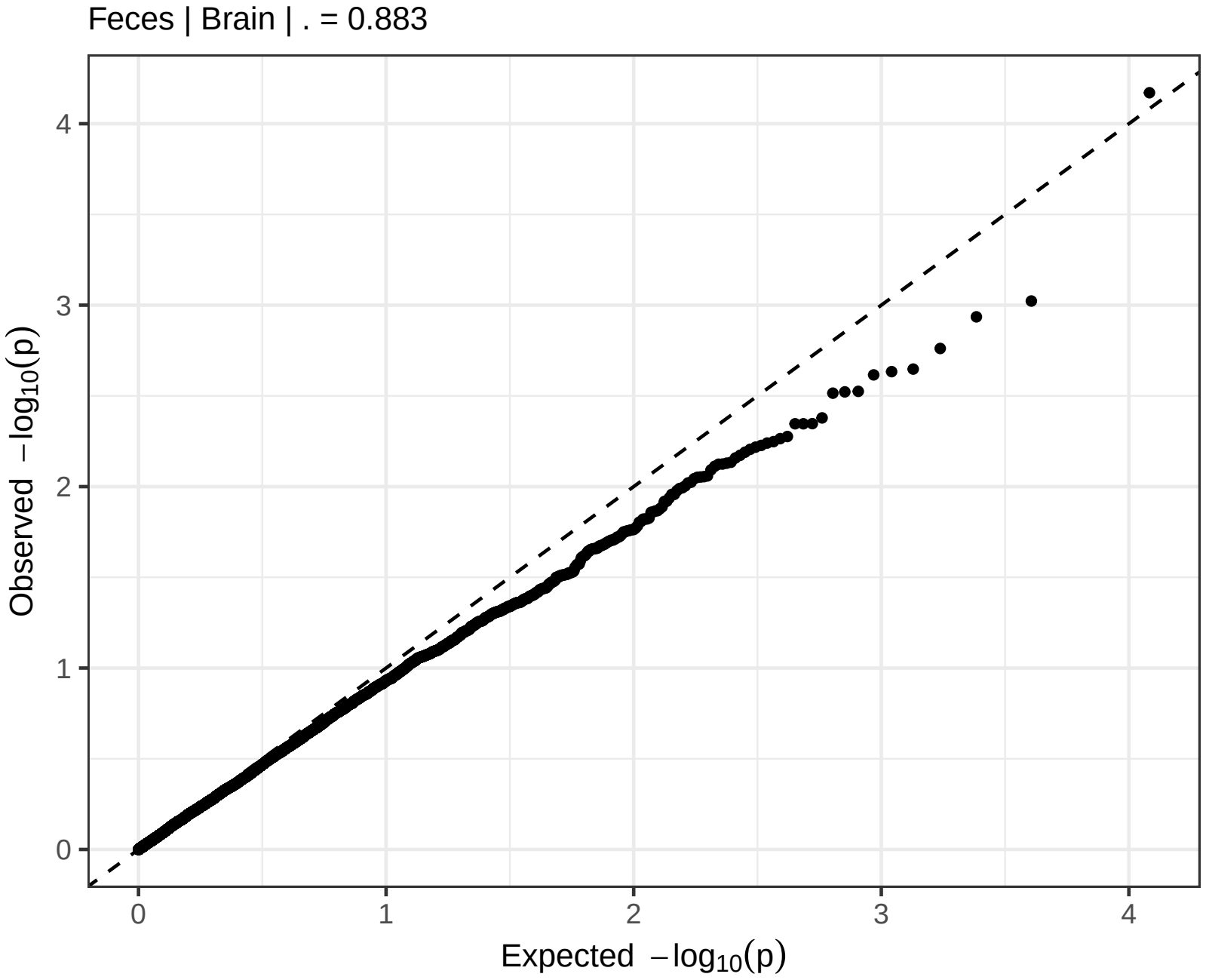

Phascolarctobacterium

Feces | Brain | . = 0.965

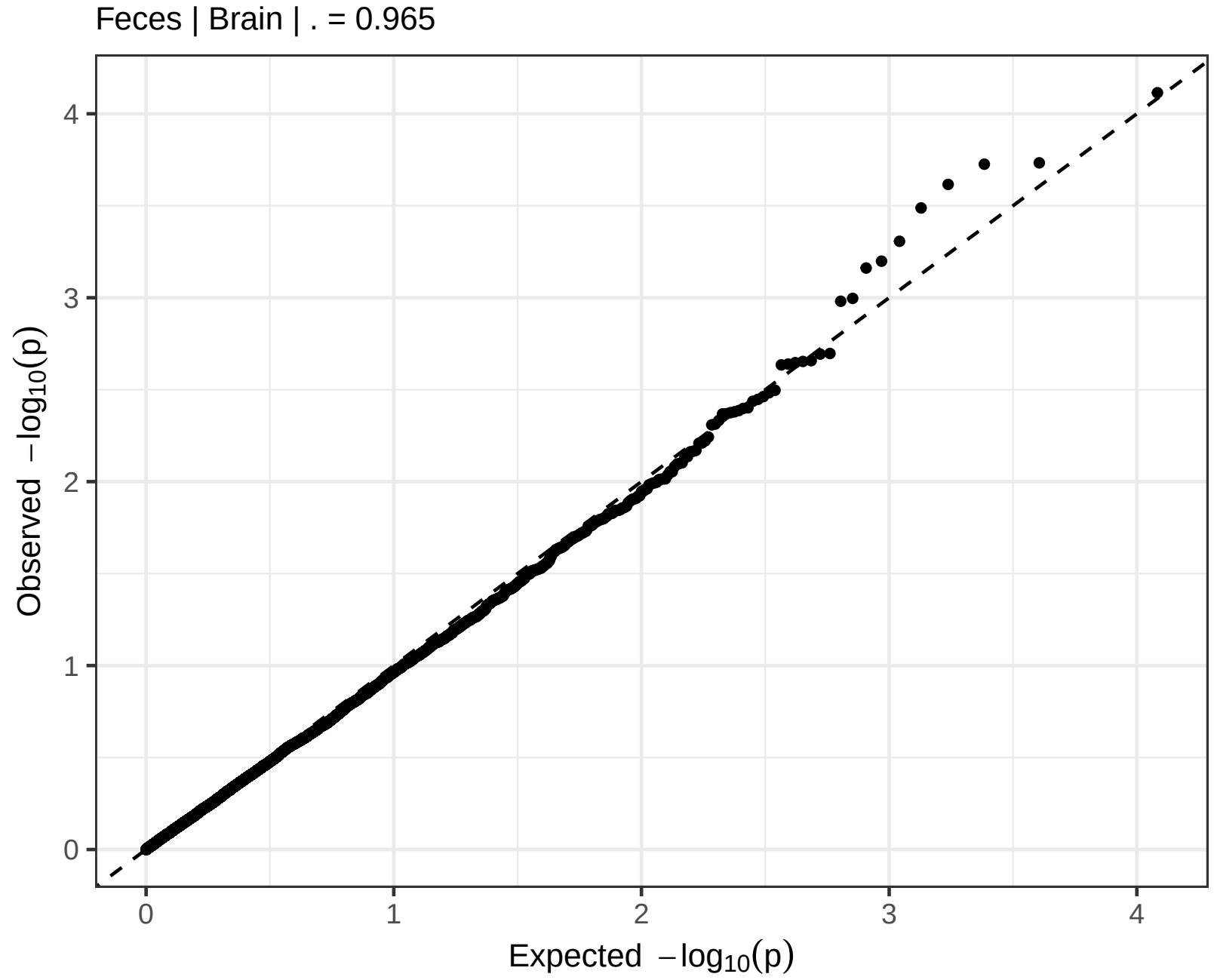

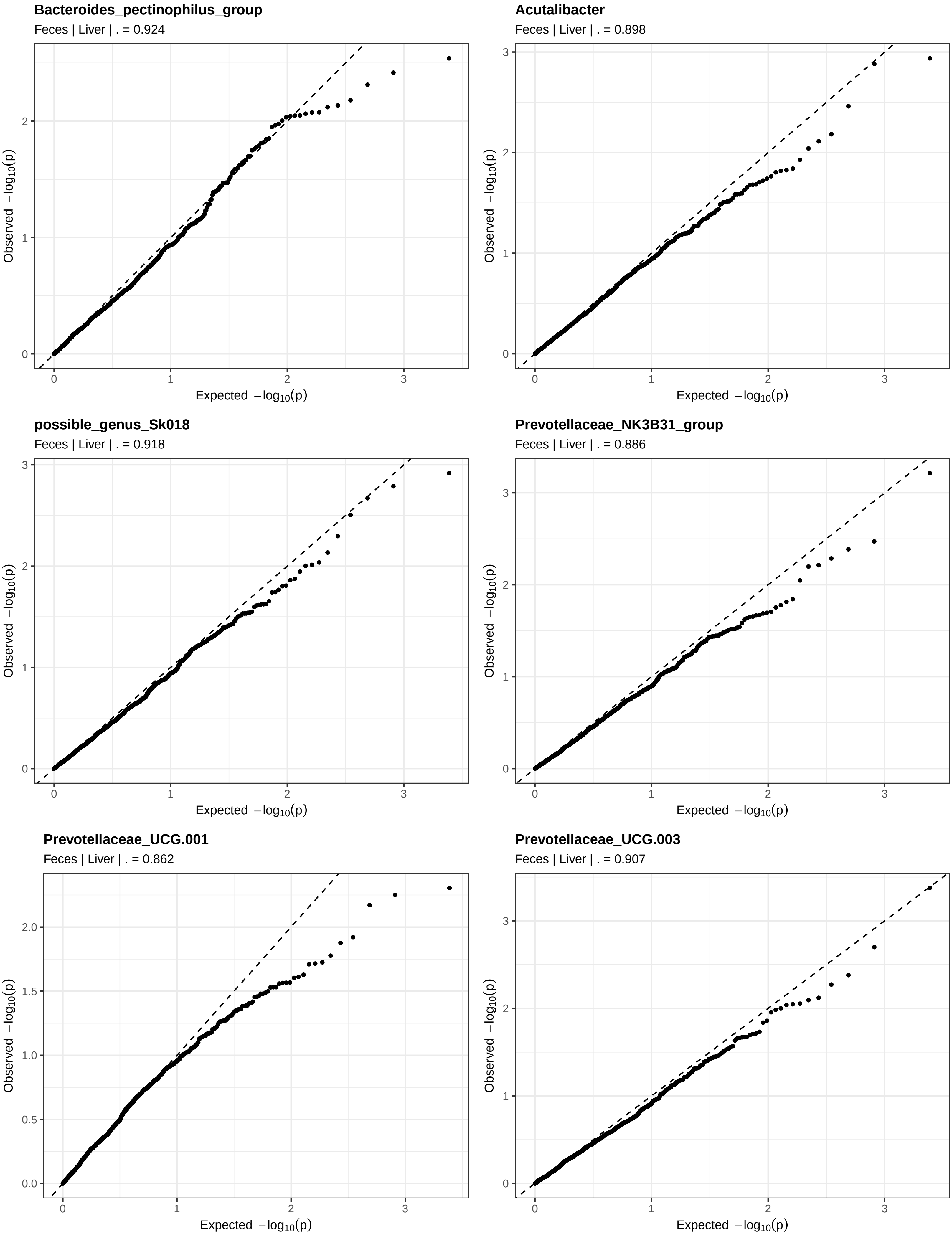

Bacteroides_pectinophilus_group
Feces | Liver | . = 0.924
Acutalibacter
Feces | Liver | . = 0.898
possible_genus_Sk018
Feces | Liver | . = 0.918
Prevotellaceae_NK3B31_group
Feces | Liver | . = 0.886
Prevotellaceae_UCG.001
Feces | Liver | . = 0.862
Prevotellaceae_UCG.003
Feces | Liver | . = 0.907
Observed $-\log_{10}(p)$
Expected $-\log_{10}(p)$

Prevotellaceae_UCG.004

Feces | Liver | . = 0.866

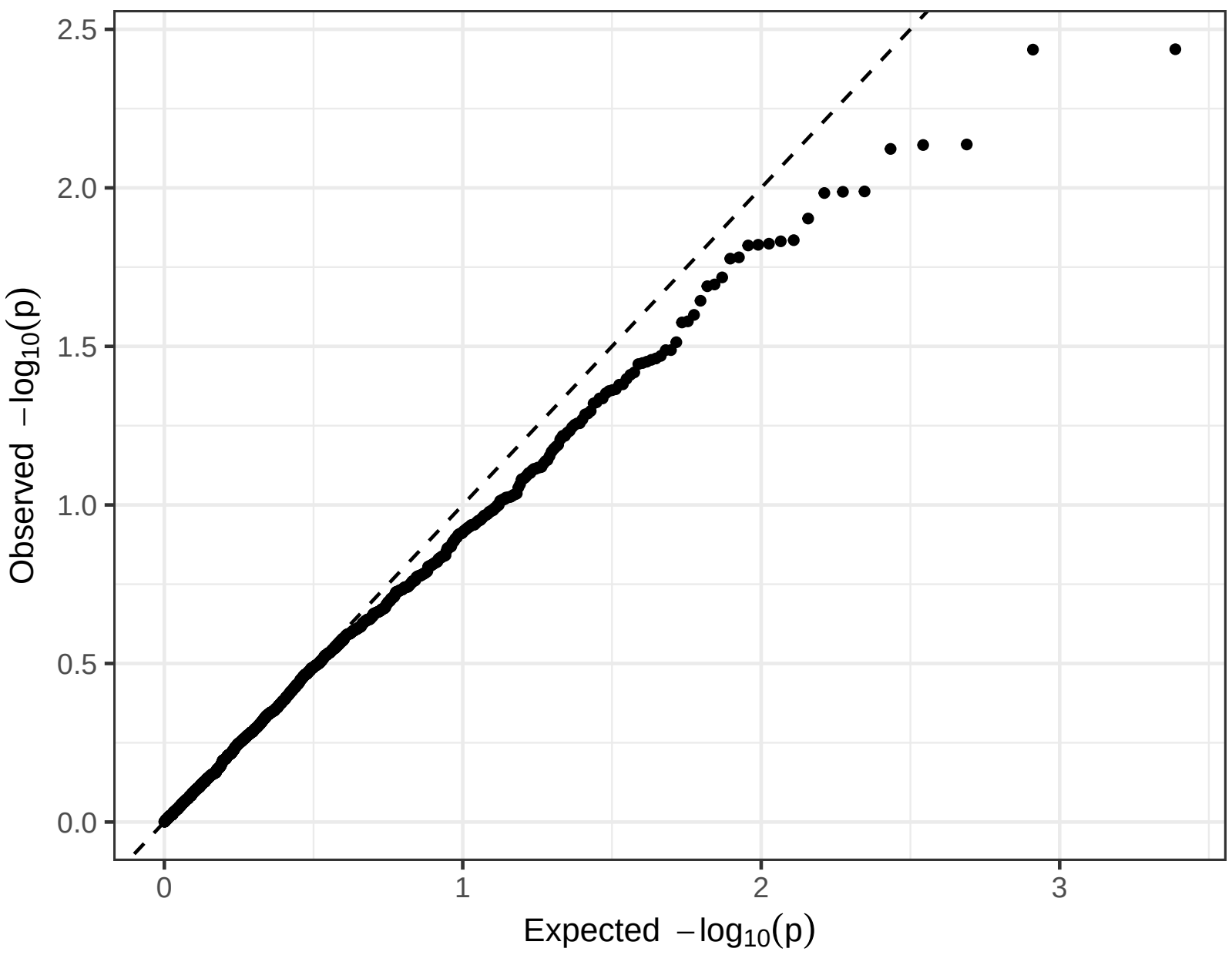

Pseudobutyrivibrio

Feces | Liver | . = 0.966

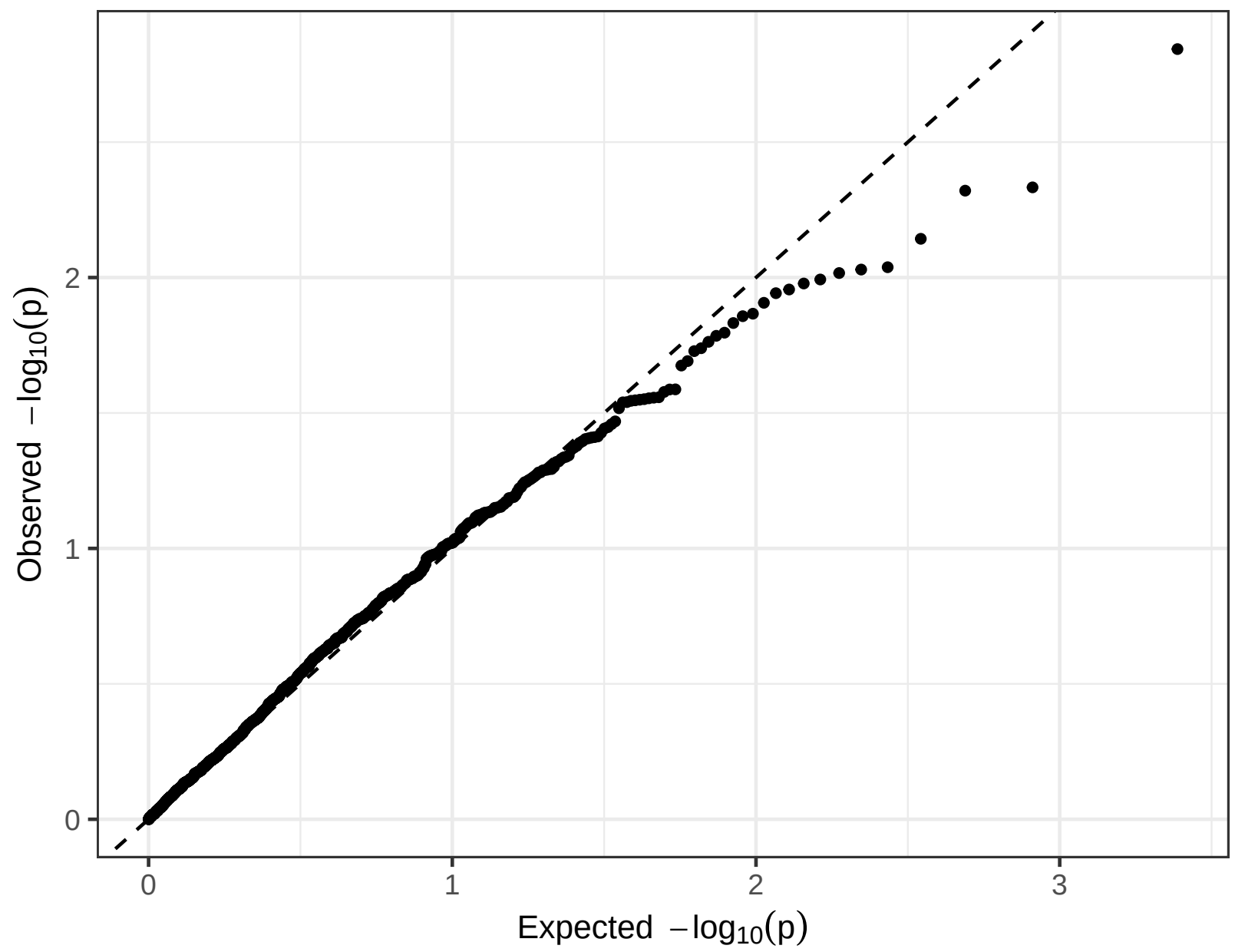

Pygmaiobacter

Feces | Liver | . = 0.921

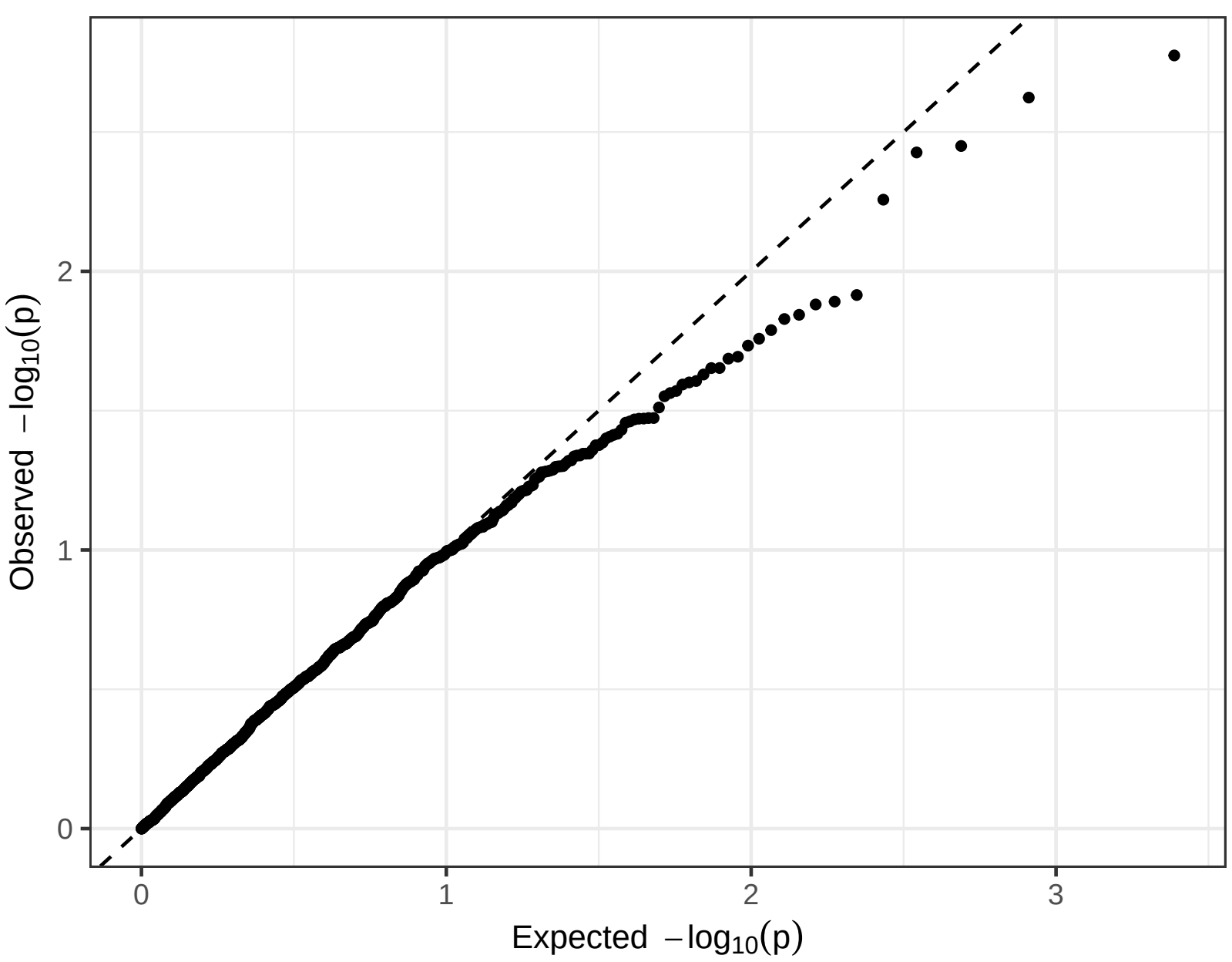

Pyramidobacter

Feces | Liver | . = 0.944

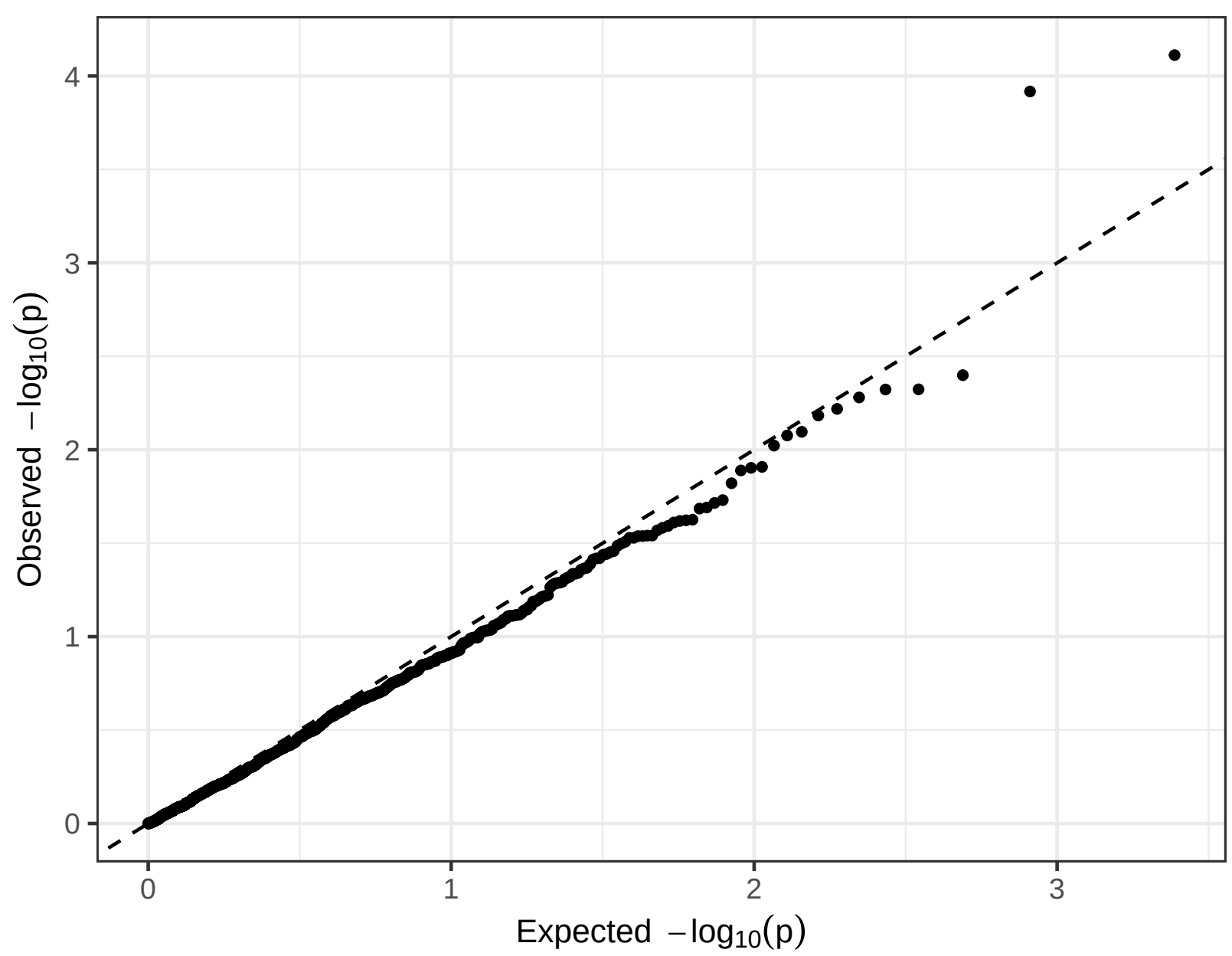

Quinella

Feces | Liver | . = 0.87

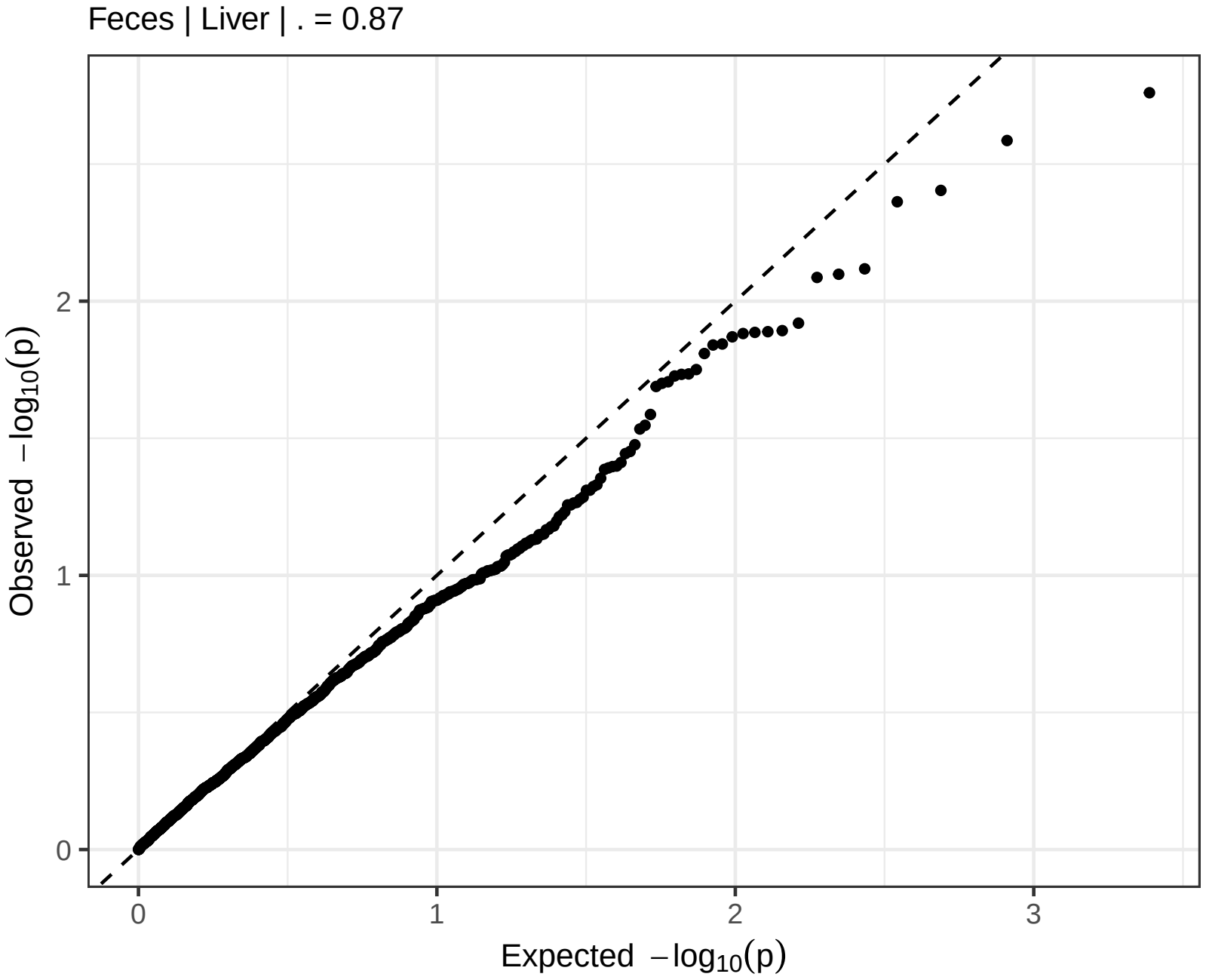

Rikenellaceae_RC9_gut_group

Feces | Liver | . = 0.854

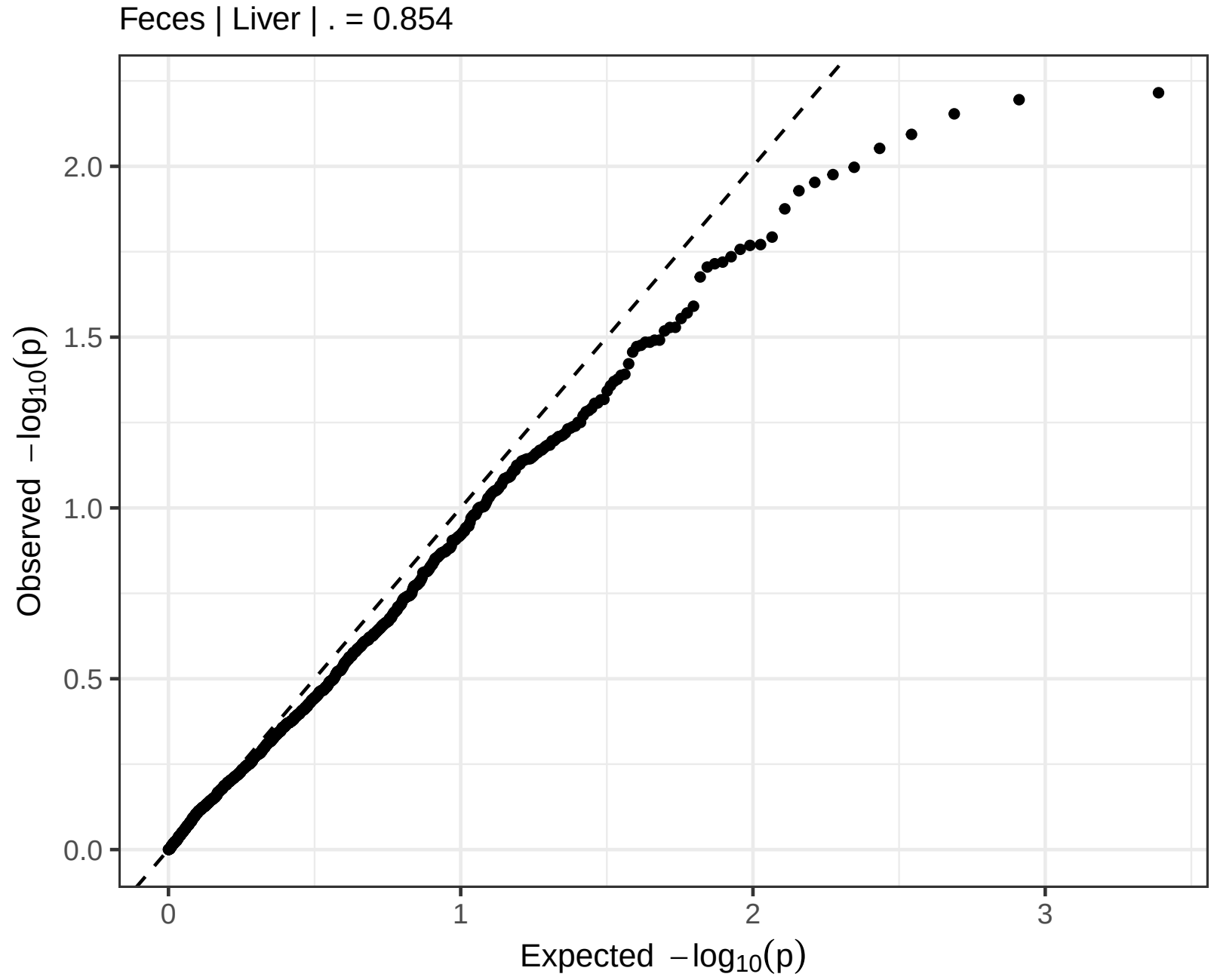

Supplement: Supplementary file 3 [file 10142_2026_2009_MOESM3_ESM.pdf]
